# Composite mobile genetic elements disseminating macrolide resistance in *Streptococcus pneumoniae*

**DOI:** 10.3389/fmicb.2015.00026

**Published:** 2015-02-09

**Authors:** Scott T. Chancey, Sonia Agrawal, Max R. Schroeder, Monica M. Farley, Hervé Tettelin, David S. Stephens

**Affiliations:** ^1^Division of Infectious Diseases, Department of Medicine, Emory University School of MedicineAtlanta, GA, USA; ^2^Laboratories of Microbial Pathogenesis, Department of Veterans Affairs Medical CenterAtlanta, GA, USA; ^3^Institute for Genome Sciences, University of Maryland School of MedicineBaltimore, MD, USA; ^4^Department of Microbiology and Immunology, University of Maryland School of MedicineBaltimore, MD, USA

**Keywords:** mobile genetic elements, transposons, integrative and conjugative elements, macrolides, antibiotic resistance, *Streptococcus pneumoniae*

## Abstract

Macrolide resistance in *Streptococcus pneumoniae* emerged in the U.S. and globally during the early 1990's. The RNA methylase encoded by *erm*(B) and the macrolide efflux genes *mef*(E) and *mel* were identified as the resistance determining factors. These genes are disseminated in the pneumococcus on mobile, often chimeric elements consisting of multiple smaller elements. To better understand the variety of elements encoding macrolide resistance and how they have evolved in the pre- and post-conjugate vaccine eras, the genomes of 121 invasive and ten carriage isolates from Atlanta from 1994 to 2011 were analyzed for mobile elements involved in the dissemination of macrolide resistance. The isolates were selected to provide broad coverage of the genetic variability of antibiotic resistant pneumococci and included 100 invasive isolates resistant to macrolides. Tn*916*-like elements carrying *mef*(E) and *mel* on the Macrolide Genetic Assembly (Mega) and *erm*(B) on the *erm*(B) element and Tn*917* were integrated into the pneumococcal chromosome backbone and into larger Tn*5253*-like composite elements. The results reported here include identification of novel insertion sites for Mega and characterization of the insertion sites of Tn*91*6-like elements in the pneumococcal chromosome and in larger composite elements. The data indicate that integration of elements by conjugation was infrequent compared to recombination. Thus, it appears that conjugative mobile elements allow the pneumococcus to acquire DNA from distantly related bacteria, but once integrated into a pneumococcal genome, transformation and recombination is the primary mechanism for transmission of novel DNA throughout the pneumococcal population.

## Introduction

*Streptococcus pneumoniae*, the pneumococcus, remains a significant risk to human health. Treatment of pneumococcal disease has been hindered by emergence of resistance to the key antibiotics (Klugman and Lonks, 2005). Macrolide resistance in pneumococci emerged throughout the 1990's, a process that has been documented in the Atlanta, Georgia, USA metropolitan area by an ongoing prospective-based surveillance of invasive pneumococcal disease by the Georgia Emerging Infections Program (Farley et al., 2002). A high incidence of efflux-mediated macrolide resistance was observed in serotypes prior to the introduction of the Seven-valent Pneumococcal Conjugate Vaccine (PCV7) in the Atlanta area in late 2000 (Gay et al., 2000; Stephens et al., 2005). PCV7 targets capsular polysaccharides of seven serotypes 4, 6B, 9V, 14, 18C, 19F, and 23F (Stephens et al., 2005). Expansion of these serotypes prior to PCV7 was a major driver of increased macrolide resistance in Atlanta (Stephens et al., 2005).

Most macrolide resistance in *S. pneumoniae* is conferred by the efflux genes *mef*(E) and *mel* or the target-modifying RNA methylase gene *erm*(B). The *mef*(E)/*mel* operon confers the M-phenotype, that is, resistance to 14- and 15-membered macrolides but not lincosamides or streptogramin B (Leclercq and Courvalin, 1991). Erm(B) confers resistance to macrolides, lincosamides and streptogramin B (MLS_B_ antibiotics) which is the MLS_B_-phenotype (Syrogiannopoulos et al., 2003). The dissemination of macrolide resistance genes in pneumococci has been aided by mobile genetic elements (MGE). The *mef*(E) and *mel* genes are located on the Macrolide Genetic Assembly, Mega (Gay and Stephens, 2001). Mega can be 5.5 kb (Mega-1) or 5.4 kb (Mega-2) based on the size of the *mef*(E)/*mel* intergenic region. Expression of the *mef*(E)/*mel* operon is controlled by transcriptional attenuation and is inducible by 15- and 16-membered macrolides (Ambrose et al., 2005; Wierzbowski et al., 2005). Immediately downstream and convergent to the *mef*(E)/*mel* operon are four co-transcribed open reading frames, *orfs 3-6*. This operon has organization and sequence similarity to a SOS-responsive operon on the pneumococcal conjugative transposons Tn*5252* and Tn*5253* (Munoz-Najar and Vijayakumar, 1999; Gay and Stephens, 2001). Tn*5253*, which is Tn*5252* with a Tn*916*-like insertion, is the prototype for a series of Tn*5253*-like integrative and conjugative element (ICE). It is one of two ICE demonstrated to carry macrolide resistance determinants in pneumococci (Ayoubi et al., 1991; Croucher et al., 2014; Mingoia et al., 2014). The other ICE commonly associated with macrolide resistance is ICE*Sp*23FST81 (Croucher et al., 2009).

The Mega element has integrated into at least four loci in the pneumococcal chromosome. Mega insertion classes I–IV were originally identified in US isolates (Gay and Stephens, 2001) and were also observed in Europe (Del Grosso et al., 2006). The class I Mega insertion site is in a homolog of the *S. pneumoniae* TIGR4 gene SP_1598 encoding a phosphomethylpyrimidine kinase predicted to be involved in thiamine biosynthesis (Gay and Stephens, 2001). Class II insertions are in a DNA-methyladenine glycosidase (SP_0180) and class III are in *capA* (SP_0103) located in the capsule biosynthesis locus. Class IV is in the RNA-methyltransferase *rumA* (SP_1029) located at the left flank of the pneumococcal pathogenicity island (PPI-1) (Brown et al., 2001).

**Figure 1:**
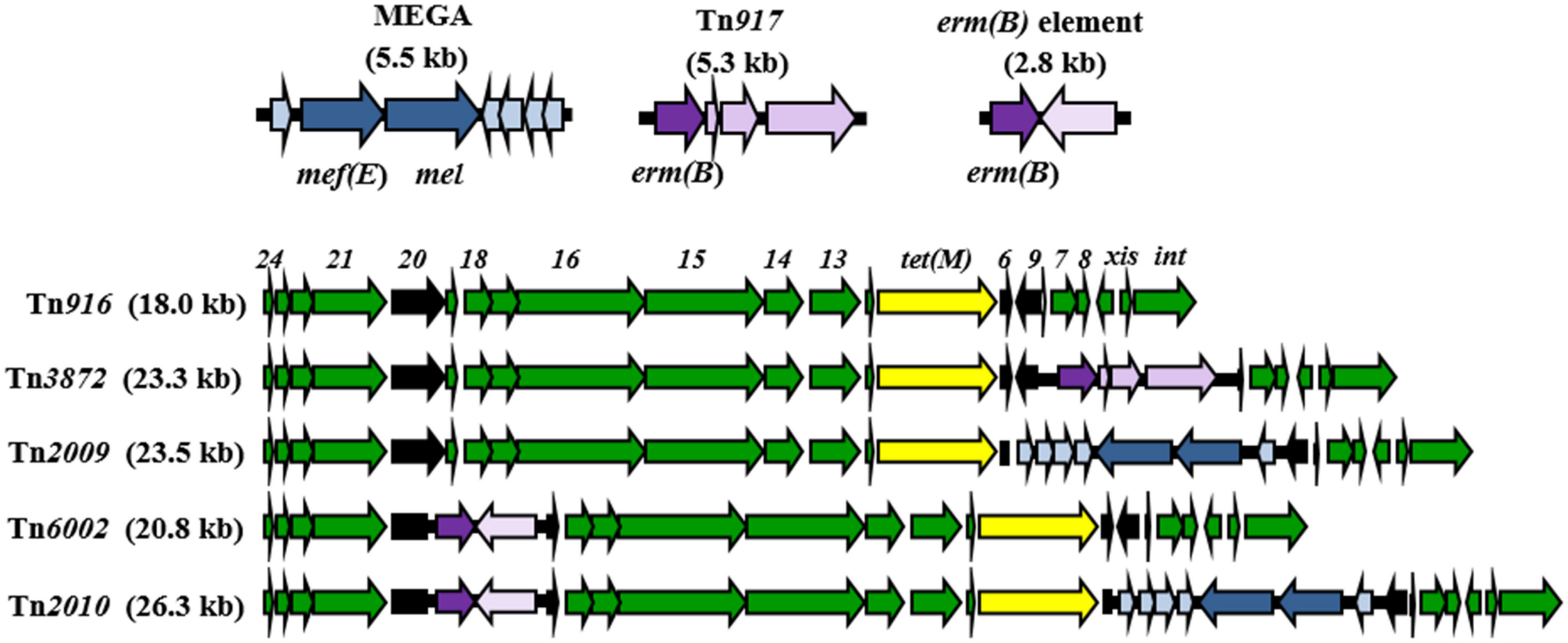
**Macrolide resistance determinants associated with mobile elements in pneumococci**. Conjugative transposon Tn*916* and Tn*91*6-like mosaic elements carrying Mega (blue), and *erm*(B) on the *erm*(B) element and Tn*917* (purple). The yellow arrows indicate *tet*(M) encoding tetracycline resistance. Black arrows indicate insertion sites.

In addition to the locations in the pneumococcal chromosome backbone, Mega has integrated into *orf6* of Tn*916*, a 18.0 kb conjugative transposon associated with tetracycline resistance due to the presence of *tet*(M) (Roberts and Mullany, 2011). Tn*916* has been enlisted to disseminate macrolide resistance in many forms. The Tn*916*-like element carrying Mega inserted into *orf6* is Tn*2009* (Del Grosso et al., 2004). A Tn*916*-like element with a 2.8 kb fragment carrying *erm*(B), named “*erm*(B) element,” inserted into Tn*916 orf20* is Tn*6002* (Cochetti et al., 2007) (Figure 1). A Tn*916*-like element harboring both *orf6*::Mega and *orf20*::*erm*(B) is Tn*2010* (Del Grosso et al., 2007) (Figure 1). The fact that the two smaller cassettes are inserted into the same locations in the different Tn*916*-like elements suggests either a very specific target sequence leading to multiple identical insertions, or that Tn*2010* is linked to Tn*2009* and Tn*6002* through recombination of macrolide resistance-conferring MGE. The same is true for Tn*2009*, Tn*3872*, and Tn*2017*. Tn*3872* and Tn*2017* are Tn*916*-like elements with *orf9* disrupted by Tn*917*, a 5.3 kb transposon-related element carrying *erm*(B) (McDougal et al., 1998). Tn*2017* is nearly identical to Tn*3872*, but also contains Mega integrated into Tn*916 orf6* (Del Grosso et al., 2009). The apparent interchangeability of the smaller macrolide resistance elements raises a question regarding the relative roles of conjugation and recombination in the dissemination of macrolide resistance and the evolution in the pneumococcus. *S. pneumoniae* is a naturally competent bacterium and is extremely proficient at acquiring and incorporating exogenous DNA into its genome (Berge et al., 2002; Claverys et al., 2006; Prudhomme et al., 2006). On the other hand, macrolide resistance-encoding Tn*916*-like elements appear to be incapable of pneumococcus-to-pneumococcus conjugation (Del Grosso et al., 2004; Zhou et al., 2014). Tn*2009* and Tn*2010* are non-conjugative while Tn*6002* is conjugative but at very low frequencies (Del Grosso et al., 2004, 2007; Cochetti et al., 2007). Conjugative transfer of Mega from *S. salivarius*, however, has been observed, but only from a host strain with a co-resident conjugative transposon (Santagati et al., 2009). How important is DNA acquisition by conjugation in the pneumococcus, which is so proficient at transformation and recombination?

In the present study we utilized comparative genomics to identify the mobile elements involved in dissemination of macrolide resistance in a geographically defined population. We sequenced the genomes of 147 pneumococcal isolates, including 131 invasive and carriage isolates collected in Atlanta, Georgia over an 18 year period spanning the pre- and post-conjugative vaccine era (McDougal et al., 1998; Cochetti et al., 2008). The goal of this study was to make a broad survey of the genomic diversity in the pneumococcal clones circulating in the Atlanta metropolitan area in pre- and post-PCV7 eras, and to document the MGE that promoted the dissemination of macrolide resistance genes. By analyzing the genetic organization and the integration sites of the elements in relation to the clonal lineage and serotype of the isolates that harbor them, we hoped to gain a better understanding of the relative roles of conjugation and transformation in the evolution and dissemination of macrolide resistance in *S. pneumoniae*.

## Materials and methods

### Bacterial strains

Atlanta invasive *S. pneumoniae* isolates were obtained from the Georgia Emerging Infections Program and were isolated from patients in Atlanta, Georgia as part of an ongoing, prospective, population-based surveillance study (Farley et al., 2002). Macrolide resistant isolates were assayed for the presence of macrolide resistance genes *mef*(E), *mel* and *erm*(B) by PCR as previously described (Stephens et al., 2005). One hundred and twenty one isolates recovered from patients between 1994 and 2011 were non-randomly selected for whole genome sequencing (Table S1). Selection criteria included capsule serotype, antibiotic resistance, macrolide resistance genes, and date of isolation. In addition to macrolide resistance, clindamycin, tetracycline, and chloramphenicol were considered indicators of mobile elements. Sequence type was also considered if available (see below). Thirteen invasive pneumococcal isolates from U.S. states other than Georgia were provided by the Antimicrobial Bacterial Core Surveillance Program and the Centers for Disease Control and Prevention (CDC) (McCormick et al., 2003). These included four isolates from Maryland, two each from Connecticut, Oregon, and Minnesota, and one each from California, New York, and Tennessee. Three invasive isolates, two from Europe and one from the North Carolina, were provided by the Pneumococcal Molecular Epidemiology Network (PMEN) (McGee et al., 2001). Finally, the study was supplemented with 10 carriage isolates collected from the Atlanta population; five each isolated pre-PCV7 (1995) and post-PCV7 (2009) (Sharma et al., 2013). Carriage isolates were collected from nasopharyngeal swabs of healthy children between the ages of 5 months and 5 years old (Sharma et al., 2013). The total number of sequenced pneumococcal genomes was 147 (Table S1).

### Characterization of isolates

The capsule serotype of each isolate was determined by the institution from which it was acquired and verified by analyses of the capsule locus in the genome sequence. Minimal inhibitory concentrations (MIC) of erythromycin, clindamycin, tetracycline, and chloramphenicol were determined by microdilution or Etest (bioMerieux Inc., Durham, NC). The Multi Locus Sequence Type (MLST) of selected isolates was determined by Sanger nucleotide sequencing of PCR products with MLST allele-specific primers as previously described (Enright and Spratt, 1998). The sequence types of the remaining isolates were determined from allelic data extracted from their genomes. Isolates were assigned to a clonal complex based on eBURST analysis of the complete *S. pneumoniae* MLST database as of September, 2014 (http://pubmlst.org/spneumoniae/).

### Genome sequencing

For each isolate sequenced, two shotgun libraries were constructed for 454 sequencing: one rapid non-paired-end library for coverage and one 3 kb paired-end library for assembly scaffolding. All isolates were sequenced using the 454 GS FLX Titanium to a depth of sequence coverage ranging from 16× to 57×. Shotgun reads were assembled into 4–32 contigs per isolate using Celera Assembler v6.1 (Myers et al., 2000). Genome sequences have been deposited in the GenBank Nucleotide Sequence Database (http://www.ncbi.nlm.nih.gov/) (Table S1) and the PathoSystems Resource Integration Center (PATRIC) (Wattam et al., 2014). Isolates are available through the American Type Culture Collection-managed Biodefense and Emerging Infections Resources repository (http://www.beiresources.org).

### Whole genome alignment and phylogenetic tree construction

Two high throughput reference-based pipelines were used to perform single nucleotide polymorphism (SNP) discovery and validation in 166 genomes using the TIGR4 genome as a reference (Tettelin et al., 2001). The genomes included the 147 from the present project plus 15 publicly available, closed, pneumococcal genomes and four whole genome shotgun sequences from this project that were later excluded from this study due to the lack of associated meta-data (Table S1). The analysis pipelines made extensive use of Perl (https://www.perl.org) scripts and were implemented within the Ergatis framework (https://github.com/jorvis/ergatis) (Orvis et al., 2010). The alignment-based SNP discovery pipeline named Skirret mainly executes a series of utilities from the MUMmer 3.0 package (http://mummer.sourceforge.net/) (Kurtz et al., 2004) namely nucmer, delta-filter and show-SNPs to determine SNP positions. This pipeline was run on all 166 assembled genomes with a minimum alignment identity of 98%. A k-mer based algorithm called kSNP (http://sourceforge.net/projects/ksnp/) (Gardner and Hall, 2013) was also employed to identify SNPs in the assembled genomes using 41 bp as the length of the k-mer. Next, a BLAST-based validation pipeline was used to merge the SNP panels from the Skirret and kSNP pipelines and 41 bp sequence surrounding each SNP position was extracted from the reference TIGR4 genome. These short sequences were searched against all 166 genomes using all-vs.-all BLASTN searches (*e*-value cut-off 0.0001) to eliminate false positive SNP calls, only core SNPs in short sequences found in all 166 genomes were selected. Putative recombinogenic regions were not excluded. The set of 10,120 core SNP bases were concatenated from each of the input genomes to create a multi-FASTA file. This file was used for phylogenetic tree construction using the Geneious Basic software version 5.6.4 with neighbor-joining and the Hasegawa, Kishino and Yano (HKY) genetic distance model. The HKY model assumes that every base has a different equilibrium base frequency, and also assumes that transitions evolve at a different rate to transversions. A consensus tree was built based on 100 iterations to provide an estimate for the level of support for each clade in the final tree. We used a 100% consensus support threshold that resulted in a strict consensus tree where the clades included were those that were present in all the trees of the original set.

### Identification and characterization of mobile elements

Mega and Mega insertion sites were located by comparing genomes with the 5,511 bp Mega element from strain GA17457 (Data File S1). ICE were identified by BLASTN (Altschul et al., 1997) search of the terminal direct repeats localized at the ends transposons ICE*Sp*23FST81 (81.0 kb; Accession no. FM211187), Tn*5253* (66.2 kb; Accession no. EU351020) (Iannelli et al., 2014), Tn*916* (18.0 kb; Accession no. U49939) and others. Elements were characterized by the *int* genes they carried. Integrase genes were identified by BLASTN search with query sequences from described ICE including those mentioned above. All query sequences used are supplied as supporting information (Data File S1). Comparisons of composite ICE were visualized with the Artemis Comparison Tool (Carver et al., 2005). Genomes were also searched for antibiotic resistance genes, including *mef*(E), *mef*(A), *mel(msr*(D)), *erm*(B), *erm*(A), *tet*(M) and *cat* (Data File S1). The nucleotide sequences of each Mega, Tn*916*-like and composite elements are provided as supporting information (Data Files S2, S3 and S4, respectively).

## Results

### Whole genome comparisons

Isolates of *S. pneumoniae* were selected from the >13,000 strain collection of the Georgia Emerging Infections Program. Isolates were collected as part of an ongoing, prospective, population-based surveillance of invasive pneumococcal disease (IPD) in the Atlanta metropolitan area (Farley et al., 2002). One hundred and twenty one invasive *S. pneumoniae* isolates from 1994 to 2011 were non-randomly selected for genome sequencing. Isolates were selected to provide broad coverage of the genotypes existing in the population during the surveillance period, but with a focus on macrolide resistance, with 100 resistant to erythromycin (MIC >1 μg/ml). 74 displayed the M-phenotype as indicated by susceptibility to the lincosamide clindamycin and 24 were also resistant to clindamycin (MLS_B_-phenotype) (Table S1). In addition to the Atlanta invasive isolates, 10 carriage isolates from Atlanta were included. Also included were 16 strains not from Atlanta. For more detail, please see the Material Methods and supporting information (Table S1). In total 147 pneumococcal genomes were sequenced including 23 different serotypes, 77 multi-locus sequence types (ST), 24 clonal complexes (CC) and two singletons (Table S1).

**Figure 2:**
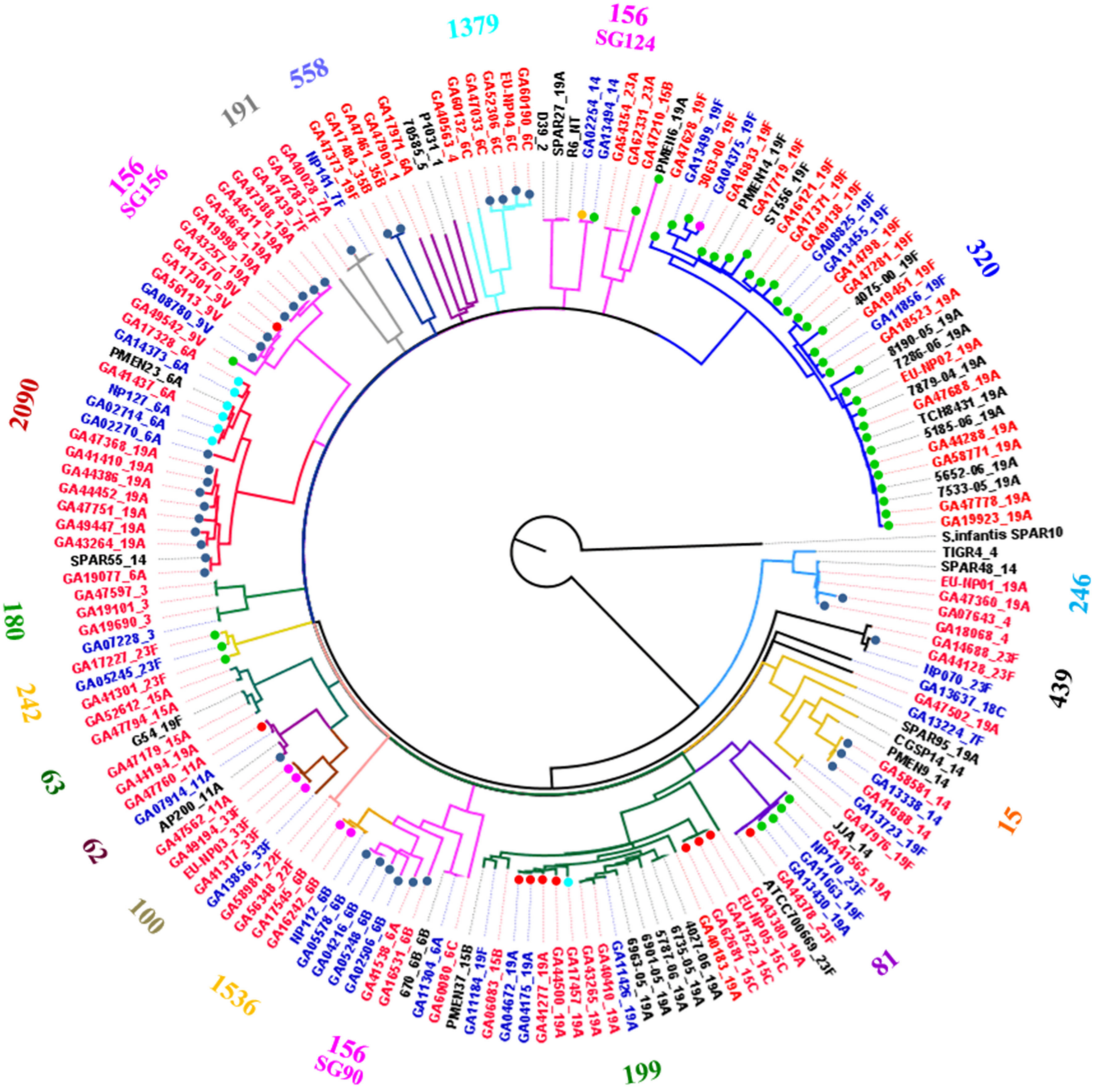
**Whole genome SNP-derived phylogenetic tree**. Whole genome single nucleotide polymorphism analysis of 166 genomes, including 147 from the present study and nine publically available closed genomes, using the TIGR4 genome as reference. The tree was rooted using *S. infantis* SPAR10 as the outgroup. Numbers on the perimeter indicate the MLST clonal complex of the isolates and are colored the same as the branches leading to the isolates in each complex. The clonal complex of each sequence type was defined as the founder ST of a clonal group, as predicted by eBURST analyses of the entire *S. pneumoniae* MLST databased accessed in September, 2014 (http://pubmlst.org/spneumoniae/). Isolate names and serotype labels colored blue indicates that the strain was isolated prior to the introduction of PCV7 in 2000. Red colored names and serotypes indicate the strains were isolated in 2000 or later. Colored dots indicate a Mega insertion in the isolate and are color coded by Mega insertion site, light blue, class I; dark blue, class II; red, class III; pink, class IV; green, class V (Tn*916 orf6*); and gold, novel insertion site.

The 147 isolates from this study and 15 publically available genomes were compared using whole genome single nucleotide polymorphism (SNP) analyses. The resulting phylogenetic tree is shown in Figure 2. The genomes clustered into MLST clonal complexes with the exception of CC156 which was divided into three clades (Figure 2). This may be an example of a limitation of MLST typing as the three clusters appear to be unrelated. CC156 is a large complex consisting of multiple subgroups thus not all were as closely related as the label CC156 implied (Figure S1). For example ST90 has only one allele in common with ST156. However, it was interesting to observe that two of the CC156 subgroups appear to be linked by a common Mega insertion event (see below). Further, the eBURST-predicted subgroups (SG90, SG124 and SG156) are well-established independent clonal complexes (CC90, CC124 and CC156, respectively) indicating that their inclusion into a single clonal complex is an artifact of eBURST and not a true indication of common ancestry.

### Distribution of the macrolide resistance-encoding element Mega

Of the Atlanta invasive isolates, 86 contained Mega integrated into the pneumococcal chromosome backbone or nested within larger mobile elements. Mega was found in 17 serotypes, 16 clonal complexes and two singletons (Table 1). Of these, 48 carried the 5,511 kb Mega type 1 (Mega-1) with a 119 bp *mef*(E)/*mel* intergenic region and 38 carried the 5,412 kb Mega type 2 (Mega-2) with a 99 bp deletion in the intergenic region (Table 1). The genome of GA07643 contained a previously unidentified 5,617 bp Mega variant, designated Mega-3, characterized by a 107 bp tandem duplication that included the first 90 bp of *mel* and 23 bp upstream containing its ribosomal binding site (RBS). Analyses of the 454 raw reads revealed that both copies of the repeated sequence were contained on single reads and the tandem duplication region had greater than 40× sequence coverage. This confirmed that the duplication was not an assembly error. The authentic duplication resulted in an intergenic region that contained the 119 bp that immediately follows *mef*(E) in Mega-1, followed by a 38 codon ORF that includes the first 30 amino acids of Mel. The ORF had the *mel* RBS and appeared suitable for protein expression. Theoretically this could have a polar effect of *mel* translation, however, GA07643 had an erythromycin MIC of >16 μg/ml so it does not appear to have had a negative impact on resistance (Table S1). The full-length copy of *mel* overlapped the 3′ end of the duplicated ORF by 10 bp and maintained the native *mel* RBS. There was no correlation between the size of the *mef*(E)/*mel* intergenic region and the level of efflux-mediated erythromycin expressed by that strain (data not shown).

**Table 1:** **Insertion sites of Mega**.

**Element (no.)**	**Insert^a^ (no.)**	**TIGR4 homolog^b^**	**Function**	**CC^c^**	**Serotype(s)^d^ (no.)**
Mega-1 (48)	I (6)	SP_1598	Phosphomethylpyrimidine kinase	2090	6A (5)
				199	19A (1)
	II (2)	SP_0180	DNA-3-methyladenine glycosylase	1379	6C (1)
				62	11A (1)
	III (10)	SP_0103	Capsule biosynthesis	156	9V (1)
				62	11A (1)
				199	15C (3), 19A (4)
				81	23F (1)
	IVb (2)	SP_1029	RNA methyltransferase	100	33F (2)
	V (27)	na	Transcriptional regulation	156	9V (1)
				124	14 (1)
				2218	15B (1)
				320	19A (6), 19F (15)
				242	23F (3)
	Novel (1)	*S. suis* element^e^			14 (1)
Mega-2 (38)	II (35)	SP_0180	DNA-3-methyladenine glycosylase	156	6A (1), 6B (5), 9V (2), 19A (5), 23A (2)
				2090	6A (2), 19A (5)
				15	14 (3)
				558	35B (2)
				62	11A (1)
				191	7A (1)
				66	4 (1)
				320	19F (1)
				433	22F (1)
				439	23F (1)
				singletons	19A (2)
	IVa/c (3)	SP_1029/SP_1067	Pneumococcal Pathogenicity Island 1	1536	6B (2)
				320	19F (1)
Mega-3 (1)	II (1)	SP_0180	DNA-3-methyladenine glycosylase	66	4 (1)

a *Class of Mega insertion site*.

b *Genes disrupted by Mega insertion. Classes I–IV are disrupted genes and are shown by S. pneumoniae TIGR4 locus tag*.

c *CC, clonal complex*.

d *PCV7 capsule serotypes are underlined*.

e *Integrative and conjugative element closely related to an element in Streptococcus suis 05HAS68 (Accession no. CP002007)*.

Mega was found inserted into each of the four previously identified insertion sites within the pneumococcal chromosome backbone (classes I-IV) (Gay and Stephens, 2001), in Tn*916*-like elements (class V), and in one novel site (Table 1). Six genomes contained Mega integrated into the class I insertion site, five of which were clonal (6A, CC2090) indicating clonal dissemination (Figure 2, light blue dots; Table 1). The exception was GA17457, a serotype 19A, CC199 isolate that has been used as the genetic background for studying the regulation of *mef*(E) and *mel* (Zähner et al., 2010; Chancey et al., 2011). GA17457 was closely related to four isolates in CC199 that contained Mega-1 in class III sites (Mega-1.III) and was the only CC199 strain to have Mega-1 integrated into class I (Mega-1.I) (Figure 2). Class II Mega insertions were identified in 38 genomes (Table 1). The elements inserted into this site include Mega-1 (*n* = 2), Mega-2 (*n* = 35) and the novel Mega-3 in strain GA07643 (Table 1). Mega-1.III was found in ten genomes in diverse backgrounds: five serotypes and four clonal complexes (Table 1). Mega class III insertions clustered mainly in CC199 but single isolates containing Mega-1.III were identified in CC156, CC81, and CC62 (Figure 2). Five genomes contained Mega-1 or Mega-2 inserted into the class IV site which was divided into three subclasses, IVa, IVb, and IVc (see below).

**Figure 3:**
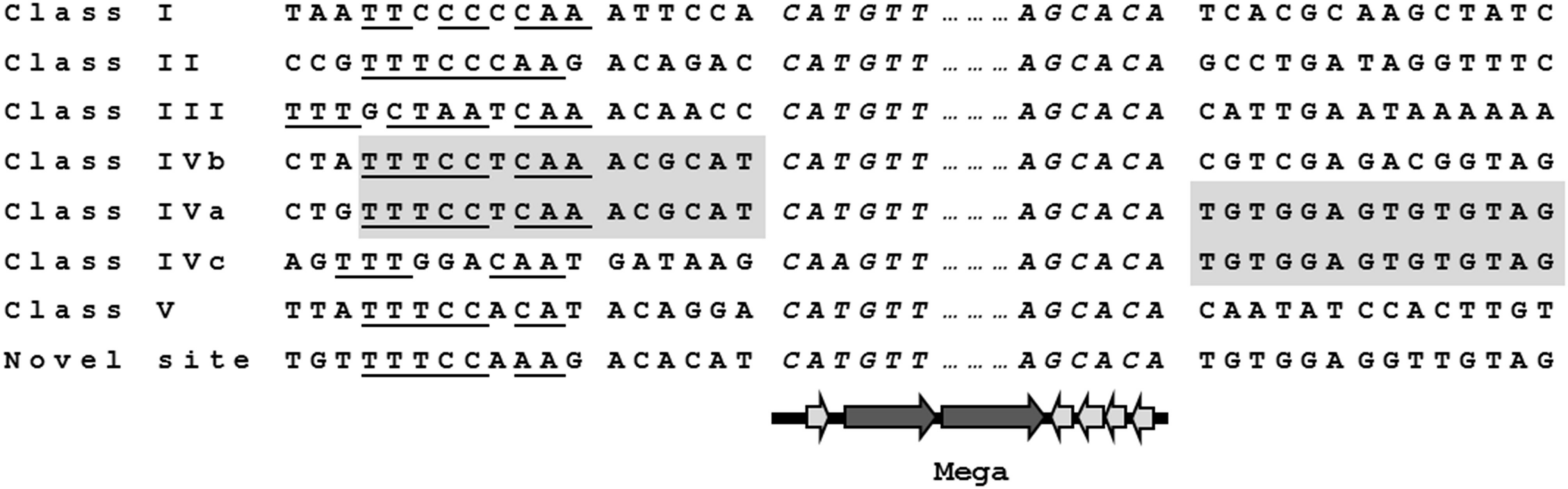
**Mega insertion sites**. Target sites of the mobile element Mega encoding macrolide efflux genes *mef*(E) and *mel*. Putative six base coupling sequences (C.S.) are shown adjacent to both ends of Mega (italics). Underlined letters indicate conserved nucleotides that may be the consensus recognition sequence 5′ TTTCCNCAA 3′. Class IV was composed of three distinct, subclasses, IVa, IVb, and IVc. Class IVb and IVa share the same left junction and IVa and IVc same right junction (shaded).

Comparison of the nucleotide sequences flanking Mega inserted into each site revealed conserved sequences upstream of the Mega insertion site that may indicate a target sequence (Figure 3). The potential consensus was 5′ TTTCCNCAA 3′ and was located approximately six bp upstream of Mega in all sites (Figure 3). The six base pairs flanking Mega on either side resembled coupling sequences as described for conjugative transposons such as Tn*916* (Figure 3). The coupling sequences and Mega flanking sequences illustrated in Figure 3 were representative of all Mega elements inserted into the respective sites. This implies that the Mega integrated into each site were descended from a single, or very few, ancestral transposition event(s). An example of this can be seen in the whole genome phylogeny (Figure 2). The class II Mega insertion was present in almost all CC156 isolates (Figure 2, dark blue dots). Genomes from isolates of this large complex clustered into three clades correlating to CC156 subgroups 90, 124, and 156 (Figure 2). Almost all genomes in subgroups 90 and 156 contained Mega (Figure 2). All 10 subgroup 156 genomes contained Mega; eight Mega-2, class II, one Mega-1 class III insertion and one integration into *orf6* of Tn*916* (Table 1; Figure 2). Further, CC2090, which branches from CC156, has two clades in one of which nine of nine isolates contain Mega class II. The other branch of CC2090 contains almost all isolates with Mega class I indicating a second conjugation event in this complex (Figure 2). Again this strongly implies that Mega inserted into the class II site early in the history of these lineages.

**Figure 4:**
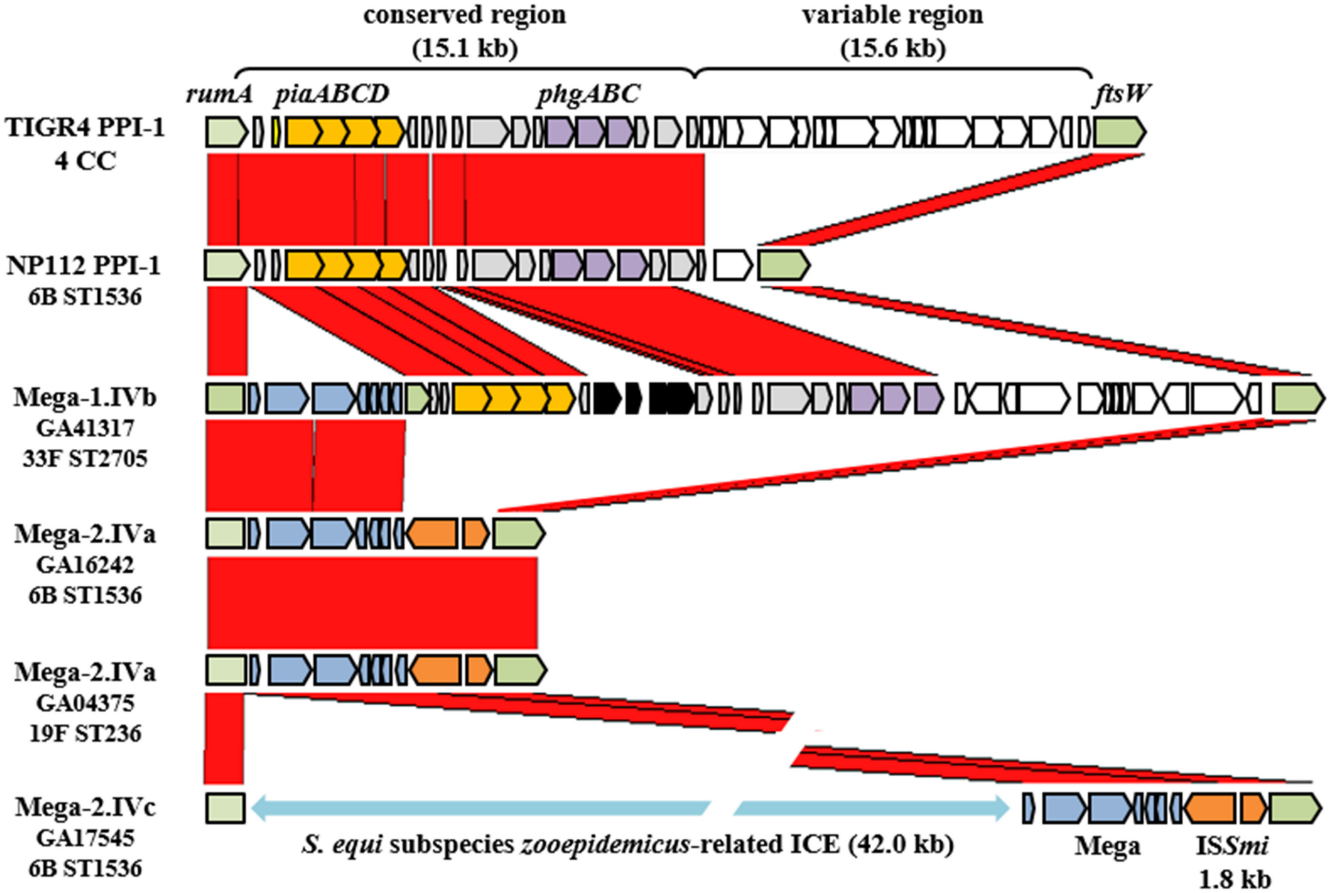
**Variations of the Pneumococcal Pathogenicity Island-1 associated with Mega**. The Pneumococcal Pathogenicity Island-1 in TIGR4 extends from *rumA* (SP_1029) to *ftsW* (SP_1065). Strain NP112 is closely related to GA16242 and GA17545, the Mega-2.IVa and IVc strains, but contains the conserved region of PPI-1. Red blocks represent regions of homology. Arrows indicate genes. Green, PPI-1 borders; yellow, iron uptake operon *piaABCD*; purple, osmotic stress tolerance operon *phgABC*; gray, PPI-1 conserved region genes not otherwise labeled; white, PPI-1 variable region genes; blue, Mega; orange, IS*Smi*1. The light blue double arrow indicates the presence of an undefined transposon-like sequence from *S. equi* subspecies *zooepidemicus*.

The Mega class IV insertion site, as previously described (Gay and Stephens, 2001), was near the 3′ end of *rumA* (SP_1029, TIGR4 annotation) that encoded a tRNA methyltransferase and was located at the left end of the Pneumococcal Pathogenicity Island-1 (PPI-1) (Brown et al., 2004) (Figure 4). Large deletions and multiple MGEs were associated with Mega integrated into this site, giving rise to the three class IV subclasses (Table 1). Starting with the simplest, the class IVb insertion contained Mega-1 integrated into *rumA* leaving Mega flanked by the fragments of the gene (Figure 4). Two class IVb isolates were identified, all serotype 33F, CC100 (Table 1). The class IVa insertion contained Mega-2 integrated into the same location in *rumA* and had an identical sequence to the left junction of Mega in class IVb (Figure 4). However, the downstream remnant of *rumA* was deleted along with the entire 30.7 kb PPI-1 (SP_1029 to SP_1067) (Figure 4). The island was replaced by Mega-2 and a 1,801 bp insertion sequence IS*Smi*2 found in *S. mitis* strain B6 (Accession no. FN568063.1) (Figure 4). The class IVc Mega-2 insertion was similar to class IVa in that PPI-1 was deleted and IS*Smi2* was located at the right junction with the chromosome (Figure 4). However, a 41,995 bp transposon-like sequence most closely related to ICE*Sz1* from *S. equi* subspecies *zooepidemicus* strain H70 (Accession no. FM204884) (Holden et al., 2009) was inserted between the *rumA* insertion site and the left end of Mega-2. Thus, the class IVc Mega site organization consists of, in tandem, a transposon previously undefined in pneumococci, Mega-2 and ISS*mi*2. The Atlanta invasive isolate GA17545 (6B, CC1536) contained the only class IVc Mega insertion identified (Tables S1, S2). Class IVa isolates are also typically 6B, CC100, however, a single Atlanta invasive isolate, GA04375, with serotype 19F, and in CC320 was also found to contain Mega-2.IVa (Table 1; Table S1). GA04375 was isolated in 1995 and was the earliest isolated CC320 in the genome collection and only one of two CC320 that contained Mega inserted into the pneumococcal chromosome backbone. The other was 3063-00, an invasive 19F, CC320 collected in 1999 in Tennessee (Table S1, S2). The serotype 14, CC156 isolate GA02254 from 1994 contained Mega-1 inserted into an unidentified transposon-like sequence and was most closely related to a sequence in *S. suis* 05HAS68 (Accession no. CP002007). Twenty-seven genomes contained Mega-1 inserted into *orf6* of a Tn*916*-like element (class V) (Table 1). Tn*916*-like elements are discussed further in the next section. These data suggest that PPI-1 is a hotspot for integration by recombination and conjugation. This locus could be an entry point for non-pneumococcal DNA to integrate into the pneumococcal genome. Conversely, the conservation of the target site in *rumA* across non-pneumococcal streptococci makes it possible that pneumococcus has been the donor and a non-pneumococcal streptococcus the recipient. The high degree of recombination in the pneumococcus makes it difficult to determine directionality of gene flow, but it likely can flow in both directions. With just these few isolates, we showed evidence for HGT with three non-pneumococcal streptococci; *S. equi*, *S. mitis*, and *S. suis*.

### Tn*916*-like elements carrying macrolide resistance determinants

The genomes were searched for Tn*916*-like elements by BLASTN using the conserved ends of Tn*916* (Data File S1). Of the 86 macrolide resistant invasive isolates from Atlanta, 53 contained a full-length Tn*916*-like element (Table S3). A macrolide resistance determinant was nested within the Tn*916* element in all but six, which instead contained Tn*916* and an unlinked Mega-2.II (*n* = 5) or Mega-1.III (*n* = 1) (Table S3). Four distinct Tn*916*-like elements containing one or two macrolide resistance determinants were identified: Tn*2009*, Tn*2010*, Tn*6002*, and Tn*3872* (Figure 1; Table 2). Tn*2009* was found in 15 Atlanta invasive isolates spanning six CC and six serotypes (Table S3). Tn*6002* was carried by nine Atlanta invasive isolates including five CC (and a singleton) and seven serotypes (Table S3). Tn*2010* was found in 13 Atlanta invasive isolates, two Atlanta carriage isolates, and eight non-Atlanta invasive isolates (Table 2; Table S3).

**Table 2:** **Tn*916* and Tn*916*-like elements inserted directly into the chromosome backbone of invasive *S. pneumoniae* isolates from Atlanta, GA**.

**Element (no.)**	**Serotype (no.)**	**CC ^a^**	**Insertion Sites ^b^ (no.)**	**Function**	**Resistance genes**
Tn*916*-like	6B (5)	384	SP_1937	*lytA*, autolysin	*tet*(M)
Tn*6002*-like	22F (1)	433	SP_1438	ABC transporter/ATP-binding subunit	*erm*(B) *tet*(M)
Tn*6002*-like	15A (3)	63	SP_1947	Hypothetical protein	*erm*(B) *tet*(M)
	19A (1)				
Tn*2009*-like	9V (1)	156	SP_1638	Iron-dependent transcriptional regulator	*mef*(E) *mel tet*(M)
Tn*2009*-like	19F (6)	320	SP_1947	Hypothetical protein	*mef*(E) *mel tet*(M)
Tn*2010*-like	19A (6)	320	SP_1947	Hypothetical protein	*erm*(B) *mef*(E) *mel tet*(M)
	19F (7)				

a *Clonal complex as defined by eBURST analyses of multi-locus sequence type data*.

b *Chromosomal insertion sites are presented as the locus tag for homologous genes in S. pneumoniae TIGR4 (Tettelin et al., 2001)*.

**Figure 5:**
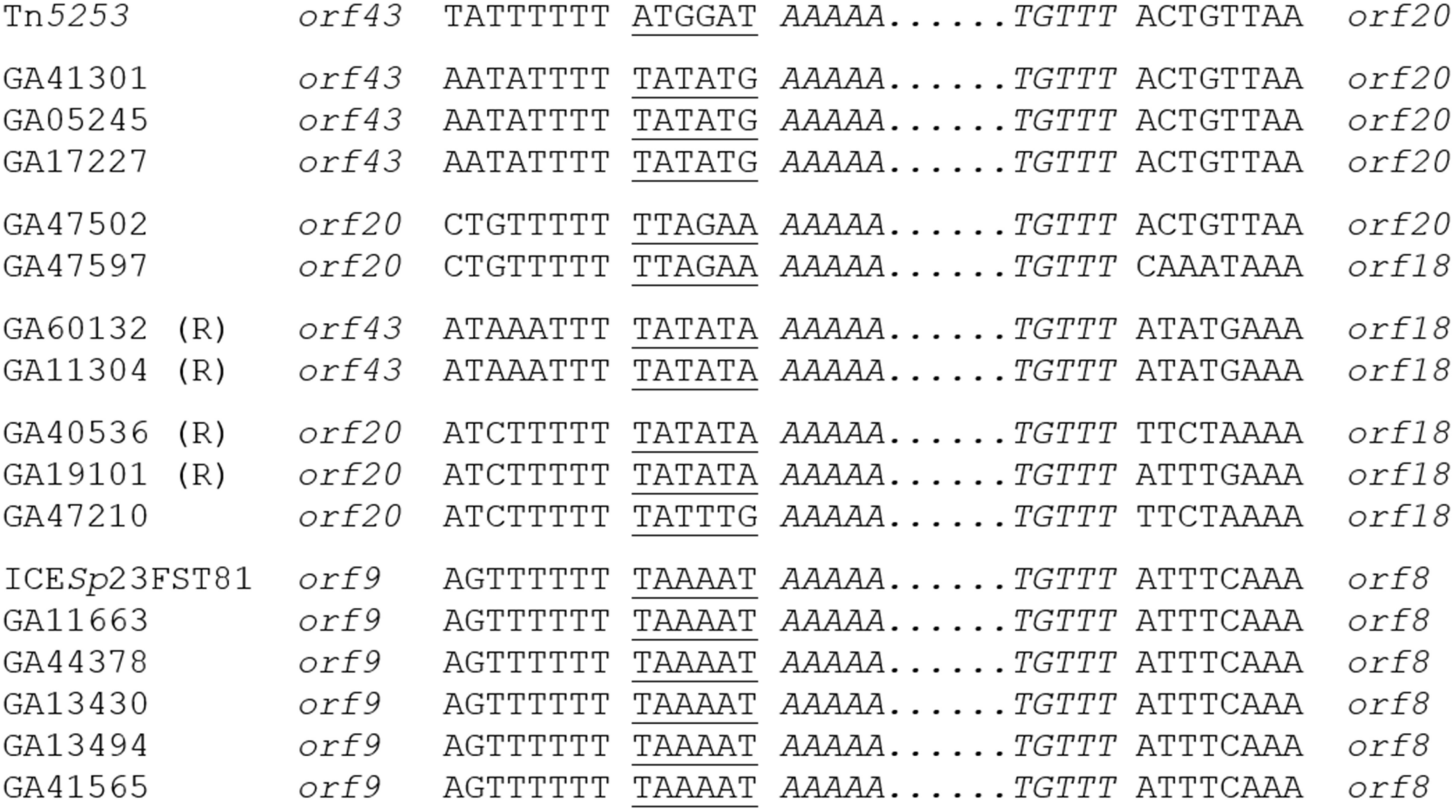
**Insertion sites of Tn*916*-like elements**. Insertion sites within composite elements. The terminal sequences of Tn*916* are shown in italics. Coupling sequences are underlined. Plain text indicates the sequences of ICE flanking the Tn*916*-like element. (R) indicates that the Tn*916*-like element is in the reverse orientation. The ORFs that flank the Tn*916*-like elements are indicated adjacent to the ICE sequences on either side.

To better understand the evolutionary relationships between these elements, the insertion site of each was determined. *Tn916*-like elements were integrated into four unique loci in the pneumococcal chromosome backbone and into several loci within Tn*5253*-like elements (Figure 5). Every Tn*2010* element discovered was inserted into SP_1947 (TIGR4 annotation) encoding a hypothetical secreted protein (Table 2; Figure 5). This is the same location where Tn*2010* has been previously reported to be integrated (Zhou et al., 2014). Six of 15 Tn*2009* elements and four of nine Tn*6002* elements found in invasive Atlanta isolates were inserted into the same locus as Tn*2010* (Table 2). The 6-base coupling sequences were identical for all elements in this locus suggesting that the elements found integrated into this locus were derived from a single transposition event (Figure 5). This further implies that the macrolide resistance elements Mega-1, Tn*917* and *erm*(B) were independently acquired by a Tn*916* residing in the SP_1947 locus. In addition, elements in this locus were identified in two clonal complexes (CC320, CC156) indicating transformation and recombination of the element in this locus between clonal complexes.

Tn*2009* was also identified integrated into SP_1638 (TIGR4), an iron-dependent transcriptional regulator, in a single serotype 9V, CC156 isolate from 2007. The fact that no element was identified in this locus from an earlier collected isolate may indicate that this integration event occurred more recently than the SP_1947 integration. Likewise, a single isolate (22F, CC433) from 2010 contained Tn*6002* integrated into the ABC transporter gene SP_1438. Five serotype 6B, CC384 isolates contained Tn*916* integrated into *lytA* (SP_1937) encoding an autolysin (Table 2). Each of these closely related isolates contained unlinked Mega-2.II (Table S2). The other Tn*916*-like elements identified were integrated into a composite ICE resembling Tn*5253* and are discussed below.

### Composite ICE containing macrolide resistance determinants

Tn*916*-like elements with macrolide resistance determinants have been associated with large conjugative transposons also known as ICE (Henderson-Begg et al., 2009; Mingoia et al., 2014). To determine if ICE have been involved in macrolide resistance dissemination in Atlanta, the genomes of 100 macrolide resistant invasive pneumococcal isolates from Atlanta were searched by BLASTN for the conserved ends of known ICE (Data File S1). The genomes of 15 macrolide resistant isolates contained composite ICE-like elements that ranged in size from approximately 57 to 88 kb (Table 3). Comparison of the integrase genes encoded on the ICE revealed two distinct families of conjugative transposons. Nine belonged to the Tn*5253* family and six belonged to the ICE*Sp*23FST81 family (Table 3). Each of the Tn*5253*-family transposons was inserted into the pneumococcal chromosome backbone in the gene for ribobiogenesis GTPase, *rbgA* (SP_1155, TIGR4 annotation) involved in assembly of 23S ribosomes. Downstream of Tn*5253*-like elements in this locus is the immunoglobulin A1 protease gene *iga* as has been described previously (Croucher et al., 2009). Each of the ICE*Sp*23FST81-like elements was inserted into the chromosome backbone gene *rplL* (SP_1354) which encodes ribosomal protein L7/L12 (Croucher et al., 2009). The correlation of ICE family and insertion sites reflects the specificity of the integrases Int_Tn*5253*_ and Int_ICE*Sp*23FST81_ (Mingoia et al., 2007; Wyres et al., 2013).

**Table 3:** **Atlanta invasive isolates containing composite ICE encoding macrolide resistance**.

**Strain**	**Year**	**Type**	**CC^a^**	**Size (bp)**	***Int* Family^b^**	**Tn*916*-like**	**Tn*916*-like I.S.^c^**	**Resistance genes^d^**	**Antibiogram^d^**
GA05245	1995	23F	242	70,161	Tn*5253*	Tn*2009*-like	*orf43/orf20*	*mef*(E) *mel tet*(M)	ERY TET
GA11304	1999	06A	156	82,623	Tn*5253*	Tn*6002*-like	*orf43/orf18* (R)	*erm*(B) *tet*(M) *cat*	ERY CLI TET CHL
GA11663	1999	19F	81	86,725	ICE*Sp*23FST81	Tn*2009*-like	*orf9/orf8*	*mef*(E) *mel tet*(M) *cat*	ERY TET CHL
GA13430	1999	19F	81	86,674	ICE*Sp*23FST81	Tn*2009*-like	*orf9/orf8*	*mef*(E) *mel tet*(M) *cat*	ERY TET CHL
GA13494	1999	14	156	86,593	ICE*Sp*23FST81	Tn*2009*-like	*orf9/orf8*	*mef*(E) *mel tet*(M) *cat*	ERY TET CHL
GA17227	2000	23F	242	67,131	Tn*5253*	Tn*2009*-like	*orf43/orf20*	*mef*(E) *mel tet*(M)	ERY TET
GA19101	2002	03	180	67,673	Tn*5253*	Tn*6002*-like	*orf20/orf18* (R)	*erm*(B) *tet*(M) *cat*	ERY CLI TET CHL
GA40563	2003	04	S	60,190	Tn*5253*	Tn*6002*-like	*orf20/orf18* (R)	*erm*(B) *tet*(M) *cat*	ERY CLI TET CHL
GA41301	2004	23F	242	67,191	Tn*5253*	Tn*2009*-like	*orf43/orf20*	*mef*(E) *mel tet*(M)	ERY TET
GA41565	2004	19A	81	88,320	ICE*Sp*23FST81	Tn*2009*-like	*orf9/orf8*	*mef*(E) *mel tet*(M) *cat*	ERY TET CHL
GA44378	2005	23F	81	81,138	ICE*Sp*23FST81	Tn*916-like*	*orf9/orf8*	*mef*(E) *mel tet*(M) *cat*	ERY TET CHL
GA47210	2006	15B	156	56,904	Tn*5253*	Tn*2009*-like	*orf20/orf18*	*mef*(E) *mel tet*(M)	ERY TET
GA47502	2006	19A	S	70,931	ICE*Sp*23FST81	Tn*3872*-like	*orf9/orf8*	*erm*(B) *tet*(M) *cat*	ERY CLI TET CHL
GA47597	2006	03	180	67,106	Tn*5253*	Tn*3872*-like	*orf20/orf18*	*erm*(B) *tet*(M) *cat*	ERY CLI TET CHL
GA60132	2010	06C	1390	71,603	Tn*5253*	Tn*6002*-like	*orf43/orf18* (R)	*erm*(B) *tet*(M)	ERY CLI TET

a *CC, clonal complex. Clonal complex was defined as the predicted founder of a complex as predicted by eBURST analyses (default settings)*.

b *Int, integrase gene. int_Tn5253_ (Accession no. EU351020); int_ICESp23FST81_, (Accession no. FR671403)*.

c *I.S., insertion site of Tn916-like element in the ICE. Orfs refer to the annotation of Tn5253. (R) indicates that the Tn916-like element is in the reverse orientation*.

d *Resistance to key antibiotics. ERY, erythromycin; CLI, clindamycin; TET, tetracycline; CHL, chloramphenicol. Minimum inhibitory concentrations for each isolate are provided as supporting information*.

*The horizontal line divides the pre-and post-PCV7 isolate*.

**Figure 6:**
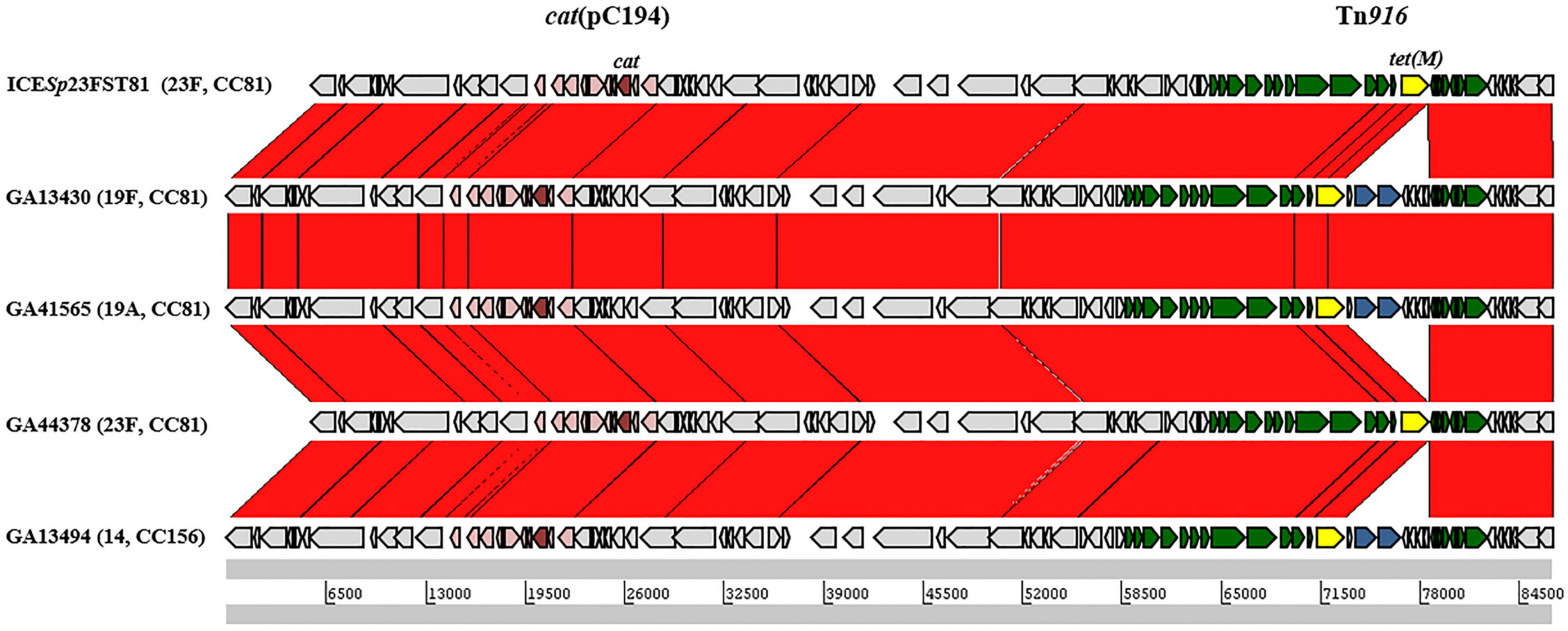
**Comparison of ICE*Sp*23FST81-like elements encoding macrolide resistance**. The location of the Tn*916*-like element in each is indicated by the Tn*5253* genes shown as arrows above each track. Green arrows, Tn*916*-genes; Blue arrows, *mef*(E) and *mel*; yellow arrows, *tet*(M); pink arrows, integrated plasmid pC194; red arrows, *cat*.

The five ICE*Sp*23FST81-like elements contained Tn*916*-like insertions, including a Tn*916*-like, a Tn*3872*-like and four Tn*2009*-like elements (Table 3). Each of the smaller elements was integrated between *orf9* and *orf8* of *Tn5253* and contained identical flanking and coupling sequences, indicating that each was derived from a single conjugation event and subsequent transfer of Mega-1 between Tn*916* and Tn*2009* (Figure 6). GA44378 containing Tn*916* integrated into the ICE*Sp*23FST81-like element was macrolide resistant due to an unlinked Mega-1.III insertion (Table 3; Table S2). ICE*Sp*23FST81 was originally described in a 23F, ST81 isolate (Croucher et al., 2009). Five of six ICE*Sp*23FST81 family elements were also in CC81, however only two were serotype 23F (Table 3). The others were serotype 19A (*n* = 1) and 19F (*n* = 2) and represent capsule switching from a PCV7 vaccine serotype to a non-vaccine serotype and a different vaccine serotype, respectively. This conclusion is supported by the close relationship between the isolates, all but one of which was also serotype 23F and CC81 (Figure 6). The lone exception was GA13494, a serotype 14, CC156 isolate that contained Tn*2009*, indicating horizontal transfer of the composite element between the clones (Figure 6). These were isolated over a 9-year period spanning the pre-PCV7 and post-PCV7 eras (1995–2004). This demonstrated clonal expansion and the long-term stability of the clone and the Tn*5253*-like element harboring Tn*2009*.

In the other composites identified, the Tn*916*-like element was inserted between *orf43* and *orf20* of Tn*5253*. Tn*3872* was inserted in this locus in GA47597, a serotype 3, CC180 isolate from 2006 (Table 3). Tn*3872* was also inserted downstream of *orf20* and contained the same flanking DNA sequences, however, the right junction was flanked by *orf18*. This indicated that GA47597, a serotype 3, CC180 isolate from 2006, also contained Tn*3872* which had a sequence identical to GA47502 flanking the left junction of Tn*3872* (Figure 6). Tn*6002* was also found flanked by *orf20* on the left and *orf18* on the right in two isolates (Table 3; Figure 5). Like GA47597, GA19101 was a serotype CC180 isolate suggesting a common integration event for a Tn*916*-like element (Figure 6). However, Tn*6002* in both of these isolates was integrated in the opposite orientation from Tn*2009* and Tn*3872* (Figure 6). This suggests separate conjugation events (one for each orientation) followed by homologous recombination of one junction to create a chimeric insertion site (i.e., *orf20/orf18*). In both isolates with Tn*3872*, the element was integrated into *orf20*/*orf18* in the same orientation as Tn*2009* in this location (Table 3). Again, Tn*2009* and Tn*6002* in this location had identical coupling sequences indicating a single event integration event of a Tn*916*-like element followed by acquisition of nested macrolide resistance elements by homologous recombination. Finally, *orf43/orf18* was identified as the site of insertion of Tn*6002* in four isolates (Table 2). Tn*5253* is also flanked by these genes and with conserved coupling sequences, indicating that each is descended from a single conjugation event followed by integration of the *erm*(B) element between *orf43* and *orf20*. These data suggest that *de novo* integration Tn*916*-like elements into pneumococcal chromosome backbones or ICE was infrequent relative to the rate of horizontal transfer by transformation and recombination.

## Discussion

The genome of *S. pneumoniae* is extremely plastic due to its natural competency and possession of numerous MGE. While transformation and conjugation are both involved in shaping the pneumococcal chromosome, the diversity of macrolide resistance elements, both independent and integrated into larger conjugative elements, raises the question of the relative role of each mechanism in the dissemination of macrolide resistance in pneumococci. Recombination events involving various macrolide resistance determinants were evidenced by an apparent interchangeability of Mega, the *erm*(B) element and Tn*917* in Tn*916*-like elements. The conversion of Tn*6002* and Tn*6003* (containing aminoglycoside resistance gene *aphA-3*) has been reported (Palmieri et al., 2012). These elements in turn were integrated into the chromosome backbone or were inserted into larger integrative and conjugative transposons. In this study, comparative genomics were used to assess the elements existing in a geographically defined location before and after the introduction of PCV7, to better understand the complex history of macrolide dissemination.

We identified Mega inserted in the four previously described locations in the pneumococcal chromosome backbone (Gay and Stephens, 2001) and inserted into *orf6* of Tn*916*-like elements. We also found a novel insertion site within an undefined transposon-like sequence similar to an element in *S. suis*. It is not clear if Mega integrated into the element before or after it made the cross-species horizontal transfer into the pneumococcus. Either way, it is an apparent example of pneumococci acquiring novel DNA from a non-pneumococcal streptococci, likely by conjugation event introducing the *S. suis* element into pneumococci or by Mega inserting into a novel locus in pneumococci. We also identified a transposon-like element most closely related to ICE*Sz*1 from *S. equi* subsp. *zooepidemicus*. This element was located in the Pneumococcal Pathogenicity Island 1 (PPI-1) and was associated with Mega. However, the possibility of *S. suis* and *S. equi* having acquired these elements from the pneumococcus cannot be disregarded. In fact, there is no data to suggest that these elements cannot flow equally in both directions.

PPI-1 appears to be a hotspot for recombination and/or integration of MGE. Not only was Mega inserted in PPI-1 but it appeared that integration of Mega at this location created, or retained, target sites for mobile elements. The Mega class IVb insertion contains Mega with no deletion of the target site. This may have been the original integrated structure. Subsequently, each flank of Mega-1.IVb may have been dislocated by insertion of additional MGE. Downstream of Mega in classes IVa and IVc was an insertion sequence, IS*Sm1*, originally identified in *S. mitis* (Denapaite et al., 2010). Interestingly, IS*Smi* precisely replaced the variable region of the pathogenicity island, which was a remnant of a Tn*5252*-like ICE (Brown et al., 2001). The variability at this locus and the presence of multiple integrated MGE-related sequences suggested this to be a hotspot for recombination or a common target site for conjugative elements. Upstream of Mega in class IVc was a large unidentified conjugative transposon-like sequence related most closely to a transposon-like sequence from *S. equi* subspecies *zooepidemicus*. The undefined element was integrated into the exact location within *rumA* as Mega and was integrated immediately upstream of Mega resulting in three MGEs linked in tandem: the unidentified transposon, Mega, and IS*Smi*1. Both undefined pneumococcal transposons are currently under investigation to identify their origin, understand where they came from, how they moved, what cargo they carry and their ability to transfer by conjugation from pneumococci to pneumococci and non-pneumococcal streptococci.

Closed circle intermediates of excised Tn*916*-like elements are formed by binding of semi-complementary coupling sequences creating a heteroduplex. This process produces novel coupling sequences with each integration event. Based on the observation that coupling sequences are altered upon integration, we used predicted coupling sequences as indicators of unique integration events. Mega and *erm*(B) often integrate in Tn*916*-like elements (Del Grosso et al., 2004, 2007, 2011). In the present study we identified many of the previously reported macrolide resistant Tn*916*-like elements in the Atlanta population including Tn*2009*, Tn*2010*, Tn*6002*, and Tn*3872*. The mechanism of conjugation of Tn*916* is well-understood. The Tn*916* integrase is promiscuous, that is, targeting sequences that are A-T rich, but not sequence specific (Rudy et al., 1997; Rocco and Churchward, 2006). Tn*916*-like transposons excise imperfectly from a donor strain to carry some flanking genomic bases and create a closed-circle intermediate with a mismatched junction (Caparon and Scott, 1989). The resulting overlapping region is the coupling sequence for subsequent integration, and thus each transposition event may create a new coupling sequence (Lu and Churchward, 1995). These facts and the observation by our laboratory and others that Tn*916*-like elements are found integrated within relatively few sites in the pneumococcus and with little variation in the coupling sequences, suggest that conjugation into and between pneumococci is relatively infrequent compared to transformation and recombination events.

ICE appear to be well-established in the pneumococcal population. They were present during a rapid emergence of macrolide resistant invasive pneumococcal disease during the early to mid-1990's (Wyres et al., 2013) in the United States. In addition, ICE have been reported in isolates from the 1960's (Wyres et al., 2013). ICE were identified in invasive and carriage pneumococcal isolates in Atlanta (Table 3). The ICE carrying the shorter macrolide resistance elements were of two types as has been reported previously (Roberts and Kreth, 2014). The first was a group of Tn*5253*-like elements containing different combinations of Mega, the *erm*(B) element, and Tn*917* also carrying *erm*(B). All of these elements were integrated into the same location in the pneumococcal chromosome backbone: the ribobiogenesis GTPase, *rbgA*. The second group was similar to ICE*Sp*23FST81 and were all inserted into the ribosome protein encoding gene *rpl*L. The conservation of insertion sites reflects the specificity of different integrases encoded on each element. Because of the specificity of these integrases, it is to be expected that they would integrate into the exact location repeatedly. Therefore, we can make no conclusions on the frequency of transfer of the element into or out of pneumococci.

Comparison of the sequence types and serotypes of the pneumococcal isolates carrying macrolide resistance determinants showed closely related isolates with variable macrolide resistance elements indicating horizontal transfer. For example, in Figure 6 GA41565 and GA44378 are both CC81, but the former is serotype 19A and the latter is 23F. These isolates cluster together in the whole genome analyses further indicating their similarity. Both contain ICE*Sp*23FST81-like ICE that are nearly identical and both have a Tn*916*-like element with no macrolide resistance determinant, while GA41565 contains Tn*2009*. This indicates the acquisition or loss of Mega by recombination. Analyses of the insertion sites suggested that conjugative insertion events of the elements into the pneumococcus are infrequent leading to the conclusion that these elements have been largely disseminated by lateral gene transfer through transformation and homologous recombination. Because homologous recombination requires homology between donor and host DNAs, gene flow between closely related isolates may occur more frequently than between distantly related clones. However, the presence of macrolide elements in composite transposons and the wide dissemination of composite transposons may aid in horizontal gene transfer between distant clones by creating 50–90 kb regions of homology that act as landing sites for the small nested elements carrying macrolide resistance determinants. These additional regions of homology may have aided in the dissemination of macrolide resistance not only because of the binding sites created by the composite elements, but also because of the multidrug resistance nature of the chimeric genetic elements. For example, macrolide resistance dissemination is aided by the non-macrolide antibiotic resistance determinants tetracycline and chloramphenicol due to soft selective sweeps of macrolide elements linked to *cat* and *tet*(M) genes carried on composite mobile elements.

The 7-valent Pneumococcal Conjugate Vaccine PCV7 was introduced into Atlanta in late 2000. We analyzed the genomic data in respect to the pre-vaccine and post-vaccine years. There appeared to be little effect on the types of elements harboring macrolide resistance determinants, however, the sequence types and serotypes harboring these elements were changed significantly. Some examples of this can be seen in the whole genome phylogenetic analyses where blue text indicated isolation in the pre-vaccine era and red indicated post-vaccine isolation (Figure 2). Note that in clonal complex 320, all isolates from before the vaccine were serotype 19F, a PCV7 serotype (Figure 2). After the vaccine was introduced serotype 19A, a non-PCV7 serotype, appeared. The shift to 19A was concomitant with the appearance of Tn*2010*, which was not detected in the pre-vaccine era. Interestingly, Tn*2010* was detected in only one chromosome backbone site and was not found in any ICE (Table 2). Likewise, CC2090 appears to have switched from the vaccine-associated serotype 6A to the non-vaccine serotype 19A (Figure 2). Every serotype 19A CC2090 strain was isolated after the introduction of the vaccine (Figure 2).

In conclusion, macrolide resistance dissemination in pneumococci has been greatly influenced by conjugation and transformation. Because Mega and Tn*916*-like element integration events appear infrequent, transformation and homologous recombination appear to be the primary means of horizontal transfer into the pneumococcus. With limited sites for insertion, the element has multiplied its capacity to disseminate throughout the pneumococcal population by co-opting the insertion sites of other elements. Thus, conjugation allows acquisition of DNA from more distantly related bacteria. Once in a pneumococcal genome, if beneficial and selected, the conjugated transposon is subsequently disseminated by transformation throughout the pneumococcal population.

### Conflict of interest statement

The authors declare that the research was conducted in the absence of any commercial or financial relationships that could be construed as a potential conflict of interest.
