# Supplementary material for: Composite mobile genetic elements disseminating macrolide resistance in Streptococcus pneumoniae
Source: Front Microbiol. 2015 Feb 9;6:26. doi: 10.3389/fmicb.2015.00026 (PMC4321634; doi:10.3389/fmicb.2015.00026)
Supplement: Supplementary file 1 [file DataSheet1.ZIP › Supplementary Material/Data File S1.DOCX]

>Tn5253_orf43

ATGGTTAATAATGTAGCTGTTAAAGTCTCAAACCTTTCAAAAGAATTTTTGTTAGGGCAAGACAAAACTGTCTCTATTTTGAAAGATGTTTCTTTATCTGTCAATTATGGGGAATTTATATCAATACTTGGTGTCAGTGGTTCTGGAAAGTCTACTTTGCTAAGTTGCTTATCAAGTTTATCTGAACCAACAAGTGGCGAAGTTGTTATCAATGGTATAAATCCATATACCTTAAAAGAAGGGAAGCTTGCTAAATTTAGAAGGCAAGATATTGCAATTATTTTTCAAAACTATAATCTGGTACCCGCTCTACCTGTACTAGAAAATGTTACACTTCCTCTGAGACTCTCAGGAAAGAGTGTTGATAGCAATAAAGTAAAAAAAATGTTGGATAGTTTGAATTTTAAAGCAGAACTATCATCATTAGTTGCTACCTTATCAGGTGGAGAACAGCAAAAAGTGGCTATTACTCGTGCAATAATAGCTGATAGTAAAATTATTTTTGCTGATGAGCCAACAGGAGCTCTGGACAGCGTCTCAAGAAAACTTATTTTTGAAACACTTCGTAATTTAGCGAGTCAAGGAAAATGTGTGTTTATGGTCACTCATGATATTGAGTTGGCTTCAAAAACTGACAGAGCACTCATTTTAAAAGACGGAAAAATATCTCAACAAATTATTAAACCTTCAGCTGATGAGCTCTACCAAGCACTTGAAAGTAGTAAAGATTAA

>Tn5253_orf79

ATGTATTATGTAACTAAAACAAATTCAAAAGGGCAACCCTTATATCAAGTGGTTGAAAAGTACAAAGATCCACTAACAGGAAAGTGGAAATCAGTAACTGTAAGTTACACTAGGAATACTAGTAGGGCTAGGAAACAAGCTGAAAGAGAGGTTCTTGATAAGATAGATAGACTAACTACTTCATTTGAAAGTCAGTTTAGCCCTGAACTGATTACAACATTTGGAGAGTTAAAAGAAAATTGGTTTCAGACTTGGTGTGTCTCTGTTAAACCACAAACAATTCAGAGAGAACTACTGGTGATGAAGCGTCTTGGGAAAATTATTGGAGATGATTTTTTATTAGACAGGATTACTCCACTTCTGATGAAAAAAAGTCTCAATAAATATTTAGAAATATATGATGCATCACCTTCAACAATGACTCATATAAAAAGTACTTGTAATAAGATTTTTAATCATGGTGTGTTATATAATGTCATTAAGTTCTCTCCTATGACTGCGGTAAAACTAGATATTTCACTAGAGAAAAGGCGTAAAGCAAAAGAAAGACATGATTCTAAATTTCTAGAAATCCATGAATTACACGCATTTTTTGATGTATTAAGCCAATGCAGAAATGCAAACTATTATGATCTTGCTATAGTATTGTTGCTTACAGGTATTCGAATTAGTGAAGCTGCTTTTTTACCATCAGATATTGATTTTGAAAAAGGAATCTTGCATATTGATAAGGCACTTCAATATCATTGTTTAAAAGTTGAACAATTTCACTTTGATACAACTAAAACACTCAATTCAATTAGAGAAGTAGCTTTGCCTGAAGCTGCAAGCGAAGCTATTAAAAGGACAATACAGAGAAATAAAGAGTTTGATGCTTATATGGAGAAACATCCCTGTCCTGCTTTTACACATTCTGAAAGTGTATTTAGAACAGAATACGGCTCTCCAATAACATCAAGCACTTTTCGTCAAATTTTGAAACGAATAGAAGGAAAATTATTGACAAATTGTTTAAGTGACTATGGTTTTAAGTGGGTAAAACATGTTACTCCCCATTCGTTTAGGCATATGCATATTAGTTACCTTCAAAGTAATGAGATGCACATAGCAGTGAAAGATATTATGACTAGAGTAGGACACGCTAACTTTGAGACAACAATGGGCTATACACATAATATAAATCGTTCACAAGAAAATACTGTAAAAGCCTTAAATCAATTTGTAGAAAATCACAATTTCCATTTTGAAGAATTGAAAAGTTATACCTGTAAATATTCCAGAATAATTGAAAAATTCATTGAAACTAGTGATAATAGCAATAAAGTAGAATTAAGTGTCGATGAGTTCAAAGACTTGTTACATCTTAGTCCACGTTACTCACCTAAAAATATTATTTCGAATTTACTATTAAAAATCAAAAAAGATATTGTCAAATACCACCCACAGTTTGATATAAAGATTGTGAAATCAAGTGAGAATCAAATCAGAGGTTTTTCCATTGCATGGTAG

>Tn5253_orf9

atggttgataaaagagagaaactgatgaactctttcaatcagtatggttttttaacttttaaacaagtaatagatgaaaatttacactacaaaaccttattaaaaatggttgcagaaggaaaaatcgatgctgaagaaaaagggttatatcgcttacctgatatttatttagatgagtggtttgtccttcagtatcgatttccaaagggaatcttttctttggagacagcactttggttacatggtttatctttgactatcccttttaatatgacgatgagttttccttatggtacgaataccaaaaacattaaggaagcagatatatgtcctattattttacgctctcactatagtgaaggaattattgaaattgagcgccttcctggtcaatttattaaagtttatgaagttgaacgagttttggttgaatgtctaagaccagttcatcaggtggatcttcaaattattgcaccagcgtttaaaaaatattttcaacaaaataaaattcatttacacaaattattttattatgcccagctatttaaagtaactgataagttacaatcttatacggaggtactatcttaa

>Tn916orf13

atgatgaaatttagaaaaaatcagaataaagaaaaacagataccaaaggaaaagaaacctcgtgtctataaggtcaatcctcataaaaaggttgtgattgccttgtgggtacttttagggcttagtttcagctttgcgatattcaagcactttacagctatagatactcatactattcacgaaacaactatcatagaaaaggaatacgttgatactcatcatgtagaaaattttgtagagaactttgcgaaagtctactattcatgggagcaatccgataagtccattgataatcgaatggaaagtctaaaaggctatctgacagatgaacttcaagctctcaatgttgatacagtacgcaaagatattcctgtatcgtcttctgtaagaggatttcagatatggacggtagagccaactggcgacaatgagtttaatgtaacctacagtgtagaccagctcattacagagggagaaaatacaaagaccgtccactctgcttatatagtgagtgtctatgtagatggttctggaaatatggtactggttaagaatccgaccattaccaacatacctaagaaatcaagttataaaccaaaagccattgaaagtgaggggacggttgattccattacaaccaatgaaatcaatgagtttttaacgacgttcttcaagctctatcctacagcgacagccagtgaactttcctactatgtgaatgacgggatattaaaaccaatcggaaaagagtacatctttcaagaactggtaaatcctattcacaatcgtaaggataatcaagtcacggtatcgctgacagtggagtatatcgaccagcagaccaaagcaacgcaggtatctcaatttgatttggtacttgaaaagaacgggagtaattggaagattatagaataa

>tetM

atgaaaattattaatattggagttttagctcatgttgatgcaggaaaaactaccttaacagaaagcttattatataacagtggagcgattacagaattaggaagcgtggacaaaggtacaacgaggacggataatacgcttttagaacgtcagagaggaattacaattcagacaggaataacctcttttcagtgggaaaatacgaaggtgaacatcatagacacgccaggacatatggatttcttagcagaagtatatcgttcattatcagttttagatggggcaattctactgatttctgcaaaagatggcgtacaagcacaaactcgtatattatttcatgcacttaggaaaatggggattcccacaatcttttttatcaataagattgaccaaaatggaattgatttatcaacggtttatcaggatattaaagagaaactttctgccgaaattgtaatcaaacagaaggtagaactgtatcctaatatgtgtgtgacgaactttaccgaatctgaacaatgggatacggtaatagagggaaacgatgaccttttagagaaatatatgtccggtaaatcattagaagcattggaactcgaacaagaggaaagcataagatttcagaattgttctctgttccctctttatcatggaagtgcaaaaagtaatatagggattgataaccttatagaagtgattacgaataaattttattcatcaacacatcgaggtcagtctgaactttgcggaaaagttttcaaaattgagtattcggaaaaaagacagcgtcttgcatatatacgtctttatagtggcgtactgcatttgcgagattcggttagaatatcggaaaaggaaaaaataaaaattacagaaatgtatacttcaataaatggtgaattatgtaaaatcgataaggcttattccggggaaattgttattttgcagaatgagtttttgaagttaaatagtgttcttggagatacaaagctattgccacagagagagagaattgaaaatcccctccctctgctgcaaacgactgttgaaccgagcaaacctcaacaaagggaaatgttacttgatgcacttttagaaatctccgacagtgacccgcttctgcgatattatgtggattctgcgacacatgaaatcatactttctttcttagggaaagtacaaatggaagtgacttgtgctctgctgcaagaaaagtatcatgtggagatagaaataaaagagcctacagtcatttatatggaaagaccgttaaaaaaagcagagtataccattcacatcgaagttccaccgaatcctttctgggcttccattggtctatctgtagcacagcttccattagggagcggagtacagtatgagagctcggtttctcttggatacttaaatcaatcgtttcaaaatgcagttatggaggggatacgctatggctgtgaacaaggattgtatggttggaatgtgacggactgtaaaatctgttttaagtatggcttatactatagccctgttagtaccccagcagattttcggatgcttgctcctattgtattggaacaagtcttaaaaaaagctggaacagaattgttagagccatatcttagttttaaaatttatgcgccacaggaatatctttcacgagcatacaacgatgctcctaaatattgtgcgaacatcgtagacactcaattgaaaaataatgaggtcattcttagtggagaaatccctgctcggtgtattcaagaatatcgtagtgatttaactttctttacaaatggacgtagtgtttgtttaacagagttaaaagggtaccatgttactaccggtgaacctgtttgccagccccgtcgtccaaatagtcggatagataaagtacgatatatgttcaataaaataacttag

>Tn916_orf20

ttggagggatttttactgaatgaacaaacttggttacagcatttaaaagaaaaacgcttggcttatggactatctcaaaaccgtttagctgttgcgactggtattacaaggcagtatctaagcgatattgaaacaggaaaagtcaagccatcagaggatttacagcagtccctttgggaagctctggaacgcttcaatcccgacgctccccttgaaatgctgtttgattatgtaaggattcgctttccgacaacagacgtacagcaggtggtcgaaaacatcttacaactgaaactgtcctattttcttcatgaggactatggtttctattcttattcagagcattatgctttaggcgacatattcgtcctttgctcccatgaactggacaaaggagttctggtggaattgaaaggtcgtgggtgcagacaatttgaaagctatcttctggcacaacaaagaagctggtatgagttctttatggacgttttggtggctggcggtgtgatgaaacgccttgaccttgccattaacgataagacagggattttgaatatccctgtactcactgaaaagtgccaacaggaagaatgtatctccgtcttccgcagttttaaaagctatcgcagtggcgaactggtacgcaaagaggaaaaggaatgtatgggaaacaccctctatatcggttcattacaaagtgaagtttatttctgtatctatgaaaaggactacgagcagtacaagaaaaatgatattcccattgaagacgcagaagtaaaaaaccgttttgagattcgattgaaaaatgagcgtgcctattatgcagtccgtgatttactcgtctatgacaatccagagcataccgcctttaaaattatcaatcggtatatccgttttgtagataaagacgattccaaacctcgttctgattggaaactgaatgaagaatgggcttggtttattgggaacaatcgtgaacgattaaaactaaccacaaaaccagagccttactccttccaaaggacgctgaactggctatctcatcaagttgccccgaccttaaaggttgcgattaaacttgatgaaatcaaccagacgcaggttgtaaaagacattctcgaccatgcgaaactgacagaccgacacaagcagattttgaagcaacagtcagtaaaagaacaggacgtgataacaacaaaaaaataa

>Tn5253_orf1

atgacaaaagaattacaatcatcacgctatattgtcatttcatttttagtacgtgaaatgggaattgacattgttgaagccatctctcttatggctgaattagaaaaaagtggcttggttcgattggaatcaagtggagatttaatactcaaagaacttggaggagcgctatga

>cat

atgaactttaataaaattgatttagacaattggaagagaaaagagatatttaatcattatttgaaccaacaaacgacttttagtataaccacagaaattgatattagtgttttataccgaaacataaaacaagaaggatataaattttaccctgcatttattttcttagtgacaagggtgataaactcaaatacagcttttagaactggttacaatagcgacggagagttaggttattgggataagttagagccactttatacaatttttgatggtgtatctaaaacattctctggtatttggactcctgtaaagaatgacttcaaagagttttatgatttatacctttctgatgtagagaaatataatggttcggggaaattgtttcccaaaacacctatacctgaaaatgctttttctctttctattattccatggacttcatttactgggtttaacttaaatatcaataataatagtaattaccttctacccattattacagcaggaaaattcattaataaaggtaattcaatatatttaccgctatctttacaggtacatcattctgtttgtgatggttatcatgcaggattgtttatgaactctattcaggaattgtcagataggcctaatgactggcttttataa

>Tn916_orf23

atggaacttaaatttgtgattcccaacatggaaaaaacattcggcaatttagaatttgctggcgaggataaagtcgttcagcgaagaatcaacggacggctaactgtcttatcaagaagctataatctctattctgatgttcaaagagcagatgatattgtggtggtgcttcctgctgaagctggcgaaaaacatttcggctttgaggaacgtgtgaagttagtcaatccacgtattaccgcagagggctacaaaatcggcactcgtggttttacaaattaccttttacatgctgacgacatgataaaagaataa

>Tn916_orf24_end

aaaatagcataaaaatctagttatccgcataaaaactggacttatcacactttatcaaggtcaaaaccactcaatttactactaatttactacttatgaatgagctttgatacgacgatttatccttgaaaagtgaagatataaagatacttccaataaaatttgaatatttaataggtagacacttcaaaaaatgaggtgtctatttttttacccgattttgaaaggaagtgaacttatgaaaacaaaaaatcaagaatcaaaaggtcgttccccactctttaagaccatcaaacattcattcagccaataaaaaagaaaggataggtaaaaatatggaacttaaatttgtgattcccaacatggaaaaaacattcggcaatttagaatttgctggcgaggataaagtcgttcagcgaagaatcaacggacggctaactgtcttatcaagaagctataatctctattctgatgttcaaagagcagatgatattgtggtggtgcttcctgctgaagctggcgaaaaacatttcggctttgaggaacgtgtgaagttagtcaatccacgtattaccgcagagggctacaaaatcggcactcgtggttttacaaattaccttttacatgctgacgacatgataaaagaataaagaaagagaggaaaaatgatgagattagcaaatggcattgtattagataaagacacgacttttggagaattgaaattctctgctctacgtcgtgaagtgagaatccaaaatgaagacgggtcggtttcagatgaaatcaaggaacgtacctatgacttaaaatccaaaggacaaggacgcatgattcaagtaagtattcctgccagcgtgcctttgaaagagtttgattataacgcacgggtggaacttatcaatcccattgcggacaccgttgctactgccacctatcaaggagcagatgttgactggtatatcaaggcagacgatattgtgctgacaaaggattctagttcattcaaagctcaaccac

>Tn916_int_xis_end

gggctattagaatgagtgaaaatggttatgcttatcaaaccatcaataactacaaacgttctttaaaggcttcattctatattgctatacaagatgattgtgttcggaagaatccatttgactttcaactgaaagcagttcttgatgatgatactgtccctaagaccgtactaacagaagaacaggaagaaaaactgttagcctttgcaaaagctgataaaacctacagcaaaaattatgatgaaattctgatactcttaaaaacaggtcttcgtatttcagagtttggtggtttgacacttccagatttagattttgagaatcgtcttgtcaatatagaccatcagctattgagagatactgaaattgggtactacattgaaacaccaaagaccaaaagtggcgaacgtcaagttcctatggttgaagaagcctatcaagcatttaagcgagtgttagcgaatcgaaagaatgataagcgtgttgagattgatggatatagtgatttcctctttcttaatagaaagaactatccaaaagtggcaagtgattacaacggcatgatgaaaggtcttgttaagaaatacaataagtataacgaggataaattgccacacatcactccacatagtttgcgacatacattctgtaccaactatgcaaatgcaggaatgaatccaaaggcattacagtacattatgggacatgctaatatagccatgacgctgaactattacgcacatgcaacattcgattctgcaatggcagaaatgaaacgcttgaataaagagaagcaacaggagcgtcttgttgcttagtagtacaaatgaatttactacttatttaccacttctgacagctaagacatgaggaaatatgcaaagaaacgtgaagtatcttcctacagtaaaaatactcgaaagcacatagaataaggctttacgagcatttaagaaaatataaaaagataattagaaatttatactttgttt

>ICESpn11876_end1

tttttggtaggaaactaaattaatttatcagtttcta

>ICESpn11876_end2

tttttggcaggaaaccaaatcaatttatcagtttcta

>Tn5253_attL

tgttcaaactatagtaaaataaaataggggatctaaatccttgctacgaaaggaaaaaaactcaatggctactattcaatggtt

>Tn5253_attP

tgtttagacaatagtaaaataaaataggggctctaaacccttgctacgaaaggaaaaaaactcaatggctactattcaatggtt

>Tn916orf2_Tn5253orf20_junction

atgaaaagaataattccagtttatatattccaacaagtaaatgtcttattggtatccctatacttactgaaattgctttgtattagtgagttaaccatactacagattctctattgtgcgtcgctcatttcttttttatggatgtatggtcaacgaaaacaagttgtcaaggtcaatatgaaaactaggatgaaatggcttggtattggattcgttagcctactgattataaatctatgttttagcctaatccatgctcaaggaactacgaatcaagcaaacttaattggccttcaacatcaagtcccttggttttcatttttattgttattaatcaatgcgagtatggttgaagaatttctgtatcgagaaattttatggaacttggttagaaaattagatattcgagttgctttgacaagtattttatttgtcttagctcatcatccaggaaccattttagcttggtgtttgtatgtttcacttgggatgtttttagggcttgtgcgctacaaatcggacttatggggcagtatggggctacatttggtgtggaacctatcagtctatgtcttatttttctttaataatagctggtctgtcttaagcatggattaattttgaaataaacaaagtataaatttctaattatctttttatattttcttaaatgctcgtaaagccttattctatgtgctttcgagtatttttactgtaggaagatacttcacgtttctttgcatatttcctcatgtcttagctgtcagaagtggtaaataagtagtaaattcatttgtactactaagcaacaagacgctcctgttgcttctctttattcaagcgtttcatttctgccattgcagaatcgaatgttgcatgtgcgtaatagttcagcgtcatggctatattagcatgtcccataatgtactgtaatgcctttggattcattcctgcatttgcatagttggtacagaatgtatgtcgcaaactatgtggagtgatgtgtggcaatttatcctcgttatacttattgtatttcttaacaagacctttcatcatgccgttgtaatcacttgccacttttggatagttctttctattaagaaagaggaaatcactatatccatcaatctcaacacgcttatcattctttcgattcgctaacactcgcttaaatgcttgataggcttcttcaaccataggaacttgacgttcgccacttttggtctttggtgtttcaatgtagtacccaatttcagtatctctcaatagctgatggtctatattgacaagacgattctcaaaatctaaatctggaagtgtcaaaccaccaaactctgaaatacgaagacctgtttttaagagtatcagaatttcatcataatttttgctgtaggttttatcagcttttgcaaaggctaacagtttttcttcctgttcttctgttagtacggtcttagggacagtatcatcatcaagaactgctttcagttgaaagtcaaatggattcttccgaacacaatcatcttgtatagcaatatagaatgaagcctttaaagaacgtttgtagttattgatggtttgataagcataaccattttcactcattctaatagcccattctttagcgtctgatggcttaatactgtcaatacttcttacacctaacttgtctttcttcaaaatatccataagatatttgcgtccagtttcagtgttttttctaacctttggtctttgagcgttctgttttgcgtaaagctggcagagtgtcattttctttcc

>Tn5397_left_end_Tn916_0rf23

gatggaaatgtaccatcaagacacctgctaagaaccgcataacagcaggcaaacaacttcatatcaaaattggaataaatacatataaagagccatttgatttttcatatcaagtggtttttgttatgtatatggagcaagacgcttactaattccattatgggaaataggagcgtctatttttttacccgattttgaaaggaagtgaacttatgaaaacaaaaaaatcaaggtcgttccccactcttagaccatcaaacaaattagattcatgaaaccattaaacagaaaggataggtaaaaatatggaacttaaatttgt

>ICESpn700669_81094bp_insertion site_element_removed

tctgaagaatagctcatcatctcaattaatttgtcctttgtcatttcagaaactgaatga

caagatacctcgatgccatagttttggaagaagtctaaaagaagttgatttcttcggcta

tttttacttagatagagatcaatcataggagacctcccaaagattcggttccatttgata

ttctgagacgattaaggaatctaataaatttgcgaagttaatcggtttcttgtcttcatc

ataagcttttacagttacttgggttgtaagtattccctcttttccctcggctcgatagcc

ttgtccatataaaacaaaaacgagattttgatgatcatctacaaaggcatcaaccccatt

ctttatgtcttgactttcaaggaattccataacgttttgaagataggattcgtaaaatag

tgggtagttatgttttttatggtaatcatctaaaaatgtcacttcaaactcacatggaga

gtaattttgactttgaacagcctaaaagtgccatcaaatttgaattggaataaatcaaat

aaatagccccatcctcatcaatccaacctttgctcaaagacaactccaaccgatctttta

aaactgagtaaaccaccttaacctccagtttcatattcttataccgttcactctcaaata

aaagtttggggagcttataataacgctctgatgtctgatattgattagcggtaatacgct

tcattattgtccctccaagactaaaattccaacatttccaaattcatcaaatcggattaa

acctacttgttccatttcatcaactaactgagttgcttttacaatatcaactcccatgat

agtcatgagatgactcttcacgaattgacgtgatgactgtccttccttttgcataattac

ctccgaaacacaaaaaaaggggtagacaatctagtgtctacccccgaaagtttattaaaa

caaaaatcctgccaaagaatttttggcaggaaaccaaatcnnnnnnnaaatgtgttcacg

attctaaaaggctgatactatagtattccgaattctaattggtatatgcctcttatttaa

gagtaactgaagctccagcttcttccaatttagctttgatttcttcagcttctgcagttg

caacgccttctttaacaagtgctggtgcaccgtcaacaagttctttagcttctttaagac

caagaccagtgatttcacgtacaactttgataacgccaacttttttgtcgcctgcagatg

tcaattcaacgtcgaatgaatctttagcagcaccagcatcagctgcatcagctgcagcaa

cagctacaggagcagctgcagttacaccaaattcttcttcgatagcttttacaaggtcgt

tcaattcaaggattgaagcttctttaatttcagcaataatgttttcaatgttcaatgcca

ttgttatttcctccaaataagttttaaattttataatagtttttttcgtagctaggctac

gctgtgtagcttaagattaagccgcgtcttctttgctttctgcaaccgctttgactgcaa

gagcaacgttgcgcactggcgcttgaagtacagaaaggagcatagaaagaagtccttcgc

ggtttggaagagttgcaagtgcaagaatctcttctttagatgcgacagcgccttcgattg

caccacctttaatttcaagtgcttcagcgtttttagaaaagtcattcaagattttcgctg

gtgcgataacatcttcattagaaaatgctactgcagatggtccaacaaatacagatgcaa

gatcttcaagaccagctttttcagctgcacgacgcaagattgagtttttaataactttat

actcaacttcgcttccacgaagctcacgacgaagaactgtatcttgctcaactgtcaaac

cacgagcgtctacaacgacgatagatgcagcagctttcattttttcagctactacgtcaa

ctagttccgcttttttagcaataattg

>Tn5252_RJ_

TTCTCAGAATAAAACGAAGAATGTGAATCTTCGACATATAATATTGACACAAACTCAACA

CCAAAAAGTTTTATTGATGATTATTGCACATATAACAATGTGAATAATTAAAATCTAAAC

CAATAAAAGTCCTTGAATTTCAAGGAATTTAAGTATTTATTCTACTTCACAATGTTTTAT

TATTTTAAATCTTCTAGACCAACCATTGAATAGTAGCCATTGAGTTTTTTTCCTTCTCGT

AGCAAGGATTTAGATCCCCTATTTTATTTTACTATAGTTTGAACATAGAGAGTTTTCAAA

TGAATGATGTCCAAATAGTTCTTGTTTTTATGAC

>Tn525_LJ

AATTTTAAATTCTCCTGCACTTGTCGACGCGCTTTAGACATGTGACCAGGAACCATTGAA

TAGTAGCCATTGAGTTTTTTTCCTTTCGTAGCAAGGGTTTAGAGCCCCTATTTTATTTTA

CTATTGTCTAAACACCAAGCGAACACCAAAACTACCATGCAATGGAAAAACCTCTGATTT

GATTCTCACTTGATTTCACAATCTTTATATCAAACTGTGGGTGGTATTTGACAATATCTT

TTTTGATTTTTAATAGTAAATTCGAAATAATATTTTAGGTGAGTAACGTGGACTAAGATG

TAACAAGTCTTTGAACTCATCGA
