# Supplementary material for: Composite mobile genetic elements disseminating macrolide resistance in Streptococcus pneumoniae
Source: Front Microbiol. 2015 Feb 9;6:26. doi: 10.3389/fmicb.2015.00026 (PMC4321634; doi:10.3389/fmicb.2015.00026)
Supplement: Supplementary file 1 [file DataSheet1.ZIP › Supplementary Material/Data File S2.DOCX]

>EU-NP01/Mega-1.III

CATGTTGAGGCGGTAAGTTTGCTAGTCAAGGAGTAAAACGACGAAGATTAGCATTTACTTCCGCCCATGCGA

TAGCTGTCCGTGATTGACAAGTGCTAGCACGCAGACAGAACGGAGATAGCGAACCGCTGAGTGTGTCGCTCT

GCTCGTAAAAGCTTAGAAACCTTTGAACGAAAGGGATAATGAAAGCCTTGATTGCAAGGCTTTTTGCTTTAT

GGTGGGTAAGTATCAGAGTGAGAAAATTTTTGGAATGAGTAGAAGTGATAGCTAGAAATTATCAGTTTCTA

TTTCCATTTACCCTGTGGGTACGTGTTTGTTTCCATTGACAAGGAGTTTGTGGGAATAGAAATGT

ACCCACCTTGTTTGAATCAAGTGAAGTGTAGTTGAAGGAAATCTGTTGAAAGCAATACTTCATTTTACCG

AATAAGTAATAATTTAGGCAACTTCAAATCGATTAAAAAAAACTATTTTAAAGGTTAAGAGTAGACAAAA

ATTGTCCACTCTTTTTTGCAAACTCAATTTATCAATAAATGAAATGAGGGAATGTAAAATG

AAATATTTTGAGGTTGAGTTAGAAAATCCTGATGAATTTTTAAAACTACAAACAGAAGATTTTGTGAAAG

CTAATCGCTTGCTACTAAGGAAGATAATCCAGAGCGTTACAGTCTATGAAGAAAACTTCGTCATATCCTT

TAAATCTGGCATCGAATTGGAAGTATGAGTCTCATTCCATAACTTTTATATTGAACATATCATCTTTTTGTG

TTATACTATAAATTGATATAAACAAAGATGTAGGAGGAACCGAAACTATGACAGCCTCAATGCGTTTAAGAT

AAGCTGGCAATAAAAAAAGCAGAATCTATACCCGATGATAGGCTTTTTTGTTGTGCTTATTTATACGATATT

GAGCATTCATTAGTTACGGTGAGGATATTGGTTATTTAACTATACCTTTATTTAACTATGTCTTTAATATGA

ATGTTTCCAAATTGTATGTATGCAGACCAAAAGCCACATTGTGGGGTTTGGCCTGCATTTTTTTTGCCTA

GAATGCTATTCAAAATAGAAATTCAAGCAAAATAATATGCAGGAGATAATATAAATGGAAAAATACAACAAT

TGGAAACGAAAATTTTATGCAATATGGGCAGGGCAAGCAGTATCATTAATCACTAGTGCCATCCTGCAAATG

GCGATTATTTTTTACCTTACAGAAAAAACAGGATCTGCGATGGTCTTGTCTATGGCTTCATTAGTAGGTTTT

TTACCCTATGCGATTTTGGGACCTGCCATTGGTGTGCTAGTGGATCGTCATGATAGGAAGAAGATAATGATT

GGTGCCGATTTAATTATCGCAGCAGCTGGTGCAGTGCTTGCTATTGTTGCATTCTGTATGGAGCTACCTGTC

TGGATGATTATGATAGTATTGTTTATCCGTAGCATTGGAACAGCTTTTCATACCCCAGCACTCAATGCGGTT

ACACCACTTTTAGTACCAGAAGAACAGCTAACGAAATGCGCAGGCTATAGTCAGTCTTTGCAGTCTATAAGC

TATATTGTTAGTCCGGCAGTTGCAGCACTCTTATACTCCGTTTGGGATTTAAATGCTATTATTGCCATCGAC

GTATTGGGTGCTGTGATTGCATCTATTACGGTAGCAATTGTACGTATACCTAAGCTGGGTAATCAAGTGCAA

AGTTTAGAACCAAATTTCATAAGGGAGATGAAAGAAGGAGTTGTGGTTCTGAGACAAAACAAAGGATTGTTT

GCCTTATTACTCTTAGGAACACTATATACTTTTGTTTATATGCCAATCAATGCACTATTTCCTTTAATAAGC

ATGGAACACTTTAATGGAACGCCTGTGCATATTTCTATTACGGAAATTTCCTTTGCATTTGGGATGCTAGCA

GGAGGCTTATTATTAGGAAGATTAGGGGGCTTCGAAAAGCATGTATTACTAATAACAAGTTCATTTTTTATA

ATGGGGACCAGTTTAGCCGTTTCGGGAATACTTCCTCCAAATGGATTTGTAATATTCGTAGTTTGCTGTGCA

ATAATGGGGCTTTCGGTGCCATTTTATAGCGGTGTGCAAACAGCTCTTTTTCAGGAGAAAATTAAGCCTGAA

TATTTAGGACGTGTATTTTCTTTGATCGGAAGTATCATGTCACTTGCTATGCCAATTGGGTTAATTCTTTCT

GGATTCTTTGCTGATAAAATCGGTGTAAATCATTGGTTTTTACTATCAGGTATTTTAATTATTGGCATTGCT

ATAGTTTGCCAAATGATAACTGAGGTTAGAAAATTAGATTTAAAATAAACAATATTGGAGGAATATTTATGT

ATCTTATTTTCATGTAACTCTTCCTGCTAAAATCGCAGGGTTTTCCCTGCATACAAGCAAATGAAAGCATGC

GATTATAGACAGGAGGAAATGTTATGGAATTAATATT

AAAAGCAAAAGACATTCGTGTGGAATTCAAAGGACGCGATGTTTTAGATATAAATGAATTAGAAGTATATGA

TTATGACCGTATTGGTTTAGTAGGAGCAAATGGTGCTGGAAAAAGCACTTTACTCAGGGTACTTTTAGGAGA

ATTAACTCCCCCAGGATGTAAAATGAATCGTCTGGGTGAACTTGCCTATATTCCCCAGTTGGACGAAGTAAC

TCTGCAGGAGGAAAAAGATTTTGCACTTGTAGGCAAGCTAGGTGTTGAGCAATTAAATATACAGACTATGAG

CGGTGGTGAAGAAACAAGGCTTAAAATAGCACAGGCCTTATCGGCACAGGTTCATGGTATTTTAGCGGATGA

ACCTACGAGCCATTTAGACCGTGAAGGAATTGATTTTCTAATAGGACAGCTAAAATATTTTACAGGTGCACT

GTTAGTTATTAGCCATGACCGCTATTTTCTTGATGAAATAGTAGATAAAATATGGGAACTGAAAGATGGCAA

AATCACTGAGTATTGGGGAAACTATTCTGATTATCTTCGTCAGAAAGAGGAAGAACGTAAGAGCCAAGCTGC

AGAATACGAACAATTTATTGCGGAACGTGCCCGATTGGAAAGGGCTGCGGAGGAAAAGCGAAAACAGGCTCG

TAAAATAGAACAGAAGGCAAAAGGTTCTTCAAAGAAAAAAAGTACTGAAGACGGAGGGCGTTTAGCTCATCA

AAAATCAATAGGAAGTAAGGAAAAAAAGATGTATAATGCTGCTAAAACCCTAGAGCACAGGATTGCGGCCTT

AGGAAAAGTAGAAGCTCCGGAAGGCATTCGCAGAATTCGTTTCAGGCAAAGTAAAGCATTGGAGCTCCATAA

TCCATACCCTATAGTCGGTGCAGAAATTAATAAAGTATTTGGGGATAAGGCTCTGTTTGAAAATGCATCTTT

TCAAATTCCGTTAGGAGCAAAAGTGGCGTTAACTGGTGGTAATGGAATCGGAAAAACAACTTTAATCCAAAT

GATCTTAAACCATGAAGAAGGAATTTCTATTTCGCCTAAGGCAAAAATAGGTTACTTTGCACAGAATGGTTA

CAAGTACAACAGTAATCAGAATGTTATGGAGTTTATGCAGAAGGATTGTGACTACAATATATCAGAAATTCG

TTCAGTGCTAGCATCTATGGGGTTCAAACAGAACGATATTGGAAAAAGTTTATCTGTTTTAAGCGGTGGAGA

AATTATAAAATTGTTGCTTGCTAAAATGCTCATGGGTAGATATAACATCCTAATAATGGATGAACCCAGTAA

CTTCCTTGACATACCAAGTTTAGAGGCTTTGGAAATACTAATGAAGGAGTACACCGGAACTATCGTGTTTAT

CACCCACGATAAACGATTACTCGAAAATGTAGCAGATGTAGTTTATGAAATTAGAGATAAGAAAATAAATCT

GAAACATTAAATTTAAGGTAGTCGCTGGTCAGTATAGTCTGTTCTGGTTGGCGACTCCATTGTTAAAGAGTA

TAAAGACTTTAGATTTTATGAATATTAAAAATAGGAACAGTCAATTGAACTGCTCCTATTTTTCTGCTAAAT

ATATTGTAGTTTTCTTATATGTATAATGATAGATTAGCGGATTCTCATCTACGGTACTTACTTCAAATATGA

AGAAGTGATCGCGGTTATCTCTGGACTTTTCCTTATTGAGGACAAAGTAATTCTTACGTGAAGTCGCCATTG

TTTTTAGGATATCATCAGTTAGGAAGGTCAATGGAATATTCATGTTAGAGTAGCGGTAGAAGTCACGTTCAA

AATCTTGGTAGCTCTCGCTATAATAGTCCATTTGTAGGTGATTACGCTGAAACTCAAGCTGATTCATAGAGC

ACCTCCTCGACAAGTTCAATACTAATAATGTCTTTTAATTTCAAATTGATGTGACCTGTTGTAGTTTTTATC

AAAATGAAATCTTTGGTCAGACTTGGTATTGTTCCAGTGTAGGAAACACGCTTGTTTTTTTCAATCACTTGA

ATGCGTGTGCGTAGCTGCCCGGCGTATACTTGACTGAGGAGTAATAATTTCTTCTCTAGTGATAAGTCAGAC

ATGTACGTTACTTTGTTTGTATCATCAGAGAGTGCTGATGCATGTTCAGATAGGAAAAAGCCCATCCATTTT

TGCATCTTTGTATCCTGGTACTCTCTTGCTGATTGAAATGGTAAATATGAACGGTCAATCATATCAAATCCT

TTCTATGCAGAGGCAAGGGTATTTTTATCAAATTGAATCGTAAAACCTTGAATTCCCCCACCTGTGTAACAT

TCTTTAAAGCGATTGATTACCTCAGTATAGATTATCACAGATGAGCTTGTTGGCTTAATGCTAAATGTAAAT

TCCAATGGTAATCGGTTTTCAGATTTAGCATGTACTAGTCGTATCGATATTTCAGTTGTTTTGAGTTTTCTC

TGACGAAGTTTTGAAGTTGCTGTTTCAACGATTCCATGTAAAAATCTTTCAAGCATTTCAATATCATTACAT

CCTTTGCTACGGATTTCTGAAAATTGTACTGTATTTTTTTCTTGTTTCATTTTAATCCCTCCAATCCACCCG

CGGAATGACCACCGATAAGTTTACTGCGTTCAATATTTCTGGAACCTTCAGTTAGGACGGTTCCTTTTTGTA

TGGCTAAAAAACCAAACTGTTCTCTGACAACATCAATAGCTGTCTGAAGTCTATTATCTTTTTCAATTTGTT

CTACATCATCAAAGAGTGATAGTAGAGTATAGCTTTCATCTACGAAGCCACTATAAGATACACCAATTTGTC

TCACTGCACCAGAGGTATATTTTTTCGGAATAATACAAGTACATGACTCACCATTGTTTTGGGGAGATTTG

CGGGTTCAATTTTATTCTGAGCATTTATAGATTTTTTCATCTCAGTCCTAGAATAGCCAATATGAATAGAAA

CGACAGTAGTCAATACTAGGGCTACTGTTCCGTTCCACAGTATCATTTAAAAATCATT-TTCACACCCTTTC

GTCTATTAGTATAGAAGAAAGCTCTCAGCACA

>EU-NP05/Mega-1.III

CATGTTGAGGCGGTAAGTTTGCTAGTCAAGGAGTAAAACGACGAAGATTAGCATTTACTTCCGCCCATGCGA

TAGCTGTCCGTGATTGACAAGTGCTAGCACGCAGACAGAACGGAGATAGCGAACCGCTGAGTGTGTCGCTCT

GCTCGTAAAAGCTTAGAAACCTTTGAACGAAAGGGATAATGAAAGCCTTGATTGCAAGGCTTTTTGCTTTAT

GGTGGGTAAGTATCAGAGTGAGAAAATTTTTGGAATGAGTAGAAGTGATAGCTAGAAATTATCAGTTTCTA

TTTCCATTTACCCTGTGGGTACGTGTTTGTTTCCATTGACAAGGAGTTTGTGGGAATAGAAATGT

ACCCACCTTGTTTGAATCAAGTGAAGTGTAGTTGAAGGAAATCTGTTGAAAGCAATACTTCATTTTACCG

AATAAGTAATAATTTAGGCAACTTCAAATCGATTAAAAAAAACTATTTTAAAGGTTAAGAGTAGACAAAA

ATTGTCCACTCTTTTTTGCAAACTCAATTTATCAATAAATGAAATGAGGGAATGTAAAATG

AAATATTTTGAGGTTGAGTTAGAAAATCCTGATGAATTTTTAAAACTACAAACAGAAGATTTTGTGAAAG

CTAATCGCTTGCTACTAAGGAAGATAATCCAGAGCGTTACAGTCTATGAAGAAAACTTCGTCATATCCTT

TAAATCTGGCATCGAATTGGAAGTATGAGTCTCATTCCATAACTTTTATATTGAACATATCATCTTTTTGTG

TTATACTATAAATTGATATAAACAAAGATGTAGGAGGAACCGAAACTATGACAGCCTCAATGCGTTTAAGAT

AAGCTGGCAATAAAAAAAGCAGAATCTATACCCGATGATAGGCTTTTTTGTTGTGCTTATTTATACGATATT

GAGCATTCATTAGTTACGGTGAGGATATTGGTTATTTAACTATACCTTTATTTAACTATGTCTTTAATATGA

ATGTTTCCAAATTGTATGTATGCAGACCAAAAGCCACATTGTGGGGTTTGGCCTGCATTTTTTTTGCCTA

GAATGCTATTCAAAATAGAAATTCAAGCAAAATAATATGCAGGAGATAATATAAATGGAAAAATACAACAAT

TGGAAACGAAAATTTTATGCAATATGGGCAGGGCAAGCAGTATCATTAATCACTAGTGCCATCCTGCAAATG

GCGATTATTTTTTACCTTACAGAAAAAACAGGATCTGCGATGGTCTTGTCTATGGCTTCATTAGTAGGTTTT

TTACCCTATGCGATTTTGGGACCTGCCATTGGTGTGCTAGTGGATCGTCATGATAGGAAGAAGATAATGATT

GGTGCCGATTTAATTATCGCAGCAGCTGGTGCAGTGCTTGCTATTGTTGCATTCTGTATGGAGCTACCTGTC

TGGATGATTATGATAGTATTGTTTATCCGTAGCATTGGAACAGCTTTTCATACCCCAGCACTCAATGCGGTT

ACACCACTTTTAGTACCAGAAGAACAGCTAACGAAATGCGCAGGCTATAGTCAGTCTTTGCAGTCTATAAGC

TATATTGTTAGTCCGGCAGTTGCAGCACTCTTATACTCCGTTTGGGATTTAAATGCTATTATTGCCATCGAC

GTATTGGGTGCTGTGATTGCATCTATTACGGTAGCAATTGTACGTATACCTAAGCTGGGTAATCAAGTGCAA

AGTTTAGAACCAAATTTCATAAGGGAGATGAAAGAAGGAGTTGTGGTTCTGAGACAAAACAAAGGATTGTTT

GCCTTATTACTCTTAGGAACACTATATACTTTTGTTTATATGCCAATCAATGCACTATTTCCTTTAATAAGC

ATGGAACACTTTAATGGAACGCCTGTGCATATTTCTATTACGGAAATTTCCTTTGCATTTGGGATGCTAGCA

GGAGGCTTATTATTAGGAAGATTAGGGGGCTTCGAAAAGCATGTATTACTAATAACAAGTTCATTTTTTATA

ATGGGGACCAGTTTAGCCGTTTCGGGAATACTTCCTCCAAATGGATTTGTAATATTCGTAGTTTGCTGTGCA

ATAATGGGGCTTTCGGTGCCATTTTATAGCGGTGTGCAAACAGCTCTTTTTCAGGAGAAAATTAAGCCTGAA

TATTTAGGACGTGTATTTTCTTTGATCGGAAGTATCATGTCACTTGCTATGCCAATTGGGTTAATTCTTTCT

GGATTCTTTGCTGATAAAATCGGTGTAAATCATTGGTTTTTACTATCAGGTATTTTAATTATTGGCATTGCT

ATAGTTTGCCAAATGATAACTGAGGTTAGAAAATTAGATTTAAAATAAACAATATTGGAGGAATATTTATGT

ATCTTATTTTCATGTAACTCTTCCTGCTAAAATCGCAGGGTTTTCCCTGCATACAAGCAAATGAAAGCATGC

GATTATAGACAGGAGGAAATGTTATGGAATTAATATT

AAAAGCAAAAGACATTCGTGTGGAATTCAAAGGACGCGATGTTTTAGATATAAATGAATTAGAAGTATATGA

TTATGACCGTATTGGTTTAGTAGGAGCAAATGGTGCTGGAAAAAGCACTTTACTCAGGGTACTTTTAGGAGA

ATTAACTCCCCCAGGATGTAAAATGAATCGTCTGGGTGAACTTGCCTATATTCCCCAGTTGGACGAAGTAAC

TCTGCAGGAGGAAAAAGATTTTGCACTTGTAGGCAAGCTAGGTGTTGAGCAATTAAATATACAGACTATGAG

CGGTGGTGAAGAAACAAGGCTTAAAATAGCACAGGCCTTATCGGCACAGGTTCATGGTATTTTAGCGGATGA

ACCTACGAGCCATTTAGACCGTGAAGGAATTGATTTTCTAATAGGACAGCTAAAATATTTTACAGGTGCACT

GTTAGTTATTAGCCATGACCGCTATTTTCTTGATGAAATAGTAGATAAAATATGGGAACTGAAAGATGGCAA

AATCACTGAGTATTGGGGAAACTATTCTGATTATCTTCGTCAGAAAGAGGAAGAACGTAAGAGCCAAGCTGC

AGAATACGAACAATTTATTGCGGAACGTGCCCGATTGGAAAGGGCTGCGGAGGAAAAGCGAAAACAGGCTCG

TAAAATAGAACAGAAGGCAAAAGGTTCTTCAAAGAAAAAAAGTACTGAAGACGGAGGGCGTTTAGCTCATCA

AAAATCAATAGGAAGTAAGGAAAAAAAGATGTATAATGCTGCTAAAACCCTAGAGCACAGGATTGCGGCCTT

AGGAAAAGTAGAAGCTCCGGAAGGCATTCGCAGAATTCGTTTCAGGCAAAGTAAAGCATTGGAGCTCCATAA

TCCATACCCTATAGTCGGTGCAGAAATTAATAAAGTATTTGGGGATAAGGCTCTGTTTGAAAATGCATCTTT

TCAAATTCCGTTAGGAGCAAAAGTGGCGTTAACTGGTGGTAATGGAATCGGAAAAACAACTTTAATCCAAAT

GATCTTAAACCATGAAGAAGGAATTTCTATTTCGCCTAAGGCAAAAATAGGTTACTTTGCACAGAATGGTTA

CAAGTACAACAGTAATCAGAATGTTATGGAGTTTATGCAGAAGGATTGTGACTACAATATATCAGAAATTCG

TTCAGTGCTAGCATCTATGGGGTTCAAACAGAACGATATTGGAAAAAGTTTATCTGTTTTAAGCGGTGGAGA

AATTATAAAATTGTTGCTTGCTAAAATGCTCATGGGTAGATATAACATCCTAATAATGGATGAACCCAGTAA

CTTCCTTGACATACCAAGTTTAGAGGCTTTGGAAATACTAATGAAGGAGTACACCGGAACTATCGTGTTTAT

CACCCACGATAAACGATTACTCGAAAATGTAGCAGATGTAGTTTATGAAATTAGAGATAAGAAAATAAATCT

GAAACATTAAATTTAAGGTAGTCGCTGGTCAGTATAGTCTGTTCTGGTTGGCGACTCCATTGTTAAAGAGTA

TAAAGACTTTAGATTTTATGAATATTAAAAATAGGAACAGTCAATTGAACTGCTCCTATTTTTCTGCTAAAT

ATATTGTAGTTTTCTTATATGTATAATGATAGATTAGCGGATTCTCATCTACGGTACTTACTTCAAATATGA

AGAAGTGATCGCGGTTATCTCTGGACTTTTCCTTATTGAGGACAAAGTAATTCTTACGTGAAGTCGCCATTG

TTTTTAGGATATCATCAGTTAGGAAGGTCAATGGAATATTCATGTTAGAGTAGCGGTAGAAGTCACGTTCAA

AATCTTGGTAGCTCTCGCTATAATAGTCCATTTGTAGGTGATTACGCTGAAACTCAAGCTGATTCATAGAGC

ACCTCCTCGACAAGTTCAATACTAATAATGTCTTTTAATTTCAAATTGATGTGACCTGTTGTAGTTTTTATC

AAAATGAAATCTTTGGTCAGACTTGGTATTGTTCCAGTGTAGGAAACACGCTTGTTTTTTTCAATCACTTGA

ATGCGTGTGCGTAGCTGCCCGGCGTATACTTGACTGAGGAGTAATAATTTCTTCTCTAGTGATAAGTCAGAC

ATGTACGTTACTTTGTTTGTATCATCAGAGAGTGCTGATGCATGTTCAGATAGGAAAAAGCCCATCCATTTT

TGCATCTTTGTATCCTGGTACTCTCTTGCTGATTGAAATGGTAAATATGAACGGTCAATCATATCAAATCCT

TTCTATGCAGAGGCAAGGGTATTTTTATCAAATTGAATCGTAAAACCTTGAATTCCCCCACCTGTGTAACAT

TCTTTAAAGCGATTGATTACCTCAGTATAGATTATCACAGATGAGCTTGTTGGCTTAATGCTAAATGTAAAT

TCCAATGGTAATCGGTTTTCAGATTTAGCATGTACTAGTCGTATCGATATTTCAGTTGTTTTGAGTTTTCTC

TGACGAAGTTTTGAAGTTGCTGTTTCAACGATTCCATGTAAAAATCTTTCAAGCATTTCAATATCATTACAT

CCTTTGCTACGGATTTCTGAAAATTGTACTGTATTTTTTTCTTGTTTCATTTTAATCCCTCCAATCCACCCG

CGGAATGACCACCGATAAGTTTACTGCGTTCAATATTTCTGGAACCTTCAGTTAGGACGGTTCCTTTTTGTA

TGGCTAAAAAACCAAACTGTTCTCTGACAACATCAATAGCTGTCTGAAGTCTATTATCTTTTTCAATTTGTT

CTACATCATCAAAGAGTGATAGTAGAGTATAGCTTTCATCTACGAAGCCACTATAAGATACACCAATTTGTC

TCACTGCACCAGAGGTATATTTTTTCGGAATAATACAAGTACATGACTCACCATTGTTTTGGGGAGATTTG

CGGGTTCAATTTTATTCTGAGCATTTATAGATTTTTTCATCTCAGTCCTAGAATAGCCAATATGAATAGAAA

CGACAGTAGTCAATACTAGGGCTACTGTTCCGTTCCACAGTATCATTTAAAAATCATT-TTCACACCCTTTC

GTCTATTAGTATAGAAGAAAGCTCTCAGCACA

>GA62681/Mega-1.III

CATGTTGAGGCGGTAAGTTTGCTAGTCAAGGAGTAAAACGACGAAGATTAGCATTTACTTCCGCCCATGCGA

TAGCTGTCCGTGATTGACAAGTGCTAGCACGCAGACAGAACGGAGATAGCGAACCGCTGAGTGTGTCGCTCT

GCTCGTAAAAGCTTAGAAACCTTTGAACGAAAGGGATAATGAAAGCCTTGATTGCAAGGCTTTTTGCTTTAT

GGTGGGTAAGTATCAGAGTGAGAAAATTTTTGGAATGAGTAGAAGTGATAGCTAGAAATTATCAGTTTCTA

TTTCCATTTACCCTGTGGGTACGTGTTTGTTTCCATTGACAAGGAGTTTGTGGGAATAGAAATGT

ACCCACCTTGTTTGAATCAAGTGAAGTGTAGTTGAAGGAAATCTGTTGAAAGCAATACTTCATTTTACCG

AATAAGTAATAATTTAGGCAACTTCAAATCGATTAAAAAAAACTATTTTAAAGGTTAAGAGTAGACAAAA

ATTGTCCACTCTTTTTTGCAAACTCAATTTATCAATAAATGAAATGAGGGAATGTAAAATG

AAATATTTTGAGGTTGAGTTAGAAAATCCTGATGAATTTTTAAAACTACAAACAGAAGATTTTGTGAAAG

CTAATCGCTTGCTACTAAGGAAGATAATCCAGAGCGTTACAGTCTATGAAGAAAACTTCGTCATATCCTT

TAAATCTGGCATCGAATTGGAAGTATGAGTCTCATTCCATAACTTTTATATTGAACATATCATCTTTTTGTG

TTATACTATAAATTGATATAAACAAAGATGTAGGAGGAACCGAAACTATGACAGCCTCAATGCGTTTAAGAT

AAGCTGGCAATAAAAAAAGCAGAATCTATACCCGATGATAGGCTTTTTTGTTGTGCTTATTTATACGATATT

GAGCATTCATTAGTTACGGTGAGGATATTGGTTATTTAACTATACCTTTATTTAACTATGTCTTTAATATGA

ATGTTTCCAAATTGTATGTATGCAGACCAAAAGCCACATTGTGGGGTTTGGCCTGCATTTTTTTTGCCTA

GAATGCTATTCAAAATAGAAATTCAAGCAAAATAATATGCAGGAGATAATATAAATGGAAAAATACAACAAT

TGGAAACGAAAATTTTATGCAATATGGGCAGGGCAAGCAGTATCATTAATCACTAGTGCCATCCTGCAAATG

GCGATTATTTTTTACCTTACAGAAAAAACAGGATCTGCGATGGTCTTGTCTATGGCTTCATTAGTAGGTTTT

TTACCCTATGCGATTTTGGGACCTGCCATTGGTGTGCTAGTGGATCGTCATGATAGGAAGAAGATAATGATT

GGTGCCGATTTAATTATCGCAGCAGCTGGTGCAGTGCTTGCTATTGTTGCATTCTGTATGGAGCTACCTGTC

TGGATGATTATGATAGTATTGTTTATCCGTAGCATTGGAACAGCTTTTCATACCCCAGCACTCAATGCGGTT

ACACCACTTTTAGTACCAGAAGAACAGCTAACGAAATGCGCAGGCTATAGTCAGTCTTTGCAGTCTATAAGC

TATATTGTTAGTCCGGCAGTTGCAGCACTCTTATACTCCGTTTGGGATTTAAATGCTATTATTGCCATCGAC

GTATTGGGTGCTGTGATTGCATCTATTACGGTAGCAATTGTACGTATACCTAAGCTGGGTAATCAAGTGCAA

AGTTTAGAACCAAATTTCATAAGGGAGATGAAAGAAGGAGTTGTGGTTCTGAGACAAAACAAAGGATTGTTT

GCCTTATTACTCTTAGGAACACTATATACTTTTGTTTATATGCCAATCAATGCACTATTTCCTTTAATAAGC

ATGGAACACTTTAATGGAACGCCTGTGCATATTTCTATTACGGAAATTTCCTTTGCATTTGGGATGCTAGCA

GGAGGCTTATTATTAGGAAGATTAGGGGGCTTCGAAAAGCATGTATTACTAATAACAAGTTCATTTTTTATA

ATGGGGACCAGTTTAGCCGTTTCGGGAATACTTCCTCCAAATGGATTTGTAATATTCGTAGTTTGCTGTGCA

ATAATGGGGCTTTCGGTGCCATTTTATAGCGGTGTGCAAACAGCTCTTTTTCAGGAGAAAATTAAGCCTGAA

TATTTAGGACGTGTATTTTCTTTGATCGGAAGTATCATGTCACTTGCTATGCCAATTGGGTTAATTCTTTCT

GGATTCTTTGCTGATAAAATCGGTGTAAATCATTGGTTTTTACTATCAGGTATTTTAATTATTGGCATTGCT

ATAGTTTGCCAAATGATAACTGAGGTTAGAAAATTAGATTTAAAATAAACAATATTGGAGGAATATTTATGT

ATCTTATTTTCATGTAACTCTTCCTGCTAAAATCGCAGGGTTTTCCCTGCATACAAGCAAATGAAAGCATGC

GATTATAGACAGGAGGAAATGTTATGGAATTAATATT

AAAAGCAAAAGACATTCGTGTGGAATTCAAAGGACGCGATGTTTTAGATATAAATGAATTAGAAGTATATGA

TTATGACCGTATTGGTTTAGTAGGAGCAAATGGTGCTGGAAAAAGCACTTTACTCAGGGTACTTTTAGGAGA

ATTAACTCCCCCAGGATGTAAAATGAATCGTCTGGGTGAACTTGCCTATATTCCCCAGTTGGACGAAGTAAC

TCTGCAGGAGGAAAAAGATTTTGCACTTGTAGGCAAGCTAGGTGTTGAGCAATTAAATATACAGACTATGAG

CGGTGGTGAAGAAACAAGGCTTAAAATAGCACAGGCCTTATCGGCACAGGTTCATGGTATTTTAGCGGATGA

ACCTACGAGCCATTTAGACCGTGAAGGAATTGATTTTCTAATAGGACAGCTAAAATATTTTACAGGTGCACT

GTTAGTTATTAGCCATGACCGCTATTTTCTTGATGAAATAGTAGATAAAATATGGGAACTGAAAGATGGCAA

AATCACTGAGTATTGGGGAAACTATTCTGATTATCTTCGTCAGAAAGAGGAAGAACGTAAGAGCCAAGCTGC

AGAATACGAACAATTTATTGCGGAACGTGCCCGATTGGAAAGGGCTGCGGAGGAAAAGCGAAAACAGGCTCG

TAAAATAGAACAGAAGGCAAAAGGTTCTTCAAAGAAAAAAAGTACTGAAGACGGAGGGCGTTTAGCTCATCA

AAAATCAATAGGAAGTAAGGAAAAAAAGATGTATAATGCTGCTAAAACCCTAGAGCACAGGATTGCGGCCTT

AGGAAAAGTAGAAGCTCCGGAAGGCATTCGCAGAATTCGTTTCAGGCAAAGTAAAGCATTGGAGCTCCATAA

TCCATACCCTATAGTCGGTGCAGAAATTAATAAAGTATTTGGGGATAAGGCTCTGTTTGAAAATGCATCTTT

TCAAATTCCGTTAGGAGCAAAAGTGGCGTTAACTGGTGGTAATGGAATCGGAAAAACAACTTTAATCCAAAT

GATCTTAAACCATGAAGAAGGAATTTCTATTTCGCCTAAGGCAAAAATAGGTTACTTTGCACAGAATGGTTA

CAAGTACAACAGTAATCAGAATGTTATGGAGTTTATGCAGAAGGATTGTGACTACAATATATCAGAAATTCG

TTCAGTGCTAGCATCTATGGGGTTCAAACAGAACGATATTGGAAAAAGTTTATCTGTTTTAAGCGGTGGAGA

AATTATAAAATTGTTGCTTGCTAAAATGCTCATGGGTAGATATAACATCCTAATAATGGATGAACCCAGTAA

CTTCCTTGACATACCAAGTTTAGAGGCTTTGGAAATACTAATGAAGGAGTACACCGGAACTATCGTGTTTAT

CACCCACGATAAACGATTACTCGAAAATGTAGCAGATGTAGTTTATGAAATTAGAGATAAGAAAATAAATCT

GAAACATTAAATTTAAGGTAGTCGCTGGTCAGTATAGTCTGTTCTGGTTGGCGACTCCATTGTTAAAGAGTA

TAAAGACTTTAGATTTTATGAATATTAAAAATAGGAACAGTCAATTGAACTGCTCCTATTTTTCTGCTAAAT

ATATTGTAGTTTTCTTATATGTATAATGATAGATTAGCGGATTCTCATCTACGGTACTTACTTCAAATATGA

AGAAGTGATCGCGGTTATCTCTGGACTTTTCCTTATTGAGGACAAAGTAATTCTTACGTGAAGTCGCCATTG

TTTTTAGGATATCATCAGTTAGGAAGGTCAATGGAATATTCATGTTAGAGTAGCGGTAGAAGTCACGTTCAA

AATCTTGGTAGCTCTCGCTATAATAGTCCATTTGTAGGTGATTACGCTGAAACTCAAGCTGATTCATAGAGC

ACCTCCTCGACAAGTTCAATACTAATAATGTCTTTTAATTTCAAATTGATGTGACCTGTTGTAGTTTTTATC

AAAATGAAATCTTTGGTCAGACTTGGTATTGTTCCAGTGTAGGAAACACGCTTGTTTTTTTCAATCACTTGA

ATGCGTGTGCGTAGCTGCCCGGCGTATACTTGACTGAGGAGTAATAATTTCTTCTCTAGTGATAAGTCAGAC

ATGTACGTTACTTTGTTTGTATCATCAGAGAGTGCTGATGCATGTTCAGATAGGAAAAAGCCCATCCATTTT

TGCATCTTTGTATCCTGGTACTCTCTTGCTGATTGAAATGGTAAATATGAACGGTCAATCATATCAAATCCT

TTCTATGCAGAGGCAAGGGTATTTTTATCAAATTGAATCGTAAAACCTTGAATTCCCCCACCTGTGTAACAT

TCTTTAAAGCGATTGATTACCTCAGTATAGATTATCACAGATGAGCTTGTTGGCTTAATGCTAAATGTAAAT

TCCAATGGTAATCGGTTTTCAGATTTAGCATGTACTAGTCGTATCGATATTTCAGTTGTTTTGAGTTTTCTC

TGACGAAGTTTTGAAGTTGCTGTTTCAACGATTCCATGTAAAAATCTTTCAAGCATTTCAATATCATTACAT

CCTTTGCTACGGATTTCTGAAAATTGTACTGTATTTTTTTCTTGTTTCATTTTAATCCCTCCAATCCACCCG

CGGAATGACCACCGATAAGTTTACTGCGTTCAATATTTCTGGAACCTTCAGTTAGGACGGTTCCTTTTTGTA

TGGCTAAAAAACCAAACTGTTCTCTGACAACATCAATAGCTGTCTGAAGTCTATTATCTTTTTCAATTTGTT

CTACATCATCAAAGAGTGATAGTAGAGTATAGCTTTCATCTACGAAGCCACTATAAGATACACCAATTTGTC

TCACTGCACCAGAGGTATATTTTTTCGGAATAATACAAGTACATGACTCACCATTGTTTTGGGGAGATTTG

CGGGTTCAATTTTATTCTGAGCATTTATAGATTTTTTCATCTCAGTCCTAGAATAGCCAATATGAATAGAAA

CGACAGTAGTCAATACTAGGGCTACTGTTCCGTTCCACAGTATCATTTAAAAATCATT-TTCACACCCTTTC

GTCTATTAGTATAGAAGAAAGCTCTCAGCACA

>GA47522/Mega-1.III

CATGTTGAGGCGGTAAGTTTGCTAGTCAAGGAGTAAAACGACGAAGATTAGCATTTACTTCCGCCCATGCGA

TAGCTGTCCGTGATTGACAAGTGCTAGCACGCAGACAGAACGGAGATAGCGAACCGCTGAGTGTGTCGCTCT

GCTCGTAAAAGCTTAGAAACCTTTGAACGAAAGGGATAATGAAAGCCTTGATTGCAAGGCTTTTTGCTTTAT

GGTGGGTAAGTATCAGAGTGAGAAAATTTTTGGAATGAGTAGAAGTGATAGCTAGAAATTATCAGTTTCTA

TTTCCATTTACCCTGTGGGTACGTGTTTGTTTCCATTGACAAGGAGTTTGTGGGAATAGAAATGT

ACCCACCTTGTTTGAATCAAGTGAAGTGTAGTTGAAGGAAATCTGTTGAAAGCAATACTTCATTTTACCG

AATAAGTAATAATTTAGGCAACTTCAAATCGATTAAAAAAAACTATTTTAAAGGTTAAGAGTAGACAAAA

ATTGTCCACTCTTTTTTGCAAACTCAATTTATCAATAAATGAAATGAGGGAATGTAAAATG

AAATATTTTGAGGTTGAGTTAGAAAATCCTGATGAATTTTTAAAACTACAAACAGAAGATTTTGTGAAAG

CTAATCGCTTGCTACTAAGGAAGATAATCCAGAGCGTTACAGTCTATGAAGAAAACTTCGTCATATCCTT

TAAATCTGGCATCGAATTGGAAGTATGAGTCTCATTCCATAACTTTTATATTGAACATATCATCTTTTTGTG

TTATACTATAAATTGATATAAACAAAGATGTAGGAGGAACCGAAACTATGACAGCCTCAATGCGTTTAAGAT

AAGCTGGCAATAAAAAAAGCAGAATCTATACCCGATGATAGGCTTTTTTGTTGTGCTTATTTATACGATATT

GAGCATTCATTAGTTACGGTGAGGATATTGGTTATTTAACTATACCTTTATTTAACTATGTCTTTAATATGA

ATGTTTCCAAATTGTATGTATGCAGACCAAAAGCCACATTGTGGGGTTTGGCCTGCATTTTTTTTGCCTA

GAATGCTATTCAAAATAGAAATTCAAGCAAAATAATATGCAGGAGATAATATAAATGGAAAAATACAACAAT

TGGAAACGAAAATTTTATGCAATATGGGCAGGGCAAGCAGTATCATTAATCACTAGTGCCATCCTGCAAATG

GCGATTATTTTTTACCTTACAGAAAAAACAGGATCTGCGATGGTCTTGTCTATGGCTTCATTAGTAGGTTTT

TTACCCTATGCGATTTTGGGACCTGCCATTGGTGTGCTAGTGGATCGTCATGATAGGAAGAAGATAATGATT

GGTGCCGATTTAATTATCGCAGCAGCTGGTGCAGTGCTTGCTATTGTTGCATTCTGTATGGAGCTACCTGTC

TGGATGATTATGATAGTATTGTTTATCCGTAGCATTGGAACAGCTTTTCATACCCCAGCACTCAATGCGGTT

ACACCACTTTTAGTACCAGAAGAACAGCTAACGAAATGCGCAGGCTATAGTCAGTCTTTGCAGTCTATAAGC

TATATTGTTAGTCCGGCAGTTGCAGCACTCTTATACTCCGTTTGGGATTTAAATGCTATTATTGCCATCGAC

GTATTGGGTGCTGTGATTGCATCTATTACGGTAGCAATTGTACGTATACCTAAGCTGGGTAATCAAGTGCAA

AGTTTAGAACCAAATTTCATAAGGGAGATGAAAGAAGGAGTTGTGGTTCTGAGACAAAACAAAGGATTGTTT

GCCTTATTACTCTTAGGAACACTATATACTTTTGTTTATATGCCAATCAATGCACTATTTCCTTTAATAAGC

ATGGAACACTTTAATGGAACGCCTGTGCATATTTCTATTACGGAAATTTCCTTTGCATTTGGGATGCTAGCA

GGAGGCTTATTATTAGGAAGATTAGGGGGCTTCGAAAAGCATGTATTACTAATAACAAGTTCATTTTTTATA

ATGGGGACCAGTTTAGCCGTTTCGGGAATACTTCCTCCAAATGGATTTGTAATATTCGTAGTTTGCTGTGCA

ATAATGGGGCTTTCGGTGCCATTTTATAGCGGTGTGCAAACAGCTCTTTTTCAGGAGAAAATTAAGCCTGAA

TATTTAGGACGTGTATTTTCTTTGATCGGAAGTATCATGTCACTTGCTATGCCAATTGGGTTAATTCTTTCT

GGATTCTTTGCTGATAAAATCGGTGTAAATCATTGGTTTTTACTATCAGGTATTTTAATTATTGGCATTGCT

ATAGTTTGCCAAATGATAACTGAGGTTAGAAAATTAGATTTAAAATAAACAATATTGGAGGAATATTTATGT

ATCTTATTTTCATGTAACTCTTCCTGCTAAAATCGCAGGGTTTTCCCTGCATACAAGCAAATGAAAGCATGC

GATTATAGACAGGAGGAAATGTTATGGAATTAATATT

AAAAGCAAAAGACATTCGTGTGGAATTCAAAGGACGCGATGTTTTAGATATAAATGAATTAGAAGTATATGA

TTATGACCGTATTGGTTTAGTAGGAGCAAATGGTGCTGGAAAAAGCACTTTACTCAGGGTACTTTTAGGAGA

ATTAACTCCCCCAGGATGTAAAATGAATCGTCTGGGTGAACTTGCCTATATTCCCCAGTTGGACGAAGTAAC

TCTGCAGGAGGAAAAAGATTTTGCACTTGTAGGCAAGCTAGGTGTTGAGCAATTAAATATACAGACTATGAG

CGGTGGTGAAGAAACAAGGCTTAAAATAGCACAGGCCTTATCGGCACAGGTTCATGGTATTTTAGCGGATGA

ACCTACGAGCCATTTAGACCGTGAAGGAATTGATTTTCTAATAGGACAGCTAAAATATTTTACAGGTGCACT

GTTAGTTATTAGCCATGACCGCTATTTTCTTGATGAAATAGTAGATAAAATATGGGAACTGAAAGATGGCAA

AATCACTGAGTATTGGGGAAACTATTCTGATTATCTTCGTCAGAAAGAGGAAGAACGTAAGAGCCAAGCTGC

AGAATACGAACAATTTATTGCGGAACGTGCCCGATTGGAAAGGGCTGCGGAGGAAAAGCGAAAACAGGCTCG

TAAAATAGAACAGAAGGCAAAAGGTTCTTCAAAGAAAAAAAGTACTGAAGACGGAGGGCGTTTAGCTCATCA

AAAATCAATAGGAAGTAAGGAAAAAAAGATGTATAATGCTGCTAAAACCCTAGAGCACAGGATTGCGGCCTT

AGGAAAAGTAGAAGCTCCGGAAGGCATTCGCAGAATTCGTTTCAGGCAAAGTAAAGCATTGGAGCTCCATAA

TCCATACCCTATAGTCGGTGCAGAAATTAATAAAGTATTTGGGGATAAGGCTCTGTTTGAAAATGCATCTTT

TCAAATTCCGTTAGGAGCAAAAGTGGCGTTAACTGGTGGTAATGGAATCGGAAAAACAACTTTAATCCAAAT

GATCTTAAACCATGAAGAAGGAATTTCTATTTCGCCTAAGGCAAAAATAGGTTACTTTGCACAGAATGGTTA

CAAGTACAACAGTAATCAGAATGTTATGGAGTTTATGCAGAAGGATTGTGACTACAATATATCAGAAATTCG

TTCAGTGCTAGCATCTATGGGGTTCAAACAGAACGATATTGGAAAAAGTTTATCTGTTTTAAGCGGTGGAGA

AATTATAAAATTGTTGCTTGCTAAAATGCTCATGGGTAGATATAACATCCTAATAATGGATGAACCCAGTAA

CTTCCTTGACATACCAAGTTTAGAGGCTTTGGAAATACTAATGAAGGAGTACACCGGAACTATCGTGTTTAT

CACCCACGATAAACGATTACTCGAAAATGTAGCAGATGTAGTTTATGAAATTAGAGATAAGAAAATAAATCT

GAAACATTAAATTTAAGGTAGTCGCTGGTCAGTATAGTCTGTTCTGGTTGGCGACTCCATTGTTAAAGAGTA

TAAAGACTTTAGATTTTATGAATATTAAAAATAGGAACAGTCAATTGAACTGCTCCTATTTTTCTGCTAAAT

ATATTGTAGTTTTCTTATATGTATAATGATAGATTAGCGGATTCTCATCTACGGTACTTACTTCAAATATGA

AGAAGTGATCGCGGTTATCTCTGGACTTTTCCTTATTGAGGACAAAGTAATTCTTACGTGAAGTCGCCATTG

TTTTTAGGATATCATCAGTTAGGAAGGTCAATGGAATATTCATGTTAGAGTAGCGGTAGAAGTCACGTTCAA

AATCTTGGTAGCTCTCGCTATAATAGTCCATTTGTAGGTGATTACGCTGAAACTCAAGCTGATTCATAGAGC

ACCTCCTCGACAAGTTCAATACTAATAATGTCTTTTAATTTCAAATTGATGTGACCTGTTGTAGTTTTTATC

AAAATGAAATCTTTGGTCAGACTTGGTATTGTTCCAGTGTAGGAAACACGCTTGTTTTTTTCAATCACTTGA

ATGCGTGTGCGTAGCTGCCCGGCGTATACTTGACTGAGGAGTAATAATTTCTTCTCTAGTGATAAGTCAGAC

ATGTACGTTACTTTGTTTGTATCATCAGAGAGTGCTGATGCATGTTCAGATAGGAAAAAGCCCATCCATTTT

TGCATCTTTGTATCCTGGTACTCTCTTGCTGATTGAAATGGTAAATATGAACGGTCAATCATATCAAATCCT

TTCTATGCAGAGGCAAGGGTATTTTTATCAAATTGAATCGTAAAACCTTGAATTCCCCCACCTGTGTAACAT

TCTTTAAAGCGATTGATTACCTCAGTATAGATTATCACAGATGAGCTTGTTGGCTTAATGCTAAATGTAAAT

TCCAATGGTAATCGGTTTTCAGATTTAGCATGTACTAGTCGTATCGATATTTCAGTTGTTTTGAGTTTTCTC

TGACGAAGTTTTGAAGTTGCTGTTTCAACGATTCCATGTAAAAATCTTTCAAGCATTTCAATATCATTACAT

CCTTTGCTACGGATTTCTGAAAATTGTACTGTATTTTTTTCTTGTTTCATTTTAATCCCTCCAATCCACCCG

CGGAATGACCACCGATAAGTTTACTGCGTTCAATATTTCTGGAACCTTCAGTTAGGACGGTTCCTTTTTGTA

TGGCTAAAAAACCAAACTGTTCTCTGACAACATCAATAGCTGTCTGAAGTCTATTATCTTTTTCAATTTGTT

CTACATCATCAAAGAGTGATAGTAGAGTATAGCTTTCATCTACGAAGCCACTATAAGATACACCAATTTGTC

TCACTGCACCAGAGGTATATTTTTTCGGAATAATACAAGTACATGACTCACCATTGTTTTGGGGAGATTTG

CGGGTTCAATTTTATTCTGAGCATTTATAGATTTTTTCATCTCAGTCCTAGAATAGCCAATATGAATAGAAA

CGACAGTAGTCAATACTAGGGCTACTGTTCCGTTCCACAGTATCATTTAAAAATCATT-TTCACACCCTTTC

GTCTATTAGTATAGAAGAAAGCTCTCAGCACA

>GA17570/Mega-1.III

CATGTTGAGGCGGTAAGTTTGCTAGTCAAGGAGTAAAACGACGAAGATTAGCATTTACTTCCGCCCATGCGA

TAGCTGTCCGTGATTGACAAGTGCTAGCACGCAGACAGAACGGAGATAGCGAACCGCTGAGTGTGTCGCTCT

GCTCGTAAAAGCTTAGAAACCTTTGAACGAAAGGGATAATGAAAGCCTTGATTGCAAGGCTTTTTGCTTTAT

GGTGGGTAAGTATCAGAGTGAGAAAATTTTTGGAATGAGTAGAAGTGATAGCTAGAAATTATCAGTTTCTA

TTTCCATTTACCCTGTGGGTACGTGTTTGTTTCCATTGACAAGGAGTTTGTGGGAATAGAAATGT

ACCCACCTTGTTTGAATCAAGTGAAGTGTAGTTGAAGGAAATCTGTTGAAAGCAATACTTCATTTTACCG

AATAAGTAATAATTTAGGCAACTTCAAATCGATTAAAAAAAACTATTTTAAAGGTTAAGAGTAGACAAAA

ATTGTCCACTCTTTTTTGCAAACTCAATTTATCAATAAATGAAATGAGGGAATGTAAAATG

AAATATTTTGAGGTTGAGTTAGAAAATCCTGATGAATTTTTAAAACTACAAACAGAAGATTTTGTGAAAG

CTAATCGCTTGCTACTAAGGAAGATAATCCAGAGCGTTACAGTCTATGAAGAAAACTTCGTCATATCCTT

TAAATCTGGCATCGAATTGGAAGTATGAGTCTCATTCCATAACTTTTATATTGAACATATCATCTTTTTGTG

TTATACTATAAATTGATATAAACAAAGATGTAGGAGGAACCGAAACTATGACAGCCTCAATGCGTTTAAGAT

AAGCTGGCAATAAAAAAAGCAGAATCTATACCCGATGATAGGCTTTTTTGTTGTGCTTATTTATACGATATT

GAGCATTCATTAGTTACGGTGAGGATATTGGTTATTTAACTATACCTTTATTTAACTATGTCTTTAATATGA

ATGTTTCCAAATTGTATGTATGCAGACCAAAAGCCACATTGTGGGGTTTGGCCTGCATTTTTTTTGCCTA

GAATGCTATTCAAAATAGAAATTCAAGCAAAATAATATGCAGGAGATAATATAAATGGAAAAATACAACAAT

TGGAAACGAAAATTTTATGCAATATGGGCAGGGCAAGCAGTATCATTAATCACTAGTGCCATCCTGCAAATG

GCGATTATTTTTTACCTTACAGAAAAAACAGGATCTGCGATGGTCTTGTCTATGGCTTCATTAGTAGGTTTT

TTACCCTATGCGATTTTGGGACCTGCCATTGGTGTGCTAGTGGATCGTCATGATAGGAAGAAGATAATGATT

GGTGCCGATTTAATTATCGCAGCAGCTGGTGCAGTGCTTGCTATTGTTGCATTCTGTATGGAGCTACCTGTC

TGGATGATTATGATAGTATTGTTTATCCGTAGCATTGGAACAGCTTTTCATACCCCAGCACTCAATGCGGTT

ACACCACTTTTAGTACCAGAAGAACAGCTAACGAAATGCGCAGGCTATAGTCAGTCTTTGCAGTCTATAAGC

TATATTGTTAGTCCGGCAGTTGCAGCACTCTTATACTCCGTTTGGGATTTAAATGCTATTATTGCCATCGAC

GTATTGGGTGCTGTGATTGCATCTATTACGGTAGCAATTGTACGTATACCTAAGCTGGGTAATCAAGTGCAA

AGTTTAGAACCAAATTTCATAAGGGAGATGAAAGAAGGAGTTGTGGTTCTGAGACAAAACAAAGGATTGTTT

GCCTTATTACTCTTAGGAACACTATATACTTTTGTTTATATGCCAATCAATGCACTATTTCCTTTAATAAGC

ATGGAACACTTTAATGGAACGCCTGTGCATATTTCTATTACGGAAATTTCCTTTGCATTTGGGATGCTAGCA

GGAGGCTTATTATTAGGAAGATTAGGGGGCTTCGAAAAGCATGTATTACTAATAACAAGTTCATTTTTTATA

ATGGGGACCAGTTTAGCCGTTTCGGGAATACTTCCTCCAAATGGATTTGTAATATTCGTAGTTTGCTGTGCA

ATAATGGGGCTTTCGGTGCCATTTTATAGCGGTGTGCAAACAGCTCTTTTTCAGGAGAAAATTAAGCCTGAA

TATTTAGGACGTGTATTTTCTTTGATCGGAAGTATCATGTCACTTGCTATGCCAATTGGGTTAATTCTTTCT

GGATTCTTTGCTGATAAAATCGGTGTAAATCATTGGTTTTTACTATCAGGTATTTTAATTATTGGCATTGCT

ATAGTTTGCCAAATGATAACTGAGGTTAGAAAATTAGATTTAAAATAAACAATATTGGAGGAATATTTATGT

ATCTTATTTTCATGTAACTCTTCCTGCTAAAATCGCAGGGTTTTCCCTGCATACAAGCAAATGAAAGCATGC

GATTATAGACAGGAGGAAATGTTATGGAATTAATATT

AAAAGCAAAAGACATTCGTGTGGAATTCAAAGGACGCGATGTTTTAGATATAAATGAATTAGAAGTATATGA

TTATGACCGTATTGGTTTAGTAGGAGCAAATGGTGCTGGAAAAAGCACTTTACTCAGGGTACTTTTAGGAGA

ATTAACTCCCCCAGGATGTAAAATGAATCGTCTGGGTGAACTTGCCTATATTCCCCAGTTGGACGAAGTAAC

TCTGCAGGAGGAAAAAGATTTTGCACTTGTAGGCAAGCTAGGTGTTGAGCAATTAAATATACAGACTATGAG

CGGTGGTGAAGAAACAAGGCTTAAAATAGCACAGGCCTTATCGGCACAGGTTCATGGTATTTTAGCGGATGA

ACCTACGAGCCATTTAGACCGTGAAGGAATTGATTTTCTAATAGGACAGCTAAAATATTTTACAGGTGCACT

GTTAGTTATTAGCCATGACCGCTATTTTCTTGATGAAATAGTAGATAAAATATGGGAACTGAAAGATGGCAA

AATCACTGAGTATTGGGGAAACTATTCTGATTATCTTCGTCAGAAAGAGGAAGAACGTAAGAGCCAAGCTGC

AGAATACGAACAATTTATTGCGGAACGTGCCCGATTGGAAAGGGCTGCGGAGGAAAAGCGAAAACAGGCTCG

TAAAATAGAACAGAAGGCAAAAGGTTCTTCAAAGAAAAAAAGTACTGAAGACGGAGGGCGTTTAGCTCATCA

AAAATCAATAGGAAGTAAGGAAAAAAAGATGTATAATGCTGCTAAAACCCTAGAGCACAGGATTGCGGCCTT

AGGAAAAGTAGAAGCTCCGGAAGGCATTCGCAGAATTCGTTTCAGGCAAAGTAAAGCATTGGAGCTCCATAA

TCCATACCCTATAGTCGGTGCAGAAATTAATAAAGTATTTGGGGATAAGGCTCTGTTTGAAAATGCATCTTT

TCAAATTCCGTTAGGAGCAAAAGTGGCGTTAACTGGTGGTAATGGAATCGGAAAAACAACTTTAATCCAAAT

GATCTTAAACCATGAAGAAGGAATTTCTATTTCGCCTAAGGCAAAAATAGGTTACTTTGCACAGAATGGTTA

CAAGTACAACAGTAATCAGAATGTTATGGAGTTTATGCAGAAGGATTGTGACTACAATATATCAGAAATTCG

TTCAGTGCTAGCATCTATGGGGTTCAAACAGAACGATATTGGAAAAAGTTTATCTGTTTTAAGCGGTGGAGA

AATTATAAAATTGTTGCTTGCTAAAATGCTCATGGGTAGATATAACATCCTAATAATGGATGAACCCAGTAA

CTTCCTTGACATACCAAGTTTAGAGGCTTTGGAAATACTAATGAAGGAGTACACCGGAACTATCGTGTTTAT

CACCCACGATAAACGATTACTCGAAAATGTAGCAGATGTAGTTTATGAAATTAGAGATAAGAAAATAAATCT

GAAACATTAAATTTAAGGTAGTCGCTGGTCAGTATAGTCTGTTCTGGTTGGCGACTCCATTGTTAAAGAGTA

TAAAGACTTTAGATTTTATGAATATTAAAAATAGGAACAGTCAATTGAACTGCTCCTATTTTTCTGCTAAAT

ATATTGTAGTTTTCTTATATGTATAATGATAGATTAGCGGATTCTCATCTACGGTACTTACTTCAAATATGA

AGAAGTGATCGCGGTTATCTCTGGACTTTTCCTTATTGAGGACAAAGTAATTCTTACGTGAAGTCGCCATTG

TTTTTAGGATATCATCAGTTAGGAAGGTCAATGGAATATTCATGTTAGAGTAGCGGTAGAAGTCACGTTCAA

AATCTTGGTAGCTCTCGCTATAATAGTCCATTTGTAGGTGATTACGCTGAAACTCAAGCTGATTCATAGAGC

ACCTCCTCGACAAGTTCAATACTAATAATGTCTTTTAATTTCAAATTGATGTGACCTGTTGTAGTTTTTATC

AAAATGAAATCTTTGGTCAGACTTGGTATTGTTCCAGTGTAGGAAACACGCTTGTTTTTTTCAATCACTTGA

ATGCGTGTGCGTAGCTGCCCGGCGTATACTTGACTGAGGAGTAATAATTTCTTCTCTAGTGATAAGTCAGAC

ATGTACGTTACTTTGTTTGTATCATCAGAGAGTGCTGATGCATGTTCAGATAGGAAAAAGCCCATCCATTTT

TGCATCTTTGTATCCTGGTACTCTCTTGCTGATTGAAATGGTAAATATGAACGGTCAATCATATCAAATCCT

TTCTATGCAGAGGCAAGGGTATTTTTATCAAATTGAATCGTAAAACCTTGAATTCCCCCACCTGTGTAACAT

TCTTTAAAGCGATTGATTACCTCAGTATAGATTATCACAGATGAGCTTGTTGGCTTAATGCTAAATGTAAAT

TCCAATGGTAATCGGTTTTCAGATTTAGCATGTACTAGTCGTATCGATATTTCAGTTGTTTTGAGTTTTCTC

TGACGAAGTTTTGAAGTTGCTGTTTCAACGATTCCATGTAAAAATCTTTCAAGCATTTCAATATCATTACAT

CCTTTGCTACGGATTTCTGAAAATTGTACTGTATTTTTTTCTTGTTTCATTTTAATCCCTCCAATCCACCCG

CGGAATGACCACCGATAAGTTTACTGCGTTCAATATTTCTGGAACCTTCAGTTAGGACGGTTCCTTTTTGTA

TGGCTAAAAAACCAAACTGTTCTCTGACAACATCAATAGCTGTCTGAAGTCTATTATCTTTTTCAATTTGTT

CTACATCATCAAAGAGTGATAGTAGAGTATAGCTTTCATCTACGAAGCCACTATAAGATACACCAATTTGTC

TCACTGCACCAGAGGTGTATTTTTTCGGAATAATACAAGTACATGACTCACCATTGTTTTGGGGAGATTTG

CGGGTTCAATTTTATTCTGAGCATTTATAGATTTTTTCATCTCAGTCCTAGAATAGCCAATATGAATAGAAA

CGACAGTAGTCAATACTAGGGCTACTGTTCCGTTCCACAGTATCATTTAAAAATCATT-TTCACACCCTTTC

GTCTATTAGTATAGAAGAAAGCTCTCAGCACA

>GA41277/Mega-1.III

CATGTTGAGGCGGTAAGTTTGCTAGTCAAGGAGTAAAACGACGAAGATTAGCATTTACTTCCGCCCATGCGA

TAGCTGTCCGTGATTGACAAGTGCTAGCACGCAGACAGAACGGAGATAGCGAACCGCTGAGTGTGTCGCTCT

GCTCGTAAAAGCTTAGAAACCTTTGAACGAAAGGGATAATGAAAGCCTTGATTGCAAGGCTTTTTGCTTTAT

GGTGGGTAAGTATCAGAGTGAGAAAATTTTTGGAATGAGTAGAAGTGATAGCTAGAAATTATCAGTTTCTA

TTTCCATTTACCCTGTGGGTACGTGTTTGTTTCCATTGACAAGGAGTTTGTGGGAATAGAAATGT

ACCCACCTTGTTTGAATCAAGTGAAGTGTAGTTGAAGGAAATCTGTTGAAAGCAATACTTCATTTTACCG

AATAAGTAATAATTTAGGCAACTTCAAATCGATTAAAAAAAACTATTTTAAAGGTTAAGAGTAGACAAAA

ATTGTCCACTCTTTTTTGCAAACTCAATTTATCAATAAATGAAATGAGGGAATGTAAAATG

AAATATTTTGAGGTTGAGTTAGAAAATCCTGATGAATTTTTAAAACTACAAACAGAAGATTTTGTGAAAG

CTAATCGCTTGCTACTAAGGAAGATAATCCAGAGCGTTACAGTCTATGAAGAAAACTTCGTCATATCCTT

TAAATCTGGCATCGAATTGGAAGTATGAGTCTCATTCCATAACTTTTATATTGAACATATCATCTTTTTGTG

TTATACTATAAATTGATATAAACAAAGATGTAGGAGGAACCGAAACTATGACAGCCTCAATGCGTTTAAGAT

AAGCTGGCAATAAAAAAAGCAGAATCTATACCCGATGATAGGCTTTTTTGTTGTGCTTATTTATACGATATT

GAGCATTCATTAGTTACGGTGAGGATATTGGTTATTTAACTATACCTTTATTTAACTATGTCTTTAATATGA

ATGTTTCCAAATTGTATGTATGCAGACCAAAAGCCACATTGTGGGGTTTGGCCTGCATTTTTTTTGCCTA

GAATGCTATTCAAAATAGAAATTCAAGCAAAATAATATGCAGGAGATAATATAAATGGAAAAATACAACAAT

TGGAAACGAAAATTTTATGCAATATGGGCAGGGCAAGCAGTATCATTAATCACTAGTGCCATCCTGCAAATG

GCGATTATTTTTTACCTTACAGAAAAAACAGGATCTGCGATGGTCTTGTCTATGGCTTCATTAGTAGGTTTT

TTACCCTATGCGATTTTGGGACCTGCCATTGGTGTGCTAGTGGATCGTCATGATAGGAAGAAGATAATGATT

GGTGCCGATTTAATTATCGCAGCAGCTGGTGCAGTGCTTGCTATTGTTGCATTCTGTATGGAGCTACCTGTC

TGGATGATTATGATAGTATTGTTTATCCGTAGCATTGGAACAGCTTTTCATACCCCAGCACTCAATGCGGTT

ACACCACTTTTAGTACCAGAAGAACAGCTAACGAAATGCGCAGGCTATAGTCAGTCTTTGCAGTCTATAAGC

TATATTGTTAGTCCGGCAGTTGCAGCACTCTTATACTCCGTTTGGGATTTAAATGCTATTATTGCCATCGAC

GTATTGGGTGCTGTGATTGCATCTATTACGGTAGCAATTGTACGTATACCTAAGCTGGGTAATCAAGTGCAA

AGTTTAGAACCAAATTTCATAAGGGAGATGAAAGAAGGAGTTGTGGTTCTGAGACAAAACAAAGGATTGTTT

GCCTTATTACTCTTAGGAACACTATATACTTTTGTTTATATGCCAATCAATGCACTATTTCCTTTAATAAGC

ATGGAACACTTTAATGGAACGCCTGTGCATATTTCTATTACGGAAATTTCCTTTGCATTTGGGATGCTAGCA

GGAGGCTTATTATTAGGAAGATTAGGGGGCTTCGAAAAGCATGTATTACTAATAACAAGTTCATTTTTTATA

ATGGGGACCAGTTTAGCCGTTTCGGGAATACTTCCTCCAAATGGATTTGTAATATTCGTAGTTTGCTGTGCA

ATAATGGGGCTTTCGGTGCCATTTTATAGCGGTGTGCAAACAGCTCTTTTTCAGGAGAAAATTAAGCCTGAA

TATTTAGGACGTGTATTTTCTTTGATCGGAAGTATCATGTCACTTGCTATGCCAATTGGGTTAATTCTTTCT

GGATTCTTTGCTGATAAAATCGGTGTAAATCATTGGTTTTTACTATCAGGTATTTTAATTATTGGCATTGCT

ATAGTTTGCCAAATGATAACTGAGGTTAGAAAATTAGATTTAAAATAAACAATATTGGAGGAATATTTATGT

ATCTTATTTTCATGTAACTCTTCCTGCTAAAATCGCAGGGTTTTCCCTGCATACAAGCAAATGAAAGCATGC

GATTATAGACAGGAGGAAATGTTATGGAATTAATATT

AAAAGCAAAAGACATTCGTGTGGAATTCAAAGGACGCGATGTTTTAGATATAAATGAATTAGAAGTATATGA

TTATGACCGTATTGGTTTAGTAGGAGCAAATGGTGCTGGAAAAAGCACTTTACTCAGGGTACTTTTAGGAGA

ATTAACTCCCCCAGGATGTAAAATGAATCGTCTGGGTGAACTTGCCTATATTCCCCAGTTGGACGAAGTAAC

TCTGCAGGAGGAAAAAGATTTTGCACTTGTAGGCAAGCTAGGTGTTGAGCAATTAAATATACAGACTATGAG

CGGTGGTGAAGAAACAAGGCTTAAAATAGCACAGGCCTTATCGGCACAGGTTCATGGTATTTTAGCGGATGA

ACCTACGAGCCATTTAGACCGTGAAGGAATTGATTTTCTAATAGGACAGCTAAAATATTTTACAGGTGCACT

GTTAGTTATTAGCCATGACCGCTATTTTCTTGATGAAATAGTAGATAAAATATGGGAACTGAAAGATGGCAA

AATCACTGAGTATTGGGGAAACTATTCTGATTATCTTCGTCAGAAAGAGGAAGAACGTAAGAGCCAAGCTGC

AGAATACGAACAATTTATTGCGGAACGTGCCCGATTGGAAAGGGCTGCGGAGGAAAAGCGAAAACAGGCTCG

TAAAATAGAACAGAAGGCAAAAGGTTCTTCAAAGAAAAAAAGTACTGAAGACGGAGGGCGTTTAGCTCATCA

AAAATCAATAGGAAGTAAGGAAAAAAAGATGTATAATGCTGCTAAAACCCTAGAGCACAGGATTGCGGCCTT

AGGAAAAGTAGAAGCTCCGGAAGGCATTCGCAGAATTCGTTTCAGGCAAAGTAAAGCATTGGAGCTCCATAA

TCCATACCCTATAGTCGGTGCAGAAATTAATAAAGTATTTGGGGATAAGGCTCTGTTTGAAAATGCATCTTT

TCAAATTCCGTTAGGAGCAAAAGTGGCGTTAACTGGTGGTAATGGAATCGGAAAAACAACTTTAATCCAAAT

GATCTTAAACCATGAAGAAGGAATTTCTATTTCGCCTAAGGCAAAAATAGGTTACTTTGCACAGAATGGTTA

CAAGTACAACAGTAATCAGAATGTTATGGAGTTTATGCAGAAGGATTGTGACTACAATATATCAGAAATTCG

TTCAGTGCTAGCATCTATGGGGTTCAAACAGAACGATATTGGAAAAAGTTTATCTGTTTTAAGCGGTGGAGA

AATTATAAAATTGTTGCTTGCTAAAATGCTCATGGGTAGATATAACATCCTAATAATGGATGAACCCAGTAA

CTTCCTTGACATACCAAGTTTAGAGGCTTTGGAAATACTAATGAAGGAGTACACCGGAACTATCGTGTTTAT

CACCCACGATAAACGATTACTCGAAAATGTAGCAGATGTAGTTTATGAAATTAGAGATAAGAAAATAAATCT

GAAACATTAAATTTAAGGTAGTCGCTGGTCAGTATAGTCTGTTCTGGTTGGCGACTCCATTGTTAAAGAGTA

TAAAGACTTTAGATTTTATGAATATTAAAAATAGGAACAGTCAATTGAACTGCTCCTATTTTTCTGCTAAAT

ATATTGTAGTTTTCTTATATGTATAATGATAGATTAGCGGATTCTCATCTACGGTACTTACTTCAAATATGA

AGAAGTGATCGCGGTTATCTCTGGACTTTTCCTTATTGAGGACAAAGTAATTCTTACGTGAAGTCGCCATTG

TTTTTAGGATATCATCAGTTAGGAAGGTCAATGGAATATTCATGTTAGAGTAGCGGTAGAAGTCACGTTCAA

AATCTTGGTAGCTCTCGCTATAATAGTCCATTTGTAGGTGATTACGCTGAAACTCAAGCTGATTCATAGAGC

ACCTCCTCGACAAGTTCAATACTAATAATGTCTTTTAATTTCAAATTGATGTGACCTGTTGTAGTTTTTATC

AAAATGAAATCTTTGGTCAGACTTGGTATTGTTCCAGTGTAGGAAACACGCTTGTTTTTTTCAATCACTTGA

ATGCGTGTGCGTAGCTGCCCGGCGTATACTTGACTGAGGAGTAATAATTTCTTCTCTAGTGATAAGTCAGAC

ATGTACGTTACTTTGTTTGTATCATCAGAGAGTGCTGATGCATGTTCAGATAGGAAAAAGCCCATCCATTTT

TGCATCTTTGTATCCTGGTACTCTCTTGCTGATTGAAATGGTAAATATGAACGGTCAATCATATCAAATCCT

TTCTATGCAGAGGCAAGGGTATTTTTATCAAATTGAATCGTAAAACCTTGAATTCCCCCACCTGTGTAACAT

TCTTTAAAGCGATTGATTACCTCAGTATAGATTATCACAGATGAGCTTGTTGGCTTAATGCTAAATGTAAAT

TCCAATGGTAATCGGTTTTCAGATTTAGCATGTACTAGTCGTATCGATATTTCAGTTGTTTTGAGTTTTCTC

TGACGAAGTTTTGAAGTTGCTGTTTCAACGATTCCATGTAAAAATCTTTCAAGCATTTCAATATCATTACAT

CCTTTGCTACGGATTTCTGAAAATTGTACTGTATTTTTTTCTTGTTTCATTTTAATCCCTCCAATCCACCCG

CGGAATGACCACCGATAAGTTTACTGCGTTCAATATTTCTGGAACCTTCAGTTAGGACGGTTCCTTTTTGTA

TGGCTAAAAAACCAAACTGTTCTCTGACAACATCAATAGCTGTCTGAAGTCTATTATCTTTTTCAATTTGTT

CTACATCATCAAAGAGTGATAGTAGAGTATAGCTTTCATCTACGAAGCCACTATAAGATACACCAATTTGTC

TCACTGCACCAGAGGTGTATTTTTTCGGAATAATACAAGTACATGACTCACCATTGTTTTGGGGAGATTTG

CGGGTTCAATTTTATTCTGAGCATTTATAGATTTTTTCATCTCAGTCCTAGAATAGCCAATATGAATAGAAA

CGACAGTAGTCAATACTAGGGCTACTGTTCCGTTCCACAGTATCATTTAAAAATCATT-TTCACACCCTTTC

GTCTATTAGTATAGAAGAAAGCTCTCAGCACA

>GA44500/Mega-1.III

CATGTTGAGGCGGTAAGTTTGCTAGTCAAGGAGTAAAACGACGAAGATTAGCATTTACTTCCGCCCATGCGA

TAGCTGTCCGTGATTGACAAGTGCTAGCACGCAGACAGAACGGAGATAGCGAACCGCTGAGTGTGTCGCTCT

GCTCGTAAAAGCTTAGAAACCTTTGAACGAAAGGGATAATGAAAGCCTTGATTGCAAGGCTTTTTGCTTTAT

GGTGGGTAAGTATCAGAGTGAGAAAATTTTTGGAATGAGTAGAAGTGATAGCTAGAAATTATCAGTTTCTA

TTTCCATTTACCCTGTGGGTACGTGTTTGTTTCCATTGACAAGGAGTTTGTGGGAATAGAAATGT

ACCCACCTTGTTTGAATCAAGTGAAGTGTAGTTGAAGGAAATCTGTTGAAAGCAATACTTCATTTTACCG

AATAAGTAATAATTTAGGCAACTTCAAATCGATTAAAAAAAACTATTTTAAAGGTTAAGAGTAGACAAAA

ATTGTCCACTCTTTTTTGCAAACTCAATTTATCAATAAATGAAATGAGGGAATGTAAAATG

AAATATTTTGAGGTTGAGTTAGAAAATCCTGATGAATTTTTAAAACTACAAACAGAAGATTTTGTGAAAG

CTAATCGCTTGCTACTAAGGAAGATAATCCAGAGCGTTACAGTCTATGAAGAAAACTTCGTCATATCCTT

TAAATCTGGCATCGAATTGGAAGTATGAGTCTCATTCCATAACTTTTATATTGAACATATCATCTTTTTGTG

TTATACTATAAATTGATATAAACAAAGATGTAGGAGGAACCGAAACTATGACAGCCTCAATGCGTTTAAGAT

AAGCTGGCAATAAAAAAAGCAGAATCTATACCCGATGATAGGCTTTTTTGTTGTGCTTATTTATACGATATT

GAGCATTCATTAGTTACGGTGAGGATATTGGTTATTTAACTATACCTTTATTTAACTATGTCTTTAATATGA

ATGTTTCCAAATTGTATGTATGCAGACCAAAAGCCACATTGTGGGGTTTGGCCTGCATTTTTTTTGCCTA

GAATGCTATTCAAAATAGAAATTCAAGCAAAATAATATGCAGGAGATAATATAAATGGAAAAATACAACAAT

TGGAAACGAAAATTTTATGCAATATGGGCAGGGCAAGCAGTATCATTAATCACTAGTGCCATCCTGCAAATG

GCGATTATTTTTTACCTTACAGAAAAAACAGGATCTGCGATGGTCTTGTCTATGGCTTCATTAGTAGGTTTT

TTACCCTATGCGATTTTGGGACCTGCCATTGGTGTGCTAGTGGATCGTCATGATAGGAAGAAGATAATGATT

GGTGCCGATTTAATTATCGCAGCAGCTGGTGCAGTGCTTGCTATTGTTGCATTCTGTATGGAGCTACCTGTC

TGGATGATTATGATAGTATTGTTTATCCGTAGCATTGGAACAGCTTTTCATACCCCAGCACTCAATGCGGTT

ACACCACTTTTAGTACCAGAAGAACAGCTAACGAAATGCGCAGGCTATAGTCAGTCTTTGCAGTCTATAAGC

TATATTGTTAGTCCGGCAGTTGCAGCACTCTTATACTCCGTTTGGGATTTAAATGCTATTATTGCCATCGAC

GTATTGGGTGCTGTGATTGCATCTATTACGGTAGCAATTGTACGTATACCTAAGCTGGGTAATCAAGTGCAA

AGTTTAGAACCAAATTTCATAAGGGAGATGAAAGAAGGAGTTGTGGTTCTGAGACAAAACAAAGGATTGTTT

GCCTTATTACTCTTAGGAACACTATATACTTTTGTTTATATGCCAATCAATGCACTATTTCCTTTAATAAGC

ATGGAACACTTTAATGGAACGCCTGTGCATATTTCTATTACGGAAATTTCCTTTGCATTTGGGATGCTAGCA

GGAGGCTTATTATTAGGAAGATTAGGGGGCTTCGAAAAGCATGTATTACTAATAACAAGTTCATTTTTTATA

ATGGGGACCAGTTTAGCCGTTTCGGGAATACTTCCTCCAAATGGATTTGTAATATTCGTAGTTTGCTGTGCA

ATAATGGGGCTTTCGGTGCCATTTTATAGCGGTGTGCAAACAGCTCTTTTTCAGGAGAAAATTAAGCCTGAA

TATTTAGGACGTGTATTTTCTTTGATCGGAAGTATCATGTCACTTGCTATGCCAATTGGGTTAATTCTTTCT

GGATTCTTTGCTGATAAAATCGGTGTAAATCATTGGTTTTTACTATCAGGTATTTTAATTATTGGCATTGCT

ATAGTTTGCCAAATGATAACTGAGGTTAGAAAATTAGATTTAAAATAAACAATATTGGAGGAATATTTATGT

ATCTTATTTTCATGTAACTCTTCCTGCTAAAATCGCAGGGTTTTCCCTGCATACAAGCAAATGAAAGCATGC

GATTATAGACAGGAGGAAATGTTATGGAATTAATATT

AAAAGCAAAAGACATTCGTGTGGAATTCAAAGGACGCGATGTTTTAGATATAAATGAATTAGAAGTATATGA

TTATGACCGTATTGGTTTAGTAGGAGCAAATGGTGCTGGAAAAAGCACTTTACTCAGGGTACTTTTAGGAGA

ATTAACTCCCCCAGGATGTAAAATGAATCGTCTGGGTGAACTTGCCTATATTCCCCAGTTGGACGAAGTAAC

TCTGCAGGAGGAAAAAGATTTTGCACTTGTAGGCAAGCTAGGTGTTGAGCAATTAAATATACAGACTATGAG

CGGTGGTGAAGAAACAAGGCTTAAAATAGCACAGGCCTTATCGGCACAGGTTCATGGTATTTTAGCGGATGA

ACCTACGAGCCATTTAGACCGTGAAGGAATTGATTTTCTAATAGGACAGCTAAAATATTTTACAGGTGCACT

GTTAGTTATTAGCCATGACCGCTATTTTCTTGATGAAATAGTAGATAAAATATGGGAACTGAAAGATGGCAA

AATCACTGAGTATTGGGGAAACTATTCTGATTATCTTCGTCAGAAAGAGGAAGAACGTAAGAGCCAAGCTGC

AGAATACGAACAATTTATTGCGGAACGTGCCCGATTGGAAAGGGCTGCGGAGGAAAAGCGAAAACAGGCTCG

TAAAATAGAACAGAAGGCAAAAGGTTCTTCAAAGAAAAAAAGTACTGAAGACGGAGGGCGTTTAGCTCATCA

AAAATCAATAGGAAGTAAGGAAAAAAAGATGTATAATGCTGCTAAAACCCTAGAGCACAGGATTGCGGCCTT

AGGAAAAGTAGAAGCTCCGGAAGGCATTCGCAGAATTCGTTTCAGGCAAAGTAAAGCATTGGAGCTCCATAA

TCCATACCCTATAGTCGGTGCAGAAATTAATAAAGTATTTGGGGATAAGGCTCTGTTTGAAAATGCATCTTT

TCAAATTCCGTTAGGAGCAAAAGTGGCGTTAACTGGTGGTAATGGAATCGGAAAAACAACTTTAATCCAAAT

GATCTTAAACCATGAAGAAGGAATTTCTATTTCGCCTAAGGCAAAAATAGGTTACTTTGCACAGAATGGTTA

CAAGTACAACAGTAATCAGAATGTTATGGAGTTTATGCAGAAGGATTGTGACTACAATATATCAGAAATTCG

TTCAGTGCTAGCATCTATGGGGTTCAAACAGAACGATATTGGAAAAAGTTTATCTGTTTTAAGCGGTGGAGA

AATTATAAAATTGTTGCTTGCTAAAATGCTCATGGGTAGATATAACATCCTAATAATGGATGAACCCAGTAA

CTTCCTTGACATACCAAGTTTAGAGGCTTTGGAAATACTAATGAAGGAGTACACCGGAACTATCGTGTTTAT

CACCCACGATAAACGATTACTCGAAAATGTAGCAGATGTAGTTTATGAAATTAGAGATAAGAAAATAAATCT

GAAACATTAAATTTAAGGTAGTCGCTGGTCAGTATAGTCTGTTCTGGTTGGCGACTCCATTGTTAAAGAGTA

TAAAGACTTTAGATTTTATGAATATTAAAAATAGGAACAGTCAATTGAACTGCTCCTATTTTTCTGCTAAAT

ATATTGTAGTTTTCTTATATGTATAATGATAGATTAGCGGATTCTCATCTACGGTACTTACTTCAAATATGA

AGAAGTGATCGCGGTTATCTCTGGACTTTTCCTTATTGAGGACAAAGTAATTCTTACGTGAAGTCGCCATTG

TTTTTAGGATATCATCAGTTAGGAAGGTCAATGGAATATTCATGTTAGAGTAGCGGTAGAAGTCACGTTCAA

AATCTTGGTAGCTCTCGCTATAATAGTCCATTTGTAGGTGATTACGCTGAAACTCAAGCTGATTCATAGAGC

ACCTCCTCGACAAGTTCAATACTAATAATGTCTTTTAATTTCAAATTGATGTGACCTGTTGTAGTTTTTATC

AAAATGAAATCTTTGGTCAGACTTGGTATTGTTCCAGTGTAGGAAACACGCTTGTTTTTTTCAATCACTTGA

ATGCGTGTGCGTAGCTGCCCGGCGTATACTTGACTGAGGAGTAATAATTTCTTCTCTAGTGATAAGTCAGAC

ATGTACGTTACTTTGTTTGTATCATCAGAGAGTGCTGATGCATGTTCAGATAGGAAAAAGCCCATCCATTTT

TGCATCTTTGTATCCTGGTACTCTCTTGCTGATTGAAATGGTAAATATGAACGGTCAATCATATCAAATCCT

TTCTATGCAGAGGCAAGGGTATTTTTATCAAATTGAATCGTAAAACCTTGAATTCCCCCACCTGTGTAACAT

TCTTTAAAGCGATTGATTACCTCAGTATAGATTATCACAGATGAGCTTGTTGGCTTAATGCTAAATGTAAAT

TCCAATGGTAATCGGTTTTCAGATTTAGCATGTACTAGTCGTATCGATATTTCAGTTGTTTTGAGTTTTCTC

TGACGAAGTTTTGAAGTTGCTGTTTCAACGATTCCATGTAAAAATCTTTCAAGCATTTCAATATCATTACAT

CCTTTGCTACGGATTTCTGAAAATTGTACTGTATTTTTTTCTTGTTTCATTTTAATCCCTCCAATCCACCCG

CGGAATGACCACCGATAAGTTTACTGCGTTCAATATTTCTGGAACCTTCAGTTAGGACGGTTCCTTTTTGTA

TGGCTAAAAAACCAAACTGTTCTCTGACAACATCAATAGCTGTCTGAAGTCTATTATCTTTTTCAATTTGTT

CTACATCATCAAAGAGTGATAGTAGAGTATAGCTTTCATCTACGAAGCCACTATAAGATACACCAATTTGTC

TCACTGCACCAGAGGTGTATTTTTTCGGAATAATACAAGTACATGACTCACCATTGTTTTGGGGAGATTTG

CGGGTTCAATTTTATTCTGAGCATTTATAGATTTTTTCATCTCAGTCCTAGAATAGCCAATATGAATAGAAA

CGACAGTAGTCAATACTAGGGCTACTGTTCCGTTCCACAGTATCATTTAAAAATCATT-TTCACACCCTTTC

GTCTATTAGTATAGAAGAAAGCTCTCAGCACA

>GA44378/Mega-1.III

CATGTTGAGGCGGTAAGTTTGCTAGTCAAGGAGTAAAACGACGAAGATTAGCATTTACTTCCGCCCATGCGA

TAGCTGTCCGTGATTGACAAGTGCTAGCACGCAGACAGAACGGAGATAGCGAACCGCTGAGTGTGTCGCTCT

GCTCGTAAAAGCTTAGAAACCTTTGAACGAAAGGGATAATGAAAGCCTTGATTGCAAGGCTTTTTGCTTTAT

GGTGGGTAAGTATCAGAGTGAGAAAATTTTTGGAATGAGTAGAAGTGATAGCTAGAAATTATCAGTTTCTA

TTTCCATTTACCCTGTGGGTACGTGTTTGTTTCCATTGACAAGGAGTTTGTGGGAATAGAAATGT

ACCCACCTTGTTTGAATCAAGTGAAGTGTAGTTGAAGGAAATCTGTTGAAAGCAATACTTCATTTTACCG

AATAAGTAATAATTTAGGCAACTTCAAATCGATTAAAAAAAACTATTTTAAAGGTTAAGAGTAGACAAAA

ATTGTCCACTCTTTTTTGCAAACTCAATTTATCAATAAATGAAATGAGGGAATGTAAAATG

AAATATTTTGAGGTTGAGTTAGAAAATCCTGATGAATTTTTAAAACTACAAACAGAAGATTTTGTGAAAG

CTAATCGCTTGCTACTAAGGAAGATAATCCAGAGCGTTACAGTCTATGAAGAAAACTTCGTCATATCCTT

TAAATCTGGCATCGAATTGGAAGTATGAGTCTCATTCCATAACTTTTATATTGAACATATCATCTTTTTGTG

TTATACTATAAATTGATATAAACAAAGATGTAGGAGGAACCGAAACTATGACAGCCTCAATGCGTTTAAGAT

AAGCTGGCAATAAAAAAAGCAGAATCTATACCCGATGATAGGCTTTTTTGTTGTGCTTATTTATACGATATT

GAGCATTCATTAGTTACGGTGAGGATATTGGTTATTTAACTATACCTTTATTTAACTATGTCTTTAATATGA

ATGTTTCCAAATTGTATGTATGCAGACCAAAAGCCACATTGTGGGGTTTGGCCTGCATTTTTTTTGCCTA

GAATGCTATTCAAAATAGAAATTCAAGCAAAATAATATGCAGGAGATAATATAAATGGAAAAATACAACAAT

TGGAAACGAAAATTTTATGCAATATGGGCAGGGCAAGCAGTATCATTAATCACTAGTGCCATCCTGCAAATG

GCGATTATTTTTTACCTTACAGAAAAAACAGGATCTGCGATGGTCTTGTCTATGGCTTCATTAGTAGGTTTT

TTACCCTATGCGATTTTGGGACCTGCCATTGGTGTGCTAGTGGATCGTCATGATAGGAAGAAGATAATGATT

GGTGCCGATTTAATTATCGCAGCAGCTGGTGCAGTGCTTGCTATTGTTGCATTCTGTATGGAGCTACCTGTC

TGGATGATTATGATAGTATTGTTTATCCGTAGCATTGGAACAGCTTTTCATACCCCAGCACTCAATGCGGTT

ACACCACTTTTAGTACCAGAAGAACAGCTAACGAAATGCGCAGGCTATAGTCAGTCTTTGCAGTCTATAAGC

TATATTGTTAGTCCGGCAGTTGCAGCACTCTTATACTCCGTTTGGGATTTAAATGCTATTATTGCCATCGAC

GTATTGGGTGCTGTGATTGCATCTATTACGGTAGCAATTGTACGTATACCTAAGCTGGGTAATCAAGTGCAA

AGTTTAGAACCAAATTTCATAAGGGAGATGAAAGAAGGAGTTGTGGTTCTGAGACAAAACAAAGGATTGTTT

GCCTTATTACTCTTAGGAACACTATATACTTTTGTTTATATGCCAATCAATGCACTATTTCCTTTAATAAGC

ATGGAACACTTTAATGGAACGCCTGTGCATATTTCTATTACGGAAATTTCCTTTGCATTTGGGATGCTAGCA

GGAGGCTTATTATTAGGAAGATTAGGGGGCTTCGAAAAGCATGTATTACTAATAACAAGTTCATTTTTTATA

ATGGGGACCAGTTTAGCCGTTTCGGGAATACTTCCTCCAAATGGATTTGTAATATTCGTAGTTTGCTGTGCA

ATAATGGGGCTTTCGGTGCCATTTTATAGCGGTGTGCAAACAGCTCTTTTTCAGGAGAAAATTAAGCCTGAA

TATTTAGGACGTGTATTTTCTTTGATCGGAAGTATCATGTCACTTGCTATGCCAATTGGGTTAATTCTTTCT

GGATTCTTTGCTGATAAAATCGGTGTAAATCATTGGTTTTTACTATCAGGTATTTTAATTATTGGCATTGCT

ATAGTTTGCCAAATGATAACTGAGGTTAGAAAATTAGATTTAAAATAAACAATATTGGAGGAATATTTATGT

ATCTTATTTTCATGTAACTCTTCCTGCTAAAATCGCAGGGTTTTCCCTGCATACAAGCAAATGAAAGCATGC

GATTATAGACAGGAGGAAATGTTATGGAATTAATATT

AAAAGCAAAAGACATTCGTGTGGAATTCAAAGGACGCGATGTTTTAGATATAAATGAATTAGAAGTATATGA

TTATGACCGTATTGGTTTAGTAGGAGCAAATGGTGCTGGAAAAAGCACTTTACTCAGGGTACTTTTAGGAGA

ATTAACTCCCCCAGGATGTAAAATGAATCGTCTGGGTGAACTTGCCTATATTCCCCAGTTGGACGAAGTAAC

TCTGCAGGAGGAAAAAGATTTTGCACTTGTAGGCAAGCTAGGTGTTGAGCAATTAAATATACAGACTATGAG

CGGTGGTGAAGAAACAAGGCTTAAAATAGCACAGGCCTTATCGGCACAGGTTCATGGTATTTTAGCGGATGA

ACCTACGAGCCATTTAGACCGTGAAGGAATTGATTTTCTAATAGGACAGCTAAAATATTTTACAGGTGCACT

GTTAGTTATTAGCCATGACCGCTATTTTCTTGATGAAATAGTAGATAAAATATGGGAACTGAAAGATGGCAA

AATCACTGAGTATTGGGGAAACTATTCTGATTATCTTCGTCAGAAAGAGGAAGAACGTAAGAGCCAAGCTGC

AGAATACGAACAATTTATTGCGGAACGTGCCCGATTGGAAAGGGCTGCGGAGGAAAAGCGAAAACAGGCTCG

TAAAATAGAACAGAAGGCAAAAGGTTCTTCAAAGAAAAAAAGTACTGAAGACGGAGGGCGTTTAGCTCATCA

AAAATCAATAGGAAGTAAGGAAAAAAAGATGTATAATGCTGCTAAAACCCTAGAGCACAGGATTGCGGCCTT

AGGAAAAGTAGAAGCTCCGGAAGGCATTCGCAGAATTCGTTTCAGGCAAAGTAAAGCATTGGAGCTCCATAA

TCCATACCCTATAGTCGGTGCAGAAATTAATAAAGTATTTGGGGATAAGGCTCTGTTTGAAAATGCATCTTT

TCAAATTCCGTTAGGAGCAAAAGTGGCGTTAACTGGTGGTAATGGAATCGGAAAAACAACTTTAATCCAAAT

GATCTTAAACCATGAAGAAGGAATTTCTATTTCGCCTAAGGCAAAAATAGGTTACTTTGCACAGAATGGTTA

CAAGTACAACAGTAATCAGAATGTTATGGAGTTTATGCAGAAGGATTGTGACTACAATATATCAGAAATTCG

TTCAGTGCTAGCATCTATGGGGTTCAAACAGAACGATATTGGAAAAAGTTTATCTGTTTTAAGCGGTGGAGA

AATTATAAAATTGTTGCTTGCTAAAATGCTCATGGGTAGATATAACATCCTAATAATGGATGAACCCAGTAA

CTTCCTTGACATACCAAGTTTAGAGGCTTTGGAAATACTAATGAAGGAGTACACCGGAACTATCGTGTTTAT

CACCCACGATAAACGATTACTCGAAAATGTAGCAGATGTAGTTTATGAAATTAGAGATAAGAAAATAAATCT

GAAACATTAAATTTAAGGTAGTCGCTGGTCAGTATAGTCTGTTCTGGTTGGCGACTCCATTGTTAAAGAGTA

TAAAGACTTTAGATTTTATGAATATTAAAAATAGGAACAGTCAATTGAACTGCTCCTATTTTTCTGCTAAAT

ATATTGTAGTTTTCTTATATGTATAATGATAGATTAGCGGATTCTCATCTACGGTACTTACTTCAAATATGA

AGAAGTGATCGCGGTTATCTCTGGACTTTTCCTTATTGAGGACAAAGTAATTCTTACGTGAAGTCGCCATTG

TTTTTAGGATATCATCAGTTAGGAAGGTCAATGGAATATTCATGTTAGAGTAGCGGTAGAAGTCACGTTCAA

AATCTTGGTAGCTCTCGCTATAATAGTCCATTTGTAGGTGATTACGCTGAAACTCAAGCTGATTCATAGAGC

ACCTCCTCGACAAGTTCAATACTAATAATGTCTTTTAATTTCAAATTGATGTGACCTGTTGTAGTTTTTATC

AAAATGAAATCTTTGGTCAGACTTGGTATTGTTCCAGTGTAGGAAACACGCTTGTTTTTTTCAATCACTTGA

ATGCGTGTGCGTAGCTGCCCGGCGTATACTTGACTGAGGAGTAATAATTTCTTCTCTAGTGATAAGTCAGAC

ATGTACGTTACTTTGTTTGTATCATCAGAGAGTGCTGATGCATGTTCAGATAGGAAAAAGCCCATCCATTTT

TGCATCTTTGTATCCTGGTACTCTCTTGCTGATTGAAATGGTAAATATGAACGGTCAATCATATCAAATCCT

TTCTATGCAGAGGCAAGGGTATTTTTATCAAATTGAATCGTAAAACCTTGAATTCCCCCACCTGTGTAACAT

TCTTTAAAGCGATTGATTACCTCAGTATAGATTATCACAGATGAGCTTGTTGGCTTAATGCTAAATGTAAAT

TCCAATGGTAATCGGTTTTCAGATTTAGCATGTACTAGTCGTATCGATATTTCAGTTGTTTTGAGTTTTCTC

TGACGAAGTTTTGAAGTTGCTGTTTCAACGATTCCATGTAAAAATCTTTCAAGCATTTCAATATCATTACAT

CCTTTGCTACGGATTTCTGAAAATTGTACTGTATTTTTTTCTTGTTTCATTTTAATCCCTCCAATCCACCCG

CGGAATGACCACCGATAAGTTTACTGCGTTCAATATTTCTGGAACCTTCAGTTAGGACGGTTCCTTTTTGTA

TGGCTAAAAAACCAAACTGTTCTCTGACAACATCAATAGCTGTCTGAAGTCTATTATCTTTTTCAATTTGTT

CTACATCATCAAAGAGTGATAGTAGAGTATAGCTTTCATCTACGAAGCCACTATAAGATACACCAATTTGTC

TCACTGCACCAGAGGTGTATTTTTTCGGAATAATACAAGTACATGACTCACCATTGTTTTGGGGAGATTTG

CGGGTTCAATTTTATTCTGAGCATTTATAGATTTTTTCATCTCAGTCCTAGAATAGCCAATATGAATAGAAA

CGACAGTAGTCAATACTAGGGCTACTGTTCCGTTCCACAGTATCATTTAAAAATCATT-TTCACACCCTTTC

GTCTATTAGTATAGAAGAAAGCTCTCAGCACA

>GA47760/Mega-1.III

CATGTTGAGGCGGTAAGTTTGCTAGTCAAGGAGTAAAACGACGAAGATTAGCATTTACTTCCGCCCATGCGA

TAGCTGTCCGTGATTGACAAGTGCTAGCACGCAGACAGAACGGAGATAGCGAACCGCTGAGTGTGTCGCTCT

GCTCGTAAAAGCTTAGAAACCTTTGAACGAAAGGGATAATGAAAGCCTTGATTGCAAGGCTTTTTGCTTTAT

GGTGGGTAAGTATCAGAGTGAGAAAATTTTTGGAATGAGTAGAAGTGATAGCTAGAAATTATCAGTTTCTA

TTTCCATTTACCCTGTGGGTACGTGTTTGTTTCCATTGACAAGGAGTTTGTGGGAATAGAAATGT

ACCCACCTTGTTTGAATCAAGTGAAGTGTAGTTGAAGGAAATCTGTTGAAAGCAATACTTCATTTTACCG

AATAAGTAATAATTTAGGCAACTTCAAATCGATTAAAAAAAACTATTTTAAAGGTTAAGAGTAGACAAAA

ATTGTCCACTCTTTTTTGCAAACTCAATTTATCAATAAATGAAATGAGGGAATGTAAAATG

AAATATTTTGAGGTTGAGTTAGAAAATCCTGATGAATTTTTAAAACTACAAACAGAAGATTTTGTGAAAG

CTAATCGCTTGCTACTAAGGAAGATAATCCAGAGCGTTACAGTCTATGAAGAAAACTTCGTCATATCCTT

TAAATCTGGCATCGAATTGGAAGTATGAGTCTCATTCCATAACTTTTATATTGAACATATCATCTTTTTGTG

TTATACTATAAATTGATATAAACAAAGATGTAGGAGGAACCGAAACTATGACAGCCTCAATGCGTTTAAGAT

AAGCTGGCAATAAAAAAAGCAGAATCTATACCCGATGATAGGCTTTTTTGTTGTGCTTATTTATACGATATT

GAGCATTCATTAGTTACGGTGAGGATATTGGTTATTTAACTATACCTTTATTTAACTATGTCTTTAATATGA

ATGTTTCCAAATTGTATGTATGCAGACCAAAAGCCACATTGTGGGGTTTGGCCTGCATTTTTTTTGCCTA

GAATGCTATTCAAAATAGAAATTCAAGCAAAATAATATGCAGGAGATAATATAAATGGAAAAATACAACAAT

TGGAAACGAAAATTTTATGCAATATGGGCAGGGCAAGCAGTATCATTAATCACTAGTGCCATCCTGCAAATG

GCGATTATTTTTTACCTTACAGAAAAAACAGGATCTGCGATGGTCTTGTCTATGGCTTCATTAGTAGGTTTT

TTACCCTATGCGATTTTGGGACCTGCCATTGGTGTGCTAGTGGATCGTCATGATAGGAAGAAGATAATGATT

GGTGCCGATTTAATTATCGCAGCAGCTGGTGCAGTGCTTGCTATTGTTGCATTCTGTATGGAGCTACCTGTC

TGGATGATTATGATAGTATTGTTTATCCGTAGCATTGGAACAGCTTTTCATACCCCAGCACTCAATGCGGTT

ACACCACTTTTAGTACCAGAAGAACAGCTAACGAAATGCGCAGGCTATAGTCAGTCTTTGCAGTCTATAAGC

TATATTGTTAGTCCGGCAGTTGCAGCACTCTTATACTCCGTTTGGGATTTAAATGCTATTATTGCCATCGAC

GTATTGGGTGCTGTGATTGCATCTATTACGGTAGCAATTGTACGTATACCTAAGCTGGGTAATCAAGTGCAA

AGTTTAGAACCAAATTTCATAAGGGAGATGAAAGAAGGAGTTGTGGTTCTGAGACAAAACAAAGGATTGTTT

GCCTTATTACTCTTAGGAACACTATATACTTTTGTTTATATGCCAATCAATGCACTATTTCCTTTAATAAGC

ATGGAACACTTTAATGGAACGCCTGTGCATATTTCTATTACGGAAATTTCCTTTGCATTTGGGATGCTAGCA

GGAGGCTTATTATTAGGAAGATTAGGGGGCTTCGAAAAGCATGTATTACTAATAACAAGTTCATTTTTTATA

ATGGGGACCAGTTTAGCCGTTTCGGGAATACTTCCTCCAAATGGATTTGTAATATTCGTAGTTTGCTGTGCA

ATAATGGGGCTTTCGGTGCCATTTTATAGCGGTGTGCAAACAGCTCTTTTTCAGGAGAAAATTAAGCCTGAA

TATTTAGGACGTGTATTTTCTTTGATCGGAAGTATCATGTCACTTGCTATGCCAATTGGGTTAATTCTTTCT

GGATTCTTTGCTGATAAAATCGGTGTAAATCATTGGTTTTTACTATCAGGTATTTTAATTATTGGCATTGCT

ATAGTTTGCCAAATGATAACTGAGGTTAGAAAATTAGATTTAAAATAAACAATATTGGAGGAATATTTATGT

ATCTTATTTTCATGTAACTCTTCCTGCTAAAATCGCAGGGTTTTCCCTGCATACAAGCAAATGAAAGCATGC

GATTATAGACAGGAGGAAATGTTATGGAATTAATATT

AAAAGCAAAAGACATTCGTGTGGAATTCAAAGGACGCGATGTTTTAGATATAAATGAATTAGAAGTATATGA

TTATGACCGTATTGGTTTAGTAGGAGCAAATGGTGCTGGAAAAAGCACTTTACTCAGGGTACTTTTAGGAGA

ATTAACTCCCCCAGGATGTAAAATGAATCGTCTGGGTGAACTTGCCTATATTCCCCAGTTGGACGAAGTAAC

TCTGCAGGAGGAAAAAGATTTTGCACTTGTAGGCAAGCTAGGTGTTGAGCAATTAAATATACAGACTATGAG

CGGTGGTGAAGAAACAAGGCTTAAAATAGCACAGGCCTTATCGGCACAGGTTCATGGTATTTTAGCGGATGA

ACCTACGAGCCATTTAGACCGTGAAGGAATTGATTTTCTAATAGGACAGCTAAAATATTTTACAGGTGCACT

GTTAGTTATTAGCCATGACCGCTATTTTCTTGATGAAATAGTAGATAAAATATGGGAACTGAAAGATGGCAA

AATCACTGAGTATTGGGGAAACTATTCTGATTATCTTCGTCAGAAAGAGGAAGAACGTAAGAGCCAAGCTGC

AGAATACGAACAATTTATTGCGGAACGTGCCCGATTGGAAAGGGCTGCGGAGGAAAAGCGAAAACAGGCTCG

TAAAATAGAACAGAAGGCAAAAGGTTCTTCAAAGAAAAAAAGTACTGAAGACGGAGGGCGTTTAGCTCATCA

AAAATCAATAGGAAGTAAGGAAAAAAAGATGTATAATGCTGCTAAAACCCTAGAGCACAGGATTGCGGCCTT

AGGAAAAGTAGAAGCTCCGGAAGGCATTCGCAGAATTCGTTTCAGGCAAAGTAAAGCATTGGAGCTCCATAA

TCCATACCCTATAGTCGGTGCAGAAATTAATAAAGTATTTGGGGATAAGGCTCTGTTTGAAAATGCATCTTT

TCAAATTCCGTTAGGAGCAAAAGTGGCGTTAACTGGTGGTAATGGAATCGGAAAAACAACTTTAATCCAAAT

GATCTTAAACCATGAAGAAGGAATTTCTATTTCGCCTAAGGCAAAAATAGGTTACTTTGCACAGAATGGTTA

CAAGTACAACAGTAATCAGAATGTTATGGAGTTTATGCAGAAGGATTGTGACTACAATATATCAGAAATTCG

TTCAGTGCTAGCATCTATGGGGTTCAAACAGAACGATATTGGAAAAAGTTTATCTGTTTTAAGCGGTGGAGA

AATTATAAAATTGTTGCTTGCTAAAATGCTCATGGGTAGATATAACATCCTAATAATGGATGAACCCAGTAA

CTTCCTTGACATACCAAGTTTAGAGGCTTTGGAAATACTAATGAAGGAGTACACCGGAACTATCGTGTTTAT

CACCCACGATAAACGATTACTCGAAAATGTAGCAGATGTAGTTTATGAAATTAGAGATAAGAAAATAAATCT

GAAACATTAAATTTAAGGTAGTCGCTGGTCAGTATAGTCTGTTCTGGTTGGCGACTCCATTGTTAAAGAGTA

TAAAGACTTTAGATTTTATGAATATTAAAAATAGGAACAGTCAATTGAACTGCTCCTATTTTTCTGCTAAAT

ATATTGTAGTTTTCTTATATGTATAATGATAGATTAGCGGATTCTCATCTACGGTACTTACTTCAAATATGA

AGAAGTGATCGCGGTTATCTCTGGACTTTTCCTTATTGAGGACAAAGTAATTCTTACGTGAAGTCGCCATTG

TTTTTAGGATATCATCAGTTAGGAAGGTCAATGGAATATTCATGTTAGAGTAGCGGTAGAAGTCACGTTCAA

AATCTTGGTAGCTCTCGCTATAATAGTCCATTTGTAGGTGATTACGCTGAAACTCAAGCTGATTCATAGAGC

ACCTCCTCGACAAGTTCAATACTAATAATGTCTTTTAATTTCAAATTGATGTGACCTGTTGTAGTTTTTATC

AAAATGAAATCTTTGGTCAGACTTGGTATTGTTCCAGTGTAGGAAACACGCTTGTTTTTTTCAATCACTTGA

ATGCGTGTGCGTAGCTGCCCGGCGTATACTTGACTGAGGAGTAATAATTTCTTCTCTAGTGATAAGTCAGAC

ATGTACGTTACTTTGTTTGTATCATCAGAGAGTGCTGATGCATGTTCAGATAGGAAAAAGCCCATCCATTTT

TGCATCTTTGTATCCTGGTACTCTCTTGCTGATTGAAATGGTAAATATGAACGGTCAATCATATCAAATCCT

TTCTATGCAGAGGCAAGGGTATTTTTATCAAATTGAATCGTAAAACCTTGAATTCCCCCACCTGTGTAACAT

TCTTTAAAGCGATTGATTACCTCAGTATAGATTATCACAGATGAGCTTGTTGGCTTAATGCTAAATGTAAAT

TCCAATGGTAATCGGTTTTCAGATTTAGCATGTACTAGTCGTATCGATATTTCAGTTGTTTTGAGTTTTCTC

TGACGAAGTTTTGAAGTTGCTGTTTCAACGATTCCATGTAAAAATCTTTCAAGCATTTCAATATCATTACAT

CCTTTGCTACGGATTTCTGAAAATTGTACTGTATTTTTTTCTTGTTTCATTTTAATCCCTCCAATCCACCCG

CGGAATGACCACCGATAAGTTTACTGCGTTCAATATTTCTGGAACCTTCAGTTAGGACGGTTCCTTTTTGTA

TGGCTAAAAAACCAAACTGTTCTCTGACAACATCAATAGCTGTCTGAAGTCTATTATCTTTTTCAATTTGTT

CTACATCATCAAAGAGTGATAGTAGAGTATAGCTTTCATCTACGAAGCCACTATAAGATACACCAATTTGTC

TCACTGCACCAGAGGTGTATTTTTTCGGAATAATACAAGTACATGACTCACCATTGTTTTGGGGAGATTTG

CGGGTTCAATTTTATTCTGAGCATTTATAGATTTTTTCATCTCAGTCCTAGAATAGCCAATATGAATAGAAA

CGACAGTAGTCAATACTAGGGCTACTGTTCCGTTCCACAGTATCATTTAAAAATCATT-TTCACACCCTTTC

GTCTATTAGTATAGAAGAAAGCTCTCAGCACA

>GA04175/Mega-1.III

CATGTTGAGGCGGTAAGTTTGCTAGTCAAGGAGTAAAACGACGAAGATTAGCATTTACTTCCGCCCATGCGA

TAGCTGTCCGTGATTGACAAGTGCTAGCACGCAGACAGAACGGAGATAGCGAACCGCTGAGTGTGTCGCTCT

GCTCGTAAAAGCTTAGAAACCTTTGAACGAAAGGGATAATGAAAGCCTTGATTGCAAGGCTTTTTGCTTTAT

GGTGGGTAAGTATCAGAGTGAGAAAATTTTTGGAATGAGTAGAAGTGATAGCTAGAAATTATCAGTTTCTA

TTTCCATTTACCCTGTGGGTACGTGTTTGTTTCCATTGACAAGGAGTTTGTGGGAATAGAAATGT

ACCCACCTTGTTTGAATCAAGTGAAGTGTAGTTGAAGGAAATCTGTTGAAAGCAATACTTCATTTTACCG

AATAAGTAATAATTTAGGCAACTTCAAATCGATTAAAAAAAACTATTTTAAAGGTTAAGAGTAGACAAAA

ATTGTCCACTCTTTTTTGCAAACTCAATTTATCAATAAATGAAATGAGGGAATGTAAAATG

AAATATTTTGAGGTTGAGTTAGAAAATCCTGATGAATTTTTAAAACTACAAACAGAAGATTTTGTGAAAG

CTAATCGCTTGCTACTAAGGAAGATAATCCAGAGCGTTACAGTCTATGAAGAAAACTTCGTCATATCCTT

TAAATCTGGCATCGAATTGGAAGTATGAGTCTCATTCCATAACTTTTATATTGAACATATCATCTTTTTGTG

TTATACTATAAATTGATATAAACAAAGATGTAGGAGGAACCGAAACTATGACAGCCTCAATGCGTTTAAGAT

AAGCTGGCAATAAAAAAAGCAGAATCTATACCCGATGATAGGCTTTTTTGTTGTGCTTATTTATACGATATT

GAGCATTCATTAGTTACGGTGAGGATATTGGTTATTTAACTATACCTTTATTTAACTATGTCTTTAATATGA

ATGTTTCCAAATTGTATGTATGCAGACCAAAAGCCACATTGTGGGGTTTGGCCTGCATTTTTTTTGCCTA

GAATGCTATTCAAAATAGAAATTCAAGCAAAATAATATGCAGGAGATAATATAAATGGAAAAATACAACAAT

TGGAAACGAAAATTTTATGCAATATGGGCAGGGCAAGCAGTATCATTAATCACTAGTGCCATCCTGCAAATG

GCGATTATTTTTTACCTTACAGAAAAAACAGGATCTGCGATGGTCTTGTCTATGGCTTCATTAGTAGGTTTT

TTACCCTATGCGATTTTGGGACCTGCCATTGGTGTGCTAGTGGATCGTCATGATAGGAAGAAGATAATGATT

GGTGCCGATTTAATTATCGCAGCAGCTGGTGCAGTGCTTGCTATTGTTGCATTCTGTATGGAGCTACCTGTC

TGGATGATTATGATAGTATTGTTTATCCGTAGCATTGGAACAGCTTTTCATACCCCAGCACTCAATGCGGTT

ACACCACTTTTAGTACCAGAAGAACAGCTAACGAAATGCGCAGGCTATAGTCAGTCTTTGCAGTCTATAAGC

TATATTGTTAGTCCGGCAGTTGCAGCACTCTTATACTCCGTTTGGGATTTAAATGCTATTATTGCCATCGAC

GTATTGGGTGCTGTGATTGCATCTATTACGGTAGCAATTGTACGTATACCTAAGCTGGGTAATCAAGTGCAA

AGTTTAGAACCAAATTTCATAAGGGAGATGAAAGAAGGAGTTGTGGTTCTGAGACAAAACAAAGGATTGTTT

GCCTTATTACTCTTAGGAACACTATATACTTTTGTTTATATGCCAATCAATGCACTATTTCCTTTAATAAGC

ATGGAACACTTTAATGGAACGCCTGTGCATATTTCTATTACGGAAATTTCCTTTGCATTTGGGATGCTAGCA

GGAGGCTTATTATTAGGAAGATTAGGGGGCTTCGAAAAGCATGTATTACTAATAACAAGTTCATTTTTTATA

ATGGGGACCAGTTTAGCCGTTTCGGGAATACTTCCTCCAAATGGATTTGTAATATTCGTAGTTTGCTGTGCA

ATAATGGGGCTTTCGGTGCCATTTTATAGCGGTGTGCAAACAGCTCTTTTTCAGGAGAAAATTAAGCCTGAA

TATTTAGGACGTGTATTTTCTTTGATCGGAAGTATCATGTCACTTGCTATGCCAATTGGGTTAATTCTTTCT

GGATTCTTTGCTGATAAAATCGGTGTAAATCATTGGTTTTTACTATCAGGTATTTTAATTATTGGCATTGCT

ATAGTTTGCCAAATGATAACTGAGGTTAGAAAATTAGATTTAAAATAAACAATATTGGAGGAATATTTATGT

ATCTTATTTTCATGTAACTCTTCCTGCTAAAATCGCAGGGTTTTCCCTGCATACAAGCAAATGAAAGCATGC

GATTATAGACAGGAGGAAATGTTATGGAATTAATATT

AAAAGCAAAAGACATTCGTGTGGAATTCAAAGGACGCGATGTTTTAGATATAAATGAATTAGAAGTATATGA

TTATGACCGTATTGGTTTAGTAGGAGCAAATGGTGCTGGAAAAAGCACTTTACTCAGGGTACTTTTAGGAGA

ATTAACTCCCCCAGGATGTAAAATGAATCGTCTGGGTGAACTTGCCTATATTCCCCAGTTGGACGAAGTAAC

TCTGCAGGAGGAAAAAGATTTTGCACTTGTAGGCAAGCTAGGTGTTGAGCAATTAAATATACAGACTATGAG

CGGTGGTGAAGAAACAAGGCTTAAAATAGCACAGGCCTTATCGGCACAGGTTCATGGTATTTTAGCGGATGA

ACCTACGAGCCATTTAGACCGTGAAGGAATTGATTTTCTAATAGGACAGCTAAAATATTTTACAGGTGCACT

GTTAGTTATTAGCCATGACCGCTATTTTCTTGATGAAATAGTAGATAAAATATGGGAACTGAAAGATGGCAA

AATCACTGAGTATTGGGGAAACTATTCTGATTATCTTCGTCAGAAAGAGGAAGAACGTAAGAGCCAAGCTGC

AGAATACGAACAATTTATTGCGGAACGTGCCCGATTGGAAAGGGCTGCGGAGGAAAAGCGAAAACAGGCTCG

TAAAATAGAACAGAAGGCAAAAGGTTCTTCAAAGAAAAAAAGTACTGAAGACGGAGGGCGTTTAGCTCATCA

AAAATCAATAGGAAGTAAGGAAAAAAAGATGTATAATGCTGCTAAAACCCTAGAGCACAGGATTGCGGCCTT

AGGAAAAGTAGAAGCTCCGGAAGGCATTCGCAGAATTCGTTTCAGGCAAAGTAAAGCATTGGAGCTCCATAA

TCCATACCCTATAGTCGGTGCAGAAATTAATAAAGTATTTGGGGATAAGGCTCTGTTTGAAAATGCATCTTT

TCAAATTCCGTTAGGAGCAAAAGTGGCGTTAACTGGTGGTAATGGAATCGGAAAAACAACTTTAATCCAAAT

GATCTTAAACCATGAAGAAGGAATTTCTATTTCGCCTAAGGCAAAAATAGGTTACTTTGCACAGAATGGTTA

CAAGTACAACAGTAATCAGAATGTTATGGAGTTTATGCAGAAGGATTGTGACTACAATATATCAGAAATTCG

TTCAGTGCTAGCATCTATGGGGTTCAAACAGAACGATATTGGAAAAAGTTTATCTGTTTTAAGCGGTGGAGA

AATTATAAAATTGTTGCTTGCTAAAATGCTCATGGGTAGATATAACATCCTAATAATGGATGAACCCAGTAA

CTTCCTTGACATACCAAGTTTAGAGGCTTTGGAAATACTAATGAAGGAGTACACCGGAACTATCGTGTTTAT

CACCCACGATAAACGATTACTCGAAAATGTAGCAGATGTAGTTTATGAAATTAGAGATAAGAAAATAAATCT

GAAACATTAAATTTAAGGTAGTCGCTGGTCAGTATAGTCTGTTCTGGTTGGCGACTCCATTGTTAAAGAGTA

TAAAGACTTTAGATTTTATGAATATTAAAAATAGGAACAGTCAATTGAACTGCTCCTATTTTTCTGCTAAAT

ATATTGTAGTTTTCTTATATGTATAATGATAGATTAGCGGATTCTCATCTACGGTACTTACTTCAAATATGA

AGAAGTGATCGCGGTTATCTCTGGACTTTTCCTTATTGAGGACAAAGTAATTCTTACGTGAAGTCGCCATTG

TTTTTAGGATATCATCAGTTAGGAAGGTCAATGGAATATTCATGTTAGAGTAGCGGTAGAAGTCACGTTCAA

AATCTTGGTAGCTCTCGCTATAATAGTCCATTTGTAGGTGATTACGCTGAAACTCAAGCTGATTCATAGAGC

ACCTCCTCGACAAGTTCAATACTAATAATGTCTTTTAATTTCAAATTGATGTGACCTGTTGTAGTTTTTATC

AAAATGAAATCTTTGGTCAGACTTGGTATTGTTCCAGTGTAGGAAACACGCTTGTTTTTTTCAATCACTTGA

ATGCGTGTGCGTAGCTGCCCGGCGTATACTTGACTGAGGAGTAATAATTTCTTCTCTAGTGATAAGTCAGAC

ATGTACGTTACTTTGTTTGTATCATCAGAGAGTGCTGATGCATGTTCAGATAGGAAAAAGCCCATCCATTTT

TGCATCTTTGTATCCTGGTACTCTCTTGCTGATTGAAATGGTAAATATGAACGGTCAATCATATCAAATCCT

TTCTATGCAGAGGCAAGGGTATTTTTATCAAATTGAATCGTAAAACCTTGAATTCCCCCACCTGTGTAACAT

TCTTTAAAGCGATTGATTACCTCAGTATAGATTATCACAGATGAGCTTGTTGGCTTAATGCTAAATGTAAAT

TCCAATGGTAATCGGTTTTCAGATTTAGCATGTACTAGTCGTATCGATATTTCAGTTGTTTTGAGTTTTCTC

TGACGAAGTTTTGAAGTTGCTGTTTCAACGATTCCATGTAAAAATCTTTCAAGCATTTCAATATCATTACAT

CCTTTGCTACGGATTTCTGAAAATTGTACTGTATTTTTTTCTTGTTTCATTTTAATCCCTCCAATCCACCCG

CGGAATGACCACCGATAAGTTTACTGCGTTCAATATTTCTGGAACCTTCAGTTAGGACGGTTCCTTTTTGTA

TGGCTAAAAAACCAAACTGTTCTCTGACAACATCAATAGCTGTCTGAAGTCTATTATCTTTTTCAATTTGTT

CTACATCATCAAAGAGTGATAGTAGAGTATAGCTTTCATCTACGAAGCCACTATAAGATACACCAATTTGTC

TCACTGCACCAGAGGTGTATTTTTTCGGAATAATACAAGTACATGACTCACCATTGTTTTGGGGAGATTTG

CGGGTTCAATTTTATTCTGAGCATTTATAGATTTTTTCATCTCAGTCCTAGAATAGCCAATATGAATAGAAA

CGACAGTAGTCAATACTAGGGCTACTGTTCCGTTCCACAGTATCATTTAAAAATCATT-TTCACACCCTTTC

GTCTATTAGTATAGAAGAAAGCTCTCAGCACA

>GA04672/Mega-1.III

CATGTTGAGGCGGTAAGTTTGCTAGTCAAGGAGTAAAACGACGAAGATTAGCATTTACTTCCGCCCATGCGA

TAGCTGTCCGTGATTGACAAGTGCTAGCACGCAGACAGAACGGAGATAGCGAACCGCTGAGTGTGTCGCTCT

GCTCGTAAAAGCTTAGAAACCTTTGAACGAAAGGGATAATGAAAGCCTTGATTGCAAGGCTTTTTGCTTTAT

GGTGGGTAAGTATCAGAGTGAGAAAATTTTTGGAATGAGTAGAAGTGATAGCTAGAAATTATCAGTTTCTA

TTTCCATTTACCCTGTGGGTACGTGTTTGTTTCCATTGACAAGGAGTTTGTGGGAATAGAAATGT

ACCCACCTTGTTTGAATCAAGTGAAGTGTAGTTGAAGGAAATCTGTTGAAAGCAATACTTCATTTTACCG

AATAAGTAATAATTTAGGCAACTTCAAATCGATTAAAAAAAACTATTTTAAAGGTTAAGAGTAGACAAAA

ATTGTCCACTCTTTTTTGCAAACTCAATTTATCAATAAATGAAATGAGGGAATGTAAAATG

AAATATTTTGAGGTTGAGTTAGAAAATCCTGATGAATTTTTAAAACTACAAACAGAAGATTTTGTGAAAG

CTAATCGCTTGCTACTAAGGAAGATAATCCAGAGCGTTACAGTCTATGAAGAAAACTTCGTCATATCCTT

TAAATCTGGCATCGAATTGGAAGTATGAGTCTCATTCCATAACTTTTATATTGAACATATCATCTTTTTGTG

TTATACTATAAATTGATATAAACAAAGATGTAGGAGGAACCGAAACTATGACAGCCTCAATGCGTTTAAGAT

AAGCTGGCAATAAAAAAAGCAGAATCTATACCCGATGATAGGCTTTTTTGTTGTGCTTATTTATACGATATT

GAGCATTCATTAGTTACGGTGAGGATATTGGTTATTTAACTATACCTTTATTTAACTATGTCTTTAATATGA

ATGTTTCCAAATTGTATGTATGCAGACCAAAAGCCACATTGTGGGGTTTGGCCTGCATTTTTTTTGCCTA

GAATGCTATTCAAAATAGAAATTCAAGCAAAATAATATGCAGGAGATAATATAAATGGAAAAATACAACAAT

TGGAAACGAAAATTTTATGCAATATGGGCAGGGCAAGCAGTATCATTAATCACTAGTGCCATCCTGCAAATG

GCGATTATTTTTTACCTTACAGAAAAAACAGGATCTGCGATGGTCTTGTCTATGGCTTCATTAGTAGGTTTT

TTACCCTATGCGATTTTGGGACCTGCCATTGGTGTGCTAGTGGATCGTCATGATAGGAAGAAGATAATGATT

GGTGCCGATTTAATTATCGCAGCAGCTGGTGCAGTGCTTGCTATTGTTGCATTCTGTATGGAGCTACCTGTC

TGGATGATTATGATAGTATTGTTTATCCGTAGCATTGGAACAGCTTTTCATACCCCAGCACTCAATGCGGTT

ACACCACTTTTAGTACCAGAAGAACAGCTAACGAAATGCGCAGGCTATAGTCAGTCTTTGCAGTCTATAAGC

TATATTGTTAGTCCGGCAGTTGCAGCACTCTTATACTCCGTTTGGGATTTAAATGCTATTATTGCCATCGAC

GTATTGGGTGCTGTGATTGCATCTATTACGGTAGCAATTGTACGTATACCTAAGCTGGGTAATCAAGTGCAA

AGTTTAGAACCAAATTTCATAAGGGAGATGAAAGAAGGAGTTGTGGTTCTGAGACAAAACAAAGGATTGTTT

GCCTTATTACTCTTAGGAACACTATATACTTTTGTTTATATGCCAATCAATGCACTATTTCCTTTAATAAGC

ATGGAACACTTTAATGGAACGCCTGTGCATATTTCTATTACGGAAATTTCCTTTGCATTTGGGATGCTAGCA

GGAGGCTTATTATTAGGAAGATTAGGGGGCTTCGAAAAGCATGTATTACTAATAACAAGTTCATTTTTTATA

ATGGGGACCAGTTTAGCCGTTTCGGGAATACTTCCTCCAAATGGATTTGTAATATTCGTAGTTTGCTGTGCA

ATAATGGGGCTTTCGGTGCCATTTTATAGCGGTGTGCAAACAGCTCTTTTTCAGGAGAAAATTAAGCCTGAA

TATTTAGGACGTGTATTTTCTTTGATCGGAAGTATCATGTCACTTGCTATGCCAATTGGGTTAATTCTTTCT

GGATTCTTTGCTGATAAAATCGGTGTAAATCATTGGTTTTTACTATCAGGTATTTTAATTATTGGCATTGCT

ATAGTTTGCCAAATGATAACTGAGGTTAGAAAATTAGATTTAAAATAAACAATATTGGAGGAATATTTATGT

ATCTTATTTTCATGTAACTCTTCCTGCTAAAATCGCAGGGTTTTCCCTGCATACAAGCAAATGAAAGCATGC

GATTATAGACAGGAGGAAATGTTATGGAATTAATATT

AAAAGCAAAAGACATTCGTGTGGAATTCAAAGGACGCGATGTTTTAGATATAAATGAATTAGAAGTATATGA

TTATGACCGTATTGGTTTAGTAGGAGCAAATGGTGCTGGAAAAAGCACTTTACTCAGGGTACTTTTAGGAGA

ATTAACTCCCCCAGGATGTAAAATGAATCGTCTGGGTGAACTTGCCTATATTCCCCAGTTGGACGAAGTAAC

TCTGCAGGAGGAAAAAGATTTTGCACTTGTAGGCAAGCTAGGTGTTGAGCAATTAAATATACAGACTATGAG

CGGTGGTGAAGAAACAAGGCTTAAAATAGCACAGGCCTTATCGGCACAGGTTCATGGTATTTTAGCGGATGA

ACCTACGAGCCATTTAGACCGTGAAGGAATTGATTTTCTAATAGGACAGCTAAAATATTTTACAGGTGCACT

GTTAGTTATTAGCCATGACCGCTATTTTCTTGATGAAATAGTAGATAAAATATGGGAACTGAAAGATGGCAA

AATCACTGAGTATTGGGGAAACTATTCTGATTATCTTCGTCAGAAAGAGGAAGAACGTAAGAGCCAAGCTGC

AGAATACGAACAATTTATTGCGGAACGTGCCCGATTGGAAAGGGCTGCGGAGGAAAAGCGAAAACAGGCTCG

TAAAATAGAACAGAAGGCAAAAGGTTCTTCAAAGAAAAAAAGTACTGAAGACGGAGGGCGTTTAGCTCATCA

AAAATCAATAGGAAGTAAGGAAAAAAAGATGTATAATGCTGCTAAAACCCTAGAGCACAGGATTGCGGCCTT

AGGAAAAGTAGAAGCTCCGGAAGGCATTCGCAGAATTCGTTTCAGGCAAAGTAAAGCATTGGAGCTCCATAA

TCCATACCCTATAGTCGGTGCAGAAATTAATAAAGTATTTGGGGATAAGGCTCTGTTTGAAAATGCATCTTT

TCAAATTCCGTTAGGAGCAAAAGTGGCGTTAACTGGTGGTAATGGAATCGGAAAAACAACTTTAATCCAAAT

GATCTTAAACCATGAAGAAGGAATTTCTATTTCGCCTAAGGCAAAAATAGGTTACTTTGCACAGAATGGTTA

CAAGTACAACAGTAATCAGAATGTTATGGAGTTTATGCAGAAGGATTGTGACTACAATATATCAGAAATTCG

TTCAGTGCTAGCATCTATGGGGTTCAAACAGAACGATATTGGAAAAAGTTTATCTGTTTTAAGCGGTGGAGA

AATTATAAAATTGTTGCTTGCTAAAATGCTCATGGGTAGATATAACATCCTAATAATGGATGAACCCAGTAA

CTTCCTTGACATACCAAGTTTAGAGGCTTTGGAAATACTAATGAAGGAGTACACCGGAACTATCGTGTTTAT

CACCCACGATAAACGATTACTCGAAAATGTAGCAGATGTAGTTTATGAAATTAGAGATAAGAAAATAAATCT

GAAACATTAAATTTAAGGTAGTCGCTGGTCAGTATAGTCTGTTCTGGTTGGCGACTCCATTGTTAAAGAGTA

TAAAGACTTTAGATTTTATGAATATTAAAAATAGGAACAGTCAATTGAACTGCTCCTATTTTTCTGCTAAAT

ATATTGTAGTTTTCTTATATGTATAATGATAGATTAGCGGATTCTCATCTACGGTACTTACTTCAAATATGA

AGAAGTGATCGCGGTTATCTCTGGACTTTTCCTTATTGAGGACAAAGTAATTCTTACGTGAAGTCGCCATTG

TTTTTAGGATATCATCAGTTAGGAAGGTCAATGGAATATTCATGTTAGAGTAGCGGTAGAAGTCACGTTCAA

AATCTTGGTAGCTCTCGCTATAATAGTCCATTTGTAGGTGATTACGCTGAAACTCAAGCTGATTCATAGAGC

ACCTCCTCGACAAGTTCAATACTAATAATGTCTTTTAATTTCAAATTGATGTGACCTGTTGTAGTTTTTATC

AAAATGAAATCTTTGGTCAGACTTGGTATTGTTCCAGTGTAGGAAACACGCTTGTTTTTTTCAATCACTTGA

ATGCGTGTGCGTAGCTGCCCGGCGTATACTTGACTGAGGAGTAATAATTTCTTCTCTAGTGATAAGTCAGAC

ATGTACGTTACTTTGTTTGTATCATCAGAGAGTGCTGATGCATGTTCAGATAGGAAAAAGCCCATCCATTTT

TGCATCTTTGTATCCTGGTACTCTCTTGCTGATTGAAATGGTAAATATGAACGGTCAATCATATCAAATCCT

TTCTATGCAGAGGCAAGGGTATTTTTATCAAATTGAATCGTAAAACCTTGAATTCCCCCACCTGTGTAACAT

TCTTTAAAGCGATTGATTACCTCAGTATAGATTATCACAGATGAGCTTGTTGGCTTAATGCTAAATGTAAAT

TCCAATGGTAATCGGTTTTCAGATTTAGCATGTACTAGTCGTATCGATATTTCAGTTGTTTTGAGTTTTCTC

TGACGAAGTTTTGAAGTTGCTGTTTCAACGATTCCATGTAAAAATCTTTCAAGCATTTCAATATCATTACAT

CCTTTGCTACGGATTTCTGAAAATTGTACTGTATTTTTTTCTTGTTTCATTTTAATCCCTCCAATCCACCCG

CGGAATGACCACCGATAAGTTTACTGCGTTCAATATTTCTGGAACCTTCAGTTAGGACGGTTCCTTTTTGTA

TGGCTAAAAAACCAAACTGTTCTCTGACAACATCAATAGCTGTCTGAAGTCTATTATCTTTTTCAATTTGTT

CTACATCATCAAAGAGTGATAGTAGAGTATAGCTTTCATCTACGAAGCCACTATAAGATACACCAATTTGTC

TCACTGCACCAGAGGTGTATTTTTTCGGAATAATACAAGTACATGACTCACCATTGTTTTGGGGAGATTTG

CGGGTTCAATTTTATTCTGAGCATTTATAGATTTTTTCATCTCAGTCCTAGAATAGCCAATATGAATAGAAA

CGACAGTAGTCAATACTAGGGCTACTGTTCCGTTCCACAGTATCATTTAAAAATCATT-TTCACACCCTTTC

GTCTATTAGTATAGAAGAAAGCTCTCAGCACA

>GA17301/Mega-1.III

CATGTTGAGGCGGTAAGTTTGCTAGTCAAGGAGTAAAACGACGAAGATTAGCATTTACTTCCGCCCATGCGA

TAGCTGTCCGTGATTGACAAGTGCTAGCACGCAGACAGAACGGAGATAGCGAACCGCTGAGTGTGTCGCTCT

GCTCGTAAAAGCTTAGAAACCTTTGAACGAAAGGGATAATGAAAGCCTTGATTGCAAGGCTTTTTGCTTTAT

GGTGGGTAAGTATCAGAGTGAGAAAATTTTTGGAATGAGTAGAAGTGATAGCTAGAAATTATCAGTTTCTA

TTTCCATTTACCCTGTGGGTACGTGTTTGTTTCCATTGACAAGGAGTTTGTGGGAATAGAAATGT

ACCCACCTTGTTTGAATCAAGTGAAGTGTAGTTGAAGGAAATCTGTTGAAAGCAATACTTCATTTTACCG

AATAAGTAATAATTTAGGCAACTTCAAATCGATTAAAAAAAACTATTTTAAAGGTTAAGAGTAGACAAAA

ATTGTCCACTCTTTTTTGCAAACTCAATTTATCAATAAATGAAATGAGGGAATGTAAAATG

AAATATTTTGAGGTTGAGTTAGAAAATCCTGATGAATTTTTAAAACTACAAACAGAAGATTTTGTGAAAG

CTAATCGCTTGCTACTAAGGAAGATAATCCAGAGCGTTACAGTCTATGAAGAAAACTTCGTCATATCCTT

TAAATCTGGCATCGAATTGGAAGTATGAGTCTCATTCCATAACTTTTATATTGAACATATCATCTTTTTGTG

TTATACTATAAATTGATATAAACAAAGATGTAGGAGGAACCGAAACTATGACAGCCTCAATGCGTTTAAGAT

AAGCTGGCAATAAAAAAAGCAGAATCTATACCCGATGATAGGCTTTTTTGTTGTGCTTATTTATACGATATT

GAGCATTCATTAGTTACGGTGAGGATATTGGTTATTTAACTATACCTTTATTTAACTATGTCTTTAATATGA

ATGTTTCCAAATTGTATGTATGCAGACCAAAAGCCACATTGTGGGGTTTGGCCTGCATTTTTTTTGCCTA

GAATGCTATTCAAAATAGAAATTCAAGCAAAATAATATGCAGGAGATAATATAAATGGAAAAATACAACAAT

TGGAAACGAAAATTTTATGCAATATGGGCAGGGCAAGCAGTATCATTAATCACTAGTGCCATCCTGCAAATG

GCGATTATTTTTTACCTTACAGAAAAAACAGGATCTGCGATGGTCTTGTCTATGGCTTCATTAGTAGGTTTT

TTACCCTATGCGATTTTGGGACCTGCCATTGGTGTGCTAGTGGATCGTCATGATAGGAAGAAGATAATGATT

GGTGCCGATTTAATTATCGCAGCAGCTGGTGCAGTGCTTGCTATTGTTGCATTCTGTATGGAGCTACCTGTC

TGGATGATTATGATAGTATTGTTTATCCGTAGCATTGGAACAGCTTTTCATACCCCAGCACTCAATGCGGTT

ACACCACTTTTAGTACCAGAAGAACAGCTAACGAAATGCGCAGGCTATAGTCAGTCTTTGCAGTCTATAAGC

TATATTGTTAGTCCGGCAGTTGCAGCACTCTTATACTCCGTTTGGGATTTAAATGCTATTATTGCCATCGAC

GTATTGGGTGCTGTGATTGCATCTATTACGGTAGCAATTGTACGTATACCTAAGCTGGGTAATCAAGTGCAA

AGTTTAGAACCAAATTTCATAAGGGAGATGAAAGAAGGAGTTGTGGTTCTGAGACAAAACAAAGGATTGTTT

GCCTTATTACTCTTAGGAACACTATATACTTTTGTTTATATGCCAATCAATGCACTATTTCCTTTAATAAGC

ATGGAACACTTTAATGGAACGCCTGTGCATATTTCTATTACGGAAATTTCCTTTGCATTTGGGATGCTAGCA

GGAGGCTTATTATTAGGAAGATTAGGGGGCTTCGAAAAGCATGTATTACTAATAACAAGTTCATTTTTTATA

ATGGGGACCAGTTTAGCCGTTTCGGGAATACTTCCTCCAAATGGATTTGTAATATTCGTAGTTTGCTGTGCA

ATAATGGGGCTTTCGGTGCCATTTTATAGCGGTGTGCAAACAGCTCTTTTTCAGGAGAAAATTAAGCCTGAA

TATTTAGGACGTGTATTTTCTTTGATCGGAAGTATCATGTCACTTGCTATGCCAATTGGGTTAATTCTTTCT

GGATTCTTTGCTGATAAAATCGGTGTAAATCATTGGTTTTTACTATCAGGTATTTTAATTATTGGCATTGCT

ATAGTTTGCCAAATGATAACTGAGGTTAGAAAATTAGATTTAAAATAAACAATATTGGAGGAATATTTATGT

ATCTTATTTTCATGTAACTCTTCCTGCTAAAATCGCAGGGTTTTCCCTGCATACAAGCAAATGAAAGCATGC

GATTATAGACAGGAGGAAATGTTATGGAATTAATATT

AAAAGCAAAAGACATTCGTGTGGAATTCAAAGGACGCGATGTTTTAGATATAAATGAATTAGAAGTATATGA

TTATGACCGTATTGGTTTAGTAGGAGCAAATGGTGCTGGAAAAAGCACTTTACTCAGGGTACTTTTAGGAGA

ATTAACTCCCCCAGGATGTAAAATGAATCGTCTGGGTGAACTTGCCTATATTCCCCAGTTGGACGAAGTAAC

TCTGCAGGAGGAAAAAGATTTTGCACTTGTAGGCAAGCTAGGTGTTGAGCAATTAAATATACAGACTATGAG

CGGTGGTGAAGAAACAAGGCTTAAAATAGCACAGGCCTTATCGGCACAGGTTCATGGTATTTTAGCGGATGA

ACCTACGAGCCATTTAGACCGTGAAGGAATTGATTTTCTAATAGGACAGCTAAAATATTTTACAGGTGCACT

GTTAGTTATTAGCCATGACCGCTATTTTCTTGATGAAATAGTAGATAAAATATGGGAACTGAAAGATGGCAA

AATCACTGAGTATTGGGGAAACTATTCTGATTATCTTCGTCAGAAAGAGGAAGAACGTAAGAGCCAAGCTGC

AGAATACGAACAATTTATTGCGGAACGTGCCCGATTGGAAAGGGCTGCGGAGGAAAAGCGAAAACAGGCTCG

TAAAATAGAACAGAAGGCAAAAGGTTCTTCAAAGAAAAAAAGTACTGAAGACGGAGGGCGTTTAGCTCATCA

AAAATCAATAGGAAGTAAGGAAAAAAAGATGTATAATGCTGCTAAAACCCTAGAGCACAGGATTGCGGCCTT

AGGAAAAGTAGAAGCTCCGGAAGGCATTCGCAGAATTCGTTTCAGGCAAAGTAAAGCATTGGAGCTCCATAA

TCCATACCCTATAGTCGGTGCAGAAATTAATAAAGTATTTGGGGATAAGGCTCTGTTTGAAAATGCATCTTT

TCAAATTCCGTTAGGAGCAAAAGTGGCGTTAACTGGTGGTAATGGAATCGGAAAAACAACTTTAATCCAAAT

GATCTTAAACCATGAAGAAGGAATTTCTATTTCGCCTAAGGCAAAAATAGGTTACTTTGCACAGAATGGTTA

CAAGTACAACAGTAATCAGAATGTTATGGAGTTTATGCAGAAGGATTGTGACTACAATATATCAGAAATTCG

TTCAGTGCTAGCATCTATGGGGTTCAAACAGAACGATATTGGAAAAAGTTTATCTGTTTTAAGCGGTGGAGA

AATTATAAAATTGTTGCTTGCTAAAATGCTCATGGGTAGATATAACATCCTAATAATGGATGAACCCAGTAA

CTTCCTTGACATACCAAGTTTAGAGGCTTTGGAAATACTAATGAAGGAGTACACCGGAACTATCGTGTTTAT

CACCCACGATAAACGATTACTCGAAAATGTAGCAGATGTAGTTTATGAAATTAGAGATAAGAAAATAAATCT

GAAACATTAAATTTAAGGTAGTCGCTGGTCAGTATAGTCTGTTCTGGTTGGCGACTCCATTGTTAAAGAGTA

TAAAGACTTTAGATTTTATGAATATTAAAAATAGGAACAGTCAATTGAACTGCTCCTATTTTTCTGCTAAAT

ATATTGTAGTTTTCTTATATGTATAATGATAGATTAGCGGATTCTCATCTACGGTACTTACTTCAAATATGA

AGAAGTGATCGCGGTTATCTCTGGACTTTTCCTTATTGAGGACAAAGTAATTCTTACGTGAAGTCGCCATTG

TTTTTAGGATATCATCAGTTAGGAAGGTCAATGGAATATTCATGTTAGAGTAGCGGTAGAAGTCACGTTCAA

AATCTTGGTAGCTCTCGCTATAATAGTCCATTTGTAGGTGATTACGCTGAAACTCAAGCTGATTCATAGAGC

ACCTCCTCGACAAGTTCAATACTAATAATGTCTTTTAATTTCAAATTGATGTGACCTGTTGTAGTTTTTATC

AAAATGAAATCTTTGGTCAGACTTGGTATTGTTCCAGTGTAGGAAACACGCTTGTTTTTTTCAATCACTTGA

ATGCGTGTGCGTAGCTGCCCGGCGTATACTTGACTGAGGAGTAATAATTTCTTCTCTAGTGATAAGTCAGAC

ATGTACGTTACTTTGTTTGTATCATCAGAGAGTGCTGATGCATGTTCAGATAGGAAAAAGCCCATCCATTTT

TGCATCTTTGTATCCTGGTACTCTCTTGCTGATTGAAATGGTAAATATGAACGGTCAATCATATCAAATCCT

TTCTATGCAGAGGCAAGGGTATTTTTATCAAATTGAATCGTAAAACCTTGAATTCCCCCACCTGTGTAACAT

TCTTTAAAGCGATTGATTACCTCAGTATAGATTATCACAGATGAGCTTGTTGGCTTAATGCTAAATGTAAAT

TCCAATGGTAATCGGTTTTCAGATTTAGCATGTACTAGTCGTATCGATATTTCAGTTGTTTTGAGTTTTCTC

TGACGAAGTTTTGAAGTTGCTGTTTCAACGATTCCATGTAAAAATCTTTCAAGCATTTCAATATCATTACAT

CCTTTGCTACGGATTTCTGAAAATTGTACTGTATTTTTTTCTTGTTTCATTTTAATCCCTCCAATCCACCCG

CGGAATGACCACCGATAAGTTTACTGCGTTCAATATTTCTGGAACCTTCAGTTAGGACGGTTCCTTTTTGTA

TGGCTAAAAAACCAAACTGTTCTCTGACAACATCAATAGCTGTCTGAAGTCTATTATCTTTTTCAATTTGTT

CTACATCATCAAAGAGTGATAGTAGAGTATAGCTTTCATCTACGAAGCCACTATAAGATACACCAATTTGTC

TCACTGCACCAGAGGTGTATTTTTTCGGAATAATACAAGTACATGACTCACCATTGTTTTGGGGAGATTTG

CGGGTTCAATTTTATTCTGAGCATTTATAGATTTTTTCATCTCAGTCCTAGAATAGCCAATATGAATAGAAA

CGACAGTAGTCAATACTAGGGCTACTGTTCCGTTCCACAGTATCATTTAAAAATCATT-TTCACACCCTTTC

GTCTATTAGTATAGAAGAAAGCTCTCAGCACA

>GA05248/Mega-2.II

CATGTTGAGGCGGTAAGTTTGCTAGTCAAGGAGTAAAACGACGAAGATTAGCATTTACTTCCGCCCATGCGA

TAGCTGTCCGTGATTGACAAGTGCTAGCACGCAGACAGAACGGAGATAGCGAACCGCTGAGTGTGTCGCTCT

GCTCGTAAAAGCTTAGAAACCTTTGAACGAAAGGGATAATGAAAGCCTTGATTGCAAGGCTTTTTGCTTTAT

GGTGGGTAAGTATCAGAGTGAGAAAATTTTTGGAATGAGTAGAAGTGATAGCTAGAAATTATCAGTTTCTA

TTTCCATTTACCCTGTGGGTACGTGTTTGTTTCCATTGACAAGGAGTTTGTGGGAATAGAAATGT

ACCCACCTTGTTTGAATCAAGTGAAGTGTAGTTGAAGGAAATCTGTTGAAAGCAATACTTCATTTTACCG

AATAAGTAATAATTTAGGCAACTTCAAATCGATTAAAAAAAACTATTTTAAAGGTTAAGAGTAGACAAAA

ATTGTCCACTCTTTTTTGCAAACTCAATTTATCAATAAATGAAATGAGGGAATGTAAAATG

AAATATTTTGAGGTTGAGTTAGAAAATCCTGATGAATTTTTAAAACTACAAACAGAAGATTTTGTGAAAG

CTAATCGCTTGCTACTAAGGAAGATAATCCAGAGCGTTACAGTCTATGAAGAAAACTTCGTCATATCCTT

TAAATCTGGCATCGAATTGGAAGTATGAGTCTCATTCCATAACTTTTATATTGAACATATCATCTTGTTGTG

TTATACTATAAATTGATATAAACAAAGATGTAGGAGGAACCGAAACTATGACAGCCTCAATGCGTTTAAGAT

AAGCTGGCAATAAAAAAAGCAGAATCTATACCCGATGATAGGCTTTTTTGTTGTGCTTATTTATACGATATT

GAGCATTCATTAGTTACGGTGAGGATATTGGTTATTTAACTATACCTTTATTTAACTATGTCTTTAATATGA

ATGTTTCCAAATTGTATGTATGCAGACCAAAAGCCACATTGTGGGGTTTGGCCTGCATTTTTTTTGCCTA

GAATGCTATTCAAAATAGAAATTCAAGCAAAATAATATGCAGGAGATAATATAAATGGAAAAATACAACAAT

TGGAAACGAAAATTTTATGCAATATGGGCAGGGCAAGCAGTATCATTAATCACTAGTGCCATCCTGCAAATG

GCGATTATTTTTTACCTTACAGAAAAAACAGGATCTGCGATGGTCTTGTCTATGGCTTCATTAGTAGGTTTT

TTACCCTATGCGATTTTGGGACCTGCCATTGGTGTGCTAGTGGATCGTCATGATAGGAAGAAGATAATGATT

GGTGCCGATTTAATTATCGCAGCAGCTGGTGCAGTGCTTGCTATTGTTGCATTCTGTATGGAGCTACCTGTC

TGGATGATTATGATAGTATTGTTTATCCGTAGCATTGGAACAGCTTTTCATACCCCAGCACTCAATGCGGTT

ACACCACTTTTAGTACCAGAAGAACAGCTAACGAAATGCGCAGGCTATAGTCAGTCTTTGCAGTCTATAAGC

TATATTGTTAGTCCGGCAGTTGCAGCACTCTTATACTCCGTTTGGGATTTAAATGCTATTATTGCCATCGAC

GTATTGGGTGCTGTGATTGCATCTATTACGGTAGCAATTGTACGTATACCTAAGCTGGGTAATCAAGTGCAA

AGTTTAGAACCAAATTTCATAAGGGAGATGAAAGAAGGAGTTGTGGTTCTGAGACAAAACAAAGGATTGTTT

GCCTTATTACTCTTAGGAACACTATATACTTTTGTTTATATGCCAATCAATGCACTATTTCCTTTAATAAGC

ATGGAACACTTTAATGGAACGCCTGTGCATATTTCTATTACGGAAATTTCCTTTGCATTTGGGATGCTAGCA

GGAGGCTTATTATTAGGAAGATTAGGGGGCTTCGAAAAGCATGTATTACTAATAACAAGTTCATTTTTTATA

ATGGGGACCAGTTTAGCCGTTTCGGGAATACTTCCTCCAAATGGATTTGTAATATTCGTAGTTTGCTGTGCA

ATAATGGGGCTTTCGGTGCCATTTTATAGCGGTGTGCAAACAGCTCTTTTTCAGGAGAAAATTAAGCCTGAA

TATTTAGGACGTGTATTTTCTTTGATCGGAAGTATCATGTCACTTGCTATGCCAATTGGGTTAATTCTTTCT

GGATTCTTTGCTGATAAAATCGGTGTAAATCATTGGTTTTTACTATCAGGTATTTTAATTATTGGCATTGCT

ATAGTTTGCCAAATGATAACTGAGGTTAGAAAATTAGATTTAAAATAAACAATATTGGAGGAA

ATGTTATGGAATTAATATT

AAAAGCAAAAGACATTCGTGTGGAATTCAAAGGACGCGATGTTTTAGATATAAATGAATTAGAAGTATATGA

TTATGACCGTATTGGTTTAGTAGGAGCAAATGGTGCTGGAAAAAGCACTTTACTCAGGGTACTTTTAGGAGA

ATTAACTCCCCCAGGATGTAAAATGAATCGTCTGGGTGAACTTGCCTATATTCCCCAGTTGGACGAAGTAAC

TCTGCAGGAGGAAAAAGATTTTGCACTTGTAGGCAAGCTAGGTGTTGAGCAATTAAATATACAGACTATGAG

CGGTGGTGAAGAAACAAGGCTTAAAATAGCACAGGCCTTATCGGCACAGGTTCATGGTATTTTAGCGGATGA

ACCTACGAGCCATTTAGACCGTGAAGGAATTGATTTTCTAATAGGACAGCTAAAATATTTTACAGGTGCACT

GTTAGTTATTAGCCATGACCGCTATTTTCTTGATGAAATAGTAGATAAAATATGGGAACTGAAAGATGGCAA

AATCACTGAGTATTGGGGAAACTATTCTGATTATCTTCGTCAGAAAGAGGAAGAACGTAAGAGCCAAGCTGC

AGAATACGAACAATTTATTGCGGAACGTGCCCGATTGGAAAGGGCTGCGGAGGAAAAGCGAAAACAGGCTCG

TAAAATAGAACAGAAGGCAAAAGGTTCTTCAAAGAAAAAAAGTACTGAAGACGGAGGGCGTTTAGCTCATCA

AAAATCAATAGGAAGTAAGGAAAAAAAGATGTATAATGCTGCTAAAACCCTAGAGCACAGGATTGCGGCCTT

AGGAAAAGTAGAAGCTCCGGAAGGCATTCGCAGAATTCGTTTCAGGCAAAGTAAAGCATTGGAGCTCCATAA

TCCATACCCTATAGTCGGTGCAGAAATTAATAAAGTATTTGGGGATAAGGCTCTGTTTGAAAATGCATCTTT

TCAAATTCCGTTAGGAGCAAAAGTGGCGTTAACTGGTGGTAATGGAATCGGAAAAACAACTTTAATCCAAAT

GATCTTAAACCATGAAGAAGGAATTTCTATTTCGCCTAAGGCAAAAATAGGTTACTTTGCACAGAATGGTTA

CAAGTACAACAGTAATCAGAATGTTATGGAGTTTATGCAGAAGGATTGTGACTACAATATATCAGAAATTCG

TTCAGTGCTAGCATCTATGGGGTTCAAACAGAACGATATTGGAAAAAGTTTATCTGTTTTAAGCGGTGGAGA

AATTATAAAATTGTTGCTTGCTAAAATGCTCATGGGTAGATATAACATCCTAATAATGGATGAACCCAGTAA

CTTCCTTGACATACCAAGTTTAGAGGCTTTGGAAATACTAATGAAGGAGTACACCGGAACTATCGTGTTTAT

CACCCACGATAAACGATTACTCGAAAATGTAGCAGATGTAGTTTATGAAATTAGAGATAAGAAAATAAATCT

GAAACATTAAATTTAAGGTAGTCGCTGGTCAGTATAGTCTGTTCTGGTTGGCGACTCCATTGTTAAAGAGTA

TAAAGACTTTAGATTTTATGAATATTAAAAATAGGAACAGTCAATTGAACTGCTCCTATTTTTCTGCTAAAT

ATATTGTAGTTTTCTTATATGTATAATGATAGATTAGCGGATTCTCATCTACGGTACTTACTTCAAATATGA

AGAAGTGATCGCGGTTATCTCTGGACTTTTCCTTATTGAGGACAAAGTAATTCTTACGTGAAGTCGCCATTG

TTTTTAGGATATCATCAGTTAGGAAGGTCAATGGAATATTCATGTTAGAGTAGCGGTAGAAGTCACGTTCAA

AATCTTGGTAGCTCTCGCTATAATAGTCCATTTGTAGGTGATTACGCTGAAACTCAAGCTGATTCATAGAGC

ACCTCCTCGACAAGTTCAATACTAATAATGTCTTTTAATTTCAAATTGATGTGACCTGTTGTAGTTTTTATC

AAAATGAAATCTTTGGTCAGACTTGGTATTGTTCCAGTGTAGGAAACACGCTTGTTTTTTTCAATCACTTGA

ATGCGTGTGCGTAGCTGCCCGGCGTATACTTGACTGAGGAGTAATAATTTCTTCTCTAGTGATAAGTCAGAC

ATGTACGTTACTTTGTTTGTATCATCAGAGAGTGCTGATGCATGTTCAGATAGGAAAAAGCCCATCCATTTT

TGCATCTTTGTATCCTGGTACTCTCTTGCTGATTGAAATGGTAAATATGAACGGTCAATCATATCAAATCCT

TTCTATGCAGAGGCAAGGGTATTTTTATCAAATTGAATCGTAAAACCTTGAATTCCCCCACCTGTGTAACAT

TCTTTAAAGCGATTGATTACCTCAGTATAGATTATCACAGATGAGCTTGTTGGCTTAATGCTAAATGTAAAT

TCCAATGGTAATCGGTTTTCAGATTTAGCATGTACTAGTCGTATCGATATTTCAGTTGTTTTGAGTTTTCTC

TGACGAAGTTTTGAAGTTGCTGTTTCAACGATTCCATGTAAAAATCTTTCAAGCATTTCAATATCATTACAT

CCTTTGCTACGGATTTCTGAAAATTGTACTGTATTTTTTTCTTGTTTCATTTTAATCCCTCCAATCCACCCG

CGGAATGACCACCGATAAGTTTACTGCGTTCAATATTTCTGGAACCTTCAGTTAGGACGGTTCCTTTTTGTA

TGGCTAAAAAACCAAACTGTTCTCTGACAACATCAATAGCTGTCTGAAGTCTATTATCTTTTTCAATTTGTT

CTACATCATCAAAGAGTGATAGTAGAGTATAGCTTTCATCTACGAAGCCACTATAAGATACACCAATTTGTC

TCACTGCACCAGAGGTGTATTTTTTCGGAATAATACAAGTACATGACTCACCATTGTTTTGGGGAGATTTG

CGGGTTCAATTTTATTCTGAGCATTTATAGATTTTTTCATCTCAGTCCTAGAATAGCCAATATGAATAGAAA

CGACAGTAGTCAATACTAGGGCTACTGTTCCGTTCCACAGTATCATTTAAAAATCATT-TTCACACCCTTTC

GTCTATTAGTATAGAAGAAAGCTCTCAGCACA

>GA49447/Mega-2.II

CATGTTGAGGCGGTAAGTTTGCTAGTCAAGGAGTAAAACGACGAAGATTAGCATTTACTTCCGCCCATGCGA

TAGCTGTCCGTGATTGACAAGTGCTAGCACGCAGACAGAACGGAGATAGCGAACCGCTGAGTGTGTCGCTCT

GCTCGTAAAAGCTTAGAAACCTTTGAACGAAAGGGATAATGAAAGCCTTGATTGCAAGGCTTTTTGCTTTAT

GGTGGGTAAGTATCAGAGTGAGAAAATTTTTGGAATGAGTAGAAGTGATAGCTAGAAATTATCAGTTTCTA

TTTCCATTTACCCTGTGGGTACGTGTTTGTTTCCATTGACAAGGAGTTTGTGGGAATAGAAATGT

ACCCACCTTGTTTGAATCAAGTGAAGTGTAGTTGAAGGAAATCTGTTGAAAGCAATACTTCATTTTACCG

AATAAGTAATAATTTAGGCAACTTCAAATCGATTAAAAAAAACTATTTTAAAGGTTAAGAGTAGACAAAA

ATTGTCCACTCTTTTTTGCAAACTCAATTTATCAATAAATGAAATGAGGGAATGTAAAATG

AAATATTTTGAGGTTGAGTTAGAAAATCCTGATGAATTTTTAAAACTACAAACAGAAGATTTTGTGAAAG

CTAATCGCTTGCTACTAAGGAAGATAATCCAGAGCGTTACAGTCTATGAAGAAAACTTCGTCATATCCTT

TAAATCTGGCATCGAATTGGAAGTATGAGTCTCATTCCATAACTTTTATATTGAACATATCATCTTGTTGTG

TTATACTATAAATTGATATAAACAAAGATGTAGGAGGAACCGAAACTATGACAGCCTCAATGCGTTTAAGAT

AAGCTGGCAATAAAAAAAGCAGAATCTATACCCGATGATAGGCTTTTTTGTTGTGCTTATTTATACGATATT

GAGCATTCATTAGTTACGGTGAGGATATTGGTTATTTAACTATACCTTTATTTAACTATGTCTTTAATATGA

ATGTTTCCAAATTGTATGTATGCAGACCAAAAGCCACATTGTGGGGTTTGGCCTGCATTTTTTTTGCCTA

GAATGCTATTCAAAATAGAAATTCAAGCAAAATAATATGCAGGAGATAATATAAATGGAAAAATACAACAAT

TGGAAACGAAAATTTTATGCAATATGGGCAGGGCAAGCAGTATCATTAATCACTAGTGCCATCCTGCAAATG

GCGATTATTTTTTACCTTACAGAAAAAACAGGATCTGCGATGGTCTTGTCTATGGCTTCATTAGTAGGTTTT

TTACCCTATGCGATTTTGGGACCTGCCATTGGTGTGCTAGTGGATCGTCATGATAGGAAGAAGATAATGATT

GGTGCCGATTTAATTATCGCAGCAGCTGGTGCAGTGCTTGCTATTGTTGCATTCTGTATGGAGCTACCTGTC

TGGATGATTATGATAGTATTGTTTATCCGTAGCATTGGAACAGCTTTTCATACCCCAGCACTCAATGCGGTT

ACACCACTTTTAGTACCAGAAGAACAGCTAACGAAATGCGCAGGCTATAGTCAGTCTTTGCAGTCTATAAGC

TATATTGTTAGTCCGGCAGTTGCAGCACTCTTATACTCCGTTTGGGATTTAAATGCTATTATTGCCATCGAC

GTATTGGGTGCTGTGATTGCATCTATTACGGTAGCAATTGTACGTATACCTAAGCTGGGTAATCAAGTGCAA

AGTTTAGAACCAAATTTCATAAGGGAGATGAAAGAAGGAGTTGTGGTTCTGAGACAAAACAAAGGATTGTTT

GCCTTATTACTCTTAGGAACACTATATACTTTTGTTTATATGCCAATCAATGCACTATTTCCTTTAATAAGC

ATGGAACACTTTAATGGAACGCCTGTGCATATTTCTATTACGGAAATTTCCTTTGCATTTGGGATGCTAGCA

GGAGGCTTATTATTAGGAAGATTAGGGGGCTTCGAAAAGCATGTATTACTAATAACAAGTTCATTTTTTATA

ATGGGGACCAGTTTAGCCGTTTCGGGAATACTTCCTCCAAATGGATTTGTAATATTCGTAGTTTGCTGTGCA

ATAATGGGGCTTTCGGTGCCATTTTATAGCGGTGTGCAAACAGCTCTTTTTCAGGAGAAAATTAAGCCTGAA

TATTTAGGACGTGTATTTTCTTTGATCGGAAGTATCATGTCACTTGCTATGCCAATTGGGTTAATTCTTTCT

GGATTCTTTGCTGATAAAATCGGTGTAAATCATTGGTTTTTACTATCAGGTATTTTAATTATTGGCATTGCT

ATAGTTTGCCAAATGATAACTGAGGTTAGAAAATTAGATTTAAAATAAACAATATTGGAGGAA

ATGTTATGGAATTAATATT

AAAAGCAAAAGACATTCGTGTGGAATTCAAAGGACGCGATGTTTTAGATATAAATGAATTAGAAGTATATGA

TTATGACCGTATTGGTTTAGTAGGAGCAAATGGTGCTGGAAAAAGCACTTTACTCAGGGTACTTTTAGGAGA

ATTAACTCCCCCAGGATGTAAAATGAATCGTCTGGGTGAACTTGCCTATATTCCCCAGTTGGACGAAGTAAC

TCTGCAGGAGGAAAAAGATTTTGCACTTGTAGGCAAGCTAGGTGTTGAGCAATTAAATATACAGACTATGAG

CGGTGGTGAAGAAACAAGGCTTAAAATAGCACAGGCCTTATCGGCACAGGTTCATGGTATTTTAGCGGATGA

ACCTACGAGCCATTTAGACCGTGAAGGAATTGATTTTCTAATAGGACAGCTAAAATATTTTACAGGTGCACT

GTTAGTTATTAGCCATGACCGCTATTTTCTTGATGAAATAGTAGATAAAATATGGGAACTGAAAGATGGCAA

AATCACTGAGTATTGGGGAAACTATTCTGATTATCTTCGTCAGAAAGAGGAAGAACGTAAGAGCCAAGCTGC

AGAATACGAACAATTTATTGCGGAACGTGCCCGATTGGAAAGGGCTGCGGAGGAAAAGCGAAAACAGGCTCG

TAAAATAGAACAGAAGGCAAAAGGTTCTTCAAAGAAAAAAAGTACTGAAGACGGAGGGCGTTTAGCTCATCA

AAAATCAATAGGAAGTAAGGAAAAAAAGATGTATAATGCTGCTAAAACCCTAGAGCACAGGATTGCGGCCTT

AGGAAAAGTAGAAGCTCCGGAAGGCATTCGCAGAATTCGTTTCAGGCAAAGTAAAGCATTGGAGCTCCATAA

TCCATACCCTATAGTCGGTGCAGAAATTAATAAAGTATTTGGGGATAAGGCTCTGTTTGAAAATGCATCTTT

TCAAATTCCGTTAGGAGCAAAAGTGGCGTTAACTGGTGGTAATGGAATCGGAAAAACAACTTTAATCCAAAT

GATCTTAAACCATGAAGAAGGAATTTCTATTTCGCCTAAGGCAAAAATAGGTTACTTTGCACAGAATGGTTA

CAAGTACAACAGTAATCAGAATGTTATGGAGTTTATGCAGAAGGATTGTGACTACAATATATCAGAAATTCG

TTCAGTGCTAGCATCTATGGGGTTCAAACAGAACGATATTGGAAAAAGTTTATCTGTTTTAAGCGGTGGAGA

AATTATAAAATTGTTGCTTGCTAAAATGCTCATGGGTAGATATAACATCCTAATAATGGATGAACCCAGTAA

CTTCCTTGACATACCAAGTTTAGAGGCTTTGGAAATACTAATGAAGGAGTACACCGGAACTATCGTGTTTAT

CACCCACGATAAACGATTACTCGAAAATGTAGCAGATGTAGTTTATGAAATTAGAGATAAGAAAATAAATCT

GAAACATTAAATTTAAGGTAGTCGCTGGTCAGTATAGTCTGTTCTGGTTGGCGACTCCATTGTTAAAGAGTA

TAAAGACTTTAGATTTTATGAATATTAAAAATAGGAACAGTCAATTGAACTGCTCCTATTTTTCTGCTAAAT

ATATTGTAGTTTTCTTATATGTATAATGATAGATTAGCGGATTCTCATCTACGGTACTTACTTCAAATATGA

AGAAGTGATCGCGGTTATCTCTGGACTTTTCCTTATTGAGGACAAAGTAATTCTTACGTGAAGTCGCCATTG

TTTTTAGGATATCATCAGTTAGGAAGGTCAATGGAATATTCATGTTAGAGTAGCGGTAGAAGTCACGTTCAA

AATCTTGGTAGCTCTCGCTATAATAGTCCATTTGTAGGTGATTACGCTGAAACTCAAGCTGATTCATAGAGC

ACCTCCTCGACAAGTTCAATACTAATAATGTCTTTTAATTTCAAATTGATGTGACCTGTTGTAGTTTTTATC

AAAATGAAATCTTTGGTCAGACTTGGTATTGTTCCAGTGTAGGAAACACGCTTGTTTTTTTCAATCACTTGA

ATGCGTGTGCGTAGCTGCCCGGCGTATACTTGACTGAGGAGTAATAATTTCTTCTCTAGTGATAAGTCAGAC

ATGTACGTTACTTTGTTTGTATCATCAGAGAGTGCTGATGCATGTTCAGATAGGAAAAAGCCCATCCATTTT

TGCATCTTTGTATCCTGGTACTCTCTTGCTGATTGAAATGGTAAATATGAACGGTCAATCATATCAAATCCT

TTCTATGCAGAGGCAAGGGTATTTTTATCAAATTGAATCGTAAAACCTTGAATTCCCCCACCTGTGTAACAT

TCTTTAAAGCGATTGATTACCTCAGTATAGATTATCACAGATGAGCTTGTTGGCTTAATGCTAAATGTAAAT

TCCAATGGTAATCGGTTTTCAGATTTAGCATGTACTAGTCGTATCGATATTTCAGTTGTTTTGAGTTTTCTC

TGACGAAGTTTTGAAGTTGCTGTTTCAACGATTCCATGTAAAAATCTTTCAAGCATTTCAATATCATTACAT

CCTTTGCTACGGATTTCTGAAAATTGTACTGTATTTTTTTCTTGTTTCATTTTAATCCCTCCAATCCACCCG

CGGAATGACCACCGATAAGTTTACTGCGTTCAATATTTCTGGAACCTTCAGTTAGGACGGTTCCTTTTTGTA

TGGCTAAAAAACCAAACTGTTCTCTGACAACATCAATAGCTGTCTGAAGTCTATTATCTTTTTCAATTTGTT

CTACATCATCAAAGAGTGATAGTAGAGTATAGCTTTCATCTACGAAGCCACTATAAGATACACCAATTTGTC

TCACTGCACCAGAGGTGTATTTTTTCGGAATAATACAAGTACATGACTCACCATTGTTTTGGGGAGATTTG

CGGGTTCAATTTTATTCTGAGCATTTATAGATTTTTTCATCTCAGTCCTAGAATAGCCAATATGAATAGAAA

CGACAGTAGTCAATACTAGGGCTACTGTTCCGTTCCACAGTATCATTTAAAAATCATT-TTCACACCCTTTC

GTCTATTAGTATAGAAGAAAGCTCTCAGCACA

>GA43264/Mega-2.II

CATGTTGAGGCGGTAAGTTTGCTAGTCAAGGAGTAAAACGACGAAGATTAGCATTTACTTCCGCCCATGCGA

TAGCTGTCCGTGATTGACAAGTGCTAGCACGCAGACAGAACGGAGATAGCGAACCGCTGAGTGTGTCGCTCT

GCTCGTAAAAGCTTAGAAACCTTTGAACGAAAGGGATAATGAAAGCCTTGATTGCAAGGCTTTTTGCTTTAT

GGTGGGTAAGTATCAGAGTGAGAAAATTTTTGGAATGAGTAGAAGTGATAGCTAGAAATTATCAGTTTCTA

TTTCCATTTACCCTGTGGGTACGTGTTTGTTTCCATTGACAAGGAGTTTGTGGGAATAGAAATGT

ACCCACCTTGTTTGAATCAAGTGAAGTGTAGTTGAAGGAAATCTGTTGAAAGCAATACTTCATTTTACCG

AATAAGTAATAATTTAGGCAACTTCAAATCGATTAAAAAAAACTATTTTAAAGGTTAAGAGTAGACAAAA

ATTGTCCACTCTTTTTTGCAAACTCAATTTATCAATAAATGAAATGAGGGAATGTAAAATG

AAATATTTTGAGGTTGAGTTAGAAAATCCTGATGAATTTTTAAAACTACAAACAGAAGATTTTGTGAAAG

CTAATCGCTTGCTACTAAGGAAGATAATCCAGAGCGTTACAGTCTATGAAGAAAACTTCGTCATATCCTT

TAAATCTGGCATCGAATTGGAAGTATGAGTCTCATTCCATAACTTTTATATTGAACATATCATCTTGTTGTG

TTATACTATAAATTGATATAAACAAAGATGTAGGAGGAACCGAAACTATGACAGCCTCAATGCGTTTAAGAT

AAGCTGGCAATAAAAAAAGCAGAATCTATACCCGATGATAGGCTTTTTTGTTGTGCTTATTTATACGATATT

GAGCATTCATTAGTTACGGTGAGGATATTGGTTATTTAACTATACCTTTATTTAACTATGTCTTTAATATGA

ATGTTTCCAAATTGTATGTATGCAGACCAAAAGCCACATTGTGGGGTTTGGCCTGCATTTTTTTTGCCTA

GAATGCTATTCAAAATAGAAATTCAAGCAAAATAATATGCAGGAGATAATATAAATGGAAAAATACAACAAT

TGGAAACGAAAATTTTATGCAATATGGGCAGGGCAAGCAGTATCATTAATCACTAGTGCCATCCTGCAAATG

GCGATTATTTTTTACCTTACAGAAAAAACAGGATCTGCGATGGTCTTGTCTATGGCTTCATTAGTAGGTTTT

TTACCCTATGCGATTTTGGGACCTGCCATTGGTGTGCTAGTGGATCGTCATGATAGGAAGAAGATAATGATT

GGTGCCGATTTAATTATCGCAGCAGCTGGTGCAGTGCTTGCTATTGTTGCATTCTGTATGGAGCTACCTGTC

TGGATGATTATGATAGTATTGTTTATCCGTAGCATTGGAACAGCTTTTCATACCCCAGCACTCAATGCGGTT

ACACCACTTTTAGTACCAGAAGAACAGCTAACGAAATGCGCAGGCTATAGTCAGTCTTTGCAGTCTATAAGC

TATATTGTTAGTCCGGCAGTTGCAGCACTCTTATACTCCGTTTGGGATTTAAATGCTATTATTGCCATCGAC

GTATTGGGTGCTGTGATTGCATCTATTACGGTAGCAATTGTACGTATACCTAAGCTGGGTAATCAAGTGCAA

AGTTTAGAACCAAATTTCATAAGGGAGATGAAAGAAGGAGTTGTGGTTCTGAGACAAAACAAAGGATTGTTT

GCCTTATTACTCTTAGGAACACTATATACTTTTGTTTATATGCCAATCAATGCACTATTTCCTTTAATAAGC

ATGGAACACTTTAATGGAACGCCTGTGCATATTTCTATTACGGAAATTTCCTTTGCATTTGGGATGCTAGCA

GGAGGCTTATTATTAGGAAGATTAGGGGGCTTCGAAAAGCATGTATTACTAATAACAAGTTCATTTTTTATA

ATGGGGACCAGTTTAGCCGTTTCGGGAATACTTCCTCCAAATGGATTTGTAATATTCGTAGTTTGCTGTGCA

ATAATGGGGCTTTCGGTGCCATTTTATAGCGGTGTGCAAACAGCTCTTTTTCAGGAGAAAATTAAGCCTGAA

TATTTAGGACGTGTATTTTCTTTGATCGGAAGTATCATGTCACTTGCTATGCCAATTGGGTTAATTCTTTCT

GGATTCTTTGCTGATAAAATCGGTGTAAATCATTGGTTTTTACTATCAGGTATTTTAATTATTGGCATTGCT

ATAGTTTGCCAAATGATAACTGAGGTTAGAAAATTAGATTTAAAATAAACAATATTGGAGGAAATGTTATGG

AATTAATATT

AAAAGCAAAAGACATTCGTGTGGAATTCAAAGGACGCGATGTTTTAGATATAAATGAATTAGAAGTATATGA

TTATGACCGTATTGGTTTAGTAGGAGCAAATGGTGCTGGAAAAAGCACTTTACTCAGGGTACTTTTAGGAGA

ATTAACTCCCCCAGGATGTAAAATGAATCGTCTGGGTGAACTTGCCTATATTCCCCAGTTGGACGAAGTAAC

TCTGCAGGAGGAAAAAGATTTTGCACTTGTAGGCAAGCTAGGTGTTGAGCAATTAAATATACAGACTATGAG

CGGTGGTGAAGAAACAAGGCTTAAAATAGCACAGGCCTTATCGGCACAGGTTCATGGTATTTTAGCGGATGA

ACCTACGAGCCATTTAGACCGTGAAGGAATTGATTTTCTAATAGGACAGCTAAAATATTTTACAGGTGCACT

GTTAGTTATTAGCCATGACCGCTATTTTCTTGATGAAATAGTAGATAAAATATGGGAACTGAAAGATGGCAA

AATCACTGAGTATTGGGGAAACTATTCTGATTATCTTCGTCAGAAAGAGGAAGAACGTAAGAGCCAAGCTGC

AGAATACGAACAATTTATTGCGGAACGTGCCCGATTGGAAAGGGCTGCGGAGGAAAAGCGAAAACAGGCTCG

TAAAATAGAACAGAAGGCAAAAGGTTCTTCAAAGAAAAAAAGTACTGAAGACGGAGGGCGTTTAGCTCATCA

AAAATCAATAGGAAGTAAGGAAAAAAAGATGTATAATGCTGCTAAAACCCTAGAGCACAGGATTGCGGCCTT

AGGAAAAGTAGAAGCTCCGGAAGGCATTCGCAGAATTCGTTTCAGGCAAAGTAAAGCATTGGAGCTCCATAA

TCCATACCCTATAGTCGGTGCAGAAATTAATAAAGTATTTGGGGATAAGGCTCTGTTTGAAAATGCATCTTT

TCAAATTCCGTTAGGAGCAAAAGTGGCGTTAACTGGTGGTAATGGAATCGGAAAAACAACTTTAATCCAAAT

GATCTTAAACCATGAAGAAGGAATTTCTATTTCGCCTAAGGCAAAAATAGGTTACTTTGCACAGAATGGTTA

CAAGTACAACAGTAATCAGAATGTTATGGAGTTTATGCAGAAGGATTGTGACTACAATATATCAGAAATTCG

TTCAGTGCTAGCATCTATGGGGTTCAAACAGAACGATATTGGAAAAAGTTTATCTGTTTTAAGCGGTGGAGA

AATTATAAAATTGTTGCTTGCTAAAATGCTCATGGGTAGATATAACATCCTAATAATGGATGAACCCAGTAA

CTTCCTTGACATACCAAGTTTAGAGGCTTTGGAAATACTAATGAAGGAGTACACCGGAACTATCGTGTTTAT

CACCCACGATAAACGATTACTCGAAAATGTAGCAGATGTAGTTTATGAAATTAGAGATAAGAAAATAAATCT

GAAACATTAAATTTAAGGTAGTCGCTGGTCAGTATAGTCTGTTCTGGTTGGCGACTCCATTGTTAAAGAGTA

TAAAGACTTTAGATTTTATGAATATTAAAAATAGGAACAGTCAATTGAACTGCTCCTATTTTTCTGCTAAAT

ATATTGTAGTTTTCTTATATGTATAATGATAGATTAGCGGATTCTCATCTACGGTACTTACTTCAAATATGA

AGAAGTGATCGCGGTTATCTCTGGACTTTTCCTTATTGAGGACAAAGTAATTCTTACGTGAAGTCGCCATTG

TTTTTAGGATATCATCAGTTAGGAAGGTCAATGGAATATTCATGTTAGAGTAGCGGTAGAAGTCACGTTCAA

AATCTTGGTAGCTCTCGCTATAATAGTCCATTTGTAGGTGATTACGCTGAAACTCAAGCTGATTCATAGAGC

ACCTCCTCGACAAGTTCAATACTAATAATGTCTTTTAATTTCAAATTGATGTGACCTGTTGTAGTTTTTATC

AAAATGAAATCTTTGGTCAGACTTGGTATTGTTCCAGTGTAGGAAACACGCTTGTTTTTTTCAATCACTTGA

ATGCGTGTGCGTAGCTGCCCGGCGTATACTTGACTGAGGAGTAATAATTTCTTCTCTAGTGATAAGTCAGAC

ATGTACGTTACTTTGTTTGTATCATCAGAGAGTGCTGATGCATGTTCAGATAGGAAAAAGCCCATCCATTTT

TGCATCTTTGTATCCTGGTACTCTCTTGCTGATTGAAATGGTAAATATGAACGGTCAATCATATCAAATCCT

TTCTATGCAGAGGCAAGGGTATTTTTATCAAATTGAATCGTAAAACCTTGAATTCCCCCACCTGTGTAACAT

TCTTTAAAGCGATTGATTACCTCAGTATAGATTATCACAGATGAGCTTGTTGGCTTAATGCTAAATGTAAAT

TCCAATGGTAATCGGTTTTCAGATTTAGCATGTACTAGTCGTATCGATATTTCAGTTGTTTTGAGTTTTCTC

TGACGAAGTTTTGAAGTTGCTGTTTCAACGATTCCATGTAAAAATCTTTCAAGCATTTCAATATCATTACAT

CCTTTGCTACGGATTTCTGAAAATTGTACTGTATTTTTTTCTTGTTTCATTTTAATCCCTCCAATCCACCCG

CGGAATGACCACCGATAAGTTTACTGCGTTCAATATTTCTGGAACCTTCAGTTAGGACGGTTCCTTTTTGTA

TGGCTAAAAAACCAAACTGTTCTCTGACAACATCAATAGCTGTCTGAAGTCTATTATCTTTTTCAATTTGTT

CTACATCATCAAAGAGTGATAGTAGAGTATAGCTTTCATCTACGAAGCCACTATAAGATACACCAATTTGTC

TCACTGCACCAGAGGTGTATTTTTTCGGAATAATACAAGTACATGACTCACCATTGTTTTGGGGAGATTTG

CGGGTTCAATTTTATTCTGAGCATTTATAGATTTTTTCATCTCAGTCCTAGAATAGCCAATATGAATAGAAA

CGACAGTAGTCAATACTAGGGCTACTGTTCCGTTCCACAGTATCATTTAAAAATCATT-TTCACACCCTTTC

GTCTATTAGTATAGAAGAAAGCTCTCAGCACA

>GA16531/Mega-2.II

CATGTTGAGGCGGTAAGTTTGCTAGTCAAGGAGTAAAACGACGAAGATTAGCATTTACTTCCGCCCATGCGA

TAGCTGTCCGTGATTGACAAGTGCTAGCACGCAGACAGAACGGAGATAGCGAACCGCTGAGTGTGTCGCTCT

GCTCGTAAAAGCTTAGAAACCTTTGAACGAAAGGGATAATGAAAGCCTTGATTGCAAGGCTTTTTGCTTTAT

GGTGGGTAAGTATCAGAGTGAGAAAATTTTTGGAATGAGTAGAAGTGATAGCTAGAAATTATCAGTTTCTA

TTTCCATTTACCCTGTGGGTACGTGTTTGTTTCCATTGACAAGGAGTTTGTGGGAATAGAAATGT

ACCCACCTTGTTTGAATCAAGTGAAGTGTAGTTGAAGGAAATCTGTTGAAAGCAATACTTCATTTTACCG

AATAAGTAATAATTTAGGCAACTTCAAATCGATTAAAAAAAACTATTTTAAAGGTTAAGAGTAGACAAAA

ATTGTCCACTCTTTTTTGCAAACTCAATTTATCAATAAATGAAATGAGGGAATGTAAAATG

AAATATTTTGAGGTTGAGTTAGAAAATCCTGATGAATTTTTAAAACTACAAACAGAAGATTTTGTGAAAG

CTAATCGCTTGCTACTAAGGAAGATAATCCAGAGCGTTACAGTCTATGAAGAAAACTTCGTCATATCCTT

TAAATCTGGCATCGAATTGGAAGTATGAGTCTCATTCCATAACTTTTATATTGAACATATCATCTTGTTGTG

TTATACTATAAATTGATATAAACAAAGATGTAGGAGGAACCGAAACTATGACAGCCTCAATGCGTTTAAGAT

AAGCTGGCAATAAAAAAAGCAGAATCTATACCCGATGATAGGCTTTTTTGTTGTGCTTATTTATACGATATT

GAGCATTCATTAGTTACGGTGAGGATATTGGTTATTTAACTATACCTTTATTTAACTATGTCTTTAATATGA

ATGTTTCCAAATTGTATGTATGCAGACCAAAAGCCACATTGTGGGGTTTGGCCTGCATTTTTTTTGCCTA

GAATGCTATTCAAAATAGAAATTCAAGCAAAATAATATGCAGGAGATAATATAAATGGAAAAATACAACAAT

TGGAAACGAAAATTTTATGCAATATGGGCAGGGCAAGCAGTATCATTAATCACTAGTGCCATCCTGCAAATG

GCGATTATTTTTTACCTTACAGAAAAAACAGGATCTGCGATGGTCTTGTCTATGGCTTCATTAGTAGGTTTT

TTACCCTATGCGATTTTGGGACCTGCCATTGGTGTGCTAGTGGATCGTCATGATAGGAAGAAGATAATGATT

GGTGCCGATTTAATTATCGCAGCAGCTGGTGCAGTGCTTGCTATTGTTGCATTCTGTATGGAGCTACCTGTC

TGGATGATTATGATAGTATTGTTTATCCGTAGCATTGGAACAGCTTTTCATACCCCAGCACTCAATGCGGTT

ACACCACTTTTAGTACCAGAAGAACAGCTAACGAAATGCGCAGGCTATAGTCAGTCTTTGCAGTCTATAAGC

TATATTGTTAGTCCGGCAGTTGCAGCACTCTTATACTCCGTTTGGGATTTAAATGCTATTATTGCCATCGAC

GTATTGGGTGCTGTGATTGCATCTATTACGGTAGCAATTGTACGTATACCTAAGCTGGGTAATCAAGTGCAA

AGTTTAGAACCAAATTTCATAAGGGAGATGAAAGAAGGAGTTGTGGTTCTGAGACAAAACAAAGGATTGTTT

GCCTTATTACTCTTAGGAACACTATATACTTTTGTTTATATGCCAATCAATGCACTATTTCCTTTAATAAGC

ATGGAACACTTTAATGGAACGCCTGTGCATATTTCTATTACGGAAATTTCCTTTGCATTTGGGATGCTAGCA

GGAGGCTTATTATTAGGAAGATTAGGGGGCTTCGAAAAGCATGTATTACTAATAACAAGTTCATTTTTTATA

ATGGGGACCAGTTTAGCCGTTTCGGGAATACTTCCTCCAAATGGATTTGTAATATTCGTAGTTTGCTGTGCA

ATAATGGGGCTTTCGGTGCCATTTTATAGCGGTGTGCAAACAGCTCTTTTTCAGGAGAAAATTAAGCCTGAA

TATTTAGGACGTGTATTTTCTTTGATCGGAAGTATCATGTCACTTGCTATGCCAATTGGGTTAATTCTTTCT

GGATTCTTTGCTGATAAAATCGGTGTAAATCATTGGTTTTTACTATCAGGTATTTTAATTATTGGCATTGCT

ATAGTTTGCCAAATGATAACTGAGGTTAGAAAATTAGATTTAAAATAAACAATATTGGAGGAA

ATGTTATGGAATTAATATT

AAAAGCAAAAGACATTCGTGTGGAATTCAAAGGACGCGATGTTTTAGATATAAATGAATTAGAAGTATATGA

TTATGACCGTATTGGTTTAGTAGGAGCAAATGGTGCTGGAAAAAGCACTTTACTCAGGGTACTTTTAGGAGA

ATTAACTCCCCCAGGATGTAAAATGAATCGTCTGGGTGAACTTGCCTATATTCCCCAGTTGGACGAAGTAAC

TCTGCAGGAGGAAAAAGATTTTGCACTTGTAGGCAAGCTAGGTGTTGAGCAATTAAATATACAGACTATGAG

CGGTGGTGAAGAAACAAGGCTTAAAATAGCACAGGCCTTATCGGCACAGGTTCATGGTATTTTAGCGGATGA

ACCTACGAGCCATTTAGACCGTGAAGGAATTGATTTTCTAATAGGACAGCTAAAATATTTTACAGGTGCACT

GTTAGTTATTAGCCATGACCGCTATTTTCTTGATGAAATAGTAGATAAAATATGGGAACTGAAAGATGGCAA

AATCACTGAGTATTGGGGAAACTATTCTGATTATCTTCGTCAGAAAGAGGAAGAACGTAAGAGCCAAGCTGC

AGAATACGAACAATTTATTGCGGAACGTGCCCGATTGGAAAGGGCTGCGGAGGAAAAGCGAAAACAGGCTCG

TAAAATAGAACAGAAGGCAAAAGGTTCTTCAAAGAAAAAAAGTACTGAAGACGGAGGGCGTTTAGCTCATCA

AAAATCAATAGGAAGTAAGGAAAAAAAGATGTATAATGCTGCTAAAACCCTAGAGCACAGGATTGCGGCCTT

AGGAAAAGTAGAAGCTCCGGAAGGCATTCGCAGAATTCGTTTCAGGCAAAGTAAAGCATTGGAGCTCCATAA

TCCATACCCTATAGTCGGTGCAGAAATTAATAAAGTATTTGGGGATAAGGCTCTGTTTGAAAATGCATCTTT

TCAAATTCCGTTAGGAGCAAAAGTGGCGTTAACTGGTGGTAATGGAATCGGAAAAACAACTTTAATCCAAAT

GATCTTAAACCATGAAGAAGGAATTTCTATTTCGCCTAAGGCAAAAATAGGTTACTTTGCACAGAATGGTTA

CAAGTACAACAGTAATCAGAATGTTATGGAGTTTATGCAGAAGGATTGTGACTACAATATATCAGAAATTCG

TTCAGTGCTAGCATCTATGGGGTTCAAACAGAACGATATTGGAAAAAGTTTATCTGTTTTAAGCGGTGGAGA

AATTATAAAATTGTTGCTTGCTAAAATGCTCATGGGTAGATATAACATCCTAATAATGGATGAACCCAGTAA

CTTCCTTGACATACCAAGTTTAGAGGCTTTGGAAATACTAATGAAGGAGTACACCGGAACTATCGTGTTTAT

CACCCACGATAAACGATTACTCGAAAATGTAGCAGATGTAGTTTATGAAATTAGAGATAAGAAAATAAATCT

GAAACATTAAATTTAAGGTAGTCGCTGGTCAGTATAGTCTGTTCTGGTTGGCGACTCCATTGTTAAAGAGTA

TAAAGACTTTAGATTTTATGAATATTAAAAATAGGAACAGTCAATTGAACTGCTCCTATTTTTCTGCTAAAT

ATATTGTAGTTTTCTTATATGTATAATGATAGATTAGCGGATTCTCATCTACGGTACTTACTTCAAATATGA

AGAAGTGATCGCGGTTATCTCTGGACTTTTCCTTATTGAGGACAAAGTAATTCTTACGTGAAGTCGCCATTG

TTTTTAGGATATCATCAGTTAGGAAGGTCAATGGAATATTCATGTTAGAGTAGCGGTAGAAGTCACGTTCAA

AATCTTGGTAGCTCTCGCTATAATAGTCCATTTGTAGGTGATTACGCTGAAACTCAAGCTGATTCATAGAGC

ACCTCCTCGACAAGTTCAATACTAATAATGTCTTTTAATTTCAAATTGATGTGACCTGTTGTAGTTTTTATC

AAAATGAAATCTTTGGTCAGACTTGGTATTGTTCCAGTGTAGGAAACACGCTTGTTTTTTTCAATCACTTGA

ATGCGTGTGCGTAGCTGCCCGGCGTATACTTGACTGAGGAGTAATAATTTCTTCTCTAGTGATAAGTCAGAC

ATGTACGTTACTTTGTTTGTATCATCAGAGAGTGCTGATGCATGTTCAGATAGGAAAAAGCCCATCCATTTT

TGCATCTTTGTATCCTGGTACTCTCTTGCTGATTGAAATGGTAAATATGAACGGTCAATCATATCAAATCCT

TTCTATGCAGAGGCAAGGGTATTTTTATCAAATTGAATCGTAAAACCTTGAATTCCCCCACCTGTGTAACAT

TCTTTAAAGCGATTGATTACCTCAGTATAGATTATCACAGATGAGCTTGTTGGCTTAATGCTAAATGTAAAT

TCCAATGGTAATCGGTTTTCAGATTTAGCATGTACTAGTCGTATCGATATTTCAGTTGTTTTGAGTTTTCTC

TGACGAAGTTTTGAAGTTGCTGTTTCAACGATTCCATGTAAAAATCTTTCAAGCATTTCAATATCATTACAT

CCTTTGCTACGGATTTCTGAAAATTGTACTGTATTTTTTTCTTGTTTCATTTTAATCCCTCCAATCCACCCG

CGGAATGACCACCGATAAGTTTACTGCGTTCAATATTTCTGGAACCTTCAGTTAGGACGGTTCCTTTTTGTA

TGGCTAAAAAACCAAACTGTTCTCTGACAACATCAATAGCTGTCTGAAGTCTATTATCTTTTTCAATTTGTT

CTACATCATCAAAGAGTGATAGTAGAGTATAGCTTTCATCTACGAAGCCACTATAAGATACACCAATTTGTC

TCACTGCACCAGAGGTGTATTTTTTCGGAATAATACAAGTACATGACTCACCATTGTTTTGGGGAGATTTG

CGGGTTCAATTTTATTCTGAGCATTTATAGATTTTTTCATCTCAGTCCTAGAATAGCCAATATGAATAGAAA

CGACAGTAGTCAATACTAGGGCTACTGTTCCGTTCCACAGTATCATTTAAAAATCATT-TTCACACCCTTTC

GTCTATTAGTATAGAAGAAAGCTCTCAGCACA

>GA58581/Mega-2.II

CATGTTGAGGCGGTAAGTTTGCTAGTCAAGGAGTAAAACGACGAAGATTAGCATTTACTTCCGCCCATGCGA

TAGCTGTCCGTGATTGACAAGTGCTAGCACGCAGACAGAACGGAGATAGCGAACCGCTGAGTGTGTCGCTCT

GCTCGTAAAAGCTTAGAAACCTTTGAACGAAAGGGATAATGAAAGCCTTGATTGCAAGGCTTTTTGCTTTAT

GGTGGGTAAGTATCAGAGTGAGAAAATTTTTGGAATGAGTAGAAGTGATAGCTAGAAATTATCAGTTTCTA

TTTCCATTTACCCTGTGGGTACGTGTTTGTTTCCATTGACAAGGAGTTTGTGGGAATAGAAATGT

ACCCACCTTGTTTGAATCAAGTGAAGTGTAGTTGAAGGAAATCTGTTGAAAGCAATACTTCATTTTACCG

AATAAGTAATAATTTAGGCAACTTCAAATCGATTAAAAAAAACTATTTTAAAGGTTAAGAGTAGACAAAA

ATTGTCCACTCTTTTTTGCAAACTCAATTTATCAATAAATGAAATGAGGGAATGTAAAATG

AAATATTTTGAGGTTGAGTTAGAAAATCCTGATGAATTTTTAAAACTACAAACAGAAGATTTTGTGAAAG

CTAATCGCTTGCTACTAAGGAAGATAATCCAGAGCGTTACAGTCTATGAAGAAAACTTCGTCATATCCTT

TAAATCTGGCATCGAATTGGAAGTATGAGTCTCATTCCATAACTTTTATATTGAACATATCATCTTGTTGTG

TTATACTATAAATTGATATAAACAAAGATGTAGGAGGAACCGAAACTATGACAGCCTCAATGCGTTTAAGAT

AAGCTGGCAATAAAAAAAGCAGAATCTATACCCGATGATAGGCTTTTTTGTTGTGCTTATTTATACGATATT

GAGCATTCATTAGTTACGGTGAGGATATTGGTTATTTAACTATACCTTTATTTAACTATGTCTTTAATATGA

ATGTTTCCAAATTGTATGTATGCAGACCAAAAGCCACATTGTGGGGTTTGGCCTGCATTTTTTTTGCCTA

GAATGCTATTCAAAATAGAAATTCAAGCAAAATAATATGCAGGAGATAATATAAATGGAAAAATACAACAAT

TGGAAACGAAAATTTTATGCAATATGGGCAGGGCAAGCAGTATCATTAATCACTAGTGCCATCCTGCAAATG

GCGATTATTTTTTACCTTACAGAAAAAACAGGATCTGCGATGGTCTTGTCTATGGCTTCATTAGTAGGTTTT

TTACCCTATGCGATTTTGGGACCTGCCATTGGTGTGCTAGTGGATCGTCATGATAGGAAGAAGATAATGATT

GGTGCCGATTTAATTATCGCAGCAGCTGGTGCAGTGCTTGCTATTGTTGCATTCTGTATGGAGCTACCTGTC

TGGATGATTATGATAGTATTGTTTATCCGTAGCATTGGAACAGCTTTTCATACCCCAGCACTCAATGCGGTT

ACACCACTTTTAGTACCAGAAGAACAGCTAACGAAATGCGCAGGCTATAGTCAGTCTTTGCAGTCTATAAGC

TATATTGTTAGTCCGGCAGTTGCAGCACTCTTATACTCCGTTTGGGATTTAAATGCTATTATTGCCATCGAC

GTATTGGGTGCTGTGATTGCATCTATTACGGTAGCAATTGTACGTATACCTAAGCTGGGTAATCAAGTGCAA

AGTTTAGAACCAAATTTCATAAGGGAGATGAAAGAAGGAGTTGTGGTTCTGAGACAAAACAAAGGATTGTTT

GCCTTATTACTCTTAGGAACACTATATACTTTTGTTTATATGCCAATCAATGCACTATTTCCTTTAATAAGC

ATGGAACACTTTAATGGAACGCCTGTGCATATTTCTATTACGGAAATTTCCTTTGCATTTGGGATGCTAGCA

GGAGGCTTATTATTAGGAAGATTAGGGGGCTTCGAAAAGCATGTATTACTAATAACAAGTTCATTTTTTATA

ATGGGGACCAGTTTAGCCGTTTCGGGAATACTTCCTCCAAATGGATTTGTAATATTCGTAGTTTGCTGTGCA

ATAATGGGGCTTTCGGTGCCATTTTATAGCGGTGTGCAAACAGCTCTTTTTCAGGAGAAAATTAAGCCTGAA

TATTTAGGACGTGTATTTTCTTTGATCGGAAGTATCATGTCACTTGCTATGCCAATTGGGTTAATTCTTTCT

GGATTCTTTGCTGATAAAATCGGTGTAAATCATTGGTTTTTACTATCAGGTATTTTAATTATTGGCATTGCT

ATAGTTTGCCAAATGATAACTGAGGTTAGAAAATTAGATTTAAAATAAACAATATTGGAGGAA

ATGTTATGGAATTAATATT

AAAAGCAAAAGACATTCGTGTGGAATTCAAAGGACGCGATGTTTTAGATATAAATGAATTAGAAGTATATGA

TTATGACCGTATTGGTTTAGTAGGAGCAAATGGTGCTGGAAAAAGCACTTTACTCAGGGTACTTTTAGGAGA

ATTAACTCCCCCAGGATGTAAAATGAATCGTCTGGGTGAACTTGCCTATATTCCCCAGTTGGACGAAGTAAC

TCTGCAGGAGGAAAAAGATTTTGCACTTGTAGGCAAGCTAGGTGTTGAGCAATTAAATATACAGACTATGAG

CGGTGGTGAAGAAACAAGGCTTAAAATAGCACAGGCCTTATCGGCACAGGTTCATGGTATTTTAGCGGATGA

ACCTACGAGCCATTTAGACCGTGAAGGAATTGATTTTCTAATAGGACAGCTAAAATATTTTACAGGTGCACT

GTTAGTTATTAGCCATGACCGCTATTTTCTTGATGAAATAGTAGATAAAATATGGGAACTGAAAGATGGCAA

AATCACTGAGTATTGGGGAAACTATTCTGATTATCTTCGTCAGAAAGAGGAAGAACGTAAGAGCCAAGCTGC

AGAATACGAACAATTTATTGCGGAACGTGCCCGATTGGAAAGGGCTGCGGAGGAAAAGCGAAAACAGGCTCG

TAAAATAGAACAGAAGGCAAAAGGTTCTTCAAAGAAAAAAAGTACTGAAGACGGAGGGCGTTTAGCTCATCA

AAAATCAATAGGAAGTAAGGAAAAAAAGATGTATAATGCTGCTAAAACCCTAGAGCACAGGATTGCGGCCTT

AGGAAAAGTAGAAGCTCCGGAAGGCATTCGCAGAATTCGTTTCAGGCAAAGTAAAGCATTGGAGCTCCATAA

TCCATACCCTATAGTCGGTGCAGAAATTAATAAAGTATTTGGGGATAAGGCTCTGTTTGAAAATGCATCTTT

TCAAATTCCGTTAGGAGCAAAAGTGGCGTTAACTGGTGGTAATGGAATCGGAAAAACAACTTTAATCCAAAT

GATCTTAAACCATGAAGAAGGAATTTCTATTTCGCCTAAGGCAAAAATAGGTTACTTTGCACAGAATGGTTA

CAAGTACAACAGTAATCAGAATGTTATGGAGTTTATGCAGAAGGATTGTGACTACAATATATCAGAAATTCG

TTCAGTGCTAGCATCTATGGGGTTCAAACAGAACGATATTGGAAAAAGTTTATCTGTTTTAAGCGGTGGAGA

AATTATAAAATTGTTGCTTGCTAAAATGCTCATGGGTAGATATAACATCCTAATAATGGATGAACCCAGTAA

CTTCCTTGACATACCAAGTTTAGAGGCTTTGGAAATACTAATGAAGGAGTACACCGGAACTATCGTGTTTAT

CACCCACGATAAACGATTACTCGAAAATGTAGCAGATGTAGTTTATGAAATTAGAGATAAGAAAATAAATCT

GAAACATTAAATTTAAGGTAGTCGCTGGTCAGTATAGTCTGTTCTGGTTGGCGACTCCATTGTTAAAGAGTA

TAAAGACTTTAGATTTTATGAATATTAAAAATAGGAACAGTCAATTGAACTGCTCCTATTTTTCTGCTAAAT

ATATTGTAGTTTTCTTATATGTATAATGATAGATTAGCGGATTCTCATCTACGGTACTTACTTCAAATATGA

AGAAGTGATCGCGGTTATCTCTGGACTTTTCCTTATTGAGGACAAAGTAATTCTTACGTGAAGTCGCCATTG

TTTTTAGGATATCATCAGTTAGGAAGGTCAATGGAATATTCATGTTAGAGTAGCGGTAGAAGTCACGTTCAA

AATCTTGGTAGCTCTCGCTATAATAGTCCATTTGTAGGTGATTACGCTGAAACTCAAGCTGATTCATAGAGC

ACCTCCTCGACAAGTTCAATACTAATAATGTCTTTTAATTTCAAATTGATGTGACCTGTTGTAGTTTTTATC

AAAATGAAATCTTTGGTCAGACTTGGTATTGTTCCAGTGTAGGAAACACGCTTGTTTTTTTCAATCACTTGA

ATGCGTGTGCGTAGCTGCCCGGCGTATACTTGACTGAGGAGTAATAATTTCTTCTCTAGTGATAAGTCAGAC

ATGTACGTTACTTTGTTTGTATCATCAGAGAGTGCTGATGCATGTTCAGATAGGAAAAAGCCCATCCATTTT

TGCATCTTTGTATCCTGGTACTCTCTTGCTGATTGAAATGGTAAATATGAACGGTCAATCATATCAAATCCT

TTCTATGCAGAGGCAAGGGTATTTTTATCAAATTGAATCGTAAAACCTTGAATTCCCCCACCTGTGTAACAT

TCTTTAAAGCGATTGATTACCTCAGTATAGATTATCACAGATGAGCTTGTTGGCTTAATGCTAAATGTAAAT

TCCAATGGTAATCGGTTTTCAGATTTAGCATGTACTAGTCGTATCGATATTTCAGTTGTTTTGAGTTTTCTC

TGACGAAGTTTTGAAGTTGCTGTTTCAACGATTCCATGTAAAAATCTTTCAAGCATTTCAATATCATTACAT

CCTTTGCTACGGATTTCTGAAAATTGTACTGTATTTTTTTCTTGTTTCATTTTAATCCCTCCAATCCACCCG

CGGAATGACCACCGATAAGTTTACTGCGTTCAATATTTCTGGAACCTTCAGTTAGGACGGTTCCTTTTTGTA

TGGCTAAAAAACCAAACTGTTCTCTGACAACATCAATAGCTGTCTGAAGTCTATTATCTTTTTCAATTTGTT

CTACATCATCAAAGAGTGATAGTAGAGTATAGCTTTCATCTACGAAGCCACTATAAGATACACCAATTTGTC

TCACTGCACCAGAGGTGTATTTTTTCGGAATAATACAAGTACATGACTCACCATTGTTTTGGGGAGATTTG

CGGGTTCAATTTTATTCTGAGCATTTATAGATTTTTTCATCTCAGTCCTAGAATAGCCAATATGAATAGAAA

CGACAGTAGTCAATACTAGGGCTACTGTTCCGTTCCACAGTATCATTTAAAAATCATT-TTCACACCCTTTC

GTCTATTAGTATAGAAGAAAGCTCTCAGCACA

>GA56348/Mega-2.II

CATGTTGAGGCGGTAAGTTTGCTAGTCAAGGAGTAAAACGACGAAGATTAGCATTTACTTCCGCCCATGCGA

TAGCTGTCCGTGATTGACAAGTGCTAGCACGCAGACAGAACGGAGATAGCGAACCGCTGAGTGTGTCGCTCT

GCTCGTAAAAGCTTAGAAACCTTTGAACGAAAGGGATAATGAAAGCCTTGATTGCAAGGCTTTTTGCTTTAT

GGTGGGTAAGTATCAGAGTGAGAAAATTTTTGGAATGAGTAGAAGTGATAGCTAGAAATTATCAGTTTCTA

TTTCCATTTACCCTGTGGGTACGTGTTTGTTTCCATTGACAAGGAGTTTGTGGGAATAGAAATGT

ACCCACCTTGTTTGAATCAAGTGAAGTGTAGTTGAAGGAAATCTGTTGAAAGCAATACTTCATTTTACCG

AATAAGTAATAATTTAGGCAACTTCAAATCGATTAAAAAAAACTATTTTAAAGGTTAAGAGTAGACAAAA

ATTGTCCACTCTTTTTTGCAAACTCAATTTATCAATAAATGAAATGAGGGAATGTAAAATG

AAATATTTTGAGGTTGAGTTAGAAAATCCTGATGAATTTTTAAAACTACAAACAGAAGATTTTGTGAAAG

CTAATCGCTTGCTACTAAGGAAGATAATCCAGAGCGTTACAGTCTATGAAGAAAACTTCGTCATATCCTT

TAAATCTGGCATCGAATTGGAAGTATGAGTCTCATTCCATAACTTTTATATTGAACATATCATCTTGTTGTG

TTATACTATAAATTGATATAAACAAAGATGTAGGAGGAACCGAAACTATGACAGCCTCAATGCGTTTAAGAT

AAGCTGGCAATAAAAAAAGCAGAATCTATACCCGATGATAGGCTTTTTTGTTGTGCTTATTTATACGATATT

GAGCATTCATTAGTTACGGTGAGGATATTGGTTATTTAACTATACCTTTATTTAACTATGTCTTTAATATGA

ATGTTTCCAAATTGTATGTATGCAGACCAAAAGCCACATTGTGGGGTTTGGCCTGCATTTTTTTTGCCTA

GAATGCTATTCAAAATAGAAATTCAAGCAAAATAATATGCAGGAGATAATATAAATGGAAAAATACAACAAT

TGGAAACGAAAATTTTATGCAATATGGGCAGGGCAAGCAGTATCATTAATCACTAGTGCCATCCTGCAAATG

GCGATTATTTTTTACCTTACAGAAAAAACAGGATCTGCGATGGTCTTGTCTATGGCTTCATTAGTAGGTTTT

TTACCCTATGCGATTTTGGGACCTGCCATTGGTGTGCTAGTGGATCGTCATGATAGGAAGAAGATAATGATT

GGTGCCGATTTAATTATCGCAGCAGCTGGTGCAGTGCTTGCTATTGTTGCATTCTGTATGGAGCTACCTGTC

TGGATGATTATGATAGTATTGTTTATCCGTAGCATTGGAACAGCTTTTCATACCCCAGCACTCAATGCGGTT

ACACCACTTTTAGTACCAGAAGAACAGCTAACGAAATGCGCAGGCTATAGTCAGTCTTTGCAGTCTATAAGC

TATATTGTTAGTCCGGCAGTTGCAGCACTCTTATACTCCGTTTGGGATTTAAATGCTATTATTGCCATCGAC

GTATTGGGTGCTGTGATTGCATCTATTACGGTAGCAATTGTACGTATACCTAAGCTGGGTAATCAAGTGCAA

AGTTTAGAACCAAATTTCATAAGGGAGATGAAAGAAGGAGTTGTGGTTCTGAGACAAAACAAAGGATTGTTT

GCCTTATTACTCTTAGGAACACTATATACTTTTGTTTATATGCCAATCAATGCACTATTTCCTTTAATAAGC

ATGGAACACTTTAATGGAACGCCTGTGCATATTTCTATTACGGAAATTTCCTTTGCATTTGGGATGCTAGCA

GGAGGCTTATTATTAGGAAGATTAGGGGGCTTCGAAAAGCATGTATTACTAATAACAAGTTCATTTTTTATA

ATGGGGACCAGTTTAGCCGTTTCGGGAATACTTCCTCCAAATGGATTTGTAATATTCGTAGTTTGCTGTGCA

ATAATGGGGCTTTCGGTGCCATTTTATAGCGGTGTGCAAACAGCTCTTTTTCAGGAGAAAATTAAGCCTGAA

TATTTAGGACGTGTATTTTCTTTGATCGGAAGTATCATGTCACTTGCTATGCCAATTGGGTTAATTCTTTCT

GGATTCTTTGCTGATAAAATCGGTGTAAATCATTGGTTTTTACTATCAGGTATTTTAATTATTGGCATTGCT

ATAGTTTGCCAAATGATAACTGAGGTTAGAAAATTAGATTTAAAATAAACAATATTGGAGGAA

ATGTTATGGAATTAATATT

AAAAGCAAAAGACATTCGTGTGGAATTCAAAGGACGCGATGTTTTAGATATAAATGAATTAGAAGTATATGA

TTATGACCGTATTGGTTTAGTAGGAGCAAATGGTGCTGGAAAAAGCACTTTACTCAGGGTACTTTTAGGAGA

ATTAACTCCCCCAGGATGTAAAATGAATCGTCTGGGTGAACTTGCCTATATTCCCCAGTTGGACGAAGTAAC

TCTGCAGGAGGAAAAAGATTTTGCACTTGTAGGCAAGCTAGGTGTTGAGCAATTAAATATACAGACTATGAG

CGGTGGTGAAGAAACAAGGCTTAAAATAGCACAGGCCTTATCGGCACAGGTTCATGGTATTTTAGCGGATGA

ACCTACGAGCCATTTAGACCGTGAAGGAATTGATTTTCTAATAGGACAGCTAAAATATTTTACAGGTGCACT

GTTAGTTATTAGCCATGACCGCTATTTTCTTGATGAAATAGTAGATAAAATATGGGAACTGAAAGATGGCAA

AATCACTGAGTATTGGGGAAACTATTCTGATTATCTTCGTCAGAAAGAGGAAGAACGTAAGAGCCAAGCTGC

AGAATACGAACAATTTATTGCGGAACGTGCCCGATTGGAAAGGGCTGCGGAGGAAAAGCGAAAACAGGCTCG

TAAAATAGAACAGAAGGCAAAAGGTTCTTCAAAGAAAAAAAGTACTGAAGACGGAGGGCGTTTAGCTCATCA

AAAATCAATAGGAAGTAAGGAAAAAAAGATGTATAATGCTGCTAAAACCCTAGAGCACAGGATTGCGGCCTT

AGGAAAAGTAGAAGCTCCGGAAGGCATTCGCAGAATTCGTTTCAGGCAAAGTAAAGCATTGGAGCTCCATAA

TCCATACCCTATAGTCGGTGCAGAAATTAATAAAGTATTTGGGGATAAGGCTCTGTTTGAAAATGCATCTTT

TCAAATTCCGTTAGGAGCAAAAGTGGCGTTAACTGGTGGTAATGGAATCGGAAAAACAACTTTAATCCAAAT

GATCTTAAACCATGAAGAAGGAATTTCTATTTCGCCTAAGGCAAAAATAGGTTACTTTGCACAGAATGGTTA

CAAGTACAACAGTAATCAGAATGTTATGGAGTTTATGCAGAAGGATTGTGACTACAATATATCAGAAATTCG

TTCAGTGCTAGCATCTATGGGGTTCAAACAGAACGATATTGGAAAAAGTTTATCTGTTTTAAGCGGTGGAGA

AATTATAAAATTGTTGCTTGCTAAAATGCTCATGGGTAGATATAACATCCTAATAATGGATGAACCCAGTAA

CTTCCTTGACATACCAAGTTTAGAGGCTTTGGAAATACTAATGAAGGAGTACACCGGAACTATCGTGTTTAT

CACCCACGATAAACGATTACTCGAAAATGTAGCAGATGTAGTTTATGAAATTAGAGATAAGAAAATAAATCT

GAAACATTAAATTTAAGGTAGTCGCTGGTCAGTATAGTCTGTTCTGGTTGGCGACTCCATTGTTAAAGAGTA

TAAAGACTTTAGATTTTATGAATATTAAAAATAGGAACAGTCAATTGAACTGCTCCTATTTTTCTGCTAAAT

ATATTGTAGTTTTCTTATATGTATAATGATAGATTAGCGGATTCTCATCTACGGTACTTACTTCAAATATGA

AGAAGTGATCGCGGTTATCTCTGGACTTTTCCTTATTGAGGACAAAGTAATTCTTACGTGAAGTCGCCATTG

TTTTTAGGATATCATCAGTTAGGAAGGTCAATGGAATATTCATGTTAGAGTAGCGGTAGAAGTCACGTTCAA

AATCTTGGTAGCTCTCGCTATAATAGTCCATTTGTAGGTGATTACGCTGAAACTCAAGCTGATTCATAGAGC

ACCTCCTCGACAAGTTCAATACTAATAATGTCTTTTAATTTCAAATTGATGTGACCTGTTGTAGTTTTTATC

AAAATGAAATCTTTGGTCAGACTTGGTATTGTTCCAGTGTAGGAAACACGCTTGTTTTTTTCAATCACTTGA

ATGCGTGTGCGTAGCTGCCCGGCGTATACTTGACTGAGGAGTAATAATTTCTTCTCTAGTGATAAGTCAGAC

ATGTACGTTACTTTGTTTGTATCATCAGAGAGTGCTGATGCATGTTCAGATAGGAAAAAGCCCATCCATTTT

TGCATCTTTGTATCCTGGTACTCTCTTGCTGATTGAAATGGTAAATATGAACGGTCAATCATATCAAATCCT

TTCTATGCAGAGGCAAGGGTATTTTTATCAAATTGAATCGTAAAACCTTGAATTCCCCCACCTGTGTAACAT

TCTTTAAAGCGATTGATTACCTCAGTATAGATTATCACAGATGAGCTTGTTGGCTTAATGCTAAATGTAAAT

TCCAATGGTAATCGGTTTTCAGATTTAGCATGTACTAGTCGTATCGATATTTCAGTTGTTTTGAGTTTTCTC

TGACGAAGTTTTGAAGTTGCTGTTTCAACGATTCCATGTAAAAATCTTTCAAGCATTTCAATATCATTACAT

CCTTTGCTACGGATTTCTGAAAATTGTACTGTATTTTTTTCTTGTTTCATTTTAATCCCTCCAATCCACCCG

CGGAATGACCACCGATAAGTTTACTGCGTTCAATATTTCTGGAACCTTCAGTTAGGACGGTTCCTTTTTGTA

TGGCTAAAAAACCAAACTGTTCTCTGACAACATCAATAGCTGTCTGAAGTCTATTATCTTTTTCAATTTGTT

CTACATCATCAAAGAGTGATAGTAGAGTATAGCTTTCATCTACGAAGCCACTATAAGATACACCAATTTGTC

TCACTGCACCAGAGGTGTATTTTTTCGGAATAATACAAGTACATGACTCACCATTGTTTTGGGGAGATTTG

CGGGTTCAATTTTATTCTGAGCATTTATAGATTTTTTCATCTCAGTCCTAGAATAGCCAATATGAATAGAAA

CGACAGTAGTCAATACTAGGGCTACTGTTCCGTTCCACAGTATCATTTAAAAATCATT-TTCACACCCTTTC

GTCTATTAGTATAGAAGAAAGCTCTCAGCACA

>GA56113/Mega-2.II

CATGTTGAGGCGGTAAGTTTGCTAGTCAAGGAGTAAAACGACGAAGATTAGCATTTACTTCCGCCCATGCGA

TAGCTGTCCGTGATTGACAAGTGCTAGCACGCAGACAGAACGGAGATAGCGAACCGCTGAGTGTGTCGCTCT

GCTCGTAAAAGCTTAGAAACCTTTGAACGAAAGGGATAATGAAAGCCTTGATTGCAAGGCTTTTTGCTTTAT

GGTGGGTAAGTATCAGAGTGAGAAAATTTTTGGAATGAGTAGAAGTGATAGCTAGAAATTATCAGTTTCTA

TTTCCATTTACCCTGTGGGTACGTGTTTGTTTCCATTGACAAGGAGTTTGTGGGAATAGAAATGT

ACCCACCTTGTTTGAATCAAGTGAAGTGTAGTTGAAGGAAATCTGTTGAAAGCAATACTTCATTTTACCG

AATAAGTAATAATTTAGGCAACTTCAAATCGATTAAAAAAAACTATTTTAAAGGTTAAGAGTAGACAAAA

ATTGTCCACTCTTTTTTGCAAACTCAATTTATCAATAAATGAAATGAGGGAATGTAAAATG

AAATATTTTGAGGTTGAGTTAGAAAATCCTGATGAATTTTTAAAACTACAAACAGAAGATTTTGTGAAAG

CTAATCGCTTGCTACTAAGGAAGATAATCCAGAGCGTTACAGTCTATGAAGAAAACTTCGTCATATCCTT

TAAATCTGGCATCGAATTGGAAGTATGAGTCTCATTCCATAACTTTTATATTGAACATATCATCTTGTTGTG

TTATACTATAAATTGATATAAACAAAGATGTAGGAGGAACCGAAACTATGACAGCCTCAATGCGTTTAAGAT

AAGCTGGCAATAAAAAAAGCAGAATCTATACCCGATGATAGGCTTTTTTGTTGTGCTTATTTATACGATATT

GAGCATTCATTAGTTACGGTGAGGATATTGGTTATTTAACTATACCTTTATTTAACTATGTCTTTAATATGA

ATGTTTCCAAATTGTATGTATGCAGACCAAAAGCCACATTGTGGGGTTTGGCCTGCATTTTTTTTGCCTA

GAATGCTATTCAAAATAGAAATTCAAGCAAAATAATATGCAGGAGATAATATAAATGGAAAAATACAACAAT

TGGAAACGAAAATTTTATGCAATATGGGCAGGGCAAGCAGTATCATTAATCACTAGTGCCATCCTGCAAATG

GCGATTATTTTTTACCTTACAGAAAAAACAGGATCTGCGATGGTCTTGTCTATGGCTTCATTAGTAGGTTTT

TTACCCTATGCGATTTTGGGACCTGCCATTGGTGTGCTAGTGGATCGTCATGATAGGAAGAAGATAATGATT

GGTGCCGATTTAATTATCGCAGCAGCTGGTGCAGTGCTTGCTATTGTTGCATTCTGTATGGAGCTACCTGTC

TGGATGATTATGATAGTATTGTTTATCCGTAGCATTGGAACAGCTTTTCATACCCCAGCACTCAATGCGGTT

ACACCACTTTTAGTACCAGAAGAACAGCTAACGAAATGCGCAGGCTATAGTCAGTCTTTGCAGTCTATAAGC

TATATTGTTAGTCCGGCAGTTGCAGCACTCTTATACTCCGTTTGGGATTTAAATGCTATTATTGCCATCGAC

GTATTGGGTGCTGTGATTGCATCTATTACGGTAGCAATTGTACGTATACCTAAGCTGGGTAATCAAGTGCAA

AGTTTAGAACCAAATTTCATAAGGGAGATGAAAGAAGGAGTTGTGGTTCTGAGACAAAACAAAGGATTGTTT

GCCTTATTACTCTTAGGAACACTATATACTTTTGTTTATATGCCAATCAATGCACTATTTCCTTTAATAAGC

ATGGAACACTTTAATGGAACGCCTGTGCATATTTCTATTACGGAAATTTCCTTTGCATTTGGGATGCTAGCA

GGAGGCTTATTATTAGGAAGATTAGGGGGCTTCGAAAAGCATGTATTACTAATAACAAGTTCATTTTTTATA

ATGGGGACCAGTTTAGCCGTTTCGGGAATACTTCCTCCAAATGGATTTGTAATATTCGTAGTTTGCTGTGCA

ATAATGGGGCTTTCGGTGCCATTTTATAGCGGTGTGCAAACAGCTCTTTTTCAGGAGAAAATTAAGCCTGAA

TATTTAGGACGTGTATTTTCTTTGATCGGAAGTATCATGTCACTTGCTATGCCAATTGGGTTAATTCTTTCT

GGATTCTTTGCTGATAAAATCGGTGTAAATCATTGGTTTTTACTATCAGGTATTTTAATTATTGGCATTGCT

ATAGTTTGCCAAATGATAACTGAGGTTAGAAAATTAGATTTAAAATAAACAATATTGGAGGAA

ATGTTATGGAATTAATATT

AAAAGCAAAAGACATTCGTGTGGAATTCAAAGGACGCGATGTTTTAGATATAAATGAATTAGAAGTATATGA

TTATGACCGTATTGGTTTAGTAGGAGCAAATGGTGCTGGAAAAAGCACTTTACTCAGGGTACTTTTAGGAGA

ATTAACTCCCCCAGGATGTAAAATGAATCGTCTGGGTGAACTTGCCTATATTCCCCAGTTGGACGAAGTAAC

TCTGCAGGAGGAAAAAGATTTTGCACTTGTAGGCAAGCTAGGTGTTGAGCAATTAAATATACAGACTATGAG

CGGTGGTGAAGAAACAAGGCTTAAAATAGCACAGGCCTTATCGGCACAGGTTCATGGTATTTTAGCGGATGA

ACCTACGAGCCATTTAGACCGTGAAGGAATTGATTTTCTAATAGGACAGCTAAAATATTTTACAGGTGCACT

GTTAGTTATTAGCCATGACCGCTATTTTCTTGATGAAATAGTAGATAAAATATGGGAACTGAAAGATGGCAA

AATCACTGAGTATTGGGGAAACTATTCTGATTATCTTCGTCAGAAAGAGGAAGAACGTAAGAGCCAAGCTGC

AGAATACGAACAATTTATTGCGGAACGTGCCCGATTGGAAAGGGCTGCGGAGGAAAAGCGAAAACAGGCTCG

TAAAATAGAACAGAAGGCAAAAGGTTCTTCAAAGAAAAAAAGTACTGAAGACGGAGGGCGTTTAGCTCATCA

AAAATCAATAGGAAGTAAGGAAAAAAAGATGTATAATGCTGCTAAAACCCTAGAGCACAGGATTGCGGCCTT

AGGAAAAGTAGAAGCTCCGGAAGGCATTCGCAGAATTCGTTTCAGGCAAAGTAAAGCATTGGAGCTCCATAA

TCCATACCCTATAGTCGGTGCAGAAATTAATAAAGTATTTGGGGATAAGGCTCTGTTTGAAAATGCATCTTT

TCAAATTCCGTTAGGAGCAAAAGTGGCGTTAACTGGTGGTAATGGAATCGGAAAAACAACTTTAATCCAAAT

GATCTTAAACCATGAAGAAGGAATTTCTATTTCGCCTAAGGCAAAAATAGGTTACTTTGCACAGAATGGTTA

CAAGTACAACAGTAATCAGAATGTTATGGAGTTTATGCAGAAGGATTGTGACTACAATATATCAGAAATTCG

TTCAGTGCTAGCATCTATGGGGTTCAAACAGAACGATATTGGAAAAAGTTTATCTGTTTTAAGCGGTGGAGA

AATTATAAAATTGTTGCTTGCTAAAATGCTCATGGGTAGATATAACATCCTAATAATGGATGAACCCAGTAA

CTTCCTTGACATACCAAGTTTAGAGGCTTTGGAAATACTAATGAAGGAGTACACCGGAACTATCGTGTTTAT

CACCCACGATAAACGATTACTCGAAAATGTAGCAGATGTAGTTTATGAAATTAGAGATAAGAAAATAAATCT

GAAACATTAAATTTAAGGTAGTCGCTGGTCAGTATAGTCTGTTCTGGTTGGCGACTCCATTGTTAAAGAGTA

TAAAGACTTTAGATTTTATGAATATTAAAAATAGGAACAGTCAATTGAACTGCTCCTATTTTTCTGCTAAAT

ATATTGTAGTTTTCTTATATGTATAATGATAGATTAGCGGATTCTCATCTACGGTACTTACTTCAAATATGA

AGAAGTGATCGCGGTTATCTCTGGACTTTTCCTTATTGAGGACAAAGTAATTCTTACGTGAAGTCGCCATTG

TTTTTAGGATATCATCAGTTAGGAAGGTCAATGGAATATTCATGTTAGAGTAGCGGTAGAAGTCACGTTCAA

AATCTTGGTAGCTCTCGCTATAATAGTCCATTTGTAGGTGATTACGCTGAAACTCAAGCTGATTCATAGAGC

ACCTCCTCGACAAGTTCAATACTAATAATGTCTTTTAATTTCAAATTGATGTGACCTGTTGTAGTTTTTATC

AAAATGAAATCTTTGGTCAGACTTGGTATTGTTCCAGTGTAGGAAACACGCTTGTTTTTTTCAATCACTTGA

ATGCGTGTGCGTAGCTGCCCGGCGTATACTTGACTGAGGAGTAATAATTTCTTCTCTAGTGATAAGTCAGAC

ATGTACGTTACTTTGTTTGTATCATCAGAGAGTGCTGATGCATGTTCAGATAGGAAAAAGCCCATCCATTTT

TGCATCTTTGTATCCTGGTACTCTCTTGCTGATTGAAATGGTAAATATGAACGGTCAATCATATCAAATCCT

TTCTATGCAGAGGCAAGGGTATTTTTATCAAATTGAATCGTAAAACCTTGAATTCCCCCACCTGTGTAACAT

TCTTTAAAGCGATTGATTACCTCAGTATAGATTATCACAGATGAGCTTGTTGGCTTAATGCTAAATGTAAAT

TCCAATGGTAATCGGTTTTCAGATTTAGCATGTACTAGTCGTATCGATATTTCAGTTGTTTTGAGTTTTCTC

TGACGAAGTTTTGAAGTTGCTGTTTCAACGATTCCATGTAAAAATCTTTCAAGCATTTCAATATCATTACAT

CCTTTGCTACGGATTTCTGAAAATTGTACTGTATTTTTTTCTTGTTTCATTTTAATCCCTCCAATCCACCCG

CGGAATGACCACCGATAAGTTTACTGCGTTCAATATTTCTGGAACCTTCAGTTAGGACGGTTCCTTTTTGTA

TGGCTAAAAAACCAAACTGTTCTCTGACAACATCAATAGCTGTCTGAAGTCTATTATCTTTTTCAATTTGTT

CTACATCATCAAAGAGTGATAGTAGAGTATAGCTTTCATCTACGAAGCCACTATAAGATACACCAATTTGTC

TCACTGCACCAGAGGTGTATTTTTTCGGAATAATACAAGTACATGACTCACCATTGTTTTGGGGAGATTTG

CGGGTTCAATTTTATTCTGAGCATTTATAGATTTTTTCATCTCAGTCCTAGAATAGCCAATATGAATAGAAA

CGACAGTAGTCAATACTAGGGCTACTGTTCCGTTCCACAGTATCATTTAAAAATCATT-TTCACACCCTTTC

GTCTATTAGTATAGAAGAAAGCTCTCAGCACA

>GA47562/Mega-2.II

CATGTTGAGGCGGTAAGTTTGCTAGTCAAGGAGTAAAACGACGAAGATTAGCATTTACTTCCGCCCATGCGA

TAGCTGTCCGTGATTGACAAGTGCTAGCACGCAGACAGAACGGAGATAGCGAACCGCTGAGTGTGTCGCTCT

GCTCGTAAAAGCTTAGAAACCTTTGAACGAAAGGGATAATGAAAGCCTTGATTGCAAGGCTTTTTGCTTTAT

GGTGGGTAAGTATCAGAGTGAGAAAATTTTTGGAATGAGTAGAAGTGATAGCTAGAAATTATCAGTTTCTA

TTTCCATTTACCCTGTGGGTACGTGTTTGTTTCCATTGACAAGGAGTTTGTGGGAATAGAAATGT

ACCCACCTTGTTTGAATCAAGTGAAGTGTAGTTGAAGGAAATCTGTTGAAAGCAATACTTCATTTTACCG

AATAAGTAATAATTTAGGCAACTTCAAATCGATTAAAAAAAACTATTTTAAAGGTTAAGAGTAGACAAAA

ATTGTCCACTCTTTTTTGCAAACTCAATTTATCAATAAATGAAATGAGGGAATGTAAAATG

AAATATTTTGAGGTTGAGTTAGAAAATCCTGATGAATTTTTAAAACTACAAACAGAAGATTTTGTGAAAG

CTAATCGCTTGCTACTAAGGAAGATAATCCAGAGCGTTACAGTCTATGAAGAAAACTTCGTCATATCCTT

TAAATCTGGCATCGAATTGGAAGTATGAGTCTCATTCCATAACTTTTATATTGAACATATCATCTTGTTGTG

TTATACTATAAATTGATATAAACAAAGATGTAGGAGGAACCGAAACTATGACAGCCTCAATGCGTTTAAGAT

AAGCTGGCAATAAAAAAAGCAGAATCTATACCCGATGATAGGCTTTTTTGTTGTGCTTATTTATACGATATT

GAGCATTCATTAGTTACGGTGAGGATATTGGTTATTTAACTATACCTTTATTTAACTATGTCTTTAATATGA

ATGTTTCCAAATTGTATGTATGCAGACCAAAAGCCACATTGTGGGGTTTGGCCTGCATTTTTTTTGCCTA

GAATGCTATTCAAAATAGAAATTCAAGCAAAATAATATGCAGGAGATAATATAAATGGAAAAATACAACAAT

TGGAAACGAAAATTTTATGCAATATGGGCAGGGCAAGCAGTATCATTAATCACTAGTGCCATCCTGCAAATG

GCGATTATTTTTTACCTTACAGAAAAAACAGGATCTGCGATGGTCTTGTCTATGGCTTCATTAGTAGGTTTT

TTACCCTATGCGATTTTGGGACCTGCCATTGGTGTGCTAGTGGATCGTCATGATAGGAAGAAGATAATGATT

GGTGCCGATTTAATTATCGCAGCAGCTGGTGCAGTGCTTGCTATTGTTGCATTCTGTATGGAGCTACCTGTC

TGGATGATTATGATAGTATTGTTTATCCGTAGCATTGGAACAGCTTTTCATACCCCAGCACTCAATGCGGTT

ACACCACTTTTAGTACCAGAAGAACAGCTAACGAAATGCGCAGGCTATAGTCAGTCTTTGCAGTCTATAAGC

TATATTGTTAGTCCGGCAGTTGCAGCACTCTTATACTCCGTTTGGGATTTAAATGCTATTATTGCCATCGAC

GTATTGGGTGCTGTGATTGCATCTATTACGGTAGCAATTGTACGTATACCTAAGCTGGGTAATCAAGTGCAA

AGTTTAGAACCAAATTTCATAAGGGAGATGAAAGAAGGAGTTGTGGTTCTGAGACAAAACAAAGGATTGTTT

GCCTTATTACTCTTAGGAACACTATATACTTTTGTTTATATGCCAATCAATGCACTATTTCCTTTAATAAGC

ATGGAACACTTTAATGGAACGCCTGTGCATATTTCTATTACGGAAATTTCCTTTGCATTTGGGATGCTAGCA

GGAGGCTTATTATTAGGAAGATTAGGGGGCTTCGAAAAGCATGTATTACTAATAACAAGTTCATTTTTTATA

ATGGGGACCAGTTTAGCCGTTTCGGGAATACTTCCTCCAAATGGATTTGTAATATTCGTAGTTTGCTGTGCA

ATAATGGGGCTTTCGGTGCCATTTTATAGCGGTGTGCAAACAGCTCTTTTTCAGGAGAAAATTAAGCCTGAA

TATTTAGGACGTGTATTTTCTTTGATCGGAAGTATCATGTCACTTGCTATGCCAATTGGGTTAATTCTTTCT

GGATTCTTTGCTGATAAAATCGGTGTAAATCATTGGTTTTTACTATCAGGTATTTTAATTATTGGCATTGCT

ATAGTTTGCCAAATGATAACTGAGGTTAGAAAATTAGATTTAAAATAAACAATATTGGAGGAA

ATGTTATGGAATTAATATT

AAAAGCAAAAGACATTCGTGTGGAATTCAAAGGACGCGATGTTTTAGATATAAATGAATTAGAAGTATATGA

TTATGACCGTATTGGTTTAGTAGGAGCAAATGGTGCTGGAAAAAGCACTTTACTCAGGGTACTTTTAGGAGA

ATTAACTCCCCCAGGATGTAAAATGAATCGTCTGGGTGAACTTGCCTATATTCCCCAGTTGGACGAAGTAAC

TCTGCAGGAGGAAAAAGATTTTGCACTTGTAGGCAAGCTAGGTGTTGAGCAATTAAATATACAGACTATGAG

CGGTGGTGAAGAAACAAGGCTTAAAATAGCACAGGCCTTATCGGCACAGGTTCATGGTATTTTAGCGGATGA

ACCTACGAGCCATTTAGACCGTGAAGGAATTGATTTTCTAATAGGACAGCTAAAATATTTTACAGGTGCACT

GTTAGTTATTAGCCATGACCGCTATTTTCTTGATGAAATAGTAGATAAAATATGGGAACTGAAAGATGGCAA

AATCACTGAGTATTGGGGAAACTATTCTGATTATCTTCGTCAGAAAGAGGAAGAACGTAAGAGCCAAGCTGC

AGAATACGAACAATTTATTGCGGAACGTGCCCGATTGGAAAGGGCTGCGGAGGAAAAGCGAAAACAGGCTCG

TAAAATAGAACAGAAGGCAAAAGGTTCTTCAAAGAAAAAAAGTACTGAAGACGGAGGGCGTTTAGCTCATCA

AAAATCAATAGGAAGTAAGGAAAAAAAGATGTATAATGCTGCTAAAACCCTAGAGCACAGGATTGCGGCCTT

AGGAAAAGTAGAAGCTCCGGAAGGCATTCGCAGAATTCGTTTCAGGCAAAGTAAAGCATTGGAGCTCCATAA

TCCATACCCTATAGTCGGTGCAGAAATTAATAAAGTATTTGGGGATAAGGCTCTGTTTGAAAATGCATCTTT

TCAAATTCCGTTAGGAGCAAAAGTGGCGTTAACTGGTGGTAATGGAATCGGAAAAACAACTTTAATCCAAAT

GATCTTAAACCATGAAGAAGGAATTTCTATTTCGCCTAAGGCAAAAATAGGTTACTTTGCACAGAATGGTTA

CAAGTACAACAGTAATCAGAATGTTATGGAGTTTATGCAGAAGGATTGTGACTACAATATATCAGAAATTCG

TTCAGTGCTAGCATCTATGGGGTTCAAACAGAACGATATTGGAAAAAGTTTATCTGTTTTAAGCGGTGGAGA

AATTATAAAATTGTTGCTTGCTAAAATGCTCATGGGTAGATATAACATCCTAATAATGGATGAACCCAGTAA

CTTCCTTGACATACCAAGTTTAGAGGCTTTGGAAATACTAATGAAGGAGTACACCGGAACTATCGTGTTTAT

CACCCACGATAAACGATTACTCGAAAATGTAGCAGATGTAGTTTATGAAATTAGAGATAAGAAAATAAATCT

GAAACATTAAATTTAAGGTAGTCGCTGGTCAGTATAGTCTGTTCTGGTTGGCGACTCCATTGTTAAAGAGTA

TAAAGACTTTAGATTTTATGAATATTAAAAATAGGAACAGTCAATTGAACTGCTCCTATTTTTCTGCTAAAT

ATATTGTAGTTTTCTTATATGTATAATGATAGATTAGCGGATTCTCATCTACGGTACTTACTTCAAATATGA

AGAAGTGATCGCGGTTATCTCTGGACTTTTCCTTATTGAGGACAAAGTAATTCTTACGTGAAGTCGCCATTG

TTTTTAGGATATCATCAGTTAGGAAGGTCAATGGAATATTCATGTTAGAGTAGCGGTAGAAGTCACGTTCAA

AATCTTGGTAGCTCTCGCTATAATAGTCCATTTGTAGGTGATTACGCTGAAACTCAAGCTGATTCATAGAGC

ACCTCCTCGACAAGTTCAATACTAATAATGTCTTTTAATTTCAAATTGATGTGACCTGTTGTAGTTTTTATC

AAAATGAAATCTTTGGTCAGACTTGGTATTGTTCCAGTGTAGGAAACACGCTTGTTTTTTTCAATCACTTGA

ATGCGTGTGCGTAGCTGCCCGGCGTATACTTGACTGAGGAGTAATAATTTCTTCTCTAGTGATAAGTCAGAC

ATGTACGTTACTTTGTTTGTATCATCAGAGAGTGCTGATGCATGTTCAGATAGGAAAAAGCCCATCCATTTT

TGCATCTTTGTATCCTGGTACTCTCTTGCTGATTGAAATGGTAAATATGAACGGTCAATCATATCAAATCCT

TTCTATGCAGAGGCAAGGGTATTTTTATCAAATTGAATCGTAAAACCTTGAATTCCCCCACCTGTGTAACAT

TCTTTAAAGCGATTGATTACCTCAGTATAGATTATCACAGATGAGCTTGTTGGCTTAATGCTAAATGTAAAT

TCCAATGGTAATCGGTTTTCAGATTTAGCATGTACTAGTCGTATCGATATTTCAGTTGTTTTGAGTTTTCTC

TGACGAAGTTTTGAAGTTGCTGTTTCAACGATTCCATGTAAAAATCTTTCAAGCATTTCAATATCATTACAT

CCTTTGCTACGGATTTCTGAAAATTGTACTGTATTTTTTTCTTGTTTCATTTTAATCCCTCCAATCCACCCG

CGGAATGACCACCGATAAGTTTACTGCGTTCAATATTTCTGGAACCTTCAGTTAGGACGGTTCCTTTTTGTA

TGGCTAAAAAACCAAACTGTTCTCTGACAACATCAATAGCTGTCTGAAGTCTATTATCTTTTTCAATTTGTT

CTACATCATCAAAGAGTGATAGTAGAGTATAGCTTTCATCTACGAAGCCACTATAAGATACACCAATTTGTC

TCACTGCACCAGAGGTGTATTTTTTCGGAATAATACAAGTACATGACTCACCATTGTTTTGGGGAGATTTG

CGGGTTCAATTTTATTCTGAGCATTTATAGATTTTTTCATCTCAGTCCTAGAATAGCCAATATGAATAGAAA

CGACAGTAGTCAATACTAGGGCTACTGTTCCGTTCCACAGTATCATTTAAAAATCATT-TTCACACCCTTTC

GTCTATTAGTATAGAAGAAAGCTCTCAGCACA

>GA47461/Mega-2.II

CATGTTGAGGCGGTAAGTTTGCTAGTCAAGGAGTAAAACGACGAAGATTAGCATTTACTTCCGCCCATGCGA

TAGCTGTCCGTGATTGACAAGTGCTAGCACGCAGACAGAACGGAGATAGCGAACCGCTGAGTGTGTCGCTCT

GCTCGTAAAAGCTTAGAAACCTTTGAACGAAAGGGATAATGAAAGCCTTGATTGCAAGGCTTTTTGCTTTAT

GGTGGGTAAGTATCAGAGTGAGAAAATTTTTGGAATGAGTAGAAGTGATAGCTAGAAATTATCAGTTTCTA

TTTCCATTTACCCTGTGGGTACGTGTTTGTTTCCATTGACAAGGAGTTTGTGGGAATAGAAATGT

ACCCACCTTGTTTGAATCAAGTGAAGTGTAGTTGAAGGAAATCTGTTGAAAGCAATACTTCATTTTACCG

AATAAGTAATAATTTAGGCAACTTCAAATCGATTAAAAAAAACTATTTTAAAGGTTAAGAGTAGACAAAA

ATTGTCCACTCTTTTTTGCAAACTCAATTTATCAATAAATGAAATGAGGGAATGTAAAATG

AAATATTTTGAGGTTGAGTTAGAAAATCCTGATGAATTTTTAAAACTACAAACAGAAGATTTTGTGAAAG

CTAATCGCTTGCTACTAAGGAAGATAATCCAGAGCGTTACAGTCTATGAAGAAAACTTCGTCATATCCTT

TAAATCTGGCATCGAATTGGAAGTATGAGTCTCATTCCATAACTTTTATATTGAACATATCATCTTGTTGTG

TTATACTATAAATTGATATAAACAAAGATGTAGGAGGAACCGAAACTATGACAGCCTCAATGCGTTTAAGAT

AAGCTGGCAATAAAAAAAGCAGAATCTATACCCGATGATAGGCTTTTTTGTTGTGCTTATTTATACGATATT

GAGCATTCATTAGTTACGGTGAGGATATTGGTTATTTAACTATACCTTTATTTAACTATGTCTTTAATATGA

ATGTTTCCAAATTGTATGTATGCAGACCAAAAGCCACATTGTGGGGTTTGGCCTGCATTTTTTTTGCCTA

GAATGCTATTCAAAATAGAAATTCAAGCAAAATAATATGCAGGAGATAATATAAATGGAAAAATACAACAAT

TGGAAACGAAAATTTTATGCAATATGGGCAGGGCAAGCAGTATCATTAATCACTAGTGCCATCCTGCAAATG

GCGATTATTTTTTACCTTACAGAAAAAACAGGATCTGCGATGGTCTTGTCTATGGCTTCATTAGTAGGTTTT

TTACCCTATGCGATTTTGGGACCTGCCATTGGTGTGCTAGTGGATCGTCATGATAGGAAGAAGATAATGATT

GGTGCCGATTTAATTATCGCAGCAGCTGGTGCAGTGCTTGCTATTGTTGCATTCTGTATGGAGCTACCTGTC

TGGATGATTATGATAGTATTGTTTATCCGTAGCATTGGAACAGCTTTTCATACCCCAGCACTCAATGCGGTT

ACACCACTTTTAGTACCAGAAGAACAGCTAACGAAATGCGCAGGCTATAGTCAGTCTTTGCAGTCTATAAGC

TATATTGTTAGTCCGGCAGTTGCAGCACTCTTATACTCCGTTTGGGATTTAAATGCTATTATTGCCATCGAC

GTATTGGGTGCTGTGATTGCATCTATTACGGTAGCAATTGTACGTATACCTAAGCTGGGTAATCAAGTGCAA

AGTTTAGAACCAAATTTCATAAGGGAGATGAAAGAAGGAGTTGTGGTTCTGAGACAAAACAAAGGATTGTTT

GCCTTATTACTCTTAGGAACACTATATACTTTTGTTTATATGCCAATCAATGCACTATTTCCTTTAATAAGC

ATGGAACACTTTAATGGAACGCCTGTGCATATTTCTATTACGGAAATTTCCTTTGCATTTGGGATGCTAGCA

GGAGGCTTATTATTAGGAAGATTAGGGGGCTTCGAAAAGCATGTATTACTAATAACAAGTTCATTTTTTATA

ATGGGGACCAGTTTAGCCGTTTCGGGAATACTTCCTCCAAATGGATTTGTAATATTCGTAGTTTGCTGTGCA

ATAATGGGGCTTTCGGTGCCATTTTATAGCGGTGTGCAAACAGCTCTTTTTCAGGAGAAAATTAAGCCTGAA

TATTTAGGACGTGTATTTTCTTTGATCGGAAGTATCATGTCACTTGCTATGCCAATTGGGTTAATTCTTTCT

GGATTCTTTGCTGATAAAATCGGTGTAAATCATTGGTTTTTACTATCAGGTATTTTAATTATTGGCATTGCT

ATAGTTTGCCAAATGATAACTGAGGTTAGAAAATTAGATTTAAAATAAACAATATTGGAGGAA

ATGTTATGGAATTAATATT

AAAAGCAAAAGACATTCGTGTGGAATTCAAAGGACGCGATGTTTTAGATATAAATGAATTAGAAGTATATGA

TTATGACCGTATTGGTTTAGTAGGAGCAAATGGTGCTGGAAAAAGCACTTTACTCAGGGTACTTTTAGGAGA

ATTAACTCCCCCAGGATGTAAAATGAATCGTCTGGGTGAACTTGCCTATATTCCCCAGTTGGACGAAGTAAC

TCTGCAGGAGGAAAAAGATTTTGCACTTGTAGGCAAGCTAGGTGTTGAGCAATTAAATATACAGACTATGAG

CGGTGGTGAAGAAACAAGGCTTAAAATAGCACAGGCCTTATCGGCACAGGTTCATGGTATTTTAGCGGATGA

ACCTACGAGCCATTTAGACCGTGAAGGAATTGATTTTCTAATAGGACAGCTAAAATATTTTACAGGTGCACT

GTTAGTTATTAGCCATGACCGCTATTTTCTTGATGAAATAGTAGATAAAATATGGGAACTGAAAGATGGCAA

AATCACTGAGTATTGGGGAAACTATTCTGATTATCTTCGTCAGAAAGAGGAAGAACGTAAGAGCCAAGCTGC

AGAATACGAACAATTTATTGCGGAACGTGCCCGATTGGAAAGGGCTGCGGAGGAAAAGCGAAAACAGGCTCG

TAAAATAGAACAGAAGGCAAAAGGTTCTTCAAAGAAAAAAAGTACTGAAGACGGAGGGCGTTTAGCTCATCA

AAAATCAATAGGAAGTAAGGAAAAAAAGATGTATAATGCTGCTAAAACCCTAGAGCACAGGATTGCGGCCTT

AGGAAAAGTAGAAGCTCCGGAAGGCATTCGCAGAATTCGTTTCAGGCAAAGTAAAGCATTGGAGCTCCATAA

TCCATACCCTATAGTCGGTGCAGAAATTAATAAAGTATTTGGGGATAAGGCTCTGTTTGAAAATGCATCTTT

TCAAATTCCGTTAGGAGCAAAAGTGGCGTTAACTGGTGGTAATGGAATCGGAAAAACAACTTTAATCCAAAT

GATCTTAAACCATGAAGAAGGAATTTCTATTTCGCCTAAGGCAAAAATAGGTTACTTTGCACAGAATGGTTA

CAAGTACAACAGTAATCAGAATGTTATGGAGTTTATGCAGAAGGATTGTGACTACAATATATCAGAAATTCG

TTCAGTGCTAGCATCTATGGGGTTCAAACAGAACGATATTGGAAAAAGTTTATCTGTTTTAAGCGGTGGAGA

AATTATAAAATTGTTGCTTGCTAAAATGCTCATGGGTAGATATAACATCCTAATAATGGATGAACCCAGTAA

CTTCCTTGACATACCAAGTTTAGAGGCTTTGGAAATACTAATGAAGGAGTACACCGGAACTATCGTGTTTAT

CACCCACGATAAACGATTACTCGAAAATGTAGCAGATGTAGTTTATGAAATTAGAGATAAGAAAATAAATCT

GAAACATTAAATTTAAGGTAGTCGCTGGTCAGTATAGTCTGTTCTGGTTGGCGACTCCATTGTTAAAGAGTA

TAAAGACTTTAGATTTTATGAATATTAAAAATAGGAACAGTCAATTGAACTGCTCCTATTTTTCTGCTAAAT

ATATTGTAGTTTTCTTATATGTATAATGATAGATTAGCGGATTCTCATCTACGGTACTTACTTCAAATATGA

AGAAGTGATCGCGGTTATCTCTGGACTTTTCCTTATTGAGGACAAAGTAATTCTTACGTGAAGTCGCCATTG

TTTTTAGGATATCATCAGTTAGGAAGGTCAATGGAATATTCATGTTAGAGTAGCGGTAGAAGTCACGTTCAA

AATCTTGGTAGCTCTCGCTATAATAGTCCATTTGTAGGTGATTACGCTGAAACTCAAGCTGATTCATAGAGC

ACCTCCTCGACAAGTTCAATACTAATAATGTCTTTTAATTTCAAATTGATGTGACCTGTTGTAGTTTTTATC

AAAATGAAATCTTTGGTCAGACTTGGTATTGTTCCAGTGTAGGAAACACGCTTGTTTTTTTCAATCACTTGA

ATGCGTGTGCGTAGCTGCCCGGCGTATACTTGACTGAGGAGTAATAATTTCTTCTCTAGTGATAAGTCAGAC

ATGTACGTTACTTTGTTTGTATCATCAGAGAGTGCTGATGCATGTTCAGATAGGAAAAAGCCCATCCATTTT

TGCATCTTTGTATCCTGGTACTCTCTTGCTGATTGAAATGGTAAATATGAACGGTCAATCATATCAAATCCT

TTCTATGCAGAGGCAAGGGTATTTTTATCAAATTGAATCGTAAAACCTTGAATTCCCCCACCTGTGTAACAT

TCTTTAAAGCGATTGATTACCTCAGTATAGATTATCACAGATGAGCTTGTTGGCTTAATGCTAAATGTAAAT

TCCAATGGTAATCGGTTTTCAGATTTAGCATGTACTAGTCGTATCGATATTTCAGTTGTTTTGAGTTTTCTC

TGACGAAGTTTTGAAGTTGCTGTTTCAACGATTCCATGTAAAAATCTTTCAAGCATTTCAATATCATTACAT

CCTTTGCTACGGATTTCTGAAAATTGTACTGTATTTTTTTCTTGTTTCATTTTAATCCCTCCAATCCACCCG

CGGAATGACCACCGATAAGTTTACTGCGTTCAATATTTCTGGAACCTTCAGTTAGGACGGTTCCTTTTTGTA

TGGCTAAAAAACCAAACTGTTCTCTGACAACATCAATAGCTGTCTGAAGTCTATTATCTTTTTCAATTTGTT

CTACATCATCAAAGAGTGATAGTAGAGTATAGCTTTCATCTACGAAGCCACTATAAGATACACCAATTTGTC

TCACTGCACCAGAGGTGTATTTTTTCGGAATAATACAAGTACATGACTCACCATTGTTTTGGGGAGATTTG

CGGGTTCAATTTTATTCTGAGCATTTATAGATTTTTTCATCTCAGTCCTAGAATAGCCAATATGAATAGAAA

CGACAGTAGTCAATACTAGGGCTACTGTTCCGTTCCACAGTATCATTTAAAAATCATT-TTCACACCCTTTC

GTCTATTAGTATAGAAGAAAGCTCTCAGCACA

>GA44452/Mega-2.II

CATGTTGAGGCGGTAAGTTTGCTAGTCAAGGAGTAAAACGACGAAGATTAGCATTTACTTCCGCCCATGCGA

TAGCTGTCCGTGATTGACAAGTGCTAGCACGCAGACAGAACGGAGATAGCGAACCGCTGAGTGTGTCGCTCT

GCTCGTAAAAGCTTAGAAACCTTTGAACGAAAGGGATAATGAAAGCCTTGATTGCAAGGCTTTTTGCTTTAT

GGTGGGTAAGTATCAGAGTGAGAAAATTTTTGGAATGAGTAGAAGTGATAGCTAGAAATTATCAGTTTCTA

TTTCCATTTACCCTGTGGGTACGTGTTTGTTTCCATTGACAAGGAGTTTGTGGGAATAGAAATGT

ACCCACCTTGTTTGAATCAAGTGAAGTGTAGTTGAAGGAAATCTGTTGAAAGCAATACTTCATTTTACCG

AATAAGTAATAATTTAGGCAACTTCAAATCGATTAAAAAAAACTATTTTAAAGGTTAAGAGTAGACAAAA

ATTGTCCACTCTTTTTTGCAAACTCAATTTATCAATAAATGAAATGAGGGAATGTAAAATG

AAATATTTTGAGGTTGAGTTAGAAAATCCTGATGAATTTTTAAAACTACAAACAGAAGATTTTGTGAAAG

CTAATCGCTTGCTACTAAGGAAGATAATCCAGAGCGTTACAGTCTATGAAGAAAACTTCGTCATATCCTT

TAAATCTGGCATCGAATTGGAAGTATGAGTCTCATTCCATAACTTTTATATTGAACATATCATCTTGTTGTG

TTATACTATAAATTGATATAAACAAAGATGTAGGAGGAACCGAAACTATGACAGCCTCAATGCGTTTAAGAT

AAGCTGGCAATAAAAAAAGCAGAATCTATACCCGATGATAGGCTTTTTTGTTGTGCTTATTTATACGATATT

GAGCATTCATTAGTTACGGTGAGGATATTGGTTATTTAACTATACCTTTATTTAACTATGTCTTTAATATGA

ATGTTTCCAAATTGTATGTATGCAGACCAAAAGCCACATTGTGGGGTTTGGCCTGCATTTTTTTTGCCTA

GAATGCTATTCAAAATAGAAATTCAAGCAAAATAATATGCAGGAGATAATATAAATGGAAAAATACAACAAT

TGGAAACGAAAATTTTATGCAATATGGGCAGGGCAAGCAGTATCATTAATCACTAGTGCCATCCTGCAAATG

GCGATTATTTTTTACCTTACAGAAAAAACAGGATCTGCGATGGTCTTGTCTATGGCTTCATTAGTAGGTTTT

TTACCCTATGCGATTTTGGGACCTGCCATTGGTGTGCTAGTGGATCGTCATGATAGGAAGAAGATAATGATT

GGTGCCGATTTAATTATCGCAGCAGCTGGTGCAGTGCTTGCTATTGTTGCATTCTGTATGGAGCTACCTGTC

TGGATGATTATGATAGTATTGTTTATCCGTAGCATTGGAACAGCTTTTCATACCCCAGCACTCAATGCGGTT

ACACCACTTTTAGTACCAGAAGAACAGCTAACGAAATGCGCAGGCTATAGTCAGTCTTTGCAGTCTATAAGC

TATATTGTTAGTCCGGCAGTTGCAGCACTCTTATACTCCGTTTGGGATTTAAATGCTATTATTGCCATCGAC

GTATTGGGTGCTGTGATTGCATCTATTACGGTAGCAATTGTACGTATACCTAAGCTGGGTAATCAAGTGCAA

AGTTTAGAACCAAATTTCATAAGGGAGATGAAAGAAGGAGTTGTGGTTCTGAGACAAAACAAAGGATTGTTT

GCCTTATTACTCTTAGGAACACTATATACTTTTGTTTATATGCCAATCAATGCACTATTTCCTTTAATAAGC

ATGGAACACTTTAATGGAACGCCTGTGCATATTTCTATTACGGAAATTTCCTTTGCATTTGGGATGCTAGCA

GGAGGCTTATTATTAGGAAGATTAGGGGGCTTCGAAAAGCATGTATTACTAATAACAAGTTCATTTTTTATA

ATGGGGACCAGTTTAGCCGTTTCGGGAATACTTCCTCCAAATGGATTTGTAATATTCGTAGTTTGCTGTGCA

ATAATGGGGCTTTCGGTGCCATTTTATAGCGGTGTGCAAACAGCTCTTTTTCAGGAGAAAATTAAGCCTGAA

TATTTAGGACGTGTATTTTCTTTGATCGGAAGTATCATGTCACTTGCTATGCCAATTGGGTTAATTCTTTCT

GGATTCTTTGCTGATAAAATCGGTGTAAATCATTGGTTTTTACTATCAGGTATTTTAATTATTGGCATTGCT

ATAGTTTGCCAAATGATAACTGAGGTTAGAAAATTAGATTTAAAATAAACAATATTGGAGGAA

ATGTTATGGAATTAATATT

AAAAGCAAAAGACATTCGTGTGGAATTCAAAGGACGCGATGTTTTAGATATAAATGAATTAGAAGTATATGA

TTATGACCGTATTGGTTTAGTAGGAGCAAATGGTGCTGGAAAAAGCACTTTACTCAGGGTACTTTTAGGAGA

ATTAACTCCCCCAGGATGTAAAATGAATCGTCTGGGTGAACTTGCCTATATTCCCCAGTTGGACGAAGTAAC

TCTGCAGGAGGAAAAAGATTTTGCACTTGTAGGCAAGCTAGGTGTTGAGCAATTAAATATACAGACTATGAG

CGGTGGTGAAGAAACAAGGCTTAAAATAGCACAGGCCTTATCGGCACAGGTTCATGGTATTTTAGCGGATGA

ACCTACGAGCCATTTAGACCGTGAAGGAATTGATTTTCTAATAGGACAGCTAAAATATTTTACAGGTGCACT

GTTAGTTATTAGCCATGACCGCTATTTTCTTGATGAAATAGTAGATAAAATATGGGAACTGAAAGATGGCAA

AATCACTGAGTATTGGGGAAACTATTCTGATTATCTTCGTCAGAAAGAGGAAGAACGTAAGAGCCAAGCTGC

AGAATACGAACAATTTATTGCGGAACGTGCCCGATTGGAAAGGGCTGCGGAGGAAAAGCGAAAACAGGCTCG

TAAAATAGAACAGAAGGCAAAAGGTTCTTCAAAGAAAAAAAGTACTGAAGACGGAGGGCGTTTAGCTCATCA

AAAATCAATAGGAAGTAAGGAAAAAAAGATGTATAATGCTGCTAAAACCCTAGAGCACAGGATTGCGGCCTT

AGGAAAAGTAGAAGCTCCGGAAGGCATTCGCAGAATTCGTTTCAGGCAAAGTAAAGCATTGGAGCTCCATAA

TCCATACCCTATAGTCGGTGCAGAAATTAATAAAGTATTTGGGGATAAGGCTCTGTTTGAAAATGCATCTTT

TCAAATTCCGTTAGGAGCAAAAGTGGCGTTAACTGGTGGTAATGGAATCGGAAAAACAACTTTAATCCAAAT

GATCTTAAACCATGAAGAAGGAATTTCTATTTCGCCTAAGGCAAAAATAGGTTACTTTGCACAGAATGGTTA

CAAGTACAACAGTAATCAGAATGTTATGGAGTTTATGCAGAAGGATTGTGACTACAATATATCAGAAATTCG

TTCAGTGCTAGCATCTATGGGGTTCAAACAGAACGATATTGGAAAAAGTTTATCTGTTTTAAGCGGTGGAGA

AATTATAAAATTGTTGCTTGCTAAAATGCTCATGGGTAGATATAACATCCTAATAATGGATGAACCCAGTAA

CTTCCTTGACATACCAAGTTTAGAGGCTTTGGAAATACTAATGAAGGAGTACACCGGAACTATCGTGTTTAT

CACCCACGATAAACGATTACTCGAAAATGTAGCAGATGTAGTTTATGAAATTAGAGATAAGAAAATAAATCT

GAAACATTAAATTTAAGGTAGTCGCTGGTCAGTATAGTCTGTTCTGGTTGGCGACTCCATTGTTAAAGAGTA

TAAAGACTTTAGATTTTATGAATATTAAAAATAGGAACAGTCAATTGAACTGCTCCTATTTTTCTGCTAAAT

ATATTGTAGTTTTCTTATATGTATAATGATAGATTAGCGGATTCTCATCTACGGTACTTACTTCAAATATGA

AGAAGTGATCGCGGTTATCTCTGGACTTTTCCTTATTGAGGACAAAGTAATTCTTACGTGAAGTCGCCATTG

TTTTTAGGATATCATCAGTTAGGAAGGTCAATGGAATATTCATGTTAGAGTAGCGGTAGAAGTCACGTTCAA

AATCTTGGTAGCTCTCGCTATAATAGTCCATTTGTAGGTGATTACGCTGAAACTCAAGCTGATTCATAGAGC

ACCTCCTCGACAAGTTCAATACTAATAATGTCTTTTAATTTCAAATTGATGTGACCTGTTGTAGTTTTTATC

AAAATGAAATCTTTGGTCAGACTTGGTATTGTTCCAGTGTAGGAAACACGCTTGTTTTTTTCAATCACTTGA

ATGCGTGTGCGTAGCTGCCCGGCGTATACTTGACTGAGGAGTAATAATTTCTTCTCTAGTGATAAGTCAGAC

ATGTACGTTACTTTGTTTGTATCATCAGAGAGTGCTGATGCATGTTCAGATAGGAAAAAGCCCATCCATTTT

TGCATCTTTGTATCCTGGTACTCTCTTGCTGATTGAAATGGTAAATATGAACGGTCAATCATATCAAATCCT

TTCTATGCAGAGGCAAGGGTATTTTTATCAAATTGAATCGTAAAACCTTGAATTCCCCCACCTGTGTAACAT

TCTTTAAAGCGATTGATTACCTCAGTATAGATTATCACAGATGAGCTTGTTGGCTTAATGCTAAATGTAAAT

TCCAATGGTAATCGGTTTTCAGATTTAGCATGTACTAGTCGTATCGATATTTCAGTTGTTTTGAGTTTTCTC

TGACGAAGTTTTGAAGTTGCTGTTTCAACGATTCCATGTAAAAATCTTTCAAGCATTTCAATATCATTACAT

CCTTTGCTACGGATTTCTGAAAATTGTACTGTATTTTTTTCTTGTTTCATTTTAATCCCTCCAATCCACCCG

CGGAATGACCACCGATAAGTTTACTGCGTTCAATATTTCTGGAACCTTCAGTTAGGACGGTTCCTTTTTGTA

TGGCTAAAAAACCAAACTGTTCTCTGACAACATCAATAGCTGTCTGAAGTCTATTATCTTTTTCAATTTGTT

CTACATCATCAAAGAGTGATAGTAGAGTATAGCTTTCATCTACGAAGCCACTATAAGATACACCAATTTGTC

TCACTGCACCAGAGGTGTATTTTTTCGGAATAATACAAGTACATGACTCACCATTGTTTTGGGGAGATTTG

CGGGTTCAATTTTATTCTGAGCATTTATAGATTTTTTCATCTCAGTCCTAGAATAGCCAATATGAATAGAAA

CGACAGTAGTCAATACTAGGGCTACTGTTCCGTTCCACAGTATCATTTAAAAATCATT-TTCACACCCTTTC

GTCTATTAGTATAGAAGAAAGCTCTCAGCACA

>GA44386/Mega-I.III

CATGTTGAGGCGGTAAGTTTGCTAGTCAAGGAGTAAAACGACGAAGATTAGCATTTACTTCCGCCCATGCGA

TAGCTGTCCGTGATTGACAAGTGCTAGCACGCAGACAGAACGGAGATAGCGAACCGCTGAGTGTGTCGCTCT

GCTCGTAAAAGCTTAGAAACCTTTGAACGAAAGGGATAATGAAAGCCTTGATTGCAAGGCTTTTTGCTTTAT

GGTGGGTAAGTATCAGAGTGAGAAAATTTTTGGAATGAGTAGAAGTGATAGCTAGAAATTATCAGTTTCTA

TTTCCATTTACCCTGTGGGTACGTGTTTGTTTCCATTGACAAGGAGTTTGTGGGAATAGAAATGT

ACCCACCTTGTTTGAATCAAGTGAAGTGTAGTTGAAGGAAATCTGTTGAAAGCAATACTTCATTTTACCG

AATAAGTAATAATTTAGGCAACTTCAAATCGATTAAAAAAAACTATTTTAAAGGTTAAGAGTAGACAAAA

ATTGTCCACTCTTTTTTGCAAACTCAATTTATCAATAAATGAAATGAGGGAATGTAAAATG

AAATATTTTGAGGTTGAGTTAGAAAATCCTGATGAATTTTTAAAACTACAAACAGAAGATTTTGTGAAAG

CTAATCGCTTGCTACTAAGGAAGATAATCCAGAGCGTTACAGTCTATGAAGAAAACTTCGTCATATCCTT

TAAATCTGGCATCGAATTGGAAGTATGAGTCTCATTCCATAACTTTTATATTGAACATATCATCTTGTTGTG

TTATACTATAAATTGATATAAACAAAGATGTAGGAGGAACCGAAACTATGACAGCCTCAATGCGTTTAAGAT

AAGCTGGCAATAAAAAAAGCAGAATCTATACCCGATGATAGGCTTTTTTGTTGTGCTTATTTATACGATATT

GAGCATTCATTAGTTACGGTGAGGATATTGGTTATTTAACTATACCTTTATTTAACTATGTCTTTAATATGA

ATGTTTCCAAATTGTATGTATGCAGACCAAAAGCCACATTGTGGGGTTTGGCCTGCATTTTTTTTGCCTA

GAATGCTATTCAAAATAGAAATTCAAGCAAAATAATATGCAGGAGATAATATAAATGGAAAAATACAACAAT

TGGAAACGAAAATTTTATGCAATATGGGCAGGGCAAGCAGTATCATTAATCACTAGTGCCATCCTGCAAATG

GCGATTATTTTTTACCTTACAGAAAAAACAGGATCTGCGATGGTCTTGTCTATGGCTTCATTAGTAGGTTTT

TTACCCTATGCGATTTTGGGACCTGCCATTGGTGTGCTAGTGGATCGTCATGATAGGAAGAAGATAATGATT

GGTGCCGATTTAATTATCGCAGCAGCTGGTGCAGTGCTTGCTATTGTTGCATTCTGTATGGAGCTACCTGTC

TGGATGATTATGATAGTATTGTTTATCCGTAGCATTGGAACAGCTTTTCATACCCCAGCACTCAATGCGGTT

ACACCACTTTTAGTACCAGAAGAACAGCTAACGAAATGCGCAGGCTATAGTCAGTCTTTGCAGTCTATAAGC

TATATTGTTAGTCCGGCAGTTGCAGCACTCTTATACTCCGTTTGGGATTTAAATGCTATTATTGCCATCGAC

GTATTGGGTGCTGTGATTGCATCTATTACGGTAGCAATTGTACGTATACCTAAGCTGGGTAATCAAGTGCAA

AGTTTAGAACCAAATTTCATAAGGGAGATGAAAGAAGGAGTTGTGGTTCTGAGACAAAACAAAGGATTGTTT

GCCTTATTACTCTTAGGAACACTATATACTTTTGTTTATATGCCAATCAATGCACTATTTCCTTTAATAAGC

ATGGAACACTTTAATGGAACGCCTGTGCATATTTCTATTACGGAAATTTCCTTTGCATTTGGGATGCTAGCA

GGAGGCTTATTATTAGGAAGATTAGGGGGCTTCGAAAAGCATGTATTACTAATAACAAGTTCATTTTTTATA

ATGGGGACCAGTTTAGCCGTTTCGGGAATACTTCCTCCAAATGGATTTGTAATATTCGTAGTTTGCTGTGCA

ATAATGGGGCTTTCGGTGCCATTTTATAGCGGTGTGCAAACAGCTCTTTTTCAGGAGAAAATTAAGCCTGAA

TATTTAGGACGTGTATTTTCTTTGATCGGAAGTATCATGTCACTTGCTATGCCAATTGGGTTAATTCTTTCT

GGATTCTTTGCTGATAAAATCGGTGTAAATCATTGGTTTTTACTATCAGGTATTTTAATTATTGGCATTGCT

ATAGTTTGCCAAATGATAACTGAGGTTAGAAAATTAGATTTAAAATAAACAATATTGGAGGAA

ATGTTATGGAATTAATATT

AAAAGCAAAAGACATTCGTGTGGAATTCAAAGGACGCGATGTTTTAGATATAAATGAATTAGAAGTATATGA

TTATGACCGTATTGGTTTAGTAGGAGCAAATGGTGCTGGAAAAAGCACTTTACTCAGGGTACTTTTAGGAGA

ATTAACTCCCCCAGGATGTAAAATGAATCGTCTGGGTGAACTTGCCTATATTCCCCAGTTGGACGAAGTAAC

TCTGCAGGAGGAAAAAGATTTTGCACTTGTAGGCAAGCTAGGTGTTGAGCAATTAAATATACAGACTATGAG

CGGTGGTGAAGAAACAAGGCTTAAAATAGCACAGGCCTTATCGGCACAGGTTCATGGTATTTTAGCGGATGA

ACCTACGAGCCATTTAGACCGTGAAGGAATTGATTTTCTAATAGGACAGCTAAAATATTTTACAGGTGCACT

GTTAGTTATTAGCCATGACCGCTATTTTCTTGATGAAATAGTAGATAAAATATGGGAACTGAAAGATGGCAA

AATCACTGAGTATTGGGGAAACTATTCTGATTATCTTCGTCAGAAAGAGGAAGAACGTAAGAGCCAAGCTGC

AGAATACGAACAATTTATTGCGGAACGTGCCCGATTGGAAAGGGCTGCGGAGGAAAAGCGAAAACAGGCTCG

TAAAATAGAACAGAAGGCAAAAGGTTCTTCAAAGAAAAAAAGTACTGAAGACGGAGGGCGTTTAGCTCATCA

AAAATCAATAGGAAGTAAGGAAAAAAAGATGTATAATGCTGCTAAAACCCTAGAGCACAGGATTGCGGCCTT

AGGAAAAGTAGAAGCTCCGGAAGGCATTCGCAGAATTCGTTTCAGGCAAAGTAAAGCATTGGAGCTCCATAA

TCCATACCCTATAGTCGGTGCAGAAATTAATAAAGTATTTGGGGATAAGGCTCTGTTTGAAAATGCATCTTT

TCAAATTCCGTTAGGAGCAAAAGTGGCGTTAACTGGTGGTAATGGAATCGGAAAAACAACTTTAATCCAAAT

GATCTTAAACCATGAAGAAGGAATTTCTATTTCGCCTAAGGCAAAAATAGGTTACTTTGCACAGAATGGTTA

CAAGTACAACAGTAATCAGAATGTTATGGAGTTTATGCAGAAGGATTGTGACTACAATATATCAGAAATTCG

TTCAGTGCTAGCATCTATGGGGTTCAAACAGAACGATATTGGAAAAAGTTTATCTGTTTTAAGCGGTGGAGA

AATTATAAAATTGTTGCTTGCTAAAATGCTCATGGGTAGATATAACATCCTAATAATGGATGAACCCAGTAA

CTTCCTTGACATACCAAGTTTAGAGGCTTTGGAAATACTAATGAAGGAGTACACCGGAACTATCGTGTTTAT

CACCCACGATAAACGATTACTCGAAAATGTAGCAGATGTAGTTTATGAAATTAGAGATAAGAAAATAAATCT

GAAACATTAAATTTAAGGTAGTCGCTGGTCAGTATAGTCTGTTCTGGTTGGCGACTCCATTGTTAAAGAGTA

TAAAGACTTTAGATTTTATGAATATTAAAAATAGGAACAGTCAATTGAACTGCTCCTATTTTTCTGCTAAAT

ATATTGTAGTTTTCTTATATGTATAATGATAGATTAGCGGATTCTCATCTACGGTACTTACTTCAAATATGA

AGAAGTGATCGCGGTTATCTCTGGACTTTTCCTTATTGAGGACAAAGTAATTCTTACGTGAAGTCGCCATTG

TTTTTAGGATATCATCAGTTAGGAAGGTCAATGGAATATTCATGTTAGAGTAGCGGTAGAAGTCACGTTCAA

AATCTTGGTAGCTCTCGCTATAATAGTCCATTTGTAGGTGATTACGCTGAAACTCAAGCTGATTCATAGAGC

ACCTCCTCGACAAGTTCAATACTAATAATGTCTTTTAATTTCAAATTGATGTGACCTGTTGTAGTTTTTATC

AAAATGAAATCTTTGGTCAGACTTGGTATTGTTCCAGTGTAGGAAACACGCTTGTTTTTTTCAATCACTTGA

ATGCGTGTGCGTAGCTGCCCGGCGTATACTTGACTGAGGAGTAATAATTTCTTCTCTAGTGATAAGTCAGAC

ATGTACGTTACTTTGTTTGTATCATCAGAGAGTGCTGATGCATGTTCAGATAGGAAAAAGCCCATCCATTTT

TGCATCTTTGTATCCTGGTACTCTCTTGCTGATTGAAATGGTAAATATGAACGGTCAATCATATCAAATCCT

TTCTATGCAGAGGCAAGGGTATTTTTATCAAATTGAATCGTAAAACCTTGAATTCCCCCACCTGTGTAACAT

TCTTTAAAGCGATTGATTACCTCAGTATAGATTATCACAGATGAGCTTGTTGGCTTAATGCTAAATGTAAAT

TCCAATGGTAATCGGTTTTCAGATTTAGCATGTACTAGTCGTATCGATATTTCAGTTGTTTTGAGTTTTCTC

TGACGAAGTTTTGAAGTTGCTGTTTCAACGATTCCATGTAAAAATCTTTCAAGCATTTCAATATCATTACAT

CCTTTGCTACGGATTTCTGAAAATTGTACTGTATTTTTTTCTTGTTTCATTTTAATCCCTCCAATCCACCCG

CGGAATGACCACCGATAAGTTTACTGCGTTCAATATTTCTGGAACCTTCAGTTAGGACGGTTCCTTTTTGTA

TGGCTAAAAAACCAAACTGTTCTCTGACAACATCAATAGCTGTCTGAAGTCTATTATCTTTTTCAATTTGTT

CTACATCATCAAAGAGTGATAGTAGAGTATAGCTTTCATCTACGAAGCCACTATAAGATACACCAATTTGTC

TCACTGCACCAGAGGTGTATTTTTTCGGAATAATACAAGTACATGACTCACCATTGTTTTGGGGAGATTTG

CGGGTTCAATTTTATTCTGAGCATTTATAGATTTTTTCATCTCAGTCCTAGAATAGCCAATATGAATAGAAA

CGACAGTAGTCAATACTAGGGCTACTGTTCCGTTCCACAGTATCATTTAAAAATCATT-TTCACACCCTTTC

GTCTATTAGTATAGAAGAAAGCTCTCAGCACA

>GA43257/Mega-2.II

CATGTTGAGGCGGTAAGTTTGCTAGTCAAGGAGTAAAACGACGAAGATTAGCATTTACTTCCGCCCATGCGA

TAGCTGTCCGTGATTGACAAGTGCTAGCACGCAGACAGAACGGAGATAGCGAACCGCTGAGTGTGTCGCTCT

GCTCGTAAAAGCTTAGAAACCTTTGAACGAAAGGGATAATGAAAGCCTTGATTGCAAGGCTTTTTGCTTTAT

GGTGGGTAAGTATCAGAGTGAGAAAATTTTTGGAATGAGTAGAAGTGATAGCTAGAAATTATCAGTTTCTA

TTTCCATTTACCCTGTGGGTACGTGTTTGTTTCCATTGACAAGGAGTTTGTGGGAATAGAAATGT

ACCCACCTTGTTTGAATCAAGTGAAGTGTAGTTGAAGGAAATCTGTTGAAAGCAATACTTCATTTTACCG

AATAAGTAATAATTTAGGCAACTTCAAATCGATTAAAAAAAACTATTTTAAAGGTTAAGAGTAGACAAAA

ATTGTCCACTCTTTTTTGCAAACTCAATTTATCAATAAATGAAATGAGGGAATGTAAAATG

AAATATTTTGAGGTTGAGTTAGAAAATCCTGATGAATTTTTAAAACTACAAACAGAAGATTTTGTGAAAG

CTAATCGCTTGCTACTAAGGAAGATAATCCAGAGCGTTACAGTCTATGAAGAAAACTTCGTCATATCCTT

TAAATCTGGCATCGAATTGGAAGTATGAGTCTCATTCCATAACTTTTATATTGAACATATCATCTTGTTGTG

TTATACTATAAATTGATATAAACAAAGATGTAGGAGGAACCGAAACTATGACAGCCTCAATGCGTTTAAGAT

AAGCTGGCAATAAAAAAAGCAGAATCTATACCCGATGATAGGCTTTTTTGTTGTGCTTATTTATACGATATT

GAGCATTCATTAGTTACGGTGAGGATATTGGTTATTTAACTATACCTTTATTTAACTATGTCTTTAATATGA

ATGTTTCCAAATTGTATGTATGCAGACCAAAAGCCACATTGTGGGGTTTGGCCTGCATTTTTTTTGCCTA

GAATGCTATTCAAAATAGAAATTCAAGCAAAATAATATGCAGGAGATAATATAAATGGAAAAATACAACAAT

TGGAAACGAAAATTTTATGCAATATGGGCAGGGCAAGCAGTATCATTAATCACTAGTGCCATCCTGCAAATG

GCGATTATTTTTTACCTTACAGAAAAAACAGGATCTGCGATGGTCTTGTCTATGGCTTCATTAGTAGGTTTT

TTACCCTATGCGATTTTGGGACCTGCCATTGGTGTGCTAGTGGATCGTCATGATAGGAAGAAGATAATGATT

GGTGCCGATTTAATTATCGCAGCAGCTGGTGCAGTGCTTGCTATTGTTGCATTCTGTATGGAGCTACCTGTC

TGGATGATTATGATAGTATTGTTTATCCGTAGCATTGGAACAGCTTTTCATACCCCAGCACTCAATGCGGTT

ACACCACTTTTAGTACCAGAAGAACAGCTAACGAAATGCGCAGGCTATAGTCAGTCTTTGCAGTCTATAAGC

TATATTGTTAGTCCGGCAGTTGCAGCACTCTTATACTCCGTTTGGGATTTAAATGCTATTATTGCCATCGAC

GTATTGGGTGCTGTGATTGCATCTATTACGGTAGCAATTGTACGTATACCTAAGCTGGGTAATCAAGTGCAA

AGTTTAGAACCAAATTTCATAAGGGAGATGAAAGAAGGAGTTGTGGTTCTGAGACAAAACAAAGGATTGTTT

GCCTTATTACTCTTAGGAACACTATATACTTTTGTTTATATGCCAATCAATGCACTATTTCCTTTAATAAGC

ATGGAACACTTTAATGGAACGCCTGTGCATATTTCTATTACGGAAATTTCCTTTGCATTTGGGATGCTAGCA

GGAGGCTTATTATTAGGAAGATTAGGGGGCTTCGAAAAGCATGTATTACTAATAACAAGTTCATTTTTTATA

ATGGGGACCAGTTTAGCCGTTTCGGGAATACTTCCTCCAAATGGATTTGTAATATTCGTAGTTTGCTGTGCA

ATAATGGGGCTTTCGGTGCCATTTTATAGCGGTGTGCAAACAGCTCTTTTTCAGGAGAAAATTAAGCCTGAA

TATTTAGGACGTGTATTTTCTTTGATCGGAAGTATCATGTCACTTGCTATGCCAATTGGGTTAATTCTTTCT

GGATTCTTTGCTGATAAAATCGGTGTAAATCATTGGTTTTTACTATCAGGTATTTTAATTATTGGCATTGCT

ATAGTTTGCCAAATGATAACTGAGGTTAGAAAATTAGATTTAAAATAAACAATATTGGAGGAA

ATGTTATGGAATTAATATT

AAAAGCAAAAGACATTCGTGTGGAATTCAAAGGACGCGATGTTTTAGATATAAATGAATTAGAAGTATATGA

TTATGACCGTATTGGTTTAGTAGGAGCAAATGGTGCTGGAAAAAGCACTTTACTCAGGGTACTTTTAGGAGA

ATTAACTCCCCCAGGATGTAAAATGAATCGTCTGGGTGAACTTGCCTATATTCCCCAGTTGGACGAAGTAAC

TCTGCAGGAGGAAAAAGATTTTGCACTTGTAGGCAAGCTAGGTGTTGAGCAATTAAATATACAGACTATGAG

CGGTGGTGAAGAAACAAGGCTTAAAATAGCACAGGCCTTATCGGCACAGGTTCATGGTATTTTAGCGGATGA

ACCTACGAGCCATTTAGACCGTGAAGGAATTGATTTTCTAATAGGACAGCTAAAATATTTTACAGGTGCACT

GTTAGTTATTAGCCATGACCGCTATTTTCTTGATGAAATAGTAGATAAAATATGGGAACTGAAAGATGGCAA

AATCACTGAGTATTGGGGAAACTATTCTGATTATCTTCGTCAGAAAGAGGAAGAACGTAAGAGCCAAGCTGC

AGAATACGAACAATTTATTGCGGAACGTGCCCGATTGGAAAGGGCTGCGGAGGAAAAGCGAAAACAGGCTCG

TAAAATAGAACAGAAGGCAAAAGGTTCTTCAAAGAAAAAAAGTACTGAAGACGGAGGGCGTTTAGCTCATCA

AAAATCAATAGGAAGTAAGGAAAAAAAGATGTATAATGCTGCTAAAACCCTAGAGCACAGGATTGCGGCCTT

AGGAAAAGTAGAAGCTCCGGAAGGCATTCGCAGAATTCGTTTCAGGCAAAGTAAAGCATTGGAGCTCCATAA

TCCATACCCTATAGTCGGTGCAGAAATTAATAAAGTATTTGGGGATAAGGCTCTGTTTGAAAATGCATCTTT

TCAAATTCCGTTAGGAGCAAAAGTGGCGTTAACTGGTGGTAATGGAATCGGAAAAACAACTTTAATCCAAAT

GATCTTAAACCATGAAGAAGGAATTTCTATTTCGCCTAAGGCAAAAATAGGTTACTTTGCACAGAATGGTTA

CAAGTACAACAGTAATCAGAATGTTATGGAGTTTATGCAGAAGGATTGTGACTACAATATATCAGAAATTCG

TTCAGTGCTAGCATCTATGGGGTTCAAACAGAACGATATTGGAAAAAGTTTATCTGTTTTAAGCGGTGGAGA

AATTATAAAATTGTTGCTTGCTAAAATGCTCATGGGTAGATATAACATCCTAATAATGGATGAACCCAGTAA

CTTCCTTGACATACCAAGTTTAGAGGCTTTGGAAATACTAATGAAGGAGTACACCGGAACTATCGTGTTTAT

CACCCACGATAAACGATTACTCGAAAATGTAGCAGATGTAGTTTATGAAATTAGAGATAAGAAAATAAATCT

GAAACATTAAATTTAAGGTAGTCGCTGGTCAGTATAGTCTGTTCTGGTTGGCGACTCCATTGTTAAAGAGTA

TAAAGACTTTAGATTTTATGAATATTAAAAATAGGAACAGTCAATTGAACTGCTCCTATTTTTCTGCTAAAT

ATATTGTAGTTTTCTTATATGTATAATGATAGATTAGCGGATTCTCATCTACGGTACTTACTTCAAATATGA

AGAAGTGATCGCGGTTATCTCTGGACTTTTCCTTATTGAGGACAAAGTAATTCTTACGTGAAGTCGCCATTG

TTTTTAGGATATCATCAGTTAGGAAGGTCAATGGAATATTCATGTTAGAGTAGCGGTAGAAGTCACGTTCAA

AATCTTGGTAGCTCTCGCTATAATAGTCCATTTGTAGGTGATTACGCTGAAACTCAAGCTGATTCATAGAGC

ACCTCCTCGACAAGTTCAATACTAATAATGTCTTTTAATTTCAAATTGATGTGACCTGTTGTAGTTTTTATC

AAAATGAAATCTTTGGTCAGACTTGGTATTGTTCCAGTGTAGGAAACACGCTTGTTTTTTTCAATCACTTGA

ATGCGTGTGCGTAGCTGCCCGGCGTATACTTGACTGAGGAGTAATAATTTCTTCTCTAGTGATAAGTCAGAC

ATGTACGTTACTTTGTTTGTATCATCAGAGAGTGCTGATGCATGTTCAGATAGGAAAAAGCCCATCCATTTT

TGCATCTTTGTATCCTGGTACTCTCTTGCTGATTGAAATGGTAAATATGAACGGTCAATCATATCAAATCCT

TTCTATGCAGAGGCAAGGGTATTTTTATCAAATTGAATCGTAAAACCTTGAATTCCCCCACCTGTGTAACAT

TCTTTAAAGCGATTGATTACCTCAGTATAGATTATCACAGATGAGCTTGTTGGCTTAATGCTAAATGTAAAT

TCCAATGGTAATCGGTTTTCAGATTTAGCATGTACTAGTCGTATCGATATTTCAGTTGTTTTGAGTTTTCTC

TGACGAAGTTTTGAAGTTGCTGTTTCAACGATTCCATGTAAAAATCTTTCAAGCATTTCAATATCATTACAT

CCTTTGCTACGGATTTCTGAAAATTGTACTGTATTTTTTTCTTGTTTCATTTTAATCCCTCCAATCCACCCG

CGGAATGACCACCGATAAGTTTACTGCGTTCAATATTTCTGGAACCTTCAGTTAGGACGGTTCCTTTTTGTA

TGGCTAAAAAACCAAACTGTTCTCTGACAACATCAATAGCTGTCTGAAGTCTATTATCTTTTTCAATTTGTT

CTACATCATCAAAGAGTGATAGTAGAGTATAGCTTTCATCTACGAAGCCACTATAAGATACACCAATTTGTC

TCACTGCACCAGAGGTGTATTTTTTCGGAATAATACAAGTACATGACTCACCATTGTTTTGGGGAGATTTG

CGGGTTCAATTTTATTCTGAGCATTTATAGATTTTTTCATCTCAGTCCTAGAATAGCCAATATGAATAGAAA

CGACAGTAGTCAATACTAGGGCTACTGTTCCGTTCCACAGTATCATTTAAAAATCATT-TTCACACCCTTTC

GTCTATTAGTATAGAAGAAAGCTCTCAGCACA

>GA19998/Mega-2.II

CATGTTGAGGCGGTAAGTTTGCTAGTCAAGGAGTAAAACGACGAAGATTAGCATTTACTTCCGCCCATGCGA

TAGCTGTCCGTGATTGACAAGTGCTAGCACGCAGACAGAACGGAGATAGCGAACCGCTGAGTGTGTCGCTCT

GCTCGTAAAAGCTTAGAAACCTTTGAACGAAAGGGATAATGAAAGCCTTGATTGCAAGGCTTTTTGCTTTAT

GGTGGGTAAGTATCAGAGTGAGAAAATTTTTGGAATGAGTAGAAGTGATAGCTAGAAATTATCAGTTTCTA

TTTCCATTTACCCTGTGGGTACGTGTTTGTTTCCATTGACAAGGAGTTTGTGGGAATAGAAATGT

ACCCACCTTGTTTGAATCAAGTGAAGTGTAGTTGAAGGAAATCTGTTGAAAGCAATACTTCATTTTACCG

AATAAGTAATAATTTAGGCAACTTCAAATCGATTAAAAAAAACTATTTTAAAGGTTAAGAGTAGACAAAA

ATTGTCCACTCTTTTTTGCAAACTCAATTTATCAATAAATGAAATGAGGGAATGTAAAATG

AAATATTTTGAGGTTGAGTTAGAAAATCCTGATGAATTTTTAAAACTACAAACAGAAGATTTTGTGAAAG

CTAATCGCTTGCTACTAAGGAAGATAATCCAGAGCGTTACAGTCTATGAAGAAAACTTCGTCATATCCTT

TAAATCTGGCATCGAATTGGAAGTATGAGTCTCATTCCATAACTTTTATATTGAACATATCATCTTGTTGTG

TTATACTATAAATTGATATAAACAAAGATGTAGGAGGAACCGAAACTATGACAGCCTCAATGCGTTTAAGAT

AAGCTGGCAATAAAAAAAGCAGAATCTATACCCGATGATAGGCTTTTTTGTTGTGCTTATTTATACGATATT

GAGCATTCATTAGTTACGGTGAGGATATTGGTTATTTAACTATACCTTTATTTAACTATGTCTTTAATATGA

ATGTTTCCAAATTGTATGTATGCAGACCAAAAGCCACATTGTGGGGTTTGGCCTGCATTTTTTTTGCCTA

GAATGCTATTCAAAATAGAAATTCAAGCAAAATAATATGCAGGAGATAATATAAATGGAAAAATACAACAAT

TGGAAACGAAAATTTTATGCAATATGGGCAGGGCAAGCAGTATCATTAATCACTAGTGCCATCCTGCAAATG

GCGATTATTTTTTACCTTACAGAAAAAACAGGATCTGCGATGGTCTTGTCTATGGCTTCATTAGTAGGTTTT

TTACCCTATGCGATTTTGGGACCTGCCATTGGTGTGCTAGTGGATCGTCATGATAGGAAGAAGATAATGATT

GGTGCCGATTTAATTATCGCAGCAGCTGGTGCAGTGCTTGCTATTGTTGCATTCTGTATGGAGCTACCTGTC

TGGATGATTATGATAGTATTGTTTATCCGTAGCATTGGAACAGCTTTTCATACCCCAGCACTCAATGCGGTT

ACACCACTTTTAGTACCAGAAGAACAGCTAACGAAATGCGCAGGCTATAGTCAGTCTTTGCAGTCTATAAGC

TATATTGTTAGTCCGGCAGTTGCAGCACTCTTATACTCCGTTTGGGATTTAAATGCTATTATTGCCATCGAC

GTATTGGGTGCTGTGATTGCATCTATTACGGTAGCAATTGTACGTATACCTAAGCTGGGTAATCAAGTGCAA

AGTTTAGAACCAAATTTCATAAGGGAGATGAAAGAAGGAGTTGTGGTTCTGAGACAAAACAAAGGATTGTTT

GCCTTATTACTCTTAGGAACACTATATACTTTTGTTTATATGCCAATCAATGCACTATTTCCTTTAATAAGC

ATGGAACACTTTAATGGAACGCCTGTGCATATTTCTATTACGGAAATTTCCTTTGCATTTGGGATGCTAGCA

GGAGGCTTATTATTAGGAAGATTAGGGGGCTTCGAAAAGCATGTATTACTAATAACAAGTTCATTTTTTATA

ATGGGGACCAGTTTAGCCGTTTCGGGAATACTTCCTCCAAATGGATTTGTAATATTCGTAGTTTGCTGTGCA

ATAATGGGGCTTTCGGTGCCATTTTATAGCGGTGTGCAAACAGCTCTTTTTCAGGAGAAAATTAAGCCTGAA

TATTTAGGACGTGTATTTTCTTTGATCGGAAGTATCATGTCACTTGCTATGCCAATTGGGTTAATTCTTTCT

GGATTCTTTGCTGATAAAATCGGTGTAAATCATTGGTTTTTACTATCAGGTATTTTAATTATTGGCATTGCT

ATAGTTTGCCAAATGATAACTGAGGTTAGAAAATTAGATTTAAAATAAACAATATTGGAGGAA

ATGTTATGGAATTAATATT

AAAAGCAAAAGACATTCGTGTGGAATTCAAAGGACGCGATGTTTTAGATATAAATGAATTAGAAGTATATGA

TTATGACCGTATTGGTTTAGTAGGAGCAAATGGTGCTGGAAAAAGCACTTTACTCAGGGTACTTTTAGGAGA

ATTAACTCCCCCAGGATGTAAAATGAATCGTCTGGGTGAACTTGCCTATATTCCCCAGTTGGACGAAGTAAC

TCTGCAGGAGGAAAAAGATTTTGCACTTGTAGGCAAGCTAGGTGTTGAGCAATTAAATATACAGACTATGAG

CGGTGGTGAAGAAACAAGGCTTAAAATAGCACAGGCCTTATCGGCACAGGTTCATGGTATTTTAGCGGATGA

ACCTACGAGCCATTTAGACCGTGAAGGAATTGATTTTCTAATAGGACAGCTAAAATATTTTACAGGTGCACT

GTTAGTTATTAGCCATGACCGCTATTTTCTTGATGAAATAGTAGATAAAATATGGGAACTGAAAGATGGCAA

AATCACTGAGTATTGGGGAAACTATTCTGATTATCTTCGTCAGAAAGAGGAAGAACGTAAGAGCCAAGCTGC

AGAATACGAACAATTTATTGCGGAACGTGCCCGATTGGAAAGGGCTGCGGAGGAAAAGCGAAAACAGGCTCG

TAAAATAGAACAGAAGGCAAAAGGTTCTTCAAAGAAAAAAAGTACTGAAGACGGAGGGCGTTTAGCTCATCA

AAAATCAATAGGAAGTAAGGAAAAAAAGATGTATAATGCTGCTAAAACCCTAGAGCACAGGATTGCGGCCTT

AGGAAAAGTAGAAGCTCCGGAAGGCATTCGCAGAATTCGTTTCAGGCAAAGTAAAGCATTGGAGCTCCATAA

TCCATACCCTATAGTCGGTGCAGAAATTAATAAAGTATTTGGGGATAAGGCTCTGTTTGAAAATGCATCTTT

TCAAATTCCGTTAGGAGCAAAAGTGGCGTTAACTGGTGGTAATGGAATCGGAAAAACAACTTTAATCCAAAT

GATCTTAAACCATGAAGAAGGAATTTCTATTTCGCCTAAGGCAAAAATAGGTTACTTTGCACAGAATGGTTA

CAAGTACAACAGTAATCAGAATGTTATGGAGTTTATGCAGAAGGATTGTGACTACAATATATCAGAAATTCG

TTCAGTGCTAGCATCTATGGGGTTCAAACAGAACGATATTGGAAAAAGTTTATCTGTTTTAAGCGGTGGAGA

AATTATAAAATTGTTGCTTGCTAAAATGCTCATGGGTAGATATAACATCCTAATAATGGATGAACCCAGTAA

CTTCCTTGACATACCAAGTTTAGAGGCTTTGGAAATACTAATGAAGGAGTACACCGGAACTATCGTGTTTAT

CACCCACGATAAACGATTACTCGAAAATGTAGCAGATGTAGTTTATGAAATTAGAGATAAGAAAATAAATCT

GAAACATTAAATTTAAGGTAGTCGCTGGTCAGTATAGTCTGTTCTGGTTGGCGACTCCATTGTTAAAGAGTA

TAAAGACTTTAGATTTTATGAATATTAAAAATAGGAACAGTCAATTGAACTGCTCCTATTTTTCTGCTAAAT

ATATTGTAGTTTTCTTATATGTATAATGATAGATTAGCGGATTCTCATCTACGGTACTTACTTCAAATATGA

AGAAGTGATCGCGGTTATCTCTGGACTTTTCCTTATTGAGGACAAAGTAATTCTTACGTGAAGTCGCCATTG

TTTTTAGGATATCATCAGTTAGGAAGGTCAATGGAATATTCATGTTAGAGTAGCGGTAGAAGTCACGTTCAA

AATCTTGGTAGCTCTCGCTATAATAGTCCATTTGTAGGTGATTACGCTGAAACTCAAGCTGATTCATAGAGC

ACCTCCTCGACAAGTTCAATACTAATAATGTCTTTTAATTTCAAATTGATGTGACCTGTTGTAGTTTTTATC

AAAATGAAATCTTTGGTCAGACTTGGTATTGTTCCAGTGTAGGAAACACGCTTGTTTTTTTCAATCACTTGA

ATGCGTGTGCGTAGCTGCCCGGCGTATACTTGACTGAGGAGTAATAATTTCTTCTCTAGTGATAAGTCAGAC

ATGTACGTTACTTTGTTTGTATCATCAGAGAGTGCTGATGCATGTTCAGATAGGAAAAAGCCCATCCATTTT

TGCATCTTTGTATCCTGGTACTCTCTTGCTGATTGAAATGGTAAATATGAACGGTCAATCATATCAAATCCT

TTCTATGCAGAGGCAAGGGTATTTTTATCAAATTGAATCGTAAAACCTTGAATTCCCCCACCTGTGTAACAT

TCTTTAAAGCGATTGATTACCTCAGTATAGATTATCACAGATGAGCTTGTTGGCTTAATGCTAAATGTAAAT

TCCAATGGTAATCGGTTTTCAGATTTAGCATGTACTAGTCGTATCGATATTTCAGTTGTTTTGAGTTTTCTC

TGACGAAGTTTTGAAGTTGCTGTTTCAACGATTCCATGTAAAAATCTTTCAAGCATTTCAATATCATTACAT

CCTTTGCTACGGATTTCTGAAAATTGTACTGTATTTTTTTCTTGTTTCATTTTAATCCCTCCAATCCACCCG

CGGAATGACCACCGATAAGTTTACTGCGTTCAATATTTCTGGAACCTTCAGTTAGGACGGTTCCTTTTTGTA

TGGCTAAAAAACCAAACTGTTCTCTGACAACATCAATAGCTGTCTGAAGTCTATTATCTTTTTCAATTTGTT

CTACATCATCAAAGAGTGATAGTAGAGTATAGCTTTCATCTACGAAGCCACTATAAGATACACCAATTTGTC

TCACTGCACCAGAGGTGTATTTTTTCGGAATAATACAAGTACATGACTCACCATTGTTTTGGGGAGATTTG

CGGGTTCAATTTTATTCTGAGCATTTATAGATTTTTTCATCTCAGTCCTAGAATAGCCAATATGAATAGAAA

CGACAGTAGTCAATACTAGGGCTACTGTTCCGTTCCACAGTATCATTTAAAAATCATT-TTCACACCCTTTC

GTCTATTAGTATAGAAGAAAGCTCTCAGCACA

>GA18068/Mega-2.II

CATGTTGAGGCGGTAAGTTTGCTAGTCAAGGAGTAAAACGACGAAGATTAGCATTTACTTCCGCCCATGCGA

TAGCTGTCCGTGATTGACAAGTGCTAGCACGCAGACAGAACGGAGATAGCGAACCGCTGAGTGTGTCGCTCT

GCTCGTAAAAGCTTAGAAACCTTTGAACGAAAGGGATAATGAAAGCCTTGATTGCAAGGCTTTTTGCTTTAT

GGTGGGTAAGTATCAGAGTGAGAAAATTTTTGGAATGAGTAGAAGTGATAGCTAGAAATTATCAGTTTCTA

TTTCCATTTACCCTGTGGGTACGTGTTTGTTTCCATTGACAAGGAGTTTGTGGGAATAGAAATGT

ACCCACCTTGTTTGAATCAAGTGAAGTGTAGTTGAAGGAAATCTGTTGAAAGCAATACTTCATTTTACCG

AATAAGTAATAATTTAGGCAACTTCAAATCGATTAAAAAAAACTATTTTAAAGGTTAAGAGTAGACAAAA

ATTGTCCACTCTTTTTTGCAAACTCAATTTATCAATAAATGAAATGAGGGAATGTAAAATG

AAATATTTTGAGGTTGAGTTAGAAAATCCTGATGAATTTTTAAAACTACAAACAGAAGATTTTGTGAAAG

CTAATCGCTTGCTACTAAGGAAGATAATCCAGAGCGTTACAGTCTATGAAGAAAACTTCGTCATATCCTT

TAAATCTGGCATCGAATTGGAAGTATGAGTCTCATTCCATAACTTTTATATTGAACATATCATCTTGTTGTG

TTATACTATAAATTGATATAAACAAAGATGTAGGAGGAACCGAAACTATGACAGCCTCAATGCGTTTAAGAT

AAGCTGGCAATAAAAAAAGCAGAATCTATACCCGATGATAGGCTTTTTTGTTGTGCTTATTTATACGATATT

GAGCATTCATTAGTTACGGTGAGGATATTGGTTATTTAACTATACCTTTATTTAACTATGTCTTTAATATGA

ATGTTTCCAAATTGTATGTATGCAGACCAAAAGCCACATTGTGGGGTTTGGCCTGCATTTTTTTTGCCTA

GAATGCTATTCAAAATAGAAATTCAAGCAAAATAATATGCAGGAGATAATATAAATGGAAAAATACAACAAT

TGGAAACGAAAATTTTATGCAATATGGGCAGGGCAAGCAGTATCATTAATCACTAGTGCCATCCTGCAAATG

GCGATTATTTTTTACCTTACAGAAAAAACAGGATCTGCGATGGTCTTGTCTATGGCTTCATTAGTAGGTTTT

TTACCCTATGCGATTTTGGGACCTGCCATTGGTGTGCTAGTGGATCGTCATGATAGGAAGAAGATAATGATT

GGTGCCGATTTAATTATCGCAGCAGCTGGTGCAGTGCTTGCTATTGTTGCATTCTGTATGGAGCTACCTGTC

TGGATGATTATGATAGTATTGTTTATCCGTAGCATTGGAACAGCTTTTCATACCCCAGCACTCAATGCGGTT

ACACCACTTTTAGTACCAGAAGAACAGCTAACGAAATGCGCAGGCTATAGTCAGTCTTTGCAGTCTATAAGC

TATATTGTTAGTCCGGCAGTTGCAGCACTCTTATACTCCGTTTGGGATTTAAATGCTATTATTGCCATCGAC

GTATTGGGTGCTGTGATTGCATCTATTACGGTAGCAATTGTACGTATACCTAAGCTGGGTAATCAAGTGCAA

AGTTTAGAACCAAATTTCATAAGGGAGATGAAAGAAGGAGTTGTGGTTCTGAGACAAAACAAAGGATTGTTT

GCCTTATTACTCTTAGGAACACTATATACTTTTGTTTATATGCCAATCAATGCACTATTTCCTTTAATAAGC

ATGGAACACTTTAATGGAACGCCTGTGCATATTTCTATTACGGAAATTTCCTTTGCATTTGGGATGCTAGCA

GGAGGCTTATTATTAGGAAGATTAGGGGGCTTCGAAAAGCATGTATTACTAATAACAAGTTCATTTTTTATA

ATGGGGACCAGTTTAGCCGTTTCGGGAATACTTCCTCCAAATGGATTTGTAATATTCGTAGTTTGCTGTGCA

ATAATGGGGCTTTCGGTGCCATTTTATAGCGGTGTGCAAACAGCTCTTTTTCAGGAGAAAATTAAGCCTGAA

TATTTAGGACGTGTATTTTCTTTGATCGGAAGTATCATGTCACTTGCTATGCCAATTGGGTTAATTCTTTCT

GGATTCTTTGCTGATAAAATCGGTGTAAATCATTGGTTTTTACTATCAGGTATTTTAATTATTGGCATTGCT

ATAGTTTGCCAAATGATAACTGAGGTTAGAAAATTAGATTTAAAATAAACAATATTGGAGGAA

ATGTTATGGAATTAATATT

AAAAGCAAAAGACATTCGTGTGGAATTCAAAGGACGCGATGTTTTAGATATAAATGAATTAGAAGTATATGA

TTATGACCGTATTGGTTTAGTAGGAGCAAATGGTGCTGGAAAAAGCACTTTACTCAGGGTACTTTTAGGAGA

ATTAACTCCCCCAGGATGTAAAATGAATCGTCTGGGTGAACTTGCCTATATTCCCCAGTTGGACGAAGTAAC

TCTGCAGGAGGAAAAAGATTTTGCACTTGTAGGCAAGCTAGGTGTTGAGCAATTAAATATACAGACTATGAG

CGGTGGTGAAGAAACAAGGCTTAAAATAGCACAGGCCTTATCGGCACAGGTTCATGGTATTTTAGCGGATGA

ACCTACGAGCCATTTAGACCGTGAAGGAATTGATTTTCTAATAGGACAGCTAAAATATTTTACAGGTGCACT

GTTAGTTATTAGCCATGACCGCTATTTTCTTGATGAAATAGTAGATAAAATATGGGAACTGAAAGATGGCAA

AATCACTGAGTATTGGGGAAACTATTCTGATTATCTTCGTCAGAAAGAGGAAGAACGTAAGAGCCAAGCTGC

AGAATACGAACAATTTATTGCGGAACGTGCCCGATTGGAAAGGGCTGCGGAGGAAAAGCGAAAACAGGCTCG

TAAAATAGAACAGAAGGCAAAAGGTTCTTCAAAGAAAAAAAGTACTGAAGACGGAGGGCGTTTAGCTCATCA

AAAATCAATAGGAAGTAAGGAAAAAAAGATGTATAATGCTGCTAAAACCCTAGAGCACAGGATTGCGGCCTT

AGGAAAAGTAGAAGCTCCGGAAGGCATTCGCAGAATTCGTTTCAGGCAAAGTAAAGCATTGGAGCTCCATAA

TCCATACCCTATAGTCGGTGCAGAAATTAATAAAGTATTTGGGGATAAGGCTCTGTTTGAAAATGCATCTTT

TCAAATTCCGTTAGGAGCAAAAGTGGCGTTAACTGGTGGTAATGGAATCGGAAAAACAACTTTAATCCAAAT

GATCTTAAACCATGAAGAAGGAATTTCTATTTCGCCTAAGGCAAAAATAGGTTACTTTGCACAGAATGGTTA

CAAGTACAACAGTAATCAGAATGTTATGGAGTTTATGCAGAAGGATTGTGACTACAATATATCAGAAATTCG

TTCAGTGCTAGCATCTATGGGGTTCAAACAGAACGATATTGGAAAAAGTTTATCTGTTTTAAGCGGTGGAGA

AATTATAAAATTGTTGCTTGCTAAAATGCTCATGGGTAGATATAACATCCTAATAATGGATGAACCCAGTAA

CTTCCTTGACATACCAAGTTTAGAGGCTTTGGAAATACTAATGAAGGAGTACACCGGAACTATCGTGTTTAT

CACCCACGATAAACGATTACTCGAAAATGTAGCAGATGTAGTTTATGAAATTAGAGATAAGAAAATAAATCT

GAAACATTAAATTTAAGGTAGTCGCTGGTCAGTATAGTCTGTTCTGGTTGGCGACTCCATTGTTAAAGAGTA

TAAAGACTTTAGATTTTATGAATATTAAAAATAGGAACAGTCAATTGAACTGCTCCTATTTTTCTGCTAAAT

ATATTGTAGTTTTCTTATATGTATAATGATAGATTAGCGGATTCTCATCTACGGTACTTACTTCAAATATGA

AGAAGTGATCGCGGTTATCTCTGGACTTTTCCTTATTGAGGACAAAGTAATTCTTACGTGAAGTCGCCATTG

TTTTTAGGATATCATCAGTTAGGAAGGTCAATGGAATATTCATGTTAGAGTAGCGGTAGAAGTCACGTTCAA

AATCTTGGTAGCTCTCGCTATAATAGTCCATTTGTAGGTGATTACGCTGAAACTCAAGCTGATTCATAGAGC

ACCTCCTCGACAAGTTCAATACTAATAATGTCTTTTAATTTCAAATTGATGTGACCTGTTGTAGTTTTTATC

AAAATGAAATCTTTGGTCAGACTTGGTATTGTTCCAGTGTAGGAAACACGCTTGTTTTTTTCAATCACTTGA

ATGCGTGTGCGTAGCTGCCCGGCGTATACTTGACTGAGGAGTAATAATTTCTTCTCTAGTGATAAGTCAGAC

ATGTACGTTACTTTGTTTGTATCATCAGAGAGTGCTGATGCATGTTCAGATAGGAAAAAGCCCATCCATTTT

TGCATCTTTGTATCCTGGTACTCTCTTGCTGATTGAAATGGTAAATATGAACGGTCAATCATATCAAATCCT

TTCTATGCAGAGGCAAGGGTATTTTTATCAAATTGAATCGTAAAACCTTGAATTCCCCCACCTGTGTAACAT

TCTTTAAAGCGATTGATTACCTCAGTATAGATTATCACAGATGAGCTTGTTGGCTTAATGCTAAATGTAAAT

TCCAATGGTAATCGGTTTTCAGATTTAGCATGTACTAGTCGTATCGATATTTCAGTTGTTTTGAGTTTTCTC

TGACGAAGTTTTGAAGTTGCTGTTTCAACGATTCCATGTAAAAATCTTTCAAGCATTTCAATATCATTACAT

CCTTTGCTACGGATTTCTGAAAATTGTACTGTATTTTTTTCTTGTTTCATTTTAATCCCTCCAATCCACCCG

CGGAATGACCACCGATAAGTTTACTGCGTTCAATATTTCTGGAACCTTCAGTTAGGACGGTTCCTTTTTGTA

TGGCTAAAAAACCAAACTGTTCTCTGACAACATCAATAGCTGTCTGAAGTCTATTATCTTTTTCAATTTGTT

CTACATCATCAAAGAGTGATAGTAGAGTATAGCTTTCATCTACGAAGCCACTATAAGATACACCAATTTGTC

TCACTGCACCAGAGGTGTATTTTTTCGGAATAATACAAGTACATGACTCACCATTGTTTTGGGGAGATTTG

CGGGTTCAATTTTATTCTGAGCATTTATAGATTTTTTCATCTCAGTCCTAGAATAGCCAATATGAATAGAAA

CGACAGTAGTCAATACTAGGGCTACTGTTCCGTTCCACAGTATCATTTAAAAATCATT-TTCACACCCTTTC

GTCTATTAGTATAGAAGAAAGCTCTCAGCACA

>GA17484/Mega-2.II

CATGTTGAGGCGGTAAGTTTGCTAGTCAAGGAGTAAAACGACGAAGATTAGCATTTACTTCCGCCCATGCGA

TAGCTGTCCGTGATTGACAAGTGCTAGCACGCAGACAGAACGGAGATAGCGAACCGCTGAGTGTGTCGCTCT

GCTCGTAAAAGCTTAGAAACCTTTGAACGAAAGGGATAATGAAAGCCTTGATTGCAAGGCTTTTTGCTTTAT

GGTGGGTAAGTATCAGAGTGAGAAAATTTTTGGAATGAGTAGAAGTGATAGCTAGAAATTATCAGTTTCTA

TTTCCATTTACCCTGTGGGTACGTGTTTGTTTCCATTGACAAGGAGTTTGTGGGAATAGAAATGT

ACCCACCTTGTTTGAATCAAGTGAAGTGTAGTTGAAGGAAATCTGTTGAAAGCAATACTTCATTTTACCG

AATAAGTAATAATTTAGGCAACTTCAAATCGATTAAAAAAAACTATTTTAAAGGTTAAGAGTAGACAAAA

ATTGTCCACTCTTTTTTGCAAACTCAATTTATCAATAAATGAAATGAGGGAATGTAAAATG

AAATATTTTGAGGTTGAGTTAGAAAATCCTGATGAATTTTTAAAACTACAAACAGAAGATTTTGTGAAAG

CTAATCGCTTGCTACTAAGGAAGATAATCCAGAGCGTTACAGTCTATGAAGAAAACTTCGTCATATCCTT

TAAATCTGGCATCGAATTGGAAGTATGAGTCTCATTCCATAACTTTTATATTGAACATATCATCTTGTTGTG

TTATACTATAAATTGATATAAACAAAGATGTAGGAGGAACCGAAACTATGACAGCCTCAATGCGTTTAAGAT

AAGCTGGCAATAAAAAAAGCAGAATCTATACCCGATGATAGGCTTTTTTGTTGTGCTTATTTATACGATATT

GAGCATTCATTAGTTACGGTGAGGATATTGGTTATTTAACTATACCTTTATTTAACTATGTCTTTAATATGA

ATGTTTCCAAATTGTATGTATGCAGACCAAAAGCCACATTGTGGGGTTTGGCCTGCATTTTTTTTGCCTA

GAATGCTATTCAAAATAGAAATTCAAGCAAAATAATATGCAGGAGATAATATAAATGGAAAAATACAACAAT

TGGAAACGAAAATTTTATGCAATATGGGCAGGGCAAGCAGTATCATTAATCACTAGTGCCATCCTGCAAATG

GCGATTATTTTTTACCTTACAGAAAAAACAGGATCTGCGATGGTCTTGTCTATGGCTTCATTAGTAGGTTTT

TTACCCTATGCGATTTTGGGACCTGCCATTGGTGTGCTAGTGGATCGTCATGATAGGAAGAAGATAATGATT

GGTGCCGATTTAATTATCGCAGCAGCTGGTGCAGTGCTTGCTATTGTTGCATTCTGTATGGAGCTACCTGTC

TGGATGATTATGATAGTATTGTTTATCCGTAGCATTGGAACAGCTTTTCATACCCCAGCACTCAATGCGGTT

ACACCACTTTTAGTACCAGAAGAACAGCTAACGAAATGCGCAGGCTATAGTCAGTCTTTGCAGTCTATAAGC

TATATTGTTAGTCCGGCAGTTGCAGCACTCTTATACTCCGTTTGGGATTTAAATGCTATTATTGCCATCGAC

GTATTGGGTGCTGTGATTGCATCTATTACGGTAGCAATTGTACGTATACCTAAGCTGGGTAATCAAGTGCAA

AGTTTAGAACCAAATTTCATAAGGGAGATGAAAGAAGGAGTTGTGGTTCTGAGACAAAACAAAGGATTGTTT

GCCTTATTACTCTTAGGAACACTATATACTTTTGTTTATATGCCAATCAATGCACTATTTCCTTTAATAAGC

ATGGAACACTTTAATGGAACGCCTGTGCATATTTCTATTACGGAAATTTCCTTTGCATTTGGGATGCTAGCA

GGAGGCTTATTATTAGGAAGATTAGGGGGCTTCGAAAAGCATGTATTACTAATAACAAGTTCATTTTTTATA

ATGGGGACCAGTTTAGCCGTTTCGGGAATACTTCCTCCAAATGGATTTGTAATATTCGTAGTTTGCTGTGCA

ATAATGGGGCTTTCGGTGCCATTTTATAGCGGTGTGCAAACAGCTCTTTTTCAGGAGAAAATTAAGCCTGAA

TATTTAGGACGTGTATTTTCTTTGATCGGAAGTATCATGTCACTTGCTATGCCAATTGGGTTAATTCTTTCT

GGATTCTTTGCTGATAAAATCGGTGTAAATCATTGGTTTTTACTATCAGGTATTTTAATTATTGGCATTGCT

ATAGTTTGCCAAATGATAACTGAGGTTAGAAAATTAGATTTAAAATAAACAATATTGGAGGAA

ATGTTATGGAATTAATATT

AAAAGCAAAAGACATTCGTGTGGAATTCAAAGGACGCGATGTTTTAGATATAAATGAATTAGAAGTATATGA

TTATGACCGTATTGGTTTAGTAGGAGCAAATGGTGCTGGAAAAAGCACTTTACTCAGGGTACTTTTAGGAGA

ATTAACTCCCCCAGGATGTAAAATGAATCGTCTGGGTGAACTTGCCTATATTCCCCAGTTGGACGAAGTAAC

TCTGCAGGAGGAAAAAGATTTTGCACTTGTAGGCAAGCTAGGTGTTGAGCAATTAAATATACAGACTATGAG

CGGTGGTGAAGAAACAAGGCTTAAAATAGCACAGGCCTTATCGGCACAGGTTCATGGTATTTTAGCGGATGA

ACCTACGAGCCATTTAGACCGTGAAGGAATTGATTTTCTAATAGGACAGCTAAAATATTTTACAGGTGCACT

GTTAGTTATTAGCCATGACCGCTATTTTCTTGATGAAATAGTAGATAAAATATGGGAACTGAAAGATGGCAA

AATCACTGAGTATTGGGGAAACTATTCTGATTATCTTCGTCAGAAAGAGGAAGAACGTAAGAGCCAAGCTGC

AGAATACGAACAATTTATTGCGGAACGTGCCCGATTGGAAAGGGCTGCGGAGGAAAAGCGAAAACAGGCTCG

TAAAATAGAACAGAAGGCAAAAGGTTCTTCAAAGAAAAAAAGTACTGAAGACGGAGGGCGTTTAGCTCATCA

AAAATCAATAGGAAGTAAGGAAAAAAAGATGTATAATGCTGCTAAAACCCTAGAGCACAGGATTGCGGCCTT

AGGAAAAGTAGAAGCTCCGGAAGGCATTCGCAGAATTCGTTTCAGGCAAAGTAAAGCATTGGAGCTCCATAA

TCCATACCCTATAGTCGGTGCAGAAATTAATAAAGTATTTGGGGATAAGGCTCTGTTTGAAAATGCATCTTT

TCAAATTCCGTTAGGAGCAAAAGTGGCGTTAACTGGTGGTAATGGAATCGGAAAAACAACTTTAATCCAAAT

GATCTTAAACCATGAAGAAGGAATTTCTATTTCGCCTAAGGCAAAAATAGGTTACTTTGCACAGAATGGTTA

CAAGTACAACAGTAATCAGAATGTTATGGAGTTTATGCAGAAGGATTGTGACTACAATATATCAGAAATTCG

TTCAGTGCTAGCATCTATGGGGTTCAAACAGAACGATATTGGAAAAAGTTTATCTGTTTTAAGCGGTGGAGA

AATTATAAAATTGTTGCTTGCTAAAATGCTCATGGGTAGATATAACATCCTAATAATGGATGAACCCAGTAA

CTTCCTTGACATACCAAGTTTAGAGGCTTTGGAAATACTAATGAAGGAGTACACCGGAACTATCGTGTTTAT

CACCCACGATAAACGATTACTCGAAAATGTAGCAGATGTAGTTTATGAAATTAGAGATAAGAAAATAAATCT

GAAACATTAAATTTAAGGTAGTCGCTGGTCAGTATAGTCTGTTCTGGTTGGCGACTCCATTGTTAAAGAGTA

TAAAGACTTTAGATTTTATGAATATTAAAAATAGGAACAGTCAATTGAACTGCTCCTATTTTTCTGCTAAAT

ATATTGTAGTTTTCTTATATGTATAATGATAGATTAGCGGATTCTCATCTACGGTACTTACTTCAAATATGA

AGAAGTGATCGCGGTTATCTCTGGACTTTTCCTTATTGAGGACAAAGTAATTCTTACGTGAAGTCGCCATTG

TTTTTAGGATATCATCAGTTAGGAAGGTCAATGGAATATTCATGTTAGAGTAGCGGTAGAAGTCACGTTCAA

AATCTTGGTAGCTCTCGCTATAATAGTCCATTTGTAGGTGATTACGCTGAAACTCAAGCTGATTCATAGAGC

ACCTCCTCGACAAGTTCAATACTAATAATGTCTTTTAATTTCAAATTGATGTGACCTGTTGTAGTTTTTATC

AAAATGAAATCTTTGGTCAGACTTGGTATTGTTCCAGTGTAGGAAACACGCTTGTTTTTTTCAATCACTTGA

ATGCGTGTGCGTAGCTGCCCGGCGTATACTTGACTGAGGAGTAATAATTTCTTCTCTAGTGATAAGTCAGAC

ATGTACGTTACTTTGTTTGTATCATCAGAGAGTGCTGATGCATGTTCAGATAGGAAAAAGCCCATCCATTTT

TGCATCTTTGTATCCTGGTACTCTCTTGCTGATTGAAATGGTAAATATGAACGGTCAATCATATCAAATCCT

TTCTATGCAGAGGCAAGGGTATTTTTATCAAATTGAATCGTAAAACCTTGAATTCCCCCACCTGTGTAACAT

TCTTTAAAGCGATTGATTACCTCAGTATAGATTATCACAGATGAGCTTGTTGGCTTAATGCTAAATGTAAAT

TCCAATGGTAATCGGTTTTCAGATTTAGCATGTACTAGTCGTATCGATATTTCAGTTGTTTTGAGTTTTCTC

TGACGAAGTTTTGAAGTTGCTGTTTCAACGATTCCATGTAAAAATCTTTCAAGCATTTCAATATCATTACAT

CCTTTGCTACGGATTTCTGAAAATTGTACTGTATTTTTTTCTTGTTTCATTTTAATCCCTCCAATCCACCCG

CGGAATGACCACCGATAAGTTTACTGCGTTCAATATTTCTGGAACCTTCAGTTAGGACGGTTCCTTTTTGTA

TGGCTAAAAAACCAAACTGTTCTCTGACAACATCAATAGCTGTCTGAAGTCTATTATCTTTTTCAATTTGTT

CTACATCATCAAAGAGTGATAGTAGAGTATAGCTTTCATCTACGAAGCCACTATAAGATACACCAATTTGTC

TCACTGCACCAGAGGTGTATTTTTTCGGAATAATACAAGTACATGACTCACCATTGTTTTGGGGAGATTTG

CGGGTTCAATTTTATTCTGAGCATTTATAGATTTTTTCATCTCAGTCCTAGAATAGCCAATATGAATAGAAA

CGACAGTAGTCAATACTAGGGCTACTGTTCCGTTCCACAGTATCATTTAAAAATCATT-TTCACACCCTTTC

GTCTATTAGTATAGAAGAAAGCTCTCAGCACA

>GA08780/Mega-2.II

CATGTTGAGGCGGTAAGTTTGCTAGTCAAGGAGTAAAACGACGAAGATTAGCATTTACTTCCGCCCATGCGA

TAGCTGTCCGTGATTGACAAGTGCTAGCACGCAGACAGAACGGAGATAGCGAACCGCTGAGTGTGTCGCTCT

GCTCGTAAAAGCTTAGAAACCTTTGAACGAAAGGGATAATGAAAGCCTTGATTGCAAGGCTTTTTGCTTTAT

GGTGGGTAAGTATCAGAGTGAGAAAATTTTTGGAATGAGTAGAAGTGATAGCTAGAAATTATCAGTTTCTA

TTTCCATTTACCCTGTGGGTACGTGTTTGTTTCCATTGACAAGGAGTTTGTGGGAATAGAAATGT

ACCCACCTTGTTTGAATCAAGTGAAGTGTAGTTGAAGGAAATCTGTTGAAAGCAATACTTCATTTTACCG

AATAAGTAATAATTTAGGCAACTTCAAATCGATTAAAAAAAACTATTTTAAAGGTTAAGAGTAGACAAAA

ATTGTCCACTCTTTTTTGCAAACTCAATTTATCAATAAATGAAATGAGGGAATGTAAAATG

AAATATTTTGAGGTTGAGTTAGAAAATCCTGATGAATTTTTAAAACTACAAACAGAAGATTTTGTGAAAG

CTAATCGCTTGCTACTAAGGAAGATAATCCAGAGCGTTACAGTCTATGAAGAAAACTTCGTCATATCCTT

TAAATCTGGCATCGAATTGGAAGTATGAGTCTCATTCCATAACTTTTATATTGAACATATCATCTTGTTGTG

TTATACTATAAATTGATATAAACAAAGATGTAGGAGGAACCGAAACTATGACAGCCTCAATGCGTTTAAGAT

AAGCTGGCAATAAAAAAAGCAGAATCTATACCCGATGATAGGCTTTTTTGTTGTGCTTATTTATACGATATT

GAGCATTCATTAGTTACGGTGAGGATATTGGTTATTTAACTATACCTTTATTTAACTATGTCTTTAATATGA

ATGTTTCCAAATTGTATGTATGCAGACCAAAAGCCACATTGTGGGGTTTGGCCTGCATTTTTTTTGCCTA

GAATGCTATTCAAAATAGAAATTCAAGCAAAATAATATGCAGGAGATAATATAAATGGAAAAATACAACAAT

TGGAAACGAAAATTTTATGCAATATGGGCAGGGCAAGCAGTATCATTAATCACTAGTGCCATCCTGCAAATG

GCGATTATTTTTTACCTTACAGAAAAAACAGGATCTGCGATGGTCTTGTCTATGGCTTCATTAGTAGGTTTT

TTACCCTATGCGATTTTGGGACCTGCCATTGGTGTGCTAGTGGATCGTCATGATAGGAAGAAGATAATGATT

GGTGCCGATTTAATTATCGCAGCAGCTGGTGCAGTGCTTGCTATTGTTGCATTCTGTATGGAGCTACCTGTC

TGGATGATTATGATAGTATTGTTTATCCGTAGCATTGGAACAGCTTTTCATACCCCAGCACTCAATGCGGTT

ACACCACTTTTAGTACCAGAAGAACAGCTAACGAAATGCGCAGGCTATAGTCAGTCTTTGCAGTCTATAAGC

TATATTGTTAGTCCGGCAGTTGCAGCACTCTTATACTCCGTTTGGGATTTAAATGCTATTATTGCCATCGAC

GTATTGGGTGCTGTGATTGCATCTATTACGGTAGCAATTGTACGTATACCTAAGCTGGGTAATCAAGTGCAA

AGTTTAGAACCAAATTTCATAAGGGAGATGAAAGAAGGAGTTGTGGTTCTGAGACAAAACAAAGGATTGTTT

GCCTTATTACTCTTAGGAACACTATATACTTTTGTTTATATGCCAATCAATGCACTATTTCCTTTAATAAGC

ATGGAACACTTTAATGGAACGCCTGTGCATATTTCTATTACGGAAATTTCCTTTGCATTTGGGATGCTAGCA

GGAGGCTTATTATTAGGAAGATTAGGGGGCTTCGAAAAGCATGTATTACTAATAACAAGTTCATTTTTTATA

ATGGGGACCAGTTTAGCCGTTTCGGGAATACTTCCTCCAAATGGATTTGTAATATTCGTAGTTTGCTGTGCA

ATAATGGGGCTTTCGGTGCCATTTTATAGCGGTGTGCAAACAGCTCTTTTTCAGGAGAAAATTAAGCCTGAA

TATTTAGGACGTGTATTTTCTTTGATCGGAAGTATCATGTCACTTGCTATGCCAATTGGGTTAATTCTTTCT

GGATTCTTTGCTGATAAAATCGGTGTAAATCATTGGTTTTTACTATCAGGTATTTTAATTATTGGCATTGCT

ATAGTTTGCCAAATGATAACTGAGGTTAGAAAATTAGATTTAAAATAAACAATATTGGAGGAA

ATGTTATGGAATTAATATT

AAAAGCAAAAGACATTCGTGTGGAATTCAAAGGACGCGATGTTTTAGATATAAATGAATTAGAAGTATATGA

TTATGACCGTATTGGTTTAGTAGGAGCAAATGGTGCTGGAAAAAGCACTTTACTCAGGGTACTTTTAGGAGA

ATTAACTCCCCCAGGATGTAAAATGAATCGTCTGGGTGAACTTGCCTATATTCCCCAGTTGGACGAAGTAAC

TCTGCAGGAGGAAAAAGATTTTGCACTTGTAGGCAAGCTAGGTGTTGAGCAATTAAATATACAGACTATGAG

CGGTGGTGAAGAAACAAGGCTTAAAATAGCACAGGCCTTATCGGCACAGGTTCATGGTATTTTAGCGGATGA

ACCTACGAGCCATTTAGACCGTGAAGGAATTGATTTTCTAATAGGACAGCTAAAATATTTTACAGGTGCACT

GTTAGTTATTAGCCATGACCGCTATTTTCTTGATGAAATAGTAGATAAAATATGGGAACTGAAAGATGGCAA

AATCACTGAGTATTGGGGAAACTATTCTGATTATCTTCGTCAGAAAGAGGAAGAACGTAAGAGCCAAGCTGC

AGAATACGAACAATTTATTGCGGAACGTGCCCGATTGGAAAGGGCTGCGGAGGAAAAGCGAAAACAGGCTCG

TAAAATAGAACAGAAGGCAAAAGGTTCTTCAAAGAAAAAAAGTACTGAAGACGGAGGGCGTTTAGCTCATCA

AAAATCAATAGGAAGTAAGGAAAAAAAGATGTATAATGCTGCTAAAACCCTAGAGCACAGGATTGCGGCCTT

AGGAAAAGTAGAAGCTCCGGAAGGCATTCGCAGAATTCGTTTCAGGCAAAGTAAAGCATTGGAGCTCCATAA

TCCATACCCTATAGTCGGTGCAGAAATTAATAAAGTATTTGGGGATAAGGCTCTGTTTGAAAATGCATCTTT

TCAAATTCCGTTAGGAGCAAAAGTGGCGTTAACTGGTGGTAATGGAATCGGAAAAACAACTTTAATCCAAAT

GATCTTAAACCATGAAGAAGGAATTTCTATTTCGCCTAAGGCAAAAATAGGTTACTTTGCACAGAATGGTTA

CAAGTACAACAGTAATCAGAATGTTATGGAGTTTATGCAGAAGGATTGTGACTACAATATATCAGAAATTCG

TTCAGTGCTAGCATCTATGGGGTTCAAACAGAACGATATTGGAAAAAGTTTATCTGTTTTAAGCGGTGGAGA

AATTATAAAATTGTTGCTTGCTAAAATGCTCATGGGTAGATATAACATCCTAATAATGGATGAACCCAGTAA

CTTCCTTGACATACCAAGTTTAGAGGCTTTGGAAATACTAATGAAGGAGTACACCGGAACTATCGTGTTTAT

CACCCACGATAAACGATTACTCGAAAATGTAGCAGATGTAGTTTATGAAATTAGAGATAAGAAAATAAATCT

GAAACATTAAATTTAAGGTAGTCGCTGGTCAGTATAGTCTGTTCTGGTTGGCGACTCCATTGTTAAAGAGTA

TAAAGACTTTAGATTTTATGAATATTAAAAATAGGAACAGTCAATTGAACTGCTCCTATTTTTCTGCTAAAT

ATATTGTAGTTTTCTTATATGTATAATGATAGATTAGCGGATTCTCATCTACGGTACTTACTTCAAATATGA

AGAAGTGATCGCGGTTATCTCTGGACTTTTCCTTATTGAGGACAAAGTAATTCTTACGTGAAGTCGCCATTG

TTTTTAGGATATCATCAGTTAGGAAGGTCAATGGAATATTCATGTTAGAGTAGCGGTAGAAGTCACGTTCAA

AATCTTGGTAGCTCTCGCTATAATAGTCCATTTGTAGGTGATTACGCTGAAACTCAAGCTGATTCATAGAGC

ACCTCCTCGACAAGTTCAATACTAATAATGTCTTTTAATTTCAAATTGATGTGACCTGTTGTAGTTTTTATC

AAAATGAAATCTTTGGTCAGACTTGGTATTGTTCCAGTGTAGGAAACACGCTTGTTTTTTTCAATCACTTGA

ATGCGTGTGCGTAGCTGCCCGGCGTATACTTGACTGAGGAGTAATAATTTCTTCTCTAGTGATAAGTCAGAC

ATGTACGTTACTTTGTTTGTATCATCAGAGAGTGCTGATGCATGTTCAGATAGGAAAAAGCCCATCCATTTT

TGCATCTTTGTATCCTGGTACTCTCTTGCTGATTGAAATGGTAAATATGAACGGTCAATCATATCAAATCCT

TTCTATGCAGAGGCAAGGGTATTTTTATCAAATTGAATCGTAAAACCTTGAATTCCCCCACCTGTGTAACAT

TCTTTAAAGCGATTGATTACCTCAGTATAGATTATCACAGATGAGCTTGTTGGCTTAATGCTAAATGTAAAT

TCCAATGGTAATCGGTTTTCAGATTTAGCATGTACTAGTCGTATCGATATTTCAGTTGTTTTGAGTTTTCTC

TGACGAAGTTTTGAAGTTGCTGTTTCAACGATTCCATGTAAAAATCTTTCAAGCATTTCAATATCATTACAT

CCTTTGCTACGGATTTCTGAAAATTGTACTGTATTTTTTTCTTGTTTCATTTTAATCCCTCCAATCCACCCG

CGGAATGACCACCGATAAGTTTACTGCGTTCAATATTTCTGGAACCTTCAGTTAGGACGGTTCCTTTTTGTA

TGGCTAAAAAACCAAACTGTTCTCTGACAACATCAATAGCTGTCTGAAGTCTATTATCTTTTTCAATTTGTT

CTACATCATCAAAGAGTGATAGTAGAGTATAGCTTTCATCTACGAAGCCACTATAAGATACACCAATTTGTC

TCACTGCACCAGAGGTGTATTTTTTCGGAATAATACAAGTACATGACTCACCATTGTTTTGGGGAGATTTG

CGGGTTCAATTTTATTCTGAGCATTTATAGATTTTTTCATCTCAGTCCTAGAATAGCCAATATGAATAGAAA

CGACAGTAGTCAATACTAGGGCTACTGTTCCGTTCCACAGTATCATTTAAAAATCATT-TTCACACCCTTTC

GTCTATTAGTATAGAAGAAAGCTCTCAGCACA

>GA47388/Mega-2.II

CATGTTGAGGCGGTAAGTTTGCTAGTCAAGGAGTAAAACGACGAAGATTAGCATTTACTTCCGCCCATGCGA

TAGCTGTCCGTGATTGACAAGTGCTAGCACGCAGACAGAACGGAGATAGCGAACCGCTGAGTGTGTCGCTCT

GCTCGTAAAAGCTTAGAAACCTTTGAACGAAAGGGATAATGAAAGCCTTGATTGCAAGGCTTTTTGCTTTAT

GGTGGGTAAGTATCAGAGTGAGAAAATTTTTGGAATGAGTAGAAGTGATAGCTAGAAATTATCAGTTTCTA

TTTCCATTTACCCTGTGGGTACGTGTTTGTTTCCATTGACAAGGAGTTTGTGGGAATAGAAATGT

ACCCACCTTGTTTGAATCAAGTGAAGTGTAGTTGAAGGAAATCTGTTGAAAGCAATACTTCATTTTACCG

AATAAGTAATAATTTAGGCAACTTCAAATCGATTAAAAAAAACTATTTTAAAGGTTAAGAGTAGACAAAA

ATTGTCCACTCTTTTTTGCAAACTCAATTTATCAATAAATGAAATGAGGGAATGTAAAATG

AAATATTTTGAGGTTGAGTTAGAAAATCCTGATGAATTTTTAAAACTACAAACAGAAGATTTTGTGAAAG

CTAATCGCTTGCTACTAAGGAAGATAATCCAGAGCGTTACAGTCTATGAAGAAAACTTCGTCATATCCTT

TAAATCTGGCATCGAATTGGAAGTATGAGTCTCATTCCATAACTTTTATATTGAACATATCATCTTGTTGTG

TTATACTATAAATTGATATAAACAAAGATGTAGGAGGAACCGAAACTATGACAGCCTCAATGCGTTTAAGAT

AAGCTGGCAATAAAAAAAGCAGAATCTATACCCGATGATAGGCTTTTTTGTTGTGCTTATTTATACGATATT

GAGCATTCATTAGTTACGGTGAGGATATTGGTTATTTAACTATACCTTTATTTAACTATGTCTTTAATATGA

ATGTTTCCAAATTGTATGTATGCAGACCAAAAGCCACATTGTGGGGTTTGGCCTGCATTTTTTTTGCCTA

GAATGCTATTCAAAATAGAAATTCAAGCAAAATAATATGCAGGAGATAATATAAATGGAAAAATACAACAAT

TGGAAACGAAAATTTTATGCAATATGGGCAGGGCAAGCAGTATCATTAATCACTAGTGCCATCCTGCAAATG

GCGATTATTTTTTACCTTACAGAAAAAACAGGATCTGCGATGGTCTTGTCTATGGCTTCATTAGTAGGTTTT

TTACCCTATGCGATTTTGGGACCTGCCATTGGTGTGCTAGTGGATCGTCATGATAGGAAGAAGATAATGATT

GGTGCCGATTTAATTATCGCAGCAGCTGGTGCAGTGCTTGCTATTGTTGCATTCTGTATGGAGCTACCTGTC

TGGATGATTATGATAGTATTGTTTATCCGTAGCATTGGAACAGCTTTTCATACCCCAGCACTCAATGCGGTT

ACACCACTTTTAGTACCAGAAGAACAGCTAACGAAATGCGCAGGCTATAGTCAGTCTTTGCAGTCTATAAGC

TATATTGTTAGTCCGGCAGTTGCAGCACTCTTATACTCCGTTTGGGATTTAAATGCTATTATTGCCATCGAC

GTATTGGGTGCTGTGATTGCATCTATTACGGTAGCAATTGTACGTATACCTAAGCTGGGTAATCAAGTGCAA

AGTTTAGAACCAAATTTCATAAGGGAGATGAAAGAAGGAGTTGTGGTTCTGAGACAAAACAAAGGATTGTTT

GCCTTATTACTCTTAGGAACACTATATACTTTTGTTTATATGCCAATCAATGCACTATTTCCTTTAATAAGC

ATGGAACACTTTAATGGAACGCCTGTGCATATTTCTATTACGGAAATTTCCTTTGCATTTGGGATGCTAGCA

GGAGGCTTATTATTAGGAAGATTAGGGGGCTTCGAAAAGCATGTATTACTAATAACAAGTTCATTTTTTATA

ATGGGGACCAGTTTAGCCGTTTCGGGAATACTTCCTCCAAATGGATTTGTAATATTCGTAGTTTGCTGTGCA

ATAATGGGGCTTTCGGTGCCATTTTATAGCGGTGTGCAAACAGCTCTTTTTCAGGAGAAAATTAAGCCTGAA

TATTTAGGACGTGTATTTTCTTTGATCGGAAGTATCATGTCACTTGCTATGCCAATTGGGTTAATTCTTTCT

GGATTCTTTGCTGATAAAATCGGTGTAAATCATTGGTTTTTACTATCAGGTATTTTAATTATTGGCATTGCT

ATAGTTTGCCAAATGATAACTGAGGTTAGAAAATTAGATTTAAAATAAACAATATTGGAGGAA

ATGTTATGGAATTAATATT

AAAAGCAAAAGACATTCGTGTGGAATTCAAAGGACGCGATGTTTTAGATATAAATGAATTAGAAGTATATGA

TTATGACCGTATTGGTTTAGTAGGAGCAAATGGTGCTGGAAAAAGCACTTTACTCAGGGTACTTTTAGGAGA

ATTAACTCCCCCAGGATGTAAAATGAATCGTCTGGGTGAACTTGCCTATATTCCCCAGTTGGACGAAGTAAC

TCTGCAGGAGGAAAAAGATTTTGCACTTGTAGGCAAGCTAGGTGTTGAGCAATTAAATATACAGACTATGAG

CGGTGGTGAAGAAACAAGGCTTAAAATAGCACAGGCCTTATCGGCACAGGTTCATGGTATTTTAGCGGATGA

ACCTACGAGCCATTTAGACCGTGAAGGAATTGATTTTCTAATAGGACAGCTAAAATATTTTACAGGTGCACT

GTTAGTTATTAGCCATGACCGCTATTTTCTTGATGAAATAGTAGATAAAATATGGGAACTGAAAGATGGCAA

AATCACTGAGTATTGGGGAAACTATTCTGATTATCTTCGTCAGAAAGAGGAAGAACGTAAGAGCCAAGCTGC

AGAATACGAACAATTTATTGCGGAACGTGCCCGATTGGAAAGGGCTGCGGAGGAAAAGCGAAAACAGGCTCG

TAAAATAGAACAGAAGGCAAAAGGTTCTTCAAAGAAAAAAAGTACTGAAGACGGAGGGCGTTTAGCTCATCA

AAAATCAATAGGAAGTAAGGAAAAAAAGATGTATAATGCTGCTAAAACCCTAGAGCACAGGATTGCGGCCTT

AGGAAAAGTAGAAGCTCCGGAAGGCATTCGCAGAATTCGTTTCAGGCAAAGTAAAGCATTGGAGCTCCATAA

TCCATACCCTATAGTCGGTGCAGAAATTAATAAAGTATTTGGGGATAAGGCTCTGTTTGAAAATGCATCTTT

TCAAATTCCGTTAGGAGCAAAAGTGGCGTTAACTGGTGGTAATGGAATCGGAAAAACAACTTTAATCCAAAT

GATCTTAAACCATGAAGAAGGAATTTCTATTTCGCCTAAGGCAAAAATAGGTTACTTTGCACAGAATGGTTA

CAAGTACAACAGTAATCAGAATGTTATGGAGTTTATGCAGAAGGATTGTGACTACAATATATCAGAAATTCG

TTCAGTGCTAGCATCTATGGGGTTCAAACAGAACGATATTGGAAAAAGTTTATCTGTTTTAAGCGGTGGAGA

AATTATAAAATTGTTGCTTGCTAAAATGCTCATGGATAGATATAACATCCTAATAATGGATGAACCCAGTAA

CTTCCTTGACATACCAAGTTTAGAGGCTTTGGAAATACTAATGAAGGAGTACACCGGAACTATCGTGTTTAT

CACCCACGATAAACGATTACTCGAAAATGTAGCAGATGTAGTTTATGAAATTAGAGATAAGAAAATAAATCT

GAAACATTAAATTTAAGGTAGTCGCTGGTCAGTATAGTCTGTTCTGGTTGGCGACTCCATTGTTAAAGAGTA

TAAAGACTTTAGATTTTATGAATATTAAAAATAGGAACAGTCAATTGAACTGCTCCTATTTTTCTGCTAAAT

ATATTGTAGTTTTCTTATATGTATAATGATAGATTAGCGGATTCTCATCTACGGTACTTACTTCAAATATGA

AGAAGTGATCGCGGTTATCTCTGGACTTTTCCTTATTGAGGACAAAGTAATTCTTACGTGAAGTCGCCATTG

TTTTTAGGATATCATCAGTTAGGAAGGTCAATGGAATATTCATGTTAGAGTAGCGGTAGAAGTCACGTTCAA

AATCTTGGTAGCTCTCGCTATAATAGTCCATTTGTAGGTGATTACGCTGAAACTCAAGCTGATTCATAGAGC

ACCTCCTCGACAAGTTCAATACTAATAATGTCTTTTAATTTCAAATTGATGTGACCTGTTGTAGTTTTTATC

AAAATGAAATCTTTGGTCAGACTTGGTATTGTTCCAGTGTAGGAAACACGCTTGTTTTTTTCAATCACTTGA

ATGCGTGTGCGTAGCTGCCCGGCGTATACTTGACTGAGGAGTAATAATTTCTTCTCTAGTGATAAGTCAGAC

ATGTACGTTACTTTGTTTGTATCATCAGAGAGTGCTGATGCATGTTCAGATAGGAAAAAGCCCATCCATTTT

TGCATCTTTGTATCCTGGTACTCTCTTGCTGATTGAAATGGTAAATATGAACGGTCAATCATATCAAATCCT

TTCTATGCAGAGGCAAGGGTATTTTTATCAAATTGAATCGTAAAACCTTGAATTCCCCCACCTGTGTAACAT

TCTTTAAAGCGATTGATTACCTCAGTATAGATTATCACAGATGAGCTTGTTGGCTTAATGCTAAATGTAAAT

TCCAATGGTAATCGGTTTTCAGATTTAGCATGTACTAGTCGTATCGATATTTCAGTTGTTTTGAGTTTTCTC

TGACGAAGTTTTGAAGTTGCTGTTTCAACGATTCCATGTAAAAATCTTTCAAGCATTTCAATATCATTACAT

CCTTTGCTACGGATTTCTGAAAATTGTACTGTATTTTTTTCTTGTTTCATTTTAATCCCTCCAATCCACCCG

CGGAATGACCACCGATAAGTTTACTGCGTTCAATATTTCTGGAACCTTCAGTTAGGACGGTTCCTTTTTGTA

TGGCTAAAAAACCAAACTGTTCTCTGACAACATCAATAGCTGTCTGAAGTCTATTATCTTTTTCAATTTGTT

CTACATCATCAAAGAGTGATAGTAGAGTATAGCTTTCATCTACGAAGCCACTATAAGATACACCAATTTGTC

TCACTGCACCAGAGGTGTATTTTTTCGGAATAATACAAGTACATGACTCACCATTGTTTTGGGGAGATTTG

CGGGTTCAATTTTATTCTGAGCATTTATAGATTTTTTCATCTCAGTCCTAGAATAGCCAATATGAATAGAAA

CGACAGTAGTCAATACTAGGGCTACTGTTCCGTTCCACAGTATCATTTAAAAATCATT-TTCACACCCTTTC

GTCTATTAGTATAGAAGAAAGCTCTCAGCACA

>GA05578/Mega-2.II

CATGTTGAGGCGGTAAGTTTGCTAGTCAAGGAGTAAAACGACGAAGATTAGCATTTACTTCCGCCCATGCGA

TAGCTGTCCGTGATTGACAAGTGCTAGCACGCAGACAGAACGGAGATAGCGAACCGCTGAGTGTGTCGCTCT

GCTCGTAAAAGCTTAGAAACCTTTGAACGAAAGGGATAATGAAAGCCTTGATTGCAAGGCTTTTTGCTTTAT

GGTGGGTAAGTATCAGAGTGAGAAAATTTTTGGAATGAGTAGAAGTGATAGCTAGAAATTATCAGTTTCTA

TTTCCATTTACCCTGTGGGTACGTGTTTGTTTCCATTGACAAGGAGTTTGTGGGAATAGAAATGT

ACCCACCTTGTTTGAATCAAGTGAAGTGTAGTTGAAGGAAATCTGTTGAAAGCAATACTTCATTTTACCG

AATAAGTAATAATTTAGGCAACTTCAAATCGATTAAAAAAAACTATTTTAAAGGTTAAGAGTAGACAAAA

ATTGTCCACTCTTTTTTGCAAACTCAATTTATCAATAAATGAAATGAGGGAATGTAAAATG

AAATATTTTGAGGTTGAGTTAGAAAATCCTGATGAATTTTTAAAACTACAAACAGAAGATTTTGTGAAAG

CTAATCGCTTGCTACTAAGGAAGATAATCCAGAGCGTTACAGTCTATGAAGAAAACTTCGTCATATCCTT

TAAATCTGGCATCGAATTGGAAGTATGAGTCTCATTCCATAACTTTTATATTGAACATATCATCTTGTTGTG

TTATACTATAAATTGATATAAACAAAGATGTAGGAGGAACCGAAACTATGACAGCCTCAATGCGTTTAAGAT

AAGCTGGCAATAAAAAAAGCAGAATCTATACCCGATGATAGGCTTTTTTGTTGTGCTTATTTATACGATATT

GAGCATTCATTAGTTACGGTGAGGATATTGGTTATTTAACTATACCTTTATTTAACTATGTCTTTAATATGA

ATGTTTCCAAATTGTATGTATGCAGACCAAAAGCCACATTGTGGGGTTTGGCCTGCATTTTTTTTGCCTA

GAATGCTATTCAAAATAGAAATTCAAGCAAAATAATATGCAGGAGATAATATAAATGGAAAAATACAACAAT

TGGAAACGAAAATTTTATGCAATATGGGCAGGGCAAGCAGTATCATTAATCACTAGTGCCATCCTGCAAATG

GCGATTATTTTTTACCTTACAGAAAAAACAGGATCTGCGATGGTCTTGTCTATGGCTTCATTAGTAGGTTTT

TTACCCTATGCGATTTTGGGACCTGCCATTGGTGTGCTAGTGGATCGTCATGATAGGAAGAAGATAATGATT

GGTGCCGATTTAATTATCGCAGCAGCTGGTGCAGTGCTTGCTATTGTTGCATTCTGTATGGAGCTACCTGTC

TGGATGATTATGATAGTATTGTTTATCCGTAGCATTGGAACAGCTTTTCATACCCCAGCACTCAATGCGGTT

ACACCACTTTTAGTACCAGAAGAACAGCTAACGAAATGCGCAGGCTATAGTCAGTCTTTGCAGTCTATAAGC

TATATTGTTAGTCCGGCAGTTGCAGCACTCTTATACTCCGTTTGGGATTTAAATGCTATTATTGCCATCGAC

GTATTGGGTGCTGTGATTGCATCTATTACGGTAGCAATTGTACGTATACCTAAGCTGGGTAATCAAGTGCAA

AGTTTAGAACCAAATTTCATAAGGGAGATGAAAGAAGGAGTTGTGGTTCTGAGACAAAACAAAGGATTGTTT

GCCTTATTACTCTTAGGAACACTATATACTTTTGTTTATATGCCAATCAATGCACTATTTCCTTTAATAAGC

ATGGAACACTTTAATGGAACGCCTGTGCATATTTCTATTACGGAAATTTCCTTTGCATTTGGGATGCTAGCA

GGAGGCTTATTATTAGGAAGATTAGGGGGCTTCGAAAAGCATGTATTACTAATAACAAGTTCATTTTTTATA

ATGGGGACCAGTTTAGCCGTTTCGGGAATACTTCCTCCAAATGGATTTGTAATATTCGTAGTTTGCTGTGCA

ATAATGGGGCTTTCGGTGCCATTTTATAGCGGTGTGCAAACAGCTCTTTTTCAGGAGAAAATTAAGCCTGAA

TATTTAGGACGTGTATTTTCTTTGATCGGAAGTATCATGTCACTTGCTATGCCAATTGGGTTAATTCTTTCT

GGATTCTTTGCTGATAAAATCGGTGTAAATCATTGGTTTTTACTATCAGGTATTTTAATTATTGGCATTGCT

ATAGTTTGCCAAATGATAACTGAGGTTAGAAAATTAGATTTAAAATAAACAATATTGGAGGAA

ATGTTATGGAATTAATATT

AAAAGCAAAAGACATTCGTGTGGAATTCAAAGGACGCGATGTTTTAGATATAAATGAATTAGAAGTATATGA

TTATGACCGTATTGGTTTAGTAGGAGCAAATGGTGCTGGAAAAAGCACTTTACTCAGGGTACTTTTAGGAGA

ATTAACTCCCCCAGGATGTAAAATGAATCGTCTGGGTGAACTTGCCTATATTCCCCAGTTGGACGAAGTAAC

TCTGCAGGAGGAAAAAGATTTTGCACTTGTAGGCAAGCTAGGTGTTGAGCAATTAAATATACAGACTATGAG

CGGTGGTGAAGAAACAAGGCTTAAAATAGCACAGGCCTTATCGGCACAGGTTCATGGTATTTTAGCGGATGA

ACCTACGAGCCATTTAGACCGTGAAGGAATTGATTTTCTAATAGGACAGCTAAAATATTTTACAGGTGCACT

GTTAGTTATTAGCCATGACCGCTATTTTCTTGATGAAATAGTAGATAAAATATGGGAACTGAAAGATGGCAA

AATCACTGAGTATTGGGGAAACTATTCTGATTATCTTCGTCAGAAAGAGGAAGAACGTAAGAGCCAAGCTGC

AGAATACGAACAATTTATTGCGGAACGTGCCCGATTGGAAAGGGCTGCGGAGGAAAAGCGAAAACAGGCTCG

TAAAATAGAACAGAAGGCAAAAGGTTCTTCAAAGAAAAAAAGTACTGAAGACGGAGGGCGTTTAGCTCATCA

AAAATCAATAGGAAGTAAGGAAAAAAAGATGTATAATGCTGCTAAAACCCTAGAGCACAGGATTGCGGCCTT

AGGAAAAGTAGAAGCTCCGGAAGGCATTCGCAGAATTCGTTTCAGGCAAAGTAAAGCATTGGAGCTCCATAA

TCCATACCCTATAGTCGGTGCAGAAATTAATAAAGTATTTGGGGATAAGGCTCTGTTTGAAAATGCATCTTT

TCAAATTCCGTTAGGAGCAAAAGTGGCGTTAACTGGTGGTAATGGAATCGGAAAAACAACTTTAATCCAAAT

GATCTTAAACCATGAAGAAGGAATTTCTATTTCGCCTAAGGCAAAAATAGGTTACTTTGCACAGAATGGTTA

CAAGTACAACAGTAATCAGAATGTTATGGAGTTTATGCAGAAGGATTGTGACTACAATATATCAGAAATTCG

TTCAGTGCTAGCATCTATGGGGTTCAAACAGAACGATATTGGAAAAAGTTTATCTGTTTTAAGCGGTGGAGA

AATTATAAAATTGTTGCTTGCTAAAATGCTCATGGGTAGATATAACATCCTAATAATGGATGAACCCAGTAA

CTTCCTTGACATACCAAGTTTAGAGGCTTTGGAAATACTAATGAAGGAGTACACCGGAACTATCGTGTTTAT

CACCCACGATAAACGATTACTCGAAAATGTAGCAGATGTAGTTTATGAAATTAGAGATAAGAAAATAAATCT

GAAACATTAAATTTAAGGTAGTCGCTGGTCAGTATAGTCTGTTCTGGTTGGCGACTCCATTGTTAAAGAGTA

TAAAGACTTTAGATTTTATGAATATTAAAAATAGGAACAGTCAATTGAACTGCTCCTATTTTTCTGCTAAAT

ATATTGTAGTTTTCTTATATGTATAATGATAGATTAGCGGATTCTCATCTACGGTACTTACTTCAAATATGA

AGAAGTGATCGCGGTTATCTCTGGACTTTTCCTTATTGAGGACAAAGTAATTCTTACGTGAAGTCGCCATTG

TTTTTAGGATATCATCAGTTAGGAAGGTCAATGGAATATTCATGTTAGAGTAGCGGTAGAAGTCACGTTCAA

AATCTTGGTAGCTCTCGCTATAATAGTCCATTTGTAGGTGATTACGCTGAAACTCAAGCTGATTCATAGAGC

ACCTCCTCGACAAGTTCAATACTAATAATGTCTTTTAATTTCAAATTGATGTGACCTGTTGTAGTTTTTATC

AAAATGAAATCTTTGGTCAGACTTGGTATTGTTCCAGTGTAGGAAACACGCTTGTTTTTTTCAATCACTTGA

ATGCGTGTGCGTAGCTGCCCGGCGTATACTTGACTGAGGAGTAATAATTTCTTCTCTAGTGATAAGTCAGAC

ATGTACGTTACTTTGTTTGTATCATCAGAGAGTGCTGATGCATGTTCAGATAGGAAAAAGCCCATCCATTTT

TGCATCTTTGTATCCTGGTACTCTCTTGCTGATTGAAATGGTAAATATGAACGGTCAATCATATCAAATCCT

TTCTATGCAGAGGCAAGGGTATTTTTATCAAATTGAATCGTAAAACCTTGAATTCCCCCACCTGTGTAACAT

TCTTTAAAGCGATTGATTACCTCAGTATAGATTATCACAGATGAGCTTGTTGGCTTAATGCTAAATGTAAAT

TCCAATGGTAATCGGTTTTCAGATTTAGCATGTACTAGTCGTATCGATATTTCAGTTGTTTTGAGTTTTCTC

TGACGAAGTTTTGAAGTTGCTGTTTCAACGATTCCATGTAAAAATCTTTCAAGCATTTCAATATCATTACAT

CCTTTGCTACGGATTTCTGAAAATTGTACTGTATTTTTTTCTTGTTTCATTTTAATCCCTCCAATCCACCCG

CGGAATGACCACCGATAAGTTTACTGCGTTCAATATTTCTGGAACCTTCAGTTAGGACGGTTCCTTTTTGTA

TGGCTAAAAAACCAAACTGTTCTCTGACAACATCAATAGCTGTCTGAAGTCTATTATCTTTTTCAATTTGTT

CTACATCATCAAAGAGTGATAGTAGAGTATAGCTTTCATCTACGAAGCCACTATAAGATACACCAATTTGTC

TCACTGCACCAGAGGTGTATTTTTTCGGAATAATACAAGTACATGACTCACCATTGTTTTGGGGAGATTTG

CGGGTTCAATTTTATTCTGAGCATTTATAGATTTTTTCATCTCAGTCCTAGAATAGCCAATATGAATAGAAA

CGACAGTAGTCAATACTAGGGCTACTGTTCCGTTCCACAGTATCATTTAAAAATCATT-TTCACACCCTTTC

GTCTATTAGTATAGAAGAAAGCTCTCAGCACA

>GA04216/Mega-2.II

CATGTTGAGGCGGTAAGTTTGCTAGTCAAGGAGTAAAACGACGAAGATTAGCATTTACTTCCGCCCATGCGA

TAGCTGTCCGTGATTGACAAGTGCTAGCACGCAGACAGAACGGAGATAGCGAACCGCTGAGTGTGTCGCTCT

GCTCGTAAAAGCTTAGAAACCTTTGAACGAAAGGGATAATGAAAGCCTTGATTGCAAGGCTTTTTGCTTTAT

GGTGGGTAAGTATCAGAGTGAGAAAATTTTTGGAATGAGTAGAAGTGATAGCTAGAAATTATCAGTTTCTA

TTTCCATTTACCCTGTGGGTACGTGTTTGTTTCCATTGACAAGGAGTTTGTGGGAATAGAAATGT

ACCCACCTTGTTTGAATCAAGTGAAGTGTAGTTGAAGGAAATCTGTTGAAAGCAATACTTCATTTTACCG

AATAAGTAATAATTTAGGCAACTTCAAATCGATTAAAAAAAACTATTTTAAAGGTTAAGAGTAGACAAAA

ATTGTCCACTCTTTTTTGCAAACTCAATTTATCAATAAATGAAATGAGGGAATGTAAAATG

AAATATTTTGAGGTTGAGTTAGAAAATCCTGATGAATTTTTAAAACTACAAACAGAAGATTTTGTGAAAG

CTAATCGCTTGCTACTAAGGAAGATAATCCAGAGCGTTACAGTCTATGAAGAAAACTTCGTCATATCCTT

TAAATCTGGCATCGAATTGGAAGTATGAGTCTCATTCCATAACTTTTATATTGAACATATCATCTTGTTGTG

TTATACTATAAATTGATATAAACAAAGATGTAGGAGGAACCGAAACTATGACAGCCTCAATGCGTTTAAGAT

AAGCTGGCAATAAAAAAAGCAGAATCTATACCCGATGATAGGCTTTTTTGTTGTGCTTATTTATACGATATT

GAGCATTCATTAGTTACGGTGAGGATATTGGTTATTTAACTATACCTTTATTTAACTATGTCTTTAATATGA

ATGTTTCCAAATTGTATGTATGCAGACCAAAAGCCACATTGTGGGGTTTGGCCTGCATTTTTTTTGCCTA

GAATGCTATTCAAAATAGAAATTCAAGCAAAATAATATGCAGGAGATAATATAAATGGAAAAATACAACAAT

TGGAAACGAAAATTTTATGCAATATGGGCAGGGCAAGCAGTATCATTAATCACTAGTGCCATCCTGCAAATG

GCGATTATTTTTTACCTTACAGAAAAAACAGGATCTGCGATGGTCTTGTCTATGGCTTCATTAGTAGGTTTT

TTACCCTATGCGATTTTGGGACCTGCCATTGGTGTGCTAGTGGATCGTCATGATAGGAAGAAGATAATGATT

GGTGCCGATTTAATTATCGCAGCAGCTGGTGCAGTGCTTGCTATTGTTGCATTCTGTATGGAGCTACCTGTC

TGGATGATTATGATAGTATTGTTTATCCGTAGCATTGGAACAGCTTTTCATACCCCAGCACTCAATGCGGTT

ACACCACTTTTAGTACCAGAAGAACAGCTAACGAAATGCGCAGGCTATAGTCAGTCTTTGCAGTCTATAAGC

TATATTGTTAGTCCGGCAGTTGCAGCACTCTTATACTCCGTTTGGGATTTAAATGCTATTATTGCCATCGAC

GTATTGGGTGCTGTGATTGCATCTATTACGGTAGCAATTGTACGTATACCTAAGCTGGGTAATCAAGTGCAA

AGTTTAGAACCAAATTTCATAAGGGAGATGAAAGAAGGAGTTGTGGTTCTGAGACAAAACAAAGGATTGTTT

GCCTTATTACTCTTAGGAACACTATATACTTTTGTTTATATGCCAATCAATGCACTATTTCCTTTAATAAGC

ATGGAACACTTTAATGGAACGCCTGTGCATATTTCTATTACGGAAATTTCCTTTGCATTTGGGATGCTAGCA

GGAGGCTTATTATTAGGAAGATTAGGGGGCTTCGAAAAGCATGTATTACTAATAACAAGTTCATTTTTTATA

ATGGGGACCAGTTTAGCCGTTTCGGGAATACTTCCTCCAAATGGATTTGTAATATTCGTAGTTTGCTGTGCA

ATAATGGGGCTTTCGGTGCCATTTTATAGCGGTGTGCAAACAGCTCTTTTTCAGGAGAAAATTAAGCCTGAA

TATTTAGGACGTGTATTTTCTTTGATCGGAAGTATCATGTCACTTGCTATGCCAATTGGGTTAATTCTTTCT

GGATTCTTTGCTGATAAAATCGGTGTAAATCATTGGTTTTTACTATCAGGTATTTTAATTATTGGCATTGCT

ATAGTTTGCCAAATGATAACTGAGGTTAGAAAATTAGATTTAAAATAAACAATATTGGAGGAA

ATGTTATGGAATTAATATT

AAAAGCAAAAGACATTCGTGTGGAATTCAAAGGACGCGATGTTTTAGATATAAATGAATTAGAAGTATATGA

TTATGACCGTATTGGTTTAGTAGGAGCAAATGGTGCTGGAAAAAGCACTTTACTCAGGGTACTTTTAGGAGA

ATTAACTCCCCCAGGATGTAAAATGAATCGTCTGGGTGAACTTGCCTATATTCCCCAGTTGGACGAAGTAAC

TCTGCAGGAGGAAAAAGATTTTGCACTTGTAGGCAAGCTAGGTGTTGAGCAATTAAATATACAGACTATGAG

CGGTGGTGAAGAAACAAGGCTTAAAATAGCACAGGCCTTATCGGCACAGGTTCATGGTATTTTAGCGGATGA

ACCTACGAGCCATTTAGACCGTGAAGGAATTGATTTTCTAATAGGACAGCTAAAATATTTTACAGGTGCACT

GTTAGTTATTAGCCATGACCGCTATTTTCTTGATGAAATAGTAGATAAAATATGGGAACTGAAAGATGGCAA

AATCACTGAGTATTGGGGAAACTATTCTGATTATCTTCGTCAGAAAGAGGAAGAACGTAAGAGCCAAGCTGC

AGAATACGAACAATTTATTGCGGAACGTGCCCGATTGGAAAGGGCTGCGGAGGAAAAGCGAAAACAGGCTCG

TAAAATAGAACAGAAGGCAAAAGGTTCTTCAAAGAAAAAAAGTACTGAAGACGGAGGGCGTTTAGCTCATCA

AAAATCAATAGGAAGTAAGGAAAAAAAGATGTATAATGCTGCTAAAACCCTAGAGCACAGGATTGCGGCCTT

AGGAAAAGTAGAAGCTCCGGAAGGCATTCGCAGAATTCGTTTCAGGCAAAGTAAAGCATTGGAGCTCCATAA

TCCATACCCTATAGTCGGTGCAGAAATTAATAAAGTATTTGGGGATAAGGCTCTGTTTGAAAATGCATCTTT

TCAAATTCCGTTAGGAGCAAAAGTGGCGTTAACTGGTGGTAATGGAATCGGAAAAACAACTTTAATCCAAAT

GATCTTAAACCATGAAGAAGGAATTTCTATTTCGCCTAAGGCAAAAATAGGTTACTTTGCACAGAATGGTTA

CAAGTACAACAGTAATCAGAATGTTATGGAGTTTATGCAGAAGGATTGTGACTACAATATATCAGAAATTCG

TTCAGTGCTAGCATCTATGGGGTTCAAACAGAACGATATTGGAAAAAGTTTATCTGTTTTAAGCGGTGGAGA

AATTATAAAATTGTTGCTTGCTAAAATGCTCATGGGTAGATATAACATCCTAATAATGGATGAACCCAGTAA

CTTCCTTGACATACCAAGTTTAGAGGCTTTGGAAATACTAATGAAGGAGTACACCGGAACTATCGTGTTTAT

CACCCACGATAAACGATTACTCGAAAATGTAGCAGATGTAGTTTATGAAATTAGAGATAAGAAAATAAATCT

GAAACATTAAATTTAAGGTAGTCGCTGGTCAGTATAGTCTGTTCTGGTTGGCGACTCCATTGTTAAAGAGTA

TAAAGACTTTAGATTTTATGAATATTAAAAATAGGAACAGTCAATTGAACTGCTCCTATTTTTCTGCTAAAT

ATATTGTAGTTTTCTTATATGTATAATGATAGATTAGCGGATTCTCATCTACGGTACTTACTTCAAATATGA

AGAAGTGATCGCGGTTATCTCTGGACTTTTCCTTATTGAGGACAAAGTAATTCTTACGTGAAGTCGCCATTG

TTTTTAGGATATCATCAGTTAGGAAGGTCAATGGAATATTCATGTTAGAGTAGCGGTAGAAGTCACGTTCAA

AATCTTGGTAGCTCTCGCTATAATAGTCCATTTGTAGGTGATTACGCTGAAACTCAAGCTGATTCATAGAGC

ACCTCCTCGACAAGTTCAATACTAATAATGTCTTTTAATTTCAAATTGATGTGACCTGTTGTAGTTTTTATC

AAAATGAAATCTTTGGTCAGACTTGGTATTGTTCCAGTGTAGGAAACACGCTTGTTTTTTTCAATCACTTGA

ATGCGTGTGCGTAGCTGCCCGGCGTATACTTGACTGAGGAGTAATAATTTCTTCTCTAGTGATAAGTCAGAC

ATGTACGTTACTTTGTTTGTATCATCAGAGAGTGCTGATGCATGTTCAGATAGGAAAAAGCCCATCCATTTT

TGCATCTTTGTATCCTGGTACTCTCTTGCTGATTGAAATGGTAAATATGAACGGTCAATCATATCAAATCCT

TTCTATGCAGAGGCAAGGGTATTTTTATCAAATTGAATCGTAAAACCTTGAATTCCCCCACCTGTGTAACAT

TCTTTAAAGCGATTGATTACCTCAGTATAGATTATCACAGATGAGCTTGTTGGCTTAATGCTAAATGTAAAT

TCCAATGGTAATCGGTTTTCAGATTTAGCATGTACTAGTCGTATCGATATTTCAGTTGTTTTGAGTTTTCTC

TGACGAAGTTTTGAAGTTGCTGTTTCAACGATTCCATGTAAAAATCTTTCAAGCATTTCAATATCATTACAT

CCTTTGCTACGGATTTCTGAAAATTGTACTGTATTTTTTTCTTGTTTCATTTTAATCCCTCCAATCCACCCG

CGGAATGACCACCGATAAGTTTACTGCGTTCAATATTTCTGGAACCTTCAGTTAGGACGGTTCCTTTTTGTA

TGGCTAAAAAACCAAACTGTTCTCTGACAACATCAATAGCTGTCTGAAGTCTATTATCTTTTTCAATTTGTT

CTACATCATCAAAGAGTGATAGTAGAGTATAGCTTTCATCTACGAAGCCACTATAAGATACACCAATTTGTC

TCACTGCACCAGAGGTGTATTTTTTCGGAATAATACAAGTACATGACTCACCATTGTTTTGGGGAGATTTG

CGGGTTCAATTTTATTCTGAGCATTTATAGATTTTTTCATCTCAGTCCTAGAATAGCCAATATGAATAGAAA

CGACAGTAGTCAATACTAGGGCTACTGTTCCGTTCCACAGTATCATTTAAAAATCATT-TTCACACCCTTTC

GTCTATTAGTATAGAAGAAAGCTCTCAGCACA

>GA02506/Mega-2.II

CATGTTGAGGCGGTAAGTTTGCTAGTCAAGGAGTAAAACGACGAAGATTAGCATTTACTTCCGCCCATGCGA

TAGCTGTCCGTGATTGACAAGTGCTAGCACGCAGACAGAACGGAGATAGCGAACCGCTGAGTGTGTCGCTCT

GCTCGTAAAAGCTTAGAAACCTTTGAACGAAAGGGATAATGAAAGCCTTGATTGCAAGGCTTTTTGCTTTAT

GGTGGGTAAGTATCAGAGTGAGAAAATTTTTGGAATGAGTAGAAGTGATAGCTAGAAATTATCAGTTTCTA

TTTCCATTTACCCTGTGGGTACGTGTTTGTTTCCATTGACAAGGAGTTTGTGGGAATAGAAATGT

ACCCACCTTGTTTGAATCAAGTGAAGTGTAGTTGAAGGAAATCTGTTGAAAGCAATACTTCATTTTACCG

AATAAGTAATAATTTAGGCAACTTCAAATCGATTAAAAAAAACTATTTTAAAGGTTAAGAGTAGACAAAA

ATTGTCCACTCTTTTTTGCAAACTCAATTTATCAATAAATGAAATGAGGGAATGTAAAATG

AAATATTTTGAGGTTGAGTTAGAAAATCCTGATGAATTTTTAAAACTACAAACAGAAGATTTTGTGAAAG

CTAATCGCTTGCTACTAAGGAAGATAATCCAGAGCGTTACAGTCTATGAAGAAAACTTCGTCATATCCTT

TAAATCTGGCATCGAATTGGAAGTATGAGTCTCATTCCATAACTTTTATATTGAACATATCATCTTGTTGTG

TTATACTATAAATTGATATAAACAAAGATGTAGGAGGAACCGAAACTATGACAGCCTCAATGCGTTTAAGAT

AAGCTGGCAATAAAAAAAGCAGAATCTATACCCGATGATAGGCTTTTTTGTTGTGCTTATTTATACGATATT

GAGCATTCATTAGTTACGGTGAGGATATTGGTTATTTAACTATACCTTTATTTAACTATGTCTTTAATATGA

ATGTTTCCAAATTGTATGTATGCAGACCAAAAGCCACATTGTGGGGTTTGGCCTGCATTTTTTTTGCCTA

GAATGCTATTCAAAATAGAAATTCAAGCAAAATAATATGCAGGAGATAATATAAATGGAAAAATACAACAAT

TGGAAACGAAAATTTTATGCAATATGGGCAGGGCAAGCAGTATCATTAATCACTAGTGCCATCCTGCAAATG

GCGATTATTTTTTACCTTACAGAAAAAACAGGATCTGCGATGGTCTTGTCTATGGCTTCATTAGTAGGTTTT

TTACCCTATGCGATTTTGGGACCTGCCATTGGTGTGCTAGTGGATCGTCATGATAGGAAGAAGATAATGATT

GGTGCCGATTTAATTATCGCAGCAGCTGGTGCAGTGCTTGCTATTGTTGCATTCTGTATGGAGCTACCTGTC

TGGATGATTATGATAGTATTGTTTATCCGTAGCATTGGAACAGCTTTTCATACCCCAGCACTCAATGCGGTT

ACACCACTTTTAGTACCAGAAGAACAGCTAACGAAATGCGCAGGCTATAGTCAGTCTTTGCAGTCTATAAGC

TATATTGTTAGTCCGGCAGTTGCAGCACTCTTATACTCCGTTTGGGATTTAAATGCTATTATTGCCATCGAC

GTATTGGGTGCTGTGATTGCATCTATTACGGTAGCAATTGTACGTATACCTAAGCTGGGTAATCAAGTGCAA

AGTTTAGAACCAAATTTCATAAGGGAGATGAAAGAAGGAGTTGTGGTTCTGAGACAAAACAAAGGATTGTTT

GCCTTATTACTCTTAGGAACACTATATACTTTTGTTTATATGCCAATCAATGCACTATTTCCTTTAATAAGC

ATGGAACACTTTAATGGAACGCCTGTGCATATTTCTATTACGGAAATTTCCTTTGCATTTGGGATGCTAGCA

GGAGGCTTATTATTAGGAAGATTAGGGGGCTTCGAAAAGCATGTATTACTAATAACAAGTTCATTTTTTATA

ATGGGGACCAGTTTAGCCGTTTCGGGAATACTTCCTCCAAATGGATTTGTAATATTCGTAGTTTGCTGTGCA

ATAATGGGGCTTTCGGTGCCATTTTATAGCGGTGTGCAAACAGCTCTTTTTCAGGAGAAAATTAAGCCTGAA

TATTTAGGACGTGTATTTTCTTTGATCGGAAGTATCATGTCACTTGCTATGCCAATTGGGTTAATTCTTTCT

GGATTCTTTGCTGATAAAATCGGTGTAAATCATTGGTTTTTACTATCAGGTATTTTAATTATTGGCATTGCT

ATAGTTTGCCAAATGATAACTGAGGTTAGAAAATTAGATTTAAAATAAACAATATTGGAGGAA

ATGTTATGGAATTAATATT

AAAAGCAAAAGACATTCGTGTGGAATTCAAAGGACGCGATGTTTTAGATATAAATGAATTAGAAGTATATGA

TTATGACCGTATTGGTTTAGTAGGAGCAAATGGTGCTGGAAAAAGCACTTTACTCAGGGTACTTTTAGGAGA

ATTAACTCCCCCAGGATGTAAAATGAATCGTCTGGGTGAACTTGCCTATATTCCCCAGTTGGACGAAGTAAC

TCTGCAGGAGGAAAAAGATTTTGCACTTGTAGGCAAGCTAGGTGTTGAGCAATTAAATATACAGACTATGAG

CGGTGGTGAAGAAACAAGGCTTAAAATAGCACAGGCCTTATCGGCACAGGTTCATGGTATTTTAGCGGATGA

ACCTACGAGCCATTTAGACCGTGAAGGAATTGATTTTCTAATAGGACAGCTAAAATATTTTACAGGTGCACT

GTTAGTTATTAGCCATGACCGCTATTTTCTTGATGAAATAGTAGATAAAATATGGGAACTGAAAGATGGCAA

AATCACTGAGTATTGGGGAAACTATTCTGATTATCTTCGTCAGAAAGAGGAAGAACGTAAGAGCCAAGCTGC

AGAATACGAACAATTTATTGCGGAACGTGCCCGATTGGAAAGGGCTGCGGAGGAAAAGCGAAAACAGGCTCG

TAAAATAGAACAGAAGGCAAAAGGTTCTTCAAAGAAAAAAAGTACTGAAGACGGAGGGCGTTTAGCTCATCA

AAAATCAATAGGAAGTAAGGAAAAAAAGATGTATAATGCTGCTAAAACCCTAGAGCACAGGATTGCGGCCTT

AGGAAAAGTAGAAGCTCCGGAAGGCATTCGCAGAATTCGTTTCAGGCAAAGTAAAGCATTGGAGCTCCATAA

TCCATACCCTATAGTCGGTGCAGAAATTAATAAAGTATTTGGGGATAAGGCTCTGTTTGAAAATGCATCTTT

TCAAATTCCGTTAGGAGCAAAAGTGGCGTTAACTGGTGGTAATGGAATCGGAAAAACAACTTTAATCCAAAT

GATCTTAAACCATGAAGAAGGAATTTCTATTTCGCCTAAGGCAAAAATAGGTTACTTTGCACAGAATGGTTA

CAAGTACAACAGTAATCAGAATGTTATGGAGTTTATGCAGAAGGATTGTGACTACAATATATCAGAAATTCG

TTCAGTGCTAGCATCTATGGGGTTCAAACAGAACGATATTGGAAAAAGTTTATCTGTTTTAAGCGGTGGAGA

AATTATAAAATTGTTGCTTGCTAAAATGCTCATGGGTAGATATAACATCCTAATAATGGATGAACCCAGTAA

CTTCCTTGACATACCAAGTTTAGAGGCTTTGGAAATACTAATGAAGGAGTACACCGGAACTATCGTGTTTAT

CACCCACGATAAACGATTACTCGAAAATGTAGCAGATGTAGTTTATGAAATTAGAGATAAGAAAATAAATCT

GAAACATTAAATTTAAGGTAGTCGCTGGTCAGTATAGTCTGTTCTGGTTGGCGACTCCATTGTTAAAGAGTA

TAAAGACTTTAGATTTTATGAATATTAAAAATAGGAACAGTCAATTGAACTGCTCCTATTTTTCTGCTAAAT

ATATTGTAGTTTTCTTATATGTATAATGATAGATTAGCGGATTCTCATCTACGGTACTTACTTCAAATATGA

AGAAGTGATCGCGGTTATCTCTGGACTTTTCCTTATTGAGGACAAAGTAATTCTTACGTGAAGTCGCCATTG

TTTTTAGGATATCATCAGTTAGGAAGGTCAATGGAATATTCATGTTAGAGTAGCGGTAGAAGTCACGTTCAA

AATCTTGGTAGCTCTCGCTATAATAGTCCATTTGTAGGTGATTACGCTGAAACTCAAGCTGATTCATAGAGC

ACCTCCTCGACAAGTTCAATACTAATAATGTCTTTTAATTTCAAATTGATGTGACCTGTTGTAGTTTTTATC

AAAATGAAATCTTTGGTCAGACTTGGTATTGTTCCAGTGTAGGAAACACGCTTGTTTTTTTCAATCACTTGA

ATGCGTGTGCGTAGCTGCCCGGCGTATACTTGACTGAGGAGTAATAATTTCTTCTCTAGTGATAAGTCAGAC

ATGTACGTTACTTTGTTTGTATCATCAGAGAGTGCTGATGCATGTTCAGATAGGAAAAAGCCCATCCATTTT

TGCATCTTTGTATCCTGGTACTCTCTTGCTGATTGAAATGGTAAATATGAACGGTCAATCATATCAAATCCT

TTCTATGCAGAGGCAAGGGTATTTTTATCAAATTGAATCGTAAAACCTTGAATTCCCCCACCTGTGTAACAT

TCTTTAAAGCGATTGATTACCTCAGTATAGATTATCACAGATGAGCTTGTTGGCTTAATGCTAAATGTAAAT

TCCAATGGTAATCGGTTTTCAGATTTAGCATGTACTAGTCGTATCGATATTTCAGTTGTTTTGAGTTTTCTC

TGACGAAGTTTTGAAGTTGCTGTTTCAACGATTCCATGTAAAAATCTTTCAAGCATTTCAATATCATTACAT

CCTTTGCTACGGATTTCTGAAAATTGTACTGTATTTTTTTCTTGTTTCATTTTAATCCCTCCAATCCACCCG

CGGAATGACCACCGATAAGTTTACTGCGTTCAATATTTCTGGAACCTTCAGTTAGGACGGTTCCTTTTTGTA

TGGCTAAAAAACCAAACTGTTCTCTGACAACATCAATAGCTGTCTGAAGTCTATTATCTTTTTCAATTTGTT

CTACATCATCAAAGAGTGATAGTAGAGTATAGCTTTCATCTACGAAGCCACTATAAGATACACCAATTTGTC

TCACTGCACCAGAGGTGTATTTTTTCGGAATAATACAAGTACATGACTCACCATTGTTTTGGGGAGATTTG

CGGGTTCAATTTTATTCTGAGCATTTATAGATTTTTTCATCTCAGTCCTAGAATAGCCAATATGAATAGAAA

CGACAGTAGTCAATACTAGGGCTACTGTTCCGTTCCACAGTATCATTTAAAAATCATT-TTCACACCCTTTC

GTCTATTAGTATAGAAGAAAGCTCTCAGCACA

>GA41538/Mega-2.II

CATGTTGAGGCGGTAAGTTTGCTAGTCAAGGAGTAAAACGACGAAGATTAGCATTTACTTCCGCCCATGCGA

TAGCTGTCCGTGATTGACAAGTGCTAGCACGCAGACAGAACGGAGATAGCGAACCGCTGAGTGTGTCGCTCT

GCTCGTAAAAGCTTAGAAACCTTTGAACGAAAGGGATAATGAAAGCCTTGATTGCAAGGCTTTTTGCTTTAT

GGTGGGTAAGTATCAGAGTGAGAAAATTTTTGGAATGAGTAGAAGTGATAGCTAGAAATTATCAGTTTCTA

TTTCCATTTACCCTGTGGGTACGTGTTTGTTTCCATTGACAAGGAGTTTGTGGGAATAGAAATGT

ACCCACCTTGTTTGAATCAAGTGAAGTGTAGTTGAAGGAAATCTGTTGAAAGCAATACTTCATTTTACCG

AATAAGTAATAATTTAGGCAACTTCAAATCGATTAAAAAAAACTATTTTAAAGGTTAAGAGTAGACAAAA

ATTGTCCACTCTTTTTTGCAAACTCAATTTATCAATAAATGAAATGAGGGAATGTAAAATG

AAATATTTTGAGGTTGAGTTAGAAAATCCTGATGAATTTTTAAAACTACAAACAGAAGATTTTGTGAAAG

CTAATCGCTTGCTACTAAGGAAGATAATCCAGAGCGTTACAGTCTATGAAGAAAACTTCGTCATATCCTT

TAAATCTGGCATCGAATTGGAAGTATGAGTCTCATTCCATAACTTTTATATTGAACATATCATCTTGTTGTG

TTATACTATAAATTGATATAAACAAAGATGTAGGAGGAACCGAAACTATGACAGCCTCAATGCGTTTAAGAT

AAGCTGGCAATAAAAAAAGCAGAATCTATACCCGATGATAGGCTTTTTTGTTGTGCTTATTTATACGATATT

GAGCATTCATTAGTTACGGTGAGGATATTGGTTATTTAACTATACCTTTATTTAACTATGTCTTTAATATGA

ATGTTTCCAAATTGTATGTATGCAGACCAAAAGCCACATTGTGGGGTTTGGCCTGCATTTTTTTTGCCTA

GAATGCTATTCAAAATAGAAATTCAAGCAAAATAATATGCAGGAGATAATATAAATGGAAAAATACAACAAT

TGGAAACGAAAATTTTATGCAATATGGGCAGGGCAAGCAGTATCATTAATCACTAGTGCCATCCTGCAAATG

GCGATTATTTTTTACCTTACAGAAAAAACAGGATCTGCGATGGTCTTGTCTATGGCTTCATTAGTAGGTTTT

TTACCCTATGCGATTTTGGGACCTGCCATTGGTGTGCTAGTGGATCGTCATGATAGGAAGAAGATAATGATT

GGTGCCGATTTAATTATCGCAGCAGCTGGTGCAGTGCTTGCTATTGTTGCATTCTGTATGGAGCTACCTGTC

TGGATGATTATGATAGTATTGTTTATCCGTAGCATTGGAACAGCTTTTCATACCCCAGCACTCAATGCGGTT

ACACCACTTTTAGTACCAGAAGAACAGCTAACGAAATGCGCAGGCTATAGTCAGTCTTTGCAGTCTATAAGC

TATATTGTTAGTCCGGCAGTTGCAGCACTCTTATACTCCGTTTGGGATTTAAATGCTATTATTGCCATCGAC

GTATTGGGTGCTGTGATTGCATCTATTACGGTAGCAATTGTACGTATACCTAAGCTGGGTAATCAAGTGCAA

AGTTTAGAACCAAATTTCATAAGGGAGATGAAAGAAGGAGTTGTGGTTCTGAGACAAAACAAAGGATTGTTT

GCCTTATTACTCTTAGGAACACTATATACTTTTGTTTATATGCCAATCAATGCACTATTTCCTTTAATAAGC

ATGGAACACTTTAATGGAACGCCTGTGCATATTTCTATTACGGAAATTTCCTTTGCATTTGGGATGCTAGCA

GGAGGCTTATTATTAGGAAGATTAGGGGGCTTCGAAAAGCATGTATTACTAATAACAAGTTCATTTTTTATA

ATGGGGACCAGTTTAGCCGTTTCGGGAATACTTCCTCCAAATGGATTTGTAATATTCGTAGTTTGCTGTGCA

ATAATGGGGCTTTCGGTGCCATTTTATAGCGGTGTGCAAACAGCTCTTTTTCAGGAGAAAATTAAGCCTGAA

TATTTAGGACGTGTATTTTCTTTGATCGGAAGTATCATGTCACTTGCTATGCCAATTGGGTTAATTCTTTCT

GGATTCTTTGCTGATAAAATCGGTGTAAATCATTGGTTTTTACTATCAGGTATTTTAATTATTGGCATTGCT

ATAGTTTGCCAAATGATAACTGAGGTTAGAAAATTAGATTTAAAATAAACAATATTGGAGGAA

ATGTTATGGAATTAATATT

AAAAGCAAAAGACATTCGTGTGGAATTCAAAGGACGCGATGTTTTAGATATAAATGAATTAGAAGTATATGA

TTATGACCGTATTGGTTTAGTAGGAGCAAATGGTGCTGGAAAAAGCACTTTACTCAGGGTACTTTTAGGAGA

ATTAACTCCCCCAGGATGTAAAATGAATCGTCTGGGTGAACTTGCCTATATTCCCCAGTTGGACGAAGTAAC

TCTGCAGGAGGAAAAAGATTTTGCACTTGTAGGCAAGCTAGGTGTTGAGCAATTAAATATACAGACTATGAG

CGGTGGTGAAGAAACAAGGCTTAAAATAGCACAGGCCTTATCGGCACAGGTTCATGGTATTTTAGCGGATGA

ACCTACGAGCCATTTAGACCGTGAAGGAATTGATTTTCTAATAGGACAGCTAAAATATTTTACAGGTGCACT

GTTAGTTATTAGCCATGACCGCTATTTTCTTGATGAAATAGTAGATAAAATATGGGAACTGAAAGATGGCAA

AATCACTGAGTATTGGGGAAACTATTCTGATTATCTTCGTCAGAAAGAGGAAGAACGTAAGAGCCAAGCTGC

AGAATACGAACAATTTATTGCGGAACGTGCCCGATTGGAAAGGGCTGCGGAGGAAAAGCGAAAACAGGCTCG

TAAAATAGAACAGAAGGCAAAAGGTTCTTCAAAGAAAAAAAGTACTGAAGACGGAGGGCGTTTAGCTCATCA

AAAATCAATAGGAAGTAAGGAAAAAAAGATGTATAATGCTGCTAAAACCCTAGAGCACAGGATTGCGGCCTT

AGGAAAAGTAGAAGCTCCGGAAGGCATTCGCAGAATTCGTTTCAGGCAAAGTAAAGCATTGGAGCTCCATAA

TCCATACCCTATAGTCGGTGCAGAAATTAATAAAGTATTTGGGGATAAGGCTCTGTTTGAAAATGCATCTTT

TCAAATTCCGTTAGGAGCAAAAGTGGCGTTAACTGGTGGTAATGGAATCGGAAAAACAACTTTAATCCAAAT

GATCTTAAACCATGAAGAAGGAATTTCTATTTCGCCTAAGGCAAAAATAGGTTACTTTGCACAGAATGGTTA

CAAGTACAACAGTAATCAGAATGTTATGGAGTTTATGCAGAAGGATTGTGACTACAATATATCAGAAATTCG

TTCAGTGCTAGCATCTATGGGGTTCAAACAGAACGATATTGGAAAAAGTTTATCTGTTTTAAGCGGTGGAGA

AATTATAAAATTGTTGCTTGCTAAAATGCTCATGGGTAGATATAACATCCTAATAATGGATGAACCCAGTAA

CTTCCTTGACATACCAAGTTTAGAGGCTTTGGAAATACTAATGAAGGAGTACACCGGAACTATCGTGTTTAT

CACCCACGATAAACGATTACTCGAAAATGTAGCAGATGTAGTTTATGAAATTAGAGATAAGAAAATAAATCT

GAAACATTAAATTTAAGGTAGTCGCTGGTCAGTATAGTCTGTTCTGGTTGGCGACTCCATTGTTAAAGAGTA

TAAAGACTTTAGATTTTATGAATATTAAAAATAGGAACAGTCAATTGAACTGCTCCTATTTTTCTGCTAAAT

ATATTGTAGTTTTCTTATATGTATAATGATAGATTAGCGGATTCTCATCTACGGTACTTACTTCAAATATGA

AGAAGTGATCGCGGTTATCTCTGGACTTTTCCTTATTGAGGACAAAGTAATTCTTACGTGAAGTCGCCATTG

TTTTTAGGATATCATCAGTTAGGAAGGTCAATGGAATATTCATGTTAGAGTAGCGGTAGAAGTCACGTTCAA

AATCTTGGTAGCTCTCGCTATAATAGTCCATTTGTAGGTGATTACGCTGAAACTCAAGCTGATTCATAGAGC

ACCTCCTCGACAAGTTCAATACTAATAATGTCTTTTAATTTCAAATTGATGTGACCTGTTGTAGTTTTTATC

AAAATGAAATCTTTGGTCAGACTTGGTATTGTTCCAGTGTAGGAAACACGCTTGTTTTTTTCAATCACTTGA

ATGCGTGTGCGTAGCTGCCCGGCGTATACTTGACTGAGGAGTAATAATTTCTTCTCTAGTGATAAGTCAGAC

ATGTACGTTACTTTGTTTGTATCATCAGAGAGTGCTGATGCATGTTCAGATAGGAAAAAGCCCATCCATTTT

TGCATCTTTGTATCCTGGTACTCTCTTGCTGATTGAAATGGTAAATATGAACGGTCAATCATATCAAATCCT

TTCTATGCAGAGGCAAGGGTATTTTTATCAAATTGAATCGTAAAACCTTGAATTCCCCCACCTGTGTAACAT

TCTTTAAAGCGATTGATTACCTCAGTATAGATTATCACAGATGAGCTTGTTGGCTTAATGCTAAATGTAAAT

TCCAATGGTAATCGGTTTTCAGATTTAGCATGTACTAGTCGTATCGATATTTCAGTTGTTTTGAGTTTTCTC

TGACGAAGTTTTGAAGTTGCTGTTTCAACGATTCCATGTAAAAATCTTTCAAGCATTTCAATATCATTACAT

CCTTTGCTACGGATTTCTGAAAATTGTACTGTATTTTTTTCTTGTTTCATTTTAATCCCTCCAATCCACCCG

CGGAATGACCACCGATAAGTTTACTGCGTTCAATATTTCTGGAACCTTCAGTTAGGACGGTTCCTTTTTGTA

TGGCTAAAAAACCAAACTGTTCTCTGACAACATCAATAGCTGTCTGAAGTCTATTATCTTTTTCAATTTGTT

CTACATCATCAAAGAGTGATAGTAGAGTATAGCTTTCATCTACGAAGCCACTATAAGATACACCAATTTGTC

TCACTGCACCAGAGGTGTATTTTTTCGGAATAATACAAGTACATGACTCACCATTGTTTTGGGGAGATTTG

CGGGTTCAATTTTATTCTGAGCATTTATAGATTTTTTCATCTCAGTCCTAGAATAGCCAATATGAATAGAAA

CGACAGTAGTCAATACTAGGGCTACTGTTCCGTTCCACAGTATCATTTAAAAATCATT-TTCACACCCTTTC

GTCTATTAGTATAGAAGAAAGCTCTCAGCACA

>GA40028/Mega-2.II

CATGTTGAGGCGGTAAGTTTGCTAGTCAAGGAGTAAAACGACGAAGATTAGCATTTACTTCCGCCCATGCGA

TAGCTGTCCGTGATTGACAAGTGCTAGCACGCAGACAGAACGGAGATAGCGAACCGCTGAGTGTGTCGCTCT

GCTCGTAAAAGCTTAGAAACCTTTGAACGAAAGGGATAATGAAAGCCTTGATTGCAAGGCTTTTTGCTTTAT

GGTGGGTAAGTATCAGAGTGAGAAAATTTTTGGAATGAGTAGAAGTGATAGCTAGAAATTATCAGTTTCTA

TTTCCATTTACCCTGTGGGTACGTGTTTGTTTCCATTGACAAGGAGTTTGTGGGAATAGAAATGT

ACCCACCTTGTTTGAATCAAGTGAAGTGTAGTTGAAGGAAATCTGTTGAAAGCAATACTTCATTTTACCG

AATAAGTAATAATTTAGGCAACTTCAAATCGATTAAAAAAAACTATTTTAAAGGTTAAGAGTAGACAAAA

ATTGTCCACTCTTTTTTGCAAACTCAATTTATCAATAAATGAAATGAGGGAATGTAAAATG

AAATATTTTGAGGTTGAGTTAGAAAATCCTGATGAATTTTTAAAACTACAAACAGAAGATTTTGTGAAAG

CTAATCGCTTGCTACTAAGGAAGATAATCCAGAGCGTTACAGTCTATGAAGAAAACTTCGTCATATCCTT

TAAATCTGGCATCGAATTGGAAGTATGAGTCTCATTCCATAACTTTTATATTGAACATATCATCTTGTTGTG

TTATACTATAAATTGATATAAACAAAGATGTAGGAGGAACCGAAACTATGACAGCCTCAATGCGTTTAAGAT

AAGCTGGCAATAAAAAAAGCAGAATCTATACCCGATGATAGGCTTTTTTGTTGTGCTTATTTATACGATATT

GAGCATTCATTAGTTACGGTGAGGATATTGGTTATTTAACTATACCTTTATTTAACTATGTCTTTAATATGA

ATGTTTCCAAATTGTATGTATGCAGACCAAAAGCCACATTGTGGGGTTTGGCCTGCATTTTTTTTGCCTA

GAATGCTATTCAAAATAGAAATTCAAGCAAAATAATATGCAGGAGATAATATAAATGGAAAAATACAACAAT

TGGAAACGAAAATTTTATGCAATATGGGCAGGGCAAGCAGTATCATTAATCACTAGTGCCATCCTGCAAATG

GCGATTATTTTTTACCTTACAGAAAAAACAGGATCTGCGATGGTCTTGTCTATGGCTTCATTAGTAGGTTTT

TTACCCTATGCGATTTTGGGACCTGCCATTGGTGTGCTAGTGGATCGTCATGATAGGAAGAAGATAATGATT

GGTGCCGATTTAATTATCGCAGCAGCTGGTGCAGTGCTTGCTATTGTTGCATTCTGTATGGAGCTACCTGTC

TGGATGATTATGATAGTATTGTTTATCCGTAGCATTGGAACAGCTTTTCATACCCCAGCACTCAATGCGGTT

ACACCACTTTTAGTACCAGAAGAACAGCTAACGAAATGCGCAGGCTATAGTCAGTCTTTGCAGTCTATAAGC

TATATTGTTAGTCCGGCAGTTGCAGCACTCTTATACTCCGTTTGGGATTTAAATGCTATTATTGCCATCGAC

GTATTGGGTGCTGTGATTGCATCTATTACGGTAGCAATTGTACGTATACCTAAGCTGGGTAATCAAGTGCAA

AGTTTAGAACCAAATTTCATAAGGGAGATGAAAGAAGGAGTTGTGGTTCTGAGACAAAACAAAGGATTGTTT

GCCTTATTACTCTTAGGAACACTATATACTTTTGTTTATATGCCAATCAATGCACTATTTCCTTTAATAAGC

ATGGAACACTTTAATGGAACGCCTGTGCATATTTCTATTACGGAAATTTCCTTTGCATTTGGGATGCTAGCA

GGAGGCTTATTATTAGGAAGATTAGGGGGCTTCGAAAAGCATGTATTACTAATAACAAGTTCATTTTTTATA

ATGGGGACCAGTTTAGCCGTTTCGGGAATACTTCCTCCAAATGGATTTGTAATATTCGTAGTTTGCTGTGCA

ATAATGGGGCTTTCGGTGCCATTTTATAGCGGTGTGCAAACAGCTCTTTTTCAGGAGAAAATTAAGCCTGAA

TATTTAGGACGTGTATTTTCTTTGATCGGAAGTATCATGTCACTTGCTATGCCAATTGGGTTAATTCTTTCT

GGATTCTTTGCTGATAAAATCGGTGTAAATCATTGGTTTTTACTATCAGGTATTTTAATTATTGGCATTGCT

ATAGTTTGCCAAATGATAACTGAGGTTAGAAAATTAGATTTAAAATAAACAATATTGGAGGAA

ATGTTATGGAATTAATATT

AAAAGCAAAAGACATTCGTGTGGAATTCAAAGGACGCGATGTTTTAGATATAAATGAATTAGAAGTATATGA

TTATGACCGTATTGGTTTAGTAGGAGCAAATGGTGCTGGAAAAAGCACTTTACTCAGGGTACTTTTAGGAGA

ATTAACTCCCCCAGGATGTAAAATGAATCGTCTGGGTGAACTTGCCTATATTCCCCAGTTGGACGAAGTAAC

TCTGCAGGAGGAAAAAGATTTTGCACTTGTAGGCAAGCTAGGTGTTGAGCAATTAAATATACAGACTATGAG

CGGTGGTGAAGAAACAAGGCTTAAAATAGCACAGGCCTTATCGGCACAGGTTCATGGTATTTTAGCGGATGA

ACCTACGAGCCATTTAGACCGTGAAGGAATTGATTTTCTAATAGGACAGCTAAAATATTTTACAGGTGCACT

GTTAGTTATTAGCCATGACCGCTATTTTCTTGATGAAATAGTAGATAAAATATGGGAACTGAAAGATGGCAA

AATCACTGAGTATTGGGGAAACTATTCTGATTATCTTCGTCAGAAAGAGGAAGAACGTAAGAGCCAAGCTGC

AGAATACGAACAATTTATTGCGGAACGTGCCCGATTGGAAAGGGCTGCGGAGGAAAAGCGAAAACAGGCTCG

TAAAATAGAACAGAAGGCAAAAGGTTCTTCAAAGAAAAAAAGTACTGAAGACGGAGGGCGTTTAGCTCATCA

AAAATCAATAGGAAGTAAGGAAAAAAAGATGTATAATGCTGCTAAAACCCTAGAGCACAGGATTGCGGCCTT

AGGAAAAGTAGAAGCTCCGGAAGGCATTCGCAGAATTCGTTTCAGGCAAAGTAAAGCATTGGAGCTCCATAA

TCCATACCCTATAGTCGGTGCAGAAATTAATAAAGTATTTGGGGATAAGGCTCTGTTTGAAAATGCATCTTT

TCAAATTCCGTTAGGAGCAAAAGTGGCGTTAACTGGTGGTAATGGAATCGGAAAAACAACTTTAATCCAAAT

GATCTTAAACCATGAAGAAGGAATTTCTATTTCGCCTAAGGCAAAAATAGGTTACTTTGCACAGAATGGTTA

CAAGTACAACAGTAATCAGAATGTTATGGAGTTTATGCAGAAGGATTGTGACTACAATATATCAGAAATTCG

TTCAGTGCTAGCATCTATGGGGTTCAAACAGAACGATATTGGAAAAAGTTTATCTGTTTTAAGCGGTGGAGA

AATTATAAAATTGTTGCTTGCTAAAATGCTCATGGGTAGATATAACATCCTAATAATGGATGAACCCAGTAA

CTTCCTTGACATACCAAGTTTAGAGGCTTTGGAAATACTAATGAAGGAGTACACCGGAACTATCGTGTTTAT

CACCCACGATAAACGATTACTCGAAAATGTAGCAGATGTAGTTTATGAAATTAGAGATAAGAAAATAAATCT

GAAACATTAAATTTAAGGTAGTCGCTGGTCAGTATAGTCTGTTCTGGTTGGCGACTCCATTGTTAAAGAGTA

TAAAGACTTTAGATTTTATGAATATTAAAAATAGGAACAGTCAATTGAACTGCTCCTATTTTTCTGCTAAAT

ATATTGTAGTTTTCTTATATGTATAATGATAGATTAGCGGATTCTCATCTACGGTACTTACTTCAAATATGA

AGAAGTGATCGCGGTTATCTCTGGACTTTTCCTTATTGAGGACAAAGTAATTCTTACGTGAAGTCGCCATTG

TTTTTAGGATATCATCAGTTAGGAAGGTCAATGGAATATTCATGTTAGAGTAGCGGTAGAAGTCACGTTCAA

AATCTTGGTAGCTCTCGCTATAATAGTCCATTTGTAGGTGATTACGCTGAAACTCAAGCTGATTCATAGAGC

ACCTCCTCGACAAGTTCAATACTAATAATGTCTTTTAATTTCAAATTGATGTGACCTGTTGTAGTTTTTATC

AAAATGAAATCTTTGGTCAGACTTGGTATTGTTCCAGTGTAGGAAACACGCTTGTTTTTTTCAATCACTTGA

ATGCGTGTGCGTAGCTGCCCGGCGTATACTTGACTGAGGAGTAATAATTTCTTCTCTAGTGATAAGTCAGAC

ATGTACGTTACTTTGTTTGTATCATCAGAGAGTGCTGATGCATGTTCAGATAGGAAAAAGCCCATCCATTTT

TGCATCTTTGTATCCTGGTACTCTCTTGCTGATTGAAATGGTAAATATGAACGGTCAATCATATCAAATCCT

TTCTATGCAGAGGCAAGGGTATTTTTATCAAATTGAATCGTAAAACCTTGAATTCCCCCACCTGTGTAACAT

TCTTTAAAGCGATTGATTACCTCAGTATAGATTATCACAGATGAGCTTGTTGGCTTAATGCTAAATGTAAAT

TCCAATGGTAATCGGTTTTCAGATTTAGCATGTACTAGTCGTATCGATATTTCAGTTGTTTTGAGTTTTCTC

TGACGAAGTTTTGAAGTTGCTGTTTCAACGATTCCATGTAAAAATCTTTCAAGCATTTCAATATCATTACAT

CCTTTGCTACGGATTTCTGAAAATTGTACTGTATTTTTTTCTTGTTTCATTTTAATCCCTCCAATCCACCCG

CGGAATGACCACCGATAAGTTTACTGCGTTCAATATTTCTGGAACCTTCAGTTAGGACGGTTCCTTTTTGTA

TGGCTAAAAAACCAAACTGTTCTCTGACAACATCAATAGCTGTCTGAAGTCTATTATCTTTTTCAATTTGTT

CTACATCATCAAAGAGTGATAGTAGAGTATAGCTTTCATCTACGAAGCCACTATAAGATACACCAATTTGTC

TCACTGCACCAGAGGTGTATTTTTTCGGAATAATACAAGTACATGACTCACCATTGTTTTGGGGAGATTTG

CGGGTTCAATTTTATTCTGAGCATTTATAGATTTTTTCATCTCAGTCCTAGAATAGCCAATATGAATAGAAA

CGACAGTAGTCAATACTAGGGCTACTGTTCCGTTCCACAGTATCATTTAAAAATCATT-TTCACACCCTTTC

GTCTATTAGTATAGAAGAAAGCTCTCAGCACA

>GA60190/Mega-1.II

CATGTTGAGGCGGTAAGTTTGCTAGTCAAGGAGTAAAACGACGAAGATTAGCATTTACTTCCGCCCATGCGA

TAGCTGTCCGTGATTGACAAGTGCTAGCACGCAGACAGAACGGAGATAGCGAACCGCTGAGTGTGTCGCTCT

GCTCGTAAAAGCTTAGAAACCTTTGAACGAAAGGGATAATGAAAGCCTTGATTGCAAGGCTTTTTGCTTTAT

GGTGGGTAAGTATCAGAGTGAGAAAATTTTTGGAATGAGTAGAAGTGATAGCTAGAAATTATCAGTTTCTA

TTTCCATTTACCCTGTGGGTACGTGTTTGTTTCCATTGACAAGGAGTTTGTGGGAATAGAAATGT

ACCCACCTTGTTTGAATCAAGTGAAGTGTAGTTGAAGGAAATCTGTTGAAAGCAATACTTCATTTTACCG

AATAAGTAATAATTTAGGCAACTTCAAATCGATTAAAAAAAACTATTTTAAAGGTTAAGAGTAGACAAAA

ATTGTCCACTCTTTTTTGCAAACTCAATTTATCAATAAATGAAATGAGGGAATGTAAAATG

AAATATTTTGAGGTTGAGTTAGAAAATCCTGATGAATTTTTAAAACTACAAACAGAAGATTTTGTGAAAG

CTAATCGCTTGCTACTAAGGAAGATAATCCAGAGCGTTACAGTCTATGAAGAAAACTTCGTCATATCCTT

TAAATCTGGCATCGAATTGGAAGTATGAGTCTCATTCCATAACTTTTATATTGAACATATCATCTTGTTGTG

TTATACTATAAATTGATATAAACAAAGATGTAGGAGGAACCGAAACTATGACAGCCTCAATGCGTTTAAGAT

AAGCTGGCAATAAAAAAAGCAGAATCTATACCCGATGATAGGCTTTTTTGTTGTGCTTATTTATACGATATT

GAGCATTCATTAGTTACGGTGAGGATATTGGTTATTTAACTATACCTTTATTTAACTATGTCTTTAATATGA

ATGTTTCCAAATTGTATGTATGCAGACCAAAAGCCACATTGTGGGGTTTGGCCTGCATTTTTTTTGCCTA

GAATGCTATTCAAAATAGAAATTCAAGCAAAATAATATGCAGGAGATAATATAAATGGAAAAATACAACAAT

TGGAAACGAAAATTTTATGCAATATGGGCAGGGCAAGCAGTATCATTAATCACTAGTGCCATCCTGCAAATG

GCGATTATTTTTTACCTTACAGAAAAAACAGGATCTGCGATGGTCTTGTCTATGGCTTCATTAGTAGGTTTT

TTACCCTATGCGATTTTGGGACCTGCCATTGGTGTGCTAGTGGATCGTCATGATAGGAAGAAGATAATGATT

GGTGCCGATTTAATTATCGCAGCAGCTGGTGCAGTGCTTGCTATTGTTGCATTCTGTATGGAGCTACCTGTC

TGGATGATTATGATAGTATTGTTTATCCGTAGCATTGGAACAGCTTTTCATACCCCAGCACTCAATGCGGTT

ACACCACTTTTAGTACCAGAAGAACAGCTAACGAAATGCGCAGGCTATAGTCAGTCTTTGCAGTCTATAAGC

TATATTGTTAGTCCGGCAGTTGCAGCACTCTTATACTCCGTTTGGGATTTAAATGCTATTATTGCCATCGAC

GTATTGGGTGCTGTGATTGCATCTATTACGGTAGCAATTGTACGTATACCTAAGCTGGGTAATCAAGTGCAA

AGTTTAGAACCAAATTTCATAAGGGAGATGAAAGAAGGAGTTGTGGTTCTGAGACAAAACAAAGGATTGTTT

GCCTTATTACTCTTAGGAACACTATATACTTTTGTTTATATGCCAATCAATGCACTATTTCCTTTAATAAGC

ATGGAACACTTTAATGGAACGCCTGTGCATATTTCTATTACGGAAATTTCCTTTGCATTTGGGATGCTAGCA

GGAGGCTTATTATTAGGAAGATTAGGGGGCTTCGAAAAGCATGTATTACTAATAACAAGTTCATTTTTTATA

ATGGGGACCAGTTTAGCCGTTTCGGGAATACTTCCTCCAAATGGATTTGTAATATTCGTAGTTTGCTGTGCA

ATAATGGGGCTTTCGGTGCCATTTTATAGCGGTGTGCAAACAGCTCTTTTTCAGGAGAAAATTAAGCCTGAA

TATTTAGGACGTGTATTTTCTTTGATCGGAAGTATCATGTCACTTGCTATGCCAATTGGGTTAATTCTTTCT

GGATTCTTTGCTGATAAAATCGGTGTAAATCATTGGTTTTTACTATCAGGTATTTTAATTATTGGCATTGCT

ATAGTTTGCCAAATGATAACTGAGGTTAGAAAATTAGATTTAAAATAAACAATATTGGAGGAATATTTATGT

ATCTTATTTTCATGTAACTCTTCCTGCTAAAATCGCAGGGTTTTCCCTGCATACAAGCAAATGAAAGCATGC

GATTATAGACAGGAGGAAATGTTATGGAATTAATATT

AAAAGCAAAAGACATTCGTGTGGAATTCAAAGGACGCGATGTTTTAGATATAAATGAATTAGAAGTATATGA

TTATGACCGTATTGGTTTAGTAGGAGCAAATGGTGCTGGAAAAAGCACTTTACTCAGGGTACTTTTAGGAGA

ATTAACTCCCCCAGGATGTAAAATGAATCGTCTGGGTGAACTTGCCTATATTCCCCAGTTGGACGAAGTAAC

TCTGCAGGAGGAAAAAGATTTTGCACTTGTAGGCAAGCTAGGTGTTGAGCAATTAAATATACAGACTATGAG

CGGTGGTGAAGAAACAAGGCTTAAAATAGCACAGGCCTTATCGGCACAGGTTCATGGTATTTTAGCGGATGA

ACCTACGAGCCATTTAGACCGTGAAGGAATTGATTTTCTAATAGGACAGCTAAAATATTTTACAGGTGCACT

GTTAGTTATTAGCCATGACCGCTATTTTCTTGATGAAATAGTAGATAAAATATGGGAACTGAAAGATGGCAA

AATCACTGAGTATTGGGGAAACTATTCTGATTATCTTCGTCAGAAAGAGGAAGAACGTAAGAGCCAAGCTGC

AGAATACGAACAATTTATTGCGGAACGTGCCCGATTGGAAAGGGCTGCGGAGGAAAAGCGAAAACAGGCTCG

TAAAATAGAACAGAAGGCAAAAGGTTCTTCAAAGAAAAAAAGTACTGAAGACGGAGGGCGTTTAGCTCATCA

AAAATCAATAGGAAGTAAGGAAAAAAAGATGTATAATGCTGCTAAAACCCTAGAGCACAGGATTGCGGCCTT

AGGAAAAGTAGAAGCTCCGGAAGGCATTCGCAGAATTCGTTTCAGGCAAAGTAAAGCATTGGAGCTCCATAA

TCCATACCCTATAGTCGGTGCAGAAATTAATAAAGTATTTGGGGATAAGGCTCTGTTTGAAAATGCATCTTT

TCAAATTCCGTTAGGAGCAAAAGTGGCGTTAACTGGTGGTAATGGAATCGGAAAAACAACTTTAATCCAAAT

GATCTTAAACCATGAAGAAGGAATTTCTATTTCGCCTAAGGCAAAAATAGGTTACTTTGCACAGAATGGTTA

CAAGTACAACAGTAATCAGAATGTTATGGAGTTTATGCAGAAGGATTGTGACTACAATATATCAGAAATTCG

TTCAGTGCTAGCATCTATGGGGTTCAAACAGAACGATATTGGAAAAAGTTTATCTGTTTTAAGCGGTGGAGA

AATTATAAAATTGTTGCTTGCTAAAATGCTCATGGGTAGATATAACATCCTAATAATGGATGAACCCAGTAA

CTTCCTTGACATACCAAGTTTAGAGGCTTTGGAAATACTAATGAAGGAGTACACCGGAACTATCGTGTTTAT

CACCCACGATAAACGATTACTCGAAAATGTAGCAGATGTAGTTTATGAAATTAGAGATAAGAAAATAAATCT

GAAACATTAAATTTAAGGTAGTCGCTGGTCAGTATAGTCTGTTCTGGTTGGCGACTCCATTGTTAAAGAGTA

TAAAGACTTTAGATTTTATGAATATTAAAAATAGGAACAGTCAATTGAACTGCTCCTATTTTTCTGCTAAAT

ATATTGTAGTTTTCTTATATGTATAATGATAGATTAGCGGATTCTCATCTACGGTACTTACTTCAAATATGA

AGAAGTGATCGCGGTTATCTCTGGACTTTTCCTTATTGAGGACAAAGTAATTCTTACGTGAAGTCGCCATTG

TTTTTAGGATATCATCAGTTAGGAAGGTCAATGGAATATTCATGTTAGAGTAGCGGTAGAAGTCACGTTCAA

AATCTTGGTAGCTCTCGCTATAATAGTCCATTTGTAGGTGATTACGCTGAAACTCAAGCTGATTCATAGAGC

ACCTCCTCGACAAGTTCAATACTAATAATGTCTTTTAATTTCAAATTGATGTGACCTGTTGTAGTTTTTATC

AAAATGAAATCTTTGGTCAGACTTGGTATTGTTCCAGTGTAGGAAACACGCTTGTTTTTTTCAATCACTTGA

ATGCGTGTGCGTAGCTGCCCGGCGTATACTTGACTGAGGAGTAATAATTTCTTCTCTAGTGATAAGTCAGAC

ATGTACGTTACTTTGTTTGTATCATCAGAGAGTGCTGATGCATGTTCAGATAGGAAAAAGCCCATCCATTTT

TGCATCTTTGTATCCTGGTACTCTCTTGCTGATTGAAATGGTAAATATGAACGGTCAATCATATCAAATCCT

TTCTATGCAGAGGCAAGGGTATTTTTATCAAATTGAATCGTAAAACCTTGAATTCCCCCACCTGTGTAACAT

TCTTTAAAGCGATTGATTACCTCAGTATAGATTATCACAGATGAGCTTGTTGGCTTAATGCTAAATGTAAAT

TCCAATGGTAATCGGTTTTCAGATTTAGCATGTACTAGTCGTATCGATATTTCAGTTGTTTTGAGTTTTCTC

TGACGAAGTTTTGAAGTTGCTGTTTCAACGATTCCATGTAAAAATCTTTCAAGCATTTCAATATCATTACAT

CCTTTGCTACGGATTTCTGAAAATTGTACTGTATTTTTTTCTTGTTTCATTTTAATCCCTCCAATCCACCCG

CGGAATGACCACCGATAAGTTTACTGCGTTCAATATTTCTGGAACCTTCAGTTAGGACGGTTCCTTTTTGTA

TGGCTAAAAAACCAAACTGTTCTCTGACAACATCAATAGCTGTCTGAAGTCTATTATCTTTTTCAATTTGTT

CTACATCATCAAAGAGTGATAGTAGAGTATAGCTTTCATCTACGAAGCCACTATAAGATACACCAATTTGTC

TCACTGCACCAGAGGTGTATTTTTTCGGAATAATACAAGTACATGACTCACCATTGTTTTGGGGAGATTTG

CGGGTTCAATTTTATTCTGAGCATTTATAGATTTTTTCATCTCAGTCCTAGAATAGCCAATATGAATAGAAA

CGACAGTAGTCAATACTAGGGCTACTGTTCCGTTCCACAGTATCATTTAAAAATCATT-TTCACACCCTTTC

GTCTATTAGTATAGAAGAAAGCTCTCAGCACA

>EU-NP04/Mega-1.II

CATGTTGAGGCGGTAAGTTTGCTAGTCAAGGAGTAAAACGACGAAGATTAGCATTTACTTCCGCCCATGCGA

TAGCTGTCCGTGATTGACAAGTGCTAGCACGCAGACAGAACGGAGATAGCGAACCGCTGAGTGTGTCGCTCT

GCTCGTAAAAGCTTAGAAACCTTTGAACGAAAGGGATAATGAAAGCCTTGATTGCAAGGCTTTTTGCTTTAT

GGTGGGTAAGTATCAGAGTGAGAAAATTTTTGGAATGAGTAGAAGTGATAGCTAGAAATTATCAGTTTCTA

TTTCCATTTACCCTGTGGGTACGTGTTTGTTTCCATTGACAAGGAGTTTGTGGGAATAGAAATGT

ACCCACCTTGTTTGAATCAAGTGAAGTGTAGTTGAAGGAAATCTGTTGAAAGCAATACTTCATTTTACCG

AATAAGTAATAATTTAGGCAACTTCAAATCGATTAAAAAAAACTATTTTAAAGGTTAAGAGTAGACAAAA

ATTGTCCACTCTTTTTTGCAAACTCAATTTATCAATAAATGAAATGAGGGAATGTAAAATG

AAATATTTTGAGGTTGAGTTAGAAAATCCTGATGAATTTTTAAAACTACAAACAGAAGATTTTGTGAAAG

CTAATCGCTTGCTACTAAGGAAGATAATCCAGAGCGTTACAGTCTATGAAGAAAACTTCGTCATATCCTT

TAAATCTGGCATCGAATTGGAAGTATGAGTCTCATTCCATAACTTTTATATTGAACATATCATCTTGTTGTG

TTATACTATAAATTGATATAAACAAAGATGTAGGAGGAACCGAAACTATGACAGCCTCAATGCGTTTAAGAT

AAGCTGGCAATAAAAAAAGCAGAATCTATACCCGATGATAGGCTTTTTTGTTGTGCTTATTTATACGATATT

GAGCATTCATTAGTTACGGTGAGGATATTGGTTATTTAACTATACCTTTATTTAACTATGTCTTTAATATGA

ATGTTTCCAAATTGTATGTATGCAGACCAAAAGCCACATTGTGGGGTTTGGCCTGCATTTTTTTTGCCTA

GAATGCTATTCAAAATAGAAATTCAAGCAAAATAATATGCAGGAGATAATATAAATGGAAAAATACAACAAT

TGGAAACGAAAATTTTATGCAATATGGGCAGGGCAAGCAGTATCATTAATCACTAGTGCCATCCTGCAAATG

GCGATTATTTTTTACCTTACAGAAAAAACAGGATCTGCGATGGTCTTGTCTATGGCTTCATTAGTAGGTTTT

TTACCCTATGCGATTTTGGGACCTGCCATTGGTGTGCTAGTGGATCGTCATGATAGGAAGAAGATAATGATT

GGTGCCGATTTAATTATCGCAGCAGCTGGTGCAGTGCTTGCTATTGTTGCATTCTGTATGGAGCTACCTGTC

TGGATGATTATGATAGTATTGTTTATCCGTAGCATTGGAACAGCTTTTCATACCCCAGCACTCAATGCGGTT

ACACCACTTTTAGTACCAGAAGAACAGCTAACGAAATGCGCAGGCTATAGTCAGTCTTTGCAGTCTATAAGC

TATATTGTTAGTCCGGCAGTTGCAGCACTCTTATACTCCGTTTGGGATTTAAATGCTATTATTGCCATCGAC

GTATTGGGTGCTGTGATTGCATCTATTACGGTAGCAATTGTACGTATACCTAAGCTGGGTAATCAAGTGCAA

AGTTTAGAACCAAATTTCATAAGGGAGATGAAAGAAGGAGTTGTGGTTCTGAGACAAAACAAAGGATTGTTT

GCCTTATTACTCTTAGGAACACTATATACTTTTGTTTATATGCCAATCAATGCACTATTTCCTTTAATAAGC

ATGGAACACTTTAATGGAACGCCTGTGCATATTTCTATTACGGAAATTTCCTTTGCATTTGGGATGCTAGCA

GGAGGCTTATTATTAGGAAGATTAGGGGGCTTCGAAAAGCATGTATTACTAATAACAAGTTCATTTTTTATA

ATGGGGACCAGTTTAGCCGTTTCGGGAATACTTCCTCCAAATGGATTTGTAATATTCGTAGTTTGCTGTGCA

ATAATGGGGCTTTCGGTGCCATTTTATAGCGGTGTGCAAACAGCTCTTTTTCAGGAGAAAATTAAGCCTGAA

TATTTAGGACGTGTATTTTCTTTGATCGGAAGTATCATGTCACTTGCTATGCCAATTGGGTTAATTCTTTCT

GGATTCTTTGCTGATAAAATCGGTGTAAATCATTGGTTTTTACTATCAGGTATTTTAATTATTGGCATTGCT

ATAGTTTGCCAAATGATAACTGAGGTTAGAAAATTAGATTTAAAATAAACAATATTGGAGGAATATTTATGT

ATCTTATTTTCATGTAACTCTTCCTGCTAAAATCGCAGGGTTTTCCCTGCATACAAGCAAATGAAAGCATGC

GATTATAGACAGGAGGAAATGTTATGGAATTAATATT

AAAAGCAAAAGACATTCGTGTGGAATTCAAAGGACGCGATGTTTTAGATATAAATGAATTAGAAGTATATGA

TTATGACCGTATTGGTTTAGTAGGAGCAAATGGTGCTGGAAAAAGCACTTTACTCAGGGTACTTTTAGGAGA

ATTAACTCCCCCAGGATGTAAAATGAATCGTCTGGGTGAACTTGCCTATATTCCCCAGTTGGACGAAGTAAC

TCTGCAGGAGGAAAAAGATTTTGCACTTGTAGGCAAGCTAGGTGTTGAGCAATTAAATATACAGACTATGAG

CGGTGGTGAAGAAACAAGGCTTAAAATAGCACAGGCCTTATCGGCACAGGTTCATGGTATTTTAGCGGATGA

ACCTACGAGCCATTTAGACCGTGAAGGAATTGATTTTCTAATAGGACAGCTAAAATATTTTACAGGTGCACT

GTTAGTTATTAGCCATGACCGCTATTTTCTTGATGAAATAGTAGATAAAATATGGGAACTGAAAGATGGCAA

AATCACTGAGTATTGGGGAAACTATTCTGATTATCTTCGTCAGAAAGAGGAAGAACGTAAGAGCCAAGCTGC

AGAATACGAACAATTTATTGCGGAACGTGCCCGATTGGAAAGGGCTGCGGAGGAAAAGCGAAAACAGGCTCG

TAAAATAGAACAGAAGGCAAAAGGTTCTTCAAAGAAAAAAAGTACTGAAGACGGAGGGCGTTTAGCTCATCA

AAAATCAATAGGAAGTAAGGAAAAAAAGATGTATAATGCTGCTAAAACCCTAGAGCACAGGATTGCGGCCTT

AGGAAAAGTAGAAGCTCCGGAAGGCATTCGCAGAATTCGTTTCAGGCAAAGTAAAGCATTGGAGCTCCATAA

TCCATACCCTATAGTCGGTGCAGAAATTAATAAAGTATTTGGGGATAAGGCTCTGTTTGAAAATGCATCTTT

TCAAATTCCGTTAGGAGCAAAAGTGGCGTTAACTGGTGGTAATGGAATCGGAAAAACAACTTTAATCCAAAT

GATCTTAAACCATGAAGAAGGAATTTCTATTTCGCCTAAGGCAAAAATAGGTTACTTTGCACAGAATGGTTA

CAAGTACAACAGTAATCAGAATGTTATGGAGTTTATGCAGAAGGATTGTGACTACAATATATCAGAAATTCG

TTCAGTGCTAGCATCTATGGGGTTCAAACAGAACGATATTGGAAAAAGTTTATCTGTTTTAAGCGGTGGAGA

AATTATAAAATTGTTGCTTGCTAAAATGCTCATGGGTAGATATAACATCCTAATAATGGATGAACCCAGTAA

CTTCCTTGACATACCAAGTTTAGAGGCTTTGGAAATACTAATGAAGGAGTACACCGGAACTATCGTGTTTAT

CACCCACGATAAACGATTACTCGAAAATGTAGCAGATGTAGTTTATGAAATTAGAGATAAGAAAATAAATCT

GAAACATTAAATTTAAGGTAGTCGCTGGTCAGTATAGTCTGTTCTGGTTGGCGACTCCATTGTTAAAGAGTA

TAAAGACTTTAGATTTTATGAATATTAAAAATAGGAACAGTCAATTGAACTGCTCCTATTTTTCTGCTAAAT

ATATTGTAGTTTTCTTATATGTATAATGATAGATTAGCGGATTCTCATCTACGGTACTTACTTCAAATATGA

AGAAGTGATCGCGGTTATCTCTGGACTTTTCCTTATTGAGGACAAAGTAATTCTTACGTGAAGTCGCCATTG

TTTTTAGGATATCATCAGTTAGGAAGGTCAATGGAATATTCATGTTAGAGTAGCGGTAGAAGTCACGTTCAA

AATCTTGGTAGCTCTCGCTATAATAGTCCATTTGTAGGTGATTACGCTGAAACTCAAGCTGATTCATAGAGC

ACCTCCTCGACAAGTTCAATACTAATAATGTCTTTTAATTTCAAATTGATGTGACCTGTTGTAGTTTTTATC

AAAATGAAATCTTTGGTCAGACTTGGTATTGTTCCAGTGTAGGAAACACGCTTGTTTTTTTCAATCACTTGA

ATGCGTGTGCGTAGCTGCCCGGCGTATACTTGACTGAGGAGTAATAATTTCTTCTCTAGTGATAAGTCAGAC

ATGTACGTTACTTTGTTTGTATCATCAGAGAGTGCTGATGCATGTTCAGATAGGAAAAAGCCCATCCATTTT

TGCATCTTTGTATCCTGGTACTCTCTTGCTGATTGAAATGGTAAATATGAACGGTCAATCATATCAAATCCT

TTCTATGCAGAGGCAAGGGTATTTTTATCAAATTGAATCGTAAAACCTTGAATTCCCCCACCTGTGTAACAT

TCTTTAAAGCGATTGATTACCTCAGTATAGATTATCACAGATGAGCTTGTTGGCTTAATGCTAAATGTAAAT

TCCAATGGTAATCGGTTTTCAGATTTAGCATGTACTAGTCGTATCGATATTTCAGTTGTTTTGAGTTTTCTC

TGACGAAGTTTTGAAGTTGCTGTTTCAACGATTCCATGTAAAAATCTTTCAAGCATTTCAATATCATTACAT

CCTTTGCTACGGATTTCTGAAAATTGTACTGTATTTTTTTCTTGTTTCATTTTAATCCCTCCAATCCACCCG

CGGAATGACCACCGATAAGTTTACTGCGTTCAATATTTCTGGAACCTTCAGTTAGGACGGTTCCTTTTTGTA

TGGCTAAAAAACCAAACTGTTCTCTGACAACATCAATAGCTGTCTGAAGTCTATTATCTTTTTCAATTTGTT

CTACATCATCAAAGAGTGATAGTAGAGTATAGCTTTCATCTACGAAGCCACTATAAGATACACCAATTTGTC

TCACTGCACCAGAGGTGTATTTTTTCGGAATAATACAAGTACATGACTCACCATTGTTTTGGGGAGATTTG

CGGGTTCAATTTTATTCTGAGCATTTATAGATTTTTTCATCTCAGTCCTAGAATAGCCAATATGAATAGAAA

CGACAGTAGTCAATACTAGGGCTACTGTTCCGTTCCACAGTATCATTTAAAAATCATT-TTCACACCCTTTC

GTCTATTAGTATAGAAGAAAGCTCTCAGCACA

>GA02270/Mega-2.II

CATGTTGAGGCGGTAAGTTTGCTAGTCAAGGAGTAAAACGACGAAGATTAGCATTTACTTCCGCCCATGCGA

TAGCTGTCCGTGATTGACAAGTGCTAGCACGCAGACAGAACGGAGATAGCGAACCGCTGAGTGTGTCGCTCT

GCTCGTAAAAGCTTAGAAACCTTTGAACGAAAGGGATAATGAAAGCCTTGATTGCAAGGCTTTTTGCTTTAT

GGTGGGTAAGTATCAGAGTGAGAAAATTTTTGGAATGAGTAGAAGTGATAGCTAGAAATTATCAGTTTCTA

TTTCCATTTACCCTGTGGGTACGTGTTTGTTTCCATTGACAAGGAGTTTGTGGGAATAGAAATGT

ACCCACCTTGTTTGAATCAAGTGAAGTGTAGTTGAAGGAAATCTGTTGAAAGCAATACTTCATTTTACCG

AATAAGTAATAATTTAGGCAACTTCAAATCGATTAAAAAAAACTATTTTAAAGGTTAAGAGTAGACAAAA

ATTGTCCACTCTTTTTTGCAAACTCAATTTATCAATAAATGAAATGAGGGAATGTAAAATG

AAATATTTTGAGGTTGAGTTAGAAAATCCTGATGAATTTTTAAAACTACAAACAGAAGATTTTGTGAAAG

CTAATCGCTTGCTACTAAGGAAGATAATCCAGAGCGTTACAGTCTATGAAGAAAACTTCGTCATATCCTT

TAAATCTGGCATCGAATTGGAAGTATGAGTCTCATTCCATAACTTTTATATTGAACATATCATCTTGTTGTG

TTATACTATAAATTGATATAAACAAAGATGTAGGAGGAACCGAAACTATGACAGCCTCAATGCGTTTAAGAT

AAGCTGGCAATAAAAAAAGCAGAATCTATACCCGATGATAGGCTTTTTTGTTGTGCTTATTTATACGATATT

GAGCATTCATTAGTTACGGTGAGGATATTGGTTATTTAACTATACCTTTATTTAACTATGTCTTTAATATGA

ATGTTTCCAAATTGTATGTATGCAGACCAAAAGCCACATTGTGGGGTTTGGCCTGCATTTTTTTTGCCTA

GAATGCTATTCAAAATAGAAATTCAAGCAAAATAATATGCAGGAGATAATATAAATGGAAAAATACAACAAT

TGGAAACGAAAATTTTATGCAATATGGGCAGGGCAAGCAGTATCATTAATCACTAGTGCCATCCTGCAAATG

GCGATTATTTTTTACCTTACAGAAAAAACAGGATCTGCGATGGTCTTGTCTATGGCTTCATTAGTAGGTTTT

TTACCCTATGCGATTTTGGGACCTGCCATTGGTGTGCTAGTGGATCGTCATGATAGGAAGAAGATAATGATT

GGTGCCGATTTAATTATCGCAGCAGCTGGTGCAGTGCTTGCTATTGTTGCATTCTGTATGGAGCTACCTGTC

TGGATGATTATGATAGTATTGTTTATCCGTAGCATTGGAACAGCTTTTCATACCCCAGCACTCAATGCGGTT

ACACCACTTTTAGTACCAGAAGAACAGCTAACGAAATGCGCAGGCTATAGTCAGTCTTTGCAGTCTATAAGC

TATATTGTTAGTCCGGCAGTTGCAGCACTCTTATACTCCGTTTGGGATTTAAATGCTATTATTGCCATCGAC

GTATTGGGTGCTGTGATTGCATCTATTACGGTAGCAATTGTACGTATACCTAAGCTGGGTAATCAAGTGCAA

AGTTTAGAACCAAATTTCATAAGGGAGATGAAAGAAGGAGTTGTGGTTCTGAGACAAAACAAAGGATTGTTT

GCCTTATTACTCTTAGGAACACTATATACTTTTGTTTATATGCCAATCAATGCACTATTTCCTTTAATAAGC

ATGGAACACTTTAATGGAACGCCTGTGCATATTTCTATTACGGAAATTTCCTTTGCATTTGGGATGCTAGCA

GGAGGCTTATTATTAGGAAGATTAGGGGGCTTCGAAAAGCATGTATTACTAATAACAAGTTCATTTTTTATA

ATGGGGACCAGTTTAGCCGTTTCGGGAATACTTCCTCCAAATGGATTTGTAATATTCGTAGTTTGCTGTGCA

ATAATGGGGCTTTCGGTGCCATTTTATAGCGGTGTGCAAACAGCTCTTTTTCAGGAGAAAATTAAGCCTGAA

TATTTAGGACGTGTATTTTCTTTGATCGGAAGTATCATGTCACTTGCTATGCCAATTGGGTTAATTCTTTCT

GGATTCTTTGCTGATAAAATCGGTGTAAATCATTGGTTTTTACTATCAGGTATTTTAATTATTGGCATTGCT

ATAGTTTGCCAAATGATAACTGAGGTTAGAAAATTAGATTTAAAATAAACAATATTGGAGGAA

ATGTTATGGAATTAATATT

AAAAGCAAAAGACATTCGTGTGGAATTCAAAGGACGCGATGTTTTAGATATAAATGAATTAGAAGTATATGA

TTATGACCGTATTGGTTTAGTAGGAGCAAATGGTGCTGGAAAAAGCACTTTACTCAGGGTACTTTTAGGAGA

ATTAACTCCCCCAGGATGTAAAATGAATCGTCTGGGTGAACTTGCCTATATTCCCCAGTTGGACGAAGTAAC

TCTGCAGGAGGAAAAAGATTTTGCACTTGTAGGCAAGCTAGGTGTTGAGCAATTAAATATACAGACTATGAG

CGGTGGTGAAGAAACAAGGCTTAAAATAGCACAGGCCTTATCGGCACAGGTTCATGGTATTTTAGCGGATGA

ACCTACGAGCCATTTAGACCGTGAAGGAATTGATTTTCTAATAGGACAGCTAAAATATTTTACAGGTGCACT

GTTAGTTATTAGCCATGACCGCTATTTTCTTGATGAAATAGTAGATAAAATATGGGAACTGAAAGATGGCAA

AATCACTGAGTATTGGGGAAACTATTCTGATTATCTTCGTCAGAAAGAGGAAGAACGTAAGAGCCAAGCTGC

AGAATACGAACAATTTATTGCGGAACGTGCCCGATTGGAAAGGGCTGCGGAGGAAAAGCGAAAACAGGCTCG

TAAAATAGAACAGAAGGCAAAAGGTTCTTCAAAGAAAAAAAGTACTGAAGACGGAGGGCGTTTAGCTCATCA

AAAATCAATAGGAAGTAAGGAAAAAAAGATGTATAATGCTGCTAAAACCCTAGAGCACAGGATTGCGGCCTT

AGGAAAAGTAGAAGCTCCGGAAGGCATTCGCAGAATTCGTTTCAGGCAAAGTAAAGCATTGGAGCTCCATAA

TCCATACCCTATAGTCGGTGCAGAAATTAATAAAGTATTTGGGGATAAGGCTCTGTTTGAAAATGCATCTTT

TCAAATTCCGTTAGGAGCAAAAGTGGCGTTAACTGGTGGTAATGGAATCGGAAAAACAACTTTAATCCAAAT

GATCTTAAACCATGAAGAAGGAATTTCTATTTCGCCTAAGGCAAAAATAGGTTACTTTGCACAGAATGGTTA

CAAGTACAACAGTAATCAGAATGTTATGGAGTTTATGCAGAAGGATTGTGACTACAATATATCAGAAATTCG

TTCAGTGCTAGCATCTATGGGGTTCAAACAGAACGATATTGGAAAAAGTTTATCTGTTTTAAGCGGTGGAGA

AATTATAAAATTGTTGCTTGCTAAAATGCTCATGGGTAGATATAACATCCTAATAATGGATGAACCCAGTAA

CTTCCTTGACATACCAAGTTTAGAGGCTTTGGAAATACTAATGAAGGAGTACACCGGAACTATCGTGTTTAT

CACCCACGATAAACGATTACTCGAAAATGTAGCAGATGTAGTTTATGAAATTAGAGATAAGAAAATAAATCT

GAAACATTAAATTTAAGGTAGTCGCTGGTCAGTATAGTCTGTTCTGGTTGGCGACTCCATTGTTAAAGAGTA

TAAAGACTTTAGATTTTATGAATATTAAAAATAGGAACAGTCAATTGAACTGCTCCTATTTTTCTGCTAAAT

ATATTGTAGTTTTCTTATATGTATAATGATAGATTAGCGGATTCTCATCTACGGTACTTACTTCAAATATGA

AGAAGTGATCGCGGTTATCTCTGGACTTTTCCTTATTGAGGACAAAGTAATTCTTACGTGAAGTCGCCATTG

TTTTTAGGATATCATCAGTTAGGAAGGTCAATGGAATATTCATGTTAGAGTAGCGGTAGAAGTCACGTTCAA

AATCTTGGTAGCTCTCGCTATAATAGTCCATTTGTAGGTGATTACGCTGAAACTCAAGCTGATTCATAGAGC

ACCTCCTCGACAAGTTCAATACTAATAATGTCTTTTAATTTCAAATTGATGTGACCTGTTGTAGTTTTTATC

AAAATGAAATCTTTGGTCAGACTTGGTATTGTTCCAGTGTAGGAAACACGCTTGTTTTTTTCAATCACTTGA

ATGCGTGTGCGTAGCTGCCCGGCGTATACTTGACTGAGGAGTAATAATTTCTTCTCTAGTGATAAGTCAGAC

ATGTACGTTACTTTGTTTGTATCATCAGAGAGTGCTGATGCATGTTCAGATAGGAAAAAGCCCATCCATTTT

TGCATCTTTGTATCCTGGTACTCTCTTGCTGATTGAAATGGTAAATATGAACGGTCAATCATATCAAATCCT

TTCTATGCAGAGGCAAGGGTATTTTTATCAAATTGAATCGTAAAACCTTGAATTCCCCCACCTGTGTAACAT

TCTTTAAAGCGATTGATTACCTCAGTATAGATTATCACAGATGAGCTTGTTGGCTTAATGCTAAATGTAAAT

TCCAATGGTAATCGGTTTTCAGATTTAGCATGTACTAGTCGTATCGATATTTCAGTTGTTTTGAGTTTTCTC

TGACGAAGTTTTGAAGTTGCTGTTTCAACGATTCCATGTAAAAATCTTTCAAGCATTTCAATATCATTACAT

CCTTTGCTACGGATTTCTGAAAATTGTACTGTATTTTTTTCTTGTTTCATTTTAATCCCTCCAATCCACCCG

CGGAATGACCACCGATAAGTTTACTGCGTTCAATATTTCTGGAACCTTCAGTTAGGACGGTTCCTTTTTGTA

TGGCTAAAAAACCAAACTGTTCTCTGACAACATCAATAGCTGTCTGAAGTCTATTATCTTTTTCAATTTGTT

CTACATCATCAAAGAGTGATAGTAGAGTATAGCTTTCATCTACGAAGCCACTATAAGATACACCAATTTGTC

TCACTGCACCAGAGGTGTATTTTTTCGGAATAATACAAGTACATGACTCACCATTGTTTTGGGGAGATTTG

CGGGTTCAATTTTATTCTGAGCATTTATAGATTTTTTCATCTCAGTCCTAGAATAGCCAATATGAATAGAAA

CGACAGTAGTCAATACTAGGGCTACTGTTCCGTTCCACAGTATCATTTAAAAATCATT-TTCACACCCTTTC

GTCTATTAGTATAGAAGAAAGCTCTCAGCACA

>GA41688/Mega-2.II

CATGTTGAGGCGGTAAGTTTGCTAGTCAAGGAGTAAAACGACGAAGATTAGCATTTACTTCCGCCCATGCGA

TAGCTGTCCGTGATTGACAAGTGCTAGCACGCAGACAGAACGGAGATAGCGAACCGCTGAGTGTGTCGCTCT

GCTCGTAAAAGCTTAGAAACCTTTGAACGAAAGGGATAATGAAAGCCTTGATTGCAAGGCTTTTTGCTTTAT

GGTGGGTAAGTATCAGAGTGAGAAAATTTTTGGAATGAGTAGAAGTGATAGCTAGAAATTATCAGTTTCTA

TTTCCATTTACCCTGTGGGTACGTGTTTGTTTCCATTGACAAGGAGTTTGTGGGAATAGAAATGT

ACCCACCTTGTTTGAATCAAGTGAAGTGTAGTTGAAGGAAATCTGTTGAAAGCAATACTTCATTTTACCG

AATAAGTAATAATTTAGGCAACTTCAAATCGATTAAAAAAAACTATTTTAAAGGTTAAGAGTAGACAAAA

ATTGTCCACTCTTTTTTGCAAACTCAATTTATCAATAAATGAAATGAGGGAATGTAAAATG

AAATATTTTGAGGTTGAGTTAGAAAATCCTGATGAATTTTTAAAACTACAAACAGAAGATTTTGTGAAAG

CTAATCGCTTGCTACTAAGGAAGATAATCCAGAGCGTTACAGTCTATGAAGAAAACTTCGTCATATCCTT

TAAATCTGGCATCGAATTGGAAGTATGAGTCTCATTCCATAACTTTTATATTGAACATATCATCTTGTTGTG

TTATACTATAAATTGATATAAACAAAGATGTAGGAGGAACCGAAACTATGACAGCCTCAATGCGTTTAAGAT

AAGCTGGCAATAAAAAAAGCAGAATCTATACCCGATGATAGGCTTTTTTGTTGTGCTTATTTATACGATATT

GAGCATTCATTAGTTACGGTGAGGATATTGGTTATTTAACTATACCTTTATTTAACTATGTCTTTAATATGA

ATGTTTCCAAATTGTATGTATGCAGACCAAAAGCCACATTGTGGGGTTTGGCCTGCATTTTTTTTGCCTA

GAATGCTATTCAAAATAGAAATTCAAGCAAAATAATATGCAGGAGATAATATAAATGGAAAAATACAACAAT

TGGAAACGAAAATTTTATGCAATATGGGCAGGGCAAGCAGTATCATTAATCACTAGTGCCATCCTGCAAATG

GCGATTATTTTTTACCTTACAGAAAAAACAGGATCTGCGATGGTCTTGTCTATGGCTTCATTAGTAGGTTTT

TTACCCTATGCGATTTTGGGACCTGCCATTGGTGTGCTAGTGGATCGTCATGATAGGAAGAAGATAATGATT

GGTGCCGATTTAATTATCGCAGCAGCTGGTGCAGTGCTTGCTATTGTTGCATTCTGTATGGAGCTACCTGTC

TGGATGATTATGATAGTATTGTTTATCCGTAGCATTGGAACAGCTTTTCATACCCCAGCACTCAATGCGGTT

ACACCACTTTTAGTACCAGAAGAACAGCTAACGAAATGCGCAGGCTATAGTCAGTCTTTGCAGTCTATAAGC

TATATTGTTAGTCCGGCAGTTGCAGCACTCTTATACTCCGTTTGGGATTTAAATGCTATTATTGCCATCGAC

GTATTGGGTGCTGTGATTGCATCTATTACGGTAGCAATTGTACGTATACCTAAGCTGGGTAATCAAGTGCAA

AGTTTAGAACCAAATTTCATAAGGGAGATGAAAGAAGGAGTTGTGGTTCTGAGACAAAACAAAGGATTGTTT

GCCTTATTACTCTTAGGAACACTATATACTTTTGTTTATATGCCAATCAATGCACTATTTCCTTTAATAAGC

ATGGAACACTTTAATGGAACGCCTGTGCATATTTCTATTACGGAAATTTCCTTTGCATTTGGGATGCTAGCA

GGAGGCTTATTATTAGGAAGATTAGGGGGCTTCGAAAAGCATGTATTACTAATAACAAGTTCATTTTTTATA

ATGGGGACCAGTTTAGCCGTTTCGGGAATACTTCCTCCAAATGGATTTGTAATATTCGTAGTTTGCTGTGCA

ATAATGGGGCTTTCGGTGCCATTTTATAGCGGTGTGCAAACAGCTCTTTTTCAGGAGAAAATTAAGCCTGAA

TATTTAGGACGTGTATTTTCTTTGATCGGAAGTATCATGTCACTTGCTATGCCAATTGGGTTAATTCTTTCT

GGATTCTTTGCTGATAAAATCGGTGTAAATCATTGGTTTTTACTATCAGGTATTTTAATTATTGGCATTGCT

ATAGTTTGCCAAATGATAACTGAGGTTAGAAAATTAGATTTAAAATAAACAATATTGGAGGAA

ATGTTATGGAATTAATATT

AAAAGCAAAAGACATTCGTGTGGAATTCAAAGGACGCGATGTTTTAGATATAAATGAATTAGAAGTATATGA

TTATGACCGTATTGGTTTAGTAGGAGCAAATGGTGCTGGAAAAAGCACTTTACTCAGGGTACTTTTAGGAGA

ATTAACTCCCCCAGGATGTAAAATGAATCGTCTGGGTGAACTTGCCTATATTCCCCAGTTGGACGAAGTAAC

TCTGCAGGAGGAAAAAGATTTTGCACTTGTAGGCAAGCTAGGTGTTGAGCAATTAAATATACAGACTATGAG

CGGTGGTGAAGAAACAAGGCTTAAAATAGCACAGGCCTTATCGGCACAGGTTCATGGTATTTTAGCGGATGA

ACCTACGAGCCATTTAGACCGTGAAGGAATTGATTTTCTAATAGGACAGCTAAAATATTTTACAGGTGCACT

GTTAGTTATTAGCCATGACCGCTATTTTCTTGATGAAATAGTAGATAAAATATGGGAACTGAAAGATGGCAA

AATCACTGAGTATTGGGGAAACTATTCTGATTATCTTCGTCAGAAAGAGGAAGAACGTAAGAGCCAAGCTGC

AGAATACGAACAATTTATTGCGGAACGTGCCCGATTGGAAAGGGCTGCGGAGGAAAAGCGAAAACAGGCTCG

TAAAATAGAACAGAAGGCAAAAGGTTCTTCAAAGAAAAAAAGTACTGAAGACGGAGGGCGTTTAGCTCATCA

AAAATCAATAGGAAGTAAGGAAAAAAAGATGTATAATGCTGCTAAAACCCTAGAGCACAGGATTGCGGCCTT

AGGAAAAGTAGAAGCTCCGGAAGGCATTCGCAGAATTCGTTTCAGGCAAAGTAAAGCATTGGAGCTCCATAA

TCCATACCCTATAGTCGGTGCAGAAATTAATAAAGTATTTGGGGATAAGGCTCTGTTTGAAAATGCATCTTT

TCAAATTCCGTTAGGAGCAAAAGTGGCGTTAACTGGTGGTAATGGAATCGGAAAAACAACTTTAATCCAAAT

GATCTTAAACCATGAAGAAGGAATTTCTATTTCGCCTAAGGCAAAAATAGGTTACTTTGCACAGAATGGTTA

CAAGTACAACAGTAATCAGAATGTTATGGAGTTTATGCAGAAGGATTGTGACTACAATATATCAGAAATTCG

TTCAGTGCTAGCATCTATGGGGTTCAAACAGAACGATATTGGAAAAAGTTTATCTGTTTTAAGCGGTGGAGA

AATTATAAAATTGTTGCTTGCTAAAATGCTCATGGGTAGATATAACATCCTAATAATGGATGAACCCAGTAA

CTTCCTTGACATACCAAGTTTAGAGGCTTTGGAAATACTAATGAAGGAGTACACCGGAACTATCGTGTTTAT

CACCCACGATAAACGATTACTCGAAAATGTAGCAGATGTAGTTTATGAAATTAGAGATAAGAAAATAAATCT

GAAACATTAAATTTAAGGTAGTCGCTGGTCAGTATAGTCTGTTCTGGTTGGCGACTCCATTGTTAAAGAGTA

TAAAGACTTTAGATTTTATGAATATTAAAAATAGGAACAGTCAATTGAACTGCTCCTATTTTTCTGCTAAAT

ATATTGTAGTTTTCTTATATGTATAATGATAGATTAGCGGATTCTCATCTACGGTACTTACTTCAAATATGA

AGAAGTGATCGCGGTTATCTCTGGACTTTTCCTTATTGAGGACAAAGTAATTCTTACGTGAAGTCGCCATTG

TTTTTAGGATATCATCAGTTAGGAAGGTCAATGGAATATTCATGTTAGAGTAGCGGTAGAAGTCACGTTCAA

AATCTTGGTAGCTCTCGCTATAATAGTCCATTTGTAGGTGATTACGCTGAAACTCAAGCTGATTCATAGAGC

ACCTCCTCGACAAGTTCAATACTAATAATGTCTTTTAATTTCAAATTGATGTGACCTGTTGTAGTTTTTATC

AAAATGAAATCTTTGGTCAGACTTGGTATTGTTCCAGTGTAGGAAACACGCTTGTTTTTTTCAATCACTTGA

ATGCGTGTGCGTAGCTGCCCGGCGTATACTTGACTGAGGAGTAATAATTTCTTCTCTAGTGATAAGTCAGAC

ATGTACGTTACTTTGTTTGTATCATCAGAGAGTGCTGATGCATGTTCAGATAGGAAAAAGCCCATCCATTTT

TGCATCTTTGTATCCTGGTACTCTCTTGCTGATTGAAATGGTAAATATGAACGGTCAATCATATCAAATCCT

TTCTATGCAGAGGCAAGGGTATTTTTATCAAATTGAATCGTAAAACCTTGAATTCCCCCACCTGTGTAACAT

TCTTTAAAGCGATTGATTACCTCAGTATAGATTATCACAGATGAGCTTGTTGGCTTAATGCTAAATGTAAAT

TCCAATGGTAATCGGTTTTCAGATTTAGCATGTACTAGTCGTATCGATATTTCAGTTGTTTTGAGTTTTCTC

TGACGAAGTTTTGAAGTTGCTGTTTCAACGATTCCATGTAAAAATCTTTCAAGCATTTCAATATCATTACAT

CCTTTGCTACGGATTTCTGAAAATTGTACTGTATTTTTTTCTTGTTTCATTTTAATCCCTCCAATCCACCCG

CGGAATGACCACCGATAAGTTTACTGCGTTCAATATTTCTGGAACCTTCAGTTAGGACGGTTCCTTTTTGTA

TGGCTAAAAAACCAAACTGTTCTCTGACAACATCAATAGCTGTCTGAAGTCTATTATCTTTTTCAATTTGTT

CTACATCATCAAAGAGTGATAGTAGAGTATAGCTTTCATCTACGAAGCCACTATAAGATACACCAATTTGTC

TCACTGCACCAGAGGTGTATTTTTTCGGAATAATACAAGTACATGACTCACCATTGTTTTGGGGAGATTTG

CGGGTTCAATTTTATTCTGAGCATTTATAGATTTTTTCATCTCAGTCCTAGAATAGCCAATATGAATAGAAA

CGACAGTAGTCAATACTAGGGCTACTGTTCCGTTCCACAGTATCATTTAAAAATCATT-TTCACACCCTTTC

GTCTATTAGTATAGAAGAAAGCTCTCAGCACA

>GA19074/Mega-2.II

CATGTTGAGGCGGTAAGTTTGCTAGTCAAGGAGTAAAACGACGAAGATTAGCATTTACTTCCGCCCATGCGA

TAGCTGTCCGTGATTGACAAGTGCTAGCACGCAGACAGAACGGAGATAGCGAACCGCTGAGTGTGTCGCTCT

GCTCGTAAAAGCTTAGAAACCTTTGAACGAAAGGGATAATGAAAGCCTTGATTGCAAGGCTTTTTGCTTTAT

GGTGGGTAAGTATCAGAGTGAGAAAATTTTTGGAATGAGTAGAAGTGATAGCTAGAAATTATCAGTTTCTA

TTTCCATTTACCCTGTGGGTACGTGTTTGTTTCCATTGACAAGGAGTTTGTGGGAATAGAAATGT

ACCCACCTTGTTTGAATCAAGTGAAGTGTAGTTGAAGGAAATCTGTTGAAAGCAATACTTCATTTTACCG

AATAAGTAATAATTTAGGCAACTTCAAATCGATTAAAAAAAACTATTTTAAAGGTTAAGAGTAGACAAAA

ATTGTCCACTCTTTTTTGCAAACTCAATTTATCAATAAATGAAATGAGGGAATGTAAAATG

AAATATTTTGAGGTTGAGTTAGAAAATCCTGATGAATTTTTAAAACTACAAACAGAAGATTTTGTGAAAG

CTAATCGCTTGCTACTAAGGAAGATAATCCAGAGCGTTACAGTCTATGAAGAAAACTTCGTCATATCCTT

TAAATCTGGCATCGAATTGGAAGTATGAGTCTCATTCCATAACTTTTATATTGAACATATCATCTTGTTGTG

TTATACTATAAATTGATATAAACAAAGATGTAGGAGGAACCGAAACTATGACAGCCTCAATGCGTTTAAGAT

AAGCTGGCAATAAAAAAAGCAGAATCTATACCCGATGATAGGCTTTTTTGTTGTGCTTATTTATACGATATT

GAGCATTCATTAGTTACGGTGAGGATATTGGTTATTTAACTATACCTTTATTTAACTATGTCTTTAATATGA

ATGTTTCCAAATTGTATGTATGCAGACCAAAAGCCACATTGTGGGGTTTGGCCTGCATTTTTTTTGCCTA

GAATGCTATTCAAAATAGAAATTCAAGCAAAATAATATGCAGGAGATAATATAAATGGAAAAATACAACAAT

TGGAAACGAAAATTTTATGCAATATGGGCAGGGCAAGCAGTATCATTAATCACTAGTGCCATCCTGCAAATG

GCGATTATTTTTTACCTTACAGAAAAAACAGGATCTGCGATGGTCTTGTCTATGGCTTCATTAGTAGGTTTT

TTACCCTATGCGATTTTGGGACCTGCCATTGGTGTGCTAGTGGATCGTCATGATAGGAAGAAGATAATGATT

GGTGCCGATTTAATTATCGCAGCAGCTGGTGCAGTGCTTGCTATTGTTGCATTCTGTATGGAGCTACCTGTC

TGGATGATTATGATAGTATTGTTTATCCGTAGCATTGGAACAGCTTTTCATACCCCAGCACTCAATGCGGTT

ACACCACTTTTAGTACCAGAAGAACAGCTAACGAAATGCGCAGGCTATAGTCAGTCTTTGCAGTCTATAAGC

TATATTGTTAGTCCGGCAGTTGCAGCACTCTTATACTCCGTTTGGGATTTAAATGCTATTATTGCCATCGAC

GTATTGGGTGCTGTGATTGCATCTATTACGGTAGCAATTGTACGTATACCTAAGCTGGGTAATCAAGTGCAA

AGTTTAGAACCAAATTTCATAAGGGAGATGAAAGAAGGAGTTGTGGTTCTGAGACAAAACAAAGGATTGTTT

GCCTTATTACTCTTAGGAACACTATATACTTTTGTTTATATGCCAATCAATGCACTATTTCCTTTAATAAGC

ATGGAACACTTTAATGGAACGCCTGTGCATATTTCTATTACGGAAATTTCCTTTGCATTTGGGATGCTAGCA

GGAGGCTTATTATTAGGAAGATTAGGGGGCTTCGAAAAGCATGTATTACTAATAACAAGTTCATTTTTTATA

ATGGGGACCAGTTTAGCCGTTTCGGGAATACTTCCTCCAAATGGATTTGTAATATTCGTAGTTTGCTGTGCA

ATAATGGGGCTTTCGGTGCCATTTTATAGCGGTGTGCAAACAGCTCTTTTTCAGGAGAAAATTAAGCCTGAA

TATTTAGGACGTGTATTTTCTTTGATCGGAAGTATCATGTCACTTGCTATGCCAATTGGGTTAATTCTTTCT

GGATTCTTTGCTGATAAAATCGGTGTAAATCATTGGTTTTTACTATCAGGTATTTTAATTATTGGCATTGCT

ATAGTTTGCCAAATGATAACTGAGGTTAGAAAATTAGATTTAAAATAAACAATATTGGAGGAA

ATGTTATGGAATTAATATT

AAAAGCAAAAGACATTCGTGTGGAATTCAAAGGACGCGATGTTTTAGATATAAATGAATTAGAAGTATATGA

TTATGACCGTATTGGTTTAGTAGGAGCAAATGGTGCTGGAAAAAGCACTTTACTCAGGGTACTTTTAGGAGA

ATTAACTCCCCCAGGATGTAAAATGAATCGTCTGGGTGAACTTGCCTATATTCCCCAGTTGGACGAAGTAAC

TCTGCAGGAGGAAAAAGATTTTGCACTTGTAGGCAAGCTAGGTGTTGAGCAATTAAATATACAGACTATGAG

CGGTGGTGAAGAAACAAGGCTTAAAATAGCACAGGCCTTATCGGCACAGGTTCATGGTATTTTAGCGGATGA

ACCTACGAGCCATTTAGACCGTGAAGGAATTGATTTTCTAATAGGACAGCTAAAATATTTTACAGGTGCACT

GTTAGTTATTAGCCATGACCGCTATTTTCTTGATGAAATAGTAGATAAAATATGGGAACTGAAAGATGGCAA

AATCACTGAGTATTGGGGAAACTATTCTGATTATCTTCGTCAGAAAGAGGAAGAACGTAAGAGCCAAGCTGC

AGAATACGAACAATTTATTGCGGAACGTGCCCGATTGGAAAGGGCTGCGGAGGAAAAGCGAAAACAGGCTCG

TAAAATAGAACAGAAGGCAAAAGGTTCTTCAAAGAAAAAAAGTACTGAAGACGGAGGGCGTTTAGCTCATCA

AAAATCAATAGGAAGTAAGGAAAAAAAGATGTATAATGCTGCTAAAACCCTAGAGCACAGGATTGCGGCCTT

AGGAAAAGTAGAAGCTCCGGAAGGCATTCGCAGAATTCGTTTCAGGCAAAGTAAAGCATTGGAGCTCCATAA

TCCATACCCTATAGTCGGTGCAGAAATTAATAAAGTATTTGGGGATAAGGCTCTGTTTGAAAATGCATCTTT

TCAAATTCCGTTAGGAGCAAAAGTGGCGTTAACTGGTGGTAATGGAATCGGAAAAACAACTTTAATCCAAAT

GATCTTAAACCATGAAGAAGGAATTTCTATTTCGCCTAAGGCAAAAATAGGTTACTTTGCACAGAATGGTTA

CAAGTACAACAGTAATCAGAATGTTATGGAGTTTATGCAGAAGGATTGTGACTACAATATATCAGAAATTCG

TTCAGTGCTAGCATCTATGGGGTTCAAACAGAACGATATTGGAAAAAGTTTATCTGTTTTAAGCGGTGGAGA

AATTATAAAATTGTTGCTTGCTAAAATGCTCATGGGTAGATATAACATCCTAATAATGGATGAACCCAGTAA

CTTCCTTGACATACCAAGTTTAGAGGCTTTGGAAATACTAATGAAGGAGTACACCGGAACTATCGTGTTTAT

CACCCACGATAAACGATTACTCGAAAATGTAGCAGATGTAGTTTATGAAATTAGAGATAAGAAAATAAATCT

GAAACATTAAATTTAAGGTAGTCGCTGGTCAGTATAGTCTGTTCTGGTTGGCGACTCCATTGTTAAAGAGTA

TAAAGACTTTAGATTTTATGAATATTAAAAATAGGAACAGTCAATTGAACTGCTCCTATTTTTCTGCTAAAT

ATATTGTAGTTTTCTTATATGTATAATGATAGATTAGCGGATTCTCATCTACGGTACTTACTTCAAATATGA

AGAAGTGATCGCGGTTATCTCTGGACTTTTCCTTATTGAGGACAAAGTAATTCTTACGTGAAGTCGCCATTG

TTTTTAGGATATCATCAGTTAGGAAGGTCAATGGAATATTCATGTTAGAGTAGCGGTAGAAGTCACGTTCAA

AATCTTGGTAGCTCTCGCTATAATAGTCCATTTGTAGGTGATTACGCTGAAACTCAAGCTGATTCATAGAGC

ACCTCCTCGACAAGTTCAATACTAATAATGTCTTTTAATTTCAAATTGATGTGACCTGTTGTAGTTTTTATC

AAAATGAAATCTTTGGTCAGACTTGGTATTGTTCCAGTGTAGGAAACACGCTTGTTTTTTTCAATCACTTGA

ATGCGTGTGCGTAGCTGCCCGGCGTATACTTGACTGAGGAGTAATAATTTCTTCTCTAGTGATAAGTCAGAC

ATGTACGTTACTTTGTTTGTATCATCAGAGAGTGCTGATGCATGTTCAGATAGGAAAAAGCCCATCCATTTT

TGCATCTTTGTATCCTGGTACTCTCTTGCTGATTGAAATGGTAAATATGAACGGTCAATCATATCAAATCCT

TTCTATGCAGAGGCAAGGGTATTTTTATCAAATTGAATCGTAAAACCTTGAATTCCCCCACCTGTGTAACAT

TCTTTAAAGCGATTGATTACCTCAGTATAGATTATCACAGATGAGCTTGTTGGCTTAATGCTAAATGTAAAT

TCCAATGGTAATCGGTTTTCAGATTTAGCATGTACTAGTCGTATCGATATTTCAGTTGTTTTGAGTTTTCTC

TGACGAAGTTTTGAAGTTGCTGTTTCAACGATTCCATGTAAAAATCTTTCAAGCATTTCAATATCATTACAT

CCTTTGCTACGGATTTCTGAAAATTGTACTGTATTTTTTTCTTGTTTCATTTTAATCCCTCCAATCCACCCG

CGGAATGACCACCGATAAGTTTACTGCGTTCAATATTTCTGGAACCTTCAGTTAGGACGGTTCCTTTTTGTA

TGGCTAAAAAACCAAACTGTTCTCTGACAACATCAATAGCTGTCTGAAGTCTATTATCTTTTTCAATTTGTT

CTACATCATCAAAGAGTGATAGTAGAGTATAGCTTTCATCTACGAAGCCACTATAAGATACACCAATTTGTC

TCACTGCACCAGAGGTGTATTTTTTCGGAATAATACAAGTACATGACTCACCATTGTTTTGGGGAGATTTG

CGGGTTCAATTTTATTCTGAGCATTTATAGATTTTTTCATCTCAGTCCTAGAATAGCCAATATGAATAGAAA

CGACAGTAGTCAATACTAGGGCTACTGTTCCGTTCCACAGTATCATTTAAAAATCATT-TTCACACCCTTTC

GTCTATTAGTATAGAAGAAAGCTCTCAGCACA

>GA41410/Mega-2.II

CATGTTGAGGCGGTAAGTTTGCTAGTCAAGGAGTAAAACGACGAAGATTAGCATTTACTTCCGCCCATGCGA

TAGCTGTCCGTGATTGACAAGTGCTAGCACGCAGACAGAACGGAGATAGCGAACCGCTGAGTGTGTCGCTCT

GCTCGTAAAAGCTTAGAAACCTTTGAACGAAAGGGATAATGAAAGCCTTGATTGCAAGGCTTTTTGCTTTAT

GGTGGGTAAGTATCAGAGTGAGAAAATTTTTGGAATGAGTAGAAGTGATAGCTAGAAATTATCAGTTTCTA

TTTCCATTTACCCTGTGGGTACGTGTTTGTTTCCATTGACAAGGAGTTTGTGGGAATAGAAATGT

ACCCACCTTGTTTGAATCAAGTGAAGTGTAGTTGAAGGAAATCTGTTGAAAGCAATACTTCATTTTACCG

AATAAGTAATAATTTAGGCAACTTCAAATCGATTAAAAAAAACTATTTTAAAGGTTAAGAGTAGACAAAA

ATTGTCCACTCTTTTTTGCAAACTCAATTTATCAATAAATGAAATGAGGGAATGTAAAATG

AAATATTTTGAGGTTGAGTTAGAAAATCCTGATGAATTTTTAAAACTACAAACAGAAGATTTTGTGAAAG

CTAATCGCTTGCTACTAAGGAAGATAATCCAGAGCGTTACAGTCTATGAAGAAAACTTCGTCATATCCTT

TAAATCTGGCATCGAATTGGAAGTATGAGTCTCATTCCATAACTTTTATATTGAACATATCATCTTGTTGTG

TTATACTATAAATTGATATAAACAAAGATGTAGGAGGAACCGAAACTATGACAGCCTCAATGCGTTTAAGAT

AAGCTGGCAATAAAAAAAGCAGAATCTATACCCGATGATAGGCTTTTTTGTTGTGCTTATTTATACGATATT

GAGCATTCATTAGTTACGGTGAGGATATTGGTTATTTAACTATACCTTTATTTAACTATGTCTTTAATATGA

ATGTTTCCAAATTGTATGTATGCAGACCAAAAGCCACATTGTGGGGTTTGGCCTGCATTTTTTTTGCCTA

GAATGCTATTCAAAATAGAAATTCAAGCAAAATAATATGCAGGAGATAATATAAATGGAAAAATACAACAAT

TGGAAACGAAAATTTTATGCAATATGGGCAGGGCAAGCAGTATCATTAATCACTAGTGCCATCCTGCAAATG

GCGATTATTTTTTACCTTACAGAAAAAACAGGATCTGCGATGGTCTTGTCTATGGCTTCATTAGTAGGTTTT

TTACCCTATGCGATTTTGGGACCTGCCATTGGTGTGCTAGTGGATCGTCATGATAGGAAGAAGATAATGATT

GGTGCCGATTTAATTATCGCAGCAGCTGGTGCAGTGCTTGCTATTGTTGCATTCTGTATGGAGCTACCTGTC

TGGATGATTATGATAGTATTGTTTATCCGTAGCATTGGAACAGCTTTTCATACCCCAGCACTCAATGCGGTT

ACACCACTTTTAGTACCAGAAGAACAGCTAACGAAATGCGCAGGCTATAGTCAGTCTTTGCAGTCTATAAGC

TATATTGTTAGTCCGGCAGTTGCAGCACTCTTATACTCCGTTTGGGATTTAAATGCTATTATTGCCATCGAC

GTATTGGGTGCTGTGATTGCATCTATTACGGTAGCAATTGTACGTATACCTAAGCTGGGTAATCAAGTGCAA

AGTTTAGAACCAAATTTCATAAGGGAGATGAAAGAAGGAGTTGTGGTTCTGAGACAAAACAAAGGATTGTTT

GCCTTATTACTCTTAGGAACACTATATACTTTTGTTTATATGCCAATCAATGCACTATTTCCTTTAATAAGC

ATGGAACACTTTAATGGAACGCCTGTGCATATTTCTATTACGGAAATTTCCTTTGCATTTGGGATGCTAGCA

GGAGGCTTATTATTAGGAAGATTAGGGGGCTTCGAAAAGCATGTATTACTAATAACAAGTTCATTTTTTATA

ATGGGGACCAGTTTAGCCGTTTCGGGAATACTTCCTCCAAATGGATTTGTAATATTCGTAGTTTGCTGTGCA

ATAATGGGGCTTTCGGTGCCATTTTATAGCGGTGTGCAAACAGCTCTTTTTCAGGAGAAAATTAAGCCTGAA

TATTTAGGACGTGTATTTTCTTTGATCGGAAGTATCATGTCACTTGCTATGCCAATTGGGTTAATTCTTTCT

GGATTCTTTGCTGATAAAATCGGTGTAAATCATTGGTTTTTACTATCAGGTATTTTAATTATTGGCATTGCT

ATAGTTTGCCAAATGATAACTGAGGTTAGAAAATTAGATTTAAAATAAACAATATTGGAGGAA

ATGTTATGGAATTAATATT

AAAAGCAAAAGACATTCGTGTGGAATTCAAAGGACGCGATGTTTTAGATATAAATGAATTAGAAGTATATGA

TTATGACCGTATTGGTTTAGTAGGAGCAAATGGTGCTGGAAAAAGCACTTTACTCAGGGTACTTTTAGGAGA

ATTAACTCCCCCAGGATGTAAAATGAATCGTCTGGGTGAACTTGCCTATATTCCCCAGTTGGACGAAGTAAC

TCTGCAGGAGGAAAAAGATTTTGCACTTGTAGGCAAGCTAGGTGTTGAGCAATTAAATATACAGACTATGAG

CGGTGGTGAAGAAACAAGGCTTAAAATAGCACAGGCCTTATCGGCACAGGTTCATGGTATTTTAGCGGATGA

ACCTACGAGCCATTTAGACCGTGAAGGAATTGATTTTCTAATAGGACAGCTAAAATATTTTACAGGTGCACT

GTTAGTTATTAGCCATGACCGCTATTTTCTTGATGAAATAGTAGATAAAATATGGGAACTGAAAGATGGCAA

AATCACTGAGTATTGGGGAAACTATTCTGATTATCTTCGTCAGAAAGAGGAAGAACGTAAGAGCCAAGCTGC

AGAATACGAACAATTTATTGCGGAACGTGCCCGATTGGAAAGGGCTGCGGAGGAAAAGCGAAAACAGGCTCG

TAAAATAGAACAGAAGGCAAAAGGTTCTTCAAAGAAAAAAAGTACTGAAGACGGAGGGCGTTTAGCTCATCA

AAAATCAATAGGAAGTAAGGAAAAAAAGATGTATAATGCTGCTAAAACCCTAGAGCACAGGATTGCGGCCTT

AGGAAAAGTAGAAGCTCCGGAAGGCATTCGCAGAATTCGTTTCAGGCAAAGTAAAGCATTGGAGCTCCATAA

TCCATACCCTATAGTCGGTGCAGAAATTAATAAAGTATTTGGGGATAAGGCTCTGTTTGAAAATGCATCTTT

TCAAATTCCGTTAGGAGCAAAAGTGGCGTTAACTGGTGGTAATGGAATCGGAAAAACAACTTTAATCCAAAT

GATCTTAAACCATGAAGAAGGAATTTCTATTTCGCCTAAGGCAAAAATAGGTTACTTTGCACAGAATGGTTA

CAAGTACAACAGTAATCAGAATGTTATGGAGTTTATGCAGAAGGATTGTGACTACAATATATCAGAAATTCG

TTCAGTGCTAGCATCTATGGGGTTCAAACAGAACGATATTGGAAAAAGTTTATCTGTTTTAAGCGGTGGAGA

AATTATAAAATTGTTGCTTGCTAAAATGCTCATGGGTAGATATAACATCCTAATAATGGATGAACCCAGTAA

CTTCCTTGACATACCAAGTTTAGAGGCTTTGGAAATACTAATGAAGGAGTACACCGGAACTATCGTGTTTAT

CACCCACGATAAACGATTACTCGAAAATGTAGCAGATGTAGTTTATGAAATTAGAGATAAGAAAATAAATCT

GAAACATTAAATTTAAGGTAGTCGCTGGTCAGTATAGTCTGTTCTGGTTGGCGACTCCATTGTTAAAGAGTA

TAAAGACTTTAGATTTTATGAATATTAAAAATAGGAACAGTCAATTGAACTGCTCCTATTTTTCTGCTAAAT

ATATTGTAGTTTTCTTATATGTATAATGATAGATTAGCGGATTCTCATCTACGGTACTTACTTCAAATATGA

AGAAGTGATCGCGGTTATCTCTGGACTTTTCCTTATTGAGGACAAAGTAATTCTTACGTGAAGTCGCCATTG

TTTTTAGGATATCATCAGTTAGGAAGGTCAATGGAATATTCATGTTAGAGTAGCGGTAGAAGTCACGTTCAA

AATCTTGGTAGCTCTCGCTATAATAGTCCATTTGTAGGTGATTACGCTGAAACTCAAGCTGATTCATAGAGC

ACCTCCTCGACAAGTTCAATACTAATAATGTCTTTTAATTTCAAATTGATGTGACCTGTTGTAGTTTTTATC

AAAATGAAATCTTTGGTCAGACTTGGTATTGTTCCAGTGTAGGAAACACGCTTGTTTTTTTCAATCACTTGA

ATGCGTGTGCGTAGCTGCCCGGCGTATACTTGACTGAGGAGTAATAATTTCTTCTCTAGTGATAAGTCAGAC

ATGTACGTTACTTTGTTTGTATCATCAGAGAGTGCTGATGCATGTTCAGATAGGAAAAAGCCCATCCATTTT

TGCATCTTTGTATCCTGGTACTCTCTTGCTGATTGAAATGGTAAATATGAACGGTCAATCATATCAAATCCT

TTCTATGCAGAGGCAAGGGTATTTTTATCAAATTGAATCGTAAAACCTTGAATTCCCCCACCTGTGTAACAT

TCTTTAAAGCGATTGATTACCTCAGTATAGATTATCACAGATGAGCTTGTTGGCTTAATGCTAAATGTAAAT

TCCAATGGTAATCGGTTTTCAGATTTAGCATGTACTAGTCGTATCGATATTTCAGTTGTTTTGAGTTTTCTC

TGACGAAGTTTTGAAGTTGCTGTTTCAACGATTCCATGTAAAAATCTTTCAAGCATTTCAATATCATTACAT

CCTTTGCTACGGATTTCTGAAAATTGTACTGTATTTTTTTCTTGTTTCATTTTAATCCCTCCAATCCACCCG

CGGAATGACCACCGATAAGTTTACTGCGTTCAATATTTCTGGAACCTTCAGTTAGGACGGTTCCTTTTTGTA

TGGCTAAAAAACCAAACTGTTCTCTGACAACATCAATAGCTGTCTGAAGTCTATTATCTTTTTCAATTTGTT

CTACATCATCAAAGAGTGATAGTAGAGTATAGCTTTCATCTACGAAGCCACTATAAGATACACCAATTTGTC

TCACTGCACCAGAGGTGTATTTTTTCGGAATAATACAAGTACATGACTCACCATTGTTTTGGGGAGATTTG

CGGGTTCAATTTTATTCTGAGCATTTATAGATTTTTTCATCTCAGTCCTAGAATAGCCAATATGAATAGAAA

CGACAGTAGTCAATACTAGGGCTACTGTTCCGTTCCACAGTATCATTTAAAAATCATT-TTCACACCCTTTC

GTCTATTAGTATAGAAGAAAGCTCTCAGCACA

>GA47368/Mega-2.II

CATGTTGAGGCGGTAAGTTTGCTAGTCAAGGAGTAAAACGACGAAGATTAGCATTTACTTCCGCCCATGCGA

TAGCTGTCCGTGATTGACAAGTGCTAGCACGCAGACAGAACGGAGATAGCGAACCGCTGAGTGTGTCGCTCT

GCTCGTAAAAGCTTAGAAACCTTTGAACGAAAGGGATAATGAAAGCCTTGATTGCAAGGCTTTTTGCTTTAT

GGTGGGTAAGTATCAGAGTGAGAAAATTTTTGGAATGAGTAGAAGTGATAGCTAGAAATTATCAGTTTCTA

TTTCCATTTACCCTGTGGGTACGTGTTTGTTTCCATTGACAAGGAGTTTGTGGGAATAGAAATGT

ACCCACCTTGTTTGAATCAAGTGAAGTGTAGTTGAAGGAAATCTGTTGAAAGCAATACTTCATTTTACCG

AATAAGTAATAATTTAGGCAACTTCAAATCGATTAAAAAAAACTATTTTAAAGGTTAAGAGTAGACAAAA

ATTGTCCACTCTTTTTTGCAAACTCAATTTATCAATAAATGAAATGAGGGAATGTAAAATG

AAATATTTTGAGGTTGAGTTAGAAAATCCTGATGAATTTTTAAAACTACAAACAGAAGATTTTGTGAAAG

CTAATCGCTTGCTACTAAGGAAGATAATCCAGAGCGTTACAGTCTATGAAGAAAACTTCGTCATATCCTT

TAAATCTGGCATCGAATTGGAAGTATGAGTCTCATTCCATAACTTTTATATTGAACATATCATCTTGTTGTG

TTATACTATAAATTGATATAAACAAAGATGTAGGAGGAACCGAAACTATGACAGCCTCAATGCGTTTAAGAT

AAGCTGGCAATAAAAAAAGCAGAATCTATACCCGATGATAGGCTTTTTTGTTGTGCTTATTTATACGATATT

GAGCATTCATTAGTTACGGTGAGGATATTGGTTATTTAACTATACCTTTATTTAACTATGTCTTTAATATGA

ATGTTTCCAAATTGTATGTATGCAGACCAAAAGCCACATTGTGGGGTTTGGCCTGCATTTTTTTTGCCTA

GAATGCTATTCAAAATAGAAATTCAAGCAAAATAATATGCAGGAGATAATATAAATGGAAAAATACAACAAT

TGGAAACGAAAATTTTATGCAATATGGGCAGGGCAAGCAGTATCATTAATCACTAGTGCCATCCTGCAAATG

GCGATTATTTTTTACCTTACAGAAAAAACAGGATCTGCGATGGTCTTGTCTATGGCTTCATTAGTAGGTTTT

TTACCCTATGCGATTTTGGGACCTGCCATTGGTGTGCTAGTGGATCGTCATGATAGGAAGAAGATAATGATT

GGTGCCGATTTAATTATCGCAGCAGCTGGTGCAGTGCTTGCTATTGTTGCATTCTGTATGGAGCTACCTGTC

TGGATGATTATGATAGTATTGTTTATCCGTAGCATTGGAACAGCTTTTCATACCCCAGCACTCAATGCGGTT

ACACCACTTTTAGTACCAGAAGAACAGCTAACGAAATGCGCAGGCTATAGTCAGTCTTTGCAGTCTATAAGC

TATATTGTTAGTCCGGCAGTTGCAGCACTCTTATACTCCGTTTGGGATTTAAATGCTATTATTGCCATCGAC

GTATTGGGTGCTGTGATTGCATCTATTACGGTAGCAATTGTACGTATACCTAAGCTGGGTAATCAAGTGCAA

AGTTTAGAACCAAATTTCATAAGGGAGATGAAAGAAGGAGTTGTGGTTCTGAGACAAAACAAAGGATTGTTT

GCCTTATTACTCTTAGGAACACTATATACTTTTGTTTATATGCCAATCAATGCACTATTTCCTTTAATAAGC

ATGGAACACTTTAATGGAACGCCTGTGCATATTTCTATTACGGAAATTTCCTTTGCATTTGGGATGCTAGCA

GGAGGCTTATTATTAGGAAGATTAGGGGGCTTCGAAAAGCATGTATTACTAATAACAAGTTCATTTTTTATA

ATGGGGACCAGTTTAGCCGTTTCGGGAATACTTCCTCCAAATGGATTTGTAATATTCGTAGTTTGCTGTGCA

ATAATGGGGCTTTCGGTGCCATTTTATAGCGGTGTGCAAACAGCTCTTTTTCAGGAGAAAATTAAGCCTGAA

TATTTAGGACGTGTATTTTCTTTGATCGGAAGTATCATGTCACTTGCTATGCCAATTGGGTTAATTCTTTCT

GGATTCTTTGCTGATAAAATCGGTGTAAATCATTGGTTTTTACTATCAGGTATTTTAATTATTGGCATTGCT

ATAGTTTGCCAAATGATAACTGAGGTTAGAAAATTAGATTTAAAATAAACAATATTGGAGGAA

ATGTTATGGAATTAATATT

AAAAGCAAAAGACATTCGTGTGGAATTCAAAGGACGCGATGTTTTAGATATAAATGAATTAGAAGTATATGA

TTATGACCGTATTGGTTTAGTAGGAGCAAATGGTGCTGGAAAAAGCACTTTACTCAGGGTACTTTTAGGAGA

ATTAACTCCCCCAGGATGTAAAATGAATCGTCTGGGTGAACTTGCCTATATTCCCCAGTTGGACGAAGTAAC

TCTGCAGGAGGAAAAAGATTTTGCACTTGTAGGCAAGCTAGGTGTTGAGCAATTAAATATACAGACTATGAG

CGGTGGTGAAGAAACAAGGCTTAAAATAGCACAGGCCTTATCGGCACAGGTTCATGGTATTTTAGCGGATGA

ACCTACGAGCCATTTAGACCGTGAAGGAATTGATTTTCTAATAGGACAGCTAAAATATTTTACAGGTGCACT

GTTAGTTATTAGCCATGACCGCTATTTTCTTGATGAAATAGTAGATAAAATATGGGAACTGAAAGATGGCAA

AATCACTGAGTATTGGGGAAACTATTCTGATTATCTTCGTCAGAAAGAGGAAGAACGTAAGAGCCAAGCTGC

AGAATACGAACAATTTATTGCGGAACGTGCCCGATTGGAAAGGGCTGCGGAGGAAAAGCGAAAACAGGCTCG

TAAAATAGAACAGAAGGCAAAAGGTTCTTCAAAGAAAAAAAGTACTGAAGACGGAGGGCGTTTAGCTCATCA

AAAATCAATAGGAAGTAAGGAAAAAAAGATGTATAATGCTGCTAAAACCCTAGAGCACAGGATTGCGGCCTT

AGGAAAAGTAGAAGCTCCGGAAGGCATTCGCAGAATTCGTTTCAGGCAAAGTAAAGCATTGGAGCTCCATAA

TCCATACCCTATAGTCGGTGCAGAAATTAATAAAGTATTTGGGGATAAGGCTCTGTTTGAAAATGCATCTTT

TCAAATTCCGTTAGGAGCAAAAGTGGCGTTAACTGGTGGTAATGGAATCGGAAAAACAACTTTAATCCAAAT

GATCTTAAACCATGAAGAAGGAATTTCTATTTCGCCTAAGGCAAAAATAGGTTACTTTGCACAGAATGGTTA

CAAGTACAACAGTAATCAGAATGTTATGGAGTTTATGCAGAAGGATTGTGACTACAATATATCAGAAATTCG

TTCAGTGCTAGCATCTATGGGGTTCAAACAGAACGATATTGGAAAAAGTTTATCTGTTTTAAGCGGTGGAGA

AATTATAAAATTGTTGCTTGCTAAAATGCTCATGGGTAGATATAACATCCTAATAATGGATGAACCCAGTAA

CTTCCTTGACATACCAAGTTTAGAGGCTTTGGAAATACTAATGAAGGAGTACACCGGAACTATCGTGTTTAT

CACCCACGATAAACGATTACTCGAAAATGTAGCAGATGTAGTTTATGAAATTAGAGATAAGAAAATAAATCT

GAAACATTAAATTTAAGGTAGTCGCTGGTCAGTATAGTCTGTTCTGGTTGGCGACTCCATTGTTAAAGAGTA

TAAAGACTTTAGATTTTATGAATATTAAAAATAGGAACAGTCAATTGAACTGCTCCTATTTTTCTGCTAAAT

ATATTGTAGTTTTCTTATATGTATAATGATAGATTAGCGGATTCTCATCTACGGTACTTACTTCAAATATGA

AGAAGTGATCGCGGTTATCTCTGGACTTTTCCTTATTGAGGACAAAGTAATTCTTACGTGAAGTCGCCATTG

TTTTTAGGATATCATCAGTTAGGAAGGTCAATGGAATATTCATGTTAGAGTAGCGGTAGAAGTCACGTTCAA

AATCTTGGTAGCTCTCGCTATAATAGTCCATTTGTAGGTGATTACGCTGAAACTCAAGCTGATTCATAGAGC

ACCTCCTCGACAAGTTCAATACTAATAATGTCTTTTAATTTCAAATTGATGTGACCTGTTGTAGTTTTTATC

AAAATGAAATCTTTGGTCAGACTTGGTATTGTTCCAGTGTAGGAAACACGCTTGTTTTTTTCAATCACTTGA

ATGCGTGTGCGTAGCTGCCCGGCGTATACTTGACTGAGGAGTAATAATTTCTTCTCTAGTGATAAGTCAGAC

ATGTACGTTACTTTGTTTGTATCATCAGAGAGTGCTGATGCATGTTCAGATAGGAAAAAGCCCATCCATTTT

TGCATCTTTGTATCCTGGTACTCTCTTGCTGATTGAAATGGTAAATATGAACGGTCAATCATATCAAATCCT

TTCTATGCAGAGGCAAGGGTATTTTTATCAAATTGAATCGTAAAACCTTGAATTCCCCCACCTGTGTAACAT

TCTTTAAAGCGATTGATTACCTCAGTATAGATTATCACAGATGAGCTTGTTGGCTTAATGCTAAATGTAAAT

TCCAATGGTAATCGGTTTTCAGATTTAGCATGTACTAGTCGTATCGATATTTCAGTTGTTTTGAGTTTTCTC

TGACGAAGTTTTGAAGTTGCTGTTTCAACGATTCCATGTAAAAATCTTTCAAGCATTTCAATATCATTACAT

CCTTTGCTACGGATTTCTGAAAATTGTACTGTATTTTTTTCTTGTTTCATTTTAATCCCTCCAATCCACCCG

CGGAATGACCACCGATAAGTTTACTGCGTTCAATATTTCTGGAACCTTCAGTTAGGACGGTTCCTTTTTGTA

TGGCTAAAAAACCAAACTGTTCTCTGACAACATCAATAGCTGTCTGAAGTCTATTATCTTTTTCAATTTGTT

CTACATCATCAAAGAGTGATAGTAGAGTATAGCTTTCATCTACGAAGCCACTATAAGATACACCAATTTGTC

TCACTGCACCAGAGGTGTATTTTTTCGGAATAATACAAGTACATGACTCACCATTGTTTTGGGGAGATTTG

CGGGTTCAATTTTATTCTGAGCATTTATAGATTTTTTCATCTCAGTCCTAGAATAGCCAATATGAATAGAAA

CGACAGTAGTCAATACTAGGGCTACTGTTCCGTTCCACAGTATCATTTAAAAATCATT-TTCACACCCTTTC

GTCTATTAGTATAGAAGAAAGCTCTCAGCACA

>GA13338/Mega-2.II

CATGTTGAGGCGGTAAGTTTGCTAGTCAAGGAGTAAAACGACGAAGATTAGCATTTACTTCCGCCCATGCGA

TAGCTGTCCGTGATTGACAAGTGCTAGCACGCAGACAGAACGGAGATAGCGAACCGCTGAGTGTGTCGCTCT

GCTCGTAAAAGCTTAGAAACCTTTGAACGAAAGGGATAATGAAAGCCTTGATTGCAAGGCTTTTTGCTTTAT

GGTGGGTAAGTATCAGAGTGAGAAAATTTTTGGAATGAGTAGAAGTGATAGCTAGAAATTATCAGTTTCTA

TTTCCATTTACCCTGTGGGTACGTGTTTGTTTCCATTGACAAGGAGTTTGTGGGAATAGAAATGT

ACCCACCTTGTTTGAATCAAGTGAAGTGTAGTTGAAGGAAATCTGTTGAAAGCAATACTTCATTTTACCG

AATAAGTAATAATTTAGGCAACTTCAAATCGATTAAAAAAAACTATTTTAAAGGTTAAGAGTAGACAAAA

ATTGTCCACTCTTTTTTGCAAACTCAATTTATCAATAAATGAAATGAGGGAATGTAAAATG

AAATATTTTGAGGTTGAGTTAGAAAATCCTGATGAATTTTTAAAACTACAAACAGAAGATTTTGTGAAAG

CTAATCGCTTGCTACTAAGGAAGATAATCCAGAGCGTTACAGTCTATGAAGAAAACTTCGTCATATCCTT

TAAATCTGGCATCGAATTGGAAGTATGAGTCTCATTCCATAACTTTTATATTGAACATATCATCTTGTTGTG

TTATACTATAAATTGATATAAACAAAGATGTAGGAGGAACCGAAACTATGACAGCCTCAATGCGTTTAAGAT

AAGCTGGCAATAAAAAAAGCAGAATCTATACCCGATGATAGGCTTTTTTGTTGTGCTTATTTATACGATATT

GAGCATTCATTAGTTACGGTGAGGATATTGGTTATTTAACTATACCTTTATTTAACTATGTCTTTAATATGA

ATGTTTCCAAATTGTATGTATGCAGACCAAAAGCCACATTGTGGGGTTTGGCCTGCATTTTTTTTGCCTA

GAATGCTATTCAAAATAGAAATTCAAGCAAAATAATATGCAGGAGATAATATAAATGGAAAAATACAACAAT

TGGAAACGAAAATTTTATGCAATATGGGCAGGGCAAGCAGTATCATTAATCACTAGTGCCATCCTGCAAATG

GCGATTATTTTTTACCTTACAGAAAAAACAGGATCTGCGATGGTCTTGTCTATGGCTTCATTAGTAGGTTTT

TTACCCTATGCGATTTTGGGACCTGCCATTGGTGTGCTAGTGGATCGTCATGATAGGAAGAAGATAATGATT

GGTGCCGATTTAATTATCGCAGCAGCTGGTGCAGTGCTTGCTATTGTTGCATTCTGTATGGAGCTACCTGTC

TGGATGATTATGATAGTATTGTTTATCCGTAGCATTGGAACAGCTTTTCATACCCCAGCACTCAATGCGGTT

ACACCACTTTTAGTACCAGAAGAACAGCTAACGAAATGCGCAGGCTATAGTCAGTCTTTGCAGTCTATAAGC

TATATTGTTAGTCCGGCAGTTGCAGCACTCTTATACTCCGTTTGGGATTTAAATGCTATTATTGCCATCGAC

GTATTGGGTGCTGTGATTGCATCTATTACGGTAGCAATTGTACGTATACCTAAGCTGGGTAATCAAGTGCAA

AGTTTAGAACCAAATTTCATAAGGGAGATGAAAGAAGGAGTTGTGGTTCTGAGACAAAACAAAGGATTGTTT

GCCTTATTACTCTTAGGAACACTATATACTTTTGTTTATATGCCAATCAATGCACTATTTCCTTTAATAAGC

ATGGAACACTTTAATGGAACGCCTGTGCATATTTCTATTACGGAAATTTCCTTTGCATTTGGGATGCTAGCA

GGAGGCTTATTATTAGGAAGATTAGGGGGCTTCGAAAAGCATGTATTACTAATAACAAGTTCATTTTTTATA

ATGGGGACCAGTTTAGCCGTTTCGGGAATACTTCCTCCAAATGGATTTGTAATATTCGTAGTTTGCTGTGCA

ATAATGGGGCTTTCGGTGCCATTTTATAGCGGTGTGCAAACAGCTCTTTTTCAGGAGAAAATTAAGCCTGAA

TATTTAGGACGTGTATTTTCTTTGATCGGAAGTATCATGTCACTTGCTATGCCAATTGGGTTAATTCTTTCT

GGATTCTTTGCTGATAAAATCGGTGTAAATCATTGGTTTTTACTATCAGGTATTTTAATTATTGGCATTGCT

ATAGTTTGCCAAATGATAACTGAGGTTAGAAAATTAGATTTAAAATAAACAATATTGGAGGAA

ATGTTATGGAATTAATATT

AAAAGCAAAAGACATTCGTGTGGAATTCAAAGGACGCGATGTTTTAGATATAAATGAATTAGAAGTATATGA

TTATGACCGTATTGGTTTAGTAGGAGCAAATGGTGCTGGAAAAAGCACTTTACTCAGGGTACTTTTAGGAGA

ATTAACTCCCCCAGGATGTAAAATGAATCGTCTGGGTGAACTTGCCTATATTCCCCAGTTGGACGAAGTAAC

TCTGCAGGAGGAAAAAGATTTTGCACTTGTAGGCAAGCTAGGTGTTGAGCAATTAAATATACAGACTATGAG

CGGTGGTGAAGAAACAAGGCTTAAAATAGCACAGGCCTTATCGGCACAGGTTCATGGTATTTTAGCGGATGA

ACCTACGAGCCATTTAGACCGTGAAGGAATTGATTTTCTAATAGGACAGCTAAAATATTTTACAGGTGCACT

GTTAGTTATTAGCCATGACCGCTATTTTCTTGATGAAATAGTAGATAAAATATGGGAACTGAAAGATGGCAA

AATCACTGAGTATTGGGGAAACTATTCTGATTATCTTCGTCAGAAAGAGGAAGAACGTAAGAGCCAAGCTGC

AGAATACGAACAATTTATTGCGGAACGTGCCCGATTGGAAAGGGCTGCGGAGGAAAAGCGAAAACAGGCTCG

TAAAATAGAACAGAAGGCAAAAGGTTCTTCAAAGAAAAAAAGTACTGAAGACGGAGGGCGTTTAGCTCATCA

AAAATCAATAGGAAGTAAGGAAAAAAAGATGTATAATGCTGCTAAAACCCTAGAGCACAGGATTGCGGCCTT

AGGAAAAGTAGAAGCTCCGGAAGGCATTCGCAGAATTCGTTTCAGGCAAAGTAAAGCATTGGAGCTCCATAA

TCCATACCCTATAGTCGGTGCAGAAATTAATAAAGTATTTGGGGATAAGGCTCTGTTTGAAAATGCATCTTT

TCAAATTCCGTTAGGAGCAAAAGTGGCGTTAACTGGTGGTAATGGAATCGGAAAAACAACTTTAATCCAAAT

GATCTTAAACCATGAAGAAGGAATTTCTATTTCGCCTAAGGCAAAAATAGGTTACTTTGCACAGAATGGTTA

CAAGTACAACAGTAATCAGAATGTTATGGAGTTTATGCAGAAGGATTGTGACTACAATATATCAGAAATTCG

TTCAGTGCTAGCATCTATGGGGTTCAAACAGAACGATATTGGAAAAAGTTTATCTGTTTTAAGCGGTGGAGA

AATTATAAAATTGTTGCTTGCTAAAATGCTCATGGGTAGATATAACATCCTAATAATGGATGAACCCAGTAA

CTTCCTTGACATACCAAGTTTAGAGGCTTTGGAAATACTAATGAAGGAGTACACCGGAACTATCGTGTTTAT

CACCCACGATAAACGATTACTCGAAAATGTAGCAGATGTAGTTTATGAAATTAGAGATAAGAAAATAAATCT

GAAACATTAAATTTAAGGTAGTCGCTGGTCAGTATAGTCTGTTCTGGTTGGCGACTCCATTGTTAAAGAGTA

TAAAGACTTTAGATTTTATGAATATTAAAAATAGGAACAGTCAATTGAACTGCTCCTATTTTTCTGCTAAAT

ATATTGTAGTTTTCTTATATGTATAATGATAGATTAGCGGATTCTCATCTACGGTACTTACTTCAAATATGA

AGAAGTGATCGCGGTTATCTCTGGACTTTTCCTTATTGAGGACAAAGTAATTCTTACGTGAAGTCGCCATTG

TTTTTAGGATATCATCAGTTAGGAAGGTCAATGGAATATTCATGTTAGAGTAGCGGTAGAAGTCACGTTCAA

AATCTTGGTAGCTCTCGCTATAATAGTCCATTTGTAGGTGATTACGCTGAAACTCAAGCTGATTCATAGAGC

ACCTCCTCGACAAGTTCAATACTAATAATGTCTTTTAATTTCAAATTGATGTGACCTGTTGTAGTTTTTATC

AAAATGAAATCTTTGGTCAGACTTGGTATTGTTCCAGTGTAGGAAACACGCTTGTTTTTTTCAATCACTTGA

ATGCGTGTGCGTAGCTGCCCGGCGTATACTTGACTGAGGAGTAATAATTTCTTCTCTAGTGATAAGTCAGAC

ATGTACGTTACTTTGTTTGTATCATCAGAGAGTGCTGATGCATGTTCAGATAGGAAAAAGCCCATCCATTTT

TGCATCTTTGTATCCTGGTACTCTCTTGCTGATTGAAATGGTAAATATGAACGGTCAATCATATCAAATCCT

TTCTATGCAGAGGCAAGGGTATTTTTATCAAATTGAATCGTAAAACCTTGAATTCCCCCACCTGTGTAACAT

TCTTTAAAGCGATTGATTACCTCAGTATAGATTATCACAGATGAGCTTGTTGGCTTAATGCTAAATGTAAAT

TCCAATGGTAATCGGTTTTCAGATTTAGCATGTACTAGTCGTATCGATATTTCAGTTGTTTTGAGTTTTCTC

TGACGAAGTTTTGAAGTTGCTGTTTCAACGATTCCATGTAAAAATCTTTCAAGCATTTCAATATCATTACAT

CCTTTGCTACGGATTTCTGAAAATTGTACTGTATTTTTTTCTTGTTTCATTTTAATCCCTCCAATCCACCCG

CGGAATGACCACCGATAAGTTTACTGCGTTCAATATTTCTGGAACCTTCAGTTAGGACGGTTCCTTTTTGTA

TGGCTAAAAAACCAAACTGTTCTCTGACAACATCAATAGCTGTCTGAAGTCTATTATCTTTTTCAATTTGTT

CTACATCATCAAAGAGTGATAGTAGAGTATAGCTTTCATCTACGAAGCCACTATAAGATACACCAATTTGTC

TCACTGCACCAGAGGTGTATTTTTTCGGAATAATACAAGTACATGACTCACCATTGTTTTGGGGAGATTTG

CGGGTTCAATTTTATTCTGAGCATTTATAGATTTTTTCATCTCAGTCCTAGAATAGCCAATATGAATAGAAA

CGACAGTAGTCAATACTAGGGCTACTGTTCCGTTCCACAGTATCATTTAAAAATCATT-TTCACACCCTTTC

GTCTATTAGTATAGAAGAAAGCTCTCAGCACA

>GA19077/Mega-2.II

CATGTTGAGGCGGTAAGTTTGCTAGTCAAGGAGTAAAACGACGAAGATTAGCATTTACTTCCGCCCATGCGA

TAGCTGTCCGTGATTGACAAGTGCTAGCACGCAGACAGAACGGAGATAGCGAACCGCTGAGTGTGTCGCTCT

GCTCGTAAAAGCTTAGAAACCTTTGAACGAAAGGGATAATGAAAGCCTTGATTGCAAGGCTTTTTGCTTTAT

GGTGGGTAAGTATCAGAGTGAGAAAATTTTTGGAATGAGTAGAAGTGATAGCTAGAAATTATCAGTTTCTA

TTTCCATTTACCCTGTGGGTACGTGTTTGTTTCCATTGACAAGGAGTTTGTGGGAATAGAAATGT

ACCCACCTTGTTTGAATCAAGTGAAGTGTAGTTGAAGGAAATCTGTTGAAAGCAATACTTCATTTTACCG

AATAAGTAATAATTTAGGCAACTTCAAATCGATTAAAAAAAACTATTTTAAAGGTTAAGAGTAGACAAAA

ATTGTCCACTCTTTTTTGCAAACTCAATTTATCAATAAATGAAATGAGGGAATGTAAAATG

AAATATTTTGAGGTTGAGTTAGAAAATCCTGATGAATTTTTAAAACTACAAACAGAAGATTTTGTGAAAG

CTAATCGCTTGCTACTAAGGAAGATAATCCAGAGCGTTACAGTCTATGAAGAAAACTTCGTCATATCCTT

TAAATCTGGCATCGAATTGGAAGTATGAGTCTCATTCCATAACTTTTATATTGAACATATCATCTTGTTGTG

TTATACTATAAATTGATATAAACAAAGATGTAGGAGGAACCGAAACTATGACAGCCTCAATGCGTTTAAGAT

AAGCTGGCAATAAAAAAAGCAGAATCTATACCCGATGATAGGCTTTTTTGTTGTGCTTATTTATACGATATT

GAGCATTCATTAGTTACGGTGAGGATATTGGTTATTTAACTATACCTTTATTTAACTATGTCTTTAATATGA

ATGTTTCCAAATTGTATGTATGCAGACCAAAAGCCACATTGTGGGGTTTGGCCTGCATTTTTTTTGCCTA

GAATGCTATTCAAAATAGAAATTCAAGCAAAATAATATGCAGGAGATAATATAAATGGAAAAATACAACAAT

TGGAAACGAAAATTTTATGCAATATGGGCAGGGCAAGCAGTATCATTAATCACTAGTGCCATCCTGCAAATG

GCGATTATTTTTTACCTTACAGAAAAAACAGGATCTGCGATGGTCTTGTCTATGGCTTCATTAGTAGGTTTT

TTACCCTATGCGATTTTGGGACCTGCCATTGGTGTGCTAGTGGATCGTCATGATAGGAAGAAGATAATGATT

GGTGCCGATTTAATTATCGCAGCAGCTGGTGCAGTGCTTGCTATTGTTGCATTCTGTATGGAGCTACCTGTC

TGGATGATTATGATAGTATTGTTTATCCGTAGCATTGGAACAGCTTTTCATACCCCAGCACTCAATGCGGTT

ACACCACTTTTAGTACCAGAAGAACAGCTAACGAAATGCGCAGGCTATAGTCAGTCTTTGCAGTCTATAAGC

TATATTGTTAGTCCGGCAGTTGCAGCACTCTTATACTCCGTTTGGGATTTAAATGCTATTATTGCCATCGAC

GTATTGGGTGCTGTGATTGCATCTATTACGGTAGCAATTGTACGTATACCTAAGCTGGGTAATCAAGTGCAA

AGTTTAGAACCAAATTTCATAAGGGAGATGAAAGAAGGAGTTGTGGTTCTGAGACAAAACAAAGGATTGTTT

GCCTTATTACTCTTAGGAACACTATATACTTTTGTTTATATGCCAATCAATGCACTATTTCCTTTAATAAGC

ATGGAACACTTTAATGGAACGCCTGTGCATATTTCTATTACGGAAATTTCCTTTGCATTTGGGATGCTAGCA

GGAGGCTTATTATTAGGAAGATTAGGGGGCTTCGAAAAGCATGTATTACTAATAACAAGTTCATTTTTTATA

ATGGGGACCAGTTTAGCCGTTTCGGGAATACTTCCTCCAAATGGATTTGTAATATTCGTAGTTTGCTGTGCA

ATAATGGGGCTTTCGGTGCCATTTTATAGCGGTGTGCAAACAGCTCTTTTTCAGGAGAAAATTAAGCCTGAA

TATTTAGGACGTGTATTTTCTTTGATCGGAAGTATCATGTCACTTGCTATGCCAATTGGGTTAATTCTTTCT

GGATTCTTTGCTGATAAAATCGGTGTAAATCATTGGTTTTTACTATCAGGTATTTTAATTATTGGCATTGCT

ATAGTTTGCCAAATGATAACTGAGGTTAGAAAATTAGATTTAAAATAAACAATATTGGAGGAA

ATGTTATGGAATTAATATT

AAAAGCAAAAGACATTCGTGTGGAATTCAAAGGACGCGATGTTTTAGATATAAATGAATTAGAAGTATATGA

TTATGACCGTATTGGTTTAGTAGGAGCAAATGGTGCTGGAAAAAGCACTTTACTCAGGGTACTTTTAGGAGA

ATTAACTCCCCCAGGATGTAAAATGAATCGTCTGGGTGAACTTGCCTATATTCCCCAGTTGGACGAAGTAAC

TCTGCAGGAGGAAAAAGATTTTGCACTTGTAGGCAAGCTAGGTGTTGAGCAATTAAATATACAGACTATGAG

CGGTGGTGAAGAAACAAGGCTTAAAATAGCACAGGCCTTATCGGCACAGGTTCATGGTATTTTAGCGGATGA

ACCTACGAGCCATTTAGACCGTGAAGGAATTGATTTTCTAATAGGACAGCTAAAATATTTTACAGGTGCACT

GTTAGTTATTAGCCATGACCGCTATTTTCTTGATGAAATAGTAGATAAAATATGGGAACTGAAAGATGGCAA

AATCACTGAGTATTGGGGAAACTATTCTGATTATCTTCGTCAGAAAGAGGAAGAACGTAAGAGCCAAGCTGC

AGAATACGAACAATTTATTGCGGAACGTGCCCGATTGGAAAGGGCTGCGGAGGAAAAGCGAAAACAGGCTCG

TAAAATAGAACAGAAGGCAAAAGGTTCTTCAAAGAAAAAAAGTACTGAAGACGGAGGGCGTTTAGCTCATCA

AAAATCAATAGGAAGTAAGGAAAAAAAGATGTATAATGCTGCTAAAACCCTAGAGCACAGGATTGCGGCCTT

AGGAAAAGTAGAAGCTCCGGAAGGCATTCGCAGAATTCGTTTCAGGCAAAGTAAAGCATTGGAGCTCCATAA

TCCATACCCTATAGTCGGTGCAGAAATTAATAAAGTATTTGGGGATAAGGCTCTGTTTGAAAATGCATCTTT

TCAAATTCCGTTAGGAGCAAAAGTGGCGTTAACTGGTGGTAATGGAATCGGAAAAACAACTTTAATCCAAAT

GATCTTAAACCATGAAGAAGGAATTTCTATTTCGCCTAAGGCAAAAATAGGTTACTTTGCACAGAATGGTTA

CAAGTACAACAGTAATCAGAATGTTATGGAGTTTATGCAGAAGGATTGTGACTACAATATATCAGAAATTCG

TTCAGTGCTAGCATCTATGGGGTTCAAACAGAACGATATTGGAAAAAGTTTATCTGTTTTAAGCGGTGGAGA

AATTATAAAATTGTTGCTTGCTAAAATGCTCATGGGTAGATATAACATCCTAATAATGGATGAACCCAGTAA

CTTCCTTGACATACCAAGTTTAGAGGCTTTGGAAATACTAATGAAGGAGTACACCGGAACTATCGTGTTTAT

CACCCACGATAAACGATTACTCGAAAATGTAGCAGATGTAGTTTATGAAATTAGAGATAAGAAAATAAATCT

GAAACATTAAATTTAAGGTAGTCGCTGGTCAGTATAGTCTGTTCTGGTTGGCGACTCCATTGTTAAAGAGTA

TAAAGACTTTAGATTTTATGAATATTAAAAATAGGAACAGTCAATTGAACTGCTCCTATTTTTCTGCTAAAT

ATATTGTAGTTTTCTTATATGTATAATGATAGATTAGCGGATTCTCATCTACGGTACTTACTTCAAATATGA

AGAAGTGATCGCGGTTATCTCTGGACTTTTCCTTATTGAGGACAAAGTAATTCTTACGTGAAGTCGCCATTG

TTTTTAGGATATCATCAGTTAGGAAGGTCAATGGAATATTCATGTTAGAGTAGCGGTAGAAGTCACGTTCAA

AATCTTGGTAGCTCTCGCTATAATAGTCCATTTGTAGGTGATTACGCTGAAACTCAAGCTGATTCATAGAGC

ACCTCCTCGACAAGTTCAATACTAATAATGTCTTTTAATTTCAAATTGATGTGACCTGTTGTAGTTTTTATC

AAAATGAAATCTTTGGTCAGACTTGGTATTGTTCCAGTGTAGGAAACACGCTTGTTTTTTTCAATCACTTGA

ATGCGTGTGCGTAGCTGCCCGGCGTATACTTGACTGAGGAGTAATAATTTCTTCTCTAGTGATAAGTCAGAC

ATGTACGTTACTTTGTTTGTATCATCAGAGAGTGCTGATGCATGTTCAGATAGGAAAAAGCCCATCCATTTT

TGCATCTTTGTATCCTGGTACTCTCTTGCTGATTGAAATGGTAAATATGAACGGTCAATCATATCAAATCCT

TTCTATGCAGAGGCAAGGGTATTTTTATCAAATTGAATCGTAAAACCTTGAATTCCCCCACCTGTGTAACAT

TCTTTAAAGCGATTGATTACCTCAGTATAGATTATCACAGATGAGCTTGTTGGCTTAATGCTAAATGTAAAT

TCCAATGGTAATCGGTTTTCAGATTTAGCATGTACTAGTCGTATCGATATTTCAGTTGTTTTGAGTTTTCTC

TGACGAAGTTTTGAAGTTGCTGTTTCAACGATTCCATGTAAAAATCTTTCAAGCATTTCAATATCATTACAT

CCTTTGCTACGGATTTCTGAAAATTGTACTGTATTTTTTTCTTGTTTCATTTTAATCCCTCCAATCCACCCG

CGGAATGACCACCGATAAGTTTACTGCGTTCAATATTTCTGGAACCTTCAGTTAGGACGGTTCCTTTTTGTA

TGGCTAAAAAACCAAACTGTTCTCTGACAACATCAATAGCTGTCTGAAGTCTATTATCTTTTTCAATTTGTT

CTACATCATCAAAGAGTGATAGTAGAGTATAGCTTTCATCTACGAAGCCACTATAAGATACACCAATTTGTC

TCACTGCACCAGAGGTGTATTTTTTCGGAATAATACAAGTACATGACTCACCATTGTTTTGGGGAGATTTG

CGGGTTCAATTTTATTCTGAGCATTTATAGATTTTTTCATCTCAGTCCTAGAATAGCCAATATGAATAGAAA

CGACAGTAGTCAATACTAGGGCTACTGTTCCGTTCCACAGTATCATTTAAAAATCATT-TTCACACCCTTTC

GTCTATTAGTATAGAAGAAAGCTCTCAGCACA

>GA44511/Mega-2.II

CATGTTGAGGCGGTAAGTTTGCTAGTCAAGGAGTAAAACGACGAAGATTAGCATTTACTTCCGCCCATGCGA

TAGCTGTCCGTGATTGACAAGTGCTAGCACGCAGACAGAACGGAGATAGCGAACCGCTGAGTGTGTCGCTCT

GCTCGTAAAAGCTTAGAAACCTTTGAACGAAAGGGATAATGAAAGCCTTGATTGCAAGGCTTTTTGCTTTAT

GGTGGGTAAGTATCAGAGTGAGAAAATTTTTGGAATGAGTAGAAGTGATAGCTAGAAATTATCAGTTTCTA

TTTCCATTTACCCTGTGGGTACGTGTTTGTTTCCATTGACAAGGAGTTTGTGGGAATAGAAATGT

ACCCACCTTGTTTGAATCAAGTGAAGTGTAGTTGAAGGAAATCTGTTGAAAGCAATACTTCATTTTACCG

AATAAGTAATAATTTAGGCAACTTCAAATCGATTAAAAAAAACTATTTTAAAGGTTAAGAGTAGACAAAA

ATTGTCCACTCTTTTTTGCAAACTCAATTTATCAATAAATGAAATGAGGGAATGTAAAATG

AAATATTTTGAGGTTGAGTTAGAAAATCCTGATGAATTTTTAAAACTACAAACAGAAGATTTTGTGAAAG

CTAATCGCTTGCTACTAAGGAAGATAATCCAGAGCGTTACAGTCTATGAAGAAAACTTCGTCATATCCTT

TAAATCTGGCATCGAATTGGAAGTATGAGTCTCATTCCATAACTTTTATATTGAACATATCATCTTGTTGTG

TTATACTATAAATTGATATAAACAAAGATGTAGGAGGAACCGAAACTATGACAGCCTCAATGCGTTTAAGAT

AAGCTGGCAATAAAAAAAGCAGAATCTATACCCGATGATAGGCTTTTTTGTTGTGCTTATTTATACGATATT

GAGCATTCATTAGTTACGGTGAGGATATTGGTTATTTAACTATACCTTTATTTAACTATGTCTTTAATATGA

ATGTTTCCAAATTGTATGTATGCAGACCAAAAGCCACATTGTGGGGTTTGGCCTGCATTTTTTTTGCCTA

GAATGCTATTCAAAATAGAAATTCAAGCAAAATAATATGCAGGAGATAATATAAATGGAAAAATACAACAAT

TGGAAACGAAAATTTTATGCAATATGGGCAGGGCAAGCAGTATCATTAATCACTAGTGCCATCCTGCAAATG

GCGATTATTTTTTACCTTACAGAAAAAACAGGATCTGCGATGGTCTTGTCTATGGCTTCATTAGTAGGTTTT

TTACCCTATGCGATTTTGGGACCTGCCATTGGTGTGCTAGTGGATCGTCATGATAGGAAGAAGATAATGATT

GGTGCCGATTTAATTATCGCAGCAGCTGGTGCAGTGCTTGCTATTGTTGCATTCTGTATGGAGCTACCTGTC

TGGATGATTATGATAGTATTGTTTATCCGTAGCATTGGAACAGCTTTTCATACCCCAGCACTCAATGCGGTT

ACACCACTTTTAGTACCAGAAGAACAGCTAACGAAATGCGCAGGCTATAGTCAGTCTTTGCAGTCTATAAGC

TATATTGTTAGTCCGGCAGTTGCAGCACTCTTATACTCCGTTTGGGATTTAAATGCTATTATTGCCATCGAC

GTATTGGGTGCTGTGATTGCATCTATTACGGTAGCAATTGTACGTATACCTAAGCTGGGTAATCAAGTGCAA

AGTTTAGAACCAAATTTCATAAGGGAGATGAAAGAAGGAGTTGTGGTTCTGAGACAAAACAAAGGATTGTTT

GCCTTATTACTCTTAGGAACACTATATACTTTTGTTTATATGCCAATCAATGCACTATTTCCTTTAATAAGC

ATGGAACACTTTAATGGAACGCCTGTGCATATTTCTATTACGGAAATTTCCTTTGCATTTGGGATGCTAGCA

GGAGGCTTATTATTAGGAAGATTAGGGGGCTTCGAAAAGCATGTATTACTAATAACAAGTTCATTTTTTATA

ATGGGGACCAGTTTAGCCGTTTCGGGAATACTTCCTCCAAATGGATTTGTAATATTCGTAGTTTGCTGTGCA

ATAATGGGGCTTTCGGTGCCATTTTATAGCGGTGTGCAAACAGCTCTTTTTCAGGAGAAAATTAAGCCTGAA

TATTTAGGACGTGTATTTTCTTTGATCGGAAGTATCATGTCACTTGCTATGCCAATTGGGTTAATTCTTTCT

GGATTCTTTGCTGATAAAATCGGTGTAAATCATTGGTTTTTACTATCAGGTATTTTAATTATTGGCATTGCT

ATAGTTTGCCAAATGATAACTGAGGTTAGAAAATTAGATTTAAAATAAACAATATTGGAGGAA

ATGTTATGGAATTAATATT

AAAAGCAAAAGACATTCGTGTGGAATTCAAAGGACGCGATGTTTTAGATATAAATGAATTAGAAGTATATGA

TTATGACCGTATTGGTTTAGTAGGAGCAAATGGTGCTGGAAAAAGCACTTTACTCAGGGTACTTTTAGGAGA

ATTAACTCCCCCAGGATGTAAAATGAATCGTCTGGGTGAACTTGCCTATATTCCCCAGTTGGACGAAGTAAC

TCTGCAGGAGGAAAAAGATTTTGCACTTGTAGGCAAGCTAGGTGTTGAGCAATTAAATATACAGACTATGAG

CGGTGGTGAAGAAACAAGGCTTAAAATAGCACAGGCCTTATCGGCACAGGTTCATGGTATTTTAGCGGATGA

ACCTACGAGCCATTTAGACCGTGAAGGAATTGATTTTCTAATAGGACAGCTAAAATATTTTACAGGTGCACT

GTTAGTTATTAGCCATGACCGCTATTTTCTTGATGAAATAGTAGATAAAATATGGGAACTGAAAGATGGCAA

AATCACTGAGTATTGGGGAAACTATTCTGATTATCTTCGTCAGAAAGAGGAAGAACGTAAGAGCCAAGCTGC

AGAATACGAACAATTTATTGCGGAACGTGCCCGATTGGAAAGGGCTGCGGAGGAAAAGCGAAAACAGGCTCG

TAAAATAGAACAGAAGGCAAAAGGTTCTTCAAAGAAAAAAAGTACTGAAGACGGAGGGCGTTTAGCTCATCA

AAAATCAATAGGAAGTAAGGAAAAAAAGATGTATAATGCTGCTAAAACCCTAGAGCACAGGATTGCGGCCTT

AGGAAAAGTAGAAGCTCCGGAAGGCATTCGCAGAATTCGTTTCAGGCAAAGTAAAGCATTGGAGCTCCATAA

TCCATACCCTATAGTCGGTGCAGAAATTAATAAAGTATTTGGGGATAAGGCTCTGTTTGAAAATGCATCTTT

TCAAATTCCGTTAGGAGCAAAAGTGGCGTTAACTGGTGGTAATGGAATCGGAAAAACAACTTTAATCCAAAT

GATCTTAAACCATGAAGAAGGAATTTCTATTTCGCCTAAGGCAAAAATAGGTTACTTTGCACAGAATGGTTA

CAAGTACAACAGTAATCAGAATGTTATGGAGTTTATGCAGAAGGATTGTGACTACAATATATCAGAAATTCG

TTCAGTGCTAGCATCTATGGGGTTCAAACAGAACGATATTGGAAAAAGTTTATCTGTTTTAAGCGGTGGAGA

AATTATAAAATTGTTGCTTGCTAAAATGCTCATGGGTAGATATAACATCCTAATAATGGATGAACCCAGTAA

CTTCCTTGACATACCAAGTTTAGAGGCTTTGGAAATACTAATGAAGGAGTACACCGGAACTATCGTGTTTAT

CACCCACGATAAACGATTACTCGAAAATGTAGCAGATGTAGTTTATGAAATTAGAGATAAGAAAATAAATCT

GAAACATTAAATTTAAGGTAGTCGCTGGTCAGTATAGTCTGTTCTGGTTGGCGACTCCATTGTTAAAGAGTA

TAAAGACTTTAGATTTTATGAATATTAAAAATAGGAACAGTCAATTGAACTGCTCCTATTTTTCTGCTAAAT

ATATTGTAGTTTTCTTATATGTATAATGATAGATTAGCGGATTCTCATCTACGGTACTTACTTCAAATATGA

AGAAGTGATCGCGGTTATCTCTGGACTTTTCCTTATTGAGGACAAAGTAATTCTTACGTGAAGTCGCCATTG

TTTTTAGGATATCATCAGTTAGGAAGGTCAATGGAATATTCATGTTAGAGTAGCGGTAGAAGTCACGTTCAA

AATCTTGGTAGCTCTCGCTATAATAGTCCATTTGTAGGTGATTACGCTGAAACTCAAGCTGATTCATAGAGC

ACCTCCTCGACAAGTTCAATACTAATAATGTCTTTTAATTTCAAATTGATGTGACCTGTTGTAGTTTTTATC

AAAATGAAATCTTTGGTCAGACTTGGTATTGTTCCAGTGTAGGAAACACGCTTGTTTTTTTCAATCACTTGA

ATGCGTGTGCGTAGCTGCCCGGCGTATACTTGACTGAGGAGTAATAATTTCTTCTCTAGTGATAAGTCAGAC

ATGTACGTTACTTTGTTTGTATCATCAGAGAGTGCTGATGCATGTTCAGATAGGAAAAAGCCCATCCATTTT

TGCATCTTTGTATCCTGGTACTCTCTTGCTGATTGAAATGGTAAATATGAACGGTCAATCATATCAAATCCT

TTCTATGCAGAGGCAAGGGTATTTTTATCAAATTGAATCGTAAAACCTTGAATTCCCCCACCTGTGTAACAT

TCTTTAAAGCGATTGATTACCTCAGTATAGATTATCACAGATGAGCTTGTTGGCTTAATGCTAAATGTAAAT

TCCAATGGTAATCGGTTTTCAGATTTAGCATGTACTAGTCGTATCGATATTTCAGTTGTTTTGAGTTTTCTC

TGACGAAGTTTTGAAGTTGCTGTTTCAACGATTCCATGTAAAAATCTTTCAAGCATTTCAATATCATTACAT

CCTTTGCTACGGATTTCTGAAAATTGTACTGTATTTTTTTCTTGTTTCATTTTAATCCCTCCAATCCACCCG

CGGAATGACCACCGATAAGTTTACTGCGTTCAATATTTCTGGAACCTTCAGTTAGGACGGTTCCTTTTTGTA

TGGCTAAAAAACCAAACTGTTCTCTGACAACATCAATAGCTGTCTGAAGTCTATTATCTTTTTCAATTTGTT

CTACATCATCAAAGAGTGATAGTAGAGTATAGCTTTCATCTACGAAGCCACTATAAGATACACCAATTTGTC

TCACTGCACCAGAGGTGTATTTTTTCGGAATAATACAAGTACATGACTCACCATTGTTTTGGGGAGATTTG

CGGGTTCAATTTTATTCTGAGCATTTATAGATTTTTTCATCTCAGTCCTAGAATAGCCAATATGAATAGAAA

CGACAGTAGTCAATACTAGGGCTACTGTTCCGTTCCACAGTATCATTTAAAAATCATT-TTCACACCCTTTC

GTCTATTAGTATAGAAGAAAGCTCTCAGCACA

>GA47751/Mega-2.II

CATGTTGAGGCGGTAAGTTTGCTAGTCAAGGAGTAAAACGACGAAGATTAGCATTTACTTCCGCCCATGCGA

TAGCTGTCCGTGATTGACAAGTGCTAGCACGCAGACAGAACGGAGATAGCGAACCGCTGAGTGTGTCGCTCT

GCTCGTAAAAGCTTAGAAACCTTTGAACGAAAGGGATAATGAAAGCCTTGATTGCAAGGCTTTTTGCTTTAT

GGTGGGTAAGTATCAGAGTGAGAAAATTTTTGGAATGAGTAGAAGTGATAGCTAGAAATTATCAGTTTCTA

TTTCCATTTACCCTGTGGGTACGTGTTTGTTTCCATTGACAAGGAGTTTGTGGGAATAGAAATGT

ACCCACCTTGTTTGAATCAAGTGAAGTGTAGTTGAAGGAAATCTGTTGAAAGCAATACTTCATTTTACCG

AATAAGTAATAATTTAGGCAACTTCAAATCGATTAAAAAAAACTATTTTAAAGGTTAAGAGTAGACAAAA

ATTATCCACTCTTTTTTGCAAACTCAATTTATCAATAAATGAAATGAGGGAATGTAAAATG

AAATATTTTGAGGTTGAGTTAGAAAATCCTGATGAATTTTTAAAACTACAAACAGAAGATTTTGTGAAAG

CTAATCGCTTGCTACTAAGGAAGATAATCCAGAGCGTTACAGTCTATGAAGAAAACTTCGTCATATCCTT

TAAATCTGGCATCGAATTGGAAGTATGAGTCTCATTCCATAACTTTTATATTGAACATATCATCTTGTTGTG

TTATACTATAAATTGATATAAACAAAGATGTAGGAGGAACCGAAACTATGACAGCCTCAATGCGTTTAAGAT

AAGCTGGCAATAAAAAAAGCAGAATCTATACCCGATGATAGGCTTTTTTGTTGTGCTTATTTATACGATATT

GAGCATTCATTAGTTACGGTGAGGATATTGGTTATTTAACTATACCTTTATTTAACTATGTCTTTAATATGA

ATGTTTCCAAATTGTATGTATGCAGACCAAAAGCCACATTGTGGGGTTTGGCCTGCATTTTTTTTGCCTA

GAATGCTATTCAAAATAGAAATTCAAGCAAAATAATATGCAGGAGATAATATAAATGGAAAAATACAACAAT

TGGAAACGAAAATTTTATGCAATATGGGCAGGGCAAGCAGTATCATTAATCACTAGTGCCATCCTGCAAATG

GCGATTATTTTTTACCTTACAGAAAAAACAGGATCTGCGATGGTCTTGTCTATGGCTTCATTAGTAGGTTTT

TTACCCTATGCGATTTTGGGACCTGCCATTGGTGTGCTAGTGGATCGTCATGATAGGAAGAAGATAATGATT

GGTGCCGATTTAATTATCGCAGCAGCTGGTGCAGTGCTTGCTATTGTTGCATTCTGTATGGAGCTACCTGTC

TGGATGATTATGATAGTATTGTTTATCCGTAGCATTGGAACAGCTTTTCATACCCCAGCACTCAATGCGGTT

ACACCACTTTTAGTACCAGAAGAACAGCTAACGAAATGCGCAGGCTATAGTCAGTCTTTGCAGTCTATAAGC

TATATTGTTAGTCCGGCAGTTGCAGCACTCTTATACTCCGTTTGGGATTTAAATGCTATTATTGCCATCGAC

GTATTGGGTGCTGTGATTGCATCTATTACGGTAGCAATTGTACGTATACCTAAGCTGGGTAATCAAGTGCAA

AGTTTAGAACCAAATTTCATAAGGGAGATGAAAGAAGGAGTTGTGGTTCTGAGACAAAACAAAGGATTGTTT

GCCTTATTACTCTTAGGAACACTATATACTTTTGTTTATATGCCAATCAATGCACTATTTCCTTTAATAAGC

ATGGAACACTTTAATGGAACGCCTGTGCATATTTCTATTACGGAAATTTCCTTTGCATTTGGGATGCTAGCA

GGAGGCTTATTATTAGGAAGATTAGGGGGCTTCGAAAAGCATGTATTACTAATAACAAGTTCATTTTTTATA

ATGGGGACCAGTTTAGCCGTTTCGGGAATACTTCCTCCAAATGGATTTGTAATATTCGTAGTTTGCTGTGCA

ATAATGGGGCTTTCGGTGCCATTTTATAGCGGTGTGCAAACAGCTCTTTTTCAGGAGAAAATTAAGCCTGAA

TATTTAGGACGTGTATTTTCTTTGATCGGAAGTATCATGTCACTTGCTATGCCAATTGGGTTAATTCTTTCT

GGATTCTTTGCTGATAAAATCGGTGTAAATCATTGGTTTTTACTATCAGGTATTTTAATTATTGGCATTGCT

ATAGTTTGCCAAATGATAACTGAGGTTAGAAAATTAGATTTAAAATAAACAATATTGGAGGAA

ATGTTATGGAATTAATATT

AAAAGCAAAAGACATTCGTGTGGAATTCAAAGGACGCGATGTTTTAGATATAAATGAATTAGAAGTATATGA

TTATGACCGTATTGGTTTAGTAGGAGCAAATGGTGCTGGAAAAAGCACTTTACTCAGGGTACTTTTAGGAGA

ATTAACTCCCCCAGGATGTAAAATGAATCGTCTGGGTGAACTTGCCTATATTCCCCAGTTGGACGAAGTAAC

TCTGCAGGAGGAAAAAGATTTTGCACTTGTAGGCAAGCTAGGTGTTGAGCAATTAAATATACAGACTATGAG

CGGTGGTGAAGAAACAAGGCTTAAAATAGCACAGGCCTTATCGGCACAGGTTCATGGTATTTTAGCGGATGA

ACCTACGAGCCATTTAGACCGTGAAGGAATTGATTTTCTAATAGGACAGCTAAAATATTTTACAGGTGCACT

GTTAGTTATTAGCCATGACCGCTATTTTCTTGATGAAATAGTAGATAAAATATGGGAACTGAAAGATGGCAA

AATCACTGAGTATTGGGGAAACTATTCTGATTATCTTCGTCAGAAAGAGGAAGAACGTAAGAGCCAAGCTGC

AGAATACGAACAATTTATTGCGGAACGTGCCCGATTGGAAAGGGCTGCGGAGGAAAAGCGAAAACAGGCTCG

TAAAATAGAACAGAAGGCAAAAGGTTCTTCAAAGAAAAAAAGTACTGAAGACGGAGGGCGTTTAGCTCATCA

AAAATCAATAGGAAGTAAGGAAAAAAAGATGTATAATGCTGCTAAAACCCTAGAGCACAGGATTGCGGCCTT

AGGAAAAGTAGAAGCTCCGGAAGGCATTCGCAGAATTCGTTTCAGGCAAAGTAAAGCATTGGAGCTCCATAA

TCCATACCCTATAGTCGGTGCAGAAATTAATAAAGTATTTGGGGATAAGGCTCTGTTTGAAAATGCATCTTT

TCAAATTCCGTTAGGAGCAAAAGTGGCGTTAACTGGTGGTAATGGAATCGGAAAAACAACTTTAATCCAAAT

GATCTTAAACCATGAAGAAGGAATTTCTATTTCGCCTAAGGCAAAAATAGGTTACTTTGCACAGAATGGTTA

CAAGTACAACAGTAATCAGAATGTTATGGAGTTTATGCAGAAGGATTGTGACTACAATATATCAGAAATTCG

TTCAGTGCTAGCATCTATGGGGTTCAAACAGAACGATATTGGAAAAAGTTTATCTGTTTTAAGCGGTGGAGA

AATTATAAAATTGTTGCTTGCTAAAATGCTCATGGGTAGATATAACATCCTAATAATGGATGAACCCAGTAA

CTTCCTTGACATACCAAGTTTAGAGGCTTTGGAAATACTAATGAAGGAGTACACCGGAACTATCGTGTTTAT

CACCCACGATAAACGATTACTCGAAAATGTAGCAGATGTAGTTTATGAAATTAGAGATAAGAAAATAAATCT

GAAACATTAAATTTAAGGTAGTCGCTGGTCAGTATAGTCTGTTCTGGTTGGCGACTCCATTGTTAAAGAGTA

TAAAGACTTTAGATTTTATGAATATTAAAAATAGGAACAGTCAATTGAACTGCTCCTATTTTTCTGCTAAAT

ATATTGTAGTTTTCTTATATGTATAATGATAGATTAGCGGATTCTCATCTACGGTACTTACTTCAAATATGA

AGAAGTGATCGCGGTTATCTCTGGACTTTTCCTTATTGAGGACAAAGTAATTCTTACGTGAAGTCGCCATTG

TTTTTAGGATATCATCAGTTAGGAAGGTCAATGGAATATTCATGTTAGAGTAGCGGTAGAAGTCACGTTCAA

AATCTTGGTAGCTCTCGCTATAATAGTCCATTTGTAGGTGATTACGCTGAAACTCAAGCTGATTCATAGAGC

ACCTCCTCGACAAGTTCAATACTAATAATGTCTTTTAATTTCAAATTGATGTGACCTGTTGTAGTTTTTATC

AAAATGAAATCTTTGGTCAGACTTGGTATTGTTCCAGTGTAGGAAACACGCTTGTTTTTTTCAATCACTTGA

ATGCGTGTGCGTAGCTGCCCGGCGTATACTTGACTGAGGAGTAATAATTTCTTCTCTAGTGATAAGTCAGAC

ATGTACGTTACTTTGTTTGTATCATCAGAGAGTGCTGATGCATGTTCAGATAGGAAAAAGCCCATCCATTTT

TGCATCTTTGTATCCTGGTACTCTCTTGCTGATTGAAATGGTAAATATGAACGGTCAATCATATCAAATCCT

TTCTATGCAGAGGCAAGGGTATTTTTATCAAATTGAATCGTAAAACCTTGAATTCCCCCACCTGTGTAACAT

TCTTTAAAGCGATTGATTACCTCAGTATAGATTATCACAGATGAGCTTGTTGGCTTAATGCTAAATGTAAAT

TCCAATGGTAATCGGTTTTCAGATTTAGCATGTACTAGTCGTATCGATATTTCAGTTGTTTTGAGTTTTCTC

TGACGAAGTTTTGAAGTTGCTGTTTCAACGATTCCATGTAAAAATCTTTCAAGCATTTCAATATCATTACAT

CCTTTGCTACGGATTTCTGAAAATTGTACTGTATTTTTTTCTTGTTTCATTTTAATCCCTCCAATCCACCCG

CGGAATGACCACCGATAAGTTTACTGCGTTCAATATTTCTGGAACCTTCAGTTAGGACGGTTCCTTTTTGTA

TGGCTAAAAAACCAAACTGTTCTCTGACAACATCAATAGCTGTCTGAAGTCTATTATCTTTTTCAATTTGTT

CTACATCATCAAAGAGTGATAGTAGAGTATAGCTTTCATCTACGAAGCCACTATAAGATACACCAATTTGTC

TCACTGCACCAGAGGTGTATTTTTTCGGAATAATACAAGTACATGACTCACCATTGTTTTGGGGAGATTTG

CGGGTTCAATTTTATTCTGAGCATTTATAGATTTTTTCATCTCAGTCCTAGAATAGCCAATATGAATAGAAA

CGACAGTAGTCAATACTAGGGCTACTGTTCCGTTCCACAGTATCATTTAAAAATCATT-TTCACACCCTTTC

GTCTATTAGTATAGAAGAAAGCTCTCAGCACA

>GA44128/Mega-2.II

CATGTTGAGGCGGTAAGTTTGCTAGTCAAGGAGTAAAACGACGAAGATTAGCATTTACTTCCGCCCATGCGA

TAGCTGTCCGTGATTGACAAGTGCTAGCACGCAGACAGAACGGAGATAGCGAACCGCTGAGTGTGTCGCTCT

GCTCGTAAAAGCTTAGAAACCTTTGAACGAAAGGGATAATGAAAGCCTTGATTGCAAGGCTTTTTGCTTTAT

GGTGGGTAAGTATCAGAGTGAGAAAATTTTTGGAATGAGTAGAAGTGATAGCTAGAAATTATCAGTTTCTA

TTTCCATTTACCCTGTGGGTACGTGTTTGTTTCCATTGACAAGGAGTTTGTGGGAATAGAAATGT

ACCCACCTTGTTTGAATCAAGTGAAGTGTAGTTGAAGGAAATCTGTTGAAAGCAATACTTCATTTTACCG

AATAAGTAATAATTTAGGCAACTTCAAATCGATTAAAAAAAACTATTTTAAAGGTTAAGAGTAGACAAAA

ATTGTCCACTCTTTTTTGCAAACTCAATTTATCAATAAATGAAATGAGGGAATGTAAAATG

AAATATTTTGAGGTTGAGTTAGAAAATCCTGATGAATTTTTAAAACTACAAACAGAAGATTTTGTGAAAG

CTAATCGCTTGCTACTAAGGAAGATAATCCAGAGCGTTACAGTCTATGAAGAAAACTTCGTCATATCCTT

TAAATCTGGCATCGAATTGGAAGTATGAGTCTCATTCCATAACTTTTATATTGAACATATCATCTTGTTGTG

TTATACTATAAATTGATATAAACAAAGATGTAGGAGGAACCGAAACTATGACAGCCTCAATGCGTTTAAGAT

AAGCTGGCAATAAAAAAAGCAGAATCTATACCCGATGATAGGCTTTTTTGTTGTGCTTATTTATACGATATT

GAGCATTCATTAGTTACGGTGAGGATATTGGTTATTTAACTATACCTTTATTTAACTATGTCTTTAATATGA

ATGTTTCCAAATTGTATGTATGCAGACCAAAAGCCACATTGTGGGGTTTGGCCTGCATTTTTTTTGCCTA

GAATGCTATTCAAAATAGAAATTCAAGCAAAATAATATGCAGGAGATAATATAAATGGAAAAATACAACAAT

TGGAAACGAAAATTTTATGCAATATGGGCAGGGCAAGCAGTATCATTAATCACTAGTGCCATCCTGCAAATG

GCGATTATTTTTTACCTTACAGAAAAAACAGGATCTGCGATGGTCTTGTCTATGGCTTCATTAGTAGGTTTT

TTACCCTATGCGATTTTGGGACCTGCCATTGGTGTGCTAGTGGATCGTCATGATAGGAAGAAGATAATGATT

GGTGCCGATTTAATTATCGCAGCAGCTGGTGCAGTGCTTGCTATTGTTGCATTCTGTATGGAGCTACCTGTC

TGGATGATTATGATAGTATTGTTTATCCGTAGCATTGGAACAGCTTTTCATACCCCAGCACTCAATGCGGTT

ACACCACTTTTAGTACCAGAAGAACAGCTAACGAAATGCGCAGGCTATAGTCAGTCTTTGCAGTCTATAAGC

TATATTGTTAGTCCGGCAGTTGCAGCACTCTTATACTCCGTTTGGGATTTAAATGCTATTATTGCCATCGAC

GTATTGGGTGCTGTGATTGCATCTATTACGGTAGCAATTGTACGTATACCTAAGCTGGGTAATCAAGTGCAA

AGTTTAGAACCAAATTTCATAAGGGAGATGAAAGAAGGAGTTGTGGTTCTGAGACAAAACAAAGGATTGTTT

GCCTTATTACTCTTAGGAACACTATATACTTTTGTTTATATGCCAATCAATGCACTATTTCCTTTAATAAGC

ATGGAATACTTTAATGGAACGCCTGTGCATATTTCTATTACGGAAATTTCCTTTGCATTTGGGATGCTAGCA

GGAGGCTTATTATTAGGAAGATTAGGGGGCTTCGAAAAGCATGTATTACTAATAACAAGTTCATTTTTTATA

ATGGGGACCAGTTTAGCCGTTTCGGGAATACTTCCTCCAAATGGATTTGTAATATTCGTAGTTTGCTGTGCA

ATAATGGGGCTTTCGGTGCCATTTTATAGCGGTGTGCAAACAGCTCTTTTTCAGGAGAAAATTAAGCCTGAA

TATTTAGGACGTGTATTTTCTTTGATCGGAAGTATCATGTCACTTGCTATGCCAATTGGGTTAATTCTTTCT

GGATTCTTTGCTGATAAAATCGGTGTAAATCATTGGTTTTTACTATCAGGTATTTTAATTATTGGCATTGCT

ATAGTTTGCCAAATGATAACTGAGGTTAGAAAATTAGATTTAAAATAAACAATATTGGAGGAA

ATGTTATGGAATTAATATT

AAAAGCAAAAGACATTCGTGTGGAATTCAAAGGACGCGATGTTTTAGATATAAATGAATTAGAAGTATATGA

TTATGACCGTATTGGTTTAGTAGGAGCAAATGGTGCTGGAAAAAGCACTTTACTCAGGGTACTTTTAGGAGA

ATTAACTCCCCCAGGATGTAAAATGAATCGTCTGGGTGAACTTGCCTATATTCCCCAGTTGGACGAAGTAAC

TCTGCAGGAGGAAAAAGATTTTGCACTTGTAGGCAAGCTAGGTGTTGAGCAATTAAATATACAGACTATGAG

CGGTGGTGAAGAAACAAGGCTTAAAATAGCACAGGCCTTATCGGCACAGGTTCATGGTATTTTAGCGGATGA

ACCTACGAGCCATTTAGACCGTGAAGGAATTGATTTTCTAATAGGACAGCTAAAATATTTTACAGGTGCACT

GTTAGTTATTAGCCATGACCGCTATTTTCTTGATGAAATAGTAGATAAAATATGGGAACTGAAAGATGGCAA

AATCACTGAGTATTGGGGAAACTATTCTGATTATCTTCGTCAGAAAGAGGAAGAACGTAAGAGCCAAGCTGC

AGAATACGAACAATTTATTGCGGAACGTGCCCGATTGGAAAGGGCTGCGGAGGAAAAGCGAAAACAGGCTCG

TAAAATAGAACAGAAGGCAAAAGGTTCTTCAAAGAAAAAAAGTACTGAAGACGGAGGGCGTTTAGCTCATCA

AAAATCAATAGGAAGTAAGGAAAAAAAGATGTATAATGCTGCTAAAACCCTAGAGCACAGGATTGCGGCCTT

AGGAAAAGTAGAAGCTCCGGAAGGCATTCGCAGAATTCGTTTCAGGCAAAGTAAAGCATTGGAGCTCCATAA

TCCATACCCTATAGTCGGTGCAGAAATTAATAAAGTATTTGGGGATAAGGCTCTGTTTGAAAATGCATCTTT

TCAAATTCCGTTAGGAGCAAAAGTGGCGTTAACTGGTGGTAATGGAATCGGAAAAACAACTTTAATCCAAAT

GATCTTAAACCATGAAGAAGGAATTTCTATTTCGCCTAAGGCAAAAATAGGTTACTTTGCACAGAATGGTTA

CAAGTACAACAGTAATCAGAATGTTATGGAGTTTATGCAGAAGGATTGTGACTACAATATATCAGAAATTCG

TTCAGTGCTAGCATCTATGGGGTTCAAACAGAACGATATTGGAAAAAGTTTATCTGTTTTAAGCGGTGGAGA

AATTATAAAATTGTTGCTTGCTAAAATGCTCATGGGTAGATATAACATCCTAATAATGGATGAACCCAGTAA

CTTCCTTGACATACCAAGTTTAGAGGCTTTGGAAATACTAATGAAGGAGTACACCGGAACTATCGTGTTTAT

CACCCACGATAAACGATTACTCGAAAATGTAGCAGATGTAGTTTATGAAATTAGAGATAAGAAAATAAATCT

GAAACATTAAATTTAAGGTAGTCGCTGGTCAGTATAGTCTGTTCTGGTTGGCGACTCCATTGTTAAAGAGTA

TAAAGACTTTAGATTTTATGAATATTAAAAATAGGAACAGTCAATTGAACTGCTCCTATTTTTCTGCTAAAT

ATATTGTAGTTTTCTTATATGTATAATGATAGATTAGCGGATTCTCATCTACGGTACTTACTTCAAATATGA

AGAAGTGATCGCGGTTATCTCTGGACTTTTCCTTATTGAGGACAAAGTAATTCTTACGTGAAGTCGCCATTG

TTTTTAGGATATCATCAGTTAGGAAGGTCAATGGAATATTCATGTTAGAGTAGCGGTAGAAGTCACGTTCAA

AATCTTGGTAGCTCTCGCTATAATAGTCCATTTGTAGGTGATTACGCTGAAACTCAAGCTGATTCATAGAGC

ACCTCCTCGACAAGTTCAATACTAATAATGTCTTTTAATTTCAAATTGATGTGACCTGTTGTAGTTTTTATC

AAAATGAAATCTTTGGTCAGACTTGGTATTGTTCCAGTGTAGGAAACACGCTTGTTTTTTTCAATCACTTGA

ATGCGTGTGCGTAGCTGCCCGGCGTATACTTGACTGAGGAGTAATAATTTCTTCTCTAGTGATAAGTCAGAC

ATGTACGTTACTTTGTTTGTATCATCAGAGAGTGCTGATGCATGTTCAGATAGGAAAAAGCCCATCCATTTT

TGCATCTTTGTATCCTGGTACTCTCTTGCTGATTGAAATGGTAAATATGAACGGTCAATCATATCAAATCCT

TTCTATGCAGAGGCAAGGGTATTTTTATCAAATTGAATCGTAAAACCTTGAATTCCCCCACCTGTGTAACAT

TCTTTAAAGCGATTGATTACCTCAGTATAGATTATCACAGATGAGCTTGTTGGCTTAATGCTAAATGTAAAT

TCCAATGGTAATCGGTTTTCAGATTTAGCATGTACTAGTCGTATCGATATTTCAGTTGTTTTGAGTTTTCTC

TGACGAAGTTTTGAAGTTGCTGTTTCAACGATTCCATGTAAAAATCTTTCAAGCATTTCAATATCATTACAT

CCTTTGCTACGGATTTCTGAAAATTGTACTGTATTTTTTTCTTGTTTCATTTTAATCCCTCCAATCCACCCG

CGGAATGACCACCGATAAGTTTACTGCGTTCAATATTTCTGGAACCTTCAGTTAGGACGGTTCCTTTTTGTA

TGGCTAAAAAACCAAACTGTTCTCTGACAACATCAATAGCTGTCTGAAGTCTATTATCTTTTTCAATTTGTT

CTACATCATCAAAGAGTGATAGTAGAGTATAGCTTTCATCTACGAAGCCACTATAAGATACACCAATTTGTC

TCACTGCACCAGAGGTGTATTTTTTCGGAATAATACAAGTACATGACTCACCATTGTTTTGGGGAGATTTG

CGGGTTCAATTTTATTCTGAGCATTTATAGATTTTTTCATCTCAGTCCTAGAATAGCCAATATGAATAGAAA

CGACAGTAGTCAATACTAGGGCTACTGTTCCGTTCCACAGTATCATTTAAAAATCATT-TTCACACCCTTTC

GTCTATTAGTATAGAAGAAAGCTCTCAGCACA

>3063-00/Mega-2.II

CATGTTGAGGCGGTAAGTTTGCTAGTCAAGGAGTAAAACGACGAAGATTAGCATTTACTTCCGCCCATGCGA

TAGCTGTCCGTGATTGACAAGTGCTAGCACGCAGACAGAACGGAGATAGCGAACCGCTGAGTGTGTCGCTCT

GCTCGTAAAAGCTTAGAAACCTTTGAACGAAAGGGATAATGAAAGCCTTGATTGCAAGGCTTTTTGCTTTAT

GGTGGGTAAGTATCAGAGTGAGAAAATTTTTGGAATGAGTAGAAGTGATAGCTAGAAATTATCAGTTTCTA

TTTCCATTTACCCTGTGGGTACGTGTTTGTTTCCATTGACAAGGAGTTTGTGGGAATAGAAATGT

ACCCACCTTGTTTGAATCAAGTGAAGTGTAGTTGAAGGAAATCTGTTGAAAGCAATACTTCATTTTACCG

AATAAGTAATAATTTAGGCAACTTCAAATCGATTAAAAAAAACTATTTTAAAGGTTAAGAGTAGACAAAA

ATTGTCCACTCTTTTTTGCAAACTCAATTTATCAATAAATGAAATGAGGGAATGTAAAATG

AAATATTTTGAGGTTGAGTTAGAAAATCCTGATGAATTTTTAAAACTACAAACAGAAGATTTTGTGAAAG

CTAATCGCTTGCTACTAAGGAAGATAATCCAGAGCGTTACAGTCTATGAAGAAAACTTCGTCATATCCTT

TAAATCTGGCATCGAATTGGAAGTATGAGTCTCATTCCATAACTTTTATATTGAACATATCATCTTGTTGTG

TTATACTATAAATTGATATAAACAAAGATGTAGGAGGAACCGAAACTATGACAGCCTCAATGCGTTTAAGAT

AAGCTGGCAATAAAAAAAGCAGAATCTATACCCGATGATAGGCTTTTTTGTTGTGCTTATTTATACGATATT

GAGCATTCATTAGTTACGGTGAGGATATTGGTTATTTAACTATACCTTTATTTAACTATGTCTTTAATATGA

ATGTTTCCAAATTGTATGTATGCAGACCAAAAGCCACATTGTGGGGTTTGGCCTGCATTTTTTTTGCCTA

GAATGCTATTCAAAATAGAAATTCAAGCAAAATAATATGCAGGAGATAATATAAATGGAAAAATACAACAAT

TGGAAACGAAAATTTTATGCAATATGGGCAGGGCAAGCAGTATCATTAATCACTAGTGCCATCCTGCAAATG

GCGATTATTTTTTACCTTACAGAAAAAACAGGATCTGCGATGGTCTTGTCTATGGCTTCATTAGTAGGTTTT

TTACCCTATGCGATTTTGGGACCTGCCATTGGTGTGCTAGTGGATCGTCATGATAGGAAGAAGATAATGATT

GGTGCCGATTTAATTATCGCAGCAGCTGGTGCAGTGCTTGCTATTGTTGCATTCTGTATGGAGCTACCTGTC

TGGATGATTATGATAGTATTGTTTATCCGTAGCATTGGAACAGCTTTTCATACCCCAGCACTCAATGCGGTT

ACACCACTTTTAGTACCAGAAGAACAGCTAACGAAATGCGCAGGCTATAGTCAGTCTTTGCAGTCTATAAGC

TATATTGTTAGTCCGGCAGTTGCAGCACTCTTATACTCCGTTTGGGATTTAAATGCTATTATTGCCATCGAC

GTATTGGGTGCTGTGATTGCATCTATTACGGTAGCAATTGTACGTATACCTAAGCTGGGTAATCAAGTGCAA

AGTTTAGAACCAAATTTCATAAGGGAGATGAAAGAAGGAGTTGTGGTTCTGAGACAAAACAAAGGATTGTTT

GCCTTATTACTCTTAGGAACACTATATACTTTTGTTTATATGCCAATCAATGCACTATTTCCTTTAATAAGC

ATGGAACACTTTAATGGAACGCCTGTGCATATTTCTATTACGGAAATTTCCTTTGCATTTGGGATGCTAGCA

GGAGGCTTATTATTAGGAAGATTAGGGGGCTTCGAAAAGCATGTATTACTAATAACAAGTTCATTTTTTATA

ATGGGGACCAGTTTAGCCGTTTCGGGAATACTTCCTCCAAATGGATTTGTAATATTCGTAGTTTGCTGTGCA

ATAATGGGGCTTTCGGTGCCATTTTATAGCGGTGTGCAAACAGCTCTTTTTCAGGAGAAAATTAAGCCTGAA

TATTTAGGACGTGTATTTTCTTTGATCGGAAGTATCATGTCACTTGCTATGCCAATTGGGTTAATTCTTTCT

GGATTCTTTGCTGATAAAATCGGTGTAAATCATTGGTTTTTACTATCAGGTATTTTAATTATTGGCATTGCT

ATAGTTTGCCAAATGATAACTGAGGTTAGAAAATTAGATTTAAAATAAACAATATTGGAGGAA

ATGTTATGGAATTAATATT

AAAAGCAAAAGACATTCGTGTGGAATTCAAAGGACGCGATGTTTTAGATATAAATGAATTAGAAGTATATGA

TTATGACCGTATTGGTTTAGTAGGAGCAAATGGTGCTGGAAAAAGCACTTTACTCAGGGTACTTTTAGGAGA

ATTAACTCCCCCAGGATGTAAAATGAATCGTCTGGGTGAACTTGCCTATATTCCCCAGTTGGACGAAGTAAC

TCTGCAGGAGGAAAAAGATTTTGCACTTGTAGGCAAGCTAGGTGTTGAGCAATTAAATATACAGACTATGAG

CGGTGGTGAAGAAACAAGGCTTAAAATAGCACAGGCCTTATCGGCACAGGTTCATGGTATTTTAGCGGATGA

ACCTACGAGCCATTTAGACCGTGAAGGAATTGATTTTCTAATAGGACAGCTAAAATATTTTACAGGTGCACT

GTTAGTTATTAGCCATGACCGCTATTTTCTTGATGAAATAGTAGATAAAATATGGGAACTGAAAGATGGCAA

AATCACTGAGTATTGGGGAAACTATTCTGATTATCTTCGTCAGAAAGAGGAAGAACGTAAGAGCCAAGCTGC

AGAATACGAACAATTTATTGCGGAACGTGCCCGATTGGAAAGGGCTGCGGAGGAAAAGCGAAAACAGGCTCG

TAAAATAGAACAGAAGGCAAAAGGTTCTTCAAAGAAAAAAAGTACTGAAGACGGAGGGCGTTTAGCTCATCA

AAAATCAATAGGAAGTAAGGAAAAAAAGATGTATAATGCTGCTAAAACCCTAGAGCACAGGATTGCGGCCTT

AGGAAAAGTAGAAGCTCCGGAAGGCATTCGCAGAATTCGTTTCAGGCAAAGTAAAGCATTGGAGCTCCATAA

TCCATACCCTATAGTCGGTGCAGAAATTAATAAAGTATTTGGGGATAAGGCTCTGTTTGAAAATGCATCTTT

TCAAATTCCGTTAGGAGCAAAAGTGGCGTTAACTGGTGGTAATGGAATCGGAAAAACAACTTTAATCCAAAT

GATCTTAAACCATGAAGAAGGAATTTCTATTTCGCCTAAGGCAAAAATAGGTTACTTTGCACAGAATGGTTA

CAAGTACAACAGTAATCAGAATGTTATGGAGTTTATGCAGAAGGATTGTGACTACAATATATCAGAAATTCG

TTCAGTGCTAGCATCTATGGGGTTCAAACAGAACGATATTGGAAAAAGTTTATCTGTTTTAAGCGGTGGAGA

AATTATAAAATTGTTGCTTGCTAAAATGCTCATGGGTAGATATAACATCCTAATAATGGATGAACCCAGTAA

CTTCCTTGACATACCAAGTTTAGAGGCTTTGGAAATACTAATGAAGGAGTACACCGGAACTATCGTGTTTAT

CACCCACGATAAACGATTACTCGAAAATGTAGCAGATGTAGTTTATGAAATTAGAGATAAGAAAATAAATCT

GAAACATTAAATTTAAGGTAGTCGCTGGTCAGTATAGTCTGTTCTGGTTGGCGACTCCATTGTTAAAGAGTA

TAAAGACTTTAGATTTTATGAATATTAAAAATAGGAACAGTCAATTGAACTGCTCCTATTTTTCTGCTAAAT

ATATTGTAGTTTTCTTATATGTATAATGATAGATTAGCGGATTCTCATCTACGGTACTTACTTCAAATATGA

AGAAGTGATCGCGGTTATCTCTGGACTTTTCCTTATTGAGGACAAAGTAATTCTTACGTGAAGTCGCCATTG

TTTTTAGGATATCATCAGTTAGGAAGGTCAATGGAATATTCATGTTAGAGTAGCGGTAGAAGTCACGTTCAA

AATCTTGGTAGCTCTCGCTATAATAGTCCATTTGTAGGTGATTACGCTGAAACTCAAGCTGATTCATAGAGC

ACCTCCTCGACAAGTTCAATACTAATAATGTCTTTTAATTTCAAATTGATGTGACCTGTTGTAGTTTTTATC

AAAATGAAATCTTTGGTCAGACTTGGTATTGTTCCAGTGTAGGAAACACGCTTGTTTTTTTCAATCACTTGA

ATGCGTGTGCGTAGCTGCCCGGCGTATACTTGACTGAGGAGTAATAATTTCTTCTCTAGTGATAAGTCAGAC

ATGTACGTTACTTTGTTTGTATCATCAGAGAGTGCTGATGCATGTTCAGATAGGAAAAAGCCCATCCATTTT

TGCATCTTTGTATCCTGGTACTCTCTTGCTGATTGAAATGGTAAATATGAACGGTCAATCATATCAAATCCT

TTCTATGCAGAGGCAAGGGTATTTTTATCAAATTGAATCGTAAAACCTTGAATTCCCCCACCTGTGTAACAT

TCTTTAAAGCGATTGATTACCTCAGTATAGATTATCACAGATGAGCTTGTTGGCTTAATGCTAAATGTAAAT

TCCAATGGTAATCGGTTTTCAGATTTAGCATGTACTAGTCGTATCGATATTTCAGTTGTTTTGAGTTTTCTC

TGACGAAGTTTTGAAGTTGCTGTTTCAACGATTCCATGTAAAAATCTTTCAAGCATTTCAATATCATTACAT

CCTTTGCTACGGATTTCTGAAAATTGTACTGTATTTTTTTCTTGTTTCATTTTAATCCCTCCAATCCACCCG

CGGAATGACCACCGATAAGTTTACTGCGTTCAATATTTCTGGAACCTTCAGTTAGGACGGTTCCTTTTTGTA

TGGCTAAAAAACCAAACTGTTCTCTGACAACATCAATAGCTGTCTGAAGTCTATTATCTTTTTCAATTTGTT

CTACATCATCAAAGAGTGATAGTAGAGTATAGCTTTCATCTACGAAGCCACTATAAGATACACCAATTTGTC

TCACTGCACCAGAGGTGTATTTTTTCGGAATAATACAAGTACATGACTCACCATTGTTTTGGGGAGATTTG

CGGGTTCAATTTTATTCTGAGCATTTATAGATTTTTTCATCTCAGTCCTAGAATAGCCAATATGAATAGAAA

CGACAGTAGTCAATACTAGGGCTACTGTTCCGTTCCACAGTATCATTTAAAAATCATT-TTCACACCCTTTC

GTCTATTAGTATAGAAGAAAGCTCTCAGCACA

>GA54644/Mega-2.II

CATGTTGAGGCGGTAAGTTTGCTAGTCAAGGAGTAAAACGACGAAGATTAGCATTTACTTCCGCCCATGCGA

TAGCTGTCCGTGATTGACAAGTGCTAGCACGCAGACAGAACGGAGATAGCGAACCGCTGAGTGTGTCGCTCT

GCTCGTAAAAGCTTAGAAACCTTTGAACGAAAGGGATAATGAAAGCCTTGATTGCAAGGCTTTTTGCTTTAT

GGTGGGTAAGTATCAGAGTGAGAAAATTTTTGGAATGAGTAGAAGTGATAGCTAGAAATTATCAGTTTCTA

TTTCCATTTACCCTGTGGGTACGTGTTTGTTTCCATTGACAAGGAGTTTGTGGGAATAGAAATGT

ACCCACCTTGTTTGAATCAAGTGAAGTGTAGTTGAAGGAAATCTGTTGAAAGCAATACTTCATTTTACCG

AATAAGTAATAATTTAGGCAACTTCAAATCGATTAAAAAAAACTATTTTAAAGGTTAAGAGTAGACAAAA

ATTGTCCACTCTTTTTTGCAAACTCAATTTATCAATAAATGAAATGAGGGAATGTAAAATG

AAATATTTTGAGGTTGAGTTAGAAAATCCTGATGAATTTTTAAAACTACAAACAGAAGATTTTGTGAAAG

CTAATCGCTTGCTACTAAGGAAGATAATCCAGAGCGTTACAGTCTATGAAGAAAACTTCGTCATATCCTT

TAAATCTGGCATCGAATTGGAAGTATGAGTCTCATTCCATAACTTTTATATTGAACATATCATCTTGTTGTG

TTATACTATAAATTGATATAAACAAAGATGTAGGAGGAACCGAAACTATGACAGCCTCAATGCGTTTAAGAT

AAGCTGGCAATAAAAAAAGCAGAATCTATACCCGATGATAGGCTTTTTTGTTGTGCTTATTTATACGATATT

GAGCATTCATTAGTTACGGTGAGGATATTGGTTATTTAACTATACCTTTATTTAACTATGTCTTTAATATGA

ATGTTTCCAAATTGTATGTATGCAGACCAAAAGCCACATTGTGGGGTTTGGCCTGCATTTTTTTTGCCTA

GAATGCTATTCAAAATAGAAATTCAAGCAAAATAATATGCAGGAGATAATATAAATGGAAAAATACAACAAT

TGGAAACGAAAATTTTATGCAATATGGGCAGGGCAAGCAGTATCATTAATCACTAGTGCCATCCTGCAAATG

GCGATTATTTTTTACCTTACAGAAAAAACAGGATCTGCGATGGTCTTGTCTATGGCTTCATTAGTAGGTTTT

TTACCCTATGCGATTTTGGGACCTGCCATTGGTGTGCTAGTGGATCGTCATGATAGGAAGAAGATAATGATT

GGTGCCGATTTAATTATCGCAGCAGCTGGTGCAGTGCTTGCTATTGTTGCATTCTGTATGGAGCTACCTGTC

TGGATGATTATGATAGTATTGTTTATCCGTAGCATTGGAACAGCTTTTCATACCCCAGCACTCAATGCGGTT

ACACCACTTTTAGTACCAGAAGAACAGCTAACGAAATGCGCAGGCTATAGTCAGTCTTTGCAGTCTATAAGC

TATATTGTTAGTCCGGCAGTTGCAGCACTCTTATACTCCGTTTGGGATTTAAATGCTATTATTGCCATCGAC

GTATTGGGTGCTGTGATTGCATCTATTACGGTAGCAATTGTACGTATACCTAAGCTGGGTAATCAAGTGCAA

AGTTTAGAACCAAATTTCATAAGGGAGATGAAAGAAGGAGTTGTGGTTCTGAGACAAAACAAAGGATTGTTT

GCCTTATTACTCTTAGGAACACTATATACTTTTGTTTATATGCCAATCAATGCACTATTTCCTTTAATAAGC

ATGGAACACTTTAATGGAACGCCTGTGCATATTTCTATTACGGAAATTTCCTTTGCATTTGGGATGCTAGCA

GGAGGCTTATTATTAGGAAGATTAGGGGGCTTCGAAAAGCATGTATTACTAATAACAAGTTCATTTTTTATA

ATGGGGACCAGTTTAGCCGTTTCGGGAATACTTCCTCCAAATGGATTTGTAATATTCGTAGTTTGCTGTGCA

ATAATGGGGCTTTCGGTGCCATTTTATAGCGGTGTGCAAACAGCTCTTTTTCAGGAGAAAATTAAGCCTGAA

TATTTAGGACGTGTATTTTCTTTGATCGGAAGTATCATGTCACTTGCTATGCCAATTGGGTTAATTCTTTCT

GGATTCTTTGCTGATAAAATCGGTGTAAATCATTGGTTTTTACTATCAGGTATTTTAATTATTGGCATTGCT

ATAGTTTGCCAAATGATAACTGAGGTTAGAAAATTAGATTTAAAATAAACAATATTGGAGGAA

ATGTTATGGAATTAATATT

AAAAGCAAAAGACATTCGTGTGGAATTCAAAGGACGCGATGTTTTAGATATAAATGAATTAGAAGTATATGA

TTATGACCGTATTGGTTTAGTAGGAGCAAATGGTGCTGGAAAAAGCACTTTACTCAGGGTACTTTTAGGAGA

ATTAACTCCCCCAGGATGTAAAATGAATCGTCTGGGTGAACTTGCCTATATTCCCCAGTTGGACGAAGTAAC

TCTGCAGGAGGAAAAAGATTTTGCACTTGTAGGCAAGCTAGGTGTTGAGCAATTAAATATACAGACTATGAG

CGGTGGTGAAGAAACAAGGCTTAAAATAGCACAGGCCTTATCGGCACAGGTTCATGGTATTTTAGCGGATGA

ACCTACGAGCCATTTAGACCGTGAAGGAATTGATTTTCTAATAGGACAGCTAAAATATTTTACAGGTGCACT

GTTAGTTATTAGCCATGACCGCTATTTTCTTGATGAAATAGTAGATAAAATATGGGAACTGAAAGATGGCAA

AATCACTGAGTATTGGGGAAACTATTCTGATTATCTTCGTCAGAAAGAGGAAGAACGTAAGAGCCAAGCTGC

AGAATACGAACAATTTATTGCGGAACGTGCCCGATTGGAAAGGGCTGCGGAGGAAAAGCGAAAACAGGCTCG

TAAAATAGAACAGAAGGCAAAAGGTTCTTCAAAGAAAAAAAGTACTGAAGACGGAGGGCGTTTAGCTCATCA

AAAATCAATAGGAAGTAAGGAAAAAAAGATGTATAATGCTGCTAAAACCCTAGAGCACAGGATTGCGGCCTT

AGGAAAAGTAGAAGCTCCGGAAGGCATTCGCAGAATTCGTTTCAGGCAAAGTAAAGCATTGGAGCTCCATAA

TCCATACCCTATAGTCGGTGCAGAAATTAATAAAGTATTTGGGGATAAGGCTCTGTTTGAAAATGCATCTTT

TCAAATTCCGTTAGGAGCAAAAGTGGCGTTAACTGGTGGTAATGGAATCGGAAAAACAACTTTAATCCAAAT

GATCTTAAACCATGAAGAAGGAATTTCTATTTCGCCTAAGGCAAAAATAGGTTACTTTGCACAGAATGGTTA

CAAGTACAACAGTAATCAGAATGTTATGGAGTTTATGCAGAAGGATTGTGACTACAATATATCAGAAATTCG

TTCAGTGCTAGCATCTATGGGGTTCAAACAGAACGATATTGGAAAAAGTTTATCTGTTTTAAGCGGTGGAGA

AATTATAAAATTGTTGCTTGCTAAAATGCTCATGGGTAGATATAACATCCTAATAATGGATGAACCCAGTAA

CTTCCTTGACATACCAAGTTTAGAGGCTTTGGAAATACTAATGAAGGAGTACACCGGAACTATCGTGTTTAT

CACCCACGATAAACGATTACTCGAAAATGTAGCAGATGTAGTTTATGAAATTAGAGATAAGAAAATAAATCT

GAAACATTAAATTTAAGGTAGTCGCTGGTCAGTATAGTCTGTTCTGGTTGGCGACTCCATTGTTAAAGAGTA

TAAAGACTTTAGATTTTATGAATATTAAAAATAGGAACAGTCAATTGAACTGCTCCTATTTTTCTGCTAAAT

ATATTGTAGTTTTCTTATATGTATAATGATAGATTAGCGGATTCTCATCTACGGTACTTACTTCAAATATGA

AGAAGTGATCGCGGTTATCTCTGGACTTTTCCTTATTGAGGACAAAGTAATTCTTACGTGAAGTCGCCATTG

TTTTTAGGATATCATCAGTTAGGAAGGTCAATGGAATATTCATGTTAGAGTAGCGGTAGAAGTCACGTTCAA

AATCTTGGTAGCTCTCGCTATAATAGTCCATTTGTAGGTGATTACGCTGAAACTCAAGCTGATTCATAGAGC

ACCTCCTCGACAAGTTCAATACTAATAATGTCTTTTAATTTCAAATTGATGTGACCTGTTGTAGTTTTTATC

AAAATGAAATCTTTGGTCAGACTTGGTATTGTTCCAGTGTAGGAAACACGCTTGTTTTTTTCAATCACTTGA

ATGCGTGTGCGTAGCTGCCCGGCGTATACTTGACTGAGGAGTAATAATTTCTTCTCTAGTGATAAGTCAGAC

ATGTACGTTACTTTGTTTGTATCATCAGAGAGTGCTGATGCATGTTCAGATAGGAAAAAGCCCATCCATTTT

TGCATCTTTGTATCCTGGTACTCTCTTGCTGATTGAAATGGTAAATATGAACGGTCAATCATATCAAATCCT

TTCTATGCAGAGGCAAGGGTATTTTTATCAAATTGAATCGTAAAACCTTGAATTCCCCCACCTGTGTAACAT

TCTTTAAAGCGATTGATTACCTCAGTATAGATTATCACAGATGAGCTTGTTGGCTTAATGCTAAATGTAAAT

TCCAATGGTAATCGGTTTTCAGATTTAGCATGTACTAGTCGTATCGATATTTCAGTTGTTTTGAGTTTTCTC

TGACGAAGTTTTGAAGTTGCTGTTTCAACGATTCCATGTAAAAATCTTTCAAGCATTTCAATATCATTACAT

CCTTTGCTACGGATTTCTGAAAATTGTACTGTATTTTTTTCTTGTTTCATTTTAATCCCTCCAATCCACCCG

CGGAATGACCACCGATAAGTTTACTGCGTTCAATATTTCTGGAACCTTCAGTTAGGACGGTTCCTTTTTGTA

TGGCTAAAAAACCAAACTGTTCTCTGACAACATCAATAGCTGTCTGAAGTCTATTATCTTTTTCAATTTGTT

CTACATCATCAAAGAGTGATAGTAGAGTATAGCTTTCATCTACGAAGCCACTATAAGATACACCAATTTGTC

TCACTGCACCAGAGGTGTATTTTTTCGGAATAATACAAGTACATGACTCACCATTGTTTTGGGGAGATTTG

CGGGTTCAATTTTATTCTGAGCATTTATAGATTTTTTCATCTCAGTCCTAGAATAGCCAATATGAATAGAAA

CGACAGTAGTCAATACTAGGGCTACTGTTCCGTTCCACAGTATCATTTAAAAATCATT-TTCACACCCTTTC

GTCTATTAGTATAGAAGAAAGCTCTCAGCACA

>GA47033/Mega-1.II

CATGTTGAGGCGGTAAGTTTGCTAGTCAAGGAGTAAAACGACGAAGATTAGCATTTACTTCCGCCCATGCGA

TAGCTGTCCGTGATTGACAAGTGCTAGCACGCAGACAGAACGGAGATAGCGAACCGCTGAGTGTGTCGCTCT

GCTCGTAAAAGCTTAGAAACCTTTGAACGAAAGGGATAATGAAAGCCTTGATTGCAAGGCTTTTTGCTTTAT

GGTGGGTAAGTATCAGAGTGAGAAAATTTTTGGAATGAGTAGAAGTGATAGCTAGAAATTATCAGTTTCTA

TTTCCATTTACCCTGTGGGTACGTGTTTGTTTCCATTGACAAGGAGTTTGTGGGAATAGAAATGT

ACCCACCTTGTTTGAATCAAGTGAAGTGTAGTTGAAGGAAATCTGTTGAAAGCAATACTTCATTTTACCG

AATAAGTAATAATTTAGGCAACTTCAAATCGATTAAAAAAAACTATTTTAAAGGTTAAGAGTAGACAAAA

ATTGTCCACTCTTTTTTGCAAACTCAATTTATCAATAAATGAAATGAGGGAATGTAAAATG

AAATATTTTGAGGTTGAGTTAGAAAATCCTGATGAATTTTTAAAACTACAAACAGAAGATTTTGTGAAAG

CTAATCGCTTGCTACTAAGGAAGATAATCCAGAGCGTTACAGTCTATGAAGAAAACTTCGTCATATCCTT

TAAATCTGGCATCGAATTGGAAGTATGAGTCTCATTCCATAACTTTTATATTGAACATATCATCTTGTTGTG

TTATACTATAAATTGATATAAACAAAGATGTAGGAGGAACCGAAACTATGACAGCCTCAATGCGTTTAAGAT

AAGCTGGCAATAAAAAAAGCAGAATCTATACCCGATGATAGGCTTTTTTGTTGTGCTTATTTATACGATATT

GAGCATTCATTAGTTACGGTGAGGATATTGGTTATTTAACTATACCTTTATTTAACTATGTCTTTAATATGA

ATGTTTCCAAATTGTATGTATGCAGACCAAAAGCCACATTGTGGGGTTTGGCCTGCATTTTTTTTGCCTA

GAATGCTATTCAAAATAGAAATTCAAGCAAAATAATATGCAGGAGATAATATAAATGGAAAAATACAACAAT

TGGAAACGAAAATTTTATGCAATATGGGCAGGGCAAGCAGTATCATTAATCACTAGTGCCATCCTGCAAATG

GCGATTATTTTTTACCTTACAGAAAAAACAGGATCTGCGATGGTCTTGTCTATGGCTTCATTAGTAGGTTTT

TTACCCTATGCGATTTTGGGACCTGCCATTGGTGTGCTAGTGGATCGTCATGATAGGAAGAAGATAATGATT

GGTGCCGATTTAATTATCGCAGCAGCTGGTGCAGTGCTTGCTATTGTTGCATTCTGTATGGAGCTACCTGTC

TGGATGATTATGATAGTATTGTTTATCCGTAGCATTGGAACAGCTTTTCATACCCCAGCACTCAATGCGGTT

ACACCACTTTTAGTACCAGAAGAACAGCTAACGAAATGCGCAGGCTATAGTCAGTCTTTGCAGTCTATAAGC

TATATTGTTAGTCCGGCAGTTGCAGCACTCTTATACTCCGTTTGGGATTTAAATGCTATTATTGCCATCGAC

GTATTGGGTGCTGTGATTGCATCTATTACGGTAGCAATTGTACGTATACCTAAGCTGGGTAATCAAGTGCAA

AGTTTAGAACCAAATTTCATAAGGGAGATGAAAGAAGGAGTTGTGGTTCTGAGACAAAACAAAGGATTGTTT

GCCTTATTACTCTTAGGAACACTATATACTTTTGTTTATATGCCAATCAATGCACTATTTCCTTTAATAAGC

ATGGAACACTTTAATGGAACGCCTGTGCATATTTCTATTACGGAAATTTCCTTTGCATTTGGGATGCTAGCA

GGAGGCTTATTATTAGGAAGATTAGGGGGCTTCGAAAAGCATGTATTACTAATAACAAGTTCATTTTTTATA

ATGGGGACCAGTTTAGCCGTTTCGGGAATACTTCCTCCAAATGGATTTGTAATATTCGTAGTTTGCTGTGCA

ATAATGGGGCTTTCGGTGCCATTTTATAGCGGTGTGCAAACAGCTCTTTTTCAGGAGAAAATTAAGCCTGAA

TATTTAGGACGTGTATTTTCTTTGATCGGAAGTATCATGTCACTTGCTATGCCAATTGGGTTAATTCTTTCT

GGATTCTTTGCTGATAAAATCGGTGTAAATCATTGGTTTTTACTATCAGGTATTTTAATTATTGGCATTGCT

ATAGTTTGCCAAATGATAACTGAGGTTAGAAAATTAGATTTAAAATAAACAATATTGGAGGAATATTTATGT

ATCTTATTTTCATGTAACTCTTCCTGCTAAAATCGCAGGGTTTTCCCTGCATACAAGCAAATGAAAGCATGC

GATTATAGACAGGAGGAAATGTTATGGAATTAATATT

AAAAGCAAAAGACATTCGTGTGGAATTCAAAGGACGCGATGTTTTAGATATAAATGAATTAGAAGTATATGA

TTATGACCGTATTGGTTTAGTAGGAGCAAATGGTGCTGGAAAAAGCACTTTACTCAGGGTACTTTTAGGAGA

ATTAACTCCCCCAGGATGTAAAATGAATCGTCTGGGTGAACTTGCCTATATTCCCCAGTTGGACGAAGTAAC

TCTGCAGGAGGAAAAAGATTTTGCACTTGTAGGCAAGCTAGGTGTTGAGCAATTAAATATACAGACTATGAG

CGGTGGTGAAGAAACAAGGCTTAAAATAGCACAGGCCTTATCGGCACAGGTTCATGGTATTTTAGCGGATGA

ACCTACGAGCCATTTAGACCGTGAAGGAATTGATTTTCTAATAGGACAGCTAAAATATTTTACAGGTGCACT

GTTAGTTATTAGCCATGACCGCTATTTTCTTGATGAAATAGTAGATAAAATATGGGAACTGAAAGATGGCAA

AATCACTGAGTATTGGGGAAACTATTCTGATTATCTTCGTCAGAAAGAGGAAGAACGTAAGAGCCAAGCTGC

AGAATACGAACAATTTATTGCGGAACGTGCCCGATTGGAAAGGGCTGCGGAGGAAAAGCGAAAACAGGCTCG

TAAAATAGAACAGAAGGCAAAAGGTTCTTCAAAGAAAAAAAGTACTGAAGACGGAGGGCGTTTAGCTCATCA

AAAATCAATAGGAAGTAAGGAAAAAAAGATGTATAATGCTGCTAAAACCCTAGAGCACAGGATTGCGGCCTT

AGGAAAAGTAGAAGCTCCGGAAGGCATTCGCAGAATTCGTTTCAGGCAAAGTAAAGCATTGGAGCTCCATAA

TCCATACCCTATAGTCGGTGCAGAAATTAATAAAGTATTTGGGGATAAGGCTCTGTTTGAAAATGCATCTTT

TCAAATTCCGTTAGGAGCAAAAGTGGCGTTAACTGGTGGTAATGGAATCGGAAAAACAACTTTAATCCAAAT

GATCTTAAACCATGAAGAAGGAATTTCTATTTCGCCTAAGGCAAAAATAGGTTACTTTGCACAGAATGGTTA

CAAGTACAACAGTAATCAGAATGTTATGGAGTTTATGCAGAAGGATTGTGACTACAATATATCAGAAATTCG

TTCAGTGCTAGCATCTATGGGGTTCAAACAGAACGATATTGGAAAAAGTTTATCTGTTTTAAGCGGTGGAGA

AATTATAAAATTGTTGCTTGCTAAAATGCTCATGGGTAGATATAACATCCTAATAATGGATGAACCCAGTAA

CTTCCTTGACATACCAAGTTTAGAGGCTTTGGAAATACTAATGAAGGAGTACACCGGAACTATCGTGTTTAT

CACCCACGATAAACGATTACTCGAAAATGTAGCAGATGTAGTTTATGAAATTAGAGATAAGAAAATAAATCT

GAAACATTAAATTTAAGGTAGTCGCTGGTCAGTATAGTCTGTTCTGGTTGGCGACTCCATTGTTAAAGAGTA

TAAAGACTTTAGATTTTATGAATATTAAAAATAGGAACAGTCAATTGAACTGCTCCTATTTTTCTGCTAAAT

ATATTGTAGTTTTCTTATATGTATAATGATAGATTAGCGGATTCTCATCTACGGTACTTACTTCAAATATGA

AGAAGTGATCGCGGTTATCTCTGGACTTTTCCTTATTGAGGACAAAGTAATTCTTACGTGAAGTCGCCATTG

TTTTTAGGATATCATCAGTTAGGAAGGTCAATGGAATATTCATGTTAGAGTAGCGGTAGAAGTCACGTTCAA

AATCTTGGTAGCTCTCGCTATAATAGTCCATTTGTAGGTGATTACGCTGAAACTCAAGCTGATTCATAGAGC

ACCTCCTCGACAAGTTCAATACTAATAATGTCTTTTAATTTCAAATTGATGTGACCTGTTGTAGTTTTTATC

AAAATGAAATCTTTGGTCAGACTTGGTATTGTTCCAGTGTAGGAAACACGCTTGTTTTTTTCAATCACTTGA

ATGCGTGTGCGTAGCTGCCCGGCGTATACTTGACTGAGGAGTAATAATTTCTTCTCTAGTGATAAGTCAGAC

ATGTACGTTACTTTGTTTGTATCATCAGAGAGTGCTGATGCATGTTCAGATAGGAAAAAGCCCATCCATTTT

TGCATCTTTGTATCCTGGTACTCTCTTGCTGATTGAAATGGTAAATATGAACGGTCAATCATATCAAATCCT

TTCTATGCAGAGGCAAGGGTATTTTTATCAAATTGAATCGTAAAACCTTGAATTCCCCCACCTGTGTAACAT

TCTTTAAAGCGATTGATTACCTCAGTATAGATTATCACAGATGAGCTTGTTGGCTTAATGCTAAATGTAAAT

TCCAATGGTAATCGGTTTTCAGATTTAGCATGTACTAGTCGTATCGATATTTCAGTTGTTTTGAGTTTTCTC

TGACGAAGTTTTGAAGTTGCTGTTTCAACGATTCCATGTAAAAATCTTTCAAGCATTTCAATATCATTACAT

CCTTTGCTACGGATTTCTGAAAATTGTACTGTATTTTTTTCTTGTTTCATTTTAATCCCTCCAATCCACCCG

CGGAATGACCACCGATAAGTTTACTGCGTTCAATATTTCTGGAACCTTCAGTTAGGACGGTTCCTTTTTGTA

TGGCTAAAAAACCAAACTGTTCTCTGACAACATCAATAGCTGTCTGAAGTCTATTATCTTTTTCAATTTGTT

CTACATCATCAAAGAGTGATAGTAGAGTATAGCTTTCATCTACGAAGCCACTATAAGATACACCAATTTGTC

TCACTGCACCAGAGGTGTATTTTTTCGGAATAATACAAGTACATGACTCACCATTGTTTTGGGGAGATTTG

CGGGTTCAATTTTATTCTGAGCATTTATAGATTTTTTCATCTCAGTCCTAGAATAGCCAATATGAATAGAAA

CGACAGTAGTCAATACTAGGGCTACTGTTCCGTTCCACAGTATCATTTAAAAATCATT-TTCACACCCTTTC

GTCTATTAGTATAGAAGAAAGCTCTCAGCACA

>GA52306/Mega-1.II

CATGTTGAGGCGGTAAGTTTGCTAGTCAAGGAGTAAAACGACGAAGATTAGCATTTACTTCCGCCCATGCGA

TAGCTGTCCGTGATTGACAAGTGCTAGCACGCAGACAGAACGGAGATAGCGAACCGCTGAGTGTGTCGCTCT

GCTCGTAAAAGCTTAGAAACCTTTGAACGAAAGGGATAATGAAAGCCTTGATTGCAAGGCTTTTTGCTTTAT

GGTGGGTAAGTATCAGAGTGAGAAAATTTTTGGAATGAGTAGAAGTGATAGCTAGAAATTATCAGTTTCTA

TTTCCATTTACCCTGTGGGTACGTGTTTGTTTCCATTGACAAGGAGTTTGTGGGAATAGAAATGT

ACCCACCTTGTTTGAATCAAGTGAAGTGTAGTTGAAGGAAATCTGTTGAAAGCAATACTTCATTTTACCG

AATAAGTAATAATTTAGGCAACTTCAAATCGATTAAAAAAAACTATTTTAAAGGTTAAGAGTAGACAAAA

ATTGTCCACTCTTTTTTGCAAACTCAATTTATCAATAAATGAAATGAGGGAATGTAAAATG

AAATATTTTGAGGTTGAGTTAGAAAATCCTGATGAATTTTTAAAACTACAAACAGAAGATTTTGTGAAAG

CTAATCGCTTGCTACTAAGGAAGATAATCCAGAGCGTTACAGTCTATGAAGAAAACTTCGTCATATCCTT

TAAATCTGGCATCGAATTGGAAGTATGAGTCTCATTCCATAACTTTTATATTGAACATATCATCTTGTTGTG

TTATACTATAAATTGATATAAACAAAGATGTAGGAGGAACCGAAACTATGACAGCCTCAATGCGTTTAAGAT

AAGCTGGCAATAAAAAAAGCAGAATCTATACCCGATGATAGGCTTTTTTGTTGTGCTTATTTATACGATATT

GAGCATTCATTAGTTACGGTGAGGATATTGGTTATTTAACTATACCTTTATTTAACTATGTCTTTAATATGA

ATGTTTCCAAATTGTATGTATGCAGACCAAAAGCCACATTGTGGGGTTTGGCCTGCATTTTTTTTGCCTA

GAATGCTATTCAAAATAGAAATTCAAGCAAAATAATATGCAGGAGATAATATAAATGGAAAAATACAACAAT

TGGAAACGAAAATTTTATGCAATATGGGCAGGGCAAGCAGTATCATTAATCACTAGTGCCATCCTGCAAATG

GCGATTATTTTTTACCTTACAGAAAAAACAGGATCTGCGATGGTCTTGTCTATGGCTTCATTAGTAGGTTTT

TTACCCTATGCGATTTTGGGACCTGCCATTGGTGTGCTAGTGGATCGTCATGATAGGAAGAAGATAATGATT

GGTGCCGATTTAATTATCGCAGCAGCTGGTGCAGTGCTTGCTATTGTTGCATTCTGTATGGAGCTACCTGTC

TGGATGATTATGATAGTATTGTTTATCCGTAGCATTGGAACAGCTTTTCATACCCCAGCACTCAATGCGGTT

ACACCACTTTTAGTACCAGAAGAACAGCTAACGAAATGCGCAGGCTATAGTCAGTCTTTGCAGTCTATAAGC

TATATTGTTAGTCCGGCAGTTGCAGCACTCTTATACTCCGTTTGGGATTTAAATGCTATTATTGCCATCGAC

GTATTGGGTGCTGTGATTGCATCTATTACGGTAGCAATTGTACGTATACCTAAGCTGGGTAATCAAGTGCAA

AGTTTAGAACCAAATTTCATAAGGGAGATGAAAGAAGGAGTTGTGGTTCTGAGACAAAACAAAGGATTGTTT

GCCTTATTACTCTTAGGAACACTATATACTTTTGTTTATATGCCAATCAATGCACTATTTCCTTTAATAAGC

ATGGAACACTTTAATGGAACGCCTGTGCATATTTCTATTACGGAAATTTCCTTTGCATTTGGGATGCTAGCA

GGAGGCTTATTATTAGGAAGATTAGGGGGCTTCGAAAAGCATGTATTACTAATAACAAGTTCATTTTTTATA

ATGGGGACCAGTTTAGCCGTTTCGGGAATACTTCCTCCAAATGGATTTGTAATATTCGTAGTTTGCTGTGCA

ATAATGGGGCTTTCGGTGCCATTTTATAGCGGTGTGCAAACAGCTCTTTTTCAGGAGAAAATTAAGCCTGAA

TATTTAGGACGTGTATTTTCTTTGATCGGAAGTATCATGTCACTTGCTATGCCAATTGGGTTAATTCTTTCT

GGATTCTTTGCTGATAAAATCGGTGTAAATCATTGGTTTTTACTATCAGGTATTTTAATTATTGGCATTGCT

ATAGTTTGCCAAATGATAACTGAGGTTAGAAAATTAGATTTAAAATAAACAATATTGGAGGAATATTTATGT

ATCTTATTTTCATGTAACTCTTCCTGCTAAAATCGCAGGGTTTTCCCTGCATACAAGCAAATGAAAGCATGC

GATTATAGACAGGAGGAAATGTTATGGAATTAATATT

AAAAGCAAAAGACATTCGTGTGGAATTCAAAGGACGCGATGTTTTAGATATAAATGAATTAGAAGTATATGA

TTATGACCGTATTGGTTTAGTAGGAGCAAATGGTGCTGGAAAAAGCACTTTACTCAGGGTACTTTTAGGAGA

ATTAACTCCCCCAGGATGTAAAATGAATCGTCTGGGTGAACTTGCCTATATTCCCCAGTTGGACGAAGTAAC

TCTGCAGGAGGAAAAAGATTTTGCACTTGTAGGCAAGCTAGGTGTTGAGCAATTAAATATACAGACTATGAG

CGGTGGTGAAGAAACAAGGCTTAAAATAGCACAGGCCTTATCGGCACAGGTTCATGGTATTTTAGCGGATGA

ACCTACGAGCCATTTAGACCGTGAAGGAATTGATTTTCTAATAGGACAGCTAAAATATTTTACAGGTGCACT

GTTAGTTATTAGCCATGACCGCTATTTTCTTGATGAAATAGTAGATAAAATATGGGAACTGAAAGATGGCAA

AATCACTGAGTATTGGGGAAACTATTCTGATTATCTTCGTCAGAAAGAGGAAGAACGTAAGAGCCAAGCTGC

AGAATACGAACAATTTATTGCGGAACGTGCCCGATTGGAAAGGGCTGCGGAGGAAAAGCGAAAACAGGCTCG

TAAAATAGAACAGAAGGCAAAAGGTTCTTCAAAGAAAAAAAGTACTGAAGACGGAGGGCGTTTAGCTCATCA

AAAATCAATAGGAAGTAAGGAAAAAAAGATGTATAATGCTGCTAAAACCCTAGAGCACAGGATTGCGGCCTT

AGGAAAAGTAGAAGCTCCGGAAGGCATTCGCAGAATTCGTTTCAGGCAAAGTAAAGCATTGGAGCTCCATAA

TCCATACCCTATAGTCGGTGCAGAAATTAATAAAGTATTTGGGGATAAGGCTCTGTTTGAAAATGCATCTTT

TCAAATTCCGTTAGGAGCAAAAGTGGCGTTAACTGGTGGTAATGGAATCGGAAAAACAACTTTAATCCAAAT

GATCTTAAACCATGAAGAAGGAATTTCTATTTCGCCTAAGGCAAAAATAGGTTACTTTGCACAGAATGGTTA

CAAGTACAACAGTAATCAGAATGTTATGGAGTTTATGCAGAAGGATTGTGACTACAATATATCAGAAATTCG

TTCAGTGCTAGCATCTATGGGGTTCAAACAGAACGATATTGGAAAAAGTTTATCTGTTTTAAGCGGTGGAGA

AATTATAAAATTGTTGCTTGCTAAAATGCTCATGGGTAGATATAACATCCTAATAATGGATGAACCCAGTAA

CTTCCTTGACATACCAAGTTTAGAGGCTTTGGAAATACTAATGAAGGAGTACACCGGAACTATCGTGTTTAT

CACCCACGATAAACGATTACTCGAAAATGTAGCAGATGTAGTTTATGAAATTAGAGATAAGAAAATAAATCT

GAAACATTAAATTTAAGGTAGTCGCTGGTCAGTATAGTCTGTTCTGGTTGGCGACTCCATTGTTAAAGAGTA

TAAAGACTTTAGATTTTATGAATATTAAAAATAGGAACAGTCAATTGAACTGCTCCTATTTTTCTGCTAAAT

ATATTGTAGTTTTCTTATATGTATAATGATAGATTAGCGGATTCTCATCTACGGTACTTACTTCAAATATGA

AGAAGTGATCGCGGTTATCTCTGGACTTTTCCTTATTGAGGACAAAGTAATTCTTACGTGAAGTCGCCATTG

TTTTTAGGATATCATCAGTTAGGAAGGTCAATGGAATATTCATGTTAGAGTAGCGGTAGAAGTCACGTTCAA

AATCTTGGTAGCTCTCGCTATAATAGTCCATTTGTAGGTGATTACGCTGAAACTCAAGCTGATTCATAGAGC

ACCTCCTCGACAAGTTCAATACTAATAATGTCTTTTAATTTCAAATTGATGTGACCTGTTGTAGTTTTTATC

AAAATGAAATCTTTGGTCAGACTTGGTATTGTTCCAGTGTAGGAAACACGCTTGTTTTTTTCAATCACTTGA

ATGCGTGTGCGTAGCTGCCCGGCGTATACTTGACTGAGGAGTAATAATTTCTTCTCTAGTGATAAGTCAGAC

ATGTACGTTACTTTGTTTGTATCATCAGAGAGTGCTGATGCATGTTCAGATAGGAAAAAGCCCATCCATTTT

TGCATCTTTGTATCCTGGTACTCTCTTGCTGATTGAAATGGTAAATATGAACGGTCAATCATATCAAATCCT

TTCTATGCAGAGGCAAGGGTATTTTTATCAAATTGAATCGTAAAACCTTGAATTCCCCCACCTGTGTAACAT

TCTTTAAAGCGATTGATTACCTCAGTATAGATTATCACAGATGAGCTTGTTGGCTTAATGCTAAATGTAAAT

TCCAATGGTAATCGGTTTTCAGATTTAGCATGTACTAGTCGTATCGATATTTCAGTTGTTTTGAGTTTTCTC

TGACGAAGTTTTGAAGTTGCTGTTTCAACGATTCCATGTAAAAATCTTTCAAGCATTTCAATATCATTACAT

CCTTTGCTACGGATTTCTGAAAATTGTACTGTATTTTTTTCTTGTTTCATTTTAATCCCTCCAATCCACCCG

CGGAATGACCACCGATAAGTTTACTGCGTTCAATATTTCTGGAACCTTCAGTTAGGACGGTTCCTTTTTGTA

TGGCTAAAAAACCAAACTGTTCTCTGACAACATCAATAGCTGTCTGAAGTCTATTATCTTTTTCAATTTGTT

CTACATCATCAAAGAGTGATAGTAGAGTATAGCTTTCATCTACGAAGCCACTATAAGATACACCAATTTGTC

TCACTGCACCAGAGGTGTATTTTTTCGGAATAATACAAGTACATGACTCACCATTGTTTTGGGGAGATTTG

CGGGTTCAATTTTATTCTGAGCATTTATAGATTTTTTCATCTCAGTCCTAGAATAGCCAATATGAATAGAAA

CGACAGTAGTCAATACTAGGGCTACTGTTCCGTTCCACAGTATCATTTAAAAATCATT-TTCACACCCTTTC

GTCTATTAGTATAGAAGAAAGCTCTCAGCACA

>GA07643/Mega-1.II

CATGTTGAGGCGGTAAGTTTGCTAGTCAAGGAGTAAAACGACGAAGATTAGCATTTACTTCCGCCCATGCGA

TAGCTGTCCGTGATTGACAAGTGCTAGCACGCAGACAGAACGGAGATAGCGAACCGCTGAGTGTGTCGCTCT

GCTCGTAAAAGCTTAGAAACCTTTGAACGAAAGGGATAATGAAAGCCTTGATTGCAAGGCTTTTTGCTTTAT

GGTGGGTAAGTATCAGAGTGAGAAAATTTTTGGAATGAGTAGAAGTGATAGCTAGAAATTATCAGTTTCTA

TTTCCATTTACCCTGTGGGTACGTGTTTGTTTCCATTGACAAGGAGTTTGTGGGAATAGAAATGT

ACCCACCTTGTTTGAATCAAGTGAAGTGTAGTTGAAGGAAATCTGTTGAAAGCAATACTTCATTTTACCG

AATAAGTAATAATTTAGGCAACTTCAAATCGATTAAAAAAAACTATTTTAAAGGTTAAGAGTAGACAAAA

ATTGTCCACTCTTTTTTGCAAACTCAATTTATCAATAAATGAAATGAGGGAATGTAAAATG

AAATATTTTGAGGTTGAGTTAGAAAATCCTGATGAATTTTTAAAACTACAAACAGAAGATTTTGTGAAAG

CTAATCGCTTGCTACTAAGGAAGATAATCCAGAGCGTTACAGTCTATGAAGAAAACTTCGTCATATCCTT

TAAATCTGGCATCGAATTGGAAGTATGAGTCTCATTCCATAACTTTTATATTGAACATATCATCTTTTTGTG

TTATACTATAAATTGATATAAACAAAGATGTAGGAGGAACCGAAACTATGACAGCCTCAATGCGTTTAAGAT

AAGCTGGCAATAAAAAAAGCAGAATCTATACCCGATGATAGGCTTTTTTGTTGTGCTTATTTATACGATATT

GAGCATTCATTAGTTACGGTGAGGATATTGGTTATTTAACTATACCTTTATTTAACTATGTCTTTAATATGA

ATGTTTCCAAATTGTATGTATGCAGACCAAAAGCCACATTGTGGGGTTTGGCCTGCATTTTTTTTGCCTA

GAATGCTATTCAAAATAGAAATTCAAGCAAAATAATATGCAGGAGATAATATAAATGGAAAAATACAACAAT

TGGAAACGAAAATTTTATGCAATATGGGCAGGGCAAGCAGTATCATTAATCACTAGTGCCATCCTGCAAATG

GCGATTATTTTTTACCTTACAGAAAAAACAGGATCTGCGATGGTCTTGTCTATGGCTTCATTAGTAGGTTTT

TTACCCTATGCGATTTTGGGACCTGCCATTGGTGTGCTAGTGGATCGTCATGATAGGAAGAAGATAATGATT

GGTGCCGATTTAATTATCGCAGCAGCTGGTGCAGTGCTTGCTATTGTTGCATTCTGTATGGAGCTACCTGTC

TGGATGATTATGATAGTATTGTTTATCCGTAGCATTGGAACAGCTTTTCATACCCCAGCACTCAATGCGGTT

ACACCACTTTTAGTACCAGAAGAACAGCTAACGAAATGCGCAGGCTATAGTCAGTCTTTGCAGTCTATAAGC

TATATTGTTAGTCCGGCAGTTGCAGCACTCTTATACTCCGTTTGGGATTTAAATGCTATTATTGCCATCGAC

GTATTGGGTGCTGTGATTGCATCTATTACGGTAGCAATTGTACGTATACCTAAGCTGGGTAATCAAGTGCAA

AGTTTAGAACCAAATTTCATAAGGGAGATGAAAGAAGGAGTTGTGGTTCTGAGACAAAACAAAGGATTGTTT

GCCTTATTACTCTTAGGAACACTATATACTTTTGTTTATATGCCAATCAATGCACTATTTCCTTTAATAAGC

ATGGAACACTTTAATGGAACGCCTGTGCATATTTCTATTACGGAAATTTCCTTTGCATTTGGGATGCTAGCA

GGAGGCTTATTATTAGGAAGATTAGGGGGCTTCGAAAAGCATGTATTACTAATAACAAGTTCATTTTTTATA

ATGGGGACCAGTTTAGCCGTTTCGGGAATACTTCCTCCAAATGGATTTGTAATATTCGTAGTTTGCTGTGCA

ATAATGGGGCTTTCGGTGCCATTTTATAGCGGTGTGCAAACAGCTCTTTTTCAGGAGAAAATTAAGCCTGAA

TATTTAGGACGTGTATTTTCTTTGATCGGAAGTATCATGTCACTTGCTATGCCAATTGGGTTAATTCTTTCT

GGATTCTTTGCTGATAAAATCGGTGTAAATCATTGGTTTTTACTATCAGGTATTTTAATTATTGGCATTGCT

ATAGTTTGCCAAATGATAACTGAGGTTAGAAAATTAGATTTAAAATAAACAATATTGGAGGAATATTTATGT

ATCTTATTTTCATGTAACTCTTCCTGCTAAAATCGCAGGGTTTTCCCTGCATACAAGCAAATGAAAGCATGC

GATTATAGACAGGAGGAAATGTTATGGAATTAATATTAAAAGCAAAAGACATTCGTGTGGAATTCAAAGGAC

GCGATGTTTTAGATATAAATGAATTAGAAGTATATGATTATAGACAGGAGGAAATGTTATGGAATTAATATT

AAAAGCAAAAGACATTCGTGTGGAATTCAAAGGACGCGATGTTTTAGATATAAATGAATTAGAAGTATATGA

TTATGACCGTATTGGTTTAGTAGGAGCAAATGGTGCTGGAAAAAGCACTTTACTCAGGGTACTTTTAGGAGA

ATTAACTCCCCCAGGATGTAAAATGAATCGTCTGGGTGAACTTGCCTATATTCCCCAGTTGGACGAAGTAAC

TCTGCAGGAGGAAAAAGATTTTGCACTTGTAGGCAAGCTAGGTGTTGAGCAATTAAATATACAGACTATGAG

CGGTGGTGAAGAAACAAGGCTTAAAATAGCACAGGCCTTATCGGCACAGGTTCATGGTATTTTAGCGGATGA

ACCTACGAGCCATTTAGACCGTGAAGGAATTGATTTTCTAATAGGACAGCTAAAATATTTTACAGGTGCACT

GTTAGTTATTAGCCATGACCGCTATTTTCTTGATGAAATAGTAGATAAAATATGGGAACTGAAAGATGGCAA

AATCACTGAGTATTGGGGAAACTATTCTGATTATCTTCGTCAGAAAGAGGAAGAACGTAAGAGCCAAGCTGC

AGAATACGAACAATTTATTGCGGAACGTGCCCGATTGGAAAGGGCTGCGGAGGAAAAGCGAAAACAGGCTCG

TAAAATAGAACAGAAGGCAAAAGGTTCTTCAAAGAAAAAAAGTACTGAAGACGGAGGGCGTTTAGCTCATCA

AAAATCAATAGGAAGTAAGGAAAAAAAGATGTATAATGCTGCTAAAACCCTAGAGCACAGGATTGCGGCCTT

AGGAAAAGTAGAAGCTCCGGAAGGCATTCGCAGAATTCGTTTCAGGCAAAGTAAAGCATTGGAGCTCCATAA

TCCATACCCTATAGTCGGTGCAGAAATTAATAAAGTATTTGGGGATAAGGCTCTGTTTGAAAATGCATCTTT

TCAAATTCCGTTAGGAGCAAAAGTGGCGTTAACTGGTGGTAATGGAATCGGAAAAACAACTTTAATCCAAAT

GATCTTAAACCATGAAGAAGGAATTTCTATTTCGCCTAAGGCAAAAATAGGTTACTTTGCACAGAATGGTTA

CAAGTACAACAGTAATCAGAATGTTATGGAGTTTATGCAGAAGGATTGTGACTACAATATATCAGAAATTCG

TTCAGTGCTAGCATCTATGGGGTTCAAACAGAACGATATTGGAAAAAGTTTATCTGTTTTAAGCGGTGGAGA

AATTATAAAATTGTTGCTTGCTAAAATGCTCATGGGTAGATATAACATCCTAATAATGGATGAACCCAGTAA

CTTCCTTGACATACCAAGTTTAGAGGCTTTGGAAATACTAATGAAGGAGTACACCGGAACTATCGTGTTTAT

CACCCACGATAAACGATTACTCGAAAATGTAGCAGATGTAGTTTATGAAATTAGAGATAAGAAAATAAATCT

GAAACATTAAATTTAAGGTAGTCGCTGGTCAGTATAGTCTGTTCTGGTTGGCGACTCCATTGTTAAAGAGTA

TAAAGACTTTAGATTTTATGAATATTAAAAATAGGAACAGTCAATTGAACTGCTCCTATTTTTCTGCTAAAT

ATATTGTAGTTTTCTTATATGTATAATGATAGATTAGCGGATTCTCATCTACGGTACTTACTTCAAATATGA

AGAAGTGATCGCGGTTATCTCTGGACTTTTCCTTATTGAGGACAAAGTAATTCTTACGTGAAGTCGCCATTG

TTTTTAGGATATCATCAGTTAGGAAGGTCAATGGAATATTCATGTTAGAGTAGCGGTAGAAGTCACGTTCAA

AATCTTGGTAGCTCTCGCTATAATAGTCCATTTGTAGATGATTACGCTGAAACTCAAGCTGATTCATAGAGC

ACCTCCTCGACAAGTTCAATACTAATAATGTCTTTTAATTTCAAATTGATGTGACCTGTTGTAGTTTTTATC

AAAATGAAATCTTTGGTCAGACTTGGTATTGTTCCAGTGTAGGAAACACGCTTGTTTTTTTCAATCACTTGA

ATGCGTGTGCGTAGCTGCCCGGCGTATACTTGACTGAGGAGTAATAATTTCTTCTCTAGTGATAAGTCAGAC

ATGTACGTTACTTTGTTTGTATCATCAGAGAGTGCTGATGCATGTTCAGATAGGAAAAAGCCCATCCATTTT

TGCATCTTTGTATCCTGGTACTCTCTTGCTGATTGAAATGGTAAATATGAACGGTCAATCATATCAAATCCT

TTCTATGCAGAGGCAAGGGTATTTTTATCAAATTGAATCGTAAAACCTTGAATTCCCCCACCTGTGTAACAT

TCTTTAAAGCGATTGATTACCTCAGTATAGATTATCACAGATGAGCTTGTTGGCTTAATGCTAAATGTAAAT

TCCAATGGTAATCGGTTTTCAGATTTAGCATGTACTAGTCGTATCGATATTTCAGTTGTTTTGAGTTTTCTC

TGACGAAGTTTTGAAGTTGCTGTTTCAACGATTCCATGTAAAAATCTTTCAAGCATTTCAATATCATTACAT

CCTTTGCTACGGATTTCTGAAAATTGTACTGTATTTTTTTCTTGTTTCATTTTAATCCCTCCAATCCACCCG

CGGAATGACCACCGATAAGTTTACTGCGTTCAATATTTCTGGAACCTTCAGTTAGGACGGTTCCTTTTTGTA

TGGCTAAAAAACCAAACTGTTCTCTGACAACATCAATAGCTGTCTGAAGTCTATTATCTTTTTCAATTTGTT

CTACATCATCAAAGAGTGATAGTAGAGTATAGCTTTCATCTACGAAGCCACTATAAGATACACCAATTTGTC

TCACTGCACCAGAGGTGTATTTTTTCGGAATAATACAAGTACATGACTCACCATTGTTTTGGGGAGATTTG

CGGGTTCAATTTTATTCTGAGCATTTATAGATTTTTTCATCTCAGTCCTAGAATAGCCAATATGAATAGAAA

CGACAGTAGTCAATACTAGGGCTACTGTTCCGTTCCACAGTATCATTTAAAAATCATT-TTCACACCCTTTC

GTCTATTAGTATAGAAGAAAGCTCTCAGCACA

>GA54354/Mega-2.II

CATGTTGAGGCGGTAAGTTTGCTAGTCAAGGAGTAAAACGACGAAGATTAGCATTTACTTCCGCCCATGCGA

TAGCTGTCCGTGATTGACAAGTGCTAGCACGCAGACAGAACGGAGATAGCGAACCGCTGAGTGTGTCGCTCT

GCTCGTAAAAGCTTAGAAACCTTTGAACGAAAGGGATAATGAAAGCCTTGATTGCAAGGCTTTTTGCTTTAT

GGTGGGTAAGTATCAGAGTGAGAAAATTTTTGGAATGAGTAGAAGTGATAGCTAGAAATTATCAGTTTCTA

TTTCCATTTACCCTGTGGGTACGTGTTTGTTTCCATTGACAAGGAGTTTGTGGGAATAGAAATGT

ACCCACCTTGTTTGAATCAAGTGAAGTGTAGTTGAAGGAAATCTGTTGAAAGCAATACTTCATTTTACCG

AATAAGTAATAATTTAGGCAACTTCAAATCGATTAAAAAAAACTATTTTAAAGGTTAAGAGTAGACAAAA

ATTGTCCACTCTTTTTTGCAAACTCAATTTATCAATAAATGAAATGAGGGAATGTAAAATG

AAATATTTTGAGGTTGAGTTAGAAAATCCTGATGAATTTTTAAAACTACAAACAGAAGATTTTGTGAAAG

CTAATCGCTTGCTACTAAGGAAGATAATCCAGAGCGTTACAGTCTATGAAGAAAACTTCGTCATATCCTT

TAAATCTGGCATCGAATTGGAAGTATGAGTCTCATTCCATAACTTTTATATTGAACATATCATCTTTTTGTG

TTATACTATAAATTGATATAAACAAAGATGTAGGAGGAACCGAAACTATGACAGCCTCAATGCGTTTAAGAT

AAGCTGGCAATAAAAAAAGCAGAATCTATACCCGATGATAGGCTTTTTTGTTGTGCTTATTTATACGATATT

GAGCATTCATTAGTTACGGTGAGGATATTGGTTATTTAACTATACCTTTATTTAACTATGTCTTTAATATGA

ATGTTTCCAAATTGTATGTATGCAGACCAAAAGCCACATTGTGGGGTTTGGCCTGCATTTTTTTTGCCTA

GAATGCTATTCAAAATAGAAATTCAAGCAAAATAATATGCAGGAGATAATATAAATGGAAAAATACAACAAT

TGGAAACGAAAATTTTATGCAATATGGGCAGGGCAAGCAGTATCATTAATCACTAGTGCCATCCTGCAAATG

GCGATTATTTTTTACCTTACAGAAAAAACAGGATCTGCGATGGTCTTGTCTATGGCTTCATTAGTAGGTTTT

TTACCCTATGCGATTTTGGGACCTGCCATTGGTGTGCTAGTGGATCGTCATGATAGGAAGAAGATAATGATT

GGTGCCGATTTAATTATCGCAGCAGCTGGTGCAGTGCTTGCTATTGTTGCATTCTGTATGGAGCTACCTGTC

TGGATGATTATGATAGTATTGTTTATCCGTAGCATTGGAACAGCTTTTCATACCCCAGCACTCAATGCGGTT

ACACCACTTTTAGTACCAGAAGAACAGCTAACGAAATGCGCAGGCTATAGTCAGTCTTTGCAGTCTATAAGC

TATATTGTTAGTCCGGCAGTTGCAGCACTCTTATACTCCGTTTGGGATTTAAATGCTATTATTGCCATCGAC

GTATTGGGTGCTGTGATTGCATCTATTACGGTAGCAATTGTACGTATACCTAAGCTGGGTAATCAAGTGCAA

AGTTTAGAACCAAATTTCATAAGGGAGATGAAAGAAGGAGTTGTGGTTCTGAGACAAAACAAAGGATTGTTT

GCCTTATTACTCTTAGGAACACTATATACTTTTGTTTATATGCCAATCAATGCACTATTTCCTTTAATAAGC

ATGGAACACTTTAATGGAACGCCTGTGCATATTTCTATTACGGAAATTTCCTTTGCATTTGGGATGCTAGCA

GGAGGCTTATTATTAGGAAGATTAGGGGGCTTCGAAAAGCATGTATTACTAATAACAAGTTCATTTTTTATA

ATGGGGACCAGTTTAGCCGTTTCGGGAATACTTCCTCCAAATGGATTTGTAATATTCGTAGTTTGCTGTGCA

ATAATGGGGCTTTCGGTGCCATTTTATAGCGGTGTGCAAACAGCTCTTTTTCAGGAGAAAATTAAGCCTGAA

TATTTAGGACGTGTATTTTCTTTGATCGGAAGTATCATGTCACTTGCTATGCCAATTGGGTTAATTCTTTCT

GGATTCTTTGCTGATAAAATCGGTGTAAATCATTGGTTTTTACTATCAGGTATTTTAATTATTGGCATTGCT

ATAGTTTGCCAAATGATAACTGAGGTTAGAAAATTAGATTTAAAATAAACAATATTGGAGGAA

ATGTTATGGAATTAATATT

AAAAGCAAAAGACATTCGTGTGGAATTCAAAGGACGCGATGTTTTAGATATAAATGAATTAGAAGTATATGA

TTATGACCGTATTGGTTTAGTAGGAGCAAATGGTGCTGGAAAAAGCACTTTACTCAGGGTACTTTTAGGAGA

ATTAACTCCCCCAGGATGTAAAATGAATCGTCTGGGTGAACTTGCCTATATTCCCCAGTTGGACGAAGTAAC

TCTGCAGGAGGAAAAAGATTTTGCACTTGTAGGCAAGCTAGGTGTTGAGCAATTAAATATACAGACTATGAG

CGGTGGTGAAGAAACAAGGCTTAAAATAGCACAGGCCTTATCGGCACAGGTTCATGGTATTTTAGCGGATGA

ACCTACGAGCCATTTAGACCGTGAAGGAATTGATTTTCTAATAGGACAGCTAAAATATTTTACAGGTGCACT

GTTAGTTATTAGCCATGACCGCTATTTTCTTGATGAAATAGTAGATAAAATATGGGAACTGAAAGATGGCAA

AATCACTGAGTATTGGGGAAACTATTCTGATTATCTTCGTCAGAAAGAGGAAGAACGTAAGAGCCAAGCTGC

AGAATACGAACAATTTATTGCGGAACGTGCCCGATTGGAAAGGGCTGCGGAGAAAAAGCGAAAACAGGCTCG

TAAAATAGAACAGAAGGCAAAAGGTTCTTCAAAGAAAAAAAGTACTGAAGACGGAGGGCGTTTAGCTCATCA

AAAATCAATAGGAAGTAAGGAAAAAAAGATGTATAATGCTGCTAAAACCCTAGAGCACAGGATTGCGGCCTT

AGGAAAAGTAGAAGCTCCGGAAGGCATTCGCAGAATTCGTTTCAGGCAAAGTAAAGCATTGGAGCTCCATAA

TCCATACCCTATAGTCGGTGCAGAAATTAATAAAGTATTTGGGGATAAGGCTCTGTTTGAAAATGCATCTTT

TCAAATTCCGTTAGGAGCAAAAGTGGCGTTAACTGGTGGTAATGGAATCGGAAAAACAACTTTAATCCAAAT

GATCTTAAACCATGAAGAAGGAATTTCTATTTCGCCTAAGGCAAAAATAGGTTACTTTGCACAGAATGGTTA

TAAGTACAACAGTAATCAGAATGTTATGGAGTTTATGCAGAAGGATTGTGACTACAATATATCAGAAATTCG

TTCAGTGCTAGCATCTATGGGGTTCAAACAGAACGATATTGGAAAAAGTTTATCTGTTTTAAGCGGTGGAGA

AATTATAAAATTGTTGCTTGCTAAAATGCTCATGGGTAGATATAACATCCTAATAATGGATGAACCCAGTAA

CTTCCTTGACATACCAAGTTTAGAGGCTTTGGAAATACTAATGAAGGAGTACACCGGAACTATCGTGTTTAT

CACCCACGATAAACGATTACTCGAAAATGTAGCAGATGTAGTTTATGAAATTAGAGATAAGAAAATAAATCT

GAAACATTAAATTTAAGGTAGTCGCTGGTCAGTATAGTCTGTTCTGGTTGGCGACTCCATTGTTAAAGAGTA

TAAAGACTTTAGATTTTATGAATATTAAAAATAGGAACAGTCAATTGAACTGCTCCTATTTTTCTGCTAAAT

ATATTGTAGTTTTCTTATATGTATAATGATAGATTAGCGGATTCTCATCTACGGTACTTACTTCAAATATGA

AGAAGTGATCGCGGTTATCTCTGGACTTTTCCTTATTGAGGACAAAGTAATTCTTACGTGAAGTCGCCATTG

TTTTTAGGATATCATCAGTTAGGAAGGTCAATGGAATATTCATGTTAGAGTAGCGGTAGAAGTCACGTTCAA

AATCTTGGTAGCTCTCGCTATAATAGTCCATTTGTAGGTGATTACGCTGAAACTCAAGCTGATTCATAGAGC

ACCTCCTCGACAAGTTCAATACTAATAATGTCTTTTAATTTCAAATTGATGTGACCTGTTGTAGTTTTTATC

AAAATGAAATCTTTGGTCAGACTTGGTATTGTTCCAGTGTAGGAAACACGCTTGTTTTTTTCAATCACTTGA

ATGCGTGTGCGTAGCTGCCCGGCGTATACTTGACTGAGGAGTAATAATTTCTTCTCTAGTGATAAGTCAGAC

ATGTACGTTACTTTGTTTGTATCATCAGAGAGTGCTGATGCATGTTCAGATAGGAAAAAGCCCATCCATTTT

TGCATCTTTGTATCCTGGTACTCTCTTGCTGATTGAAATGGTAAATATGAACGGTCAATCATATCAAATCCT

TTCTATGCAGAGGCAAGGGTATTTTTATCAAATTGAATCGTAAAACCTTGAATTCCCCCACCTGTGTAACAT

TCTTTAAAGCGATTGATTACCTCAGTATAGATTATCACAGATGAGCTTGTTGGCTTAATGCTAAATGTAAAT

TCCAATGGTAATCGGTTTTCAGATTTAGCATGTACTAGTCGTATCGATATTTCAGTTGTTTTGAGTTTTCTC

TGACGAAGTTTTGAAGTTGCTGTTTCAACGATTCCATGTAAAAATCTTTCAAGCATTTCAATATCATTACAT

CCTTTGCTACGGATTTCTGAAAATTGTACTGTATTTTTTTCTTGTTTCATTTTAATCCCTCCAATCCACCCG

CGGAATGACCACCGATAAGTTTACTGCGTTCAATATTTCTGGAACCTTCAGTTAGGACGGTTCCTTTTTGTA

TGGCTAAAAAACCAAACTGTTCTCTGACAACATCAATAGCTGTCTGAAGTCTATTATCTTTTTCAATTTGTT

CTACATCATCAAAGAGTGATAGTAGAGTATAGCTTTCATCTACGAAGCCACTATAAGATACACCAATTTGTC

TCACTGCACCAGAGGTGTATTTTTTCGGAATAATACAAGTACATGACTCACCATTGTTTTGGGGAGATTTG

CGGGTTCAATTTTATTCTGAGCATTTATAGATTTTTTCATCTCAGTCCTAGAATAGCCAATATGAATAGAAA

CGACAGTAGTCAATACTAGGGCTACTGTTCCGTTCCACAGTATCATTTAAAAATCATT-TTCACACCCTTTC

GTCTATTAGTATAGAAGAAAGCTCTCAGCACA

>GA62331/Mega-2.II

CATGTTGAGGCGGTAAGTTTGCTAGTCAAGGAGTAAAACGACGAAGATTAGCATTTACTTCCGCCCATGCGA

TAGCTGTCCGTGATTGACAAGTGCTAGCACGCAGACAGAACGGAGATAGCGAACCGCTGAGTGTGTCGCTCT

GCTCGTAAAAGCTTAGAAACCTTTGAACGAAAGGGATAATGAAAGCCTTGATTGCAAGGCTTTTTGCTTTAT

GGTGGGTAAGTATCAGAGTGAGAAAATTTTTGGAATGAGTAGAAGTGATAGCTAGAAATTATCAGTTTCTA

TTTCCATTTACCCTGTGGGTACGTGTTTGTTTCCATTGACAAGGAGTTTGTGGGAATAGAAATGT

ACCCACCTTGTTTGAATCAAGTGAAGTGTAGTTGAAGGAAATCTGTTGAAAGCAATACTTCATTTTACCG

AATAAGTAATAATTTAGGCAACTTCAAATCGATTAAAAAAAACTATTTTAAAGGTTAAGAGTAGACAAAA

ATTGTCCACTCTTTTTTGCAAACTCAATTTATCAATAAATGAAATGAGGGAATGTAAAATG

AAATATTTTGAGGTTGAGTTAGAAAATCCTGATGAATTTTTAAAACTACAAACAGAAGATTTTGTGAAAG

CTAATCGCTTGCTACTAAGGAAGATAATCCAGAGCGTTACAGTCTATGAAGAAAACTTCGTCATATCCTT

TAAATCTGGCATCGAATTGGAAGTATGAGTCTCATTCCATAACTTTTATATTGAACATATCATCTTTTTGTG

TTATACTATAAATTGATATAAACAAAGATGTAGGAGGAACCGAAACTATGACAGCCTCAATGCGTTTAAGAT

AAGCTGGCAATAAAAAAAGCAGAATCTATACCCGATGATAGGCTTTTTTGTTGTGCTTATTTATACGATATT

GAGCATTCATTAGTTACGGTGAGGATATTGGTTATTTAACTATACCTTTATTTAACTATGTCTTTAATATGA

ATGTTTCCAAATTGTATGTATGCAGACCAAAAGCCACATTGTGGGGTTTGGCCTGCATTTTTTTTGCCTA

GAATGCTATTCAAAATAGAAATTCAAGCAAAATAATATGCAGGAGATAATATAAATGGAAAAATACAACAAT

TGGAAACGAAAATTTTATGCAATATGGGCAGGGCAAGCAGTATCATTAATCACTAGTGCCATCCTGCAAATG

GCGATTATTTTTTACCTTACAGAAAAAACAGGATCTGCGATGGTCTTGTCTATGGCTTCATTAGTAGGTTTT

TTACCCTATGCGATTTTGGGACCTGCCATTGGTGTGCTAGTGGATCGTCATGATAGGAAGAAGATAATGATT

GGTGCCGATTTAATTATCGCAGCAGCTGGTGCAGTGCTTGCTATTGTTGCATTCTGTATGGAGCTACCTGTC

TGGATGATTATGATAGTATTGTTTATCCGTAGCATTGGAACAGCTTTTCATACCCCAGCACTCAATGCGGTT

ACACCACTTTTAGTACCAGAAGAACAGCTAACGAAATGCGCAGGCTATAGTCAGTCTTTGCAGTCTATAAGC

TATATTGTTAGTCCGGCAGTTGCAGCACTCTTATACTCCGTTTGGGATTTAAATGCTATTATTGCCATCGAC

GTATTGGGTGCTGTGATTGCATCTATTACGGTAGCAATTGTACGTATACCTAAGCTGGGTAATCAAGTGCAA

AGTTTAGAACCAAATTTCATAAGGGAGATGAAAGAAGGAGTTGTGGTTCTGAGACAAAACAAAGGATTGTTT

GCCTTATTACTCTTAGGAACACTATATACTTTTGTTTATATGCCAATCAATGCACTATTTCCTTTAATAAGC

ATGGAACACTTTAATGGAACGCCTGTGCATATTTCTATTACGGAAATTTCCTTTGCATTTGGGATGCTAGCA

GGAGGCTTATTATTAGGAAGATTAGGGGGCTTCGAAAAGCATGTATTACTAATAACAAGTTCATTTTTTATA

ATGGGGACCAGTTTAGCCGTTTCGGGAATACTTCCTCCAAATGGATTTGTAATATTCGTAGTTTGCTGTGCA

ATAATGGGGCTTTCGGTGCCATTTTATAGCGGTGTGCAAACAGCTCTTTTTCAGGAGAAAATTAAGCCTGAA

TATTTAGGACGTGTATTTTCTTTGATCGGAAGTATCATGTCACTTGCTATGCCAATTGGGTTAATTCTTTCT

GGATTCTTTGCTGATAAAATCGGTGTAAATCATTGGTTTTTACTATCAGGTATTTTAATTATTGGCATTGCT

ATAGTTTGCCAAATGATAACTGAGGTTAGAAAATTAGATTTAAAATAAACAATATTGGAGGAA

ATGTTATGGAATTAATATT

AAAAGCAAAAGACATTCGTGTGGAATTCAAAGGACGCGATGTTTTAGATATAAATGAATTAGAAGTATATGA

TTATGACCGTATTGGTTTAGTAGGAGCAAATGGTGCTGGAAAAAGCACTTTACTCAGGGTACTTTTAGGAGA

ATTAACTCCCCCAGGATGTAAAATGAATCGTCTGGGTGAACTTGCCTATATTCCCCAGTTGGACGAAGTAAC

TCTGCAGGAGGAAAAAGATTTTGCACTTGTAGGCAAGCTAGGTGTTGAGCAATTAAATATACAGACTATGAG

CGGTGGTGAAGAAACAAGGCTTAAAATAGCACAGGCCTTATCGGCACAGGTTCATGGTATTTTAGCGGATGA

ACCTACGAGCCATTTAGACCGTGAAGGAATTGATTTTCTAATAGGACAGCTAAAATATTTTACAGGTGCACT

GTTAGTTATTAGCCATGACCGCTATTTTCTTGATGAAATAGTAGATAAAATATGGGAACTGAAAGATGGCAA

AATCACTGAGTATTGGGGAAACTATTCTGATTATCTTCGTCAGAAAGAGGAAGAACGTAAGAGCCAAGCTGC

AGAATACGAACAATTTATTGCGGAACGTGCCCGATTGGAAAGGGCTGCGGAGAAAAAGCGAAAACAGGCTCG

TAAAATAGAACAGAAGGCAAAAGGTTCTTCAAAGAAAAAAAGTACTGAAGACGGAGGGCGTTTAGCTCATCA

AAAATCAATAGGAAGTAAGGAAAAAAAGATGTATAATGCTGCTAAAACCCTAGAGCACAGGATTGCGGCCTT

AGGAAAAGTAGAAGCTCCGGAAGGCATTCGCAGAATTCGTTTCAGGCAAAGTAAAGCATTGGAGCTCCATAA

TCCATACCCTATAGTCGGTGCAGAAATTAATAAAGTATTTGGGGATAAGGCTCTGTTTGAAAATGCATCTTT

TCAAATTCCGTTAGGAGCAAAAGTGGCGTTAACTGGTGGTAATGGAATCGGAAAAACAACTTTAATCCAAAT

GATCTTAAACCATGAAGAAGGAATTTCTATTTCGCCTAAGGCAAAAATAGGTTACTTTGCACAGAATGGTTA

TAAGTACAACAGTAATCAGAATGTTATGGAGTTTATGCAGAAGGATTGTGACTACAATATATCAGAAATTCG

TTCAGTGCTAGCATCTATGGGGTTCAAACAGAACGATATTGGAAAAAGTTTATCTGTTTTAAGCGGTGGAGA

AATTATAAAATTGTTGCTTGCTAAAATGCTCATGGGTAGATATAACATCCTAATAATGGATGAACCCAGTAA

CTTCCTTGACATACCAAGTTTAGAGGCTTTGGAAATACTAATGAAGGAGTACACCGGAACTATCGTGTTTAT

CACCCACGATAAACGATTACTCGAAAATGTAGCAGATGTAGTTTATGAAATTAGAGATAAGAAAATAAATCT

GAAACATTAAATTTAAGGTAGTCGCTGGTCAGTATAGTCTGTTCTGGTTGGCGACTCCATTGTTAAAGAGTA

TAAAGACTTTAGATTTTATGAATATTAAAAATAGGAACAGTCAATTGAACTGCTCCTATTTTTCTGCTAAAT

ATATTGTAGTTTTCTTATATGTATAATGATAGATTAGCGGATTCTCATCTACGGTACTTACTTCAAATATGA

AGAAGTGATCGCGGTTATCTCTGGACTTTTCCTTATTGAGGACAAAGTAATTCTTACGTGAAGTCGCCATTG

TTTTTAGGATATCATCAGTTAGGAAGGTCAATGGAATATTCATGTTAGAGTAGCGGTAGAAGTCACGTTCAA

AATCTTGGTAGCTCTCGCTATAATAGTCCATTTGTAGGTGATTACGCTGAAACTCAAGCTGATTCATAGAGC

ACCTCCTCGACAAGTTCAATACTAATAATGTCTTTTAATTTCAAATTGATGTGACCTGTTGTAGTTTTTATC

AAAATGAAATCTTTGGTCAGACTTGGTATTGTTCCAGTGTAGGAAACACGCTTGTTTTTTTCAATCACTTGA

ATGCGTGTGCGTAGCTGCCCGGCGTATACTTGACTGAGGAGTAATAATTTCTTCTCTAGTGATAAGTCAGAC

ATGTACGTTACTTTGTTTGTATCATCAGAGAGTGCTGATGCATGTTCAGATAGGAAAAAGCCCATCCATTTT

TGCATCTTTGTATCCTGGTACTCTCTTGCTGATTGAAATGGTAAATATGAACGGTCAATCATATCAAATCCT

TTCTATGCAGAGGCAAGGGTATTTTTATCAAATTGAATCGTAAAACCTTGAATTCCCCCACCTGTGTAACAT

TCTTTAAAGCGATTGATTACCTCAGTATAGATTATCACAGATGAGCTTGTTGGCTTAATGCTAAATGTAAAT

TCCAATGGTAATCGGTTTTCAGATTTAGCATGTACTAGTCGTATCGATATTTCAGTTGTTTTGAGTTTTCTC

TGACGAAGTTTTGAAGTTGCTGTTTCAACGATTCCATGTAAAAATCTTTCAAGCATTTCAATATCATTACAT

CCTTTGCTACGGATTTCTGAAAATTGTACTGTATTTTTTTCTTGTTTCATTTTAATCCCTCCAATCCACCCG

CGGAATGACCACCGATAAGTTTACTGCGTTCAATATTTCTGGAACCTTCAGTTAGGACGGTTCCTTTTTGTA

TGGCTAAAAAACCAAACTGTTCTCTGACAACATCAATAGCTGTCTGAAGTCTATTATCTTTTTCAATTTGTT

CTACATCATCAAAGAGTGATAGTAGAGTATAGCTTTCATCTACGAAGCCACTATAAGATACACCAATTTGTC

TCACTGCACCAGAGGTGTATTTTTTCGGAATAATACAAGTACATGACTCACCATTGTTTTGGGGAGATTTG

CGGGTTCAATTTTATTCTGAGCATTTATAGATTTTTTCATCTCAGTCCTAGAATAGCCAATATGAATAGAAA

CGACAGTAGTCAATACTAGGGCTACTGTTCCGTTCCACAGTATCATTTAAAAATCATT-TTCACACCCTTTC

GTCTATTAGTATAGAAGAAAGCTCTCAGCACA

>GA16242/Mega-2.IVa

CATGTTGAGGCGGTAAGTTTGCTAGTCAAGGAGTAAAACGACGAAGATTAGCATTTACTTCCGCCCATGCGA

TAGCTGTCCGTGATTGACAAGTGCTAGCACGCAGACAGAACGGAGATAGCGAACCGCTGAGTGTGTCGCTCT

GCTCGTAAAAGCTTAGAAACCTTTGAACGAAAGGGATAATGAAAGCCTTGATTGCAAGGCTTTTTGCTTTAT

GGTGGGTAAGTATCAGAGTGAGAAAATTTTTGGAATGAGTAGAAGTGATAGCTAGAAATTATCAGTTTCTA

TTTCCATTTACCCTGTGGGTACGTGTTTGTTTCCATTGACAAGGAGTTTGTGGGAATAGAAATGT

ACCCACCTTGTTTGAATCAAGTGAAGTGTAGTTGAAGGAAATCTGTTGAAAGCAATACTTCATTTTACCG

AATAAGTAATAATTTAGGCAACTTCAAATCGATTAAAAAAAACTATTTTAAAGGTTAAGAGTAGACAAAA

ATTGTCCACTCTTTTTTGCAAACTCAATTTATCAATAAATGAAATGAGGGAATGTAAAATG

AAATATTTTGAGGTTGAGTTAGAAAATCCTGATGAATTTTTAAAACTACAAACAGAAGATTTTGTGAAAG

CTAATCGCTTGCTACTAAGGAAGATAATCCAGAGCGTTACAGTCTATGAAGAAAACTTCGTCATATCCTT

TAAATCTGGCATCGAATTGGAAGTATGAGTCTCATTCCATAACTTTTATATTGAACATATCATCTTTTTGTG

TTATACTATAAATTGATATAAACAAAGATGTAGGAGGAACCGAAACTATGACAGCCTCAATGCGTTTAAGAT

AAGCTGGCAATAAAAAAAGCAGAATCTATACCCGATGATAGGCTTTTTTGTTGTGCTTATTTATACGATATT

GAGCATTCATTAGTTACGGTGAGGATATTGGTTATTTAACTATACCTTTATTTAACTATGTCTTTAATATGA

ATGTTTCCAAATTGTATGTATGCAGACCAAAAGCCACATTGTGGGGTTTGGCCTGCATTTTTTTTGCCTA

GAATGCTATTCAAAATAGAAATTCAAGCAAAATAATATGCAGGAGATAATATAAATGGAAAAATACAACAAT

TGGAAACGAAAATTTTATGCAATATGGGCAGGGCAAGCAGTATCATTAATCACTAGTGCCATCCTGCAAATG

GCGATTATTTTTTACCTTACAGAAAAAACAGGATCTGCGATGGTCTTGTCTATGGCTTCATTAGTAGGTTTT

TTACCCTATGCGATTTTGGGACCTGCCATTGGTGTGCTAGTGGATCGTCATGATAGGAAGAAGATAATGATT

GGTGCCGATTTAATTATCGCAGCAGCTGGTGCAGTGCTTGCTATTGTTGCATTCTGTATGGAGCTACCTGTC

TGGATGATTATGATAGTATTGTTTATCCGTAGCATTGGAACAGCTTTTCATACCCCAGCACTCAATGCGGTT

ACACCACTTTTAGTACCAGAAGAACAGCTAACGAAATGCGCAGGCTATAGTCAGTCTTTGCAGTCTATAAGC

TATATTGTTAGTCCGGCAGTTGCAGCACTCTTATACTCCGTTTGGGATTTAAATGCTATTATTGCCATCGAC

GTATTGGGTGCTGTGATTGCATCTATTACGGTAGCAATTGTACGTATACCTAAGCTGGGTAATCAAGTGCAA

AGTTTAGAACCAAATTTCATAAGGGAGATGAAAGAAGGAGTTGTGGTTCTGAGACAAAACAAAGGATTGTTT

GCCTTATTACTCTTAGGAACACTATATACTTTTGTTTATATGCCAATCAATGCACTATTTCCTTTAATAAGC

ATGGAACACTTTAATGGAACGCCTGTGCATATTTCTATTACGGAAATTTCCTTTGCATTTGGGATGCTAGCA

GGAGGCTTATTATTAGGAAGATTAGGGGGCTTCGAAAAGCATGTATTACTAATAACAAGTTCATTTTTTATA

ATGGGGACCAGTTTAGCCGTTTCGGGAATACTTCCTCCAAATGGATTTGTAATATTCGTAGTTTGCTGTGCA

ATAATGGGGCTTTCGGTGCCATTTTATAGCGGTGTGCAAACAGCTCTTTTTCAGGAGAAAATTAAGCCTGAA

TATTTAGGACGTGTATTTTCTTTGATCGGAAGTATCATGTCACTTGCTATGCCAATTGGGTTAATTCTTTCT

GGATTCTTTGCTGATAAAATCGGTGTAAATCATTGGTTTTTACTATCAGGTATTTTAATTATTGGCATTGCT

ATAGTTTGCCAAATGATAACTGAGGTTAGAAAATTAGATTTAAAATAAACAATATTGGAGGAA

ATGTTATGGAATTAATATT

AAAAGCAAAAGACATTCGTGTGGAATTCAAAGGACGCGATGTTTTAGATATAAATGAATTAGAAGTATATGA

TTATGACCGTATTGGTTTAGTAGGAGCAAATGGTGCTGGAAAAAGCACTTTACTCAGGGTACTTTTAGGAGA

ATTAACTCCCCCAGGATGTAAAATGAATCGTCTGGGTGAACTTGCCTATATTCCCCAGTTGGACGAAGTAAC

TCTGCAGGAGGAAAAAGATTTTGCACTTGTAGGCAAGCTAGGTGTTGAGCAATTAAATATACAGACTATGAG

CGGTGGTGAAGAAACAAGGCTTAAAATAGCACAGGCCTTATCGGCACAGGTTCATGGTATTTTAGCGGATGA

ACCTACGAGCCATTTAGACCGTGAAGGAATTGATTTTCTAATAGGACAGCTAAAATATTTTACAGGTGCACT

GTTAGTTATTAGCCATGACCGCTATTTTCTTGATGAAATAGTAGATAAAATATGGGAACTGAAAGATGGCAA

AATCACTGAGTATTGGGGAAACTATTCTGATTATCTTCGTCAGAAAGAGGAAGAACGTAAGAGCCAAGCTGC

AGAATACGAACAATTTATTGCGGAACGTGCCCGATTGGAAAGGGCTGCGGAGAAAAAGCGAAAACAGGCTCG

TAAAATAGAACAGAAGGCAAAAGGTTCTTCAAAGAAAAAAAGTACTGAAGACGGAGGGCGTTTAGCTCATCA

AAAATCAATAGGAAGTAAGGAAAAAAAGATGTATAATGCTGCTAAAACCCTAGAGCACAGGATTGCGGCCTT

AGGAAAAGTAGAAGCTCCGGAAGGCATTCGCAGAATTCGTTTCAGGCAAAGTAAAGCATTGGAGCTCCATAA

TCCATACCCTATAGTCGGTGCAGAAATTAATAAAGTATTTGGGGATAAGGCTCTGTTTGAAAATGCATCTTT

TCAAATTCCGTTAGGAGCAAAAGTGGCGTTAACTGGTGGTAATGGAATCGGAAAAACAACTTTAATCCAAAT

GATCTTAAACCATGAAGAAGGAATTTCTATTTCGCCTAAGGCAAAAATAGGTTACTTTGCACAGAATGGTTA

CAAGTACAACAGTAATCAGAATGTTATGGAGTTTATGCAGAAGGATTGTGACTACAATATATCAGAAATTCG

TTCAGTGCTAGCATCTATGGGGTTCAAACAGAACGATATTGGAAAAAGTTTATCTGTTTTAAGCGGTGGAGA

AATTATAAAATTGTTGCTTGCTAAAATGCTCATGGGTAGATATAACATCCTAATAATGGATGAACCCAGTAA

CTTCCTTGACATACCAAGTTTAGAGGCTTTGGAAATACTAATGAAGGAGTACACCGGAACTATCGTGTTTAT

CACCCACGATAAACGATTACTCGAAAATGTAGCAGATGTAGTTTATGAAATTAGAGATAAGAAAATAAATCT

GAAACATTAAATTTAAGGTAGTCGCTGGTCAGTATAGTCTGTTCTGGTTGGCGACTCCATTGTTAAAGAGTA

TAAAGACTTTAGATTTTATGAATATTAAAAATAGGAACAGTCAATTGAACTGCTCCTATTTTTCTGCTAAAT

ATATTGTAGTTTTCTTATATGTATAATGATAGATTAGCGGATTCTCATCTACGGTACTTACTTCAAATATGA

AGAAGTGATCGCGGTTATCTCTGGACTTTTCCTTATTGAGGACAAAGTAATTCTTACGTGAAGTCGCCATTG

TTTTTAGGATATCATCAGTTAGGAAGGTCAATGGAATATTCATGTTAGAGTAGCGGTAGAAGTCACGTTCAA

AATCTTGGTAGCTCTCGCTATAATAGTCCATTTGTAGGTGATTACGCTGAAACTCAAGCTGATTCATAGAGC

ACCTCCTCGACAAGTTCAATACTAATAATGTCTTTTAATTTCAAATTGATGTGACCTGTTGTAGTTTTTATC

AAAATGAAATCTTTGGTCAGACTTGGTATTGTTCCAGTGTAGGAAACACGCTTGTTTTTTTCAATCACTTGA

ATGCGTGTGCGTAGCTGCCCGGCGTATACTTGACTGAGGAGTAATAATTTCTTCTCTAGTGATAAGTCAGAC

ATGTACGTTACTTTGTTTGTATCATCAGAGAGTGCTGATGCATGTTCAGATAGGAAAAAGCCCATCCATTTT

TGCATCTTTGTATCCTGGTACTCTCTTGCTGATTGAAATGGTAAATATGAACGGTCAATCATATCAAATCCT

TTCTATGCAGAGGCAAGGGTATTTTTATCAAATTGAATCGTAAAACCTTGAATTCCCCCACCTGTGTAACAT

TCTTTAAAGCGATTGATTACCTCAGTATAGATTATCACAGATGAGCTTGTTGGCTTAATGCTAAATGTAAAT

TCCAATGGTAATCGGTTTTCAGATTTAGCATGTACTAGTCGTATCGATATTTCAGTTGTTTTGAGTTTTCTC

TGACGAAGTTTTGAAGTTGCTGTTTCAACGATTCCATGTAAAAATCTTTCAAGCATTTCAATATCATTACAT

CCTTTGCTACGGATTTCTGAAAATTGTACTGTATTTTTTTCTTGTTTCATTTTAATCCCTCCAATCCACCCG

CGGAATGACCACCGATAAGTTTACTGCGTTCAATATTTCTGGAACCTTCAGTTAGGACGGTTCCTTTTTGTA

TGGCTAAAAAACCAAACTGTTCTCTGACAACATCAATAGCTGTCTGAAGTCTATTATCTTTTTCAATTTGTT

CTACATCATCAAAGAGTGATAGTAGAGTATAGCTTTCATCTACGAAGCCACTATAAGATACACCAATTTGTC

TCACTGCACCAGAGGTGTATTTTTTCGGAATAATACAAGTACATGACTCACCATTGTTTTGGGGAGATTTG

CGGGTTCAATTTTATTCTGAGCATTTATAGATTTTTTCATCTCAGTCCTAGAATAGCCAATATGAATAGAAA

CGACAGTAGTCAATACTAGGGCTACTGTTCCGTTCCACAGTATCATTTAAAAATCATT-TTCACACCCTTTC

GTCTATTAGTATAGAAGAAAGCTCTCAGCACA

>GA4375/Mega-2.IVa

CATGTTGAGGCGGTAAGTTTGCTAGTCAAGGAGTAAAACGACGAAGATTAGCATTTACTTCCGCCCATGCGA

TAGCTGTCCGTGATTGACAAGTGCTAGCACGCAGACAGAACGGAGATAGCGAACCGCTGAGTGTGTCGCTCT

GCTCGTAAAAGCTTAGAAACCTTTGAACGAAAGGGATAATGAAAGCCTTGATTGCAAGGCTTTTTGCTTTAT

GGTGGGTAAGTATCAGAGTGAGAAAATTTTTGGAATGAGTAGAAGTGATAGCTAGAAATTATCAGTTTCTA

TTTCCATTTACCCTGTGGGTACGTGTTTGTTTCCATTGACAAGGAGTTTGTGGGAATAGAAATGT

ACCCACCTTGTTTGAATCAAGTGAAGTGTAGTTGAAGGAAATCTGTTGAAAGCAATACTTCATTTTACCG

AATAAGTAATAATTTAGGCAACTTCAAATCGATTAAAAAAAACTATTTTAAAGGTTAAGAGTAGACAAAA

ATTGTCCACTCTTTTTTGCAAACTCAATTTATCAATAAATGAAATGAGGGAATGTAAAATG

AAATATTTTGAGGTTGAGTTAGAAAATCCTGATGAATTTTTAAAACTACAAACAGAAGATTTTGTGAAAG

CTAATCGCTTGCTACTAAGGAAGATAATCCAGAGCGTTACAGTCTATGAAGAAAACTTCGTCATATCCTT

TAAATCTGGCATCGAATTGGAAGTATGAGTCTCATTCCATAACTTTTATATTGAACATATCATCTTTTTGTG

TTATACTATAAATTGATATAAACAAAGATGTAGGAGGAACCGAAACTATGACAGCCTCAATGCGTTTAAGAT

AAGCTGGCAATAAAAAAAGCAGAATCTATACCCGATGATAGGCTTTTTTGTTGTGCTTATTTATACGATATT

GAGCATTCATTAGTTACGGTGAGGATATTGGTTATTTAACTATACCTTTATTTAACTATGTCTTTAATATGA

ATGTTTCCAAATTGTATGTATGCAGACCAAAAGCCACATTGTGGGGTTTGGCCTGCATTTTTTTTGCCTA

GAATGCTATTCAAAATAGAAATTCAAGCAAAATAATATGCAGGAGATAATATAAATGGAAAAATACAACAAT

TGGAAACGAAAATTTTATGCAATATGGGCAGGGCAAGCAGTATCATTAATCACTAGTGCCATCCTGCAAATG

GCGATTATTTTTTACCTTACAGAAAAAACAGGATCTGCGATGGTCTTGTCTATGGCTTCATTAGTAGGTTTT

TTACCCTATGCGATTTTGGGACCTGCCATTGGTGTGCTAGTGGATCGTCATGATAGGAAGAAGATAATGATT

GGTGCCGATTTAATTATCGCAGCAGCTGGTGCAGTGCTTGCTATTGTTGCATTCTGTATGGAGCTACCTGTC

TGGATGATTATGATAGTATTGTTTATCCGTAGCATTGGAACAGCTTTTCATACCCCAGCACTCAATGCGGTT

ACACCACTTTTAGTACCAGAAGAACAGCTAACGAAATGCGCAGGCTATAGTCAGTCTTTGCAGTCTATAAGC

TATATTGTTAGTCCGGCAGTTGCAGCACTCTTATACTCCGTTTGGGATTTAAATGCTATTATTGCCATCGAC

GTATTGGGTGCTGTGATTGCATCTATTACGGTAGCAATTGTACGTATACCTAAGCTGGGTAATCAAGTGCAA

AGTTTAGAACCAAATTTCATAAGGGAGATGAAAGAAGGAGTTGTGGTTCTGAGACAAAACAAAGGATTGTTT

GCCTTATTACTCTTAGGAACACTATATACTTTTGTTTATATGCCAATCAATGCACTATTTCCTTTAATAAGC

ATGGAACACTTTAATGGAACGCCTGTGCATATTTCTATTACGGAAATTTCCTTTGCATTTGGGATGCTAGCA

GGAGGCTTATTATTAGGAAGATTAGGGGGCTTCGAAAAGCATGTATTACTAATAACAAGTTCATTTTTTATA

ATGGGGACCAGTTTAGCCGTTTCGGGAATACTTCCTCCAAATGGATTTGTAATATTCGTAGTTTGCTGTGCA

ATAATGGGGCTTTCGGTGCCATTTTATAGCGGTGTGCAAACAGCTCTTTTTCAGGAGAAAATTAAGCCTGAA

TATTTAGGACGTGTATTTTCTTTGATCGGAAGTATCATGTCACTTGCTATGCCAATTGGGTTAATTCTTTCT

GGATTCTTTGCTGATAAAATCGGTGTAAATCATTGGTTTTTACTATCAGGTATTTTAATTATTGGCATTGCT

ATAGTTTGCCAAATGATAACTGAGGTTAGAAAATTAGATTTAAAATAAACAATATTGGAGGAA

ATGTTATGGAATTAATATT

AAAAGCAAAAGACATTCGTGTGGAATTCAAAGGACGCGATGTTTTAGATATAAATGAATTAGAAGTATATGA

TTATGACCGTATTGGTTTAGTAGGAGCAAATGGTGCTGGAAAAAGCACTTTACTCAGGGTACTTTTAGGAGA

ATTAACTCCCCCAGGATGTAAAATGAATCGTCTGGGTGAACTTGCCTATATTCCCCAGTTGGACGAAGTAAC

TCTGCAGGAGGAAAAAGATTTTGCACTTGTAGGCAAGCTAGGTGTTGAGCAATTAAATATACAGACTATGAG

CGGTGGTGAAGAAACAAGGCTTAAAATAGCACAGGCCTTATCGGCACAGGTTCATGGTATTTTAGCGGATGA

ACCTACGAGCCATTTAGACCGTGAAGGAATTGATTTTCTAATAGGACAGCTAAAATATTTTACAGGTGCACT

GTTAGTTATTAGCCATGACCGCTATTTTCTTGATGAAATAGTAGATAAAATATGGGAACTGAAAGATGGCAA

AATCACTGAGTATTGGGGAAACTATTCTGATTATCTTCGTCAGAAAGAGGAAGAACGTAAGAGCCAAGCTGC

AGAATACGAACAATTTATTGCGGAACGTGCCCGATTGGAAAGGGCTGCGGAGGAAAAGCGAAAACAGGCTCG

TAAAATAGAACAGAAGGCAAAAGGTTCTTCAAAGAAAAAAAGTACTGAAGACGGAGGGCGTTTAGCTCATCA

AAAATCAATAGGAAGTAAGGAAAAAAAGATGTATAATGCTGCTAAAACCCTAGAGCACAGGATTGCGGCCTT

AGGAAAAGTAGAAGCTCCGGAAGGCATTCGCAGAATTCGTTTCAGGCAAAGTAAAGCATTGGAGCTCCATAA

TCCATACCCTATAGTCGGTGCAGAAATTAATAAAGTATTTGGGGATAAGGCTCTGTTTGAAAATGCATCTTT

TCAAATTCCGTTAGGAGCAAAAGTGGCGTTAACTGGTGGTAATGGAATCGGAAAAACAACTTTAATCCAAAT

GATCTTAAACCATGAAGAAGGAATTTCTATTTCGCCTAAGGCAAAAATAGGTTACTTTGCACAGAATGGTTA

CAAGTACAACAGTAATCAGAATGTTATGGAGTTTATGCAGAAGGATTGTGACTACAATATATCAGAAATTCG

TTCAGTGCTAGCATCTATGGGGTTCAAACAGAACGATATTGGAAAAAGTTTATCTGTTTTAAGCGGTGGAGA

AATTATAAAATTGTTGCTTGCTAAAATGCTCATGGGTAGATATAACATCCTAATAATGGATGAACCCAGTAA

CTTCCTTGACATACCAAGTTTAGAGGCTTTGGAAATACTAATGAAGGAGTACACCGGAACTATCGTGTTTAT

CACCCACGATAAACGATTACTCGAAAATGTAGCAGATGTAGTTTATGAAATTAGAGATAAGAAAATAAATCT

GAAACATTAAATTTAAGGTAGTCGCTGGTCAGTATAGTCTGTTCTGGTTGGCGACTCCATTGTTAAAGAGTA

TAAAGACTTTAGATTTTATGAATATTAAAAATAGGAACAGTCAATTGAACTGCTCCTATTTTTCTGCTAAAT

ATATTGTAGTTTTCTTATATGTATAATGATAGATTAGCGGATTCTCATCTACGGTACTTACTTCAAATATGA

AGAAGTGATCGCGGTTATCTCTGGACTTTTCCTTATTGAGGACAAAGTAATTCTTACGTGAAGTCGCCATTG

TTTTTAGGATATCATCAGTTAGGAAGGTCAATGGAATATTCATGTTAGAGTAGCGGTAGAAGTCACGTTCAA

AATCTTGGTAGCTCTCGCTATAATAGTCCATTTGTAGGTGATTACGCTGAAACTCAAGCTGATTCATAGAGC

ACCTCCTCGACAAGTTCAATACTAATAATGTCTTTTAATTTCAAATTGATGTGACCTGTTGTAGTTTTTATC

AAAATGAAATCTTTGGTCAGACTTGGTATTGTTCCAGTGTAGGAAACACGCTTGTTTTTTTCAATCACTTGA

ATGCGTGTGCGTAGCTGCCCGGCGTATACTTGACTGAGGAGTAATAATTTCTTCTCTAGTGATAAGTCAGAC

ATGTACGTTACTTTGTTTGTATCATCAGAGAGTGCTGATGCATGTTCAGATAGGAAAAAGCCCATCCATTTT

TGCATCTTTGTATCCTGGTACTCTCTTGCTGATTGAAATGGTAAATATGAACGGTCAATCATATCAAATCCT

TTCTATGCAGAGGCAAGGGTATTTTTATCAAATTGAATCGTAAAACCTTGAATTCCCCCACCTGTGTAACAT

TCTTTAAAGCGATTGATTACCTCAGTATAGATTATCACAGATGAGCTTGTTGGCTTAATGCTAAATGTAAAT

TCCAATGGTAATCGGTTTTCAGATTTAGCATGTACTAGTCGTATCGATATTTCAGTTGTTTTGAGTTTTCTC

TGACGAAGTTTTGAAGTTGCTGTTTCAACGATTCCATGTAAAAATCTTTCAAGCATTTCAATATCATTACAT

CCTTTGCTACGGATTTCTGAAAATTGTACTGTATTTTTTTCTTGTTTCATTTTAATCCCTCCAATCCACCCG

CGGAATGACCACCGATAAGTTTACTGCGTTCAATATTTCTGGAACCTTCAGTTAGGACGGTTCCTTTTTGTA

TGGCTAAAAAACCAAACTGTTCTCTGACAACATCAATAGCTGTCTGAAGTCTATTATCTTTTTCAATTTGTT

CTACATCATCAAAGAGTGATAGTAGAGTATAGCTTTCATCTACGAAGCCACTATAAGATACACCAATTTGTC

TCACTGCACCAGAGGTGTATTTTTTCGGAATAATACAAGTACATGACTCACCATTGTTTTGGGGAGATTTG

CGGGTTCAATTTTATTCTGAGCATTTATAGATTTTTTCATCTCAGTCCTAGAATAGCCAATATGAATAGAAA

CGACAGTAGTCAATACTAGGGCTACTGTTCCGTTCCACAGTATCATTTAAAAATCATT-TTCACACCCTTTC

GTCTATTAGTATAGAAGAAAGCTCTCAGCACA

>GA17545/Mega-2.IVc

CAAGTTGAGGCGGTAAGTTTGCTAGTCAAGGAGTAAAACGACGAAGATTAGCATTTACTTCCGCCCATGCGA

TAGCTGTCCGTGATTGACAAGTGCTAGCACGCAGACAGAACGGAGATAGCGAACCGCTGAGTGTGTCGCTCT

GCTCGTAAAAGCTTAGAAACCTTTGAACGAAAGGGATAATGAAAGCCTTGATTGCAAGGCTTTTTGCTTTAT

GGTGGGTAAGTATCAGAGTGAGAAAATTTTTGGAATGAGTAGAAGTGATAGCTAGAAATTATCAGTTTCTA

TTTCCATTTACCCTGTGGGTACGTGTTTGTTTCCATTGACAAGGAGTTTGTGGGAATAGAAATGT

ACCCACCTTGTTTGAATCAAGTGAAGTGTAGTTGAAGGAAATCTGTTGAAAGCAATACTTCATTTTACCG

AATAAGTAATAATTTAGGCAACTTCAAATCGATTAAAAAAAACTATTTTAAAGGTTAAGAGTAGACAAAA

ATTGTCCACTCTTTTTTGCAAACTCAATTTATCAATAAATGAAATGAGGGAATGTAAAATG

AAATATTTTGAGGTTGAGTTAGAAAATCCTGATGAATTTTTAAAACTACAAACAGAAGATTTTGTGAAAG

CTAATCGCTTGCTACTAAGGAAGATAATCCAGAGCGTTACAGTCTATGAAGAAAACTTCGTCATATCCTT

TAAATCTGGCATCGAATTGGAAGTATGAGTCTCATTCCATAACTTTTATATTGAACATATCATCTTTTTGTG

TTATACTATAAATTGATATAAACAAAGATGTAGGAGGAACCGAAACTATGACAGCCTCAATGCGTTTAAGAT

AAGCTGGCAATAAAAAAAGCAGAATCTATACCCGATGATAGGCTTTTTTGTTGTGCTTATTTATACGATATT

GAGCATTCATTAGTTACGGTGAGGATATTGGTTATTTAACTATACCTTTATTTAACTATGTCTTTAATATGA

ATGTTTCCAAATTGTATGTATGCAGACCAAAAGCCACATTGTGGGGTTTGGCCTGCATTTTTTATTGCCTA

GAATGCTATTCAAAATAGAAATTCAAGCAAAATAATATGCAGGAGATAATATAAATGGAAAAATACAACAAT

TGGAAACGAAAATTTTATGCAATATGGGCAGGGCAAGCAGTATCATTAATCACTAGTGCCATCCTGCAAATG

GCGATTATTTTTTACCTTACAGAAAAAACAGGATCTGCGATGGTCTTGTCTATGGCTTCATTAGTAGGTTTT

TTACCCTATGCGATTTTGGGACCTGCCATTGGTGTGCTAGTGGATCGTCATGATAGGAAGAAGATAATGATT

GGTGCCGATTTAATTATCGCAGCAGCTGGTGCAGTGCTTGCTATTGTTGCATTCTGTATGGAGCTACCTGTC

TGGATGATTATGATAGTATTGTTTATCCGTAGCATTGGAACAGCTTTTCATACCCCAGCACTCAATGCGGTT

ACACCACTTTTAGTACCAGAAGAACAGCTAACGAAATGCGCAGGCTATAGTCAGTCTTTGCAGTCTATAAGC

TATATTGTTAGTCCGGCAGTTGCAGCACTCTTATACTCCGTTTGGGATTTAAATGCTATTATTGCCATCGAC

GTATTGGGTGCTGTGATTGCATCTATTACGGTAGCAATTGTACGTATACCTAAGCTGGGTAATCAAGTGCAA

AGTTTAGAACCAAATTTCATAAGGGAGATGAAAGAAGGAGTTGTGGTTCTGAGACAAAACAAAGGATTGTTT

GCCTTATTACTCTTAGGAACACTATATACTTTTGTTTATATGCCAATCAATGCACTATTTCCTTTAATAAGC

ATGGAACACTTTAATGGAACGCCTGTGCATATTTCTATTACGGAAATTTCCTTTGCATTTGGGATGCTAGCA

GGAGGCTTATTATTAGGAAGATTAGGGGGCTTCGAAAAGCATGTATTACTAATAACAAGTTCATTTTTTATA

ATGGGGACCAGTTTAGCCGTTTCGGGAATACTTCCTCCAAATGGATTTGTAATATTCGTAGTTTGCTGTGCA

ATAATGGGGCTTTCGGTGCCATTTTATAGCGGTGTGCAAACAGCTCTTTTTCAGGAGAAAATTAAGCCTGAA

TATTTAGGACGTGTATTTTCTTTGATCGGAAGTATCATGTCACTTGCTATGCCAATTGGGTTAATTCTTTCT

GGATTCTTTGCTGATAAAATCGGTGTAAATCATTGGTTTTTACTATCAGGTATTTTAATTATTGGCATTGCT

ATAGTTTGCCAAATGATAACTGAGGTTAGAAAATTAGATTTAAAATAAACAATATTGGAGGAA

ATGTTATGGAATTAATATT

AAAAGCAAAAGACATTCGTGTGGAATTCAAAGGACGCGATGTTTTAGATATAAATGAATTAGAAGTATATGA

TTATGACCGTATTGGTTTAGTAGGAGCAAATGGTGCTGGAAAAAGCACTTTACTCAGGGTACTTTTAGGAGA

ATTAACTCCCCCAGGATGTAAAATGAATCGTCTGGGTGAACTTGCCTATATTCCCCAGTTGGACGAAGTAAC

TCTGCAGGAGGAAAAAGATTTTGCACTTGTAGGCAAGCTAGGTGTTGAGCAATTAAATATACAGACTATGAG

CGGTGGTGAAGAAACAAGGCTTAAAATAGCACAGGCCTTATCGGCACAGGTTCATGGTATTTTAGCGGATGA

ACCTACGAGCCATTTAGACCGTGAAGGAATTGATTTTCTAATAGGACAGCTAAAATATTTTACAGGTGCACT

GTTAGTTATTAGCCATGACCGCTATTTTCTTGATGAAATAGTAGATAAAATATGGGAACTGAAAGATGGCAA

AATCACTGAGTATTGGGGAAACTATTCTGATTATCTTCGTCAGAAAGAGGAAGAACGTAAGAGCCAAGCTGC

AGAATACGAACAATTTATTGCGGAACGTGCCCGATTGGAAAGGGCTGCGGAGAAAAAGCGAAAACAGGCTCG

TAAAATAGAACAGAAGGCAAAAGGTTCTTCAAAGAAAAAAAGTACTGAAGACGGAGGGCGTTTAGCTCATCA

AAAATCAATAGGAAGTAAGGAAAAAAAGATGTATAATGCTGCTAAAACCCTAGAGCACAGGATTGCGGCCTT

AGGAAAAGTAGAAGCTCCGGAAGGCATTCGCAGAATTCGTTTCAGGCAAAGTAAAGCATTGGAGCTCCATAA

TCCATACCCTATAGTCGGTGCAGAAATTAATAAAGTATTTGGGGATAAGGCTCTGTTTGAAAATGCATCTTT

TCAAATTCCGTTAGGAGCAAAAGTGGCGTTAACTGGTGGTAATGGAATCGGAAAAACAACTTTAATCCAAAT

GATCTTAAACCATGAAGAAGGAATTTCTATTTCGCCTAAGGCAAAAATAGGTTACTTTGCACAGAATGGTTA

CAAGTACAACAGTAATCAGAATGTTATGGAGTTTATGCAGAAGGATTGTGACTACAATATATCAGAAATTCG

TTCAGTGCTAGCATCTATGGGGTTCAAACAGAACGATATTGGAAAAAGTTTATCTGTTTTAAGCGGTGGAGA

AATTATAAAATTGTTGCTTGCTAAAATGCTCATGGGTAGATATAACATCCTAATAATGGATGAACCCAGTAA

CTTCCTTGACATACCAAGTTTAGAGGCTTTGGAAATACTAATGAAGGAGTACACCGGAACTATCGTGTTTAT

CACCCACGATAAACGATTACTCGAAAATGTAGCAGATGTAGTTTATGAAATTAGAGATAAGAAAATAAATCT

GAAACATTAAATTTAAGGTAGTCGCTGGTCAGTATAGTCTGTTCTGGTTGGCGACTCCATTGTTAAAGAGTA

TAAAGACTTTAGATTTTATGAATATTAAAAATAGGAACAGTCAATTGAACTGCTCCTATTTTTCTGCTAAAT

ATATTGTAGTTTTCTTATATGTATAATGATAGATTAGCGGATTCTCATCTACGGTACTTACTTCAAATATGA

AGAAGTGATCGCGGTTATCTCTGGACTTTTCCTTATTGAGGACAAAGTAATTCTTACGTGAAGTCGCCATTG

TTTTTAGGATATCATCAGTTAGGAAGGTCAATGGAATATTCATGTTAGAGTAGCGGTAGAAGTCACGTTCAA

AATCTTGGTAGCTCTCGCTATAATAGTCCATTTGTAGGTGATTACGCTGAAACTCAAGCTGATTCATAGAGC

ACCTCCTCGACAAGTTCAATACTAATAATGTCTTTTAATTTCAAATTGATGTGACCTGTTGTAGTTTTTATC

AAAATGAAATCTTTGGTCAGACTTGGTATTGTTCCAGTGTAGGAAACACGCTTGTTTTTTTCAATCACTTGA

ATGCGTGTGCGTAGCTGCCCGGCGTATACTTGACTGAGGAGTAATAATTTCTTCTCTAGTGATAAGTCAGAC

ATGTACGTTACTTTGTTTGTATCATCAGAGAGTGCTGATGCATGTTCAGATAGGAAAAAGCCCATCCATTTT

TGCATCTTTGTATCCTGGTACTCTCTTGCTGATTGAAATGGTAAATATGAACGGTCAATCATATCAAATCCT

TTCTATGCAGAGGCAAGGGTATTTTTATCAAATTGAATCGTAAAACCTTGAATTCCCCCACCTGTGTAACAT

TCTTTAAAGCGATTGATTACCTCAGTATAGATTATCACAGATGAGCTTGTTGGCTTAATGCTAAATGTAAAT

TCCAATGGTAATCGGTTTTCAGATTTAGCATGTACTAGTCGTATCGATATTTCAGTTGTTTTGAGTTTTCTC

TGACGAAGTTTTGAAGTTGCTGTTTCAACGATTCCATGTAAAAATCTTTCAAGCATTTCAATATCATTACAT

CCTTTGCTACGGATTTCTGAAAATTGTACTGTATTTTTTTCTTGTTTCATTTTAATCCCTCCAATCCACCCG

CGGAATGACCACCGATAAGTTTACTGCGTTCAATATTTCTGGAACCTTCAGTTAGGACGGTTCCTTTTTGTA

TGGCTAAAAAACCAAACTGTTCTCTGACAACATCAATAGCTGTCTGAAGTCTATTATCTTTTTCAATTTGTT

CTACATCATCAAAGAGTGATAGTAGAGTATAGCTTTCATCTACGAAGCCACTATAAGATACACCAATTTGTC

TCACTGCACCAGAGGTGTATTTTTTCGGAATAATACAAGTACATGACTCACCATTGTTTTGGGGAGATTTG

CGGGTTCAATTTTATTCTGAGCATTTATAGATTTTTTCATCTCAGTCCTAGAATAGCCAATATGAATAGAAA

CGACAGTAGTCAATACTAGGGCTACTGTTCCGTTCCACAGTATCATTTAAAAATCATT TTCACACCCTTTC

GTCTATTAGTATAGAAGAAAGCTCTCAGCACA

>GA13494/Mega-1.V

CATGTTGAGGCGGTAAGTTTGCTAGTCAAGGAGTAAAACGACGAAGATTAGCATTTACTTCCGCCCATGCGA

TAGCTGTCCGTGATTGACAAGTGCTAGCACGCAGACAGAACGGAGATAGCGAACCGCTGAGTGTGTCGCTCT

GCTCGTAAAAGCTTAGAAACCTTTGAACGAAAGGGATAATGAAAGCCTTGATTGCAAGGCTTTTTGCTTTAT

GGTGGGTAAGTATCAGAGTGAGAAAATTTTTGGAATGAGTAGAAGTGATAGCTAGAAATTATCAGTTTCTA

TTTCCATTTACCCTGTGGGTACGTGTTTGTTTCCATTGACAAGGAGTTTGTGGGAATAGAAATGT

ACCCACCTTGTTTGAATCAAGTGAAGTGTAGTTGAAGGAAATCTGTTGAAAGCAATACTTCATTTTACCG

AATAAGTAATAATTTAGGCAACTTCAAATCGATTAAAAAAAACTATTTTAAAGGTTAAGAGTAGACAAAA

ATTGTCCACTCTTTTTTGCAAACTCAATTTATCAATAAATGAAATGAGGGAATGTAAAATG

AAATATTTTGAGGTTGAGTTAGAAAATCCTGATGAATTTTTAAAACTACAAACAGAAGATTTTGTGAAAG

CTAATCGCTTGCTACTAAGGAAGATAATCCAGAGCGTTACAGTCTATGAAGAAAACTTCGTCATATCCTT

TAAATCTGGCATCGAATTGGAAGTATGAGTCTCATTCCATAACTTTTATATTGAACATATCATCTTGTTGTG

TTATACTATAAATTGATATAAACAAAGATGTAGGAGGAACCGAAACTATGACAGCCTCAATGCGTTTAAGAT

AAGCTGGCAATAAAAAAAGCAGAATCTATACCCGATGATAGGCTTTTTTGTTGTGCTTATTTATACGATATT

GAGCATTCATTAGTTACGGTGAGGATATTGGTTATTTAACTATACCTTTATTTAACTATGTCTTTAATATGA

ATGTTTCCAAATTGTATGTATGCAGACCAAAAGCCACATTGTGGGGTTTGGCCTGCATTTTTATTGCCTA

GAATGCTATTCAAAATAGAAATTCAAGCAAAATAATATGCAGGAGATAATATAAATGGAAAAATACAACAAT

TGGAAACGAAAATTTTATGCAATATGGGCAGGGCAAGCAGTATCATTAATCACTAGTGCCATCCTGCAAATG

GCGATTATTTTTTACCTTACAGAAAAAACAGGATCTGCGATGGTCTTGTCTATGGCTTCATTAGTAGGTTTT

TTACCCTATGCGATTTTGGGACCTGCCATTGGTGTGCTAGTGGATCGTCATGATAGGAAGAAGATAATGATT

GGTGCCGATTTAATTATCGCAGCAGCTGGTGCAGTGCTTGCTATTGTTGCATTCTGTATGGAGCTACCTGTC

TGGATGATTATGATAGTATTGTTTATCCGTAGCATTGGAACAGCTTTTCATACCCCAGCACTCAATGCGGTT

ACACCACTTTTAGTACCAGAAGAACAGCTAACGAAATGCGCAGGCTATAGTCAGTCTTTGCAGTCTATAAGC

TATATTGTTAGTCCGGCAGTTGCAGCACTCTTATACTCCGTTTGGGATTTAAATGCTATTATTGCCATCGAC

GTATTGGGTGCTGTGATTGCATCTATTACGGTAGCAATTGTACGTATACCTAAGCTGGGTAATCAAGTGCAA

AGTTTAGAACCAAATTTCATAAGGGAGATGAAAGAAGGAGTTGTGGTTCTGAGACAAAACAAAGGATTGTTT

GCCTTATTACTCTTAGGAACACTATATACTTTTGTTTATATGCCAATCAATGCACTATTTCCTTTAATAAGC

ATGGAACACTTTAATGGAACGCCTGTGCATATTTCTATTACGGAAATTTCCTTTGCATTTGGGATGCTAGCA

GGAGGCTTATTATTAGGAAGATTAGGGGGCTTCGAAAAGCATGTATTACTAATAACAAGTTCATTTTTTATA

ATGGGGACCAGTTTAGCCGTTTCGGGAATACTTCCTCCAAATGGATTTGTAATATTCGTAGTTTGCTGTGCA

ATAATGGGGCTTTCGGTGCCATTTTATAGCGGTGTGCAAACAGCTCTTTTTCAGGAGAAAATTAAGCCTGAA

TATTTAGGACGTGTATTTTCTTTGATCGGAAGTATCATGTCACTTGCTATGCCAATTGGGTTAATTCTTTCT

GGATTCTTTGCTGATAAAATCGGTGTAAATCATTGGTTTTTACTATCAGGTATTTTAATTATTGGCATTGCT

ATAGTTTGCCAAATGATAACTGAGGTTAGAAAATTAGATTTAAAATAAACAATATTGGAGGAATATTTATGT

ATCTTATTTTCATGTAACTCTTCCTGCTAAAATCGCAGGGTTTTCCCTGCATACAAGCAAATGAAAGCATGC

GATTATAGACAGGAGGAAATGTTATGGAATTAATATT

AAAAGCAAAAGACATTCGTGTGGAATTCAAAGGACGCGATGTTTTAGATATAAATGAATTAGAAGTATATGA

TTATGACCGTATTGGTTTAGTAGGAGCAAATGGTGCTGGAAAAAGCACTTTACTCAGGGTACTTTTAGGAGA

ATTAACTCCCCCAGGATGTAAAATGAATCGTCTGGGTGAACTTGCCTATATTCCCCAGTTGGACGAAGTAAC

TCTGCAGGAGGAAAAAGATTTTGCACTTGTAGGCAAGCTAGGTGTTGAGCAATTAAATATACAGACTATGAG

CGGTGGTGAAGAAACAAGGCTTAAAATAGCACAGGCCTTATCGGCACAGGTTCATGGTATTTTAGCGGATGA

ACCTACGAGCCATTTAGACCGTGAAGGAATTGATTTTCTAATAGGACAGCTAAAATATTTTACAGGTGCACT

GTTAGTTATTAGCCATGACCGCTATTTTCTTGATGAAATAGTAGATAAAATATGGGAACTGAAAGATGGCAA

AATCACTGAGTATTGGGGAAACTATTCTGATTATCTTCGTCAGAAAGAGGAAGAACGTAAGAGCCAAGCTGC

AGAATACGAACAATTTATTGCGGAACGTGCCCGATTGGAAAGGGCTGCGGAGGAAAAGCGAAAACAGGCTCG

TAAAATAGAACAGAAGGCAAAAGGTTCTTCAAAGAAAAAAAGTACTGAAGACGGAGGGCGTTTAGCTCATCA

AAAATCAATAGGAAGTAAGGAAAAAAAGATGTATAATGCTGCTAAAACCCTAGAGCACAGGATTGCGGCCTT

AGGAAAAGTAGAAGCTCCGGAAGGCATTCGCAGAATTCGTTTCAGGCAAAGTAAAGCATTGGAGCTCCATAA

TCCATACCCTATAGTCGGTGCAGAAATTAATAAAGTATTTGGGGATAAGGCTCTGTTTGAAAATGCATCTTT

TCAAATTCCGTTAGGAGCAAAAGTGGCGTTAACTGGTGGTAATGGAATCGGAAAAACAACTTTAATCCAAAT

GATCTTAAACCATGAAGAAGGAATTTCTATTTCGCCTAAGGCAAAAATAGGTTACTTTGCACAGAATGGTTA

CAAGTACAACAGTAATCAGAATGTTATGGAGTTTATGCAGAAGGATTGTGACTACAATATATCAGAAATTCG

TTCAGTGCTAGCATCTATGGGGTTCAAACAGAACGATATTGGAAAAAGTTTATCTGTTTTAAGCGGTGGAGA

AATTATAAAATTGTTGCTTGCTAAAATGCTCATGGGTAGATATAACATCCTAATAATGGATGAACCCAGTAA

CTTCCTTGACATACCAAGTTTAGAGGCTTTGGAAATACTAATGAAGGAGTACACCGGAACTATCGTGTTTAT

CACCCACGATAAACGATTACTCGAAAATGTAGCAGATGTAGTTTATGAAATTAGAGATAAGAAAATAAATCT

GAAACATTAAATTTAAGGTAGTCGCTGGTCAGTATAGTCTGTTCTGGTTGGCGACTCCATTGTTAAAGAGTA

TAAAGACTTTAGATTTTATGAATATTAAAAATAGGAACAGTCAATTGAACTGCTCCTATTTTTCTGCTAAAT

ATATTGTAGTTTTCTTATATGTATAATGATAGATTAGCGGATTCTCATCTACGGTACTTACTTCAAATATGA

AGAAGTGATCGCGGTTATCTCTGGACTTTTCCTTATTGAGGACAAAGTAATTCTTACGTGAAGTCGCCATTG

TTTTTAGGATATCATCAGTTAGGAAGGTCAATGGAATATTCATGTTAGAGTAGCGGTAGAAGTCACGTTCAA

AATCTTGGTAGCTCTCGCTATAATAGTCCATTTGTAGGTGATTACGCTGAAACTCAAGCTGATTCATAGAGC

ACCTCCTCGACAAGTTCAATACTAATAATGTCTTTTAATTTCAAATTGATGTGACCTGTTGTAGTTTTTATC

AAAATGAAATCTTTGGTCAGACTTGGTATTGTTCCAGTGTAGGAAACACGCTTGTTTTTTTCAATCACTTGA

ATGCGTGTGCGTAGCTGCCCGGCGTATACTTGACTGAGGAGTAATAATTTCTTCTCTAGTGATAAGTCAGAC

ATGTACGTTACTTTGTTTGTATCATCAGAGAGTGCTGATGCATGTTCAGATAGGAAAAAGCCCATCCATTTT

TGCATCTTTGTATCCTGGTACTCTCTTGCTGATTGAAATGGTAAATATGAACGGTCAATCATATCAAATCCT

TTCTATGCAGAGGCAAGGGTATTTTTATCAAATTGAATCGTAAAACCTTGAATTCCCCCACCTGTGTAACAT

TCTTTAAAGCGATTGATTACCTCAGTATAGATTATCACAGATGAGCTTGTTGGCTTAATGCTAAATGTAAAT

TCCAATGGTAATCGGTTTTCAGATTTAGCATGTACTAGTCGTATCGATATTTCAGTTGTTTTGAGTTTTCTC

TGACGAAGTTTTGAAGTTGCTGTTTCAACGATTCCATGTAAAAATCTTTCAAGCATTTCAATATCATTACAT

CCTTTGCTACGGATTTCTGAAAATTGTACTGTATTTTTTTCTTGTTTCATTTTAATCCCTCCAATCCACCCG

CGGAATGACCACCGATAAGTTTACTGCGTTCAATATTTCTGGAACCTTCAGTTAGGACGGTTCCTTTTTGTA

TGGCTAAAAAACCAAACTGTTCTCTGACAACATCAATAGCTGTCTGAAGTCTATTATCTTTTTCAATTTGTT

CTACATCATCAAAGAGTGATAGTAGAGTATAGCTTTCATCTACGAAGCCACTATAAGATACACCAATTTGTC

TCACTGCACCAGAGGTGTATTTTTTCGGAATAATACAAGTACATGACTCACCATTGTTTTGGGGAGATTTG

CGGGTTCAATTTTATTCTGAGCATTTATAGATTTTTTCATCTCAGTCCTAGAATAGCCAATATGAATAGAAA

CGACAGTAGTCAATACTAGGGCTACTGTTCCGTTCCACAGTATCATTTAAAAATCATT TTCACACCCTTTC

GTCTATTAGTATAGAAGAAAGCTCTCAGCACA

>GA16833/Mega-1.V

CATGTTGAGGCGGTAAGTTTGCTAGTCAAGGAGTAAAACGACGAAGATTAGCATTTACTTCCGCCCATGCGA

TAGCTGTCCGTGATTGACAAGTGCTAGCACGCAGACAGAACGGAGATAGCGAACCGCTGAGTGTGTCGCTCT

GCTCGTAAAAGCTTAGAAACCTTTGAACGAAAGGGATAATGAAAGCCTTGATTGCAAGGCTTTTTGCTTTAT

GGTGGGTAAGTATCAGAGTGAGAAAATTTTTGGAATGAGTAGAAGTGATAGCTAGAAATTATCAGTTTCTA

TTTCCATTTACCCTGTGGGTACGTGTTTGTTTCCATTGACAAGGAGTTTGTGGGAATAGAAATGT

ACCCACCTTGTTTGAATCAAGTGAAGTGTAGTTGAAGGAAATCTGTTGAAAGCAATACTTCATTTTACCG

AATAAGTAATAATTTAGGCAACTTCAAATCGATTAAAAAAAACTATTTTAAAGGTTAAGAGTAGACAAAA

ATTGTCCACTCTTTTTTGCAAACTCAATTTATCAATAAATGAAATGAGGGAATGTAAAATG

AAATATTTTGAGGTTGAGTTAGAAAATCCTGATGAATTTTTAAAACTACAAACAGAAGATTTTGTGAAAG

CTAATCGCTTGCTACTAAGGAAGATAATCCAGAGCGTTACAGTCTATGAAGAAAACTTCGTCATATCCTT

TAAATCTGGCATCGAATTGGAAGTATGAGTCTCATTCCATAACTTTTATATTGAACATATCATCTTGTTGTG

TTATACTATAAATTGATATAAACAAAGATGTAGGAGGAACCGAAACTATGACAGCCTCAATGCGTTTAAGAT

AAGCTGGCAATAAAAAAAGCAGAATCTATACCCGATGATAGGCTTTTTTGTTGTGCTTATTTATACGATATT

GAGCATTCATTAGTTACGGTGAGGATATTGGTTATTTAACTATACCTTTATTTAACTATGTCTTTAATATGA

ATGTTTCCAAATTGTATGTATGCAGACCAAAAGCCACATTGTGGGGTTTGGCCTGCATTTTTT ATTGCCTA

GAATGCTATTCAAAATAGAAATTCAAGCAAAATAATATGCAGGAGATAATATAAATGGAAAAATACAACAAT

TGGAAACGAAAATTTTATGCAATATGGGCAGGGCAAGCAGTATCATTAATCACTAGTGCCATCCTGCAAATG

GCGATTATTTTTTACCTTACAGAAAAAACAGGATCTGCGATGGTCTTGTCTATGGCTTCATTAGTAGGTTTT

TTACCCTATGCGATTTTGGGACCTGCCATTGGTGTGCTAGTGGATCGTCATGATAGGAAGAAGATAATGATT

GGTGCCGATTTAATTATCGCAGCAGCTGGTGCAGTGCTTGCTATTGTTGCATTCTGTATGGAGCTACCTGTC

TGGATGATTATGATAGTATTGTTTATCCGTAGCATTGGAACAGCTTTTCATACCCCAGCACTCAATGCGGTT

ACACCACTTTTAGTACCAGAAGAACAGCTAACGAAATGCGCAGGCTATAGTCAGTCTTTGCAGTCTATAAGC

TATATTGTTAGTCCGGCAGTTGCAGCACTCTTATACTCCGTTTGGGATTTAAATGCTATTATTGCCATCGAC

GTATTGGGTGCTGTGATTGCATCTATTACGGTAGCAATTGTACGTATACCTAAGCTGGGTAATCAAGTGCAA

AGTTTAGAACCAAATTTCATAAGGGAGATGAAAGAAGGAGTTGTGGTTCTGAGACAAAACAAAGGATTGTTT

GCCTTATTACTCTTAGGAACACTATATACTTTTGTTTATATGCCAATCAATGCACTATTTCCTTTAATAAGC

ATGGAACACTTTAATGGAACGCCTGTGCATATTTCTATTACGGAAATTTCCTTTGCATTTGGGATGCTAGCA

GGAGGCTTATTATTAGGAAGATTAGGGGGCTTCGAAAAGCATGTATTACTAATAACAAGTTCATTTTTTATA

ATGGGGACCAGTTTAGCCGTTTCGGGAATACTTCCTCCAAATGGATTTGTAATATTCGTAGTTTGCTGTGCA

ATAATGGGGCTTTCGGTGCCATTTTATAGCGGTGTGCAAACAGCTCTTTTTCAGGAGAAAATTAAGCCTGAA

TATTTAGGACGTGTATTTTCTTTGATCGGAAGTATCATGTCACTTGCTATGCCAATTGGGTTAATTCTTTCT

GGATTCTTTGCTGATAAAATCGGTGTAAATCATTGGTTTTTACTATCAGGTATTTTAATTATTGGCATTGCT

ATAGTTTGCCAAATGATAACTGAGGTTAGAAAATTAGATTTAAAATAAACAATATTGGAGGAATATTTATGT

ATCTTATTTTCATGTAACTCTTCCTGCTAAAATCGCAGGGTTTTCCCTGCATACAAGCAAATGAAAGCATGC

GATTATAGACAGGAGGAAATGTTATGGAATTAATATT

AAAAGCAAAAGACATTCGTGTGGAATTCAAAGGACGCGATGTTTTAGATATAAATGAATTAGAAGTATATGA

TTATGACCGTATTGGTTTAGTAGGAGCAAATGGTGCTGGAAAAAGCACTTTACTCAGGGTACTTTTAGGAGA

ATTAACTCCCCCAGGATGTAAAATGAATCGTCTGGGTGAACTTGCCTATATTCCCCAGTTGGACGAAGTAAC

TCTGCAGGAGGAAAAAGATTTTGCACTTGTAGGCAAGCTAGGTGTTGAGCAATTAAATATACAGACTATGAG

CGGTGGTGAAGAAACAAGGCTTAAAATAGCACAGGCCTTATCGGCACAGGTTCATGGTATTTTAGCGGATGA

ACCTACGAGCCATTTAGACCGTGAAGGAATTGATTTTCTAATAGGACAGCTAAAATATTTTACAGGTGCACT

GTTAGTTATTAGCCATGACCGCTATTTTCTTGATGAAATAGTAGATAAAATATGGGAACTGAAAGATGGCAA

AATCACTGAGTATTGGGGAAACTATTCTGATTATCTTCGTCAGAAAGAGGAAGAACGTAAGAGCCAAGCTGC

AGAATACGAACAATTTATTGCGGAACGTGCCCGATTGGAAAGGGCTGCGGAGGAAAAGCGAAAACAGGCTCG

TAAAATAGAACAGAAGGCAAAAGGTTCTTCAAAGAAAAAAAGTACTGAAGACGGAGGGCGTTTAGCTCATCA

AAAATCAATAGGAAGTAAGGAAAAAAAGATGTATAATGCTGCTAAAACCCTAGAGCACAGGATTGCGGCCTT

AGGAAAAGTAGAAGCTCCGGAAGGCATTCGCAGAATTCGTTTCAGGCAAAGTAAAGCATTGGAGCTCCATAA

TCCATACCCTATAGTCGGTGCAGAAATTAATAAAGTATTTGGGGATAAGGCTCTGTTTGAAAATGCATCTTT

TCAAATTCCGTTAGGAGCAAAAGTGGCGTTAACTGGTGGTAATGGAATCGGAAAAACAACTTTAATCCAAAT

GATCTTAAACCATGAAGAAGGAATTTCTATTTCGCCTAAGGCAAAAATAGGTTACTTTGCACAGAATGGTTA

CAAGTACAACAGTAATCAGAATGTTATGGAGTTTATGCAGAAGGATTGTGACTACAATATATCAGAAATTCG

TTCAGTGCTAGCATCTATGGGGTTCAAACAGAACGATATTGGAAAAAGTTTATCTGTTTTAAGCGGTGGAGA

AATTATAAAATTGTTGCTTGCTAAAATGCTCATGGGTAGATATAACATCCTAATAATGGATGAACCCAGTAA

CTTCCTTGACATACCAAGTTTAGAGGCTTTGGAAATACTAATGAAGGAGTACACCGGAACTATCGTGTTTAT

CACCCACGATAAACGATTACTCGAAAATGTAGCAGATGTAGTTTATGAAATTAGAGATAAGAAAATAAATCT

GAAACATTAAATTTAAGGTAGTCGCTGGTCAGTATAGTCTGTTCTGGTTGGCGACTCCATTGTTAAAGAGTA

TAAAGACTTTAGATTTTATGAATATTAAAAATAGGAACAGTCAATTGAACTGCTCCTATTTTTCTGCTAAAT

ATATTGTAGTTTTCTTATATGTATAATGATAGATTAGCGGATTCTCATCTACGGTACTTACTTCAAATATGA

AGAAGTGATCGCGGTTATCTCTGGACTTTTCCTTATTGAGGACAAAGTAATTCTTACGTGAAGTCGCCATTG

TTTTTAGGATATCATCAGTTAGGAAGGTCAATGGAATATTCATGTTAGAGTAGCGGTAGAAGTCACGTTCAA

AATCTTGGTAGCTCTCGCTATAATAGTCCATTTGTAGGTGATTACGCTGAAACTCAAGCTGATTCATAGAGC

ACCTCCTCGACAAGTTCAATACTAATAATGTCTTTTAATTTCAAATTGATGTGACCTGTTGTAGTTTTTATC

AAAATGAAATCTTTGGTCAGACTTGGTATTGTTCCAGTGTAGGAAACACGCTTGTTTTTTTCAATCACTTGA

ATGCGTGTGCGTAGCTGCCCGGCGTATACTTGACTGAGGAGTAATAATTTCTTCTCTAGTGATAAGTCAGAC

ATGTACGTTACTTTGTTTGTATCATCAGAGAGTGCTGATGCATGTTCAGATAGGAAAAAGCCCATCCATTTT

TGCATCTTTGTATCCTGGTACTCTCTTGCTGATTGAAATGGTAAATATGAACGGTCAATCATATCAAATCCT

TTCTATGCAGAGGCAAGGGTATTTTTATCAAATTGAATCGTAAAACCTTGAATTCCCCCACCTGTGTAACAT

TCTTTAAAGCGATTGATTACCTCAGTATAGATTATCACAGATGAGCTTGTTGGCTTAATGCTAAATGTAAAT

TCCAATGGTAATCGGTTTTCAGATTTAGCATGTACTAGTCGTATCGATATTTCAGTTGTTTTGAGTTTTCTC

TGACGAAGTTTTGAAGTTGCTGTTTCAACGATTCCATGTAAAAATCTTTCAAGCATTTCAATATCATTACAT

CCTTTGCTACGGATTTCTGAAAATTGTACTGTATTTTTTTCTTGTTTCATTTTAATCCCTCCAATCCACCCG

CGGAATGACCACCGATAAGTTTACTGCGTTCAATATTTCTGGAACCTTCAGTTAGGACGGTTCCTTTTTGTA

TGGCTAAAAAACCAAACTGTTCTCTGACAACATCAATAGCTGTCTGAAGTCTATTATCTTTTTCAATTTGTT

CTACATCATCAAAGAGTGATAGTAGAGTATAGCTTTCATCTACGAAGCCACTATAAGATACACCAATTTGTC

TCACTGCACCAGAGGTGTATTTTTTCGGAATAATACAAGTACATGACTCACCATTGTTTTGGGGAGATTTG

CGGGTTCAATTTTATTCTGAGCATTTATAGATTTTTTCATCTCAGTCCTAGAATAGCCAATATGAATAGAAA

CGACAGTAGTCAATACTAGGGCTACTGTTCCGTTCCACAGTATCATTTAAAAATCATT-TTCACACCCTTTC

GTCTATTAGTATAGAAGAAAGCTCTCAGCACA

>GA18523/Mega-1.V

CATGTTGAGGCGGTAAGTTTGCTAGTCAAGGAGTAAAACGACGAAGATTAGCATTTACTTCCGCCCATGCGA

TAGCTGTCCGTGATTGACAAGTGCTAGCACGCAGACAGAACGGAGATAGCGAACCGCTGAGTGTGTCGCTCT

GCTCGTAAAAGCTTAGAAACCTTTGAACGAAAGGGATAATGAAAGCCTTGATTGCAAGGCTTTTTGCTTTAT

GGTGGGTAAGTATCAGAGTGAGAAAATTTTTGGAATGAGTAGAAGTGATAGCTAGAAATTATCAGTTTCTA

TTTCCATTTACCCTGTGGGTACGTGTTTGTTTCCATTGACAAGGAGTTTGTGGGAATAGAAATGT

ACCCACCTTGTTTGAATCAAGTGAAGTGTAGTTGAAGGAAATCTGTTGAAAGCAATACTTCATTTTACCG

AATAAGTAATAATTTAGGCAACTTCAAATCGATTAAAAAAAACTATTTTAAAGGTTAAGAGTAGACAAAA

ATTGTCCACTCTTTTTTGCAAACTCAATTTATCAATAAATGAAATGAGGGAATGTAAAATG

AAATATTTTGAGGTTGAGTTAGAAAATCCTGATGAATTTTTAAAACTACAAACAGAAGATTTTGTGAAAG

CTAATCGCTTGCTACTAAGGAAGATAATCCAGAGCGTTACAGTCTATGAAGAAAACTTCGTCATATCCTT

TAAATCTGGCATCGAATTGGAAGTATGAGTCTCATTCCATAACTTTTATATTGAACATATCATCTTGTTGTG

TTATACTATAAATTGATATAAACAAAGATGTAGGAGGAACCGAAACTATGACAGCCTCAATGCGTTTAAGAT

AAGCTGGCAATAAAAAAAGCAGAATCTATACCCGATGATAGGCTTTTTTGTTGTGCTTATTTATACGATATT

GAGCATTCATTAGTTACGGTGAGGATATTGGTTATTTAACTATACCTTTATTTAACTATGTCTTTAATATGA

ATGTTTCCAAATTGTATGTATGCAGACCAAAAGCCACATTGTGGGGTTTGGCCTGCATTTTTTTTGCCTA

GAATGCTATTCAAAATAGAAATTCAAGCAAAATAATATGCAGGAGATAATATAAATGGAAAAATACAACAAT

TGGAAACGAAAATTTTATGCAATATGGGCAGGGCAAGCAGTATCATTAATCACTAGTGCCATCCTGCAAATG

GCGATTATTTTTTACCTTACAGAAAAAACAGGATCTGCGATGGTCTTGTCTATGGCTTCATTAGTAGGTTTT

TTACCCTATGCGATTTTGGGACCTGCCATTGGTGTGCTAGTGGATCGTCATGATAGGAAGAAGATAATGATT

GGTGCCGATTTAATTATCGCAGCAGCTGGTGCAGTGCTTGCTATTGTTGCATTCTGTATGGAGCTACCTGTC

TGGATGATTATGATAGTATTGTTTATCCGTAGCATTGGAACAGCTTTTCATACCCCAGCACTCAATGCGGTT

ACACCACTTTTAGTACCAGAAGAACAGCTAACGAAATGCGCAGGCTATAGTCAGTCTTTGCAGTCTATAAGC

TATATTGTTAGTCCGGCAGTTGCAGCACTCTTATACTCCGTTTGGGATTTAAATGCTATTATTGCCATCGAC

GTATTGGGTGCTGTGATTGCATCTATTACGGTAGCAATTGTACGTATACCTAAGCTGGGTAATCAAGTGCAA

AGTTTAGAACCAAATTTCATAAGGGAGATGAAAGAAGGAGTTGTGGTTCTGAGACAAAACAAAGGATTGTTT

GCCTTATTACTCTTAGGAACACTATATACTTTTGTTTATATGCCAATCAATGCACTATTTCCTTTAATAAGC

ATGGAACACTTTAATGGAACGCCTGTGCATATTTCTATTACGGAAATTTCCTTTGCATTTGGGATGCTAGCA

GGAGGCTTATTATTAGGAAGATTAGGGGGCTTCGAAAAGCATGTATTACTAATAACAAGTTCATTTTTTATA

ATGGGGACCAGTTTAGCCGTTTCGGGAATACTTCCTCCAAATGGATTTGTAATATTCGTAGTTTGCTGTGCA

ATAATGGGGCTTTCGGTGCCATTTTATAGCGGTGTGCAAACAGCTCTTTTTCAGGAGAAAATTAAGCCTGAA

TATTTAGGACGTGTATTTTCTTTGATCGGAAGTATCATGTCACTTGCTATGCCAATTGGGTTAATTCTTTCT

GGATTCTTTGCTGATAAAATCGGTGTAAATCATTGGTTTTTACTATCAGGTATTTTAATTATTGGCATTGCT

ATAGTTTGCCAAATGATAACTGAGGTTAGAAAATTAGATTTAAAATAAACAATATTGGAGGAATATTTATGT

ATCTTATTTTCATGTAACTCTTCCTGCTAAAATCGCAGGGTTTTCCCTGCATACAAGCAAATGAAAGCATGC

GATTATAGACAGGAGGAAATGTTATGGAATTAATATT

AAAAGCAAAAGACATTCGTGTGGAATTCAAAGGACGCGATGTTTTAGATATAAATGAATTAGAAGTATATGA

TTATGACCGTATTGGTTTAGTAGGAGCAAATGGTGCTGGAAAAAGCACTTTACTCAGGGTACTTTTAGGAGA

ATTAACTCCCCCAGGATGTAAAATGAATCGTCTGGGTGAACTTGCCTATATTCCCCAGTTGGACGAAGTAAC

TCTGCAGGAGGAAAAAGATTTTGCACTTGTAGGCAAGCTAGGTGTTGAGCAATTAAATATACAGACTATGAG

CGGTGGTGAAGAAACAAGGCTTAAAATAGCACAGGCCTTATCGGCACAGGTTCATGGTATTTTAGCGGATGA

ACCTACGAGCCATTTAGACCGTGAAGGAATTGATTTTCTAATAGGACAGCTAAAATATTTTACAGGTGCACT

GTTAGTTATTAGCCATGACCGCTATTTTCTTGATGAAATAGTAGATAAAATATGGGAACTGAAAGATGGCAA

AATCACTGAGTATTGGGGAAACTATTCTGATTATCTTCGTCAGAAAGAGGAAGAACGTAAGAGCCAAGCTGC

AGAATACGAACAATTTATTGCGGAACGTGCCCGATTGGAAAGGGCTGCGGAGGAAAAGCGAAAACAGGCTCG

TAAAATAGAACAGAAGGCAAAAGGTTCTTCAAAGAAAAAAAGTACTGAAGACGGAGGGCGTTTAGCTCATCA

AAAATCAATAGGAAGTAAGGAAAAAAAGATGTATAATGCTGCTAAAACCCTAGAGCACAGGATTGCGGCCTT

AGGAAAAGTAGAAGCTCCGGAAGGCATTCGCAGAATTCGTTTCAGGCAAAGTAAAGCATTGGAGCTCCATAA

TCCATACCCTATAGTCGGTGCAGAAATTAATAAAGTATTTGGGGATAAGGCTCTGTTTGAAAATGCATCTTT

TCAAATTCCGTTAGGAGCAAAAGTGGCGTTAACTGGTGGTAATGGAATCGGAAAAACAACTTTAATCCAAAT

GATCTTAAACCATGAAGAAGGAATTTCTATTTCGCCTAAGGCAAAAATAGGTTACTTTGCACAGAATGGTTA

CAAGTACAACAGTAATCAGAATGTTATGGAGTTTATGCAGAAGGATTGTGACTACAATATATCAGAAATTCG

TTCAGTGCTAGCATCTATGGGGTTCAAACAGAACGATATTGGAAAAAGTTTATCTGTTTTAAGCGGTGGAGA

AATTATAAAATTGTTGCTTGCTAAAATGCTCATGGGTAGATATAACATCCTAATAATGGATGAACCCAGTAA

CTTCCTTGACATACCAAGTTTAGAGGCTTTGGAAATACTAATGAAGGAGTACACCGGAACTATCGTGTTTAT

CACCCACGATAAACGATTACTCGAAAATGTAGCAGATGTAGTTTATGAAATTAGAGATAAGAAAATAAATCT

GAAACATTAAATTTAAGGTAGTCGCTGGTCAGTATAGTCTGTTCTGGTTGGCGACTCCATTGTTAAAGAGTA

TAAAGACTTTAGATTTTATGAATATTAAAAATAGGAACAGTCAATTGAACTGCTCCTATTTTTCTGCTAAAT

ATATTGTAGTTTTCTTATATGTATAATGATAGATTAGCGGATTCTCATCTACGGTACTTACTTCAAATATGA

AGAAGTGATCGCGGTTATCTCTGGACTTTTCCTTATTGAGGACAAAGTAATTCTTACGTGAAGTCGCCATTG

TTTTTAGGATATCATCAGTTAGGAAGGTCAATGGAATATTCATGTTAGAGTAGCGGTAGAAGTCACGTTCAA

AATCTTGGTAGCTCTCGCTATAATAGTCCATTTGTAGGTGATTACGCTGAAACTCAAGCTGATTCATAGAGC

ACCTCCTCGACAAGTTCAATACTAATAATGTCTTTTAATTTCAAATTGATGTGACCTGTTGTAGTTTTTATC

AAAATGAAATCTTTGGTCAGACTTGGTATTGTTCCAGTGTAGGAAACACGCTTGTTTTTTTCAATCACTTGA

ATGCGTGTGCGTAGCTGCCCGGCGTATACTTGACTGAGGAGTAATAATTTCTTCTCTAGTGATAAGTCAGAC

ATGTACGTTACTTTGTTTGTATCATCAGAGAGTGCTGATGCATGTTCAGATAGGAAAAAGCCCATCCATTTT

TGCATCTTTGTATCCTGGTACTCTCTTGCTGATTGAAATGGTAAATATGAACGGTCAATCATATCAAATCCT

TTCTATGCAGAGGCAAGGGTATTTTTATCAAATTGAATCGTAAAACCTTGAATTCCCCCACCTGTGTAACAT

TCTTTAAAGCGATTGATTACCTCAGTATAGATTATCACAGATGAGCTTGTTGGCTTAATGCTAAATGTAAAT

TCCAATGGTAATCGGTTTTCAGATTTAGCATGTACTAGTCGTATCGATATTTCAGTTGTTTTGAGTTTTCTC

TGACGAAGTTTTGAAGTTGCTGTTTCAACGATTCCATGTAAAAATCTTTCAAGCATTTCAATATCATTACAT

CCTTTGCTACGGATTTCTGAAAATTGTACTGTATTTTTTTCTTGTTTCATTTTAATCCCTCCAATCCACCCG

CGGAATGACCACCGATAAGTTTACTGCGTTCAATATTTCTGGAACCTTCAGTTAGGACGGTTCCTTTTTGTA

TGGCTAAAAAACCAAACTGTTCTCTGACAACATCAATAGCTGTCTGAAGTCTATTATCTTTTTCAATTTGTT

CTACATCATCAAAGAGTGATAGTAGAGTATAGCTTTCATCTACGAAGCCACTATAAGATACACCAATTTGTC

TCACTGCACCAGAGGTGTATTTTTTCGGAATAATACAAGTACATGACTCACCATTGTTTTGGGGAGATTTG

CGGGTTCAATTTTATTCTGAGCATTTATAGATTTTTTCATCTCAGTCCTAGAATAGCCAATATGAATAGAAA

CGACAGTAGTCAATACTAGGGCTACTGTTCCGTTCCACAGTATCATTTAAAAATCATTTTCACACCCTTTC

GTCTATTAGTATAGAAGAAAGCTCTCAGCACA

>GA16121/Mega-1.V

CATGTTGAGGCGGTAAGTTTGCTAGTCAAGGAGTAAAACGACGAAGATTAGCATTTACTTCCGCCCATGCGA

TAGCTGTCCGTGATTGACAAGTGCTAGCACGCAGACAGAACGGAGATAGCGAACCGCTGAGTGTGTCGCTCT

GCTCGTAAAAGCTTAGAAACCTTTGAACGAAAGGGATAATGAAAGCCTTGATTGCAAGGCTTTTTGCTTTAT

GGTGGGTAAGTATCAGAGTGAGAAAATTTTTGGAATGAGTAGAAGTGATAGCTAGAAATTATCAGTTTCTA

TTTCCATTTACCCTGTGGGTACGTGTTTGTTTCCATTGACAAGGAGTTTGTGGGAATAGAAATGT

ACCCACCTTGTTTGAATCAAGTGAAGTGTAGTTGAAGGAAATCTGTTGAAAGCAATACTTCATTTTACCG

AATAAGTAATAATTTAGGCAACTTCAAATCGATTAAAAAAAACTATTTTAAAGGTTAAGAGTAGACAAAA

ATTGTCCACTCTTTTTTGCAAACTCAATTTATCAATAAATGAAATGAGGGAATGTAAAATG

AAATATTTTGAGGTTGAGTTAGAAAATCCTGATGAATTTTTAAAACTACAAACAGAAGATTTTGTGAAAG

CTAATCGCTTGCTACTAAGGAAGATAATCCAGAGCGTTACAGTCTATGAAGAAAACTTCGTCATATCCTT

TAAATCTGGCATCGAATTGGAAGTATGAGTCTCATTCCATAACTTTTATATTGAACATATCATCTTGTTGTG

TTATACTATAAATTGATATAAACAAAGATGTAGGAGGAACCGAAACTATGACAGCCTCAATGCGTTTAAGAT

AAGCTGGCAATAAAAAAAGCAGAATCTATACCCGATGATAGGCTTTTTTGTTGTGCTTATTTATACGATATT

GAGCATTCATTAGTTACGGTGAGGATATTGGTTATTTAACTATACCTTTATTTAACTATGTCTTTAATATGA

ATGTTTCCAAATTGTATGTATGCAGACCAAAAGCCACATTGTGGGGTTTGGCCTGCATTTTTTATTGCCTA

GAATGCTATTCAAAATAGAAATTCAAGCAAAATAATATGCAGGAGATAATATAAATGGAAAAATACAACAAT

TGGAAACGAAAATTTTATGCAATATGGGCAGGGCAAGCAGTATCATTAATCACTAGTGCCATCCTGCAAATG

GCGATTATTTTTTACCTTACAGAAAAAACAGGATCTGCGATGGTCTTGTCTATGGCTTCATTAGTAGGTTTT

TTACCCTATGCGATTTTGGGACCTGCCATTGGTGTGCTAGTGGATCGTCATGATAGGAAGAAGATAATGATT

GGTGCCGATTTAATTATCGCAGCAGCTGGTGCAGTGCTTGCTATTGTTGCATTCTGTATGGAGCTACCTGTC

TGGATGATTATGATAGTATTGTTTATCCGTAGCATTGGAACAGCTTTTCATACCCCAGCACTCAATGCGGTT

ACACCACTTTTAGTACCAGAAGAACAGCTAACGAAATGCGCAGGCTATAGTCAGTCTTTGCAGTCTATAAGC

TATATTGTTAGTCCGGCAGTTGCAGCACTCTTATACTCCGTTTGGGATTTAAATGCTATTATTGCCATCGAC

GTATTGGGTGCTGTGATTGCATCTATTACGGTAGCAATTGTACGTATACCTAAGCTGGGTAATCAAGTGCAA

AGTTTAGAACCAAATTTCATAAGGGAGATGAAAGAAGGAGTTGTGGTTCTGAGACAAAACAAAGGATTGTTT

GCCTTATTACTCTTAGGAACACTATATACTTTTGTTTATATGCCAATCAATGCACTATTTCCTTTAATAAGC

ATGGAACACTTTAATGGAACGCCTGTGCATATTTCTATTACGGAAATTTCCTTTGCATTTGGGATGCTAGCA

GGAGGCTTATTATTAGGAAGATTAGGGGGCTTCGAAAAGCATGTATTACTAATAACAAGTTCATTTTTTATA

ATGGGGACCAGTTTAGCCGTTTCGGGAATACTTCCTCCAAATGGATTTGTAATATTCGTAGTTTGCTGTGCA

ATAATGGGGCTTTCGGTGCCATTTTATAGCGGTGTGCAAACAGCTCTTTTTCAGGAGAAAATTAAGCCTGAA

TATTTAGGACGTGTATTTTCTTTGATCGGAAGTATCATGTCACTTGCTATGCCAATTGGGTTAATTCTTTCT

GGATTCTTTGCTGATAAAATCGGTGTAAATCATTGGTTTTTACTATCAGGTATTTTAATTATTGGCATTGCT

ATAGTTTGCCAAATGATAACTGAGGTTAGAAAATTAGATTTAAAATAAACAATATTGGAGGAATATTTATGT

ATCTTATTTTCATGTAACTCTTCCTGCTAAAATCGCAGGGTTTTCCCTGCATACAAGCAAATGAAAGCATGC

GATTATAGACAGGAGGAAATGTTATGGAATTAATATT

AAAAGCAAAAGACATTCGTGTGGAATTCAAAGGACGCGATGTTTTAGATATAAATGAATTAGAAGTATATGA

TTATGACCGTATTGGTTTAGTAGGAGCAAATGGTGCTGGAAAAAGCACTTTACTCAGGGTACTTTTAGGAGA

ATTAACTCCCCCAGGATGTAAAATGAATCGTCTGGGTGAACTTGCCTATATTCCCCAGTTGGACGAAGTAAC

TCTGCAGGAGGAAAAAGATTTTGCACTTGTAGGCAAGCTAGGTGTTGAGCAATTAAATATACAGACTATGAG

CGGTGGTGAAGAAACAAGGCTTAAAATAGCACAGGCCTTATCGGCACAGGTTCATGGTATTTTAGCGGATGA

ACCTACGAGCCATTTAGACCGTGAAGGAATTGATTTTCTAATAGGACAGCTAAAATATTTTACAGGTGCACT

GTTAGTTATTAGCCATGACCGCTATTTTCTTGATGAAATAGTAGATAAAATATGGGAACTGAAAGATGGCAA

AATCACTGAGTATTGGGGAAACTATTCTGATTATCTTCGTCAGAAAGAGGAAGAACGTAAGAGCCAAGCTGC

AGAATACGAACAATTTATTGCGGAACGTGCCCGATTGGAAAGGGCTGCGGAGGAAAAGCGAAAACAGGCTCG

TAAAATAGAACAGAAGGCAAAAGGTTCTTCAAAGAAAAAAAGTACTGAAGACGGAGGGCGTTTAGCTCATCA

AAAATCAATAGGAAGTAAGGAAAAAAAGATGTATAATGCTGCTAAAACCCTAGAGCACAGGATTGCGGCCTT

AGGAAAAGTAGAAGCTCCGGAAGGCATTCGCAGAATTCGTTTCAGGCAAAGTAAAGCATTGGAGCTCCATAA

TCCATACCCTATAGTCGGTGCAGAAATTAATAAAGTATTTGGGGATAAGGCTCTGTTTGAAAATGCATCTTT

TCAAATTCCGTTAGGAGCAAAAGTGGCGTTAACTGGTGGTAATGGAATCGGAAAAACAACTTTAATCCAAAT

GATCTTAAACCATGAAGAAGGAATTTCTATTTCGCCTAAGGCAAAAATAGGTTACTTTGCACAGAATGGTTA

CAAGTACAACAGTAATCAGAATGTTATGGAGTTTATGCAGAAGGATTGTGACTACAATATATCAGAAATTCG

TTCAGTGCTAGCATCTATGGGGTTCAAACAGAACGATATTGGAAAAAGTTTATCTGTTTTAAGCGGTGGAGA

AATTATAAAATTGTTGCTTGCTAAAATGCTCATGGGTAGATATAACATCCTAATAATGGATGAACCCAGTAA

CTTCCTTGACATACCAAGTTTAGAGGCTTTGGAAATACTAATGAAGGAGTACACCGGAACTATCGTGTTTAT

CACCCACGATAAACGATTACTCGAAAATGTAGCAGATGTAGTTTATGAAATTAGAGATAAGAAAATAAATCT

GAAACATTAAATTTAAGGTAGTCGCTGGTCAGTATAGTCTGTTCTGGTTGGCGACTCCATTGTTAAAGAGTA

TAAAGACTTTAGATTTTATGAATATTAAAAATAGGAACAGTCAATTGAACTGCTCCTATTTTTCTGCTAAAT

ATATTGTAGTTTTCTTATATGTATAATGATAGATTAGCGGATTCTCATCTACGGTACTTACTTCAAATATGA

AGAAGTGATCGCGGTTATCTCTGGACTTTTCCTTATTGAGGACAAAGTAATTCTTACGTGAAGTCGCCATTG

TTTTTAGGATATCATCAGTTAGGAAGGTCAATGGAATATTCATGTTAGAGTAGCGGTAGAAGTCACGTTCAA

AATCTTGGTAGCTCTCGCTATAATAGTCCATTTGTAGGTGATTACGCTGAAACTCAAGCTGATTCATAGAGC

ACCTCCTCGACAAGTTCAATACTAATAATGTCTTTTAATTTCAAATTGATGTGACCTGTTGTAGTTTTTATC

AAAATGAAATCTTTGGTCAGACTTGGTATTGTTCCAGTGTAGGAAACACGCTTGTTTTTTTCAATCACTTGA

ATGCGTGTGCGTAGCTGCCCGGCGTATACTTGACTGAGGAGTAATAATTTCTTCTCTAGTGATAAGTCAGAC

ATGTACGTTACTTTGTTTGTATCATCAGAGAGTGCTGATGCATGTTCAGATAGGAAAAAGCCCATCCATTTT

TGCATCTTTGTATCCTGGTACTCTCTTGCTGATTGAAATGGTAAATATGAACGGTCAATCATATCAAATCCT

TTCTATGCAGAGGCAAGGGTATTTTTATCAAATTGAATCGTAAAACCTTGAATTCCCCCACCTGTGTAACAT

TCTTTAAAGCGATTGATTACCTCAGTATAGATTATCACAGATGAGCTTGTTGGCTTAATGCTAAATGTAAAT

TCCAATGGTAATCGGTTTTCAGATTTAGCATGTACTAGTCGTATCGATATTTCAGTTGTTTTGAGTTTTCTC

TGACGAAGTTTTGAAGTTGCTGTTTCAACGATTCCATGTAAAAATCTTTCAAGCATTTCAATATCATTACAT

CCTTTGCTACGGATTTCTGAAAATTGTACTGTATTTTTTTCTTGTTTCATTTTAATCCCTCCAATCCACCCG

CGGAATGACCACCGATAAGTTTACTGCGTTCAATATTTCTGGAACCTTCAGTTAGGACGGTTCCTTTTTGTA

TGGCTAAAAAACCAAACTGTTCTCTGACAACATCAATAGCTGTCTGAAGTCTATTATCTTTTTCAATTTGTT

CTACATCATCAAAGAGTGATAGTAGAGTATAGCTTTCATCTACGAAGCCACTATAAGATACACCAATTTGTC

TCACTGCACCAGAGGTGTATTTTTTCGGAATAATACAAGTACATGACTCACCATTGTTTTGGGGAGATTTG

CGGGTTCAATTTTATTCTGAGCATTTATAGATTTTTTCATCTCAGTCCTAGAATAGCCAATATGAATAGAAA

CGACAGTAGTCAATACTAGGGCTACTGTTCCGTTCCACAGTATCATTTAAAAATCATTTTCACACCCTTTC

GTCTATTAGTATAGAAGAAAGCTCTCAGCACA

>GA17227/Mega-1.V

CATGTTGAGGCGGTAAGTTTGCTAGTCAAGGAGTAAAACGACGAAGATTAGCATTTACTTCCGCCCATGCGA

TAGCTGTCCGTGATTGACAAGTGCTAGCACGCAGACAGAACGGAGATAGCGAACCGCTGAGTGTGTCGCTCT

GCTCGTAAAAGCTTAGAAACCTTTGAACGAAAGGGATAATGAAAGCCTTGATTGCAAGGCTTTTTGCTTTAT

GGTGGGTAAGTATCAGAGTGAGAAAATTTTTGGAATGAGTAGAAGTGATAGCTAGAAATTATCAGTTTCTA

TTTCCATTTACCCTGTGGGTACGTGTTTGTTTCCATTGACAAGGAGTTTGTGGGAATAGAAATGT

ACCCACCTTGTTTGAATCAAGTGAAGTGTAGTTGAAGGAAATCTGTTGAAAGCAATACTTCATTTTACCG

AATAAGTAATAATTTAGGCAACTTCAAATCGATTAAAAAAAACTATTTTAAAGGTTAAGAGTAGACAAAA

ATTGTCCACTCTTTTTTGCAAACTCAATTTATCAATAAATGAAATGAGGGAATGTAAAATG

AAATATTTTGAGGTTGAGTTAGAAAATCCTGATGAATTTTTAAAACTACAAACAGAAGATTTTGTGAAAG

CTAATCGCTTGCTACTAAGGAAGATAATCCAGAGCGTTACAGTCTATGAAGAAAACTTCGTCATATCCTT

TAAATCTGGCATCGAATTGGAAGTATGAGTCTCATTCCATAACTTTTATATTGAACATATCATCTTGTTGTG

TTATACTATAAATTGATATAAACAAAGATGTAGGAGGAACCGAAACTATGACAGCCTCAATGCGTTTAAGAT

AAGCTGGCAATAAAAAAAGCAGAATCTATACCCGATGATAGGCTTTTTTGTTGTGCTTATTTATACGATATT

GAGCATTCATTAGTTACGGTGAGGATATTGGTTATTTAACTATACCTTTATTTAACTATGTCTTTAATATGA

ATGTTTCCAAATTGTATGTATGCAGACCAAAAGCCACATTGTGGGGTTTGGCCTGCATTTTTTATTGCCTA

GAATGCTATTCAAAATAGAAATTCAAGCAAAATAATATGCAGGAGATAATATAAATGGAAAAATACAACAAT

TGGAAACGAAAATTTTATGCAATATGGGCAGGGCAAGCAGTATCATTAATCACTAGTGCCATCCTGCAAATG

GCGATTATTTTTTACCTTACAGAAAAAACAGGATCTGCGATGGTCTTGTCTATGGCTTCATTAGTAGGTTTT

TTACCCTATGCGATTTTGGGACCTGCCATTGGTGTGCTAGTGGATCGTCATGATAGGAAGAAGATAATGATT

GGTGCCGATTTAATTATCGCAGCAGCTGGTGCAGTGCTTGCTATTGTTGCATTCTGTATGGAGCTACCTGTC

TGGATGATTATGATAGTATTGTTTATCCGTAGCATTGGAACAGCTTTTCATACCCCAGCACTCAATGCGGTT

ACACCACTTTTAGTACCAGAAGAACAGCTAACGAAATGCGCAGGCTATAGTCAGTCTTTGCAGTCTATAAGC

TATATTGTTAGTCCGGCAGTTGCAGCACTCTTATACTCCGTTTGGGATTTAAATGCTATTATTGCCATCGAC

GTATTGGGTGCTGTGATTGCATCTATTACGGTAGCAATTGTACGTATACCTAAGCTGGGTAATCAAGTGCAA

AGTTTAGAACCAAATTTCATAAGGGAGATGAAAGAAGGAGTTGTGGTTCTGAGACAAAACAAAGGATTGTTT

GCCTTATTACTCTTAGGAACACTATATACTTTTGTTTATATGCCAATCAATGCACTATTTCCTTTAATAAGC

ATGGAACACTTTAATGGAACGCCTGTGCATATTTCTATTACGGAAATTTCCTTTGCATTTGGGATGCTAGCA

GGAGGCTTATTATTAGGAAGATTAGGGGGCTTCGAAAAGCATGTATTACTAATAACAAGTTCATTTTTTATA

ATGGGGACCAGTTTAGCCGTTTCGGGAATACTTCCTCCAAATGGATTTGTAATATTCGTAGTTTGCTGTGCA

ATAATGGGGCTTTCGGTGCCATTTTATAGCGGTGTGCAAACAGCTCTTTTTCAGGAGAAAATTAAGCCTGAA

TATTTAGGACGTGTATTTTCTTTGATCGGAAGTATCATGTCACTTGCTATGCCAATTGGGTTAATTCTTTCT

GGATTCTTTGCTGATAAAATCGGTGTAAATCATTGGTTTTTACTATCAGGTATTTTAATTATTGGCATTGCT

ATAGTTTGCCAAATGATAACTGAGGTTAGAAAATTAGATTTAAAATAAACAATATTGGAGGAATATTTATGT

ATCTTATTTTCATGTAACTCTTCCTGCTAAAATCGCAGGGTTTTCCCTGCATACAAGCAAATGAAAGCATGC

GATTATAGACAGGAGGAAATGTTATGGAATTAATATT

AAAAGCAAAAGACATTCGTGTGGAATTCAAAGGACGCGATGTTTTAGATATAAATGAATTAGAAGTATATGA

TTATGACCGTATTGGTTTAGTAGGAGCAAATGGTGCTGGAAAAAGCACTTTACTCAGGGTACTTTTAGGAGA

ATTAACTCCCCCAGGATGTAAAATGAATCGTCTGGGTGAACTTGCCTATATTCCCCAGTTGGACGAAGTAAC

TCTGCAGGAGGAAAAAGATTTTGCACTTGTAGGCAAGCTAGGTGTTGAGCAATTAAATATACAGACTATGAG

CGGTGGTGAAGAAACAAGGCTTAAAATAGCACAGGCCTTATCGGCACAGGTTCATGGTATTTTAGCGGATGA

ACCTACGAGCCATTTAGACCGTGAAGGAATTGATTTTCTAATAGGACAGCTAAAATATTTTACAGGTGCACT

GTTAGTTATTAGCCATGACCGCTATTTTCTTGATGAAATAGTAGATAAAATATGGGAACTGAAAGATGGCAA

AATCACTGAGTATTGGGGAAACTATTCTGATTATCTTCGTCAGAAAGAGGAAGAACGTAAGAGCCAAGCTGC

AGAATACGAACAATTTATTGCGGAACGTGCCCGATTGGAAAGGGCTGCGGAGGAAAAGCGAAAACAGGCTCG

TAAAATAGAACAGAAGGCAAAAGGTTCTTCAAAGAAAAAAAGTACTGAAGACGGAGGGCGTTTAGCTCATCA

AAAATCAATAGGAAGTAAGGAAAAAAAGATGTATAATGCTGCTAAAACCCTAGAGCACAGGATTGCGGCCTT

AGGAAAAGTAGAAGCTCCGGAAGGCATTCGCAGAATTCGTTTCAGGCAAAGTAAAGCATTGGAGCTCCATAA

TCCATACCCTATAGTCGGTGCAGAAATTAATAAAGTATTTGGGGATAAGGCTCTGTTTGAAAATGCATCTTT

TCAAATTCCGTTAGGAGCAAAAGTGGCGTTAACTGGTGGTAATGGAATCGGAAAAACAACTTTAATCCAAAT

GATCTTAAACCATGAAGAAGGAATTTCTATTTCGCCTAAGGCAAAAATAGGTTACTTTGCACAGAATGGTTA

CAAGTACAACAGTAATCAGAATGTTATGGAGTTTATGCAGAAGGATTGTGACTACAATATATCAGAAATTCG

TTCAGTGCTAGCATCTATGGGGTTCAAACAGAACGATATTGGAAAAAGTTTATCTGTTTTAAGCGGTGGAGA

AATTATAAAATTGTTGCTTGCTAAAATGCTCATGGGTAGATATAACATCCTAATAATGGATGAACCCAGTAA

CTTCCTTGACATACCAAGTTTAGAGGCTTTGGAAATACTAATGAAGGAGTACACCGGAACTATCGTGTTTAT

CACCCACGATAAACGATTACTCGAAAATGTAGCAGATGTAGTTTATGAAATTAGAGATAAGAAAATAAATCT

GAAACATTAAATTTAAGGTAGTCGCTGGTCAGTATAGTCTGTTCTGGTTGGCGACTCCATTGTTAAAGAGTA

TAAAGACTTTAGATTTTATGAATATTAAAAATAGGAACAGTCAATTGAACTGCTCCTATTTTTCTGCTAAAT

ATATTGTAGTTTTCTTATATGTATAATGATAGATTAGCGGATTCTCATCTACGGTACTTACTTCAAATATGA

AGAAGTGATCGCGGTTATCTCTGGACTTTTCCTTATTGAGGACAAAGTAATTCTTACGTGAAGTCGCCATTG

TTTTTAGGATATCATCAGTTAGGAAGGTCAATGGAATATTCATGTTAGAGTAGCGGTAGAAGTCACGTTCAA

AATCTTGGTAGCTCTCGCTATAATAGTCCATTTGTAGGTGATTACGCTGAAACTCAAGCTGATTCATAGAGC

ACCTCCTCGACAAGTTCAATACTAATAATGTCTTTTAATTTCAAATTGATGTGACCTGTTGTAGTTTTTATC

AAAATGAAATCTTTGGTCAGACTTGGTATTGTTCCAGTGTAGGAAACACGCTTGTTTTTTTCAATCACTTGA

ATGCGTGTGCGTAGCTGCCCGGCGTATACTTGACTGAGGAGTAATAATTTCTTCTCTAGTGATAAGTCAGAC

ATGTACGTTACTTTGTTTGTATCATCAGAGAGTGCTGATGCATGTTCAGATAGGAAAAAGCCCATCCATTTT

TGCATCTTTGTATCCTGGTACTCTCTTGCTGATTGAAATGGTAAATATGAACGGTCAATCATATCAAATCCT

TTCTATGCAGAGGCAAGGGTATTTTTATCAAATTGAATCGTAAAACCTTGAATTCCCCCACCTGTGTAACAT

TCTTTAAAGCGATTGATTACCTCAGTATAGATTATCACAGATGAGCTTGTTGGCTTAATGCTAAATGTAAAT

TCCAATGGTAATCGGTTTTCAGATTTAGCATGTACTAGTCGTATCGATATTTCAGTTGTTTTGAGTTTTCTC

TGACGAAGTTTTGAAGTTGCTGTTTCAACGATTCCATGTAAAAATCTTTCAAGCATTTCAATATCATTACAT

CCTTTGCTACGGATTTCTGAAAATTGTACTGTATTTTTTTCTTGTTTCATTTTAATCCCTCCAATCCACCCG

CGGAATGACCACCGATAAGTTTACTGCGTTCAATATTTCTGGAACCTTCAGTTAGGACGGTTCCTTTTTGTA

TGGCTAAAAAACCAAACTGTTCTCTGACAACATCAATAGCTGTCTGAAGTCTATTATCTTTTTCAATTTGTT

CTACATCATCAAAGAGTGATAGTAGAGTATAGCTTTCATCTACGAAGCCACTATAAGATACACCAATTTGTC

TCACTGCACCAGAGGTGTATTTTTTCGGAATAATACAAGTACATGACTCACCATTGTTTTGGGGAGATTTG

CGGGTTCAATTTTATTCTGAGCATTTATAGATTTTTTCATCTCAGTCCTAGAATAGCCAATATGAATAGAAA

CGACAGTAGTCAATACTAGGGCTACTGTTCCGTTCCACAGTATCATTTAAAAATCATTTTCACACCCTTTC

GTCTATTAGTATAGAAGAAAGCTCTCAGCACA

>GA17371/Mega-1.V

CATGTTGAGGCGGTAAGTTTGCTAGTCAAGGAGTAAAACGACGAAGATTAGCATTTACTTCCGCCCATGCGA

TAGCTGTCCGTGATTGACAAGTGCTAGCACGCAGACAGAACGGAGATAGCGAACCGCTGAGTGTGTCGCTCT

GCTCGTAAAAGCTTAGAAACCTTTGAACGAAAGGGATAATGAAAGCCTTGATTGCAAGGCTTTTTGCTTTAT

GGTGGGTAAGTATCAGAGTGAGAAAATTTTTGGAATGAGTAGAAGTGATAGCTAGAAATTATCAGTTTCTA

TTTCCATTTACCCTGTGGGTACGTGTTTGTTTCCATTGACAAGGAGTTTGTGGGAATAGAAATGT

ACCCACCTTGTTTGAATCAAGTGAAGTGTAGTTGAAGGAAATCTGTTGAAAGCAATACTTCATTTTACCG

AATAAGTAATAATTTAGGCAACTTCAAATCGATTAAAAAAAACTATTTTAAAGGTTAAGAGTAGACAAAA

ATTGTCCACTCTTTTTTGCAAACTCAATTTATCAATAAATGAAATGAGGGAATGTAAAATG

AAATATTTTGAGGTTGAGTTAGAAAATCCTGATGAATTTTTAAAACTACAAACAGAAGATTTTGTGAAAG

CTAATCGCTTGCTACTAAGGAAGATAATCCAGAGCGTTACAGTCTATGAAGAAAACTTCGTCATATCCTT

TAAATCTGGCATCGAATTGGAAGTATGAGTCTCATTCCATAACTTTTATATTGAACATATCATCTTGTTGTG

TTATACTATAAATTGATATAAACAAAGATGTAGGAGGAACCGAAACTATGACAGCCTCAATGCGTTTAAGAT

AAGCTGGCAATAAAAAAAGCAGAATCTATACCCGATGATAGGCTTTTTTGTTGTGCTTATTTATACGATATT

GAGCATTCATTAGTTACGGTGAGGATATTGGTTATTTAACTATACCTTTATTTAACTATGTCTTTAATATGA

ATGTTTCCAAATTGTATGTATGCAGACCAAAAGCCACATTGTGGGGTTTGGCCTGCATTTTTTATTGCCTA

GAATGCTATTCAAAATAGAAATTCAAGCAAAATAATATGCAGGAGATAATATAAATGGAAAAATACAACAAT

TGGAAACGAAAATTTTATGCAATATGGGCAGGGCAAGCAGTATCATTAATCACTAGTGCCATCCTGCAAATG

GCGATTATTTTTTACCTTACAGAAAAAACAGGATCTGCGATGGTCTTGTCTATGGCTTCATTAGTAGGTTTT

TTACCCTATGCGATTTTGGGACCTGCCATTGGTGTGCTAGTGGATCGTCATGATAGGAAGAAGATAATGATT

GGTGCCGATTTAATTATCGCAGCAGCTGGTGCAGTGCTTGCTATTGTTGCATTCTGTATGGAGCTACCTGTC

TGGATGATTATGATAGTATTGTTTATCCGTAGCATTGGAACAGCTTTTCATACCCCAGCACTCAATGCGGTT

ACACCACTTTTAGTACCAGAAGAACAGCTAACGAAATGCGCAGGCTATAGTCAGTCTTTGCAGTCTATAAGC

TATATTGTTAGTCCGGCAGTTGCAGCACTCTTATACTCCGTTTGGGATTTAAATGCTATTATTGCCATCGAC

GTATTGGGTGCTGTGATTGCATCTATTACGGTAGCAATTGTACGTATACCTAAGCTGGGTAATCAAGTGCAA

AGTTTAGAACCAAATTTCATAAGGGAGATGAAAGAAGGAGTTGTGGTTCTGAGACAAAACAAAGGATTGTTT

GCCTTATTACTCTTAGGAACACTATATACTTTTGTTTATATGCCAATCAATGCACTATTTCCTTTAATAAGC

ATGGAACACTTTAATGGAACGCCTGTGCATATTTCTATTACGGAAATTTCCTTTGCATTTGGGATGCTAGCA

GGAGGCTTATTATTAGGAAGATTAGGGGGCTTCGAAAAGCATGTATTACTAATAACAAGTTCATTTTTTATA

ATGGGGACCAGTTTAGCCGTTTCGGGAATACTTCCTCCAAATGGATTTGTAATATTCGTAGTTTGCTGTGCA

ATAATGGGGCTTTCGGTGCCATTTTATAGCGGTGTGCAAACAGCTCTTTTTCAGGAGAAAATTAAGCCTGAA

TATTTAGGACGTGTATTTTCTTTGATCGGAAGTATCATGTCACTTGCTATGCCAATTGGGTTAATTCTTTCT

GGATTCTTTGCTGATAAAATCGGTGTAAATCATTGGTTTTTACTATCAGGTATTTTAATTATTGGCATTGCT

ATAGTTTGCCAAATGATAACTGAGGTTAGAAAATTAGATTTAAAATAAACAATATTGGAGGAATATTTATGT

ATCTTATTTTCATGTAACTCTTCCTGCTAAAATCGCAGGGTTTTCCCTGCATACAAGCAAATGAAAGCATGC

GATTATAGACAGGAGGAAATGTTATGGAATTAATATT

AAAAGCAAAAGACATTCGTGTGGAATTCAAAGGACGCGATGTTTTAGATATAAATGAATTAGAAGTATATGA

TTATGACCGTATTGGTTTAGTAGGAGCAAATGGTGCTGGAAAAAGCACTTTACTCAGGGTACTTTTAGGAGA

ATTAACTCCCCCAGGATGTAAAATGAATCGTCTGGGTGAACTTGCCTATATTCCCCAGTTGGACGAAGTAAC

TCTGCAGGAGGAAAAAGATTTTGCACTTGTAGGCAAGCTAGGTGTTGAGCAATTAAATATACAGACTATGAG

CGGTGGTGAAGAAACAAGGCTTAAAATAGCACAGGCCTTATCGGCACAGGTTCATGGTATTTTAGCGGATGA

ACCTACGAGCCATTTAGACCGTGAAGGAATTGATTTTCTAATAGGACAGCTAAAATATTTTACAGGTGCACT

GTTAGTTATTAGCCATGACCGCTATTTTCTTGATGAAATAGTAGATAAAATATGGGAACTGAAAGATGGCAA

AATCACTGAGTATTGGGGAAACTATTCTGATTATCTTCGTCAGAAAGAGGAAGAACGTAAGAGCCAAGCTGC

AGAATACGAACAATTTATTGCGGAACGTGCCCGATTGGAAAGGGCTGCGGAGGAAAAGCGAAAACAGGCTCG

TAAAATAGAACAGAAGGCAAAAGGTTCTTCAAAGAAAAAAAGTACTGAAGACGGAGGGCGTTTAGCTCATCA

AAAATCAATAGGAAGTAAGGAAAAAAAGATGTATAATGCTGCTAAAACCCTAGAGCACAGGATTGCGGCCTT

AGGAAAAGTAGAAGCTCCGGAAGGCATTCGCAGAATTCGTTTCAGGCAAAGTAAAGCATTGGAGCTCCATAA

TCCATACCCTATAGTCGGTGCAGAAATTAATAAAGTATTTGGGGATAAGGCTCTGTTTGAAAATGCATCTTT

TCAAATTCCGTTAGGAGCAAAAGTGGCGTTAACTGGTGGTAATGGAATCGGAAAAACAACTTTAATCCAAAT

GATCTTAAACCATGAAGAAGGAATTTCTATTTCGCCTAAGGCAAAAATAGGTTACTTTGCACAGAATGGTTA

CAAGTACAACAGTAATCAGAATGTTATGGAGTTTATGCAGAAGGATTGTGACTACAATATATCAGAAATTCG

TTCAGTGCTAGCATCTATGGGGTTCAAACAGAACGATATTGGAAAAAGTTTATCTGTTTTAAGCGGTGGAGA

AATTATAAAATTGTTGCTTGCTAAAATGCTCATGGGTAGATATAACATCCTAATAATGGATGAACCCAGTAA

CTTCCTTGACATACCAAGTTTAGAGGCTTTGGAAATACTAATGAAGGAGTACACCGGAACTATCGTGTTTAT

CACCCACGATAAACGATTACTCGAAAATGTAGCAGATGTAGTTTATGAAATTAGAGATAAGAAAATAAATCT

GAAACATTAAATTTAAGGTAGTCGCTGGTCAGTATAGTCTGTTCTGGTTGGCGACTCCATTGTTAAAGAGTA

TAAAGACTTTAGATTTTATGAATATTAAAAATAGGAACAGTCAATTGAACTGCTCCTATTTTTCTGCTAAAT

ATATTGTAGTTTTCTTATATGTATAATGATAGATTAGCGGATTCTCATCTACGGTACTTACTTCAAATATGA

AGAAGTGATCGCGGTTATCTCTGGACTTTTCCTTATTGAGGACAAAGTAATTCTTACGTGAAGTCGCCATTG

TTTTTAGGATATCATCAGTTAGGAAGGTCAATGGAATATTCATGTTAGAGTAGCGGTAGAAGTCACGTTCAA

AATCTTGGTAGCTCTCGCTATAATAGTCCATTTGTAGGTGATTACGCTGAAACTCAAGCTGATTCATAGAGC

ACCTCCTCGACAAGTTCAATACTAATAATGTCTTTTAATTTCAAATTGATGTGACCTGTTGTAGTTTTTATC

AAAATGAAATCTTTGGTCAGACTTGGTATTGTTCCAGTGTAGGAAACACGCTTGTTTTTTTCAATCACTTGA

ATGCGTGTGCGTAGCTGCCCGGCGTATACTTGACTGAGGAGTAATAATTTCTTCTCTAGTGATAAGTCAGAC

ATGTACGTTACTTTGTTTGTATCATCAGAGAGTGCTGATGCATGTTCAGATAGGAAAAAGCCCATCCATTTT

TGCATCTTTGTATCCTGGTACTCTCTTGCTGATTGAAATGGTAAATATGAACGGTCAATCATATCAAATCCT

TTCTATGCAGAGGCAAGGGTATTTTTATCAAATTGAATCGTAAAACCTTGAATTCCCCCACCTGTGTAACAT

TCTTTAAAGCGATTGATTACCTCAGTATAGATTATCACAGATGAGCTTGTTGGCTTAATGCTAAATGTAAAT

TCCAATGGTAATCGGTTTTCAGATTTAGCATGTACTAGTCGTATCGATATTTCAGTTGTTTTGAGTTTTCTC

TGACGAAGTTTTGAAGTTGCTGTTTCAACGATTCCATGTAAAAATCTTTCAAGCATTTCAATATCATTACAT

CCTTTGCTACGGATTTCTGAAAATTGTACTGTATTTTTTTCTTGTTTCATTTTAATCCCTCCAATCCACCCG

CGGAATGACCACCGATAAGTTTACTGCGTTCAATATTTCTGGAACCTTCAGTTAGGACGGTTCCTTTTTGTA

TGGCTAAAAAACCAAACTGTTCTCTGACAACATCAATAGCTGTCTGAAGTCTATTATCTTTTTCAATTTGTT

CTACATCATCAAAGAGTGATAGTAGAGTATAGCTTTCATCTACGAAGCCACTATAAGATACACCAATTTGTC

TCACTGCACCAGAGGTGTATTTTTTCGGAATAATACAAGTACATGACTCACCATTGTTTTGGGGAGATTTG

CGGGTTCAATTTTATTCTGAGCATTTATAGATTTTTTCATCTCAGTCCTAGAATAGCCAATATGAATAGAAA

CGACAGTAGTCAATACTAGGGCTACTGTTCCGTTCCACAGTATCATTTAAAAATCATTTTCACACCCTTTC

GTCTATTAGTATAGAAGAAAGCTCTCAGCACA

>GA47778/Mega-1.V

CATGTTGAGGCGGTAAGTTTGCTAGTCAAGGAGTAAAACGACGAAGATTAGCATTTACTTCCGCCCATGCGA

TAGCTGTCCGTGATTGACAAGTGCTAGCACGCAGACAGAACGGAGATAGCGAACCGCTGAGTGTGTCGCTCT

GCTCGTAAAAGCTTAGAAACCTTTGAACGAAAGGGATAATGAAAGCCTTGATTGCAAGGCTTTTTGCTTTAT

GGTGGGTAAGTATCAGAGTGAGAAAATTTTTGGAATGAGTAGAAGTGATAGCTAGAAATTATCAGTTTCTA

TTTCCATTTACCCTGTGGGTACGTGTTTGTTTCCATTGACAAGGAGTTTGTGGGAATAGAAATGT

ACCCACCTTGTTTGAATCAAGTGAAGTGTAGTTGAAGGAAATCTGTTGAAAGCAATACTTCATTTTACCG

AATAAGTAATAATTTAGGCAACTTCAAATCGATTAAAAAAAACTATTTTAAAGGTTAAGAGTAGACAAAA

ATTGTCCACTCTTTTTTGCAAACTCAATTTATCAATAAATGAAATGAGGGAATGTAAAATG

AAATATTTTGAGGTTGAGTTAGAAAATCCTGATGAATTTTTAAAACTACAAACAGAAGATTTTGTGAAAG

CTAATCGCTTGCTACTAAGGAAGATAATCCAGAGCGTTACAGTCTATGAAGAAAACTTCGTCATATCCTT

TAAATCTGGCATCGAATTGGAAGTATGAGTCTCATTCCATAACTTTTATATTGAACATATCATCTTGTTGTG

TTATACTATAAATTGATATAAACAAAGATGTAGGAGGAACCGAAACTATGACAGCCTCAATGCGTTTAAGAT

AAGCTGGCAATAAAAAAAGCAGAATCTATACCCGATGATAGGCTTTTTTGTTGTGCTTATTTATACGATATT

GAGCATTCATTAGTTACGGTGAGGATATTGGTTATTTAACTATACCTTTATTTAACTATGTCTTTAATATGA

ATGTTTCCAAATTGTATGTATGCAGACCAAAAGCCACATTGTGGGGTTTGGCCTGCATTTTTTATTGCCTA

GAATGCTATTCAAAATAGAAATTCAAGCAAAATAATATGCAGGAGATAATATAAATGGAAAAATACAACAAT

TGGAAACGAAAATTTTATGCAATATGGGCAGGGCAAGCAGTATCATTAATCACTAGTGCCATCCTGCAAATG

GCGATTATTTTTTACCTTACAGAAAAAACAGGATCTGCGATGGTCTTGTCTATGGCTTCATTAGTAGGTTTT

TTACCCTATGCGATTTTGGGACCTGCCATTGGTGTGCTAGTGGATCGTCATGATAGGAAGAAGATAATGATT

GGTGCCGATTTAATTATCGCAGCAGCTGGTGCAGTGCTTGCTATTGTTGCATTCTGTATGGAGCTACCTGTC

TGGATGATTATGATAGTATTGTTTATCCGTAGCATTGGAACAGCTTTTCATACCCCAGCACTCAATGCGGTT

ACACCACTTTTAGTACCAGAAGAACAGCTAACGAAATGCGCAGGCTATAGTCAGTCTTTGCAGTCTATAAGC

TATATTGTTAGTCCGGCAGTTGCAGCACTCTTATACTCCGTTTGGGATTTAAATGCTATTATTGCCATCGAC

GTATTGGGTGCTGTGATTGCATCTATTACGGTAGCAATTGTACGTATACCTAAGCTGGGTAATCAAGTGCAA

AGTTTAGAACCAAATTTCATAAGGGAGATGAAAGAAGGAGTTGTGGTTCTGAGACAAAACAAAGGATTGTTT

GCCTTATTACTCTTAGGAACACTATATACTTTTGTTTATATGCCAATCAATGCACTATTTCCTTTAATAAGC

ATGGAACACTTTAATGGAACGCCTGTGCATATTTCTATTACGGAAATTTCCTTTGCATTTGGGATGCTAGCA

GGAGGCTTATTATTAGGAAGATTAGGGGGCTTCGAAAAGCATGTATTACTAATAACAAGTTCATTTTTTATA

ATGGGGACCAGTTTAGCCGTTTCGGGAATACTTCCTCCAAATGGATTTGTAATATTCGTAGTTTGCTGTGCA

ATAATGGGGCTTTCGGTGCCATTTTATAGCGGTGTGCAAACAGCTCTTTTTCAGGAGAAAATTAAGCCTGAA

TATTTAGGACGTGTATTTTCTTTGATCGGAAGTATCATGTCACTTGCTATGCCAATTGGGTTAATTCTTTCT

GGATTCTTTGCTGATAAAATCGGTGTAAATCATTGGTTTTTACTATCAGGTATTTTAATTATTGGCATTGCT

ATAGTTTGCCAAATGATAACTGAGGTTAGAAAATTAGATTTAAAATAAACAATATTGGAGGAATATTTATGT

ATCTTATTTTCATGTAACTCTTCCTGCTAAAATCGCAGGGTTTTCCCTGCATACAAGCAAATGAAAGCATGC

GATTATAGACAGGAGGAAATGTTATGGAATTAATATT

AAAAGCAAAAGACATTCGTGTGGAATTCAAAGGACGCGATGTTTTAGATATAAATGAATTAGAAGTATATGA

TTATGACCGTATTGGTTTAGTAGGAGCAAATGGTGCTGGAAAAAGCACTTTACTCAGGGTACTTTTAGGAGA

ATTAACTCCCCCAGGATGTAAAATGAATCGTCTGGGTGAACTTGCCTATATTCCCCAGTTGGACGAAGTAAC

TCTGCAGGAGGAAAAAGATTTTGCACTTGTAGGCAAGCTAGGTGTTGAGCAATTAAATATACAGACTATGAG

CGGTGGTGAAGAAACAAGGCTTAAAATAGCACAGGCCTTATCGGCACAGGTTCATGGTATTTTAGCGGATGA

ACCTACGAGCCATTTAGACCGTGAAGGAATTGATTTTCTAATAGGACAGCTAAAATATTTTACAGGTGCACT

GTTAGTTATTAGCCATGACCGCTATTTTCTTGATGAAATAGTAGATAAAATATGGGAACTGAAAGATGGCAA

AATCACTGAGTATTGGGGAAACTATTCTGATTATCTTCGTCAGAAAGAGGAAGAACGTAAGAGCCAAGCTGC

AGAATACGAACAATTTATTGCGGAACGTGCCCGATTGGAAAGGGCTGCGGAGGAAAAGCGAAAACAGGCTCG

TAAAATAGAACAGAAGGCAAAAGGTTCTTCAAAGAAAAAAAGTACTGAAGACGGAGGGCGTTTAGCTCATCA

AAAATCAATAGGAAGTAAGGAAAAAAAGATGTATAATGCTGCTAAAACCCTAGAGCACAGGATTGCGGCCTT

AGGAAAAGTAGAAGCTCCGGAAGGCATTCGCAGAATTCGTTTCAGGCAAAGTAAAGCATTGGAGCTCCATAA

TCCATACCCTATAGTCGGTGCAGAAATTAATAAAGTATTTGGGGATAAGGCTCTGTTTGAAAATGCATCTTT

TCAAATTCCGTTAGGAGCAAAAGTGGCGTTAACTGGTGGTAATGGAATCGGAAAAACAACTTTAATCCAAAT

GATCTTAAACCATGAAGAAGGAATTTCTATTTCGCCTAAGGCAAAAATAGGTTACTTTGCACAGAATGGTTA

CAAGTACAACAGTAATCAGAATGTTATGGAGTTTATGCAGAAGGATTGTGACTACAATATATCAGAAATTCG

TTCAGTGCTAGCATCTATGGGGTTCAAACAGAACGATATTGGAAAAAGTTTATCTGTTTTAAGCGGTGGAGA

AATTATAAAATTGTTGCTTGCTAAAATGCTCATGGGTAGATATAACATCCTAATAATGGATGAACCCAGTAA

CTTCCTTGACATACCAAGTTTAGAGGCTTTGGAAATACTAATGAAGGAGTACACCGGAACTATCGTGTTTAT

CACCCACGATAAACGATTACTCGAAAATGTAGCAGATGTAGTTTATGAAATTAGAGATAAGAAAATAAATCT

GAAACATTAAATTTAAGGTAGTCGCTGGTCAGTATAGTCTGTTCTGGTTGGCGACTCCATTGTTAAAGAGTA

TAAAGACTTTAGATTTTATGAATATTAAAAATAGGAACAGTCAATTGAACTGCTCCTATTTTTCTGCTAAAT

ATATTGTAGTTTTCTTATATGTATAATGATAGATTAGCGGATTCTCATCTACGGTACTTACTTCAAATATGA

AGAAGTGATCGCGGTTATCTCTGGACTTTTCCTTATTGAGGACAAAGTAATTCTTACGTGAAGTCGCCATTG

TTTTTAGGATATCATCAGTTAGGAAGGTCAATGGAATATTCATGTTAGAGTAGCGGTAGAAGTCACGTTCAA

AATCTTGGTAGCTCTCGCTATAATAGTCCATTTGTAGGTGATTACGCTGAAACTCAAGCTGATTCATAGAGC

ACCTCCTCGACAAGTTCAATACTAATAATGTCTTTTAATTTCAAATTGATGTGACCTGTTGTAGTTTTTATC

AAAATGAAATCTTTGGTCAGACTTGGTATTGTTCCAGTGTAGGAAACACGCTTGTTTTTTTCAATCACTTGA

ATGCGTGTGCGTAGCTGCCCGGCGTATACTTGACTGAGGAGTAATAATTTCTTCTCTAGTGATAAGTCAGAC

ATGTACGTTACTTTGTTTGTATCATCAGAGAGTGCTGATGCATGTTCAGATAGGAAAAAGCCCATCCATTTT

TGCATCTTTGTATCCTGGTACTCTCTTGCTGATTGAAATGGTAAATATGAACGGTCAATCATATCAAATCCT

TTCTATGCAGAGGCAAGGGTATTTTTATCAAATTGAATCGTAAAACCTTGAATTCCCCCACCTGTGTAACAT

TCTTTAAAGCGATTGATTACCTCAGTATAGATTATCACAGATGAGCTTGTTGGCTTAATGCTAAATGTAAAT

TCCAATGGTAATCGGTTTTCAGATTTAGCATGTACTAGTCGTATCGATATTTCAGTTGTTTTGAGTTTTCTC

TGACGAAGTTTTGAAGTTGCTGTTTCAACGATTCCATGTAAAAATCTTTCAAGCATTTCAATATCATTACAT

CCTTTGCTACGGATTTCTGAAAATTGTACTGTATTTTTTTCTTGTTTCATTTTAATCCCTCCAATCCACCCG

CGGAATGACCACCGATAAGTTTACTGCGTTCAATATTTCTGGAACCTTCAGTTAGGACGGTTCCTTTTTGTA

TGGCTAAAAAACCAAACTGTTCTCTGACAACATCAATAGCTGTCTGAAGTCTATTATCTTTTTCAATTTGTT

CTACATCATCAAAGAGTGATAGTAGAGTATAGCTTTCATCTACGAAGCCACTATAAGATACACCAATTTGTC

TCACTGCACCAGAGGTGTATTTTTTCGGAATAATACAAGTACATGACTCACCATTGTTTTGGGGAGATTTG

CGGGTTCAATTTTATTCTGAGCATTTATAGATTTTTTCATCTCAGTCCTAGAATAGCCAATATGAATAGAAA

CGACAGTAGTCAATACTAGGGCTACTGTTCCGTTCCACAGTATCATTTAAAAATCATTTTCACACCCTTTC

GTCTATTAGTATAGAAGAAAGCTCTCAGCACA

>8190-05/Mega-1.V

CATGTTGAGGCGGTAAGTTTGCTAGTCAAGGAGTAAAACGACGAAGATTAGCATTTACTTCCGCCCATGCGA

TAGCTGTCCGTGATTGACAAGTGCTAGCACGCAGACAGAACGGAGATAGCGAACCGCTGAGTGTGTCGCTCT

GCTCGTAAAAGCTTAGAAACCTTTGAACGAAAGGGATAATGAAAGCCTTGATTGCAAGGCTTTTTGCTTTAT

GGTGGGTAAGTATCAGAGTGAGAAAATTTTTGGAATGAGTAGAAGTGATAGCTAGAAATTATCAGTTTCTA

TTTCCATTTACCCTGTGGGTACGTGTTTGTTTCCATTGACAAGGAGTTTGTGGGAATAGAAATGT

ACCCACCTTGTTTGAATCAAGTGAAGTGTAGTTGAAGGAAATCTGTTGAAAGCAATACTTCATTTTACCG

AATAAGTAATAATTTAGGCAACTTCAAATCGATTAAAAAAAACTATTTTAAAGGTTAAGAGTAGACAAAA

ATTGTCCACTCTTTTTTGCAAACTCAATTTATCAATAAATGAAATGAGGGAATGTAAAATG

AAATATTTTGAGGTTGAGTTAGAAAATCCTGATGAATTTTTAAAACTACAAACAGAAGATTTTGTGAAAG

CTAATCGCTTGCTACTAAGGAAGATAATCCAGAGCGTTACAGTCTATGAAGAAAACTTCGTCATATCCTT

TAAATCTGGCATCGAATTGGAAGTATGAGTCTCATTCCATAACTTTTATATTGAACATATCATCTTGTTGTG

TTATACTATAAATTGATATAAACAAAGATGTAGGAGGAACCGAAACTATGACAGCCTCAATGCGTTTAAGAT

AAGCTGGCAATAAAAAAAGCAGAATCTATACCCGATGATAGGCTTTTTTGTTGTGCTTATTTATACGATATT

GAGCATTCATTAGTTACGGTGAGGATATTGGTTATTTAACTATACCTTTGTTTAACTATGTCTTTAATATGA

ATGTTTCCAAATTGTATGTATGCAGACCAAAAGCCACATTGTGGGGTTTGGCCTGCATTTTTTTTGCCTA

GAATGCTATTCAAAATAGAAATTCAAGCAAAATAATATGCAGGAGATAATATAAATGGAAAAATACAACAAT

TGGAAACGAAAATTTTATGCAATATGGGCAGGGCAAGCAGTATCATTAATCACTAGTGCCATCCTGCAAATG

GCGATTATTTTTTACCTTACAGAAAAAACAGGATCTGCGATGGTCTTGTCTATGGCTTCATTAGTAGGTTTT

TTACCCTATGCGATTTTGGGACCTGCCATTGGTGTGCTAGTGGATCGTCATGATAGGAAGAAGATAATGATT

GGTGCCGATTTAATTATCGCAGCAGCTGGTGCAGTGCTTGCTATTGTTGCATTCTGTATGGAGCTACCTGTC

TGGATGATTATGATAGTATTGTTTATCCGTAGCATTGGAACAGCTTTTCATACCCCAGCACTCAATGCGGTT

ACACCACTTTTAGTACCAGAAGAACAGCTAACGAAATGCGCAGGCTATAGTCAGTCTTTGCAGTCTATAAGC

TATATTGTTAGTCCGGCAGTTGCAGCACTCTTATACTCCGTTTGGGATTTAAATGCTATTATTGCCATCGAC

GTATTGGGTGCTGTGATTGCATCTATTACGGTAGCAATTGTACGTATACCTAAGCTGGGTAATCAAGTGCAA

AGTTTAGAACCAAATTTCATAAGGGAGATGAAAGAAGGAGTTGTGGTTCTGAGACAAAACAAAGGATTGTTT

GCCTTATTACTCTTAGGAACACTATATACTTTTGTTTATATGCCAATCAATGCACTATTTCCTTTAATAAGC

ATGGAACACTTTAATGGAACGCCTGTGCATATTTCTATTACGGAAATTTCCTTTGCATTTGGGATGCTAGCA

GGAGGCTTATTATTAGGAAGATTAGGGGGCTTCGAAAAGCATGTATTACTAATAACAAGTTCATTTTTTATA

ATGGGGACCAGTTTAGCCGTTTCGGGAATACTTCCTCCAAATGGATTTGTAATATTCGTAGTTTGCTGTGCA

ATAATGGGGCTTTCGGTGCCATTTTATAGCGGTGTGCAAACAGCTCTTTTTCAGGAGAAAATTAAGCCTGAA

TATTTAGGACGTGTATTTTCTTTGATCGGAAGTATCATGTCACTTGCTATGCCAATTGGGTTAATTCTTTCT

GGATTCTTTGCTGATAAAATCGGTGTAAATCATTGGTTTTTACTATCAGGTATTTTAATTATTGGCATTGCT

ATAGTTTGCCAAATGATAACTGAGGTTAGAAAATTAGATTTAAAATAAACAATATTGGAGGAATATTTATGT

ATCTTATTTTCATGTAACTCTTCCTGCTAAAATCGCAGGGTTTTCCCTGCATACAAGCAAATGAAAGCATGC

GATTATAGACAGGAGGAAATGTTATGGAATTAATATT

AAAAGCAAAAGACATTCGTGTGGAATTCAAAGGACGCGATGTTTTAGATATAAATGAATTAGAAGTATATGA

TTATGACCGTATTGGTTTAGTAGGAGCAAATGGTGCTGGAAAAAGCACTTTACTCAGGGTACTTTTAGGAGA

ATTAACTCCCCCAGGATGTAAAATGAATCGTCTGGGTGAACTTGCCTATATTCCCCAGTTGGACGAAGTAAC

TCTGCAGGAGGAAAAAGATTTTGCACTTGTAGGCAAGCTAGGTGTTGAGCAATTAAATATACAGACTATGAG

CGGTGGTGAAGAAACAAGGCTTAAAATAGCACAGGCCTTATCGGCACAGGTTCATGGTATTTTAGCGGATGA

ACCTACGAGCCATTTAGACCGTGAAGGAATTGATTTTCTAATAGGACAGCTAAAATATTTTACAGGTGCACT

GTTAGTTATTAGCCATGACCGCTATTTTCTTGATGAAATAGTAGATAAAATATGGGAACTGAAAGATGGCAA

AATCACTGAGTATTGGGGAAACTATTCTGATTATCTTCGTCAGAAAGAGGAAGAACGTAAGAGCCAAGCTGC

AGAATACGAACAATTTATTGCGGAACGTGCCCGATTGGAAAGGGCTGCGGAGGAAAAGCGAAAACAGGCTCG

TAAAATAGAACAGAAGGCAAAAGGTTCTTCAAAGAAAAAAAGTACTGAAGACGGAGGGCGTTTAGCTCATCA

AAAATCAATAGGAAGTAAGGAAAAAAAGATGTATAATGCTGCTAAAACCCTAGAGCACAGGATTGCGGCCTT

AGGAAAAGTAGAAGCTCCGGAAGGCATTCGCAGAATTCGTTTCAGGCAAAGTAAAGCATTGGAGCTCCATAA

TCCATACCCTATAGTCGGTGCAGAAATTAATAAAGTATTTGGGGATAAGGCTCTGTTTGAAAATGCATCTTT

TCAAATTCCGTTAGGAGCAAAAGTGGCGTTAACTGGTGGTAATGGAATCGGAAAAACAACTTTAATCCAAAT

GATCTTAAACCATGAAGAAGGAATTTCTATTTCGCCTAAGGCAAAAATAGGTTACTTTGCACAGAATGGTTA

CAAGTACAACAGTAATCAGAATGTTATGGAGTTTATGCAGAAGGATTGTGACTACAATATATCAGAAATTCG

TTCAGTGCTAGCATCTATGGGGTTCAAACAGAACGATATTGGAAAAAGTTTATCTGTTTTAAGCGGTGGAGA

AATTATAAAATTGTTGCTTGCTAAAATGCTCATGGGTAGATATAACATCCTAATAATGGATGAACCCAGTAA

CTTCCTTGACATACCAAGTTTAGAGGCTTTGGAAATACTAATGAAGGAGTACACCGGAACTATCGTGTTTAT

CACCCACGATAAACGATTACTCGAAAATGTAGCAGATGTAGTTTATGAAATTAGAGATAAGAAAATAAATCT

GAAACATTAAATTTAAGGTAGTCGCTGGTCAGTATAGTCTGTTCTGGTTGGCGACTCCATTGTTAAAGAGTA

TAAAGACTTTAGATTTTATGAATATTAAAAATAGGAACAGTCAATTGAACTGCTCCTATTTTTCTGCTAAAT

ATATTGTAGTTTTCTTATATGTATAATGATAGATTAGCGGATTCTCATCTACGGTACTTACTTCAAATATGA

AGAAGTGATCGCGGTTATCTCTGGACTTTTCCTTATTGAGGACAAAGTAATTCTTACGTGAAGTCGCCATTG

TTTTTAGGATATCATCAGTTAGGAAGGTCAATGGAATATTCATGTTAGAGTAGCGGTAGAAGTCACGTTCAA

AATCTTGGTAGCTCTCGCTATAATAGTCCATTTGTAGGTGATTACGCTGAAACTCAAGCTGATTCATAGAGC

ACCTCCTCGACAAGTTCAATACTAATAATGTCTTTTAATTTCAAATTGATGTGACCTGTTGTAGTTTTTATC

AAAATGAAATCTTTGGTCAGACTTGGTATTGTTCCAGTGTAGGAAACACGCTTGTTTTTTTCAATCACTTGA

ATGCGTGTGCGTAGCTGCCCGGCGTATACTTGACTGAGGAGTAATAATTTCTTCTCTAGTGATAAGTCAGAC

ATGTACGTTACTTTGTTTGTATCATCAGAGAGTGCTGATGCATGTTCAGATAGGAAAAAGCCCATCCATTTT

TGCATCTTTGTATCCTGGTACTCTCTTGCTGATTGAAATGGTAAATATGAACGGTCAATCATATCAAATCCT

TTCTATGCAGAGGCAAGGGTATTTTTATCAAATTGAATCGTAAAACCTTGAATTCCCCCACCTGTGTAACAT

TCTTTAAAGCGATTGATTACCTCAGTATAGATTATCACAGATGAGCTTGTTGGCTTAATGCTAAATGTAAAT

TCCAATGGTAATCGGTTTTCAGATTTAGCATGTACTAGTCGTATCGATATTTCAGTTGTTTTGAGTTTTCTC

TGACGAAGTTTTGAAGTTGCTGTTTCAACGATTCCATGTAAAAATCTTTCAAGCATTTCAATATCATTACAT

CCTTTGCTACGGATTTCTGAAAATTGTACTGTATTTTTTTCTTGTTTCATTTTAATCCCTCCAATCCACCCG

CGGAATGACCACCGATAAGTTTACTGCGTTCAATATTTCTGGAACCTTCAGTTAGGACGGTTCCTTTTTGTA

TGGCTAAAAAACCAAACTGTTCTCTGACAACATCAATAGCTGTCTGAAGTCTATTATCTTTTTCAATTTGTT

CTACATCATCAAAGAGTGATAGTAGAGTATAGCTTTCATCTACGAAGCCACTATAAGATACACCAATTTGTC

TCACTGCACCAGAGGTGTATTTTTTCGGAATAATACAAGTACATGACTCACCATTGTTTTGGGGAGATTTG

CGGGTTCAATTTTATTCTGAGCATTTATAGATTTTTTCATCTCAGTCCTAGAATAGCCAATATGAATAGAAA

CGACAGTAGTCAATACTAGGGCTACTGTTCCGTTCCACAGTATCATTTAAAAATCATT-TTCACACCCTTTC

GTCTATTAGTATAGAAGAAAGCTCTCAGCACA

>GA41301/Mega-1.V

CATGTTGAGGCGGTAAGTTTGCTAGTCAAGGAGTAAAACGACGAAGATTAGCATTTACTTCCGCCCATGCGA

TAGCTGTCCGTGATTGACAAGTGCTAGCACGCAGACAGAACGGAGATAGCGAACCGCTGAGTGTGTCGCTCT

GCTCGTAAAAGCTTAGAAACCTTTGAACGAAAGGGATAATGAAAGCCTTGATTGCAAGGCTTTTTGCTTTAT

GGTGGGTAAGTATCAGAGTGAGAAAATTTTTGGAATGAGTAGAAGTGATAGCTAGAAATTATCAGTTTTTA

TTTCCATTTACCCTGTGGGTACGTGTTTGTTTCCATTGACAAGGAGTTTGTGGGAATAGAAATGT

ACCCACCTTGTTTGAATCAAGTGAAGTGTAGTTGAAGGAAATCTGTTGAAAGCAATACTTCATTTTACCG

AATAAGTAATAATTTAGGCAACTTCAAATCGATTAAAAAAAACTATTTTAAAGGTTAAGAGTAGACAAAA

ATTGTCCACTCTTTTTTGCAAACTCAATTTATCAATAAATGAAATGAGGGAATGTAAAATG

AAATATTTTGAGGTTGAGTTAGAAAATCCTGATGAATTTTTAAAACTACAAACAGAAGATTTTGTGAAAG

CTAATCGCTTGCTACTAAGGAAGATAATCCAGAGCGTTACAGTCTATGAAGAAAACTTCGTCATATCCTT

TAAATCTGGCATCGAATTGGAAGTATGAGTCTCATTCCATAACTTTTATATTGAACATATCATCTTGTTGTG

TTATACTATAAATTGATATAAACAAAGATGTAGGAGGAACCGAAACTATGACAGCCTCAATGCGTTTAAGAT

AAGCTGGCAATAAAAAAAGCAGAATCTATACCCGATGATAGGCTTTTTTGTTGTGCTTATTTATACGATATT

GAGCATTCATTAGTTACGGTGAGGATATTGGTTATTTAACTATACCTTTATTTAACTATGTCTTTAATATGA

ATGTTTCCAAATTGTATGTATGCAGACCAAAAGCCACATTGTGGGGTTTGGCCTGCATTTTTTTTGCCTA

GAATGCTATTCAAAATAGAAATTCAAGCAAAATAATATGCAGGAGATAATATAAATGGAAAAATACAACAAT

TGGAAACGAAAATTTTATGCAATATGGGCAGGGCAAGCAGTATCATTAATCACTAGTGCCATCCTGCAAATG

GCGATTATTTTTTACCTTACAGAAAAAACAGGATCTGCGATGGTCTTGTCTATGGCTTCATTAGTAGGTTTT

TTACCCTATGCGATTTTGGGACCTGCCATTGGTGTGCTAGTGGATCGTCATGATAGGAAGAAGATAATGATT

GGTGCCGATTTAATTATCGCAGCAGCTGGTGCAGTGCTTGCTATTGTTGCATTCTGTATGGAGCTACCTGTC

TGGATGATTATGATAGTATTGTTTATCCGTAGCATTGGAACAGCTTTTCATACCCCAGCACTCAATGCGGTT

ACACCACTTTTAGTACCAGAAGAACAGCTAACGAAATGCGCAGGCTATAGTCAGTCTTTGCAGTCTATAAGC

TATATTGTTAGTCCGGCAGTTGCAGCACTCTTATACTCCGTTTGGGATTTAAATGCTATTATTGCCATCGAC

GTATTGGGTGCTGTGATTGCATCTATTACGGTAGCAATTGTACGTATACCTAAGCTGGGTAATCAAGTGCAA

AGTTTAGAACCAAATTTCATAAGGGAGATGAAAGAAGGAGTTGTGGTTCTGAGACAAAACAAAGGATTGTTT

GCCTTATTACTCTTAGGAACACTATATACTTTTGTTTATATGCCAATCAATGCACTATTTCCTTTAATAAGC

ATGGAACACTTTAATGGAACGCCTGTGCATATTTCTATTACGGAAATTTCCTTTGCATTTGGGATGCTAGCA

GGAGGCTTATTATTAGGAAGATTAGGGGGCTTCGAAAAGCATGTATTACTAATAACAAGTTCATTTTTTATA

ATGGGGACCAGTTTAGCCGTTTCGGGAATACTTCCTCCAAATGGATTTGTAATATTCGTAGTTTGCTGTGCA

ATAATGGGGCTTTCGGTGCCATTTTATAGCGGTGTGCAAACAGCTCTTTTTCAGGAGAAAATTAAGCCTGAA

TATTTAGGACGTGTATTTTCTTTGATCGGAAGTATCATGTCACTTGCTATGCCAATTGGGTTAATTCTTTCT

GGATTCTTTGCTGATAAAATCGGTGTAAATCATTGGTTTTTACTATCAGGTATTTTAATTATTGGCATTGCT

ATAGTTTGCCAAATGATAACTGAGGTTAGAAAATTAGATTTAAAATAAACAATATTGGAGGAATATTTATGT

ATCTTATTTTCATGTAACTCTTCCTGCTAAAATCGCAGGGTTTTCCCTGCATACAAGCAAATGAAAGCATGC

GATTATAGACAGGAGGAAATGTTATGGAATTAATATT

AAAAGCAAAAGACATTCGTGTGGAATTCAAAGGACGCGATGTTTTAGATATAAATGAATTAGAAGTATATGA

TTATGACCGTATTGGTTTAGTAGGAGCAAATGGTGCTGGAAAAAGCACTTTACTCAGGGTACTTTTAGGAGA

ATTAACTCCCCCAGGATGTAAAATGAATCGTCTGGGTGAACTTGCCTATATTCCCCAGTTGGACGAAGTAAC

TCTGCAGGAGGAAAAAGATTTTGCACTTGTAGGCAAGCTAGGTGTTGAGCAATTAAATATACAGACTATGAG

CGGTGGTGAAGAAACAAGGCTTAAAATAGCACAGGCCTTATCGGCACAGGTTCATGGTATTTTAGCGGATGA

ACCTACGAGCCATTTAGACCGTGAAGGAATTGATTTTCTAATAGGACAGCTAAAATATTTTACAGGTGCACT

GTTAGTTATTAGCCATGACCGCTATTTTCTTGATGAAATAGTAGATAAAATATGGGAACTGAAAGATGGCAA

AATCACTGAGTATTGGGGAAACTATTCTGATTATCTTCGTCAGAAAGAGGAAGAACGTAAGAGCCAAGCTGC

AGAATACGAACAATTTATTGCGGAACGTGCCCGATTGGAAAGGGCTGCGGAGGAAAAGCGAAAACAGGCTCG

TAAAATAGAACAGAAGGCAAAAGGTTCTTCAAAGAAAAAAAGTACTGAAGACGGAGGGCGTTTAGCTCATCA

AAAATCAATAGGAAGTAAGGAAAAAAAGATGTATAATGCTGCTAAAACCCTAGAGCACAGGATTGCGGCCTT

AGGAAAAGTAGAAGCTCCGGAAGGCATTCGCAGAATTCGTTTCAGGCAAAGTAAAGCATTGGAGCTCCATAA

TCCATACCCTATAGTCGGTGCAGAAATTAATAAAGTATTTGGGGATAAGGCTCTGTTTGAAAATGCATCTTT

TCAAATTCCGTTAGGAGCAAAAGTGGCGTTAACTGGTGGTAATGGAATCGGAAAAACAACTTTAATCCAAAT

GATCTTAAACCATGAAGAAGGAATTTCTATTTCGCCTAAGGCAAAAATAGGTTACTTTGCACAGAATGGTTA

CAAGTACAACAGTAATCAGAATGTTATGGAGTTTATGCAGAAGGATTGTGACTACAATATATCAGAAATTCG

TTCAGTGCTAGCATCTATGGGGTTCAAACAGAACGATATTGGAAAAAGTTTATCTGTTTTAAGCGGTGGAGA

AATTATAAAATTGTTGCTTGCTAAAATGCTCATGGGTAGATATAACATCCTAATAATGGATGAACCCAGTAA

CTTCCTTGACATACCAAGTTTAGAGGCTTTGGAAATACTAATGAAGGAGTACACCGGAACTATCGTGTTTAT

CACCCACGATAAACGATTACTCGAAAATGTAGCAGATGTAGTTTATGAAATTAGAGATAAGAAAATAAATCT

GAAACATTAAATTTAAGGTAGTCGCTGGTCAGTATAGTCTGTTCTGGTTGGCGACTCCATTGTTAAAGAGTA

TAAAGACTTTAGATTTTATGAATATTAAAAATAGGAACAGTCAATTGAACTGCTCCTATTTTTCTGCTAAAT

ATATTGTAGTTTTCTTATATGTATAATGATAGATTAGCGGATTCTCATCTACGGTACTTACTTCAAATATGA

AGAAGTGATCGCGGTTATCTCTGGACTTTTCCTTATTGAGGACAAAGTAATTCTTACGTGAAGTCGCCATTG

TTTTTAGGATATCATCAGTTAGGAAGGTCAATGGAATATTCATGTTAGAGTAGCGGTAGAAGTCACGTTCAA

AATCTTGGTAGCTCTCGCTATAATAGTCCATTTGTAGGTGATTACGCTGAAACTCAAGCTGATTCATAGAGC

ACCTCCTCGACAAGTTCAATACTAATAATGTCTTTTAATTTCAAATTGATGTGACCTGTTGTAGTTTTTATC

AAAATGAAATCTTTGGTCAGACTTGGTATTGTTCCAGTGTAGGAAACACGCTTGTTTTTTTCAATCACTTGA

ATGCGTGTGCGTAGCTGCCCGGCGTATACTTGACTGAGGAGTAATAATTTCTTCTCTAGTGATAAGTCAGAC

ATGTACGTTACTTTGTTTGTATCATCAGAGAGTGCTGATGCATGTTCAGATAGGAAAAAGCCCATCCATTTT

TGCATCTTTGTATCCTGGTACTCTCTTGCTGATTGAAATGGTAAATATGAACGGTCAATCATATCAAATCCT

TTCTATGCAGAGGCAAGGGTATTTTTATCAAATTGAATCGTAAAACCTTGAATTCCCCCACCTGTGTAACAT

TCTTTAAAGCGATTGATTACCTCAGTATAGATTATCACAGATGAGCTTGTTGGCTTAATGCTAAATGTAAAT

TCCAATGGTAATCGGTTTTCAGATTTAGCATGTACTAGTCGTATCGATATTTCAGTTGTTTTGAGTTTTCTC

TGACGAAGTTTTGAAGTTGCTGTTTCAACGATTCCATGTAAAAATCTTTCAAGCATTTCAATATCATTACAT

CCTTTGCTACGGATTTCTGAAAATTGTACTGTATTTTTTTCTTGTTTCATTTTAATCCCTCCAATCCACCCG

CGGAATGACCACCGATAAGTTTACTGCGTTCAATATTTCTGGAACCTTCAGTTAGGACGGTTCCTTTTTGTA

TGGCTAAAAAACCAAACTGTTCTCTGACAACATCAATAGCTGTCTGAAGTCTATTATCTTTTTCAATTTGTT

CTACATCATCAAAGAGTGATAGTAGAGTATAGCTTTCATCTACGAAGCCACTATAAGATACACCAATTTGTC

TCACTGCACCAGAGGTGTATTTTTTCGGAATAATACAAGTACATGACTCACCATTGTTTTGGGGAGATTTG

CGGGTTCAATTTTATTCTGAGCATTTATAGATTTTTTCATCTCAGTCCTAGAATAGCCAATATGAATAGAAA

CGACAGTAGTCAATACTAGGGCTACTGTTCCGTTCCACAGTATCATTTAAAAATCATT-TTCACACCCTTTC

GTCTATTAGTATAGAAGAAAGCTCTCAGCACA

>7879-04/Mega-1.V

CATGTTGAGGCGGTAAGTTTGCTAGTCAAGGAGTAAAACGACGAAGATTAGCATTTACTTCCGCCCATGCGA

TAGCTGTCCGTGATTGACAAGTGCTAGCACGCAGACAGAACGGAGATAGCGAACCGCTGAGTGTGTCGCTCT

GCTCGTAAAAGCTTAGAAACCTTTGAACGAAAGGGATAATGAAAGCCTTGATTGCAAGGCTTTTTGCTTTAT

GGTGGGTAAGTATCAGAGTGAGAAAATTTTTGGAATGAGTAGAAGTGATAGCTAGAAATTATCAGTTTCTA

TTTCCATTTACCCTGTGGGTACGTGTTTGTTTCCATTGACAAGGAGTTTGTGGGAATAGAAATGT

ACCCACCTTGTTTGAATCAAGTGAAGTGTAGTTGAAGGAAATCTGTTGAAAGCAATACTTCATTTTACCG

AATAAGTAATAATTTAGGCAACTTCAAATCGATTAAAAAAAACTATTTTAAAGGTTAAGAGTAGACAAAA

ATTGTCCACTCTTTTTTGCAAACTCAATTTATCAATAAATGAAATGAGGGAATGTAAAATG

AAATATTTTGAGGTTGAGTTAGAAAATCCTGATGAATTTTTAAAACTACAAACAGAAGATTTTGTGAAAG

CTAATCGCTTGCTACTAAGGAAGATAATCCAGAGCGTTACAGTCTATGAAGAAAACTTCGTCATATCCTT

TAAATCTGGCATCGAATTGGAAGTATGAGTCTCATTCCATAACTTTTATATTGAACATATCATCTTGTTGTG

TTATACTATAAATTGATATAAACAAAGATGTAGGAGGAACCGAAACTATGACAGCCTCAATGCGTTTAAGAT

AAGCTGGCAATAAAAAAAGCAGAATCTATACCCGATGATAGGCTTTTTTGTTGTGCTTATTTATACGATATT

GAGCATTCATTAGTTACGGTGAGGATATTGGTTATTTAACTATACCTTTATTTAACTATGTCTTTAATATGA

ATGTTTCCAAATTGTATGTATGCAGACCAAAAGCCACATTGTGGGGTTTGGCCTGCATTTTTTTTGCCTA

GAATGCTATTCAAAATAGAAATTCAAGCAAAATAATATGCAGGAGATAATATAAATGGAAAAATACAACAAT

TGGAAACGAAAATTTTATGCAATATGGGCAGGGCAAGCAGTATCATTAATCACTAGTGCCATCCTGCAAATG

GCGATTATTTTTTACCTTACAGAAAAAACAGGATCTGCGATGGTCTTGTCTATGGCTTCATTAGTAGGTTTT

TTACCCTATGCGATTTTGGGACCTGCCATTGGTGTGCTAGTGGATCGTCATGATAGGAAGAAGATAATGATT

GGTGCCGATTTAATTATCGCAGCAGCTGGTGCAGTGCTTGCTATTGTTGCATTCTGTATGGAGCTACCTGTC

TGGATGATTATGATAGTATTGTTTATCCGTAGCATTGGAACAGCTTTTCATACCCCAGCACTCAATGCGGTT

ACACCACTTTTAGTACCAGAAGAACAGCTAACGAAATGCGCAGGCTATAGTCAGTCTTTGCAGTCTATAAGC

TATATTGTTAGTCCGGCAGTTGCAGCACTCTTATACTCCGTTTGGGATTTAAATGCTATTATTGCCATCGAC

GTATTGGGTGCTGTGATTGCATCTATTACGGTAGCAATTGTACGTATACCTAAGCTGGGTAATCAAGTGCAA

AGTTTAGAACCAAATTTCATAAGGGAGATGAAAGAAGGAGTTGTGGTTCTGAGACAAAACAAAGGATTGTTT

GCCTTATTACTCTTAGGAACACTATATACTTTTGTTTATATGCCAATCAATGCACTATTTCCTTTAATAAGC

ATGGAACACTTTAATGGAACGCCTGTGCATATTTCTATTACGGAAATTTCCTTTGCATTTGGGATGCTAGCA

GGAGGCTTATTATTAGGAAGATTAGGGGGCTTCGAAAAGCATGTATTACTAATAACAAGTTCATTTTTTATA

ATGGGGACCAGTTTAGCCGTTTCGGGAATACTTCCTCCAAATGGATTTGTAATATTCGTAGTTTGCTGTGCA

ATAATGGGGCTTTCGGTGCCATTTTATAGCGGTGTGCAAACAGCTCTTTTTCAGGAGAAAATTAAGCCTGAA

TATTTAGGACGTGTATTTTCTTTGATCGGAAGTATCATGTCACTTGCTATGCCAATTGGGTTAATTCTTTCT

GGATTCTTTGCTGATAAAATCGGTGTAAATCATTGGTTTTTACTATCAGGTATTTTAATTATTGGCATTGCT

ATAGTTTGCCAAATGATAACTGAGGTTAGAAAATTAGATTTAAAATAAACAATATTGGAGGAATATTTATGT

ATCTTATTTTCATGTAACTCTTCCTGCTAAAATCGCAGGGTTTTCCCTGCATACAAGCAAATGAAAGCATGC

GATTATAGACAGGAGGAAATGTTATGGAATTAATATT

AAAAGCAAAAGACATTCGTGTGGAATTCAAAGGACGCGATGTTTTAGATATAAATGAATTAGAAGTATATGA

TTATGACCGTATTGGTTTAGTAGGAGCAAATGGTGCTGGAAAAAGCACTTTACTCAGGGTACTTTTAGGAGA

ATTAACTCCCCCAGGATGTAAAATGAATCGTCTGGGTGAACTTGCCTATATTCCCCAGTTGGACGAAGTAAC

TCTGCAGGAGGAAAAAGATTTTGCACTTGTAGGCAAGCTAGGTGTTGAGCAATTAAATATACAGACTATGAG

CGGTGGTGAAGAAACAAGGCTTAAAATAGCACAGGCCTTATCGGCACAGGTTCATGGTATTTTAGCGGATGA

ACCTACGAGCCATTTAGACCGTGAAGGAATTGATTTTCTAATAGGACAGCTAAAATATTTTACAGGTGCACT

GTTAGTTATTAGCCATGACCGCTATTTTCTTGATGAAATAGTAGATAAAATATGGGAACTGAAAGATGGCAA

AATCACTGAGTATTGGGGAAACTATTCTGATTATCTTCGTCAGAAAGAGGAAGAACGTAAGAGCCAAGCTGC

AGAATACGAACAATTTATTGCGGAACGTGCCCGATTGGAAAGGGCTGCGGAGGAAAAGCGAAAACAGGCTCG

TAAAATAGAACAGAAGGCAAAAGGTTCTTCAAAGAAAAAAAGTACTGAAGACGGAGGGCGTTTAGCTCATCA

AAAATCAATAGGAAGTAAGGAAAAAAAGATGTATAATGCTGCTAAAACCCTAGAGCACAGGATTGCGGCCTT

AGGAAAAGTAGAAGCTCCGGAAGGCATTCGCAGAATTCGTTTCAGGCAAAGTAAAGCATTGGAGCTCCATAA

TCCATACCCTATAGTCGGTGCAGAAATTAATAAAGTATTTGGGGATAAGGCTCTGTTTGAAAATGCATCTTT

TCAAATTCCGTTAGGAGCAAAAGTGGCGTTAACTGGTGGTAATGGAATCGGAAAAACAACTTTAATCCAAAT

GATCTTAAACCATGAAGAAGGAATTTCTATTTCGCCTAAGGCAAAAATAGGTTACTTTGCACAGAATGGTTA

CAAGTACAACAGTAATCAGAATGTTATGGAGTTTATGCAGAAGGATTGTGACTACAATATATCAGAAATTCG

TTCAGTGCTAGCATCTATGGGGTTCAAACAGAACGATATTGGAAAAAGTTTATCTGTTTTAAGCGGTGGAGA

AATTATAAAATTGTTGCTTGCTAAAATGCTCATGGGTAGATATAACATCCTAATAATGGATGAACCCAGTAA

CTTCCTTGACATACCAAGTTTAGAGGCTTTGGAAATACTAATGAAGGAGTACACCGGAACTATCGTGTTTAT

CACCCACGATAAACGATTACTCGAAAATGTAGCAGATGTAGTTTATGAAATTAGAGATAAGAAAATAAATCT

GAAACATTAAATTTAAGGTAGTCGCTGGTCAGTATAGTCTGTTCTGGTTGGCGACTCCATTGTTAAAGAGTA

TAAAGACTTTAGATTTTATGAATATTAAAAATAGGAACAGTCAATTGAACTGCTCCTATTTTTCTGCTAAAT

ATATTGTAGTTTTCTTATATGTATAATGATAGATTAGCGGATTCTCATCTACGGTACTTACTTCAAATATGA

AGAAGTGATCGCGGTTATCTCTGGACTTTTCCTTATTGAGGACAAAGTAATTCTTACGTGAAGTCGCCATTG

TTTTTAGGATATCATCAGTTAGGAAGGTCAATGGAATATTCATGTTAGAGTAGCGGTAGAAGTCACGTTCAA

AATCTTGGTAGCTCTCGCTATAATAGTCCATTTGTAGGTGATTACGCTGAAACTCAAGCTGATTCATAGAGC

ACCTCCTCGACAAGTTCAATACTAATAATGTCTTTTAATTTCAAATTGATGTGACCTGTTGTAGTTTTTATC

AAAATGAAATCTTTGGTCAGACTTGGTATTGTTCCAGTGTAGGAAACACGCTTGTTTTTTTCAATCACTTGA

ATGCGTGTGCGTAGCTGCCCGGCGTATACTTGACTGAGGAGTAATAATTTCTTCTCTAGTGATAAGTCAGAC

ATGTACGTTACTTTGTTTGTATCATCAGAGAGTGCTGATGCATGTTCAGATAGGAAAAAGCCCATCCATTTT

TGCATCTTTGTATCCTGGTACTCTCTTGCTGATTGAAATGGTAAATATGAACGGTCAATCATATCAAATCCT

TTCTATGCAGAGGCAAGGGTATTTTTATCAAATTGAATCGTAAAACCTTGAATTCCCCCACCTGTGTAACAT

TCTTTAAAGCGATTGATTACCTCAGTATAGATTATCACAGATGAGCTTGTTGGCTTAATGCTAAATGTAAAT

TCCAATGGTAATCGGTTTTCAGATTTAGCATGTACTAGTCGTATCGATATTTCAGTTGTTTTGAGTTTTCTC

TGACGAAGTTTTGAAGTTGCTGTTTCAACGATTCCATGTAAAAATCTTTCAAGCATTTCAATATCATTACAT

CCTTTGCTACGGATTTCTGAAAATTGTACTGTATTTTTTTCTTGTTTCATTTTAATCCCTCCAATCCACCCG

CGGAATGACCACCGATAAGTTTACTGCGTTCAATATTTCTGGAACCTTCAGTTAGGACGGTTCCTTTTTGTA

TGGCTAAAAAACCAAACTGTTCTCTGACAACATCAATAGCTGTCTGAAGTCTATTATCTTTTTCAATTTGTT

CTACATCATCAAAGAGTGATAGTAGAGTATAGCTTTCATCTACGAAGCCACTATAAGATACACCAATTTGTC

TCACTGCACCAGAGGTGTATTTTTTCGGAATAATACAAGTACATGACTCACCATTGTTTTGGGGAGATTTG

CGGGTTCAATTTTATTCTGAGCATTTATAGATTTTTTCATCTCAGTCCTAGAATAGCCAATATGAATAGAAA

CGACAGTAGTCAATACTAGGGCTACTGTTCCGTTCCACAGTATCATTTAAAAATCATT-TTCACACCCTTTC

GTCTATTAGTATAGAAGAAAGCTCTCAGCACA

>EU-NP02/Mega-1.V

CATGTTGAGGCGGTAAGTTTGCTAGTCAAGGAGTAAAACGACGAAGATTAGCATTTACTTCCGCCCATGCGA

TAGCTGTCCGTGATTGACAAGTGCTAGCACGCAGACAGAACGGAGATAGCGAACCGCTGAGTGTGTCGCTCT

GCTCGTAAAAGCTTAGAAACCTTTGAACGAAAGGGATAATGAAAGCCTTGATTGCAAGGCTTTTTGCTTTAT

GGTGGGTAAGTATCAGAGTGAGAAAATTTTTGGAATGAGTAGAAGTGATAGCTAGAAATTATCAGTTTCTA

TTTCCATTTACCCTGTGGGTACGTGTTTGTTTCCATTGACAAGGAGTTTGTGGGAATAGAAATGT

ACCCACCTTGTTTGAATCAAGTGAAGTGTAGTTGAAGGAAATCTGTTGAAAGCAATACTTCATTTTACCG

AATAAGTAATAATTTAGGCAACTTCAAATCGATTAAAAAAAACTATTTTAAAGGTTAAGAGTAGACAAAA

ATTGTCCACTCTTTTTTGCAAACTCAATTTATCAATAAATGAAATGAGGGAATGTAAAATG

AAATATTTTGAGGTTGAGTTAGAAAATCCTGATGAATTTTTAAAACTACAAACAGAAGATTTTGTGAAAG

CTAATCGCTTGCTACTAAGGAAGATAATCCAGAGCGTTACAGTCTATGAAGAAAACTTCGTCATATCCTT

TAAATCTGGCATCGAATTGGAAGTATGAGTCTCATTCCATAACTTTTATATTGAACATATCATCTTGTTGTG

TTATACTATAAATTGATATAAACAAAGATGTAGGAGGAACCGAAACTATGACAGCCTCAATGCGTTTAAGAT

AAGCTGGCAATAAAAAAAGCAGAATCTATACCCGATGATAGGCTTTTTTGTTGTGCTTATTTATACGATATT

GAGCATTCATTAGTTACGGTGAGGATATTGGTTATTTAACTATACCTTTATTTAACTATGTCTTTAATATGA

ATGTTTCCAAATTGTATGTATGCAGACCAAAAGCCACATTGTGGGGTTTGGCCTGCATTTTTTTTGCCTA

GAATGCTATTCAAAATAGAAATTCAAGCAAAATAATATGCAGGAGATAATATAAATGGAAAAATACAACAAT

TGGAAACGAAAATTTTATGCAATATGGGCAGGGCAAGCAGTATCATTAATCACTAGTGCCATCCTGCAAATG

GCGATTATTTTTTACCTTACAGAAAAAACAGGATCTGCGATGGTCTTGTCTATGGCTTCATTAGTAGGTTTT

TTACCCTATGCGATTTTGGGACCTGCCATTGGTGTGCTAGTGGATCGTCATGATAGGAAGAAGATAATGATT

GGTGCCGATTTAATTATCGCAGCAGCTGGTGCAGTGCTTGCTATTGTTGCATTCTGTATGGAGCTACCTGTC

TGGATGATTATGATAGTATTGTTTATCCGTAGCATTGGAACAGCTTTTCATACCCCAGCACTCAATGCGGTT

ACACCACTTTTAGTACCAGAAGAACAGCTAACGAAATGCGCAGGCTATAGTCAGTCTTTGCAGTCTATAAGC

TATATTGTTAGTCCGGCAGTTGCAGCACTCTTATACTCCGTTTGGGATTTAAATGCTATTATTGCCATCGAC

GTATTGGGTGCTGTGATTGCATCTATTACGGTAGCAATTGTACGTATACCTAAGCTGGGTAATCAAGTGCAA

AGTTTAGAACCAAATTTCATAAGGGAGATGAAAGAAGGAGTTGTGGTTCTGAGACAAAACAAAGGATTGTTT

GCCTTATTACTCTTAGGAACACTATATACTTTTGTTTATATGCCAATCAATGCACTATTTCCTTTAATAAGC

ATGGAACACTTTAATGGAACGCCTGTGCATATTTCTATTACGGAAATTTCCTTTGCATTTGGGATGCTAGCA

GGAGGCTTATTATTAGGAAGATTAGGGGGCTTCGAAAAGCATGTATTACTAATAACAAGTTCATTTTTTATA

ATGGGGACCAGTTTAGCCGTTTCGGGAATACTTCCTCCAAATGGATTTGTAATATTCGTAGTTTGCTGTGCA

ATAATGGGGCTTTCGGTGCCATTTTATAGCGGTGTGCAAACAGCTCTTTTTCAGGAGAAAATTAAGCCTGAA

TATTTAGGACGTGTATTTTCTTTGATCGGAAGTATCATGTCACTTGCTATGCCAATTGGGTTAATTCTTTCT

GGATTCTTTGCTGATAAAATCGGTGTAAATCATTGGTTTTTACTATCAGGTATTTTAATTATTGGCATTGCT

ATAGTTTGCCAAATGATAACTGAGGTTAGAAAATTAGATTTAAAATAAACAATATTGGAGGAATATTTATGT

ATCTTATTTTCATGTAACTCTTCCTGCTAAAATCGCAGGGTTTTCCCTGCATACAAGCAAATGAAAGCATGC

GATTATAGACAGGAGGAAATGTTATGGAATTAATATT

AAAAGCAAAAGACATTCGTGTGGAATTCAAAGGACGCGATGTTTTAGATATAAATGAATTAGAAGTATATGA

TTATGACCGTATTGGTTTAGTAGGAGCAAATGGTGCTGGAAAAAGCACTTTACTCAGGGTACTTTTAGGAGA

ATTAACTCCCCCAGGATGTAAAATGAATCGTCTGGGTGAACTTGCCTATATTCCCCAGTTGGACGAAGTAAC

TCTGCAGGAGGAAAAAGATTTTGCACTTGTAGGCAAGCTAGGTGTTGAGCAATTAAATATACAGACTATGAG

CGGTGGTGAAGAAACAAGGCTTAAAATAGCACAGGCCTTATCGGCACAGGTTCATGGTATTTTAGCGGATGA

ACCTACGAGCCATTTAGACCGTGAAGGAATTGATTTTCTAATAGGACAGCTAAAATATTTTACAGGTGCACT

GTTAGTTATTAGCCATGACCGCTATTTTCTTGATGAAATAGTAGATAAAATATGGGAACTGAAAGATGGCAA

AATCACTGAGTATTGGGGAAACTATTCTGATTATCTTCGTCAGAAAGAGGAAGAACGTAAGAGCCAAGCTGC

AGAATACGAACAATTTATTGCGGAACGTGCCCGATTGGAAAGGGCTGCGGAGGAAAAGCGAAAACAGGCTCG

TAAAATAGAACAGAAGGCAAAAGGTTCTTCAAAGAAAAAAAGTACTGAAGACGGAGGGCGTTTAGCTCATCA

AAAATCAATAGGAAGTAAGGAAAAAAAGATGTATAATGCTGCTAAAACCCTAGAGCACAGGATTGCGGCCTT

AGGAAAAGTAGAAGCTCCGGAAGGCATTCGCAGAATTCGTTTCAGGCAAAGTAAAGCATTGGAGCTCCATAA

TCCATACCCTATAGTCGGTGCAGAAATTAATAAAGTATTTGGGGATAAGGCTCTGTTTGAAAATGCATCTTT

TCAAATTCCGTTAGGAGCAAAAGTGGCGTTAACTGGTGGTAATGGAATCGGAAAAACAACTTTAATCCAAAT

GATCTTAAACCATGAAGAAGGAATTTCTATTTCGCCTAAGGCAAAAATAGGTTACTTTGCACAGAATGGTTA

CAAGTACAACAGTAATCAGAATGTTATGGAGTTTATGCAGAAGGATTGTGACTACAATATATCAGAAATTCG

TTCAGTGCTAGCATCTATGGGGTTCAAACAGAACGATATTGGAAAAAGTTTATCTGTTTTAAGCGGTGGAGA

AATTATAAAATTGTTGCTTGCTAAAATGCTCATGGGTAGATATAACATCCTAATAATGGATGAACCCAGTAA

CTTCCTTGACATACCAAGTTTAGAGGCTTTGGAAATACTAATGAAGGAGTACACCGGAACTATCGTGTTTAT

CACCCACGATAAACGATTACTCGAAAATGTAGCAGATGTAGTTTATGAAATTAGAGATAAGAAAATAAATCT

GAAACATTAAATTTAAGGTAGTCGCTGGTCAGTATAGTCTGTTCTGGTTGGCGACTCCATTGTTAAAGAGTA

TAAAGACTTTAGATTTTATGAATATTAAAAATAGGAACAGTCAATTGAACTGCTCCTATTTTTCTGCTAAAT

ATATTGTAGTTTTCTTATATGTATAATGATAGATTAGCGGATTCTCATCTACGGTACTTACTTCAAATATGA

AGAAGTGATCGCGGTTATCTCTGGACTTTTCCTTATTGAGGACAAAGTAATTCTTACGTGAAGTCGCCATTG

TTTTTAGGATATCATCAGTTAGGAAGGTCAATGGAATATTCATGTTAGAGTAGCGGTAGAAGTCACGTTCAA

AATCTTGGTAGCTCTCGCTATAATAGTCCATTTGTAGGTGATTACGCTGAAACTCAAGCTGATTCATAGAGC

ACCTCCTCGACAAGTTCAATACTAATAATGTCTTTTAATTTCAAATTGATGTGACCTGTTGTAGTTTTTATC

AAAATGAAATCTTTGGTCAGACTTGGTATTGTTCCAGTGTAGGAAACACGCTTGTTTTTTTCAATCACTTGA

ATGCGTGTGCGTAGCTGCCCGGCGTATACTTGACTGAGGAGTAATAATTTCTTCTCTAGTGATAAGTCAGAC

ATGTACGTTACTTTGTTTGTATCATCAGAGAGTGCTGATGCATGTTCAGATAGGAAAAAGCCCATCCATTTT

TGCATCTTTGTATCCTGGTACTCTCTTGCTGATTGAAATGGTAAATATGAACGGTCAATCATATCAAATCCT

TTCTATGCAGAGGCAAGGGTATTTTTATCAAATTGAATCGTAAAACCTTGAATTCCCCCACCTGTGTAACAT

TCTTTAAAGCGATTGATTACCTCAGTATAGATTATCACAGATGAGCTTGTTGGCTTAATGCTAAATGTAAAT

TCCAATGGTAATCGGTTTTCAGATTTAGCATGTACTAGTCGTATCGATATTTCAGTTGTTTTGAGTTTTCTC

TGACGAAGTTTTGAAGTTGCTGTTTCAACGATTCCATGTAAAAATCTTTCAAGCATTTCAATATCATTACAT

CCTTTGCTACGGATTTCTGAAAATTGTACTGTATTTTTTTCTTGTTTCATTTTAATCCCTCCAATCCACCCG

CGGAATGACCACCGATAAGTTTACTGCGTTCAATATTTCTGGAACCTTCAGTTAGGACGGTTCCTTTTTGTA

TGGCTAAAAAACCAAACTGTTCTCTGACAACATCAATAGCTGTCTGAAGTCTATTATCTTTTTCAATTTGTT

CTACATCATCAAAGAGTGATAGTAGAGTATAGCTTTCATCTACGAAGCCACTATAAGATACACCAATTTGTC

TCACTGCACCAGAGGTGTATTTTTTCGGAATAATACAAGTACATGACTCACCATTGTTTTGGGGAGATTTG

CGGGTTCAATTTTATTCTGAGCATTTATAGATTTTTTCATCTCAGTCCTAGAATAGCCAATATGAATAGAAA

CGACAGTAGTCAATACTAGGGCTACTGTTCCGTTCCACAGTATCATTTAAAAATCATT-TTCACACCCTTTC

GTCTATTAGTATAGAAGAAAGCTCTCAGCACA

>GA44288/Mega-1.V

CATGTTGAGGCGGTAAGTTTGCTAGTCAAGGAGTAAAACGACGAAGATTAGCATTTACTTCCGCCCATGCGA

TAGCTGTCCGTGATTGACAAGTGCTAGCACGCAGACAGAACGGAGATAGCGAACCGCTGAGTGTGTCGCTCT

GCTCGTAAAAGCTTAGAAACCTTTGAACGAAAGGGATAATGAAAGCCTTGATTGCAAGGCTTTTTGCTTTAT

GGTGGGTAAGTATCAGAGTGAGAAAATTTTTGGAATGAGTAGAAGTGATAGCTAGAAATTATCAGTTTCTA

TTTCCATTTACCCTGTGGGTACGTGTTTGTTTCCATTGACAAGGAGTTTGTGGGAATAGAAATGT

ACCCACCTTGTTTGAATCAAGTGAAGTGTAGTTGAAGGAAATCTGTTGAAAGCAATACTTCATTTTACCG

AATAAGTAATAATTTAGGCAACTTCAAATCGATTAAAAAAAACTATTTTAAAGGTTAAGAGTAGACAAAA

ATTGTCCACTCTTTTTTGCAAACTCAATTTATCAATAAATGAAATGAGGGAATGTAAAATG

AAATATTTTGAGGTTGAGTTAGAAAATCCTGATGAATTTTTAAAACTACAAACAGAAGATTTTGTGAAAG

CTAATCGCTTGCTACTAAGGAAGATAATCCAGAGCGTTACAGTCTATGAAGAAAACTTCGTCATATCCTT

TAAATCTGGCATCGAATTGGAAGTATGAGTCTCATTCCATAACTTTTATATTGAACATATCATCTTGTTGTG

TTATACTATAAATTGATATAAACAAAGATGTAGGAGGAACCGAAACTATGACAGCCTCAATGCGTTTAAGAT

AAGCTGGCAATAAAAAAAGCAGAATCTATACCCGATGATAGGCTTTTTTGTTGTGCTTATTTATACGATATT

GAGCATTCATTAGTTACGGTGAGGATATTGGTTATTTAACTATACCTTTATTTAACTATGTCTTTAATATGA

ATGTTTCCAAATTGTATGTATGCAGACCAAAAGCCACATTGTGGGGTTTGGCCTGCATTTTTTTTGCCTA

GAATGCTATTCAAAATAGAAATTCAAGCAAAATAATATGCAGGAGATAATATAAATGGAAAAATACAACAAT

TGGAAACGAAAATTTTATGCAATATGGGCAGGGCAAGCAGTATCATTAATCACTAGTGCCATCCTGCAAATG

GCGATTATTTTTTACCTTACAGAAAAAACAGGATCTGCGATGGTCTTGTCTATGGCTTCATTAGTAGGTTTT

TTACCCTATGCGATTTTGGGACCTGCCATTGGTGTGCTAGTGGATCGTCATGATAGGAAGAAGATAATGATT

GGTGCCGATTTAATTATCGCAGCAGCTGGTGCAGTGCTTGCTATTGTTGCATTCTGTATGGAGCTACCTGTC

TGGATGATTATGATAGTATTGTTTATCCGTAGCATTGGAACAGCTTTTCATACCCCAGCACTCAATGCGGTT

ACACCACTTTTAGTACCAGAAGAACAGCTAACGAAATGCGCAGGCTATAGTCAGTCTTTGCAGTCTATAAGC

TATATTGTTAGTCCGGCAGTTGCAGCACTCTTATACTCCGTTTGGGATTTAAATGCTATTATTGCCATCGAC

GTATTGGGTGCTGTGATTGCATCTATTACGGTAGCAATTGTACGTATACCTAAGCTGGGTAATCAAGTGCAA

AGTTTAGAACCAAATTTCATAAGGGAGATGAAAGAAGGAGTTGTGGTTCTGAGACAAAACAAAGGATTGTTT

GCCTTATTACTCTTAGGAACACTATATACTTTTGTTTATATGCCAATCAATGCACTATTTCCTTTAATAAGC

ATGGAACACTTTAATGGAACGCCTGTGCATATTTCTATTACGGAAATTTCCTTTGCATTTGGGATGCTAGCA

GGAGGCTTATTATTAGGAAGATTAGGGGGCTTCGAAAAGCATGTATTACTAATAACAAGTTCATTTTTTATA

ATGGGGACCAGTTTAGCCGTTTCGGGAATACTTCCTCCAAATGGATTTGTAATATTCGTAGTTTGCTGTGCA

ATAATGGGGCTTTCGGTGCCATTTTATAGCGGTGTGCAAACAGCTCTTTTTCAGGAGAAAATTAAGCCTGAA

TATTTAGGACGTGTATTTTCTTTGATCGGAAGTATCATGTCACTTGCTATGCCAATTGGGTTAATTCTTTCT

GGATTCTTTGCTGATAAAATCGGTGTAAATCATTGGTTTTTACTATCAGGTATTTTAATTATTGGCATTGCT

ATAGTTTGCCAAATGATAACTGAGGTTAGAAAATTAGATTTAAAATAAACAATATTGGAGGAATATTTATGT

ATCTTATTTTCATGTAACTCTTCCTGCTAAAATCGCAGGGTTTTCCCTGCATACAAGCAAATGAAAGCATGC

GATTATAGACAGGAGGAAATGTTATGGAATTAATATT

AAAAGCAAAAGACATTCGTGTGGAATTCAAAGGACGCGATGTTTTAGATATAAATGAATTAGAAGTATATGA

TTATGACCGTATTGGTTTAGTAGGAGCAAATGGTGCTGGAAAAAGCACTTTACTCAGGGTACTTTTAGGAGA

ATTAACTCCCCCAGGATGTAAAATGAATCGTCTGGGTGAACTTGCCTATATTCCCCAGTTGGACGAAGTAAC

TCTGCAGGAGGAAAAAGATTTTGCACTTGTAGGCAAGCTAGGTGTTGAGCAATTAAATATACAGACTATGAG

CGGTGGTGAAGAAACAAGGCTTAAAATAGCACAGGCCTTATCGGCACAGGTTCATGGTATTTTAGCGGATGA

ACCTACGAGCCATTTAGACCGTGAAGGAATTGATTTTCTAATAGGACAGCTAAAATATTTTACAGGTGCACT

GTTAGTTATTAGCCATGACCGCTATTTTCTTGATGAAATAGTAGATAAAATATGGGAACTGAAAGATGGCAA

AATCACTGAGTATTGGGGAAACTATTCTGATTATCTTCGTCAGAAAGAGGAAGAACGTAAGAGCCAAGCTGC

AGAATACGAACAATTTATTGCGGAACGTGCCCGATTGGAAAGGGCTGCGGAGGAAAAGCGAAAACAGGCTCG

TAAAATAGAACAGAAGGCAAAAGGTTCTTCAAAGAAAAAAAGTACTGAAGACGGAGGGCGTTTAGCTCATCA

AAAATCAATAGGAAGTAAGGAAAAAAAGATGTATAATGCTGCTAAAACCCTAGAGCACAGGATTGCGGCCTT

AGGAAAAGTAGAAGCTCCGGAAGGCATTCGCAGAATTCGTTTCAGGCAAAGTAAAGCATTGGAGCTCCATAA

TCCATACCCTATAGTCGGTGCAGAAATTAATAAAGTATTTGGGGATAAGGCTCTGTTTGAAAATGCATCTTT

TCAAATTCCGTTAGGAGCAAAAGTGGCGTTAACTGGTGGTAATGGAATCGGAAAAACAACTTTAATCCAAAT

GATCTTAAACCATGAAGAAGGAATTTCTATTTCGCCTAAGGCAAAAATAGGTTACTTTGCACAGAATGGTTA

CAAGTACAACAGTAATCAGAATGTTATGGAGTTTATGCAGAAGGATTGTGACTACAATATATCAGAAATTCG

TTCAGTGCTAGCATCTATGGGGTTCAAACAGAACGATATTGGAAAAAGTTTATCTGTTTTAAGCGGTGGAGA

AATTATAAAATTGTTGCTTGCTAAAATGCTCATGGGTAGATATAACATCCTAATAATGGATGAACCCAGTAA

CTTCCTTGACATACCAAGTTTAGAGGCTTTGGAAATACTAATGAAGGAGTACACCGGAACTATCGTGTTTAT

CACCCACGATAAACGATTACTCGAAAATGTAGCAGATGTAGTTTATGAAATTAGAGATAAGAAAATAAATCT

GAAACATTAAATTTAAGGTAGTCGCTGGTCAGTATAGTCTGTTCTGGTTGGCGACTCCATTGTTAAAGAGTA

TAAAGACTTTAGATTTTATGAATATTAAAAATAGGAACAGTCAATTGAACTGCTCCTATTTTTCTGCTAAAT

ATATTGTAGTTTTCTTATATGTATAATGATAGATTAGCGGATTCTCATCTACGGTACTTACTTCAAATATGA

AGAAGTGATCGCGGTTATCTCTGGACTTTTCCTTATTGAGGACAAAGTAATTCTTACGTGAAGTCGCCATTG

TTTTTAGGATATCATCAGTTAGGAAGGTCAATGGAATATTCATGTTAGAGTAGCGGTAGAAGTCACGTTCAA

AATCTTGGTAGCTCTCGCTATAATAGTCCATTTGTAGGTGATTACGCTGAAACTCAAGCTGATTCATAGAGC

ACCTCCTCGACAAGTTCAATACTAATAATGTCTTTTAATTTCAAATTGATGTGACCTGTTGTAGTTTTTATC

AAAATGAAATCTTTGGTCAGACTTGGTATTGTTCCAGTGTAGGAAACACGCTTGTTTTTTTCAATCACTTGA

ATGCGTGTGCGTAGCTGCCCGGCGTATACTTGACTGAGGAGTAATAATTTCTTCTCTAGTGATAAGTCAGAC

ATGTACGTTACTTTGTTTGTATCATCAGAGAGTGCTGATGCATGTTCAGATAGGAAAAAGCCCATCCATTTT

TGCATCTTTGTATCCTGGTACTCTCTTGCTGATTGAAATGGTAAATATGAACGGTCAATCATATCAAATCCT

TTCTATGCAGAGGCAAGGGTATTTTTATCAAATTGAATCGTAAAACCTTGAATTCCCCCACCTGTGTAACAT

TCTTTAAAGCGATTGATTACCTCAGTATAGATTATCACAGATGAGCTTGTTGGCTTAATGCTAAATGTAAAT

TCCAATGGTAATCGGTTTTCAGATTTAGCATGTACTAGTCGTATCGATATTTCAGTTGTTTTGAGTTTTCTC

TGACGAAGTTTTGAAGTTGCTGTTTCAACGATTCCATGTAAAAATCTTTCAAGCATTTCAATATCATTACAT

CCTTTGCTACGGATTTCTGAAAATTGTACTGTATTTTTTTCTTGTTTCATTTTAATCCCTCCAATCCACCCG

CGGAATGACCACCGATAAGTTTACTGCGTTCAATATTTCTGGAACCTTCAGTTAGGACGGTTCCTTTTTGTA

TGGCTAAAAAACCAAACTGTTCTCTGACAACATCAATAGCTGTCTGAAGTCTATTATCTTTTTCAATTTGTT

CTACATCATCAAAGAGTGATAGTAGAGTATAGCTTTCATCTACGAAGCCACTATAAGATACACCAATTTGTC

TCACTGCACCAGAGGTGTATTTTTTCGGAATAATACAAGTACATGACTCACCATTGTTTTGGGGAGATTTG

CGGGTTCAATTTTATTCTGAGCATTTATAGATTTTTTCATCTCAGTCCTAGAATAGCCAATATGAATAGAAA

CGACAGTAGTCAATACTAGGGCTACTGTTCCGTTCCACAGTATCATTTAAAAATCATT-TTCACACCCTTTC

GTCTATTAGTATAGAAGAAAGCTCTCAGCACA

>GA47688/Mega-1.V

CATGTTGAGGCGGTAAGTTTGCTAGTCAAGGAGTAAAACGACGAAGATTAGCATTTACTTCCGCCCATGCGA

TAGCTGTCCGTGATTGACAAGTGCTAGCACGCAGACAGAACGGAGATAGCGAACCGCTGAGTGTGTCGCTCT

GCTCGTAAAAGCTTAGAAACCTTTGAACGAAAGGGATAATGAAAGCCTTGATTGCAAGGCTTTTTGCTTTAT

GGTGGGTAAGTATCAGAGTGAGAAAATTTTTGGAATGAGTAGAAGTGATAGCTAGAAATTATCAGTTTCTA

TTTCCATTTACCCTGTGGGTACGTGTTTGTTTCCATTGACAAGGAGTTTGTGGGAATAGAAATGT

ACCCACCTTGTTTGAATCAAGTGAAGTGTAGTTGAAGGAAATCTGTTGAAAGCAATACTTCATTTTACCG

AATAAGTAATAATTTAGGCAACTTCAAATCGATTAAAAAAAACTATTTTAAAGGTTAAGAGTAGACAAAA

ATTGTCCACTCTTTTTTGCAAACTCAATTTATCAATAAATGAAATGAGGGAATGTAAAATG

AAATATTTTGAGGTTGAGTTAGAAAATCCTGATGAATTTTTAAAACTACAAACAGAAGATTTTGTGAAAG

CTAATCGCTTGCTACTAAGGAAGATAATCCAGAGCGTTACAGTCTATGAAGAAAACTTCGTCATATCCTT

TAAATCTGGCATCGAATTGGAAGTATGAGTCTCATTCCATAACTTTTATATTGAACATATCATCTTGTTGTG

TTATACTATAAATTGATATAAACAAAGATGTAGGAGGAACCGAAACTATGACAGCCTCAATGCGTTTAAGAT

AAGCTGGCAATAAAAAAAGCAGAATCTATACCCGATGATAGGCTTTTTTGTTGTGCTTATTTATACGATATT

GAGCATTCATTAGTTACGGTGAGGATATTGGTTATTTAACTATACCTTTATTTAACTATGTCTTTAATATGA

ATGTTTCCAAATTGTATGTATGCAGACCAAAAGCCACATTGTGGGGTTTGGCCTGCATTTTTTTTGCCTA

GAATGCTATTCAAAATAGAAATTCAAGCAAAATAATATGCAGGAGATAATATAAATGGAAAAATACAACAAT

TGGAAACGAAAATTTTATGCAATATGGGCAGGGCAAGCAGTATCATTAATCACTAGTGCCATCCTGCAAATG

GCGATTATTTTTTACCTTACAGAAAAAACAGGATCTGCGATGGTCTTGTCTATGGCTTCATTAGTAGGTTTT

TTACCCTATGCGATTTTGGGACCTGCCATTGGTGTGCTAGTGGATCGTCATGATAGGAAGAAGATAATGATT

GGTGCCGATTTAATTATCGCAGCAGCTGGTGCAGTGCTTGCTATTGTTGCATTCTGTATGGAGCTACCTGTC

TGGATGATTATGATAGTATTGTTTATCCGTAGCATTGGAACAGCTTTTCATACCCCAGCACTCAATGCGGTT

ACACCACTTTTAGTACCAGAAGAACAGCTAACGAAATGCGCAGGCTATAGTCAGTCTTTGCAGTCTATAAGC

TATATTGTTAGTCCGGCAGTTGCAGCACTCTTATACTCCGTTTGGGATTTAAATGCTATTATTGCCATCGAC

GTATTGGGTGCTGTGATTGCATCTATTACGGTAGCAATTGTACGTATACCTAAGCTGGGTAATCAAGTGCAA

AGTTTAGAACCAAATTTCATAAGGGAGATGAAAGAAGGAGTTGTGGTTCTGAGACAAAACAAAGGATTGTTT

GCCTTATTACTCTTAGGAACACTATATACTTTTGTTTATATGCCAATCAATGCACTATTTCCTTTAATAAGC

ATGGAACACTTTAATGGAACGCCTGTGCATATTTCTATTACGGAAATTTCCTTTGCATTTGGGATGCTAGCA

GGAGGCTTATTATTAGGAAGATTAGGGGGCTTCGAAAAGCATGTATTACTAATAACAAGTTCATTTTTTATA

ATGGGGACCAGTTTAGCCGTTTCGGGAATACTTCCTCCAAATGGATTTGTAATATTCGTAGTTTGCTGTGCA

ATAATGGGGCTTTCGGTGCCATTTTATAGCGGTGTGCAAACAGCTCTTTTTCAGGAGAAAATTAAGCCTGAA

TATTTAGGACGTGTATTTTCTTTGATCGGAAGTATCATGTCACTTGCTATGCCAATTGGGTTAATTCTTTCT

GGATTCTTTGCTGATAAAATCGGTGTAAATCATTGGTTTTTACTATCAGGTATTTTAATTATTGGCATTGCT

ATAGTTTGCCAAATGATAACTGAGGTTAGAAAATTAGATTTAAAATAAACAATATTGGAGGAATATTTATGT

ATCTTATTTTCATGTAACTCTTCCTGCTAAAATCGCAGGGTTTTCCCTGCATACAAGCAAATGAAAGCATGC

GATTATAGACAGGAGGAAATGTTATGGAATTAATATT

AAAAGCAAAAGACATTCGTGTGGAATTCAAAGGACGCGATGTTTTAGATATAAATGAATTAGAAGTATATGA

TTATGACCGTATTGGTTTAGTAGGAGCAAATGGTGCTGGAAAAAGCACTTTACTCAGGGTACTTTTAGGAGA

ATTAACTCCCCCAGGATGTAAAATGAATCGTCTGGGTGAACTTGCCTATATTCCCCAGTTGGACGAAGTAAC

TCTGCAGGAGGAAAAAGATTTTGCACTTGTAGGCAAGCTAGGTGTTGAGCAATTAAATATACAGACTATGAG

CGGTGGTGAAGAAACAAGGCTTAAAATAGCACAGGCCTTATCGGCACAGGTTCATGGTATTTTAGCGGATGA

ACCTACGAGCCATTTAGACCGTGAAGGAATTGATTTTCTAATAGGACAGCTAAAATATTTTACAGGTGCACT

GTTAGTTATTAGCCATGACCGCTATTTTCTTGATGAAATAGTAGATAAAATATGGGAACTGAAAGATGGCAA

AATCACTGAGTATTGGGGAAACTATTCTGATTATCTTCGTCAGAAAGAGGAAGAACGTAAGAGCCAAGCTGC

AGAATACGAACAATTTATTGCGGAACGTGCCCGATTGGAAAGGGCTGCGGAGGAAAAGCGAAAACAGGCTCG

TAAAATAGAACAGAAGGCAAAAGGTTCTTCAAAGAAAAAAAGTACTGAAGACGGAGGGCGTTTAGCTCATCA

AAAATCAATAGGAAGTAAGGAAAAAAAGATGTATAATGCTGCTAAAACCCTAGAGCACAGGATTGCGGCCTT

AGGAAAAGTAGAAGCTCCGGAAGGCATTCGCAGAATTCGTTTCAGGCAAAGTAAAGCATTGGAGCTCCATAA

TCCATACCCTATAGTCGGTGCAGAAATTAATAAAGTATTTGGGGATAAGGCTCTGTTTGAAAATGCATCTTT

TCAAATTCCGTTAGGAGCAAAAGTGGCGTTAACTGGTGGTAATGGAATCGGAAAAACAACTTTAATCCAAAT

GATCTTAAACCATGAAGAAGGAATTTCTATTTCGCCTAAGGCAAAAATAGGTTACTTTGCACAGAATGGTTA

CAAGTACAACAGTAATCAGAATGTTATGGAGTTTATGCAGAAGGATTGTGACTACAATATATCAGAAATTCG

TTCAGTGCTAGCATCTATGGGGTTCAAACAGAACGATATTGGAAAAAGTTTATCTGTTTTAAGCGGTGGAGA

AATTATAAAATTGTTGCTTGCTAAAATGCTCATGGGTAGATATAACATCCTAATAATGGATGAACCCAGTAA

CTTCCTTGACATACCAAGTTTAGAGGCTTTGGAAATACTAATGAAGGAGTACACCGGAACTATCGTGTTTAT

CACCCACGATAAACGATTACTCGAAAATGTAGCAGATGTAGTTTATGAAATTAGAGATAAGAAAATAAATCT

GAAACATTAAATTTAAGGTAGTCGCTGGTCAGTATAGTCTGTTCTGGTTGGCGACTCCATTGTTAAAGAGTA

TAAAGACTTTAGATTTTATGAATATTAAAAATAGGAACAGTCAATTGAACTGCTCCTATTTTTCTGCTAAAT

ATATTGTAGTTTTCTTATATGTATAATGATAGATTAGCGGATTCTCATCTACGGTACTTACTTCAAATATGA

AGAAGTGATCGCGGTTATCTCTGGACTTTTCCTTATTGAGGACAAAGTAATTCTTACGTGAAGTCGCCATTG

TTTTTAGGATATCATCAGTTAGGAAGGTCAATGGAATATTCATGTTAGAGTAGCGGTAGAAGTCACGTTCAA

AATCTTGGTAGCTCTCGCTATAATAGTCCATTTGTAGGTGATTACGCTGAAACTCAAGCTGATTCATAGAGC

ACCTCCTCGACAAGTTCAATACTAATAATGTCTTTTAATTTCAAATTGATGTGACCTGTTGTAGTTTTTATC

AAAATGAAATCTTTGGTCAGACTTGGTATTGTTCCAGTGTAGGAAACACGCTTGTTTTTTTCAATCACTTGA

ATGCGTGTGCGTAGCTGCCCGGCGTATACTTGACTGAGGAGTAATAATTTCTTCTCTAGTGATAAGTCAGAC

ATGTACGTTACTTTGTTTGTATCATCAGAGAGTGCTGATGCATGTTCAGATAGGAAAAAGCCCATCCATTTT

TGCATCTTTGTATCCTGGTACTCTCTTGCTGATTGAAATGGTAAATATGAACGGTCAATCATATCAAATCCT

TTCTATGCAGAGGCAAGGGTATTTTTATCAAATTGAATCGTAAAACCTTGAATTCCCCCACCTGTGTAACAT

TCTTTAAAGCGATTGATTACCTCAGTATAGATTATCACAGATGAGCTTGTTGGCTTAATGCTAAATGTAAAT

TCCAATGGTAATCGGTTTTCAGATTTAGCATGTACTAGTCGTATCGATATTTCAGTTGTTTTGAGTTTTCTC

TGACGAAGTTTTGAAGTTGCTGTTTCAACGATTCCATGTAAAAATCTTTCAAGCATTTCAATATCATTACAT

CCTTTGCTACGGATTTCTGAAAATTGTACTGTATTTTTTTCTTGTTTCATTTTAATCCCTCCAATCCACCCG

CGGAATGACCACCGATAAGTTTACTGCGTTCAATATTTCTGGAACCTTCAGTTAGGACGGTTCCTTTTTGTA

TGGCTAAAAAACCAAACTGTTCTCTGACAACATCAATAGCTGTCTGAAGTCTATTATCTTTTTCAATTTGTT

CTACATCATCAAAGAGTGATAGTAGAGTATAGCTTTCATCTACGAAGCCACTATAAGATACACCAATTTGTC

TCACTGCACCAGAGGTGTATTTTTTCGGAATAATACAAGTACATGACTCACCATTGTTTTGGGGAGATTTG

CGGGTTCAATTTTATTCTGAGCATTTATAGATTTTTTCATCTCAGTCCTAGAATAGCCAATATGAATAGAAA

CGACAGTAGTCAATACTAGGGCTACTGTTCCGTTCCACAGTATCATTTAAAAATCATT-TTCACACCCTTTC

GTCTATTAGTATAGAAGAAAGCTCTCAGCACA

>GA49138/Mega-1.V

CATGTTGAGGCGGTAAGTTTGCTAGTCAAGGAGTAAAACGACGAAGATTAGCATTTACTTCCGCCCATGCGA

TAGCTGTCCGTGATTGACAAGTGCTAGCACGCAGACAGAACGGAGATAGCGAACCGCTGAGTGTGTCGCTCT

GCTCGTAAAAGCTTAGAAACCTTTGAACGAAAGGGATAATGAAAGCCTTGATTGCAAGGCTTTTTGCTTTAT

GGTGGGTAAGTATCAGAGTGAGAAAATTTTTGGAATGAGTAGAAGTGATAGCTAGAAATTATCAGTTTCTA

TTTCCATTTACCCTGTGGGTACGTGTTTGTTTCCATTGACAAGGAGTTTGTGGGAATAGAAATGT

ACCCACCTTGTTTGAATCAAGTGAAGTGTAGTTGAAGGAAATCTGTTGAAAGCAATACTTCATTTTACCG

AATAAGTAATAATTTAGGCAACTTCAAATCGATTAAAAAAAACTATTTTAAAGGTTAAGAGTAGACAAAA

ATTGTCCACTCTTTTTTGCAAACTCAATTTATCAATAAATGAAATGAGGGAATGTAAAATG

AAATATTTTGAGGTTGAGTTAGAAAATCCTGATGAATTTTTAAAACTACAAACAGAAGATTTTGTGAAAG

CTAATCGCTTGCTACTAAGGAAGATAATCCAGAGCGTTACAGTCTATGAAGAAAACTTCGTCATATCCTT

TAAATCTGGCATCGAATTGGAAGTATGAGTCTCATTCCATAACTTTTATATTGAACATATCATCTTGTTGTG

TTATACTATAAATTGATATAAACAAAGATGTAGGAGGAACCGAAACTATGACAGCCTCAATGCGTTTAAGAT

AAGCTGGCAATAAAAAAAGCAGAATCTATACCCGATGATAGGCTTTTTTGTTGTGCTTATTTATACGATATT

GAGCATTCATTAGTTACGGTGAGGATATTGGTTATTTAACTATACCTTTATTTAACTATGTCTTTAATATGA

ATGTTTCCAAATTGTATGTATGCAGACCAAAAGCCACATTGTGGGGTTTGGCCTGCATTTTTTTTGCCTA

GAATGCTATTCAAAATAGAAATTCAAGCAAAATAATATGCAGGAGATAATATAAATGGAAAAATACAACAAT

TGGAAACGAAAATTTTATGCAATATGGGCAGGGCAAGCAGTATCATTAATCACTAGTGCCATCCTGCAAATG

GCGATTATTTTTTACCTTACAGAAAAAACAGGATCTGCGATGGTCTTGTCTATGGCTTCATTAGTAGGTTTT

TTACCCTATGCGATTTTGGGACCTGCCATTGGTGTGCTAGTGGATCGTCATGATAGGAAGAAGATAATGATT

GGTGCCGATTTAATTATCGCAGCAGCTGGTGCAGTGCTTGCTATTGTTGCATTCTGTATGGAGCTACCTGTC

TGGATGATTATGATAGTATTGTTTATCCGTAGCATTGGAACAGCTTTTCATACCCCAGCACTCAATGCGGTT

ACACCACTTTTAGTACCAGAAGAACAGCTAACGAAATGCGCAGGCTATAGTCAGTCTTTGCAGTCTATAAGC

TATATTGTTAGTCCGGCAGTTGCAGCACTCTTATACTCCGTTTGGGATTTAAATGCTATTATTGCCATCGAC

GTATTGGGTGCTGTGATTGCATCTATTACGGTAGCAATTGTACGTATACCTAAGCTGGGTAATCAAGTGCAA

AGTTTAGAACCAAATTTCATAAGGGAGATGAAAGAAGGAGTTGTGGTTCTGAGACAAAACAAAGGATTGTTT

GCCTTATTACTCTTAGGAACACTATATACTTTTGTTTATATGCCAATCAATGCACTATTTCCTTTAATAAGC

ATGGAACACTTTAATGGAACGCCTGTGCATATTTCTATTACGGAAATTTCCTTTGCATTTGGGATGCTAGCA

GGAGGCTTATTATTAGGAAGATTAGGGGGCTTCGAAAAGCATGTATTACTAATAACAAGTTCATTTTTTATA

ATGGGGACCAGTTTAGCCGTTTCGGGAATACTTCCTCCAAATGGATTTGTAATATTCGTAGTTTGCTGTGCA

ATAATGGGGCTTTCGGTGCCATTTTATAGCGGTGTGCAAACAGCTCTTTTTCAGGAGAAAATTAAGCCTGAA

TATTTAGGACGTGTATTTTCTTTGATCGGAAGTATCATGTCACTTGCTATGCCAATTGGGTTAATTCTTTCT

GGATTCTTTGCTGATAAAATCGGTGTAAATCATTGGTTTTTACTATCAGGTATTTTAATTATTGGCATTGCT

ATAGTTTGCCAAATGATAACTGAGGTTAGAAAATTAGATTTAAAATAAACAATATTGGAGGAATATTTATGT

ATCTTATTTTCATGTAACTCTTCCTGCTAAAATCGCAGGGTTTTCCCTGCATACAAGCAAATGAAAGCATGC

GATTATAGACAGGAGGAAATGTTATGGAATTAATATT

AAAAGCAAAAGACATTCGTGTGGAATTCAAAGGACGCGATGTTTTAGATATAAATGAATTAGAAGTATATGA

TTATGACCGTATTGGTTTAGTAGGAGCAAATGGTGCTGGAAAAAGCACTTTACTCAGGGTACTTTTAGGAGA

ATTAACTCCCCCAGGATGTAAAATGAATCGTCTGGGTGAACTTGCCTATATTCCCCAGTTGGACGAAGTAAC

TCTGCAGGAGGAAAAAGATTTTGCACTTGTAGGCAAGCTAGGTGTTGAGCAATTAAATATACAGACTATGAG

CGGTGGTGAAGAAACAAGGCTTAAAATAGCACAGGCCTTATCGGCACAGGTTCATGGTATTTTAGCGGATGA

ACCTACGAGCCATTTAGACCGTGAAGGAATTGATTTTCTAATAGGACAGCTAAAATATTTTACAGGTGCACT

GTTAGTTATTAGCCATGACCGCTATTTTCTTGATGAAATAGTAGATAAAATATGGGAACTGAAAGATGGCAA

AATCACTGAGTATTGGGGAAACTATTCTGATTATCTTCGTCAGAAAGAGGAAGAACGTAAGAGCCAAGCTGC

AGAATACGAACAATTTATTGCGGAACGTGCCCGATTGGAAAGGGCTGCGGAGGAAAAGCGAAAACAGGCTCG

TAAAATAGAACAGAAGGCAAAAGGTTCTTCAAAGAAAAAAAGTACTGAAGACGGAGGGCGTTTAGCTCATCA

AAAATCAATAGGAAGTAAGGAAAAAAAGATGTATAATGCTGCTAAAACCCTAGAGCACAGGATTGCGGCCTT

AGGAAAAGTAGAAGCTCCGGAAGGCATTCGCAGAATTCGTTTCAGGCAAAGTAAAGCATTGGAGCTCCATAA

TCCATACCCTATAGTCGGTGCAGAAATTAATAAAGTATTTGGGGATAAGGCTCTGTTTGAAAATGCATCTTT

TCAAATTCCGTTAGGAGCAAAAGTGGCGTTAACTGGTGGTAATGGAATCGGAAAAACAACTTTAATCCAAAT

GATCTTAAACCATGAAGAAGGAATTTCTATTTCGCCTAAGGCAAAAATAGGTTACTTTGCACAGAATGGTTA

CAAGTACAACAGTAATCAGAATGTTATGGAGTTTATGCAGAAGGATTGTGACTACAATATATCAGAAATTCG

TTCAGTGCTAGCATCTATGGGGTTCAAACAGAACGATATTGGAAAAAGTTTATCTGTTTTAAGCGGTGGAGA

AATTATAAAATTGTTGCTTGCTAAAATGCTCATGGGTAGATATAACATCCTAATAATGGATGAACCCAGTAA

CTTCCTTGACATACCAAGTTTAGAGGCTTTGGAAATACTAATGAAGGAGTACACCGGAACTATCGTGTTTAT

CACCCACGATAAACGATTACTCGAAAATGTAGCAGATGTAGTTTATGAAATTAGAGATAAGAAAATAAATCT

GAAACATTAAATTTAAGGTAGTCGCTGGTCAGTATAGTCTGTTCTGGTTGGCGACTCCATTGTTAAAGAGTA

TAAAGACTTTAGATTTTATGAATATTAAAAATAGGAACAGTCAATTGAACTGCTCCTATTTTTCTGCTAAAT

ATATTGTAGTTTTCTTATATGTATAATGATAGATTAGCGGATTCTCATCTACGGTACTTACTTCAAATATGA

AGAAGTGATCGCGGTTATCTCTGGACTTTTCCTTATTGAGGACAAAGTAATTCTTACGTGAAGTCGCCATTG

TTTTTAGGATATCATCAGTTAGGAAGGTCAATGGAATATTCATGTTAGAGTAGCGGTAGAAGTCACGTTCAA

AATCTTGGTAGCTCTCGCTATAATAGTCCATTTGTAGGTGATTACGCTGAAACTCAAGCTGATTCATAGAGC

ACCTCCTCGACAAGTTCAATACTAATAATGTCTTTTAATTTCAAATTGATGTGACCTGTTGTAGTTTTTATC

AAAATGAAATCTTTGGTCAGACTTGGTATTGTTCCAGTGTAGGAAACACGCTTGTTTTTTTCAATCACTTGA

ATGCGTGTGCGTAGCTGCCCGGCGTATACTTGACTGAGGAGTAATAATTTCTTCTCTAGTGATAAGTCAGAC

ATGTACGTTACTTTGTTTGTATCATCAGAGAGTGCTGATGCATGTTCAGATAGGAAAAAGCCCATCCATTTT

TGCATCTTTGTATCCTGGTACTCTCTTGCTGATTGAAATGGTAAATATGAACGGTCAATCATATCAAATCCT

TTCTATGCAGAGGCAAGGGTATTTTTATCAAATTGAATCGTAAAACCTTGAATTCCCCCACCTGTGTAACAT

TCTTTAAAGCGATTGATTACCTCAGTATAGATTATCACAGATGAGCTTGTTGGCTTAATGCTAAATGTAAAT

TCCAATGGTAATCGGTTTTCAGATTTAGCATGTACTAGTCGTATCGATATTTCAGTTGTTTTGAGTTTTCTC

TGACGAAGTTTTGAAGTTGCTGTTTCAACGATTCCATGTAAAAATCTTTCAAGCATTTCAATATCATTACAT

CCTTTGCTACGGATTTCTGAAAATTGTACTGTATTTTTTTCTTGTTTCATTTTAATCCCTCCAATCCACCCG

CGGAATGACCACCGATAAGTTTACTGCGTTCAATATTTCTGGAACCTTCAGTTAGGACGGTTCCTTTTTGTA

TGGCTAAAAAACCAAACTGTTCTCTGACAACATCAATAGCTGTCTGAAGTCTATTATCTTTTTCAATTTGTT

CTACATCATCAAAGAGTGATAGTAGAGTATAGCTTTCATCTACGAAGCCACTATAAGATACACCAATTTGTC

TCACTGCACCAGAGGTGTATTTTTTCGGAATAATACAAGTACATGACTCACCATTGTTTTGGGGAGATTTG

CGGGTTCAATTTTATTCTGAGCATTTATAGATTTTTTCATCTCAGTCCTAGAATAGCCAATATGAATAGAAA

CGACAGTAGTCAATACTAGGGCTACTGTTCCGTTCCACAGTATCATTTAAAAATCATT-TTCACACCCTTTC

GTCTATTAGTATAGAAGAAAGCTCTCAGCACA

>5652-06/Mega-1.V

CATGTTGAGGCGGTAAGTTTGCTAGTCAAGGAGTAAAACGACGAAGATTAGCATTTACTTCCGCCCATGCGA

TAGCTGTCCGTGATTGACAAGTGCTAGCACGCAGACAGAACGGAGATAGCGAACCGCTGAGTGTGTCGCTCT

GCTCGTAAAAGCTTAGAAACCTTTGAACGAAAGGGATAATGAAAGCCTTGATTGCAAGGCTTTTTGCTTTAT

GGTGGGTAAGTATCAGAGTGAGAAAATTTTTGGAATGAGTAGAAGTGATAGCTAGAAATTATCAGTTTCTA

TTTCCATTTACCCTGTGGGTACGTGTTTGTTTCCATTGACAAGGAGTTTGTGGGAATAGAAATGT

ACCCACCTTGTTTGAATCAAGTGAAGTGTAGTTGAAGGAAATCTGTTGAAAGCAATACTTCATTTTACCG

AATAAGTAATAATTTAGGCAACTTCAAATCGATTAAAAAAAACTATTTTAAAGGTTAAGAGTAGACAAAA

ATTGTCCACTCTTTTTTGCAAACTCAATTTATCAATAAATGAAATGAGGGAATGTAAAATG

AAATATTTTGAGGTTGAGTTAGAAAATCCTGATGAATTTTTAAAACTACAAACAGAAGATTTTGTGAAAG

CTAATCGCTTGCTACTAAGGAAGATAATCCAGAGCGTTACAGTCTATGAAGAAAACTTCGTCATATCCTT

TAAATCTGGCATCGAATTGGAAGTATGAGTCTCATTCCATAACTTTTATATTGAACATATCATCTTGTTGTG

TTATACTATAAATTGATATAAACAAAGATGTAGGAGGAACCGAAACTATGACAGCCTCAATGCGTTTAAGAT

AAGCTGGCAATAAAAAAAGCAGAATCTATACCCGATGATAGGCTTTTTTGTTGTGCTTATTTATACGATATT

GAGCATTCATTAGTTACGGTGAGGATATTGGTTATTTAACTATACCTTTATTTAACTATGTCTTTAATATGA

ATGTTTCCAAATTGTATGTATGCAGACCAAAAGCCACATTGTGGGGTTTGGCCTGCATTTTTTTTGCCTA

GAATGCTATTCAAAATAGAAATTCAAGCAAAATAATATGCAGGAGATAATATAAATGGAAAAATACAACAAT

TGGAAACGAAAATTTTATGCAATATGGGCAGGGCAAGCAGTATCATTAATCACTAGTGCCATCCTGCAAATG

GCGATTATTTTTTACCTTACAGAAAAAACAGGATCTGCGATGGTCTTGTCTATGGCTTCATTAGTAGGTTTT

TTACCCTATGCGATTTTGGGACCTGCCATTGGTGTGCTAGTGGATCGTCATGATAGGAAGAAGATAATGATT

GGTGCCGATTTAATTATCGCAGCAGCTGGTGCAGTGCTTGCTATTGTTGCATTCTGTATGGAGCTACCTGTC

TGGATGATTATGATAGTATTGTTTATCCGTAGCATTGGAACAGCTTTTCATACCCCAGCACTCAATGCGGTT

ACACCACTTTTAGTACCAGAAGAACAGCTAACGAAATGCGCAGGCTATAGTCAGTCTTTGCAGTCTATAAGC

TATATTGTTAGTCCGGCAGTTGCAGCACTCTTATACTCCGTTTGGGATTTAAATGCTATTATTGCCATCGAC

GTATTGGGTGCTGTGATTGCATCTATTACGGTAGCAATTGTACGTATACCTAAGCTGGGTAATCAAGTGCAA

AGTTTAGAACCAAATTTCATAAGGGAGATGAAAGAAGGAGTTGTGGTTCTGAGACAAAACAAAGGATTGTTT

GCCTTATTACTCTTAGGAACACTATATACTTTTGTTTATATGCCAATCAATGCACTATTTCCTTTAATAAGC

ATGGAACACTTTAATGGAACGCCTGTGCATATTTCTATTACGGAAATTTCCTTTGCATTTGGGATGCTAGCA

GGAGGCTTATTATTAGGAAGATTAGGGGGCTTCGAAAAGCATGTATTACTAATAACAAGTTCATTTTTTATA

ATGGGGACCAGTTTAGCCGTTTCGGGAATACTTCCTCCAAATGGATTTGTAATATTCGTAGTTTGCTGTGCA

ATAATGGGGCTTTCGGTGCCATTTTATAGCGGTGTGCAAACAGCTCTTTTTCAGGAGAAAATTAAGCCTGAA

TATTTAGGACGTGTATTTTCTTTGATCGGAAGTATCATGTCACTTGCTATGCCAATTGGGTTAATTCTTTCT

GGATTCTTTGCTGATAAAATCGGTGTAAATCATTGGTTTTTACTATCAGGTATTTTAATTATTGGCATTGCT

ATAGTTTGCCAAATGATAACTGAGGTTAGAAAATTAGATTTAAAATAAACAATATTGGAGGAATATTTATGT

ATCTTATTTTCATGTAACTCTTCCTGCTAAAATCGCAGGGTTTTCCCTGCATACAAGCAAATGAAAGCATGC

GATTATAGACAGGAGGAAATGTTATGGAATTAATATT

AAAAGCAAAAGACATTCGTGTGGAATTCAAAGGACGCGATGTTTTAGATATAAATGAATTAGAAGTATATGA

TTATGACCGTATTGGTTTAGTAGGAGCAAATGGTGCTGGAAAAAGCACTTTACTCAGGGTACTTTTAGGAGA

ATTAACTCCCCCAGGATGTAAAATGAATCGTCTGGGTGAACTTGCCTATATTCCCCAGTTGGACGAAGTAAC

TCTGCAGGAGGAAAAAGATTTTGCACTTGTAGGCAAGCTAGGTGTTGAGCAATTAAATATACAGACTATGAG

CGGTGGTGAAGAAACAAGGCTTAAAATAGCACAGGCCTTATCGGCACAGGTTCATGGTATTTTAGCGGATGA

ACCTACGAGCCATTTAGACCGTGAAGGAATTGATTTTCTAATAGGACAGCTAAAATATTTTACAGGTGCACT

GTTAGTTATTAGCCATGACCGCTATTTTCTTGATGAAATAGTAGATAAAATATGGGAACTGAAAGATGGCAA

AATCACTGAGTATTGGGGAAACTATTCTGATTATCTTCGTCAGAAAGAGGAAGAACGTAAGAGCCAAGCTGC

AGAATACGAACAATTTATTGCGGAACGTGCCCGATTGGAAAGGGCTGCGGAGGAAAAGCGAAAACAGGCTCG

TAAAATAGAACAGAAGGCAAAAGGTTCTTCAAAGAAAAAAAGTACTGAAGACGGAGGGCGTTTAGCTCATCA

AAAATCAATAGGAAGTAAGGAAAAAAAGATGTATAATGCTGCTAAAACCCTAGAGCACAGGATTGCGGCCTT

AGGAAAAGTAGAAGCTCCGGAAGGCATTCGCAGAATTCGTTTCAGGCAAAGTAAAGCATTGGAGCTCCATAA

TCCATACCCTATAGTCGGTGCAGAAATTAATAAAGTATTTGGGGATAAGGCTCTGTTTGAAAATGCATCTTT

TCAAATTCCGTTAGGAGCAAAAGTGGCGTTAACTGGTGGTAATGGAATCGGAAAAACAACTTTAATCCAAAT

GATCTTAAACCATGAAGAAGGAATTTCTATTTCGCCTAAGGCAAAAATAGGTTACTTTGCACAGAATGGTTA

CAAGTACAACAGTAATCAGAATGTTATGGAGTTTATGCAGAAGGATTGTGACTACAATATATCAGAAATTCG

TTCAGTGCTAGCATCTATGGGGTTCAAACAGAACGATATTGGAAAAAGTTTATCTGTTTTAAGCGGTGGAGA

AATTATAAAATTGTTGCTTGCTAAAATGCTCATGGGTAGATATAACATCCTAATAATGGATGAACCCAGTAA

CTTCCTTGACATACCAAGTTTAGAGGCTTTGGAAATACTAATGAAGGAGTACACCGGAACTATCGTGTTTAT

CACCCACGATAAACGATTACTCGAAAATGTAGCAGATGTAGTTTATGAAATTAGAGATAAGAAAATAAATCT

GAAACATTAAATTTAAGGTAGTCGCTGGTCAGTATAGTCTGTTCTGGTTGGCGACTCCATTGTTAAAGAGTA

TAAAGACTTTAGATTTTATGAATATTAAAAATAGGAACAGTCAATTGAACTGCTCCTATTTTTCTGCTAAAT

ATATTGTAGTTTTCTTATATGTATAATGATAGATTAGCGGATTCTCATCTACGGTACTTACTTCAAATATGA

AGAAGTGATCGCGGTTATCTCTGGACTTTTCCTTATTGAGGACAAAGTAATTCTTACGTGAAGTCGCCATTG

TTTTTAGGATATCATCAGTTAGGAAGGTCAATGGAATATTCATGTTAGAGTAGCGGTAGAAGTCACGTTCAA

AATCTTGGTAGCTCTCGCTATAATAGTCCATTTGTAGGTGATTACGCTGAAACTCAAGCTGATTCATAGAGC

ACCTCCTCGACAAGTTCAATACTAATAATGTCTTTTAATTTCAAATTGATGTGACCTGTTGTAGTTTTTATC

AAAATGAAATCTTTGGTCAGACTTGGTATTGTTCCAGTGTAGGAAACACGCTTGTTTTTTTCAATCACTTGA

ATGCGTGTGCGTAGCTGCCCGGCGTATACTTGACTGAGGAGTAATAATTTCTTCTCTAGTGATAAGTCAGAC

ATGTACGTTACTTTGTTTGTATCATCAGAGAGTGCTGATGCATGTTCAGATAGGAAAAAGCCCATCCATTTT

TGCATCTTTGTATCCTGGTACTCTCTTGCTGATTGAAATGGTAAATATGAACGGTCAATCATATCAAATCCT

TTCTATGCAGAGGCAAGGGTATTTTTATCAAATTGAATCGTAAAACCTTGAATTCCCCCACCTGTGTAACAT

TCTTTAAAGCGATTGATTACCTCAGTATAGATTATCACAGATGAGCTTGTTGGCTTAATGCTAAATGTAAAT

TCCAATGGTAATCGGTTTTCAGATTTAGCATGTACTAGTCGTATCGATATTTCAGTTGTTTTGAGTTTTCTC

TGACGAAGTTTTGAAGTTGCTGTTTCAACGATTCCATGTAAAAATCTTTCAAGCATTTCAATATCATTACAT

CCTTTGCTACGGATTTCTGAAAATTGTACTGTATTTTTTTCTTGTTTCATTTTAATCCCTCCAATCCACCCG

CGGAATGACCACCGATAAGTTTACTGCGTTCAATATTTCTGGAACCTTCAGTTAGGACGGTTCCTTTTTGTA

TGGCTAAAAAACCAAACTGTTCTCTGACAACATCAATAGCTGTCTGAAGTCTATTATCTTTTTCAATTTGTT

CTACATCATCAAAGAGTGATAGTAGAGTATAGCTTTCATCTACGAAGCCACTATAAGATACACCAATTTGTC

TCACTGCACCAGAGGTGTATTTTTTCGGAATAATACAAGTACATGACTCACCATTGTTTTGGGGAGATTTG

CGGGTTCAATTTTATTCTGAGCATTTATAGATTTTTTCATCTCAGTCCTAGAATAGCCAATATGAATAGAAA

CGACAGTAGTCAATACTAGGGCTACTGTTCCGTTCCACAGTATCATTTAAAAATCATT-TTCACACCCTTTC

GTCTATTAGTATAGAAGAAAGCTCTCAGCACA

>7286-06/Mega-1.V
[truncated: 193,325 more chars]
