# Supplementary material for: Composite mobile genetic elements disseminating macrolide resistance in Streptococcus pneumoniae
Source: Front Microbiol. 2015 Feb 9;6:26. doi: 10.3389/fmicb.2015.00026 (PMC4321634; doi:10.3389/fmicb.2015.00026)
Supplement: Supplementary file 1 [file DataSheet1.ZIP › Supplementary Material/Data File S3.DOCX]

>GA02506/Tn916

AAAATAGCATAAAAATCTAGTTATCCGCATAAAAACTGGACTTATCACACTTTATCAAGG

TCAAAACCACTCAATTTACTACTAATTTACTACTTATGAATGAGCTTTGATACGACGATT

TATCCTTGAAAAGTGAAGATATAAAGATACTTCCAATAAAATTTGAATATTTAATAGGTA

GACACTTCAAAAAATGAGGTGTCTATTTTTTTACCCGATTTTGAAAGGAAGTGAACTTAT

GAAAACAAAAAATCAAGAATCAAAAGGTCGTTCCCCACTCTTTAAGACCATCAAACATTC

ATTCAGCCAATAAAAAAGAAAGGATAGGTAAAAATATGGAACTTAAATTTGTGATTCCCA

ACATGGAAAAAACATTCGGCAATTTAGAATTTGCTGGCGAGGATAAAGTCGTTCAGCGAA

GAATCAACGGACGGCTAACTGTCTTATCAAGAAGCTATAATCTCTATTCTGATGTTCAAA

GAGCAGATGATATTGTGGTGGTGCTTCCTGCTGAAGCTGGCGAAAAACATTTCGGCTTTG

AGGAACGTGTGAAGTTAGTCAATCCACGTATTACCGCAGAGGGCTACAAAATCGGCACTC

GTGGTTTTACAAATTACCTTTTACATGCTGACGACATGATAAAAGAATAAAGAAAGAGAG

GAAAAATGATGAGATTAGCAAATGGCATTGTATTAGATAAAGACACGACTTTTGGAGAAT

TGAAATTCTCTGCTCTACGTCGTGAAGTGAGAATCCAAAATGAAGACGGGTCGGTTTCAG

ATGAAATCAAGGAACGTACCTATGACTTAAAATCCAAAGGACAAGGACGCATGATTCAAG

TAAGTATTCCTGCCAGCGTGCCTTTGAAAGAGTTTGATTATAACGCACGGGTGGAACTTA

TCAATCCCATTGCGGACACCGTTGCTACTGCCACCTATCAAGGAGCAGATGTTGACTGGT

ATATCAAGGCAGACGATATTGTGCTGACAAAGGATTCTAGTTCATTCAAAGCTCAACCAC

AAGCAAAGAAAGAACCGACACAAGACAAATAGTCGCTAGGTAGAAAGGAGACTTTTTCGC

ATGAAACAGCGTGGTAAAAGGATTCGCCCATCTGGTAAAGATTTAGTCTTTCATTTTACG

ATAGCGTCACTCCTGCCTGTTTTCCTGCTGGTTGTCGGACTGTTTCATGTGAAGACAATC

CAGCAGATCAACTGGCAGGATTTTAACCTATCACAAGCAGATAAGATTGACATTCCCTAT

TTAATTATCAGTTTCAGTGTCGCAATTCTTATCTGCTTGCTGGTAGCGTTTGTATTCAAA

CGGGTTCGCTATGATACGGTTAAACAACTTTACCACCGTCAAAAACTGGCAAAGATGATA

CTTGAAAACAAGTGGTATGAATCTGAACAGGTCAAAACAGAGGGTTTCTTTAAAGATAGT

GCTGGTCGTACAAAGGAAAAGATAACCTACTTCCCTAAAATGTATTATCGACTTAAAAAT

GGCTTGATACAGATACGGGTGGAAATCACGCTGGGAAAATATCAAGACCAACTCTTACAC

TTGGAAAAGAAATTAGAGAGTGGCTTGTACTGTGAGCTGACGGATAAAGAGTTAAAGGAT

TCCTATGTGGAATATACTTTGCTCTATGACACCATAGCCAGTCGTATTTCTATTGATGAA

GTAGAAGCTAAAGATGGTAAACTTCGCTTAATGAAAAACGTATGGTGGGAATATGATAAG

CTCCCTCATATGTTGATTGCTGGTGGTACAGGTGGCGGTAAAACTTACTTTATACTGACA

CTGATTGAAGCCTTGCTTCATACAGATTCAAAACTGTATATTCTTGACCCGAAAAATGCT

GACCTTGCGGACTTAGGTTCTGTGATGGCAAATGTCTACTATAGAAAAGAAGACTTGCTT

TCTTGCATTGAAACATTCTATGAAGAAATGATGAAACGTAGTGAGGAAATGAAGCAGATG

AAGAACTATAAGACTGGCAAAAATTATGCTTACTTAGGTCTCCCGGCACACTTCTTAATC

TTTGATGAATACGTCGCTTTCATGGAAATGCTGGGAACAAAAGAAAACACCGCAGTTATG

AATAAGCTGAAACAGATTGTCATGTTAGGTCGTCAAGCTGGCTTCTTTCTAATACTGGCT

TGTCAACGTCCAGACGCAAAATATTTAGGCGACGGAATCCGTGATCAGTTTAATTTCAGA

GTGGCTTTAGGTCGTATGTCTGAAATGGGCTATGGCATGATGTTTGGCAGTGACGTACAA

AAGGATTTCTTCTTAAAGCGAATCAAAGGTCGTGGCTATGTTGATGTAGGAACAAGTGTC

ATATCAGAGTTTTATACTCCCCTTGTACCAAAAGGATATGATTTCTTGGAGGAAATTAAA

AAGTTATCCAACAGCAGACAGTCCACGCAGGCGACGTGCGAAGCGGAAGTCGCAGGTGTG

GACTGATCTTGCTGGCTGGTGTGGCAATAGCCACGCCAGCACTTAACCCCCCGTATCTAA

CAGGGGGGTACAAATCGACAGGAAACAGTCAAAAAAACATTAGAAAATCCTTTGGTTACA

AGGGATTTACAAAATTTCAGCGTATGTCAAATGGGCTTTAAAAGTTGACATACGCCTTTT

TGATTGGAGGGATTTTTACTGAATGAACAAACTTGGTTACAGCATTTAAAAGAAAAACGC

TTGGCTTATGGACTATCTCAAAACCGTTTAGCTGTTGCGACTGGTATTACAAGGCAGTAT

CTAAGCGATATTGAAACAGGAAAAGTCAAGCCATCAGAGGATTTACAGCAGTCCCTTTGG

GAAGCTCTGGAACGCTTCAATCCCGACGCTCCCCTTGAAATGCTGTTTGATTATGTAAGG

ATTCGCTTTCCGACAACAGACGTACAGCAGGTGGTCGAAAACATCTTACAACTGAAACTG

TCCTATTTTCTTCATGAGGACTATGGTTTCTATTCTTATTCAGAGCATTATGCTTTAGGC

GACATATTCGTCCTTTGCTCCCATGAACTGGACAAAGGAGTTCTGGTGGAATTGAAAGGT

CGTGGGTGCAGACAATTTGAAAGCTATCTTCTGGCACAACAAAGAAGCTGGTATGAGTTC

TTTATGGACGTTTTGGTGGCTGGCGGTGTGATGAAACGCCTTGACCTTGCCATTAACGAT

AAGACAGGGATTTTGAATATCCCTGTACTCACTGAAAAGTGCCAACAGGAAGAATGTATC

TCCGTCTTCCGCAGTTTTAAAAGCTATCGCAGTGGCGAACTGGTACGCAAAGAGGAAAAG

GAATGTATGGGAAACACCCTCTATATCGGTTCATTACAAAGTGAAGTTTATTTCTGTATC

TATGAAAAGGACTACGAGCAGTACAAGAAAAATGATATTCCCATTGAAGACGCAGAAGTA

AAAAACCGTTTTGAGATTCGATTGAAAAATGAGCGTGCCTATTATGCAGTCCGTGATTTA

CTCGTCTATGACAATCCAGAGCATACCGCCTTTAAAATTATCAATCGGTATATCCGTTTT

GTAGATAAAGACGATTCCAAACCTCGTTCTGATTGGAAACTGAATGAAGAATGGGCTTGG

TTTATTGGGAACAATCGTGAACGATTAAAACTAACCACAAAACCAGAGCCTTACTCCTTC

CAAAGGACGCTGAACTGGCTATCTCATCAAGTTGCCCCGACCTTAAAGGTTGCGATTAAA

CTTGATGAAATCAACCAGACGCAGGTTGTAAAAGACATTCTCGACCATGCGAAACTGACA

GACCGACACAAGCAGATTTTGAAGCAACAGTCAGTAAAAGAACAGGACGTGATAACAACA

AAAAAATAACTCAAATACAAATTCATTGAATATAGAGAGGAGAACATTTTTATGAATTTT

GGACAAAACCTTTATAACTGGTTTCTATCAAACGCTCAATCACTGGTGCTTTTAGCAATC

GTTGTGATTGGCTTGTATCTTGGCTTCAAGCGTGAGTTTAGCAAACTGATTGGCTTTTTA

ATTATTGCGATTATTGCGGTTGGCTTAGTCTTCAACGCTGCTGGAGTAAAAGACATTTTA

CTAGAGCTATTCAATCGCATTATTGGTGCTTAAATAAAACCGTTCTTTTGTGGAATATAA

GTGGTTTTCTTATGTTCCGCAAAGGAATGGTACACCAAACGAAGTGCGGTAGGGATTTTT

GAATCTCTACAAAGAAAGGACGTGAATATATGGACGATATGCAAGTCTATATTGCGAATT

TAGGCAAATACAATGAGGGCGAATTGGTCGGTGCGTGGTTTACCTTTCCCATTGACTTTG

AGGAAGTCAAAGAGAAAATCGGCTTGAATGATGAATATGAGGAATACGCCATTCATGACT

ACGAGTTACCCTTTACGGTTGACGAATACACTTCCATTGGCGAACTCAATCGACTATGGG

AAATGGTATCGGAATTACCCGAAGAATTACAATCGGAGCTATCTGCTCTGCTCACTCATT

TTTCAAGCATTGAAGAACTAAGCGAACATCAAGAGGATATTATCATTCATTCCGATTGTG

ATGATATGTATGACGTGGCACGCTACTACATTGAAGAAACGGGTGCTTTAGGCGAAGTAC

CAGCTAGTCTTCAAAACTATATTGATTATCAAGCCTATGGTCGGGATTTAGACCTTTCAG

GAACGTTTATCTCAACCAATCATGGGATTTTTGAAATCGTCTATTAAATCTGTCGGTACA

TTACTACTGGCAGATTTTCTATTTTACGGGGTGGCTCAATCAGCTACCCCTATTTTTTAT

GAAAGGATTGATTACATGAAGAAAATACGAAGCTATACCAGTATCTGGTCTGTGGAAAAG

GTACTGTATTCTATCAATGATTTTAGACTTCCGTTTCCCATAACCTTTACGCAAATGACA

TGGTTTGTCGTGTCACTCTTTGCAGTGATGATACTTGGCAACTTGCCCCCTCTTTCCATG

ATAGAGGGAGCATTTCTCAAATACTTTGGGATTCCTGTGGCTTTCACATGGTTTATGTCT

ACAAAAACTTTTGATGGTAAAAAGCCTTATGGATTTTTGAAGTCTGTCATTGCTTATGCA

CTGCGACCAAAGCTGACCTATGCAGGAAAAAAAGTAACGCTTGGCAGAAACCAGCCACAA

GAAGCCATTACAGCAGTTAGGAGTGAATTTTATGGCATATCCAATTAAATACATTGAAAA

CAATCTCGTCTGGAATAAAGACGGGGAATGTTATGCTTACTATGAGCTTGTTCCTTACAA

TTACTCATTTCTAAGTCCAGAACAGAAAATACAAGTGCATGATTCTTTCAGACAGCTTAT

CGCACAAAATCGTGATGGCAAAATTCATGCTTTACAAATCAGTACAGAATCCAGCATACG

TTCTGCACAAGAGCGTTCCAAAAATGAAGTCACTGGCAAGCTCAAAGCGGTTGCCTATGA

CAAAATCGACCAACAGACAGACGCTTTAATATCCATGATTGGCGAAAATCAAGTGAACTA

CCGTTTCTTTATCGGCTTTAAGTTGCTTCTCAACGATCAGGAGTTTTCTATGAAAAGTCT

TACCGTTGAAGCAAAAAATGCTTTGTCTGATTTTGTCTATGATGTGAACCATAAGCTGAT

GGGCGATTTTGTTAGTATGAGTAATGATGAAATCCTGCGTTTTCAGAAGATGGAAAAGCT

CTTAGAAAATAAAATCTCTCGTCGTTTCAAAATCCGCAGGTTAGATAAGGACGACTTCGG

CTATCTGATTGAACACCTTTACGGACAGACAGGCACTGCCTATGAAGAGTATGAGTACCA

TCTATCAAAGAAAAAGCTGGATAATGAAACGCTGATTAAATACTATGACTTGATTAAGCC

TACTCGCTGTTTGGTGGAAGAAAAACAGCGATATTTGAAAATCCAGCAGGAAGATGAAAC

CGTCTATGTAGCTTACTTTACCATTAACAGCATTGTCGGAGAACTGGACTTCCCGTCCTC

TGAAATCTTCTACTACCAGCAACAGCAATTTACATTCCCGATTGATACGTCAATGAATGT

GGAAATTGTAGCGAATCGTAAAGCCCTATCTACTGTCCGCAATAAAAAGAAAGAACTGAA

AGACTTGGATAACCACGCTTGGCAAAGTGATAATGAAACCAGCTCCAATGTGGCGGAAGC

TCTGGAAAGTGTGAATGAGCTGGAAACCAATTTAGACCAAAGCAAGGAATCTATGTACAA

GCTGTCTTATGTGGTAAGGGTATCAGCAAATGATCTTGACGAACTCAAACGTCGTTGTAA

TGAAGTGAAAGATTTTTATGACGATTTAAGCGTAAAACTGGTACGACCATTTGGGGATAT

GCTCGGCTTACATGAAGAATTTTTACCTGCCAGCAAGCGTTATATGAATGATTATATTCA

ATACGTGACCTCTGATTTCCTCGCTGGTTTAGGTTTTGGTGCTACTCAAATGCTGGGGGA

AAATGAGGGGATTTATGTTGGCTACAGCTTAGATACTGGACGCAATGTCTATCTGAAACC

TGCTCTTGCCAGTCAAGGGGTTAAGGGTTCAGTAACCAATGCGTTAGCGTCGGCTTTTGT

TGGTTCGCTGGGTGGTGGTAAATCCTTTGCGAATAACCTTATCGTCTATTATGCGGTGCT

TTATGGGGCACAAGCAGTGATTGTAGACCCAAAAGCAGAACGTGGCAGATGGAAAGAAAC

CTTGCCAGAGATTTCCCATGAAATCAATATCGTCACTCTGACTTCTGATGAGAAAAACAA

AGGCTTACTTGACCCTTATGTGATTATGAAAAATCCCAAAGATTCTGAATCACTGGCTAT

TGATATTCTGACATTCCTTACGGGGATTTCCTCTCGTGATGGGGAACGCTTCCCAATCCT

TAGAAAAGCCATTCGTGCAGTAACCAATAGTGAAGTACGAGGGTTGATGAAAGTGATTGA

GGAATTACGGGTTGAGAATACGCCACTAAGTACCAGTATAGCCGACCATATCGAAAGTTT

TACAGACTATGACTTTGCACATTTATTATTCAGTAATGGTTATGTGGAGCAGTCTATCAG

CTTAGAAAAACAACTGAACATTATACAGGTTGCGGACTTGGTACTTCCCGACAAGGAAAC

TTCCTTTGAGGAATATACCACTATGGAGCTTTTATCCGTTGCTATGCTGATTGTCATTAG

TACCTTTGCTTTAGACTTTATCCATACAGACCGAAGCATTTTCAAGATTGTAGATTTAGA

CGAAGCATGGAGCTTTTTACAGGTAGCACAAGGAAAAACACTATCTATGAAGCTGGTTCG

GGCTGGTCGTGCTATGAACGCTGGGGTATATTTCGTGACCCAAAATACAGACGACCTCTT

AGATGAAAAACTGAAAAATAACCTCGGCTTAAAATTTGCATTTCGTTCCACTGACCTTAA

CGAGATTAAAAAGACCTTAGCCTTTTTTGGTGTAGACCCAGAGGACGAAAACAATCAGAA

GCGATTGCGTGATTTGGAAAACGGGCAATGCCTTATCAGTGATTTATATGGTCGTGTCGG

TGTGATACAGTTCCACCCTGTATTTGAAGAACTGCTCCATGCCTTTGATACCAGACCACC

TGTGCGAAAAGAGGTGTAAATGTGAAACCATCAATAGTAAACAGAATAAAATCAAACTGG

ACGCTGAAACGTCTAGGTAAAGTGGCAATGACAGTGGCTTTCACACTTGTGATTGCCATT

TTTCTTTTAGCCATGCTGGGAACGGTGGTTCAAGCTGCGGGCTTGGTAGATGATACGGTC

AATGTGGCAAATGAATACAGCCGATACCCACTTGAAAACTATCAACTGGATTTTTATGTG

GATAATAGCTGGGGCTGGCTTCCGTGGAACTGGTCGGACGGGATTGGAAAACAGGTCATG

TATGGACTATATGCCATTACCAATTTTATTTGGACAATCAGTTTGTATGTTTCCAATGCG

ACAGGTTACTTAGTACAGGAAGCCTATTCCTTAGACTTCATTTCCGCTACAGCAGATTCC

ATTGGTAAGAATATGCAGACCTTAGCTGGTGTGAGTGCAAACGGATTTTCAACAGAGGGT

TTCTATGTTGGATTCCTCTTACTCTTGATTTTGGTTCTTGGGGTTTATGTTGCCTATACG

GGACTGATAAAGAGAGAAACCACAAAGGCAATTCATGCCATTATGAATTTTGTGCTGGTG

TTTATCCTATCGGCTTCCTTTATTGCCTACGCTCCCGACTACATTAAAAAAATCAATGAC

TTTTCATCAGACATCAGTAATGCCAGTTTATCACTTGGCACGAAGATTGTCATGCCCCAT

TCCGATAGTCAAGGCAAGGACAGCGTGGACTTAATCAGAGATAGCCTGTTTTCCATACAG

GTTCAGCAACCGTGGCTACTGCTTCAATACAACAGTTCAGACATTGAAAGTATCGGTATT

GACCGTGTGGAAAGCCTGCTCTCCACCAGCCCAGATTCCAACAATGGCGAAGACAGAGAA

AAAATTGTTGCGGAAGAAATTGAAGACAGAAGCAATACCAATCTAACCATTACAAAGACC

ATTAACCGTTTAGGTACAGTCTTCTTCCTATTTGTCTTCAATATTGGGATTTCCATATTT

GTATTCCTATTAACAGGAATCATGATTTTCTCGCAGGTACTTTTTATCATCTATGCTATG

TTTCTGCCTGTGAGCTTTATTTTAAGCATGATTCCATCATTTGATGGTATGTCAAAACGA

GCCATAACAAAGCTCTTTAATACCATTTTGACACGAGCTGGAATCACATTGATTATTACG

ACAGCATTTAGTATTTCAACCATGCTCTATACCTTATCGGCTGGTTATCCGTTCTTTTTG

ATTGCTTTTCTACAGATTGTGACCTTTGCAGGAATCTACTTCAAGCTGGGCGATTTAATG

AGTATGTTTTCTCTACAGAGTAACGATTCTCAAAGTGTGGGAAGTCGTGTGATGAGAAAA

CCTCGTATGCTTATGCACGCTCACATGCACCGTCTACAGCGGAAACTTGGACGTTCCATG

ACTACTCTAGGGGCTGGGTCTGCCATTGTTACAGGTAAAAAAGGACAGTCGGGTTCGGGG

AGTTCTGCAAGGACACAAGCAGATCACTCCCGACCAGACGGAAAGGAAAAATCAACACTT

GGAAAACGTATCGGTCAAACCATCGGTACAGTAGCTGATACCAAAGACAGAATGGTAGAC

ACTGCTAGTGGTTTGAAAGAACAGGTTAAAGATTTGCCGACCAATGCAAGATATGCAGTA

TATCAAGGAAAATCCAAAGTAAAAGAGAATGTCCGTGATTTAACCAGTAGTATTTCTCAA

ACCAAAGCGGACAGAGCCAGTGGACGCAAGGAACAGCAGGAACAAAGGCGAAAAACCATT

GCGAAGCGTCGCTCTGAAATGGAACAGGTCAAACAGAAAAAACAGCCTGCTTCTTCTGTT

CATGAAAGACCGACTACAAGACAAGAACAATATCATGATGAACAGACCTCAAAACAGTCT

AATATTCAGACTTCATATAAGGAATCTCAACAAGCCAAACAAGAGCGTCCAGCAGTTAAG

TCCGATTTTTCAAGTCCAAAAGTGGAACGCCAAGGCAATACCGTTCAAGAAAAAACCGTT

CAAAAGCCAGCAACTTCAACCACTACAGCAGATAGAACTTCACAACGTCCAATCACAAAA

GAACGTCCGTCTACTGTTCAAAGAGTACCACTACAAAATACAAGAAGTAGACCACCAATC

AAAACCGCCACCATTAAGAAAGTCGGTAAGAAACCATGAAGTTGAAAACTTTAGTGATTG

GTGGTTCTGGATTATTCTTGATGGTCTTCTCACTGCTTCTGTTTGTTGCCATTTTATTTT

CAGATGAACAGGACAGCGGAATTTCCAATATTCATTATGGAGGTGTGAATGTTTCCGCAG

AAGTGCTGGCTCATAAGCCTATGGTAGAAAAATATGCCAAAGAATATGGCGTTGAAGAAT

ATGTCAACATACTTCTTGCGATTATACAGGTGGAATCGGGCGGTACTGCGGAAGATGTTA

TGCAGTCCTCGGAATCCCTCGGTCTTCCACCTAATTCATTGAGTACAGAAGAATCCATTA

AGCAAGGTGTGAAGTATTTCAGTGAATTATTAGCCAGTAGCGAAAGGCTCAGTGTAGATT

TAGAATCGGTTATCCAGTCCTACAATTATGGTGGTGGTTTCTTAGGGTATGTGGCTAATC

GTGGAAATAAATATACCTTTGAACTGGCTCAAAGTTTCTCAAAAGAGTATTCAGGTGGCG

AAAAAGTGTCTTACCCCAATCCCATAGCCATACCTATCAATGGGGGCTGGCGATACAACT

ATGGCAATATGTTTTATGTGCAACTGGTAACGCAGTATCTTGTCACAACAGAGTTTGATG

ATGATACGGTACAAGCCATCATGGACGAAGCACTGAAATATGAGGGCTGGCGATACGTTT

ACGGTGGAGCTTCCCCGACTACTTCTTTTGATTGTAGCGGACTGACACAATGGACGTATG

GAAAAGCTGGAATTAACTTACCACGAACCGCACAACAGCAATATGATGTGACCCAGCATA

TCCCACTATCGGAAGCACAAGCTGGCGATTTGGTTTTCTTTCATTCTACCTATAACGCTG

GCTCTTATATTACTCATGTTGGGATATACCTTGGCAATAACCGTATGTTTCATGCAGGCG

ACCCAATCGGTTATGCCGACTTAACAAGCCCCTACTGGCAACAGCATTTAGTGGGAGCAG

GACGAATCAAACAATGAGAAAGGAAGATTTAATGATGAAATTTAGAAAAAATCAGAATAA

AGAAAAACAGATACCAAAGGAAAAGAAACCTCGTGTCTACTATAAGGTCAATCCTCATAA

AAAGGTTGTGATTGCCTTGTGGGTACTTTTAGGGCTTAGTTTCAGCTTTGCGATATTCAA

GCACTTTACAGCTATAGATACTCATACTATTCACGAAACAACTATCATAGAAAAGGAATA

CGTTGATACTCATCATGTAGAAAATTTTGTAGAGAACTTTGCGAAAGTCTACTATTCATG

GGAGCAATCCGATAAGTCCATTGATAATCGAATGGAAAGTCTAAAAGGCTATCTGACAGA

TGAACTTCAAGCTCTCAATGTTGATACAGTACGCAAAGATATTCCTGTATCGTCTTCTGT

AAGAGGATTTCAGATATGGACGGTAGAGCCAACTGGCGACAATGAGTTTAATGTAACCTA

CAGTGTAGACCAGCTCATTACAGAGGGAGAAAATACAAAGACCGTCCACTCTGCTTATAT

AGTGAGTGTCTATGTAGATGGTTCTGGAAATATGGTACTGGTTAAGAATCCGACCATTAC

CAACATACCTAAGAAATCAAGTTATAAACCAAAAGCCATTGAAAGTGAGGGGACGGTTGA

TTCCATTACAACCAATGAAATCAATGAGTTTTTAACGACGTTCTTCAAGCTCTATCCTAC

AGCGACAGCCAGTGAACTTTCCTACTATGTGAATGACGGGATATTAAAACCAATCGGAAA

AGAGTACATCTTTCAAGAACTGGTAAATCCTATTCACAATCGTAAGGATAATCAAGTCAC

GGTATCGCTGACAGTGGAGTATATCGACCAGCAGACCAAAGCAACGCAGGTATCTCAATT

TGATTTGGTACTTGAAAAGAACGGGAGTAATTGGAAGATTATAGAATAACAAATATTGGT

ACATTATTACAGCTATTTTGTAATCACGTACTCTCTTTGATAAAAAATTGGAGATTCCTT

TACAAATATGCTCTTACGTGCTATTATTTAAGTATCTATTTAAAAGGAGTTAATAAATAT

GCGGCAAGGTATTCTTAAATAAACTGTCAATTTGATAGTGGGAACAAATAATTGGATGCC

CTTTTGGGCTTTTGAATGGAGGAAAATCACATGAAAATTATTAATATTGGAGTTTTAGCT

CATGTTGATGCGGGAAAAACTACCTTAACAGAAAGCTTATTATATAACAGTGGAGCGATT

ACAGAATTAGGAAGCGTGGACAGAGGTACAACGAAAACGGATAATACGCTTTTAGAACGT

CAGAGAGGAATTACAATTCAGACGGCGATAACCTCTTTTCAGTGGAAAAATACTAAGATG

AACATCATAGACACGCCAGGACATATGGATTTTTTAGCAGAAGTATATCGTTCATTATCA

GTATTAGATGGGGCAATTCTACTGATTTCTGCAAAAGATGGCGTACAAGCACAAACTCGT

ATATTGTTTCATGCACTTAGGAAAATAGGTATTCCCACAATCTTTTTTATCAATAAGATT

GACCAAAATGGAATTGATTTATCAACGGTTTATCAGGATATTAAAGAGAAACTTTCTGCG

GAAATTGTAATCAAACAGAAGGTAGAACTGCATCCTAATATGCGTGTAATGAACTTTACC

GAATCTGAACAATGGGATATGGTAATAGAAGGAAATGATTACCTTTTGGAGAAATATACG

TCTGGGAAATTATTGGAAGCATTAGAACTCGAACAAGAGGAAAGCATAAGATTTCATAAT

TGTTCCCTGTTCCCTGTTTATCACGGAAGTGCAAAAAACAATATAGGGATTGATAACCTT

ATAGAAGTGATTACGAATAAATTTTATTCATCAACACATCGAGGTCAGTCTGAACTTTGC

GGAAAAGTTTTCAAAATTGAGTATTCGGAAAAAAGACAGCGTCTTGCATATATACGTCTT

TATAGTGGCGTACTGCATTTGCGAGATTCGGTTAGAATATCGGAAAAGGAAAAAATAAAA

ATTACAGAAATGTATACTTCAATAAATGGTGAATTATGTAAAATCGATAAGGCTTATTCC

GGGGAAATTGTTATTTTGCAGAATGAGTTTTTGAAGTTAAATAGTGTTCTTGGAGATACA

AAGCTATTGCCACAGAGAGAGAATTGAAAATCCCCTCCCTCTGCTGCAAACGACTGTTGA

ACCGAGCAAACCTCAACAAAGGGAAATGTTACTTGATGCACTTTTAGAAATCTCCGACAG

TGACCCGCTTCTGCGATATTATGTGGATTCTGCGACACATGAAATCATACTTTCTTTCTT

AGGGAAAGTACAAATGGAAGTGACTTGTGCTCTGCTGCAAGAAAAGTATCATGTGGAGAT

AGAAATAAAAGAGCCTACAGTCATTTATATGGAAAGACCGTTAAAAAAAGCAGAGTATAC

CATTCACATCGAAGTTCCACCGAATCCTTTCTGGGCTTCCATTGGTCTATCTGTAGCACA

GCTTCCATTAGGGAGCGGAGTACAGTATGAGAGCTCGGTTTCTCTTGGATACTTAAATCA

ATCGTTTCAAAATGCAGTTATGGAGGGGATACGCTATGGCTGTGAACAAGGATTGTATGG

TTGGAATGTGACGGACTGTAAAATCTGTTTTAAGTATGGCTTATACTATAGCCCTGTTAG

TACCCCAGCAGATTTTCGGATGCTTGCTCCTATTGTATTGGAACAAGTCTTAAAAAAAGC

TGGAACAGAATTGTTAGAGCCATATCTTAGTTTTAAAATTTATGCGCCACAGGAATATCT

TTCACGAGCATACAACGATGCTCCTAAATATTGTGCGAACATCGTAGACACTCAATTGAA

AAATAATGAGGTCATTCTTAGTGGAGAAATCCCTGCTCGGTGTATTCAAGAATATCGTAG

TGATTTAACTTTCTTTACAAATGGACGTAGTGTTTGTTTAACAGAGTTAAAAGGGTACCA

TGTTACTACCGGTGAACCTGTTTGCCAGCCCCGTCGTCCAAATAGTCGGATAGATAAAGT

ACGATATATGTTCAATAAAATAACTTAGTGTATTTTATGTTGTTATATAAATATGGTTTC

TTGTTAAATAAGATGAAATATTTTTTAATAAAGATTTGAATTAAAGTGTAAAGGAGGAGA

TAGTTATTATAAACTACAAGTGGATATTGTGTCCTGTATGTGGAAATAAAACACGATTAA

AGATAAGGGAAGATACTGAATTAAAAAAATTCCCCCTCTATTGTCCGAAATGCAGACAAG

AAAATTTAATTGAAATAAAGCAGTTCAAAGTAACTGTGATTACAGAGCCAGACGCAAAGA

CGCAGAGCCGATAAAATGAGATTAATACAATCTCATTTTATCGGCTCTTTCCGTTATGTA

TGGATTCTTTTAATTAGTCTTCGATGTTTCTTGCTTCGTTGATACCGCTGGCTAAAGATT

CCATTAAGGATAGTTCTTTGTCTGTAAAGCTATCCATGTATTTCTCTATCTGTAATCGTC

GGGTGCTTTTTACCAAGTTATTAGCAGGTAAGAAAAATTCATCAACGGAAACATGAAGTA

ACGATACAAGGTCATAAAGAACTTGTATGCTGGGGTGTTGCCCTTTATTTTCAATATTAG

TTAAGTACCGTGGGTCAATTTCAATCAATGCTCCCACTTGTTCACGAGTTAAACCTCGTT

TCAATCGAGCTTCTTTAATGGCTAAACCAAAGGCTCTAAAATCATATTTATCTTCTTTTT

TACGCATAGTAGACCACCTCTATACATTTTATTGTTCCTACTGAATTAAAAACAGGTATA

GAAAAACGTGTTATATGGTTTATAGGTTTATATTTAATAAAAAGCACTACTAAACGCCAA

TAAAAAAAACCGTTATATGGTAGTGCTATTTACGCTGTTAAAATATTGTATATTACTTCC

AAATGGCGGTTTGTTGGAGGTCAACGTCGCCATGAAGTACATCATATACAATAAATTTCC

TTACATTGGGTTCTTGTCAAAAAAAGTCGTCTATCTGCAATAGATAAGTACGTCCACCAA

TGTGGTTTTATAAATCATATAGATAGAATAACAGAAGCATGTAAACAGAGAAATAAATCT

GTTTATATGCTTTTTTGGCTATTCAGAACTTTTTTACAAAGTTTATTTATCAGTAATGCA

ACAAATCCCCCTTTCACATTGGGACTAAGAGTGAAAGGAGATAAACGAGCAAGGCTCACT

TCCTTTCCTAGACAGAAAGGGGGTGAGAAACATGAAACCATCTTCTTTTCAGACCACAAT

AGAAAATCAGTTTGACTATATCTGTAAACGTGCTATGGAAGACGAGCGAAAGAATTATAT

GCTTTATCTTTCAAGGATTGCAAAGCGTGAGGTGTCCTTTTCGGATGTTGGCGATTATCT

TGTTAGCCAGTTTGCGACAACAGATAACTATTCAACTGACTTTCAGATTTTTACACTCAA

TGGGTTATCAGTAGGCGTTGAAAATGATTTGTTGAGTGAAGCATTACGTGAGTTGCCAGA

CAAGAAACGTGAAATTCTACTGCTGTTTTACTTTATGGACATGAGCGATTCAGAAATTGC

AGACCTGTTGAAATTGAACCGTTCTACTGTCTATCGGCATAGAACCAGTGGACTAGCCTT

AATTAAAAAGTTTATGGAGGAATTTGAAGAATGAAAACACAATATCCTATGATTCCCTTT

CCTCTCATTGTAAAGGCAACAGATGGCGATACCGAAGCGATTAACCAGATTCTACATCAT

TACAGAGGGTACATAACGAAGCGTTCCCTACGACTTATGAAAGATGAATATGGCAATCAA

AGTATGGTCGTTGATGAAGTCTTACGTGGAAGAATGGAAACCAGACTGATTACAAAGATT

TTGTCATTTGAAATTAAGTAATATCCTCTCTCCTTTCGTGGAAGCGTGCTAAACCATTCC

ACGCTTCCCGAACAGGGAGGTTTGTTATTCCACCAAAGCATATTGAGCTTTCAATGTGTT

TTGATAGGCTAACGAGCCATTGTTCTTTGAAAACTGAATAAAAGTAATCGAATACGTTTC

GATAAGAAAAGAGCCAACGGAACTAACCGCCATGACCTATCTTATAAAGATAGCGAGCGA

TTCATGTTAGTGATCCGAGAAGCAATCTTTAGCAGGATTGCCTGCAACGACATTCTTATC

GTGATAATGATACTCCCATACAGTCAATAGTCCGAGCGTGATAAAACCGTCGCAGGCAAT

GAGTATGGCTACATGAGAACCATGCAGGGGTGGAACTCCCGTGAGCTTTGCTAAAGCTGT

TCGATTGCTGGTAAAACAACTTTTATGAAATCCAAATAAGTGATTTGGAAAGGAGGATTT

TATGAAGCAGACTGACATTCCTATTTGGGAACGTTATACCCTAACCATTGAAGAAGCGTC

AAAATATTTTCGTATTGGCGAAAACAAGCTACGACGCTTGGCAGAGGAAAATAAAAATGC

AAATTGGCTGATTATGAATGGCAATCGTATTCAGATTAAACGAAAACAATTTGAAAAAAT

TATAGATACATTGGACGCAATCTAGCGTCGCCAAAGGGTCTTGTATATGATAAAATAGTA

TTAAGTCGTATCAAGGCTCTTTCCATAAAGGAAAGGAGCAAATGCCATGTCAGAAAAAAG

ACGTGACAATAAAGGTCGAATCTTAAAGACTGGAGAGAGCCAACGAAAAGACGGAAGATA

CTTATACAAATATATAGATTCATTTGGAGAACCGCAATTTGTTTACTCGTGGAAACTTGT

GGCTACAGACCGAGTACCAGCAGGAAAGCGTGATTGTATCTCACTTAGAGAGAAAATCGC

AGAGTTACAGAAAGACATTCATGATGGTATTGATGTTGTAGGAAAGAAAATGACACTCTG

CCAGCTTTACGCAAAACAGAACGCTCAAAGACCAAAGGTTAGAAAAAACACTGAAACTGG

ACGCAAATATCTTATGGATATTTTGAAGAAAGACAAGTTAGGTGTAAGAAGTATTGACAG

TATTAAGCCATCAGACGCTAAAGAATGGGCTATTAGAATGAGTGAAAATGGTTATGCTTA

TCAAACCATCAATAACTACAAACGTTCTTTAAAGGCTTCATTCTATATTGCTATACAAGA

TGATTGTGTTCGGAAGAATCCATTTGACTTTCAACTGAAAGCAGTTCTTGATGATGATAC

TGTCCCTAAGACCGTACTAACAGAAGAACAGGAAGAAAAACTGTTAGCCTTTGCAAAAGC

TGATAAAACCTACAGCAAAAATTATGATGAAATTCTGATACTCTTAAAAACAGGTCTTCG

TATTTCAGAGTTTGGTGGTTTGACACTTCCAGATTTAGATTTTGAGAATCGTCTTGTCAA

TATAGACCATCAGCTATTGAGAGATACTGAAATTGGGTACTACATTGAAACACCAAAGAC

CAAAAGTGGCGAACGTCAAGTTCCTATGGTTGAAGAAGCCTATCAAGCATTTAAGCGAGT

GTTAGCGAATCGAAAGAATGATAAGCGTGTTGAGATTGATGGATATAGTGATTTCCTCTT

TCTTAATAGAAAGAACTATCCAAAAGTGGCAAGTGATTACAACGGCATGATGAAAGGTCT

TGTTAAGAAATACAATAAGTATAACGAGGATAAATTGCCACACATCACTCCACATAGTTT

GCGACATACATTCTGTACCAACTATGCAAATGCAGGAATGAATCCAAAGGCATTACAGTA

CATTATGGGACATGCTAATATAGCCATGACGCTGAACTATTACGCACATGCAACATTCGA

TTCTGCAATGGCAGAAATGAAACGCTTGAATAAAGAGAAGCAACAGGAGCGTCTTGTTGC

TTAGTAGTACAAATGAATTTACTACTTATTTACCACTTCTGACAGCTAAGACATGAGGAA

ATATGCAAAGAAACGTGAAGTATCTTCCTACAGTAAAAATACTCGAAAGCACATAGAATA

AGGCTTTACGAGCATTTAAGAAAATATAAAAAGATAATTAGAAATTTATACTTTGTTT

>GA04216/Tn916

AAAATAGCATAAAAATCTAGTTATCCGCATAAAAACTGGACTTATCACACTTTATCAAGG

TCAAAACCACTCAATTTACTACTAATTTACTACTTATGAATGAGCTTTGATACGACGATT

TATCCTTGAAAAGTGAAGATATAAAGATACTTCCAATAAAATTTGAATATTTAATAGGTA

GACACTTCAAAAAATGAGGTGTCTATTTTTTTACCCGATTTTGAAAGGAAGTGAACTTAT

GAAAACAAAAAATCAAGAATCAAAAGGTCGTTCCCCACTCTTTAAGACCATCAAACATTC

ATTCAGCCAATAAAAAAGAAAGGATAGGTAAAAATATGGAACTTAAATTTGTGATTCCCA

ACATGGAAAAAACATTCGGCAATTTAGAATTTGCTGGCGAGGATAAAGTCGTTCAGCGAA

GAATCAACGGACGGCTAACTGTCTTATCAAGAAGCTATAATCTCTATTCTGATGTTCAAA

GAGCAGATGATATTGTGGTGGTGCTTCCTGCTGAAGCTGGCGAAAAACATTTCGGCTTTG

AGGAACGTGTGAAGTTAGTCAATCCACGTATTACCGCAGAGGGCTACAAAATCGGCACTC

GTGGTTTTACAAATTACCTTTTACATGCTGACGACATGATAAAAGAATAAAGAAAGAGAG

GAAAAATGATGAGATTAGCAAATGGCATTGTATTAGATAAAGACACGACTTTTGGAGAAT

TGAAATTCTCTGCTCTACGTCGTGAAGTGAGAATCCAAAATGAAGACGGGTCGGTTTCAG

ATGAAATCAAGGAACGTACCTATGACTTAAAATCCAAAGGACAAGGACGCATGATTCAAG

TAAGTATTCCTGCCAGCGTGCCTTTGAAAGAGTTTGATTATAACGCACGGGTGGAACTTA

TCAATCCCATTGCGGACACCGTTGCTACTGCCACCTATCAAGGAGCAGATGTTGACTGGT

ATATCAAGGCAGACGATATTGTGCTGACAAAGGATTCTAGTTCATTCAAAGCTCAACCAC

AAGCAAAGAAAGAACCGACACAAGACAAATAGTCGCTAGGTAGAAAGGAGACTTTTTCGC

ATGAAACAGCGTGGTAAAAGGATTCGCCCATCTGGTAAAGATTTAGTCTTTCATTTTACG

ATAGCGTCACTCCTGCCTGTTTTCCTGCTGGTTGTCGGACTGTTTCATGTGAAGACAATC

CAGCAGATCAACTGGCAGGATTTTAACCTATCACAAGCAGATAAGATTGACATTCCCTAT

TTAATTATCAGTTTCAGTGTCGCAATTCTTATCTGCTTGCTGGTAGCGTTTGTATTCAAA

CGGGTTCGCTATGATACGGTTAAACAACTTTACCACCGTCAAAAACTGGCAAAGATGATA

CTTGAAAACAAGTGGTATGAATCTGAACAGGTCAAAACAGAGGGTTTCTTTAAAGATAGT

GCTGGTCGTACAAAGGAAAAGATAACCTACTTCCCTAAAATGTATTATCGACTTAAAAAT

GGCTTGATACAGATACGGGTGGAAATCACGCTGGGAAAATATCAAGACCAACTCTTACAC

TTGGAAAAGAAATTAGAGAGTGGCTTGTACTGTGAGCTGACGGATAAAGAGTTAAAGGAT

TCCTATGTGGAATATACTTTGCTCTATGACACCATAGCCAGTCGTATTTCTATTGATGAA

GTAGAAGCTAAAGATGGTAAACTTCGCTTAATGAAAAACGTATGGTGGGAATATGATAAG

CTCCCTCATATGTTGATTGCTGGTGGTACAGGTGGCGGTAAAACTTACTTTATACTGACA

CTGATTGAAGCCTTGCTTCATACAGATTCAAAACTGTATATTCTTGACCCGAAAAATGCT

GACCTTGCGGACTTAGGTTCTGTGATGGCAAATGTCTACTATAGAAAAGAAGACTTGCTT

TCTTGCATTGAAACATTCTATGAAGAAATGATGAAACGTAGTGAGGAAATGAAGCAGATG

AAGAACTATAAGACTGGCAAAAATTATGCTTACTTAGGTCTCCCGGCACACTTCTTAATC

TTTGATGAATACGTCGCTTTCATGGAAATGCTGGGAACAAAAGAAAACACCGCAGTTATG

AATAAGCTGAAACAGATTGTCATGTTAGGTCGTCAAGCTGGCTTCTTTCTAATACTGGCT

TGTCAACGTCCAGACGCAAAATATTTAGGCGACGGAATCCGTGATCAGTTTAATTTCAGA

GTGGCTTTAGGTCGTATGTCTGAAATGGGCTATGGCATGATGTTTGGCAGTGACGTACAA

AAGGATTTCTTCTTAAAGCGAATCAAAGGTCGTGGCTATGTTGATGTAGGAACAAGTGTC

ATATCAGAGTTTTATACTCCCCTTGTACCAAAAGGATATGATTTCTTGGAGGAAATTAAA

AAGTTATCCAACAGCAGACAGTCCACGCAGGCGACGTGCGAAGCGGAAGTCGCAGGTGTG

GACTGATCTTGCTGGCTGGTGTGGCAATAGCCACGCCAGCACTTAACCCCCCGTATCTAA

CAGGGGGGTACAAATCGACAGGAAACAGTCAAAAAAACATTAGAAAATCCTTTGGTTACA

AGGGATTTACAAAATTTCAGCGTATGTCAAATGGGCTTTAAAAGTTGACATACGCCTTTT

TGATTGGAGGGATTTTTACTGAATGAACAAACTTGGTTACAGCATTTAAAAGAAAAACGC

TTGGCTTATGGACTATCTCAAAACCGTTTAGCTGTTGCGACTGGTATTACAAGGCAGTAT

CTAAGCGATATTGAAACAGGAAAAGTCAAGCCATCAGAGGATTTACAGCAGTCCCTTTGG

GAAGCTCTGGAACGCTTCAATCCCGACGCTCCCCTTGAAATGCTGTTTGATTATGTAAGG

ATTCGCTTTCCGACAACAGACGTACAGCAGGTGGTCGAAAACATCTTACAACTGAAACTG

TCCTATTTTCTTCATGAGGACTATGGTTTCTATTCTTATTCAGAGCATTATGCTTTAGGC

GACATATTCGTCCTTTGCTCCCATGAACTGGACAAAGGAGTTCTGGTGGAATTGAAAGGT

CGTGGGTGCAGACAATTTGAAAGCTATCTTCTGGCACAACAAAGAAGCTGGTATGAGTTC

TTTATGGACGTTTTGGTGGCTGGCGGTGTGATGAAACGCCTTGACCTTGCCATTAACGAT

AAGACAGGGATTTTGAATATCCCTGTACTCACTGAAAAGTGCCAACAGGAAGAATGTATC

TCCGTCTTCCGCAGTTTTAAAAGCTATCGCAGTGGCGAACTGGTACGCAAAGAGGAAAAG

GAATGTATGGGAAACACCCTCTATATCGGTTCATTACAAAGTGAAGTTTATTTCTGTATC

TATGAAAAGGACTACGAGCAGTACAAGAAAAATGATATTCCCATTGAAGACGCAGAAGTA

AAAAACCGTTTTGAGATTCGATTGAAAAATGAGCGTGCCTATTATGCAGTCCGTGATTTA

CTCGTCTATGACAATCCAGAGCATACCGCCTTTAAAATTATCAATCGGTATATCCGTTTT

GTAGATAAAGACGATTCCAAACCTCGTTCTGATTGGAAACTGAATGAAGAATGGGCTTGG

TTTATTGGGAACAATCGTGAACGATTAAAACTAACCACAAAACCAGAGCCTTACTCCTTC

CAAAGGACGCTGAACTGGCTATCTCATCAAGTTGCCCCGACCTTAAAGGTTGCGATTAAA

CTTGATGAAATCAACCAGACGCAGGTTGTAAAAGACATTCTCGACCATGCGAAACTGACA

GACCGACACAAGCAGATTTTGAAGCAACAGTCAGTAAAAGAACAGGACGTGATAACAACA

AAAAAATAACTCAAATACAAATTCATTGAATATAGAGAGGAGAACATTTTTATGAATTTT

GGACAAAACCTTTATAACTGGTTTCTATCAAACGCTCAATCACTGGTGCTTTTAGCAATC

GTTGTGATTGGCTTGTATCTTGGCTTCAAGCGTGAGTTTAGCAAACTGATTGGCTTTTTA

ATTATTGCGATTATTGCGGTTGGCTTAGTCTTCAACGCTGCTGGAGTAAAAGACATTTTA

CTAGAGCTATTCAATCGCATTATTGGTGCTTAAATAAAACCGTTCTTTTGTGGAATATAA

GTGGTTTTCTTATGTTCCGCAAAGGAATGGTACACCAAACGAAGTGCGGTAGGGATTTTT

GAATCTCTACAAAGAAAGGACGTGAATATATGGACGATATGCAAGTCTATATTGCGAATT

TAGGCAAATACAATGAGGGCGAATTGGTCGGTGCGTGGTTTACCTTTCCCATTGACTTTG

AGGAAGTCAAAGAGAAAATCGGCTTGAATGATGAATATGAGGAATACGCCATTCATGACT

ACGAGTTACCCTTTACGGTTGACGAATACACTTCCATTGGCGAACTCAATCGACTATGGG

AAATGGTATCGGAATTACCCGAAGAATTACAATCGGAGCTATCTGCTCTGCTCACTCATT

TTTCAAGCATTGAAGAACTAAGCGAACATCAAGAGGATATTATCATTCATTCCGATTGTG

ATGATATGTATGACGTGGCACGCTACTACATTGAAGAAACGGGTGCTTTAGGCGAAGTAC

CAGCTAGTCTTCAAAACTATATTGATTATCAAGCCTATGGTCGGGATTTAGACCTTTCAG

GAACGTTTATCTCAACCAATCATGGGATTTTTGAAATCGTCTATTAAATCTGTCGGTACA

TTACTACTGGCAGATTTTCTATTTTACGGGGTGGCTCAATCAGCTACCCCTATTTTTTAT

GAAAGGATTGATTACATGAAGAAAATACGAAGCTATACCAGTATCTGGTCTGTGGAAAAG

GTACTGTATTCTATCAATGATTTTAGACTTCCGTTTCCCATAACCTTTACGCAAATGACA

TGGTTTGTCGTGTCACTCTTTGCAGTGATGATACTTGGCAACTTGCCCCCTCTTTCCATG

ATAGAGGGAGCATTTCTCAAATACTTTGGGATTCCTGTGGCTTTCACATGGTTTATGTCT

ACAAAAACTTTTGATGGTAAAAAGCCTTATGGATTTTTGAAGTCTGTCATTGCTTATGCA

CTGCGACCAAAGCTGACCTATGCAGGAAAAAAAGTAACGCTTGGCAGAAACCAGCCACAA

GAAGCCATTACAGCAGTTAGGAGTGAATTTTATGGCATATCCAATTAAATACATTGAAAA

CAATCTCGTCTGGAATAAAGACGGGGAATGTTATGCTTACTATGAGCTTGTTCCTTACAA

TTACTCATTTCTAAGTCCAGAACAGAAAATACAAGTGCATGATTCTTTCAGACAGCTTAT

CGCACAAAATCGTGATGGCAAAATTCATGCTTTACAAATCAGTACAGAATCCAGCATACG

TTCTGCACAAGAGCGTTCCAAAAATGAAGTCACTGGCAAGCTCAAAGCGGTTGCCTATGA

CAAAATCGACCAACAGACAGACGCTTTAATATCCATGATTGGCGAAAATCAAGTGAACTA

CCGTTTCTTTATCGGCTTTAAGTTGCTTCTCAACGATCAGGAGTTTTCTATGAAAAGTCT

TACCGTTGAAGCAAAAAATGCTTTGTCTGATTTTGTCTATGATGTGAACCATAAGCTGAT

GGGCGATTTTGTTAGTATGAGTAATGATGAAATCCTGCGTTTTCAGAAGATGGAAAAGCT

CTTAGAAAATAAAATCTCTCGTCGTTTCAAAATCCGCAGGTTAGATAAGGACGACTTCGG

CTATCTGATTGAACACCTTTACGGACAGACAGGCACTGCCTATGAAGAGTATGAGTACCA

TCTATCAAAGAAAAAGCTGGATAATGAAACGCTGATTAAATACTATGACTTGATTAAGCC

TACTCGCTGTTTGGTGGAAGAAAAACAGCGATATTTGAAAATCCAGCAGGAAGATGAAAC

CGTCTATGTAGCTTACTTTACCATTAACAGCATTGTCGGAGAACTGGACTTCCCGTCCTC

TGAAATCTTCTACTACCAGCAACAGCAATTTACATTCCCGATTGATACGTCAATGAATGT

GGAAATTGTAGCGAATCGTAAAGCCCTATCTACTGTCCGCAATAAAAAGAAAGAACTGAA

AGACTTGGATAACCACGCTTGGCAAAGTGATAATGAAACCAGCTCCAATGTGGCGGAAGC

TCTGGAAAGTGTGAATGAGCTGGAAACCAATTTAGACCAAAGCAAGGAATCTATGTACAA

GCTGTCTTATGTGGTAAGGGTATCAGCAAATGATCTTGACGAACTCAAACGTCGTTGTAA

TGAAGTGAAAGATTTTTATGACGATTTAAGCGTAAAACTGGTACGACCATTTGGGGATAT

GCTCGGCTTACATGAAGAATTTTTACCTGCCAGCAAGCGTTATATGAATGATTATATTCA

ATACGTGACCTCTGATTTCCTCGCTGGTTTAGGTTTTGGTGCTACTCAAATGCTGGGGGA

AAATGAGGGGATTTATGTTGGCTACAGCTTAGATACTGGACGCAATGTCTATCTGAAACC

TGCTCTTGCCAGTCAAGGGGTTAAGGGTTCAGTAACCAATGCGTTAGCGTCGGCTTTTGT

TGGTTCGCTGGGTGGTGGTAAATCCTTTGCGAATAACCTTATCGTCTATTATGCGGTGCT

TTATGGGGCACAAGCAGTGATTGTAGACCCAAAAGCAGAACGTGGCAGATGGAAAGAAAC

CTTGCCAGAGATTTCCCATGAAATCAATATCGTCACTCTGACTTCTGATGAGAAAAACAA

AGGCTTACTTGACCCTTATGTGATTATGAAAAATCCCAAAGATTCTGAATCACTGGCTAT

TGATATTCTGACATTCCTTACGGGGATTTCCTCTCGTGATGGGGAACGCTTCCCAATCCT

TAGAAAAGCCATTCGTGCAGTAACCAATAGTGAAGTACGAGGGTTGATGAAAGTGATTGA

GGAATTACGGGTTGAGAATACGCCACTAAGTACCAGTATAGCCGACCATATCGAAAGTTT

TACAGACTATGACTTTGCACATTTATTATTCAGTAATGGTTATGTGGAGCAGTCTATCAG

CTTAGAAAAACAACTGAACATTATACAGGTTGCGGACTTGGTACTTCCCGACAAGGAAAC

TTCCTTTGAGGAATATACCACTATGGAGCTTTTATCCGTTGCTATGCTGATTGTCATTAG

TACCTTTGCTTTAGACTTTATCCATACAGACCGAAGCATTTTCAAGATTGTAGATTTAGA

CGAAGCATGGAGCTTTTTACAGGTAGCACAAGGAAAAACACTATCTATGAAGCTGGTTCG

GGCTGGTCGTGCTATGAACGCTGGGGTATATTTCGTGACCCAAAATACAGACGACCTCTT

AGATGAAAAACTGAAAAATAACCTCGGCTTAAAATTTGCATTTCGTTCCACTGACCTTAA

CGAGATTAAAAAGACCTTAGCCTTTTTTGGTGTAGACCCAGAGGACGAAAACAATCAGAA

GCGATTGCGTGATTTGGAAAACGGGCAATGCCTTATCAGTGATTTATATGGTCGTGTCGG

TGTGATACAGTTCCACCCTGTATTTGAAGAACTGCTCCATGCCTTTGATACCAGACCACC

TGTGCGAAAAGAGGTGTAAATGTGAAACCATCAATAGTAAACAGAATAAAATCAAACTGG

ACGCTGAAACGTCTAGGTAAAGTGGCAATGACAGTGGCTTTCACACTTGTGATTGCCATT

TTTCTTTTAGCCATGCTGGGAACGGTGGTTCAAGCTGCGGGCTTGGTAGATGATACGGTC

AATGTGGCAAATGAATACAGCCGATACCCACTTGAAAACTATCAACTGGATTTTTATGTG

GATAATAGCTGGGGCTGGCTTCCGTGGAACTGGTCGGACGGGATTGGAAAACAGGTCATG

TATGGACTATATGCCATTACCAATTTTATTTGGACAATCAGTTTGTATGTTTCCAATGCG

ACAGGTTACTTAGTACAGGAAGCCTATTCCTTAGACTTCATTTCCGCTACAGCAGATTCC

ATTGGTAAGAATATGCAGACCTTAGCTGGTGTGAGTGCAAACGGATTTTCAACAGAGGGT

TTCTATGTTGGATTCCTCTTACTCTTGATTTTGGTTCTTGGGGTTTATGTTGCCTATACG

GGACTGATAAAGAGAGAAACCACAAAGGCAATTCATGCCATTATGAATTTTGTGCTGGTG

TTTATCCTATCGGCTTCCTTTATTGCCTACGCTCCCGACTACATTAAAAAAATCAATGAC

TTTTCATCAGACATCAGTAATGCCAGTTTATCACTTGGCACGAAGATTGTCATGCCCCAT

TCCGATAGTCAAGGCAAGGACAGCGTGGACTTAATCAGAGATAGCCTGTTTTCCATACAG

GTTCAGCAACCGTGGCTACTGCTTCAATACAACAGTTCAGACATTGAAAGTATCGGTATT

GACCGTGTGGAAAGCCTGCTCTCCACCAGCCCAGATTCCAACAATGGCGAAGACAGAGAA

AAAATTGTTGCGGAAGAAATTGAAGACAGAAGCAATACCAATCTAACCATTACAAAGACC

ATTAACCGTTTAGGTACAGTCTTCTTCCTATTTGTCTTCAATATTGGGATTTCCATATTT

GTATTCCTATTAACAGGAATCATGATTTTCTCGCAGGTACTTTTTATCATCTATGCTATG

TTTCTGCCTGTGAGCTTTATTTTAAGCATGATTCCATCATTTGATGGTATGTCAAAACGA

GCCATAACAAAGCTCTTTAATACCATTTTGACACGAGCTGGAATCACATTGATTATTACG

ACAGCATTTAGTATTTCAACCATGCTCTATACCTTATCGGCTGGTTATCCGTTCTTTTTG

ATTGCTTTTCTACAGATTGTGACCTTTGCAGGAATCTACTTCAAGCTGGGCGATTTAATG

AGTATGTTTTCTCTACAGAGTAACGATTCTCAAAGTGTGGGAAGTCGTGTGATGAGAAAA

CCTCGTATGCTTATGCACGCTCACATGCACCGTCTACAGCGGAAACTTGGACGTTCCATG

ACTACTCTAGGGGCTGGGTCTGCCATTGTTACAGGTAAAAAAGGACAGTCGGGTTCGGGG

AGTTCTGCAAGGACACAAGCAGATCACTCCCGACCAGACGGAAAGGAAAAATCAACACTT

GGAAAACGTATCGGTCAAACCATCGGTACAGTAGCTGATACCAAAGACAGAATGGTAGAC

ACTGCTAGTGGTTTGAAAGAACAGGTTAAAGATTTGCCGACCAATGCAAGATATGCAGTA

TATCAAGGAAAATCCAAAGTAAAAGAGAATGTCCGTGATTTAACCAGTAGTATTTCTCAA

ACCAAAGCGGACAGAGCCAGTGGACGCAAGGAACAGCAGGAACAAAGGCGAAAAACCATT

GCGAAGCGTCGCTCTGAAATGGAACAGGTCAAACAGAAAAAACAGCCTGCTTCTTCTGTT

CATGAAAGACCGACTACAAGACAAGAACAATATCATGATGAACAGACCTCAAAACAGTCT

AATATTCAGACTTCATATAAGGAATCTCAACAAGCCAAACAAGAGCGTCCAGCAGTTAAG

TCCGATTTTTCAAGTCCAAAAGTGGAACGCCAAGGCAATACCGTTCAAGAAAAAACCGTT

CAAAAGCCAGCAACTTCAACCACTACAGCAGATAGAACTTCACAACGTCCAATCACAAAA

GAACGTCCGTCTACTGTTCAAAGAGTACCACTACAAAATACAAGAAGTAGACCACCAATC

AAAACCGCCACCATTAAGAAAGTCGGTAAGAAACCATGAAGTTGAAAACTTTAGTGATTG

GTGGTTCTGGATTATTCTTGATGGTCTTCTCACTGCTTCTGTTTGTTGCCATTTTATTTT

CAGATGAACAGGACAGCGGAATTTCCAATATTCATTATGGAGGTGTGAATGTTTCCGCAG

AAGTGCTGGCTCATAAGCCTATGGTAGAAAAATATGCCAAAGAATATGGCGTTGAAGAAT

ATGTCAACATACTTCTTGCGATTATACAGGTGGAATCGGGCGGTACTGCGGAAGATGTTA

TGCAGTCCTCGGAATCCCTCGGTCTTCCACCTAATTCATTGAGTACAGAAGAATCCATTA

AGCAAGGTGTGAAGTATTTCAGTGAATTATTAGCCAGTAGCGAAAGGCTCAGTGTAGATT

TAGAATCGGTTATCCAGTCCTACAATTATGGTGGTGGTTTCTTAGGGTATGTGGCTAATC

GTGGAAATAAATATACCTTTGAACTGGCTCAAAGTTTCTCAAAAGAGTATTCAGGTGGCG

AAAAAGTGTCTTACCCCAATCCCATAGCCATACCTATCAATGGGGGCTGGCGATACAACT

ATGGCAATATGTTTTATGTGCAACTGGTAACGCAGTATCTTGTCACAACAGAGTTTGATG

ATGATACGGTACAAGCCATCATGGACGAAGCACTGAAATATGAGGGCTGGCGATACGTTT

ACGGTGGAGCTTCCCCGACTACTTCTTTTGATTGTAGCGGACTGACACAATGGACGTATG

GAAAAGCTGGAATTAACTTACCACGAACCGCACAACAGCAATATGATGTGACCCAGCATA

TCCCACTATCGGAAGCACAAGCTGGCGATTTGGTTTTCTTTCATTCTACCTATAACGCTG

GCTCTTATATTACTCATGTTGGGATATACCTTGGCAATAACCGTATGTTTCATGCAGGCG

ACCCAATCGGTTATGCCGACTTAACAAGCCCCTACTGGCAACAGCATTTAGTGGGAGCAG

GACGAATCAAACAATGAGAAAGGAAGATTTAATGATGAAATTTAGAAAAAATCAGAATAA

AGAAAAACAGATACCAAAGGAAAAGAAACCTCGTGTCTACTATAAGGTCAATCCTCATAA

AAAGGTTGTGATTGCCTTGTGGGTACTTTTAGGGCTTAGTTTCAGCTTTGCGATATTCAA

GCACTTTACAGCTATAGATACTCATACTATTCACGAAACAACTATCATAGAAAAGGAATA

CGTTGATACTCATCATGTAGAAAATTTTGTAGAGAACTTTGCGAAAGTCTACTATTCATG

GGAGCAATCCGATAAGTCCATTGATAATCGAATGGAAAGTCTAAAAGGCTATCTGACAGA

TGAACTTCAAGCTCTCAATGTTGATACAGTACGCAAAGATATTCCTGTATCGTCTTCTGT

AAGAGGATTTCAGATATGGACGGTAGAGCCAACTGGCGACAATGAGTTTAATGTAACCTA

CAGTGTAGACCAGCTCATTACAGAGGGAGAAAATACAAAGACCGTCCACTCTGCTTATAT

AGTGAGTGTCTATGTAGATGGTTCTGGAAATATGGTACTGGTTAAGAATCCGACCATTAC

CAACATACCTAAGAAATCAAGTTATAAACCAAAAGCCATTGAAAGTGAGGGGACGGTTGA

TTCCATTACAACCAATGAAATCAATGAGTTTTTAACGACGTTCTTCAAGCTCTATCCTAC

AGCGACAGCCAGTGAACTTTCCTACTATGTGAATGACGGGATATTAAAACCAATCGGAAA

AGAGTACATCTTTCAAGAACTGGTAAATCCTATTCACAATCGTAAGGATAATCAAGTCAC

GGTATCGCTGACAGTGGAGTATATCGACCAGCAGACCAAAGCAACGCAGGTATCTCAATT

TGATTTGGTACTTGAAAAGAACGGGAGTAATTGGAAGATTATAGAATAACAAATATTGGT

ACATTATTACAGCTATTTTGTAATCACGTACTCTCTTTGATAAAAAATTGGAGATTCCTT

TACAAATATGCTCTTACGTGCTATTATTTAAGTATCTATTTAAAAGGAGTTAATAAATAT

GCGGCAAGGTATTCTTAAATAAACTGTCAATTTGATAGTGGGAACAAATAATTGGATGCC

CTTTTGGGCTTTTGAATGGAGGAAAATCACATGAAAATTATTAATATTGGAGTTTTAGCT

CATGTTGATGCGGGAAAAACTACCTTAACAGAAAGCTTATTATATAACAGTGGAGCGATT

ACAGAATTAGGAAGCGTGGACAGAGGTACAACGAAAACGGATAATACGCTTTTAGAACGT

CAGAGAGGAATTACAATTCAGACGGCGATAACCTCTTTTCAGTGGAAAAATACTAAGATG

AACATCATAGACACGCCAGGACATATGGATTTTTTAGCAGAAGTATATCGTTCATTATCA

GTATTAGATGGGGCAATTCTACTGATTTCTGCAAAAGATGGCGTACAAGCACAAACTCGT

ATATTGTTTCATGCACTTAGGAAAATAGGTATTCCCACAATCTTTTTTATCAATAAGATT

GACCAAAATGGAATTGATTTATCAACGGTTTATCAGGATATTAAAGAGAAACTTTCTGCG

GAAATTGTAATCAAACAGAAGGTAGAACTGCATCCTAATATGCGTGTAATGAACTTTACC

GAATCTGAACAATGGGATATGGTAATAGAAGGAAATGATTACCTTTTGGAGAAATATACG

TCTGGGAAATTATTGGAAGCATTAGAACTCGAACAAGAGGAAAGCATAAGATTTCATAAT

TGTTCCCTGTTCCCTGTTTATCACGGAAGTGCAAAAAACAATATAGGGATTGATAACCTT

ATAGAAGTGATTACGAATAAATTTTATTCATCAACACATCGAGGTCAGTCTGAACTTTGC

GGAAAAGTTTTCAAAATTGAGTATTCGGAAAAAAGACAGCGTCTTGCATATATACGTCTT

TATAGTGGCGTACTGCATTTGCGAGATTCGGTTAGAATATCGGAAAAGGAAAAAATAAAA

ATTACAGAAATGTATACTTCAATAAATGGTGAATTATGTAAAATCGATAAGGCTTATTCC

GGGGAAATTGTTATTTTGCAGAATGAGTTTTTGAAGTTAAATAGTGTTCTTGGAGATACA

AAGCTATTGCCACAGAGAGAGAATTGAAAATCCCCTCCCTCTGCTGCAAACGACTGTTGA

ACCGAGCAAACCTCAACAAAGGGAAATGTTACTTGATGCACTTTTAGAAATCTCCGACAG

TGACCCGCTTCTGCGATATTATGTGGATTCTGCGACACATGAAATCATACTTTCTTTCTT

AGGGAAAGTACAAATGGAAGTGACTTGTGCTCTGCTGCAAGAAAAGTATCATGTGGAGAT

AGAAATAAAAGAGCCTACAGTCATTTATATGGAAAGACCGTTAAAAAAAGCAGAGTATAC

CATTCACATCGAAGTTCCACCGAATCCTTTCTGGGCTTCCATTGGTCTATCTGTAGCACA

GCTTCCATTAGGGAGCGGAGTACAGTATGAGAGCTCGGTTTCTCTTGGATACTTAAATCA

ATCGTTTCAAAATGCAGTTATGGAGGGGATACGCTATGGCTGTGAACAAGGATTGTATGG

TTGGAATGTGACGGACTGTAAAATCTGTTTTAAGTATGGCTTATACTATAGCCCTGTTAG

TACCCCAGCAGATTTTCGGATGCTTGCTCCTATTGTATTGGAACAAGTCTTAAAAAAAGC

TGGAACAGAATTGTTAGAGCCATATCTTAGTTTTAAAATTTATGCGCCACAGGAATATCT

TTCACGAGCATACAACGATGCTCCTAAATATTGTGCGAACATCGTAGACACTCAATTGAA

AAATAATGAGGTCATTCTTAGTGGAGAAATCCCTGCTCGGTGTATTCAAGAATATCGTAG

TGATTTAACTTTCTTTACAAATGGACGTAGTGTTTGTTTAACAGAGTTAAAAGGGTACCA

TGTTACTACCGGTGAACCTGTTTGCCAGCCCCGTCGTCCAAATAGTCGGATAGATAAAGT

ACGATATATGTTCAATAAAATAACTTAGTGTATTTTATGTTGTTATATAAATATGGTTTC

TTGTTAAATAAGATGAAATATTTTTTAATAAAGATTTGAATTAAAGTGTAAAGGAGGAGA

TAGTTATTATAAACTACAAGTGGATATTGTGTCCTGTATGTGGAAATAAAACACGATTAA

AGATAAGGGAAGATACTGAATTAAAAAAATTCCCCCTCTATTGTCCGAAATGCAGACAAG

AAAATTTAATTGAAATAAAGCAGTTCAAAGTAACTGTGATTACAGAGCCAGACGCAAAGA

CGCAGAGCCGATAAAATGAGATTAATACAATCTCATTTTATCGGCTCTTTCCGTTATGTA

TGGATTCTTTTAATTAGTCTTCGATGTTTCTTGCTTCGTTGATACCGCTGGCTAAAGATT

CCATTAAGGATAGTTCTTTGTCTGTAAAGCTATCCATGTATTTCTCTATCTGTAATCGTC

GGGTGCTTTTTACCAAGTTATTAGCAGGTAAGAAAAATTCATCAACGGAAACATGAAGTA

ACGATACAAGGTCATAAAGAACTTGTATGCTGGGGTGTTGCCCTTTATTTTCAATATTAG

TTAAGTACCGTGGGTCAATTTCAATCAATGCTCCCACTTGTTCACGAGTTAAACCTCGTT

TCAATCGAGCTTCTTTAATGGCTAAACCAAAGGCTCTAAAATCATATTTATCTTCTTTTT

TACGCATAGTAGACCACCTCTATACATTTTATTGTTCCTACTGAATTAAAAACAGGTATA

GAAAAACGTGTTATATGGTTTATAGGTTTATATTTAATAAAAAGCACTACTAAACGCCAA

TAAAAAAAACCGTTATATGGTAGTGCTATTTACGCTGTTAAAATATTGTATATTACTTCC

AAATGGCGGTTTGTTGGAGGTCAACGTCGCCATGAAGTACATCATATACAATAAATTTCC

TTACATTGGGTTCTTGTCAAAAAAAGTCGTCTATCTGCAATAGATAAGTACGTCCACCAA

TGTGGTTTTATAAATCATATAGATAGAATAACAGAAGCATGTAAACAGAGAAATAAATCT

GTTTATATGCTTTTTTGGCTATTCAGAACTTTTTTACAAAGTTTATTTATCAGTAATGCA

ACAAATCCCCCTTTCACATTGGGACTAAGAGTGAAAGGAGATAAACGAGCAAGGCTCACT

TCCTTTCCTAGACAGAAAGGGGGTGAGAAACATGAAACCATCTTCTTTTCAGACCACAAT

AGAAAATCAGTTTGACTATATCTGTAAACGTGCTATGGAAGACGAGCGAAAGAATTATAT

GCTTTATCTTTCAAGGATTGCAAAGCGTGAGGTGTCCTTTTCGGATGTTGGCGATTATCT

TGTTAGCCAGTTTGCGACAACAGATAACTATTCAACTGACTTTCAGATTTTTACACTCAA

TGGGTTATCAGTAGGCGTTGAAAATGATTTGTTGAGTGAAGCATTACGTGAGTTGCCAGA

CAAGAAACGTGAAATTCTACTGCTGTTTTACTTTATGGACATGAGCGATTCAGAAATTGC

AGACCTGTTGAAATTGAACCGTTCTACTGTCTATCGGCATAGAACCAGTGGACTAGCCTT

AATTAAAAAGTTTATGGAGGAATTTGAAGAATGAAAACACAATATCCTATGATTCCCTTT

CCTCTCATTGTAAAGGCAACAGATGGCGATACCGAAGCGATTAACCAGATTCTACATCAT

TACAGAGGGTACATAACGAAGCGTTCCCTACGACTTATGAAAGATGAATATGGCAATCAA

AGTATGGTCGTTGATGAAGTCTTACGTGGAAGAATGGAAACCAGACTGATTACAAAGATT

TTGTCATTTGAAATTAAGTAATATCCTCTCTCCTTTCGTGGAAGCGTGCTAAACCATTCC

ACGCTTCCCGAACAGGGAGGTTTGTTATTCCACCAAAGCATATTGAGCTTTCAATGTGTT

TTGATAGGCTAACGAGCCATTGTTCTTTGAAAACTGAATAAAAGTAATCGAATACGTTTC

GATAAGAAAAGAGCCAACGGAACTAACCGCCATGACCTATCTTATAAAGATAGCGAGCGA

TTCATGTTAGTGATCCGAGAAGCAATCTTTAGCAGGATTGCCTGCAACGACATTCTTATC

GTGATAATGATACTCCCATACAGTCAATAGTCCGAGCGTGATAAAACCGTCGCAGGCAAT

GAGTATGGCTACATGAGAACCATGCAGGGGTGGAACTCCCGTGAGCTTTGCTAAAGCTGT

TCGATTGCTGGTAAAACAACTTTTATGAAATCCAAATAAGTGATTTGGAAAGGAGGATTT

TATGAAGCAGACTGACATTCCTATTTGGGAACGTTATACCCTAACCATTGAAGAAGCGTC

AAAATATTTTCGTATTGGCGAAAACAAGCTACGACGCTTGGCAGAGGAAAATAAAAATGC

AAATTGGCTGATTATGAATGGCAATCGTATTCAGATTAAACGAAAACAATTTGAAAAAAT

TATAGATACATTGGACGCAATCTAGCGTCGCCAAAGGGTCTTGTATATGATAAAATAGTA

TTAAGTCGTATCAAGGCTCTTTCCATAAAGGAAAGGAGCAAATGCCATGTCAGAAAAAAG

ACGTGACAATAAAGGTCGAATCTTAAAGACTGGAGAGAGCCAACGAAAAGACGGAAGATA

CTTATACAAATATATAGATTCATTTGGAGAACCGCAATTTGTTTACTCGTGGAAACTTGT

GGCTACAGACCGAGTACCAGCAGGAAAGCGTGATTGTATCTCACTTAGAGAGAAAATCGC

AGAGTTACAGAAAGACATTCATGATGGTATTGATGTTGTAGGAAAGAAAATGACACTCTG

CCAGCTTTACGCAAAACAGAACGCTCAAAGACCAAAGGTTAGAAAAAACACTGAAACTGG

ACGCAAATATCTTATGGATATTTTGAAGAAAGACAAGTTAGGTGTAAGAAGTATTGACAG

TATTAAGCCATCAGACGCTAAAGAATGGGCTATTAGAATGAGTGAAAATGGTTATGCTTA

TCAAACCATCAATAACTACAAACGTTCTTTAAAGGCTTCATTCTATATTGCTATACAAGA

TGATTGTGTTCGGAAGAATCCATTTGACTTTCAACTGAAAGCAGTTCTTGATGATGATAC

TGTCCCTAAGACCGTACTAACAGAAGAACAGGAAGAAAAACTGTTAGCCTTTGCAAAAGC

TGATAAAACCTACAGCAAAAATTATGATGAAATTCTGATACTCTTAAAAACAGGTCTTCG

TATTTCAGAGTTTGGTGGTTTGACACTTCCAGATTTAGATTTTGAGAATCGTCTTGTCAA

TATAGACCATCAGCTATTGAGAGATACTGAAATTGGGTACTACATTGAAACACCAAAGAC

CAAAAGTGGCGAACGTCAAGTTCCTATGGTTGAAGAAGCCTATCAAGCATTTAAGCGAGT

GTTAGCGAATCGAAAGAATGATAAGCGTGTTGAGATTGATGGATATAGTGATTTCCTCTT

TCTTAATAGAAAGAACTATCCAAAAGTGGCAAGTGATTACAACGGCATGATGAAAGGTCT

TGTTAAGAAATACAATAAGTATAACGAGGATAAATTGCCACACATCACTCCACATAGTTT

GCGACATACATTCTGTACCAACTATGCAAATGCAGGAATGAATCCAAAGGCATTACAGTA

CATTATGGGACATGCTAATATAGCCATGACGCTGAACTATTACGCACATGCAACATTCGA

TTCTGCAATGGCAGAAATGAAACGCTTGAATAAAGAGAAGCAACAGGAGCGTCTTGTTGC

TTAGTAGTACAAATGAATTTACTACTTATTTACCACTTCTGACAGCTAAGACATGAGGAA

ATATGCAAAGAAACGTGAAGTATCTTCCTACAGTAAAAATACTCGAAAGCACATAGAATA

AGGCTTTACGAGCATTTAAGAAAATATAAAAAGATAATTAGAAATTTATACTTTGTTT

>GA05248/Tn916

AAAATAGCATAAAAATCTAGTTATCCGCATAAAAACTGGACTTATCACACTTTATCAAGG

TCAAAACCACTCAATTTACTACTAATTTACTACTTATGAATGAGCTTTGATACGACGATT

TATCCTTGAAAAGTGAAGATATAAAGATACTTCCAATAAAATTTGAATATTTAATAGGTA

GACACTTCAAAAAATGAGGTGTCTATTTTTTTACCCGATTTTGAAAGGAAGTGAACTTAT

GAAAACAAAAAATCAAGAATCAAAAGGTCGTTCCCCACTCTTTAAGACCATCAAACATTC

ATTCAGCCAATAAAAAAGAAAGGATAGGTAAAAATATGGAACTTAAATTTGTGATTCCCA

ACATGGAAAAAACATTCGGCAATTTAGAATTTGCTGGCGAGGATAAAGTCGTTCAGCGAA

GAATCAACGGACGGCTAACTGTCTTATCAAGAAGCTATAATCTCTATTCTGATGTTCAAA

GAGCAGATGATATTGTGGTGGTGCTTCCTGCTGAAGCTGGCGAAAAACATTTCGGCTTTG

AGGAACGTGTGAAGTTAGTCAATCCACGTATTACCGCAGAGGGCTACAAAATCGGCACTC

GTGGTTTTACAAATTACCTTTTACATGCTGACGACATGATAAAAGAATAAAGAAAGAGAG

GAAAAATGATGAGATTAGCAAATGGCATTGTATTAGATAAAGACACGACTTTTGGAGAAT

TGAAATTCTCTGCTCTACGTCGTGAAGTGAGAATCCAAAATGAAGACGGGTCGGTTTCAG

ATGAAATCAAGGAACGTACCTATGACTTAAAATCCAAAGGACAAGGACGCATGATTCAAG

TAAGTATTCCTGCCAGCGTGCCTTTGAAAGAGTTTGATTATAACGCACGGGTGGAACTTA

TCAATCCCATTGCGGACACCGTTGCTACTGCCACCTATCAAGGAGCAGATGTTGACTGGT

ATATCAAGGCAGACGATATTGTGCTGACAAAGGATTCTAGTTCATTCAAAGCTCAACCAC

AAGCAAAGAAAGAACCGACACAAGACAAATAGTCGCTAGGTAGAAAGGAGACTTTTTCGC

ATGAAACAGCGTGGTAAAAGGATTCGCCCATCTGGTAAAGATTTAGTCTTTCATTTTACG

ATAGCGTCACTCCTGCCTGTTTTCCTGCTGGTTGTCGGACTGTTTCATGTGAAGACAATC

CAGCAGATCAACTGGCAGGATTTTAACCTATCACAAGCAGATAAGATTGACATTCCCTAT

TTAATTATCAGTTTCAGTGTCGCAATTCTTATCTGCTTGCTGGTAGCGTTTGTATTCAAA

CGGGTTCGCTATGATACGGTTAAACAACTTTACCACCGTCAAAAACTGGCAAAGATGATA

CTTGAAAACAAGTGGTATGAATCTGAACAGGTCAAAACAGAGGGTTTCTTTAAAGATAGT

GCTGGTCGTACAAAGGAAAAGATAACCTACTTCCCTAAAATGTATTATCGACTTAAAAAT

GGCTTGATACAGATACGGGTGGAAATCACGCTGGGAAAATATCAAGACCAACTCTTACAC

TTGGAAAAGAAATTAGAGAGTGGCTTGTACTGTGAGCTGACGGATAAAGAGTTAAAGGAT

TCCTATGTGGAATATACTTTGCTCTATGACACCATAGCCAGTCGTATTTCTATTGATGAA

GTAGAAGCTAAAGATGGTAAACTTCGCTTAATGAAAAACGTATGGTGGGAATATGATAAG

CTCCCTCATATGTTGATTGCTGGTGGTACAGGTGGCGGTAAAACTTACTTTATACTGACA

CTGATTGAAGCCTTGCTTCATACAGATTCAAAACTGTATATTCTTGACCCGAAAAATGCT

GACCTTGCGGACTTAGGTTCTGTGATGGCAAATGTCTACTATAGAAAAGAAGACTTGCTT

TCTTGCATTGAAACATTCTATGAAGAAATGATGAAACGTAGTGAGGAAATGAAGCAGATG

AAGAACTATAAGACTGGCAAAAATTATGCTTACTTAGGTCTCCCGGCACACTTCTTAATC

TTTGATGAATACGTCGCTTTCATGGAAATGCTGGGAACAAAAGAAAACACCGCAGTTATG

AATAAGCTGAAACAGATTGTCATGTTAGGTCGTCAAGCTGGCTTCTTTCTAATACTGGCT

TGTCAACGTCCAGACGCAAAATATTTAGGCGACGGAATCCGTGATCAGTTTAATTTCAGA

GTGGCTTTAGGTCGTATGTCTGAAATGGGCTATGGCATGATGTTTGGCAGTGACGTACAA

AAGGATTTCTTCTTAAAGCGAATCAAAGGTCGTGGCTATGTTGATGTAGGAACAAGTGTC

ATATCAGAGTTTTATACTCCCCTTGTACCAAAAGGATATGATTTCTTGGAGGAAATTAAA

AAGTTATCCAACAGCAGACAGTCCACGCAGGCGACGTGCGAAGCGGAAGTCGCAGGTGTG

GACTGATCTTGCTGGCTGGTGTGGCAATAGCCACGCCAGCACTTAACCCCCCGTATCTAA

CAGGGGGGTACAAATCGACAGGAAACAGTCAAAAAAACATTAGAAAATCCTTTGGTTACA

AGGGATTTACAAAATTTCAGCGTATGTCAAATGGGCTTTAAAAGTTGACATACGCCTTTT

TGATTGGAGGGATTTTTACTGAATGAACAAACTTGGTTACAGCATTTAAAAGAAAAACGC

TTGGCTTATGGACTATCTCAAAACCGTTTAGCTGTTGCGACTGGTATTACAAGGCAGTAT

CTAAGCGATATTGAAACAGGAAAAGTCAAGCCATCAGAGGATTTACAGCAGTCCCTTTGG

GAAGCTCTGGAACGCTTCAATCCCGACGCTCCCCTTGAAATGCTGTTTGATTATGTAAGG

ATTCGCTTTCCGACAACAGACGTACAGCAGGTGGTCGAAAACATCTTACAACTGAAACTG

TCCTATTTTCTTCATGAGGACTATGGTTTCTATTCTTATTCAGAGCATTATGCTTTAGGC

GACATATTCGTCCTTTGCTCCCATGAACTGGACAAAGGAGTTCTGGTGGAATTGAAAGGT

CGTGGGTGCAGACAATTTGAAAGCTATCTTCTGGCACAACAAAGAAGCTGGTATGAGTTC

TTTATGGACGTTTTGGTGGCTGGCGGTGTGATGAAACGCCTTGACCTTGCCATTAACGAT

AAGACAGGGATTTTGAATATCCCTGTACTCACTGAAAAGTGCCAACAGGAAGAATGTATC

TCCGTCTTCCGCAGTTTTAAAAGCTATCGCAGTGGCGAACTGGTACGCAAAGAGGAAAAG

GAATGTATGGGAAACACCCTCTATATCGGTTCATTACAAAGTGAAGTTTATTTCTGTATC

TATGAAAAGGACTACGAGCAGTACAAGAAAAATGATATTCCCATTGAAGACGCAGAAGTA

AAAAACCGTTTTGAGATTCGATTGAAAAATGAGCGTGCCTATTATGCAGTCCGTGATTTA

CTCGTCTATGACAATCCAGAGCATACCGCCTTTAAAATTATCAATCGGTATATCCGTTTT

GTAGATAAAGACGATTCCAAACCTCGTTCTGATTGGAAACTGAATGAAGAATGGGCTTGG

TTTATTGGGAACAATCGTGAACGATTAAAACTAACCACAAAACCAGAGCCTTACTCCTTC

CAAAGGACGCTGAACTGGCTATCTCATCAAGTTGCCCCGACCTTAAAGGTTGCGATTAAA

CTTGATGAAATCAACCAGACGCAGGTTGTAAAAGACATTCTCGACCATGCGAAACTGACA

GACCGACACAAGCAGATTTTGAAGCAACAGTCAGTAAAAGAACAGGACGTGATAACAACA

AAAAAATAACTCAAATACAAATTCATTGAATATAGAGAGGAGAACATTTTTATGAATTTT

GGACAAAACCTTTATAACTGGTTTCTATCAAACGCTCAATCACTGGTGCTTTTAGCAATC

GTTGTGATTGGCTTGTATCTTGGCTTCAAGCGTGAGTTTAGCAAACTGATTGGCTTTTTA

ATTATTGCGATTATTGCGGTTGGCTTAGTCTTCAACGCTGCTGGAGTAAAAGACATTTTA

CTAGAGCTATTCAATCGCATTATTGGTGCTTAAATAAAACCGTTCTTTTGTGGAATATAA

GTGGTTTTCTTATGTTCCGCAAAGGAATGGTACACCAAACGAAGTGCGGTAGGGATTTTT

GAATCTCTACAAAGAAAGGACGTGAATATATGGACGATATGCAAGTCTATATTGCGAATT

TAGGCAAATACAATGAGGGCGAATTGGTCGGTGCGTGGTTTACCTTTCCCATTGACTTTG

AGGAAGTCAAAGAGAAAATCGGCTTGAATGATGAATATGAGGAATACGCCATTCATGACT

ACGAGTTACCCTTTACGGTTGACGAATACACTTCCATTGGCGAACTCAATCGACTATGGG

AAATGGTATCGGAATTACCCGAAGAATTACAATCGGAGCTATCTGCTCTGCTCACTCATT

TTTCAAGCATTGAAGAACTAAGCGAACATCAAGAGGATATTATCATTCATTCCGATTGTG

ATGATATGTATGACGTGGCACGCTACTACATTGAAGAAACGGGTGCTTTAGGCGAAGTAC

CAGCTAGTCTTCAAAACTATATTGATTATCAAGCCTATGGTCGGGATTTAGACCTTTCAG

GAACGTTTATCTCAACCAATCATGGGATTTTTGAAATCGTCTATTAAATCTGTCGGTACA

TTACTACTGGCAGATTTTCTATTTTACGGGGTGGCTCAATCAGCTACCCCTATTTTTTAT

GAAAGGATTGATTACATGAAGAAAATACGAAGCTATACCAGTATCTGGTCTGTGGAAAAG

GTACTGTATTCTATCAATGATTTTAGACTTCCGTTTCCCATAACCTTTACGCAAATGACA

TGGTTTGTCGTGTCACTCTTTGCAGTGATGATACTTGGCAACTTGCCCCCTCTTTCCATG

ATAGAGGGAGCATTTCTCAAATACTTTGGGATTCCTGTGGCTTTCACATGGTTTATGTCT

ACAAAAACTTTTGATGGTAAAAAGCCTTATGGATTTTTGAAGTCTGTCATTGCTTATGCA

CTGCGACCAAAGCTGACCTATGCAGGAAAAAAAGTAACGCTTGGCAGAAACCAGCCACAA

GAAGCCATTACAGCAGTTAGGAGTGAATTTTATGGCATATCCAATTAAATACATTGAAAA

CAATCTCGTCTGGAATAAAGACGGGGAATGTTATGCTTACTATGAGCTTGTTCCTTACAA

TTACTCATTTCTAAGTCCAGAACAGAAAATACAAGTGCATGATTCTTTCAGACAGCTTAT

CGCACAAAATCGTGATGGCAAAATTCATGCTTTACAAATCAGTACAGAATCCAGCATACG

TTCTGCACAAGAGCGTTCCAAAAATGAAGTCACTGGCAAGCTCAAAGCGGTTGCCTATGA

CAAAATCGACCAACAGACAGACGCTTTAATATCCATGATTGGCGAAAATCAAGTGAACTA

CCGTTTCTTTATCGGCTTTAAGTTGCTTCTCAACGATCAGGAGTTTTCTATGAAAAGTCT

TACCGTTGAAGCAAAAAATGCTTTGTCTGATTTTGTCTATGATGTGAACCATAAGCTGAT

GGGCGATTTTGTTAGTATGAGTAATGATGAAATCCTGCGTTTTCAGAAGATGGAAAAGCT

CTTAGAAAATAAAATCTCTCGTCGTTTCAAAATCCGCAGGTTAGATAAGGACGACTTCGG

CTATCTGATTGAACACCTTTACGGACAGACAGGCACTGCCTATGAAGAGTATGAGTACCA

TCTATCAAAGAAAAAGCTGGATAATGAAACGCTGATTAAATACTATGACTTGATTAAGCC

TACTCGCTGTTTGGTGGAAGAAAAACAGCGATATTTGAAAATCCAGCAGGAAGATGAAAC

CGTCTATGTAGCTTACTTTACCATTAACAGCATTGTCGGAGAACTGGACTTCCCGTCCTC

TGAAATCTTCTACTACCAGCAACAGCAATTTACATTCCCGATTGATACGTCAATGAATGT

GGAAATTGTAGCGAATCGTAAAGCCCTATCTACTGTCCGCAATAAAAAGAAAGAACTGAA

AGACTTGGATAACCACGCTTGGCAAAGTGATAATGAAACCAGCTCCAATGTGGCGGAAGC

TCTGGAAAGTGTGAATGAGCTGGAAACCAATTTAGACCAAAGCAAGGAATCTATGTACAA

GCTGTCTTATGTGGTAAGGGTATCAGCAAATGATCTTGACGAACTCAAACGTCGTTGTAA

TGAAGTGAAAGATTTTTATGACGATTTAAGCGTAAAACTGGTACGACCATTTGGGGATAT

GCTCGGCTTACATGAAGAATTTTTACCTGCCAGCAAGCGTTATATGAATGATTATATTCA

ATACGTGACCTCTGATTTCCTCGCTGGTTTAGGTTTTGGTGCTACTCAAATGCTGGGGGA

AAATGAGGGGATTTATGTTGGCTACAGCTTAGATACTGGACGCAATGTCTATCTGAAACC

TGCTCTTGCCAGTCAAGGGGTTAAGGGTTCAGTAACCAATGCGTTAGCGTCGGCTTTTGT

TGGTTCGCTGGGTGGTGGTAAATCCTTTGCGAATAACCTTATCGTCTATTATGCGGTGCT

TTATGGGGCACAAGCAGTGATTGTAGACCCAAAAGCAGAACGTGGCAGATGGAAAGAAAC

CTTGCCAGAGATTTCCCATGAAATCAATATCGTCACTCTGACTTCTGATGAGAAAAACAA

AGGCTTACTTGACCCTTATGTGATTATGAAAAATCCCAAAGATTCTGAATCACTGGCTAT

TGATATTCTGACATTCCTTACGGGGATTTCCTCTCGTGATGGGGAACGCTTCCCAATCCT

TAGAAAAGCCATTCGTGCAGTAACCAATAGTGAAGTACGAGGGTTGATGAAAGTGATTGA

GGAATTACGGGTTGAGAATACGCCACTAAGTACCAGTATAGCCGACCATATCGAAAGTTT

TACAGACTATGACTTTGCACATTTATTATTCAGTAATGGTTATGTGGAGCAGTCTATCAG

CTTAGAAAAACAACTGAACATTATACAGGTTGCGGACTTGGTACTTCCCGACAAGGAAAC

TTCCTTTGAGGAATATACCACTATGGAGCTTTTATCCGTTGCTATGCTGATTGTCATTAG

TACCTTTGCTTTAGACTTTATCCATACAGACCGAAGCATTTTCAAGATTGTAGATTTAGA

CGAAGCATGGAGCTTTTTACAGGTAGCACAAGGAAAAACACTATCTATGAAGCTGGTTCG

GGCTGGTCGTGCTATGAACGCTGGGGTATATTTCGTGACCCAAAATACAGACGACCTCTT

AGATGAAAAACTGAAAAATAACCTCGGCTTAAAATTTGCATTTCGTTCCACTGACCTTAA

CGAGATTAAAAAGACCTTAGCCTTTTTTGGTGTAGACCCAGAGGACGAAAACAATCAGAA

GCGATTGCGTGATTTGGAAAACGGGCAATGCCTTATCAGTGATTTATATGGTCGTGTCGG

TGTGATACAGTTCCACCCTGTATTTGAAGAACTGCTCCATGCCTTTGATACCAGACCACC

TGTGCGAAAAGAGGTGTAAATGTGAAACCATCAATAGTAAACAGAATAAAATCAAACTGG

ACGCTGAAACGTCTAGGTAAAGTGGCAATGACAGTGGCTTTCACACTTGTGATTGCCATT

TTTCTTTTAGCCATGCTGGGAACGGTGGTTCAAGCTGCGGGCTTGGTAGATGATACGGTC

AATGTGGCAAATGAATACAGCCGATACCCACTTGAAAACTATCAACTGGATTTTTATGTG

GATAATAGCTGGGGCTGGCTTCCGTGGAACTGGTCGGACGGGATTGGAAAACAGGTCATG

TATGGACTATATGCCATTACCAATTTTATTTGGACAATCAGTTTGTATGTTTCCAATGCG

ACAGGTTACTTAGTACAGGAAGCCTATTCCTTAGACTTCATTTCCGCTACAGCAGATTCC

ATTGGTAAGAATATGCAGACCTTAGCTGGTGTGAGTGCAAACGGATTTTCAACAGAGGGT

TTCTATGTTGGATTCCTCTTACTCTTGATTTTGGTTCTTGGGGTTTATGTTGCCTATACG

GGACTGATAAAGAGAGAAACCACAAAGGCAATTCATGCCATTATGAATTTTGTGCTGGTG

TTTATCCTATCGGCTTCCTTTATTGCCTACGCTCCCGACTACATTAAAAAAATCAATGAC

TTTTCATCAGACATCAGTAATGCCAGTTTATCACTTGGCACGAAGATTGTCATGCCCCAT

TCCGATAGTCAAGGCAAGGACAGCGTGGACTTAATCAGAGATAGCCTGTTTTCCATACAG

GTTCAGCAACCGTGGCTACTGCTTCAATACAACAGTTCAGACATTGAAAGTATCGGTATT

GACCGTGTGGAAAGCCTGCTCTCCACCAGCCCAGATTCCAACAATGGCGAAGACAGAGAA

AAAATTGTTGCGGAAGAAATTGAAGACAGAAGCAATACCAATCTAACCATTACAAAGACC

ATTAACCGTTTAGGTACAGTCTTCTTCCTATTTGTCTTCAATATTGGGATTTCCATATTT

GTATTCCTATTAACAGGAATCATGATTTTCTCGCAGGTACTTTTTATCATCTATGCTATG

TTTCTGCCTGTGAGCTTTATTTTAAGCATGATTCCATCATTTGATGGTATGTCAAAACGA

GCCATAACAAAGCTCTTTAATACCATTTTGACACGAGCTGGAATCACATTGATTATTACG

ACAGCATTTAGTATTTCAACCATGCTCTATACCTTATCGGCTGGTTATCCGTTCTTTTTG

ATTGCTTTTCTACAGATTGTGACCTTTGCAGGAATCTACTTCAAGCTGGGCGATTTAATG

AGTATGTTTTCTCTACAGAGTAACGATTCTCAAAGTGTGGGAAGTCGTGTGATGAGAAAA

CCTCGTATGCTTATGCACGCTCACATGCACCGTCTACAGCGGAAACTTGGACGTTCCATG

ACTACTCTAGGGGCTGGGTCTGCCATTGTTACAGGTAAAAAAGGACAGTCGGGTTCGGGG

AGTTCTGCAAGGACACAAGCAGATCACTCCCGACCAGACGGAAAGGAAAAATCAACACTT

GGAAAACGTATCGGTCAAACCATCGGTACAGTAGCTGATACCAAAGACAGAATGGTAGAC

ACTGCTAGTGGTTTGAAAGAACAGGTTAAAGATTTGCCGACCAATGCAAGATATGCAGTA

TATCAAGGAAAATCCAAAGTAAAAGAGAATGTCCGTGATTTAACCAGTAGTATTTCTCAA

ACCAAAGCGGACAGAGCCAGTGGACGCAAGGAACAGCAGGAACAAAGGCGAAAAACCATT

GCGAAGCGTCGCTCTGAAATGGAACAGGTCAAACAGAAAAAACAGCCTGCTTCTTCTGTT

CATGAAAGACCGACTACAAGACAAGAACAATATCATGATGAACAGACCTCAAAACAGTCT

AATATTCAGACTTCATATAAGGAATCTCAACAAGCCAAACAAGAGCGTCCAGCAGTTAAG

TCCGATTTTTCAAGTCCAAAAGTGGAACGCCAAGGCAATACCGTTCAAGAAAAAACCGTT

CAAAAGCCAGCAACTTCAACCACTACAGCAGATAGAACTTCACAACGTCCAATCACAAAA

GAACGTCCGTCTACTGTTCAAAGAGTACCACTACAAAATACAAGAAGTAGACCACCAATC

AAAACCGCCACCATTAAGAAAGTCGGTAAGAAACCATGAAGTTGAAAACTTTAGTGATTG

GTGGTTCTGGATTATTCTTGATGGTCTTCTCACTGCTTCTGTTTGTTGCCATTTTATTTT

CAGATGAACAGGACAGCGGAATTTCCAATATTCATTATGGAGGTGTGAATGTTTCCGCAG

AAGTGCTGGCTCATAAGCCTATGGTAGAAAAATATGCCAAAGAATATGGCGTTGAAGAAT

ATGTCAACATACTTCTTGCGATTATACAGGTGGAATCGGGCGGTACTGCGGAAGATGTTA

TGCAGTCCTCGGAATCCCTCGGTCTTCCACCTAATTCATTGAGTACAGAAGAATCCATTA

AGCAAGGTGTGAAGTATTTCAGTGAATTATTAGCCAGTAGCGAAAGGCTCAGTGTAGATT

TAGAATCGGTTATCCAGTCCTACAATTATGGTGGTGGTTTCTTAGGGTATGTGGCTAATC

GTGGAAATAAATATACCTTTGAACTGGCTCAAAGTTTCTCAAAAGAGTATTCAGGTGGCG

AAAAAGTGTCTTACCCCAATCCCATAGCCATACCTATCAATGGGGGCTGGCGATACAACT

ATGGCAATATGTTTTATGTGCAACTGGTAACGCAGTATCTTGTCACAACAGAGTTTGATG

ATGATACGGTACAAGCCATCATGGACGAAGCACTGAAATATGAGGGCTGGCGATACGTTT

ACGGTGGAGCTTCCCCGACTACTTCTTTTGATTGTAGCGGACTGACACAATGGACGTATG

GAAAAGCTGGAATTAACTTACCACGAACCGCACAACAGCAATATGATGTGACCCAGCATA

TCCCACTATCGGAAGCACAAGCTGGCGATTTGGTTTTCTTTCATTCTACCTATAACGCTG

GCTCTTATATTACTCATGTTGGGATATACCTTGGCAATAACCGTATGTTTCATGCAGGCG

ACCCAATCGGTTATGCCGACTTAACAAGCCCCTACTGGCAACAGCATTTAGTGGGAGCAG

GACGAATCAAACAATGAGAAAGGAAGATTTAATGATGAAATTTAGAAAAAATCAGAATAA

AGAAAAACAGATACCAAAGGAAAAGAAACCTCGTGTCTACTATAAGGTCAATCCTCATAA

AAAGGTTGTGATTGCCTTGTGGGTACTTTTAGGGCTTAGTTTCAGCTTTGCGATATTCAA

GCACTTTACAGCTATAGATACTCATACTATTCACGAAACAACTATCATAGAAAAGGAATA

CGTTGATACTCATCATGTAGAAAATTTTGTAGAGAACTTTGCGAAAGTCTACTATTCATG

GGAGCAATCCGATAAGTCCATTGATAATCGAATGGAAAGTCTAAAAGGCTATCTGACAGA

TGAACTTCAAGCTCTCAATGTTGATACAGTACGCAAAGATATTCCTGTATCGTCTTCTGT

AAGAGGATTTCAGATATGGACGGTAGAGCCAACTGGCGACAATGAGTTTAATGTAACCTA

CAGTGTAGACCAGCTCATTACAGAGGGAGAAAATACAAAGACCGTCCACTCTGCTTATAT

AGTGAGTGTCTATGTAGATGGTTCTGGAAATATGGTACTGGTTAAGAATCCGACCATTAC

CAACATACCTAAGAAATCAAGTTATAAACCAAAAGCCATTGAAAGTGAGGGGACGGTTGA

TTCCATTACAACCAATGAAATCAATGAGTTTTTAACGACGTTCTTCAAGCTCTATCCTAC

AGCGACAGCCAGTGAACTTTCCTACTATGTGAATGACGGGATATTAAAACCAATCGGAAA

AGAGTACATCTTTCAAGAACTGGTAAATCCTATTCACAATCGTAAGGATAATCAAGTCAC

GGTATCGCTGACAGTGGAGTATATCGACCAGCAGACCAAAGCAACGCAGGTATCTCAATT

TGATTTGGTACTTGAAAAGAACGGGAGTAATTGGAAGATTATAGAATAACAAATATTGGT

ACATTATTACAGCTATTTTGTAATCACGTACTCTCTTTGATAAAAAATTGGAGATTCCTT

TACAAATATGCTCTTACGTGCTATTATTTAAGTATCTATTTAAAAGGAGTTAATAAATAT

GCGGCAAGGTATTCTTAAATAAACTGTCAATTTGATAGTGGGAACAAATAATTGGATGCC

CTTTTGGGCTTTTGAATGGAGGAAAATCACATGAAAATTATTAATATTGGAGTTTTAGCT

CATGTTGATGCGGGAAAAACTACCTTAACAGAAAGCTTATTATATAACAGTGGAGCGATT

ACAGAATTAGGAAGCGTGGACAGAGGTACAACGAAAACGGATAATACGCTTTTAGAACGT

CAGAGAGGAATTACAATTCAGACGGCGATAACCTCTTTTCAGTGGAAAAATACTAAGATG

AACATCATAGACACGCCAGGACATATGGATTTTTTAGCAGAAGTATATCGTTCATTATCA

GTATTAGATGGGGCAATTCTACTGATTTCTGCAAAAGATGGCGTACAAGCACAAACTCGT

ATATTGTTTCATGCACTTAGGAAAATAGGTATTCCCACAATCTTTTTTATCAATAAGATT

GACCAAAATGGAATTGATTTATCAACGGTTTATCAGGATATTAAAGAGAAACTTTCTGCG

GAAATTGTAATCAAACAGAAGGTAGAACTGCATCCTAATATGCGTGTAATGAACTTTACC

GAATCTGAACAATGGGATATGGTAATAGAAGGAAATGATTACCTTTTGGAGAAATATACG

TCTGGGAAATTATTGGAAGCATTAGAACTCGAACAAGAGGAAAGCATAAGATTTCATAAT

TGTTCCCTGTTCCCTGTTTATCACGGAAGTGCAAAAAACAATATAGGGATTGATAACCTT

ATAGAAGTGATTACGAATAAATTTTATTCATCAACACATCGAGGTCAGTCTGAACTTTGC

GGAAAAGTTTTCAAAATTGAGTATTCGGAAAAAAGACAGCGTCTTGCATATATACGTCTT

TATAGTGGCGTACTGCATTTGCGAGATTCGGTTAGAATATCGGAAAAGGAAAAAATAAAA

ATTACAGAAATGTATACTTCAATAAATGGTGAATTATGTAAAATCGATAAGGCTTATTCC

GGGGAAATTGTTATTTTGCAGAATGAGTTTTTGAAGTTAAATAGTGTTCTTGGAGATACA

AAGCTATTGCCACAGAGAGAGAATTGAAAATCCCCTCCCTCTGCTGCAAACGACTGTTGA

ACCGAGCAAACCTCAACAAAGGGAAATGTTACTTGATGCACTTTTAGAAATCTCCGACAG

TGACCCGCTTCTGCGATATTATGTGGATTCTGCGACACATGAAATCATACTTTCTTTCTT

AGGGAAAGTACAAATGGAAGTGACTTGTGCTCTGCTGCAAGAAAAGTATCATGTGGAGAT

AGAAATAAAAGAGCCTACAGTCATTTATATGGAAAGACCGTTAAAAAAAGCAGAGTATAC

CATTCACATCGAAGTTCCACCGAATCCTTTCTGGGCTTCCATTGGTCTATCTGTAGCACA

GCTTCCATTAGGGAGCGGAGTACAGTATGAGAGCTCGGTTTCTCTTGGATACTTAAATCA

ATCGTTTCAAAATGCAGTTATGGAGGGGATACGCTATGGCTGTGAACAAGGATTGTATGG

TTGGAATGTGACGGACTGTAAAATCTGTTTTAAGTATGGCTTATACTATAGCCCTGTTAG

TACCCCAGCAGATTTTCGGATGCTTGCTCCTATTGTATTGGAACAAGTCTTAAAAAAAGC

TGGAACAGAATTGTTAGAGCCATATCTTAGTTTTAAAATTTATGCGCCACAGGAATATCT

TTCACGAGCATACAACGATGCTCCTAAATATTGTGCGAACATCGTAGACACTCAATTGAA

AAATAATGAGGTCATTCTTAGTGGAGAAATCCCTGCTCGGTGTATTCAAGAATATCGTAG

TGATTTAACTTTCTTTACAAATGGACGTAGTGTTTGTTTAACAGAGTTAAAAGGGTACCA

TGTTACTACCGGTGAACCTGTTTGCCAGCCCCGTCGTCCAAATAGTCGGATAGATAAAGT

ACGATATATGTTCAATAAAATAACTTAGTGTATTTTATGTTGTTATATAAATATGGTTTC

TTGTTAAATAAGATGAAATATTTTTTAATAAAGATTTGAATTAAAGTGTAAAGGAGGAGA

TAGTTATTATAAACTACAAGTGGATATTGTGTCCTGTATGTGGAAATAAAACACGATTAA

AGATAAGGGAAGATACTGAATTAAAAAAATTCCCCCTCTATTGTCCGAAATGCAGACAAG

AAAATTTAATTGAAATAAAGCAGTTCAAAGTAACTGTGATTACAGAGCCAGACGCAAAGA

CGCAGAGCCGATAAAATGAGATTAATACAATCTCATTTTATCGGCTCTTTCCGTTATGTA

TGGATTCTTTTAATTAGTCTTCGATGTTTCTTGCTTCGTTGATACCGCTGGCTAAAGATT

CCATTAAGGATAGTTCTTTGTCTGTAAAGCTATCCATGTATTTCTCTATCTGTAATCGTC

GGGTGCTTTTTACCAAGTTATTAGCAGGTAAGAAAAATTCATCAACGGAAACATGAAGTA

ACGATACAAGGTCATAAAGAACTTGTATGCTGGGGTGTTGCCCTTTATTTTCAATATTAG

TTAAGTACCGTGGGTCAATTTCAATCAATGCTCCCACTTGTTCACGAGTTAAACCTCGTT

TCAATCGAGCTTCTTTAATGGCTAAACCAAAGGCTCTAAAATCATATTTATCTTCTTTTT

TACGCATAGTAGACCACCTCTATACATTTTATTGTTCCTACTGAATTAAAAACAGGTATA

GAAAAACGTGTTATATGGTTTATAGGTTTATATTTAATAAAAAGCACTACTAAACGCCAA

TAAAAAAAACCGTTATATGGTAGTGCTATTTACGCTGTTAAAATATTGTATATTACTTCC

AAATGGCGGTTTGTTGGAGGTCAACGTCGCCATGAAGTACATCATATACAATAAATTTCC

TTACATTGGGTTCTTGTCAAAAAAAGTCGTCTATCTGCAATAGATAAGTACGTCCACCAA

TGTGGTTTTATAAATCATATAGATAGAATAACAGAAGCATGTAAACAGAGAAATAAATCT

GTTTATATGCTTTTTTGGCTATTCAGAACTTTTTTACAAAGTTTATTTATCAGTAATGCA

ACAAATCCCCCTTTCACATTGGGACTAAGAGTGAAAGGAGATAAACGAGCAAGGCTCACT

TCCTTTCCTAGACAGAAAGGGGGTGAGAAACATGAAACCATCTTCTTTTCAGACCACAAT

AGAAAATCAGTTTGACTATATCTGTAAACGTGCTATGGAAGACGAGCGAAAGAATTATAT

GCTTTATCTTTCAAGGATTGCAAAGCGTGAGGTGTCCTTTTCGGATGTTGGCGATTATCT

TGTTAGCCAGTTTGCGACAACAGATAACTATTCAACTGACTTTCAGATTTTTACACTCAA

TGGGTTATCAGTAGGCGTTGAAAATGATTTGTTGAGTGAAGCATTACGTGAGTTGCCAGA

CAAGAAACGTGAAATTCTACTGCTGTTTTACTTTATGGACATGAGCGATTCAGAAATTGC

AGACCTGTTGAAATTGAACCGTTCTACTGTCTATCGGCATAGAACCAGTGGACTAGCCTT

AATTAAAAAGTTTATGGAGGAATTTGAAGAATGAAAACACAATATCCTATGATTCCCTTT

CCTCTCATTGTAAAGGCAACAGATGGCGATACCGAAGCGATTAACCAGATTCTACATCAT

TACAGAGGGTACATAACGAAGCGTTCCCTACGACTTATGAAAGATGAATATGGCAATCAA

AGTATGGTCGTTGATGAAGTCTTACGTGGAAGAATGGAAACCAGACTGATTACAAAGATT

TTGTCATTTGAAATTAAGTAATATCCTCTCTCCTTTCGTGGAAGCGTGCTAAACCATTCC

ACGCTTCCCGAACAGGGAGGTTTGTTATTCCACCAAAGCATATTGAGCTTTCAATGTGTT

TTGATAGGCTAACGAGCCATTGTTCTTTGAAAACTGAATAAAAGTAATCGAATACGTTTC

GATAAGAAAAGAGCCAACGGAACTAACCGCCATGACCTATCTTATAAAGATAGCGAGCGA

TTCATGTTAGTGATCCGAGAAGCAATCTTTAGCAGGATTGCCTGCAACGACATTCTTATC

GTGATAATGATACTCCCATACAGTCAATAGTCCGAGCGTGATAAAACCGTCGCAGGCAAT

GAGTATGGCTACATGAGAACCATGCAGGGGTGGAACTCCCGTGAGCTTTGCTAAAGCTGT

TCGATTGCTGGTAAAACAACTTTTATGAAATCCAAATAAGTGATTTGGAAAGGAGGATTT

TATGAAGCAGACTGACATTCCTATTTGGGAACGTTATACCCTAACCATTGAAGAAGCGTC

AAAATATTTTCGTATTGGCGAAAACAAGCTACGACGCTTGGCAGAGGAAAATAAAAATGC

AAATTGGCTGATTATGAATGGCAATCGTATTCAGATTAAACGAAAACAATTTGAAAAAAT

TATAGATACATTGGACGCAATCTAGCGTCGCCAAAGGGTCTTGTATATGATAAAATAGTA

TTAAGTCGTATCAAGGCTCTTTCCATAAAGGAAAGGAGCAAATGCCATGTCAGAAAAAAG

ACGTGACAATAAAGGTCGAATCTTAAAGACTGGAGAGAGCCAACGAAAAGACGGAAGATA

CTTATACAAATATATAGATTCATTTGGAGAACCGCAATTTGTTTACTCGTGGAAACTTGT

GGCTACAGACCGAGTACCAGCAGGAAAGCGTGATTGTATCTCACTTAGAGAGAAAATCGC

AGAGTTACAGAAAGACATTCATGATGGTATTGATGTTGTAGGAAAGAAAATGACACTCTG

CCAGCTTTACGCAAAACAGAACGCTCAAAGACCAAAGGTTAGAAAAAACACTGAAACTGG

ACGCAAATATCTTATGGATATTTTGAAGAAAGACAAGTTAGGTGTAAGAAGTATTGACAG

TATTAAGCCATCAGACGCTAAAGAATGGGCTATTAGAATGAGTGAAAATGGTTATGCTTA

TCAAACCATCAATAACTACAAACGTTCTTTAAAGGCTTCATTCTATATTGCTATACAAGA

TGATTGTGTTCGGAAGAATCCATTTGACTTTCAACTGAAAGCAGTTCTTGATGATGATAC

TGTCCCTAAGACCGTACTAACAGAAGAACAGGAAGAAAAACTGTTAGCCTTTGCAAAAGC

TGATAAAACCTACAGCAAAAATTATGATGAAATTCTGATACTCTTAAAAACAGGTCTTCG

TATTTCAGAGTTTGGTGGTTTGACACTTCCAGATTTAGATTTTGAGAATCGTCTTGTCAA

TATAGACCATCAGCTATTGAGAGATACTGAAATTGGGTACTACATTGAAACACCAAAGAC

CAAAAGTGGCGAACGTCAAGTTCCTATGGTTGAAGAAGCCTATCAAGCATTTAAGCGAGT

GTTAGCGAATCGAAAGAATGATAAGCGTGTTGAGATTGATGGATATAGTGATTTCCTCTT

TCTTAATAGAAAGAACTATCCAAAAGTGGCAAGTGATTACAACGGCATGATGAAAGGTCT

TGTTAAGAAATACAATAAGTATAACGAGGATAAATTGCCACACATCACTCCACATAGTTT

GCGACATACATTCTGTACCAACTATGCAAATGCAGGAATGAATCCAAAGGCATTACAGTA

CATTATGGGACATGCTAATATAGCCATGACGCTGAACTATTACGCACATGCAACATTCGA

TTCTGCAATGGCAGAAATGAAACGCTTGAATAAAGAGAAGCAACAGGAGCGTCTTGTTGC

TTAGTAGTACAAATGAATTTACTACTTATTTACCACTTCTGACAGCTAAGACATGAGGAA

ATATGCAAAGAAACGTGAAGTATCTTCCTACAGTAAAAATACTCGAAAGCACATAGAATA

AGGCTTTACGAGCATTTAAGAAAATATAAAAAGATAATTAGAAATTTATACTTTGTTT

>GA05578/Tn916

AAAATAGCATAAAAATCTAGTTATCCGCATAAAAACTGGACTTATCACACTTTATCAAGG

TCAAAACCACTCAATTTACTACTAATTTACTACTTATGAATGAGCTTTGATACGACGATT

TATCCTTGAAAAGTGAAGATATAAAGATACTTCCAATAAAATTTGAATATTTAATAGGTA

GACACTTCAAAAAATGAGGTGTCTATTTTTTTACCCGATTTTGAAAGGAAGTGAACTTAT

GAAAACAAAAAATCAAGAATCAAAAGGTCGTTCCCCACTCTTTAAGACCATCAAACATTC

ATTCAGCCAATAAAAAAGAAAGGATAGGTAAAAATATGGAACTTAAATTTGTGATTCCCA

ACATGGAAAAAACATTCGGCAATTTAGAATTTGCTGGCGAGGATAAAGTCGTTCAGCGAA

GAATCAACGGACGGCTAACTGTCTTATCAAGAAGCTATAATCTCTATTCTGATGTTCAAA

GAGCAGATGATATTGTGGTGGTGCTTCCTGCTGAAGCTGGCGAAAAACATTTCGGCTTTG

AGGAACGTGTGAAGTTAGTCAATCCACGTATTACCGCAGAGGGCTACAAAATCGGCACTC

GTGGTTTTACAAATTACCTTTTACATGCTGACGACATGATAAAAGAATAAAGAAAGAGAG

GAAAAATGATGAGATTAGCAAATGGCATTGTATTAGATAAAGACACGACTTTTGGAGAAT

TGAAATTCTCTGCTCTACGTCGTGAAGTGAGAATCCAAAATGAAGACGGGTCGGTTTCAG

ATGAAATCAAGGAACGTACCTATGACTTAAAATCCAAAGGACAAGGACGCATGATTCAAG

TAAGTATTCCTGCCAGCGTGCCTTTGAAAGAGTTTGATTATAACGCACGGGTGGAACTTA

TCAATCCCATTGCGGACACCGTTGCTACTGCCACCTATCAAGGAGCAGATGTTGACTGGT

ATATCAAGGCAGACGATATTGTGCTGACAAAGGATTCTAGTTCATTCAAAGCTCAACCAC

AAGCAAAGAAAGAACCGACACAAGACAAATAGTCGCTAGGTAGAAAGGAGACTTTTTCGC

ATGAAACAGCGTGGTAAAAGGATTCGCCCATCTGGTAAAGATTTAGTCTTTCATTTTACG

ATAGCGTCACTCCTGCCTGTTTTCCTGCTGGTTGTCGGACTGTTTCATGTGAAGACAATC

CAGCAGATCAACTGGCAGGATTTTAACCTATCACAAGCAGATAAGATTGACATTCCCTAT

TTAATTATCAGTTTCAGTGTCGCAATTCTTATCTGCTTGCTGGTAGCGTTTGTATTCAAA

CGGGTTCGCTATGATACGGTTAAACAACTTTACCACCGTCAAAAACTGGCAAAGATGATA

CTTGAAAACAAGTGGTATGAATCTGAACAGGTCAAAACAGAGGGTTTCTTTAAAGATAGT

GCTGGTCGTACAAAGGAAAAGATAACCTACTTCCCTAAAATGTATTATCGACTTAAAAAT

GGCTTGATACAGATACGGGTGGAAATCACGCTGGGAAAATATCAAGACCAACTCTTACAC

TTGGAAAAGAAATTAGAGAGTGGCTTGTACTGTGAGCTGACGGATAAAGAGTTAAAGGAT

TCCTATGTGGAATATACTTTGCTCTATGACACCATAGCCAGTCGTATTTCTATTGATGAA

GTAGAAGCTAAAGATGGTAAACTTCGCTTAATGAAAAACGTATGGTGGGAATATGATAAG

CTCCCTCATATGTTGATTGCTGGTGGTACAGGTGGCGGTAAAACTTACTTTATACTGACA

CTGATTGAAGCCTTGCTTCATACAGATTCAAAACTGTATATTCTTGACCCGAAAAATGCT

GACCTTGCGGACTTAGGTTCTGTGATGGCAAATGTCTACTATAGAAAAGAAGACTTGCTT

TCTTGCATTGAAACATTCTATGAAGAAATGATGAAACGTAGTGAGGAAATGAAGCAGATG

AAGAACTATAAGACTGGCAAAAATTATGCTTACTTAGGTCTCCCGGCACACTTCTTAATC

TTTGATGAATACGTCGCTTTCATGGAAATGCTGGGAACAAAAGAAAACACCGCAGTTATG

AATAAGCTGAAACAGATTGTCATGTTAGGTCGTCAAGCTGGCTTCTTTCTAATACTGGCT

TGTCAACGTCCAGACGCAAAATATTTAGGCGACGGAATCCGTGATCAGTTTAATTTCAGA

GTGGCTTTAGGTCGTATGTCTGAAATGGGCTATGGCATGATGTTTGGCAGTGACGTACAA

AAGGATTTCTTCTTAAAGCGAATCAAAGGTCGTGGCTATGTTGATGTAGGAACAAGTGTC

ATATCAGAGTTTTATACTCCCCTTGTACCAAAAGGATATGATTTCTTGGAGGAAATTAAA

AAGTTATCCAACAGCAGACAGTCCACGCAGGCGACGTGCGAAGCGGAAGTCGCAGGTGTG

GACTGATCTTGCTGGCTGGTGTGGCAATAGCCACGCCAGCACTTAACCCCCCGTATCTAA

CAGGGGGGTACAAATCGACAGGAAACAGTCAAAAAAACATTAGAAAATCCTTTGGTTACA

AGGGATTTACAAAATTTCAGCGTATGTCAAATGGGCTTTAAAAGTTGACATACGCCTTTT

TGATTGGAGGGATTTTTACTGAATGAACAAACTTGGTTACAGCATTTAAAAGAAAAACGC

TTGGCTTATGGACTATCTCAAAACCGTTTAGCTGTTGCGACTGGTATTACAAGGCAGTAT

CTAAGCGATATTGAAACAGGAAAAGTCAAGCCATCAGAGGATTTACAGCAGTCCCTTTGG

GAAGCTCTGGAACGCTTCAATCCCGACGCTCCCCTTGAAATGCTGTTTGATTATGTAAGG

ATTCGCTTTCCGACAACAGACGTACAGCAGGTGGTCGAAAACATCTTACAACTGAAACTG

TCCTATTTTCTTCATGAGGACTATGGTTTCTATTCTTATTCAGAGCATTATGCTTTAGGC

GACATATTCGTCCTTTGCTCCCATGAACTGGACAAAGGAGTTCTGGTGGAATTGAAAGGT

CGTGGGTGCAGACAATTTGAAAGCTATCTTCTGGCACAACAAAGAAGCTGGTATGAGTTC

TTTATGGACGTTTTGGTGGCTGGCGGTGTGATGAAACGCCTTGACCTTGCCATTAACGAT

AAGACAGGGATTTTGAATATCCCTGTACTCACTGAAAAGTGCCAACAGGAAGAATGTATC

TCCGTCTTCCGCAGTTTTAAAAGCTATCGCAGTGGCGAACTGGTACGCAAAGAGGAAAAG

GAATGTATGGGAAACACCCTCTATATCGGTTCATTACAAAGTGAAGTTTATTTCTGTATC

TATGAAAAGGACTACGAGCAGTACAAGAAAAATGATATTCCCATTGAAGACGCAGAAGTA

AAAAACCGTTTTGAGATTCGATTGAAAAATGAGCGTGCCTATTATGCAGTCCGTGATTTA

CTCGTCTATGACAATCCAGAGCATACCGCCTTTAAAATTATCAATCGGTATATCCGTTTT

GTAGATAAAGACGATTCCAAACCTCGTTCTGATTGGAAACTGAATGAAGAATGGGCTTGG

TTTATTGGGAACAATCGTGAACGATTAAAACTAACCACAAAACCAGAGCCTTACTCCTTC

CAAAGGACGCTGAACTGGCTATCTCATCAAGTTGCCCCGACCTTAAAGGTTGCGATTAAA

CTTGATGAAATCAACCAGACGCAGGTTGTAAAAGACATTCTCGACCATGCGAAACTGACA

GACCGACACAAGCAGATTTTGAAGCAACAGTCAGTAAAAGAACAGGACGTGATAACAACA

AAAAAATAACTCAAATACAAATTCATTGAATATAGAGAGGAGAACATTTTTATGAATTTT

GGACAAAACCTTTATAACTGGTTTCTATCAAACGCTCAATCACTGGTGCTTTTAGCAATC

GTTGTGATTGGCTTGTATCTTGGCTTCAAGCGTGAGTTTAGCAAACTGATTGGCTTTTTA

ATTATTGCGATTATTGCGGTTGGCTTAGTCTTCAACGCTGCTGGAGTAAAAGACATTTTA

CTAGAGCTATTCAATCGCATTATTGGTGCTTAAATAAAACCGTTCTTTTGTGGAATATAA

GTGGTTTTCTTATGTTCCGCAAAGGAATGGTACACCAAACGAAGTGCGGTAGGGATTTTT

GAATCTCTACAAAGAAAGGACGTGAATATATGGACGATATGCAAGTCTATATTGCGAATT

TAGGCAAATACAATGAGGGCGAATTGGTCGGTGCGTGGTTTACCTTTCCCATTGACTTTG

AGGAAGTCAAAGAGAAAATCGGCTTGAATGATGAATATGAGGAATACGCCATTCATGACT

ACGAGTTACCCTTTACGGTTGACGAATACACTTCCATTGGCGAACTCAATCGACTATGGG

AAATGGTATCGGAATTACCCGAAGAATTACAATCGGAGCTATCTGCTCTGCTCACTCATT

TTTCAAGCATTGAAGAACTAAGCGAACATCAAGAGGATATTATCATTCATTCCGATTGTG

ATGATATGTATGACGTGGCACGCTACTACATTGAAGAAACGGGTGCTTTAGGCGAAGTAC

CAGCTAGTCTTCAAAACTATATTGATTATCAAGCCTATGGTCGGGATTTAGACCTTTCAG

GAACGTTTATCTCAACCAATCATGGGATTTTTGAAATCGTCTATTAAATCTGTCGGTACA

TTACTACTGGCAGATTTTCTATTTTACGGGGTGGCTCAATCAGCTACCCCTATTTTTTAT

GAAAGGATTGATTACATGAAGAAAATACGAAGCTATACCAGTATCTGGTCTGTGGAAAAG

GTACTGTATTCTATCAATGATTTTAGACTTCCGTTTCCCATAACCTTTACGCAAATGACA

TGGTTTGTCGTGTCACTCTTTGCAGTGATGATACTTGGCAACTTGCCCCCTCTTTCCATG

ATAGAGGGAGCATTTCTCAAATACTTTGGGATTCCTGTGGCTTTCACATGGTTTATGTCT

ACAAAAACTTTTGATGGTAAAAAGCCTTATGGATTTTTGAAGTCTGTCATTGCTTATGCA

CTGCGACCAAAGCTGACCTATGCAGGAAAAAAAGTAACGCTTGGCAGAAACCAGCCACAA

GAAGCCATTACAGCAGTTAGGAGTGAATTTTATGGCATATCCAATTAAATACATTGAAAA

CAATCTCGTCTGGAATAAAGACGGGGAATGTTATGCTTACTATGAGCTTGTTCCTTACAA

TTACTCATTTCTAAGTCCAGAACAGAAAATACAAGTGCATGATTCTTTCAGACAGCTTAT

CGCACAAAATCGTGATGGCAAAATTCATGCTTTACAAATCAGTACAGAATCCAGCATACG

TTCTGCACAAGAGCGTTCCAAAAATGAAGTCACTGGCAAGCTCAAAGCGGTTGCCTATGA

CAAAATCGACCAACAGACAGACGCTTTAATATCCATGATTGGCGAAAATCAAGTGAACTA

CCGTTTCTTTATCGGCTTTAAGTTGCTTCTCAACGATCAGGAGTTTTCTATGAAAAGTCT

TACCGTTGAAGCAAAAAATGCTTTGTCTGATTTTGTCTATGATGTGAACCATAAGCTGAT

GGGCGATTTTGTTAGTATGAGTAATGATGAAATCCTGCGTTTTCAGAAGATGGAAAAGCT

CTTAGAAAATAAAATCTCTCGTCGTTTCAAAATCCGCAGGTTAGATAAGGACGACTTCGG

CTATCTGATTGAACACCTTTACGGACAGACAGGCACTGCCTATGAAGAGTATGAGTACCA

TCTATCAAAGAAAAAGCTGGATAATGAAACGCTGATTAAATACTATGACTTGATTAAGCC

TACTCGCTGTTTGGTGGAAGAAAAACAGCGATATTTGAAAATCCAGCAGGAAGATGAAAC

CGTCTATGTAGCTTACTTTACCATTAACAGCATTGTCGGAGAACTGGACTTCCCGTCCTC

TGAAATCTTCTACTACCAGCAACAGCAATTTACATTCCCGATTGATACGTCAATGAATGT

GGAAATTGTAGCGAATCGTAAAGCCCTATCTACTGTCCGCAATAAAAAGAAAGAACTGAA

AGACTTGGATAACCACGCTTGGCAAAGTGATAATGAAACCAGCTCCAATGTGGCGGAAGC

TCTGGAAAGTGTGAATGAGCTGGAAACCAATTTAGACCAAAGCAAGGAATCTATGTACAA

GCTGTCTTATGTGGTAAGGGTATCAGCAAATGATCTTGACGAACTCAAACGTCGTTGTAA

TGAAGTGAAAGATTTTTATGACGATTTAAGCGTAAAACTGGTACGACCATTTGGGGATAT

GCTCGGCTTACATGAAGAATTTTTACCTGCCAGCAAGCGTTATATGAATGATTATATTCA

ATACGTGACCTCTGATTTCCTCGCTGGTTTAGGTTTTGGTGCTACTCAAATGCTGGGGGA

AAATGAGGGGATTTATGTTGGCTACAGCTTAGATACTGGACGCAATGTCTATCTGAAACC

TGCTCTTGCCAGTCAAGGGGTTAAGGGTTCAGTAACCAATGCGTTAGCGTCGGCTTTTGT

TGGTTCGCTGGGTGGTGGTAAATCCTTTGCGAATAACCTTATCGTCTATTATGCGGTGCT

TTATGGGGCACAAGCAGTGATTGTAGACCCAAAAGCAGAACGTGGCAGATGGAAAGAAAC

CTTGCCAGAGATTTCCCATGAAATCAATATCGTCACTCTGACTTCTGATGAGAAAAACAA

AGGCTTACTTGACCCTTATGTGATTATGAAAAATCCCAAAGATTCTGAATCACTGGCTAT

TGATATTCTGACATTCCTTACGGGGATTTCCTCTCGTGATGGGGAACGCTTCCCAATCCT

TAGAAAAGCCATTCGTGCAGTAACCAATAGTGAAGTACGAGGGTTGATGAAAGTGATTGA

GGAATTACGGGTTGAGAATACGCCACTAAGTACCAGTATAGCCGACCATATCGAAAGTTT

TACAGACTATGACTTTGCACATTTATTATTCAGTAATGGTTATGTGGAGCAGTCTATCAG

CTTAGAAAAACAACTGAACATTATACAGGTTGCGGACTTGGTACTTCCCGACAAGGAAAC

TTCCTTTGAGGAATATACCACTATGGAGCTTTTATCCGTTGCTATGCTGATTGTCATTAG

TACCTTTGCTTTAGACTTTATCCATACAGACCGAAGCATTTTCAAGATTGTAGATTTAGA

CGAAGCATGGAGCTTTTTACAGGTAGCACAAGGAAAAACACTATCTATGAAGCTGGTTCG

GGCTGGTCGTGCTATGAACGCTGGGGTATATTTCGTGACCCAAAATACAGACGACCTCTT

AGATGAAAAACTGAAAAATAACCTCGGCTTAAAATTTGCATTTCGTTCCACTGACCTTAA

CGAGATTAAAAAGACCTTAGCCTTTTTTGGTGTAGACCCAGAGGACGAAAACAATCAGAA

GCGATTGCGTGATTTGGAAAACGGGCAATGCCTTATCAGTGATTTATATGGTCGTGTCGG

TGTGATACAGTTCCACCCTGTATTTGAAGAACTGCTCCATGCCTTTGATACCAGACCACC

TGTGCGAAAAGAGGTGTAAATGTGAAACCATCAATAGTAAACAGAATAAAATCAAACTGG

ACGCTGAAACGTCTAGGTAAAGTGGCAATGACAGTGGCTTTCACACTTGTGATTGCCATT

TTTCTTTTAGCCATGCTGGGAACGGTGGTTCAAGCTGCGGGCTTGGTAGATGATACGGTC

AATGTGGCAAATGAATACAGCCGATACCCACTTGAAAACTATCAACTGGATTTTTATGTG

GATAATAGCTGGGGCTGGCTTCCGTGGAACTGGTCGGACGGGATTGGAAAACAGGTCATG

TATGGACTATATGCCATTACCAATTTTATTTGGACAATCAGTTTGTATGTTTCCAATGCG

ACAGGTTACTTAGTACAGGAAGCCTATTCCTTAGACTTCATTTCCGCTACAGCAGATTCC

ATTGGTAAGAATATGCAGACCTTAGCTGGTGTGAGTGCAAACGGATTTTCAACAGAGGGT

TTCTATGTTGGATTCCTCTTACTCTTGATTTTGGTTCTTGGGGTTTATGTTGCCTATACG

GGACTGATAAAGAGAGAAACCACAAAGGCAATTCATGCCATTATGAATTTTGTGCTGGTG

TTTATCCTATCGGCTTCCTTTATTGCCTACGCTCCCGACTACATTAAAAAAATCAATGAC

TTTTCATCAGACATCAGTAATGCCAGTTTATCACTTGGCACGAAGATTGTCATGCCCCAT

TCCGATAGTCAAGGCAAGGACAGCGTGGACTTAATCAGAGATAGCCTGTTTTCCATACAG

GTTCAGCAACCGTGGCTACTGCTTCAATACAACAGTTCAGACATTGAAAGTATCGGTATT

GACCGTGTGGAAAGCCTGCTCTCCACCAGCCCAGATTCCAACAATGGCGAAGACAGAGAA

AAAATTGTTGCGGAAGAAATTGAAGACAGAAGCAATACCAATCTAACCATTACAAAGACC

ATTAACCGTTTAGGTACAGTCTTCTTCCTATTTGTCTTCAATATTGGGATTTCCATATTT

GTATTCCTATTAACAGGAATCATGATTTTCTCGCAGGTACTTTTTATCATCTATGCTATG

TTTCTGCCTGTGAGCTTTATTTTAAGCATGATTCCATCATTTGATGGTATGTCAAAACGA

GCCATAACAAAGCTCTTTAATACCATTTTGACACGAGCTGGAATCACATTGATTATTACG

ACAGCATTTAGTATTTCAACCATGCTCTATACCTTATCGGCTGGTTATCCGTTCTTTTTG

ATTGCTTTTCTACAGATTGTGACCTTTGCAGGAATCTACTTCAAGCTGGGCGATTTAATG

AGTATGTTTTCTCTACAGAGTAACGATTCTCAAAGTGTGGGAAGTCGTGTGATGAGAAAA

CCTCGTATGCTTATGCACGCTCACATGCACCGTCTACAGCGGAAACTTGGACGTTCCATG

ACTACTCTAGGGGCTGGGTCTGCCATTGTTACAGGTAAAAAAGGACAGTCGGGTTCGGGG

AGTTCTGCAAGGACACAAGCAGATCACTCCCGACCAGACGGAAAGGAAAAATCAACACTT

GGAAAACGTATCGGTCAAACCATCGGTACAGTAGCTGATACCAAAGACAGAATGGTAGAC

ACTGCTAGTGGTTTGAAAGAACAGGTTAAAGATTTGCCGACCAATGCAAGATATGCAGTA

TATCAAGGAAAATCCAAAGTAAAAGAGAATGTCCGTGATTTAACCAGTAGTATTTCTCAA

ACCAAAGCGGACAGAGCCAGTGGACGCAAGGAACAGCAGGAACAAAGGCGAAAAACCATT

GCGAAGCGTCGCTCTGAAATGGAACAGGTCAAACAGAAAAAACAGCCTGCTTCTTCTGTT

CATGAAAGACCGACTACAAGACAAGAACAATATCATGATGAACAGACCTCAAAACAGTCT

AATATTCAGACTTCATATAAGGAATCTCAACAAGCCAAACAAGAGCGTCCAGCAGTTAAG

TCCGATTTTTCAAGTCCAAAAGTGGAACGCCAAGGCAATACCGTTCAAGAAAAAACCGTT

CAAAAGCCAGCAACTTCAACCACTACAGCAGATAGAACTTCACAACGTCCAATCACAAAA

GAACGTCCGTCTACTGTTCAAAGAGTACCACTACAAAATACAAGAAGTAGACCACCAATC

AAAACCGCCACCATTAAGAAAGTCGGTAAGAAACCATGAAGTTGAAAACTTTAGTGATTG

GTGGTTCTGGATTATTCTTGATGGTCTTCTCACTGCTTCTGTTTGTTGCCATTTTATTTT

CAGATGAACAGGACAGCGGAATTTCCAATATTCATTATGGAGGTGTGAATGTTTCCGCAG

AAGTGCTGGCTCATAAGCCTATGGTAGAAAAATATGCCAAAGAATATGGCGTTGAAGAAT

ATGTCAACATACTTCTTGCGATTATACAGGTGGAATCGGGCGGTACTGCGGAAGATGTTA

TGCAGTCCTCGGAATCCCTCGGTCTTCCACCTAATTCATTGAGTACAGAAGAATCCATTA

AGCAAGGTGTGAAGTATTTCAGTGAATTATTAGCCAGTAGCGAAAGGCTCAGTGTAGATT

TAGAATCGGTTATCCAGTCCTACAATTATGGTGGTGGTTTCTTAGGGTATGTGGCTAATC

GTGGAAATAAATATACCTTTGAACTGGCTCAAAGTTTCTCAAAAGAGTATTCAGGTGGCG

AAAAAGTGTCTTACCCCAATCCCATAGCCATACCTATCAATGGGGGCTGGCGATACAACT

ATGGCAATATGTTTTATGTGCAACTGGTAACGCAGTATCTTGTCACAACAGAGTTTGATG

ATGATACGGTACAAGCCATCATGGACGAAGCACTGAAATATGAGGGCTGGCGATACGTTT

ACGGTGGAGCTTCCCCGACTACTTCTTTTGATTGTAGCGGACTGACACAATGGACGTATG

GAAAAGCTGGAATTAACTTACCACGAACCGCACAACAGCAATATGATGTGACCCAGCATA

TCCCACTATCGGAAGCACAAGCTGGCGATTTGGTTTTCTTTCATTCTACCTATAACGCTG

GCTCTTATATTACTCATGTTGGGATATACCTTGGCAATAACCGTATGTTTCATGCAGGCG

ACCCAATCGGTTATGCCGACTTAACAAGCCCCTACTGGCAACAGCATTTAGTGGGAGCAG

GACGAATCAAACAATGAGAAAGGAAGATTTAATGATGAAATTTAGAAAAAATCAGAATAA

AGAAAAACAGATACCAAAGGAAAAGAAACCTCGTGTCTACTATAAGGTCAATCCTCATAA

AAAGGTTGTGATTGCCTTGTGGGTACTTTTAGGGCTTAGTTTCAGCTTTGCGATATTCAA

GCACTTTACAGCTATAGATACTCATACTATTCACGAAACAACTATCATAGAAAAGGAATA

CGTTGATACTCATCATGTAGAAAATTTTGTAGAGAACTTTGCGAAAGTCTACTATTCATG

GGAGCAATCCGATAAGTCCATTGATAATCGAATGGAAAGTCTAAAAGGCTATCTGACAGA

TGAACTTCAAGCTCTCAATGTTGATACAGTACGCAAAGATATTCCTGTATCGTCTTCTGT

AAGAGGATTTCAGATATGGACGGTAGAGCCAACTGGCGACAATGAGTTTAATGTAACCTA

CAGTGTAGACCAGCTCATTACAGAGGGAGAAAATACAAAGACCGTCCACTCTGCTTATAT

AGTGAGTGTCTATGTAGATGGTTCTGGAAATATGGTACTGGTTAAGAATCCGACCATTAC

CAACATACCTAAGAAATCAAGTTATAAACCAAAAGCCATTGAAAGTGAGGGGACGGTTGA

TTCCATTACAACCAATGAAATCAATGAGTTTTTAACGACGTTCTTCAAGCTCTATCCTAC

AGCGACAGCCAGTGAACTTTCCTACTATGTGAATGACGGGATATTAAAACCAATCGGAAA

AGAGTACATCTTTCAAGAACTGGTAAATCCTATTCACAATCGTAAGGATAATCAAGTCAC

GGTATCGCTGACAGTGGAGTATATCGACCAGCAGACCAAAGCAACGCAGGTATCTCAATT

TGATTTGGTACTTGAAAAGAACGGGAGTAATTGGAAGATTATAGAATAACAAATATTGGT

ACATTATTACAGCTATTTTGTAATCACGTACTCTCTTTGATAAAAAATTGGAGATTCCTT

TACAAATATGCTCTTACGTGCTATTATTTAAGTATCTATTTAAAAGGAGTTAATAAATAT

GCGGCAAGGTATTCTTAAATAAACTGTCAATTTGATAGTGGGAACAAATAATTGGATGCC

CTTTTGGGCTTTTGAATGGAGGAAAATCACATGAAAATTATTAATATTGGAGTTTTAGCT

CATGTTGATGCGGGAAAAACTACCTTAACAGAAAGCTTATTATATAACAGTGGAGCGATT

ACAGAATTAGGAAGCGTGGACAGAGGTACAACGAAAACGGATAATACGCTTTTAGAACGT

CAGAGAGGAATTACAATTCAGACGGCGATAACCTCTTTTCAGTGGAAAAATACTAAGATG

AACATCATAGACACGCCAGGACATATGGATTTTTTAGCAGAAGTATATCGTTCATTATCA

GTATTAGATGGGGCAATTCTACTGATTTCTGCAAAAGATGGCGTACAAGCACAAACTCGT

ATATTGTTTCATGCACTTAGGAAAATAGGTATTCCCACAATCTTTTTTATCAATAAGATT

GACCAAAATGGAATTGATTTATCAACGGTTTATCAGGATATTAAAGAGAAACTTTCTGCG

GAAATTGTAATCAAACAGAAGGTAGAACTGCATCCTAATATGCGTGTAATGAACTTTACC

GAATCTGAACAATGGGATATGGTAATAGAAGGAAATGATTACCTTTTGGAGAAATATACG

TCTGGGAAATTATTGGAAGCATTAGAACTCGAACAAGAGGAAAGCATAAGATTTCATAAT

TGTTCCCTGTTCCCTGTTTATCACGGAAGTGCAAAAAACAATATAGGGATTGATAACCTT

ATAGAAGTGATTACGAATAAATTTTATTCATCAACACATCGAGGTCAGTCTGAACTTTGC

GGAAAAGTTTTCAAAATTGAGTATTCGGAAAAAAGACAGCGTCTTGCATATATACGTCTT

TATAGTGGCGTACTGCATTTGCGAGATTCGGTTAGAATATCGGAAAAGGAAAAATAAAAA

TTACAGAAATGTATACTTCAATAAATGGTGAATTATGTAAAATCGATAAGGCTTATTCCG

GGGAAATTGTTATTTTGCAGAATGAGTTTTTGAAGTTAAATAGTGTTCTTGGAGATACAA

AGCTATTGCCACAGAGAGAGAATTGAAAATCCCCTCCCTCTGCTGCAAACGACTGTTGAA

CCGAGCAAACCTCAACAAAGGGAAATGTTACTTGATGCACTTTTAGAAATCTCCGACAGT

GACCCGCTTCTGCGATATTATGTGGATTCTGCGACACATGAAATCATACTTTCTTTCTTA

GGGAAAGTACAAATGGAAGTGACTTGTGCTCTGCTGCAAGAAAAGTATCATGTGGAGATA

GAAATAAAAGAGCCTACAGTCATTTATATGGAAAGACCGTTAAAAAAAGCAGAGTATACC

ATTCACATCGAAGTTCCACCGAATCCTTTCTGGGCTTCCATTGGTCTATCTGTAGCACAG

CTTCCATTAGGGAGCGGAGTACAGTATGAGAGCTCGGTTTCTCTTGGATACTTAAATCAA

TCGTTTCAAAATGCAGTTATGGAGGGGATACGCTATGGCTGTGAACAAGGATTGTATGGT

TGGAATGTGACGGACTGTAAAATCTGTTTTAAGTATGGCTTATACTATAGCCCTGTTAGT

ACCCCAGCAGATTTTCGGATGCTTGCTCCTATTGTATTGGAACAAGTCTTAAAAAAAGCT

GGAACAGAATTGTTAGAGCCATATCTTAGTTTTAAAATTTATGCGCCACAGGAATATCTT

TCACGAGCATACAACGATGCTCCTAAATATTGTGCGAACATCGTAGACACTCAATTGAAA

AATAATGAGGTCATTCTTAGTGGAGAAATCCCTGCTCGGTGTATTCAAGAATATCGTAGT

GATTTAACTTTCTTTACAAATGGACGTAGTGTTTGTTTAACAGAGTTAAAAGGGTACCAT

GTTACTACCGGTGAACCTGTTTGCCAGCCCCGTCGTCCAAATAGTCGGATAGATAAAGTA

CGATATATGTTCAATAAAATAACTTAGTGTATTTTATGTTGTTATATAAATATGGTTTCT

TGTTAAATAAGATGAAATATTTTTTAATAAAGATTTGAATTAAAGTGTAAAGGAGGAGAT

AGTTATTATAAACTACAAGTGGATATTGTGTCCTGTATGTGGAAATAAAACACGATTAAA

GATAAGGGAAGATACTGAATTAAAAAAATTCCCCCTCTATTGTCCGAAATGCAGACAAGA

AAATTTAATTGAAATAAAGCAGTTCAAAGTAACTGTGATTACAGAGCCAGACGCAAAGAC

GCAGAGCCGATAAAATGAGATTAATACAATCTCATTTTATCGGCTCTTTCCGTTATGTAT

GGATTCTTTTAATTAGTCTTCGATGTTTCTTGCTTCGTTGATACCGCTGGCTAAAGATTC

CATTAAGGATAGTTCTTTGTCTGTAAAGCTATCCATGTATTTCTCTATCTGTAATCGTCG

GGTGCTTTTTACCAAGTTATTAGCAGGTAAGAAAAATTCATCAACGGAAACATGAAGTAA

CGATACAAGGTCATAAAGAACTTGTATGCTGGGGTGTTGCCCTTTATTTTCAATATTAGT

TAAGTACCGTGGGTCAATTTCAATCAATGCTCCCACTTGTTCACGAGTTAAACCTCGTTT

CAATCGAGCTTCTTTAATGGCTAAACCAAAGGCTCTAAAATCATATTTATCTTCTTTTTT

ACGCATAGTAGACCACCTCTATACATTTTATTGTTCCTACTGAATTAAAAACAGGTATAG

AAAAACGTGTTATATGGTTTATAGGTTTATATTTAATAAAAAGCACTACTAAACGCCAAT

AAAAAAAACCGTTATATGGTAGTGCTATTTACGCTGTTAAAATATTGTATATTACTTCCA

AATGGCGGTTTGTTGGAGGTCAACGTCGCCATGAAGTACATCATATACAATAAATTTCCT

TACATTGGGTTCTTGTCAAAAAAAGTCGTCTATCTGCAATAGATAAGTACGTCCACCAAT

GTGGTTTTATAAATCATATAGATAGAATAACAGAAGCATGTAAACAGAGAAATAAATCTG

TTTATATGCTTTTTTGGCTATTCAGAACTTTTTTACAAAGTTTATTTATCAGTAATGCAA

CAAATCCCCCTTTCACATTGGGACTAAGAGTGAAAGGAGATAAACGAGCAAGGCTCACTT

CCTTTCCTAGACAGAAAGGGGGTGAGAAACATGAAACCATCTTCTTTTCAGACCACAATA

GAAAATCAGTTTGACTATATCTGTAAACGTGCTATGGAAGACGAGCGAAAGAATTATATG

CTTTATCTTTCAAGGATTGCAAAGCGTGAGGTGTCCTTTTCGGATGTTGGCGATTATCTT

GTTAGCCAGTTTGCGACAACAGATAACTATTCAACTGACTTTCAGATTTTTACACTCAAT

GGGTTATCAGTAGGCGTTGAAAATGATTTGTTGAGTGAAGCATTACGTGAGTTGCCAGAC

AAGAAACGTGAAATTCTACTGCTGTTTTACTTTATGGACATGAGCGATTCAGAAATTGCA

GACCTGTTGAAATTGAACCGTTCTACTGTCTATCGGCATAGAACCAGTGGACTAGCCTTA

ATTAAAAAGTTTATGGAGGAATTTGAAGAATGAAAACACAATATCCTATGATTCCCTTTC

CTCTCATTGTAAAGGCAACAGATGGCGATACCGAAGCGATTAACCAGATTCTACATCATT

ACAGAGGGTACATAACGAAGCGTTCCCTACGACTTATGAAAGATGAATATGGCAATCAAA

GTATGGTCGTTGATGAAGTCTTACGTGGAAGAATGGAAACCAGACTGATTACAAAGATTT

TGTCATTTGAAATTAAGTAATATCCTCTCTCCTTTCGTGGAAGCGTGCTAAACCATTCCA

CGCTTCCCGAACAGGGAGGTTTGTTATTCCACCAAAGCATATTGAGCTTTCAATGTGTTT

TGATAGGCTAACGAGCCATTGTTCTTTGAAAACTGAATAAAAGTAATCGAATACGTTTCG

ATAAGAAAAGAGCCAACGGAACTAACCGCCATGACCTATCTTATAAAGATAGCGAGCGAT

TCATGTTAGTGATCCGAGAAGCAATCTTTAGCAGGATTGCCTGCAACGACATTCTTATCG

TGATAATGATACTCCCATACAGTCAATAGTCCGAGCGTGATAAAACCGTCGCAGGCAATG

AGTATGGCTACATGAGAACCATGCAGGGGTGGAACTCCCGTGAGCTTTGCTAAAGCTGTT

CGATTGCTGGTAAAACAACTTTTATGAAATCCAAATAAGTGATTTGGAAAGGAGGATTTT

ATGAAGCAGACTGACATTCCTATTTGGGAACGTTATACCCTAACCATTGAAGAAGCGTCA

AAATATTTTCGTATTGGCGAAAACAAGCTACGACGCTTGGCAGAGGAAAATAAAAATGCA

AATTGGCTGATTATGAATGGCAATCGTATTCAGATTAAACGAAAACAATTTGAAAAAATT

ATAGATACATTGGACGCAATCTAGCGTCGCCAAAGGGTCTTGTATATGATAAAATAGTAT

TAAGTCGTATCAAGGCTCTTTCCATAAAGGAAAGGAGCAAATGCCATGTCAGAAAAAAGA

CGTGACAATAAAGGTCGAATCTTAAAGACTGGAGAGAGCCAACGAAAAGACGGAAGATAC

TTATACAAATATATAGATTCATTTGGAGAACCGCAATTTGTTTACTCGTGGAAACTTGTG

GCTACAGACCGAGTACCAGCAGGAAAGCGTGATTGTATCTCACTTAGAGAGAAAATCGCA

GAGTTACAGAAAGACATTCATGATGGTATTGATGTTGTAGGAAAGAAAATGACACTCTGC

CAGCTTTACGCAAAACAGAACGCTCAAAGACCAAAGGTTAGAAAAAACACTGAAACTGGA

CGCAAATATCTTATGGATATTTTGAAGAAAGACAAGTTAGGTGTAAGAAGTATTGACAGT

ATTAAGCCATCAGACGCTAAAGAATGGGCTATTAGAATGAGTGAAAATGGTTATGCTTAT

CAAACCATCAATAACTACAAACGTTCTTTAAAGGCTTCATTCTATATTGCTATACAAGAT

GATTGTGTTCGGAAGAATCCATTTGACTTTCAACTGAAAGCAGTTCTTGATGATGATACT

GTCCCTAAGACCGTACTAACAGAAGAACAGGAAGAAAAACTGTTAGCCTTTGCAAAAGCT

GATAAAACCTACAGCAAAAATTATGATGAAATTCTGATACTCTTAAAAACAGGTCTTCGT

ATTTCAGAGTTTGGTGGTTTGACACTTCCAGATTTAGATTTTGAGAATCGTCTTGTCAAT

ATAGACCATCAGCTATTGAGAGATACTGAAATTGGGTACTACATTGAAACACCAAAGACC

AAAAGTGGCGAACGTCAAGTTCCTATGGTTGAAGAAGCCTATCAAGCATTTAAGCGAGTG

TTAGCGAATCGAAAGAATGATAAGCGTGTTGAGATTGATGGATATAGTGATTTCCTCTTT

CTTAATAGAAAGAACTATCCAAAAGTGGCAAGTGATTACAACGGCATGATGAAAGGTCTT

GTTAAGAAATACAATAAGTATAACGAGGATAAATTGCCACACATCACTCCACATAGTTTG

CGACATACATTCTGTACCAACTATGCAAATGCAGGAATGAATCCAAAGGCATTACAGTAC

ATTATGGGACATGCTAATATAGCCATGACGCTGAACTATTACGCACATGCAACATTCGAT

TCTGCAATGGCAGAAATGAAACGCTTGAATAAAGAGAAGCAACAGGAGCGTCTTGTTGCT

TAGTAGTACAAATGAATTTACTACTTATTTACCACTTCTGACAGCTAAGACATGAGGAAA

TATGCAAAGAAACGTGAAGTATCTTCCTACAGTAAAAATACTCGAAAGCACATAGAATAA

GGCTTTACGAGCATTTAAGAAAATATAAAAAGATAATTAGAAATTTATACTTTGTTT

>GA41538/Tn916

AAAATAGCATAAAAATCTAGTTATCCGCATAAAAACTGGACTTATCACACTTTATCAAGG

TCAAAACCACTCAATTTACTACTAATTTACTACTTATGAATGAGCTTTGATACGACGATT

TATCCTTGAAAAGTGAAGATATAAAGATACTTCCAATAAAATTTGAATATTTAATAGGTA

GACACTTCAAAAAATGAGGTGTCTATTTTTTTACCCGATTTTGAAAGGAAGTGAACTTAT

GAAAACAAAAAATCAAGAATCAAAAGGTCGTTCCCCACTCTTTAAGACCATCAAACATTC

ATTCAGCCAATAAAAAGAAAGGATAGGTAAAAATATGGAACTTAAATTTGTGATTCCCAA

CATGGAAAAAACATTCGGCAATTTAGAATTTGCTGGCGAGGATAAAGTCGTTCAGCGAAG

AATCAACGGACGGCTAACTGTCTTATCAAGAAGCTATAATCTCTATTCTGATGTTCAAAG

AGCAGATGATATTGTGGTGGTGCTTCCTGCTGAAGCTGGCGAAAAACATTTCGGCTTTGA

GGAACGTGTGAAGTTAGTCAATCCACGTATTACCGCAGAGGGCTACAAAATCGGCACTCG

TGGTTTTACAAATTACCTTTTACATGCTGACGACATGATAAAAGAATAAAGAAAGAGAGG

AAAAATGATGAGATTAGCAAATGGCATTGTATTAGATAAAGACACGACTTTTGGAGAATT

GAAATTCTCTGCTCTACGTCGTGAAGTGAGAATCCAAAATGAAGACGGGTCGGTTTCAGA

TGAAATCAAGGAACGTACCTATGACTTAAAATCCAAAGGACAAGGACGCATGATTCAAGT

AAGTATTCCTGCCAGCGTGCCTTTGAAAGAGTTTGATTATAACGCACGGGTGGAACTTAT

CAATCCCATTGCGGACACCGTTGCTACTGCCACCTATCAAGGAGCAGATGTTGACTGGTA

TATCAAGGCAGACGATATTGTGCTGACAAAGGATTCTAGTTCATTCAAAGCTCAACCACA

AGCAAAGAAAGAACCGACACAAGACAAATAGTCGCTAGGTAGAAAGGAGACTTTTTCGCA

TGAAACAGCGTGGTAAAAGGATTCGCCCATCTGGTAAAGATTTAGTCTTTCATTTTACGA

TAGCGTCACTCCTGCCTGTTTTCCTGCTGGTTGTCGGACTGTTTCATGTGAAGACAATCC

AGCAGATCAACTGGCAGGATTTTAACCTATCACAAGCAGATAAGATTGACATTCCCTATT

TAATTATCAGTTTCAGTGTCGCAATTCTTATCTGCTTGCTGGTAGCGTTTGTATTCAAAC

GGGTTCGCTATGATACGGTTAAACAACTTTACCACCGTCAAAAACTGGCAAAGATGATAC

TTGAAAACAAGTGGTATGAATCTGAACAGGTCAAAACAGAGGGTTTCTTTAAAGATAGTG

CTGGTCGTACAAAGGAAAAGATAACCTACTTCCCTAAAATGTATTATCGACTTAAAAATG

GCTTGATACAGATACGGGTGGAAATCACGCTGGGAAAATATCAAGACCAACTCTTACACT

TGGAAAAGAAATTAGAGAGTGGCTTGTACTGTGAGCTGACGGATAAAGAGTTAAAGGATT

CCTATGTGGAATATACTTTGCTCTATGACACCATAGCCAGTCGTATTTCTATTGATGAAG

TAGAAGCTAAAGATGGTAAACTTCGCTTAATGAAAAACGTATGGTGGGAATATGATAAGC

TCCCTCATATGTTGATTGCTGGTGGTACAGGTGGCGGTAAAACTTACTTTATACTGACAC

TGATTGAAGCCTTGCTTCATACAGATTCAAAACTGTATATTCTTGACCCGAAAAATGCTG

ACCTTGCGGACTTAGGTTCTGTGATGGCAAATGTCTACTATAGAAAAGAAGACTTGCTTT

CTTGCATTGAAACATTCTATGAAGAAATGATGAAACGTAGTGAGGAAATGAAGCAGATGA

AGAACTATAAGACTGGCAAAAATTATGCTTACTTAGGTCTCCCGGCACACTTCTTAATCT

TTGATGAATACGTCGCTTTCATGGAAATGCTGGGAACAAAAGAAAACACCGCAGTTATGA

ATAAGCTGAAACAGATTGTCATGTTAGGTCGTCAAGCTGGCTTCTTTCTAATACTGGCTT

GTCAACGTCCAGACGCAAAATATTTAGGCGACGGAATCCGTGATCAGTTTAATTTCAGAG

TGGCTTTAGGTCGTATGTCTGAAATGGGCTATGGCATGATGTTTGGCAGTGACGTACAAA

AGGATTTCTTCTTAAAGCGAATCAAAGGTCGTGGCTATGTTGATGTAGGAACAAGTGTCA

TATCAGAGTTTTATACTCCCCTTGTACCAAAAGGATATGATTTCTTGGAGGAAATTAAAA

AGTTATCCAACAGCAGACAGTCCACGCAGGCGACGTGCGAAGCGGAAGTCGCAGGTGTGG

ACTGATCTTGCTGGCTGGTGTGGCAATAGCCACGCCAGCACTTAACCCCCCGTATCTAAC

AGGGGGGTACAAATCGACAGGAAACAGTCAAAAAACATTAGAAAATCCTTTGGTTACAAG

GGATTTACAAAATTTCAGCGTATGTCAAATGGGCTTTAAAAGTTGACATACGCCTTTTTG

ATTGGAGGGATTTTTACTGAATGAACAAACTTGGTTACAGCATTTAAAAGAAAAACGCTT

GGCTTATGGACTATCTCAAAACCGTTTAGCTGTTGCGACTGGTATTACAAGGCAGTATCT

AAGCGATATTGAAACAGGAAAAGTCAAGCCATCAGAGGATTTACAGCAGTCCCTTTGGGA

AGCTCTGGAACGCTTCAATCCCGACGCTCCCCTTGAAATGCTGTTTGATTATGTAAGGAT

TCGCTTTCCGACAACAGACGTACAGCAGGTGGTCGAAAACATCTTACAACTGAAACTGTC

CTATTTTCTTCATGAGGACTATGGTTTCTATTCTTATTCAGAGCATTATGCTTTAGGCGA

CATATTCGTCCTTTGCTCCCATGAACTGGACAAAGGAGTTCTGGTGGAATTGAAAGGTCG

TGGGTGCAGACAATTTGAAAGCTATCTTCTGGCACAACAAAGAAGCTGGTATGAGTTCTT

TATGGACGTTTTGGTGGCTGGCGGTGTGATGAAACGCCTTGACCTTGCCATTAACGATAA

GACAGGGATTTTGAATATCCCTGTACTCACTGAAAAGTGCCAACAGGAAGAATGTATCTC

CGTCTTCCGCAGTTTTAAAAGCTATCGCAGTGGCGAACTGGTACGCAAAGAGGAAAAGGA

ATGTATGGGAAACACCCTCTATATCGGTTCATTACAAAGTGAAGTTTATTTCTGTATCTA

TGAAAAGGACTACGAGCAGTACAAGAAAAATGATATTCCCATTGAAGACGCAGAAGTAAA

AAACCGTTTTGAGATTCGATTGAAAAATGAGCGTGCCTATTATGCAGTCCGTGATTTACT

CGTCTATGACAATCCAGAGCATACCGCCTTTAAAATTATCAATCGGTATATCCGTTTTGT

AGATAAAGACGATTCCAAACCTCGTTCTGATTGGAAACTGAATGAAGAATGGGCTTGGTT

TATTGGGAACAATCGTGAACGATTAAAACTAACCACAAAACCAGAGCCTTACTCCTTCCA

AAGGACGCTGAACTGGCTATCTCATCAAGTTGCCCCGACCTTAAAGGTTGCGATTAAACT

TGATGAAATCAACCAGACGCAGGTTGTAAAAGACATTCTCGACCATGCGAAACTGACAGA

CCGACACAAGCAGATTTTGAAGCAACAGTCAGTAAAAGAACAGGACGTGATAACAACAAA

AAATAACTCAAATACAAATTCATTGAATATAGAGAGGAGAACATTTTTATGAATTTTGGA

CAAAACCTTTATAACTGGTTTCTATCAAACGCTCAATCACTGGTGCTTTTAGCAATCGTT

GTGATTGGCTTGTATCTTGGCTTCAAGCGTGAGTTTAGCAAACTGATTGGCTTTTTAATT

ATTGCGATTATTGCGGTTGGCTTAGTCTTCAACGCTGCTGGAGTAAAAGACATTTTACTA

GAGCTATTCAATCGCATTATTGGTGCTTAAATAAAACCGTTCTTTTGTGGAATATAAGTG

GTTTTCTTATGTTCCGCAAAGGAATGGTACACCAAACGAAGTGCGGTAGGGATTTTTGAA

TCTCTACAAAGAAAGGACGTGAATATATGGACGATATGCAAGTCTATATTGCGAATTTAG

GCAAATACAATGAGGGCGAATTGGTCGGTGCGTGGTTTACCTTTCCCATTGACTTTGAGG

AAGTCAAAGAGAAAATCGGCTTGAATGATGAATATGAGGAATACGCCATTCATGACTACG

AGTTACCCTTTACGGTTGACGAATACACTTCCATTGGCGAACTCAATCGACTATGGGAAA

TGGTATCGGAATTACCCGAAGAATTACAATCGGAGCTATCTGCTCTGCTCACTCATTTTT

CAAGCATTGAAGAACTAAGCGAACATCAAGAGGATATTATCATTCATTCCGATTGTGATG

ATATGTATGACGTGGCACGCTACTACATTGAAGAAACGGGTGCTTTAGGCGAAGTACCAG

CTAGTCTTCAAAACTATATTGATTATCAAGCCTATGGTCGGGATTTAGACCTTTCAGGAA

CGTTTATCTCAACCAATCATGGGATTTTTGAAATCGTCTATTAAATCTGTCGGTACATTA

CTACTGGCAGATTTTCTATTTTACGGGGTGGCTCAATCAGCTACCCCTATTTTTTATGAA

AGGATTGATTACATGAAGAAAATACGAAGCTATACCAGTATCTGGTCTGTGGAAAAGGTA

CTGTATTCTATCAATGATTTTAGACTTCCGTTTCCCATAACCTTTACGCAAATGACATGG

TTTGTCGTGTCACTCTTTGCAGTGATGATACTTGGCAACTTGCCCCCTCTTTCCATGATA

GAGGGAGCATTTCTCAAATACTTTGGGATTCCTGTGGCTTTCACATGGTTTATGTCTACA

AAAACTTTTGATGGTAAAAAGCCTTATGGATTTTTGAAGTCTGTCATTGCTTATGCACTG

CGACCAAAGCTGACCTATGCAGGAAAAAAGTAACGCTTGGCAGAAACCAGCCACAAGAAG

CCATTACAGCAGTTAGGAGTGAATTTTATGGCATATCCAATTAAATACATTGAAAACAAT

CTCGTCTGGAATAAAGACGGGGAATGTTATGCTTACTATGAGCTTGTTCCTTACAATTAC

TCATTTCTAAGTCCAGAACAGAAAATACAAGTGCATGATTCTTTCAGACAGCTTATCGCA

CAAAATCGTGATGGCAAAATTCATGCTTTACAAATCAGTACAGAATCCAGCATACGTTCT

GCACAAGAGCGTTCCAAAAATGAAGTCACTGGCAAGCTCAAAGCGGTTGCCTATGACAAA

ATCGACCAACAGACAGACGCTTTAATATCCATGATTGGCGAAAATCAAGTGAACTACCGT

TTCTTTATCGGCTTTAAGTTGCTTCTCAACGATCAGGAGTTTTCTATGAAAAGTCTTACC

GTTGAAGCAAAAAATGCTTTGTCTGATTTTGTCTATGATGTGAACCATAAGCTGATGGGC

GATTTTGTTAGTATGAGTAATGATGAAATCCTGCGTTTTCAGAAGATGGAAAAGCTCTTA

GAAAATAAAATCTCTCGTCGTTTCAAAATCCGCAGGTTAGATAAGGACGACTTCGGCTAT

CTGATTGAACACCTTTACGGACAGACAGGCACTGCCTATGAAGAGTATGAGTACCATCTA

TCAAAGAAAAAGCTGGATAATGAAACGCTGATTAAATACTATGACTTGATTAAGCCTACT

CGCTGTTTGGTGGAAGAAAAACAGCGATATTTGAAAATCCAGCAGGAAGATGAAACCGTC

TATGTAGCTTACTTTACCATTAACAGCATTGTCGGAGAACTGGACTTCCCGTCCTCTGAA

ATCTTCTACTACCAGCAACAGCAATTTACATTCCCGATTGATACGTCAATGAATGTGGAA

ATTGTAGCGAATCGTAAAGCCCTATCTACTGTCCGCAATAAAAAGAAAGAACTGAAAGAC

TTGGATAACCACGCTTGGCAAAGTGATAATGAAACCAGCTCCAATGTGGCGGAAGCTCTG

GAAAGTGTGAATGAGCTGGAAACCAATTTAGACCAAAGCAAGGAATCTATGTACAAGCTG

TCTTATGTGGTAAGGGTATCAGCAAATGATCTTGACGAACTCAAACGTCGTTGTAATGAA

GTGAAAGATTTTTATGACGATTTAAGCGTAAAACTGGTACGACCATTTGGGGATATGCTC

GGCTTACATGAAGAATTTTTACCTGCCAGCAAGCGTTATATGAATGATTATATTCAATAC

GTGACCTCTGATTTCCTCGCTGGTTTAGGTTTTGGTGCTACTCAAATGCTGGGGGAAAAT

GAGGGGATTTATGTTGGCTACAGCTTAGATACTGGACGCAATGTCTATCTGAAACCTGCT

CTTGCCAGTCAAGGGGTTAAGGGTTCAGTAACCAATGCGTTAGCGTCGGCTTTTGTTGGT

TCGCTGGGTGGTGGTAAATCCTTTGCGAATAACCTTATCGTCTATTATGCGGTGCTTTAT

GGGGCACAAGCAGTGATTGTAGACCCAAAAGCAGAACGTGGCAGATGGAAAGAAACCTTG

CCAGAGATTTCCCATGAAATCAATATCGTCACTCTGACTTCTGATGAGAAAAACAAAGGC

TTACTTGACCCTTATGTGATTATGAAAAATCCCAAAGATTCTGAATCACTGGCTATTGAT

ATTCTGACATTCCTTACGGGGATTTCCTCTCGTGATGGGGAACGCTTCCCAATCCTTAGA

AAAGCCATTCGTGCAGTAACCAATAGTGAAGTACGAGGGTTGATGAAAGTGATTGAGGAA

TTACGGGTTGAGAATACGCCACTAAGTACCAGTATAGCCGACCATATCGAAAGTTTTACA

GACTATGACTTTGCACATTTATTATTCAGTAATGGTTATGTGGAGCAGTCTATCAGCTTA

GAAAAACAACTGAACATTATACAGGTTGCGGACTTGGTACTTCCCGACAAGGAAACTTCC

TTTGAGGAATATACCACTATGGAGCTTTTATCCGTTGCTATGCTGATTGTCATTAGTACC

TTTGCTTTAGACTTTATCCATACAGACCGAAGCATTTTCAAGATTGTAGATTTAGACGAA

GCATGGAGCTTTTTACAGGTAGCACAAGGAAAAACACTATCTATGAAGCTGGTTCGGGCT

GGTCGTGCTATGAACGCTGGGGTATATTTCGTGACCCAAAATACAGACGACCTCTTAGAT

GAAAAACTGAAAAATAACCTCGGCTTAAAATTTGCATTTCGTTCCACTGACCTTAACGAG

ATTAAAAAGACCTTAGCCTTTTTTGGTGTAGACCCAGAGGACGAAAACAATCAGAAGCGA

TTGCGTGATTTGGAAAACGGGCAATGCCTTATCAGTGATTTATATGGTCGTGTCGGTGTG

ATACAGTTCCACCCTGTATTTGAAGAACTGCTCCATGCCTTTGATACCAGACCACCTGTG

CGAAAAGAGGTGTAAATGTGAAACCATCAATAGTAAACAGAATAAAATCAAACTGGACGC

TGAAACGTCTAGGTAAAGTGGCAATGACAGTGGCTTTCACACTTGTGATTGCCATTTTTC

TTTTAGCCATGCTGGGAACGGTGGTTCAAGCTGCGGGCTTGGTAGATGATACGGTCAATG

TGGCAAATGAATACAGCCGATACCCACTTGAAAACTATCAACTGGATTTTTATGTGGATA

ATAGCTGGGGCTGGCTTCCGTGGAACTGGTCGGACGGGATTGGAAAACAGGTCATGTATG

GACTATATGCCATTACCAATTTTATTTGGACAATCAGTTTGTATGTTTCCAATGCGACAG

GTTACTTAGTACAGGAAGCCTATTCCTTAGACTTCATTTCCGCTACAGCAGATTCCATTG

GTAAGAATATGCAGACCTTAGCTGGTGTGAGTGCAAACGGATTTTCAACAGAGGGTTTCT

ATGTTGGATTCCTCTTACTCTTGATTTTGGTTCTTGGGGTTTATGTTGCCTATACGGGAC

TGATAAAGAGAGAAACCACAAAGGCAATTCATGCCATTATGAATTTTGTGCTGGTGTTTA

TCCTATCGGCTTCCTTTATTGCCTACGCTCCCGACTACATTAAAAAATCAATGACTTTTC

ATCAGACATCAGTAATGCCAGTTTATCACTTGGCACGAAGATTGTCATGCCCCATTCCGA

TAGTCAAGGCAAGGACAGCGTGGACTTAATCAGAGATAGCCTGTTTTCCATACAGGTTCA

GCAACCGTGGCTACTGCTTCAATACAACAGTTCAGACATTGAAAGTATCGGTATTGACCG

TGTGGAAAGCCTGCTCTCCACCAGCCCAGATTCCAACAATGGCGAAGACAGAGAAAAAAT

TGTTGCGGAAGAAATTGAAGACAGAAGCAATACCAATCTAACCATTACAAAGACCATTAA

CCGTTTAGGTACAGTCTTCTTCCTATTTGTCTTCAATATTGGGATTTCCATATTTGTATT

CCTATTAACAGGAATCATGATTTTCTCGCAGGTACTTTTTATCATCTATGCTATGTTTCT

GCCTGTGAGCTTTATTTTAAGCATGATTCCATCATTTGATGGTATGTCAAAACGAGCCAT

AACAAAGCTCTTTAATACCATTTTGACACGAGCTGGAATCACATTGATTATTACGACAGC

ATTTAGTATTTCAACCATGCTCTATACCTTATCGGCTGGTTATCCGTTCTTTTTGATTGC

TTTTCTACAGATTGTGACCTTTGCAGGAATCTACTTCAAGCTGGGCGATTTAATGAGTAT

GTTTTCTCTACAGAGTAACGATTCTCAAAGTGTGGGAAGTCGTGTGATGAGAAAACCTCG

TATGCTTATGCACGCTCACATGCACCGTCTACAGCGGAAACTTGGACGTTCCATGACTAC

TCTAGGGGCTGGGTCTGCCATTGTTACAGGTAAAAAAGGACAGTCGGGTTCGGGGAGTTC

TGCAAGGACACAAGCAGATCACTCCCGACCAGACGGAAAGGAAAAATCAACACTTGGAAA

ACGTATCGGTCAAACCATCGGTACAGTAGCTGATACCAAAGACAGAATGGTAGACACTGC

TAGTGGTTTGAAAGAACAGGTTAAAGATTTGCCGACCAATGCAAGATATGCAGTATATCA

AGGAAAATCCAAAGTAAAAGAGAATGTCCGTGATTTAACCAGTAGTATTTCTCAAACCAA

AGCGGACAGAGCCAGTGGACGCAAGGAACAGCAGGAACAAAGGCGAAAAACCATTGCGAA

GCGTCGCTCTGAAATGGAACAGGTCAAACAGAAAAAACAGCCTGCTTCTTCTGTTCATGA

AAGACCGACTACAAGACAAGAACAATATCATGATGAACAGACCTCAAAACAGTCTAATAT

TCAGACTTCATATAAGGAATCTCAACAAGCCAAACAAGAGCGTCCAGCAGTTAAGTCCGA

TTTTTCAAGTCCAAAAGTGGAACGCCAAGGCAATACCGTTCAAGAAAAAACCGTTCAAAA

GCCAGCAACTTCAACCACTACAGCAGATAGAACTTCACAACGTCCAATCACAAAAGAACG

TCCGTCTACTGTTCAAAGAGTACCACTACAAAATACAAGAAGTAGACCACCAATCAAAAC

CGCCACCATTAAGAAAGTCGGTAAGAAACCATGAAGTTGAAAACTTTAGTGATTGGTGGT

TCTGGATTATTCTTGATGGTCTTCTCACTGCTTCTGTTTGTTGCCATTTTATTTTCAGAT

GAACAGGACAGCGGAATTTCCAATATTCATTATGGAGGTGTGAATGTTTCCGCAGAAGTG

CTGGCTCATAAGCCTATGGTAGAAAAATATGCCAAAGAATATGGCGTTGAAGAATATGTC

AACATACTTCTTGCGATTATACAGGTGGAATCGGGCGGTACTGCGGAAGATGTTATGCAG

TCCTCGGAATCCCTCGGTCTTCCACCTAATTCATTGAGTACAGAAGAATCCATTAAGCAA

GGTGTGAAGTATTTCAGTGAATTATTAGCCAGTAGCGAAAGGCTCAGTGTAGATTTAGAA

TCGGTTATCCAGTCCTACAATTATGGTGGTGGTTTCTTAGGGTATGTGGCTAATCGTGGA

AATAAATATACCTTTGAACTGGCTCAAAGTTTCTCAAAAGAGTATTCAGGTGGCGAAAAA

GTGTCTTACCCCAATCCCATAGCCATACCTATCAATGGGGGCTGGCGATACAACTATGGC

AATATGTTTTATGTGCAACTGGTAACGCAGTATCTTGTCACAACAGAGTTTGATGATGAT

ACGGTACAAGCCATCATGGACGAAGCACTGAAATATGAGGGCTGGCGATACGTTTACGGT

GGAGCTTCCCCGACTACTTCTTTTGATTGTAGCGGACTGACACAATGGACGTATGGAAAA

GCTGGAATTAACTTACCACGAACCGCACAACAGCAATATGATGTGACCCAGCATATCCCA

CTATCGGAAGCACAAGCTGGCGATTTGGTTTTCTTTCATTCTACCTATAACGCTGGCTCT

TATATTACTCATGTTGGGATATACCTTGGCAATAACCGTATGTTTCATGCAGGCGACCCA

ATCGGTTATGCCGACTTAACAAGCCCCTACTGGCAACAGCATTTAGTGGGAGCAGGACGA

ATCAAACAATGAGAAAGGAAGATTTAATGATGAAATTTAGAAAAAATCAGAATAAAGAAA

AACAGATACCAAAGGAAAAGAAACCTCGTGTCTACTATAAGGTCAATCCTCATAAAAAGG

TTGTGATTGCCTTGTGGGTACTTTTAGGGCTTAGTTTCAGCTTTGCGATATTCAAGCACT

TTACAGCTATAGATACTCATACTATTCACGAAACAACTATCATAGAAAAGGAATACGTTG

ATACTCATCATGTAGAAAATTTTGTAGAGAACTTTGCGAAAGTCTACTATTCATGGGAGC

AATCCGATAAGTCCATTGATAATCGAATGGAAAGTCTAAAAGGCTATCTGACAGATGAAC

TTCAAGCTCTCAATGTTGATACAGTACGCAAAGATATTCCTGTATCGTCTTCTGTAAGAG

GATTTCAGATATGGACGGTAGAGCCAACTGGCGACAATGAGTTTAATGTAACCTACAGTG

TAGACCAGCTCATTACAGAGGGAGAAAATACAAAGACCGTCCACTCTGCTTATATAGTGA

GTGTCTATGTAGATGGTTCTGGAAATATGGTACTGGTTAAGAATCCGACCATTACCAACA

TACCTAAGAAATCAAGTTATAAACCAAAAGCCATTGAAAGTGAGGGGACGGTTGATTCCA

TTACAACCAATGAAATCAATGAGTTTTTAACGACGTTCTTCAAGCTCTATCCTACAGCGA

CAGCCAGTGAACTTTCCTACTATGTGAATGACGGGATATTAAAACCAATCGGAAAAGAGT

ACATCTTTCAAGAACTGGTAAATCCTATTCACAATCGTAAGGATAATCAAGTCACGGTAT

CGCTGACAGTGGAGTATATCGACCAGCAGACCAAAGCAACGCAGGTATCTCAATTTGATT

TGGTACTTGAAAAGAACGGGAGTAATTGGAAGATTATAGAATAACAAATATTGGTACATT

ATTACAGCTATTTTGTAATCACGTACTCTCTTTGATAAAAAATTGGAGATTCCTTTACAA

ATATGCTCTTACGTGCTATTATTTAAGTATCTATTTAAAAGGAGTTAATAAATATGCGGC

AAGGTATTCTTAAATAAACTGTCAATTTGATAGTGGGAACAAATAATTGGATGCCCTTTT

GGGCTTTTGAATGGAGGAAAATCACATGAAAATTATTAATATTGGAGTTTTAGCTCATGT

TGATGCGGGAAAAACTACCTTAACAGAAAGCTTATTATATAACAGTGGAGCGATTACAGA

ATTAGGAAGCGTGGACAGAGGTACAACGAAAACGGATAATACGCTTTTAGAACGTCAGAG

AGGAATTACAATTCAGACGGCGATAACCTCTTTTCAGTGGAAAAATACTAAGATGAACAT

CATAGACACGCCAGGACATATGGATTTTTTAGCAGAAGTATATCGTTCATTATCAGTATT

AGATGGGGCAATTCTACTGATTTCTGCAAAAGATGGCGTACAAGCACAAACTCGTATATT

GTTTCATGCACTTAGGAAAATAGGTATTCCCACAATCTTTTTTATCAATAAGATTGACCA

AAATGGAATTGATTTATCAACGGTTTATCAGGATATTAAAGAGAAACTTTCTGCGGAAAT

TGTAATCAAACAGAAGGTAGAACTGCATCCTAATATGCGTGTAATGAACTTTACCGAATC

TGAACAATGGGATATGGTAATAGAAGGAAATGATTACCTTTTGGAGAAATATACGTCTGG

GAAATTATTGGAAGCATTAGAACTCGAACAAGAGGAAAGCATAAGATTTCATAATTGTTC

CCTGTTCCCTGTTTATCACGGAAGTGCAAAAAACAATATAGGGATTGATAACCTTATAGA

AGTGATTACGAATAAATTTTATTCATCAACACATCGAGGTCAGTCTGAACTTTGCGGAAA

AGTTTTCAAAATTGAGTATTCGGAAAAAAGACAGCGTCTTGCATATATACGTCTTTATAG

TGGCGTACTGCATTTGCGAGATTCGGTTAGAATATCGGAAAAGGAAAAATAAAAATTACA

GAAATGTATACTTCAATAAATGGTGAATTATGTAAAATCGATAAGGCTTATTCCGGGGAA

ATTGTTATTTTGCAGAATGAGTTTTTGAAGTTAAATAGTGTTCTTGGAGATACAAAGCTA

TTGCCACAGAGAGAGAATTGAAAATCCCCTCCCTCTGCTGCAAACGACTGTTGAACCGAG

CAAACCTCAACAAAGGGAAATGTTACTTGATGCACTTTTAGAAATCTCCGACAGTGACCC

GCTTCTGCGATATTATGTGGATTCTGCGACACATGAAATCATACTTTCTTTCTTAGGGAA

AGTACAAATGGAAGTGACTTGTGCTCTGCTGCAAGAAAAGTATCATGTGGAGATAGAAAT

AAAAGAGCCTACAGTCATTTATATGGAAAGACCGTTAAAAAAAGCAGAGTATACCATTCA

CATCGAAGTTCCACCGAATCCTTTCTGGGCTTCCATTGGTCTATCTGTAGCACAGCTTCC

ATTAGGGAGCGGAGTACAGTATGAGAGCTCGGTTTCTCTTGGATACTTAAATCAATCGTT

TCAAAATGCAGTTATGGAGGGGATACGCTATGGCTGTGAACAAGGATTGTATGGTTGGAA

TGTGACGGACTGTAAAATCTGTTTTAAGTATGGCTTATACTATAGCCCTGTTAGTACCCC

AGCAGATTTTCGGATGCTTGCTCCTATTGTATTGGAACAAGTCTTAAAAAAAGCTGGAAC

AGAATTGTTAGAGCCATATCTTAGTTTTAAAATTTATGCGCCACAGGAATATCTTTCACG

AGCATACAACGATGCTCCTAAATATTGTGCGAACATCGTAGACACTCAATTGAAAAATAA

TGAGGTCATTCTTAGTGGAGAAATCCCTGCTCGGTGTATTCAAGAATATCGTAGTGATTT

AACTTTCTTTACAAATGGACGTAGTGTTTGTTTAACAGAGTTAAAAGGGTACCATGTTAC

TACCGGTGAACCTGTTTGCCAGCCCCGTCGTCCAAATAGTCGGATAGATAAAGTACGATA

TATGTTCAATAAAATAACTTAGTGTATTTTATGTTGTTATATAAATATGGTTTCTTGTTA

AATAAGATGAAATATTTTTTAATAAAGATTTGAATTAAAGTGTAAAGGAGGAGATAGTTA

TTATAAACTACAAGTGGATATTGTGTCCTGTATGTGGAAATAAAACACGATTAAAGATAA

GGGAAGATACTGAATTAAAAAAATTCCCCCTCTATTGTCCGAAATGCAGACAAGAAAATT

TAATTGAAATAAAGCAGTTCAAAGTAACTGTGATTACAGAGCCAGACGCAAAGACGCAGA

GCCGATAAAATGAGATTAATACAATCTCATTTTATCGGCTCTTTCCGTTATGTATGGATT

CTTTTAATTAGTCTTCGATGTTTCTTGCTTCGTTGATACCGCTGGCTAAAGATTCCATTA

AGGATAGTTCTTTGTCTGTAAAGCTATCCATGTATTTCTCTATCTGTAATCGTCGGGTGC

TTTTTACCAAGTTATTAGCAGGTAAGAAAAATTCATCAACGGAAACATGAAGTAACGATA

CAAGGTCATAAAGAACTTGTATGCTGGGGTGTTGCCCTTTATTTTCAATATTAGTTAAGT

ACCGTGGGTCAATTTCAATCAATGCTCCCACTTGTTCACGAGTTAAACCTCGTTTCAATC

GAGCTTCTTTAATGGCTAAACCAAAGGCTCTAAAATCATATTTATCTTCTTTTTTACGCA

TAGTAGACCACCTCTATACATTTTATTGTTCCTACTGAATTAAAAACAGGTATAGAAAAA

CGTGTTATATGGTTTATAGGTTTATATTTAATAAAAAGCACTACTAAACGCCAATAAAAA

AACCGTTATATGGTAGTGCTATTTACGCTGTTAAAATATTGTATATTACTTCCAAATGGC

GGTTTGTTGGAGGTCAACGTCGCCATGAAGTACATCATATACAATAAATTTCCTTACATT

GGGTTCTTGTCAAAAAAAGTCGTCTATCTGCAATAGATAAGTACGTCCACCAATGTGGTT

TTATAAATCATATAGATAGAATAACAGAAGCATGTAAACAGAGAAATAAATCTGTTTATA

TGCTTTTTTGGCTATTCAGAACTTTTTTACAAAGTTTATTTATCAGTAATGCAACAAATC

CCCCTTTCACATTGGGACTAAGAGTGAAAGGAGATAAACGAGCAAGGCTCACTTCCTTTC

CTAGACAGAAAGGGGGTGAGAAACATGAAACCATCTTCTTTTCAGACCACAATAGAAAAT

CAGTTTGACTATATCTGTAAACGTGCTATGGAAGACGAGCGAAAGAATTATATGCTTTAT

CTTTCAAGGATTGCAAAGCGTGAGGTGTCCTTTTCGGATGTTGGCGATTATCTTGTTAGC

CAGTTTGCGACAACAGATAACTATTCAACTGACTTTCAGATTTTTACACTCAATGGGTTA

TCAGTAGGCGTTGAAAATGATTTGTTGAGTGAAGCATTACGTGAGTTGCCAGACAAGAAA

CGTGAAATTCTACTGCTGTTTTACTTTATGGACATGAGCGATTCAGAAATTGCAGACCTG

TTGAAATTGAACCGTTCTACTGTCTATCGGCATAGAACCAGTGGACTAGCCTTAATTAAA

AAGTTTATGGAGGAATTTGAAGAATGAAAACACAATATCCTATGATTCCCTTTCCTCTCA

TTGTAAAGGCAACAGATGGCGATACCGAAGCGATTAACCAGATTCTACATCATTACAGAG

GGTACATAACGAAGCGTTCCCTACGACTTATGAAAGATGAATATGGCAATCAAAGTATGG

TCGTTGATGAAGTCTTACGTGGAAGAATGGAAACCAGACTGATTACAAAGATTTTGTCAT

TTGAAATTAAGTAATATCCTCTCTCCTTTCGTGGAAGCGTGCTAAACCATTCCACGCTTC

CCGAACAGGGAGGTTTGTTATTCCACCAAAGCATATTGAGCTTTCAATGTGTTTTGATAG

GCTAACGAGCCATTGTTCTTTGAAAACTGAATAAAAGTAATCGAATACGTTTCGATAAGA

AAAGAGCCAACGGAACTAACCGCCATGACCTATCTTATAAAGATAGCGAGCGATTCATGT

TAGTGATCCGAGAAGCAATCTTTAGCAGGATTGCCTGCAACGACATTCTTATCGTGATAA

TGATACTCCCATACAGTCAATAGTCCGAGCGTGATAAAACCGTCGCAGGCAATGAGTATG

GCTACATGAGAACCATGCAGGGGTGGAACTCCCGTGAGCTTTGCTAAAGCTGTTCGATTG

CTGGTAAAACAACTTTTATGAAATCCAAATAAGTGATTTGGAAAGGAGGATTTTATGAAG

CAGACTGACATTCCTATTTGGGAACGTTATACCCTAACCATTGAAGAAGCGTCAAAATAT

TTTCGTATTGGCGAAAACAAGCTACGACGCTTGGCAGAGGAAAATAAAAATGCAAATTGG

CTGATTATGAATGGCAATCGTATTCAGATTAAACGAAAACAATTTGAAAAAATTATAGAT

ACATTGGACGCAATCTAGCGTCGCCAAAGGGTCTTGTATATGATAAAATAGTATTAAGTC

GTATCAAGGCTCTTTCCATAAAGGAAAGGAGCAAATGCCATGTCAGAAAAAAGACGTGAC

AATAAAGGTCGAATCTTAAAGACTGGAGAGAGCCAACGAAAAGACGGAAGATACTTATAC

AAATATATAGATTCATTTGGAGAACCGCAATTTGTTTACTCGTGGAAACTTGTGGCTACA

GACCGAGTACCAGCAGGAAAGCGTGATTGTATCTCACTTAGAGAGAAAATCGCAGAGTTA

CAGAAAGACATTCATGATGGTATTGATGTTGTAGGAAAGAAAATGACACTCTGCCAGCTT

TACGCAAAACAGAACGCTCAAAGACCAAAGGTTAGAAAAAACACTGAAACTGGACGCAAA

TATCTTATGGATATTTTGAAGAAAGACAAGTTAGGTGTAAGAAGTATTGACAGTATTAAG

CCATCAGACGCTAAAGAATGGGCTATTAGAATGAGTGAAAATGGTTATGCTTATCAAACC

ATCAATAACTACAAACGTTCTTTAAAGGCTTCATTCTATATTGCTATACAAGATGATTGT

GTTCGGAAGAATCCATTTGACTTTCAACTGAAAGCAGTTCTTGATGATGATACTGTCCCT

AAGACCGTACTAACAGAAGAACAGGAAGAAAAACTGTTAGCCTTTGCAAAAGCTGATAAA

ACCTACAGCAAAAATTATGATGAAATTCTGATACTCTTAAAAACAGGTCTTCGTATTTCA

GAGTTTGGTGGTTTGACACTTCCAGATTTAGATTTTGAGAATCGTCTTGTCAATATAGAC

CATCAGCTATTGAGAGATACTGAAATTGGGTACTACATTGAAACACCAAAGACCAAAAGT

GGCGAACGTCAAGTTCCTATGGTTGAAGAAGCCTATCAAGCATTTAAGCGAGTGTTAGCG

AATCGAAAGAATGATAAGCGTGTTGAGATTGATGGATATAGTGATTTCCTCTTTCTTAAT

AGAAAGAACTATCCAAAAGTGGCAAGTGATTACAACGGCATGATGAAAGGTCTTGTTAAG

AAATACAATAAGTATAACGAGGATAAATTGCCACACATCACTCCACATAGTTTGCGACAT

ACATTCTGTACCAACTATGCAAATGCAGGAATGAATCCAAAGGCATTACAGTACATTATG

GGACATGCTAATATAGCCATGACGCTGAACTATTACGCACATGCAACATTCGATTCTGCA

ATGGCAGAAATGAAACGCTTGAATAAAGAGAAGCAACAGGAGCGTCTTGTTGCTTAGTAG

TACAAATGAATTTACTACTTATTTACCACTTCTGACAGCTAAGACATGAGGAAATATGCA

AAGAAACGTGAAGTATCTTCCTACAGTAAAAATACTCGAAAGCACATAGAATAAGGCTTT

ACGAGCATTTAAGAAAATATAAAAAGATAATTAGAAATTTATACTTTGTTT

>GA60132/Tn6002

AAAATAGCATAAAAATCTAGTTATCCGCATAAAAACTGGACTTATCACACTTTATCAAGG

TCAAAACCACTCAATTTACTACTAATTTACTACTTATGAATGAGCTTTGATACGACGATT

TATCCTTGAAAAGTGAAGATATAAAGATACTTCCAATAAAATTTGAATATTTAATAGGTA

GACACTTCAAAAAATGAGGTGTCTATTTTTTTACCCGATTTTGAAAGGAAGTGAACTTAT

GAAAACAAAAAATCAAGAATCAAAAGGTCGTTCCCCACTCTTTAAGACCATCAAACATTC

ATTCAGCCAATAAAAAAGAAAGGATAGGTAAAAATATGGAACTTAAATTTGTGATTCCCA

ACATGGAAAAAACATTCGGCAATTTAGAATTTGCTGGCGAGGATAAAGTCGTTCAGCGAA

GAATCAACGGACGGCTAACTGTCTTATCAAGAAGCTATAATCTCTATTCTGATGTTCAAA

GAGCAGATGATATTGTGGTGGTGCTTCCTGCTGAAGCTGGCGAAAAACATTTCGGCTTTG

AGGAACGTGTGAAGTTAGTCAATCCACGTATTACCGCAGAGGGCTACAAAATCGGCACTC

GTGGTTTTACAAATTACCTTTTACATGCTGACGACATGATAAAAGAATAAAGAAAGAGAG

GAAAAATGATGAGATTAGCAAATGGCATTGTATTAGATAAAGACACGACTTTTGGAGAAT

TGAAATTCTCTGCTCTACGTCGTGAAGTGAGAATCCAAAATGAAGACGGGTCGGTTTCAG

ATGAAATCAAGGAACGTACCTATGACTTAAAATCCAAAGGACAAGGACGCATGATTCAAG

TAAGTATTCCTGCCAGCGTGCCTTTGAAAGAGTTTGATTATAACGCACGGGTGGAACTTA

TCAATCCCATTGCGGACACCGTTGCTACTGCCACCTATCAAGGAGCAGATGTTGACTGGT

ATATCAAGGCAGACGATATTGTGCTGACAAAGGATTCTAGTTCATTCAAAGCTCAACCAC

AAGCAAAGAAAGAACCGACACAAGACAAATAGTCGCTAGGTAGAAAGGAGACTTTTTCGC

ATGAAACAGCGTGGTAAAAGGATTCGCCCATCTGGTAAAGATTTAGTCTTTCATTTTACG

ATAGCGTCACTCCTGCCTGTTTTCCTGCTGGTTGTCGGACTGTTTCATGTGAAGACAATC

CAGCAGATCAACTGGCAGGATTTTAACCTATCACAAGCAGATAAGATTGACATTCCCTAT

TTAATTATCAGTTTCAGTGTCGCAATTCTTATCTGCTTGCTGGTAGCGTTTGTATTCAAA

CGGGTTCGCTATGATACGGTTAAACAACTTTACCACCGTCAAAAACTGGCAAAGATGATA

CTTGAAAACAAGTGGTATGAATCTGAACAGGTCAAAACAGAGGGTTTCTTTAAAGATAGT

GCTGGTCGTACAAAGGAAAAGATAACCTACTTCCCTAAAATGTATTATCGACTTAAAAAT

GGCTTGATACAGATACGGGTGGAAATCACGCTGGGAAAATATCAAGACCAACTCTTACAC

TTGGAAAAGAAATTAGAGAGTGGCTTGTACTGTGAGCTGACGGATAAAGAGTTAAAGGAT

TCCTATGTGGAATATACTTTGCTCTATGACACCATAGCCAGTCGTATTTCTATTGATGAA

GTAGAAGCTAAAGATGGTAAACTTCGCTTAATGAAAAACGTATGGTGGGAATATGATAAG

CTCCCTCATATGTTGATTGCTGGTGGTACAGGTGGCGGTAAAACTTACTTTATACTGACA

CTGATTGAAGCCTTGCTTCATACAGATTCAAAACTGTATATTCTTGACCCGAAAAATGCT

GACCTTGCGGACTTAGGTTCTGTGATGGCAAATGTCTACTATAGAAAAGAAGACTTGCTT

TCTTGCATTGAAACATTCTATGAAGAAATGATGAAACGTAGTGAGGAAATGAAGCAGATG

AAGAACTATAAGACTGGCAAAAATTATGCTTACTTAGGTCTCCCGGCACACTTCTTAATC

TTTGATGAATACGTCGCTTTCATGGAAATGCTGGGAACAAAAGAAAACACCGCAGTTATG

AATAAGCTGAAACAGATTGTCATGTTAGGTCGTCAAGCTGGCTTCTTTCTAATACTGGCT

TGTCAACGTCCAGACGCAAAATATTTAGGCGACGGAATCCGTGATCAGTTTAATTTCAGA

GTGGCTTTAGGTCGTATGTCTGAAATGGGCTATGGCATGATGTTTGGCAGTGACGTACAA

AAGGATTTCTTCTTAAAGCGAATCAAAGGTCGTGGCTATGTTGATGTAGGAACAAGTGTC

ATATCAGAGTTTTATACTCCCCTTGTACCAAAAGGATATGATTTCTTGGAGGAAATTAAA

AAGTTATCCAACAGCAGACAGTCCACGCAGGCGACGTGCGAAGCGGAAGTCGCAGGTGTG

GACTGATCTTGCTGGCTGGTGTGGCAATAGCCACGCCAGCACTTACCCCCCCGTATCTAA

CAGGGGGGTACAAATCGACAGGAAACAGTCAAAAAAACATTAGAAAATCCTTTGGTTACA

AGGGATTTACAAAATTTCAGCGTATGTCAAATGGGCTTTAAAAGTTGACATACGCCTTTT

TGATTGGAGGGATTTTTACTGAATGAACAAACTTGGTTACAGCATTTAAAAGAAAAACGC

TTGGCTTATGGACTATCTCAAAACCGTTTAGCTGTTGCGACTGGTATTACAAGGCAGTAT

CTAAGCGATATTGAAACAGGAAAAGTCAAGCCATCAGAGGATTTACAGCAGTCCCTTTGG

GAAGCTCTGGAACGCTTCAATCCCGACGCTCCCCTTGAAATGCTGTTTGATTATGTAAGA

ATTCGCTTTCCGACAACAGACGTACAGCAGGTGGTCGAAAACATCTTACAACTGAAACTG

TCCTATTTTCTTCATGAGGACTATGGTTTCTATTCTTATTCAGAGCATTATGCTTTAGGC

GACATATTCGTCCTTTGCTCCCATGAACTGGACAAAGGAGTTCTGGTGGAATTGAAAGGT

CGTGGGTGCAGACAATTTGAAAGCTATCTTCTGGCACAACAAAGAAGCTGGTATGAGTTC

TTTATGGACGTTTTGGTGGCTGGCGGTGTGATGAAACGCCTTGACCTTGCCATTAACGAT

AAGACAGGGATTTTAAATATCCCTGTACTCACTGAAAAGTGCCAACAGGAAGAATGTATC

TCCGTCTTCCGCAGTTTTAAAAGCTATCGCAGTGGCGAACTGGTACGCAAAGAGGAAAAG

GAATGTATGGGAAACACCCTCTATATCGGTTCATTACAAAGTGAAGTTTATTTCTGTATC

TATGAAAAGGACTACGAGCAGTACAAGAAAAATGATATTCCCATTGAAGACGCAGAAGTA

AAAAACCGTTTTGAGATTCGATTGAAAAATGAGCGTGCCTATTATGCAGTCCGTGATTTA

CTCGTCTATGACAATCCAGAGCATACCGCCTTTAAAATTATCAATCGGTATATCCGTTTT

GTAGATAAAGACGATTCCAAACCTCGTTCTGATTGGAAACTGAATGAAGAATGGGCTTGG

TTTATTGGGAACAATCGTGAACGATTAAAACTAACCACAAAACCAGAGCCTTACTCCTTC

CAAAGGACGCTGAACTGGCTATCTCATCAAGTTGCCCCGACCTTAAAGGTTGCGATTAAA

CTTGATGAAATCAACCAGACGCAGGTTGTAAAAGACATTCTCGACCATGCGAAACTGACA

GACCGACACAAGCAGATTTTGAAGCAACAGTCAGTAAAAGAACAGGACGTGATAACAACA

AAAAAAGGATATCTGTCAACCATACCAGTTGACAGATATCCAAAAAAAGATATAATGGGA

GATAAGACGGTTCGTGTTCGTGCTGACTTGCACCATATCATAAAAATCGAAACAGCAAAG

AATGGCGGAAACGTAAAAGAAGTTATGGAAATAAGACTTAGAAGCAAACTTAAGAGTGTG

TTGATAGTGCATTATCTTAAAATTTTGTATAATAGGAATTGAAGTTAAATTAGATGCTAA

AAATTTGTAATTAAGAAGGAGGGATTCGTCATGTTGGTATTCCAAATGCGTAATGTAGAT

AAAACATCTACTGTTTTGAAACAGACTAAAAACAGTGATTACGCAGATAAATAAATACGT

TAGATTAATTCCTACCAGTGACTAATCTTATGACTTTTTAAACAGATAACTAAAATTACA

AACAAATCGTTTAACTTCTGTATTTGTTTATAGATGTAATCACTTCAGGAGAGATTACAT

GAACAAAAATATAAAATATTCTCAAAACTTTTTAACGAGTGAAAAAGTACTCAACCAAAT

AATAAAACAATTGAATTTAAAAGAAACCGATACCGTTTACGAAATTGGAACAGGTAAAGG

GCATTTAACGACGAAACTGGCTAAAATAAGTAAACAGGTAACGTCTATTGAATTAGACAG

TCATCTATTCAACTTATCGTCAGAAAAATTAAAACTGAATACTCGTGTCACTTTAATTCA

CCAAGATATTCTACAGTTTCAATTCCCTAACAAACAGAGGTATAAAATTGTTGGGAATAT

TCCTTACCATTTAAGCACACAAATTATTAAAAAAGTGGTTTTTGAAAGCCGTGCGTCTGA

CATCTATCTGATTGTTGAAGAAGGATTCTACAAGCGTACCTTGGATATTCACCGAACACT

AGGGTTGCTCTTGCACACTCAAGTCTCGATTCAGCAATTGCTTAAGCTGCCAGCGGAATG

CTTTCATCCTAAACCAAAAGTAAACAGTGTCTTAATAAAACTTACCCGCCATACCACAGA

TGTTCCAGATAAATATTGGAAGCTATATACGTACTTTGTTTCAAAATGGGTCAATCGAGA

ATATCGTCAACTGTTTACTAAAAATCAGTTTCATCAAGCAATGAAACACGCCAAAGTAAA

CAATTTAAGTACCATTACTTATGAGCAAGTATTGTCTATTTTTAATAGTTATCTATTATT

TAACGGGAGGAAATAATTCTATGAGTCGCTTTTTTAAATTTGGAAAGTTACACGTTACTA

AAGGGAATGGAGATAAATTATTAGATATACTACTGACAGCTTCCAAGAAGCTAAAGAGGT

CCCTAGCGCCTACGGGGAATTTGTATCGATAAGGGGTACAAATTCCCACTAAGCGCTCGG

GACCCCTTGTAGGAAAATGTCCTAAGTGGGATATCTGTCAACTGGTATGGTTGACTAAAA

ATACTTCCTACGAAAATGTAGGGGGTATTTTTTTACGAAAAAATACAATCGATTCTTAAA

AAGAAAAATTTTTGATTGGCAAAACCATAACAAGTTCGTTTTAGGGTTTTGATTTTGCGA

TTGATGCCTTCTAAAGGACCATTAGAGTATTCAAATTTAGCGCTATTTAAGACATATTTT

CTGTTTTGACGAAGGGTTTGAATAGCAGTATCCATTTCTGTATTGGTTTTTTGGTAGTCT

AAGATGGTTGACTCTAGTAATTCACTATTGCGCTCGTTTAGGGCTTTCGTGATATCTTGG

TAAGTTTGGTATACTTCAGCGAACTTGGAAAATTTACTAGTAATGAGATCAACAGCATTT

TGGCGAGTCATATATTGTTTAACGCCGCGAAGAAAAACTACTTCTTCAGGGTGAAGATCT

TCAGCTTTTTTATGGAATAGCTTCCAATGTGACTTCATAATTTTATATTCTTGGCTCTGT

TTATCAAGTTGCTTTAGGATAGAGATACGACAATTGTCCAAAGCGCGACCAGCTAATTGT

ACAAGGTGGAAGCGATCAATAATGATATTGGCATTAGGGAAAAGGCGATAGATAAAACTT

TGATATTGAGCATTTAAATCAATTACAACTGATTGAACGCATTCGCGTTCGGCTTTTGAA

TAACGACTTTCAAAATAATCAACAATGGTAGGTGATAGACGATCCTGTAACTTTGTGACA

ATTTGGTGGGTTTCAGCGTCACAACAGATAAAGGACATCACAGACTTAATTGAACGAAAC

TCGTCAAAACATAGATGCTTAGGCAACTTAGCCACACGATAGTGTGGTTCCATGCGCTCT

AAGATTGTTCGACGAACACTGCTAGGAGAGCAGTGACACATTTCAGCAATAAGCTGACCA

GATAAGCCTTTACGAGCTAAAAGCATGATTTGATTTTTGAGATCACTGGATAAGGTTTGA

TTTTCTTTGGTTAAATTAGTAATAGCACCAAAAGTAGTATGGCATGATTTACATTTATAG

CGTTGTTTACGAAGCTCTAGTTCATATCTTCTCCCATTTAAACTTGCCAGTCGTACATGA

GTTTTGCGAAAGCCATCCTTATTAACTGTGGGAAAGCCACAGTTACGACAACGATTAATC

GGATAAGAAAGAGTAGCTGTTATTAGCGTTATATACTCTTTAACAGAATCGTTGTTGTGT

TCAGCTTCTTCAACAGAAATAATTTTAATATTTTTATCTTTAATTCCAAGAATATTTAGG

ATAGAATCATTATGGGACATTTGTTTAACCTTCTTTCATGATTTTTGTGGTGAATTGATT

GTATAACGAGGGGACAGCAAATGTCCTCTTTTTTGTATAAAAAAATCTGGCATGGAATCT

CTATCCATACCAGAAAGTGTATACCCCAAAAAAATAACTCAAATACAAATTCATTGAATA

TAGAGAGGAGAACATTTTTATGAATTTTGGACAAAACCTTTATAACTGGTTTCTATCAAA

CGCTCAATCACTGGTGCTTTTAGCAATCGTTGTGATTGGCTTGTATCTTGGCTTCAAGCG

TGAGTTTAGCAAACTGATTGGCTTTTTAATTATTGCGATTATTGCGGTTGGCTTAGTCTT

CAACGCTGCTGGAGTAAAAGACATTTTACTAGAGCTATTCAATCGCATTATTGGTGCTTA

AATAAAACCGTTCTTTTGTGGAATATAAGTGGTTTTCTTATGTTCCGCAAAGGAATGGTA

CACCAAACGAAGTGCGGTAGGGATTTTTGAATCTCTACAAAGAAAGGACGTGAATATATG

GACGATATGCAAGTCTATATTGCGAATTTAGGCAAATACAATGAGGGCGAATTGGTCGGT

GCGTGGTTTACCTTTCCCATTGACTTTGAGGAAGTCAAAGAGAAAATCGGCTTGAATGAT

GAATATGAGGAATACGCCATTCATGACTACGAGTTACCCTTTACGGTTGACGAATACACT

TCCATTGGCGAACTCAATCGACTATGGGAAATGGTATCGGAATTACCCGAAGAATTACAA

TCGGAGCTATCTGCTCTGCTCACTCATTTTTCAAGCATTGAAGAACTAAGCGAACATCAA

GAGGATATTATCATTCATTCCGATTGTGATGATATGTATGACGTGGCACGCTACTACATT

GAAGAAACGGGTGCTTTAGGCGAAGTACCAGCTAGTCTTCAAAACTATATTGATTATCAA

GCCTATGGTCGGGATTTAGACCTTTCAGGAACGTTTATCTCAACCAATCATGGGATTTTT

GAAATCGTCTATTAAATCTGTCGGTACATTACTACTGGCAGATTTTCTATTTTACGGGGT

GGCTCAATCAGCTACCCCTATTTTTTATGAAAGGATTGATTACATGAAGAAAATACGAAG

CTATACCAGTATCTGGTCTGTGGAAAAGGTACTGTATTCTATCAATGATTTTAGACTTCC

GTTTCCCATAACCTTTACGCAAATGACATGGTTTGTCGTGTCACTCTTTGCAGTGATGAT

ACTTGGCAACTTGCCCCCTCTTTCCATGATAGAGGGAGCATTTCTCAAATACTTTGGGAT

TCCTGTGGCTTTCACATGGTTTATGTCTACAAAAACTTTTGATGGTAAAAAGCCTTATGG

ATTTTTGAAGTCTGTCATTGCTTATGCACTGCGACCAAAGCTGACCTATGCAGGAAAAAA

AGTAACGCTTGGCAGAAACCAGCCACAAGAAGCCATTACAGCAGTTAGGAGTGAATTTTA

TGGCATATCCAATTAAATACATTGAAAACAATCTCGTCTGGAATAAAGACGGGGAATGTT

ATGCTTACTATGAGCTTGTTCCTTACAATTACTCATTTCTAAGTCCAGAACAGAAAATAC

AAGTGCATGATTCTTTCAGACAGCTTATCGCACAAAATCGTGATGGCAAAATTCATGCTT

TACAAATCAGTACAGAATCCAGCATACGTTCTGCACAAGAGCGTTCCAAAAATGAAGTCA

CTGGCAAGCTCAAAGCGGTTGCCTATGACAAAATCGACCAACAGACAGACGCTTTAATAT

CCATGATTGGCGAAAATCAAGTGAACTACCGTTTCTTTATCGGCTTTAAGTTGCTTCTCA

ACGATCAGGAGTTTTCTATGAAAAGTCTTACCGTTGAAGCAAAAAATGCTTTGTCTGATT

TTGTCTATGATGTGAACCATAAGCTGATGGGCGATTTTGTTAGTATGAGTAATGATGAAA

TCCTGCGTTTTCAGAAGATGGAAAAGCTCTTAGAAAATAAAATCTCTCGTCGTTTCAAAA

TCCGCAGGTTAGATAAGGACGACTTCGGCTATCTGATTGAACACCTTTACGGACAGACAG

GCACTGCCTATGAAGAGTATGAGTACCATCTATCAAAGAAAAAGCTGGATAATGAAACGC

TGATTAAATACTATGACTTGATTAAGCCTACTCGCTGTTTGGTGGAAGAAAAACAGCGAT

ATTTGAAAATCCAGCAGGAAGATGAAACCGTCTATGTAGCTTACTTTACCATTAACAGCA

TTGTCGGAGAACTGGACTTCCCGTCCTCTGAAATCTTCTACTACCAGCAACAGCAATTTA

CATTCCCGATTGATACGTCAATGAATGTGGAAATTGTAGCGAATCGTAAAGCCCTATCTA

CTGTCCGCAATAAAAAGAAAGAACTGAAAGACTTGGATAACCACGCTTGGCAAAGTGATA

ATGAAACCAGCTCCAATGTGGCGGAAGCTCTGGAAAGTGTGAATGAGCTGGAAACCAATT

TAGACCAAAGCAAGGAATCTATGTACAAGCTGTCTTATGTGGTAAGGGTATCAGCAAATG

ATCTTGACGAACTCAAACGTCGTTGTAATGAAGTGAAAGATTTTTATGACGATTTAAGCG

TAAAACTGGTACGACCATTTGGGGATATGCTCGGCTTACATGAAGAATTTTTACCTGCCA

GCAAGCGTTATATGAATGATTATATTCAATACGTGACCTCTGATTTCCTCGCTGGTTTAG

GTTTTGGTGCTACTCAAATGCTGGGGGAAAATGAGGGGATTTATGTTGGCTACAGCTTAG

ATACTGGACGCAATGTCTATCTGAAACCTGCTCTTGCCAGTCAAGGGGTTAAGGGTTCAG

TAACCAATGCGTTAGCGTCGGCTTTTGTTGGTTCGCTGGGTGGTGGTAAATCCTTTGCGA

ATAACCTTATCGTCTATTATGCGGTGCTTTATGGGGCACAAGCAGTGATTGTAGACCCAA

AAGCAGAACGTGGCAGATGGAAAGAAACCTTGCCAGAGATTTCCCATGAAATCAATATCG

TCACTCTGACTTCTGATGAGAAAAACAAAGGCTTACTTGACCCTTATGTGATTATGAAAA

ATCCCAAAGATTCTGAATCACTGGCTATTGATATTCTGACATTCCTTACGGGGATTTCCT

CTCGTGATGGGGAACGCTTCCCAATCCTTAGAAAAGCCATTCGTGCAGTAACCAATAGTG

AAGTACGAGGGTTGATGAAAGTGATTGAGGAATTACGGGTTGAGAATACGCCACTAAGTA

CCAGTATAGCCGACCATATCGAAAGTTTTACAGACTATGACTTTGCACATTTATTATTCA

GTAATGGTTATGTGGAGCAGTCTATCAGCTTAGAAAAACAACTGAACATTATACAGGTTG

CGGACTTGGTACTTCCCGACAAGGAAACTTCCTTTGAGGAATATACCACTATGGAGCTTT

TATCCGTTGCTATGCTGATTGTCATTAGTACCTTTGCTTTAGACTTTATCCATACAGACC

GAAGCATTTTCAAGATTGTAGATTTAGACGAAGCATGGAGCTTTTTACAGGTAGCACAAG

GAAAAACACTATCTATGAAGCTGGTTCGGGCTGGTCGTGCTATGAACGCTGGGGTATATT

TCGTGACCCAAAATACAGACGACCTCTTAGATGAAAAACTGAAAAATAACCTCGGCTTAA

AATTTGCATTTCGTTCCACTGACCTTAACGAGATTAAAAAGACCTTAGCCTTTTTTGGTG

TAGACCCAGAGGACGAAAACAATCAGAAGCGATTGCGTGATTTGGAAAACGGGCAATGCC

TTATCAGTGATTTATATGGTCGTGTCGGTGTGATACAGTTCCACCCTGTATTTGAAGAAC

TGCTCCATGCCTTTGATACCAGACCACCTGTGCGAAAAGAGGTGTAAATGTGAAACCATC

AATAGTAAACAGAATAAAATCAAACTGGACGCTGAAACGTCTAGGTAAAGTGGCAATGAC

AGTGGCTTTCACACTTGTGATTGCCATTTTTCTTTTAGCCATGCTGGGAACGGTGGTTCA

AGCTGCGGGCTTGGTAGATGATACGGTCAATGTGGCAAATGAATACAGCCGATACCCACT

TGAAAACTATCAACTGGATTTTTATGTGGATAATAGCTGGGGCTGGCTTCCGTGGAACTG

GTCGGACGGGATTGGAAAACAGGTCATGTATGGACTATATGCCATTACCAATTTTATTTG

GACAATCAGTTTGTATGTTTCCAATGCGACAGGTTACTTAGTACAGGAAGCCTATTCCTT

AGACTTCATTTCCGCTACAGCAGATTCCATTGGTAAGAATATGCAGACCTTAGCTGGTGT

GAGTGCAAACGGATTTTCAACAGAGGGTTTCTATGTTGGATTCCTCTTACTCTTGATTTT

GGTTCTTGGGGTTTATGTTGCCTATACGGGACTGATAAAGAGAGAAACCACAAAGGCAAT

TCATGCCATTATGAATTTTGTGCTGGTGTTTATCCTATCGGCTTCCTTTATTGCCTACGC

TCCCGACTACATTAAAAAAATCAATGACTTTTCATCAGACATCAGTAATGCCAGTTTATC

ACTTGGCACGAAGATTGTCATGCCCCATTCCGATAGTCAAGGCAAGGACAGCGTGGACTT

AATCAGAGATAGCCTGTTTTCCATACAGGTTCAGCAACCGTGGCTACTGCTTCAATACAA

CAGTTCAGACATTGAAAGTATCGGTATTGACCGTGTGGAAAGCCTGCTCTCCACCAGCCC

AGATTCCAACAATGGCGAAGACAGAGAAAAAATTGTTGCGGAAGAAATTGAAGACAGAAG

CAATACCAATCTAACCATTACAAAGACCATTAACCGTTTAGGTACAGTCTTCTTCCTATT

TGTCTTCAATATTGGGATTTCCATATTTGTATTCCTATTAACAGGAATCATGATTTTCTC

GCAGGTACTTTTTATCATCTATGCTATGTTTCTGCCTGTGAGCTTTATTTTAAGCATGAT

TCCATCATTTGATGGTATGTCAAAACGAGCCATAACAAAGCTCTTTAATACCATTTTGAC

ACGAGCTGGAATCACATTGATTATTACGACAGCATTTAGTATTTCAACCATGCTCTATAC

CTTATCGGCTGGTTATCCGTTCTTTTTGATTGCTTTTCTACAGATTGTGACCTTTGCAGG

AATCTACTTCAAGCTGGGCGATTTAATGAGTATGTTTTCTCTACAGAGTAACGATTCTCA

AAGTGTGGGAAGTCGTGTGATGAGAAAACCTCGTATGCTTATGCACGCTCACATGCACCG

TCTACAGCGGAAACTTGGACGTTCCATGACTACTCTAGGGGCTGGGTCTGCCATTGTTAC

AGGTAAAAAAGGACAGTCGGGTTCGGGGAGTTCTGCAAGGACACAAGCAGATCACTCCCG

ACCAGACGGAAAGGAAAAATCAACACTTGGAAAACGTATCGGTCAAACCATCGGTACAGT

AGCTGATACCAAAGACAGAATGGTAGACACTGCTAGTGGTTTGAAAGAACAGGTTAAAGA

TTTGCCGACCAATGCAAGATATGCAGTATATCAAGGAAAATCCAAAGTAAAAGAGAATGT

CCGTGATTTAACCAGTAGTATTTCTCAAACCAAAGCGGACAGAGCCAGTGGACGCAAGGA

ACAGCAGGAACAAAGGCGAAAAACCATTGCGAAGCGTCGCTCTGAAATGGAACAGGTCAA

ACAGAAAAAACAGCCTGCTTCTTCTGTTCATGAAAGACCGACTACAAGACAAGAACAATA

TCATGATGAACAGACCTCAAAACAGTCTAATATTCAGACTTCATATAAGGAATCTCAACA

AGCCAAACAAGAGCGTCCAGCAGTTAAGTCCGATTTTTCAAGTCCAAAAGTGGAACGCCA

AGGCAATACCGTTCAAGAAAAAACCGTTCAAAAGCCAGCAACTTCAACCACTACAGCAGA

TAGAACTTCACAACGTCCAATCACAAAAGAACGTCCGTCTACTGTTCAAAGAGTACCACT

ACAAAATACAAGAAGTAGACCACCAATCAAAACCGCCACCATTAAGAAAGTCGGTAAGAA

ACCATGAAGTTGAAAACTTTAGTGATTGGTGGTTCTGGATTATTCTTGATGGTCTTCTCA

CTGCTTCTGTTTGTTGCCATTTTATTTTCAGATGAACAGGACAGCGGAATTTCCAATATT

CATTATGGAGGTGTGAATGTTTCCGCAGAAGTGCTGGCTCATAAGCCTATGGTAGAAAAA

TATGCCAAAGAATATGGCGTTGAAGAATATGTCAACATACTTCTTGCGATTATACAGGTG

GAATCGGGCGGTACTGCGGAAGATGTTATGCAGTCCTCGGAATCCCTCGGTCTTCCACCT

AATTCATTGAGTACAGAAGAATCCATTAAGCAAGGTGTGAAGTATTTCAGTGAATTATTA

GCCAGTAGCGAAAGGCTCAGTGTAGATTTAGAATCGGTTATCCAGTCCTACAATTATGGT

GGTGGTTTCTTAGGGTATGTGGCTAATCGTGGAAATAAATATACCTTTGAACTGGCTCAA

AGTTTCTCAAAAGAGTATTCAGGTGGCGAAAAAGTGTCTTACCCCAATCCCATAGCCATA

CCTATCAATGGGGGCTGGCGATACAACTATGGCAATATGTTTTATGTGCAACTGGTAACG

CAGTATCTTGTCACAACAGAGTTTGATGATGATACGGTACAAGCCATCATGGACGAAGCA

CTGAAATATGAGGGCTGGCGATACGTTTACGGTGGAGCTTCCCCGACTACTTCTTTTGAT

TGTAGCGGACTGACACAATGGACGTATGGAAAAGCTGGAATTAACTTACCACGAACCGCA

CAACAGCAATATGATGTGACCCAGCATATCCCACTATCGGAAGCACAAGCTGGCGATTTG

GTTTTCTTTCATTCTACCTATAACGCTGGCTCTTATATTACTCATGTTGGGATATACCTT

GGCAATAACCGTATGTTTCATGCAGGCGACCCAATCGGTTATGCCGACTTAACAAGCCCC

TACTGGCAACAGCATTTAGTGGGAGCAGGACGAATCAAACAATGAGAAAGGAAGATTTAA

TGATGAAATTTAGAAAAAATCAGAATAAAGAAAAACAGATACCAAAGGAAAAGAAACCTC

GTGTCTACTATAAGGTCAATCCTCATAAAAAGGTTGTGATTGCCTTGTGGGTACTTTTAG

GGCTTAGTTTCAGCTTTGCGATATTCAAGCACTTTACAGCTATAGATACTCATACTATTC

ACGAAACAACTATCATAGAAAAGGAATACGTTGATACTCATCATGTAGAAAATTTTGTAG

AGAACTTTGCGAAAGTCTACTATTCATGGGAGCAATCCGATAAGTCCATTGATAATCGAA

TGGAAAGTCTAAAAGGCTATCTGACAGATGAACTTCAAGCTCTCAATGTTGATACAGTAC

GCAAAGATATTCCTGTATCGTCTTCTGTAAGAGGATTTCAGATATGGACGGTAGAGCCAA

CTGGCGACAATGAGTTTAATGTAACCTACAGTGTAGACCAGCTCATTACAGAGGGAGAAA

ATACAAAGACCGTCCACTCTGCTTATATAGTGAGTGTCTATGTAGATGGTTCTGGAAATA

TGGTACTGGTTAAGAATCCGACCATTACCAACATACCTAAGAAATCAAGTTATAAACCAA

AAGCCATTGAAAGTGAGGGGACGGTTGATTCCATTACAACCAATGAAATCAATGAGTTTT

TAACGACGTTCTTCAAGCTCTATCCTACAGCGACAGCCAGTGAACTTTCCTACTATGTGA

ATGACGGGATATTAAAACCAATCGGAAAAGAGTACATCTTTCAAGAACTGGTAAATCCTA

TTCACAATCGTAAGGATAATCAAGTCACGGTATCGCTGACAGTGGAGTATATCGACCAGC

AGACCAAAGCAACGCAGGTATCTCAATTTGATTTGGTACTTGAAAAGAACGGGAGTAATT

GGAAGATTATAGAATAACAAATATTGGTACATTATTACAGCTATTTTGTAATCACGTACT

CTCTTTGATAAAAAATTGGAGATTCCTTGACAAATATGCTCTTACGTGCTATTATTTAAG

TATCTATTTAAAAGGAGTTAATAAATATGCGGCAAGGTATTCTTAAATAAACTGTCAATT

TGATAGTGGGAACAAATAATTGGATGTCCTTTTTTAGGAGGGCTTAGTTTTTTGTACCCA

GTTTAAGAATACCTTTATCATGTGATTCTAAAGTATCCGGAGAATATCTGTATGCTTTGT

ATGCCTATGGTTATGCATAAAAATCNNNNNCACACACTTAATTAATTAAGTGTGTGNNNN

NATTTTTATGCCCTTTTGGGCTTTTGAATGGAGGAAAATCACATGAAAATTATTAATATT

GGAGTTTTAGCTCATGTTGATGCGGGAAAAACTACCTTAACAGAAAGCTTATTATATAAC

AGTGGAGCGATTACAGAATTAGGAAGCGTGGACAAAGGTACAACGAGGACGGATAATACG

CTTTTAGAACGTCAGAGAGGAATTACAATTCAGACAGGAATAACCTCTTTTCAGTGGGAA

AATACGAAGGTGAACATCATAGACACGCCAGGACATATGGATTTCTTAGCAGAAGTATAT

CGTTCATTATCAGTTTTAGATGGGGCAATTCTACTGATTTCTGCAAAAGATGGCGTACAA

GCACAAACTCGTATATTATTTCATGCACTTAGGAAAATGGGGATTCCCACAATCTTTTTT

ATCAATAAGATTGACCAAAATGGAATTGATTTATCAACGGTTTATCAGGATATTAAAGAG

AAACTTTCTGCCGAAATTGTAATCAAACAGAAGGTAGAACTGTATCCTAATATGTGTGTG

ACGAACTTTACCGAATCTGAACAATGGGATACGGTAATAGAGGGAAACGATGACCTTTTA

GAGAAATATATGTCCGGTAAATCATTAGAAGCATTGGAACTCGAACAAGAGGAAAGCATA

AGATTTCAGAATTGTTCTCTGTTCCCTCTTTATCATGGAAGTGCAAAAAGTAATATAGGG

ATTGATAACCTTATAGAAGTTATTACTAATAAATTTTATTCATCAACACATCGAGGTCCG

TCTGAACTTTGCGGAAATGTTTTCAAAATTGAGTATTCGGAAAAAAGACAGCGTCTTGCA

TATATACGTCTTTATAGTGGCGTACTGCATTTGCGAGATTCGGTTAGAATATCGGAAAAG

GAAAAAATAAAAATTACAGAAATGTATACTTCAATAAATGGTGAATTATGTAAAATCGAT

AAGGCTTATTCCGGGGAAATTGTTATTTTGCAGAATGAGTTTTTGAAGTTAAATAGTGTT

CTTGGAGATACAAAGCTATTGCCACAGAGAGAGAGAATTGAAAATCCCCTCCCTCTGCTG

CAAACGACTGTTGAACCGAGCAAACCTCAACAAAGGGAAATGTTACTTGATGCACTTTTA

GAAATCTCCGACAGTGACCCGCTTCTGCGATATTATGTGGATTCTGCGACACATGAAATC

ATACTTTCTTTCTTAGGGAAAGTACAAATGGAAGTGACTTGTGCTCTGCTGCAAGAAAAG

TATCATGTGGAGATAGAAATAAAAGAGCCTACAGTCATTTATATGGAAAGACCGTTAAAA

AAAGCAGAGTATACCATTCACATCGAAGTTCCACCGAATCCTTTCTGGGCTTCCATTGGT

CTATCTGTAGCACAGCTTCCATTAGGGAGCGGAGTACAGTATGAGAGCTCGGTTTCTCTT

GGATACTTAAATCAATCGTTTCAAAATGCAGTTATGGAGGGGATACGCTATGGCTGTGAA

CAAGGATTGTATGGTTGGAATGTGACGGACTGTAAAATCTGTTTTAAGTATGGCTTATAC

TATAGCCCTGTTAGTACCCCAGCAGATTTTCGGATGCTTGCTCCTATTGTATTGGAACAA

GTCTTAAAAAAAGCTGGAACAGAATTGTTAGAGCCATATCTTAGTTTTAAAATTTATGCG

CCACAGGAATATCTTTCACGAGCATACAACGATGCTCCTAAATATTGTGCGAACATCGTA

GACACTCAATTGAAAAATAATGAGGTCATTCTTAGTGGAGAAATCCCTGCTCGGTGTATT

CAAGAATATCGTAGTGATTTAACTTTCTTTACAAATGGACGTAGTGTTTGTTTAACAGAG

TTAAAAGGGTACCATGTTACTACCGGTGAACCTGTTTGCCAGCCCCGTCGTCCAAATAGT

CGGATAGATAAAGTACGATATATGTTCAATAAAATAACTTAGTGTATTTTATGTTGTTAT

ATAAATATGGTTTCTTGTTAAATAAGATGAAATATTTTTTAATAAAGATTTGAATTAAAG

TGTAAAGGAGGAGATAGTTATTATAAACTACAAGTGGATATTGTGTCCTGTATGTGGAAA

TAAAACACGATTAAAGATAAGGGAAGATACTGAATTAAAAAAATTCCCCCTCTATTGTCC

GAAATGCAGACAAGAAAATTTAATTGAAATAAAGCAGTTCAAAGTAACTGTGATTACAGA

GCCAGACGCAAAGACGCAGAGCCGATAAAATGAGATTAATACAATCTCATTTTATCGGCT

CTTTCCGTTATGTATGGATTCTTTTAATTAGTCTTCGATGTTTCTTGCTTCGTTGATACC

GCTGGCTAAAGATTCCATTAAGGATAGTTCTTTGTCTGTAAAGCTATCCATGTATTTCTC

TATCTGTAATCGTCGGGTGCTTTTTACCAAGTTATTAGCAGGTAAGAAAAATTCATCAAC

GGAAACATGAAGTAACGATACAAGGTCATAAAGAACTTGTATGCTGGGGTGTTGCCCTTT

ATTTTCAATATTAGTTAAGTACCGTGGGTCAATTTCAATCAATGCTCCCACTTGTTCACG

AGTTAAACCTCGTTTCAATCGAGCTTCTTTAATGGCTAAACCAAAGGCTCTAAAATCATA

TTTATCTTCTTTTTTACGCATAGTAGACCACCTCTATACATTTTATTGTCCCTACTGAAT

TAAAAACAGGTATAGAAAAACGTGTTATATGGTTTATAGGTTTATATTTAATAAAAAGCA

CTACTAAACGCCAATAAAAAAAACCGTTATATGGTAGTGCTATTTACGCTGTTAAAATAT

TGTATATTACTTCCAAATGGCGGTTTGTTGGAGGTCAACGTCGCCATGAAGTACATCATA

TACAATAAATTTCCTTACATTGGGTTCTTGTCAAAAAAAGTCGTCTATCTGCAATAGATA

AGTACGTCCACCAATGTGGTTTTATAAATCATATAGATAGAATAACAGAAGCATGTAAAC

AGAGAAATAAATCTGTTTATATGCTTTTTTGGCTATTCAGAACTTTTTTACAAAGTTTAT

TTATCAGTAATGCAACAAATCCCCCTTTCACATTGGGACTAAGAGTGAAAGGAGATAAAC

GAGCAAGGCTCACTTCCTTTCCTAGACAGAAAGGGGGTGAGAAACATGAAACCATCTTCT

TTTCAGACCACAATAGAAAATCAGTTTGACTATATCTGTAAACGTGCTATGGAAGACGAG

CGAAAGAATTATATGCTTTATCTTTCAAGGATTGCAAAGCGTGAGGTGTCCTTTTCGGAT

GTTGGCGATTATCTTGTTAGCCAGTTTGCGACAACAGATAACTATTCAACTGACTTTCAG

ATTTTTACACTCAATGGGTTATCAGTAGGCGTTGAAAATGATTTGTTGAGTGAAGCATTA

CGTGAGTTGCCAGACAAGAAACGTGAAATTCTACTGCTGTTTTACTTTATGGACATGAGC

GATTCAGAAATTGCAGACCTGTTGAAATTGAACCGTTCTACTGTCTATCGGCATAGAACC

AGTGGACTAGCCTTAATTAAAAAGTTTATGGAGGAATTTGAAGAATGAAAACACAATATC

CTATGATTCCCTTTCCTCTCATTGTAAAGGCAACAGATGGCGATACCGAAGCGATTAACC

AGATTCTACATCATTACAGAGGGTACATAACGAAGCGTTCCCTACGACTTATGAAAGATG

AATATGGCAATCAAAGTATGGTCGTTGATGAAGTCTTACGTGGAAGAATGGAAACCAGAC

TGATTACAAAGATTTTGTCATTTGAAATTAAGTAATATCCTCTCTCCTTTCGTGGAAGCG

TGCTAAACCATTCCACGCTTCCCGAACAGGGAGGTTTGTTATTCCACCAAAGCATATTGA

GCTTTCAATGTGTTTTGATAGGCTAACGAGCCATTGTTCTTTGAAAACTGAATAAAAGTA

ATCGAATACGTTTCGATAAGAAAAGAGCCAACGGAACTAACCGCCATGACCTATCTTATA

AAGATAGCGAGCGATTCATGTTAGTGATCCGAGAAGCAATCTTTAGCAGGATTGCCTGCA

ACGACATTCTTATCGTGATAATGATACTCCCATACAGTCAATAGTCCGAGCGTGATAAAA

CCGTCGCAGGCAATGAGTATGGCTACATGAGAACCATGCAGGGGTGGAACTCCCGTGAGC

TTTGCTAAAGCTGTTCGATTGCTGGTAAAACAACTTTTATGAAATCCAAATAAGTGATTT

GGAAAGGAGGATTTTATGAAGCAGACTGACATTCCTATTTGGGAACGTTATACCCTAACC

ATTGAAGAAGCGTCAAAATATTTTCGTATTGGCGAAAACAAGCTACGACGCTTGGCAGAG

GAAAATAAAAATGCAAATTGGCTGATTATGAATGGCAATCGTATTCAGATTAAACGAAAA

CAATTTGAAAAAATTATAGATACATTGGACGCAATCTAGCGTCGCCAAAGGGTCTTGTAT

ATGATAAAATAGTATTAAGTCGTATCAAGGCTCTTTCCATAAAGGAAAGGAGCAAATGCC

ATGTCAGAAAAAAGACGTGACAATAAAGGTCGAATCTTAAAGACTGGAGAGAGCCAACGA

AAAGACGGAAGATACTTATACAAATATATAGATTCATTTGGAGAACCGCAATTTGTTTAC

TCGTGGAAACTTGTGGCTACAGACCTAGTACCAGCAGGAAAGCGTGATTGTATCTCACTT

AGAGAGAAAATCGCAGAGTTACAGAAAGACATTCATGATGGTATTGATGTTGTAGGAAAG

AAAATGACACTCTGCCAGCTTTACGCAAAACAGAACGCTCAAAGACCAAAGGTTAGAAAA

AACACTGAAACTGGACGCAAATATCTTATGGATATTTTGAAGAAAGACAAGTTAGGTGTA

AGAAGTATTGACAGTATTAAGCCATCAGACGCTAAAGAATGGGCTATTAGAATGAGTGAA

AATGGTTATGCTTATCAAACCATCAATAACTACAAACGTTCTTTAAAGGCTTCATTCTAT

ATTGCTATACAAGATGATTGTGTTCGGAAGAATCCATTTGACTTTCAACTGAAAGCAGTT

CTTGATGATGATACTGTCCCTAAGACCGTACTAACAGAAGAACAGGAAGAAAAACTGTTA

GCCTTTGCAAAAGCTGATAAAACCTACAGCAAAAATTATGATGAAATTCTGATACTCTTA

AAAACAGGTCTTCGTATTTCAGAGTTTGGTGGTTTGACACTTCCAGATTTAGATTTTGAG

AATCGTCTTGTCAATATAGACCATCAGCTATTGAGAGATACTGAAATTGGGTACTACATT

GAAACACCAAAGACCAAAAGTGGCGAACGTCAAGTTCCTATGGTTGAAGAAGCCTATCAA

GCATTTAAGCGAGTGTTAGCGAATCGAAAGAATGATAAGCGTGTTGAGATTGATGGATAT

AGTGATTTCCTCTTTCTTAATAGAAAGAACTATCCAAAAGTGGCAAGTGATTACAACGGC

ATGATGAAAGGTCTTGTTAAGAAATACAATAAGTATAACGAGGATAAATTGCCACACATC

ACTCCACATAGTTTGCGACATACATTCTGTACCAACTATGCAAATGCAGGAATGAATCCA

AAGGCATTACAGTACATTATGGGACATGCTAATATAGCCATGACGCTGAACTATTACGCA

CATGCAACATTCGATTCTGCAATGGCAGAAATGAAACGCTTGAATAAAGAGAAGCAACAG

GAGCGTCTTGTTGCTTAGTAGTACAAATGAATTTACTACTTATTTACCACTTCTGACAGC

TAAGACATGAGGAAATATGCAAAGAAACGTGAAGTATCTTCCTACAGTAAAAATACTCGA

AAGCACATAGAATAAGGCTTTACGAGCATTTAAGAAAATATAAAAAGATAATTAGAAATT

TATACTTTGTTT

>GA11304/Tn6002

AAAATAGCATAAAAATCTAGTTATCCGCATAAAAACTGGACTTATCACACTTTATCAAGG

TCAAAACCACTCAATTTACTACTAATTTACTACTTATGAATGAGCTTTGATACGACGATT

TATCCTTGAAAAGTGAAGATATAAAGATACTTCCAATAAAATTTGAATATTTAATAGGTA

GACACTTCAAAAAATGAGGTGTCTATTTTTTTACCCGATTTTGAAAGGAAGTGAACTTAT

GAAAACAAAAAATCAAGAATCAAAAGGTCGTTCCCCACTCTTTAAGACCATCAAACATTC

ATTCAGCCAATAAAAAAGAAAGGATAGGTAAAAATATGGAACTTAAATTTGTGATTCCCA

ACATGGAAAAAACATTCGGCAATTTAGAATTTGCTGGCGAGGATAAAGTCGTTCAGCGAA

GAATCAACGGACGGCTAACTGTCTTATCAAGAAGCTATAATCTCTATTCTGATGTTCAAA

GAGCAGATGATATTGTGGTGGTGCTTCCTGCTGAAGCTGGCGAAAAACATTTCGGCTTTG

AGGAACGTGTGAAGTTAGTCAATCCACGTATTACCGCAGAGGGCTACAAAATCGGCACTC

GTGGTTTTACAAATTACCTTTTACATGCTGACGACATGATAAAAGAATAAAGAAAGAGAG

GAAAAATGATGAGATTAGCAAATGGCATTGTATTAGATAAAGACACGACTTTTGGAGAAT

TGAAATTCTCTGCTCTACGTCGTGAAGTGAGAATCCAAAATGAAGACGGGTCGGTTTCAG

ATGAAATCAAGGAACGTACCTATGACTTAAAATCCAAAGGACAAGGACGCATGATTCAAG

TAAGTATTCCTGCCAGCGTGCCTTTGAAAGAGTTTGATTATAACGCACGGGTGGAACTTA

TCAATCCCATTGCGGACACCGTTGCTACTGCCACCTATCAAGGAGCAGATGTTGACTGGT

ATATCAAGGCAGACGATATTGTGCTGACAAAGGATTCTAGTTCATTCAAAGCTCAACCAC

AAGCAAAGAAAGAACCGACACAAGACAAATAGTCGCTAGGTAGAAAGGAGACTTTTTCGC

ATGAAACAGCGTGGTAAAAGGATTCGCCCATCTGGTAAAGATTTAGTCTTTCATTTTACG

ATAGCGTCACTCCTGCCTGTTTTCCTGCTGGTTGTCGGACTGTTTCATGTGAAGACAATC

CAGCAGATCAACTGGCAGGATTTTAACCTATCACAAGCAGATAAGATTGACATTCCCTAT

TTAATTATCAGTTTCAGTGTCGCAATTCTTATCTGCTTGCTGGTAGCGTTTGTATTCAAA

CGGGTTCGCTATGATACGGTTAAACAACTTTACCACCGTCAAAAACTGGCAAAGATGATA

CTTGAAAACAAGTGGTATGAATCTGAACAGGTCAAAACAGAGGGTTTCTTTAAAGATAGT

GCTGGTCGTACAAAGGAAAAGATAACCTACTTCCCTAAAATGTATTATCGACTTAAAAAT

GGCTTGATACAGATACGGGTGGAAATCACGCTGGGAAAATATCAAGACCAACTCTTACAC

TTGGAAAAGAAATTAGAGAGTGGCTTGTACTGTGAGCTGACGGATAAAGAGTTAAAGGAT

TCCTATGTGGAATATACTTTGCTCTATGACACCATAGCCAGTCGTATTTCTATTGATGAA

GTAGAAGCTAAAGATGGTAAACTTCGCTTAATGAAAAACGTATGGTGGGAATATGATAAG

CTCCCTCATATGTTGATTGCTGGTGGTACAGGTGGCGGTAAAACTTACTTTATACTGACA

CTGATTGAAGCCTTGCTTCATACAGATTCAAAACTGTATATTCTTGACCCGAAAAATGCT

GACCTTGCGGACTTAGGTTCTGTGATGGCAAATGTCTACTATAGAAAAGAAGACTTGCTT

TCTTGCATTGAAACATTCTATGAAGAAATGATGAAACGTAGTGAGGAAATGAAGCAGATG

AAGAACTATAAGACTGGCAAAAATTATGCTTACTTAGGTCTCCCGGCACACTTCTTAATC

TTTGATGAATACGTCGCTTTCATGGAAATGCTGGGAACAAAAGAAAACACCGCAGTTATG

AATAAGCTGAAACAGATTGTCATGTTAGGTCGTCAAGCTGGCTTCTTTCTAATACTGGCT

TGTCAACGTCCAGACGCAAAATATTTAGGCGACGGAATCCGTGATCAGTTTAATTTCAGA

GTGGCTTTAGGTCGTATGTCTGAAATGGGCTATGGCATGATGTTTGGCAGTGACGTACAA

AAGGATTTCTTCTTAAAGCGAATCAAAGGTCGTGGCTATGTTGATGTAGGAACAAGTGTC

ATATCAGAGTTTTATACTCCCCTTGTACCAAAAGGATATGATTTCTTGGAGGAAATTAAA

AAGTTATCCAACAGCAGACAGTCCACGCAGGCGACGTGCGAAGCGGAAGTCGCAGGTGTG

GACTGATCTTGCTGGCTGGTGTGGCAATAGCCACGCCAGCACTTACCCCCCCGTATCTAA

CAGGGGGGTACAAATCGACAGGAAACAGTCAAAAAAACATTAGAAAATCCTTTGGTTACA

AGGGATTTACAAAATTTCAGCGTATGTCAAATGGGCTTTAAAAGTTGACATACGCCTTTT

TGATTGGAGGGATTTTTACTGAATGAACAAACTTGGTTACAGCATTTAAAAGAAAAACGC

TTGGCTTATGGACTATCTCAAAACCGTTTAGCTGTTGCGACTGGTATTACAAGGCAGTAT

CTAAGCGATATTGAAACAGGAAAAGTCAAGCCATCAGAGGATTTACAGCAGTCCCTTTGG

GAAGCTCTGGAACGCTTCAATCCCGACGCTCCCCTTGAAATGCTGTTTGATTATGTAAGA

ATTCGCTTTCCGACAACAGACGTACAGCAGGTGGTCGAAAACATCTTACAACTGAAACTG

TCCTATTTTCTTCATGAGGACTATGGTTTCTATTCTTATTCAGAGCATTATGCTTTAGGC

GACATATTCGTCCTTTGCTCCCATGAACTGGACAAAGGAGTTCTGGTGGAATTGAAAGGT

CGTGGGTGCAGACAATTTGAAAGCTATCTTCTGGCACAACAAAGAAGCTGGTATGAGTTC

TTTATGGACGTTTTGGTGGCTGGCGGTGTGATGAAACGCCTTGACCTTGCCATTAACGAT

AAGACAGGGATTTTAAATATCCCTGTACTCACTGAAAAGTGCCAACAGGAAGAATGTATC

TCCGTCTTCCGCAGTTTTAAAAGCTATCGCAGTGGCGAACTGGTACGCAAAGAGGAAAAG

GAATGTATGGGAAACACCCTCTATATCGGTTCATTACAAAGTGAAGTTTATTTCTGTATC

TATGAAAAGGACTACGAGCAGTACAAGAAAAATGATATTCCCATTGAAGACGCAGAAGTA

AAAAACCGTTTTGAGATTCGATTGAAAAATGAGCGTGCCTATTATGCAGTCCGTGATTTA

CTCGTCTATGACAATCCAGAGCATACCGCCTTTAAAATTATCAATCGGTATATCCGTTTT

GTAGATAAAGACGATTCCAAACCTCGTTCTGATTGGAAACTGAATGAAGAATGGGCTTGG

TTTATTGGGAACAATCGTGAACGATTAAAACTAACCACAAAACCAGAGCCTTACTCCTTC

CAAAGGACGCTGAACTGGCTATCTCATCAAGTTGCCCCGACCTTAAAGGTTGCGATTAAA

CTTGATGAAATCAACCAGACGCAGGTTGTAAAAGACATTCTCGACCATGCGAAACTGACA

GACCGACACAAGCAGATTTTGAAGCAACAGTCAGTAAAAGAACAGGACGTGATAACAACA

AAAAAAGGATATCTGTCAACCATACCAGTTGACAGATATCCAAAAAAAGATATAATGGGA

GATAAGACGGTTCGTGTTCGTGCTGACTTGCACCATATCATAAAAATCGAAACAGCAAAG

AATGGCGGAAACGTAAAAGAAGTTATGGAAATAAGACTTAGAAGCAAACTTAAGAGTGTG

TTGATAGTGCATTATCTTAAAATTTTGTATAATAGGAATTGAAGTTAAATTAGATGCTAA

AAATTTGTAATTAAGAAGGAGGGATTCGTCATGTTGGTATTCCAAATGCGTAATGTAGAT

AAAACATCTACTGTTTTGAAACAGACTAAAAACAGTGATTACGCAGATAAATAAATACGT

TAGATTAATTCCTACCAGTGACTAATCTTATGACTTTTTAAACAGATAACTAAAATTACA

AACAAATCGTTTAACTTCTGTATTTGTTTATAGATGTAATCACTTCAGGAGAGATTACAT

GAACAAAAATATAAAATATTCTCAAAACTTTTTAACGAGTGAAAAAGTACTCAACCAAAT

AATAAAACAATTGAATTTAAAAGAAACCGATACCGTTTACGAAATTGGAACAGGTAAAGG

GCATTTAACGACGAAACTGGCTAAAATAAGTAAACAGGTAACGTCTATTGAATTAGACAG

TCATCTATTCAACTTATCGTCAGAAAAATTAAAACTGAATACTCGTGTCACTTTAATTCA

CCAAGATATTCTACAGTTTCAATTCCCTAACAAACAGAGGTATAAAATTGTTGGGAATAT

TCCTTACCATTTAAGCACACAAATTATTAAAAAAGTGGTTTTTGAAAGCCGTGCGTCTGA

CATCTATCTGATTGTTGAAGAAGGATTCTACAAGCGTACCTTGGATATTCACCGAACACT

AGGGTTGCTCTTGCACACTCAAGTCTCGATTCAGCAATTGCTTAAGCTGCCAGCGGAATG

CTTTCATCCTAAACCAAAAGTAAACAGTGTCTTAATAAAACTTACCCGCCATACCACAGA

TGTTCCAGATAAATATTGGAAGCTATATACGTACTTTGTTTCAAAATGGGTCAATCGAGA

ATATCGTCAACTGTTTACTAAAAATCAGTTTCATCAAGCAATGAAACACGCCAAAGTAAA

CAATTTAAGTACCATTACTTATGAGCAAGTATTGTCTATTTTTAATAGTTATCTATTATT

TAACGGGAGGAAATAATTCTATGAGTCGCTTTTTTAAATTTGGAAAGTTACACGTTACTA

AAGGGAATGGAGATAAATTATTAGATATACTACTGACAGCTTCCAAGAAGCTAAAGAGGT

CCCTAGCGCCTACGGGGAATTTGTATCGATAAGGGGTACAAATTCCCACTAAGCGCTCGG

GACCCCTTGTAGGAAAATGTCCTAAGTGGGATATCTGTCAACTGGTATGGTTGACTAAAA

ATACTTCCTACGAAAATGTAGGGGGTATTTTTTACGAAAAATACAATCGATTCTTAAAAA

GAAAAATTTTTGATTGGCAAAACCATAACAAGTTCGTTTTAGGGTTTTGATTTTGCGATT

GATGCCTTCTAAAGGACCATTAGAGTATTCAAATTTAGCGCTATTTAAGACATATTTTCT

GTTTTGACGAAGGGTTTGAATAGCAGTATCCATTTCTGTATTGGTTTTTTGGTAGTCTAA

GATGGTTGACTCTAGTAATTCACTATTGCGCTCGTTTAGGGCTTTCGTGATATCTTGGTA

AGTTTGGTATACTTCAGCGAACTTGGAAAATTTACTAGTAATGAGATCAACAGCATTTTG

GCGAGTCATATATTGTTTAACGCCGCGAAGAAAAACTACTTCTTCAGGGTGAAGATCTTC

AGCTTTTTTATGGAATAGCTTCCAATGTGACTTCATAATTTTATATTCTTGGCTCTGTTT

ATCAAGTTGCTTTAGGATAGAGATACGACAATTGTCCAAAGCGCGACCAGCTAATTGTAC

AAGGTGGAAGCGATCAATAATGATATTGGCATTAGGGAAAAGGCGATAGATAAAACTTTG

ATATTGAGCATTTAAATCAATTACAACTGATTGAACGCATTCGCGTTCGGCTTTTGAATA

ACGACTTTCAAAATAATCAACAATGGTAGGTGATAGACGATCCTGTAACTTTGTGACAAT

TTGGTGGGTTTCAGCGTCACAACAGATAAAGGACATCACAGACTTAATTGAACGAAACTC

GTCAAAACATAGATGCTTAGGCAACTTAGCCACACGATAGTGTGGTTCCATGCGCTCTAA

GATTGTTCGACGAACACTGCTAGGAGAGCAGTGACACATTTCAGCAATAAGCTGACCAGA

TAAGCCTTTACGAGCTAAAAGCATGATTTGATTTTTGAGATCACTGGATAAGGTTTGATT

TTCTTTGGTTAAATTAGTAATAGCACCAAAAGTAGTATGGCATGATTTACATTTATAGCG

TTGTTTACGAAGCTCTAGTTCATATCTTCTCCCATTTAAACTTGCCAGTCGTACATGAGT

TTTGCGAAAGCCATCCTTATTAACTGTGGGAAAGCCACAGTTACGACAACGATTAATCGG

ATAAGAAAGAGTAGCTGTTATTAGCGTTATATACTCTTTAACAGAATCGTTGTTGTGTTC

AGCTTCTTCAACAGAAATAATTTTAATATTTTTATCTTTAATTCCAAGAATATTTAGGAT

AGAATCATTATGGGACATTTGTTTAACCTTCTTTCATGATTTTTGTGGTGAATTGATTGT

ATAACGAGGGGACAGCAAATGTCCTCTTTTTTGTATAAAAAAATCTGGCATGGAATCTCT

ATCCATACCAGAAAGTGTATACCCCAAAAAATAACTCAAATACAAATTCATTGAATATAG

AGAGGAGAACATTTTTATGAATTTTGGACAAAACCTTTATAACTGGTTTCTATCAAACGC

TCAATCACTGGTGCTTTTAGCAATCGTTGTGATTGGCTTGTATCTTGGCTTCAAGCGTGA

GTTTAGCAAACTGATTGGCTTTTTAATTATTGCGATTATTGCGGTTGGCTTAGTCTTCAA

CGCTGCTGGAGTAAAAGACATTTTACTAGAGCTATTCAATCGCATTATTGGTGCTTAAAT

AAAACCGTTCTTTTGTGGAATATAAGTGGTTTTCTTATGTTCCGCAAAGGAATGGTACAC

CAAACGAAGTGCGGTAGGGATTTTTGAATCTCTACAAAGAAAGGACGTGAATATATGGAC

GATATGCAAGTCTATATTGCGAATTTAGGCAAATACAATGAGGGCGAATTGGTCGGTGCG

TGGTTTACCTTTCCCATTGACTTTGAGGAAGTCAAAGAGAAAATCGGCTTGAATGATGAA

TATGAGGAATACGCCATTCATGACTACGAGTTACCCTTTACGGTTGACGAATACACTTCC

ATTGGCGAACTCAATCGACTATGGGAAATGGTATCGGAATTACCCGAAGAATTACAATCG

GAGCTATCTGCTCTGCTCACTCATTTTTCAAGCATTGAAGAACTAAGCGAACATCAAGAG

GATATTATCATTCATTCCGATTGTGATGATATGTATGACGTGGCACGCTACTACATTGAA

GAAACGGGTGCTTTAGGCGAAGTACCAGCTAGTCTTCAAAACTATATTGATTATCAAGCC

TATGGTCGGGATTTAGACCTTTCAGGAACGTTTATCTCAACCAATCATGGGATTTTTGAA

ATCGTCTATTAAATCTGTCGGTACATTACTACTGGCAGATTTTCTATTTTACGGGGTGGC

TCAATCAGCTACCCCTATTTTTTATGAAAGGATTGATTACATGAAGAAAATACGAAGCTA

TACCAGTATCTGGTCTGTGGAAAAGGTACTGTATTCTATCAATGATTTTAGACTTCCGTT

TCCCATAACCTTTACGCAAATGACATGGTTTGTCGTGTCACTCTTTGCAGTGATGATACT

TGGCAACTTGCCCCCTCTTTCCATGATAGAGGGAGCATTTCTCAAATACTTTGGGATTCC

TGTGGCTTTCACATGGTTTATGTCTACAAAAACTTTTGATGGTAAAAAGCCTTATGGATT

TTTGAAGTCTGTCATTGCTTATGCACTGCGACCAAAGCTGACCTATGCAGGAAAAAAGTA

ACGCTTGGCAGAAACCAGCCACAAGAAGCCATTACAGCAGTTAGGAGTGAATTTTATGGC

ATATCCAATTAAATACATTGAAAACAATCTCGTCTGGAATAAAGACGGGGAATGTTATGC

TTACTATGAGCTTGTTCCTTACAATTACTCATTTCTAAGTCCAGAACAGAAAATACAAGT

GCATGATTCTTTCAGACAGCTTATCGCACAAAATCGTGATGGCAAAATTCATGCTTTACA

AATCAGTACAGAATCCAGCATACGTTCTGCACAAGAGCGTTCCAAAAATGAAGTCACTGG

CAAGCTCAAAGCGGTTGCCTATGACAAAATCGACCAACAGACAGACGCTTTAATATCCAT

GATTGGCGAAAATCAAGTGAACTACCGTTTCTTTATCGGCTTTAAGTTGCTTCTCAACGA

TCAGGAGTTTTCTATGAAAAGTCTTACCGTTGAAGCAAAAAATGCTTTGTCTGATTTTGT

CTATGATGTGAACCATAAGCTGATGGGCGATTTTGTTAGTATGAGTAATGATGAAATCCT

GCGTTTTCAGAAGATGGAAAAGCTCTTAGAAAATAAAATCTCTCGTCGTTTCAAAATCCG

CAGGTTAGATAAGGACGACTTCGGCTATCTGATTGAACACCTTTACGGACAGACAGGCAC

TGCCTATGAAGAGTATGAGTACCATCTATCAAAGAAAAAGCTGGATAATGAAACGCTGAT

TAAATACTATGACTTGATTAAGCCTACTCGCTGTTTGGTGGAAGAAAAACAGCGATATTT

GAAAATCCAGCAGGAAGATGAAACCGTCTATGTAGCTTACTTTACCATTAACAGCATTGT

CGGAGAACTGGACTTCCCGTCCTCTGAAATCTTCTACTACCAGCAACAGCAATTTACATT

CCCGATTGATACGTCAATGAATGTGGAAATTGTAGCGAATCGTAAAGCCCTATCTACTGT

CCGCAATAAAAAGAAAGAACTGAAAGACTTGGATAACCACGCTTGGCAAAGTGATAATGA

AACCAGCTCCAATGTGGCGGAAGCTCTGGAAAGTGTGAATGAGCTGGAAACCAATTTAGA

CCAAAGCAAGGAATCTATGTACAAGCTGTCTTATGTGGTAAGGGTATCAGCAAATGATCT

TGACGAACTCAAACGTCGTTGTAATGAAGTGAAAGATTTTTATGACGATTTAAGCGTAAA

ACTGGTACGACCATTTGGGGATATGCTCGGCTTACATGAAGAATTTTTACCTGCCAGCAA

GCGTTATATGAATGATTATATTCAATACGTGACCTCTGATTTCCTCGCTGGTTTAGGTTT

TGGTGCTACTCAAATGCTGGGGGAAAATGAGGGGATTTATGTTGGCTACAGCTTAGATAC

TGGACGCAATGTCTATCTGAAACCTGCTCTTGCCAGTCAAGGGGTTAAGGGTTCAGTAAC

CAATGCGTTAGCGTCGGCTTTTGTTGGTTCGCTGGGTGGTGGTAAATCCTTTGCGAATAA

CCTTATCGTCTATTATGCGGTGCTTTATGGGGCACAAGCAGTGATTGTAGACCCAAAAGC

AGAACGTGGCAGATGGAAAGAAACCTTGCCAGAGATTTCCCATGAAATCAATATCGTCAC

TCTGACTTCTGATGAGAAAAACAAAGGCTTACTTGACCCTTATGTGATTATGAAAAATCC

CAAAGATTCTGAATCACTGGCTATTGATATTCTGACATTCCTTACGGGGATTTCCTCTCG

TGATGGGGAACGCTTCCCAATCCTTAGAAAAGCCATTCGTGCAGTAACCAATAGTGAAGT

ACGAGGGTTGATGAAAGTGATTGAGGAATTACGGGTTGAGAATACGCCACTAAGTACCAG

TATAGCCGACCATATCGAAAGTTTTACAGACTATGACTTTGCACATTTATTATTCAGTAA

TGGTTATGTGGAGCAGTCTATCAGCTTAGAAAAACAACTGAACATTATACAGGTTGCGGA

CTTGGTACTTCCCGACAAGGAAACTTCCTTTGAGGAATATACCACTATGGAGCTTTTATC

CGTTGCTATGCTGATTGTCATTAGTACCTTTGCTTTAGACTTTATCCATACAGACCGAAG

CATTTTCAAGATTGTAGATTTAGACGAAGCATGGAGCTTTTTACAGGTAGCACAAGGAAA

AACACTATCTATGAAGCTGGTTCGGGCTGGTCGTGCTATGAACGCTGGGGTATATTTCGT

GACCCAAAATACAGACGACCTCTTAGATGAAAAACTGAAAAATAACCTCGGCTTAAAATT

TGCATTTCGTTCCACTGACCTTAACGAGATTAAAAAGACCTTAGCCTTTTTTGGTGTAGA

CCCAGAGGACGAAAACAATCAGAAGCGATTGCGTGATTTGGAAAACGGGCAATGCCTTAT

CAGTGATTTATATGGTCGTGTCGGTGTGATACAGTTCCACCCTGTATTTGAAGAACTGCT

CCATGCCTTTGATACCAGACCACCTGTGCGAAAAGAGGTGTAAATGTGAAACCATCAATA

GTAAACAGAATAAAATCAAACTGGACGCTGAAACGTCTAGGTAAAGTGGCAATGACAGTG

GCTTTCACACTTGTGATTGCCATTTTTCTTTTAGCCATGCTGGGAACGGTGGTTCAAGCT

GCGGGCTTGGTAGATGATACGGTCAATGTGGCAAATGAATACAGCCGATACCCACTTGAA

AACTATCAACTGGATTTTTATGTGGATAATAGCTGGGGCTGGCTTCCGTGGAACTGGTCG

GACGGGATTGGAAAACAGGTCATGTATGGACTATATGCCATTACCAATTTTATTTGGACA

ATCAGTTTGTATGTTTCCAATGCGACAGGTTACTTAGTACAGGAAGCCTATTCCTTAGAC

TTCATTTCCGCTACAGCAGATTCCATTGGTAAGAATATGCAGACCTTAGCTGGTGTGAGT

GCAAACGGATTTTCAACAGAGGGTTTCTATGTTGGATTCCTCTTACTCTTGATTTTGGTT

CTTGGGGTTTATGTTGCCTATACGGGACTGATAAAGAGAGAAACCACAAAGGCAATTCAT

GCCATTATGAATTTTGTGCTGGTGTTTATCCTATCGGCTTCCTTTATTGCCTACGCTCCC

GACTACATTAAAAAAATCAATGACTTTTCATCAGACATCAGTAATGCCAGTTTATCACTT

GGCACGAAGATTGTCATGCCCCATTCCGATAGTCAAGGCAAGGACAGCGTGGACTTAATC

AGAGATAGCCTGTTTTCCATACAGGTTCAGCAACCGTGGCTACTGCTTCAATACAACAGT

TCAGACATTGAAAGTATCGGTATTGACCGTGTGGAAAGCCTGCTCTCCACCAGCCCAGAT

TCCAACAATGGCGAAGACAGAGAAAAAATTGTTGCGGAAGAAATTGAAGACAGAAGCAAT

ACCAATCTAACCATTACAAAGACCATTAACCGTTTAGGTACAGTCTTCTTCCTATTTGTC

TTCAATATTGGGATTTCCATATTTGTATTCCTATTAACAGGAATCATGATTTTCTCGCAG

GTACTTTTTATCATCTATGCTATGTTTCTGCCTGTGAGCTTTATTTTAAGCATGATTCCA

TCATTTGATGGTATGTCAAAACGAGCCATAACAAAGCTCTTTAATACCATTTTGACACGA

GCTGGAATCACATTGATTATTACGACAGCATTTAGTATTTCAACCATGCTCTATACCTTA

TCGGCTGGTTATCCGTTCTTTTTGATTGCTTTTCTACAGATTGTGACCTTTGCAGGAATC

TACTTCAAGCTGGGCGATTTAATGAGTATGTTTTCTCTACAGAGTAACGATTCTCAAAGT

GTGGGAAGTCGTGTGATGAGAAAACCTCGTATGCTTATGCACGCTCACATGCACCGTCTA

CAGCGGAAACTTGGACGTTCCATGACTACTCTAGGGGCTGGGTCTGCCATTGTTACAGGT

AAAAAAGGACAGTCGGGTTCGGGGAGTTCTGCAAGGACACAAGCAGATCACTCCCGACCA

GACGGAAAGGAAAAATCAACACTTGGAAAACGTATCGGTCAAACCATCGGTACAGTAGCT

GATACCAAAGACAGAATGGTAGACACTGCTAGTGGTTTGAAAGAACAGGTTAAAGATTTG

CCGACCAATGCAAGATATGCAGTATATCAAGGAAAATCCAAAGTAAAAGAGAATGTCCGT

GATTTAACCAGTAGTATTTCTCAAACCAAAGCGGACAGAGCCAGTGGACGCAAGGAACAG

CAGGAACAAAGGCGAAAAACCATTGCGAAGCGTCGCTCTGAAATGGAACAGGTCAAACAG

AAAAAACAGCCTGCTTCTTCTGTTCATGAAAGACCGACTACAAGACAAGAACAATATCAT

GATGAACAGACCTCAAAACAGTCTAATATTCAGACTTCATATAAGGAATCTCAACAAGCC

AAACAAGAGCGTCCAGCAGTTAAGTCCGATTTTTCAAGTCCAAAAGTGGAACGCCAAGGC

AATACCGTTCAAGAAAAAACCGTTCAAAAGCCAGCAACTTCAACCACTACAGCAGATAGA

ACTTCACAACGTCCAATCACAAAAGAACGTCCGTCTACTGTTCAAAGAGTACCACTACAA

AATACAAGAAGTAGACCACCAATCAAAACCGCCACCATTAAGAAAGTCGGTAAGAAACCA

TGAAGTTGAAAACTTTAGTGATTGGTGGTTCTGGATTATTCTTGATGGTCTTCTCACTGC

TTCTGTTTGTTGCCATTTTATTTTCAGATGAACAGGACAGCGGAATTTCCAATATTCATT

ATGGAGGTGTGAATGTTTCCGCAGAAGTGCTGGCTCATAAGCCTATGGTAGAAAAATATG

CCAAAGAATATGGCGTTGAAGAATATGTCAACATACTTCTTGCGATTATACAGGTGGAAT

CGGGCGGTACTGCGGAAGATGTTATGCAGTCCTCGGAATCCCTCGGTCTTCCACCTAATT

CATTGAGTACAGAAGAATCCATTAAGCAAGGTGTGAAGTATTTCAGTGAATTATTAGCCA

GTAGCGAAAGGCTCAGTGTAGATTTAGAATCGGTTATCCAGTCCTACAATTATGGTGGTG

GTTTCTTAGGGTATGTGGCTAATCGTGGAAATAAATATACCTTTGAACTGGCTCAAAGTT

TCTCAAAAGAGTATTCAGGTGGCGAAAAAGTGTCTTACCCCAATCCCATAGCCATACCTA

TCAATGGGGGCTGGCGATACAACTATGGCAATATGTTTTATGTGCAACTGGTAACGCAGT

ATCTTGTCACAACAGAGTTTGATGATGATACGGTACAAGCCATCATGGACGAAGCACTGA

AATATGAGGGCTGGCGATACGTTTACGGTGGAGCTTCCCCGACTACTTCTTTTGATTGTA

GCGGACTGACACAATGGACGTATGGAAAAGCTGGAATTAACTTACCACGAACCGCACAAC

AGCAATATGATGTGACCCAGCATATCCCACTATCGGAAGCACAAGCTGGCGATTTGGTTT

TCTTTCATTCTACCTATAACGCTGGCTCTTATATTACTCATGTTGGGATATACCTTGGCA

ATAACCGTATGTTTCATGCAGGCGACCCAATCGGTTATGCCGACTTAACAAGCCCCTACT

GGCAACAGCATTTAGTGGGAGCAGGACGAATCAAACAATGAGAAAGGAAGATTTAATGAT

GAAATTTAGAAAAAATCAGAATAAAGAAAAACAGATACCAAAGGAAAAGAAACCTCGTGT

CTACTATAAGGTCAATCCTCATAAAAAGGTTGTGATTGCCTTGTGGGTACTTTTAGGGCT

TAGTTTCAGCTTTGCGATATTCAAGCACTTTACAGCTATAGATACTCATACTATTCACGA

AACAACTATCATAGAAAAGGAATACGTTGATACTCATCATGTAGAAAATTTTGTAGAGAA

CTTTGCGAAAGTCTACTATTCATGGGAGCAATCCGATAAGTCCATTGATAATCGAATGGA

AAGTCTAAAAGGCTATCTGACAGATGAACTTCAAGCTCTCAATGTTGATACAGTACGCAA

AGATATTCCTGTATCGTCTTCTGTAAGAGGATTTCAGATATGGACGGTAGAGCCAACTGG

CGACAATGAGTTTAATGTAACCTACAGTGTAGACCAGCTCATTACAGAGGGAGAAAATAC

AAAGACCGTCCACTCTGCTTATATAGTGAGTGTCTATGTAGATGGTTCTGGAAATATGGT

ACTGGTTAAGAATCCGACCATTACCAACATACCTAAGAAATCAAGTTATAAACCAAAAGC

CATTGAAAGTGAGGGGACGGTTGATTCCATTACAACCAATGAAATCAATGAGTTTTTAAC

GACGTTCTTCAAGCTCTATCCTACAGCGACAGCCAGTGAACTTTCCTACTATGTGAATGA

CGGGATATTAAAACCAATCGGAAAAGAGTACATCTTTCAAGAACTGGTAAATCCTATTCA

CAATCGTAAGGATAATCAAGTCACGGTATCGCTGACAGTGGAGTATATCGACCAGCAGAC

CAAAGCAACGCAGGTATCTCAATTTGATTTGGTACTTGAAAAGAACGGGAGTAATTGGAA

GATTATAGAATAACAAATATTGGTACATTATTACAGCTATTTTGTAATCACGTACTCTCT

TTGATAAAAAATTGGAGATTCCTTGACAAATATGCTCTTACGTGCTATTATTTAAGTATC

TATTTAAAAGGAGTTAATAAATATGCGGCAAGGTATTCTTAAATAAACTGTCAATTTGAT

AGTGGGAACAAATAATTGGATGTCCTTTTTTAGGAGGGCTTAGTTTTTTGTACCCAGTTT

AAGAATACCTTTATCATGTGATTCTAAAGTATCCGGAGAATATCTGTATGCTTTGTATGC

CTATGGTTATGCATAAAAANNNNNCACACACTTAATTAATTAAGTGTGTGNNNNNATGCC

CTTTTGGGCTTTTGAATGGAGGAAAATCACATGAAAATTATTAATATTGGAGTTTTAGCT

CATGTTGATGCGGGAAAAACTACCTTAACAGAAAGCTTATTATATAACAGTGGAGCGATT

ACAGAATTAGGAAGCGTGGACAAAGGTACAACGAGGACGGATAATACGCTTTTAGAACGT

CAGAGAGGAATTACAATTCAGACAGGAATAACCTCTTTTCAGTGGGAAAATACGAAGGTG

AACATCATAGACACGCCAGGACATATGGATTTCTTAGCAGAAGTATATCGTTCATTATCA

GTTTTAGATGGGGCAATTCTACTGATTTCTGCAAAAGATGGCGTACAAGCACAAACTCGT

ATATTATTTCATGCACTTAGGAAAATGGGGATTCCCACAATCTTTTTTATCAATAAGATT

GACCAAAATGGAATTGATTTATCAACGGTTTATCAGGATATTAAAGAGAAACTTTCTGCC

GAAATTGTAATCAAACAGAAGGTAGAACTGTATCCTAATATGTGTGTGACGAACTTTACC

GAATCTGAACAATGGGATACGGTAATAGAGGGAAACGATGACCTTTTAGAGAAATATATG

TCCGGTAAATCATTAGAAGCATTGGAACTCGAACAAGAGGAAAGCATAAGATTTCAGAAT

TGTTCTCTGTTCCCTCTTTATCATGGAAGTGCAAAAAGTAATATAGGGATTGATAACCTT

ATAGAAGTTATTACTAATAAATTTTATTCATCAACACATCGAGGTCCGTCTGAACTTTGC

GGAAATGTTTTCAAAATTGAGTATTCGGAAAAAAGACAGCGTCTTGCATATATACGTCTT

TATAGTGGCGTACTGCATTTGCGAGATTCGGTTAGAATATCGGAAAAGGAAAAATAAAAA

TTACAGAAATGTATACTTCAATAAATGGTGAATTATGTAAAATCGATAAGGCTTATTCCG

GGGAAATTGTTATTTTGCAGAATGAGTTTTTGAAGTTAAATAGTGTTCTTGGAGATACAA

AGCTATTGCCACAGAGAGAGAGAATTGAAAATCCCCTCCCTCTGCTGCAAACGACTGTTG

AACCGAGCAAACCTCAACAAAGGGAAATGTTACTTGATGCACTTTTAGAAATCTCCGACA

GTGACCCGCTTCTGCGATATTATGTGGATTCTGCGACACATGAAATCATACTTTCTTTCT

TAGGGAAAGTACAAATGGAAGTGACTTGTGCTCTGCTGCAAGAAAAGTATCATGTGGAGA

TAGAAATAAAAGAGCCTACAGTCATTTATATGGAAAGACCGTTAAAAAAAGCAGAGTATA

CCATTCACATCGAAGTTCCACCGAATCCTTTCTGGGCTTCCATTGGTCTATCTGTAGCAC

AGCTTCCATTAGGGAGCGGAGTACAGTATGAGAGCTCGGTTTCTCTTGGATACTTAAATC

AATCGTTTCAAAATGCAGTTATGGAGGGGATACGCTATGGCTGTGAACAAGGATTGTATG

GTTGGAATGTGACGGACTGTAAAATCTGTTTTAAGTATGGCTTATACTATAGCCCTGTTA

GTACCCCAGCAGATTTTCGGATGCTTGCTCCTATTGTATTGGAACAAGTCTTAAAAAAAG

CTGGAACAGAATTGTTAGAGCCATATCTTAGTTTTAAAATTTATGCGCCACAGGAATATC

TTTCACGAGCATACAACGATGCTCCTAAATATTGTGCGAACATCGTAGACACTCAATTGA

AAAATAATGAGGTCATTCTTAGTGGAGAAATCCCTGCTCGGTGTATTCAAGAATATCGTA

GTGATTTAACTTTCTTTACAAATGGACGTAGTGTTTGTTTAACAGAGTTAAAAGGGTACC

ATGTTACTACCGGTGAACCTGTTTGCCAGCCCCGTCGTCCAAATAGTCGGATAGATAAAG

TACGATATATGTTCAATAAAATAACTTAGTGTATTTTATGTTGTTATATAAATATGGTTT

CTTGTTAAATAAGATGAAATATTTTTTAATAAAGATTTGAATTAAAGTGTAAAGGAGGAG

ATAGTTATTATAAACTACAAGTGGATATTGTGTCCTGTATGTGGAAATAAAACACGATTA

AAGATAAGGGAAGATACTGAATTAAAAAAATTCCCCCTCTATTGTCCGAAATGCAGACAA

GAAAATTTAATTGAAATAAAGCAGTTCAAAGTAACTGTGATTACAGAGCCAGACGCAAAG

ACGCAGAGCCGATAAAATGAGATTAATACAATCTCATTTTATCGGCTCTTTCCGTTATGT

ATGGATTCTTTTAATTAGTCTTCGATGTTTCTTGCTTCGTTGATACCGCTGGCTAAAGAT

TCCATTAAGGATAGTTCTTTGTCTGTAAAGCTATCCATGTATTTCTCTATCTGTAATCGT

CGGGTGCTTTTTACCAAGTTATTAGCAGGTAAGAAAAATTCATCAACGGAAACATGAAGT

AACGATACAAGGTCATAAAGAACTTGTATGCTGGGGTGTTGCCCTTTATTTTCAATATTA

GTTAAGTACCGTGGGTCAATTTCAATCAATGCTCCCACTTGTTCACGAGTTAAACCTCGT

TTCAATCGAGCTTCTTTAATGGCTAAACCAAAGGCTCTAAAATCATATTTATCTTCTTTT

TTACGCATAGTAGACCACCTCTATACATTTTATTGTCCCTACTGAATTAAAAACAGGTAT

AGAAAAACGTGTTATATGGTTTATAGGTTTATATTTAATAAAAAGCACTACTAAACGCCA

ATAAAAAAAACCGTTATATGGTAGTGCTATTTACGCTGTTAAAATATTGTATATTACTTC

CAAATGGCGGTTTGTTGGAGGTCAACGTCGCCATGAAGTACATCATATACAATAAATTTC

CTTACATTGGGTTCTTGTCAAAAAAAGTCGTCTATCTGCAATAGATAAGTACGTCCACCA

ATGTGGTTTTATAAATCATATAGATAGAATAACAGAAGCATGTAAACAGAGAAATAAATC

TGTTTATATGCTTTTTTGGCTATTCAGAACTTTTTTACAAAGTTTATTTATCAGTAATGC

AACAAATCCCCCTTTCACATTGGGACTAAGAGTGAAAGGAGATAAACGAGCAAGGCTCAC

TTCCTTTCCTAGACAGAAAGGGGGTGAGAAACATGAAACCATCTTCTTTTCAGACCACAA

TAGAAAATCAGTTTGACTATATCTGTAAACGTGCTATGGAAGACGAGCGAAAGAATTATA

TGCTTTATCTTTCAAGGATTGCAAAGCGTGAGGTGTCCTTTTCGGATGTTGGCGATTATC

TTGTTAGCCAGTTTGCGACAACAGATAACTATTCAACTGACTTTCAGATTTTTACACTCA

ATGGGTTATCAGTAGGCGTTGAAAATGATTTGTTGAGTGAAGCATTACGTGAGTTGCCAG

ACAAGAAACGTGAAATTCTACTGCTGTTTTACTTTATGGACATGAGCGATTCAGAAATTG

CAGACCTGTTGAAATTGAACCGTTCTACTGTCTATCGGCATAGAACCAGTGGACTAGCCT

TAATTAAAAAGTTTATGGAGGAATTTGAAGAATGAAAACACAATATCCTATGATTCCCTT

TCCTCTCATTGTAAAGGCAACAGATGGCGATACCGAAGCGATTAACCAGATTCTACATCA

TTACAGAGGGTACATAACGAAGCGTTCCCTACGACTTATGAAAGATGAATATGGCAATCA

AAGTATGGTCGTTGATGAAGTCTTACGTGGAAGAATGGAAACCAGACTGATTACAAAGAT

TTTGTCATTTGAAATTAAGTAATATCCTCTCTCCTTTCGTGGAAGCGTGCTAAACCATTC

CACGCTTCCCGAACAGGGAGGTTTGTTATTCCACCAAAGCATATTGAGCTTTCAATGTGT

TTTGATAGGCTAACGAGCCATTGTTCTTTGAAAACTGAATAAAAGTAATCGAATACGTTT

CGATAAGAAAAGAGCCAACGGAACTAACCGCCATGACCTATCTTATAAAGATAGCGAGCG

ATTCATGTTAGTGATCCGAGAAGCAATCTTTAGCAGGATTGCCTGCAACGACATTCTTAT

CGTGATAATGATACTCCCATACAGTCAATAGTCCGAGCGTGATAAAACCGTCGCAGGCAA

TGAGTATGGCTACATGAGAACCATGCAGGGGTGGAACTCCCGTGAGCTTTGCTAAAGCTG

TTCGATTGCTGGTAAAACAACTTTTATGAAATCCAAATAAGTGATTTGGAAAGGAGGATT

TTATGAAGCAGACTGACATTCCTATTTGGGAACGTTATACCCTAACCATTGAAGAAGCGT

CAAAATATTTTCGTATTGGCGAAAACAAGCTACGACGCTTGGCAGAGGAAAATAAAAATG

CAAATTGGCTGATTATGAATGGCAATCGTATTCAGATTAAACGAAAACAATTTGAAAAAA

TTATAGATACATTGGACGCAATCTAGCGTCGCCAAAGGGTCTTGTATATGATAAAATAGT

ATTAAGTCGTATCAAGGCTCTTTCCATAAAGGAAAGGAGCAAATGCCATGTCAGAAAAAA

GACGTGACAATAAAGGTCGAATCTTAAAGACTGGAGAGAGCCAACGAAAAGACGGAAGAT

ACTTATACAAATATATAGATTCATTTGGAGAACCGCAATTTGTTTACTCGTGGAAACTTG

TGGCTACAGACCTAGTACCAGCAGGAAAGCGTGATTGTATCTCACTTAGAGAGAAAATCG

CAGAGTTACAGAAAGACATTCATGATGGTATTGATGTTGTAGGAAAGAAAATGACACTCT

GCCAGCTTTACGCAAAACAGAACGCTCAAAGACCAAAGGTTAGAAAAAACACTGAAACTG

GACGCAAATATCTTATGGATATTTTGAAGAAAGACAAGTTAGGTGTAAGAAGTATTGACA

GTATTAAGCCATCAGACGCTAAAGAATGGGCTATTAGAATGAGTGAAAATGGTTATGCTT

ATCAAACCATCAATAACTACAAACGTTCTTTAAAGGCTTCATTCTATATTGCTATACAAG

ATGATTGTGTTCGGAAGAATCCATTTGACTTTCAACTGAAAGCAGTTCTTGATGATGATA

CTGTCCCTAAGACCGTACTAACAGAAGAACAGGAAGAAAAACTGTTAGCCTTTGCAAAAG

CTGATAAAACCTACAGCAAAAATTATGATGAAATTCTGATACTCTTAAAAACAGGTCTTC

GTATTTCAGAGTTTGGTGGTTTGACACTTCCAGATTTAGATTTTGAGAATCGTCTTGTCA

ATATAGACCATCAGCTATTGAGAGATACTGAAATTGGGTACTACATTGAAACACCAAAGA

CCAAAAGTGGCGAACGTCAAGTTCCTATGGTTGAAGAAGCCTATCAAGCATTTAAGCGAG

TGTTAGCGAATCGAAAGAATGATAAGCGTGTTGAGATTGATGGATATAGTGATTTCCTCT

TTCTTAATAGAAAGAACTATCCAAAAGTGGCAAGTGATTACAACGGCATGATGAAAGGTC

TTGTTAAGAAATACAATAAGTATAACGAGGATAAATTGCCACACATCACTCCACATAGTT

TGCGACATACATTCTGTACCAACTATGCAAATGCAGGAATGAATCCAAAGGCATTACAGT

ACATTATGGGACATGCTAATATAGCCATGACGCTGAACTATTACGCACATGCAACATTCG

ATTCTGCAATGGCAGAAATGAAACGCTTGAATAAAGAGAAGCAACAGGAGCGTCTTGTTG

CTTAGTAGTACAAATGAATTTACTACTTATTTACCACTTCTGACAGCTAAGACATGAGGA

AATATGCAAAGAAACGTGAAGTATCTTCCTACAGTAAAAATACTCGAAAGCACATAGAAT

AAGGCTTTACGAGCATTTAAGAAAATATAAAAAGATAATTAGAAATTTATACTTTGTTT

>GA49542/Tn2009

AAAATAGCATAAAAATCTAGTTATCCGCATAAAAACTGGACTTATCACACTTTATCAAGG

TCAAAACCACTCAATTTACTACTAATTTACTACTTATGAATGAGCTTTGATACGACGATT

TATCCTTGAAAAGTGAAGATATAAAGATACTTCCAATAAAATTTGAATATTTAATAGGTA

GACACTTCAAAAAATGAGGTGTCTATTTTTTTACCCGATTTTGAAAGGAAGTGAACTTAT

GAAAACAAAAAATCAAGAATCAAAAGGTCGTTCCCCACTCTTTAAGACCATCAAACATTC

ATTCAGCCAATAAAAAAGAAAGGATAGGTAAAAATATGGAACTTAAATTTGTGATTCCCA

ACATGGAAAAAACATTCGGCAATTTAGAATTTGCTGGCGAGGATAAAGTCGTTCAGCGAA

GAATCAACGGACGGCTAACTGTCTTATCAAGAAGCTATAATCTCTATTCTGATGTTCAAA

GAGCAGATGATATTGTGGTGGTGCTTCCTGCTGAAGCTGGCGAAAAACATTTCGGCTTTG

AGGAACGTGTGAAGTTAGTCAATCCACGTATTACCGCAGAGGGCTACAAAATCGGCACTC

GTGGTTTTACAAATTACCTTTTACATGCTGACGACATGATAAAAGAATAAAGAAAGAGAG

GAAAAATGATGAGATTAGCAAATGGCATTGTATTAGATAAAGACACGACTTTTGGAGAAT

TGAAATTCTCTGCTCTACGTCGTGAAGTGAGAATCCAAAATGAAGACGGGTCGGTTTCAG

ATGAAATCAAGGAACGTACCTATGACTTAAAATCCAAAGGACAAGGACGCATGATTCAAG

TAAGTATTCCTGCCAGCGTGCCTTTGAAAGAGTTTGATTATAACGCACGGGTGGAACTTA

TCAATCCCATTGCGGACACCGTTGCTACTGCCACCTATCAAGGAGCAGATGTTGACTGGT

ATATCAAGGCAGACGATATTGTGCTGACAAAGGATTCTAGTTCATTCAAAGCTCAACCAC

AAGCAAAGAAAGAACCGACACAAGACAAATAGTCGCTAGGTAGAAAGGAGACTTTTTCGC

ATGAAACAGCGTGGTAAAAGGATTCGCCCATCTGGTAAAGATTTAGTCTTTCATTTTACG

ATAGCGTCACTCCTGCCTGTTTTCCTGCTGGTTGTCGGACTGTTTCATGTGAAGACAATC

CAGCAGATCAACTGGCAGGATTTTAACCTATCACAAGCAGATAAGATTGACATTCCCTAT

TTAATTATCAGTTTCAGTGTCGCAATTCTTATCTGCTTGCTGGTAGCGTTTGTATTCAAA

CGGGTTCGCTATGATACGGTTAAACAACTTTACCACCGTCAAAAACTGGCAAAGATGATA

CTTGAAAACAAGTGGTATGAATCTGAACAGGTCAAAACAGAGGGTTTCTTTAAAGATAGT

GCTGGTCGTACAAAGGAAAAGATAACCTACTTCCCTAAAATGTATTATCGACTTAAAAAT

GGCTTGATACAGATACGGGTGGAAATCACGCTGGGAAAATATCAAGACCAACTCTTACAC

TTGGAAAAGAAATTAGAGAGTGGCTTGTACTGTGAGCTGACGGATAAAGAGTTAAAGGAT

TCCTATGTGGAATATACTTTGCTCTATGACACCATAGCCAGTCGTATTTCTATTGATGAA

GTAGAAGCTAAAGATGGTAAACTTCGCTTAATGAAAAACGTATGGTGGGAATATGATAAG

CTCCCTCATATGTTGATTGCTGGTGGTACAGGTGGCGGTAAAACTTACTTTATACTGACA

CTGATTGAAGCCTTGCTTCATACAGATTCAAAACTGTATATTCTTGACCCGAAAAATGCT

GACCTTGCGGACTTAGGTTCTGTGATGGCAAATGTCTACTATAGAAAAGAAGACTTGCTT

TCTTGCATTGAAACATTCTATGAAGAAATGATGAAACGTAGTGAGGAAATGAAGCAGATG

AAGAACTATAAGACTGGCAAAAATTATGCTTACTTAGGTCTCCCGGCACACTTCTTAATC

TTTGATGAATACGTCGCTTTCATGGAAATGCTGGGAACAAAAGAAAACACCGCAGTTATG

AATAAGCTGAAACAGATTGTCATGTTAGGTCGTCAAGCTGGCTTCTTTCTAATACTGGCT

TGTCAACGTCCAGACGCAAAATATTTAGGCGACGGAATCCGTGATCAGTTTAATTTCAGA

GTGGCTTTAGGTCGTATGTCTGAAATGGGCTATGGCATGATGTTTGGCAGTGACGTACAA

AAGGATTTCTTCTTAAAGCGAATCAAAGGTCGTGGCTATGTTGATGTAGGAACAAGTGTC

ATATCAGAGTTTTATACTCCCCTTGTACCAAAAGGATATGATTTCTTGGAGGAAATTAAA

AAGTTATCCAACAGCAGACAGTCCACGCAGGCGACGTGCGAAGCGGAAGTCGCAGGTGTG

GACTGATCTTGCTGGCTGGTGTGGCAATAGCCACGCCAGCACTTAACCCCCCGTATCTAA

CAGGGGGGTACAAATCGACAGGAAACAGTCAAAAAAACATTAGAAAATCCTTTGGTTACA

AGGGATTTACAAAATTTCAGCGTATGTCAAATGGGCTTTAAAAGTTGACATACGCCTTTT

TGATTGGAGGGATTTTTACTGAATGAACAAACTTGGTTACAGCATTTAAAAGAAAAACGC

TTGGCTTATGGACTATCTCAAAACCGTTTAGCTGTTGCGACTGGTATTACAAGGCAGTAT

CTAAGCGATATTGAAACAGGAAAAGTCAAGCCATCAGAGGATTTACAGCAGTCCCTTTGG

GAAGCTCTGGAACGCTTCAATCCCGACGCTCCCCTTGAAATGCTGTTTGATTATGTAAGG

ATTCGCTTTCCGACAACAGACGTACAGCAGGTGGTCGAAAACATCTTACAACTGAAACTG

TCCTATTTTCTTCATGAGGACTATGGTTTCTATTCTTATTCAGAGCATTATGCTTTAGGC

GACATATTCGTCCTTTGCTCCCATGAACTGGACAAAGGAGTTCTGGTGGAATTGAAAGGT

CGTGGGTGCAGACAATTTGAAAGCTATCTTCTGGCACAACAAAGAAGCTGGTATGAGTTC

TTTATGGACGTTTTGGTGGCTGGCGGTGTGATGAAACGCCTTGACCTTGCCATTAACGAT

AAGACAGGGATTTTGAATATCCCTGTACTCACTGAAAAGTGCCAACAGGAAGAATGTATC

TCCGTCTTCCGCAGTTTTAAAAGCTATCGCAGTGGCGAACTGGTACGCAAAGAGGAAAAG

GAATGTATGGGAAACACCCTCTATATCGGTTCATTACAAAGTGAAGTTTATTTCTGTATC

TATGAAAAGGACTACGAGCAGTACAAGAAAAATGATATTCCCATTGAAGACGCAGAAGTA

AAAAACCGTTTTGAGATTCGATTGAAAAATGAGCGTGCCTATTATGCAGTCCGTGATTTA

CTCGTCTATGACAATCCAGAGCATACCGCCTTTAAAATTATCAATCGGTATATCCGTTTT

GTAGATAAAGACGATTCCAAACCTCGTTCTGATTGGAAACTGAATGAAGAATGGGCTTGG

TTTATTGGGAACAATCGTGAACGATTAAAACTAACCACAAAACCAGAGCCTTACTCCTTC

CAAAGGACGCTGAACTGGCTATCTCATCAAGTTGCCCCGACCTTAAAGGTTGCGATTAAA

CTTGATGAAATCAACCAGACGCAGGTTGTAAAAGACATTCTCGACCATGCGAAACTGACA

GACCGACACAAGCAGATTTTGAAGCAACAGTCAGTAAAAGAACAGGACGTGATAACAACA

AAAAAATAACTCAAATACAAATTCATTGAATATAGAGAGGAGAACATTTTTATGAATTTT

GGACAAAACCTTTATAACTGGTTTCTATCAAACGCTCAATCACTGGTGCTTTTAGCAATC

GTTGTGATTGGCTTGTATCTTGGCTTCAAGCGTGAGTTTAGCAAACTGATTGGCTTTTTA

ATTATTGCGATTATTGCGGTTGGCTTAGTCTTCAACGCTGCTGGAGTAAAAGACATTTTA

CTAGAGCTATTCAATCGCATTATTGGTGCTTAAATAAAACCGTTCTTTTGTGGAATATAA

GTGGTTTTCTTATGTTCCGCAAAGGAATGGTACACCAAACGAAGTGCGGTAGGGATTTTT

GAATCTCTACAAAGAAAGGACGTGAATATATGGACGATATGCAAGTCTATATTGCGAATT

TAGGCAAATACAATGAGGGCGAATTGGTCGGTGCGTGGTTTACCTTTCCCATTGACTTTG

AGGAAGTCAAAGAGAAAATCGGCTTGAATGATGAATATGAGGAATACGCCATTCATGACT

ACGAGTTACCCTTTACGGTTGACGAATACACTTCCATTGGCGAACTCAATCGACTATGGG

AAATGGTATCGGAATTACCCGAAGAATTACAATCGGAGCTATCTGCTCTGCTCACTCATT

TTTCAAGCATTGAAGAACTAAGCGAACATCAAGAGGATATTATCATTCATTCCGATTGTG

ATGATATGTATGACGTGGCACGCTACTACATTGAAGAAACGGGTGCTTTAGGCGAAGTAC

CAGCTAGTCTTCAAAACTATATTGATTATCAAGCCTATGGTCGGGATTTAGACCTTTCAG

GAACGTTTATCTCAACCAATCATGGGATTTTTGAAATCGTCTATTAAATCTGTCGGTACA

TTACTACTGGCAGATTTTCTATTTTACGGGGTGGCTCAATCAGCTACCCCTATTTTTTAT

GAAAGGATTGATTACATGAAGAAAATACGAAGCTATACCAGTATCTGGTCTGTGGAAAAG

GTACTGTATTCTATCAATGATTTTAGACTTCCGTTTCCCATAACCTTTACGCAAATGACA

TGGTTTGTCGTGTCACTCTTTGCAGTGATGATACTTGGCAACTTGCCCCCTCTTTCCATG

ATAGAGGGAGCATTTCTCAAATACTTTGGGATTCCTGTGGCTTTCACATGGTTTATGTCT

ACAAAAACTTTTGATGGTAAAAAGCCTTATGGATTTTTGAAGTCTGTCATTGCTTATGCA

CTGCGACCAAAGCTGACCTATGCAGGAAAAAAAGTAACGCTTGGCAGAAACCAGCCACAA

GAAGCCATTACAGCAGTTAGGAGTGAATTTTATGGCATATCCAATTAAATACATTGAAAA

CAATCTCGTCTGGAATAAAGACGGGGAATGTTATGCTTACTATGAGCTTGTTCCTTACAA

TTACTCATTTCTAAGTCCAGAACAGAAAATACAAGTGCATGATTCTTTCAGACAGCTTAT

CGCACAAAATCGTGATGGCAAAATTCATGCTTTACAAATCAGTACAGAATCCAGCATACG

TTCTGCACAAGAGCGTTCCAAAAATGAAGTCACTGGCAAGCTCAAAGCGGTTGCCTATGA

CAAAATCGACCAACAGACAGACGCTTTAATATCCATGATTGGCGAAAATCAAGTGAACTA

CCGTTTCTTTATCGGCTTTAAGTTGCTTCTCAACGATCAGGAGTTTTCTATGAAAAGTCT

TACCGTTGAAGCAAAAAATGCTTTGTCTGATTTTGTCTATGATGTGAACCATAAGCTGAT

GGGCGATTTTGTTAGTATGAGTAATGATGAAATCCTGCGTTTTCAGAAGATGGAAAAGCT

CTTAGAAAATAAAATCTCTCGTCGTTTCAAAATCCGCAGGTTAGATAAGGACGACTTCGG

CTATCTGATTGAACACCTTTACGGACAGACAGGCACTGCCTATGAAGAGTATGAGTACCA

TCTATCAAAGAAAAAGCTGGATAATGAAACGCTGATTAAATACTATGACTTGATTAAGCC

TACTCGCTGTTTGGTGGAAGAAAAACAGCGATATTTGAAAATCCAGCAGGAAGATGAAAC

CGTCTATGTAGCTTACTTTACCATTAACAGCATTGTCGGAGAACTGGACTTCCCGTCCTC

TGAAATCTTCTACTACCAGCAACAGCAATTTACATTCCCGATTGATACGTCAATGAATGT

GGAAATTGTAGCGAATCGTAAAGCCCTATCTACTGTCCGCAATAAAAAGAAAGAACTGAA

AGACTTGGATAACCACGCTTGGCAAAGTGATAATGAAACCAGCTCCAATGTGGCGGAAGC

TCTGGAAAGTGTGAATGAGCTGGAAACCAATTTAGACCAAAGCAAGGAATCTATGTACAA

GCTGTCTTATGTGGTAAGGGTATCAGCAAATGATCTTGACGAACTCAAACGTCGTTGTAA

TGAAGTGAAAGATTTTTATGACGATTTAAGCGTAAAACTGGTACGACCATTTGGGGATAT

GCTCGGCTTACATGAAGAATTTTTACCTGCCAGCAAGCGTTATATGAATGATTATATTCA

ATACGTGACCTCTGATTTCCTCGCTGGTTTAGGTTTTGGTGCTACTCAAATGCTGGGGGA

AAATGAGGGGATTTATGTTGGCTACAGCTTAGATACTGGACGCAATGTCTATCTGAAACC

TGCTCTTGCCAGTCAAGGGGTTAAGGGTTCAGTAACCAATGCGTTAGCGTCGGCTTTTGT

TGGTTCGCTGGGTGGTGGTAAATCCTTTGCGAATAACCTTATCGTCTATTATGCGGTGCT

TTATGGGGCACAAGCAGTGATTGTAGACCCAAAAGCAGAACGTGGCAGATGGAAAGAAAC

CTTGCCAGAGATTTCCCATGAAATCAATATCGTCACTCTGACTTCTGATGAGAAAAACAA

AGGCTTACTTGACCCTTATGTGATTATGAAAAATCCCAAAGATTCTGAATCACTGGCTAT

TGATATTCTGACATTCCTTACGGGGATTTCCTCTCGTGATGGGGAACGCTTCCCAATCCT

TAGAAAAGCCATTCGTGCAGTAACCAATAGTGAAGTACGAGGGTTGATGAAAGTGATTGA

GGAATTACGGGTTGAGAATACGCCACTAAGTACCAGTATAGCCGACCATATCGAAAGTTT

TACAGACTATGACTTTGCACATTTATTATTCAGTAATGGTTATGTGGAGCAGTCTATCAG

CTTAGAAAAACAACTGAACATTATACAGGTTGCGGACTTGGTACTTCCCGACAAGGAAAC

TTCCTTTGAGGAATATACCACTATGGAGCTTTTATCCGTTGCTATGCTGATTGTCATTAG

TACCTTTGCTTTAGACTTTATCCATACAGACCGAAGCATTTTCAAGATTGTAGATTTAGA

CGAAGCATGGAGCTTTTTACAGGTAGCACAAGGAAAAACACTATCTATGAAGCTGGTTCG

GGCTGGTCGTGCTATGAACGCTGGGGTATATTTCGTGACCCAAAATACAGACGACCTCTT

AGATGAAAAACTGAAAAATAACCTCGGCTTAAAATTTGCATTTCGTTCCACTGACCTTAA

CGAGATTAAAAAGACCTTAGCCTTTTTTGGTGTAGACCCAGAGGACGAAAACAATCAGAA

GCGATTGCGTGATTTGGAAAACGGGCAATGCCTTATCAGTGATTTATATGGTCGTGTCGG

TGTGATACAGTTCCACCCTGTATTTGAAGAACTGCTCCATGCCTTTGATACCAGACCACC

TGTGCGAAAAGAGGTGTAAATGTGAAACCATCAATAGTAAACAGAATAAAATCAAACTGG

ACGCTGAAACGTCTAGGTAAAGTGGCAATGACAGTGGCTTTCACACTTGTGATTGCCATT

TTTCTTTTAGCCATGCTGGGAACGGTGGTTCAAGCTGCGGGCTTGGTAGATGATACGGTC

AATGTGGCAAATGAATACAGCCGATACCCACTTGAAAACTATCAACTGGATTTTTATGTG

GATAATAGCTGGGGCTGGCTTCCGTGGAACTGGTCGGACGGGATTGGAAAACAGGTCATG

TATGGACTATATGCCATTACCAATTTTATTTGGACAATCAGTTTGTATGTTTCCAATGCG

ACAGGTTACTTAGTACAGGAAGCCTATTCCTTAGACTTCATTTCCGCTACAGCAGATTCC

ATTGGTAAGAATATGCAGACCTTAGCTGGTGTGAGTGCAAACGGATTTTCAACAGAGGGT

TTCTATGTTGGATTCCTCTTACTCTTGATTTTGGTTCTTGGGGTTTATGTTGCCTATACG

GGACTGATAAAGAGAGAAACCACAAAGGCAATTCATGCCATTATGAATTTTGTGCTGGTG

TTTATCCTATCGGCTTCCTTTATTGCCTACGCTCCCGACTACATTAAAAAAATCAATGAC

TTTTCATCAGACATCAGTAATGCCAGTTTATCACTTGGCACGAAGATTGTCATGCCCCAT

TCCGATAGTCAAGGCAAGGACAGCGTGGACTTAATCAGAGATAGCCTGTTTTCCATACAG

GTTCAGCAACCGTGGCTACTGCTTCAATACAACAGTTCAGACATTGAAAGTATCGGTATT

GACCGTGTGGAAAGCCTGCTCTCCACCAGCCCAGATTCCAACAATGGCGAAGACAGAGAA

AAAATTGTTGCGGAAGAAATTGAAGACAGAAGCAATACCAATCTAACCATTACAAAGACC

ATTAACCGTTTAGGTACAGTCTTCTTCCTATTTGTCTTCAATATTGGGATTTCCATATTT

GTATTCCTATTAACAGGAATCATGATTTTCTCGCAGGTACTTTTTATCATCTATGCTATG

TTTCTGCCTGTGAGCTTTATTTTAAGCATGATTCCATCATTTGATGGTATGTCAAAACGA

GCCATAACAAAGCTCTTTAATACCATTTTGACACGAGCTGGAATCACATTGATTATTACG

ACAGCATTTAGTATTTCAACCATGCTCTATACCTTATCGGCTGGTTATCCGTTCTTTTTG

ATTGCTTTTCTACAGATTGTGACCTTTGCAGGAATCTACTTCAAGCTGGGCGATTTAATG

AGTATGTTTTCTCTACAGAGTAACGATTCTCAAAGTGTGGGAAGTCGTGTGATGAGAAAA

CCTCGTATGCTTATGCACGCTCACATGCACCGTCTACAGCGGAAACTTGGACGTTCCATG

ACTACTCTAGGGGCTGGGTCTGCCATTGTTACAGGTAAAAAAGGACAGTCGGGTTCGGGG

AGTTCTGCAAGGACACAAGCAGATCACTCCCGACCAGACGGAAAGGAAAAATCAACACTT

GGAAAACGTATCGGTCAAACCATCGGTACAGTAGCTGATACCAAAGACAGAATGGTAGAC

ACTGCTAGTGGTTTGAAAGAACAGGTTAAAGATTTGCCGACCAATGCAAGATATGCAGTA

TATCAAGGAAAATCCAAAGTAAAAGAGAATGTCCGTGATTTAACCAGTAGTATTTCTCAA

ACCAAAGCGGACAGAGCCAGTGGACGCAAGGAACAGCAGGAACAAAGGCGAAAAACCATT

GCGAAGCGTCGCTCTGAAATGGAACAGGTCAAACAGAAAAAACAGCCTGCTTCTTCTGTT

CATGAAAGACCGACTACAAGACAAGAACAATATCATGATGAACAGACCTCAAAACAGTCT

AATATTCAGACTTCATATAAGGAATCTCAACAAGCCAAACAAGAGCGTCCAGCAGTTAAG

TCCGATTTTTCAAGTCCAAAAGTGGAACGCCAAGGCAATACCGTTCAAGAAAAAACCGTT

CAAAAGCCAGCAACTTCAACCACTACAGCAGATAGAACTTCACAACGTCCAATCACAAAA

GAACGTCCGTCTACTGTTCAAAGAGTACCACTACAAAATACAAGAAGTAGACCACCAATC

AAAACCGCCACCATTAAGAAAGTCGGTAAGAAACCATGAAGTTGAAAACTTTAGTGATTG

GTGGTTCTGGATTATTCTTGATGGTCTTCTCACTGCTTCTGTTTGTTGCCATTTTATTTT

CAGATGAACAGGACAGCGGAATTTCCAATATTCATTATGGAGGTGTGAATGTTTCCGCAG

AAGTGCTGGCTCATAAGCCTATGGTAGAAAAATATGCCAAAGAATATGGCGTTGAAGAAT

ATGTCAACATACTTCTTGCGATTATACAGGTGGAATCGGGCGGTACTGCGGAAGATGTTA

TGCAGTCCTCGGAATCCCTCGGTCTTCCACCTAATTCATTGAGTACAGAAGAATCCATTA

AGCAAGGTGTGAAGTATTTCAGTGAATTATTAGCCAGTAGCGAAAGGCTCAGTGTAGATT

TAGAATCGGTTATCCAGTCCTACAATTATGGTGGTGGTTTCTTAGGGTATGTGGCTAATC

GTGGAAATAAATATACCTTTGAACTGGCTCAAAGTTTCTCAAAAGAGTATTCAGGTGGCG

AAAAAGTGTCTTACCCCAATCCCATAGCCATACCTATCAATGGGGGCTGGCGATACAACT

ATGGCAATATGTTTTATGTGCAACTGGTAACGCAGTATCTTGTCACAACAGAGTTTGATG

ATGATACGGTACAAGCCATCATGGACGAAGCACTGAAATATGAGGGCTGGCGATACGTTT

ACGGTGGAGCTTCCCCGACTACTTCTTTTGATTGTAGCGGACTGACACAATGGACGTATG

GAAAAGCTGGAATTAACTTACCACGAACCGCACAACAGCAATATGATGTGACCCAGCATA

TCCCACTATCGGAAGCACAAGCTGGCGATTTGGTTTTCTTTCATTCTACCTATAACGCTG

GCTCTTATATTACTCATGTTGGGATATACCTTGGCAATAACCGTATGTTTCATGCAGGCG

ACCCAATCGGTTATGCCGACTTAACAAGCCCCTACTGGCAACAGCATTTAGTGGGAGCAG

GACGAATCAAACAATGAGAAAGGAAGATTTAATGATGAAATTTAGAAAAAATCAGAATAA

AGAAAAACAGATACCAAAGGAAAAGAAACCTCGTGTCTACTATAAGGTCAATCCTCATAA

AAAGGTTGTGATTGCCTTGTGGGTACTTTTAGGGCTTAGTTTCAGCTTTGCGATATTCAA

GCACTTTACAGCTATAGATACTCATACTATTCACGAAACAACTATCATAGAAAAGGAATA

CGTTGATACTCATCATGTAGAAAATTTTGTAGAGAACTTTGCGAAAGTCTACTATTCATG

GGAGCAATCCGATAAGTCCATTGATAATCGAATGGAAAGTCTAAAAGGCTATCTGACAGA

TGAACTTCAAGCTCTCAATGTTGATACAGTACGCAAAGATATTCCTGTATCGTCTTCTGT

AAGAGGATTTCAGATATGGACGGTAGAGCCAACTGGCGACAATGAGTTTAATGTAACCTA

CAGTGTAGACCAGCTCATTACAGAGGGAGAAAATACAAAGACCGTCCACTCTGCTTATAT

AGTGAGTGTCTATGTAGATGGTTCTGGAAATATGGTACTGGTTAAGAATCCGACCATTAC

CAACATACCTAAGAAATCAAGTTATAAACCAAAAGCCATTGAAAGTGAGGGGACGGTTGA

TTCCATTACAACCAATGAAATCAATGAGTTTTTAACGACGTTCTTCAAGCTCTATCCTAC

AGCGACAGCCAGTGAACTTTCCTACTATGTGAATGACGGGATATTAAAACCAATCGGAAA

AGAGTACATCTTTCAAGAACTGGTAAATCCTATTCACAATCGTAAGGATAATCAAGTCAC

GGTATCGCTGACAGTGGAGTATATCGACCAGCAGACCAAAGCAACGCAGGTATCTCAATT

TGATTTGGTACTTGAAAAGAACGGGAGTAATTGGAAGATTATAGAATAACAAATATTGGT

ACATTATTACAGCTATTTTGTAATCACGTACTCTCTTTGATAAAAAATTGGAGATTCCTT

TACAAATATGCTCTTACGTGCTATTATTTAAGTATCTATTTAAAAGGAGTTAATAAATAT

GCGGCAAGGTATTATTAAATAAACTGTCAATTTGATAGTGGGAACAAATAATTGGATGTC

CTTTTTTAGGAGGGCTTAGTTTTTTGTACCCAGTTTAAGAATACCTTTATCATGTGATTC

TAAAGTATCCAGAGAATATCTGTATGCTTTGTATACCTATGGTTATGCATAAAAAATCCC

AGTGATAAANNNNNCACACACTTAATTAATTAAGTGTGTGNNNNNGATAAAAGTATTTAT

CACTGGGATTTTTATGCCCTTTTGGGTTTTTGAATGGAGGAAAATCACATGAAAATTATT

AATATTGGAGTTTTAGCTCATGTTGATGCAGGAAAAACTACCTTAACAGAAAGCTTATTA

TATAACAGTGGAGCGATTACAGAATTAGGAAGCGTGGACAAAGGTACAACGAGGACGGAT

AATACGCTTTTAGAACGTCAGAGAGGAATTACAATTCAGACAGGAATAACCTCTTTTCAG

TGGGAAAATACGAAGGTGAACATCATAGACACGCCAGGACATATGGATTTCTTAGCAGAA

GTATATCGTTCATTATCAGTTTTAGATGGGGCAATTCTACTGATTTCTGCAAAAGATGGC

GTACAAGCACAAACTCGTATATTATTTCATGCACTTAGGAAAATGGGGATTCCCACAATC

TTTTTTATCAATAAGATTGACCAAAATGGAATTGATTTATCAACGGTTTATCAGGATATT

AAAGAGAAACTTTCTGCCGAAATTGTAATCAAACAGAAGGTAGAACTGTATCCTAATATG

TGTGTGACGAACTTTACCGAATCTGAACAATGGGATACGGTAATAGAGGGAAACGATGAC

CTTTTAGAGAAATATATGTCCGGTAAATCATTAGAAGCATTGGAACTCGAACAAGAGGAA

AGCATAAGATTTCAGAATTGTTCTCTGTTCCCTCTTTATCATGGAAGTGCAAAAAGTAAT

ATAGGGATTGATAACCTTATAGAAGTTATTACTAATAAATTTTATTCATCAACACATCGA

GGTCCGTCTGAACTTTGCGGAAATGTTTTCAAAATTGAATATACAAAAAAAAGACAACGT

CTTGCATATATACGCCTTTATAGTGGAGTACTACATTTACGAGATTCGGTTAGAGTATCA

GAAAAAGAAAAATAAAAGTTACAGAAATGTATACTTCAATAAATGGTGAATTATGTAAGA

TTGATAGAGCTTATTCTGGAGAAATTGTTATTTTGCAAAATGAGTTTTTGAAGTTAAATA

GTGTTCTTGGAGATACAAAACTATTGCCACAGAGAAAAAAGATTGAAAATCCGCACCCTC

TACTACAAACAACTGTTGAACCGAGTAAACCTGAACAGAGAGAAATGTTGCTTGATGCCC

TTTTGGAAATCTCAGATAGTGATCCGCTTCTACGATATTACGTGGATTCTACGACACATG

AAATTATACTTTCTTTCTTAGGGAAAGTACAAATGGAAGTGATTAGTGCACTGTTGCAAG

AAAAGTATCATGTGGAGATAGAACTAAAAGAGCCTACAGTCATTTATATGGAGAGACCGT

TAAAAAATGCAGAATATACCATTCACATCGAAGTGCCGCCAAATCCTTTCTGGGCTTCCA

TTGGTTTATCTGTATCACCGCTTCCGTTGGGAAGTGGAATGCAGTATGAGAGCTCGGTTT

CTCTTGGATACTTAAATCAATCATTTCAAAATGCAGTTATGGAAGGGATACGCTATGGTT

GCGAACAAGGATTATATGGTTGGAATGTGACGGACTGTAAAATCTGTTTTAAGTATGGCT

TATACTATAGCCCTGTTAGTACCCCAGCAGATTTTCGGATGCTTGCTCCTATTGTATTGG

AACAAGTCTTAAAAAAAGCTGGAACAGAATTGTTAGAGCCATATCTTAGTTTTAAAATTT

ATGCGCCACAGGAATATCTTTCACGAGCATACAACGATGCTCCTAAATATTGTGCGAACA

TCGTAGACACTCAATTGAAAAATAATGAGGTCATTCTTAGTGGAGAAATCCCTGCTCGGT

GTATTCAAGAATATCGTAGTGATTTAACTTTCTTTACAAATGGACGTAGTGTTTGTTTAA

CAGAGTTAAAAGGGTACCATGTTACTACCGGTGAACCTGTTTGCCAGCCCCGTCGTCCAA

ATAGTCGGATAGATAAAGTACGATATATGTTCAATAAAATAACTTAGTGTATTTTATGTT

GTTATATAAATATGGTTTCTTGTTAAATAAGATGAAATATTTTTTAATAAAGATTTGAAT

TAAAGTGTAAAGGAGGAGATAGTTATTATAAACTACAAGTGGATATTGTGTGCTGAGAGC

TTTCTTCTATACTAATAGACGAAAGGGTGTGAAAATGATTTTTAAATGATACTGTGGAAC

GGAACAGTAGCCCTAGTATTGACTACTGTCGTTTCTATTCATATTGGCTATTCTAGGACT

GAGATGAAAAAATCTATAAATGCTCAGAATAAAATTGAACCCGCAAATCTCCCCAAAACA

ATGGTGAGTCATGTACTTGTATTATTCCGAAAAAATACACCTCTGGTGCAGTGAGACAAA

TTGGTGTATCTTATAGTGGCTTCGTAGATGAAAGCTATACTCTACTATCACTCTTTGATG

ATGTAGAACAAATTGAAAAAGATAATAGACTTCAGACAGCTATTGATGTTGTCAGAGAAC

AGTTTGGTTTTTTAGCCATACAAAAAGGAACCGTCCTAACTGAAGGTTCCAGAAATATTG

AACGCAGTAAACTTATCGGTGGTCATTCCGCGGGTGGATTGGAGGGATTAAAATGAAACA

AGAAAAAAATACAGTACAATTTTCAGAAATCCGTAGCAAAGGATGTAATGATATTGAAAT

GCTTGAAAGATTTTTACATGGAATCGTTGAAACAGCAACTTCAAAACTTCGTCAGAGAAA

ACTCAAAACAACTGAAATATCGATACGACTAGTACATGCTAAATCTGAAAACCGATTACC

ATTGGAATTTACATTTAGCATTAAGCCAACAAGCTCATCTGTGATAATCTATACTGAGGT

AATCAATCGCTTTAAAGAATGTTACACAGGTGGGGGAATTCAAGGTTTTACGATTCAATT

TGATAAAAATACCCTTGCCTCTGCATAGAAAGGATTTGATATGATTGACCGTTCATATTT

ACCATTTCAATCAGCAAGAGAGTACCAGGATACAAAGATGCAAAAATGGATGGGCTTTTT

CCTATCTGAACATGCATCAGCACTCTCTGATGATACAAACAAAGTAACGTACATGTCTGA

CTTATCACTAGAGAAGAAATTATTACTCCTCAGTCAAGTATACGCCGGGCAGCTACGCAC

ACGCATTCAAGTGATTGAAAAAAACAAGCGTGTTTCCTACACTGGAACAATACCAAGTCT

GACCAAAGATTTCATTTTGATAAAAACTACAACAGGTCACATCAATTTGAAATTAAAAGA

CATTATTAGTATTGAACTTGTCGAGGAGGTGCTCTATGAATCAGCTTGAGTTTCAGCGTA

ATCACCTACAAATGGACTATTATAGCGAGAGCTACCAAGATTTTGAACGTGACTTCTACC

GCTACTCTAACATGAATATTCCATTGACCTTCCTAACTGATGATATCCTAAAAACAATGG

CGACTTCACGTAAGAATTACTTTGTCCTCAATAAGGAAAAGTCCAGAGATAACCGCGATC

ACTTCTTCATATTTGAAGTAAGTACCGTAGATGAGAATCCGCTAATCTATCATTATACAT

ATAAGAAAACTACAATATATTTAGCAGAAAAATAGGAGCAGTTCAATTGACTGTTCCTAT

TTTTAATATTCATAAAATCTAAAGTCTTTATACTCTTTAACAATGGAGTCGCCAACCAGA

ACAGACTATACTGACCAGCGACTACCTTAAATTTAATGTTTCAGATTTATTTTCTTATCT

CTAATTTCATAAACTACATCTGCTACATTTTCGAGTAATCGTTTATCGTGGGTGATAAAC

ACGATAGTTCCGGTGTACTCCTTCATTAGTATTTCCAAAGCCTCTAAACTTGGTATGTCA

AGGAAGTTACTGGGTTCATCCATTATTAGGATGTTATATCTACCCATGAGCATTTTAGCA

AGCAACAATTTTATAATTTCTCCACCGCTTAAAACAGATAAACTTTTTCCAATATCGTTC

TGTTTGAACCCCATAGATGCTAGCACTGAACGAATTTCTGATATATTGTAGTCACAATCC

TTCTGCATAAACTCCATAACATTCTGATTACTGTTGTACTTGTAACCATTCTGTGCAAAG

TAACCTATTTTTGCCTTAGGCGAAATAGAAATTCCTTCTTCATGGTTTAAGATCATTTGG

ATTAAAGTTGTTTTTCCGATTCCATTACCACCAGTTAACGCCACTTTTGCTCCTAACGGA

ATTTGAAAAGATGCATTTTCAAACAGAGCCTTATCCCCAAATACTTTATTAATTTCTGCA

CCGACTATAGGGTATGGATTATGGAGCTCCAATGCTTTACTTTGCCTGAAACGAATTCTG

CGAATGCCTTCCGGAGCTTCTACTTTTCCTAAGGCCGCAATCCTGTGCTCTAGGGTTTTA

GCAGCATTATACATCTTTTTTTCCTTACTTCCTATTGATTTTTGATGAGCTAAACGCCCT

CCGTCTTCAGTACTTTTTTTCTTTGAAGAACCTTTTGCCTTCTGTTCTATTTTACGAGCC

TGTTTTCGCTTTTCCTCCGCAGCCCTTTCCAATCGGGCACGTTCCGCAATAAATTGTTCG

TATTCTGCAGCTTGGCTCTTACGTTCTTCCTCTTTCTGACGAAGATAATCAGAATAGTTT

CCCCAATACTCAGTGATTTTGCCATCTTTCAGTTCCCATATTTTATCTACTATTTCATCA

AGAAAATAGCGGTCATGGCTAATAACTAACAGTGCACCTGTAAAATATTTTAGCTGTCCT

ATTAGAAAATCAATTCCTTCACGGTCTAAATGGCTCGTAGGTTCATCCGCTAAAATACCA

TGAACCTGTGCCGATAAGGCCTGTGCTATTTTAAGCCTTGTTTCTTCACCACCGCTCATA

GTCTGTATATTTAATTGCTCAACACCTAGCTTGCCTACAAGTGCAAAATCTTTTTCCTCC

TGCAGAGTTACTTCGTCCAACTGGGGAATATAGGCAAGTTCACCCAGACGATTCATTTTA

CATCCTGGGGGAGTTAATTCTCCTAAAAGTACCCTGAGTAAAGTGCTTTTTCCAGCACCA

TTTGCTCCTACTAAACCAATACGGTCATAATCATATACTTCTAATTCATTTATATCTAAA

ACATCGCGTCCTTTGAATTCCACACGAATGTCTTTTGCTTTTAATATTAATTCCATAACA

TTTCCTCCTGTCTATAATCGCATGCTTTCATTTGCTTGTATGCAGGGAAAACCCTGCGAT

TTTAGCAGGAAGAGTTACATGAAAATAAGATACATAAATATTCCTCCAATATTGTTTATT

TTAAATCTAATTTTCTAACCTCAGTTATCATTTGGCAAACTATAGCAATGCCAATAATTA

AAATACCTGATAGTAAAAACCAATGATTTACACCGATTTTATCAGCAAAGAATCCAGAAA

GAATTAACCCAATTGGCATAGCAAGTGACATGATACTTCCGATCAAAGAAAATACACGTC

CTAAATATTCAGGCTTAATTTTCTCCTGAAAAAGAGCTGTTTGCACACCGCTATAAAATG

GCACCGAAAGCCCCATTATTGCACAGCAAACTACGAATATTACAAATCCATTTGGAGGAA

GTATTCCCGAAACGGCTAAACTGGTCCCCATTATAAAAAATGAACTTGTTATTAGTAATA

CATGCTTTTCGAAGCCCCCTAATCTTCCTAATAATAAGCCTCCTGCTAGCATCCCAAATG

CAAAGGAAATTTCCGTAATAGAAATATGCACAGGCGTTCCATTAAAGTGTTCCATGCTTA

TTAAAGGAAATAGTGCATTGATTGGCATATAAACAAAAGTATATAGTGTTCCTAAGAGTA

ATAAGGCAAACAATCCTTTGTTTTGTCTCAGAACCACAACTCCTTCTTTCATCTCCCTTA

TGAAATTTGGTTCTAAACTTTGCACTTGATTACCCAGCTTAGGTATACGTACAATTGCTA

CCGTAATAGATGCAATCACAGCACCCAATACGTCGATGGCAATAATAGCATTTAAATCCC

AAACGGAGTATAAGAGTGCTGCAACTGCCGGACTAACAATATAGCTTATAGACTGCAAAG

ACTGACTATAGCCTGCGCATTTCGTTAGCTGTTCTTCTGGTACTAAAAGTGGTGTAACCG

CATTGAGTGCTGGGGTATGAAAAGCTGTTCCAATGCTACGGATAAACAATACTATCATAA

TCATCCAGACAGGTAGCTCCATACAGAATGCAACAATAGCAAGCACTGCACCAGCTGCTG

CGATAATTAAATCGGCACCAATCATTATCTTCTTCCTATCATGACGATCCACTAGCACAC

CAATGGCAGGTCCCAAAATCGCATAGGGTAAAAAACCTACTAATGAAGCCATAGACAAGA

CCATCGCAGATCCTGTTTTTTCTGTAAGGTAAAAAATAATCGCCATTTGCAGGATGGCAC

TAGTGATTAATGATACTGCTTGCCCTGCCCATATTGCATAAAATTTTCGTTTCCAATTGT

TGTATTTTTCCATTTATATTATCTCCTGCATATTATTTTGCTTGAATTTCTATTTTGAAT

AGCATTCTAGGCAATAAAAAATGCAGGCCAAACCCCACAATGTGGCTTTTGGTCTGCATA

CATACAATTTGGAAACATTCATATTAAAGACATAGTTAAATAAAGGTATAGTTAAATAAC

CAATATCCTCACCGTAACTAATGAATGCTCAATATCGTATAAATAAGCACAACAAAAAAG

CCTATCATCGGGTATAGATTCTGCTTTTTTTATTGCCAGCTTATCTTAAACGCATTGAGG

CTGTCATAGTTTCGGTTCCTCCTACATCTTTGTTTATATCAATTTATAGTATAACACAAA

AAGATGATATGTTCAATATAAAAGTTATGGAATGAGACTCATACTTCCAATTCGATGCCA

GATTTAAAGGATATGACGAAGTTTTCTTCATAGACTGTAACGCTCTGGATTATCTTCCTT

AGTAGCAAGCGATTAGCTTTCACAAAATCTTCTGTTTGTAGTTTTAAAAATTCATCAGGA

TTTTCTAACTCAACCTCAAAATATTTCATTTTACATTCCCTCATTTCATTTATTGATAAA

TTGAGTTTGCAAAAAAGAGTGGACAATTTTTGTCTACTCTTAACCTTTAAAATAGTTTTT

TTTAATCGATTTGAAGTTGCCTAAATTATTACTTATTCGGTAAAATGAAGTATTGCTTTC

AACAGATTTCCTTCAACTACACTTCACTTGATTCAAACAAGGTGGGTACATTTCTATTCC

CACAAACTCCTTGTCAATGGAAACAAACACGTACCCACAGGGTAAATGGAAATAGAAACT

GATAATTTCTAGCTATCACTTCTACTCATTCCAAAAATTTTCTCACTCTGATACTTACCC

ACCATAAAGCAAAAAGCCTTGCAATCAAGGCTTTCATTATCCCTTTCGTTCAAAGGTTTC

TAAGCTTTTACGAGCAGAGCGACACACTCAGCGGTTCGCTATCTCCGTTCTGTCTGCGTG

CTAGCACTTGTCAATCACGGACAGCTATCGCATGGGCGGAAGTAAATGCTAATCTTCGTC

GTTTTACTCCTTGACTAGCAAACTTACCGCCTCAACATGTCCTGTATGTGGAAATAAAAC

ACGATTAAAGATAAGGGAAGATACTGAATTAAAAAAATTCCCCCTCTATTGTCCGAAATG

CAGACAAGAAAATTTAATTGAAATAAAGCAGTTCAAAGTAACTGTGATTACAGAGCCAGA

CGCAAAGACGCAGAGCCGATAAAATGAGATTAATACAATCTCATTTTATCGGCTCTTTCC

GTTATGTATGGATTCTTTTAATTAGTCTTCGATGTTTCTTGCTTCGTTGATACCGCTGGC

TAAAGATTCCATTAAGGATAGTTCTTTGTCTGTAAAGCTATCCATGTATTTCTCTATCTG

TAATCGTCGGGTGCTTTTTACCAAGTTATTAGCAGGTAAGAAAAATTCATCAACGGAAAC

ATGAAGTAACGATACAAGGTCATAAAGAACTTGTATGCTGGGGTGTTGCCCTTTATTTTC

AATATTAGTTAAGTACCGTGGGTCAATTTCAATCAATGCTCCCACTTGTTCACGAGTTAA

ACCTCGTTTCAATCGAGCTTCTTTAATGGCTAAACCAAAGGCTCTAAAATCATATTTATC

TTCTTTTTTACGCATAGTAGACCACCTCTATACATTTTATTGTTCCTACTGAATTAAAAA

CAGGTATAGAAAAACGTGTTATATGGTTTATAGGTTTATATTTAATAAAAAGCACTACTA

AACGCCAATAAAAAAAACCGTTATATGGTAGTGCTATTTACGCTGTTAAAATATTGTATA

TTACTTCCAAATGGCGGTTTGTTGGAGGTCAACGTCGCCATGAAGTACATCATATACAAT

AAATTTCCTTACATTGGGTTCTTGTCAAAAAAAGTCGTCTATCTGCAATAGATAAGTACG

TCCACCAATGTGGTTTTATAAATCATATAGATAGAATAACAGAAGCATGTAAACAGAGAA

ATAAATCTGTTTATATGCTTTTTTGGCTATTCAGAACTTTTTTACAAAGTTTATTTATCA

GTAATGCAACAAATCCCCCTTTCACATTGGGACTAAGAGTGAAAGGAGATAAACGAGCAA

GGCTCACTTCCTTTCCTAGACAGAAAGGGGGTGAGAAACATGAAACCATCTTCTTTTCAG

ACCACAATAGAAAATCAGTTTGACTATATCTGTAAACGTGCTATGGAAGACGAGCGAAAG

AATTATATGCTTTATCTTTCAAGGATTGCAAAGCGTGAGGTGTCCTTTTCGGATGTTGGC

GATTATCTTGTTAGCCAGTTTGCGACAACAGATAACTATTCAACTGACTTTCAGATTTTT

ACACTCAATTGGTTATCAGTAGGCGTTGAAAATGATTTGTTGAGTGAAGCATTACGTGAG

TTGCCAGACAAGAAACGTGAAATTCTACTGCTGTTTTACTTTATGGACATGAGCGATTCA

GAAATTGCAGACCTGTTGAAATTGAACCGTTCTACTGTCTATCGGCATAGAACCAGTGGA

CTAGCCTTAATTAAAAAGTTTATGGAGGAATTTGAAGAATGAAAACACAATATCCTATGA

TTCCCTTTCCTCTCATTGTAAAGGCAACAGATGGCGATACCGAAGCGATTAACCAGATTC

TACATCATTACAGAGGGTACATAACGAAGCGTTCCCTACGACTTATGAAAGATGAATATG

GCAATCAAAGTATGGTCGTTGATGAAGTCTTACGTGGAAGAATGGAAACCAGACTGATTA

CAAAGATTTTGTCATTTGAAATTAAGTAATATCCTCTCTCCTTTCGTGGAAGCGTGCTAA

ACCATTCCACGCTTCCCGAACAGGGAGGTTTGTTATTCCACCAAAGCATATTGAGCTTTC

AATGTGTTTTGATAGGCTAACGAGCCATTGTTCTTTGAAAACTGAATAAAAGTAATCGAA

TACGTTTCGATAAGAAAAGAGCCAACGGAACTAACCGCCATGACCTATCTTATAAAGATA

GCGAGCGATTCATGTTAGTGATCCGAGAAGCAATCTTTAGCAGGATTGCCTGCAACGACA

TTCTTATCGTGATAATGATACTCCCATACAGTCAATAGTCCGAGCGTGATAAAACCGTCG

CAGGCAATGAGTATGGCTACATGAGAACCATGCAGGGGTGGAACTCCCGTGAGCTTTGCT

AAAGCTGTTCGATTGCTGGTAAAACAACTTTTATGAAATCCAAATAAGTGATTTGGAAAG

GAGGATTTTATGAAGCAGACTGACATTCCTATTTGGGAACGTTATACCCTAACCATTGAA

GAAGCGTCAAAATATTTTCGTATTGGCGAAAACAAGCTACGACGCTTGGCAGAGGAAAAT

AAAAATGCAAATTGGCTGATTATGAATGGCAATCGTATTCAGATTAAACGAAAACAATTT

GAAAAAATTATAGATACATTGGACGCAATCTAGCGTCGCCAAAGGGTCTTGTATATGATA

AAATAGTATTAAGTCGTATCAAGGCTCTTTCCATAAAGGAAAGGAGCAAATGCCATGTCA

GAAAAAAGACGTGACAATAAAGGTCGAATCTTAAAGACTGGAGAGAGCCAACGAAAAGAC

GGAAGATACTTATACAAATATATAGATTCATTTGGAGAACCGCAATTTGTTTACTCGTGG

AAACTTGTGGCTACAGACCGAGTACCAGCAGGAAAGCGTGATTGTATCTCACTTAGAGAG

AAAATCGCAGAGTTACAGAAAGACATTCATGATGGTATTGATGTTGTAGGAAAGAAAATG

ACACTCTGCCAGCTTTACGCAAAACAGAACGCTCAAAGACCAAAGGTTAGAAAAAACACT

GAAACTGGACGCAAATATCTTATGGATATTTTGAAGAAAGACAAGTTAGGTGTAAGAAGT

ATTGACAGTATTAAGCCATCAGACGCTAAAGAATGGGCTATTAGAATGAGTGAAAATGGT

TATGCTTATCAAACCATCAATAACTACAAACGTTCTTTAAAGGCTTCATTCTATATTGCT

ATACAAGATGATTGTGTTCGGAAGAATCCATTTGACTTTCAACTGAAAGCAGTTCTTGAT

GATGATACTGTCCCTAAGACCGTACTAACAGAAGAACAGGAAGAAAAACTGTTAGCCTTT

GCAAAAGCTGATAAAACCTACAGCAAAAATTATGATGAAATTCTGATACTCTTAAAAACA

GGTCTTCGTATTTCAGAGTTTGGTGGTTTGACACTTCCAGATTTAGATTTTGAGAATCGT

CTTGTCAATATAGACCATCAGCTATTGAGAGATACTGAAATTGGGTACTACATTGAAACA

CCAAAGACCAAAAGTGGCGAACGTCAAGTTCCTATGGTTGAAGAAGCCTATCAAGCATTT

AAGCGAGTGTTAGCGAATCGAAAGAATGATAAGCGTGTTGAGATTGATGGATATAGTGAT

TTCCTCTTTCTTAATAGAAAGAACTATCCAAAAGTGGCAAGTGATTACAACGGCATGATG

AAAGGTCTTGTTAAGAAATACAATAAGTATAACGAGGATAAATTGCCACACATCACTCCA

CATAGTTTGCGACATACATTCTGTACCAACTATGCAAATGCAGGAATGAATCCAAAGGCA

TTACAGTACATTATGGGACATGCTAATATAGCCATGACGCTGAACTATTACGCACATGCA

ACATTCGATTCTGCAATGGCAGAAATGAAACGCTTGAATAAAGAGAAGCAACAGGAGCGT

CTTGTTGCTTAGTAGTACAAATGAATTTACTACTTATTTACCACTTCTGACAGCTAAGAC

ATGAGGAAATATGCAAAGAAACGTGAAGTATCTTCCTACAGTAAAAATACTCGAAAGCAC

ATAGAATAAGGCTTTACGAGCATTTAAGAAAATATAAAAAGATAATTAGAAATTTATACT

TTGTTT

>GA44378/Tn916

AAAATAGCATAAAAATCTAGTTATCCGCATAAAAACTGGACTTATCACACTTTATCAAGG

TCAAAACCACTCAATTTACTACTAATTTACTACTTATGAATGAGCTTTGATACGACGATT

TATCCTTGAAAAGTGAAGATATAAAGATACTTCCAATAAAATTTGAATATTTAATAGGTA

GACACTTCAAAAAATGAGGTGTCTATTTTTTTACCCGATTTTGAAAGGAAGTGAACTTAT

GAAAACAAAAAATCAAGAATCAAAAGGTCGTTCCCCACTCTTTAAGACCATCAAACATTC

ATTCAGCCAATAAAAAAGAAAGGATAGGTAAAAATATGGAACTTAAATTTGTGATTCCCA

ACATGGAAAAAACATTCGGCAATTTAGAATTTGCTGGCGAGGATAAAGTCGTTCAGCGAA

GAATCAACGGACGGCTAACTGTCTTATCAAGAAGCTATAATCTCTATTCTGATGTTCAAA

GAGCAGATGATATTGTGGTGGTGCTTCCTGCTGAAGCTGGCGAAAAACATTTCGGCTTTG

AGGAACGTGTGAAGTTAGTCAATCCACGTATTACCGCAGAGGGCTACAAAATCGGCACTC

GTGGTTTTACAAATTACCTTTTACATGCTGACGACATGATAAAAGAATAAAGAAAGAGAG

GAAAAATGATGAGATTAGCAAATGGCATTGTATTAGATAAAGACACGACTTTTGGAGAAT

TGAAATTCTCTGCTCTACGTCGTGAAGTGAGAATCCAAAATGAAGACGGGTCGGTTTCAG

ATGAAATCAAGGAACGTACCTATGACTTAAAATCCAAAGGACAAGGACGCATGATTCAAG

TAAGTATTCCTGCCAGCGTGCCTTTGAAAGAGTTTGATTATAACGCACGGGTGGAACTTA

TCAATCCCATTGCGGACACCGTTGCTACTGCCACCTATCAAGGAGCAGATGTTGACTGGT

ATATCAAGGCAGACGATATTGTGCTGACAAAGGATTCTAGTTCATTCAAAGCTCAACCAC

AAGCAAAGAAAGAACCGACACAAGACAAATAGTCGCTAGGTAGAAAGGAGACTTTTTCGC

ATGAAACAGCGTGGTAAAAGGATTCGCCCATCTGGTAAAGATTTAGTCTTTCATTTTACG

ATAGCGTCACTCCTGCCTGTTTTCCTGCTGGTTGTCGGACTGTTTCATGTGAAGACAATC

CAGCAGATCAACTGGCAGGATTTTAACCTATCACAAGCAGATAAGATTGACATTCCCTAT

TTAATTATCAGTTTCAGTGTCGCAATTCTTATCTGCTTGCTGGTAGCGTTTGTATTCAAA

CGGGTTCGCTATGATACGGTTAAACAACTTTACCACCGTCAAAAACTGGCAAAGATGATA

CTTGAAAACAAGTGGTATGAATCTGAACAGGTCAAAACAGAGGGTTTCTTTAAAGATAGT

GCTGGTCGTACAAAGGAAAAGATAACCTACTTCCCTAAAATGTATTATCGACTTAAAAAT

GGCTTGATACAGATACGGGTGGAAATCACGCTGGGAAAATATCAAGACCAACTCTTACAC

TTGGAAAAGAAATTAGAGAGTGGCTTGTACTGTGAGCTGACGGATAAAGAGTTAAAGGAT

TCCTATGTGGAATATACTTTGCTCTATGACACCATAGCCAGTCGTATTTCTATTGATGAA

GTAGAAGCTAAAGATGGTAAACTTCGCTTAATGAAAAACGTATGGTGGGAATATGATAAG

CTCCCTCATATGTTGATTGCTGGTGGTACAGGTGGCGGTAAAACTTACTTTATACTGACA

CTGATTGAAGCCTTGCTTCATACAGATTCAAAACTGTATATTCTTGACCCGAAAAATGCT

GACCTTGCGGACTTAGGTTCTGTGATGGCAAATGTCTACTATAGAAAAGAAGACTTGCTT

TCTTGCATTGAAACATTCTATGAAGAAATGATGAAACGTAGTGAGGAAATGAAGCAGATG

AAGAACTATAAGACTGGCAAAAATTATGCTTACTTAGGTCTCCCGGCACACTTCTTAATC

TTTGATGAATACGTCGCTTTCATGGAAATGCTGGGAACAAAAGAAAACACCGCAGTTATG

AATAAGCTGAAACAGATTGTCATGTTAGGTCGTCAAGCTGGCTTCTTTCTAATACTGGCT

TGTCAACGTCCAGACGCAAAATATTTAGGCGACGGAATCCGTGATCAGTTTAATTTCAGA

GTGGCTTTAGGTCGTATGTCTGAAATGGGCTATGGCATGATGTTTGGCAGTGACGTACAA

AAGGATTTCTTCTTAAAGCGAATCAAAGGTCGTGGCTATGTTGATGTAGGAACAAGTGTC

ATATCAGAGTTTTATACTCCCCTTGTACCAAAAGGATATGATTTCTTGGAGGAAATTAAA

AAGTTATCCAACAGCAGACAGTCCACGCAGGCGACGTGCGAAGCGGAAGTCGCAGGTGTG

GACTGATCTTGCTGGCTGGTGTGGCAATAGCCACGCCAGCACTTAACCCCCCGTATCTAA

CAGGGGGGTACAAATCGACAGGAAACAGTCAAAAAAACATTAGAAAATCCTTTGGTTACA

AGGGATTTACAAAATTTCAGCGTATGTCAAATGGGCTTTAAAAGTTGACATACGCCTTTT

TGATTGGAGGGATTTTTACTGAATGAACAAACTTGGTTACAGCATTTAAAAGAAAAACGC

TTGGCTTATGGACTATCTCAAAACCGTTTAGCTGTTGCGACTGGTATTACAAGGCAGTAT

CTAAGCGATATTGAAACAGGAAAAGTCAAGCCATCAGAGGATTTACAGCAGTCCCTTTGG

GAAGCTCTGGAACGCTTCAATCCCGACGCTCCCCTTGAAATGCTGTTTGATTATGTAAGG

ATTCGCTTTCCGACAACAGACGTACAGCAGGTGGTCGAAAACATCTTACAACTGAAACTG

TCCTATTTTCTTCATGAGGACTATGGTTTCTATTCTTATTCAGAGCATTATGCTTTAGGC

GACATATTCGTCCTTTGCTCCCATGAACTGGACAAAGGAGTTCTGGTGGAATTGAAAGGT

CGTGGGTGCAGACAATTTGAAAGCTATCTTCTGGCACAACAAAGAAGCTGGTATGAGTTC

TTTATGGACGTTTTGGTGGCTGGCGGTGTGATGAAACGCCTTGACCTTGCCATTAACGAT

AAGACAGGGATTTTGAATATCCCTGTACTCACTGAAAAGTGCCAACAGGAAGAATGTATC

TCCGTCTTCCGCAGTTTTAAAAGCTATCGCAGTGGCGAACTGGTACGCAAAGAGGAAAAG

GAATGTATGGGAAACACCCTCTATATCGGTTCATTACAAAGTGAAGTTTATTTCTGTATC

TATGAAAAGGACTACGAGCAGTACAAGAAAAATGATATTCCCATTGAAGACGCAGAAGTA

AAAAACCGTTTTGAGATTCGATTGAAAAATGAGCGTGCCTATTATGCAGTCCGTGATTTA

CTCGTCTATGACAATCCAGAGCATACCGCCTTTAAAATTATCAATCGGTATATCCGTTTT

GTAGATAAAGACGATTCCAAACCTCGTTCTGATTGGAAACTGAATGAAGAATGGGCTTGG

TTTATTGGGAACAATCGTGAACGATTAAAACTAACCACAAAACCAGAGCCTTACTCCTTC

CAAAGGACGCTGAACTGGCTATCTCATCAAGTTGCCCCGACCTTAAAGGTTGCGATTAAA

CTTGATGAAATCAACCAGACGCAGGTTGTAAAAGACATTCTCGACCATGCGAAACTGACA

GACCGACACAAGCAGATTTTGAAGCAACAGTCAGTAAAAGAACAGGACGTGATAACAACA

AAAAAATAACTCAAATACAAATTCATTGAATATAGAGAGGAGAACATTTTTATGAATTTT

GGACAAAACCTTTATAACTGGTTTCTATCAAACGCTCAATCACTGGTGCTTTTAGCAATC

GTTGTGATTGGCTTGTATCTTGGCTTCAAGCGTGAGTTTAGCAAACTGATTGGCTTTTTA

ATTATTGCGATTATTGCGGTTGGCTTAGTCTTCAACGCTGCTGGAGTAAAAGACATTTTA

CTAGAGCTATTCAATCGCATTATTGGTGCTTAAATAAAACCGTTCTTTTGTGGAATATAA

GTGGTTTTCTTATGTTCCGCAAAGGAATGGTACACCAAACGAAGTGCGGTAGGGATTTTT

GAATCTCTACAAAGAAAGGACGTGAATATATGGACGATATGCAAGTCTATATTGCGAATT

TAGGCAAATACAATGAGGGCGAATTGGTCGGTGCGTGGTTTACCTTTCCCATTGACTTTG

AGGAAGTCAAAGAGAAAATCGGCTTGAATGATGAATATGAGGAATACGCCATTCATGACT

ACGAGTTACCCTTTACGGTTGACGAATACACTTCCATTGGCGAACTCAATCGACTATGGG

AAATGGTATCGGAATTACCCGAAGAATTACAATCGGAGCTATCTGCTCTGCTCACTCATT

TTTCAAGCATTGAAGAACTAAGCGAACATCAAGAGGATATTATCATTCATTCCGATTGTG

ATGATATGTATGACGTGGCACGCTACTACATTGAAGAAACGGGTGCTTTAGGCGAAGTAC

CAGCTAGTCTTCAAAACTATATTGATTATCAAGCCTATGGTCGGGATTTAGACCTTTCAG

GAACGTTTATCTCAACCAATCATGGGATTTTTGAAATCGTCTATTAAATCTGTCGGTACA

TTACTACTGGCAGATTTTCTATTTTACGGGGTGGCTCAATCAGCTACCCCTATTTTTTAT

GAAAGGATTGATTACATGAAGAAAATACGAAGCTATACCAGTATCTGGTCTGTGGAAAAG

GTACTGTATTCTATCAATGATTTTAGACTTCCGTTTCCCATAACCTTTACGCAAATGACA

TGGTTTGTCGTGTCACTCTTTGCAGTGATGATACTTGGCAACTTGCCCCCTCTTTCCATG

ATAGAGGGAGCATTTCTCAAATACTTTGGGATTCCTGTGGCTTTCACATGGTTTATGTCT

ACAAAAACTTTTGATGGTAAAAAGCCTTATGGATTTTTGAAGTCTGTCATTGCTTATGCA

CTGCGACCAAAGCTGACCTATGCAGGAAAAAAAGTAACGCTTGGCAGAAACCAGCCACAA

GAAGCCATTACAGCAGTTAGGAGTGAATTTTATGGCATATCCAATTAAATACATTGAAAA

CAATCTCGTCTGGAATAAAGACGGGGAATGTTATGCTTACTATGAGCTTGTTCCTTACAA

TTACTCATTTCTAAGTCCAGAACAGAAAATACAAGTGCATGATTCTTTCAGACAGCTTAT

CGCACAAAATCGTGATGGCAAAATTCATGCTTTACAAATCAGTACAGAATCCAGCATACG

TTCTGCACAAGAGCGTTCCAAAAATGAAGTCACTGGCAAGCTCAAAGCGGTTGCCTATGA

CAAAATCGACCAACAGACAGACGCTTTAATATCCATGATTGGCGAAAATCAAGTGAACTA

CCGTTTCTTTATCGGCTTTAAGTTGCTTCTCAACGATCAGGAGTTTTCTATGAAAAGTCT

TACCGTTGAAGCAAAAAATGCTTTGTCTGATTTTGTCTATGATGTGAACCATAAGCTGAT

GGGCGATTTTGTTAGTATGAGTAATGATGAAATCCTGCGTTTTCAGAAGATGGAAAAGCT

CTTAGAAAATAAAATCTCTCGTCGTTTCAAAATCCGCAGGTTAGATAAGGACGACTTCGG

CTATCTGATTGAACACCTTTACGGACAGACAGGCACTGCCTATGAAGAGTATGAGTACCA

TCTATCAAAGAAAAAGCTGGATAATGAAACGCTGATTAAATACTATGACTTGATTAAGCC

TACTCGCTGTTTGGTGGAAGAAAAACAGCGATATTTGAAAATCCAGCAGGAAGATGAAAC

CGTCTATGTAGCTTACTTTACCATTAACAGCATTGTCGGAGAACTGGACTTCCCGTCCTC

TGAAATCTTCTACTACCAGCAACAGCAATTTACATTCCCGATTGATACGTCAATGAATGT

GGAAATTGTAGCGAATCGTAAAGCCCTATCTACTGTCCGCAATAAAAAGAAAGAACTGAA

AGACTTGGATAACCACGCTTGGCAAAGTGATAATGAAACCAGCTCCAATGTGGCGGAAGC

TCTGGAAAGTGTGAATGAGCTGGAAACCAATTTAGACCAAAGCAAGGAATCTATGTACAA

GCTGTCTTATGTGGTAAGGGTATCAGCAAATGATCTTGACGAACTCAAACGTCGTTGTAA

TGAAGTGAAAGATTTTTATGACGATTTAAGCGTAAAACTGGTACGACCATTTGGGGATAT

GCTCGGCTTACATGAAGAATTTTTACCTGCCAGCAAGCGTTATATGAATGATTATATTCA

ATACGTGACCTCTGATTTCCTCGCTGGTTTAGGTTTTGGTGCTACTCAAATGCTGGGGGA

AAATGAGGGGATTTATGTTGGCTACAGCTTAGATACTGGACGCAATGTCTATCTGAAACC

TGCTCTTGCCAGTCAAGGGGTTAAGGGTTCAGTAACCAATGCGTTAGCGTCGGCTTTTGT

TGGTTCGCTGGGTGGTGGTAAATCCTTTGCGAATAACCTTATCGTCTATTATGCGGTGCT

TTATGGGGCACAAGCAGTGATTGTAGACCCAAAAGCAGAACGTGGCAGATGGAAAGAAAC

CTTGCCAGAGATTTCCCATGAAATCAATATCGTCACTCTGACTTCTGATGAGAAAAACAA

AGGCTTACTTGACCCTTATGTGATTATGAAAAATCCCAAAGATTCTGAATCACTGGCTAT

TGATATTCTGACATTCCTTACGGGGATTTCCTCTCGTGATGGGGAACGCTTCCCAATCCT

TAGAAAAGCCATTCGTGCAGTAACCAATAGTGAAGTACGAGGGTTGATGAAAGTGATTGA

GGAATTACGGGTTGAGAATACGCCACTAAGTACCAGTATAGCCGACCATATCGAAAGTTT

TACAGACTATGACTTTGCACATTTATTATTCAGTAATGGTTATGTGGAGCAGTCTATCAG

CTTAGAAAAACAACTGAACATTATACAGGTTGCGGACTTGGTACTTCCCGACAAGGAAAC

TTCCTTTGAGGAATATACCACTATGGAGCTTTTATCCGTTGCTATGCTGATTGTCATTAG

TACCTTTGCTTTAGACTTTATCCATACAGACCGAAGCATTTTCAAGATTGTAGATTTAGA

CGAAGCATGGAGCTTTTTACAGGTAGCACAAGGAAAAACACTATCTATGAAGCTGGTTCG

GGCTGGTCGTGCTATGAACGCTGGAGTATATTTCGTGACCCAAAATACAGACGACCTCTT

AGATGAAAAACTGAAAAATAACCTCGGCTTAAAATTTGCATTTCGTTCCACTGACCTTAA

CGAGATTAAAAAGACCTTAGCCTTTTTTGGTGTAGACCCAGAGGACGAAAACAATCAGAA

GCGATTGCGTGATTTGGAAAACGGGCAATGCCTTATCAGTGATTTATATGGTCGTGTCGG

TGTGATACAGTTCCACCCTGTATTTGAAGAACTGCTCCATGCCTTTGATACCAGACCACC

TGTGCGAAAAGAGGTGTAAATGTGAAACCATCAATAGTAAACAGAATAAAATCAAACTGG

ACGCTGAAACGTCTAGGTAAAGTGGCAATGACAGTGGCTTTCACACTTGTGATTGCCATT

TTTCTTTTAGCCATGCTGGGAACGGTGGTTCAAGCTGCGGGCTTGGTAGATGATACGGTC

AATGTGGCAAATGAATACAGCCGATACCCACTTGAAAACTATCAACTGGATTTTTATGTG

GATAATAGCTGGGGCTGGCTTCCGTGGAACTGGTCGGACGGGATTGGAAAACAGGTCATG

TATGGACTATATGCCATTACCAATTTTATTTGGACAATCAGTTTGTATGTTTCCAATGCG

ACAGGTTACTTAGTACAGGAAGCCTATTCCTTAGACTTCATTTCCGCTACAGCAGATTCC

ATTGGTAAGAATATGCAGACCTTAGCTGGTGTGAGTGCAAACGGATTTTCAACAGAGGGT

TTCTATGTTGGATTCCTCTTACTCTTGATTTTGGTTCTTGGGGTTTATGTTGCCTATACG

GGACTGATAAAGAGAGAAACCACAAAGGCAATTCATGCCATTATGAATTTTGTGCTGGTG

TTTATCCTATCGGCTTCCTTTATTGCCTACGCTCCCGACTACATTAAAAAAATCAATGAC

TTTTCATCAGACATCAGTAATGCCAGTTTATCACTTGGCACGAAGATTGTCATGCCCCAT

TCCGATAGTCAAGGCAAGGACAGCGTGGACTTAATCAGAGATAGCCTGTTTTCCATACAG

GTTCAGCAACCGTGGCTACTGCTTCAATACAACAGTTCAGACATTGAAAGTATCGGTATT

GACCGTGTGGAAAGCCTGCTCTCCACCAGCCCAGATTCCAACAATGGCGAAGACAGAGAA

AAAATTGTTGCGGAAGAAATTGAAGACAGAAGCAATACCAATCTAACCATTACAAAGACC

ATTAACCGTTTAGGTACAGTCTTCTTCCTATTTGTCTTCAATATTGGGATTTCCATATTT

GTATTCCTATTAACAGGAATCATGATTTTCTCGCAGGTACTTTTTATCATCTATGCTATG

TTTCTGCCTGTGAGCTTTATTTTAAGCATGATTCCATCATTTGATGGTATGTCAAAACGA

GCCATAACAAAGCTCTTTAATACCATTTTGACACGAGCTGGAATCACATTGATTATTACG

ACAGCATTTAGTATTTCAACCATGCTCTATACCTTATCGGCTGGTTATCCGTTCTTTTTG

ATTGCTTTTCTACAGATTGTGACCTTTGCAGGAATCTACTTCAAGCTGGGCGATTTAATG

AGTATGTTTTCTCTACAGAGTAACGATTCTCAAAGTGTGGGAAGTCGTGTGATGAGAAAA

CCTCGTATGCTTATGCACGCTCACATGCACCGTCTACAGCGGAAACTTGGACGTTCCATG

ACTACTCTAGGGGCTGGGTCTGCCATTGTTACAGGTAAAAAAGGACAGTCGGGTTCGGGG

AGTTCTGCAAGGACACAAGCAGATCACTCCCGACCAGACGGAAAGGAAAAATCAACACTT

GGAAAACGTATCGGTCAAACCATCGGTACAGTAGCTGATACCAAAGACAGAATGGTAGAC

ACTGCTAGTGGTTTGAAAGAACAGGTTAAAGATTTGCCGACCAATGCAAGATATGCAGTA

TATCAAGGAAAATCCAAAGTAAAAGAGAATGTCCGTGATTTAACCAGTAGTATTTCTCAA

ACCAAAGCGGACAGAGCCAGTGGACGCAAGGAACAGCAGGAACAAAGGCGAAAAACCATT

GCGAAGCGTCGCTCTGAAATGAAACAGGTCAAACAGAAAAAACAGCCTGCTTCTTCTGTT

CATGAAAGACCGACTACAAGACAAGAACAATATCATGATGAACAGACCTCAAAACAGTCT

AATATTCAGACTTCATATAAGGAATCTCAACAAGCCAAACAAGAGCGTCCAGCAGTTAAG

TCCGATTTTTCAAGTCCAAAAGTGGAACGCCAAGGCAATACCGTTCAAGAAAAAACCGTT

CAAAAGCCAGCAACTTCAACCACTACAGCAGATAGAACTTCACAACGTCCAATCACAAAA

GAACGTCCGTCTACTGTTCAAAGAGTACCACTACAAAATACAAGAAGTAGACCACCAATC

AAAACCGCCACCATTAAGAAAGTCGGTAAGAAACCATGAAGTTGAAAACTTTAGTGATTG

GTGGTTCTGGATTATTCTTGATGGTCTTCTCACTGCTTCTGTTTGTTGCCATTTTATTTT

CAGATGAACAGGACAGCGGAATTTCCAATATTCATTATGGAGGTGTGAATGTTTCCGCAG

AAGTGCTGGCTCATAAGCCTATGGTAGAAAAATATGCCAAAGAATATGGCGTTGAAGAAT

ATGTCAACATACTTCTTGCGATTATACAGGTGGAATCGGGCGGTACTGCGGAAGATGTTA

TGCAGTCCTCGGAATCCCTCGGTCTTCCACCTAATTCATTGAGTACAGAAGAATCCATTA

AGCAAGGTGTGAAGTATTTCAGTGAATTATTAGCCAGTAGCGAAAGGCTCAGTGTAGATT

TAGAATCGGTTATCCAGTCCTACAATTATGGTGGTGGTTTCTTAGGGTATGTGGCTAATC

GTGGAAATAAATATACCTTTGAACTGGCTCAAAGTTTCTCAAAAGAGTATTCAGGTGGCG

AAAAAGTGTCTTACCCCAATCCCATAGCCATACCTATCAATGGGGGCTGGCGATACAACT

ATGGCAATATGTTTTATGTGCAACTGGTAACGCAGTATCTTGTCACAACAGAGTTTGATG

ATGATACGGTACAAGCCATCATGGACGAAGCACTGAAATATGAGGGCTGGCGATACGTTT

ACGGTGGAGCTTCCCCGACTACTTCTTTTGATTGTAGCGGACTGACACAATGGACGTATG

GAAAAGCTGGAATTAACTTACCACGAACCGCACAACAGCAATATGATGTGACCCAGCATA

TCCCACTATCGGAAGCACAAGCTGGCGATTTGGTTTTCTTTCATTCTACCTATAACGCTG

GCTCTTATATTACTCATGTTGGGATATACCTTGGCAATAACCGTATGTTTCATGCAGGCG

ACCCAATCGGTTATGCCGACTTAACAAGCCCCTACTGGCAACAGCATTTAGTGGGAGCAG

GACGAATCAAACAATGAGAAAGGAAGATTTAATGATGAAATTTAGAAAAAATCAGAATAA

AGAAAAACAGATACCAAAGGAAAAGAAACCTCGTGTCTATAAGGTCAATCCTCATAAAAA

GGTTGTGATTGCCTTGTGGGTACTTTTAGGGCTTAGTTTCAGCTTTGCGATATTCAAGCA

CTTTACAGCTATAGATACTCATACTATTCACGAAACAACTATCATAGAAAAGGAATACGT

TGATACTCATCATGTAGAAAATTTTGTAGAGAACTTTGCGAAAGTCTACTATTCATGGGA

GCAATCCGATAAGTCCATTGATAATCGAATGGAAAGTCTAAAAGGCTATCTGACAGATGA

ACTTCAAGCTCTCAATGTTGATACAGTACGCAAAGATATTCCTGTATCGTCTTCTGTAAG

AGGATTTCAGATATGGACGGTAGAGCCAACTGGCGACAATGAGTTTAATGTAACCTACAG

TGTAGACCAGCTCATTACAGAGGGAGAAAATACAAAGACCGTCCACTCTGCTTATATAGT

GAGTGTCTATGTAGATGGTTCTGGAAATATGGTACTGGTTAAGAATCCGACCATTACCAA

CATACCTAAGAAATCAAGTTATAAACCAAAAGCCATTGAAAGTGAGGGGACGGTTGATTC

CATTACAACCAATGAAATCAATGAGTTTTTAACGACGTTCTTCAAGCTCTATCCTACAGC

GACAGCCAGTGAACTTTCCTACTATGTGAATGACGGGATATTAAAACCAATCGGAAAAGA

GTACATCTTTCAAGAACTGGTAAATCCTATTCACAATCGTAAGGATAATCAAGTCACGGT

ATCGCTGACAGTGGAGTATATCGACCAGCAGACCAAAGCAACGCAGGTATCTCAATTTGA

TTTGGTACTTGAAAAGAACGGGAGTAATTGGAAGATTATAGAATAACAAATATTGGTACA

TTATTACAGCTATTTTGTAATCACGTACTCTCTTTGATAAAAAATTGGAGATTCCTTTAC

AAATATGCTCTTATGTGCTATTATTTAAGTATCTATTTAAAAGGAGTTAATAAATATGCG

GCAAGGTATTCTTAAATAAACTGTCAATTTGATAGTGGGAACAAATAATTGGATGTCCTT

TTTTAGGAGGGCTTAGTTTTTTGTACCCAGTTTAAGAATACCTTTATCATGTGATTCTAA

AGTATCCGGAGAATATCTGTATGCTTTGTATGCCTATGGTTATGCATAAAAATCCCAGTG

ATANNNNNCACACACTTAATTAATTAAGTGTGTGNNNNNATTTTTTATGCCCTTTTGGGT

TTTTGAATGGAGGAAAATCACATGAAAATTATTAATATTGGAGTTTTAGCTCATGTTGAT

GCAGGAAAAACTACCTTAACAGAAAGCTTATTATATAACAGTGGAGCGATTACAGAATTA

GGAAGCGTGGACAAAGGTACAACGAGGACGGATAATACGCTTTTAGAACGTCAGAGAGGA

ATTACAATTCAGACAGGAATAACCTCTTTTCAGTGGGAAAATACGAAGGTGAACATCATA

GACACGCCAGGACATATGGATTTCTTAGCAGAAGTATATCGTTCATTATCAGTTTTAGAT

GGGGCAATTCTACTGATTTCTGCAAAAGATGGCGTACAAGCACAAACTCGTATATTATTT

CATGCACTTAGGAAAATGGGGATTCCCACAATCTTTTTTATCAATAAGATTGACCAAAAT

GGAATTGATTTATCAACGGTTTATCAGGATATTAAAGAGAAACTTTCTGCCGAAATTGTA

ATCAAACAGAAGGTAGAACTGTATCCTAATATGTGTGTGACGAACTTTACCGAATCTGAA

CAATGGGATACGGTAATAGAGGGAAACGATGACCTTTTAGAGAAATATATGTCCGGTAAA

TCATTAGAAGCATTGGAACTCGAACAAGAGGAAAGCATAAGATTTCAGAATTGTTCTCTG

TTCCCTCTTTATCATGGAAGTGCAAAAAGTAATATAGGGATTGATAACCTTATAGAAGTG

ATTACGAATAAATTTTATTCATCAACACATCGAGGTCAGTCTGAACTTTGCGGAAAAGTT

TTCAAAATTGAGTATTCGGAAAAAAGACAGCGTCTTGCATATATACGTCTTTATAGTGGC

GTACTGCATTTGCGAGATTCGGTTAGAATATCGGAAAAGGAAAAATAAAAATTACAGAAA

TGTATACTTCAATAAATGGTGAATTATGTAAAATCGATAAGGCTTATTCCGGGGAAATTG

TTATTTTGCAGAATGAGTTTTTGAAGTTAAATAGTGTTCTTGGAGATACAAAGCTATTGC

CACAGAGAGAGAGAATTGAAAATCCCCTCCCTCTGCTGCAAACGACTGTTGAACCGAGCA

AACCTCAACAAAGGGAAATGTTACTTGATGCACTTTTAGAAATCTCCGACAGTGACCCGC

TTCTGCGATATTATGTGGATTCTGCGACACATGAAATCATACTTTCTTTCTTAGGGAAAG

TACAAATGGAAGTGACTTGTGCTCTGCTGCAAGAAAAGTATCATGTGGAGATAGAAATAA

AAGAGCCTACAGTCATTTATATGGAAAGACCGTTAAAAAAAGCAGAGTATACCATTCACA

TCGAAGTTCCACCGAATCCTTTCTGGGCTTCCATTGGTCTATCTGTAGCACAGCTTCCAT

TAGGGAGCGGAGTACAGTATGAGAGCTCGGTTTCTCTTGGATACTTAAATCAATCGTTTC

AAAATGCAGTTATGGAGGGGATACGCTATGGCTGTGAACAAGGATTGTATGGTTGGAATG

TGACGGACTGTAAAATCTGTTTTAAGTATGGCTTATACTATAGCCCTGTTAGTACCCCAG

CAGATTTTCGGATGCTTGCTCCTATTGTATTGGAACAAGTCTTAAAAAAAGCTGGAACAG

AATTGTTAGAGCCATATCTTAGTTTTAAAATTTATGCGCCACAGGAATATCTTTCACGAG

CATACAACGATGCTCCTAAATATTGTGCGAACATCGTAGACACTCAATTGAAAAATAATG

AGGTCATTCTTAGTGGAGAAATCCCTGCTCGGTGTATTCAAGAATATCGTAGTGATTTAA

CTTTCTTTACAAATGGACGTAGTGTTTGTTTAACAGAGTTAAAAGGGTACCATGTTACTA

CCGGTGAACCTGTTTGCCAGCCCCGTCGTCCAAATAGTCGGATAGATAAAGTACGATATA

TGTTCAATAAAATAACTTAGTGTATTTTATGTTGTTATATAAATATGGTTTCTTGTTAAA

TAAGATGAAATATTTTTTAATAAAGATTTGAATTAAAGTGTAAAGGAGGAGATAGTTATT

ATAAACTACAAGTGGATATTGTGTCCTGTATGTGGAAATAAAACACGATTAAAGATAAGG

GAAGATACTGAATTAAAAAAATTCCCCCTCTATTGTCCGAAATGCAGACAAGAAAATTTA

ATTGAAATAAAGCAGTTCAAAGTAACTGTGATTACAGAGCCAGACGCAAAGACGCAGAGC

CGATAAAATGAGATTAATACAATCTCATTTTATCGGCTCTTTCCGTTATGTATGGATTCT

TTTAATTAGTCTTCGATGTTTCTTGCTTCGTTGATACCGCTGGCTAAAGATTCCATTAAG

GATAGTTCTTTGTCTGTAAAGCTATCCATGTATTTCTCTATCTGTAATCGTCGGGTGCTT

TTTACCAAGTTATTAGCAGGTAAGAAAAATTCATCAACGGAAACATGAAGTAACGATACA

AGGTCATAAAGAACTTGTATGCTGGGGTGTTGCCCTTTATTTTCAATATTAGTTAAGTAC

CGTGGGTCAATTTCAATCAATGCTCCCACTTGTTCACGAGTTAAACCTCGTTTCAATCGA

GCTTCTTTAATGGCTAAACCAAAGGCTCTAAAATCATATTTATCTTCTTTTTTACGCATA

GTAGACCACCTCTATACATTTTATTGTTCCTACTGAATTAAAAACAGGTATAGAAAAACG

TGTTATATGGTTTATAGGTTTATATTTAATAAAAAGCACTACTAAACGCCAATAAAAAAA

ACCGTTATATGGTAGTGCTATTTACGCTGTTAAAATATTGTATATTACTTCCAAATGGCG

GTTTGTTGGAGGTCAACGTCGCCATGAAGTACATCATATACAATAAATTTCCTTACATTG

GGTTCTTGTCAAAAAAAGTCGTCTATCTGCAATAGATAAGTACGTCCACCAATGTGGTTT

TATAAATCATATAGATAGAATAACAGAAGCATGTAAACAGAGAAATAAATCTGTTTATAT

GCTTTTTTGGCTATTCAGAACTTTTTTACAAAGTTTATTTATCAGTAATGCAACAAATCC

CCCTTTCACATTGGGACTAAGAGTGAAAGGAGATAAACGAGCAAGGCTCACTTCCTTTCC

TAGACAGAAAGGGGGTGAGAAACATGAAACCATCTTCTTTTCAGACCACAATAGAAAATC

AGTTTGACTATATCTGTAAACGTGCTATGGAAGACGAGCGAAAGAATTATATGCTTTATC

TTTCAAGGATTGCAAAGCGTGAGGTGTCCTTTTCGGATGTTGGCGATTATCTTGTTAGCC

AGTTTGCGACAACAGATAACTATTCAACTGACTTTCAGATTTTTACACTCAATGGGTTAT

CAGTAGGCGTTGAAAATGATTTGTTGAGTGAAGCATTACGTGAGTTGCCAGACAAGAAAC

GTGAAATTCTACTGCTGTTTTACTTTATGGACATGAGCGATTCAGAAATTGCAGACCTGT

TGAAATTGAACCGTTCTACTGTCTATCGGCATAGAACCAGTGGACTAGCCTTAATTAAAA

AGTTTATGGAGGAATTTGAAGAATGAAAACACAATATCCTATGATTCCCTTTCCTCTCAT

TGTAAAGGCAACAGATGGCGATACCGAAGCGATTAACCAGATTCTACATCATTACAGAGG

GTACATAACGAAGCGTTCCCTACGACTTATGAAAGATGAATATGGCAATCAAAGTATGGT

CGTTGATGAAGTCTTACGTGGAAGAATGGAAACCAGACTGATTACAAAGATTTTGTCATT

TGAAATTAAGTAATATCCTCTCTCCTTTCGTGGAAGCGTGCTAAACCATTCCACGCTTCC

CGAACAGGGAGGTTTGTTATTCCACCAAAGCATATTGAGCTTTCAATGTGTTTTGATAGG

CTAACGAGCCATTGTTCTTTGAAAACTGAATAAAAGTAATCGAATACGTTTCGATAAGAA

AAGAGCCAACGGAACTAACCGCCATGACCTATCTTATAAAGATAGCGAGCGATTCATGTT

AGTGATCCGAGAAGCAATCTTTAGCAGGATTGCCTGCAACGACATTCTTATCGTGATAAT

GATACTCCCATACAGTCAATAGTCCGAGCGTGATAAAACCGTCGCAGGCAATGAGTATGG

CTACATGAGAACCATGCAGGGGTGGAACTCCCGTGAGCTTTGCTAAAGCTGTTCGATTGC

TGGTAAAACAACTTTTATGAAATCCAAATAAGTGATTTGGAAAGGAGGATTTTATGAAGC

AGACTGACATTCCTATTTGGGAACGTTATACCCTAACCATTGAAGAAGCGTCAAAATATT

TTCGTATTGGCGAAAACAAGCTACGACGCTTGGCAGAGGAAAATAAAAATGCAAATTGGC

TGATTATGAATGGCAATCGTATTCAGATTAAACGAAAACAATTTGAAAAAATTATAGATA

CATTGGACGCAATCTAGCGTCGCCAAAGGGTCTTGTATATGATAAAATAGTATTAAGTCG

TATCAAGGCTCTTTCCATAAAGGAAAGGAGCAAATGCCATGTCAGAAAAAAGACGTGACA

ATAAAGGTCGAATCTTAAAGACTGGAGAGAGCCAACGAAAAGACGGAAGATACTTATACA

AATATATAGATTCATTTGGAGAACCGCAATTTGTTTACTCGTGGAAACTTGTGGCTACAG

ACCGAGTACCAGCAGGAAAGCGTGATTGTATCTCACTTAGAGAGAAAATCGCAGAGTTAC

AGAAAGACATTCATGATGGTATTGATGTTGTAGGAAAGAAAATGACACTCTGCCAGCTTT

ACGCAAAACAGAACGCTCAAAGACCAAAGGTTAGAAAAAACACTGAAACTGGACGCAAAT

ATCTTATGGATATTTTGAAGAAAGACAAGTTAGGTGTAAGAAGTATTGACAGTATTAAGC

CATCAGACGCTAAAGAATGGGCTATTAGAATGAGTGAAAATGGTTATGCTTATCAAACCA

TCAATAACTACAAACGTTCTTTAAAGGCTTCATTCTATATTGCTATACAAGATGATTGTG

TTCGGAAGAATCCATTTGACTTTCAACTGAAAGCAGTTCTTGATGATGATACTGTCCCTA

AAACCGTACTAACAGAAGAACAGGAAGAAAAACTGTTAGCCTTTGCAAAAGCTGATAAAA

CCTACAGCAAAAATTATGATGAAATTCTGATACTCTTAAAAACAGGTCTTCGTATTTCAG

AGTTTGGTGGTTTGACACTTCCAGATTTAGATTTTGAGAATCGTCTTGTCAATATAGACC

ATCAGCTATTGAGAGATACTGAAATTGGGTACTACATTGAAACACCAAAGACCAAAAGTG

GCGAACGTCAAGTTCCTATGGTTGAAGAAGCCTATCAAGCATTTAAGCGAGTGTTAGCGA

ATCGAAAGAATGATAAGCGTGTTGAGATTGATGGATATAGTGATTTCCTCTTTCTTAATA

GAAAGAACTATCCAAAAGTGCAAGTGATTACAACGGCATGATGAAAGGTCTTGTTAAGAA

ATACAATAAGTATAACGAGGATAAATTGCCACACATCACTCCACATAGTTTGCGACATAC

ATTCTGTACCAACTATGCAAATGCAGGAATGAATCCAAAGGCATTACAGTACATTATGGG

ACATGCTAATATAGCCATGACGCTGAACTATTACGCACATGCAACATTCGATTCTGCAAT

GGCAGAAATGAAACGCTTGAATAAAGAGAAGCAACAGGAGCGTCTTGTTGCTTAGTAGTA

CAAATGAATTTACTACTTATTTACCACTTCTGACAGCTAAGACATGAGGAAATATGCAAA

GAAACGTGAAGTATCTTCCTACAGTAAAAATACTCGAAAGCACATAGAATAAGGCTTTAC

GAGCATTTAAGAAAATATAAAAAGATAATTAGAAATTTATACTTTGTTT

>GA11663/Tn2009

ATAAAAATAGCATAAAAATCTAGTTATCCGCATAAAAACTGGACTTATCACACTTTATCA

AGGTCAAAACCACTCAATTTACTACTAATTTACTACTTATGAATGAGCTTTGATACGACG

ATTTATCCTTGAAAAGTGAAGATATAAAGATACTTCCAATAAAATTTGAATATTTAATAG

GTAGACACTTCAAAAAATGAGGTGTCTATTTTTTTACCCGATTTTGAAAGGAAGTGAACT

TATGAAAACAAAAAATCAAGAATCAAAAGGTCGTTCCCCACTCTTTAAGACCATCAAACA

TTCATTCAGCCAATAAAAAAGAAAGGATAGGTAAAAATATGGAACTTAAATTTGTGATTC

CCAACATGGAAAAAACATTCGGCAATTTAGAATTTGCTGGCGAGGATAAAGTCGTTCAGC

GAAGAATCAACGGACGGCTAACTGTCTTATCAAGAAGCTATAATCTCTATTCTGATGTTC

AAAGAGCAGATGATATTGTGGTGGTGCTTCCTGCTGAAGCTGGCGAAAAACATTTCGGCT

TTGAGGAACGTGTGAAGTTAGTCAATCCACGTATTACCGCAGAGGGCTACAAAATCGGCA

CTCGTGGTTTTACAAATTACCTTTTACATGCTGACGACATGATAAAAGAATAAAGAAAGA

GAGGAAAAATGATGAGATTAGCAAATGGCATTGTATTAGATAAAGACACGACTTTTGGAG

AATTGAAATTCTCTGCTCTACGTCGTGAAGTGAGAATCCAAAATGAAGACGGGTCGGTTT

CAGATGAAATCAAGGAACGTACCTATGACTTAAAATCCAAAGGACAAGGACGCATGATTC

AAGTAAGTATTCCTGCCAGCGTGCCTTTGAAAGAGTTTGATTATAACGCACGGGTGGAAC

TTATCAATCCCATTGCGGACACCGTTGCTACTGCCACCTATCAAGGAGCAGATGTTGACT

GGTATATCAAGGCAGACGATATTGTGCTGACAAAGGATTCTAGTTCATTCAAAGCTCAAC

CACAAGCAAAGAAAGAACCGACACAAGACAAATAGTCGCTAGGTAGAAAGGAGACTTTTT

CGCATGAAACAGCGTGGTAAAAGGATTCGCCCATCTGGTAAAGATTTAGTCTTTCATTTT

ACGATAGCGTCACTCCTGCCTGTTTTCCTGCTGGTTGTCGGACTGTTTCATGTGAAGACA

ATCCAGCAGATCAACTGGCAGGATTTTAACCTATCACAAGCAGATAAGATTGACATTCCC

TATTTAATTATCAGTTTCAGTGTCGCAATTCTTATCTGCTTGCTGGTAGCGTTTGTATTC

AAACGGGTTCGCTATGATACGGTTAAACAACTTTACCACCGTCAAAAACTGGCAAAGATG

ATACTTGAAAACAAGTGGTATGAATCTGAACAGGTCAAAACAGAGGGTTTCTTTAAAGAT

AGTGCTGGTCGTACAAAGGAAAAGATAACCTACTTCCCTAAAATGTATTATCGACTTAAA

AATGGCTTGATACAGATACGGGTGGAAATCACGCTGGGAAAATATCAAGACCAACTCTTA

CACTTGGAAAAGAAATTAGAGAGTGGCTTGTACTGTGAGCTGACGGATAAAGAGTTAAAG

GATTCCTATGTGGAATATACTTTGCTCTATGACACCATAGCCAGTCGTATTTCTATTGAT

GAAGTAGAAGCTAAAGATGGTAAACTTCGCTTAATGAAAAACGTATGGTGGGAATATGAT

AAGCTCCCTCATATGTTGATTGCTGGTGGTACAGGTGGCGGTAAAACTTACTTTATACTG

ACACTGATTGAAGCCTTGCTTCATACAGATTCAAAACTGTATATTCTTGACCCGAAAAAT

GCTGACCTTGCGGACTTAGGTTCTGTGATGGCAAATGTCTACTATAGAAAAGAAGACTTG

CTTTCTTGCATTGAAACATTCTATGAAGAAATGATGAAACGTAGTGAGGAAATGAAGCAG

ATGAAGAACTATAAGACTGGCAAAAATTATGCTTACTTAGGTCTCCCGGCACACTTCTTA

ATCTTTGATGAATACGTCGCTTTCATGGAAATGCTGGGAACAAAAGAAAACACCGCAGTT

ATGAATAAGCTGAAACAGATTGTCATGTTAGGTCGTCAAGCTGGCTTCTTTCTAATACTG

GCTTGTCAACGTCCAGACGCAAAATATTTAGGCGACGGAATCCGTGATCAGTTTAATTTC

AGAGTGGCTTTAGGTCGTATGTCTGAAATGGGCTATGGCATGATGTTTGGCAGTGACGTA

CAAAAGGATTTCTTCTTAAAGCGAATCAAAGGTCGTGGCTATGTTGATGTAGGAACAAGT

GTCATATCAGAGTTTTATACTCCCCTTGTACCAAAAGGATATGATTTCTTGGAGGAAATT

AAAAAGTTATCCAACAGCAGACAGTCCACGCAGGCGACGTGCGAAGCGGAAGTCGCAGGT

GTGGACTGATCTTGCTGGCTGGTGTGGCAATAGCCACGCCAGCACTTAACCCCCCGTATC

TAACAGGGGGGTACAAATCGACAGGAAACAGTCAAAAAAACATTAGAAAATCCTTTGGTT

ACAAGGGATTTACAAAATTTCAGCGTATGTCAAATGGGCTTTAAAAGTTGACATACGCCT

TTTTGATTGGAGGGATTTTTACTGAATGAACAAACTTGGTTACAGCATTTAAAAGAAAAA

CGCTTGGCTTATGGACTATCTCAAAACCGTTTAGCTGTTGCGACTGGTATTACAAGGCAG

TATCTAAGCGATATTGAAACAGGAAAAGTCAAGCCATCAGAGGATTTACAGCAGTCCCTT

TGGGAAGCTCTGGAACGCTTCAATCCCGACGCTCCCCTTGAAATGCTGTTTGATTATGTA

AGGATTCGCTTTCCGACAACAGACGTACAGCAGGTGGTCGAAAACATCTTACAACTGAAA

CTGTCCTATTTTCTTCATGAGGACTATGGTTTCTATTCTTATTCAGAGCATTATGCTTTA

GGCGACATATTCGTCCTTTGCTCCCATGAACTGGACAAAGGAGTTCTGGTGGAATTGAAA

GGTCGTGGGTGCAGACAATTTGAAAGCTATCTTCTGGCACAACAAAGAAGCTGGTATGAG

TTCTTTATGGACGTTTTGGTGGCTGGCGGTGTGATGAAACGCCTTGACCTTGCCATTAAC

GATAAGACAGGGATTTTGAATATCCCTGTACTCACTGAAAAGTGCCAACAGGAAGAATGT

ATCTCCGTCTTCCGCAGTTTTAAAAGCTATCGCAGTGGCGAACTGGTACGCAAAGAGGAA

AAGGAATGTATGGGAAACACCCTCTATATCGGTTCATTACAAAGTGAAGTTTATTTCTGT

ATCTATGAAAAGGACTACGAGCAGTACAAGAAAAATGATATTCCCATTGAAGACGCAGAA

GTAAAAAACCGTTTTGAGATTCGATTGAAAAATGAGCGTGCCTATTATGCAGTCCGTGAT

TTACTCGTCTATGACAATCCAGAGCATACCGCCTTTAAAATTATCAATCGGTATATCCGT

TTTGTAGATAAAGACGATTCCAAACCTCGTTCTGATTGGAAACTGAATGAAGAATGGGCT

TGGTTTATTGGGAACAATCGTGAACGATTAAAACTAACCACAAAACCAGAGCCTTACTCC

TTCCAAAGGACGCTGAACTGGCTATCTCATCAAGTTGCCCCGACCTTAAAGGTTGCGATT

AAACTTGATGAAATCAACCAGACGCAGGTTGTAAAAGACATTCTCGACCATGCGAAACTG

ACAGACCGACACAAGCAGATTTTGAAGCAACAGTCAGTAAAAGAACAGGACGTGATAACA

ACAAAAAATAACTCAAATACAAATTCATTGAATATAGAGAGGAGAACATTTTTATGAATT

TTGGACAAAACCTTTATAACTGGTTTCTATCAAACGCTCAATCACTGGTGCTTTTAGCAA

TCGTTGTGATTGGCTTGTATCTTGGCTTCAAGCGTGAGTTTAGCAAACTGATTGGCTTTT

TAATTATTGCGATTATTGCGGTTGGCTTAGTCTTCAACGCTGCTGGAGTAAAAGACATTT

TACTAGAGCTATTCAATCGCATTATTGGTGCTTAAATAAAACCGTTCTTTTGTGGAATAT

AAGTGGTTTTCTTATGTTCCGCAAAGGAATGGTACACCAAACGAAGTGCGGTAGGGATTT

TTGAATCTCTACAAAGAAAGGACGTGAATATATGGACGATATGCAAGTCTATATTGCGAA

TTTAGGCAAATACAATGAGGGCGAATTGGTCGGTGCGTGGTTTACCTTTCCCATTGACTT

TGAGGAAGTCAAAGAGAAAATCGGCTTGAATGATGAATATGAGGAATACGCCATTCATGA

CTACGAGTTACCCTTTACGGTTGACGAATACACTTCCATTGGCGAACTCAATCGACTATG

GGAAATGGTATCGGAATTACCCGAAGAATTACAATCGGAGCTATCTGCTCTGCTCACTCA

TTTTTCAAGCATTGAAGAACTAAGCGAACATCAAGAGGATATTATCATTCATTCCGATTG

TGATGATATGTATGACGTGGCACGCTACTACATTGAAGAAACGGGTGCTTTAGGCGAAGT

ACCAGCTAGTCTTCAAAACTATATTGATTATCAAGCCTATGGTCGGGATTTAGACCTTTC

AGGAACGTTTATCTCAACCAATCATGGGATTTTTGAAATCGTCTATTAAATCTGTCGGTA

CATTACTACTGGCAGATTTTCTATTTTACGGGGTGGCTCAATCAGCTACCCCTATTTTTT

ATGAAAGGATTGATTACATGAAGAAAATACGAAGCTATACCAGTATCTGGTCTGTGGAAA

AGGTACTGTATTCTATCAATGATTTTAGACTTCCGTTTCCCATAACCTTTACGCAAATGA

CATGGTTTGTCGTGTCACTCTTTGCAGTGATGATACTTGGCAACTTGCCCCCTCTTTCCA

TGATAGAGGGAGCATTTCTCAAATACTTTGGGATTCCTGTGGCTTTCACATGGTTTATGT

CTACAAAAACTTTTGATGGTAAAAAGCCTTATGGATTTTTGAAGTCTGTCATTGCTTATG

CACTGCGACCAAAGCTGACCTATGCAGGAAAAAAAGTAACGCTTGGCAGAAACCAGCCAC

AAGAAGCCATTACAGCAGTTAGGAGTGAATTTTATGGCATATCCAATTAAATACATTGAA

AACAATCTCGTCTGGAATAAAGACGGGGAATGTTATGCTTACTATGAGCTTGTTCCTTAC

AATTACTCATTTCTAAGTCCAGAACAGAAAATACAAGTGCATGATTCTTTCAGACAGCTT

ATCGCACAAAATCGTGATGGCAAAATTCATGCTTTACAAATCAGTACAGAATCCAGCATA

CGTTCTGCACAAGAGCGTTCCAAAAATGAAGTCACTGGCAAGCTCAAAGCGGTTGCCTAT

GACAAAATCGACCAACAGACAGACGCTTTAATATCCATGATTGGCGAAAATCAAGTGAAC

TACCGTTTCTTTATCGGCTTTAAGTTGCTTCTCAACGATCAGGAGTTTTCTATGAAAAGT

CTTACCGTTGAAGCAAAAAATGCTTTGTCTGATTTTGTCTATGATGTGAACCATAAGCTG

ATGGGCGATTTTGTTAGTATGAGTAATGATGAAATCCTGCGTTTTCAGAAGATGGAAAAG

CTCTTAGAAAATAAAATCTCTCGTCGTTTCAAAATCCGCAGGTTAGATAAGGACGACTTC

GGCTATCTGATTGAACACCTTTACGGACAGACAGGCACTGCCTATGAAGAGTATGAGTAC

CATCTATCAAAGAAAAAGCTGGATAATGAAACGCTGATTAAATACTATGACTTGATTAAG

CCTACTCGCTGTTTGGTGGAAGAAAAACAGCGATATTTGAAAATCCAGCAGGAAGATGAA

ACCGTCTATGTAGCTTACTTTACCATTAACAGCATTGTCGGAGAACTGGACTTCCCGTCC

TCTGAAATCTTCTACTACCAGCAACAGCAATTTACATTCCCGATTGATACGTCAATGAAT

GTGGAAATTGTAGCGAATCGTAAAGCCCTATCTACTGTCCGCAATAAAAAGAAAGAACTG

AAAGACTTGGATAACCACGCTTGGCAAAGTGATAATGAAACCAGCTCCAATGTGGCGGAA

GCTCTGGAAAGTGTGAATGAGCTGGAAACCAATTTAGACCAAAGCAAGGAATCTATGTAC

AAGCTGTCTTATGTGGTAAGGGTATCAGCAAATGATCTTGACGAACTCAAACGTCGTTGT

AATGAAGTGAAAGATTTTTATGACGATTTAAGCGTAAAACTGGTACGACCATTTGGGGAT

ATGCTCGGCTTACATGAAGAATTTTTACCTGCCAGCAAGCGTTATATGAATGATTATATT

CAATACGTGACCTCTGATTTCCTCGCTGGTTTAGGTTTTGGTGCTACTCAAATGCTGGGG

GAAAATGAGGGGATTTATGTTGGCTACAGCTTAGATACTGGACGCAATGTCTATCTGAAA

CCTGCTCTTGCCAGTCAAGGGGTTAAGGGTTCAGTAACCAATGCGTTAGCGTCGGCTTTT

GTTGGTTCGCTGGGTGGTGGTAAATCCTTTGCGAATAACCTTATCGTCTATTATGCGGTG

CTTTATGGGGCACAAGCAGTGATTGTAGACCCAAAAGCAGAACGTGGCAGATGGAAAGAA

ACCTTGCCAGAGATTTCCCATGAAATCAATATCGTCACTCTGACTTCTGATGAGAAAAAC

AAAGGCTTACTTGACCCTTATGTGATTATGAAAAATCCCAAAGATTCTGAATCACTGGCT

ATTGATATTCTGACATTCCTTACGGGGATTTCCTCTCGTGATGGGGAACGCTTCCCAATC

CTTAGAAAAGCCATTCGTGCAGTAACCAATAGTGAAGTACGAGGGTTGATGAAAGTGATT

GAGGAATTACGGGTTGAGAATACGCCACTAAGTACCAGTATAGCCGACCATATCGAAAGT

TTTACAGACTATGACTTTGCACATTTATTATTCAGTAATGGTTATGTGGAGCAGTCTATC

AGCTTAGAAAAACAACTGAACATTATACAGGTTGCGGACTTGGTACTTCCCGACAAGGAA

ACTTCCTTTGAGGAATATACCACTATGGAGCTTTTATCCGTTGCTATGCTGATTGTCATT

AGTACCTTTGCTTTAGACTTTATCCATACAGACCGAAGCATTTTCAAGATTGTAGATTTA

GACGAAGCATGGAGCTTTTTACAGGTAGCACAAGGAAAAACACTATCTATGAAGCTGGTT

CGGGCTGGTCGTGCTATGAACGCTGGAGTATATTTCGTGACCCAAAATACAGACGACCTC

TTAGATGAAAAACTGAAAAATAACCTCGGCTTAAAATTTGCATTTCGTTCCACTGACCTT

AACGAGATTAAAAAGACCTTAGCCTTTTTTGGTGTAGACCCAGAGGACGAAAACAATCAG

AAGCGATTGCGTGATTTGGAAAACGGGCAATGCCTTATCAGTGATTTATATGGTCGTGTC

GGTGTGATACAGTTCCACCCTGTATTTGAAGAACTGCTCCATGCCTTTGATACCAGACCA

CCTGTGCGAAAAGAGGTGTAAATGTGAAACCATCAATAGTAAACAGAATAAAATCAAACT

GGACGCTGAAACGTCTAGGTAAAGTGGCAATGACAGTGGCTTTCACACTTGTGATTGCCA

TTTTTCTTTTAGCCATGCTGGGAACGGTGGTTCAAGCTGCGGGCTTGGTAGATGATACGG

TCAATGTGGCAAATGAATACAGCCGATACCCACTTGAAAACTATCAACTGGATTTTTATG

TGGATAATAGCTGGGGCTGGCTTCCGTGGAACTGGTCGGACGGGATTGGAAAACAGGTCA

TGTATGGACTATATGCCATTACCAATTTTATTTGGACAATCAGTTTGTATGTTTCCAATG

CGACAGGTTACTTAGTACAGGAAGCCTATTCCTTAGACTTCATTTCCGCTACAGCAGATT

CCATTGGTAAGAATATGCAGACCTTAGCTGGTGTGAGTGCAAACGGATTTTCAACAGAGG

GTTTCTATGTTGGATTCCTCTTACTCTTGATTTTGGTTCTTGGGGTTTATGTTGCCTATA

CGGGACTGATAAAGAGAGAAACCACAAAGGCAATTCATGCCATTATGAATTTTGTGCTGG

TGTTTATCCTATCGGCTTCCTTTATTGCCTACGCTCCCGACTACATTAAAAAAATCAATG

ACTTTTCATCAGACATCAGTAATGCCAGTTTATCACTTGGCACGAAGATTGTCATGCCCC

ATTCCGATAGTCAAGGCAAGGACAGCGTGGACTTAATCAGAGATAGCCTGTTTTCCATAC

AGGTTCAGCAACCGTGGCTACTGCTTCAATACAACAGTTCAGACATTGAAAGTATCGGTA

TTGACCGTGTGGAAAGCCTGCTCTCCACCAGCCCAGATTCCAACAATGGCGAAGACAGAG

AAAAAATTGTTGCGGAAGAAATTGAAGACAGAAGCAATACCAATCTAACCATTACAAAGA

CCATTAACCGTTTAGGTACAGTCTTCTTCCTATTTGTCTTCAATATTGGGATTTCCATAT

TTGTATTCCTATTAACAGGAATCATGATTTTCTCGCAGGTACTTTTTATCATCTATGCTA

TGTTTCTGCCTGTGAGCTTTATTTTAAGCATGATTCCATCATTTGATGGTATGTCAAAAC

GAGCCATAACAAAGCTCTTTAATACCATTTTGACACGAGCTGGAATCACATTGATTATTA

CGACAGCATTTAGTATTTCAACCATGCTCTATACCTTATCGGCTGGTTATCCGTTCTTTT

TGATTGCTTTTCTACAGATTGTGACCTTTGCAGGAATCTACTTCAAGCTGGGCGATTTAA

TGAGTATGTTTTCTCTACAGAGTAACGATTCTCAAAGTGTGGGAAGTCGTGTGATGAGAA

AACCTCGTATGCTTATGCACGCTCACATGCACCGTCTACAGCGGAAACTTGGACGTTCCA

TGACTACTCTAGGGGCTGGGTCTGCCATTGTTACAGGTAAAAAAGGACAGTCGGGTTCGG

GGAGTTCTGCAAGGACACAAGCAGATCACTCCCGACCAGACGGAAAGGAAAAATCAACAC

TTGGAAAACGTATCGGTCAAACCATCGGTACAGTAGCTGATACCAAAGACAGAATGGTAG

ACACTGCTAGTGGTTTGAAAGAACAGGTTAAAGATTTGCCGACCAATGCAAGATATGCAG

TATATCAAGGAAAATCCAAAGTAAAAGAGAATGTCCGTGATTTAACCAGTAGTATTTCTC

AAACCAAAGCGGACAGAGCCAGTGGACGCAAGGAACAGCAGGAACAAAGGCGAAAAACCA

TTGCGAAGCGTCGCTCTGAAATGAAACAGGTCAAACAGAAAAAACAGCCTGCTTCTTCTG

TTCATGAAAGACCGACTACAAGACAAGAACAATATCATGATGAACAGACCTCAAAACAGT

CTAATATTCAGACTTCATATAAGGAATCTCAACAAGCCAAACAAGAGCGTCCAGCAGTTA

AGTCCGATTTTTCAAGTCCAAAAGTGGAACGCCAAGGCAATACCGTTCAAGAAAAAACCG

TTCAAAAGCCAGCAACTTCAACCACTACAGCAGATAGAACTTCACAACGTCCAATCACAA

AAGAACGTCCGTCTACTGTTCAAAGAGTACCACTACAAAATACAAGAAGTAGACCACCAA

TCAAAACCGCCACCATTAAGAAAGTCGGTAAGAAACCATGAAGTTGAAAACTTTAGTGAT

TGGTGGTTCTGGATTATTCTTGATGGTCTTCTCACTGCTTCTGTTTGTTGCCATTTTATT

TTCAGATGAACAGGACAGCGGAATTTCCAATATTCATTATGGAGGTGTGAATGTTTCCGC

AGAAGTGCTGGCTCATAAGCCTATGGTAGAAAAATATGCCAAAGAATATGGCGTTGAAGA

ATATGTCAACATACTTCTTGCGATTATACAGGTGGAATCGGGCGGTACTGCGGAAGATGT

TATGCAGTCCTCGGAATCCCTCGGTCTTCCACCTAATTCATTGAGTACAGAAGAATCCAT

TAAGCAAGGTGTGAAGTATTTCAGTGAATTATTAGCCAGTAGCGAAAGGCTCAGTGTAGA

TTTAGAATCGGTTATCCAGTCCTACAATTATGGTGGTGGTTTCTTAGGGTATGTGGCTAA

TCGTGGAAATAAATATACCTTTGAACTGGCTCAAAGTTTCTCAAAAGAGTATTCAGGTGG

CGAAAAAGTGTCTTACCCCAATCCCATAGCCATACCTATCAATGGGGGCTGGCGATACAA

CTATGGCAATATGTTTTATGTGCAACTGGTAACGCAGTATCTTGTCACAACAGAGTTTGA

TGATGATACGGTACAAGCCATCATGGACGAAGCACTGAAATATGAGGGCTGGCGATACGT

TTACGGTGGAGCTTCCCCGACTACTTCTTTTGATTGTAGCGGACTGACACAATGGACGTA

TGGAAAAGCTGGAATTAACTTACCACGAACCGCACAACAGCAATATGATGTGACCCAGCA

TATCCCACTATCGGAAGCACAAGCTGGCGATTTGGTTTTCTTTCATTCTACCTATAACGC

TGGCTCTTATATTACTCATGTTGGGATATACCTTGGCAATAACCGTATGTTTCATGCAGG

CGACCCAATCGGTTATGCCGACTTAACAAGCCCCTACTGGCAACAGCATTTAGTGGGAGC

AGGACGAATCAAACAATGAGAAAGGAAGATTTAATGATGAAATTTAGAAAAAATCAGAAT

AAAGAAAAACAGATACCAAAGGAAAAGAAACCTCGTGTCTATAAGGTCAATCCTCATAAA

AAGGTTGTGATTGCCTTGTGGGTACTTTTAGGGCTTAGTTTCAGCTTTGCGATATTCAAG

CACTTTACAGCTATAGATACTCATACTATTCACGAAACAACTATCATAGAAAAGGAATAC

GTTGATACTCATCATGTAGAAAATTTTGTAGAGAACTTTGCGAAAGTCTACTATTCATGG

GAGCAATCCGATAAGTCCATTGATAATCGAATGGAAAGTCTAAAAGGCTATCTGACAGAT

GAACTTCAAGCTCTCAATGTTGATACAGTACGCAAAGATATTCCTGTATCGTCTTCTGTA

AGAGGATTTCAGATATGGACGGTAGAGCCAACTGGCGACAATGAGTTTAATGTAACCTAC

AGTGTAGACCAGCTCATTACAGAGGGAGAAAATACAAAGACCGTCCACTCTGCTTATATA

GTGAGTGTCTATGTAGATGGTTCTGGAAATATGGTACTGGTTAAGAATCCGACCATTACC

AACATACCTAAGAAATCAAGTTATAAACCAAAAGCCATTGAAAGTGAGGGGACGGTTGAT

TCCATTACAACCAATGAAATCAATGAGTTTTTAACGACGTTCTTCAAGCTCTATCCTACA

GCGACAGCCAGTGAACTTTCCTACTATGTGAATGACGGGATATTAAAACCAATCGGAAAA

GAGTACATCTTTCAAGAACTGGTAAATCCTATTCACAATCGTAAGGATAATCAAGTCACG

GTATCGCTGACAGTGGAGTATATCGACCAGCAGACCAAAGCAACGCAGGTATCTCAATTT

GATTTGGTACTTGAAAAGAACGGGAGTAATTGGAAGATTATAGAATAACAAATATTGGTA

CATTATTACAGCTATTTTGTAATCACGTACTCTCTTTGATAAAAAATTGGAGATTCCTTT

ACAAATATGCTCTTATGTGCTATTATTTAAGTATCTATTTAAAAGGAGTTAATAAATATG

CGGCAAGGTATTCTTAAATAAACTGTCAATTTGATAGTGGGAACAAATAATTGGATGTCC

TTTTTTAGGAGGGCTTAGTTTTTTGTACCCAGTTTAAGAATACCTTTATCATGTGATTCT

AAAGTATCCGGAGAATATCTGTATGCTTTGTATGCCTATGGTTATGCATAAAAATCCCNN

NNNCACACACTTAATTAATTAAGTGTGTGNNNNNTGCCCTTTTGGGTTTTTGAATGGAGG

AAAATCACATGAAAATTATTAATATTGGAGTTTTAGCTCATGTTGATGCAGGAAAAACTA

CCTTAACAGAAAGCTTATTATATAACAGTGGAGCGATTACAGAATTAGGAAGCGTGGACA

AAGGTACAACGAGGACGGATAATACGCTTTTAGAACGTCAGAGAGGAATTACAATTCAGA

CAGGAATAACCTCTTTTCAGTGGGAAAATACGAAGGTGAACATCATAGACACGCCAGGAC

ATATGGATTTCTTAGCAGAAGTATATCGTTCATTATCAGTTTTAGATGGGGCAATTCTAC

TGATTTCTGCAAAAGATGGCGTACAAGCACAAACTCGTATATTATTTCATGCACTTAGGA

AAATGGGGATTCCCACAATCTTTTTTATCAATAAGATTGACCAAAATGGAATTGATTTAT

CAACGGTTTATCAGGATATTAAAGAGAAACTTTCTGCCGAAATTGTAATCAAACAGAAGG

TAGAACTGTATCCTAATATGTGTGTGACGAACTTTACCGAATCTGAACAATGGGATACGG

TAATAGAGGGAAACGATGACCTTTTAGAGAAATATATGTCCGGTAAATCATTAGAAGCAT

TGGAACTCGAACAAGAGGAAAGCATAAGATTTCAGAATTGTTCTCTGTTCCCTCTTTATC

ATGGAAGTGCAAAAAGTAATATAGGGATTGATAACCTTATAGAAGTGATTACGAATAAAT

TTTATTCATCAACACATCGAGGTCAGTCTGAACTTTGCGGAAAAGTTTTCAAAATTGAGT

ATTCGGAAAAAAGACAGCGTCTTGCATATATACGTCTTTATAGTGGCGTACTGCATTTGC

GAGATTCGGTTAGAATATCGGAAAAGGAAAAATAAAAATTACAGAAATGTATACTTCAAT

AAATGGTGAATTATGTAAAATCGATAAGGCTTATTCCGGGGAAATTGTTATTTTGCAGAA

TGAGTTTTTGAAGTTAAATAGTGTTCTTGGAGATACAAAGCTATTGCCACAGAGAGAGAG

AATTGAAAATCCCCTCCCTCTGCTGCAAACGACTGTTGAACCGAGCAAACCTCAACAAAG

GGAAATGTTACTTGATGCACTTTTAGAAATCTCCGACAGTGACCCGCTTCTGCGATATTA

TGTGGATTCTGCGACACATGAAATCATACTTTCTTTCTTAGGGAAAGTACAAATGGAAGT

GACTTGTGCTCTGCTGCAAGAAAAGTATCATGTGGAGATAGAAATAAAAGAGCCTACAGT

CATTTATATGGAAAGACCGTTAAAAAAAGCAGAGTATACCATTCACATCGAAGTGCCGCC

AAATCCTTTCTGGGCTTCCATTGGTTTATCTGTATCACCGCTTCCGTTGGGAAGTGGAAT

GCAGTATGAGAGCTCGGTTTCTCTTGGATACTTAAATCAATCATTTCAAAATGCAGTTAT

GGAAGGGATACGCTATGGTTGCGAACAAGGATTATATGGTTGGAATGTGACGGACTGTAA

AATCTGTTTTAAGTATGGCTTATACTATAGCCCTGTTAGTACCCCAGCAGATTTTCGGAT

GCTTGCTCCTATTGTATTGGAACAAGTCTTAAAAAAAGCTGGAACAGAATTGTTAGAGCC

ATATCTTAGTTTTAAAATTTATGCGCCACAGGAATATCTTTCACGAGCATACAACGATGC

TCCTAAATATTGTGCGAACATCGTAGACACTCAATTGAAAAATAATGAGGTCATTCTTAG

TGGAGAAATCCCTGCTCGGTGTATTCAAGAATATCGTAGTGATTTAACTTTCTTTACAAA

TGGACGTAGTGTTTGTTTAACAGAGTTAAAAGGGTACCATGTTACTACCGGTGAACCTGT

TTGCCAGCCCCGTCGTCCAAATAGTCGGATAGATAAAGTACGATATATGTTCAATAAAAT

AACTTAGTGTATTTTATGTTGTTATATAAATATGGTTTCTTGTTAAATAAGATGAAATAT

TTTTTAATAAAGATTTGAATTAAAGTGTAAAGGAGGAGATAGTTATTATAAACTACAAGT

GGATATTGTGTGCTGAGAGCTTTCTTCTATACTAATAGACGAAAGGGTGTGAAAATGATT

TTTAAATGATACTGTGGAACGGAACAGTAGCCCTAGTATTGACTACTGTCGTTTCTATTC

ATATTGGCTATTCTAGGACTGAGATGAAAAAATCTATAAATGCTCAGAATAAAATTGAAC

CCGCAAATCTCCCCAAAACAATGGTGAGTCATGTACTTGTATTATTCCGAAAAAATACAC

CTCTGGTGCAGTGAGACAAATTGGTGTATCTTATAGTGGCTTCGTAGATGAAAGCTATAC

TCTACTATCACTCTTTGATGATGTAGAACAAATTGAAAAAGATAATAGACTTCAGACAGC

TATTGATGTTGTCAGAGAACAGTTTGGTTTTTTAGCCATACAAAAAGGAACCGTCCTAAC

TGAAGGTTCCAGAAATATTGAACGCAGTAAACTTATCGGTGGTCATTCCGCGGGTGGATT

GGAGGGATTAAAATGAAACAAGAAAAAAATACAGTACAATTTTCAGAAATCCGTAGCAAA

GGATGTAATGATATTGAAATGCTTGAAAGATTTTTACATGGAATCGTTGAAACAGCAACT

TCAAAACTTCGTCAGAGAAAACTCAAAACAACTGAAATATCGATACGACTAGTACATGCT

AAATCTGAAAACCGATTACCATTGGAATTTACATTTAGCATTAAGCCAACAAGCTCATCT

GTGATAATCTATACTGAGGTAATCAATCGCTTTAAAGAATGTTACACAGGTGGGGGAATT

CAAGGTTTTACGATTCAATTTGATAAAAATACCCTTGCCTCTGCATAGAAAGGATTTGAT

ATGATTGACCGTTCATATTTACCATTTCAATCAGCAAGAGAGTACCAGGATACAAAGATG

CAAAAATGGATGGGCTTTTTCCTATCTGAACATGCATCAGCACTCTCTGATGATACAAAC

AAAGTAACGTACATGTCTGACTTATCACTAGAGAAGAAATTATTACTCCTCAGTCAAGTA

TACGCCGGGCAGCTACGCACACGCATTCAAGTGATTGAAAAAAACAAGCGTGTTTCCTAC

ACTGGAACAATACCAAGTCTGACCAAAGATTTCATTTTGATAAAAACTACAACAGGTCAC

ATCAATTTGAAATTAAAAGACATTATTAGTATTGAACTTGTCGAGGAGGTGCTCTATGAA

TCAGCTTGAGTTTCAGCGTAATCACCTACAAATGGACTATTATAGCGAGAGCTACCAAGA

TTTTGAACGTGACTTCTACCGCTACTCTAACATGAATATTCCATTGACCTTCCTAACTGA

TGATATCCTAAAAACAATGGCGACTTCACGTAAGAATTACTTTGTCCTCAATAAGGAAAA

GTCCAGAGATAACCGCGATCACTTCTTCATATTTGAAGTAAGTACCGTAGATGAGAATCC

GCTAATCTATCATTATACATATAAGAAAACTACAATATATTTAGCAGAAAAATAGGAGCA

GTTCAATTGACTGTTCCTATTTTTAATATTCATAAAATCTAAAGTCTTTATACTCTTTAA

CAATGGAGTCGCCAACCAGAACAGACTATACTGACCAGCGACTACCTTAAATTTAATGTT

TCAGATTTATTTTCTTATCTCTAATTTCATAAACTACATCTGCTACATTTTCGAGTAATC

GTTTATCGTGGGTGATAAACACGATAGTTCCGGTGTACTCCTTCATTAGTATTTCCAAAG

CCTCTAAACTTGGTATGTCAAGGAAGTTACTGGGTTCATCCATTATTAGGATGTTATATC

TACCCATGAGCATTTTAGCAAGCAACAATTTTATAATTTCTCCACCGCTTAAAACAGATA

AACTTTTTCCAATATCGTTCTGTTTGAACCCCATAGATGCTAGCACTGAACGAATTTCTG

ATATATTGTAGTCACAATCCTTCTGCATAAACTCCATAACATTCTGATTACTGTTGTACT

TGTAACCATTCTGTGCAAAGTAACCTATTTTTGCCTTAGGCGAAATAGAAATTCCTTCTT

CATGGTTTAAGATCATTTGGATTAAAGTTGTTTTTCCGATTCCATTACCACCAGTTAACG

CCACTTTTGCTCCTAACGGAATTTGAAAAGATGCATTTTCAAACAGAGCCTTATCCCCAA

ATACTTTATTAATTTCTGCACCGACTATAGGGTATGGATTATGGAGCTCCAATGCTTTAC

TTTGCCTGAAACGAATTCTGCGAATGCCTTCCGGAGCTTCTACTTTTCCTAAGGCCGCAA

TCCTGTGCTCTAGGGTTTTAGCAGCATTATACATCTTTTTTTCCTTACTTCCTATTGATT

TTTGATGAGCTAAACGCCCTCCGTCTTCAGTACTTTTTTTCTTTGAAGAACCTTTTGCCT

TCTGTTCTATTTTACGAGCCTGTTTTCGCTTTTCCTCCGCAGCCCTTTCCAATCGGGCAC

GTTCCGCAATAAATTGTTCGTATTCTGCAGCTTGGCTCTTACGTTCTTCCTCTTTCTGAC

GAAGATAATCAGAATAGTTTCCCCAATACTCAGTGATTTTGCCATCTTTCAGTTCCCATA

TTTTATCTACTATTTCATCAAGAAAATAGCGGTCATGGCTAATAACTAACAGTGCACCTG

TAAAATATTTTAGCTGTCCTATTAGAAAATCAATTCCTTCACGGTCTAAATGGCTCGTAG

GTTCATCCGCTAAAATACCATGAACCTGTGCCGATAAGGCCTGTGCTATTTTAAGCCTTG

TTTCTTCACCACCGCTCATAGTCTGTATATTTAATTGCTCAACACCTAGCTTGCCTACAA

GTGCAAAATCTTTTTCCTCCTGCAGAGTTACTTCGTCCAACTGGGGAATATAGGCAAGTT

CACCCAGACGATTCATTTTACATCCTGGGGGAGTTAATTCTCCTAAAAGTACCCTGAGTA

AAGTGCTTTTTCCAGCACCATTTGCTCCTACTAAACCAATACGGTCATAATCATATACTT

CTAATTCATTTATATCTAAAACATCGCGTCCTTTGAATTCCACACGAATGTCTTTTGCTT

TTAATATTAATTCCATAACATTTCCTCCTGTCTATAATCGCATGCTTTCATTTGCTTGTA

TGCAGGGAAAACCCTGCGATTTTAGCAGGAAGAGTTACATGAAAATAAGATACATAAATA

TTCCTCCAATATTGTTTATTTTAAATCTAATTTTCTAACCTCAGTTATCATTTGGCAAAC

TATAGCAATGCCAATAATTAAAATACCTGATAGTAAAAACCAATGATTTACACCGATTTT

ATCAGCAAAGAATCCAGAAAGAATTAACCCAATTGGCATAGCAAGTGACATGATACTTCC

GATCAAAGAAAATACACGTCCTAAATATTCAGGCTTAATTTTCTCCTGAAAAAGAGCTGT

TTGCACACCGCTATAAAATGGCACCGAAAGCCCCATTATTGCACAGCAAACTACGAATAT

TACAAATCCATTTGGAGGAAGTATTCCCGAAACGGCTAAACTGGTCCCCATTATAAAAAA

TGAACTTGTTATTAGTAATACATGCTTTTCGAAGCCCCCTAATCTTCCTAATAATAAGCC

TCCTGCTAGCATCCCAAATGCAAAGGAAATTTCCGTAATAGAAATATGCACAGGCGTTCC

ATTAAAGTGTTCCATGCTTATTAAAGGAAATAGTGCATTGATTGGCATATAAACAAAAGT

ATATAGTGTTCCTAAGAGTAATAAGGCAAACAATCCTTTGTTTTGTCTCAGAACCACAAC

TCCTTCTTTCATCTCCCTTATGAAATTTGGTTCTAAACTTTGCACTTGATTACCCAGCTT

AGGTATACGTACAATTGCTACCGTAATAGATGCAATCACAGCACCCAATACGTCGATGGC

AATAATAGCATTTAAATCCCAAACGGAGTATAAGAGTGCTGCAACTGCCGGACTAACAAT

ATAGCTTATAGACTGCAAAGACTGACTATAGCCTGCGCATTTCGTTAGCTGTTCTTCTGG

TACTAAAAGTGGTGTAACCGCATTGAGTGCTGGGGTATGAAAAGCTGTTCCAATGCTACG

GATAAACAATACTATCATAATCATCCAGACAGGTAGCTCCATACAGAATGCAACAATAGC

AAGCACTGCACCAGCTGCTGCGATAATTAAATCGGCACCAATCATTATCTTCTTCCTATC

ATGACGATCCACTAGCACACCAATGGCAGGTCCCAAAATCGCATAGGGTAAAAAACCTAC

TAATGAAGCCATAGACAAGACCATCGCAGATCCTGTTTTTTCTGTAAGGTAAAAAATAAT

CGCCATTTGCAGGATGGCACTAGTGATTAATGATACTGCTTGCCCTGCCCATATTGCATA

AAATTTTCGTTTCCAATTGTTGTATTTTTCCATTTATATTATCTCCTGCATATTATTTTG

CTTGAATTTCTATTTTGAATAGCATTCTAGGCAATAAAAAATGCAGGCCAAACCCCACAA

TGTGGCTTTTGGTCTGCATACATACAATTTGGAAACATTCATATTAAAGACATAGTTAAA

TAAAGGTATAGTTAAATAACCAATATCCTCACCGTAACTAATGAATGCTCAATATCGTAT

AAATAAGCACAACAAAAAAGCCTATCATCGGGTATAGATTCTGCTTTTTTTATTGCCAGC

TTATCTTAAACGCATTGAGGCTGTCATAGTTTCGGTTCCTCCTACATCTTTGTTTATATC

AATTTATAGTATAACACAACAAGATGATATGTTCAATATAAAAGTTATGGAATGAGACTC

ATACTTCCAATTCGATGCCAGATTTAAAGGATATGACGAAGTTTTCTTCATAGACTGTAA

CGCTCTGGATTATCTTCCTTAGTAGCAAGCGATTAGCTTTCACAAAATCTTCTGTTTGTA

GTTTTAAAAATTCATCAGGATTTTCTAACTCAACCTCAAAATATTTCATTTTACATTCCC

TCATTTCATTTATTGATAAATTGAGTTTGCAAAAAAGAGTGGACAATTTTTGTCTACTCT

TAACCTTTAAAATAGTTTTTTTTAATCGATTTGAAGTTGCCTAAATTATTACTTATTCGG

TAAAATGAAGTATTGCTTTCAACAGATTTCCTTCAACTACACTTCACTTGATTCAAACAA

GGTGGGTACATTTCTATTCCCACAAACTCCTTGTCAATGGAAACAAACACGTACCCACAG

GGTAAATGGAAATAGAAACTGATAATTTCTAGCTATCACTTCTACTCATTCCAAAAATTT

TCTCACTCTGATACTTACCCACCATAAAGCAAAAAGCCTTGCAATCAAGGCTTTCATTAT

CCCTTTCGTTCAAAGGTTTCTAAGCTTTTACGAGCAGAGCGACACACTCAGCGGTTCGCT

ATCTCCGTTCTGTCTGCGTGCTAGCACTTGTCAATCACGGACAGCTATCGCATGGGCGGA

AGTAAATGCTAATCTTCGTCGTTTTACTCCTTGACTAGCAAACTTACCGCCTCAACATGT

CCTGTATGTGGAAATAAAACACGATTAAAGATAAGGGAAGATACTGAATTAAAAAAATTC

CCCCTCTATTGTCCGAAATGCAGACAAGAAAATTTAATTGAAATAAAGCAGTTCAAAGTA

ACTGTGATTACAGAGCCAGACGCAAAGACGCAGAGCCGATAAAATGAGATTAATACAATC

TCATTTTATCGGCTCTTTCCGTTATGTATGGATTCTTTTAATTAGTCTTCGATGTTTCTT

GCTTCGTTGATACCGCTGGCTAAAGATTCCATTAAGGATAGTTCTTTGTCTGTAAAGCTA

TCCATGTATTTCTCTATCTGTAATCGTCGGGTGCTTTTTACCAAGTTATTAGCAGGTAAG

AAAAATTCATCAACGGAAACATGAAGTAACGATACAAGGTCATAAAGAACTTGTATGCTG

GGGTGTTGCCCTTTATTTTCAATATTAGTTAAGTACCGTGGGTCAATTTCAATCAATGCT

CCCACTTGTTCACGAGTTAAACCTCGTTTCAATCGAGCTTCTTTAATGGCTAAACCAAAG

GCTCTAAAATCATATTTATCTTCTTTTTTACGCATAGTAGACCACCTCTATACATTTTAT

TGTTCCTACTGAATTAAAAACAGGTATAGAAAAACGTGTTATATGGTTTATAGGTTTATA

TTTAATAAAAAGCACTACTAAACGCCAATAAAAAAACCGTTATATGGTAGTGCTATTTAC

GCTGTTAAAATATTGTATATTACTTCCAAATGGCGGTTTGTTGGAGGTCAACGTCGCCAT

GAAGTACATCATATACAATAAATTTCCTTACATTGGGTTCTTGTCAAAAAAAGTCGTCTA

TCTGCAATAGATAAGTACGTCCACCAATGTGGTTTTATAAATCATATAGATAGAATAACA

GAAGCATGTAAACAGAGAAATAAATCTGTTTATATGCTTTTTTGGCTATTCAGAACTTTT

TTACAAAGTTTATTTATCAGTAATGCAACAAATCCCCCTTTCACATTGGGACTAAGAGTG

AAAGGAGATAAACGAGCAAGGCTCACTTCCTTTCCTAGACAGAAAGGGGGTGAGAAACAT

GAAACCATCTTCTTTTCAGACCACAATAGAAAATCAGTTTGACTATATCTGTAAACGTGC

TATGGAAGACGAGCGAAAGAATTATATGCTTTATCTTTCAAGGATTGCAAAGCGTGAGGT

GTCCTTTTCGGATGTTGGCGATTATCTTGTTAGCCAGTTTGCGACAACAGATAACTATTC

AACTGACTTTCAGATTTTTACACTCAATGGGTTATCAGTAGGCGTTGAAAATGATTTGTT

GAGTGAAGCATTACGTGAGTTGCCAGACAAGAAACGTGAAATTCTACTGCTGTTTTACTT

TATGGACATGAGCGATTCAGAAATTGCAGACCTGTTGAAATTGAACCGTTCTACTGTCTA

TCGGCATAGAACCAGTGGACTAGCCTTAATTAAAAAGTTTATGGAGGAATTTGAAGAATG

AAAACACAATATCCTATGATTCCCTTTCCTCTCATTGTAAAGGCAACAGATGGCGATACC

GAAGCGATTAACCAGATTCTACATCATTACAGAGGGTACATAACGAAGCGTTCCCTACGA

CTTATGAAAGATGAATATGGCAATCAAAGTATGGTCGTTGATGAAGTCTTACGTGGAAGA

ATGGAAACCAGACTGATTACAAAGATTTTGTCATTTGAAATTAAGTAATATCCTCTCTCC

TTTCGTGGAAGCGTGCTAAACCATTCCACGCTTCCCGAACAGGGAGGTTTGTTATTCCAC

CAAAGCATATTGAGCTTTCAATGTGTTTTGATAGGCTAACGAGCCATTGTTCTTTGAAAA

CTGAATAAAAGTAATCGAATACGTTTCGATAAGAAAAGAGCCAACGGAACTAACCGCCAT

GACCTATCTTATAAAGATAGCGAGCGATTCATGTTAGTGATCCGAGAAGCAATCTTTAGC

AGGATTGCCTGCAACGACATTCTTATCGTGATAATGATACTCCCATACAGTCAATAGTCC

GAGCGTGATAAAACCGTCGCAGGCAATGAGTATGGCTACATGAGAACCATGCAGGGGTGG

AACTCCCGTGAGCTTTGCTAAAGCTGTTCGATTGCTGGTAAAACAACTTTTATGAAATCC

AAATAAGTGATTTGGAAAGGAGGATTTTATGAAGCAGACTGACATTCCTATTTGGGAACG

TTATACCCTAACCATTGAAGAAGCGTCAAAATATTTTCGTATTGGCGAAAACAAGCTACG

ACGCTTGGCAGAGGAAAATAAAAATGCAAATTGGCTGATTATGAATGGCAATCGTATTCA

GATTAAACGAAAACAATTTGAAAAAATTATAGATACATTGGACGCAATCTAGCGTCGCCA

AAGGGTCTTGTATATGATAAAATAGTATTAAGTCGTATCAAGGCTCTTTCCATAAAGGAA

AGGAGCAAATGCCATGTCAGAAAAAAGACGTGACAATAAAGGTCGAATCTTAAAGACTGG

AGAGAGCCAACGAAAAGACGGAAGATACTTATACAAATATATAGATTCATTTGGAGAACC

GCAATTTGTTTACTCGTGGAAACTTGTGGCTACAGACCGAGTACCAGCAGGAAAGCGTGA

TTGTATCTCACTTAGAGAGAAAATCGCAGAGTTACAGAAAGACATTCATGATGGTATTGA

TGTTGTAGGAAAGAAAATGACACTCTGCCAGCTTTACGCAAAACAGAACGCTCAAAGACC

AAAGGTTAGAAAAAACACTGAAACTGGACGCAAATATCTTATGGATATTTTGAAGAAAGA

CAAGTTAGGTGTAAGAAGTATTGACAGTATTAAGCCATCAGACGCTAAAGAATGGGCTAT

TAGAATGAGTGAAAATGGTTATGCTTATCAAACCATCAATAACTACAAACGTTCTTTAAA

GGCTTCATTCTATATTGCTATACAAGATGATTGTGTTCGGAAGAATCCATTTGACTTTCA

ACTGAAAGCAGTTCTTGATGATGATACTGTCCCTAAGACCGTACTAACAGAAGAACAGGA

AGAAAAACTGTTAGCCTTTGCAAAAGCTGATAAAACCTACAGCAAAAATTATGATGAAAT

TCTGATACTCTTAAAAACAGGTCTTCGTATTTCAGAGTTTGGTGGTTTGACACTTCCAGA

TTTAGATTTTGAGAATCGTCTTGTCAATATAGACCATCAGCTATTGAGAGATACTGAAAT

TGGGTACTACATTGAAACACCAAAGACCAAAAGTGGCGAACGTCAAGTTCCTATGGTTGA

AGAAGCCTATCAAGCATTTAAGCGAGTGTTAGCGAATCGAAAGAATGATAAGCGTGTTGA

GATTGATGGATATAGTGATTTCCTCTTTCTTAATAGAAAGAACTATCCAAAAGTGGCAAG

TGATTACAACGGCATGATGAAAGGTCTTGTTAAGAAATACAATAAGTATAACGAGGATAA

ATTGCCACACATCACTCCACATAGTTTGCGACATACATTCTGTACCAACTATGCAAATGC

AGGAATGAATCCAAAGGCATTACAGTACATTATGGGACATGCTAATATAGCCATGACGCT

GAACTATTACGCACATGCAACATTCGATTCTGCAATGGCAGAAATGAAACGCTTGAATAA

AGAGAAGCAACAGGAGCGTCTTGTTGCTTAGTAGTACAAATGAATTTACTACTTATTTAC

CACTTCTGACAGCTAAGACATGAGGAAATATGCAAAGAAACGTGAAGTATCTTCCTACAG

TAAAAATACTCGAAAGCACATAGAATAAGGCTTTACGAGCATTTAAGAAAATATAAAAAG

ATAATTAGAAATTTATACTTTGTTT

>GA13430/Tn2009

AAAATAGCATAAAAATCTAGTTATCCGCATAAAAACTGGACTTATCACACTTTATCAAGG

TCAAAACCACTCAATTTACTACTAATTTACTACTTATGAATGAGCTTTGATACGACGATT

TATCCTTGAAAAGTGAAGATATAAAGATACTTCCAATAAAATTTGAATATTTAATAGGTA

GACACTTCAAAAAATGAGGTGTCTATTTTTTTACCCGATTTTGAAAGGAAGTGAACTTAT

GAAAACAAAAAATCAAGAATCAAAAGGTCGTTCCCCACTCTTTAAGACCATCAAACATTC

ATTCAGCCAATAAAAAAGAAAGGATAGGTAAAAATATGGAACTTAAATTTGTGATTCCCA

ACATGGAAAAAACATTCGGCAATTTAGAATTTGCTGGCGAGGATAAAGTCGTTCAGCGAA

GAATCAACGGACGGCTAACTGTCTTATCAAGAAGCTATAATCTCTATTCTGATGTTCAAA

GAGCAGATGATATTGTGGTGGTGCTTCCTGCTGAAGCTGGCGAAAAACATTTCGGCTTTG

AGGAACGTGTGAAGTTAGTCAATCCACGTATTACCGCAGAGGGCTACAAAATCGGCACTC

GTGGTTTTACAAATTACCTTTTACATGCTGACGACATGATAAAAGAATAAAGAAAGAGAG

GAAAAATGATGAGATTAGCAAATGGCATTGTATTAGATAAAGACACGACTTTTGGAGAAT

TGAAATTCTCTGCTCTACGTCGTGAAGTGAGAATCCAAAATGAAGACGGGTCGGTTTCAG

ATGAAATCAAGGAACGTACCTATGACTTAAAATCCAAAGGACAAGGACGCATGATTCAAG

TAAGTATTCCTGCCAGCGTGCCTTTGAAAGAGTTTGATTATAACGCACGGGTGGAACTTA

TCAATCCCATTGCGGACACCGTTGCTACTGCCACCTATCAAGGAGCAGATGTTGACTGGT

ATATCAAGGCAGACGATATTGTGCTGACAAAGGATTCTAGTTCATTCAAAGCTCAACCAC

AAGCAAAGAAAGAACCGACACAAGACAAATAGTCGCTAGGTAGAAAGGAGACTTTTTCGC

ATGAAACAGCGTGGTAAAAGGATTCGCCCATCTGGTAAAGATTTAGTCTTTCATTTTACG

ATAGCGTCACTCCTGCCTGTTTTCCTGCTGGTTGTCGGACTGTTTCATGTGAAGACAATC

CAGCAGATCAACTGGCAGGATTTTAACCTATCACAAGCAGATAAGATTGACATTCCCTAT

TTAATTATCAGTTTCAGTGTCGCAATTCTTATCTGCTTGCTGGTAGCGTTTGTATTCAAA

CGGGTTCGCTATGATACGGTTAAACAACTTTACCACCGTCAAAAACTGGCAAAGATGATA

CTTGAAAACAAGTGGTATGAATCTGAACAGGTCAAAACAGAGGGTTTCTTTAAAGATAGT

GCTGGTCGTACAAAGGAAAAGATAACCTACTTCCCTAAAATGTATTATCGACTTAAAAAT

GGCTTGATACAGATACGGGTGGAAATCACGCTGGGAAAATATCAAGACCAACTCTTACAC

TTGGAAAAGAAATTAGAGAGTGGCTTGTACTGTGAGCTGACGGATAAAGAGTTAAAGGAT

TCCTATGTGGAATATACTTTGCTCTATGACACCATAGCCAGTCGTATTTCTATTGATGAA

GTAGAAGCTAAAGATGGTAAACTTCGCTTAATGAAAAACGTATGGTGGGAATATGATAAG

CTCCCTCATATGTTGATTGCTGGTGGTACAGGTGGCGGTAAAACTTACTTTATACTGACA

CTGATTGAAGCCTTGCTTCATACAGATTCAAAACTGTATATTCTTGACCCGAAAAATGCT

GACCTTGCGGACTTAGGTTCTGTGATGGCAAATGTCTACTATAGAAAAGAAGACTTGCTT

TCTTGCATTGAAACATTCTATGAAGAAATGATGAAACGTAGTGAGGAAATGAAGCAGATG

AAGAACTATAAGACTGGCAAAAATTATGCTTACTTAGGTCTCCCGGCACACTTCTTAATC

TTTGATGAATACGTCGCTTTCATGGAAATGCTGGGAACAAAAGAAAACACCGCAGTTATG

AATAAGCTGAAACAGATTGTCATGTTAGGTCGTCAAGCTGGCTTCTTTCTAATACTGGCT

TGTCAACGTCCAGACGCAAAATATTTAGGCGACGGAATCCGTGATCAGTTTAATTTCAGA

GTGGCTTTAGGTCGTATGTCTGAAATGGGCTATGGCATGATGTTTGGCAGTGACGTACAA

AAGGATTTCTTCTTAAAGCGAATCAAAGGTCGTGGCTATGTTGATGTAGGAACAAGTGTC

ATATCAGAGTTTTATACTCCCCTTGTACCAAAAGGATATGATTTCTTGGAGGAAATTAAA

AAGTTATCCAACAGCAGACAGTCCACGCAGGCGACGTGCGAAGCGGAAGTCGCAGGTGTG

GACTGATCTTGCTGGCTGGTGTGGCAATAGCCACGCCAGCACTTAACCCCCCGTATCTAA

CAGGGGGGTACAAATCGACAGGAAACAGTCAAAAAAACATTAGAAAATCCTTTGGTTACA

AGGGATTTACAAAATTTCAGCGTATGTCAAATGGGCTTTAAAAGTTGACATACGCCTTTT

TGATTGGAGGGATTTTTACTGAATGAACAAACTTGGTTACAGCATTTAAAAGAAAAACGC

TTGGCTTATGGACTATCTCAAAACCGTTTAGCTGTTGCGACTGGTATTACAAGGCAGTAT

CTAAGCGATATTGAAACAGGAAAAGTCAAGCCATCAGAGGATTTACAGCAGTCCCTTTGG

GAAGCTCTGGAACGCTTCAATCCCGACGCTCCCCTTGAAATGCTGTTTGATTATGTAAGG

ATTCGCTTTCCGACAACAGACGTACAGCAGGTGGTCGAAAACATCTTACAACTGAAACTG

TCCTATTTTCTTCATGAGGACTATGGTTTCTATTCTTATTCAGAGCATTATGCTTTAGGC

GACATATTCGTCCTTTGCTCCCATGAACTGGACAAAGGAGTTCTGGTGGAATTGAAAGGT

CGTGGGTGCAGACAATTTGAAAGCTATCTTCTGGCACAACAAAGAAGCTGGTATGAGTTC

TTTATGGACGTTTTGGTGGCTGGCGGTGTGATGAAACGCCTTGACCTTGCCATTAACGAT

AAGACAGGGATTTTGAATATCCCTGTACTCACTGAAAAGTGCCAACAGGAAGAATGTATC

TCCGTCTTCCGCAGTTTTAAAAGCTATCGCAGTGGCGAACTGGTACGCAAAGAGGAAAAG

GAATGTATGGGAAACACCCTCTATATCGGTTCATTACAAAGTGAAGTTTATTTCTGTATC

TATGAAAAGGACTACGAGCAGTACAAGAAAAATGATATTCCCATTGAAGACGCAGAAGTA

AAAAACCGTTTTGAGATTCGATTGAAAAATGAGCGTGCCTATTATGCAGTCCGTGATTTA

CTCGTCTATGACAATCCAGAGCATACCGCCTTTAAAATTATCAATCGGTATATCCGTTTT

GTAGATAAAGACGATTCCAAACCTCGTTCTGATTGGAAACTGAATGAAGAATGGGCTTGG

TTTATTGGGAACAATCGTGAACGATTAAAACTAACCACAAAACCAGAGCCTTACTCCTTC

CAAAGGACGCTGAACTGGCTATCTCATCAAGTTGCCCCGACCTTAAAGGTTGCGATTAAA

CTTGATGAAATCAACCAGACGCAGGTTGTAAAAGACATTCTCGACCATGCGAAACTGACA

GACCGACACAAGCAGATTTTGAAGCAACAGTCAGTAAAAGAACAGGACGTGATAACAACA

AAAAAATAACTCAAATACAAATTCATTGAATATAGAGAGGAGAACATTTTTATGAATTTT

GGACAAAACCTTTATAACTGGTTTCTATCAAACGCTCAATCACTGGTGCTTTTAGCAATC

GTTGTGATTGGCTTGTATCTTGGCTTCAAGCGTGAGTTTAGCAAACTGATTGGCTTTTTA

ATTATTGCGATTATTGCGGTTGGCTTAGTCTTCAACGCTGCTGGAGTAAAAGACATTTTA

CTAGAGCTATTCAATCGCATTATTGGTGCTTAAATAAAACCGTTCTTTTGTGGAATATAA

GTGGTTTTCTTATGTTCCGCAAAGGAATGGTACACCAAACGAAGTGCGGTAGGGATTTTT

GAATCTCTACAAAGAAAGGACGTGAATATATGGACGATATGCAAGTCTATATTGCGAATT

TAGGCAAATACAATGAGGGCGAATTGGTCGGTGCGTGGTTTACCTTTCCCATTGACTTTG

AGGAAGTCAAAGAGAAAATCGGCTTGAATGATGAATATGAGGAATACGCCATTCATGACT

ACGAGTTACCCTTTACGGTTGACGAATACACTTCCATTGGCGAACTCAATCGACTATGGG

AAATGGTATCGGAATTACCCGAAGAATTACAATCGGAGCTATCTGCTCTGCTCACTCATT

TTTCAAGCATTGAAGAACTAAGCGAACATCAAGAGGATATTATCATTCATTCCGATTGTG

ATGATATGTATGACGTGGCACGCTACTACATTGAAGAAACGGGTGCTTTAGGCGAAGTAC

CAGCTAGTCTTCAAAACTATATTGATTATCAAGCCTATGGTCGGGATTTAGACCTTTCAG

GAACGTTTATCTCAACCAATCATGGGATTTTTGAAATCGTCTATTAAATCTGTCGGTACA

TTACTACTGGCAGATTTTCTATTTTACGGGGTGGCTCAATCAGCTACCCCTATTTTTTAT

GAAAGGATTGATTACATGAAGAAAATACGAAGCTATACCAGTATCTGGTCTGTGGAAAAG

GTACTGTATTCTATCAATGATTTTAGACTTCCGTTTCCCATAACCTTTACGCAAATGACA

TGGTTTGTCGTGTCACTCTTTGCAGTGATGATACTTGGCAACTTGCCCCCTCTTTCCATG

ATAGAGGGAGCATTTCTCAAATACTTTGGGATTCCTGTGGCTTTCACATGGTTTATGTCT

ACAAAAACTTTTGATGGTAAAAAGCCTTATGGATTTTTGAAGTCTGTCATTGCTTATGCA

CTGCGACCAAAGCTGACCTATGCAGGAAAAAAAGTAACGCTTGGCAGAAACCAGCCACAA

GAAGCCATTACAGCAGTTAGGAGTGAATTTTATGGCATATCCAATTAAATACATTGAAAA

CAATCTCGTCTGGAATAAAGACGGGGAATGTTATGCTTACTATGAGCTTGTTCCTTACAA

TTACTCATTTCTAAGTCCAGAACAGAAAATACAAGTGCATGATTCTTTCAGACAGCTTAT

CGCACAAAATCGTGATGGCAAAATTCATGCTTTACAAATCAGTACAGAATCCAGCATACG

TTCTGCACAAGAGCGTTCCAAAAATGAAGTCACTGGCAAGCTCAAAGCGGTTGCCTATGA

CAAAATCGACCAACAGACAGACGCTTTAATATCCATGATTGGCGAAAATCAAGTGAACTA

CCGTTTCTTTATCGGCTTTAAGTTGCTTCTCAACGATCAGGAGTTTTCTATGAAAAGTCT

TACCGTTGAAGCAAAAAATGCTTTGTCTGATTTTGTCTATGATGTGAACCATAAGCTGAT

GGGCGATTTTGTTAGTATGAGTAATGATGAAATCCTGCGTTTTCAGAAGATGGAAAAGCT

CTTAGAAAATAAAATCTCTCGTCGTTTCAAAATCCGCAGGTTAGATAAGGACGACTTCGG

CTATCTGATTGAACACCTTTACGGACAGACAGGCACTGCCTATGAAGAGTATGAGTACCA

TCTATCAAAGAAAAAGCTGGATAATGAAACGCTGATTAAATACTATGACTTGATTAAGCC

TACTCGCTGTTTGGTGGAAGAAAAACAGCGATATTTGAAAATCCAGCAGGAAGATGAAAC

CGTCTATGTAGCTTACTTTACCATTAACAGCATTGTCGGAGAACTGGACTTCCCGTCCTC

TGAAATCTTCTACTACCAGCAACAGCAATTTACATTCCCGATTGATACGTCAATGAATGT

GGAAATTGTAGCGAATCGTAAAGCCCTATCTACTGTCCGCAATAAAAAGAAAGAACTGAA

AGACTTGGATAACCACGCTTGGCAAAGTGATAATGAAACCAGCTCCAATGTGGCGGAAGC

TCTGGAAAGTGTGAATGAGCTGGAAACCAATTTAGACCAAAGCAAGGAATCTATGTACAA

GCTGTCTTATGTGGTAAGGGTATCAGCAAATGATCTTGACGAACTCAAACGTCGTTGTAA

TGAAGTGAAAGATTTTTATGACGATTTAAGCGTAAAACTGGTACGACCATTTGGGGATAT

GCTCGGCTTACATGAAGAATTTTTACCTGCCAGCAAGCGTTATATGAATGATTATATTCA

ATACGTGACCTCTGATTTCCTCGCTGGTTTAGGTTTTGGTGCTACTCAAATGCTGGGGGA

AAATGAGGGGATTTATGTTGGCTACAGCTTAGATACTGGACGCAATGTCTATCTGAAACC

TGCTCTTGCCAGTCAAGGGGTTAAGGGTTCAGTAACCAATGCGTTAGCGTCGGCTTTTGT

TGGTTCGCTGGGTGGTGGTAAATCCTTTGCGAATAACCTTATCGTCTATTATGCGGTGCT

TTATGGGGCACAAGCAGTGATTGTAGACCCAAAAGCAGAACGTGGCAGATGGAAAGAAAC

CTTGCCAGAGATTTCCCATGAAATCAATATCGTCACTCTGACTTCTGATGAGAAAAACAA

AGGCTTACTTGACCCTTATGTGATTATGAAAAATCCCAAAGATTCTGAATCACTGGCTAT

TGATATTCTGACATTCCTTACGGGGATTTCCTCTCGTGATGGGGAACGCTTCCCAATCCT

TAGAAAAGCCATTCGTGCAGTAACCAATAGTGAAGTACGAGGGTTGATGAAAGTGATTGA

GGAATTACGGGTTGAGAATACGCCACTAAGTACCAGTATAGCCGACCATATCGAAAGTTT

TACAGACTATGACTTTGCACATTTATTATTCAGTAATGGTTATGTGGAGCAGTCTATCAG

CTTAGAAAAACAACTGAACATTATACAGGTTGCGGACTTGGTACTTCCCGACAAGGAAAC

TTCCTTTGAGGAATATACCACTATGGAGCTTTTATCCGTTGCTATGCTGATTGTCATTAG

TACCTTTGCTTTAGACTTTATCCATACAGACCGAAGCATTTTCAAGATTGTAGATTTAGA

CGAAGCATGGAGCTTTTTACAGGTAGCACAAGGAAAAACACTATCTATGAAGCTGGTTCG

GGCTGGTCGTGCTATGAACGCTGGAGTATATTTCGTGACCCAAAATACAGACGACCTCTT

AGATGAAAAACTGAAAAATAACCTCGGCTTAAAATTTGCATTTCGTTCCACTGACCTTAA

CGAGATTAAAAAGACCTTAGCCTTTTTTGGTGTAGACCCAGAGGACGAAAACAATCAGAA

GCGATTGCGTGATTTGGAAAACGGGCAATGCCTTATCAGTGATTTATATGGTCGTGTCGG

TGTGATACAGTTCCACCCTGTATTTGAAGAACTGCTCCATGCCTTTGATACCAGACCACC

TGTGCGAAAAGAGGTGTAAATGTGAAACCATCAATAGTAAACAGAATAAAATCAAACTGG

ACGCTGAAACGTCTAGGTAAAGTGGCAATGACAGTGGCTTTCACACTTGTGATTGCCATT

TTTCTTTTAGCCATGCTGGGAACGGTGGTTCAAGCTGCGGGCTTGGTAGATGATACGGTC

AATGTGGCAAATGAATACAGCCGATACCCACTTGAAAACTATCAACTGGATTTTTATGTG

GATAATAGCTGGGGCTGGCTTCCGTGGAACTGGTCGGACGGGATTGGAAAACAGGTCATG

TATGGACTATATGCCATTACCAATTTTATTTGGACAATCAGTTTGTATGTTTCCAATGCG

ACAGGTTACTTAGTACAGGAAGCCTATTCCTTAGACTTCATTTCCGCTACAGCAGATTCC

ATTGGTAAGAATATGCAGACCTTAGCTGGTGTGAGTGCAAACGGATTTTCAACAGAGGGT

TTCTATGTTGGATTCCTCTTACTCTTGATTTTGGTTCTTGGGGTTTATGTTGCCTATACG

GGACTGATAAAGAGAGAAACCACAAAGGCAATTCATGCCATTATGAATTTTGTGCTGGTG

TTTATCCTATCGGCTTCCTTTATTGCCTACGCTCCCGACTACATTAAAAAAATCAATGAC

TTTTCATCAGACATCAGTAATGCCAGTTTATCACTTGGCACGAAGATTGTCATGCCCCAT

TCCGATAGTCAAGGCAAGGACAGCGTGGACTTAATCAGAGATAGCCTGTTTTCCATACAG

GTTCAGCAACCGTGGCTACTGCTTCAATACAACAGTTCAGACATTGAAAGTATCGGTATT

GACCGTGTGGAAAGCCTGCTCTCCACCAGCCCAGATTCCAACAATGGCGAAGACAGAGAA

AAAATTGTTGCGGAAGAAATTGAAGACAGAAGCAATACCAATCTAACCATTACAAAGACC

ATTAACCGTTTAGGTACAGTCTTCTTCCTATTTGTCTTCAATATTGGGATTTCCATATTT

GTATTCCTATTAACAGGAATCATGATTTTCTCGCAGGTACTTTTTATCATCTATGCTATG

TTTCTGCCTGTGAGCTTTATTTTAAGCATGATTCCATCATTTGATGGTATGTCAAAACGA

GCCATAACAAAGCTCTTTAATACCATTTTGACACGAGCTGGAATCACATTGATTATTACG

ACAGCATTTAGTATTTCAACCATGCTCTATACCTTATCGGCTGGTTATCCGTTCTTTTTG

ATTGCTTTTCTACAGATTGTGACCTTTGCAGGAATCTACTTCAAGCTGGGCGATTTAATG

AGTATGTTTTCTCTACAGAGTAACGATTCTCAAAGTGTGGGAAGTCGTGTGATGAGAAAA

CCTCGTATGCTTATGCACGCTCACATGCACCGTCTACAGCGGAAACTTGGACGTTCCATG

ACTACTCTAGGGGCTGGGTCTGCCATTGTTACAGGTAAAAAAGGACAGTCGGGTTCGGGG

AGTTCTGCAAGGACACAAGCAGATCACTCCCGACCAGACGGAAAGGAAAAATCAACACTT

GGAAAACGTATCGGTCAAACCATCGGTACAGTAGCTGATACCAAAGACAGAATGGTAGAC

ACTGCTAGTGGTTTGAAAGAACAGGTTAAAGATTTGCCGACCAATGCAAGATATGCAGTA

TATCAAGGAAAATCCAAAGTAAAAGAGAATGTCCGTGATTTAACCAGTAGTATTTCTCAA

ACCAAAGCGGACAGAGCCAGTGGACGCAAGGAACAGCAGGAACAAAGGCGAAAAACCATT

GCGAAGCGTCGCTCTGAAATGAAACAGGTCAAACAGAAAAAACAGCCTGCTTCTTCTGTT

CATGAAAGACCGACTACAAGACAAGAACAATATCATGATGAACAGACCTCAAAACAGTCT

AATATTCAGACTTCATATAAGGAATCTCAACAAGCCAAACAAGAGCGTCCAGCAGTTAAG

TCCGATTTTTCAAGTCCAAAAGTGGAACGCCAAGGCAATACCGTTCAAGAAAAAACCGTT

CAAAAGCCAGCAACTTCAACCACTACAGCAGATAGAACTTCACAACGTCCAATCACAAAA

GAACGTCCGTCTACTGTTCAAAGAGTACCACTACAAAATACAAGAAGTAGACCACCAATC

AAAACCGCCACCATTAAGAAAGTCGGTAAGAAACCATGAAGTTGAAAACTTTAGTGATTG

GTGGTTCTGGATTATTCTTGATGGTCTTCTCACTGCTTCTGTTTGTTGCCATTTTATTTT

CAGATGAACAGGACAGCGGAATTTCCAATATTCATTATGGAGGTGTGAATGTTTCCGCAG

AAGTGCTGGCTCATAAGCCTATGGTAGAAAAATATGCCAAAGAATATGGCGTTGAAGAAT

ATGTCAACATACTTCTTGCGATTATACAGGTGGAATCGGGCGGTACTGCGGAAGATGTTA

TGCAGTCCTCGGAATCCCTCGGTCTTCCACCTAATTCATTGAGTACAGAAGAATCCATTA

AGCAAGGTGTGAAGTATTTCAGTGAATTATTAGCCAGTAGCGAAAGGCTCAGTGTAGATT

TAGAATCGGTTATCCAGTCCTACAATTATGGTGGTGGTTTCTTAGGGTATGTGGCTAATC

GTGGAAATAAATATACCTTTGAACTGGCTCAAAGTTTCTCAAAAGAGTATTCAGGTGGCG

AAAAAGTGTCTTACCCCAATCCCATAGCCATACCTATCAATGGGGGCTGGCGATACAACT

ATGGCAATATGTTTTATGTGCAACTGGTAACGCAGTATCTTGTCACAACAGAGTTTGATG

ATGATACGGTACAAGCCATCATGGACGAAGCACTGAAATATGAGGGCTGGCGATACGTTT

ACGGTGGAGCTTCCCCGACTACTTCTTTTGATTGTAGCGGACTGACACAATGGACGTATG

GAAAAGCTGGAATTAACTTACCACGAACCGCACAACAGCAATATGATGTGACCCAGCATA

TCCCACTATCGGAAGCACAAGCTGGCGATTTGGTTTTCTTTCATTCTACCTATAACGCTG

GCTCTTATATTACTCATGTTGGGATATACCTTGGCAATAACCGTATGTTTCATGCAGGCG

ACCCAATCGGTTATGCCGACTTAACAAGCCCCTACTGGCAACAGCATTTAGTGGGAGCAG

GACGAATCAAACAATGAGAAAGGAAGATTTAATGATGAAATTTAGAAAAAATCAGAATAA

AGAAAAACAGATACCAAAGGAAAAGAAACCTCGTGTCTATAAGGTCAATCCTCATAAAAA

GGTTGTGATTGCCTTGTGGGTACTTTTAGGGCTTAGTTTCAGCTTTGCGATATTCAAGCA

CTTTACAGCTATAGATACTCATACTATTCACGAAACAACTATCATAGAAAAGGAATACGT

TGATACTCATCATGTAGAAAATTTTGTAGAGAACTTTGCGAAAGTCTACTATTCATGGGA

GCAATCCGATAAGTCCATTGATAATCGAATGGAAAGTCTAAAAGGCTATCTGACAGATGA

ACTTCAAGCTCTCAATGTTGATACAGTACGCAAAGATATTCCTGTATCGTCTTCTGTAAG

AGGATTTCAGATATGGACGGTAGAGCCAACTGGCGACAATGAGTTTAATGTAACCTACAG

TGTAGACCAGCTCATTACAGAGGGAGAAAATACAAAGACCGTCCACTCTGCTTATATAGT

GAGTGTCTATGTAGATGGTTCTGGAAATATGGTACTGGTTAAGAATCCGACCATTACCAA

CATACCTAAGAAATCAAGTTATAAACCAAAAGCCATTGAAAGTGAGGGGACGGTTGATTC

CATTACAACCAATGAAATCAATGAGTTTTTAACGACGTTCTTCAAGCTCTATCCTACAGC

GACAGCCAGTGAACTTTCCTACTATGTGAATGACGGGATATTAAAACCAATCGGAAAAGA

GTACATCTTTCAAGAACTGGTAAATCCTATTCACAATCGTAAGGATAATCAAGTCACGGT

ATCGCTGACAGTGGAGTATATCGACCAGCAGACCAAAGCAACGCAGGTATCTCAATTTGA

TTTGGTACTTGAAAAGAACGGGAGTAATTGGAAGATTATAGAATAACAAATATTGGTACA

TTATTACAGCTATTTTGTAATCACGTACTCTCTTTGATAAAAAATTGGAGATTCCTTTAC

AAATATGCTCTTATGTGCTATTATTTAAGTATCTATTTAAAAGGAGTTAATAAATATGCG

GCAAGGTATTCTTAAATAAACTGTCAATTTGATAGTGGGAACAAATAATTGGATGTCCTT

TTTTAGGAGGGCTTAGTTTTTTGTACCCAGTTTAAGAATACCTTTATCATGTGATTCTAA

AGTATCCGGAGAATATCTGTATGCTTTGTATGCCTATGGTTATGCATAAAAATCNNNNNN

CACACACTTAATTAATTAAGTGTGTGNNNNNTAAGAGTATTTATCACTGGGATTTTTATG

CCCTTTTGGGTTTTTGAATGGAGGAAAATCACATGAAAATTATTAATATTGGAGTTTTAG

CTCATGTTGATGCAGGAAAAACTACCTTAACAGAAAGCTTATTATATAACAGTGGAGCGA

TTACAGAATTAGGAAGCGTGGACAAAGGTACAACGAGGACGGATAATACGCTTTTAGAAC

GTCAGAGAGGAATTACAATTCAGACAGGAATAACCTCTTTTCAGTGGGAAAATACGAAGG

TGAACATCATAGACACGCCAGGACATATGGATTTCTTAGCAGAAGTATATCGTTCATTAT

CAGTTTTAGATGGGGCAATTCTACTGATTTCTGCAAAAGATGGCGTACAAGCACAAACTC

GTATATTATTTCATGCACTTAGGAAAATGGGGATTCCCACAATCTTTTTTATCAATAAGA

TTGACCAAAATGGAATTGATTTATCAACGGTTTATCAGGATATTAAAGAGAAACTTTCTG

CCGAAATTGTAATCAAACAGAAGGTAGAACTGTATCCTAATATGTGTGTGACGAACTTTA

CCGAATCTGAACAATGGGATACGGTAATAGAGGGAAACGATGACCTTTTAGAGAAATATA

TGTCCGGTAAATCATTAGAAGCATTGGAACTCGAACAAGAGGAAAGCATAAGATTTCAGA

ATTGTTCTCTGTTCCCTCTTTATCATGGAAGTGCAAAAAGTAATATAGGGATTGATAACC

TTATAGAAGTGATTACGAATAAATTTTATTCATCAACACATCGAGGTCAGTCTGAACTTT

GCGGAAAAGTTTTCAAAATTGAGTATTCGGAAAAAAGACAGCGTCTTGCATATATACGTC

TTTATAGTGGCGTACTGCATTTGCGAGATTCGGTTAGAATATCGGAAAAGGAAAAAATAA

AAATTACAGAAATGTATACTTCAATAAATGGTGAATTATGTAAAATCGATAAGGCTTATT

CCGGGGAAATTGTTATTTTGCAGAATGAGTTTTTGAAGTTAAATAGTGTTCTTGGAGATA

CAAAGCTATTGCCACAGAGAGAGAGAATTGAAAATCCCCTCCCTCTGCTGCAAACGACTG

TTGAACCGAGCAAACCTCAACAAAGGGAAATGTTACTTGATGCACTTTTAGAAATCTCCG

ACAGTGACCCGCTTCTGCGATATTATGTGGATTCTGCGACACATGAAATCATACTTTCTT

TCTTAGGGAAAGTACAAATGGAAGTGACTTGTGCTCTGCTGCAAGAAAAGTATCATGTGG

AGATAGAAATAAAAGAGCCTACAGTCATTTATATGGAAAGACCGTTAAAAAAAGCAGAGT

ATACCATTCACATCGAAGTGCCGCCAAATCCTTTCTGGGCTTCCATTGGTTTATCTGTAT

CACCGCTTCCGTTGGGAAGTGGAATGCAGTATGAGAGCTCGGTTTCTCTTGGATACTTAA

ATCAATCATTTCAAAATGCAGTTATGGAAGGGATACGCTATGGTTGCGAACAAGGATTAT

ATGGTTGGAATGTGACGGACTGTAAAATCTGTTTTAAGTATGGCTTATACTATAGCCCTG

TTAGTACCCCAGCAGATTTTCGGATGCTTGCTCCTATTGTATTGGAACAAGTCTTAAAAA

AAGCTGGAACAGAATTGTTAGAGCCATATCTTAGTTTTAAAATTTATGCGCCACAGGAAT

ATCTTTCACGAGCATACAACGATGCTCCTAAATATTGTGCGAACATCGTAGACACTCAAT

TGAAAAATAATGAGGTCATTCTTAGTGGAGAAATCCCTGCTCGGTGTATTCAAGAATATC

GTAGTGATTTAACTTTCTTTACAAATGGACGTAGTGTTTGTTTAACAGAGTTAAAAGGGT

ACCATGTTACTACCGGTGAACCTGTTTGCCAGCCCCGTCGTCCAAATAGTCGGATAGATA

AAGTACGATATATGTTCAATAAAATAACTTAGTGTATTTTATGTTGTTATATAAATATGG

TTTCTTGTTAAATAAGATGAAATATTTTTTAATAAAGATTTGAATTAAAGTGTAAAGGAG

GAGATAGTTATTATAAACTACAAGTGGATATTGTGTGCTGAGAGCTTTCTTCTATACTAA

TAGACGAAAGGGTGTGAAAATGATTTTTAAATGATACTGTGGAACGGAACAGTAGCCCTA

GTATTGACTACTGTCGTTTCTATTCATATTGGCTATTCTAGGACTGAGATGAAAAAATCT

ATAAATGCTCAGAATAAAATTGAACCCGCAAATCTCCCCAAAACAATGGTGAGTCATGTA

CTTGTATTATTCCGAAAAAATACACCTCTGGTGCAGTGAGACAAATTGGTGTATCTTATA

GTGGCTTCGTAGATGAAAGCTATACTCTACTATCACTCTTTGATGATGTAGAACAAATTG

AAAAAGATAATAGACTTCAGACAGCTATTGATGTTGTCAGAGAACAGTTTGGTTTTTTAG

CCATACAAAAAGGAACCGTCCTAACTGAAGGTTCCAGAAATATTGAACGCAGTAAACTTA

TCGGTGGTCATTCCGCGGGTGGATTGGAGGGATTAAAATGAAACAAGAAAAAAATACAGT

ACAATTTTCAGAAATCCGTAGCAAAGGATGTAATGATATTGAAATGCTTGAAAGATTTTT

ACATGGAATCGTTGAAACAGCAACTTCAAAACTTCGTCAGAGAAAACTCAAAACAACTGA

AATATCGATACGACTAGTACATGCTAAATCTGAAAACCGATTACCATTGGAATTTACATT

TAGCATTAAGCCAACAAGCTCATCTGTGATAATCTATACTGAGGTAATCAATCGCTTTAA

AGAATGTTACACAGGTGGGGGAATTCAAGGTTTTACGATTCAATTTGATAAAAATACCCT

TGCCTCTGCATAGAAAGGATTTGATATGATTGACCGTTCATATTTACCATTTCAATCAGC

AAGAGAGTACCAGGATACAAAGATGCAAAAATGGATGGGCTTTTTCCTATCTGAACATGC

ATCAGCACTCTCTGATGATACAAACAAAGTAACGTACATGTCTGACTTATCACTAGAGAA

GAAATTATTACTCCTCAGTCAAGTATACGCCGGGCAGCTACGCACACGCATTCAAGTGAT

TGAAAAAAACAAGCGTGTTTCCTACACTGGAACAATACCAAGTCTGACCAAAGATTTCAT

TTTGATAAAAACTACAACAGGTCACATCAATTTGAAATTAAAAGACATTATTAGTATTGA

ACTTGTCGAGGAGGTGCTCTATGAATCAGCTTGAGTTTCAGCGTAATCACCTACAAATGG

ACTATTATAGCGAGAGCTACCAAGATTTTGAACGTGACTTCTACCGCTACTCTAACATGA

ATATTCCATTGACCTTCCTAACTGATGATATCCTAAAAACAATGGCGACTTCACGTAAGA

ATTACTTTGTCCTCAATAAGGAAAAGTCCAGAGATAACCGCGATCACTTCTTCATATTTG

AAGTAAGTACCGTAGATGAGAATCCGCTAATCTATCATTATACATATAAGAAAACTACAA

TATATTTAGCAGAAAAATAGGAGCAGTTCAATTGACTGTTCCTATTTTTAATATTCATAA

AATCTAAAGTCTTTATACTCTTTAACAATGGAGTCGCCAACCAGAACAGACTATACTGAC

CAGCGACTACCTTAAATTTAATGTTTCAGATTTATTTTCTTATCTCTAATTTCATAAACT

ACATCTGCTACATTTTCGAGTAATCGTTTATCGTGGGTGATAAACACGATAGTTCCGGTG

TACTCCTTCATTAGTATTTCCAAAGCCTCTAAACTTGGTATGTCAAGGAAGTTACTGGGT

TCATCCATTATTAGGATGTTATATCTACCCATGAGCATTTTAGCAAGCAACAATTTTATA

ATTTCTCCACCGCTTAAAACAGATAAACTTTTTCCAATATCGTTCTGTTTGAACCCCATA

GATGCTAGCACTGAACGAATTTCTGATATATTGTAGTCACAATCCTTCTGCATAAACTCC

ATAACATTCTGATTACTGTTGTACTTGTAACCATTCTGTGCAAAGTAACCTATTTTTGCC

TTAGGCGAAATAGAAATTCCTTCTTCATGGTTTAAGATCATTTGGATTAAAGTTGTTTTT

CCGATTCCATTACCACCAGTTAACGCCACTTTTGCTCCTAACGGAATTTGAAAAGATGCA

TTTTCAAACAGAGCCTTATCCCCAAATACTTTATTAATTTCTGCACCGACTATAGGGTAT

GGATTATGGAGCTCCAATGCTTTACTTTGCCTGAAACGAATTCTGCGAATGCCTTCCGGA

GCTTCTACTTTTCCTAAGGCCGCAATCCTGTGCTCTAGGGTTTTAGCAGCATTATACATC

TTTTTTTCCTTACTTCCTATTGATTTTTGATGAGCTAAACGCCCTCCGTCTTCAGTACTT

TTTTTCTTTGAAGAACCTTTTGCCTTCTGTTCTATTTTACGAGCCTGTTTTCGCTTTTCC

TCCGCAGCCCTTTCCAATCGGGCACGTTCCGCAATAAATTGTTCGTATTCTGCAGCTTGG

CTCTTACGTTCTTCCTCTTTCTGACGAAGATAATCAGAATAGTTTCCCCAATACTCAGTG

ATTTTGCCATCTTTCAGTTCCCATATTTTATCTACTATTTCATCAAGAAAATAGCGGTCA

TGGCTAATAACTAACAGTGCACCTGTAAAATATTTTAGCTGTCCTATTAGAAAATCAATT

CCTTCACGGTCTAAATGGCTCGTAGGTTCATCCGCTAAAATACCATGAACCTGTGCCGAT

AAGGCCTGTGCTATTTTAAGCCTTGTTTCTTCACCACCGCTCATAGTCTGTATATTTAAT

TGCTCAACACCTAGCTTGCCTACAAGTGCAAAATCTTTTTCCTCCTGCAGAGTTACTTCG

TCCAACTGGGGAATATAGGCAAGTTCACCCAGACGATTCATTTTACATCCTGGGGGAGTT

AATTCTCCTAAAAGTACCCTGAGTAAAGTGCTTTTTCCAGCACCATTTGCTCCTACTAAA

CCAATACGGTCATAATCATATACTTCTAATTCATTTATATCTAAAACATCGCGTCCTTTG

AATTCCACACGAATGTCTTTTGCTTTTAATATTAATTCCATAACATTTCCTCCTGTCTAT

AATCGCATGCTTTCATTTGCTTGTATGCAGGGAAAACCCTGCGATTTTAGCAGGAAGAGT

TACATGAAAATAAGATACATAAATATTCCTCCAATATTGTTTATTTTAAATCTAATTTTC

TAACCTCAGTTATCATTTGGCAAACTATAGCAATGCCAATAATTAAAATACCTGATAGTA

AAAACCAATGATTTACACCGATTTTATCAGCAAAGAATCCAGAAAGAATTAACCCAATTG

GCATAGCAAGTGACATGATACTTCCGATCAAAGAAAATACACGTCCTAAATATTCAGGCT

TAATTTTCTCCTGAAAAAGAGCTGTTTGCACACCGCTATAAAATGGCACCGAAAGCCCCA

TTATTGCACAGCAAACTACGAATATTACAAATCCATTTGGAGGAAGTATTCCCGAAACGG

CTAAACTGGTCCCCATTATAAAAAATGAACTTGTTATTAGTAATACATGCTTTTCGAAGC

CCCCTAATCTTCCTAATAATAAGCCTCCTGCTAGCATCCCAAATGCAAAGGAAATTTCCG

TAATAGAAATATGCACAGGCGTTCCATTAAAGTGTTCCATGCTTATTAAAGGAAATAGTG

CATTGATTGGCATATAAACAAAAGTATATAGTGTTCCTAAGAGTAATAAGGCAAACAATC

CTTTGTTTTGTCTCAGAACCACAACTCCTTCTTTCATCTCCCTTATGAAATTTGGTTCTA

AACTTTGCACTTGATTACCCAGCTTAGGTATACGTACAATTGCTACCGTAATAGATGCAA

TCACAGCACCCAATACGTCGATGGCAATAATAGCATTTAAATCCCAAACGGAGTATAAGA

GTGCTGCAACTGCCGGACTAACAATATAGCTTATAGACTGCAAAGACTGACTATAGCCTG

CGCATTTCGTTAGCTGTTCTTCTGGTACTAAAAGTGGTGTAACCGCATTGAGTGCTGGGG

TATGAAAAGCTGTTCCAATGCTACGGATAAACAATACTATCATAATCATCCAGACAGGTA

GCTCCATACAGAATGCAACAATAGCAAGCACTGCACCAGCTGCTGCGATAATTAAATCGG

CACCAATCATTATCTTCTTCCTATCATGACGATCCACTAGCACACCAATGGCAGGTCCCA

AAATCGCATAGGGTAAAAAACCTACTAATGAAGCCATAGACAAGACCATCGCAGATCCTG

TTTTTTCTGTAAGGTAAAAAATAATCGCCATTTGCAGGATGGCACTAGTGATTAATGATA

CTGCTTGCCCTGCCCATATTGCATAAAATTTTCGTTTCCAATTGTTGTATTTTTCCATTT

ATATTATCTCCTGCATATTATTTTGCTTGAATTTCTATTTTGAATAGCATTCTAGGCAAT

AAAAAATGCAGGCCAAACCCCACAATGTGGCTTTTGGTCTGCATACATACAATTTGGAAA

CATTCATATTAAAGACATAGTTAAATAAAGGTATAGTTAAATAACCAATATCCTCACCGT

AACTAATGAATGCTCAATATCGTATAAATAAGCACAACAAAAAAGCCTATCATCGGGTAT

AGATTCTGCTTTTTTTATTGCCAGCTTATCTTAAACGCATTGAGGCTGTCATAGTTTCGG

TTCCTCCTACATCTTTGTTTATATCAATTTATAGTATAACACAACAAGATGATATGTTCA

ATATAAAAGTTATGGAATGAGACTCATACTTCCAATTCGATGCCAGATTTAAAGGATATG

ACGAAGTTTTCTTCATAGACTGTAACGCTCTGGATTATCTTCCTTAGTAGCAAGCGATTA

GCTTTCACAAAATCTTCTGTTTGTAGTTTTAAAAATTCATCAGGATTTTCTAACTCAACC

TCAAAATATTTCATTTTACATTCCCTCATTTCATTTATTGATAAATTGAGTTTGCAAAAA

AGAGTGGACAATTTTTGTCTACTCTTAACCTTTAAAATAGTTTTTTTTAATCGATTTGAA

GTTGCCTAAATTATTACTTATTCGGTAAAATGAAGTATTGCTTTCAACAGATTTCCTTCA

ACTACACTTCACTTGATTCAAACAAGGTGGGTACATTTCTATTCCCACAAACTCCTTGTC

AATGGAAACAAACACGTACCCACAGGGTAAATGGAAATAGAAACTGATAATTTCTAGCTA

TCACTTCTACTCATTCCAAAAATTTTCTCACTCTGATACTTACCCACCATAAAGCAAAAA

GCCTTGCAATCAAGGCTTTCATTATCCCTTTCGTTCAAAGGTTTCTAAGCTTTTACGAGC

AGAGCGACACACTCAGCGGTTCGCTATCTCCGTTCTGTCTGCGTGCTAGCACTTGTCAAT

CACGGACAGCTATCGCATGGGCGGAAGTAAATGCTAATCTTCGTCGTTTTACTCCTTGAC

TAGCAAACTTACCGCCTCAACATGTCCTGTATGTGGAAATAAAACACGATTAAAGATAAG

GGAAGATACTGAATTAAAAAAATTCCCCCTCTATTGTCCGAAATGCAGACAAGAAAATTT

AATTGAAATAAAGCAGTTCAAAGTAACTGTGATTACAGAGCCAGACGCAAAGACGCAGAG

CCGATAAAATGAGATTAATACAATCTCATTTTATCGGCTCTTTCCGTTATGTATGGATTC

TTTTAATTAGTCTTCGATGTTTCTTGCTTCGTTGATACCGCTGGCTAAAGATTCCATTAA

GGATAGTTCTTTGTCTGTAAAGCTATCCATGTATTTCTCTATCTGTAATCGTCGGGTGCT

TTTTACCAAGTTATTAGCAGGTAAGAAAAATTCATCAACGGAAACATGAAGTAACGATAC

AAGGTCATAAAGAACTTGTATGCTGGGGTGTTGCCCTTTATTTTCAATATTAGTTAAGTA

CCGTGGGTCAATTTCAATCAATGCTCCCACTTGTTCACGAGTTAAACCTCGTTTCAATCG

AGCTTCTTTAATGGCTAAACCAAAGGCTCTAAAATCATATTTATCTTCTTTTTTACGCAT

AGTAGACCACCTCTATACATTTTATTGTTCCTACTGAATTAAAAACAGGTATAGAAAAAC

GTGTTATATGGTTTATAGGTTTATATTTAATAAAAAGCACTACTAAACGCCAATAAAAAA

AACCGTTATATGGTAGTGCTATTTACGCTGTTAAAATATTGTATATTACTTCCAAATGGC

GGTTTGTTGGAGGTCAACGTCGCCATGAAGTACATCATATACAATAAATTTCCTTACATT

GGGTTCTTGTCAAAAAAAGTCGTCTATCTGCAATAGATAAGTACGTCCACCAATGTGGTT

TTATAAATCATATAGATAGAATAACAGAAGCATGTAAACAGAGAAATAAATCTGTTTATA

TGCTTTTTTGGCTATTCAGAACTTTTTTACAAAGTTTATTTATCAGTAATGCAACAAATC

CCCCTTTCACATTGGGACTAAGAGTGAAAGGAGATAAACGAGCAAGGCTCACTTCCTTTC

CTAGACAGAAAGGGGGTGAGAAACATGAAACCATCTTCTTTTCAGACCACAATAGAAAAT

CAGTTTGACTATATCTGTAAACGTGCTATGGAAGACGAGCGAAAGAATTATATGCTTTAT

CTTTCAAGGATTGCAAAGCGTGAGGTGTCCTTTTCGGATGTTGGCGATTATCTTGTTAGC

CAGTTTGCGACAACAGATAACTATTCAACTGACTTTCAGATTTTTACACTCAATGGGTTA

TCAGTAGGCGTTGAAAATGATTTGTTGAGTGAAGCATTACGTGAGTTGCCAGACAAGAAA

CGTGAAATTCTACTGCTGTTTTACTTTATGGACATGAGCGATTCAGAAATTGCAGACCTG

TTGAAATTGAACCGTTCTACTGTCTATCGGCATAGAACCAGTGGACTAGCCTTAATTAAA

AAGTTTATGGAGGAATTTGAAGAATGAAAACACAATATCCTATGATTCCCTTTCCTCTCA

TTGTAAAGGCAACAGATGGCGATACCGAAGCGATTAACCAGATTCTACATCATTACAGAG

GGTACATAACGAAGCGTTCCCTACGACTTATGAAAGATGAATATGGCAATCAAAGTATGG

TCGTTGATGAAGTCTTACGTGGAAGAATGGAAACCAGACTGATTACAAAGATTTTGTCAT

TTGAAATTAAGTAATATCCTCTCTCCTTTCGTGGAAGCGTGCTAAACCATTCCACGCTTC

CCGAACAGGGAGGTTTGTTATTCCACCAAAGCATATTGAGCTTTCAATGTGTTTTGATAG

GCTAACGAGCCATTGTTCTTTGAAAACTGAATAAAAGTAATCGAATACGTTTCGATAAGA

AAAGAGCCAACGGAACTAACCGCCATGACCTATCTTATAAAGATAGCGAGCGATTCATGT

TAGTGATCCGAGAAGCAATCTTTAGCAGGATTGCCTGCAACGACATTCTTATCGTGATAA

TGATACTCCCATACAGTCAATAGTCCGAGCGTGATAAAACCGTCGCAGGCAATGAGTATG

GCTACATGAGAACCATGCAGGGGTGGAACTCCCGTGAGCTTTGCTAAAGCTGTTCGATTG

CTGGTAAAACAACTTTTATGAAATCCAAATAAGTGATTTGGAAAGGAGGATTTTATGAAG

CAGACTGACATTCCTATTTGGGAACGTTATACCCTAACCATTGAAGAAGCGTCAAAATAT

TTTCGTATTGGCGAAAACAAGCTACGACGCTTGGCAGAGGAAAATAAAAATGCAAATTGG

CTGATTATGAATGGCAATCGTATTCAGATTAAACGAAAACAATTTGAAAAAATTATAGAT

ACATTGGACGCAATCTAGCGTCGCCAAAGGGTCTTGTATATGATAAAATAGTATTAAGTC

GTATCAAGGCTCTTTCCATAAAGGAAAGGAGCAAATGCCATGTCAGAAAAAAGACGTGAC

AATAAAGGTCGAATCTTAAAGACTGGAGAGAGCCAACGAAAAGACGGAAGATACTTATAC

AAATATATAGATTCATTTGGAGAACCGCAATTTGTTTACTCGTGGAAACTTGTGGCTACA

GACCGAGTACCAGCAGGAAAGCGTGATTGTATCTCACTTAGAGAGAAAATCGCAGAGTTA

CAGAAAGACATTCATGATGGTATTGATGTTGTAGGAAAGAAAATGACACTCTGCCAGCTT

TACGCAAAACAGAACGCTCAAAGACCAAAGGTTAGAAAAAACACTGAAACTGGACGCAAA

TATCTTATGGATATTTTGAAGAAAGACAAGTTAGGTGTAAGAAGTATTGACAGTATTAAG

CCATCAGACGCTAAAGAATGGGCTATTAGAATGAGTGAAAATGGTTATGCTTATCAAACC

ATCAATAACTACAAACGTTCTTTAAAGGCTTCATTCTATATTGCTATACAAGATGATTGT

GTTCGGAAGAATCCATTTGACTTTCAACTGAAAGCAGTTCTTGATGATGATACTGTCCCT

AAGACCGTACTAACAGAAGAACAGGAAGAAAAACTGTTAGCCTTTGCAAAAGCTGATAAA

ACCTACAGCAAAAATTATGATGAAATTCTGATACTCTTAAAAACAGGTCTTCGTATTTCA

GAGTTTGGTGGTTTGACACTTCCAGATTTAGATTTTGAGAATCGTCTTGTCAATATAGAC

CATCAGCTATTGAGAGATACTGAAATTGGGTACTACATTGAAACACCAAAGACCAAAAGT

GGCGAACGTCAAGTTCCTATGGTTGAAGAAGCCTATCAAGCATTTAAGCGAGTGTTAGCG

AATCGAAAGAATGATAAGCGTGTTGAGATTGATGGATATAGTGATTTCCTCTTTCTTAAT

AGAAAGAACTATCCAAAAGTGGCAAGTGATTACAACGGCATGATGAAAGGTCTTGTTAAG

AAATACAATAAGTATAACGAGGATAAATTGCCACACATCACTCCACATAGTTTGCGACAT

ACATTCTGTACCAACTATGCAAATGCAGGAATGAATCCAAAGGCATTACAGTACATTATG

GGACATGCTAATATAGCCATGACGCTGAACTATTACGCACATGCAACATTCGATTCTGCA

ATGGCAGAAATGAAACGCTTGAATAAAGAGAAGCAACAGGAGCGTCTTGTTGCTTAGTAG

TACAAATGAATTTACTACTTATTTACCACTTCTGACAGCTAAGACATGAGGAAATATGCA

AAGAAACGTGAAGTATCTTCCTACAGTAAAAATACTCGAAAGCACATAGAATAAGGCTTT

ACGAGCATTTAAGAAAATATAAAAAGATAATTAGAAATTTATACTTTGTTT

>GA41565/Tn2009

AAAATAGCATAAAAATCTAGTTATCCGCATAAAAACTGGACTTATCACACTTTATCAAGG

TCAAAACCACTCAATTTACTACTAATTTACTACTTATGAATGAGCTTTGATACGACGATT

TATCCTTGAAAAGTGAAGATATAAAGATACTTCCAATAAAATTTGAATATTTAATAGGTA

GACACTTCAAAAAATGAGGTGTCTATTTTTTTACCCGATTTTGAAAGGAAGTGAACTTAT

GAAAACAAAAAATCAAGAATCAAAAGGTCGTTCCCCACTCTTTAAGACCATCAAACATTC

ATTCAGCCAATAAAAAGAAAGGATAGGTAAAAATATGGAACTTAAATTTGTGATTCCCAA

CATGGAAAAAACATTCGGCAATTTAGAATTTGCTGGCGAGGATAAAGTCGTTCAGCGAAG

AATCAACGGACGGCTAACTGTCTTATCAAGAAGCTATAATCTCTATTCTGATGTTCAAAG

AGCAGATGATATTGTGGTGGTGCTTCCTGCTGAAGCTGGCGAAAAACATTTCGGCTTTGA

GGAACGTGTGAAGTTAGTCAATCCACGTATTACCGCAGAGGGCTACAAAATCGGCACTCG

TGGTTTTACAAATTACCTTTTACATGCTGACGACATGATAAAAGAATAAAGAAAGAGAGG

AAAAATGATGAGATTAGCAAATGGCATTGTATTAGATAAAGACACGACTTTTGGAGAATT

GAAATTCTCTGCTCTACGTCGTGAAGTGAGAATCCAAAATGAAGACGGGTCGGTTTCAGA

TGAAATCAAGGAACGTACCTATGACTTAAAATCCAAAGGACAAGGACGCATGATTCAAGT

AAGTATTCCTGCCAGCGTGCCTTTGAAAGAGTTTGATTATAACGCACGGGTGGAACTTAT

CAATCCCATTGCGGACACCGTTGCTACTGCCACCTATCAAGGAGCAGATGTTGACTGGTA

TATCAAGGCAGACGATATTGTGCTGACAAAGGATTCTAGTTCATTCAAAGCTCAACCACA

AGCAAAGAAAGAACCGACACAAGACAAATAGTCGCTAGGTAGAAAGGAGACTTTTTCGCA

TGAAACAGCGTGGTAAAAGGATTCGCCCATCTGGTAAAGATTTAGTCTTTCATTTTACGA

TAGCGTCACTCCTGCCTGTTTTCCTGCTGGTTGTCGGACTGTTTCATGTGAAGACAATCC

AGCAGATCAACTGGCAGGATTTTAACCTATCACAAGCAGATAAGATTGACATTCCCTATT

TAATTATCAGTTTCAGTGTCGCAATTCTTATCTGCTTGCTGGTAGCGTTTGTATTCAAAC

GGGTTCGCTATGATACGGTTAAACAACTTTACCACCGTCAAAAACTGGCAAAGATGATAC

TTGAAAACAAGTGGTATGAATCTGAACAGGTCAAAACAGAGGGTTTCTTTAAAGATAGTG

CTGGTCGTACAAAGGAAAAGATAACCTACTTCCCTAAAATGTATTATCGACTTAAAAATG

GCTTGATACAGATACGGGTGGAAATCACGCTGGGAAAATATCAAGACCAACTCTTACACT

TGGAAAAGAAATTAGAGAGTGGCTTGTACTGTGAGCTGACGGATAAAGAGTTAAAGGATT

CCTATGTGGAATATACTTTGCTCTATGACACCATAGCCAGTCGTATTTCTATTGATGAAG

TAGAAGCTAAAGATGGTAAACTTCGCTTAATGAAAAACGTATGGTGGGAATATGATAAGC

TCCCTCATATGTTGATTGCTGGTGGTACAGGTGGCGGTAAAACTTACTTTATACTGACAC

TGATTGAAGCCTTGCTTCATACAGATTCAAAACTGTATATTCTTGACCCGAAAAATGCTG

ACCTTGCGGACTTAGGTTCTGTGATGGCAAATGTCTACTATAGAAAAGAAGACTTGCTTT

CTTGCATTGAAACATTCTATGAAGAAATGATGAAACGTAGTGAGGAAATGAAGCAGATGA

AGAACTATAAGACTGGCAAAAATTATGCTTACTTAGGTCTCCCGGCACACTTCTTAATCT

TTGATGAATACGTCGCTTTCATGGAAATGCTGGGAACAAAAGAAAACACCGCAGTTATGA

ATAAGCTGAAACAGATTGTCATGTTAGGTCGTCAAGCTGGCTTCTTTCTAATACTGGCTT

GTCAACGTCCAGACGCAAAATATTTAGGCGACGGAATCCGTGATCAGTTTAATTTCAGAG

TGGCTTTAGGTCGTATGTCTGAAATGGGCTATGGCATGATGTTTGGCAGTGACGTACAAA

AGGATTTCTTCTTAAAGCGAATCAAAGGTCGTGGCTATGTTGATGTAGGAACAAGTGTCA

TATCAGAGTTTTATACTCCCCTTGTACCAAAAGGATATGATTTCTTGGAGGAAATTAAAA

AGTTATCCAACAGCAGACAGTCCACGCAGGCGACGTGCGAAGCGGAAGTCGCAGGTGTGG

ACTGATCTTGCTGGCTGGTGTGGCAATAGCCACGCCAGCACTTAACCCCCCGTATCTAAC

AGGGGGGTACAAATCGACAGGAAACAGTCAAAAAAACATTAGAAAATCCTTTGGTTACAA

GGGATTTACAAAATTTCAGCGTATGTCAAATGGGCTTTAAAAGTTGACATACGCCTTTTT

GATTGGAGGGATTTTTACTGAATGAACAAACTTGGTTACAGCATTTAAAAGAAAAACGCT

TGGCTTATGGACTATCTCAAAACCGTTTAGCTGTTGCGACTGGTATTACAAGGCAGTATC

TAAGCGATATTGAAACAGGAAAAGTCAAGCCATCAGAGGATTTACAGCAGTCCCTTTGGG

AAGCTCTGGAACGCTTCAATCCCGACGCTCCCCTTGAAATGCTGTTTGATTATGTAAGGA

TTCGCTTTCCGACAACAGACGTACAGCAGGTGGTCGAAAACATCTTACAACTGAAACTGT

CCTATTTTCTTCATGAGGACTATGGTTTCTATTCTTATTCAGAGCATTATGCTTTAGGCG

ACATATTCGTCCTTTGCTCCCATGAACTGGACAAAGGAGTTCTGGTGGAATTGAAAGGTC

GTGGGTGCAGACAATTTGAAAGCTATCTTCTGGCACAACAAAGAAGCTGGTATGAGTTCT

TTATGGACGTTTTGGTGGCTGGCGGTGTGATGAAACGCCTTGACCTTGCCATTAACGATA

AGACAGGGATTTTGAATATCCCTGTACTCACTGAAAAGTGCCAACAGGAAGAATGTATCT

CCGTCTTCCGCAGTTTTAAAAGCTATCGCAGTGGCGAACTGGTACGCAAAGAGGAAAAGG

AATGTATGGGAAACACCCTCTATATCGGTTCATTACAAAGTGAAGTTTATTTCTGTATCT

ATGAAAAGGACTACGAGCAGTACAAGAAAAATGATATTCCCATTGAAGACGCAGAAGTAA

AAAACCGTTTTGAGATTCGATTGAAAAATGAGCGTGCCTATTATGCAGTCCGTGATTTAC

TCGTCTATGACAATCCAGAGCATACCGCCTTTAAAATTATCAATCGGTATATCCGTTTTG

TAGATAAAGACGATTCCAAACCTCGTTCTGATTGGAAACTGAATGAAGAATGGGCTTGGT

TTATTGGGAACAATCGTGAACGATTAAAACTAACCACAAAACCAGAGCCTTACTCCTTCC

AAAGGACGCTGAACTGGCTATCTCATCAAGTTGCCCCGACCTTAAAGGTTGCGATTAAAC

TTGATGAAATCAACCAGACGCAGGTTGTAAAAGACATTCTCGACCATGCGAAACTGACAG

ACCGACACAAGCAGATTTTGAAGCAACAGTCAGTAAAAGAACAGGACGTGATAACAACAA

AAAAATAACTCAAATACAAATTCATTGAATATAGAGAGGAGAACATTTTTATGAATTTTG

GACAAAACCTTTATAACTGGTTTCTATCAAACGCTCAATCACTGGTGCTTTTAGCAATCG

TTGTGATTGGCTTGTATCTTGGCTTCAAGCGTGAGTTTAGCAAACTGATTGGCTTTTTAA

TTATTGCGATTATTGCGGTTGGCTTAGTCTTCAACGCTGCTGGAGTAAAAGACATTTTAC

TAGAGCTATTCAATCGCATTATTGGTGCTTAAATAAAACCGTTCTTTTGTGGAATATAAG

TGGTTTTCTTATGTTCCGCAAAGGAATGGTACACCAAACGAAGTGCGGTAGGGATTTTTG

AATCTCTACAAAGAAAGGACGTGAATATATGGACGATATGCAAGTCTATATTGCGAATTT

AGGCAAATACAATGAGGGCGAATTGGTCGGTGCGTGGTTTACCTTTCCCATTGACTTTGA

GGAAGTCAAAGAGAAAATCGGCTTGAATGATGAATATGAGGAATACGCCATTCATGACTA

CGAGTTACCCTTTACGGTTGACGAATACACTTCCATTGGCGAACTCAATCGACTATGGGA

AATGGTATCGGAATTACCCGAAGAATTACAATCGGAGCTATCTGCTCTGCTCACTCATTT

TTCAAGCATTGAAGAACTAAGCGAACATCAAGAGGATATTATCATTCATTCCGATTGTGA

TGATATGTATGACGTGGCACGCTACTACATTGAAGAAACGGGTGCTTTAGGCGAAGTACC

AGCTAGTCTTCAAAACTATATTGATTATCAAGCCTATGGTCGGGATTTAGACCTTTCAGG

AACGTTTATCTCAACCAATCATGGGATTTTTGAAATCGTCTATTAAATCTGTCGGTACAT

TACTACTGGCAGATTTTCTATTTTACGGGGTGGCTCAATCAGCTACCCCTATTTTTTATG

AAAGGATTGATTACATGAAGAAAATACGAAGCTATACCAGTATCTGGTCTGTGGAAAAGG

TACTGTATTCTATCAATGATTTTAGACTTCCGTTTCCCATAACCTTTACGCAAATGACAT

GGTTTGTCGTGTCACTCTTTGCAGTGATGATACTTGGCAACTTGCCCCCTCTTTCCATGA

TAGAGGGAGCATTTCTCAAATACTTTGGGATTCCTGTGGCTTTCACATGGTTTATGTCTA

CAAAAACTTTTGATGGTAAAAAGCCTTATGGATTTTTGAAGTCTGTCATTGCTTATGCAC

TGCGACCAAAGCTGACCTATGCAGGAAAAAAAGTAACGCTTGGCAGAAACCAGCCACAAG

AAGCCATTACAGCAGTTAGGAGTGAATTTTATGGCATATCCAATTAAATACATTGAAAAC

AATCTCGTCTGGAATAAAGACGGGGAATGTTATGCTTACTATGAGCTTGTTCCTTACAAT

TACTCATTTCTAAGTCCAGAACAGAAAATACAAGTGCATGATTCTTTCAGACAGCTTATC

GCACAAAATCGTGATGGCAAAATTCATGCTTTACAAATCAGTACAGAATCCAGCATACGT

TCTGCACAAGAGCGTTCCAAAAATGAAGTCACTGGCAAGCTCAAAGCGGTTGCCTATGAC

AAAATCGACCAACAGACAGACGCTTTAATATCCATGATTGGCGAAAATCAAGTGAACTAC

CGTTTCTTTATCGGCTTTAAGTTGCTTCTCAACGATCAGGAGTTTTCTATGAAAAGTCTT

ACCGTTGAAGCAAAAAATGCTTTGTCTGATTTTGTCTATGATGTGAACCATAAGCTGATG

GGCGATTTTGTTAGTATGAGTAATGATGAAATCCTGCGTTTTCAGAAGATGGAAAAGCTC

TTAGAAAATAAAATCTCTCGTCGTTTCAAAATCCGCAGGTTAGATAAGGACGACTTCGGC

TATCTGATTGAACACCTTTACGGACAGACAGGCACTGCCTATGAAGAGTATGAGTACCAT

CTATCAAAGAAAAAGCTGGATAATGAAACGCTGATTAAATACTATGACTTGATTAAGCCT

ACTCGCTGTTTGGTGGAAGAAAAACAGCGATATTTGAAAATCCAGCAGGAAGATGAAACC

GTCTATGTAGCTTACTTTACCATTAACAGCATTGTCGGAGAACTGGACTTCCCGTCCTCT

GAAATCTTCTACTACCAGCAACAGCAATTTACATTCCCGATTGATACGTCAATGAATGTG

GAAATTGTAGCGAATCGTAAAGCCCTATCTACTGTCCGCAATAAAAAGAAAGAACTGAAA

GACTTGGATAACCACGCTTGGCAAAGTGATAATGAAACCAGCTCCAATGTGGCGGAAGCT

CTGGAAAGTGTGAATGAGCTGGAAACCAATTTAGACCAAAGCAAGGAATCTATGTACAAG

CTGTCTTATGTGGTAAGGGTATCAGCAAATGATCTTGACGAACTCAAACGTCGTTGTAAT

GAAGTGAAAGATTTTTATGACGATTTAAGCGTAAAACTGGTACGACCATTTGGGGATATG

CTCGGCTTACATGAAGAATTTTTACCTGCCAGCAAGCGTTATATGAATGATTATATTCAA

TACGTGACCTCTGATTTCCTCGCTGGTTTAGGTTTTGGTGCTACTCAAATGCTGGGGGAA

AATGAGGGGATTTATGTTGGCTACAGCTTAGATACTGGACGCAATGTCTATCTGAAACCT

GCTCTTGCCAGTCAAGGGGTTAAGGGTTCAGTAACCAATGCGTTAGCGTCGGCTTTTGTT

GGTTCGCTGGGTGGTGGTAAATCCTTTGCGAATAACCTTATCGTCTATTATGCGGTGCTT

TATGGGGCACAAGCAGTGATTGTAGACCCAAAAGCAGAACGTGGCAGATGGAAAGAAACC

TTGCCAGAAATTTCCCATGAAATCAATATCGTCACTCTGACTTCTGATGAGAAAAACAAA

GGCTTACTTGACCCTTATGTGATTATGAAAAATCCCAAAGATTCTGAATCACTGGCTATT

GATATTCTGACATTCCTTACGGGGATTTCCTCTCGTGATGGGGAACGCTTCCCAATCCTT

AGAAAAGCCATTCGTGCAGTAACCAATAGTGAAGTACGAGGGTTGATGAAAGTGATTGAG

GAATTACGGGTTGAGAATACGCCACTAAGTACCAGTATAGCCGACCATATCGAAAGTTTT

ACAGACTATGACTTTGCACATTTATTATTCAGTAATGGTTATGTGGAGCAGTCTATCAGC

TTAGAAAAACAACTGAACATTATACAGGTTGCGGACTTGGTACTTCCCGACAAGGAAACT

TCCTTTGAGGAATATACCACTATGGAGCTTTTATCCGTTGCTATGCTGATTGTCATTAGT

ACCTTTGCTTTAGACTTTATCCATACAGACCGAAGCATTTTCAAGATTGTAGATTTAGAC

GAAGCATGGAGCTTTTTACAGGTAGCACAAGGAAAAACACTATCTATGAAGCTGGTTCGG

GCTGGTCGTGCTATGAACGCTGGAGTATATTTCGTGACCCAAAATACAGACGACCTCTTA

GATGAAAAACTGAAAAATAACCTCGGCTTAAAATTTGCATTTCGTTCCACTGACCTTAAC

GAGATTAAAAAGACCTTAGCCTTTTTTGGTGTAGACCCAGAGGACGAAAACAATCAGAAG

CGATTGCGTGATTTGGAAAACGGGCAATGCCTTATCAGTGATTTATATGGTCGTGTCGGT

GTGATACAGTTCCACCCTGTATTTGAAGAACTGCTCCATGCCTTTGATACCAGACCACCT

GTGCGAAAAGAGGTGTAAATGTGAAACCATCAATAGTAAACAGAATAAAATCAAACTGGA

CGCTGAAACGTCTAGGTAAAGTGGCAATGACAGTGGCTTTCACACTTGTGATTGCCATTT

TTCTTTTAGCCATGCTGGGAACGGTGGTTCAAGCTGCGGGCTTGGTAGATGATACGGTCA

ATGTGGCAAATGAATACAGCCGATACCCACTTGAAAACTATCAACTGGATTTTTATGTGG

ATAATAGCTGGGGCTGGCTTCCGTGGAACTGGTCGGACGGGATTGGAAAACAGGTCATGT

ATGGACTATATGCCATTACCAATTTTATTTGGACAATCAGTTTGTATGTTTCCAATGCGA

CAGGTTACTTAGTACAGGAAGCCTATTCCTTAGACTTCATTTCCGCTACAGCAGATTCCA

TTGGTAAGAATATGCAGACCTTAGCTGGTGTGAGTGCAAACGGATTTTCAACAGAGGGTT

TCTATGTTGGATTCCTCTTACTCTTGATTTTGGTTCTTGGGGTTTATGTTGCCTATACGG

GACTGATAAAGAGAGAAACCACAAAGGCAATTCATGCCATTATGAATTTTGTGCTGGTGT

TTATCCTATCGGCTTCCTTTATTGCCTACGCTCCCGACTACATTAAAAAAATCAATGACT

TTTCATCAGACATCAGTAATGCCAGTTTATCACTTGGCACGAAGATTGTCATGCCCCATT

CCGATAGTCAAGGCAAGGACAGCGTGGACTTAATCAGAGATAGCCTGTTTTCCATACAGG

TTCAGCAACCGTGGCTACTGCTTCAATACAACAGTTCAGACATTGAAAGTATCGGTATTG

ACCGTGTGGAAAGCCTGCTCTCCACCAGCCCAGATTCCAACAATGGCGAAGACAGAGAAA

AAATTGTTGCGGAAGAAATTGAAGACAGAAGCAATACCAATCTAACCATTACAAAGACCA

TTAACCGTTTAGGTACAGTCTTCTTCCTATTTGTCTTCAATATTGGGATTTCCATATTTG

TATTCCTATTAACAGGAATCATGATTTTCTCGCAGGTACTTTTTATCATCTATGCTATGT

TTCTGCCTGTGAGCTTTATTTTAAGCATGATTCCATCATTTGATGGTATGTCAAAACGAG

CCATAACAAAGCTCTTTAATACCATTTTGACACGAGCTGGAATCACATTGATTATTACGA

CAGCATTTAGTATTTCAACCATGCTCTATACCTTATCGGCTGGTTATCCGTTCTTTTTGA

TTGCTTTTCTACAGATTGTGACCTTTGCAGGAATCTACTTCAAGCTGGGCGATTTAATGA

GTATGTTTTCTCTACAGAGTAACGATTCTCAAAGTGTGGGAAGTCGTGTGATGAGAAAAC

CTCGTATGCTTATGCACGCTCACATGCACCGTCTACAGCGGAAACTTGGACGTTCCATGA

CTACTCTAGGGGCTGGGTCTGCCATTGTTACAGGTAAAAAAGGACAGTCGGGTTCGGGGA

GTTCTGCAAGGACACAAGCAGATCACTCCCGACCAGACGGAAAGGAAAAATCAACACTTG

GAAAACGTATCGGTCAAACCATCGGTACAGTAGCTGATACCAAAGACAGAATGGTAGACA

CTGCTAGTGGTTTGAAAGAACAGGTTAAAGATTTGCCGACCAATGCAAGATATGCAGTAT

ATCAAGGAAAATCCAAAGTAAAAGAGAATGTCCGTGATTTAACCAGTAGTATTTCTCAAA

CCAAAGCGGACAGAGCCAGTGGACGCAAGGAACAGCAGGAACAAAGGCGAAAAACCATTG

CGAAGCGTCGCTCTGAAATGAAACAGGTCAAACAGAAAAAACAGCCTGCTTCTTCTGTTC

ATGAAAGACCGACTACAAGACAAGAACAATATCATGATGAACAGACCTCAAAACAGTCTA

ATATTCAGACTTCATATAAGGAATCTCAACAAGCCAAACAAGAGCGTCCAGCAGTTAAGT

CCGATTTTTCAAGTCCAAAAGTGGAACGCCAAGGCAATACCGTTCAAGAAAAAACCGTTC

AAAAGCCAGCAACTTCAACCACTACAGCAGATAGAACTTCACAACGTCCAATCACAAAAG

AACGTCCGTCTACTGTTCAAAGAGTACCACTACAAAATACAAGAAGTAGACCACCAATCA

AAACCGCCACCATTAAGAAAGTCGGTAAGAAACCATGAAGTTGAAAACTTTAGTGATTGG

TGGTTCTGGATTATTCTTGATGGTCTTCTCACTGCTTCTGTTTGTTGCCATTTTATTTTC

AGATGAACAGGACAGCGGAATTTCCAATATTCATTATGGAGGTGTGAATGTTTCCGCAGA

AGTGCTGGCTCATAAGCCTATGGTAGAAAAATATGCCAAAGAATATGGCGTTGAAGAATA

TGTCAACATACTTCTTGCGATTATACAGGTGGAATCGGGCGGTACTGCGGAAGATGTTAT

GCAGTCCTCGGAATCCCTCGGTCTTCCACCTAATTCATTGAGTACAGAAGAATCCATTAA

GCAAGGTGTGAAGTATTTCAGTGAATTATTAGCCAGTAGCGAAAGGCTCAGTGTAGATTT

AGAATCGGTTATCCAGTCCTACAATTATGGTGGTGGTTTCTTAGGGTATGTGGCTAATCG

TGGAAATAAATATACCTTTGAACTGGCTCAAAGTTTCTCAAAAGAGTATTCAGGTGGCGA

AAAAGTGTCTTACCCCAATCCCATAGCCATACCTATCAATGGGGGCTGGCGATACAACTA

TGGCAATATGTTTTATGTGCAACTGGTAACGCAGTATCTTGTCACAACAGAGTTTGATGA

TGATACGGTACAAGCCATCATGGACGAAGCACTGAAATATGAGGGCTGGCGATACGTTTA

CGGTGGAGCTTCCCCGACTACTTCTTTTGATTGTAGCGGACTGACACAATGGACGTATGG

AAAAGCTGGAATTAACTTACCACGAACCGCACAACAGCAATATGATGTGACCCAGCATAT

CCCACTATCGGAAGCACAAGCTGGCGATTTGGTTTTCTTTCATTCTACCTATAACGCTGG

CTCTTATATTACTCATGTTGGGATATACCTTGGCAATAACCGTATGTTTCATGCAGGCGA

CCCAATCGGTTATGCCGACTTAACAAGCCCCTACTGGCAACAGCATTTAGTGGGAGCAGG

ACGAATCAAACAATGAGAAAGGAAGATTTAATGATGAAATTTAGAAAAAATCAGAATAAA

GAAAAACAGATACCAAAGGAAAAGAAACCTCGTGTCTATAAGGTCAATCCTCATAAAAAG

GTTGTGATTGCCTTGTGGGTACTTTTAGGGCTTAGTTTCAGCTTTGCGATATTCAAGCAC

TTTACAGCTATAGATACTCATACTATTCACGAAACAACTATCATAGAAAAGGAATACGTT

GATACTCATCATGTAGAAAATTTTGTAGAGAACTTTGCGAAAGTCTACTATTCATGGGAG

CAATCCGATAAGTCCATTGATAATCGAATGGAAAGTCTAAAAGGCTATCTGACAGATGAA

CTTCAAGCTCTCAATGTTGATACAGTACGCAAAGATATTCCTGTATCGTCTTCTGTAAGA

GGATTTCAGATATGGACGGTAGAGCCAACTGGCGACAATGAGTTTAATGTAACCTACAGT

GTAGACCAGCTCATTACAGAGGGAGAAAATACAAAGACCGTCCACTCTGCTTATATAGTG

AGTGTCTATGTAGATGGTTCTGGAAATATGGTACTGGTTAAGAATCCGACCATTACCAAC

ATACCTAAGAAATCAAGTTATAAACCAAAAGCCATTGAAAGTGAGGGGACGGTTGATTCC

ATTACAACCAATGAAATCAATGAGTTTTTAACGACGTTCTTCAAGCTCTATCCTACAGCG

ACAGCCAGTGAACTTTCCTACTATGTGAATGACGGGATATTAAAACCAATCGGAAAAGAG

TACATCTTTCAAGAACTGGTAAATCCTATTCACAATCGTAAGGATAATCAAGTCACGGTA

TCGCTGACAGTGGAGTATATCGACCAGCAGACCAAAGCAACGCAGGTATCTCAATTTGAT

TTGGTACTTGAAAAGAACGGGAGTAATTGGAAGATTATAGAATAACAAATATTGGTACAT

TATTACAGCTATTTTGTAATCACGTACTCTCTTTGATAAAAAATTGGAGATTCCTTTACA

AATATGCTCTTATGTGCTATTATTTAAGTATCTATTTAAAAGGAGTTAATAAATATGCGG

CAAGGTATTCTTAAATAAACTGTCAATTTGATAGTGGGAACAAATAATTGGATGTCCTTT

TTTAGGAGGGCTTAGTTTTTTGTACCCAGTTTAAGAATACCTTTATCATGTGATTCTAAA

GTATCCGGAGAATATCTGTATGCTTTGTATGCCTATGGTTATGCATNNNNNCACACACTT

AATTAATTAAGTGTGTGNNNNNTTTTTATGCCCCTTTTTGGGTTTTTGAATGGAGGAAAA

TCACATGAAAATTATTAATATTGGAGTTTTAGCTCATGTTGATGCAGGAAAAACTACCTT

AACAGAAAGCTTATTATATAACAGTGGAGCGATTACAGAATTAGGAAGCGTGGACAAAGG

TACAACGAGGACGGATAATACGCTTTTAGAACGTCAGAGAGGAATTACAATTCAGACAGG

AATAACCTCTTTTCAGTGGGAAAATACGAAGGTGAACATCATAGACACGCCAGGACATAT

GGATTTCTTAGCAGAAGTATATCGTTCATTATCAGTTTTAGATGGGGCAATTCTACTGAT

TTCTGCAAAAGATGGCGTACAAGCACAAACTCGTATATTATTTCATGCACTTAGGAAAAT

GGGGATTCCCACAATCTTTTTTATCAATAAGATTGACCAAAATGGAATTGATTTATCAAC

GGTTTATCAGGATATTAAAGAGAAACTTTCTGCCGAAATTGTAATCAAACAGAAGGTAGA

ACTGTATCCTAATATGTGTGTGACGAACTTTACCGAATCTGAACAATGGGATACGGTAAT

AGAGGGAAACGATGACCTTTTAGAGAAATATATGTCCGGTAAATCATTAGAAGCATTGGA

ACTCGAACAAGAGGAAAGCATAAGATTTCAGAATTGTTCTCTGTTCCCTCTTTATCATGG

AAGTGCAAAAAGTAATATAGGGATTGATAACCTTATAGAAGTGATTACGAATAAATTTTA

TTCATCAACACATCGAGGTCAGTCTGAACTTTGCGGAAAAGTTTTCAAAATTGAGTATTC

GGAAAAAAGACAGCGTCTTGCATATATACGTCTTTATAGTGGCGTACTGCATTTGCGAGA

TTCGGTTAGAATATCGGAAAAGGAAAAATAAAAATTACAGAAATGTATACTTCAATAAAT

GGTGAATTATGTAAAATCGATAAGGCTTATTCCGGGGAAATTGTTATTTTGCAGAATGAG

TTTTTGAAGTTAAATAGTGTTCTTGGAGATACAAAGCTATTGCCACAGAGAGAGAGAATT

GAAAATCCCCTCCCTCTGCTGCAAACGACTGTTGAACCGAGCAAACCTCAACAAAGGGAA

ATGTTACTTGATGCACTTTTAGAAATCTCCGACAGTGACCCGCTTCTGCGATATTATGTG

GATTCTGCGACACATGAAATCATACTTTCTTTCTTAGGGAAAGTACAAATGGAAGTGACT

TGTGCTCTGCTGCAAGAAAAGTATCATGTGGAGATAGAAATAAAAGAGCCTACAGTCATT

TATATGGAAAGACCGTTAAAAAAAGCAGAGTATACCATTCACATCGAAGTGCCGCCAAAT

CCTTTCTGGGCTTCCATTGGTTTATCTGTATCACCGCTTCCGTTGGGAAGTGGAATGCAG

TATGAGAGCTCGGTTTCTCTTGGATACTTAAATCAATCATTTCAAAATGCAGTTATGGAA

GGGATACGCTATGGTTGCGAACAAGGATTATATGGTTGGAATGTGACGGACTGTAAAATC

TGTTTTAAGTATGGCTTATACTATAGCCCTGTTAGTACCCCAGCAGATTTTCGGATGCTT

GCTCCTATTGTATTGGAACAAGTCTTAAAAAAAGCTGGAACAGAATTGTTAGAGCCATAT

CTTAGTTTTAAAATTTATGCGCCACAGGAATATCTTTCACGAGCATACAACGATGCTCCT

AAATATTGTGCGAACATCGTAGACACTCAATTGAAAAATAATGAGGTCATTCTTAGTGGA

GAAATCCCTGCTCGGTGTATTCAAGAATATCGTAGTGATTTAACTTTCTTTACAAATGGA

CGTAGTGTTTGTTTAACAGAGTTAAAAGGGTACCATGTTACTACCGGTGAACCTGTTTGC

CAGCCCCGTCGTCCAAATAGTCGGATAGATAAAGTACGATATATGTTCAATAAAATAACT

TAGTGTATTTTATGTTGTTATATAAATATGGTTTCTTGTTAAATAAGATGAAATATTTTT

TAATAAAGATTTGAATTAAAGTGTAAAGGAGGAGATAGTTATTATAAACTACAAGTGGAT

ATTGTGTGCTGAGAGCTTTCTTCTATACTAATAGACGAAAGGGTGTGAAAATGATTTTTA

AATGATACTGTGGAACGGAACAGTAGCCCTAGTATTGACTACTGTCGTTTCTATTCATAT

TGGCTATTCTAGGACTGAGATGAAAAAATCTATAAATGCTCAGAATAAAATTGAACCCGC

AAATCTCCCCAAAACAATGGTGAGTCATGTACTTGTATTATTCCGAAAAAATACACCTCT

GGTGCAGTGAGACAAATTGGTGTATCTTATAGTGGCTTCGTAGATGAAAGCTATACTCTA

CTATCACTCTTTGATGATGTAGAACAAATTGAAAAAGATAATAGACTTCAGACAGCTATT

GATGTTGTCAGAGAACAGTTTGGTTTTTTAGCCATACAAAAAGGAACCGTCCTAACTGAA

GGTTCCAGAAATATTGAACGCAGTAAACTTATCGGTGGTCATTCCGCGGGTGGATTGGAG

GGATTAAAATGAAACAAGAAAAAAATACAGTACAATTTTCAGAAATCCGTAGCAAAGGAT

GTAATGATATTGAAATGCTTGAAAGATTTTTACATGGAATCGTTGAAACAGCAACTTCAA

AACTTCGTCAGAGAAAACTCAAAACAACTGAAATATCGATACGACTAGTACATGCTAAAT

CTGAAAACCGATTACCATTGGAATTTACATTTAGCATTAAGCCAACAAGCTCATCTGTGA

TAATCTATACTGAGGTAATCAATCGCTTTAAAGAATGTTACACAGGTGGGGGAATTCAAG

GTTTTACGATTCAATTTGATAAAAATACCCTTGCCTCTGCATAGAAAGGATTTGATATGA

TTGACCGTTCATATTTACCATTTCAATCAGCAAGAGAGTACCAGGATACAAAGATGCAAA

AATGGATGGGCTTTTTCCTATCTGAACATGCATCAGCACTCTCTGATGATACAAACAAAG

TAACGTACATGTCTGACTTATCACTAGAGAAGAAATTATTACTCCTCAGTCAAGTATACG

CCGGGCAGCTACGCACACGCATTCAAGTGATTGAAAAAAACAAGCGTGTTTCCTACACTG

GAACAATACCAAGTCTGACCAAAGATTTCATTTTGATAAAAACTACAACAGGTCACATCA

ATTTGAAATTAAAAGACATTATTAGTATTGAACTTGTCGAGGAGGTGCTCTATGAATCAG

CTTGAGTTTCAGCGTAATCACCTACAAATGGACTATTATAGCGAGAGCTACCAAGATTTT

GAACGTGACTTCTACCGCTACTCTAACATGAATATTCCATTGACCTTCCTAACTGATGAT

ATCCTAAAAACAATGGCGACTTCACGTAAGAATTACTTTGTCCTCAATAAGGAAAAGTCC

AGAGATAACCGCGATCACTTCTTCATATTTGAAGTAAGTACCGTAGATGAGAATCCGCTA

ATCTATCATTATACATATAAGAAAACTACAATATATTTAGCAGAAAAATAGGAGCAGTTC

AATTGACTGTTCCTATTTTTAATATTCATAAAATCTAAAGTCTTTATACTCTTTAACAAT

GGAGTCGCCAACCAGAACAGACTATACTGACCAGCGACTACCTTAAATTTAATGTTTCAG

ATTTATTTTCTTATCTCTAATTTCATAAACTACATCTGCTACATTTTCGAGTAATCGTTT

ATCGTGGGTGATAAACACGATAGTTCCGGTGTACTCCTTCATTAGTATTTCCAAAGCCTC

TAAACTTGGTATGTCAAGGAAGTTACTGGGTTCATCCATTATTAGGATGTTATATCTACC

CATGAGCATTTTAGCAAGCAACAATTTTATAATTTCTCCACCGCTTAAAACAGATAAACT

TTTTCCAATATCGTTCTGTTTGAACCCCATAGATGCTAGCACTGAACGAATTTCTGATAT

ATTGTAGTCACAATCCTTCTGCATAAACTCCATAACATTCTGATTACTGTTGTACTTGTA

ACCATTCTGTGCAAAGTAACCTATTTTTGCCTTAGGCGAAATAGAAATTCCTTCTTCATG

GTTTAAGATCATTTGGATTAAAGTTGTTTTTCCGATTCCATTACCACCAGTTAACGCCAC

TTTTGCTCCTAACGGAATTTGAAAAGATGCATTTTCAAACAGAGCCTTATCCCCAAATAC

TTTATTAATTTCTGCACCGACTATAGGGTATGGATTATGGAGCTCCAATGCTTTACTTTG

CCTGAAACGAATTCTGCGAATGCCTTCCGGAGCTTCTACTTTTCCTAAGGCCGCAATCCT

GTGCTCTAGGGTTTTAGCAGCATTATACATCTTTTTTTCCTTACTTCCTATTGATTTTTG

ATGAGCTAAACGCCCTCCGTCTTCAGTACTTTTTTCTTTGAAGAACCTTTTGCCTTCTGT

TCTATTTTACGAGCCTGTTTTCGCTTTTCCTCCGCAGCCCTTTCCAATCGGGCACGTTCC

GCAATAAATTGTTCGTATTCTGCAGCTTGGCTCTTACGTTCTTCCTCTTTCTGACGAAGA

TAATCAGAATAGTTTCCCCAATACTCAGTGATTTTGCCATCTTTCAGTTCCCATATTTTA

TCTACTATTTCATCAAGAAAATAGCGGTCATGGCTAATAACTAACAGTGCACCTGTAAAA

TATTTTAGCTGTCCTATTAGAAAATCAATTCCTTCACGGTCTAAATGGCTCGTAGGTTCA

TCCGCTAAAATACCATGAACCTGTGCCGATAAGGCCTGTGCTATTTTAAGCCTTGTTTCT

TCACCACCGCTCATAGTCTGTATATTTAATTGCTCAACACCTAGCTTGCCTACAAGTGCA

AAATCTTTTTCCTCCTGCAGAGTTACTTCGTCCAACTGGGGAATATAGGCAAGTTCACCC

AGACGATTCATTTTACATCCTGGGGGAGTTAATTCTCCTAAAAGTACCCTGAGTAAAGTG

CTTTTTCCAGCACCATTTGCTCCTACTAAACCAATACGGTCATAATCATATACTTCTAAT

TCATTTATATCTAAAACATCGCGTCCTTTGAATTCCACACGAATGTCTTTTGCTTTTAAT

ATTAATTCCATAACATTTCCTCCTGTCTATAATCGCATGCTTTCATTTGCTTGTATGCAG

GGAAAACCCTGCGATTTTAGCAGGAAGAGTTACATGAAAATAAGATACATAAATATTCCT

CCAATATTGTTTATTTTAAATCTAATTTTCTAACCTCAGTTATCATTTGGCAAACTATAG

CAATGCCAATAATTAAAATACCTGATAGTAAAAACCAATGATTTACACCGATTTTATCAG

CAAAGAATCCAGAAAGAATTAACCCAATTGGCATAGCAAGTGACATGATACTTCCGATCA

AAGAAAATACACGTCCTAAATATTCAGGCTTAATTTTCTCCTGAAAAAGAGCTGTTTGCA

CACCGCTATAAAATGGCACCGAAAGCCCCATTATTGCACAGCAAACTACGAATATTACAA

ATCCATTTGGAGGAAGTATTCCCGAAACGGCTAAACTGGTCCCCATTATAAAAAATGAAC

TTGTTATTAGTAATACATGCTTTTCGAAGCCCCCTAATCTTCCTAATAATAAGCCTCCTG

CTAGCATCCCAAATGCAAAGGAAATTTCCGTAATAGAAATATGCACAGGCGTTCCATTAA

AGTGTTCCATGCTTATTAAAGGAAATAGTGCATTGATTGGCATATAAACAAAAGTATATA

GTGTTCCTAAGAGTAATAAGGCAAACAATCCTTTGTTTTGTCTCAGAACCACAACTCCTT

CTTTCATCTCCCTTATGAAATTTGGTTCTAAACTTTGCACTTGATTACCCAGCTTAGGTA

TACGTACAATTGCTACCGTAATAGATGCAATCACAGCACCCAATACGTCGATGGCAATAA

TAGCATTTAAATCCCAAACGGAGTATAAGAGTGCTGCAACTGCCGGACTAACAATATAGC

TTATAGACTGCAAAGACTGACTATAGCCTGCGCATTTCGTTAGCTGTTCTTCTGGTACTA

AAAGTGGTGTAACCGCATTGAGTGCTGGGGTATGAAAAGCTGTTCCAATGCTACGGATAA

ACAATACTATCATAATCATCCAGACAGGTAGCTCCATACAGAATGCAACAATAGCAAGCA

CTGCACCAGCTGCTGCGATAATTAAATCGGCACCAATCATTATCTTCTTCCTATCATGAC

GATCCACTAGCACACCAATGGCAGGTCCCAAAATCGCATAGGGTAAAAAACCTACTAATG

AAGCCATAGACAAGACCATCGCAGATCCTGTTTTTTCTGTAAGGTAAAAAATAATCGCCA

TTTGCAGGATGGCACTAGTGATTAATGATACTGCTTGCCCTGCCCATATTGCATAAAATT

TTCGTTTCCAATTGTTGTATTTTTCCATTTATATTATCTCCTGCATATTATTTTGCTTGA

ATTTCTATTTTGAATAGCATTCTAGGCAATAAAAAATGCAGGCCAAACCCCACAATGTGG

CTTTTGGTCTGCATACATACAATTTGGAAACATTCATATTAAAGACATAGTTAAATAAAG

GTATAGTTAAATAACCAATATCCTCACCGTAACTAATGAATGCTCAATATCGTATAAATA

AGCACAACAAAAAAGCCTATCATCGGGTATAGATTCTGCTTTTTTTATTGCCAGCTTATC

TTAAACGCATTGAGGCTGTCATAGTTTCGGTTCCTCCTACATCTTTGTTTATATCAATTT

ATAGTATAACACAACAAGATGATATGTTCAATATAAAAGTTATGGAATGAGACTCATACT

TCCAATTCGATGCCAGATTTAAAGGATATGACGAAGTTTTCTTCATAGACTGTAACGCTC

TGGATTATCTTCCTTAGTAGCAAGCGATTAGCTTTCACAAAATCTTCTGTTTGTAGTTTT

AAAAATTCATCAGGATTTTCTAACTCAACCTCAAAATATTTCATTTTACATTCCCTCATT

TCATTTATTGATAAATTGAGTTTGCAAAAAAGAGTGGACAATTTTTGTCTACTCTTAACC

TTTAAAATAGTTTTTTTTAATCGATTTGAAGTTGCCTAAATTATTACTTATTCGGTAAAA

TGAAGTATTGCTTTCAACAGATTTCCTTCAACTACACTTCACTTGATTCAAACAAGGTGG

GTACATTTCTATTCCCACAAACTCCTTGTCAATGGAAACAAACACGTACCCACAGGGTAA

ATGGAAATAGAAACTGATAATTTCTAGCTATCACTTCTACTCATTCCAAAAATTTTCTCA

CTCTGATACTTACCCACCATAAAGCAAAAAGCCTTGCAATCAAGGCTTTCATTATCCCTT

TCGTTCAAAGGTTTCTAAGCTTTTACGAGCAGAGCGACACACTCAGCGGTTCGCTATCTC

CGTTCTGTCTGCGTGCTAGCACTTGTCAATCACGGACAGCTATCGCATGGGCGGAAGTAA

ATGCTAATCTTCGTCGTTTTACTCCTTGACTAGCAAACTTACCGCCTCAACATGTCCTGT

ATGTGGAAATAAAACACGATTAAAGATAAGGGAAGATACTGAATTAAAAAAATTCCCCCT

CTATTGTCCGAAATGCAGACAAGAAAATTTAATTGAAATAAAGCAGTTCAAAGTAACTGT

GATTACAGAGCCAGACGCAAAGACGCAGAGCCGATAAAATGAGATTAATACAATCTCATT

TTATCGGCTCTTTCCGTTATGTATGGATTCTTTTAATTAGTCTTCGATGTTTCTTGCTTC

GTTGATACCGCTGGCTAAAGATTCCATTAAGGATAGTTCTTTGTCTGTAAAGCTATCCAT

GTATTTCTCTATCTGTAATCGTCGGGTGCTTTTTACCAAGTTATTAGCAGGTAAGAAAAA

TTCATCAACGGAAACATGAAGTAACGATACAAGGTCATAAAGAACTTGTATGCTGGGGTG

TTGCCCTTTATTTTCAATATTAGTTAAGTACCGTGGGTCAATTTCAATCAATGCTCCCAC

TTGTTCACGAGTTAAACCTCGTTTCAATCGAGCTTCTTTAATGGCTAAACCAAAGGCTCT

AAAATCATATTTATCTTCTTTTTTACGCATAGTAGACCACCTCTATACATTTTATTGTTC

CTACTGAATTAAAAACAGGTATAGAAAAACGTGTTATATGGTTTATAGGTTTATATTTAA

TAAAAAGCACTACTAAACGCCAATAAAAAAACCGTTATATGGTAGTGCTATTTACGCTGT

TAAAATATTGTATATTACTTCCAAATGGCGGTTTGTTGGAGGTCAACGTCGCCATGAAGT

ACATCATATACAATAAATTTCCTTACATTGGGTTCTTGTCAAAAAAAGTCGTCTATCTGC

AATAGATAAGTACGTCCACCAATGTGGTTTTATAAATCATATAGATAGAATAACAGAAGC

ATGTAAACAGAGAAATAAATCTGTTTATATGCTTTTTTGGCTATTCAGAACTTTTTTACA

AAGTTTATTTATCAGTAATGCAACAAATCCCCCTTTCACATTGGGACTAAGAGTGAAAGG

AGATAAACGAGCAAGGCTCACTTCCTTTCCTAGACAGAAAGGGGGTGAGAAACATGAAAC

CATCTTCTTTTCAGACCACAATAGAAAATCAGTTTGACTATATCTGTAAACGTGCTATGG

AAGACGAGCGAAAGAATTATATGCTTTATCTTTCAAGGATTGCAAAGCGTGAGGTGTCCT

TTTCGGATGTTGGCGATTATCTTGTTAGCCAGTTTGCGACAACAGATAACTATTCAACTG

ACTTTCAGATTTTTACACTCAATGGGTTATCAGTAGGCGTTGAAAATGATTTGTTGAGTG

AAGCATTACGTGAGTTGCCAGACAAGAAACGTGAAATTCTACTGCTGTTTTACTTTATGG

ACATGAGCGATTCAGAAATTGCAGACCTGTTGAAATTGAACCGTTCTACTGTCTATCGGC

ATAGAACCAGTGGACTAGCCTTAATTAAAAAGTTTATGGAGGAATTTGAAGAATGAAAAC

ACAATATCCTATGATTCCCTTTCCTCTCATTGTAAAGGCAACAGATGGCGATACCGAAGC

GATTAACCAGATTCTACATCATTACAGAGGGTACATAACGAAGCGTTCCCTACGACTTAT

GAAAGATGAATATGGCAATCAAAGTATGGTCGTTGATGAAGTCTTACGTGGAAGAATGGA

AACCAGACTGATTACAAAGATTTTGTCATTTGAAATTAAGTAATATCCTCTCTCCTTTCG

TGGAAGCGTGCTAAACCATTCCACGCTTCCCGAACAGGGAGGTTTGTTATTCCACCAAAG

CATATTGAGCTTTCAATGTGTTTTGATAGGCTAACGAGCCATTGTTCTTTGAAAACTGAA

TAAAAGTAATCGAATACGTTTCGATAAGAAAAGAGCCAACGGAACTAACCGCCATGACCT

ATCTTATAAAGATAGCGAGCGATTCATGTTAGTGATCCGAGAAGCAATCTTTAGCAGGAT

TGCCTGCAACGACATTCTTATCGTGATAATGATACTCCCATACAGTCAATAGTCCGAGCG

TGATAAAACCGTCGCAGGCAATGAGTATGGCTACATGAGAACCATGCAGGGGTGGAACTC

CCGTGAGCTTTGCTAAAGCTGTTCGATTGCTGGTAAAACAACTTTTATGAAATCCAAATA

AGTGATTTGGAAAGGAGGATTTTATGAAGCAGACTGACATTCCTATTTGGGAACGTTATA

CCCTAACCATTGAAGAAGCGTCAAAATATTTTCGTATTGGCGAAAACAAGCTACGACGCT

TGGCAGAGGAAAATAAAAATGCAAATTGGCTGATTATGAATGGCAATCGTATTCAGATTA

AACGAAAACAATTTGAAAAAATTATAGATACATTGGACGCAATCTAGCGTCGCCAAAGGG

TCTTGTATATGATAAAATAGTATTAAGTCGTATCAAGGCTCTTTCCATAAAGGAAAGGAG

CAAATGCCATGTCAGAAAAAAGACGTGACAATAAAGGTCGAATCTTAAAGACTGGAGAGA

GCCAACGAAAAGACGGAAGATACTTATACAAATATATAGATTCATTTGGAGAACCGCAAT

TTGTTTACTCGTGGAAACTTGTGGCTACAGACCGAGTACCAGCAGGAAAGCGTGATTGTA

TCTCACTTAGAGAGAAAATCGCAGAGTTACAGAAAGACATTCATGATGGTATTGATGTTG

TAGGAAAGAAAATGACACTCTGCCAGCTTTACGCAAAACAGAACGCTCAAAGACCAAAGG

TTAGAAAAAACACTGAAACTGGACGCAAATATCTTATGGATATTTTGAAGAAAGACAAGT

TAGGTGTAAGAAGTATTGACAGTATTAAGCCATCAGACGCTAAAGAATGGGCTATTAGAA

TGAGTGAAAATGGTTATGCTTATCAAACCATCAATAACTACAAACGTTCTTTAAAGGCTT

CATTCTATATTGCTATACAAGATGATTGTGTTCGGAAGAATCCATTTGACTTTCAACTGA

AAGCAGTTCTTGATGATGATACTGTCCCTAAGACCGTACTAACAGAAGAACAGGAAGAAA

AACTGTTAGCCTTTGCAAAAGCTGATAAAACCTACAGCAAAAATTATGATGAAATTCTGA

TACTCTTAAAAACAGGTCTTCGTATTTCAGAGTTTGGTGGTTTGACACTTCCAGATTTAG

ATTTTGAGAATCGTCTTGTCAATATAGACCATCAGCTATTGAGAGATACTGAAATTGGGT

ACTACATTGAAACACCAAAGACCAAAAGTGGCGAACGTCAAGTTCCTATGGTTGAAGAAG

CCTATCAAGCATTTAAGCGAGTGTTAGCGAATCGAAAGAATGATAAGCGTGTTGAGATTG

ATGGATATAGTGATTTCCTCTTTCTTAATAGAAAGAACTATCCAAAAGTGGCAAGTGATT

ACAACGGCATGATGAAAGGTCTTGTTAAGAAATACAATAAGTATAACGAGGATAAATTGC

CACACATCACTCCACATAGTTTGCGACATACATTCTGTACCAACTATGCAAATGCAGGAA

TGAATCCAAAGGCATTACAGTACATTATGGGACATGCTAATATAGCCATGACGCTGAACT

ATTACGCACATGCAACATTCGATTCTGCAATGGCAGAAATGAAACGCTTGAATAAAGAGA

AGCAACAGGAGCGTCTTGTTGCTTAGTAGTACAAATGAATTTACTACTTATTTACCACTT

CTGACAGCTAAGACATGAGGAAATATGCAAAGAAACGTGAAGTATCTTCCTACAGTAAAA

ATACTCGAAAGCACATAGAATAAGGCTTTACGAGCATTTAAGAAAATATAAAAAGATAAT

TAGAAATTTATACTTTGTTT

>NP170/Tn2009

AAAATAGCATAAAAATCTAGTTATCCGCATAAAAACTGGACTTATCACACTTTATCAAGG

TCAAAACCACTCAATTTACTACTAATTTACTACTTATGAATGAGCTTTGATACGACGATT

TATCCTTGAAAAGTGAAGATATAAAGATACTTCCAATAAAATTTGAATATTTAATAGGTA

GACACTTCAAAAAATGAGGTGTCTATTTTTTTACCCGATTTTGAAAGGAAGTGAACTTAT

GAAAACAAAAAATCAAGAATCAAAAGGTCGTTCCCCACTCTTTAAGACCATCAAACATTC

ATTCAGCCAATAAAAAAGAAAGGATAGGTAAAAATATGGAACTTAAATTTGTGATTCCCA

ACATGGAAAAAACATTCGGCAATTTAGAATTTGCTGGCGAGGATAAAGTCGTTCAGCGAA

GAATCAACGGACGGCTAACTGTCTTATCAAGAAGCTATAATCTCTATTCTGATGTTCAAA

GAGCAGATGATATTGTGGTGGTGCTTCCTGCTGAAGCTGGCGAAAAACATTTCGGCTTTG

AGGAACGTGTGAAGTTAGTCAATCCACGTATTACCGCAGAGGGCTACAAAATCGGCACTC

GTGGTTTTACAAATTACCTTTTACATGCTGACGACATGATAAAAGAATAAAGAAAGAGAG

GAAAAATGATGAGATTAGCAAATGGCATTGTATTAGATAAAGACACGACTTTTGGAGAAT

TGAAATTCTCTGCTCTACGTCGTGAAGTGAGAATCCAAAATGAAGACGGGTCGGTTTCAG

ATGAAATCAAGGAACGTACCTATGACTTAAAATCCAAAGGACAAGGACGCATGATTCAAG

TAAGTATTCCTGCCAGCGTGCCTTTGAAAGAGTTTGATTATAACGCACGGGTGGAACTTA

TCAATCCCATTGCGGACACCGTTGCTACTGCCACCTATCAAGGAGCAGATGTTGACTGGT

ATATCAAGGCAGACGATATTGTGCTGACAAAGGATTCTAGTTCATTCAAAGCTCAACCAC

AAGCAAAGAAAGAACCGACACAAGACAAATAGTCGCTAGGTAGAAAGGAGACTTTTTCGC

ATGAAACAGCGTGGTAAAAGGATTCGCCCATCTGGTAAAGATTTAGTCTTTCATTTTACG

ATAGCGTCACTCCTGCCTGTTTTCCTGCTGGTTGTCGGACTGTTTCATGTGAAGACAATC

CAGCAGATCAACTGGCAGGATTTTAACCTATCACAAGCAGATAAGATTGACATTCCCTAT

TTAATTATCAGTTTCAGTGTCGCAATTCTTATCTGCTTGCTGGTAGCGTTTGTATTCAAA

CGGGTTCGCTATGATACGGTTAAACAACTTTACCACCGTCAAAAACTGGCAAAGATGATA

CTTGAAAACAAGTGGTATGAATCTGAACAGGTCAAAACAGAGGGTTTCTTTAAAGATAGT

GCTGGTCGTACAAAGGAAAAGATAACCTACTTCCCTAAAATGTATTATCGACTTAAAAAT

GGCTTGATACAGATACGGGTGGAAATCACGCTGGGAAAATATCAAGACCAACTCTTACAC

TTGGAAAAGAAATTAGAGAGTGGCTTGTACTGTGAGCTGACGGATAAAGAGTTAAAGGAT

TCCTATGTGGAATATACTTTGCTCTATGACACCATAGCCAGTCGTATTTCTATTGATGAA

GTAGAAGCTAAAGATGGTAAACTTCGCTTAATGAAAAACGTATGGTGGGAATATGATAAG

CTCCCTCATATGTTGATTGCTGGTGGTACAGGTGGCGGTAAAACTTACTTTATACTGACA

CTGATTGAAGCCTTGCTTCATACAGATTCAAAACTGTATATTCTTGACCCGAAAAATGCT

GACCTTGCGGACTTAGGTTCTGTGATGGCAAATGTCTACTATAGAAAAGAAGACTTGCTT

TCTTGCATTGAAACATTCTATGAAGAAATGATGAAACGTAGTGAGGAAATGAAGCAGATG

AAGAACTATAAGACTGGCAAAAATTATGCTTACTTAGGTCTCCCGGCACACTTCTTAATC

TTTGATGAATACGTCGCTTTCATGGAAATGCTGGGAACAAAAGAAAACACCGCAGTTATG

AATAAGCTGAAACAGATTGTCATGTTAGGTCGTCAAGCTGGCTTCTTTCTAATACTGGCT

TGTCAACGTCCAGACGCAAAATATTTAGGCGACGGAATCCGTGATCAGTTTAATTTCAGA

GTGGCTTTAGGTCGTATGTCTGAAATGGGCTATGGCATGATGTTTGGCAGTGACGTACAA

AAGGATTTCTTCTTAAAGCGAATCAAAGGTCGTGGCTATGTTGATGTAGGAACAAGTGTC

ATATCAGAGTTTTATACTCCCCTTGTACCAAAAGGATATGATTTCTTGGAGGAAATTAAA

AAGTTATCCAACAGCAGACAGTCCACGCAGGCGACGTGCGAAGCGGAAGTCGCAGGTGTG

GACTGATCTTGCTGGCTGGTGTGGCAATAGCCACGCCAGCACTTAACCCCCCGTATCTAA

CAGGGGGGTACAAATCGACAGGAAACAGTCAAAAAAACATTAGAAAATCCTTTGGTTACA

AGGGATTTACAAAATTTCAGCGTATGTCAAATGGGCTTTAAAAGTTGACATACGCCTTTT

TGATTGGAGGGATTTTTACTGAATGAACAAACTTGGTTACAGCATTTAAAAGAAAAACGC

TTGGCTTATGGACTATCTCAAAACCGTTTAGCTGTTGCGACTGGTATTACAAGGCAGTAT

CTAAGCGATATTGAAACAGGAAAAGTCAAGCCATCAGAGGATTTACAGCAGTCCCTTTGG

GAAGCTCTGGAACGCTTCAATCCCGACGCTCCCCTTGAAATGCTGTTTGATTATGTAAGG

ATTCGCTTTCCGACAACAGACGTACAGCAGGTGGTCGAAAACATCTTACAACTGAAACTG

TCCTATTTTCTTCATGAGGACTATGGTTTCTATTCTTATTCAGAGCATTATGCTTTAGGC

GACATATTCGTCCTTTGCTCCCATGAACTGGACAAAGGAGTTCTGGTGGAATTGAAAGGT

CGTGGGTGCAGACAATTTGAAAGCTATCTTCTGGCACAACAAAGAAGCTGGTATGAGTTC

TTTATGGACGTTTTGGTGGCTGGCGGTGTGATGAAACGCCTTGACCTTGCCATTAACGAT

AAGACAGGGATTTTGAATATCCCTGTACTCACTGAAAAGTGCCAACAGGAAGAATGTATC

TCCGTCTTCCGCAGTTTTAAAAGCTATCGCAGTGGCGAACTGGTACGCAAAGAGGAAAAG

GAATGTATGGGAAACACCCTCTATATCGGTTCATTACAAAGTGAAGTTTATTTCTGTATC

TATGAAAAGGACTACGAGCAGTACAAGAAAAATGATATTCCCATTGAAGACGCAGAAGTA

AAAAACCGTTTTGAGATTCGATTGAAAAATGAGCGTGCCTATTATGCAGTCCGTGATTTA

CTCGTCTATGACAATCCAGAGCATACCGCCTTTAAAATTATCAATCGGTATATCCGTTTT

GTAGATAAAGACGATTCCAAACCTCGTTCTGATTGGAAACTGAATGAAGAATGGGCTTGG

TTTATTGGGAACAATCGTGAACGATTAAAACTAACCACAAAACCAGAGCCTTACTCCTTC

CAAAGGACGCTGAACTGGCTATCTCATCAAGTTGCCCCGACCTTAAAGGTTGCGATTAAA

CTTGATGAAATCAACCAGACGCAGGTTGTAAAAGACATTCTCGACCATGCGAAACTGACA

GACCGACACAAGCAGATTTTGAAGCAACAGTCAGTAAAAGAACAGGACGTGATAACAACA

AAAAAATAACTCAAATACAAATTCATTGAATATAGAGAGGAGAACATTTTTATGAATTTT

GGACAAAACCTTTATAACTGGTTTCTATCAAACGCTCAATCACTGGTGCTTTTAGCAATC

GTTGTGATTGGCTTGTATCTTGGCTTCAAGCGTGAGTTTAGCAAACTGATTGGCTTTTTA

ATTATTGCGATTATTGCGGTTGGCTTAGTCTTCAACGCTGCTGGAGTAAAAGACATTTTA

CTAGAGCTATTCAATCGCATTATTGGTGCTTAAATAAAACCGTTCTTTTGTGGAATATAA

GTGGTTTTCTTATGTTCCGCAAAGGAATGGTACACCAAACGAAGTGCGGTAGGGATTTTT

GAATCTCTACAAAGAAAGGACGTGAATATATGGACGATATGCAAGTCTATATTGCGAATT

TAGGCAAATACAATGAGGGCGAATTGGTCGGTGCGTGGTTTACCTTTCCCATTGACTTTG

AGGAAGTCAAAGAGAAAATCGGCTTGAATGATGAATATGAGGAATACGCCATTCATGACT

ACGAGTTACCCTTTACGGTTGACGAATACACTTCCATTGGCGAACTCAATCGACTATGGG

AAATGGTATCGGAATTACCCGAAGAATTACAATCGGAGCTATCTGCTCTGCTCACTCATT

TTTCAAGCATTGAAGAACTAAGCGAACATCAAGAGGATATTATCATTCATTCCGATTGTG

ATGATATGTATGACGTGGCACGCTACTACATTGAAGAAACGGGTGCTTTAGGCGAAGTAC

CAGCTAGTCTTCAAAACTATATTGATTATCAAGCCTATGGTCGGGATTTAGACCTTTCAG

GAACGTTTATCTCAACCAATCATGGGATTTTTGAAATCGTCTATTAAATCTGTCGGTACA

TTACTACTGGCAGATTTTCTATTTTACGGGGTGGCTCAATCAGCTACCCCTATTTTTTAT

GAAAGGATTGATTACATGAAGAAAATACGAAGCTATACCAGTATCTGGTCTGTGGAAAAG

GTACTGTATTCTATCAATGATTTTAGACTTCCGTTTCCCATAACCTTTACGCAAATGACA

TGGTTTGTCGTGTCACTCTTTGCAGTGATGATACTTGGCAACTTGCCCCCTCTTTCCATG

ATAGAGGGAGCATTTCTCAAATACTTTGGGATTCCTGTGGCTTTCACATGGTTTATGTCT

ACAAAAACTTTTGATGGTAAAAAGCCTTATGGATTTTTGAAGTCTGTCATTGCTTATGCA

CTGCGACCAAAGCTGACCTATGCAGGAAAAAAAGTAACGCTTGGCAGAAACCAGCCACAA

GAAGCCATTACAGCAGTTAGGAGTGAATTTTATGGCATATCCAATTAAATACATTGAAAA

CAATCTCGTCTGGAATAAAGACGGGGAATGTTATGCTTACTATGAGCTTGTTCCTTACAA

TTACTCATTTCTAAGTCCAGAACAGAAAATACAAGTGCATGATTCTTTCAGACAGCTTAT

CGCACAAAATCGTGATGGCAAAATTCATGCTTTACAAATCAGTACAGAATCCAGCATACG

TTCTGCACAAGAGCGTTCCAAAAATGAAGTCACTGGCAAGCTCAAAGCGGTTGCCTATGA

CAAAATCGACCAACAGACAGACGCTTTAATATCCATGATTGGCGAAAATCAAGTGAACTA

CCGTTTCTTTATCGGCTTTAAGTTGCTTCTCAACGATCAGGAGTTTTCTATGAAAAGTCT

TACCGTTGAAGCAAAAAATGCTTTGTCTGATTTTGTCTATGATGTGAACCATAAGCTGAT

GGGCGATTTTGTTAGTATGAGTAATGATGAAATCCTGCGTTTTCAGAAGATGGAAAAGCT

CTTAGAAAATAAAATCTCTCGTCGTTTCAAAATCCGCAGGTTAGATAAGGACGACTTCGG

CTATCTGATTGAACACCTTTACGGACAGACAGGCACTGCCTATGAAGAGTATGAGTACCA

TCTATCAAAGAAAAAGCTGGATAATGAAACGCTGATTAAATACTATGACTTGATTAAGCC

TACTCGCTGTTTGGTGGAAGAAAAACAGCGATATTTGAAAATCCAGCAGGAAGATGAAAC

CGTCTATGTAGCTTACTTTACCATTAACAGCATTGTCGGAGAACTGGACTTCCCGTCCTC

TGAAATCTTCTACTACCAGCAACAGCAATTTACATTCCCGATTGATACGTCAATGAATGT

GGAAATTGTAGCGAATCGTAAAGCCCTATCTACTGTCCGCAATAAAAAGAAAGAACTGAA

AGACTTGGATAACCACGCTTGGCAAAGTGATAATGAAACCAGCTCCAATGTGGCGGAAGC

TCTGGAAAGTGTGAATGAGCTGGAAACCAATTTAGACCAAAGCAAGGAATCTATGTACAA

GCTGTCTTATGTGGTAAGGGTATCAGCAAATGATCTTGACGAACTCAAACGTCGTTGTAA

TGAAGTGAAAGATTTTTATGACGATTTAAGCGTAAAACTGGTACGACCATTTGGGGATAT

GCTCGGCTTACATGAAGAATTTTTACCTGCCAGCAAGCGTTATATGAATGATTATATTCA

ATACGTGACCTCTGATTTCCTCGCTGGTTTAGGTTTTGGTGCTACTCAAATGCTGGGGGA

AAATGAGGGGATTTATGTTGGCTACAGCTTAGATACTGGACGCAATGTCTATCTGAAACC

TGCTCTTGCCAGTCAAGGGGTTAAGGGTTCAGTAACCAATGCGTTAGCGTCGGCTTTTGT

TGGTTCGCTGGGTGGTGGTAAATCCTTTGCGAATAACCTTATCGTCTATTATGCGGTGCT

TTATGGGGCACAAGCAGTGATTGTAGACCCAAAAGCAGAACGTGGCAGATGGAAAGAAAC

CTTGCCAGAGATTTCCCATGAAATCAATATCGTCACTCTGACTTCTGATGAGAAAAACAA

AGGCTTACTTGACCCTTATGTGATTATGAAAAATCCCAAAGATTCTGAATCACTGGCTAT

TGATATTCTGACATTCCTTACGGGGATTTCCTCTCGTGATGGGGAACGCTTCCCAATCCT

TAGAAAAGCCATTCGTGCAGTAACCAATAGTGAAGTACGAGGGTTGATGAAAGTGATTGA

GGAATTACGGGTTGAGAATACGCCACTAAGTACCAGTATAGCCGACCATATCGAAAGTTT

TACAGACTATGACTTTGCACATTTATTATTCAGTAATGGTTATGTGGAGCAGTCTATCAG

CTTAGAAAAACAACTGAACATTATACAGGTTGCGGACTTGGTACTTCCCGACAAGGAAAC

TTCCTTTGAGGAATATACCACTATGGAGCTTTTATCCGTTGCTATGCTGATTGTCATTAG

TACCTTTGCTTTAGACTTTATCCATACAGACCGAAGCATTTTCAAGATTGTAGATTTAGA

CGAAGCATGGAGCTTTTTACAGGTAGCACAAGGAAAAACACTATCTATGAAGCTGGTTCG

GGCTGGTCGTGCTATGAACGCTGGAGTATATTTCGTGACCCAAAATACAGACGACCTCTT

AGATGAAAAACTGAAAAATAACCTCGGCTTAAAATTTGCATTTCGTTCCACTGACCTTAA

CGAGATTAAAAAGACCTTAGCCTTTTTTGGTGTAGACCCAGAGGACGAAAACAATCAGAA

GCGATTGCGTGATTTGGAAAACGGGCAATGCCTTATCAGTGATTTATATGGTCGTGTCGG

TGTGATACAGTTCCACCCTGTATTTGAAGAACTGCTCCATGCCTTTGATACCAGACCACC

TGTGCGAAAAGAGGTGTAAATGTGAAACCATCAATAGTAAACAGAATAAAATCAAACTGG

ACGCTGAAACGTCTAGGTAAAGTGGCAATGACAGTGGCTTTCACACTTGTGATTGCCATT

TTTCTTTTAGCCATGCTGGGAACGGTGGTTCAAGCTGCGGGCTTGGTAGATGATACGGTC

AATGTGGCAAATGAATACAGCCGATACCCACTTGAAAACTATCAACTGGATTTTTATGTG

GATAATAGCTGGGGCTGGCTTCCGTGGAACTGGTCGGACGGGATTGGAAAACAGGTCATG

TATGGACTATATGCCATTACCAATTTTATTTGGACAATCAGTTTGTATGTTTCCAATGCG

ACAGGTTACTTAGTACAGGAAGCCTATTCCTTAGACTTCATTTCCGCTACAGCAGATTCC

ATTGGTAAGAATATGCAGACCTTAGCTGGTGTGAGTGCAAACGGATTTTCAACAGAGGGT

TTCTATGTTGGATTCCTCTTACTCTTGATTTTGGTTCTTGGGGTTTATGTTGCCTATACG

GGACTGATAAAGAGAGAAACCACAAAGGCAATTCATGCCATTATGAATTTTGTGCTGGTG

TTTATCCTATCGGCTTCCTTTATTGCCTACGCTCCCGACTACATTAAAAAAATCAATGAC

TTTTCATCAGACATCAGTAATGCCAGTTTATCACTTGGCACGAAGATTGTCATGCCCCAT

TCCGATAGTCAAGGCAAGGACAGCGTGGACTTAATCAGAGATAGCCTGTTTTCCATACAG

GTTCAGCAACCGTGGCTACTGCTTCAATACAACAGTTCAGACATTGAAAGTATCGGTATT

GACCGTGTGGAAAGCCTGCTCTCCACCAGCCCAGATTCCAACAATGGCGAAGACAGAGAA

AAAATTGTTGCGGAAGAAATTGAAGACAGAAGCAATACCAATCTAACCATTACAAAGACC

ATTAACCGTTTAGGTACAGTCTTCTTCCTATTTGTCTTCAATATTGGGATTTCCATATTT

GTATTCCTATTAACAGGAATCATGATTTTCTCGCAGGTACTTTTTATCATCTATGCTATG

TTTCTGCCTGTGAGCTTTATTTTAAGCATGATTCCATCATTTGATGGTATGTCAAAACGA

GCCATAACAAAGCTCTTTAATACCATTTTGACACGAGCTGGAATCACATTGATTATTACG

ACAGCATTTAGTATTTCAACCATGCTCTATACCTTATCGGCTGGTTATCCGTTCTTTTTG

ATTGCTTTTCTACAGATTGTGACCTTTGCAGGAATCTACTTCAAGCTGGGCGATTTAATG

AGTATGTTTTCTCTACAGAGTAACGATTCTCAAAGTGTGGGAAGTCGTGTGATGAGAAAA

CCTCGTATGCTTATGCACGCTCACATGCACCGTCTACAGCGGAAACTTGGACGTTCCATG

ACTACTCTAGGGGCTGGGTCTGCCATTGTTACAGGTAAAAAAGGACAGTCGGGTTCGGGG

AGTTCTGCAAGGACACAAGCAGATCACTCCCGACCAGACGGAAAGGAAAAATCAACACTT

GGAAAACGTATCGGTCAAACCATCGGTACAGTAGCTGATACCAAAGACAGAATGGTAGAC

ACTGCTAGTGGTTTGAAAGAACAGGTTAAAGATTTGCCGACCAATGCAAGATATGCAGTA

TATCAAGGAAAATCCAAAGTAAAAGAGAATGTCCGTGATTTAACCAGTAGTATTTCTCAA

ACCAAAGCGGACAGAGCCAGTGGACGCAAGGAACAGCAGGAACAAAGGCGAAAAACCATT

GCGAAGCGTCGCTCTGAAATGAAACAGGTCAAACAGAAAAAACAGCCTGCTTCTTCTGTT

CATGAAAGACCGACTACAAGACAAGAACAATATCATGATGAACAGACCTCAAAACAGTCT

AATATTCAGACTTCATATAAGGAATCTCAACAAGCCAAACAAGAGCGTCCAGCAGTTAAG

TCCGATTTTTCAAGTCCAAAAGTGGAACGCCAAGGCAATACCGTTCAAGAAAAAACCGTT

CAAAAGCCAGCAACTTCAACCACTACAGCAGATAGAACTTCACAACGTCCAATCACAAAA

GAACGTCCGTCTACTGTTCAAAGAGTACCACTACAAAATACAAGAAGTAGACCACCAATC

AAAACCGCCACCATTAAGAAAGTCGGTAAGAAACCATGAAGTTGAAAACTTTAGTGATTG

GTGGTTCTGGATTATTCTTGATGGTCTTCTCACTGCTTCTGTTTGTTGCCATTTTATTTT

CAGATGAACAGGACAGCGGAATTTCCAATATTCATTATGGAGGTGTGAATGTTTCCGCAG

AAGTGCTGGCTCATAAGCCTATGGTAGAAAAATATGCCAAAGAATATGGCGTTGAAGAAT

ATGTCAACATACTTCTTGCGATTATACAGGTGGAATCGGGCGGTACTGCGGAAGATGTTA

TGCAGTCCTCGGAATCCCTCGGTCTTCCACCTAATTCATTGAGTACAGAAGAATCCATTA

AGCAAGGTGTGAAGTATTTCAGTGAATTATTAGCCAGTAGCGAAAGGCTCAGTGTAGATT

TAGAATCGGTTATCCAGTCCTACAATTATGGTGGTGGTTTCTTAGGGTATGTGGCTAATC

GTGGAAATAAATATACCTTTGAACTGGCTCAAAGTTTCTCAAAAGAGTATTCAGGTGGCG

AAAAAGTGTCTTACCCCAATCCCATAGCCATACCTATCAATGGGGGCTGGCGATACAACT

ATGGCAATATGTTTTATGTGCAACTGGTAACGCAGTATCTTGTCACAACAGAGTTTGATG

ATGATACGGTACAAGCCATCATGGACGAAGCACTGAAATATGAGGGCTGGCGATACGTTT

ACGGTGGAGCTTCCCCGACTACTTCTTTTGATTGTAGCGGACTGACACAATGGACGTATG

GAAAAGCTGGAATTAACTTACCACGAACCGCACAACAGCAATATGATGTGACCCAGCATA

TCCCACTATCGGAAGCACAAGCTGGCGATTTGGTTTTCTTTCATTCTACCTATAACGCTG

GCTCTTATATTACTCATGTTGGGATATACCTTGGCAATAACCGTATGTTTCATGCAGGCG

ACCCAATCGGTTATGCCGACTTAACAAGCCCCTACTGGCAACAGCATTTAGTGGGAGCAG

GACGAATCAAACAATGAGAAAGGAAGATTTAATGATGAAATTTAGAAAAAATCAGAATAA

AGAAAAACAGATACCAAAGGAAAAGAAACCTCGTGTCTATAAGGTCAATCCTCATAAAAA

GGTTGTGATTGCCTTGTGGGTACTTTTAGGGCTTAGTTTCAGCTTTGCGATATTCAAGCA

CTTTACAGCTATAGATACTCATACTATTCACGAAACAACTATCATAGAAAAGGAATACGT

TGATACTCATCATGTAGAAAATTTTGTAGAGAACTTTGCGAAAGTCTACTATTCATGGGA

GCAATCCGATAAGTCCATTGATAATCGAATGGAAAGTCTAAAAGGCTATCTGACAGATGA

ACTTCAAGCTCTCAATGTTGATACAGTACGCAAAGATATTCCTGTATCGTCTTCTGTAAG

AGGATTTCAGATATGGACGGTAGAGCCAACTGGCGACAATGAGTTTAATGTAACCTACAG

TGTAGACCAGCTCATTACAGAGGGAGAAAATACAAAGACCGTCCACTCTGCTTATATAGT

GAGTGTCTATGTAGATGGTTCTGGAAATATGGTACTGGTTAAGAATCCGACCATTACCAA

CATACCTAAGAAATCAAGTTATAAACCAAAAGCCATTGAAAGTGAGGGGACGGTTGATTC

CATTACAACCAATGAAATCAATGAGTTTTTAACGACGTTCTTCAAGCTCTATCCTACAGC

GACAGCCAGTGAACTTTCCTACTATGTGAATGACGGGATATTAAAACCAATCGGAAAAGA

GTACATCTTTCAAGAACTGGTAAATCCTATTCACAATCGTAAGGATAATCAAGTCACGGT

ATCGCTGACAGTGGAGTATATCGACCAGCAGACCAAAGCAACGCAGGTATCTCAATTTGA

TTTGGTACTTGAAAAGAACGGGAGTAATTGGAAGATTATAGAATAACAAATATTGGTACA

TTATTACAGCTATTTTGTAATCACGTACTCTCTTTGATAAAAAATTGGAGATTCCTTTAC

AAATATGCTCTTATGTGCTATTATTTAAGTATCTATTTAAAAGGAGTTAATAAATATGCG

GCAAGGTATTCTTAAATAAACTGTCAATTTGATAGTGGGAACAAATAATTGGATGTCCTT

TTTTAGGAGGGCTTAGTTTTTTGTACCCAGTTTAAGAATACCTTTATCATGTGATTCTAA

AGTATCCGGAGAATATCTGTATGCTTTGTATGCCTATGGTTATGCATAAAAATCCCAGTG

ATAAGAGTATTTNNNNNCACACACTTAATTAATTAAGTGTGTGNNNNNTTTTTATGCCCT

TTTGGGTTTTTGAATGGAGGAAAATCACATGAAAATTATTAATATTGGAGTTTTAGCTCA

TGTTGATGCAGGAAAAACTACCTTAACAGAAAGCTTATTATATAACAGTGGAGCGATTAC

AGAATTAGGAAGCGTGGACAAAGGTACAACGAGGACGGATAATACGCTTTTAGAACGTCA

GAGAGGAATTACAATTCAGACAGGAATAACCTCTTTTCAGTGGGAAAATACGAAGGTGAA

CATCATAGACACGCCAGGACATATGGATTTCTTAGCAGAAGTATATCGTTCATTATCAGT

TTTAGATGGGGCAATTCTACTGATTTCTGCAAAAGATGGCGTACAAGCACAAACTCGTAT

ATTATTTCATGCACTTAGGAAAATGGGGATTCCCACAATCTTTTTTATCAATAAGATTGA

CCAAAATGGAATTGATTTATCAACGGTTTATCAGGATATTAAAGAGAAACTTTCTGCCGA

AATTGTAATCAAACAGAAGGTAGAACTGTATCCTAATATGTGTGTGACGAACTTTACCGA

ATCTGAACAATGGGATACGGTAATAGAGGGAAACGATGACCTTTTAGAGAAATATATGTC

CGGTAAATCATTAGAAGCATTGGAACTCGAACAAGAGGAAAGCATAAGATTTCAGAATTG

TTCTCTGTTCCCTCTTTATCATGGAAGTGCAAAAAGTAATATAGGGATTGATAACCTTAT

AGAAGTGATTACGAATAAATTTTATTCATCAACACATCGAGGTCAGTCTGAACTTTGCGG

AAAAGTTTTCAAAATTGAGTATTCGGAAAAAAGACAGCGTCTTGCATATATACGTCTTTA

TAGTGGCGTACTGCATTTGCGAGATTCGGTTAGAATATCGGAAAAGGAAAAATAAAAATT

ACAGAAATGTATACTTCAATAAATGGTGAATTATGTAAAATCGATAAGGCTTATTCCGGG

GAAATTGTTATTTTGCAGAATGAGTTTTTGAAGTTAAATAGTGTTCTTGGAGATACAAAG

CTATTGCCACAGAGAGAGAGAATTGAAAATCCCCTCCCTCTGCTGCAAACGACTGTTGAA

CCGAGCAAACCTCAACAAAGGGAAATGTTACTTGATGCACTTTTAGAAATCTCCGACAGT

GACCCGCTTCTGCGATATTATGTGGATTCTGCGACACATGAAATCATACTTTCTTTCTTA

GGGAAAGTACAAATGGAAGTGACTTGTGCTCTGCTGCAAGAAAAGTATCATGTGGAGATA

GAAATAAAAGAGCCTACAGTCATTTATATGGAAAGACCGTTAAAAAAAGCAGAGTATACC

ATTCACATCGAAGTGCCGCCAAATCCTTTCTGGGCTTCCATTGGTTTATCTGTATCACCG

CTTCCGTTGGGAAGTGGAATGCAGTATGAGAGCTCGGTTTCTCTTGGATACTTAAATCAA

TCATTTCAAAATGCAGTTATGGAAGGGATACGCTATGGTTGCGAACAAGGATTATATGGT

TGGAATGTGACGGACTGTAAAATCTGTTTTAAGTATGGCTTATACTATAGCCCTGTTAGT

ACCCCAGCAGATTTTCGGATGCTTGCTCCTATTGTATTGGAACAAGTCTTAAAAAAAGCT

GGAACAGAATTGTTAGAGCCATATCTTAGTTTTAAAATTTATGCGCCACAGGAATATCTT

TCACGAGCATACAACGATGCTCCTAAATATTGTGCGAACATCGTAGACACTCAATTGAAA

AATAATGAGGTCATTCTTAGTGGAGAAATCCCTGCTCGGTGTATTCAAGAATATCGTAGT

GATTTAACTTTCTTTACAAATGGACGTAGTGTTTGTTTAACAGAGTTAAAAGGGTACCAT

GTTACTACCGGTGAACCTGTTTGCCAGCCCCGTCGTCCAAATAGTCGGATAGATAAAGTA

CGATATATGTTCAATAAAATAACTTAGTGTATTTTATGTTGTTATATAAATATGGTTTCT

TGTTAAATAAGATGAAATATTTTTTAATAAAGATTTGAATTAAAGTGTAAAGGAGGAGAT

AGTTATTATAAACTACAAGTGGATATTGTGTGCTGAGAGCTTTCTTCTATACTAATAGAC

GAAAGGGTGTGAAAATGATTTTTAAATGATACTGTGGAACGGAACAGTAGCCCTAGTATT

GACTACTGTCGTTTCTATTCATATTGGCTATTCTAGGACTGAGATGAAAAAATCTATAAA

TGCTCAGAATAAAATTGAACCCGCAAATCTCCCCAAAACAATGGTGAGTCATGTACTTGT

ATTATTCCGAAAAAATACACCTCTGGTGCAGTGAGACAAATTGGTGTATCTTATAGTGGC

TTCGTAGATGAAAGCTATACTCTACTATCACTCTTTGATGATGTAGAACAAATTGAAAAA

GATAATAGACTTCAGACAGCTATTGATGTTGTCAGAGAACAGTTTGGTTTTTTAGCCATA

CAAAAAGGAACCGTCCTAACTGAAGGTTCCAGAAATATTGAACGCAGTAAACTTATCGGT

GGTCATTCCGCGGGTGGATTGGAGGGATTAAAATGAAACAAGAAAAAAATACAGTACAAT

TTTCAGAAATCCGTAGCAAAGGATGTAATGATATTGAAATGCTTGAAAGATTTTTACATG

GAATCGTTGAAACAGCAACTTCAAAACTTCGTCAGAGAAAACTCAAAACAACTGAAATAT

CGATACGACTAGTACATGCTAAATCTGAAAACCGATTACCATTGGAATTTACATTTAGCA

TTAAGCCAACAAGCTCATCTGTGATAATCTATACTGAGGTAATCAATCGCTTTAAAGAAT

GTTACACAGGTGGGGGAATTCAAGGTTTTACGATTCAATTTGATAAAAATACCCTTGCCT

CTGCATAGAAAGGATTTGATATGATTGACCGTTCATATTTACCATTTCAATCAGCAAGAG

AGTACCAGGATACAAAGATGCAAAAATGGATGGGCTTTTTCCTATCTGAACATGCATCAG

CACTCTCTGATGATACAAACAAAGTAACGTACATGTCTGACTTATCACTAGAGAAGAAAT

TATTACTCCTCAGTCAAGTATACGCCGGGCAGCTACGCACACGCATTCAAGTGATTGAAA

AAAACAAGCGTGTTTCCTACACTGGAACAATACCAAGTCTGACCAAAGATTTCATTTTGA

TAAAAACTACAACAGGTCACATCAATTTGAAATTAAAAGACATTATTAGTATTGAACTTG

TCGAGGAGGTGCTCTATGAATCAGCTTGAGTTTCAGCGTAATCACCTACAAATGGACTAT

TATAGCGAGAGCTACCAAGATTTTGAACGTGACTTCTACCGCTACTCTAACATGAATATT

CCATTGACCTTCCTAACTGATGATATCCTAAAAACAATGGCGACTTCACGTAAGAATTAC

TTTGTCCTCAATAAGGAAAAGTCCAGAGATAACCGCGATCACTTCTTCATATTTGAAGTA

AGTACCGTAGATGAGAATCCGCTAATCTATCATTATACATATAAGAAAACTACAATATAT

TTAGCAGAAAAATAGGAGCAGTTCAATTGACTGTTCCTATTTTTAATATTCATAAAATCT

AAAGTCTTTATACTCTTTAACAATGGAGTCGCCAACCAGAACAGACTATACTGACCAGCG

ACTACCTTAAATTTAATGTTTCAGATTTATTTTCTTATCTCTAATTTCATAAACTACATC

TGCTACATTTTCGAGTAATCGTTTATCGTGGGTGATAAACACGATAGTTCCGGTGTACTC

CTTCATTAGTATTTCCAAAGCCTCTAAACTTGGTATGTCAAGGAAGTTACTGGGTTCATC

CATTATTAGGATGTTATATCTACCCATGAGCATTTTAGCAAGCAACAATTTTATAATTTC

TCCACCGCTTAAAACAGATAAACTTTTTCCAATATCGTTCTGTTTGAACCCCATAGATGC

TAGCACTGAACGAATTTCTGATATATTGTAGTCACAATCCTTCTGCATAAACTCCATAAC

ATTCTGATTACTGTTGTACTTGTAACCATTCTGTGCAAAGTAACCTATTTTTGCCTTAGG

CGAAATAGAAATTCCTTCTTCATGGTTTAAGATCATTTGGATTAAAGTTGTTTTTCCGAT

TCCATTACCACCAGTTAACGCCACTTTTGCTCCTAACGGAATTTGAAAAGATGCATTTTC

AAACAGAGCCTTATCCCCAAATACTTTATTAATTTCTGCACCGACTATAGGGTATGGATT

ATGGAGCTCCAATGCTTTACTTTGCCTGAAACGAATTCTGCGAATGCCTTCCGGAGCTTC

TACTTTTCCTAAGGCCGCAATCCTGTGCTCTAGGGTTTTAGCAGCATTATACATCTTTTT

TTCCTTACTTCCTATTGATTTTTGATGAGCTAAACGCCCTCCGTCTTCAGTACTTTTTTT

CTTTGAAGAACCTTTTGCCTTCTGTTCTATTTTACGAGCCTGTTTTCGCTTTTCCTCCGC

AGCCCTTTCCAATCGGGCACGTTCCGCAATAAATTGTTCGTATTCTGCAGCTTGGCTCTT

ACGTTCTTCCTCTTTCTGACGAAGATAATCAGAATAGTTTCCCCAATACTCAGTGATTTT

GCCATCTTTCAGTTCCCATATTTTATCTACTATTTCATCAAGAAAATAGCGGTCATGGCT

AATAACTAACAGTGCACCTGTAAAATATTTTAGCTGTCCTATTAGAAAATCAATTCCTTC

ACGGTCTAAATGGCTCGTAGGTTCATCCGCTAAAATACCATGAACCTGTGCCGATAAGGC

CTGTGCTATTTTAAGCCTTGTTTCTTCACCACCGCTCATAGTCTGTATATTTAATTGCTC

AACACCTAGCTTGCCTACAAGTGCAAAATCTTTTTCCTCCTGCAGAGTTACTTCGTCCAA

CTGGGGAATATAGGCAAGTTCACCCAGACGATTCATTTTACATCCTGGGGGAGTTAATTC

TCCTAAAAGTACCCTGAGTAAAGTGCTTTTTCCAGCACCATTTGCTCCTACTAAACCAAT

ACGGTCATAATCATATACTTCTAATTCATTTATATCTAAAACATCGCGTCCTTTGAATTC

CACACGAATGTCTTTTGCTTTTAATATTAATTCCATAACATTTCCTCCTGTCTATAATCG

CATGCTTTCATTTGCTTGTATGCAGGGAAAACCCTGCGATTTTAGCAGGAAGAGTTACAT

GAAAATAAGATACATAAATATTCCTCCAATATTGTTTATTTTAAATCTAATTTTCTAACC

TCAGTTATCATTTGGCAAACTATAGCAATGCCAATAATTAAAATACCTGATAGTAAAAAC

CAATGATTTACACCGATTTTATCAGCAAAGAATCCAGAAAGAATTAACCCAATTGGCATA

GCAAGTGACATGATACTTCCGATCAAAGAAAATACACGTCCTAAATATTCAGGCTTAATT

TTCTCCTGAAAAAGAGCTGTTTGCACACCGCTATAAAATGGCACCGAAAGCCCCATTATT

GCACAGCAAACTACGAATATTACAAATCCATTTGGAGGAAGTATTCCCGAAACGGCTAAA

CTGGTCCCCATTATAAAAAATGAACTTGTTATTAGTAATACATGCTTTTCGAAGCCCCCT

AATCTTCCTAATAATAAGCCTCCTGCTAGCATCCCAAATGCAAAGGAAATTTCCGTAATA

GAAATATGCACAGGCGTTCCATTAAAGTGTTCCATGCTTATTAAAGGAAATAGTGCATTG

ATTGGCATATAAACAAAAGTATATAGTGTTCCTAAGAGTAATAAGGCAAACAATCCTTTG

TTTTGTCTCAGAACCACAACTCCTTCTTTCATCTCCCTTATGAAATTTGGTTCTAAACTT

TGCACTTGATTACCCAGCTTAGGTATACGTACAATTGCTACCGTAATAGATGCAATCACA

GCACCCAATACGTCGATGGCAATAATAGCATTTAAATCCCAAACGGAGTATAAGAGTGCT

GCAACTGCCGGACTAACAATATAGCTTATAGACTGCAAAGACTGACTATAGCCTGCGCAT

TTCGTTAGCTGTTCTTCTGGTACTAAAAGTGGTGTAACCGCATTGAGTGCTGGGGTATGA

AAAGCTGTTCCAATGCTACGGATAAACAATACTATCATAATCATCCAGACAGGTAGCTCC

ATACAGAATGCAACAATAGCAAGCACTGCACCAGCTGCTGCGATAATTAAATCGGCACCA

ATCATTATCTTCTTCCTATCATGACGATCCACTAGCACACCAATGGCAGGTCCCAAAATC

GCATAGGGTAAAAAACCTACTAATGAAGCCATAGACAAGACCATCGCAGATCCTGTTTTT

TCTGTAAGGTAAAAAATAATCGCCATTTGCAGGATGGCACTAGTGATTAATGATACTGCT

TGCCCTGCCCATATTGCATAAAATTTTCGTTTCCAATTGTTGTATTTTTCCATTTATATT

ATCTCCTGCATATTATTTTGCTTGAATTTCTATTTTGAATAGCATTCTAGGCAATAAAAA

ATGCAGGCCAAACCCCACAATGTGGCTTTTGGTCTGCATACATACAATTTGGAAACATTC

ATATTAAAGACATAGTTAAATAAAGGTATAGTTAAATAACCAATATCCTCACCGTAACTA

ATGAATGCTCAATATCGTATAAATAAGCACAACAAAAAAGCCTATCATCGGGTATAGATT

CTGCTTTTTTTATTGCCAGCTTATCTTAAACGCATTGAGGCTGTCATAGTTTCGGTTCCT

CCTACATCTTTGTTTATATCAATTTATAGTATAACACAACAAGATGATATGTTCAATATA

AAAGTTATGGAATGAGACTCATACTTCCAATTCGATGCCAGATTTAAAGGATATGACGAA

GTTTTCTTCATAGACTGTAACGCTCTGGATTATCTTCCTTAGTAGCAAGCGATTAGCTTT

CACAAAATCTTCTGTTTGTAGTTTTAAAAATTCATCAGGATTTTCTAACTCAACCTCAAA

ATATTTCATTTTACATTCCCTCATTTCATTTATTGATAAATTGAGTTTGCAAAAAAGAGT

GGACAATTTTTGTCTACTCTTAACCTTTAAAATAGTTTTTTTTAATCGATTTGAAGTTGC

CTAAATTATTACTTATTCGGTAAAATGAAGTATTGCTTTCAACAGATTTCCTTCAACTAC

ACTTCACTTGATTCAAACAAGGTGGGTACATTTCTATTCCCACAAACTCCTTGTCAATGG

AAACAAACACGTACCCACAGGGTAAATGGAAATAGAAACTGATAATTTCTAGCTATCACT

TCTACTCATTCCAAAAATTTTCTCACTCTGATACTTACCCACCATAAAGCAAAAAGCCTT

GCAATCAAGGCTTTCATTATCCCTTTCGTTCAAAGGTTTCTAAGCTTTTACGAGCAGAGC

GACACACTCAGCGGTTCGCTATCTCCGTTCTGTCTGCGTGCTAGCACTTGTCAATCACGG

ACAGCTATCGCATGGGCGGAAGTAAATGCTAATCTTCGTCGTTTTACTCCTTGACTAGCA

AACTTACCGCCTCAACATGTCCTGTATGTGGAAATAAAACACGATTAAAGATAAGGGAAG

ATACTGAATTAAAAAAATTCCCCCTCTATTGTCCGAAATGCAGACAAGAAAATTTAATTG

AAATAAAGCAGTTCAAAGTAACTGTGATTACAGAGCCAGACGCAAAGACGCAGAGCCGAT

AAAATGAGATTAATACAATCTCATTTTATCGGCTCTTTCCGTTATGTATGGATTCTTTTA

ATTAGTCTTCGATGTTTCTTGCTTCGTTGATACCGCTGGCTAAAGATTCCATTAAGGATA

GTTCTTTGTCTGTAAAGCTATCCATGTATTTCTCTATCTGTAATCGTCGGGTGCTTTTTA

CCAAGTTATTAGCAGGTAAGAAAAATTCATCAACGGAAACATGAAGTAACGATACAAGGT

CATAAAGAACTTGTATGCTGGGGTGTTGCCCTTTATTTTCAATATTAGTTAAGTACCGTG

GGTCAATTTCAATCAATGCTCCCACTTGTTCACGAGTTAAACCTCGTTTCAATCGAGCTT

CTTTAATGGCTAAACCAAAGGCTCTAAAATCATATTTATCTTCTTTTTTACGCATAGTAG

ACCACCTCTATACATTTTATTGTTCCTACTGAATTAAAAACAGGTATAGAAAAACGTGTT

ATATGGTTTATAGGTTTATATTTAATAAAAAGCACTACTAAACGCCAATAAAAAAACCGT

TATATGGTAGTGCTATTTACGCTGTTAAAATATTGTATATTACTTCCAAATGGCGGTTTG

TTGGAGGTCAACGTCGCCATGAAGTACATCATATACAATAAATTTCCTTACATTGGGTTC

TTGTCAAAAAAAGTCGTCTATCTGCAATAGATAAGTACGTCCACCAATGTGGTTTTATAA

ATCATATAGATAGAATAACAGAAGCATGTAAACAGAGAAATAAATCTGTTTATATGCTTT

TTTGGCTATTCAGAACTTTTTTACAAAGTTTATTTATCAGTAATGCAACAAATCCCCCTT

TCACATTGGGACTAAGAGTGAAAGGAGATAAACGAGCAAGGCTCACTTCCTTTCCTAGAC

AGAAAGGGGGTGAGAAACATGAAACCATCTTCTTTTCAGACCACAATAGAAAATCAGTTT

GACTATATCTGTAAACGTGCTATGGAAGACGAGCGAAAGAATTATATGCTTTATCTTTCA

AGGATTGCAAAGCGTGAGGTGTCCTTTTCGGATGTTGGCGATTATCTTGTTAGCCAGTTT

GCGACAACAGATAACTATTCAACTGACTTTCAGATTTTTACACTCAATGGGTTATCAGTA

GGCGTTGAAAATGATTTGTTGAGTGAAGCATTACGTGAGTTGCCAGACAAGAAACGTGAA

ATTCTACTGCTGTTTTACTTTATGGACATGAGCGATTCAGAAATTGCAGACCTGTTGAAA

TTGAACCGTTCTACTGTCTATCGGCATAGAACCAGTGGACTAGCCTTAATTAAAAAGTTT

ATGGAGGAATTTGAAGAATGAAAACACAATATCCTATGATTCCCTTTCCTCTCATTGTAA

AGGCAACAGATGGCGATACCGAAGCGATTAACCAGATTCTACATCATTACAGAGGGTACA

TAACGAAGCGTTCCCTACGACTTATGAAAGATGAATATGGCAATCAAAGTATGGTCGTTG

ATGAAGTCTTACGTGGAAGAATGGAAACCAGACTGATTACAAAGATTTTGTCATTTGAAA

TTAAGTAATATCCTCTCTCCTTTCGTGGAAGCGTGCTAAACCATTCCACGCTTCCCGAAC

AGGGAGGTTTGTTATTCCACCAAAGCATATTGAGCTTTCAATGTGTTTTGATAGGCTAAC

GAGCCATTGTTCTTTGAAAACTGAATAAAAGTAATCGAATACGTTTCGATAAGAAAAGAG

CCAACGGAACTAACCGCCATGACCTATCTTATAAAGATAGCGAGCGATTCATGTTAGTGA

TCCGAGAAGCAATCTTTAGCAGGATTGCCTGCAACGACATTCTTATCGTGATAATGATAC

TCCCATACAGTCAATAGTCCGAGCGTGATAAAACCGTCGCAGGCAATGAGTATGGCTACA

TGAGAACCATGCAGGGGTGGAACTCCCGTGAGCTTTGCTAAAGCTGTTCGATTGCTGGTA

AAACAACTTTTATGAAATCCAAATAAGTGATTTGGAAAGGAGGATTTTATGAAGCAGACT

GACATTCCTATTTGGGAACGTTATACCCTAACCATTGAAGAAGCGTCAAAATATTTTCGT

ATTGGCGAAAACAAGCTACGACGCTTGGCAGAGGAAAATAAAAATGCAAATTGGCTGATT

ATGAATGGCAATCGTATTCAGATTAAACGAAAACAATTTGAAAAAATTATAGATACATTG

GACGCAATCTAGCGTCGCCAAAGGGTCTTGTATATGATAAAATAGTATTAAGTCGTATCA

AGGCTCTTTCCATAAAGGAAAGGAGCAAATGCCATGTCAGAAAAAAGACGTGACAATAAA

GGTCGAATCTTAAAGACTGGAGAGAGCCAACGAAAAGACGGAAGATACTTATACAAATAT

ATAGATTCATTTGGAGAACCGCAATTTGTTTACTCGTGGAAACTTGTGGCTACAGACCGA

GTACCAGCAGGAAAGCGTGATTGTATCTCACTTAGAGAGAAAATCGCAGAGTTACAGAAA

GACATTCATGATGGTATTGATGTTGTAGGAAAGAAAATGACACTCTGCCAGCTTTACGCA

AAACAGAACGCTCAAAGACCAAAGGTTAGAAAAAACACTGAAACTGGACGCAAATATCTT

ATGGATATTTTGAAGAAAGACAAGTTAGGTGTAAGAAGTATTGACAGTATTAAGCCATCA

GACGCTAAAGAATGGGCTATTAGAATGAGTGAAAATGGTTATGCTTATCAAACCATCAAT

AACTACAAACGTTCTTTAAAGGCTTCATTCTATATTGCTATACAAGATGATTGTGTTCGG

AAGAATCCATTTGACTTTCAACTGAAAGCAGTTCTTGATGATGATACTGTCCCTAAGACC

GTACTAACAGAAGAACAGGAAGAAAAACTGTTAGCCTTTGCAAAAGCTGATAAAACCTAC

AGCAAAAATTATGATGAAATTCTGATACTCTTAAAAACAGGTCTTCGTATTTCAGAGTTT

GGTGGTTTGACACTTCCAGATTTAGATTTTGAGAATCGTCTTGTCAATATAGACCATCAG

CTATTGAGAGATACTGAAATTGGGTACTACATTGAAACACCAAAGACCAAAAGTGGCGAA

CGTCAAGTTCCTATGGTTGAAGAAGCCTATCAAGCATTTAAGCGAGTGTTAGCGAATCGA

AAGAATGATAAGCGTGTTGAGATTGATGGATATAGTGATTTCCTCTTTCTTAATAGAAAG

AACTATCCAAAAGTGGCAAGTGATTACAACGGCATGATGAAAGGTCTTGTTAAGAAATAC

AATAAGTATAACGAGGATAAATTGCCACACATCACTCCACATAGTTTGCGACATACATTC

TGTACCAACTATGCAAATGCAGGAATGAATCCAAAGGCATTACAGTACATTATGGGACAT

GCTAATATAGCCATGACGCTGAACTATTACGCACATGCAACATTCGATTCTGCAATGGCA

GAAATGAAACGCTTGAATAAAGAGAAGCAACAGGAGCGTCTTGTTGCTTAGTAGTACAAA

TGAATTTACTACTTATTTACCACTTCTGACAGCTAAGACATGAGGAAATATGCAAAGAAA

CGTGAAGTATCTTCCTACAGTAAAAATACTCGAAAGCACATAGAATAAGGCTTTACGAGC

ATTTAAGAAAATATAAAAAGATAATTAGAAATTTATACTTTGTTT

>GA13494/Tn2009

AAAATAGCATAAAAATCTAGTTATCCGCATAAAAACTGGACTTATCACACTTTATCAAGG

TCAAAACCACTCAATTTACTACTAATTTACTACTTATGAATGAGCTTTGATACGACGATT

TATCCTTGAAAAGTGAAGATATAAAGATACTTCCAATAAAATTTGAATATTTAATAGGTA

GACACTTCAAAAAATGAGGTGTCTATTTTTTTACCCGATTTTGAAAGGAAGTGAACTTAT

GAAAACAAAAAATCAAGAATCAAAAGGTCGTTCCCCACTCTTTAAGACCATCAAACATTC

ATTCAGCCAATAAAAAGAAAGGATAGGTAAAAATATGGAACTTAAATTTGTGATTCCCAA

CATGGAAAAAACATTCGGCAATTTAGAATTTGCTGGCGAGGATAAAGTCGTTCAGCGAAG

AATCAACGGACGGCTAACTGTCTTATCAAGAAGCTATAATCTCTATTCTGATGTTCAAAG

AGCAGATGATATTGTGGTGGTGCTTCCTGCTGAAGCTGGCGAAAAACATTTCGGCTTTGA

GGAACGTGTGAAGTTAGTCAATCCACGTATTACCGCAGAGGGCTACAAAATCGGCACTCG

TGGTTTTACAAATTACCTTTTACATGCTGACGACATGATAAAAGAATAAAGAAAGAGAGG

AAAAATGATGAGATTAGCAAATGGCATTGTATTAGATAAAGACACGACTTTTGGAGAATT

GAAATTCTCTGCTCTACGTCGTGAAGTGAGAATCCAAAATGAAGACGGGTCGGTTTCAGA

TGAAATCAAGGAACGTACCTATGACTTAAAATCCAAAGGACAAGGACGCATGATTCAAGT

AAGTATTCCTGCCAGCGTGCCTTTGAAAGAGTTTGATTATAACGCACGGGTGGAACTTAT

CAATCCCATTGCGGACACCGTTGCTACTGCCACCTATCAAGGAGCAGATGTTGACTGGTA

TATCAAGGCAGACGATATTGTGCTGACAAAGGATTCTAGTTCATTCAAAGCTCAACCACA

AGCAAAGAAAGAACCGACACAAGACAAATAGTCGCTAGGTAGAAAGGAGACTTTTTCGCA

TGAAACAGCGTGGTAAAAGGATTCGCCCATCTGGTAAAGATTTAGTCTTTCATTTTACGA

TAGCGTCACTCCTGCCTGTTTTCCTGCTGGTTGTCGGACTGTTTCATGTGAAGACAATCC

AGCAGATCAACTGGCAGGATTTTAACCTATCACAAGCAGATAAGATTGACATTCCCTATT

TAATTATCAGTTTCAGTGTCGCAATTCTTATCTGCTTGCTGGTAGCGTTTGTATTCAAAC

GGGTTCGCTATGATACGGTTAAACAACTTTACCACCGTCAAAAACTGGCAAAGATGATAC

TTGAAAACAAGTGGTATGAATCTGAACAGGTCAAAACAGAGGGTTTCTTTAAAGATAGTG

CTGGTCGTACAAAGGAAAAGATAACCTACTTCCCTAAAATGTATTATCGACTTAAAAATG

GCTTGATACAGATACGGGTGGAAATCACGCTGGGAAAATATCAAGACCAACTCTTACACT

TGGAAAAGAAATTAGAGAGTGGCTTGTACTGTGAGCTGACGGATAAAGAGTTAAAGGATT

CCTATGTGGAATATACTTTGCTCTATGACACCATAGCCAGTCGTATTTCTATTGATGAAG

TAGAAGCTAAAGATGGTAAACTTCGCTTAATGAAAAACGTATGGTGGGAATATGATAAGC

TCCCTCATATGTTGATTGCTGGTGGTACAGGTGGCGGTAAAACTTACTTTATACTGACAC

TGATTGAAGCCTTGCTTCATACAGATTCAAAACTGTATATTCTTGACCCGAAAAATGCTG

ACCTTGCGGACTTAGGTTCTGTGATGGCAAATGTCTACTATAGAAAAGAAGACTTGCTTT

CTTGCATTGAAACATTCTATGAAGAAATGATGAAACGTAGTGAGGAAATGAAGCAGATGA

AGAACTATAAGACTGGCAAAAATTATGCTTACTTAGGTCTCCCGGCACACTTCTTAATCT

TTGATGAATACGTCGCTTTCATGGAAATGCTGGGAACAAAAGAAAACACCGCAGTTATGA

ATAAGCTGAAACAGATTGTCATGTTAGGTCGTCAAGCTGGCTTCTTTCTAATACTGGCTT

GTCAACGTCCAGACGCAAAATATTTAGGCGACGGAATCCGTGATCAGTTTAATTTCAGAG

TGGCTTTAGGTCGTATGTCTGAAATGGGCTATGGCATGATGTTTGGCAGTGACGTACAAA

AGGATTTCTTCTTAAAGCGAATCAAAGGTCGTGGCTATGTTGATGTAGGAACAAGTGTCA

TATCAGAGTTTTATACTCCCCTTGTACCAAAAGGATATGATTTCTTGGAGGAAATTAAAA

AGTTATCCAACAGCAGACAGTCCACGCAGGCGACGTGCGAAGCGGAAGTCGCAGGTGTGG

ACTGATCTTGCTGGCTGGTGTGGCAATAGCCACGCCAGCACTTAACCCCCCGTATCTAAC

AGGGGGGTACAAATCGACAGGAAACAGTCAAAAAAACATTAGAAAATCCTTTGGTTACAA

GGGATTTACAAAATTTCAGCGTATGTCAAATGGGCTTTAAAAGTTGACATACGCCTTTTT

GATTGGAGGGATTTTTACTGAATGAACAAACTTGGTTACAGCATTTAAAAGAAAAACGCT

TGGCTTATGGACTATCTCAAAACCGTTTAGCTGTTGCGACTGGTATTACAAGGCAGTATC

TAAGCGATATTGAAACAGGAAAAGTCAAGCCATCAGAGGATTTACAGCAGTCCCTTTGGG

AAGCTCTGGAACGCTTCAATCCCGACGCTCCCCTTGAAATGCTGTTTGATTATGTAAGGA

TTCGCTTTCCGACAACAGACGTACAGCAGGTGGTCGAAAACATCTTACAACTGAAACTGT

CCTATTTTCTTCATGAGGACTATGGTTTCTATTCTTATTCAGAGCATTATGCTTTAGGCG

ACATATTCGTCCTTTGCTCCCATGAACTGGACAAAGGAGTTCTGGTGGAATTGAAAGGTC

GTGGGTGCAGACAATTTGAAAGCTATCTTCTGGCACAACAAAGAAGCTGGTATGAGTTCT

TTATGGACGTTTTGGTGGCTGGCGGTGTGATGAAACGCCTTGACCTTGCCATTAACGATA

AGACAGGGATTTTGAATATCCCTGTACTCACTGAAAAGTGCCAACAGGAAGAATGTATCT

CCGTCTTCCGCAGTTTTAAAAGCTATCGCAGTGGCGAACTGGTACGCAAAGAGGAAAAGG

AATGTATGGGAAACACCCTCTATATCGGTTCATTACAAAGTGAAGTTTATTTCTGTATCT

ATGAAAAGGACTACGAGCAGTACAAGAAAAATGATATTCCCATTGAAGACGCAGAAGTAA

AAAACCGTTTTGAGATTCGATTGAAAAATGAGCGTGCCTATTATGCAGTCCGTGATTTAC

TCGTCTATGACAATCCAGAGCATACCGCCTTTAAAATTATCAATCGGTATATCCGTTTTG

TAGATAAAGACGATTCCAAACCTCGTTCTGATTGGAAACTGAATGAAGAATGGGCTTGGT

TTATTGGGAACAATCGTGAACGATTAAAACTAACCACAAAACCAGAGCCTTACTCCTTCC

AAAGGACGCTGAACTGGCTATCTCATCAAGTTGCCCCGACCTTAAAGGTTGCGATTAAAC

TTGATGAAATCAACCAGACGCAGGTTGTAAAAGACATTCTCGACCATGCGAAACTGACAG

ACCGACACAAGCAGATTTTGAAGCAACAGTCAGTAAAAGAACAGGACGTGATAACAACAA

AAAAATAACTCAAATACAAATTCATTGAATATAGAGAGGAGAACATTTTTATGAATTTTG

GACAAAACCTTTATAACTGGTTTCTATCAAACGCTCAATCACTGGTGCTTTTAGCAATCG

TTGTGATTGGCTTGTATCTTGGCTTCAAGCGTGAGTTTAGCAAACTGATTGGCTTTTTAA

TTATTGCGATTATTGCGGTTGGCTTAGTCTTCAACGCTGCTGGAGTAAAAGACATTTTAC

TAGAGCTATTCAATCGCATTATTGGTGCTTAAATAAAACCGTTCTTTTGTGGAATATAAG

TGGTTTTCTTATGTTCCGCAAAGGAATGGTACACCAAACGAAGTGCGGTAGGGATTTTTG

AATCTCTACAAAGAAAGGACGTGAATATATGGACGATATGCAAGTCTATATTGCGAATTT

AGGCAAATACAATGAGGGCGAATTGGTCGGTGCGTGGTTTACCTTTCCCATTGACTTTGA

GGAAGTCAAAGAGAAAATCGGCTTGAATGATGAATATGAGGAATACGCCATTCATGACTA

CGAGTTACCCTTTACGGTTGACGAATACACTTCCATTGGCGAACTCAATCGACTATGGGA

AATGGTATCGGAATTACCCGAAGAATTACAATCGGAGCTATCTGCTCTGCTCACTCATTT

TTCAAGCATTGAAGAACTAAGCGAACATCAAGAGGATATTATCATTCATTCCGATTGTGA

TGATATGTATGACGTGGCACGCTACTACATTGAAGAAACGGGTGCTTTAGGCGAAGTACC

AGCTAGTCTTCAAAACTATATTGATTATCAAGCCTATGGTCGGGATTTAGACCTTTCAGG

AACGTTTATCTCAACCAATCATGGGATTTTTGAAATCGTCTATTAAATCTGTCGGTACAT

TACTACTGGCAGATTTTCTATTTTACGGGGTGGCTCAATCAGCTACCCCTATTTTTTATG

AAAGGATTGATTACATGAAGAAAATACGAAGCTATACCAGTATCTGGTCTGTGGAAAAGG

TACTGTATTCTATCAATGATTTTAGACTTCCGTTTCCCATAACCTTTACGCAAATGACAT

GGTTTGTCGTGTCACTCTTTGCAGTGATGATACTTGGCAACTTGCCCCCTCTTTCCATGA

TAGAGGGAGCATTTCTCAAATACTTTGGGATTCCTGTGGCTTTCACATGGTTTATGTCTA

CAAAAACTTTTGATGGTAAAAAGCCTTATGGATTTTTGAAGTCTGTCATTGCTTATGCAC

TGCGACCAAAGCTGACCTATGCAGGAAAAAAAGTAACGCTTGGCAGAAACCAGCCACAAG

AAGCCATTACAGCAGTTAGGAGTGAATTTTATGGCATATCCAATTAAATACATTGAAAAC

AATCTCGTCTGGAATAAAGACGGGGAATGTTATGCTTACTATGAGCTTGTTCCTTACAAT

TACTCATTTCTAAGTCCAGAACAGAAAATACAAGTGCATGATTCTTTCAGACAGCTTATC

GCACAAAATCGTGATGGCAAAATTCATGCTTTACAAATCAGTACAGAATCCAGCATACGT

TCTGCACAAGAGCGTTCCAAAAATGAAGTCACTGGCAAGCTCAAAGCGGTTGCCTATGAC

AAAATCGACCAACAGACAGACGCTTTAATATCCATGATTGGCGAAAATCAAGTGAACTAC

CGTTTCTTTATCGGCTTTAAGTTGCTTCTCAACGATCAGGAGTTTTCTATGAAAAGTCTT

ACCGTTGAAGCAAAAAATGCTTTGTCTGATTTTGTCTATGATGTGAACCATAAGCTGATG

GGCGATTTTGTTAGTATGAGTAATGATGAAATCCTGCGTTTTCAGAAGATGGAAAAGCTC

TTAGAAAATAAAATCTCTCGTCGTTTCAAAATCCGCAGGTTAGATAAGGACGACTTCGGC

TATCTGATTGAACACCTTTACGGACAGACAGGCACTGCCTATGAAGAGTATGAGTACCAT

CTATCAAAGAAAAAGCTGGATAATGAAACGCTGATTAAATACTATGACTTGATTAAGCCT

ACTCGCTGTTTGGTGGAAGAAAAACAGCGATATTTGAAAATCCAGCAGGAAGATGAAACC

GTCTATGTAGCTTACTTTACCATTAACAGCATTGTCGGAGAACTGGACTTCCCGTCCTCT

GAAATCTTCTACTACCAGCAACAGCAATTTACATTCCCGATTGATACGTCAATGAATGTG

GAAATTGTAGCGAATCGTAAAGCCCTATCTACTGTCCGCAATAAAAAGAAAGAACTGAAA

GACTTGGATAACCACGCTTGGCAAAGTGATAATGAAACCAGCTCCAATGTGGCGGAAGCT

CTGGAAAGTGTGAATGAGCTGGAAACCAATTTAGACCAAAGCAAGGAATCTATGTACAAG

CTGTCTTATGTGGTAAGGGTATCAGCAAATGATCTTGACGAACTCAAACGTCGTTGTAAT

GAAGTGAAAGATTTTTATGACGATTTAAGCGTAAAACTGGTACGACCATTTGGGGATATG

CTCGGCTTACATGAAGAATTTTTACCTGCCAGCAAGCGTTATATGAATGATTATATTCAA

TACGTGACCTCTGATTTCCTCGCTGGTTTAGGTTTTGGTGCTACTCAAATGCTGGGGGAA

AATGAGGGGATTTATGTTGGCTACAGCTTAGATACTGGACGCAATGTCTATCTGAAACCT

GCTCTTGCCAGTCAAGGGGTTAAGGGTTCAGTAACCAATGCGTTAGCGTCGGCTTTTGTT

GGTTCGCTGGGTGGTGGTAAATCCTTTGCGAATAACCTTATCGTCTATTATGCGGTGCTT

TATGGGGCACAAGCAGTGATTGTAGACCCAAAAGCAGAACGTGGCAGATGGAAAGAAACC

TTGCCAGAAATTTCCCATGAAATCAATATCGTCACTCTGACTTCTGATGAGAAAAACAAA

GGCTTACTTGACCCTTATGTGATTATGAAAAATCCCAAAGATTCTGAATCACTGGCTATT

GATATTCTGACATTCCTTACGGGGATTTCCTCTCGTGATGGGGAACGCTTCCCAATCCTT

AGAAAAGCCATTCGTGCAGTAACCAATAGTGAAGTACGAGGGTTGATGAAAGTGATTGAG

GAATTACGGGTTGAGAATACGCCACTAAGTACCAGTATAGCCGACCATATCGAAAGTTTT

ACAGACTATGACTTTGCACATTTATTATTCAGTAATGGTTATGTGGAGCAGTCTATCAGC

TTAGAAAAACAACTGAACATTATACAGGTTGCGGACTTGGTACTTCCCGACAAGGAAACT

TCCTTTGAGGAATATACCACTATGGAGCTTTTATCCGTTGCTATGCTGATTGTCATTAGT

ACCTTTGCTTTAGACTTTATCCATACAGACCGAAGCATTTTCAAGATTGTAGATTTAGAC

GAAGCATGGAGCTTTTTACAGGTAGCACAAGGAAAAACACTATCTATGAAGCTGGTTCGG

GCTGGTCGTGCTATGAACGCTGGAGTATATTTCGTGACCCAAAATACAGACGACCTCTTA

GATGAAAAACTGAAAAATAACCTCGGCTTAAAATTTGCATTTCGTTCCACTGACCTTAAC

GAGATTAAAAAGACCTTAGCCTTTTTTGGTGTAGACCCAGAGGACGAAAACAATCAGAAG

CGATTGCGTGATTTGGAAAACGGGCAATGCCTTATCAGTGATTTATATGGTCGTGTCGGT

GTGATACAGTTCCACCCTGTATTTGAAGAACTGCTCCATGCCTTTGATACCAGACCACCT

GTGCGAAAAGAGGTGTAAATGTGAAACCATCAATAGTAAACAGAATAAAATCAAACTGGA

CGCTGAAACGTCTAGGTAAAGTGGCAATGACAGTGGCTTTCACACTTGTGATTGCCATTT

TTCTTTTAGCCATGCTGGGAACGGTGGTTCAAGCTGCGGGCTTGGTAGATGATACGGTCA

ATGTGGCAAATGAATACAGCCGATACCCACTTGAAAACTATCAACTGGATTTTTATGTGG

ATAATAGCTGGGGCTGGCTTCCGTGGAACTGGTCGGACGGGATTGGAAAACAGGTCATGT

ATGGACTATATGCCATTACCAATTTTATTTGGACAATCAGTTTGTATGTTTCCAATGCGA

CAGGTTACTTAGTACAGGAAGCCTATTCCTTAGACTTCATTTCCGCTACAGCAGATTCCA

TTGGTAAGAATATGCAGACCTTAGCTGGTGTGAGTGCAAACGGATTTTCAACAGAGGGTT

TCTATGTTGGATTCCTCTTACTCTTGATTTTGGTTCTTGGGGTTTATGTTGCCTATACGG

GACTGATAAAGAGAGAAACCACAAAGGCAATTCATGCCATTATGAATTTTGTGCTGGTGT

TTATCCTATCGGCTTCCTTTATTGCCTACGCTCCCGACTACATTAAAAAAATCAATGACT

TTTCATCAGACATCAGTAATGCCAGTTTATCACTTGGCACGAAGATTGTCATGCCCCATT

CCGATAGTCAAGGCAAGGACAGCGTGGACTTAATCAGAGATAGCCTGTTTTCCATACAGG

TTCAGCAACCGTGGCTACTGCTTCAATACAACAGTTCAGACATTGAAAGTATCGGTATTG

ACCGTGTGGAAAGCCTGCTCTCCACCAGCCCAGATTCCAACAATGGCGAAGACAGAGAAA

AAATTGTTGCGGAAGAAATTGAAGACAGAAGCAATACCAATCTAACCATTACAAAGACCA

TTAACCGTTTAGGTACAGTCTTCTTCCTATTTGTCTTCAATATTGGGATTTCCATATTTG

TATTCCTATTAACAGGAATCATGATTTTCTCGCAGGTACTTTTTATCATCTATGCTATGT

TTCTGCCTGTGAGCTTTATTTTAAGCATGATTCCATCATTTGATGGTATGTCAAAACGAG

CCATAACAAAGCTCTTTAATACCATTTTGACACGAGCTGGAATCACATTGATTATTACGA

CAGCATTTAGTATTTCAACCATGCTCTATACCTTATCGGCTGGTTATCCGTTCTTTTTGA

TTGCTTTTCTACAGATTGTGACCTTTGCAGGAATCTACTTCAAGCTGGGCGATTTAATGA

GTATGTTTTCTCTACAGAGTAACGATTCTCAAAGTGTGGGAAGTCGTGTGATGAGAAAAC

CTCGTATGCTTATGCACGCTCACATGCACCGTCTACAGCGGAAACTTGGACGTTCCATGA

CTACTCTAGGGGCTGGGTCTGCCATTGTTACAGGTAAAAAAGGACAGTCGGGTTCGGGGA

GTTCTGCAAGGACACAAGCAGATCACTCCCGACCAGACGGAAAGGAAAAATCAACACTTG

GAAAACGTATCGGTCAAACCATCGGTACAGTAGCTGATACCAAAGACAGAATGGTAGACA

CTGCTAGTGGTTTGAAAGAACAGGTTAAAGATTTGCCGACCAATGCAAGATATGCAGTAT

ATCAAGGAAAATCCAAAGTAAAAGAGAATGTCCGTGATTTAACCAGTAGTATTTCTCAAA

CCAAAGCGGACAGAGCCAGTGGACGCAAGGAACAGCAGGAACAAAGGCGAAAAACCATTG

CGAAGCGTCGCTCTGAAATGAAACAGGTCAAACAGAAAAAACAGCCTGCTTCTTCTGTTC

ATGAAAGACCGACTACAAGACAAGAACAATATCATGATGAACAGACCTCAAAACAGTCTA

ATATTCAGACTTCATATAAGGAATCTCAACAAGCCAAACAAGAGCGTCCAGCAGTTAAGT

CCGATTTTTCAAGTCCAAAAGTGGAACGCCAAGGCAATACCGTTCAAGAAAAAACCGTTC

AAAAGCCAGCAACTTCAACCACTACAGCAGATAGAACTTCACAACGTCCAATCACAAAAG

AACGTCCGTCTACTGTTCAAAGAGTACCACTACAAAATACAAGAAGTAGACCACCAATCA

AAACCGCCACCATTAAGAAAGTCGGTAAGAAACCATGAAGTTGAAAACTTTAGTGATTGG

TGGTTCTGGATTATTCTTGATGGTCTTCTCACTGCTTCTGTTTGTTGCCATTTTATTTTC

AGATGAACAGGACAGCGGAATTTCCAATATTCATTATGGAGGTGTGAATGTTTCCGCAGA

AGTGCTGGCTCATAAGCCTATGGTAGAAAAATATGCCAAAGAATATGGCGTTGAAGAATA

TGTCAACATACTTCTTGCGATTATACAGGTGGAATCGGGCGGTACTGCGGAAGATGTTAT

GCAGTCCTCGGAATCCCTCGGTCTTCCACCTAATTCATTGAGTACAGAAGAATCCATTAA

GCAAGGTGTGAAGTATTTCAGTGAATTATTAGCCAGTAGCGAAAGGCTCAGTGTAGATTT

AGAATCGGTTATCCAGTCCTACAATTATGGTGGTGGTTTCTTAGGGTATGTGGCTAATCG

TGGAAATAAATATACCTTTGAACTGGCTCAAAGTTTCTCAAAAGAGTATTCAGGTGGCGA

AAAAGTGTCTTACCCCAATCCCATAGCCATACCTATCAATGGGGGCTGGCGATACAACTA

TGGCAATATGTTTTATGTGCAACTGGTAACGCAGTATCTTGTCACAACAGAGTTTGATGA

TGATACGGTACAAGCCATCATGGACGAAGCACTGAAATATGAGGGCTGGCGATACGTTTA

CGGTGGAGCTTCCCCGACTACTTCTTTTGATTGTAGCGGACTGACACAATGGACGTATGG

AAAAGCTGGAATTAACTTACCACGAACCGCACAACAGCAATATGATGTGACCCAGCATAT

CCCACTATCGGAAGCACAAGCTGGCGATTTGGTTTTCTTTCATTCTACCTATAACGCTGG

CTCTTATATTACTCATGTTGGGATATACCTTGGCAATAACCGTATGTTTCATGCAGGCGA

CCCAATCGGTTATGCCGACTTAACAAGCCCCTACTGGCAACAGCATTTAGTGGGAGCAGG

ACGAATCAAACAATGAGAAAGGAAGATTTAATGATGAAATTTAGAAAAAATCAGAATAAA

GAAAAACAGATACCAAAGGAAAAGAAACCTCGTGTCTATAAGGTCAATCCTCATAAAAAG

GTTGTGATTGCCTTGTGGGTACTTTTAGGGCTTAGTTTCAGCTTTGCGATATTCAAGCAC

TTTACAGCTATAGATACTCATACTATTCACGAAACAACTATCATAGAAAAGGAATACGTT

GATACTCATCATGTAGAAAATTTTGTAGAGAACTTTGCGAAAGTCTACTATTCATGGGAG

CAATCCGATAAGTCCATTGATAATCGAATGGAAAGTCTAAAAGGCTATCTGACAGATGAA

CTTCAAGCTCTCAATGTTGATACAGTACGCAAAGATATTCCTGTATCGTCTTCTGTAAGA

GGATTTCAGATATGGACGGTAGAGCCAACTGGCGACAATGAGTTTAATGTAACCTACAGT

GTAGACCAGCTCATTACAGAGGGAGAAAATACAAAGACCGTCCACTCTGCTTATATAGTG

AGTGTCTATGTAGATGGTTCTGGAAATATGGTACTGGTTAAGAATCCGACCATTACCAAC

ATACCTAAGAAATCAAGTTATAAACCAAAAGCCATTGAAAGTGAGGGGACGGTTGATTCC

ATTACAACCAATGAAATCAATGAGTTTTTAACGACGTTCTTCAAGCTCTATCCTACAGCG

ACAGCCAGTGAACTTTCCTACTATGTGAATGACGGGATATTAAAACCAATCGGAAAAGAG

TACATCTTTCAAGAACTGGTAAATCCTATTCACAATCGTAAGGATAATCAAGTCACGGTA

TCGCTGACAGTGGAGTATATCGACCAGCAGACCAAAGCAACGCAGGTATCTCAATTTGAT

TTGGTACTTGAAAAGAACGGGAGTAATTGGAAGATTATAGAATAACAAATATTGGTACAT

TATTACAGCTATTTTGTAATCACGTACTCTCTTTGATAAAAAATTGGAGATTCCTTTACA

AATATGCTCTTATGTGCTATTATTTAAGTATCTATTTAAAAGGAGTTAATAAATATGCGG

CAAGGTATTCTTAAATAAACTGTCAATTTGATAGTGGGAACAAATAATTGGATGTCCTTT

TTTAGGAGGGCTTAGTTTTTGTACCCAGTTTAAGAATACCTTTATCATGTGATTCTAAAG

TATCCGGAGAATATCTGTATGCTTTGTATGCCTATGGTTATGCATNNNNNCACACACTTA

ATTAATTAAGTGTGTGNNNNNATGCCCTTTTGGGTTTTTGAATGGAGGAAAATCACATGA

AAATTATTAATATTGGAGTTTTAGCTCATGTTGATGCAGGAAAAACTACCTTAACAGAAA

GCTTATTATATAACAGTGGAGCGATTACAGAATTAGGAAGCGTGGACAAAGGTACAACGA

GGACGGATAATACGCTTTTAGAACGTCAGAGAGGAATTACAATTCAGACAGGAATAACCT

CTTTTCAGTGGGAAAATACGAAGGTGAACATCATAGACACGCCAGGACATATGGATTTCT

TAGCAGAAGTATATCGTTCATTATCAGTTTTAGATGGGGCAATTCTACTGATTTCTGCAA

AAGATGGCGTACAAGCACAAACTCGTATATTATTTCATGCACTTAGGAAAATGGGGATTC

CCACAATCTTTTTTATCAATAAGATTGACCAAAATGGAATTGATTTATCAACGGTTTATC

AGGATATTAAAGAGAAACTTTCTGCCGAAATTGTAATCAAACAGAAGGTAGAACTGTATC

CTAATATGTGTGTGACGAACTTTACCGAATCTGAACAATGGGATACGGTAATAGAGGGAA

ACGATGACCTTTTAGAGAAATATATGTCCGGTAAATCATTAGAAGCATTGGAACTCGAAC

AAGAGGAAAGCATAAGATTTCAGAATTGTTCTCTGTTCCCTCTTTATCATGGAAGTGCAA

AAAGTAATATAGGGATTGATAACCTTATAGAAGTGATTACGAATAAATTTTATTCATCAA

CACATCGAGGTCAGTCTGAACTTTGCGGAAAAGTTTTCAAAATTGAGTATTCGGAAAAAA

GACAGCGTCTTGCATATATACGTCTTTATAGTGGCGTACTGCATTTGCGAGATTCGGTTA

GAATATCGGAAAAGGAAAAATAAAAATTACAGAAATGTATACTTCAATAAATGGTGAATT

ATGTAAAATCGATAAGGCTTATTCCGGGGAAATTGTTATTTTGCAGAATGAGTTTTTGAA

GTTAAATAGTGTTCTTGGAGATACAAAGCTATTGCCACAGAGAGAGAGAATTGAAAATCC

CCTCCCTCTGCTGCAAACGACTGTTGAACCGAGCAAACCTCAACAAAGGGAAATGTTACT

TGATGCACTTTTAGAAATCTCCGACAGTGACCCGCTTCTGCGATATTATGTGGATTCTGC

GACACATGAAATCATACTTTCTTTCTTAGGGAAAGTACAAATGGAAGTGACTTGTGCTCT

GCTGCAAGAAAAGTATCATGTGGAGATAGAAATAAAAGAGCCTACAGTCATTTATATGGA

AAGACCGTTAAAAAAAGCAGAGTATACCATTCACATCGAAGTGCCGCCAAATCCTTTCTG

GGCTTCCATTGGTTTATCTGTATCACCGCTTCCGTTGGGAAGTGGAATGCAGTATGAGAG

CTCGGTTTCTCTTGGATACTTAAATCAATCATTTCAAAATGCAGTTATGGAAGGGATACG

CTATGGTTGCGAACAAGGATTATATGGTTGGAATGTGACGGACTGTAAAATCTGTTTTAA

GTATGGCTTATACTATAGCCCTGTTAGTACCCCAGCAGATTTTCGGATGCTTGCTCCTAT

TGTATTGGAACAAGTCTTAAAAAAAGCTGGAACAGAATTGTTAGAGCCATATCTTAGTTT

TAAAATTTATGCGCCACAGGAATATCTTTCACGAGCATACAACGATGCTCCTAAATATTG

TGCGAACATCGTAGACACTCAATTGAAAAATAATGAGGTCATTCTTAGTGGAGAAATCCC

TGCTCGGTGTATTCAAGAATATCGTAGTGATTTAACTTTCTTTACAAATGGACGTAGTGT

TTGTTTAACAGAGTTAAAAGGGTACCATGTTACTACCGGTGAACCTGTTTGCCAGCCCCG

TCGTCCAAATAGTCGGATAGATAAAGTACGATATATGTTCAATAAAATAACTTAGTGTAT

TTTATGTTGTTATATAAATATGGTTTCTTGTTAAATAAGATGAAATATTTTTTAATAAAG

ATTTGAATTAAAGTGTAAAGGAGGAGATAGTTATTATAAACTACAAGTGGATATTGTGTG

CTGAGAGCTTTCTTCTATACTAATAGACGAAAGGGTGTGAAAATGATTTTTAAATGATAC

TGTGGAACGGAACAGTAGCCCTAGTATTGACTACTGTCGTTTCTATTCATATTGGCTATT

CTAGGACTGAGATGAAAAAATCTATAAATGCTCAGAATAAAATTGAACCCGCAAATCTCC

CCAAAACAATGGTGAGTCATGTACTTGTATTATTCCGAAAAAATACACCTCTGGTGCAGT

GAGACAAATTGGTGTATCTTATAGTGGCTTCGTAGATGAAAGCTATACTCTACTATCACT

CTTTGATGATGTAGAACAAATTGAAAAAGATAATAGACTTCAGACAGCTATTGATGTTGT

CAGAGAACAGTTTGGTTTTTTAGCCATACAAAAAGGAACCGTCCTAACTGAAGGTTCCAG

AAATATTGAACGCAGTAAACTTATCGGTGGTCATTCCGCGGGTGGATTGGAGGGATTAAA

ATGAAACAAGAAAAAATACAGTACAATTTTCAGAAATCCGTAGCAAAGGATGTAATGATA

TTGAAATGCTTGAAAGATTTTTACATGGAATCGTTGAAACAGCAACTTCAAAACTTCGTC

AGAGAAAACTCAAAACAACTGAAATATCGATACGACTAGTACATGCTAAATCTGAAAACC

GATTACCATTGGAATTTACATTTAGCATTAAGCCAACAAGCTCATCTGTGATAATCTATA

CTGAGGTAATCAATCGCTTTAAAGAATGTTACACAGGTGGGGGAATTCAAGGTTTTACGA

TTCAATTTGATAAAAATACCCTTGCCTCTGCATAGAAAGGATTTGATATGATTGACCGTT

CATATTTACCATTTCAATCAGCAAGAGAGTACCAGGATACAAAGATGCAAAAATGGATGG

GCTTTTTCCTATCTGAACATGCATCAGCACTCTCTGATGATACAAACAAAGTAACGTACA

TGTCTGACTTATCACTAGAGAAGAAATTATTACTCCTCAGTCAAGTATACGCCGGGCAGC

TACGCACACGCATTCAAGTGATTGAAAAAACAAGCGTGTTTCCTACACTGGAACAATACC

AAGTCTGACCAAAGATTTCATTTTGATAAAAACTACAACAGGTCACATCAATTTGAAATT

AAAAGACATTATTAGTATTGAACTTGTCGAGGAGGTGCTCTATGAATCAGCTTGAGTTTC

AGCGTAATCACCTACAAATGGACTATTATAGCGAGAGCTACCAAGATTTTGAACGTGACT

TCTACCGCTACTCTAACATGAATATTCCATTGACCTTCCTAACTGATGATATCCTAAAAA

CAATGGCGACTTCACGTAAGAATTACTTTGTCCTCAATAAGGAAAAGTCCAGAGATAACC

GCGATCACTTCTTCATATTTGAAGTAAGTACCGTAGATGAGAATCCGCTAATCTATCATT

ATACATATAAGAAAACTACAATATATTTAGCAGAAAAATAGGAGCAGTTCAATTGACTGT

TCCTATTTTTAATATTCATAAAATCTAAAGTCTTTATACTCTTTAACAATGGAGTCGCCA

ACCAGAACAGACTATACTGACCAGCGACTACCTTAAATTTAATGTTTCAGATTTATTTTC

TTATCTCTAATTTCATAAACTACATCTGCTACATTTTCGAGTAATCGTTTATCGTGGGTG

ATAAACACGATAGTTCCGGTGTACTCCTTCATTAGTATTTCCAAAGCCTCTAAACTTGGT

ATGTCAAGGAAGTTACTGGGTTCATCCATTATTAGGATGTTATATCTACCCATGAGCATT

TTAGCAAGCAACAATTTTATAATTTCTCCACCGCTTAAAACAGATAAACTTTTTCCAATA

TCGTTCTGTTTGAACCCCATAGATGCTAGCACTGAACGAATTTCTGATATATTGTAGTCA

CAATCCTTCTGCATAAACTCCATAACATTCTGATTACTGTTGTACTTGTAACCATTCTGT

GCAAAGTAACCTATTTTTGCCTTAGGCGAAATAGAAATTCCTTCTTCATGGTTTAAGATC

ATTTGGATTAAAGTTGTTTTTCCGATTCCATTACCACCAGTTAACGCCACTTTTGCTCCT

AACGGAATTTGAAAAGATGCATTTTCAAACAGAGCCTTATCCCCAAATACTTTATTAATT

TCTGCACCGACTATAGGGTATGGATTATGGAGCTCCAATGCTTTACTTTGCCTGAAACGA

ATTCTGCGAATGCCTTCCGGAGCTTCTACTTTTCCTAAGGCCGCAATCCTGTGCTCTAGG

GTTTTAGCAGCATTATACATCTTTTTTCCTTACTTCCTATTGATTTTTGATGAGCTAAAC

GCCCTCCGTCTTCAGTACTTTTTTCTTTGAAGAACCTTTTGCCTTCTGTTCTATTTTACG

AGCCTGTTTTCGCTTTTCCTCCGCAGCCCTTTCCAATCGGGCACGTTCCGCAATAAATTG

TTCGTATTCTGCAGCTTGGCTCTTACGTTCTTCCTCTTTCTGACGAAGATAATCAGAATA

GTTTCCCCAATACTCAGTGATTTTGCCATCTTTCAGTTCCCATATTTTATCTACTATTTC

ATCAAGAAAATAGCGGTCATGGCTAATAACTAACAGTGCACCTGTAAAATATTTTAGCTG

TCCTATTAGAAAATCAATTCCTTCACGGTCTAAATGGCTCGTAGGTTCATCCGCTAAAAT

ACCATGAACCTGTGCCGATAAGGCCTGTGCTATTTTAAGCCTTGTTTCTTCACCACCGCT

CATAGTCTGTATATTTAATTGCTCAACACCTAGCTTGCCTACAAGTGCAAAATCTTTTTC

CTCCTGCAGAGTTACTTCGTCCAACTGGGGAATATAGGCAAGTTCACCCAGACGATTCAT

TTTACATCCTGGGGGAGTTAATTCTCCTAAAAGTACCCTGAGTAAAGTGCTTTTTCCAGC

ACCATTTGCTCCTACTAAACCAATACGGTCATAATCATATACTTCTAATTCATTTATATC

TAAAACATCGCGTCCTTTGAATTCCACACGAATGTCTTTTGCTTTTAATATTAATTCCAT

AACATTTCCTCCTGTCTATAATCGCATGCTTTCATTTGCTTGTATGCAGGGAAAACCCTG

CGATTTTAGCAGGAAGAGTTACATGAAAATAAGATACATAAATATTCCTCCAATATTGTT

TATTTTAAATCTAATTTTCTAACCTCAGTTATCATTTGGCAAACTATAGCAATGCCAATA

ATTAAAATACCTGATAGTAAAAACCAATGATTTACACCGATTTTATCAGCAAAGAATCCA

GAAAGAATTAACCCAATTGGCATAGCAAGTGACATGATACTTCCGATCAAAGAAAATACA

CGTCCTAAATATTCAGGCTTAATTTTCTCCTGAAAAAGAGCTGTTTGCACACCGCTATAA

AATGGCACCGAAAGCCCCATTATTGCACAGCAAACTACGAATATTACAAATCCATTTGGA

GGAAGTATTCCCGAAACGGCTAAACTGGTCCCCATTATAAAAAATGAACTTGTTATTAGT

AATACATGCTTTTCGAAGCCCCCTAATCTTCCTAATAATAAGCCTCCTGCTAGCATCCCA

AATGCAAAGGAAATTTCCGTAATAGAAATATGCACAGGCGTTCCATTAAAGTGTTCCATG

CTTATTAAAGGAAATAGTGCATTGATTGGCATATAAACAAAAGTATATAGTGTTCCTAAG

AGTAATAAGGCAAACAATCCTTTGTTTTGTCTCAGAACCACAACTCCTTCTTTCATCTCC

CTTATGAAATTTGGTTCTAAACTTTGCACTTGATTACCCAGCTTAGGTATACGTACAATT

GCTACCGTAATAGATGCAATCACAGCACCCAATACGTCGATGGCAATAATAGCATTTAAA

TCCCAAACGGAGTATAAGAGTGCTGCAACTGCCGGACTAACAATATAGCTTATAGACTGC

AAAGACTGACTATAGCCTGCGCATTTCGTTAGCTGTTCTTCTGGTACTAAAAGTGGTGTA

ACCGCATTGAGTGCTGGGGTATGAAAAGCTGTTCCAATGCTACGGATAAACAATACTATC

ATAATCATCCAGACAGGTAGCTCCATACAGAATGCAACAATAGCAAGCACTGCACCAGCT

GCTGCGATAATTAAATCGGCACCAATCATTATCTTCTTCCTATCATGACGATCCACTAGC

ACACCAATGGCAGGTCCCAAAATCGCATAGGGTAAAAAACCTACTAATGAAGCCATAGAC

AAGACCATCGCAGATCCTGTTTTTTCTGTAAGGTAAAAATAATCGCCATTTGCAGGATGG

CACTAGTGATTAATGATACTGCTTGCCCTGCCCATATTGCATAAAATTTTCGTTTCCAAT

TGTTGTATTTTTCCATTTATATTATCTCCTGCATATTATTTTGCTTGAATTTCTATTTTG

AATAGCATTCTAGGCAATAAAAAATGCAGGCCAAACCCCACAATGTGGCTTTTGGTCTGC

ATACATACAATTTGGAAACATTCATATTAAAGACATAGTTAAATAAAGGTATAGTTAAAT

AACCAATATCCTCACCGTAACTAATGAATGCTCAATATCGTATAAATAAGCACAACAAAA

AAGCCTATCATCGGGTATAGATTCTGCTTTTTTTATTGCCAGCTTATCTTAAACGCATTG

AGGCTGTCATAGTTTCGGTTCCTCCTACATCTTTGTTTATATCAATTTATAGTATAACAC

AACAAGATGATATGTTCAATATAAAAGTTATGGAATGAGACTCATACTTCCAATTCGATG

CCAGATTTAAAGGATATGACGAAGTTTTCTTCATAGACTGTAACGCTCTGGATTATCTTC

CTTAGTAGCAAGCGATTAGCTTTCACAAAATCTTCTGTTTGTAGTTTTAAAAATTCATCA

GGATTTTCTAACTCAACCTCAAAATATTTCATTTTACATTCCCTCATTTCATTTATTGAT

AAATTGAGTTTGCAAAAAAGAGTGGACAATTTTTGTCTACTCTTAACCTTTAAAATAGTT

TTTTTAATCGATTTGAAGTTGCCTAAATTATTACTTATTCGGTAAAATGAAGTATTGCTT

TCAACAGATTTCCTTCAACTACACTTCACTTGATTCAAACAAGGTGGGTACATTTCTATT

CCCACAAACTCCTTGTCAATGGAAACAAACACGTACCCACAGGGTAAATGGAAATAGAAA

CTGATAATTTCTAGCTATCACTTCTACTCATTCCAAAAATTTTCTCACTCTGATACTTAC

CCACCATAAAGCAAAAAGCCTTGCAATCAAGGCTTTCATTATCCCTTTCGTTCAAAGGTT

TCTAAGCTTTTACGAGCAGAGCGACACACTCAGCGGTTCGCTATCTCCGTTCTGTCTGCG

TGCTAGCACTTGTCAATCACGGACAGCTATCGCATGGGCGGAAGTAAATGCTAATCTTCG

TCGTTTTACTCCTTGACTAGCAAACTTACCGCCTCAACATGTCCTGTATGTGGAAATAAA

ACACGATTAAAGATAAGGGAAGATACTGAATTAAAAAAATTCCCCCTCTATTGTCCGAAA

TGCAGACAAGAAAATTTAATTGAAATAAAGCAGTTCAAAGTAACTGTGATTACAGAGCCA

GACGCAAAGACGCAGAGCCGATAAAATGAGATTAATACAATCTCATTTTATCGGCTCTTT

CCGTTATGTATGGATTCTTTTAATTAGTCTTCGATGTTTCTTGCTTCGTTGATACCGCTG

GCTAAAGATTCCATTAAGGATAGTTCTTTGTCTGTAAAGCTATCCATGTATTTCTCTATC

TGTAATCGTCGGGTGCTTTTTACCAAGTTATTAGCAGGTAAGAAAAATTCATCAACGGAA

ACATGAAGTAACGATACAAGGTCATAAAGAACTTGTATGCTGGGGTGTTGCCCTTTATTT

TCAATATTAGTTAAGTACCGTGGGTCAATTTCAATCAATGCTCCCACTTGTTCACGAGTT

AAACCTCGTTTCAATCGAGCTTCTTTAATGGCTAAACCAAAGGCTCTAAAATCATATTTA

TCTTCTTTTTTACGCATAGTAGACCACCTCTATACATTTTATTGTTCCTACTGAATTAAA

AACAGGTATAGAAAAACGTGTTATATGGTTTATAGGTTTATATTTAATAAAAAGCACTAC

TAAACGCCAATAAAAAAACCGTTATATGGTAGTGCTATTTACGCTGTTAAAATATTGTAT

ATTACTTCCAAATGGCGGTTTGTTGGAGGTCAACGTCGCCATGAAGTACATCATATACAA

TAAATTTCCTTACATTGGGTTCTTGTCAAAAAAAGTCGTCTATCTGCAATAGATAAGTAC

GTCCACCAATGTGGTTTTATAAATCATATAGATAGAATAACAGAAGCATGTAAACAGAGA

AATAAATCTGTTTATATGCTTTTTTGGCTATTCAGAACTTTTTTACAAAGTTTATTTATC

AGTAATGCAACAAATCCCCCTTTCACATTGGGACTAAGAGTGAAAGGAGATAAACGAGCA

AGGCTCACTTCCTTTCCTAGACAGAAAGGGGGTGAGAAACATGAAACCATCTTCTTTTCA

GACCACAATAGAAAATCAGTTTGACTATATCTGTAAACGTGCTATGGAAGACGAGCGAAA

GAATTATATGCTTTATCTTTCAAGGATTGCAAAGCGTGAGGTGTCCTTTTCGGATGTTGG

CGATTATCTTGTTAGCCAGTTTGCGACAACAGATAACTATTCAACTGACTTTCAGATTTT

TACACTCAATGGGTTATCAGTAGGCGTTGAAAATGATTTGTTGAGTGAAGCATTACGTGA

GTTGCCAGACAAGAAACGTGAAATTCTACTGCTGTTTTACTTTATGGACATGAGCGATTC

AGAAATTGCAGACCTGTTGAAATTGAACCGTTCTACTGTCTATCGGCATAGAACCAGTGG

ACTAGCCTTAATTAAAAAGTTTATGGAGGAATTTGAAGAATGAAAACACAATATCCTATG

ATTCCCTTTCCTCTCATTGTAAAGGCAACAGATGGCGATACCGAAGCGATTAACCAGATT

CTACATCATTACAGAGGGTACATAACGAAGCGTTCCCTACGACTTATGAAAGATGAATAT

GGCAATCAAAGTATGGTCGTTGATGAAGTCTTACGTGGAAGAATGGAAACCAGACTGATT

ACAAAGATTTTGTCATTTGAAATTAAGTAATATCCTCTCTCCTTTCGTGGAAGCGTGCTA

AACCATTCCACGCTTCCCGAACAGGGAGGTTTGTTATTCCACCAAAGCATATTGAGCTTT

CAATGTGTTTTGATAGGCTAACGAGCCATTGTTCTTTGAAAACTGAATAAAAGTAATCGA

ATACGTTTCGATAAGAAAAGAGCCAACGGAACTAACCGCCATGACCTATCTTATAAAGAT

AGCGAGCGATTCATGTTAGTGATCCGAGAAGCAATCTTTAGCAGGATTGCCTGCAACGAC

ATTCTTATCGTGATAATGATACTCCCATACAGTCAATAGTCCGAGCGTGATAAAACCGTC

GCAGGCAATGAGTATGGCTACATGAGAACCATGCAGGGGTGGAACTCCCGTGAGCTTTGC

TAAAGCTGTTCGATTGCTGGTAAAACAACTTTTATGAAATCCAAATAAGTGATTTGGAAA

GGAGGATTTTATGAAGCAGACTGACATTCCTATTTGGGAACGTTATACCCTAACCATTGA

AGAAGCGTCAAAATATTTTCGTATTGGCGAAAACAAGCTACGACGCTTGGCAGAGGAAAA

TAAAAATGCAAATTGGCTGATTATGAATGGCAATCGTATTCAGATTAAACGAAAACAATT

TGAAAAAATTATAGATACATTGGACGCAATCTAGCGTCGCCAAAGGGTCTTGTATATGAT

AAAATAGTATTAAGTCGTATCAAGGCTCTTTCCATAAAGGAAAGGAGCAAATGCCATGTC

AGAAAAAAGACGTGACAATAAAGGTCGAATCTTAAAGACTGGAGAGAGCCAACGAAAAGA

CGGAAGATACTTATACAAATATATAGATTCATTTGGAGAACCGCAATTTGTTTACTCGTG

GAAACTTGTGGCTACAGACCGAGTACCAGCAGGAAAGCGTGATTGTATCTCACTTAGAGA

GAAAATCGCAGAGTTACAGAAAGACATTCATGATGGTATTGATGTTGTAGGAAAGAAAAT

GACACTCTGCCAGCTTTACGCAAAACAGAACGCTCAAAGACCAAAGGTTAGAAAAAACAC

TGAAACTGGACGCAAATATCTTATGGATATTTTGAAGAAAGACAAGTTAGGTGTAAGAAG

TATTGACAGTATTAAGCCATCAGACGCTAAAGAATGGGCTATTAGAATGAGTGAAAATGG

TTATGCTTATCAAACCATCAATAACTACAAACGTTCTTTAAAGGCTTCATTCTATATTGC

TATACAAGATGATTGTGTTCGGAAGAATCCATTTGACTTTCAACTGAAAGCAGTTCTTGA

TGATGATACTGTCCCTAAGACCGTACTAACAGAAGAACAGGAAGAAAAACTGTTAGCCTT

TGCAAAAGCTGATAAAACCTACAGCAAAAATTATGATGAAATTCTGATACTCTTAAAAAC

AGGTCTTCGTATTTCAGAGTTTGGTGGTTTGACACTTCCAGATTTAGATTTTGAGAATCG

TCTTGTCAATATAGACCATCAGCTATTGAGAGATACTGAAATTGGGTACTACATTGAAAC

ACCAAAGACCAAAAGTGGCGAACGTCAAGTTCCTATGGTTGAAGAAGCCTATCAAGCATT

TAAGCGAGTGTTAGCGAATCGAAAGAATGATAAGCGTGTTGAGATTGATGGATATAGTGA

TTTCCTCTTTCTTAATAGAAAGAACTATCCAAAAGTGGCAAGTGATTACAACGGCATGAT

GAAAGGTCTTGTTAAGAAATACAATAAGTATAACGAGGATAAATTGCCACACATCACTCC

ACATAGTTTGCGACATACATTCTGTACCAACTATGCAAATGCAGGAATGAATCCAAAGGC

ATTACAGTACATTATGGGACATGCTAATATAGCCATGACGCTGAACTATTACGCACATGC

AACATTCGATTCTGCAATGGCAGAAATGAAACGCTTGAATAAAGAGAAGCAACAGGAGCG

TCTTGTTGCTTAGTAGTACAAATGAATTTACTACTTATTTACCACTTCTGACAGCTAAGA

CATGAGGAAATATGCAAAGAAACGTGAAGTATCTTCCTACAGTAAAAATACTCGAAAGCA

CATAGAATAAGGCTTTACGAGCATTTAAGAAAATATAAAAAGATAATTAGAAATTTATAC

TTTGTTT

>GA19101/Tn6002

AAAATAGCATAAAAATCTAGTTATCCGCATAAAAACTGGACTTATCACACTTTATCAAGG

TCGAAACCAATCAATTTACTACTAATTTACTACTTATGAATGAGCTTTGATACGACGATT

TACCCTTGAAAAGTGAAGAAACAAAGATACTTCCAATAAAAATTGAATAGGCAAAAGGTG

GACACTTCAAAAATGAGGTGTCTATTTTTTTACCCGATTTGAAAGGACGTGATACTACGA

AAATAGAAAAACAAGAGAATAAAGGTCGCTCCCCACCGTGAAAGACCATCAAACAAACGA

GATTCATTCAACCATAAAAAAGAAAGGATAGGTAAAAATATGGAACTTAAATTTGTGATT

CCCAAGATGGAAAAAACATTCGGCAATTTAGAATTTGCTGGCGAGGATAAAGTCGTACAG

CGAAGAATCAACGGACATCTAACCGTCTTATCTCGAAGCTATAATCTCTATTCAGACGTT

CAAAGAGCAGATGATATTGTGGTCGTACTTCCTGCTGAAGCTGGCGAAAAACATTTCGGC

TTTGAGGAACGTGTTAAGTTAGTCAATCCACGTATTACCGCCGAGGGCTATAAAATCGGC

ACTCGTGGTTTTACAAATTACCTTTTACATGCTGACGACATGGTAAAAGAATAAAGAAAG

AGAGGAAAAATGATGAGATTAGCAAATGGTATCGTATTAGATAAAGACACGACTTTTGGA

GAATTAAAATTCTCTGCTCTACGTCGTGAAGTGAGAATCCAAAATGAAGACGGTACGGTT

TCAGAGGAAATCAAAGAACGTACCTATGACTTAAAATCCAAAGGGCAAGGACGCATGATT

CAAGTAAGTATTCCTGCCAGCGTGCCTTTGAAAGAGTTTGAATATAACGCACGGGTGGAA

CTTATCAATCCCATTGCGGATACTGTCGCTACTGCTACCTTTCAAGGAGCAGATGTTGAC

TGGTACATCAAGGCAGACGATATTGTGCTGACAAAGGATTCTAATTCCTTTAGAAATCAA

CAGCCACCTAAGAAAGAACCTGCTACGGACAAATAGCCACGTATCTTCCATTAGAGAGAA

AGGAGAAAATCAAACATGAAACAGCGTGTCTTTCGTGGTAAAAGGATTCGTCCGAGTGAC

AAAGATTTAGTCTTTCATTTTACAGTAGCGTCCTTACTGCCTATTTTACTGCTTGTTGTC

GGACTGTTTCATGTGAAGACAATCCAGCAGGTCAACTGGCAGGACTTTAACCTATCACAA

GCAGATAAGATTGACATTCCGTATTTAAGTATCAGTTTCAGTGTCGCAATTCTTGTCTGC

TTGCTGGTGGCGTTTCTATTCAAACGGTATCGCTATGATACGATTAAACAACTCTACCAC

CGTCAAAAGCTGGCGAAGATGGTTCTTGAAAATAAGTGGTATGAATCAGAACAGGTCAAA

ACAGATGGCTTCTTCAAGGATTCCCCCAGTCGTACCAAAGAAAAGATAACCTACTTCCCT

AAAATCTATTATCGCCTTAAAAATGGCTTAATACAGATACAAGTGGAAATCACTCTGGGG

AAATATCAAGACCAGCTCCTACACTTGGAAAAGAAATTAGAAAGTGGCTTGTACTGTGAG

CTGACGGATAAAGAGTTAAAGGATTCCTACGTGGAATATACCTTGCTCTATGATATGATA

GCCCGTCGTATTTCTATTGATGAAGTACAAGCTCATGATGGGAAACTTTGCTTAATGAAA

AATATGTGGTGGGAATATGACAATCTGCCTCACATGCTCATAGCTGGTGGTACAGGTGGC

GGTAAGACCTACTTTATCCTGACACTGATTGAAGCCTTGCTTCATACAGATTCTAAGCTG

TATATCTTAGACCCGAAAAATGCAGACTTAGCCGATTTAGGCTCTGTGATGGCAAATGTC

CACTACAGAAAAGAAGACTTGCTTTCCTGCATTGATACATTCTATGAAGAAATGATAAAA

CGCAGTGAGGAAATGAAGCAGATGGAAAATTATAAGACTGGCGAAAATTATGCTTACTTA

GGACTTCCAGCACACTTCTTAATCTTTGATGAATATGTCGCTTTCATGGAAATGCTGGGA

ACAAAAGAAAACACCGCAGTTATAAATAAGTTGAAACAGATTGTCATGTTAGGTCGTCAA

GCTGGCTTCTTTCTAATACTGGCTTGTCAGCGTCCAGACGCAAAATATCTAGGCGACGGA

ATCCGTGACCAGTTTAATTTCAGAGTGGCTTTAGGTCGTATGTCTGAAATGGGCTATGGC

ATGATGTTTGGCAGTGACGTACAAAAGGATTTCTTCTTAAAGCGAATCAAAGGTCGTGGC

TATGTTGATGTAGGAACAAGTGTCATATCAGAGTTTTATACTCCCCTTGTACCAAAAGGA

TATGATTTCTTGGAGGAAATTAAAAAGTTATCCAACAGCAGACAGTCCACGCAGGCGACG

TGCGAAGCGGAAGTCGCAGGTGTGGACTGATCTTGCTGGCTGGTGTGGCAATAGCCACGC

CAGCACTTAACCCCCCGTATCTAACAGGGGGGTACAAATCGACAGGAAACAGTCAAAAAA

ACATTAGAAAATCCTTTGGTTACAAGGGATTTACAAAATTTCAGCGTATGTAAAATGGGC

TTTAAAAGTTGACATACGCCTTTTTGATTGGAGGGATTTTTACTGAATGAACAAACTTGG

TTACAGCAGTTAAAAGAAAAACGCTTGGCTTATGGACTATCTCAAAATCGTTTAGCGGTT

GCGACTGGTATTACAAGGCAGTATCTAAGCGATATTGAAACAGGAAAAGTCAAGCCATCA

GAGGATTTACAGCAATCTCTTTTTGAAGCTCTGGAACGCTTCAATCCCGACGCTCCCCTT

GAAATGCTCTTTGATTATGTAAGGATTCGCTTTCCCACAACGGACGTACAGCATGTGGTC

GAAAACATCTTACAACTGAAACTGTCCTATTTTCTTCATGAGGACTATGGTTTCTATTCT

TATTCAGAGCATTATGCTTTAGGCGATATATTCGTTCTCTGCTCCCACGAACTGGACAAA

GGAGTTCTGGTGGAATTGAAAGGTCGTGGGTGTCGGCAATTTGAAAGCTATCTTCTGGCT

CAACAAAGAAGCTGGTATGAGTTCTTTATGGACGCTTTGGTGGCTGGCGGTGTGATGAAA

CGCCTTGACCTTGCCATTAACGATAAGACAGGGATTTTGAATATCCCTGTACTCACTGAA

AAATGCAGACAGGAAGAATGTATATCCGTCTTCCGCAGTTTCAAAAGCTATCGCAGTGGC

GAACTGGTACGCAAAGATGAAAAGGAATGTATGGGAAATACCCTCTATATCGGTTCATTA

CAGAGTGAAGTTTATTTCTGTATCTATGAAAAGGACTATGAACAGTACAAGAAAAATGAT

ATTCCCATTGAAGACGCAGAAGTAAAAAACCGTTTTGAGATTCGATTGAAAAATGAGCGT

GCCTATTATGCAGTCCGTGATTTACTCGTCTATGACAATCCAGAGCATACCGCCTTTAAA

ATTATCAATCGGTATATCCGTTTTGTAGATAAAGACGATTCCAAACCTCGTTCTGATTGG

AAACTGAATGAAGAATGGGCTTGGTTTATTGGGAACAATCGTGAACGATTAAAACTAACC

ACAAAACCAGAGCCTTACTCCTTCCAAAGGACGCTGAACTGGCTATCTCATCAAGTTGCC

CCGACCTTAAAGGTTGCGATTAAACTTGATGAAATCAACCAGACGCAGGTTGTAAAAGAC

ATTCTCGACCATGCGAAACTGACAGACCGACACAAGCAGATTTTGAAGCAACAGTCAGTA

AAAGAACAGGACGTGATAACAACAAAAAAAGGATATCTGTCAACCATACCAGTTGACAGA

TATCCAAAAAAAGATATAATGGGAGATAAGACGGTTCGTGTTCGTGCTGACTTGCACCAT

ATCATAAAAATCGAAACAGCAAAGAATGGCGGAAACGTAAAAGAAGTTATGGAAATAAGA

CTTAGAAGCAAACTTAAGAGTGTGTTGATAGTGCATTATCTTAAAATTTTGTATAATAGG

AATTGAAGTTAAATTAGATGCTAAAAATTTGTAATTAAGAAGGAGGGATTCGTCATGTTG

GTATTCCAAATGCGTAATGTAGATAAAACATCTACTGTTTTGAAATAGACTAAAAACAGT

GATTACGCAGATAAATAAATACGTTAGATTAATTCCTACCAGTGACTAATCTTATGACTT

TTTAAACAGATAACTAAAATTACAAACAAATCGTTTAACTTCTGTATTTGTTTATAGATG

TAATCACTTCAGGAGAGATTACATGAACAAAAATATAAAATATTCTCAAAACTTTTTAAC

GAGTGAAAAAGTACTCAACCAAATAATAAAACAATTGAATTTAAAAGAAACCGATACCGT

TTACGAAATTGGAACAGGTAAAGAGCATTTAACGACGAAACTGGCTAAAATAAGTAAACA

GGTAACGTCTATTGAATTAGACAGTCATCTATTCAACTTATCGTCAGAAAAATTAAAACT

GAATACTCGTGTCACTTTAATTCACCAAGATATTCTACAGTTTCAATTCCCTAACAAACA

GAGGTATAAAATTGTTGGGAATATTCCTTACCATTTAAGCACACAAATTATTAAAAAAGT

GGTTTTTGAAAGCCGTGCGTCTGACATCTATCTGATTGTTGAAGAAGGATTCTACAAGCG

TACCTTGGATATTCACCGAACACTAGGGTTGCTCTTGCACACTCAAGTCTCGATTCAGCA

ATTGCTTAAGCTGCCAGCGGAATGCTTTCATCCTAAACCAAAAGTAAACAGTGTCTTAAT

AAAACTTACCCGCCATACCACAGATGTTCCAGATAAATATTGGAAGCTATATACGTACTT

TGTTTCAAAATGGGTCAATCGAGAATATCGTCAACTGTTTACTAAAAATCAGTTTCATCA

AGCAATGAAACACGCCAAAGTAAACAATTTAAGTACCATTACTTATGAGCAAGTATTGTC

TATTTTTAATAGTTATCTATTATTTAACGGGAGGAAATAATTCTATGAGTCGCTTTTTTA

AATTTGGAAAGTTACACGTTACTAAAGGGAATGGAGATAAATTATTAGATATACTACTGA

CAGCTTCCAAGAAGCTAAAGAGGTCCCTAGCGCCTACGGGGAATTTGTATCGATAAGGGG

TACAAATTCCCACTAAGCGCTCGGGACCCCTTGTAGGAAAATGTCCTAAGTGGGATATCT

GTCAACTGGTATGGTTGACTAAAAATACTTCCTACGAAAATGTAGGGGGTATTTTTTTAC

GAAAAAATACAATCGATTCTTAAAAAGAAAAATTTTTGATTGGCAAAACCATAACAAGTT

CGTTTTAGGGTTTTGATTTTGCGATTGATGCCTTCTAAAGGACCATTAGAGTATTCAAAT

TTAGCGCTATTTAAGACATATTTTCTGTTTTGACGAAGGGTTTGAATAGCAGTATCCATT

TCTGTATTGGTTTTTTGGTAGTCTAAGATGGTTGACTCTAGTAATTCACTATTGCGCTCG

TTTAGGGCTTTCGTGATATCTTGGTAAGTTTGGTATACTTCAGCGAACTTGGAAAATTTA

CTAGTAATGAGATCAACAGCATTTTGGCGAGTCATATATTGTTTAACGCCGCGAAGAAAA

ACTACTTCTTCAGGGTGAAGATCTTCAGCTTTTTTATGGAATAGCTTCCAATGTGACTTC

ATAATTTTATATTCTTGGCTCTGTTTATCAAGTTGCTTTAGGATAGAGATACGACAATTG

TCCAAAGCGCGACCAGCTAATTGTACAAGGTGGAAGCGATCAATAATGATATTGGCATTA

GGGAAAAGGCGATAGATAAAACTTTGATATTGAGCATTTAAATCAATTACAACTGATTGA

ACGCATTCGCGTTCGGCTTTTGAATAACGACTTTCAAAATAATCAACAATGGTAGGTGAT

AGACGATCCTGTAACTTTGTGACAATTTGGTGGGTTTCAGCGTCACAACAGATAAAGGAC

ATCACAGACTTAATTGAACGAAACTCGTCAAAACATAGATGCTTAGGCAACTTAGCCACA

CGATAGTGTGGTTCCATGCGCTCTAAGATTGTTCGACGAACACTGCTAGGAGAGCAGTGA

CACATTTCAGCAATAAGCTGACCAGATAAGCCTTTACGAGCTAAAAGCATGATTTGATTT

TTGAGATCACTGGATAAGGTTTGATTTTCTTTGGTTAAATTAGTAATAGCACCAAAAGTA

GTATGGCATGATTTACATTTATAGCGTTGTTTACGAAGCTCTAGTTCATATCTTCTCCCA

TTTAAACTTGCCAGTCGTACATGAGTTTTGCGAAAGCCATCCTTATTAACTGTGGGAAAG

CCACAGTTACGACAACGATTAATCGGATAAGAAAGAGTAGCTGTTATTAGCGTTATATAC

TCTTTAACAGAATCGTTGTTGTGTTCAGCTTCTTCAACAGAAATAATTTTAATATTTTTA

TCTTTAATTCCAAGAATATTTAGGATAGAATCATTATGGGACATTTGTTTAACCTTCTTT

CATGATTTTTGTGGTGAATTGATTGTATAACGAGGGGACAGCAAATGTCCTCTTTTTTGT

ATAAAAAAATCTGGCATGGAATCTCTATCCATACCAGAAAGTGTATACCCCAAAAAAATA

ACTCAAATACAAATTCATTGAATATAGAGAGGAGAACATTTTTATGAATTTTGGACAAAA

CCTTTATAACTGGTTTCTATCAAACGCTCAATCACTGGTGCTTTTAGCAATCGTTGTGAT

TGGCTTGTATCTTGGCTTCAAGCGTGAGTTCAGTAAACTCATTGGCTTTTTAATTATTGC

GATTATTGCGGTTGGCTTAGTCTTCAACGCTGCTGGAGTAAAAGACATTTTACTAGAGCT

ATTCAATCGCATTATTGGTGCTTAAATAAAACCGTTCTTTTGTGGAATATAAGTGGTTTT

CTTATGTTCCGCAAAGGAATGGTACACCAAACGAAGTGCGGTAGGGATTTTTGAATCTCT

ACAAAGAAAGGACGTGAATATATGGACGATATGCAAGTCTATATTGCGAATTTAGGCAAA

TACAATGAGGGCGAATTGGTCGGTGCGTGGTTTACCTTTCCCATTGACTTTGAGGAAGTC

AAAGAGAAAATCGGCTTGAATGATGAATATGAGGAATACGCCATTCATGACTACGAGTTA

CCCTTTACGGTTGACGAATACACTTCCATTGGCGAACTCAATCGACTATGGGAAATGGTA

TCGGAATTACCCGAAGAATTACAATCGGAGCTATCTGCTCTGCTCACTCATTTTTCAAGC

ATTGAAGAACTAAGCGAACATCAAGAGGATATTATCATTCATTCCGATTGTGATGATATG

TATGACGTGGCACGCTACTACATTGAAGAAACGGGTGCTTTAGGCGAAGTACCAGCTAGT

CTTCAAAACTATATTGATTATCAAGCCTATGGTCGGGATTTAGACCTTTCAGGAACGTTT

ATCTCAACCAATCATGGAATTTTTGAAATCGTCTATTAAATCTGTCGGTACATTACTACT

GGCAGATTTTCTATTTTACGGGGTGGCTCAATCAGCTACCCCTATTTTTTATGAAAGGAT

TGATTACATGAAGAAAATACGAAGCTATACCAGTATCTGGTCTGTGGAGAAAGTGTTGTA

CTCCATCAATGACTTTAGACTTCCGTTTCCCATAACCTTTACGCAAATGACATGGTTTGT

CGTGTCACTCTTTGCAGTTATGATACTTGGCAACTTGCCCCCTCTTTCTATGATAGAGGG

AGCATTTCTCAAATACTTTGGGATTCCTGTGGCTTTCACATGGTTTATGTCTACAAAAAC

CTTTGATGGTAAAAAGCCTTATGGATTTTTGAAGTCTGTCATTGCTTATGCACTGCGACG

AAAACTGACCTATGCAGGAAAAAAAGTAACGCTTGGCAGAAACCAGCCACAAGAAGCCAT

TACAGCAGTTAGGAGTGAATTTTATGGCATATCCAATTAAATATATTGAAAACAATCTGG

TCTGGAATAAAGATGGGGAATGTTACGCTTACTATGAGCTTGTTCCCTACAATTACTCAT

TTCTAAGTCCGGAACAGAAAATACAAGTACATGATTCTTTCAGACAGCTTATCGCACAAA

ATCGTGATGGCAAGATTCATGCTTTACAAATCAGTACAGAATCCAGCATACTTTCTGCAC

AAGAACGTTCCAAAAATGAAGTCACTGGAAAGCTCAAAACGGTTGCCTATGACAAAATCG

ACCAACAGACAGACGCTTTAATATCCATGATTGGCGAAAATCAAGTGGACTACCGTTTCT

TTATCGGTTTTAAGTTGCTTCTCAACGATCAGGAGTTTTCTATGAAAAGTCTTACCGTTG

AAGCAAAAAATGCTTTTACTGATTTTGTCTATGATGTGAACCATAAGCTGATGGGCGATT

TTGTTAGTATGAGTAATGATGAAATCCTGCGTTTTCAGAAGATGGAAAAGCTTTTAGAAA

ATAAAATCTCCCGTCGTTTCAAAATCCGAAGATTAGATAAGGACGACTTCGGCTATCTGA

TTGAACACCTTTACGGACAGACAGGCACTGCCTATGAAGAGTATGAGTATCCTCTATCAA

AGAAAAAGCTGGAACATGAAACACTGATTAAAACCTATGACCTCATTAAGCCTACTCGCT

GTCTGGTGGAAGAAAAACAGCGATATTTGAAAATCCAGCAGGAAGACGAAACCGCCTATG

TAGCTTACTTTACCATCAACAGTATTGTCGGCGAACTGGACTTCCCGTCCTCTGAAATCT

TCTACTACCAGCAACAGCAATTTACCTTTCCGATTGATACGTCAATGAATGTGGAAATTG

TAGCGAATCGTAAAGCCCTATCTACTGTCCGCAATAAAAAGAAAGAACTGAAAGACTTGG

ATAACCACGCATGGCAAAGTGATAATGAAACCAGCTCTAATGTGGCGGAAGCTCTGGAAA

GTGTGAACGAGCTGGAAACCAATTTAGACCAAAGCAAGGAATCTATGTATAAGCTGTCCT

ATGTTGTAAGGGTATCAGCAAATGACCTTGACGAACTTAAACGTCGTTGTAATGAAGTGA

AAGATTTCTATGATGATTTGAGCGTGAAACTGGTACGACCTTTTGGGGATATGCTGGGCT

TACATGAAGAATTTTTACCTGCCAGCAAAAGATATATGAATGACTATATTCAATACGTGA

CCTCTGATTTCCTCGCTGGTTTAGGTTTTGGTGCTACTCAAATGCTGGGTGAAAATGAGG

GGATTTATGTTGGCTACAGCTTAGATACTGGACGCAATGTCTATCTGAAACCTGCTCTTG

CCAGTCAAGGGGTTAAGGGTTCAGTAACCAATGCGTTAGCGTCTGCCTTTGTCGGTTCGC

TGGGTGGTGGTAAATCCTTTGCGAATAACCTTATCGTCTATTATGCAGTGCTTTATGGGG

CACAAGCAGTGATTGTAGACCCAAAAGCAGAACGTGGCAGATGGAAAGAAACCTTGCCAG

AGATTTCCCATGAAATCAATATCGTCAATCTGACTTCTGATGAGAAAAACAAAGGCTTAC

TTGACCCTTATGTAATTATGAAAAATACCAAAGATTCTGAATCACTGGCTATTGATATTT

TGACATTCCTTACGGGGATTTCCTCTCGTGATGGGGAACGCTTCCCAATCCTTAGAAAAG

CCATTCGTGCAGTAACCAATAGTGAAGTGCGAGGGTTGATGAAAGTGATTGAAGAATTAC

GGGTTGAGAATACGCCACTAAGTACCAGCATAGCCGACCATATCGAAAGTTTTACAGACT

ATGACTTTGCCCATCTGCTTTTTAGTGATGGTTATGTAGAGCAGTCTATCAGCCTTGAAA

AACAACTGAACATTATACAGGTTGCCGACTTGGTACTTCCTGACAAGGAAACTTCCTTTG

AGGAATATACCACAATGGAGTTACTATCGGTAGCAATGCTGATTGTCATTAGTACCTTTG

CTTTAGACTTTATCCATACAGACCGAAGCATTTTCAAGATTGTAGATTTAGACGAAGCAT

GGAGCTTTTTACAGGTAGCACAAGGAAAAACACTATCTATGAAGCTGGTTCGGGCTGGTC

GTGCTATGAACGCTGGGGTATATTTCGTGACCCAAAATACAGACGACCTCTTAGATGAAA

AACTGAAAAATAACCTCGGCTTAAAATTTGCATTTCGTTCCACTGACCTTAACGAGATTA

AAAAGACCTTAGCCTTTTTTGGTGTAGACCCAGAGGACGAAAACAATCAGAAGCGATTGC

GTGATTTGGAAAACGGGCAATGCCTTATCAGTGATTTATATGGTCGTGTCGGTGTGATAC

AGTTCCACCCTGTATTTGAAGAACTGCTCCATGCCTTTGATACCAGACCACCTGTGCGAA

AAGAGGTGTAAATGTGAAACCATCAATAGTAAACAGAATAAAATCAAACTGGACGCTGAA

ACGTCTAGGTAAAGTGGCAATGACAGTGGCTTTCACACTTGTGATTGCCATTTTTCTTTT

AGCCATGCTGGGAACGGTGGTTCAAGCTGCGGGCTTGGTAGATGATACGGTCAATGTGGC

AAATGAATACAGCCGATACCCACTTGAAAACTATCAACTGGATTTTTATGTGGATAATAG

CTGGGGCTGGCTTCCGTGGAACTGGTCGGACGGGATTGGAAAACAGGTCATGTATGGACT

ATATGCCATTACCAATTTTATTTGGACAATCAGTTTGTATGTTTCCAATGCGACAGGTTA

CTTAGTACAGGAAGCCTATTCCTTAGACTTCATTTCCGCTACAGCAGATTCCATTGGTAA

GAATATGCAGACCTTAGCTGGTGTGAGTGCAAACGGATTTTCAACAGAGGGTTTCTATGT

TGGATTCCTCTTACTCTTGATTTTGGTTCTTGGGGTTTATGTTGCCTATACGGGACTGAT

AAAGAGAGAAACCACAAAGGCAATTCATGCCATTATGAATTTTGTGCTGGTGTTTATCCT

ATCGGCTTCCTTTATTGCCTACGCTCCCGACTACATTAAAAAAATCAATGACTTTTCATC

AGACATCAGTAATGCCAGTTTATCACTTGGCACGAAGATTGTCATGCCCCATTCCGATAG

TCAAGGCAAGGACAGCGTGGACTTAATCAGAGATAGCCTGTTTTCCATACAGGTTCAGCA

ACCGTGGCTACTGCTTCAATATAACAGTTCAGACATTGAAAGTATTGGTATTGACCGTGT

GGAAAGCCTGCTCTCAACCAGTCCAGATTCCAACAATGGGGAAGACAGAGAAAAAATTGT

TGCGGAAGAAATTGAAGATAGAAGCAATACCAATATGACGATTACAAAGACGATTAACCG

TTTAGGTACAGTCTTCTTCCTATTTGTCTTCAATATTGGGATTTCCATATTTGTATTCCT

ATTAACAGGAATCATGATTTTCTCGCAGGTACTTTTTATCATCTATGCTATGTTTCTGCC

TGTGAGCTTTATTTTAAGCATGATTCCATCATTTGATGGTATGTCAAAACGAGCCATAAC

AAAGCTCTTTAATACCATTTTGACACGAGCTGGAATCACATTGATTATTACGACAGCATT

TAGTATTTCAACCATGCTCTATACCTTATCGGCTGGTTATCCGTTCTTTTTGATTGCTTT

TCTACAGATTGTGACCTTTGCAGGAATCTACTTTAAGCTGGGCGATTTAATGAGTATGTT

TTCTCTACAGAGTAACGATTCTCAAAGTGTGGGAAGTCGTATGATGAGAAAACCTCGTAT

GCTTATGCACGCTCACATGCACCGTCTACAGCGGAAACTTGGACGCTCCGTGACTGCTTT

AGGGGCTGGGTCTGCTATTGCCAGTGCTACGGGTAAAAAAGGACAGTCGGGTTCGGGAAG

TTCTGCGAGTACACAAGCAGATCACTCTCGACCAAACGGGCAGGAAAAATCAACACTAGG

AAAGCGTATCGGTCAAACCATCGGTACAGTAGCTGATACCAAAGACAGAATGGTAGATAC

TGCTGGTAATTTGAAAGAACAGGTTAAGGATTTGCCGACCAATGCAAGATATGCAGTATA

TCAAGGAAAATCTAAAGTGACGGATAATGTCCATGATTTAACCAGAAGTATTTCTCAAAC

CAAAGCGGATAAAGCCAGTGGACGCAAGAAACAGCAGGAACAAAGACGGAAAACCATTGC

GGAGCGTCGCTCTGAAATGGAACAGGTCAAACAGAAAAAACAGCCAGCTTCTTCCGTTCA

TGAAAGACCAGCCACTAAGCAAGAACAATCTCATGATGGACAGACTTCAAGGCAAACAAC

TGTACAGGCTTCCTATAGGGAATCTCAACAAGCCAAACAAGAGCGTCCAACAGTTAAGTC

CGATTCTTCAAGTTTAAAAACGGAACGTCAAAGTAATACAGTTCAAGAAAGAACGGTTCA

AAAGCCAGTAACTTCAACTGCACCAACAGATAGAGCTTCACAACGTTCAATCACAAAAGA

ACGTCAGTCTACTGTTCAAAGAGTACCACTACAAAATACAAAAAGTAGACCACCAATCAA

AACCGCCACCATTAAGAAAGGCAGTAAGAAACCATGAAGCTGAAAACTTTAGTGATTGGT

GGTTCGGGATTATTCTTGATGGTCTTCTCACTGCTTCTGTTTGTCGCCATTTTATTTTCA

GATGAACAAGATGGCGGTTTTTCCAACATTCACTATGGCGGTGTGGATGTTTCCGCAGAA

GTGCTGGCTCATAAGCCTATGGTAGAAAAAGTTGCCAAAGAATATGGAATTGAAGAATAC

GTCAATATACTTCTTGCGATTATACAGGTGGAATCGGGTGGTACAGCAGAAGATGTTATG

CAGTCCTCGGAATCCCTCGGTCTTCCACCTAATTCATTGAGTACAGAAGAATCCATTAAG

CAAGGTGTGAAGTATTTCAGTGAATTATTAGCCAGTAGCGAAAGGCTCAGTGTAGATTTA

GAATCGGTTATCCAGTCCTACAATTATGGTGGTGGTTTCTTAGGGTATGTGGCTAATCGT

GGAAATAAATATACCTTTGAACTGGCTCAAAGTTTCTCAAAAGAGTATTCAGGTGGCGAA

AAAGTGTCTTACCCCAATCCCATAGCCATACCTATCAATGGGGGCTGGCGATACAACTAT

GGCAATATGTTTTATGTGCAACTGGTAACGCAGTATCTTGTCACAACAGAGTTTGATGAT

GATACGGTACAAGCCATCATGGACGAAGCACTGAAATATGAGGGCTGGCGATACGTTTAC

GGTGGAGCTTCCCCGACTACTTCTTTTGATTGTAGCGGACTGACACAATGGACGTATGGA

AAAGCTGGAATTAACTTACCACGAACCGCACAACAGCAATATGATGTGACCCAGCATATC

CCACTATCGGAAGCACAAGCTGGCGATTTGGTTTTCTTTCATTCTACCTATAACGCTGGC

TCTTATATTACTCATGTTGGGATATACCTTGGCAATAACCGTATGTTTCATGCAGGCGAC

CCAATCGGTTATGCCGACTTAACAAGCCCCTACTGGCAACAGCATTTAGTGGGAGCAGGA

CGAATCAAACAATGAGAAAGGAAGATTTAATGATGAAATTTAGAAAAAATCAGAATAAAG

AAAAACAGATACCAAAGGAAAAGAAACCTCGTGTCTACTATAAGGTCAATCCTCATAAAA

AGGTTGTGATTGCCTTGTGGGTACTTTTAGGGCTTAGTTTCAGCTTTGCGATATTCAAGC

ACTTTACAGCTATAGATACTCATACTATTCACGAAACAACTATCATAGAAAAGGAATACG

TTGATACTCATCATGTAGAAAATTTTGTAGAGAACTTTGCGAAAGTCTACTATTCATGGG

AGCAATCCGATAAGTCCATTGATAATCGAATGGAAAGTCTAAAAGGCTATCTGACAGATG

AACTTCAAGCTCTCAATGTTGATACAGTACGCAAAGATATTCCTGTATCGTCTTCTGTAA

GAGGATTTCAGATATGGACGGTAGAGCCAACTGGCGACAATGAGTTTAATGTAACCTACA

GTGTAGACCAGCTCATTACAGAGGGAGAAAATACAAAGACCGTCCACTCTGCTTATATAG

TGAGTGTCTATGTAGATGGTTCTGGAAATATGGTACTGGTTAAGAATCCGACCATTACCA

ACATACCTAAGAAATCAAGTTATAAACCAAAAGCCATTGAAAGTGAGGGGACGGTTGATT

CCATTACAACCAATGAAATCAATGAGTTTTTAACGACGTTCTTCAAGCTCTATCCTACAG

CGACAGCCAGTGAACTTTCCTACTATGTGAATGACGGGATATTAAAACCAATCGGAAAAG

AGTACATCTTTCAAGAACTGGTAAATCCTATTCACAATCGTAAGGATAATCAAGTCACGG

TATCGCTGACAGTGGAGTATATCGACCAGCAGACCAAAGCAACGCAGGTATCTCAATTTG

ATTTGGTACTTGAAAAGAACGGGAGTAATTGGAAGATTATAGAATAACAAATATTGGTAC

ATTATTACAGCTATTTTGTAATCACGTACTCTCTTTGATAAAAAATTGGAGATTCCTTTA

CAAATATGCTCTTACGTGCTATAATTTAAGTATCTATTTAAAAGGAGTTAATAAATATGC

GGCAAGGTATTCTTAAATAAACTGTCAATTTGATAGTGGGAACAAATAATTGGATGTCCT

TTTTTAGGAGGGCTTAGTTTTTTGTACCCAGTTTAAGAATACCTTTATCATGTGATTCTA

AAGTATCCAGAGAATATCTGTATGCTTTGTATACCTATGGTTATGCATAAAAATCCCAGT

NNNNNCACACACTTAATTAATTAAGTGTGTGNNNNNGGATTTTTATGCCCTTTTGGGTTT

TTGAATGGAGGAAAATCACATGAAAATTATTAATATTGGAGTTTTAGCTCATGTTGATGC

AGGAAAAACTACCTTAACAGAAAGCTTATTATATAACAGTGGAGCGATTACAGAATTAGG

AAGCGTGGACAAAGGTACAACGAGGACGGATAATACGCTTTTAGAACGTCAGAGAGGAAT

TACAATTCAGACAGGAATAACCTCTTTTCAGTGGGAAAATACGAAGGTGAACATCATAGA

CACGCCAGGACATATGGATTTCTTAGCAGAAGTATATCGTTCATTATCAGTTTTAGATGG

GGCAATTCTACTGATTTCTGCAAAAGATGGCGTACAAGCACAAACTCGTATATTGTTTCA

TGCACTTAGGAAAATAGGTATTCCCACAATCTTTTTTATCAATAAGATTGACCAAAATGG

AATTGATTTATCAACGGTTTATCAGGATATTAAAGAGAAACTTTCTGCGGAAATTGTAAT

CAAACAGAAGGTAGAACTGCATCCTAATATGCGTGTAATGAACTTTACCGAATCTGAACA

ATGGGATATGGTAATAGAAGGAAATGATTACCTTTTGGAGAAATATACGTCTGGGAAATT

ATTGGAAGCATTAGAACTCGAACAAGAGGAAAGCATAAGATTTCATAATTGTTCCCTGTT

CCCTGTTTATCACGGAAGTGCAAAAAACAATATAGGGATTGATAACCTTATAGAAGTGAT

TACGAATAAATTTTATTCATCAACACATCGAGGTCAGTCTGAACTTTGCGGAAAAGTTTT

CAAAATTGAGTATTCGGAAAAAAGACAGCGTCTTGCATATATACGTCTTTATAGTGGCGT

ACTGCATTTGCGAGATTCGGTTAGAATATCGGAAAAGGAAAAAATAAAAATTACAGAAAT

GTATACTTCAATAAATGGTGAATTATGTAAAATCGATAAGGCTTATTCCGGGGAAATTGT

TATTTTGCAGAATGAGTTTTTGAAGTTAAATAGTGTTCTTGGAGATACAAAGCTATTGCC

ACAGAGAGAGAGAATTGAAAATCCCCTCCCTCTGCTGCAAACGACTGTTGAACCGAGCAA

ACCTCAACAAAGGGAAATGTTACTTGATGCACTTTTAGAAATCTCCGACAGTGACCCGCT

TCTGCGATATTATGTGGATTCTGCGACACATGAAATCATACTTTCTTTCTTAGGGAAAGT

ACAAATGGAAGTGACTTGTGCTCTGCTGCAAGAAAAGTATCATGTGGAGATAGAAATAAA

AGAGCCTACAGTCATTTATATGGAAAGACCGTTAAAAAAAGCAGAGTATACCATTCACAT

CGAAGTTCCACCGAATCCTTTCTGGGCTTCCATTGGTCTATCTGTAGCACCGCTTCCATT

AGGGAGCGGAGTACAGTATGAGAGCTCGGTTTCTCTTGGATACTTAAATCAATCGTTTCA

AAATGCAGTTATGGAGGGGATACGCTATGGCTGTGAACAAGGATTGTATGGTTGGAATGT

GACGGACTGTAAAATCTGTTTTAAGTATGGCTTATACTATAGCCCTGTTAGTACCCCAGC

AGATTTTCGGATGCTTGCTCCTATTGTATTGGAACAAGTCTTAAAAAAAGCTGGAACAGA

ATTGTTAGAGCCATATCTTAGTTTTAAAATTTATGCGCCACAGGAATATCTTTCACGAGC

ATACAACGATGCTCCTAAATATTGTGCGAACATCGTAGACACTCAATTGAAAAATAATGA

GGTCATTCTTAGTGGAGAAATCCCTGCTCGGTGTATTCAAGAATATCGTAGTGATTTAAC

TTTCTTTACAAATGGACGTAGTGTTTGTTTAACAGAGTTAAAAGGGTACCATGTTACTAC

CGGTGAACCTGTTTGCCAGCCCCGTCGTCCAAATAGTCGGATAGATAAAGTACGATATAT

GTTCAATAAAATAACTTAGTGTATTTTATGTTGTTATATAAATATGGTTTCTTGTTAAAT

AAGATGAAATATTCTTTAATAAAGATTTGAATTAAAGTGTAAAGGAGGAGATAGTTATTA

TAAACTACAAGTGGATATTGTGTCCTGTATGTGGAAATAAAACACGATTAAAGATAAGGG

AAGATACTGAATTAAAAAAATTCCCCCTCTATTGTCCGAAATGCAGACAAGAAAATTTAA

TTGAAATAAAGCAGTTCAAAGTAACTGTGATTACAGAGCCAGACGCAAAGACGCAGAGCC

GATAAAATGAGATTAATACAATCTCATTTTATCGGCTCTTTCCGTTATGTATGGATTCTT

TTAATTAGTCTTCGATGTTTCTTGCTTCGTTGATACCGCTGGCTAAAGATTCCATTAAGG

ATAGTTCTTTGTCTGTAAAGCTATCCATGTATTTCTCTATCTGTAATCGTCGGGTGCTTT

TTACCAAGTTATTAGCAGGTAAGAAAAATTCATCAACGGAAACATGAAGTAACGATACAA

GGTCATAAAGAACTTGTATGCTGGGGTGTTGCCCCTTATTTTCAATATTAGTTAAGTAAC

GTGGGTCAATTTCAATCAATGCTCCCACTTGTTCACGAGTTAAACCTCGTTTCAATCGAG

CTTCTTTAATGGCTAAACCAAAGGCTCTAAAATCATATTTATCTTCTTTTTTACGCATAG

TAGACCACCTCTATACATTTTACTGTTCCTATTGAATTAGAAACAGGTATAGAAAAACAT

GTTATATAGTTTATAGGTTCATATTTAATAAAAAGCACTACTAAACGCCAATAAAAAAAC

CGTTATATGGTAGTGCTATTTATGCTGTTAAAATATTGTATCTTACTTCCAAATGGCGGT

TTGTTGGAGGTCAAAGTCGCCATGAAGTATATCACATACAATCAAGTTCCCCACATTGAG

TATTTATCAAAAAAAGTCGTCTATCTGCAATAGATAAGTACGTCCACCAATGTGGTTTTA

TAAATCATATAGATAGAAAAATAAAAGCATGTAAACAGAGAAATCAATCTGTTTGTGTGC

TTTTTTGGTTATTCAGAACTTTTTTACAAAGTTTATTCATCAGTAATGCAACAAATCCCC

CTTTCACATTGGGACTAAGAGTGAAAGGAGATAAAAGAGCAAGGCTCACTTCCTTTCCTA

GACAGAAAGGGGGTGAGAAACATGAAACCATCTTTTTTTCAGACCACAATAGAAAATCAG

TTTGACTATATCTGTAAACGTGCTATGGAAGACGAGCGAAAGAATTATCTGCTTTATCTT

TCAAGGATCGCAAAGCGTGAAGTGTCCTTTTCTGATGTTGGCGATTATCTTGTTAGCCAG

TTTGCGACAACAGATAACTATTCAACTGACTTTCAGATTTTTACACTCAATGGAATATCA

GTTGGTGTTGAAAATGATTTATTGAGTGAAGCATTACGTGAGTTGCCAGACAAGAAACGT

GAAATTCTACTGCTGTTTTACTTTATGGACATGAGCGATTCAGAAATTGCAGACCTGTTG

AAATTGAACCGTTCTACTGTCTATCGGCATAGAACCAGTGGACTAGCCTTAATCAAAAAG

TTTATGGAGGAATTTGAAGAATGAAAACACAATATCCTATGATTCCCTTTCCTCTCATTG

TAAAGGCAACAGATGGCGATACAGAAGCGATTAACCAGATTCTACATCATTACAGAGGGT

ACATAACGAAACGTTCCCTACGACTTATGAAAGATGAATATGGCAATCAAAGTATGGTCG

TTGATGAAGTCTTACGTGGAAGAATGGAAACCAGACTGATTACAAAGATTCTGTCATTTG

AAATTAAGTAATAACCTCTCTCTTTTCGTGGAAGCGTGCCATACTATTCCACGCTTCCCG

AACAGGGAGGTTTGTTATTCCATCAAAGCATATTGAGCTTTCAATGTGTTTTGATAGGCT

AACGAGCCATTGTTCTTTGAAAACTGAATAAAAGTAATTGAATACGTTTCGATAAGAAAA

GAGCCAACGGAGCTAACCGCCATGACCTATCTTCTAAAGATAGCGAGCGTTTCAGTTAGT

GTTCCGAAAAACAATCTTTAGCAGGATTGCCAGCGACGACTTTCTTATCGTGATAATGAT

ACTCCCATACAGTCAATAGTCCGAGCGTTAAAAGCGTCGCAGGCAATGAGTATGGCTACA

TGAGAACCATGCAGGGGTGGAACTCCCGTGAGCTTTGCTAGAGCTGTTCGATTGCTTGTA

AAACAACTTTTATGAAATCCAATAAGTGATTTGGAAAGGAGGATTTTATGAAGCAGACTG

ACATTCCGATTTGGGAGCGTTATACCCTAACTATTGAAGAAGCGTCAAAATATTTTCGTA

TTGGCGAAAACAAGCTGCGTCGTTTGGCAGAAGAAAATAAAAATGCAAACTGGCTGATTA

TGAATGGCAATCGTATTCAGGTTAAACGAAAACAATTTGAAAAAATTATAGATACATTGA

ACGCAATCTAGCGTAGCCAAAGGGTCTTGTATATGATAAAATAGTATTAAGTCGTATCAG

GGCTCTTTCCATAATGGAAAGGAGCAAATGCCATGTCAGAAAAAAGACGTGACAATAAAG

GTCGAATTTTAAAGACTGGAGAGAGCCAACGAAAAGACGGAAGATACTTATACAAATATA

CAGATTCATTTGGAGAACCGCAATTTGTTTACTCGTGGAAACTTGTGGCTACAGACAGAG

TACCAGCAGGAAAGCGTGATTGTATCTCACTTAGGGAGAAAATCGCAGAGTTACAGAAAG

ACATTCATGATGGTATTGATGTTGTAGGAAAGAAAATGACACTCTGCCAGCTTTACGCAA

AACAGAACGCTCAAAGACCAAAGGTTAGAAAAAACACTGAAACTGGACGCAAATATCTTA

TGGATATTTTGAAGAAAGACAAGTTAGGTGTAAGAAGTATTGACAGTATTAAGCCATCAG

ACGCTAAAGAATGGGCTATTAGAATGAGTGAAAATGGTTATGCTTATCAAACCATCAATA

ACTACAAACGTTCTTTAAAGGCTTCATTCTATATTGCTATACAAGATGATTGTGTTCGGA

AGAATCCATTTGACTTTCAACTGAAAGCAGTTCTTGATGATGATACTGTCCCTAAGACCG

TACTAACAGAAGAACAGGAAGAAAAACTGTTAGCCTTTGCAAAAGCTGATAAAACCTACA

GCAAAAATTATGATGAAATTCTGATACTCTTAAAAACAGGTCTTCGTATTTCAGAGTTTG

GTGGTTTGACACTTCCAGATTTAGATTTTGAGAATCGTCTTGTCAATATAGACCATCAGC

TATTGAGAGATACTGAAATTGGGTACTACATTGAAACACCAAAGACCAAAAGTGGCGAAC

GTCAAGTTCCTATGGTTGAAGAAGCCTATCAAGCATTTAAGCGAGTGTTAGCGAATCGAA

AGAATGATAAGCGTGTTGAGATTGATGGATATAGTGATTTCCTCTTTCTTAATAGAAAGA

ACTATCCAAAAGTGGCAAGTGATTACAACGGCATGATGAAAGGTCTTGTTAAGAAATACA

ATAAGTATAACGAGGATAAATTGCCACACATCACTCCACATAGTTTGCGACATACATTCT

GTACCAACTATGCAAATGCAGGAATGAATCCAAAGGCATTACAGTACATTATGGGACATG

CTAATATAGCCATGACGCTGAACTATTACGCACATGCAACATTCGATTCTGCAATGGCAG

AAATGAAACGCTTGAATAAAGAGAAGCAACAGGAGCGTCTTGTTGCTTAGTAGTACAAAT

GAATTTACTACTTATTTACCACTTCTGACAGCTAAGACATGAGGAAATATGCAAAGAAAC

GTGAAGTATCTTCCTACAGTAAAAATACTCGAAAGCACATAGAATAAGGCTTTACGAGCA

TTTAAGAAAATATAAAAAGATAATTAGAAATTTATACTTTGTTT

>GA17227/Tn2009

AAAATAGCATAAAAATCTAGTTATCCGCATAAAAACTGGACTTATCACACTTTATCAAGG

TCAAAACCACTCAATTTACTACTAATTTACTACTTATGAATGAGCTTTGATACGACGATT

TATCCTTGAAAAGTGAAGATATAAAGATACTTCCAATAAAATTTGAATATTTAATAGGTA

GACACTTCAAAAAATGAGGTGTCTATTTTTTTACCCGATTTTGAAAGGAAGTGAACTTAT

GAAAACAAAAAATCAAGAATCAAAAGGTCGTTCCCCACTCTTTAAGACCATCAAACATTC

ATTCAGCCAATAAAAAGAAAGGATAGGTAAAAATATGGAACTTAAATTTGTGATTCCCAA

CATGGAAAAAACATTCGGCAATTTAGAATTTGCTGGCGAGGATAAAGTCGTTCAGCGAAG

AATCAACGGACGGCTAACTGTCTTATCAAGAAGCTATAATCTCTATTCTGATGTTCAAAG

AGCAGATGATATTGTGGTGGTGCTTCCTGCTGAAGCTGGCGAAAAACATTTCGGCTTTGA

GGAACGTGTGAAGTTAGTCAATCCACGTATTACCGCAGAGGGCTACAAAATCGGCACTCG

TGGTTTTACAAATTACCTTTTACATGCTGACGACATGATAAAAGAATAAAGAAAGAGAGG

AAAAATGATGAGATTAGCAAATGGCATTGTATTAGATAAAGACACGACTTTTGGAGAATT

GAAATTCTCTGCTCTACGTCGTGAAGTGAGAATCCAAAATGAAGACGGGTCGGTTTCAGA

TGAAATCAAGGAACGTACCTATGACTTAAAATCCAAAGGACAAGGACGCATGATTCAAGT

AAGTATTCCTGCCAGCGTGCCTTTGAAAGAGTTTGATTATAACGCACGGGTGGAACTTAT

CAATCCCATTGCGGACACCGTTGCTACTGCCACCTATCAAGGAGCAGATGTTGACTGGTA

TATCAAGGCAGACGATATTGTGCTGACAAAGGATTCTAGTTCATTCAAAGCTCAACCACA

AGCAAAGAAAGAACCGACACAAGACAAATAGTCGCTAGGTAGAAAGGAGACTTTTTCGCA

TGAAACAGCGTGGTAAAAGGATTCGCCCATCTGGTAAAGATTTAGTCTTTCATTTTACGA

TAGCGTCACTCCTGCCTGTTTTCCTGCTGGTTGTCGGACTGTTTCATGTGAAGACAATCC

AGCAGATCAACTGGCAGGATTTTAACCTATCACAAGCAGATAAGATTGACATTCCCTATT

TAATTATCAGTTTCAGTGTCGCAATTCTTATCTGCTTGCTGGTAGCGTTTGTATTCAAAC

GGGTTCGCTATGATACGGTTAAACAACTTTACCACCGTCAAAAACTGGCAAAGATGATAC

TTGAAAACAAGTGGTATGAATCTGAACAGGTCAAAACAGAGGGTTTCTTTAAAGATAGTG

CTGGTCGTACAAAGGAAAAGATAACCTACTTCCCTAAAATGTATTATCGACTTAAAAATG

GCTTGATACAGATACGGGTGGAAATCACGCTGGGAAAATATCAAGACCAACTCTTACACT

TGGAAAAGAAATTAGAGAGTGGCTTGTACTGTGAGCTGACGGATAAAGAGTTAAAGGATT

CCTATGTGGAATATACTTTGCTCTATGACACCATAGCCAGTCGTATTTCTATTGATGAAG

TAGAAGCTAAAGATGGTAAACTTCGCTTAATGAAAAACGTATGGTGGGAATATGATAAGC

TCCCTCATATGTTGATTGCTGGTGGTACAGGTGGCGGTAAAACTTACTTTATACTGACAC

TGATTGAAGCCTTGCTTCATACAGATTCAAAACTGTATATTCTTGACCCGAAAAATGCTG

ATCTTGCGGACTTAGGTTCTGTGATGGCAAATGTCTACTATAGAAAAGAAGACTTGCTTT

CTTGCATTGAAACATTCTATGAAGAAATGATGAAACGTAGTGAGGAAATGAAGCAGATGA

AGAACTATAAGACTGGCAAAAATTATGCTTACTTAGGTCTCCCGGCACACTTCTTAATCT

TTGATGAATACGTCGCTTTCATGGAAATGCTGGGAACAAAAGAAAACACCGCAGTTATGA

ATAAGCTGAAACAGATTGTCATGTTAGGTCGTCAAGCTGGCTTCTTTCTAATACTGGCTT

GTCAACGTCCAGACGCAAAATATTTAGGCGACGGAATCCGTGATCAGTTTAATTTCAGAG

TGGCTTTAGGTCGTATGTCTGAAATGGGCTATGGCATGATGTTTGGCAGTGACGTACAAA

AGGATTTCTTCTTAAAGCGAATCAAAGGTCGTGGCTATGTTGATGTAGGAACAAGTGTCA

TATCAGAGTTTTATACTCCCCTTGTACCAAAAGGATATGATTTCTTGGAGGAAATTAAAA

AGTTATCCAACAGCAGACAGTCCACGCAGGCGACGTGCGAAGCGGAAGTCGCAGGTGTGG

ACTGATCTTGCTGGCTGGTGTGGCAATAGCCACGCCAGCACTTAACCCCCCGTATCTAAC

AGGGGGGTACAAATCGACAGGAAACAGTCAAAAAAACATTAGAAAATCCTTTGGTTACAA

GGGATTTACAAAATTTCAGCGTATGTCAAATGGGCTTTAAAAGTTGACATACGCCTTTTT

GATTGGAGGGATTTTTACTGAATGAACAAACTTGGTTACAGCATTTAAAAGAAAAACGCT

TGGCTTATGGACTATCTCAAAACCGTTTAGCTGTTGCGACTGGTATTACAAGGCAGTATC

TAAGCGATATTGAAACAGGAAAAGTCAAGCCATCAGAGGATTTACAGCAGTCCCTTTGGG

AAGCTCTGGAACGCTTCAATCCCGACGCTCCCCTTGAAATGCTGTTTGATTATGTAAGAA

TTCGCTTTCCGACAACAGACGTACAGCAGGTGGTCGAAAACATCTTACAACTGAAACTGT

CCTATTTTCTTCATGAGGACTATGGTTTCTATTCTTATTCAGAGCATTATGCTTTAGGCG

ACATATTCGTCCTTTGCTCCCATGAACTGGACAAAGGAGTTCTGGTGGAATTGAAAGGTC

GTGGGTGCAGACAATTTGAAAGCTATCTTCTGGCACAACAAAGAAGCTGGTATGAGTTCT

TTATGGACGTTTTGGTGGCTGGCGGTGTGATGAAACGCCTTGACCTTGCCATTAACGATA

AGACAGGGATTTTAAATATCCCTGTACTCACTGAAAAGTGCCAACAGGAAGAATGTATCT

CCGTCTTCCGCAGTTTTAAAAGCTATCGCAGTGGCGAACTGGTACGCAAAGAGGAAAAGG

AATGTATGGGAAACACCCTCTATATCGGTTCATTACAAAGTGAAGTTTATTTCTGTATCT

ATGAAAAGGACTACGAGCAGTACAAGAAAAATGATATTCCCATTGAAGACGCAGAAGTAA

AAAACCGTTTTGAGATTCGATTGAAAAATGAGCGTGCCTATTATGCAGTCCGTGATTTAC

TCGTCTATGACAATCCAGAGCATACCGCCTTTAAAATTATCAATCGGTATATCCGTTTTG

TAGATAAAGACGATTCCAAACCTCGTTCTGATTGGAAACTGAATGAAGAATGGGCTTGGT

TTATTGGGAACAATCGTGAACGATTAAAACTAACCACAAAACCAGAGCCTTACTCCTTCC

AAAGGACGCTGAACTGGCTATCTCATCAAGTTGCCCCGACCTTAAAGGTTGCGATTAAAC

TTGATGAAATCAACCAGACGCAGGTTGTAAAAGACATTCTCGACCATGCGAAACTGACAG

ACCGACACAAGCAGATTTTGAAGCAACAGTCAGTAAAAGAACAGGACGTGATAACAACAA

AAAAATAACTCAAATACAAATTCATTGAATATAGAGAGGAGAACATTTTTATGAATTTTG

GACAAAACCTTTATAACTGGTTTCTATCAAACGCTCAATCACTGGTGCTTTTAGCAATCG

TTGTGATTGGCTTGTATCTTGGCTTCAAGCGTGAGTTTAGCAAACTGATTGGCTTTTTAA

TTATTGCGATTATTGCGGTTGGCTTAGTCTTCAACGCTGCTGGAGTAAAAGACATTTTAC

TAGAGCTATTCAATCGCATTATTGGTGCTTAAATAAAACCGTTCTTTTGTGGAATATAAG

TGGTTTTCTTATGTTCCGCAAAGGAATGGTACACCAAACGAAGTGCGGTAGGGATTTTTG

AATCTCTACAAAGAAAGGACGTGAATATATGGACGATATGCAAGTCTATATTGCGAATTT

AGGCAAATACAATGAGGGCGAATTGGTCGGTGCGTGGTTTACCTTTCCCATTGACTTTGA

GGAAGTCAAAGAGAAAATCGGCTTGAATGATGAATATGAGGAATACGCCATTCATGACTA

CGAGTTACCCTTTACGGTTGACGAATACACTTCCATTGGCGAACTCAATCGACTATGGGA

AATGGTATCGGAATTACCCGAAGAATTACAATCGGAGCTATCTGCTCTGCTCACTCATTT

TTCAAGCATTGAAGAACTAAGCGAACATCAAGAGGATATTATCATTCATTCCGATTGTGA

TGATATGTATGACGTGGCACGCTACTACATTGAAGAAACGGGTGCTTTAGGCGAAGTACC

AGCTAGTCTTCAAAACTATATTGATTATCAAGCCTATGGTCGGGATTTAGACCTTTCAGG

AACGTTTATCTCAACCAATCATGGGATTTTTGAAATCGTCTATTAAATCTGTCGGTACAT

TACTACTGGCAGATTTTCTATTTTACGGGGTGGCTCAATCAGCTACCCCTATTTTTTATG

AAAGGATTGATTACATGAAGAAAATACGAAGCTATACCAGTATCTGGTCTGTGGAAAAGG

TACTGTATTCTATCAATGATTTTAGACTTCCGTTTCCCATAACCTTTACGCAAATGACAT

GGTTTGTCGTGTCACTCTTTGCAGTGATGATACTTGGCAACTTGCCCCCTCTTTCCATGA

TAGAGGGAGCATTTCTCAAATACTTTGGGATTCCTGTGGCTTTCACATGGTTTATGTCTA

CAAAAACTTTTGATGGTAAAAAGCCTTATGGATTTTTGAAGTCTGTCATTGCTTATGCAC

TGCGACCAAAGCTGACCTATGCAGGAAAAAAAGTAACGCTTGGCAGAAACCAGCCACAAG

AAGCCATTACAGCAGTTAGGAGTGAATTTTATGGCATATCCAATTAAATACATTGAAAAC

AATCTCGTCTGGAATAAAGACGGGGAATGTTATGCTTACTATGAGCTTGTTCCTTACAAT

TACTCATTTCTAAGTCCAGAACAGAAAATACAAGTGCATGATTCTTTCAGACAGCTTATC

GCACAAAATCGTGATGGCAAAATTCATGCTTTACAAATCAGTACAGAATCCAGCATACGT

TCTGCACAAGAGCGTTCCAAAAATGAAGTCACTGGCAAGCTCAAAGCGGTTGCCTATGAC

AAAATCGACCAACAGACAGACGCTTTAATATCCATGATTGGCGAAAATCAAGTGAACTAC

CGTTTCTTTATCGGCTTTAAGTTGCTTCTCAACGATCAGGAGTTTTCTATGAAAAGTCTT

ACCGTTGAAGCAAAAAATGCTTTGTCTGATTTTGTCTATGATGTGAACCATAAGCTGATG

GGCGATTTTGTTAGTATGAGTAATGATGAAATCCTGCGTTTTCAGAAGATGGAAAAGCTC

TTAGAAAATAAAATCTCTCGTCGTTTCAAAATCCGCAGGTTAGATAAGGACGACTTCGGC

TATCTGATTGAACACCTTTACGGACAGACAGGCACTGCCTATGAAGAGTATGAGTACCAT

CTATCAAAGAAAAAGCTGGATAATGAAACGCTGATTAAATACTATGACTTGATTAAGCCT

ACTCGCTGTTTGGTGGAAGAAAAACAGCGATATTTGAAAATCCAGCAGGAAGATGAAACC

GTCTATGTAGCTTACTTTACCATTAACAGCATTGTCGGAGAACTGGACTTCCCGTCCTCT

GAAATCTTCTACTACCAGCAACAGCAATTTACATTCCCGATTGATACGTCAATGAATGTG

GAAATTGTAGCGAATCGTAAAGCCCTATCTACTGTCCGCAATAAAAAGAAAGAACTGAAA

GACTTGGATAACCACGCTTGGCAAAGTGATAATGAAACCAGCTCCAATGTGGCGGAAGCT

CTGGAAAGTGTGAATGAGCTGGAAACCAATTTAGACCAAAGCAAGGAATCTATGTACAAG

CTGTCTTATGTGGTAAGGGTATCAGCAAATGATCTTGACGAACTCAAACGTCGTTGTAAT

GAAGTGAAAGATTTTTATGACGATTTAAGCGTAAAACTGGTACGACCATTTGGGGATATG

CTCGGCTTACATGAAGAATTTTTACCTGCCAGCAAGCGTTATATGAATGATTATATTCAA

TACGTGACCTCTGATTTCCTCGCTGGTTTAGGTTTTGGTGCTACTCAAATGCTGGGGGAA

AATGAGGGGATTTATGTTGGCTACAGCTTAGATACTGGACGCAATGTCTATCTGAAACCT

GCTCTTGCCAGTCAAGGGGTTAAGGGTTCAGTAACCAATGCGTTAGCGTCGGCTTTTGTT

GGTTCGCTGGGTGGTGGTAAATCCTTTGCGAATAACCTTATCGTCTATTATGCGGTGCTT

TATGGGGCACAAGCAGTGATTGTAGACCCAAAAGCAGAACGTGGCAGATGGAAAGAAACC

TTGCCAGAGATTTCCCATGAAATCAATATCGTCACTCTGACTTCTGATGAGAAAAACAAA

GGCTTACTTGACCCTTATGTGATTATGAAAAATCCCAAAGATTCTGAATCACTGGCTATT

GATATTCTGACATTCCTTACGGGGATTTCCTCTCGTGATGGGGAACGCTTCCCAATCCTT

AGAAAAGCCATTCGTGCAGTAACCAATAGTGAAGTACGAGGGTTGATGAAAGTGATTGAG

GAATTACGGGTTGAGAATACGCCACTAAGTACCAGTATAGCCGACCATATCGAAAGTTTT

ACAGACTATGACTTTGCACATTTATTATTCAGTAATGGTTATGTGGAGCAGTCTATCAGC

TTAGAAAAACAACTGAACATTATACAGGTTGCGGACTTGGTACTTCCCGACAAGGAAACT

TCCTTTGAGGAATATACCACTATGGAGCTTTTATCCGTTGCTATGCTGATTGTCATTAGT

ACCTTTGCTTTAGACTTTATCCATACAGACCGAAGCATTTTCAAGATTGTAGATTTAGAC

GAAGCATGGAGCTTTTTACAGGTAGCACAAGGAAAAACACTATCTATGAAGCTGGTTCGG

GCTGGTCGTGCTATGAACGCTGGGGTATATTTCGTGACCCAAAATACAGACGACCTCTTA

GATGAAAAACTGAAAAATAACCTCGGCTTAAAATTTGCATTTCGTTCCACTGACCTTAAC

GAGATTAAAAAGACCTTAGCCTTTTTTGGTGTAGACCCAGAGGACGAAAACAATCAGAAG

CGATTGCGTGATTTGGAAAACGGGCAATGCCTTATCAGTGATTTATATGGTCGTGTCGGT

GTGATACAGTTCCACCCTGTATTTGAAGAACTGCTCCATGCCTTTGATACCAGACCACCT

GTGCGAAAAGAGGTGTAAATGTGAAACCATCAATAGTAAACAGAATAAAATCAAACTGGA

CGCTGAAACGTCTAGGTAAAGTGGCAATGACAGTGGCTTTCACACTTGTGATTGCCATTT

TTCTTTTAGCCATGCTGGGAACGGTGGTTCAAGCTGCGGGCTTGGTAGATGATACGGTCA

ATGTGGCAAATGAATACAGCCGATACCCACTTGAAAACTATCAACTGGATTTTTATGTGG

ATAATAGCTGGGGCTGGCTTCCGTGGAACTGGTCGGACGGGATTGGAAAACAGGTCATGT

ATGGACTATATGCCATTACCAATTTTATTTGGACAATCAGTTTGTATGTTTCCAATGCGA

CAGGTTACTTAGTACAGGAAGCCTATTCCTTAGACTTCATTTCCGCTACAGCAGATTCCA

TTGGTAAGAATATGCAGACCTTAGCTGGTGTGAGTGCAAACGGATTTTCAACAGAGGGTT

TCTATGTTGGATTCCTCTTACTCTTGATTTTGGTTCTTGGGGTTTATGTTGCCTATACGG

GACTGATAAAGAGAGAAACCACAAAGGCAATTCATGCCATTATGAATTTTGTGCTGGTGT

TTATCCTATCGGCTTCCTTTATTGCCTACGCTCCCGACTACATTAAAAAATCAATGACTT

TTCATCAGACATCAGTAATGCCAGTTTATCACTTGGCACGAAGATTGTCATGCCCCATTC

CGATAGTCAAGGCAAGGACAGCGTGGACTTAATCAGAGATAGCCTGTTTTCCATACAGGT

TCAGCAACCGTGGCTACTGCTTCAATACAACAGTTCAGACATTGAAAGTATCGGTATTGA

CCGTGTGGAAAGCCTGCTCTCCACCAGCCCAGATTCCAACAATGGCGAAGACAGAGAAAA

AATTGTTGCGGAAGAAATTGAAGACAGAAGCAATACCAATCTAACCATTACAAAGACCAT

TAACCGTTTAGGTACAGTCTTCTTCCTATTTGTCTTCAATATTGGGATTTCCATATTTGT

ATTCCTATTAACAGGAATCATGATTTTCTCGCAGGTACTTTTTATCATCTATGCTATGTT

TCTGCCTGTGAGCTTTATTTTAAGCATGATTCCATCATTTGATGGTATGTCAAAACGAGC

CATAACAAAGCTCTTTAATACCATTTTGACACGAGCTGGAATCACATTGATTATTACGAC

AGCATTTAGTATTTCAACCATGCTCTATACCTTATCGGCTGGTTATCCGTTCTTTTTGAT

TGCTTTTCTACAGATTGTGACCTTTGCAGGAATCTACTTCAAGCTGGGCGATTTAATGAG

TATGTTTTCTCTACAGAGTAACGATTCTCAAAGTGTGGGAAGTCGTGTGATGAGAAAACC

TCGTATGCTTATGCACGCTCACATGCACCGTCTACAGCGGAAACTTGGACGTTCCATGAC

TACTCTAGGGGCTGGGTCTGCCATTGTTACAGGTAAAAAAGGACAGTCGGGTTCGGGGAG

TTCTGCAAGGACACAAGCAGATCACTCCCGACCAGACGGAAAGGAAAAATCAACACTTGG

AAAACGTATCGGTCAAACCATCGGTACAGTAGCTGATACCAAAGACAGAATGGTAGACAC

TGCTAGTGGTTTGAAAGAACAGGTTAAAGATTTGCCGACCAATGCAAGATATGCAGTATA

TCAAGGAAAATCCAAAGTAAAAGAGAATGTCCGTGATTTAACCAGTAGTATTTCTCAAAC

CAAAGCGGACAGAGCCAGTGGACGCAAGGAACAGCAGGAACAAAGGCGAAAAACCATTGC

GAAGCGTCGCTCTGAAATGGAACAGGTCAAACAGAAAAAACAGCCTGCTTCTTCTGTTCA

TGAAAGACCGACTACAAGACAAGAACAATATCATGATGAACAGACCTCAAAACAGTCTAA

TATTCAGACTTCATATAAGGAATCTCAACAAGCCAAACAAGAGCGTCCAGCAGTTAAGTC

CGATTTTTCAAGTCCAAAAGTGGAACGCCAAGGCAATACCGTTCAAGAAAAAACCGTTCA

AAAGCCAGCAACTTCAACCACTACAGCAGATAGAACTTCACAACGTCCAATCACAAAAGA

ACGTCCGTCTACTGTTCAAAGAGTACCACTACAAAATACAAGAAGTAGACCACCAATCAA

AACCGCCACCATTAAGAAAGTCGGTAAGAAACCATGAAGTTGAAAACTTTAGTGATTGGT

GGTTCTGGATTATTCTTGATGGTCTTCTCACTGCTTCTGTTTGTTGCCATTTTATTTTCA

GATGAACAGGACAGCGGAATTTCCAATATTCATTATGGAGGTGTGAATGTTTCCGCAGAA

GTGCTGGCTCATAAGCCTATGGTAGAAAAATATGCCAAAGAATATGGCGTTGAAGAATAT

GTCAACATACTTCTTGCGATTATACAGGTGGAATCGGGCGGTACTGCGGAAGATGTTATG

CAGTCCTCGGAATCCCTCGGTCTTCCACCTAATTCATTGAGTACAGAAGAATCCATTAAG

CAAGGTGTGAAGTATTTCAGTGAATTATTAGCCAGTAGCGAAAGGCTCAGTGTAGATTTA

GAATCGGTTATCCAGTCCTACAATTATGGTGGTGGTTTCTTAGGGTATGTGGCTAATCGT

GGAAATAAATATACCTTTGAACTGGCTCAAAGTTTCTCAAAAGAGTATTCAGGTGGCGAA

AAAGTGTCTTACCCCAATCCCATAGCCATACCTATCAATGGGGGCTGGCGATACAACTAT

GGCAATATGTTTTATGTGCAACTGGTAACGCAGTATCTTGTCACAACAGAGTTTGATGAT

GATACGGTACAAGCCATCATGGACGAAGCACTGAAATATGAGGGCTGGCGATACGTTTAC

GGTGGAGCTTCCCCGACTACTTCTTTTGATTGTAGCGGACTGACACAATGGACGTATGGA

AAAGCTGGAATTAACTTACCACGAACCGCACAACAGCAATATGATGTGACCCAGCATATC

CCACTATCGGAAGCACAAGCTGGCGATTTGGTTTTCTTTCATTCTACCTATAACGCTGGC

TCTTATATTACTCATGTTGGGATATACCTTGGCAATAACCGTATGTTTCATGCAGGCGAC

CCAATCGGTTATGCCGACTTAACAAGCCCCTACTGGCAACAGCATTTAGTGGGAGCAGGA

CGAATCAAACAATGAGAAAGGAAGATTTAATGATGAAATTTAGAAAAAATCAGAATAAAG

AAAAACAGATACCAAAGGAAAAGAAACCTCGTGTCTATAAGGTCAATCCTCATAAAAAGG

TTGTGATTGCCTTGTGGGTACTTTTAGGGCTTAGTTTCAGCTTTGCGATATTCAAGCACT

TTACAGCTATAGATACTCATACTATTCACGAAACAACTATCATAGAAAAGGAATACGTTG

ATACTCATCATGTAGAAAATTTTGTAGAGAACTTTGCGAAAGTCTACTATTCATGGGAGC

AATCCGATAAGTCCATTGATAATCGAATGGAAAGTCTAAAAGGCTATCTGACAGATGAAC

TTCAAGCTCTCAATGTTGATACAGTACGCAAAGATATTCCTGTATCGTCTTCTGTAAGAG

GATTTCAGATATGGACGGTAGAGCCAACTGGCGACAATGAGTTTAATGTAACCTACAGTG

TAGACCAGCTCATTACAGAGGGAGAAAATACAAAGACCGTCCACTCTGCTTATATAGTGA

GTGTCTATGTAGATGGTTCTGGAAATATGGTACTGGTTAAGAATCCGACCATTACCAACA

TACCTAAGAAATCAAGTTATAAACCAAAAGCCATTGAAAGTGAGGGGACGGTTGATTCCA

TTACAACCAATGAAATCAATGAGTTTTTAACGACGTTCTTCAAGCTCTATCCTACAGCGA

CAGCCAGTGAACTTTCCTACTATGTGAATGACGGGATATTAAAACCAATCGGAAAAGAGT

ACATCTTTCAAGAACTGGTAAATCCTATTCACAATCGTAAGGATAATCAAGTCACGGTAT

CGCTGACAGTGGAGTATATCGACCAGCAGACCAAAGCAACGCAGGTATCTCAATTTGATT

TGGTACTTGAAAAGAACGGGAGTAATTGGAAGATTGTAAAATAACAAATATTGGTACATG

ATTACAGATACTTTGTAATCATGTACTCTTTTTGATAAAAAATTGGAGATTCCTTTACAA

ATATGCTCTTATGTGCTATTATTTAAGTGACTATTTAAAAGGAGTTAATAAATATGCGGC

AAGGTATTCTTAAATAAACTGTCAATTTGATAGCGGAAACAAATAATTAGATGTCCTTTT

TTAGGAGGGCTTAGTTTTTTGTACCCAGTTTAAGAATACCTTTATCATGTGATTCTAAAG

TATCCAGAGAATATCTGTATGCTTTGTATACCTATGGTTATGCATAAAAATCCCAGTGAT

AAAAGTATTTATCACTGGGATTTTTATGCCCTTTTGGGTTTTTGAATGGAGGAAAATCAC

ATGAAAATTATTAATATTGGAGTTTTAGCTCATGTTGATGCAGGAAAAACTACCTTAACA

GAAAGCTTATTATATAACAGTGGAGCGATTACAGAATTAGGAAGCGTGGACAAAGGTACA

ACGAGGACGGATAATACGCTTTTAGAACGTCAGAGAGGAATTACAATTCAGACAGGAATA

ACCTCTTTTCAGTGGGAAAATACGAAGGTGAACATCATAGACACGCCAGGACATATGGAT

TTCTTAGCAGAAGTATATCGTTCATTATCAGTTTTAGATGGGGCAATTCTACTGATTTCT

GCAAAAGATGGCGTACAAGCACAAACTCGTATATTATTTCATGCACTTAGGAAAATGGGG

ATTCCCACAATCTTTTTTATCAATAAGATTGACCAAAATGGAATTGATTTATCAACGGTT

TATCAGGATATTAAAGAGAAACTTTCTGCCGAAATTGTAATCAAACAGAAGGTAGAACTG

TATCCTAATATGTGTGTGACGAACTTTACCGAATCTGAACAATGGGATACGGTAATAGAG

GGAAACGATGACCTTTTAGAGAAATATATGTCCGGTAAATCATTAGAAGCATTGGAACTC

GAACAAGAGGAAAGCATAAGATTTCATAATTGTTCCCTGTTCCCTGTTTATCACGGAAGT

GCAAAAAACAATATAGGGATTGATAACCTTATAGAAGTGATTACGAATAAATTTTATTCA

TCAACACATCGAGGTCCGTCTGAACTTTGCGGAAATGTTTTCAAAATTGAATATACAAAA

AAAGACAACGTCTTGCATATATACGCCTTTATAGTGGAGTACTACATTTACGAGATTCGG

TTAGAGTATCAGAAAAAGAAAAATAAAAGTTACAGAAATGTATACTTCAATAAATGGTGA

ATTATGTAAGATTGATAGAGCTTATTCTGGAGAAATTGTTATTTTGCAAAATGAGTTTTT

GAAGTTAAATAGTGTTCTTGGAGATACAAAACTATTGCCACAGAGAAAAAAGATTGAAAA

TCCGCACCCTCTACTACAAACAACTGTTGAACCGAGTAAACCTGAACAGAGAGAAATGTT

GCTTGATGCCCTTTTGGAAATCTCAGATAGTGATCCGCTTCTACGATATTACGTGGATTC

TACGACACATGAAATTATACTTTCTTTCTTAGGGAAAGTACAAATGGAAGTGATTAGTGC

ACTGTTGCAAGAAAAGTATCATGTGGAGATAGAACTAAAAGAGCCTACAGTCATTTATAT

GGAGAGACCGTTAAAAAATGCAGAATATACCATTCACATCGAAGTGCCGCCAAATCCTTT

CTGGGCTTCCATTGGTTTATCTGTATCACCGCTTCCGTTGGGAAGTGGAATGCAGTATGA

GAGCTCGGTTTCTCTTGGATACTTAAATCAATCATTTCAAAATGCAGTTATGGAAGGGAT

ACGCTATGGTTGCGAACAAGGATTATATGGTTGGAATGTGACGGATTGTAAAATCTGTTT

TAAGTATGGCTTATACTATAGCCCTGTTAGTACCCCAGCAGATTTTCGGATGCTTGCTCC

TATTGTATTGGAACAAGTCTTAAAAAAAGCTGGAACAGAATTGTTAGAGCCATATCTTAG

TTTTAAAATTTATGCGCCACAGGAATATCTTTCACGAGCATACACCGATGCTCCTAAATA

TTGTGCGAACATCGTAGACACTCAATTGAAAAATAATGAGGTCATTCTTAGTGGAGAAAT

CCCTGCTCGGTGTATTCAAGAATATCGTAGTGATTTAACTTTCTTTACAAATGGACGTAG

TGTTTGTTTAACAGAGTTAAAAGGGTACCATGTTACTACCGGTGAACCTGTTTGCCAGCC

CCGTCGTCCAAATAGTCGGATAGATAAAGTACGATATATGTTCAATAAAATAACTTAGTG

TATTTTATGTTGTTATATAAATATGGTTTCTTGTTAAATAAGATGAAATATTTTTTAATA

AAGATTTGAATTAAAGTGTAAAGGAGGAGATAGTTATTATAAACTACAAGTGGATATTGT

GTGCTGAGAGCTTTCTTCTATACTAATAGACGAAAGGGTGTGAAAATGATTTTTAAATGA

TACTGTGGAACGGAACAGTAGCCCTAGTATTGACTACTGTCGTTTCTATTCATATTGGCT

ATTCTAGGACTGAGATGAAAAAATCTATAAATGCTCAGAATAAAATTGAACCCGCAAATC

TCCCCAAAACAATGGTGAGTCATGTACTTGTATTATTCCGAAAAAATACACCTCTGGTGC

AGTGAGACAAATTGGTGTATCTTATAGTGGCTTCGTAGATGAAAGCTATACTCTACTATC

ACTCTTTGATGATGTAGAACAAATTGAAAAAGATAATAGACTTCAGACAGCTATTGATGT

TGTCAGAGAACAGTTTGGTTTTTTAGCCATACAAAAAGGAACCGTCCTAACTGAAGGTTC

CAGAAATATTGAACGCAGTAAACTTATCGGTGGTCATTCCGCGGGTGGATTGGAGGGATT

AAAATGAAACAAGAAAAAATACAGTACAATTTTCAGAAATCCGTAGCAAAGGATGTAATG

ATATTGAAATGCTTGAAAGATTTTTACATGGAATCGTTGAAACAGCAACTTCAAAACTTC

GTCAGAGAAAACTCAAAACAACTGAAATATCGATACGACTAGTACATGCTAAATCTGAAA

ACCGATTACCATTGGAATTTACATTTAGCATTAAGCCAACAAGCTCATCTGTGATAATCT

ATACTGAGGTAATCAATCGCTTTAAAGAATGTTACACAGGTGGGGGAATTCAAGGTTTTA

CGATTCAATTTGATAAAAATACCCTTGCCTCTGCATAGAAAGGATTTGATATGATTGACC

GTTCATATTTACCATTTCAATCAGCAAGAGAGTACCAGGATACAAAGATGCAAAAATGGA

TGGGCTTTTTCCTATCTGAACATGCATCAGCACTCTCTGATGATACAAACAAAGTAACGT

ACATGTCTGACTTATCACTAGAGAAGAAATTATTACTCCTCAGTCAAGTATACGCCGGGC

AGCTACGCACACGCATTCAAGTGATTGAAAAAAACAAGCGTGTTTCCTACACTGGAACAA

TACCAAGTCTGACCAAAGATTTCATTTTGATAAAAACTACAACAGGTCACATCAATTTGA

AATTAAAAGACATTATTAGTATTGAACTTGTCGAGGAGGTGCTCTATGAATCAGCTTGAG

TTTCAGCGTAATCACCTACAAATGGACTATTATAGCGAGAGCTACCAAGATTTTGAACGT

GACTTCTACCGCTACTCTAACATGAATATTCCATTGACCTTCCTAACTGATGATATCCTA

AAAACAATGGCGACTTCACGTAAGAATTACTTTGTCCTCAATAAGGAAAAGTCCAGAGAT

AACCGCGATCACTTCTTCATATTTGAAGTAAGTACCGTAGATGAGAATCCGCTAATCTAT

CATTATACATATAAGAAAACTACAATATATTTAGCAGAAAAATAGGAGCAGTTCAATTGA

CTGTTCCTATTTTTAATATTCATAAAATCTAAAGTCTTTATACTCTTTAACAATGGAGTC

GCCAACCAGAACAGACTATACTGACCAGCGACTACCTTAAATTTAATGTTTCAGATTTAT

TTTCTTATCTCTAATTTCATAAACTACATCTGCTACATTTTCGAGTAATCGTTTATCGTG

GGTGATAAACACGATAGTTCCGGTGTACTCCTTCATTAGTATTTCCAAAGCCTCTAAACT

TGGTATGTCAAGGAAGTTACTGGGTTCATCCATTATTAGGATGTTATATCTACCCATGAG

CATTTTAGCAAGCAACAATTTTATAATTTCTCCACCGCTTAAAACAGATAAACTTTTTCC

AATATCGTTCTGTTTGAACCCCATAGATGCTAGCACTGAACGAATTTCTGATATATTGTA

GTCACAATCCTTCTGCATAAACTCCATAACATTCTGATTACTGTTGTACTTGTAACCATT

CTGTGCAAAGTAACCTATTTTTGCCTTAGGCGAAATAGAAATTCCTTCTTCATGGTTTAA

GATCATTTGGATTAAAGTTGTTTTTCCGATTCCATTACCACCAGTTAACGCCACTTTTGC

TCCTAACGGAATTTGAAAAGATGCATTTTCAAACAGAGCCTTATCCCCAAATACTTTATT

AATTTCTGCACCGACTATAGGGTATGGATTATGGAGCTCCAATGCTTTACTTTGCCTGAA

ACGAATTCTGCGAATGCCTTCCGGAGCTTCTACTTTTCCTAAGGCCGCAATCCTGTGCTC

TAGGGTTTTAGCAGCATTATACATCTTTTTTCCTTACTTCCTATTGATTTTTGATGAGCT

AAACGCCCTCCGTCTTCAGTACTTTTTTTCTTTGAAGAACCTTTTGCCTTCTGTTCTATT

TTACGAGCCTGTTTTCGCTTTTCCTCCGCAGCCCTTTCCAATCGGGCACGTTCCGCAATA

AATTGTTCGTATTCTGCAGCTTGGCTCTTACGTTCTTCCTCTTTCTGACGAAGATAATCA

GAATAGTTTCCCCAATACTCAGTGATTTTGCCATCTTTCAGTTCCCATATTTTATCTACT

ATTTCATCAAGAAAATAGCGGTCATGGCTAATAACTAACAGTGCACCTGTAAAATATTTT

AGCTGTCCTATTAGAAAATCAATTCCTTCACGGTCTAAATGGCTCGTAGGTTCATCCGCT

AAAATACCATGAACCTGTGCCGATAAGGCCTGTGCTATTTTAAGCCTTGTTTCTTCACCA

CCGCTCATAGTCTGTATATTTAATTGCTCAACACCTAGCTTGCCTACAAGTGCAAAATCT

TTTTCCTCCTGCAGAGTTACTTCGTCCAACTGGGGAATATAGGCAAGTTCACCCAGACGA

TTCATTTTACATCCTGGGGGAGTTAATTCTCCTAAAAGTACCCTGAGTAAAGTGCTTTTT

CCAGCACCATTTGCTCCTACTAAACCAATACGGTCATAATCATATACTTCTAATTCATTT

ATATCTAAAACATCGCGTCCTTTGAATTCCACACGAATGTCTTTTGCTTTTAATATTAAT

TCCATAACATTTCCTCCTGTCTATAATCGCATGCTTTCATTTGCTTGTATGCAGGGAAAA

CCCTGCGATTTTAGCAGGAAGAGTTACATGAAAATAAGATACATAAATATTCCTCCAATA

TTGTTTATTTTAAATCTAATTTTCTAACCTCAGTTATCATTTGGCAAACTATAGCAATGC

CAATAATTAAAATACCTGATAGTAAAAACCAATGATTTACACCGATTTTATCAGCAAAGA

ATCCAGAAAGAATTAACCCAATTGGCATAGCAAGTGACATGATACTTCCGATCAAAGAAA

ATACACGTCCTAAATATTCAGGCTTAATTTTCTCCTGAAAAAGAGCTGTTTGCACACCGC

TATAAAATGGCACCGAAAGCCCCATTATTGCACAGCAAACTACGAATATTACAAATCCAT

TTGGAGGAAGTATTCCCGAAACGGCTAAACTGGTCCCCATTATAAAAAATGAACTTGTTA

TTAGTAATACATGCTTTTCGAAGCCCCCTAATCTTCCTAATAATAAGCCTCCTGCTAGCA

TCCCAAATGCAAAGGAAATTTCCGTAATAGAAATATGCACAGGCGTTCCATTAAAGTGTT

CCATGCTTATTAAAGGAAATAGTGCATTGATTGGCATATAAACAAAAGTATATAGTGTTC

CTAAGAGTAATAAGGCAAACAATCCTTTGTTTTGTCTCAGAACCACAACTCCTTCTTTCA

TCTCCCTTATGAAATTTGGTTCTAAACTTTGCACTTGATTACCCAGCTTAGGTATACGTA

CAATTGCTACCGTAATAGATGCAATCACAGCACCCAATACGTCGATGGCAATAATAGCAT

TTAAATCCCAAACGGAGTATAAGAGTGCTGCAACTGCCGGACTAACAATATAGCTTATAG

ACTGCAAAGACTGACTATAGCCTGCGCATTTCGTTAGCTGTTCTTCTGGTACTAAAAGTG

GTGTAACCGCATTGAGTGCTGGGGTATGAAAAGCTGTTCCAATGCTACGGATAAACAATA

CTATCATAATCATCCAGACAGGTAGCTCCATACAGAATGCAACAATAGCAAGCACTGCAC

CAGCTGCTGCGATAATTAAATCGGCACCAATCATTATCTTCTTCCTATCATGACGATCCA

CTAGCACACCAATGGCAGGTCCCAAAATCGCATAGGGTAAAAAACCTACTAATGAAGCCA

TAGACAAGACCATCGCAGATCCTGTTTTTTCTGTAAGGTAAAAAATAATCGCCATTTGCA

GGATGGCACTAGTGATTAATGATACTGCTTGCCCTGCCCATATTGCATAAAATTTTCGTT

TCCAATTGTTGTATTTTTCCATTTATATTATCTCCTGCATATTATTTTGCTTGAATTTCT

ATTTTGAATAGCATTCTAGGCAATAAAAAATGCAGGCCAAACCCCACAATGTGGCTTTTG

GTCTGCATACATACAATTTGGAAACATTCATATTAAAGACATAGTTAAATAAAGGTATAG

TTAAATAACCAATATCCTCACCGTAACTAATGAATGCTCAATATCGTATAAATAAGCACA

ACAAAAAAGCCTATCATCGGGTATAGATTCTGCTTTTTTTATTGCCAGCTTATCTTAAAC

GCATTGAGGCTGTCATAGTTTCGGTTCCTCCTACATCTTTGTTTATATCAATTTATAGTA

TAACACAACAAGATGATATGTTCAATATAAAAGTTATGGAATGAGACTCATACTTCCAAT

TCGATGCCAGATTTAAAGGATATGACGAAGTTTTCTTCATAGACTGTAACGCTCTGGATT

ATCTTCCTTAGTAGCAAGCGATTAGCTTTCACAAAATCTTCTGTTTGTAGTTTTAAAAAT

TCATCAGGATTTTCTAACTCAACCTCAAAATATTTCATTTTACATTCCCTCATTTCATTT

ATTGATAAATTGAGTTTGCAAAAAAGAGTGGACAATTTTTGTCTACTCTTAACCTTTAAA

ATAGTTTTTTTAATCGATTTGAAGTTGCCTAAATTATTACTTATTCGGTAAAATGAAGTA

TTGCTTTCAACAGATTTCCTTCAACTACACTTCACTTGATTCAAACAAGGTGGGTACATT

TCTATTCCCACAAACTCCTTGTCAATGGAAACAAACACGTACCCACAGGGTAAATGGAAA

TAGAAACTGATAATTTCTAGCTATCACTTCTACTCATTCCAAAAATTTTCTCACTCTGAT

ACTTACCCACCATAAAGCAAAAAGCCTTGCAATCAAGGCTTTCATTATCCCTTTCGTTCA

AAGGTTTCTAAGCTTTTACGAGCAGAGCGACACACTCAGCGGTTCGCTATCTCCGTTCTG

TCTGCGTGCTAGCACTTGTCAATCACGGACAGCTATCGCATGGGCGGAAGTAAATGCTAA

TCTTCGTCGTTTTACTCCTTGACTAGCAAACTTACCGCCTCAACATGTCCTGTATGTGGA

AATAAAACACGATTAAAGATAAGGGAAGATACTGAATTAAAAAAATTCCCCCTCTATTGT

CCGAAATGCAGACAAGAAAATTTAATTGAAATAAAGCAGTTCAAAGTAACTGTGATTACA

GAGCCAGACGCAAAGACGCAGAGCCGATAAAATGAGATTAATACAATCTCATTTTATCGG

CTCTTTCCGTTATGTATGGATTCTTTTAATTAGTCTTCGATGTTTCTTGCTTCGTTGATA

CCGCTGGCTAAAGATTCCATTAAGGATAGTTCTTTGTCTGTAAAGCTATCCATGTATTTC

TCTATCTGTAATCGTCGGGTGCTTTTTACCAAGTTATTAGCAGGTAAGAAAAATTCATCA

ACGGAAACATGAAGTAACGATACAAGGTCATAAAGAACTTGTATGCTGGGGTGTTGCCCT

TTATTTTCAATATTAGTTAAGTACCGTGGGTCAATTTCAATCAATGCTCCCACTTGTTCA

CGAGTTAAACCTCGTTTCAATCGAGCTTCTTTAATGGCTAAACCAAAGGCTCTAAAATCA

TATTTATCTTCTTTTTTACGCATAGTAGACCACCTCTATACATTTTATTGTTCCTACTGA

ATTAAAAACAGGTATAGAAAAACGTGTTATATGGTTTATAGGTTTATATTTAATAAAAAG

CACTACTAAACGCCAATAAAAAAACCGTTATATGGTAGTGCTATTTACGCTGTTAAAATA

TTGTATATTACTTCCAAATGGCGGTTTGTTGGAGGTCAACGTCGCCATGAAGTACATCAT

ATACAATAAATTTCCTTACATTGGGTTCTTGTCAAAAAAAGTCGTCTATCTGCAATAGAT

AAGTACGTCCACCAATGTGGTTTTATAAATCATATAGATAGAATAACAGAAGCATGTAAA

CAGAGAAATAAATCTGTTTATATGCTTTTTTGGCTATTCAGAACTTTTTTACAAAGTTTA

TTTATCAGTAATGCAACAAATCCCCCTTTCACATTGGGACTAAGAGTGAAAGGAGATAAA

CGAGCAAGGCTCACTTCCTTTCCTAGACAGAAAGGGGGTGAGAAACATGAAACCATCTTC

TTTTCAGACCACAATAGAAAATCAGTTTGACTATATCTGTAAACGTGCTATGGAAGACGA

GCGAAAGAATTATATGCTTTATCTTTCAAGGATTGCAAAGCGTGAGGTGTCCTTTTCGGA

TGTTGGCGATTATCTTGTTAGCCAGTTTGCGACAACAGATAACTATTCAACTGACTTTCA

GATTTTTACACTCAATGGGTTATCAGTAGGCGTTGAAAATGATTTGTTGAGTGAAGCATT

ACGTGAGTTGCCAGACAAGAAACGTGAAATTCTACTGCTGTTTTACTTTATGGACATGAG

CGATTCAGAAATTGCAGACCTGTTGAAATTGAACCGTTCTACTGTCTATCGGCATAGAAC

CAGTGGACTAGCCTTAATTAAAAAGTTTATGGAGGAATTTGAAGAATGAAAACACAATAT

CCTATGATTCCCTTTCCTCTCATTGTAAAGGCAACAGATGGCGATACCGAAGCGATTAAC

CAGATTCTACATCATTACAGAGGGTACATAACGAAGCGTTCCCTACGACTTATGAAAGAT

GAATATGGCAATCAAAGTATGGTCGTTGATGAAGTCTTACGTGGAAGAATGGAAACCAGA

CTGATTACAAAGATTTTGTCATTTGAAATTAAGTAATATCCTCTCTCCTTTCGTGGAAGC

GTGCTAAACCATTCCACGCTTCCCGAACAGGGAGGTTTGTTATTCCACCAAAGCATATTG

AGCTTTCAATGTGTTTTGATAGGCTAACGAGCCATTGTTCTTTGAAAACTGAATAAAAGT

AATCGAATACGTTTCGATAAGAAAAGAGCCAACGGAACTAACCGCCATGACCTATCTTAT

AAAGATAGCGAGCGATTCATGTTAGTGATCCGAGAAGCAATCTTTAGCAGGATTGCCTGC

AACGACATTCTTATCGTGATAATGATACTCCCATACAGTCAATAGTCCGAGCGTGATAAA

ACCGTCGCAGGCAATGAGTATGGCTACATGAGAACCATGCAGGGGTGGAACTCCCGTGAG

CTTTGCTAAAGCTGTTCGATTGCTGGTAAAACAACTTTTATGAAATCCAAATAAGTGATT

TGGAAAGGAGGATTTTATGAAGCAGACTGACATTCCTATTTGGGAACGTTATACCCTAAC

CATTGAAGAAGCGTCAAAATATTTTCGTATTGGCGAAAACAAGCTACGACGCTTGGCAGA

GGAAAATAAAAATGCAAATTGGCTGATTATGAATGGCAATCGTATTCAGATTAAACGAAA

ACAATTTGAAAAAATTATAGATACATTGGACGCAATCTAGCGTCGCCAAAGGGTCTTGTA

TATGATAAAATAGTATTAAGTCGTATCAAGGCTCTTTCCATAAAGGAAAGGAGCAAATGC

CATGTCAGAAAAAAGACGTGACAATAAAGGTCGAATCTTAAAGACTGGAGAGAGCCAACG

AAAAGACGGAAGATACTTATACAAATATATAGATTCATTTGGAGAACCGCAATTTGTTTA

CTCGTGGAAACTTGTGGCTACAGACCGAGTACCAGCAGGAAAGCGTGATTGTATCTCACT

TAGAGAGAAAATCGCAGAGTTACAGAAAGACATTCATGATGGTATTGATGTTGTAGGAAA

GAAAATGACACTCTGCCAGCTTTACGCAAAACAGAACGCTCAAAGACCAAAGGTTAGAAA

AAACACTGAAACTGGACGCAAATATCTTATGGATATTTTGAAGAAAGACAAGTTAGGTGT

AAGAAGTATTGACAGTATTAAGCCATCAGACGCTAAAGAATGGGCTATTAGAATGAGTGA

AAATGGTTATGCTTATCAAACCATCAATAACTACAAACGTTCTTTAAAGGCTTCATTCTA

TATTGCTATACAAGATGATTGTGTTCGGAAGAATCCATTTGACTTTCAACTGAAAGCAGT

TCTTGATGATGATACTGTCCCTAAGACCGTACTAACAGAAGAACAGGAAGAAAAACTGTT

AGCCTTTGCAAAAGCTGATAAAACCTACAGCAAAAATTATGATGAAATTCTGATACTCTT

AAAAACAGGTCTTCGTATTTCAGAGTTTGGTGGTTTGACACTTCCAGATTTAGATTTTGA

GAATCGTCTTGTCAATATAGACCATCAGCTATTGAGAGATACTGAAATTGGGTACTACAT

TGAAACACCAAAGACCAAAAGTGGCGAACGTCAAGTTCCTATGGTTGAAGAAGCCTATCA

AGCATTTAAGCGAGTGTTAGCGAATCGAAAGAATGATAAGCGTGTTGAGATTGATGGATA

TAGTGATTTCCTCTTTCTTAATAGAAAGAACTATCCAAAAGTGGCAAGTGATTACAACGG

CATGATGAAAGGTCTTGTTAAGAAATACAATAAGTATAACGAGGATAAATTGCCACACAT

CACTCCACATAGTTTGCGACATACATTCTGTACCAACTATGCAAATGCAGGAATGAATCC

AAAGGCATTACAGTACATTATGGGACATGCTAATATAGCCATGACGCTGAACTATTACGC

ACATGCAACATTCGATTCTGCAATGGCAGAAATGAAACGCTTGAATAAAGAGAAGCAACA

GGAGCGTCTTGTTGCTTAGTAGTACAAATGAATTTACTACTTATTTACCACTTCTGACAG

CTAAGACATGAGGAAATATGCAAAGAAACGTGAAGTATCTTCCTACAGTAAAAATACTCG

AAAGCACATAGAATAAGGCTTTACGAGCATTTAAGAAAATATAAAAAGATAATTAGAAAT

TTATACTTTGTTT

>GA19923/Tn2010

AAAATAGCATAAAAATCTAGTTATCCGCATAAAAACTGGACTTATCACACTTTATCAAGG

TCAAAACCACTCAATTTACTACTAATTTACTACTTATGAATGAGCTTTGATACGACGATT

TATCCTTGAAAAGTGAAGATATAAAGATACTTCCAATAAAATTTGAATATTTAATAGGTA

GACACTTCAAAAAATGAGGTGTCTATTTTTTTACCCGATTTTGAAAGGAAGTGAACTTAT

GAAAACAAAAAATCAAGAATCAAAAGGTCGTTCCCCACTCTTTAAGACCATCAAACATTC

ATTCAGCCAATAAAAAGAAAGGATAGGTAAAAATATGGAACTTAAATTTGTGATTCCCAA

CATGGAAAAAACATTCGGCAATTTAGAATTTGCTGGCGAGGATAAAGTCGTTCAGCGAAG

AATCAACGGACGGCTAACTGTCTTATCAAGAAGCTATAATCTCTATTCTGATGTTCAAAG

AGCAGATGATATTGTGGTGGTGCTTCCTGCTGAAGCTGGCGAAAAACATTTCGGCTTTGA

GGAACGTGTGAAGTTAGTCAATCCACGTATTACCGCAGAGGGCTACAAAATCGGCACTCG

TGGTTTTACAAATTACCTTTTACATGCTGACGACATGATAAAAGAATAAAGAAAGAGAGG

AAAAATGATGAGATTAGCAAATGGCATTGTATTAGATAAAGACACGACTTTTGGAGAATT

GAAATTCTCTGCTCTACGTCGTGAAGTGAGAATCCAAAATGAAGACGGGTCGGTTTCAGA

TGAAATCAAGGAACGTACCTATGACTTAAAATCCAAAGGACAAGGACGCATGATTCAAGT

AAGTATTCCTGCCAGCGTGCCTTTGAAAGAGTTTGATTATAACGCACGGGTGGAACTTAT

CAATCCCATTGCGGACACCGTTGCTACTGCCACCTATCAAGGAGCAGATGTTGACTGGTA

TATCAAGGCAGACGATATTGTGCTGACAAAGGATTCTAGTTCATTCAAAGCTCAACCACA

AGCAAAGAAAGAACCGACACAAGACAAATAGTCGCTAGGTAGAAAGGAGACTTTTTCGCA

TGAAACAGCGTGGTAAAAGGATTCGCCCATCTGGTAAAGATTTAGTCTTTCATTTTACGA

TAGCGTCACTCCTGCCTGTTTTCCTGCTGGTTGTCGGACTGTTTCATGTGAAGACAATCC

AGCAGATCAACTGGCAGGATTTTAACCTATCACAAGCAGATAAGATTGACATTCCCTATT

TAATTATCAGTTTCAGTGTCGCAATTCTTATCTGCTTGCTGGTAGCGTTTGTATTCAAAC

GGGTTCGCTATGATACGGTTAAACAACTTTACCACCGTCAAAAACTGGCAAAGATGATAC

TTGAAAACAAGTGGTATGAATCTGAACAGGTCAAAACAGAGGGTTTCTTTAAAGATAGTG

CTGGTCGTACAAAGGAAAAGATAACCTACTTCCCTAAAATGTATTATCGACTTAAAAATG

GCTTGATACAGATACGGGTGGAAATCACGCTGGGAAAATATCAAGACCAACTCTTACACT

TGGAAAAGAAATTAGAGAGTGGCTTGTACTGTGAGCTGACGGATAAAGAGTTAAAGGATT

CCTATGTGGAATATACTTTGCTCTATGACACCATAGCCAGTCGTATTTCTATTGATGAAG

TACAAGCTAAAGATGGTAAACTTCGCTTAATGAAAAACGTATGGTGGGAATATGATAAGC

TCCCTCATATGTTGATTGCTGGTGGTACAGGTGGCGGTAAAACTTACTTTATACTGACAC

TGATTGAAGCCTTGCTTCATACAGATTCAAAACTGTATATTCTTGACCCGAAAAATGCTG

ACCTTGCGGACTTAGGTTCTGTGATGGCAAATGTCTACTATAGAAAAGAAGACTTGCTTT

CTTGCATTGAAACATTCTATGAAGAAATGATGAAACGTAGTGAGGAAATGAAGCAGATGA

AGAACTATAAGACTGGCAAAAATTATGCTTACTTAGGTCTCCCGGCACACTTCTTAATCT

TTGATGAATACGTCGCTTTCATGGAAATGCTGGGAACAAAAGAAAACACCGCAGTTATGA

ATAAGCTGAAACAGATTGTCATGTTAGGTCGTCAAGCTGGCTTCTTTCTAATACTGGCTT

GTCAACGTCCAGACGCAAAATATTTAGGCGACGGAATCCGTGATCAGTTTAATTTCAGAG

TGGCTTTAGGTCGTATGTCTGAAATGGGCTATGGCATGATGTTTGGCAGTGACGTACAAA

AGGATTTCTTCTTAAAGCGAATCAAAGGTCGTGGCTATGTTGATGTAGGAACAAGTGTCA

TATCAGAGTTTTATACTCCCCTTGTACCAAAAGGATATGATTTCTTGGAGGAAATTAAAA

AGTTATCCAACAGCAGACAGTCCACGCAGGCGACGTGCGAAGCGGAAGTCGCAGGTGTGG

ACTGATCTTGCTGGCTGGTGTGGCAATAGCCACGCCAGCACTTAACCCCCCGTATCTAAC

AGGGGGGTACAAATCGACAGGAAACAGTCAAAAAAACATTAGAAAATCCTTTGGTTACAA

GGGATTTACAAAATTTCAGCGTATGTCAAATGGGCTTTAAAAGTTGACATACGCCTTTTT

GATTGGAGGGATTTTTACTGAATGAACAAACTTGGTTACAGCAGTTAAAAGAAAAACGCT

TGGCTTATGGACTATCTCAAAATCGTTTAGCGGTTGCGACTGGTATTACAAGGCAGTATC

TAAGCGATATTGAAACAGGAAAAGTCAAGCCATCAGAGGATTTACAGCAATCTCTTTTTG

AAGCTCTGGAACGCTTCAATCCCGACGCTCCCCTTGAAATGCTCTTTGATTATGTAAGGA

TTCGCTTTCCCACAACGGACGTACAGCATGTGGTCGAAAACATCTTACAACTGAAACTGT

CCTATTTTCTTCATGAGGACTATGGTTTCTATTCTTATTCAGAGCATTATGCTTTAGGCG

ATATATTCGTTCTCTGCTCCCACGAACTGGACAAAGGAGTTCTGGTGGAATTGAAAGGTC

GTGGGTGTCGGCAATTTGAAAGCTATCTTCTGGCTCAACAAAGAAGCTGGTATGAGTTCT

TTATGGACGCTTTGGTGGCTGGCGGTGTGATGAAACGCCTTGACCTTGCCATTAACGATA

AGACAGGGATTTTGAATATCCCTGTACTCACTGAAAAGTGCCAACAGGAAGAATGTATCT

CCGTCTTCCGCAGTTTTAAAAGCTATCGCAGTGGCGAACTGGTACGCAAAGAGGAAAAGG

AATGTATGGGAAACACCCTCTATATCGGTTCATTACAAAGTGAAGTTTATTTCTGTATCT

ATGAAAAGGACTACGAGCAGTACAAGAAAAATGATATTCCCATTGAAGACGCAGAAGTAA

AAAACCGTTTTGAGATTCGATTGAAAAATGAGCGTGCCTATTATGCAGTCCGTGATTTAC

TCGTCTATGACAATCCAGAGCATACCGCCTTTAAAATTATCAATCGGTATATCCGTTTTG

TAGATAAAGACGATTCCAAACCTCGTTCTGATTGGAAACTGAATGAAGAATGGGCTTGGT

TTATTGGGAACAATCGTGAACGATTAAAACTAACCACAAAACCAGAGCCTTACTCCTTCC

AAAGGACGCTGAACTGGCTATCTCATCAAGTTGCCCCGACCTTAAAGGTTGCGATTAAAC

TTGATGAAATCAACCAGACGCAGGTTGTAAAAGACATTCTCGACCATGCGAAACTGACAG

ACCGACACAAGCAGATTTTGAAGCAACAGTCAGTAAAAGAACAGGACGTGATAACAACAA

AAAAAGGATATCTGTCAACCATACCAGTTGACAGATATCCAAAAAAAAGATATAATGGGA

GATAAGACGGTTCGTGTTCGTGCTGACTTGCACCATATCATAAAAATCGAAACAGCAAAG

AATGGCGGAAACGTAAAAGAAGTTATGGAAATAAGACTTAGAAGCAAACTTAAGAGTGTG

TTGATAGTGCATTATCTTAAAATTTTGTATAATAGGAATTGAAGTTAAATTAGATGCTAA

AAATTTGTAATTAAGAAGGAGGGATTCGTCATGTTGGTATTCCAAATGCGTAATGTAGAT

AAAACATCTACTGTTTTGAAACAGACTAAAAACAGTGATTACGCAGATAAATAAATACGT

TAGATTAATTCCTACCAGTGACTAATCTTATGACTTTTTAAACAGATAACTAAAATTACA

AACAAATCGTTTAACTTCTGTATTTGTTTATAGATGTAATCACTTCAGGAGAGATTACAT

GAACAAAAATATAAAATATTCTCAAAACTTTTTAACGAGTGAAAAAGTACTCAACCAAAT

AATAAAACAATTGAATTTAAAAGAAACCGATACCGTTTACGAAATTGGAACAGGTAAAGG

GCATTTAACGACGAAACTGGCTAAAATAAGTAAACAGGTAACGTCTATTGAATTAGACAG

TCATCTATTCAACTTATCGTCAGAAAAATTAAAACTGAATACTCGTGTCACTTTAATTCA

CCAAGATATTCTACAGTTTCAATTCCCTAACAAACAGAGGTATAAAATTGTTGGGAATAT

TCCTTACCATTTAAGCACACAAATTATTAAAAAAGTGGTTTTTGAAAGCCGTGCGTCTGA

CATCTATCTGATTGTTGAAGAAGGATTCTACAAGCGTACCTTGGATATTCACCGAACACT

AGGGTTGCTCTTGCACACTCAAGTCTCGATTCAGCAATTGCTTAAGCTGCCAGCGGAATG

CTTTCATCCTAAACCAAAAGTAAACAGTGTCTTAATAAAACTTACCCGCCATACCACAGA

TGTTCCAGATAAATATTGGAAGCTATATACGTACTTTGTTTCAAAATGGGTCAATCGAGA

ATATCGTCAACTGTTTACTAAAAATCAGTTTCATCAAGCAATGAAACACGCCAAAGTAAA

CAATTTAAGTACCATTACTTATGAGCAAGTATTGTCTATTTTTAATAGTTATCTATTATT

TAACGGGAGGAAATAATTCTATGAGTCGCTTTTTTAAATTTGGAAAGTTACACGTTACTA

AAGGGAATGGAGATAAATTATTAGATATACTACTGACAGCTTCCAAGAAGCTAAAGAGGT

CCCTAGCGCCTACGGGGAATTTGTATCGATAAGGGGTACAAATTCCCACTAAGCGCTCGG

GACCCCTTGTAGGAAAATGTCCTAAGTGGGATATCTGTCAACTGGTATGGTTGACTAAAA

ATACTTCCTACGAAAATGTAGGGGGTATTTTTTTACGAAAAAATACAATCGATTCTTAAA

AAGAAAAATTTTTGATTGGCAAAACCATAACAAGTTCGTTTTAGGGTTTTGATTTTGCGA

TTGATGCCTTCTAAAGGACCATTAGAGTATTCAAATTTAGCGCTATTTAAGACATATTTT

CTGTTTTGACGAAGGGTTTGAATAGCAGTATCCATTTCTGTATTGGTTTTTTGGTAGTCT

AAGATGGTTGACTCTAGTAATTCACTATTGCGCTCGTTTAGGGCTTTCGTGATATCTTGG

TAAGTTTGGTATACTTCAGCGAACTTGGAAAATTTACTAGTAATGAGATCAACAGCATTT

TGGCGAGTCATATATTGTTTAACGCCGCGAAGAAAAACTACTTCTTCAGGGTGAAGATCT

TCAGCTTTTTTATGGAATAGCTTCCAATGTGACTTCATAATTTTATATTCTTGGCTCTGT

TTATCAAGTTGCTTTAGGATAGAGATACGACAATTGTCCAAAGCGCGACCAGCTAATTGT

ACAAGGTGGAAGCGATCAATAATGATATTGGCATTAGGGAAAAGGCGATAGATAAAACTT

TGATATTGAGCATTTAAATCAATTACAACTGATTGAACGCATTCGCGTTCGGCTTTTGAA

TAACGACTTTCAAAATAATCAACAATGGTAGGTGATAGACGATCCTGTAACTTTGTGACA

ATTTGGTGGGTTTCAGCGTCACAACAGATAAAGGACATCACAGACTTAATTGAACGAAAC

TCGTCAAAACATAGATGCTTAGGCAACTTAGCCACACGATAGTGTGGTTCCATGCGCTCT

AAGATTGTTCGACGAACACTGCTAGGAGAGCAGTGACACATTTCAGCAATAAGCTGACCA

GATAAGCCTTTACGAGCTAAAAGCATGATTTGATTTTTGAGATCACTGGATAAGGTTTGA

TTTTCTTTGGTTAAATTAGTAATAGCACCAAAAGTAGTATGGCATGATTTACATTTATAG

CGTTGTTTACGAAGCTCTAGTTCATATCTTCTCCCATTTAAACTTGCCAGTCGTACATGA

GTTTTGCGAAAGCCATCCTTATTAACTGTGGGAAAGCCACAGTTACGACAACGATTAATC

GGATAAGAAAGAGTAGCTGTTATTAGCGTTATATACTCTTTAACAGAATCGTTGTTGTGT

TCAGCTTCTTCAACAGAAATAATTTTAATATTTTTATCTTTAATTCCAAGAATATTTAGG

ATAGAATCATTATGGGACATTTGTTTAACCTTCTTTCATGATTTTTGTGGTGAATTGATT

GTATAACGAGGGGACAGCAAATGTCCTCTTTTTTGTATAAAAAAATCTGGCATGGAATCT

CTATCCATACCAGAAAGTGTATACCCCAAAAAAATAACTCAAATACAAATTCATTGAATA

TAGAGAGGAGAACATTTTTATGAATTTTGGACAAAACCTTTATAACTGGTTTCTATCAAA

CGCTCAATCACTGGTGCTTTTAGCAATCGTTGTGATTGGCTTGTATCTTGGCTTCAAGCG

TGAGTTTAGCAAACTGATTGGCTTTTTAATTATTGCGATTATTGCGGTTGGCTTAGTCTT

CAACGCTGCTGGAGTAAAAGACATTTTACTAGAGCTATTCAATCGCATTATTGGTGCTTA

AATAAAACCGTTCTTTTGTGGAATATAAGTGGTTTTCTTATGTTCCGCAAAGGAATGGTA

CACCAAACGAAGTGCGGTAGGGATTTTTGAATCTCTACAAAGAAAGGACGTGAATATATG

GACGATATGCAAGTCTATATTGCGAATTTAGGCAAATACAATGAGGGCGAATTGGTCGGT

GCGTGGTTTACCTTTCCCATTGACTTTGAGGAAGTCAAAGAGAAAATCGGCTTGAATGAT

GAATATGAGGAATACGCCATTCATGACTACGAGTTACCCTTTACGGTTGACGAATACACT

TCCATTGGCGAACTCAATCGACTATGGGAAATGGTATCGGAATTACCCGAAGAATTACAA

TCGGAGCTATCTGCTCTGCTCACTCATTTTTCAAGCATTGAAGAACTAAGCGAACATCAA

GAGGATATTATCATTCATTCCGATTGTGATGATATGTATGACGTGGCACGCTACTACATT

GAAGAAACGGGTGCTTTAGGCGAAGTACCAGCTAGTCTTCAAAACTATATTGATTATCAA

GCCTATGGTCGGGATTTAGACCTTTCAGGAACGTTTATCTCAACCAATCATGGGATTTTT

GAAATCGTCTATTAAATCTGTCGGTACATTACTACTGGCAGATTTTCTATTTTACGGGGT

GGCTCAATCAGCTACCCCTATTTTTTATGAAAGGATTGATTACATGAAGAAAATACGAAG

CTATACCAGTATCTGGTCTGTGGAAAAGGTACTGTATTCTATCAATGATTTTAGACTTCC

GTTTCCCATAACCTTTACGCAAATGACATGGTTTGTCGTGTCACTCTTTGCAGTGATGAT

ACTTGGCAACTTGCCCCCTCTTTCCATGATAGAGGGAGCATTTCTCAAATACTTTGGGAT

TCCTGTGGCTTTCACATGGTTTATGTCTACAAAAACTTTTGATGGTAAAAAGCCTTATGG

ATTTTTGAAGTCTGTCATTGCTTATGCACTGCGACCAAAGCTGACCTATGCAGGAAAAAA

AGTAACGCTTGGCAGAAACCAGCCACAAGAAGCCATTACAGCAGTTAGGAGTGAATTTTA

TGGCATATCCAATTAAATACATTGAAAACAATCTCGTCTGGAATAAAGACGGGGAATGTT

ATGCTTACTATGAGCTTGTTCCTTACAATTACTCATTTCTAAGTCCAGAACAGAAAATAC

AAGTGCATGATTCTTTCAGACAGCTTATCGCACAAAATCGTGATGGCAAAATTCATGCTT

TACAAATCAGTACAGAATCCAGCATACGTTCTGCACAAGAGCGTTCCAAAAATGAAGTCA

CTGGCAAGCTCAAAGCGGTTGCCTATGACAAAATCGACCAACAGACAGACGCTTTAATAT

CCATGATTGGCGAAAATCAAGTGAACTACCGTTTCTTTATCGGCTTTAAGTTGCTTCTCA

ACGATCAGGAGTTTTCTATGAAAAGTCTTACCGTTGAAGCAAAAAATGCTTTGTCTGATT

TTGTCTATGATGTGAACCATAAGCTGATGGGCGATTTTGTTAGTATGAGTAATGATGAAA

TCCTGCGTTTTCAGAAGATGGAAAAGCTCTTAGAAAATAAAATCTCTCGTCGTTTCAAAA

TCCGCAGGTTAGATAAGGACGACTTCGGCTATCTGATTGAACACCTTTACGGACAGACAG

GCACTGCCTATGAAGAGTATGAGTACCATCTATCAAAGAAAAAGCTGGATAATGAAACGC

TGATTAAATACTATGACTTGATTAAGCCTACTCGCTGTTTGGTGGAAGAAAAACAGCGAT

ATTTGAAAATCCAGCAGGAAGATGAAACCGTCTATGTAGCTTACTTTACCATTAACAGCA

TTGTCGGAGAACTGGACTTCCCGTCCTCTGAAATCTTCTACTACCAGCAACAGCAATTTA

CATTCCCGATTGATACGTCAATGAATGTGGAAATTGTAGCGAATCGTAAAGCCCTATCTA

CTGTCCGCAATAAAAAGAAAGAACTGAAAGACTTGGATAACCACGCATGGCAAAGTGATA

ATGAAACCAGCTCTAATGTGGCGGAAGCTCTGGAAAGTGTGAACGAGCTGGAAACCAATT

TAGACCAAAGCAAGGAATCTATGTATAAGCTGTCCTATGTTGTAAGGGTATCAGCAAATG

ACCTTGACGAACTTAAACGTCGTTGTAATGAAGTGAAAGATTTCTATGATGATTTGAGCG

TGAAACTGGTACGACCTTTTGGGGATATGCTGGGCTTACATGAAGAATTTTTACCTGCCA

GCAAAAGATATATGAATGACTATATTCAATACGTGACCTCTGATTTCCTCGCTGGTTTAG

GTTTTGGTGCTACTCAAATGCTGGGTGAAAATGAGGGGATTTATGTTGGCTACAGCTTAG

ATACTGGACGCAATGTCTATCTGAAACCTGCTCTTGCCAGTCAAGGGGTTAAGGGTTCAG

TAACCAATGCGTTAGCGTCTGCCTTTGTCGGTTCGCTGGGTGGTGGTAAATCCTTTGCGA

ATAACCTTATCGTCTATTATGCAGTGCTTTATGGGGCACAAGCAGTGATTGTAGACCCAA

AAGCAGAACGTGGCAGATGGAAAGAAACCTTGCCAGAGATTTCCCATGAAATCAATATCG

TCACTCTGACTTCTGATGAGAAAAACAAAGGCTTACTTGACCCTTATGTGATTATGAAAA

ATCCCAAAGATTCTGAATCACTGGCTATTGATATTCTGACATTCCTTACGGGGATTTCCT

CTCGTGATGGGGAACGCTTCCCAATCCTTAGAAAAGCCATTCGTGCAGTAACCAATAGTG

AAGTACGAGGGTTGATGAAAGTGATTGAGGAATTACGGGTTGAGAATACGCCACTAAGTA

CCAGTATAGCCGACCATATCGAAAGTTTTACAGACTATGACTTTGCACATTTATTATTCA

GTAATGGTTATGTGGAGCAGTCTATCAGCTTAGAAAAACAACTGAACATTATACAGGTTG

CGGACTTGGTACTTCCCGACAAGGAAACTTCCTTTGAGGAATATACCACTATGGAGCTTT

TATCCGTTGCTATGCTGATTGTCATTAGTACCTTTGCTTTAGACTTTATCCATACAGACC

GAAGCATTTTCAAGATTGTAGATTTAGACGAAGCATGGAGCTTTTTACAGGTAGCACAAG

GAAAAACACTATCTATGAAGCTGGTTCGGGCTGGTCGTGCTATGAACGCTGGGGTATATT

TCGTGACCCAAAATACAGACGACCTCTTAGATGAAAAACTGAAAAATAACCTCGGCTTAA

AATTTGCATTTCGTTCCACTGACCTTAACGAGATTAAAAAGACCTTAGCCTTTTTTGGTG

TAGACCCAGAGGACGAAAACAATCAGAAGCGATTGCGTGATTTGGAAAACGGGCAATGCC

TTATCAGTGATTTATATGGTCGTGTCGGTGTGATACAGTTCCACCCTGTATTTGAAGAAC

TGCTCCATGCCTTTGATACCAGACCACCTGTGCGAAAAGAGGTGTAAATGTGAAACCATC

AATAGTAAACAGAATAAAATCAAACTGGACGCTGAAACGTCTAGGTAAAGTGGCAATGAC

AGTGGCTTTCACACTTGTGATTGCCATTTTTCTTTTAGCCATGCTGGGAACGGTGGTTCA

AGCTGCGGGCTTGGTAGATGATACGGTCAATGTGGCAAATGAATACAGCCGATACCCACT

TGAAAACTATCAACTGGATTTTTATGTGGATAATAGCTGGGGCTGGCTTCCGTGGAACTG

GTCGGACGGGATTGGAAAACAGGTCATGTATGGACTATATGCCATTACCAATTTTATTTG

GACAATCAGTTTGTATGTTTCCAATGCGACAGGTTACTTAGTACAGGAAGCCTATTCCTT

AGACTTCATTTCCGCTACAGCAGATTCCATTGGTAAGAATATGCAGACCTTAGCTGGTGT

GAGTGCAAACGGATTTTCAACAGAGGGTTTCTATGTTGGATTCCTCTTACTCTTGATTTT

GGTTCTTGGGGTTTATGTTGCCTATACGGGACTGATAAAGAGAGAAACCACAAAGGCAAT

TCATGCCATTATGAATTTTGTGCTGGTGTTTATCCTATCGGCTTCCTTTATTGCCTACGC

TCCCGACTACATTAAAAAAATCAATGACTTTTCATCAGACATCAGTAATGCCAGTTTATC

ACTTGGCACGAAGATTGTCATGCCCCATTCCGATAGTCAAGGCAAGGACAGCGTGGACTT

AATCAGAGATAGCCTGTTTTCCATACAGGTTCAGCAACCGTGGCTACTGCTTCAATACAA

CAGTTCAGACATTGAAAGTATCGGTATTGACCGTGTGGAAAGCCTGCTCTCCACCAGCCC

AGATTCCAACAATGGCGAAGACAGAGAAAAAATTGTTGCGGAAGAAATTGAAGACAGAAG

CAATACCAATCTAACCATTACAAAGACCATTAACCGTTTAGGTACAGTCTTCTTCCTATT

TGTCTTCAATATTGGGATTTCCATATTTGTATTCCTATTAACAGGAATCATGATTTTCTC

GCAGGTACTTTTTATCATCTATGCTATGTTTCTGCCTGTGAGCTTTATTTTAAGCATGAT

TCCATCATTTGATGGTATGTCAAAACGAGCCATAACAAAGCTCTTTAATACCATTTTGAC

ACGAGCTGGAATCACATTGATTATTACGACAGCATTTAGTATTTCAACCATGCTCTATAC

CTTATCGGCTGGTTATCCGTTCTTTTTGATTGCTTTTCTACAGATTGTGACCTTTGCAGG

AATCTACTTCAAGCTGGGCGATTTAATGAGTATGTTTTCTCTACAGAGTAACGATTCTCA

AAGTGTGGGAAGTCGTGTGATGAGAAAACCTCGTATGCTTATGCACGCTCACATGCACCG

TCTACAGCGGAAACTTGGACGTTCCATGACTACTCTAGGGGCTGGGTCTGCCATTGTTAC

AGGTAAAAAAGGACAGTCGGGTTCGGGGAGTTCTGCAAGGACACAAGCAGATCACTCCCG

ACCAGACGGAAAGGAAAAATCAACACTTGGAAAACGTATCGGTCAAACCATCGGTACAGT

AGCTGATACCAAAGACAGAATGGTAGACACTGCTAGTGGTTTGAAAGAACAGGTTAAAGA

TTTGCCGACCAATGCAAGATATGCAGTATATCAAGGAAAATCCAAAGTAAAAGAGAATGT

CCGTGATTTAACCAGTAGTATTTCTCAAACCAAAGCGGAAAGAGCCAGTGGACGCAAGGA

ACAGCAGGAACAAAGGCGAAAAACCATTGCGAAGCGTCGCTCTGAAATGGAACAGGTCAA

ACAGAAAAAACAGCCTGCTTCTTCTGTTCATGAAAGACCGACTACAAGACAAGAACAATA

TCATGATGAACAGACCTCAAAACAGTCTAATATTCAGACTTCATATAAGGAATCTCAACA

AGCCAAACAAGAGCGTCCAGCAGTTAAGTCCGATTTTTCAAGTCCAAAAGTGGAACGCCA

AGGCAATACCGTTCAAGAAAAAACCGTTCAAAAGCCAGCAACTTCAACCACTACAGCAGA

TAGAACTTCACAACGTCCAATCACAAAAGAACGTCCGTCTACTGTTCAAAGAGTACCACT

ACAAAATACAAGAAGTAGACCACCAATCAAAACCGCCACCATTAAGAAAGTCGGTAAGAA

ACCATGAAGTTGAAAACTTTAGTGATTGGTGGTTCTGGATTATTCTTGATGGTCTTCTCA

CTGCTTCTGTTTGTTGCCATTTTATTTTCAGATGAACAGGACAGCGGAATTTCCAATATT

CATTATGGAGGTGTGAATGTTTCCGCAGAAGTGCTGGCTCATAAGCCTATGGTAGAAAAA

TATGCCAAAGAATATGGCGTTGAAGAATATGTCAACATACTTCTTGCGATTATACAGGTG

GAATCGGGCGGTACTGCGGAAGATGTTATGCAGTCCTCGGAATCCCTCGGTCTTCCACCT

AATTCATTGAGTACAGAAGAATCCATTAAGCAAGGTGTGAAGTATTTCAGTGAATTATTA

GCCAGTAGCGAAAGGCTCAGTGTAGATTTAGAATCGGTTATCCAGTCCTACAATTATGGT

GGTGGTTTCTTAGGGTATGTGGCTAATCGTGGAAATAAATATACCTTTGAACTGGCTCAA

AGTTTCTCAAAAGAGTATTCAGGTGGCGAAAAAGTGTCTTACCCCAATCCCATAGCCATA

CCTATCAATGGGGGCTGGCGATACAACTATGGCAATATGTTTTATGTGCAACTGGTAACG

CAGTATCTTGTCACAACAGAGTTTGATGATGATACGGTACAAGCCATCATGGACGAAGCA

CTGAAATATGAGGGCTGGCGATACGTTTACGGTGGAGCTTCCCCGACTACTTCTTTTGAT

TGTAGCGGACTGACACAATGGACGTATGGAAAAGCTGGAATTAACTTACCACGAACCGCA

CAACAGCAATATGATGTGACCCAGCATATCCCACTATCGGAAGCACAAGCTGGCGATTTG

GTTTTCTTTCATTCTACCTATAACGCTGGCTCTTATATTACTCATGTTGGGATATACCTT

GGCAATAACCGTATGTTTCATGCAGGCGACCCAATCGGTTATGCCGACTTAACAAGCCCC

TACTGGCAACAGCATTTAGTGGGAGCAGGACGAATCAAACAATGAGAAAGGAAGATTTAA

TGATGAAATTTAGAAAAAATCAGAATAAAGAAAAACAGATACCAAAGGAAAAGAAACCTC

GTGTCTATAAGGTCAATCCTCATAAAAAGGTTGTGATTGCCTTGTGGGTACTTTTAGGGC

TTAGTTTCAGCTTTGCGATATTCAAGCACTTTACAGCTATAGATACTCATACTATTCACG

AAACAACTATCATAGAAAAGGAATACGTTGATACTCATCATGTAGAAAATTTTGTAGAGA

ACTTTGCGAAAGTCTACTATTCATGGGAGCAATCCGATAAGTCCATTGATAATCGAATGG

AAAGTCTAAAAGGCTATCTGACAGATGAACTTCAAGCTCTCAATGTTGATACAGTACGCA

AAGATATTCCTGTATCGTCTTCTGTAAGAGGATTTCAGATATGGACGGTAGAGCCAACTG

GCGACAATGAGTTTAATGTAACCTACAGTGTAGACCAGCTCATTACAGAGGGAGAAAATA

CAAAGACCGTCCACTCTGCTTATATAGTGAGTGTCTATGTAGATGGTTCTGGAAATATGG

TACTGGTTAAGAATCCGACCATTACCAACATACCTAAGAAATCAAGTTATAAACCAAAAG

CCATTGAAAGTGAGGGGACGGTTGATTCCATTACAACCAATGAAATCAATGAGTTTTTAA

CGACGTTCTTCAAGCTCTATCCTACAGCGACAGCCAGTGAACTTTCCTACTATGTGAATG

ACGGGATATTAAAACCAATCGGAAAAGAGTACATCTTTCAAGAACTGGTAAATCCTATTC

ACAATCGTAAGGATAATCAAGTCACGGTATCGCTGACAGTGGAGTATATCGACCAGCAGA

CCAAAGCAACGCAGGTATCTCAATTTGATTTGGTACTTGAAAAGAACGGGAGTAATTGGA

AGATTATAGAATAACAAATATTGGTACATTATTACAGCTATTTTGTAATCACGTACTCTC

TTTGATAAAAAATTGGAGATTCCTTTACAAATATGCTCTTACGTGCTATTATTTAAGTAT

CTATTTAAAAGGAGTTAATAAATATGCGGCAAGGTATTCTTAAATAAACTGTCAATTTGA

TAGTGCGAACAAATAATTGGATGTCCTTTTTTAGGAGGGCTTAGTTTTTTGTACCCAGTT

TAAGAATACCTTTATCATGTGATTCTAAAGTATCCGGAGAATATCTGTATGCTTTGTATG

CCTATGGTTATGCATAAAAATCCCAGTGATAAGAGTATTTNNNNNCACACACTTAATTAA

TTAAGTGTGTGNNNNNTATCACTGGGATTTTTATGCCCTTTTGGGCTTTTGAATGGAGGA

AAATCACATGAAAATTATTAATATTGGAGTTTTAGCTCATGTTGATGCGGGAAAAACTAC

CTTAACAGAAAGCTTATTATATAACAGTGGAGCGATTACAGAATTAGGAAGCGTGGACAA

AGGTACAACGAGGACGGATAATACGCTTTTAGAACGTCAGAGAGGAATTACAATTCAGAC

AGGAATAACCTCTTTTCAGTGGGAAAATACGAAGGTGAACATCATAGACACGCCAGGACA

TATGGATTTCTTAGCAGAAGTATATCGTTCATTATCAGTTTTAGATGGGGCAATTCTACT

GATTTCTGCAAAAGATGGCGTACAAGCACAAACTCGTATATTATTTCATGCACTTAGGAA

AATGGGGATTCCCACAATCTTTTTTATCAATAAGATTGACCAAAATGGAATTGATTTATC

AACGGTTTATCAGGATATTAAAGAGAAACTTTCTGCCGAAATTGTAATCAAACAGAAGGT

AGAACTGTATCCTAATATGTGTGTGACGAACTTTACCGAATCTGAACAATGGGATACGGT

AATAGAGGGAAACGATGACCTTTTAGAGAAATATATGTCCGGTAAATCATTAGAAGCATT

GGAACTCGAACAAGAGGAAAGCATAAGATTTCATAATTGTTCCCTGTTCCCTGTTTATCA

CGGAAGTGCAAAAAACAATATAGGGATTGATAACCTTATAGAAGTGATTACGAATAAATT

TTATTCATCAACACATCGAGGTCAGTCTGAACTTTGCGGAAAAGTTTTCAAAATTGAGTA

TTCGGAAAAAAGACAGCGTCTTGCATATATACGTCTTTATAGTGGCGTACTGCATTTGCG

AGATTCGGTTAGAATATCGGAAAAGGAAAAATAAAAATTACAGAAATGTATACTTCAATA

AATGGTGAATTATGTAAAATCGATAAGGCTTATTCCGGGGAAATTGTTATTTTGCAGAAT

GAGTTTTTGAAGTTAAATAGTGTTCTTGGAGATACAAAGCTATTGCCACAGAGAGAGAGA

ATTGAAAATCCCCTCCCTCTGCTGCAAACAACTGTTGAACCGAGCAAACCTCAACAAAGG

GAAATGTTACTTGATGCACTTTTAGAAATCTCCGACAGTGACCCGCTTCTGCGATATTAT

GTGGATTCTGCGACACATGAAATCATACTTTCTTTCTTAGGGAAAGTACAAATGGAAGTG

ACTTGTGCTCTGCTGCAAGAAAAGTATCATGTGGAGATAGAAATAAAAGAGCCTACAGTC

ATTTATATGGAAAGACCGTTAAAAAAAGCAGAGTATACCATTCACATCGAAGTTCCACCG

AATCCTTTCTGGGCTTCCATTGGTTTATCTGTATCACCGCTTCCGTTGGGAAGTGGAATG

CAGTATGAGAGCTCGGTTTCTCTTGGATACTTAAATCAATCATTTCAAAATGCAGTTATG

GAAGGGATACGCTATGGCTGTGAACAAGGATTGTATGGTTGGAATGTGACGGACTGTAAA

ATCTGTTTTAAGTATGGCTTATACTATAGCCCTGTTAGTACCCCAGCAGATTTTCGGATG

CTTGCTCCTATTGTATTGGAACAAGTCTTAAAAAAAGCTGGAACAGAATTGTTAGAGCCA

TATCTTAGTTTTAAAATTTATGCGCCACAGGAATATCTTTCACGAGCATACACCGATGCT

CCTAAATATTGTGCGAACATCGTAGACACTCAATTGAAAAATAATGAGGTCATTCTTAGT

GGAGAAATCCCTGCTCGGTGTATTCAAGAATATCGTAGTGATTTAACTTTCTTTACAAAT

GGACGTAGTGTTTGTTTAACAGAGTTAAAAGGGTACCATGTTACTACCGGTGAACCTGTT

TGCCAGCCCCGTCGTCCAAATAGTCGGATAGATAAAGTACGATATATGTTCAATAAAATA

ACTTAGTGTATTTTATGTTGTTATATAAATATGGTTTCTTGTTAAATAAGATGAAATATT

TTTTAATAAAGATTTGAATTAAAGTGTAAAGGAGGAGATAGTTATTATAAACTACAAGTG

GATATTGTGTGCTGAGAGCTTTCTTCTATACTAATAGACGAAAGGGTGTGAAAATGATTT

TTAAATGATACTGTGGAACGGAACAGTAGCCCTAGTATTGACTACTGTCGTTTCTATTCA

TATTGGCTATTCTAGGACTGAGATGAAAAAATCTATAAATGCTCAGAATAAAATTGAACC

CGCAAATCTCCCCAAAACAATGGTGAGTCATGTACTTGTATTATTCCGAAAAAATACACC

TCTGGTGCAGTGAGACAAATTGGTGTATCTTATAGTGGCTTCGTAGATGAAAGCTATACT

CTACTATCACTCTTTGATGATGTAGAACAAATTGAAAAAGATAATAGACTTCAGACAGCT

ATTGATGTTGTCAGAGAACAGTTTGGTTTTTTAGCCATACAAAAAGGAACCGTCCTAACT

GAAGGTTCCAGAAATATTGAACGCAGTAAACTTATCGGTGGTCATTCCGCGGGTGGATTG

GAGGGATTAAAATGAAACAAGAAAAAATACAGTACAATTTTCAGAAATCCGTAGCAAAGG

ATGTAATGATATTGAAATGCTTGAAAGATTTTTACATGGAATCGTTGAAACAGCAACTTC

AAAACTTCGTCAGAGAAAACTCAAAACAACTGAAATATCGATACGACTAGTACATGCTAA

ATCTGAAAACCGATTACCATTGGAATTTACATTTAGCATTAAGCCAACAAGCTCATCTGT

GATAATCTATACTGAGGTAATCAATCGCTTTAAAGAATGTTACACAGGTGGGGGAATTCA

AGGTTTTACGATTCAATTTGATAAAAATACCCTTGCCTCTGCATAGAAAGGATTTGATAT

GATTGACCGTTCATATTTACCATTTCAATCAGCAAGAGAGTACCAGGATACAAAGATGCA

AAAATGGATGGGCTTTTTCCTATCTGAACATGCATCAGCACTCTCTGATGATACAAACAA

AGTAACGTACATGTCTGACTTATCACTAGAGAAGAAATTATTACTCCTCAGTCAAGTATA

CGCCGGGCAGCTACGCACACGCATTCAAGTGATTGAAAAAAACAAGCGTGTTTCCTACAC

TGGAACAATACCAAGTCTGACCAAAGATTTCATTTTGATAAAAACTACAACAGGTCACAT

CAATTTGAAATTAAAAGACATTATTAGTATTGAACTTGTCGAGGAGGTGCTCTATGAATC

AGCTTGAGTTTCAGCGTAATCACCTACAAATGGACTATTATAGCGAGAGCTACCAAGATT

TTGAACGTGACTTCTACCGCTACTCTAACATGAATATTCCATTGACCTTCCTAACTGATG

ATATCCTAAAAACAATGGCGACTTCACGTAAGAATTACTTTGTCCTCAATAAGGAAAAGT

CCAGAGATAACCGCGATCACTTCTTCATATTTGAAGTAAGTACCGTAGATGAGAATCCGC

TAATCTATCATTATACATATAAGAAAACTACAATATATTTAGCAGAAAAATAGGAGCAGT

TCAATTGACTGTTCCTATTTTTAATATTCATAAAATCTAAAGTCTTTATACTCTTTAACA

ATGGAGTCGCCAACCAGAACAGACTATACTGACCAGCGACTACCTTAAATTTAATGTTTC

AGATTTATTTTCTTATCTCTAATTTCATAAACTACATCTGCTACATTTTCGAGTAATCGT

TTATCGTGGGTGATAAACACGATAGTTCCGGTGTACTCCTTCATTAGTATTTCCAAAGCC

TCTAAACTTGGTATGTCAAGGAAGTTACTGGGTTCATCCATTATTAGGATGTTATATCTA

CCCATGAGCATTTTAGCAAGCAACAATTTTATAATTTCTCCACCGCTTAAAACAGATAAA

CTTTTTCCAATATCGTTCTGTTTGAACCCCATAGATGCTAGCACTGAACGAATTTCTGAT

ATATTGTAGTCACAATCCTTCTGCATAAACTCCATAACATTCTGATTACTGTTGTACTTG

TAACCATTCTGTGCAAAGTAACCTATTTTTGCCTTAGGCGAAATAGAAATTCCTTCTTCA

TGGTTTAAGATCATTTGGATTAAAGTTGTTTTTCCGATTCCATTACCACCAGTTAACGCC

ACTTTTGCTCCTAACGGAATTTGAAAAGATGCATTTTCAAACAGAGCCTTATCCCCAAAT

ACTTTATTAATTTCTGCACCGACTATAGGGTATGGATTATGGAGCTCCAATGCTTTACTT

TGCCTGAAACGAATTCTGCGAATGCCTTCCGGAGCTTCTACTTTTCCTAAGGCCGCAATC

CTGTGCTCTAGGGTTTTAGCAGCATTATACATCTTTTTTTCCTTACTTCCTATTGATTTT

TGATGAGCTAAACGCCCTCCGTCTTCAGTACTTTTTTCTTTGAAGAACCTTTTGCCTTCT

GTTCTATTTTACGAGCCTGTTTTCGCTTTTCCTCCGCAGCCCTTTCCAATCGGGCACGTT

CCGCAATAAATTGTTCGTATTCTGCAGCTTGGCTCTTACGTTCTTCCTCTTTCTGACGAA

GATAATCAGAATAGTTTCCCCAATACTCAGTGATTTTGCCATCTTTCAGTTCCCATATTT

TATCTACTATTTCATCAAGAAAATAGCGGTCATGGCTAATAACTAACAGTGCACCTGTAA

AATATTTTAGCTGTCCTATTAGAAAATCAATTCCTTCACGGTCTAAATGGCTCGTAGGTT

CATCCGCTAAAATACCATGAACCTGTGCCGATAAGGCCTGTGCTATTTTAAGCCTTGTTT

CTTCACCACCGCTCATAGTCTGTATATTTAATTGCTCAACACCTAGCTTGCCTACAAGTG

CAAAATCTTTTTCCTCCTGCAGAGTTACTTCGTCCAACTGGGGAATATAGGCAAGTTCAC

CCAGACGATTCATTTTACATCCTGGGGGAGTTAATTCTCCTAAAAGTACCCTGAGTAAAG

TGCTTTTTCCAGCACCATTTGCTCCTACTAAACCAATACGGTCATAATCATATACTTCTA

ATTCATTTATATCTAAAACATCGCGTCCTTTGAATTCCACACGAATGTCTTTTGCTTTTA

ATATTAATTCCATAACATTTCCTCCTGTCTATAATCGCATGCTTTCATTTGCTTGTATGC

AGGGAAAACCCTGCGATTTTAGCAGGAAGAGTTACATGAAAATAAGATACATAAATATTC

CTCCAATATTGTTTATTTTAAATCTAATTTTCTAACCTCAGTTATCATTTGGCAAACTAT

AGCAATGCCAATAATTAAAATACCTGATAGTAAAAACCAATGATTTACACCGATTTTATC

AGCAAAGAATCCAGAAAGAATTAACCCAATTGGCATAGCAAGTGACATGATACTTCCGAT

CAAAGAAAATACACGTCCTAAATATTCAGGCTTAATTTTCTCCTGAAAAAGAGCTGTTTG

CACACCGCTATAAAATGGCACCGAAAGCCCCATTATTGCACAGCAAACTACGAATATTAC

AAATCCATTTGGAGGAAGTATTCCCGAAACGGCTAAACTGGTCCCCATTATAAAAAATGA

ACTTGTTATTAGTAATACATGCTTTTCGAAGCCCCCTAATCTTCCTAATAATAAGCCTCC

TGCTAGCATCCCAAATGCAAAGGAAATTTCCGTAATAGAAATATGCACAGGCGTTCCATT

AAAGTGTTCCATGCTTATTAAAGGAAATAGTGCATTGATTGGCATATAAACAAAAGTATA

TAGTGTTCCTAAGAGTAATAAGGCAAACAATCCTTTGTTTTGTCTCAGAACCACAACTCC

TTCTTTCATCTCCCTTATGAAATTTGGTTCTAAACTTTGCACTTGATTACCCAGCTTAGG

TATACGTACAATTGCTACCGTAATAGATGCAATCACAGCACCCAATACGTCGATGGCAAT

AATAGCATTTAAATCCCAAACGGAGTATAAGAGTGCTGCAACTGCCGGACTAACAATATA

GCTTATAGACTGCAAAGACTGACTATAGCCTGCGCATTTCGTTAGCTGTTCTTCTGGTAC

TAAAAGTGGTGTAACCGCATTGAGTGCTGGGGTATGAAAAGCTGTTCCAATGCTACGGAT

AAACAATACTATCATAATCATCCAGACAGGTAGCTCCATACAGAATGCAACAATAGCAAG

CACTGCACCAGCTGCTGCGATAATTAAATCGGCACCAATCATTATCTTCTTCCTATCATG

ACGATCCACTAGCACACCAATGGCAGGTCCCAAAATCGCATAGGGTAAAAAACCTACTAA

TGAAGCCATAGACAAGACCATCGCAGATCCTGTTTTTTCTGTAAGGTAAAAAATAATCGC

CATTTGCAGGATGGCACTAGTGATTAATGATACTGCTTGCCCTGCCCATATTGCATAAAA

TTTTCGTTTCCAATTGTTGTATTTTTCCATTTATATTATCTCCTGCATATTATTTTGCTT

GAATTTCTATTTTGAATAGCATTCTAGGCAATAAAAAATGCAGGCCAAACCCCACAATGT

GGCTTTTGGTCTGCATACATACAATTTGGAAACATTCATATTAAAGACATAGTTAAATAA

AGGTATAGTTAAATAACCAATATCCTCACCGTAACTAATGAATGCTCAATATCGTATAAA

TAAGCACAACAAAAAAGCCTATCATCGGGTATAGATTCTGCTTTTTTTATTGCCAGCTTA

TCTTAAACGCATTGAGGCTGTCATAGTTTCGGTTCCTCCTACATCTTTGTTTATATCAAT

TTATAGTATAACACAACAAGATGATATGTTCAATATAAAAGTTATGGAATGAGACTCATA

CTTCCAATTCGATGCCAGATTTAAAGGATATGACGAAGTTTTCTTCATAGACTGTAACGC

TCTGGATTATCTTCCTTAGTAGCAAGCGATTAGCTTTCACAAAATCTTCTGTTTGTAGTT

TTAAAAATTCATCAGGATTTTCTAACTCAACCTCAAAATATTTCATTTTACATTCCCTCA

TTTCATTTATTGATAAATTGAGTTTGCAAAAAAGAGTGGACAATTTTTGTCTACTCTTAA

CCTTTAAAATAGTTTTTTTTAATCGATTTGAAGTTGCCTAAATTATTACTTATTCGGTAA

AATGAAGTATTGCTTTCAACAGATTTCCTTCAACTACACTTCACTTGATTCAAACAAGGT

GGGTACATTTCTATTCCCACAAACTCCTTGTCAATGGAAACAAACACGTACCCACAGGGT

AAATGGAAATAGAAACTGATAATTTCTAGCTATCACTTCTACTCATTCCAAAAATTTTCT

CACTCTGATACTTACCCACCATAAAGCAAAAAGCCTTGCAATCAAGGCTTTCATTATCCC

TTTCGTTCAAAGGTTTCTAAGCTTTTACGAGCAGAGCGACACACTCAGCGGTTCGCTATC

TCCGTTCTGTCTGCGTGCTAGCACTTGTCAATCACGGACAGCTATCGCATGGGCGGAAGT

AAATGCTAATCTTCGTCGTTTTACTCCTTGACTAGCAAACTTACCGCCTCAACATGTCCT

GTATGTGGAAATAAAACACGATTAAAGATAAGGGAAGATACTGAATTAAAAAAATTCCCC

CTCTATTGTCCGAAATGCAGACAAGAAAATTTAATTGAAATAAAGCAGTTCAAAGTAACT

GTGATTACAGAGCCAGACGCAAAGACGCAGAGCCGATAAAATGAGATTAATACAATCTCA

TTTTATCGGCTCTTTCCGTTATGTATGGATTCTTTTAATTAGTCTTCGATGTTTCTTGCT

TCGTTGATACCGCTGGCTAAAGATTCCATTAAGGATAGTTCTTTGTCTGTAAAGCTATCC

ATGTATTTCTCTATCTGTAATCGTCGGGTGCTTTTTACCAAGTTATTAGCAGGTAAGAAA

AATTCATCAACGGAAACATGAAGTAACGATACAAGGTCATAAAGAACTTGTATGCTGGGG

TGTTGCCCTTTATTTTCAATATTAGTTAAGTACCGTGGGTCAATTTCAATCAATGCTCCC

ACTTGTTCACGAGTTAAACCTCGTTTCAATCGAGCTTCTTTAATGGCTAAACCAAAGGCT

CTAAAATCATATTTATCTTCTTTTTTACGCATAGTAGACCACCTCTATACATTTTATTGT

TCCTACTGAATTAAAAACAGGTATAGAAAAACGTGTTATATGGTTTATAGGTTTATATTT

AATAAAAAGCACTACTAAACGCCAATAAAAAAAACCGTTATATGGTAGTGCTATTTACGC

TGTTAAAATATTGTATATTACTTCCAAATGGCGGTTTGTTGGAGGTCAACGTCGCCATGA

AGTACATCATATACAATAAATTTCCTTACATTGGGTTCTTGTCAAAAAAAGTCGTCTATC

TGCAATAGATAAGTACGTCCACCAATGTGGTTTTATAAATCATATAGATAGAATAACAGA

AGCATGTAAACAGAGAAATAAATCTGTTTATATGCTTTTTTGGCTATTCAGAACTTTTTT

ACAAAGTTTATTTATCAGTAATGCAACAAATCCCCCTTTCACATTGGGACTAAGAGTGAA

AGGAGATAAACGAGCAAGGCTCACTTCCTTTCCTAGACAGAAAGGGGGTGAGAAACATGA

AACCATCTTCTTTTCAGACCACAATAGAAAATCAGTTTGACTATATCTGTAAACGTGCTA

TGGAAGACGAGCGAAAGAATTATATGCTTTATCTTTCAAGGATTGCAAAGCGTGAGGTGT

CCTTTTCGGATGTTGGCGATTATCTTGTTAGCCAGTTTGCGACAACAGATAACTATTCAA

CTGACTTTCAGATTTTTACACTCAATGGGTTATCAGTAGGCGTTGAAAATGATTTGTTGA

GTGAAGCATTACGTGAGTTGCCAGACAAGAAACGTGAAATTCTACTGCTGTTTTACTTTA

TGGACATGAGCGATTCAGAAATTGCAGACCTGTTGAAATTGAACCGTTCTACTGTCTATC

GGCATAGAACCAGTGGACTAGCCTTAATTAAAAAGTTTATGGAGGAATTTGAAGAATGAA

AACACAATATCCTATGATTCCCTTTCCTCTCATTGTAAAGGCAACAGATGGCGATACCGA

AGCGATTAACCAGATTCTACATCATTACAGAGGGTACATAACGAAGCGTTCCCTACGACT

TATGAAAGATGAATATGGCAATCAAAGTATGGTCGTTGATGAAGTCTTACGTGGAAGAAT

GGAAACCAGACTGATTACAAAGATTTTGTCATTTGAAATTAAGTAATATCCTCTCTCCTT

TCGTGGAAGCGTGCTAAACCATTCCACGCTTCCCGAACAGGGAGGTTTGTTATTCCACCA

AAGCATATTGAGCTTTCAATGTGTTTTGATAGGCTAACGAGCCATTGTTCTTTGAAAACT

GAATAAAAGTAATCGAATACGTTTCGATAAGAAAAGAGCCAACGGAACTAACCGCCATGA

CCTATCTTATAAAGATAGCGAGCGATTCATGTTAGTGATCCGAGAAGCAATCTTTAGCAG

GATTGCCTGCAACGACATTCTTATCGTGATAATGATACTCCCATACAGTCAATAGTCCGA

GCGTGATAAAACCGTCGCAGGCAATGAGTATGGCTACATGAGAACCATGCAGGGGTGGAA

CTCCCGTGAGCTTTGCTAAAGCTGTTCGATTGCTGGTAAAACAACTTTTATGAAATCCAA

ATAAGTGATTTGGAAAGGAGGATTTTATGAAGCAGACTGACATTCCTATTTGGGAACGTT

ATACCCTAACCATTGAAGAAGCGTCAAAATATTTTCGTATTGGCGAAAACAAGCTACGAC

GCTTGGCAGAGGAAAATAAAAATGCAAATTGGCTGATTATGAATGGCAATCGTATTCAGA

TTAAACGAAAACAATTTGAAAAAATTATAGATACATTGGACGCAATCTAGCGTCGCCAAA

GGGTCTTGTATATGATAAAATAGTATTAAGTCGTATCAAGGCTCTTTCCATAAAGGAAAG

GAGCAAATGCCATGTCAGAAAAAAGACGTGACAATAAAGGTCGAATCTTAAAGACTGGAG

AGAGCCAACGAAAAGACGGAAGATACTTATACAAATATATAGATTCATTTGGAGAACCGC

AATTTGTTTACTCGTGGAAACTTGTGGCTACAGACCGAGTACCAGCAGGAAAGCGTGATT

GTATCTCACTTAGAGAGAAAATCGCAGAGTTACAGAAAGACATTCATGATGGTATTGATG

TTGTAGGAAAGAAAATGACACTCTGCCAGCTTTACGCAAAACAGAACGCTCAAAGACCAA

AGGTTAGAAAAAACACTGAAACTGGACGCAAATATCTTATGGATATTTTGAAGAAAGACA

AGTTAGGTGTAAGAAGTATTGACAGTATTAAGCCATCAGACGCTAAAGAATGGGCTATTA

GAATGAGTGAAAATGGTTATGCTTATCAAACCATCAATAACTACAAACGTTCTTTAAAGG

CTTCATTCTATATTGCTATACAAGATGATTGTGTTCGGAAGAATCCATTTGACTTTCAAC

TGAAAGCAGTTCTTGATGATGATACTGTCCCTAAGACCGTACTAACAGAAGAACAGGAAG

AAAAACTGTTAGCCTTTGCAAAAGCTGATAAAACCTACAGCAAAAATTATGATGAAATTC

TGATACTCTTAAAAACAGGTCTTCGTATTTCAGAGTTTGGTGGTTTGACACTTCCAGATT

TAGATTTTGAGAATCGTCTTGTCAATATAGACCATCAGCTATTGAGAGATACTGAAATTG

GGTACTACATTGAAACACCAAAGACCAAAAGTGGCGAACGTCAAGTTCCTATGGTTGAAG

AAGCCTATCAAGCATTTAAGCGAGTGTTAGCGAATCGAAAGAATGATAAGCGTGTTGAGA

TTGATGGATATAGTGATTTCCTCTTTCTTAATAGAAAGAACTATCCAAAAGTGGCAAGTG

ATTACAACGGCATGATGAAAGGTCTTGTTAAGAAATACAATAAGTATAACGAGGATAAAT

TGCCACACATCACTCCACATAGTTTGCGACATACATTCTGTACCAACTATGCAAATGCAG

GAATGAATCCAAAGGCATTACAGTACATTATGGGACATGCTAATATAGCCATGACGCTGA

ACTATTACGCACATGCAACATTCGATTCTGCAATGGCAGAAATGAAACGCTTGAATAAAG

AGAAGCAACAGGAGCGTCTTGTTGCTTAGTAGTACAAATGAATTTACTACTTATTTACCA

CTTCTGACAGCTAAGACATGAGGAAATATGCAAAGAAACGTGAAGTATCTTCCTACAGTA

AAAATACTCGAAAGCACATAGAATAAGGCTTTACGAGCATTTAAGAAAATATAAAAAGAT

AATTAGAAATTTATACTTTGTTT

>GA40563/Tn6002

AAAATAGCATAAAAATCTAGTTATCCGTATAAACACTGGACTTATCACACTTTATCAAGG

TCGAAACCAATCAATTTACTACTAATTTACTACTTATGAATGAGCTTTGATACGACGATT

TACCCTTGAAAAGTGAAGAAACAAAGATACTTCCAATAAAAATTGAATAGGCAAAAGGTG

GACACTTCAAAAATGAGGTGTCTATTTTTTTACCCGATTTGAAAGGACGTGATACTACGA

AAATAGAAAAACAAGAGAATAAAGGTCGCTCCCCACCGTGAAAGACCATCAAACAAACGA

GATTCATTCAACCATAAAAAAGAAAGGATAGGTAAAAATATGGAACTTAAATTTGTGATT

CCCAAGATGGAAAAAACATTCGGCAATTTAGAATTTGCTGGCGAGGATAAAGTCGTACAG

CGAAGAATCAACGGACATCTAACCGTCTTATCTCGAAGCTATAATCTCTATTCAGACGTT

CAAAGAGCAGATGATATTGTGGTCGTACTTCCTGCTGAAGCTGGCGAAAAACATTTCGGC

TTTGAGGAACGTGTTAAGTTAGTCAATCCACGTATTACCGCCGAGGGCTATAAAATCGGC

ACTCGTGGTTTTACAAATTACCTTTTACATGCTGACGACATGGTAAAAGAATAAAGAAAG

AGAGGAAAAATGATGAGATTAGCAAATGGTATCGTATTAGATAAAGACACGACTTTTGGA

GAATTAAAATTCTCTGCTCTACGTCGTGAAGTGAGAATCCAAAATGAAGACGGTACGGTT

TCAGAGGAAATCAAAGAACGTACCTATGACTTAAAATCCAAAGGGCAAGGACGCATGATT

CAAGTAAGTATTCCTGCCAGCGTGCCTTTGAAAGAGTTTGAATATAACGCACGGGTGGAA

CTTATCAATCCCATTGCGGATACTGTCGCTACTGCTACCTTTCAAGGAGCAGATGTTGAC

TGGTACATCAAGGCAGACGATATTGTGCTGACAAAGGATTCTAATTCCTTTAGAAATCAA

CAGCCACCTAAGAAAGAACCTGCTACGGACAAATAGCCACGTATCTTCCATTAGAGAGAA

AGGAGAAAATCAAACATGAAACAGCGTGTCTTTCGTGGTAAAAGGATTCGTCCGAGTGAC

AAAGATTTAGTCTTTCATTTTACAGTAGCGTCCTTACTGCCTATTTTACTGCTTGTTGTC

GGACTGTTTCATGTGAAGACAATCCAGCAGGTCAACTGGCAGGACTTTAACCTATCACAA

GCAGATAAGATTGACATTCCGTATTTAAGTATCAGTTTCAGTGTCGCAATTCTTGTCTGC

TTGCTGGTGGCGTTTCTATTCAAACGGTATCGCTATGATACGATTAAACAACTCTACCAC

CGTCAAAAGCTGGCGAAGATGGTTCTTGAAAATAAGTGGTATGAATCAGAACAGGTCAAA

ACAGATGGCTTCTTCAAGGATTCCCCCAGTCGTACCAAAGAAAAGATAACCTACTTCCCT

AAAATCTATTATCGCCTTAAAAATGGCTTAATACAGATACAAGTGGAAATCACTCTGGGG

AAATATCAAGACCAGCTCCTACACTTGGAAAAGAAATTAGAAAGTGGCTTGTACTGTGAG

CTGACGGATAAAGAGTTAAAGGATTCCTACGTGGAATATACCTTGCTCTATGATATGATA

GCCCGTCGTATTTCTATTGATGAAGTAGAAGCTAAAGATGGTAAACTTCGCTTAATGAAA

AACGTATGGTGGGAATATGATAAGCTCCCTCATATGTTGATTGCTGGTGGTACAGGTGGC

GGTAAAACTTACTTTATACTGACACTGATTGAAGCCTTGCTTCATACAGATTCAAAACTG

TATATTCTTGACCCGAAAAATGCTGACCTTGCGGACTTAGGTTCTGTGATGGCAAATGTC

TACTATAGAAAAGAAGACTTGCTTTCTTGCATTGAAACATTCTATGAAGAAATGATGAAA

CGTAGTGAGGAAATGAAGCAGATGAAGAACTATAAGACTGGCAAAAATTATGCTTACTTA

GGTCTCCCGGCACACTTCTTAATCTTTGATGAATACGTCGCTTTCATGGAAATGCTGGGA

ACAAAAGAAAACACCGCAGTTATGAATAAGCTGAAACAGATTGTCATGTTAGGTCGTCAA

GCTGGCTTCTTTCTAATACTGGCTTGTCAACGTCCAGACGCAAAATATTTAGGCGACGGA

ATCCGTGATCAGTTTAATTTCAGAGTGGCTTTAGGTCGTATGTCTGAAATGGGCTATGGC

ATGATGTTTGGCAGTGACGTACAAAAGGATTTCTTCTTAAAGCGAATCAAAGGTCGTGGC

TATGTTGATGTAGGAACAAGTGTCATATCAGAGTTTTATACTCCCCTTGTACCAAAAGGA

TATGATTTCTTGGAGGAAATTAAAAAGTTATCCAACAGCAGACAGTCCACGCAGGCGACG

TGCGAAGCGGAAGTCGCAGGTGTGGACTGATCTTGCTGGCTGGTGTGGCAATAGCCACGC

CAGCACTTAACCCCCCGTATCTAACAGGGGGGTACAAATCGACAGGAAACAGTCAAAAAA

ACATTAGAAAATCCTTTGGTTACAAGGGATTTACAAAATTTCAGCGTATGTCAAATGGGC

TTTAAAAGTTGACATACGCCTTTTTGATTGGAGGGATTTTTACTGAATGAACAAACTTGG

TTACAGCATTTAAAAGAAAAACGCTTGGCTTATGGACTATCTCAAAACCGTTTAGCTGTT

GCGACTGGTATTACAAGGCAGTATCTAAGCGATATTGAAACAGGAAAAGTCAAGCCATCA

GAGGATTTACAGCAGTCCCTTTGGGAAGCTCTGGAACGCTTCAATCCCGACGCTCCCCTT

GAAATGCTGTTTGATTATGTAAGGATTCGCTTTCCCACAACGGACGTACAGCATGTGGTC

GAAAACATCTTACAACTGAAACTGTCCTATTTTCTTCATGAGGACTATGGTTTCTATTCT

TATTCAGAGCATTATGCTTTAGGCGATATATTCGTTCTCTGCTCCCACGAACTGGACAAA

GGAGTTCTGGTGGAATTGAAAGGTCGTGGGTGTCGGCAATTTGAAAGCTATCTTCTGGCT

CAACAAAGAAGCTGGTATGAGTTCTTTATGGACGCTTTGGTGGCTGGCGGTGTGATGAAA

CGCCTTGACCTTGCCATTAACGATAAGACAGGGATTTTGAATATCCCTGTACTCACTGAA

AAATGCAGACAGGAAGAATGTATATCCGTCTTCCGCAGTTTCAAAAGCTATCGCAGTGGC

GAACTGGTACGCAAAGATGAAAAGGAATGTATGGGAAATACCCTCTATATCGGTTCATTA

CAGAGTGAAGTTTATTTCTGTATCTATGAAAAGGACTATGAACAGTACAAGAAAAATGAT

ATTCCCATTGAAGACGCAGAAGTAAAAAACCGTTTTGAGATTCGATTGAAAAATGAGCGT

GCCTATTATGCAGTCCGTGATTTACTCGTCTATGACAATCCAGAGCATACCGCCTTTAAA

ATTATCAATCGGTATATCCGTTTTGTAGATAAAGACGATTCCAAACCTCGTTCTGATTGG

AAACTGAATGAAGAATGGGCTTGGTTTATTGGGAACAATCGTGAACGATTAAAACTAACC

ACAAAACCAGAGCCTTACTCCTTCCAAAGGACGCTGAACTGGCTATCTCATCAAGTTGCC

CCGACCTTAAAGGTTGCGATTAAACTTGATGAAATCAACCAGACGCAGGTTGTAAAAGAC

ATTCTCGACCATGCGAAACTGACAGACCGACACAAGCAGATTTTGAAGCAACAGTCAGTA

AAAGAACAGGACGTGATAACAACAAAAAAAGGATATCTGTCAACCATACCAGTTGACAGA

TATCCAAAAAAAGATATAATGGGAGATAAGACGGTTCGTGTTCGTGCTGACTTGCACCAT

ATCATAAAAATCGAAACAGCAAAGAATGGCGGAAACGTAAAAGAAGTTATGGAAATAAGA

CTTAGAAGCAAACTTAAGAGTGTGTTGATAGTGCATTATCTTAAAATTTTGTATAATAGG

AATTGAAGTTAAATTAGATGCTAAAAATTTGTAATTAAGAAGGAGGGATTCGTCATGTTG

GTATTCCAAATGCGTAATGTAGATAAAACATCTACTGTTTTGAAACAGACTAAAAACAGT

GATTACGCAGATAAATAAATACGTTAGATTAATTCCTACCAGTGACTAATCTTATGACTT

TTTAAACAGATAACTAAAATTACAAACAAATCGTTTAACTTCTGTATTTGTTTATAGATG

TAATCACTTCAGGAGAGATTACATGAACAAAAATATAAAATATTCTCAAAACTTTTTAAC

GAGTGAAAAAGTACTCAACCAAATAATAAAACAATTGAATTTAAAAGAAACCGATACCGT

TTACGAAATTGGAACAGGTAAAGGGCATTTAACGACGAAACTGGCTAAAATAAGTAAACA

GGTAACGTCTATTGAATTAGACAGTCATCTATTCAACTTATCGTCAGAAAAATTAAAACT

GAATACTCGTGTCACTTTAATTCACCAAGATATTCTACAGTTTCAATTCCCTAACAAACA

GAGGTATAAAATTGTTGGGAATATTCCTTACCATTTAAGCACACAAATTATTAAAAAAGT

GGTTTTTGAAAGCCGTGCGTCTGACATCTATCTGATTGTTGAAGAAGGATTCTACAAGCG

TACCTTGGATATTCACCGAACACTAGGGTTGCTCTTGCACACTCAAGTCTCGATTCAGCA

ATTGCTTAAGCTGCCAGCGGAATGCTTTCATCCTAAACCAAAAGTAAACAGTGTCTTAAT

AAAACTTACCCGCCATACCACAGATGTTCCAGATAAATATTGGAAGCTATATACGTACTT

TGTTTCAAAATGGGTCAATCGAGAATATCGTCAACTGTTTACTAAAAATCAGTTTCATCA

AGCAATGAAACACGCCAAAGTAAACAATTTAAGTACCATTACTTATGAGCAAGTATTGTC

TATTTTTAATAGTTATCTATTATTTAACGGGAGGAAATAATTCTATGAGTCGCTTTTTTA

AATTTGGAAAGTTACACGTTACTAAAGGGAATGGAGATAAATTATTAGATATACTACTGA

CAGCTTCCAAGAAGCTAAAGAGGTCCCTAGCGCCTACGGGGAATTTGTATCGATAAGGGG

TACAAATTCCCACTAAGCGCTCGGGACCCCTTGTAGGAAAATGTCCTAAGTGGGATATCT

GTCAACTGGTATGGTTGACTAAAAATACTTCCTACGAAAATGTAGGGGGTATTTTTTTAC

GAAAAAATACAATCGATTCTTAAAAAGAAAAATTTTTGATTGGCAAAACCATAACAAGTT

CGTTTTAGGGTTTTGATTTTGCGATTGATGCCTTCTAAAGGACCATTAGAGTATTCAAAT

TTAGCGCTATTTAAGACATATTTTCTGTTTTGACGAAGGGTTTGAATAGCAGTATCCATT

TCTGTATTGGTTTTTTGGTAGTCTAAGATGGTTGACTCTAGTAATTCACTATTGCGCTCG

TTTAGGGCTTTCGTGATATCTTGGTAAGTTTGGTATACTTCAGCGAACTTGGAAAATTTA

CTAGTAATGAGATCAACAGCATTTTGGCGAGTCATATATTGTTTAACGCCGCGAAGAAAA

ACTACTTCTTCAGGGTGAAGATCTTCAGCTTTTTTATGGAATAGCTTCCAATGTGACTTC

ATAATTTTATATTCTTGGCTCTGTTTATCAAGTTGCTTTAGGATAGAGATACGACAATTG

TCCAAAGCGCGACCAGCTAATTGTACAAGGTGGAAGCGATCAATAATGATATTGGCATTA

GGGAAAAGGCGATAGATAAAACTTTGATATTGAGCATTTAAATCAATTACAACTGATTGA

ACGCATTCGCGTTCGGCTTTTGAATAACGACTTTCAAAATAATCAACAATGGTAGGTGAT

AGACGATCCTGTAACTTTGTGACAATTTGGTGGGTTTCAGCGTCACAACAGATAAAGGAC

ATCACAGACTTAATTGAACGAAACTCGTCAAAACATAGATGCTTAGGCAACTTAGCCACA

CGATAGTGTGGTTCCATGCGCTCTAAGATTGTTCGACGAACACTGCTAGGAGAGCAGTGA

CACATTTCAGCAATAAGCTGACCAGATAAGCCTTTACGAGCTAAAAGCATGATTTGATTT

TTGAGATCACTGGATAAGGTTTGATTTTCTTTGGTTAAATTAGTAATAGCACCAAAAGTA

GTATGGCATGATTTACATTTATAGCGTTGTTTACGAAGCTCTAGTTCATATCTTCTCCCA

TTTAAACTTGCCAGTCGTACATGAGTTTTGCGAAAGCCATCCTTATTAACTGTGGGAAAG

CCACAGTTACGACAACGATTAATCGGATAAGAAAGAGTAGCTGTTATTAGCGTTATATAC

TCTTTAACAGAATCGTTGTTGTGTTCAGCTTCTTCAACAGAAATAATTTTAATATTTTTA

TCTTTAATTCCAAGAATATTTAGGATAGAATCATTATGGGACATTTGTTTAACCTTCTTT

CATGATTTTTGTGGTGAATTGATTGTATAACGAGGGGACAGCAAATGTCCTCTTTTTTGT

ATAAAAAAATCTGGCATGGAATCTCTATCCATACCAGAAAGTGTATACCCCAAAAAAATA

ACTCAAATACAAATTCATTGAATATAGAGAGGAGAACATTTTTATGAATTTTGGACAAAA

CCTTTATAACTGGTTTCTATCAAACGCTCAATCACTGGTGCTTTTAGCAATCGTTGTGAT

TGGCTTGTATCTTGGCTTCAAGCGTGAGTTTAGCAAACTGATTGGCTTTTTAATTATTGC

GATTATTGCGGTTGGCTTAGTCTTCAACGCTGCTGGAGTAAAAGACATTTTACTAGAGCT

ATTCAATCGCATTATTGGTGCTTAAATAAAACCGTTCTTTTGTGGAATATAAGTGGTTTT

CTTATGTTCCGCAAAGGAATGGTACACCAAACGAAGTGCGGTAGGGATTTTTGAATCTCT

ACAAAGAAAGGACGTGAATATATGGACGATATGCAAGTCTATATTGCGAATTTAGGCAAA

TACAATGAGGGCGAATTGGTCGGTGCGTGGTTTACCTTTCCCATTGACTTTGAGGAAGTC

AAAGAGAAAATCGGCTTGAATGATGAATATGAGGAATACGCCATTCATGACTACGAGTTA

CCCTTTACGGTTGACGAATACACTTCCATTGGCGAACTCAATCGACTATGGGAAATGGTA

TCGGAATTACCCGAAGAATTACAATCGGAGCTATCTGCTCTGCTCACTCATTTTTCAAGC

ATTGAAGAACTAAGCGAACATCAAGAGGATATTATCATTCATTCCGATTGTGATGATATG

TATGACGTGGCACGCTACTACATTGAAGAAACGGGTGCTTTAGGCGAAGTACCAGCTAGT

CTTCAAAACTATATTGATTATCAAGCCTATGGTCGGGATTTAGACCTTTCAGGAACGTTT

ATCTCAACCAATCATGGGATTTTTGAAATCGTCTATTAAATCTGTCGGTACATTACTACT

GGCAGATTTTCTATTTTACGGGGTGGCTCAATCAGCTACCCCTATTTTTTATGAAAGGAT

TGATTACATGAAGAAAATACGAAGCTATACCAGTATCTGGTCTGTGGAAAAGGTACTGTA

TTCTATCAATGATTTTAGACTTCCGTTTCCCATAACCTTTACGCAAATGACATGGTTTGT

CGTGTCACTCTTTGCAGTGATGATACTTGGCAACTTGCCCCCTCTTTCCATGATAGAGGG

AGCATTTCTCAAATACTTTGGGATTCCTGTGGCTTTCACATGGTTTATGTCTACAAAAAC

CTTTGATGGTAAAAAGCCTTATGGATTTTTGAAGTCTGTCATTGCTTATGCACTGCGACG

AAAACTGACCTATGCAGGAAAAAAAGTAACGCTTGGCAGAAACCAGCCACAAGAAGCCAT

TACAGCAGTTAGGAGTGAATTTTATGGCATATCCAATTAAATATATTGAAAACAATCTGG

TCTGGAATAAAGATGGGGAATGTTACGCTTACTATGAGCTTGTTCCCTACAATTACTCAT

TTCTAAGTCCGGAACAGAAAATACAAGTACATGATTCTTTCAGACAGCTTATCGCACAAA

ATCGTGATGGCAAGATTCATGCTTTACAAATCAGTACAGAATCCAGCATACTTTCTGCAC

AAGAGCGTTCCAAAAATGAAGTCACTGGAAAGCTCAAAACGGTTGCCTATGACAAAATCG

ACCAACAGACAGACGCTTTAATATCCATGATTGGCGAAAATCAAGTGGACTACCGTTTCT

TTATCGGTTTTAAGTTGCTTCTCAACGATCAGGAGTTTTCTATGAAAAGTCTTACCGTTG

AAGCAAAAAATGCTTTTACTGATTTTGTCTATGATGTGAACCATAAGCTGATGGGCGATT

TTGTTAGTATGAGTAATGATGAAATCCTGCGTTTTCAGAAGATGGAAAAGCTTTTAGAAA

ATAAAATCTCCCGTCGTTTCAAAATCCGAAGATTAGATAAGGACGACTTCGGCTATCTGA

TTGAACACCTTTACGGACAGACAGGCACTGCCTATGAAGAGTATGAGTATCCTCTATCAA

AGAAAAAGCTGGAACATGAAACACTGATTAAAACCTATGACCTCATTAAGCCTACTCGCT

GTCTGGTGGAAGAAAAACAGCGATATTTGAAAATCCAGCAGGAAGACGAAACCGCCTATG

TAGCTTACTTTACCATTAACAGCATTGTCGGCGAACTGGACTTCCCGTCCTCTGAAATCT

TCTACTACCAGCAACAGCAATTTACCTTTCCGATTGATACGTCAATGAATGTGGAAATTG

TAGCGAATCGTAAAGCCCTATCTACTGTCCGCAATAAAAAGAAAGAACTGAAAGACTTGG

ATAACCACGCATGGCAAAGTGATAATGAAACCAGCTCTAATGTGGCGGAAGCTCTGGAAA

GTGTGAACGAGCTGGAAACCAATTTAGACCAAAGCAAGGAATCTATGTATAAGCTGTCCT

ATGTTGTAAGGGTATCAGCAAATGACCTTGACGAACTTAAACGTCGTTGTAATGAAGTGA

AAGATTTCTATGATGATTTGAGCGTGAAACTGGTACGACCTTTTGGGGATATGCTGGGCT

TACATGAAGAATTTTTACCTGCCAGCAAAAGATATATGAATGACTATATTCAATACGTGA

CCTCTGATTTCCTCGCTGGTTTAGGTTTTGGTGCTACTCAAATGCTGGGTGAAAATGAGG

GGATTTATGTTGGCTACAGCTTAGATACTGGACGCAATGTCTATCTGAAACCTGCTCTTG

CCAGTCAAGGGGTTAAGGGTTCAGTAACCAATGCGTTAGCGTCTGCCTTTGTCGGTTCGC

TGGGTGGTGGTAAATCCTTTGCGAATAACCTTATCGTCTATTATGCAGTGCTTTATGGGG

CACAAGCAGTGATTGTAGACCCAAAAGCAGAACGTGGCAGATGGAAAGAAACCTTGCCAG

AGATTTCCCATGAAATCAATATCGTCAATCTGACTTCTGATGAGAAAAACAAAGGCTTAC

TTGACCCTTATGTAATTATGAAAAATACCAAAGATTCTGAATCACTGGCTATTGATATTT

TGACATTCCTTACGGGGATTTCCTCTCGTGATGGGGAACGCTTCCCAATCCTTAGAAAAG

CCATTCGTGCAGTAACCAATAGTGAAGTGCGAGGGTTGATGAAAGTGATTGAAGAATTAC

GGGTTGAGAATACGCCACTAAGTACCAGCATAGCCGACCATATCGAAAGTTTTACAGACT

ATGACTTTGCCCATCTGCTTTTTAGTGATGGTTATGTAGAGCAGTCTATCAGCCTTGAAA

AACAACTGAACATTATACAGGTTGCCGACTTGGTACTTCCCGACAAGGAAACTTCCTTTG

AGGAATATACCACTATGGAGCTTTTATCCGTTGCTATGCTGATTGTCATTAGTACCTTTG

CTTTAGACTTTATCCATACAGACCGAAGCATTTTCAAGATTGTAGATTTAGACGAAGCAT

GGAGCTTTTTACAGGTAGCACAAGGAAAAACACTATCTATGAAGCTGGTTCGGGCTGGTC

GTGCTATGAACGCTGGGGTATATTTCGTGACCCAAAATACAGACGACCTCTTAGATGAAA

AACTGAAAAATAACCTCGGCTTAAAATTTGCATTTCGTTCCACTGACCTTAACGAGATTA

AAAAGACCTTAGCCTTTTTTGGTGTAGACCCAGAGGACGAAAACAATCAGAAGCGATTGC

GTGATTTGGAAAACGGGCAATGCCTTATCAGTGATTTATATGGTCGTGTCGGTGTGATAC

AGTTCCACCCTGTATTTGAAGAACTGCTCCATGCCTTTGATACCAGACCACCTGTGCGAA

AAGAGGTGTAAATGTGAAACCATCAATAGTAAACAGAATAAAATCAAACTGGACGCTGAA

ACGTCTAGGTAAAGTGGCAATGACAGTGGCTTTCACACTTGTGATTGCCATTTTTCTTTT

AGCCATGCTGGGAACGGTGGTTCAAGCTGCGGGCTTGGTAGATGATACGGTCAATGTGGC

AAATGAATACAGCCGATACCCACTTGAAAACTATCAACTGGATTTTTATGTGGATAATAG

CTGGGGCTGGCTTCCGTGGAACTGGTCGGACGGGATTGGAAAACAGGTCATGTATGGACT

ATATGCCATTACCAATTTTATTTGGACAATCAGTTTGTATGTTTCCAATGCGACAGGTTA

CTTAGTACAGGAAGCCTATTCCTTAGACTTCATTTCCGCTACAGCAGATTCCATTGGTAA

GAATATGCAGACCTTAGCTGGTGTGAGTGCAAACGGATTTTCAACAGAGGGTTTCTATGT

TGGATTCCTCTTACTCTTGATTTTGGTTCTTGGGGTTTATGTTGCCTATACGGGACTGAT

AAAGAGAGAAACCACAAAGGCAATTCATGCCATTATGAATTTTGTGCTGGTGTTTATCCT

ATCGGCTTCCTTTATTGCCTACGCTCCCGACTACATTAAAAAAATCAATGACTTTTCATC

AGACATCAGTAATGCCAGTTTATCACTTGGCACGAAGATTGTCATGCCCCATTCCGATAG

TCAAGGCAAGGACAGCGTGGACTTAATCAGAGATAGCCTGTTTTCCATACAGGTTCAGCA

ACCGTGGCTACTGCTTCAATACAACAGTTCAGACATTGAAAGTATCGGTATTGACCGTGT

GGAAAGCCTGCTCTCCACCAGCCCAGATTCCAACAATGGCGAAGACAGAGAAAAAATTGT

TGCGGAAGAAATTGAAGACAGAAGCAATACCAATCTAACCATTACAAAGACCATTAACCG

TTTAGGTACAGTCTTCTTCCTATTTGTCTTCAATATTGGGATTTCCATATTTGTATTCCT

ATTAACAGGAATCATGATTTTCTCGCAGGTACTTTTTATCATCTATGCTATGTTTCTGCC

TGTGAGCTTTATTTTAAGCATGATTCCATCATTTGATGGTATGTCAAAACGAGCCATAAC

AAAGCTCTTTAATACCATTTTGACACGAGCTGGAATCACATTGATTATTACGACAGCATT

TAGTATTTCAACCATGCTCTATACCTTATCGGCTGGTTATCCGTTCTTTTTGATTGCTTT

TCTACAGATTGTGACCTTTGCAGGAATCTACTTCAAGCTGGGCGATTTAATGAGTATGTT

TTCTCTACAGAGTAACGATTCTCAAAGTGTGGGAAGTCGTGTGATGAGAAAACCTCGTAT

GCTTATGCACGCTCACATGCACCGTCTACAGCGGAAACTTGGACGTTCCATGACTACTCT

AGGGGCTGGGTCTGCCATTGTTACAGGTAAAAAAGGACAGTCGGGTTCGGGGAGTTCTGC

AAGGACACAAGCAGATCACTCCCGACCAGACGGAAAGGAAAAATCAACACTTGGAAAACG

TATCGGTCAAACCATCGGTACAGTAGCTGATACCAAAGACAGAATGGTAGACACTGCTAG

TGGTTTGAAAGAACAGGTTAAAGATTTGCCGACCAATGCAAGATATGCAGTATATCAAGG

AAAATCCAAAGTAAAAGAGAATGTCCGTGATTTAACCAGTAGTATTTCTCAAACCAAAGC

GGACAGAGCCAGTGGACGCAAGGAACAGCAGGAACAAAGGCAAAAAACCATTGCGAAGCG

TCGCTCTGAAATGGAACAGGTCAAACAGAAAAAACAGCCTGCTTCTTCTGTTCATGAAAG

ACCGACTACAAGACAAGAACAATATCATGATGAACAGACCTCAAAACAGTCTAATATTCA

GACTTCATATAAGGAATCTCAACAAGCCAAACAAGAGCGTCCAGCAGTTAAGTCCGATTT

TTCAAGTCCAAAAGTGGAACGCCAAGGCAATACCGTTCAAGAAAAACCGTTCAAAAGCCA

GCAACTTCAACCACTACAGCAGATAGAACTTCACAACGTCCAATCACAAAAGAACGTCCG

TCTACTGTTCAAAGAGTACCACTACAAAATACAAGAAGTAGACCACCAATCAAAACCGCC

ACCATTAAGAAAGTCGGTAAGAAACCATGAAGTTGAAAACTTTAGTGATTGGTGGTTCTG

GATTATTCTTGATGGTCTTCTCACTGCTTCTGTTTGTTGCCATTTTATTTTCAGATGAAC

AGGACAGCGGAATTTCCAATATTCATTATGGAGGTGTGAATGTTTCCGCAGAAGTGCTGG

CTCATAAGCCTATGGTAGAAAAATATGCCAAAGAATATGGCGTTGAAGAATATGTCAACA

TACTTCTTGCGATTATACAGGTGGAATCGGGCGGTACTGCGGAAGATGTTATGCAGTCCT

CGGAATCCCTCGGTCTTCCACCTAATTCATTGAGTACAGAAGAATCCATTAAGCAAGGTG

TGAAGTATTTCAGTGAATTATTAGCCAGTAGCGAAAGGCTCAGTGTAGATTTAGAATCGG

TTATCCAGTCCTACAATTATGGTGGTGGTTTCTTAGGGTATGTGGCTAATCGTGGAAATA

AATATACCTTTGAACTGGCTCAAAGTTTCTCAAAAGAGTATTCAGGTGGCGAAAAAGTGT

CTTACCCCAATCCCATAGCCATACCTATCAATGGGGGCTGGCGATACAACTATGGCAATA

TGTTTTATGTGCAACTGGTAACGCAGTATCTTGTCACAACAGAGTTTGATGATGATACGG

TACAAGCCATCATGGACGAAGCACTGAAATATGAGGGCTGGCGATACGTTTACGGTGGAG

CTTCCCCGACTACTTCTTTTGATTGTAGCGGACTGACACAATGGACGTATGGAAAAGCTG

GAATTAACTTACCACGAACCGCACAACAGCAATATGATGTGACCCAGCATATCCCACTAT

CGGAAGCACAAGCTGGCGATTTGGTTTTCTTTCATTCTACCTATAACGCTGGCTCTTATA

TTACTCATGTTGGGATATACCTTGGCAATAACCGTATGTTTCATGCAGGCGACCCAATCG

GTTATGCCGACTTAACAAGCCCCTACTGGCAACAGCATTTAGTGGGAGCAGGACGAATCA

AACAATGAGAAAGGAAGATTTAATGATGAAATTTAGAAAAAATCAGAATAAAGAAAAACA

GATACCAAAGGAAAAGAAACCTCGTGTCTATAAGGTCAATCCTCATAAAAAGGTTGTGAT

TGCCTTGTGGGTACTTTTAGGGCTTAGTTTCAGCTTTGCGATATTCAAGCACTTTACAGC

TATAGATACTCATACTATTCACGAAACAACTATCATAGAAAAGGAATACGTTGATACTCA

TCATGTAGAAAATTTTGTAGAGAACTTTGCGAAAGTCTACTATTCATGGGAGCAATCCGA

TAAGTCCATTGATAATCGAATGGAAAGTCTAAAAGGCTATCTGACAGATGAACTTCAAGC

TCTCAATGTTGATACAGTACGCAAAGATATTCCTGTATCGTCTTCTGTAAGAGGATTTCA

GATATGGACGGTAGAGCCAACTGGCGACAATGAGTTTAATGTAACCTACAGTGTAGACCA

GCTCATTACAGAGGGAGAAAATACAAAGACCGTCCACTCTGCTTATATAGTGAGTGTCTA

TGTAGATGGTTCTGGAAATATGGTACTGGTTAAGAATCCGACCATTACCAACATACCTAA

GAAATCAAGTTATAAACCAAAAGCCATTGAAAGTGAGGGGACGGTTGATTCCATTACAAC

CAATGAAATCAATGAGTTTTTAACGACGTTCTTCAAGCTCTATCCTACAGCGACAGCCAG

TGAACTTTCCTACTATGTGAATGACGGGATATTAAAACCAATCGGAAAAGAGTACATCTT

TCAAGAACTGGTAAATCCTATTCACAATCGTAAGGATAATCAAGTCACGGTATCGCTGAC

AGTGGAGTATATCGACCAGCAGACCAAAGCAACGCAGGTATCTCAATTTGATTTGGTACT

TGAAAAGAACGGGAGTAATTGGAAGATTATAGAATAACAAATATTGGTACATTATTACAG

CTATTTTGTAATCACGTACTCTCTTTGATAAAAAATTGGAGATTCCTTTACAAATATGCT

CTTACGTGCTATTATTTAAGTATCTATTTAAAAGGAGTTAATAAATATGCGGCAAGGTAT

TCTTAAATAAACTGTCAATTTGATAGTGAGAACAAATAATTGGATGTCCTTTTTTAGGAG

GGCTTAGTTTTTTGTACCCAGTTTAAGAATACCTTTATCATGTGATTCTAAAGTATCCGG

AGAATATCTGTATGCTTTGTATGCCTATGGTTATGCATAAAAATTCCCAGTGNNNNNCAC

ACACTTAATTAATTAAGTGTGTGNNNNNNGATTTTTATGCCCTTTTGGGTTTTTGAATGG

AGGAAAATCACATGAAAATTATTAATATTGGAGTTTTAGCTCATGTTGATGCAGGAAAAA

CTACCTTAACAGAAAGCTTATTATATAACAGTGGAGCGATTACAGAATTAGGAAGCGTGG

ACAAAGGTACAACGAGGACGGATAATACGCTTTTAGAACGTCAGAGAGGAATTACAATTC

AGACAGGAATAACCTCTTTTCAGTGGGAAAATACGAAGGTGAACATCATAGACACGCCAG

GACATATGGATTTCTTAGCAGAAGTATATCGTTCATTATCAGTTTTAGATGGGGCAATTC

TACTGATTTCTGCAAAAGATGGCGTACAAGCACAAACTCGTATATTATTTCATGCACTTA

GGAAAATGGGGATTCCCACAATCTTTTTTATCAATAAGATTGACCAAAATGGAATTGATT

TATCAACGGTTTATCAGGATATTAAAGAGAAACTTTCTGCCGAAATTGTAATCAAACAGA

AGGTAGAACTGTATCCTAATATGTGTGTGACGAACTTTACCGAATCTGAACAATGGGATA

CGGTAATAGAGGGAAACGATGACCTTTTAGAGAAATATATGTCCGGTAAATCATTAGAAG

CATTGGAACTCGAACAAGAGGAAAGCATAAGATTTCAGAATTGTTCTCTGTTCCCTCTTT

ATCATGGAAGTGCAAAAAGTAATATAGGGATTGATAACCTTATAGAAGTGATTACGAATA

AATTTTATTCATCAACACATCGAGGTCAGTCTGAACTTTGCGGAAAAGTTTTCAAAATTG

AGTATTCGGAAAAAAGACAGCGTCTTGCATATATACGTCTTTATAGTGGCGTACTGCATT

TGCGAGATTCGGTTAGAATATCGGAAAAGGAAAAAATAAAAATTACAGAAATGTATACTT

CAATAAATGGTGAATTATGTAAAATCGATAAGGCTTATTCCGGGGAAATTGTTATTTTGC

AGAATGAGTTTTTGAAGTTAAATAGTGTTCTTGGAGATACAAAGCTATTGCCACAGAGAG

AGAGAATTGAAAATCCCCTCCCTCTGCTGCAAACGACTGTTGAACCGAGCAAACCTCAAC

AAAGGGAAATGTTACTTGATGCACTTTTAGAAATCTCCGACAGTGACCCGCTTCTGCGAT

ATTATGTGGATTCTGCGACACATGAAATCATACTTTCTTTCTTAGGGAAAGTACAAATGG

AAGTGACTTGTGCTCTGCTGCAAGAAAAGTATCATGTGGAGATAGAAATAAAAGAGCCTA

CAGTCATTTATATGGAAAGACCGTTAAAAAAAGCAGAGTATACCATTCACATCGAAGTTC

CACCGAATCCTTTCTGGGCTTCCATTGGTCTATCTGTAGCACAGCTTCCATTAGGGAGCG

GAGTACAGTATGAGAGCTCGGTTTCTCTTGGATACTTAAATCAATCGTTTCAAAATGCAG

TTATGGAGGGGATACGCTATGGCTGTGAACAAGGATTGTATGGTTGGAATGTGACGGACT

GTAAAATCTGTTTTAAGTATGGCTTATACTATAGCCCTGTTAGTACCCCAGCAGATTTTC

GGATGCTTGCTCCTATTGTATTGGAACAAGTCTTAAAAAAAGCTGGAACAGAATTGTTAG

AGCCATATCTTAGTTTTAAAATTTATGCGCCACAGGAATATCTTTCACGAGCATACAACG

ATGCTCCTAAATATTGTGCGAACATCGTAGACACTCAATTGAAAAATAATGAGGTCATTA

TTATTGGAGAAATTCCTGCTCGATGTATTCAAGATTATCGCAATGATTTAACTTTCTTTA

CAAATGGACGTAGTGTTTGTTTAACAGAGTTAAAAGGGTACCATGTTACTACCGGTGAAC

CTGTTTGCCAGCCCCGTCGTCCAAATAGTCGGATAGATAAAGTACGATATATGTTCAATA

AAATAACTTAGTGTATTTTATGTTGTTATATAAATATGGTTTCTTGTTAAATAAGATGAA

ATATTTTTTAATAAAGATTTGAATTAAAGTGTAAAGGAGGAGATAGTTATTATAAACTAC

AAGTGGATATTGTGTCCTGTATGTGGAAATAAAACACGATTAAAGATAAGGGAAGATACT

GAATTAAAAAAATTCCCCCTCTATTGTCCGAAATGCAGACAAGAAAATTTAATTGAAATA

AAGCAGTTCAAAGTAACTGTGATTACAGAGCCAGACGCAAAGACGCAGAGCCGATAAAAT

GAGATTAATACAATCTCATTTTATCGGCTCTTTCCGTTATGTATGGATTCTTTTAATTAG

TCTTCGATGTTTCTTGCTTCGTTGATACCGCTGGCTAAAGATTCCATTAAGGATAGTTCT

TTGTCTGTAAAGCTATCCATGTATTTCTCTATCTGTAATCGTCGGGTGCTTTTTACCAAG

TTATTAGCAGGTAAGAAAAATTCATCAACGGAAACATGAAGTAACGATACAAGGTCATAA

AGAACTTGTATGCTGGGGTGTTGCCCTTTATTTTCAATATTAGTTAAGTACCGTGGGTCA

ATTTCAATCAATGCTCCCACTTGTTCACGAGTTAAACCTCGTTTCAATCGAGCTTCTTTA

ATGGCTAAACCAAAGGCTCTAAAATCATATTTATCTTCTTTTTTACGCATAATAGACCAC

CTCTATACATTTTACTGTTCCTATTGAATTAGAAACAGGTATAGAAAAACATGTTATATA

GTTTATAGGTTCATATTTAATAAAAAGCACTACTAAACGCCAATAAAAAAAACCGTTATA

TGGTAGTGCTATTTATGCTGTTAAAATATTGTATCTTACTTCCAAATGGCGGTTTGTTGG

AGGTCAAAGTCGCCATGAAGTATATCACATACAATCAAGTTCCCCACATTGAGTATTTAT

CAAAAAAAGTCGTCTATCTGCAATAGATAAGTACGTCCACCAATGTGGTTTTATAAATCA

TATAGATAGAAAAATAAAAGCATGTAAACAGAGAAATCAATCTGTTTGTGTGCTTTTTTG

GTTATTCAGAACTTTTTTACAAAGTTTATTCATCAGTAATGCAACAAATCCCCCTTTCAC

ATTGGGACTAAGAGTGAAAGGAGATAAAAGAGCAAGGCTCACTTCCTTTCCTAGACAGAA

AGGGGGTGAGAAACATGAAACCATCTTTTTTTCAGACCACAATAGAAAATCAGTTTGACT

ATATCTGTAAACGTGCTATGGAAGACGAGCGAAAGAATTATCTGCTTTATCTTTCAAGGA

TCGCAAAGCGTGAAGTGTCCTTTTCTGATGTTGGCGATTATCTTGTTAGCCAGTTTGCGA

CAACAGATAACTATTCAACTGACTTTCAGATTTTTACACTCAATGGAATATCAGTTGGTG

TTGAAAATGATTTATTGAGTGAAGCATTACGTGAGTTGCCAGACAAGAAACGTGAAATTC

TACTGCTGTTTTACTTTATGGACATGAGCGATTCAGAAATTGCAGACCTGTTGAAATTGA

ACCGTTCTACTGTCTATCGGCATAGAACCAGTGGACTAGCCTTAATCAAAAAGTTTATGG

AGGAATTTGAAGAATGAAAACACAATATCCTATGATTCCCTTTCCTCTCATTGTAAAGGC

AACAGATGGCGATACCGAAGCGATTAACCAGATTCTACATCATTACAGAGGGTACATAAC

GAAGCGTTCCCTACGACTTATGAAAGATGAATATGGCAATCAAAGTATGGTCGTTGATGA

AGTCTTACGTGGAAGAATGGAAACCAGACTGATTACAAAGATTTTGTCATTTGAAATTAA

GTAATATCCTCTCTCCTTTCGTGGAAGCGTGCTAAACCATTCCACGCTTCCCGAACAGGG

AGGTTTGTTATTCCACCAAAGCATATTGAGCTTTCAATGTGTTTTGATAGGCTAACGAGC

CATTGTTCTTTGAAAACTGAATAAAAGTAATCGAATACGTTTCGATAAGAAAAGAGCCAA

CGGAACTAACCGCCATGACCTATCTTCTAAAGATAGCGAGCGTTTCAGTTAGTGTTCCGA

AAAACAATCTTTAGCAGGATTGCCAGCGACGACTTTCTTATCGTGATAATGATACTCCCA

TACAGTCAATAGTCCGAGCGTTAAAAGCGTCGCAGGCAATGAGTATGGCTACATGAGAAC

CATGCAGGGGTGGAACTCCCGTGAGCTTTGCTAGAGCTGTTCGATTGCTTGTAAAACAAC

TTTTATGAAATCCAATAAGTGATTTGGAAAGGAGGATTTTATGAAGCAGACTGACATTCC

GATTTGGGAGCGTTATACCCTAACTATTGAAGAAGCGTCAAAATATTTTCGTATTGGCGA

AAACAAGCTGCGTCGTTTGGCAGAAGAAAATAAAAATGCAAACTGGCTGATTATGAATGG

CAATCGTATTCAGGTTAAACGAAAACAATTTGAAAAAATTATAGATACATTGAACGCAAT

CTAGCGTAGCCAAAGGGTCTTGTATATGATAAAATAGTATTAAGTCGTATCAGGGCTCTT

TCCATAATGGAAAGGAGCAAATGCCATGTCAGAAAAAAGACGTGACAATAAAGGTCGAAT

TTTAAAGACTGGAGAGAGCCAACGAAAAGACGGAAGATACTTATACAAATATACAGATTC

ATTTGGAGAACCGCAATTTGTTTACTCGTGGAAACTTGTGGCTACAGACAGAGTACCAGC

AGGAAAGCGTGATTGTATCTCACTTAGGGAGAAAATCGCAGAGTTACAGAAAGACATTCA

TGATGGTATTGATGTTGTAGGAAAGAAAATGACACTCTGCCAGCTTTACGCAAAACAGAA

CGCTCAAAGACCAAAGGTTAGAAAAAATACTGAAACTGGACGCAAATATCTTATGGATAT

TTTGAAGAAAGACAAGTTAGGTGCAAGAAGTATTGATAGTATTAAACCATCAGACGCTAA

AGAAAGGGCGATTAGAATGAGTGAAAATGGTTATGCCTATCAAACCATCAATAACTATAA

ACGTTCTTTAAAGGCTTCATTCTACATTGCGATACAAGATGATTGTGTTCGGAAGAATCC

ATTTGACTTTCAACTGAATGCAGTTCTTGATGATGATACTGTCCCTAAGACCGTACTAAC

AGGAGAACAGGAAGAAAAACTGTTAGCCTTTGCGAAAGCTGATAAAACCTACAGCAAAAA

TTATGATGAAATTCTGATACTCTTAAAAACAGGTCTTCGTATTTCAGAGTTTGGTGGTTT

GACACTTCCAGATTTAGATTTTGAGAATCGTCTTGTCAATATAGACCATCAGCTATTGAG

AGATACTGAAATTGGGTACTACATTGAAACACCAAAGACCAAAAGTGGTGAACGTCAAGT

TCCTATGGTTGAAGAAGCCTATCAAGCATTTAAGCGAGTGTTAGCGAATCGAAAGAATGA

TAAGCGTGTTGAGATTGATGGATATAGTGATTTCCTCTTTCTTAATAGAAAGAACTATCC

AAAAGTGGCGAGTGACTATAATGGTATGATGAAAGGTCTTGTTAAGAAATACAACAAGTA

TAATGATGATAAGTTACCACACATCACTCCACATAGTTTGCGACATACATTCTGTACCAA

CTATGCAAATGCAGGAATGAACCCAAAAGCATTACAATACATTATGGGGCATGCAAATAT

AGCCATGACGCTGAACTATTACGCACACGCAACATTTGATTCCGCAATGTCAGAGATGAA

ACGCTTGAATAAAGAGAAGCAACAGGAGCGTCTTGTTGCTTAGTAGTACAAATGAATTTA

CTACTTATTTACCACTTCTGACAGCTAAGACATGAGGAAATATGCAAAGAAACGTGAAGT

ATCTTCCTACAGTAAAAATACTCGAAAGCACATAGAATAAGGCTTTACGAGCATTTAAGA

AAATATAAAAAGATAATTAGAAATTTATACTTTGTTT

>GA47210/Tn2009

AAAATAGCATAAAAATCTAGTTATCCGCATAAAAACTGGACTTATCACACTTTATCAAGG

TCAAAACCACTCAATTTACTACTAATTTACTACTTATGAATGAGCTTTGATACGACGATT

TATCCTTGAAAAGTGAAGATATAAAGATACTTCCAATAAAATTTGAATATTTAATAGGTA

GACACTTCAAAAAATGAGGTGTCTATTTTTTTACCCGATTTTGAAAGGAAGTGAACTTAT

GAAAACAAAAAATCAAGAATCAAAAGGTCGTTCCCCACTCTTTAAGACCATCAAACATTC

ATTCAGCCAATAAAAAAGAAAGGATAGGTAAAAATATGGAACTTAAATTTGTGATTCCCA

ACATGGAAAAAACATTCGGCAATTTAGAATTTGCTGGCGAGGATAAAGTCGTTCAGCGAA

GAATCAACGGACGGCTAACTGTCTTATCAAGAAGCTATAATCTCTATTCTGATGTTCAAA

GAGCAGATGATATTGTGGTGGTGCTTCCTGCTGAAGCTGGCGAAAAACATTTCGGCTTTG

AGGAACGTGTGAAGTTAGTCAATCCACGTATTACCGCAGAGGGCTACAAAATCGGCACTC

GTGGTTTTACAAATTACCTTTTACATGCTGACGACATGATAAAAGAATAAAGAAAGAGAG

GAAAAATGATGAGATTAGCAAATGGCATTGTATTAGATAAAGACACGACTTTTGGAGAAT

TGAAATTCTCTGCTCTACGTCGTGAAGTGAGAATCCAAAATGAAGACGGGTCGGTTTCAG

ATGAAATCAAGGAACGTACCTATGACTTAAAATCCAAAGGACAAGGACGCATGATTCAAG

TAAGTATTCCTGCCAGCGTGCCTTTGAAAGAGTTTGATTATAACGCACGGGTGGAACTTA

TCAATCCCATTGCGGACACCGTTGCTACTGCCACCTATCAAGGAGCAGATGTTGACTGGT

ATATCAAGGCAGACGATATTGTGCTGACAAAGGATTCTAGTTCATTCAAAGCTCAACCAC

AAGCAAAGAAAGAACCGACACAAGACAAATAGTCGCTAGGTAGAAAGGAGACTTTTTCGC

ATGAAACAGCGTGGTAAAAGGATTCGCCCATCTGGTAAAGATTTAGTCTTTCATTTTACG

ATAGCGTCACTCCTGCCTGTTTTCCTGCTGGTTGTCGGACTGTTTCATGTGAAGACAATC

CAGCAGATCAACTGGCAGGATTTTAACCTATCACAAGCAGATAAGATTGACATTCCCTAT

TTAATTATCAGTTTCAGTGTCGCAATTCTTATCTGCTTGCTGGTAGCGTTTGTATTCAAA

CGGGTTCGCTATGATACGGTTAAACAACTTTACCACCGTCAAAAACTGGCAAAGATGATA

CTTGAAAACAAGTGGTATGAATCTGAACAGGTCAAAACAGAGGGTTTCTTTAAAGATAGT

GCTGGTCGTACAAAGGAAAAGATAACCTACTTCCCTAAAATGTATTATCGACTTAAAAAT

GGCTTGATACAGATACGGGTGGAAATCACGCTGGGAAAATATCAAGACCAACTCTTACAC

TTGGAAAAGAAATTAGAGAGTGGCTTGTACTGTGAGCTGACGGATAAAGAGTTAAAGGAT

TCCTATGTGGAATATACTTTGCTCTATGACACCATAGCCAGTCGTATTTCTATTGATGAA

GTAGAAGCTAAAGATGGTAAACTTCGCTTAATGAAAAACGTATGGTGGGAATATGATAAG

CTCCCTCATATGTTGATTGCTGGTGGTACAGGTGGCGGTAAAACTTACTTTATACTGACA

CTGATTGAAGCCTTGCTTCATACAGATTCAAAACTGTATATTCTTGACCCGAAAAATGCT

GACCTTGCGGACTTAGGTTCTGTGATGGCAAATGTCTACTATAGAAAAGAAGACTTGCTT

TCTTGCATTGAAACATTCTATGAAGAAATGATGAAACGTAGTGAGGAAATGAAGCAGATG

AAGAACTATAAGACTGGCAAAAATTATGCTTACTTAGGTCTCCCGGCACACTTCTTAATC

TTTGATGAATACGTCGCTTTCATGGAAATGCTGGGAACAAAAGAAAACACCGCAGTTATG

AATAAGCTGAAACAGATTGTCATGTTAGGTCGTCAAGCTGGCTTCTTTCTAATACTGGCT

TGTCAACGTCCAGACGCAAAATATTTAGGCGACGGAATCCGTGATCAGTTTAATTTCAGA

GTGGCTTTAGGTCGTATGTCTGAAATGGGCTATGGCATGATGTTTGGCAGTGACGTACAA

AAGGATTTCTTCTTAAAGCGAATCAAAGGTCGTGGCTATGTTGATGTAGGAACAAGTGTC

ATATCAGAGTTTTATACTCCCCTTGTACCAAAAGGATATGATTTCTTGGAGGAAATTAAA

AAGTTATCCAACAGCAGACAGTCCACGCAGGCGACGTGCGAAGCGGAAGTCGCAGGTGTG

GACTGATCTTGCTGGCTGGTGTGGCAATAGCCACGCCAGCACTTAACCCCCCGTATCTAA

CAGGGGGGTACAAATCGACAGGAAACAGTCAAAAAAACATTAGAAAATCCTTTGGTTACA

AGGGATTTACAAAATTTCAGCGTATGTCAAATGGGCTTTAAAAGTTGACATACGCCTTTT

TGATTGGAGGGATTTTTACTGAATGAACAAACTTGGTTACAGCATTTAAAAGAAAAACGC

TTGGCTTATGGACTATCTCAAAACCGTTTAGCTGTTGCGACTGGTATTACAAGGCAGTAT

CTAAGCGATATTGAAACAGGAAAAGTCAAGCCATCAGAGGATTTACAGCAGTCCCTTTGG

GAAGCTCTGGAACGCTTCAATCCCGACGCTCCCCTTGAAATGCTGTTTGATTATGTAAGG

ATTCGCTTTCCGACAACAGACGTACAGCAGGTGGTCGAAAACATCTTACAACTGAAACTG

TCCTATTTTCTTCATGAGGACTATGGTTTCTATTCTTATTCAGAGCATTATGCTTTAGGC

GACATATTCGTCCTTTGCTCCCATGAACTGGACAAAGGAGTTCTGGTGGAATTGAAAGGT

CGTGGGTGCAGACAATTTGAAAGCTATCTTCTGGCACAACAAAGAAGCTGGTATGAGTTC

TTTATGGACGTTTTGGTGGCTGGCGGTGTGATGAAACGCCTTGACCTTGCCATTAACGAT

AAGACAGGGATTTTGAATATCCCTGTACTCACTGAAAAGTGCCAACAGGAAGAATGTATC

TCCGTCTTCCGCAGTTTTAAAAGCTATCGCAGTGGCGAACTGGTACGCAAAGAGGAAAAG

GAATGTATGGGAAACACCCTCTATATCGGTTCATTACAAAGTGAAGTTTATTTCTGTATC

TATGAAAAGGACTACGAGCAGTACAAGAAAAATGATATTCCCATTGAAGACGCAGAAGTA

AAAAACCGTTTTGAGATTCGATTGAAAAATGAGCGTGCCTATTATGCAGTCCGTGATTTA

CTCGTCTATGACAATCCAGAGCATACCGCCTTTAAAATTATCAATCGGTATATCCGTTTT

GTAGATAAAGACGATTCCAAACCTCGTTCTGATTGGAAACTGAATGAAGAATGGGCTTGG

TTTATTGGGAACAATCGTGAACGATTAAAACTAACCACAAAACCAGAGCCTTACTCCTTC

CAAAGGACGCTGAACTGGCTATCTCATCAAGTTGCCCCGACCTTAAAGGTTGCGATTAAA

CTTGATGAAATCAACCAGACGCAGGTTGTAAAAGACATTCTCGACCATGCGAAACTGACA

GACCGACACAAGCAGATTTTGAAGCAACAGTCAGTAAAAGAACAGGACGTGATAACAACA

AAAAAATAACTCAAATACAAATTCATTGAATATAGAGAGGAGAACATTTTTATGAATTTT

GGACAAAACCTTTATAACTGGTTTCTATCAAACGCTCAATCACTGGTGCTTTTAGCAATC

GTTGTGATTGGCTTGTATCTTGGCTTCAAGCGTGAGTTTAGCAAACTGATTGGCTTTTTA

ATTATTGCGATTATTGCGGTTGGCTTAGTCTTCAACGCTGCTGGAGTAAAAGACATTTTA

CTAGAGCTATTCAATCGCATTATTGGTGCTTAAATAAAACCGTTCTTTTGTGGAATATAA

GTGGTTTTCTTATGTTCCGCAAAGGAATGGTACACCAAACGAAGTGCGGTAGGGATTTTT

GAATCTCTACAAAGAAAGGACGTGAATATATGGACGATATGCAAGTCTATATTGCGAATT

TAGGCAAATACAATGAGGGCGAATTGGTCGGTGCGTTGTTTACCTTTCCCATTGACTTTG

AGGAAGTCAAAGAGAAAATCGGCTTGAATGATGAATATGAGGAATACGCCATTCATGACT

ACGAGTTACCCTTTACGGTTGACGAATACACTTCCATTGGCGAACTCAATCGACTATGGG
[truncated: 757,077 more chars]
