# Supplementary material for: Composite mobile genetic elements disseminating macrolide resistance in Streptococcus pneumoniae
Source: Front Microbiol. 2015 Feb 9;6:26. doi: 10.3389/fmicb.2015.00026 (PMC4321634; doi:10.3389/fmicb.2015.00026)
Supplement: Supplementary file 1 [file DataSheet1.ZIP › Supplementary Material/Data File S4.DOCX]

>ICESpnGA11304

AACCATTGAATAGTAGCCATTGAGTTTTTTCCTTTCGTAGCAAGGGTTTAGAGTCCCTAT

TTTATTTTACTATTGTCTAAACACCAAGCGAACACCAAAACTACCATGCAATGGAAAAAC

CTCTGATTTGATTCTCGCTTGATTTCACAATCTTTATATCAAACTGTGGGTGGTATTTGA

CAATATCTTTTTTGATTTTTAATAGTAAATTTGAAACAATATTTTTAGGTGAGTAACGTG

GACTAAGATGTAACAAGTCTTTGAACTCATCAACACTTAATTCTACTTTATTGCTATTAT

CACTAGTTTCAATGAATTTTTCAATCATTCTGGAATATTTACAGGTATAACTTTTCAATT

CTTCAAAATGGAAATTGTGATTTTCTACAAATTGATTTAAGGCTTTTACAGTATTTTCTT

GTGAACGATTTATATTATGTGTATAGCCCATTGTTGTCTCAAAGTTAGCATGTCCTACTC

TAGTCATAATATCTTTCACTGCTATGTGCATCTCATTGCTTTGAAGGTAACTAATATGCA

TATGCCTAAACGAATGGGGAGTAACATGTTTTACCCACTTAAAACCATAGTCACTTAAAC

AATTTGTCAATAATTTTCCTTCTATTCGTTTCAAAATTTGACGAAAAGTGCTTGATGTTA

TTGGAGAGCCGTATTCTGTTCTAAATACACTTTCAGAATGTGTAAAAGCAGGACAGGGAT

GTTTCTTCATATAATCATCAAACTCTTTATTTCTCTGTATTGTCCTTTTAATAGCTTCGC

TTGCAGCTTCAGGCAAAGCTACTTCTCTAATTGAATTGAGTGTTTTAGTTGTATCAAAAT

GAAATTGTTTAACTTTTAAACAATGATATTGAAGTGCTTTATCAATATGCAAGATTCCTT

TTTCAAAATCAATATCTGATGGTAAAAATGCTGCTTCACTAATTCGAATACCTGTAAGCA

ACAATACTATAGCAAGATCATAATAGTTTGCATTTCTGCATTGGCGTAACACATCAAAAA

ATGCATGTAATTCATGGATTTCTAGAAATTTAGAATCATGTCTTTCTTTTGCTTTACGCC

TTTTCTCTAGTGAAATATCTAGTTTTACCGCAGTCATTGGAGAAAACTTAATGACATTAT

ATAACACACCATGATTAAAAATCTTATTACAAGTACTTTTTATATGAGTCATTGTTGAAG

GCGATGCATCATACATTTCTAAATATTTATTGAGACTATTTTTCATCAGAAGTGGAGTAA

TCCTGTCTAACAAAAAATCATCTCCTATAATTTTCCCAAGACGCTTCATAACCAGTAGTT

CTCTCTGAATTGTTTGTGGTTTAACAGAGACACACCAAGTCTGAAACCAATTTTCTTTTA

ACTCTCCAAATGTTGTAATCAGTTCAGGACTATACTGACTTTCAAATGAAGTAGTTAGTC

TATCTATTTTATCAAGAACCTCTCTTTCAGCTTGTTTCCTCGCCCTACTAGTATTCTTAG

TATAACTTACAGTTACTGATTTCCACTTTCCTGTTAGTGGATCTTTGTACTTTTCAACCA

CTTGATATAAGGGCTGCCCTTTTGAATTTGTTTTAGTTACATAATACATAATTTAATTCC

TCTTAACTCTTAAAAAGAAATAAATCATTGTTGTATAGAATAAATAACTACCCTATATTA

TACCATGACTTCCTTATTTTGTCCGCTTTTGCTTCCATCTTAAGAAACTTTCAAATCCTT

CTAGATTAATGAAAACAAGTTTATGGGTTGGGTTAATTACATACATGGAAAATGTTCTGT

TTTCTCGCATTTCTTTAATCCATCTGTTCAATGTATATTGATTTAAACCATCCCATCTTT

TTAGAATTCCCTTTTTATCGGCCCATTCGGCAATGGAATTTTCGACAGATATATATTTTA

TATTCATATCATCATCCTTCCTAAAAAATATTTTCACCTCCATGGCATGACTGATGAAGT

CATCATAAGAATTTCACTTGCTGCTCTTTTACGGTGGCAGCCTGAACGGTCAGAAGTATC

ATTGTATATCATTTGTATCATGGCATATAAAGTATCGCTCTATTTTATTGATGGTTTTAA

TATCGCTCTTATCATGGCGAACCATTCAAAACTGCTCCACAAATGAGGAAAGTACCATTT

TGGACTATTCAATTGTCAATGTGCTTTCTTAACCTACTACTTTTTGGAGTAATGGTCTTC

TTTATTATCCACTCGACCTACAAGGTAATCTAAACTTACATTATAAAAATCTGCAAGTTT

GATAAGGTCATCTATAGAGATTAACCTGGTACCTGATTCCATTTTAGAATACGCTGATCT

TGTACAATTTAAGATTGTTTTTGCAACATATTCTTGTGTCAAATCATCATCCTCGCGTAA

ATCTCTAATCCTTTTCAACATTTGTTGTCTCCTAAAACAAGTATAATATAGTTATTTTCT

TATTTTTAAATATGTGACAAATTGGCACATGAAACTATTCTATATATACTTTTTAGGAAA

CTGTTTACTCTAAATGTATCTCATTTTCCTCAATAGATTTCATATTTTGTTTTTTCTTAT

ATTTCTTTATTGTTTTATTAAACATTTCAAATAAATTTATTGCTTCTCTATGTTGTTCCT

TCAGTTTTTCAATCTTTCCTTTATAAATGTATAAATCCTTCTCTATTTTATCTACTGAAC

TTTTATCTTGAATACTATTTTCTAAATTGCTAGAACTATTTGTCGTATTATTCAACAATA

CTTCCTCTAAATTAGTTAAATCATCAATTTTATTTTGGACTTGTTCAATCATATTCTCTA

GATATGATATTTGGGCAATGAAATCATTTGTTATATCTTCAAAATCATTCGGACTATTTT

CTGCAGACATAAGAAAATCTAATTGTTCTATTTTTTCTTTAATTTTGCTAAGAGAAATTC

TTCTATACATATACTGCTGTTCATACTGAAGATTAAATTGTCTAATCAAAGTTTTACCTT

TCATAAAGCGATTTTTTTCTGCTAAATCTTTATGATATACATAGTAAGAACTAGTTTCTC

TGAGAAATACTTTAACTTTTTCTTCTTCCATATTGATTTGAATATTTGGTACAAAAATAA

GTCCTTCTTGTCGGATACCAAACTGTACCTTAATGTAAATTCCATCGTCTACTACTTCTT

CTATTTGATTAAGATTCAACTCTACTTCAAACTCATGAACAGCATCCCTATTTCTCTTGA

AATCTTGATAGGATTTCCATACCATATCTTCTGTCGGCAACTTTTTTTCTTTAATTAGCT

TTTCTTCATTGTAAAGTTCAACTAAATTTTTATTATCTAATCCGAAAGTATCATTTTTGT

TATTAAAATAGTCTTGAAAATAACTAACACTATACTGATTCGATTTTACTAATTCCTGTT

CTGAAAACTTAATCCCATCAAACTCAAATAAAACATGTTTTGCTTTCAGAATTATTTTTA

AGCCAAAAAATTCTGCTTGTTGAATCAATTCATTCATATTCTTCATTTTTGGAAGCAAAA

ATTCTAAGATGTTTATGATTTCCCTTTGAACAAACTTTTTCTTAAAATATGTTTCATTAT

AAGGTTGTTTTCTATTCAATTTATTATCACGTACGACTTGTTTCATATTTGAATCAGTCA

TAAAAAAAGTAACATGCTTGTGTCTAAAATCAATTTTTAAATGTAAATCTTTTGCTTTTT

TCTTGAAATCTTCAAAATTTTTCGAGTTCTCGATTAGAAAATATACTCGTTGTTTTATTT

CATATTTGTAATTTGTTTTGCGATAAACTTCATACTGATGATGAGAATAACGATTTTCTA

TAATTTTTGCGCCTACAATTTTTGAAAGACGATCTGAAACCATTCTTAGATTACGTTCTG

CTTTATAATCCCATAGAAATTTTTTATCAGAATTCTTATCAATTGAATTTAGGATGATGT

GATTGTGGATATGATCTTTATCGACATGAGTTGCTACAATAAAACGAAATCTACCACCTG

TCAACTCTTTAGCTGTCTCATAACCAATTCGATTGATTTGTTCAGGAGTGAGATGGTCAT

CTGGAGAAAAGGACTGAATGATATGATGGGCATGAATTTTTCGTTGCTTTTCCTCTAACC

TAGCATGCCGAAAATTATAAAGCGTATCATTGCTGATAAAATTTTCATGATACATCTGCA

CCATCTCATCATAGCTAGGAAAATCCAGAAAATTTCTCATGCCATAGTCCGATACCAATG

CAAGATTCTTGGTCTTATCAGGATTAAGAATATACTTGATAATTTTTCTGCGATAACTCT

TTCCGTGAATGGCAAAATGCTTAGTGATGACCATAAAACTCCCTCAACTTTTTTGCTTGA

ACACTAAACTCTTTCTCTACCTCACTTATCAAGTCAGCGATTCCCTTTTTCAAGTCTTGC

AATTCTTCACTTGAAATCAACTGAGATTGATTAACACTTCGGGCAATCTGGTTGATGTTA

TTACCAATCCTCTTCAACTCAAATATCAAATCTTGATAACCAGTTGTATCAATGGTGATG

AAATTCATACCAGGGTCAAGTAGGGTTCGTCGAGCATATTCTGAAAAAGATTGACAACCG

CTTTGATCGATGCAATGTTTCAACTGACTCAATTCGCTATCAGATAGGAAAACTTTCTTT

AGCTTAGTTCGGTAACGATTTTCCATGTGCCTACCTCATGTATTTTTACGGAAGTCTTGA

CTAAGAGGAATGGAGTTCCCTATTTCTTGAATCAATTCCTGAACACAGGTTAAGAGAATG

GATACATGTTCTTGGGTGACTTGATGTTCTGCCCTTGATAAGATTAAAATCTCATAAACA

TCACGACTGATTTGTTCCAGCTTTTGAGAATGCCAGAAGGTGAACCACTTTTCAGCAGTC

ATTTCTAACTCATTCTCCAGTAACTTGTTTCTGGCAAATGAAGAAAAGTGTTCAAATCCT

TTATTTTTCATCATTATGTCAATCATTTCTAATTCATTTGCAGTCAAATTAACCTCTTTG

CGAATGTCTCTGTATTGTTCTGGCACACTACTTCCTCACTTTCATCGATTGTCGGATAAT

TCCCGACCCCGTGTCAACCACGGGAAACTTATAAAAACAAAATATAGCATCTTGTAAGGC

TTTGCGAGCTCAGATATTGTGTCCACAATATCCCAAAAATCATATCGCCAGCTGACTAAA

ACCTTCCAGTTTTGACAGCTAACGATAAGATAACTTGGTGGCTCCCGCCCCCAAACCCCC

ATAGAAAATCAAAAATTGATTTTCTATGAATGATATTAGGATAACAGGGAAGATTTGGAA

AAGATATCACAGCTAATATAGTTATGATTGACTTTCTCTGATTCTATGATAAAATTTCTG

TAAACTAATATTTGGAGAAATAAAAATGCTGAGTCTAGATCAAATACATTTACTATTGAA

TACTCCTGAAGATGAGTTTCATGATTTTAAACAAAAATGGCATCATTCTAAAACTGAATT

GGTGCGTGATATCTTAAATTTTGTCAATACATCACATCATGAAGATTGTTATATCATCTT

TGGAATTGATAATATCACTTTAGATATAATCGGTGTAAACAATGATGATAATAGAAGAAA

TGAAGAAGATCTAACAGATTTACTACATAAACTCTTTATATCAACAAATAATCAAATTAG

AATTAGCATACAAACTGAAACTATAGACAACAAAGAAATTGACATCTTAATTATTCATGA

TACAGATAAAGTTCCTGTATTTTTAACAAAAGATTATAAGCCTAAGAAAGATACTGCATT

ACAAAAAGGATTAATATATGCTAGAAATGGCTCTATAAATACTCCTAAAGACTCCTCTGC

TCCATTTGAGTTAATAAATAAGTTGTTTCAAAAGTTTAATCATACCGACTTAAATATCAA

AGAGCAGTATTTTCATGTTTTAAAAGACTATAAAAATTGGTTCTTCGTTGAAAATGAAGA

CGGAAGATTTTTTATTTATAATCCTAATCCCGATTTTTATATTAAACTTACCGACGATGA

TGCAAATCGTTTCAAAACTATGCCTTATAGTTTAAATCAATACCAAACCAATGTTGATTG

GCAATTAGTACAACTTCGTTATCGACACCTTACAATTATCGACTTTATGGCTTTATATTT

AGATCAGGGAAATTGTCTAGTGCCCTCCCCTGATTTAGAAGATTTTGAATGTGGTTATTC

TGACACTATTTATTACCACTGTTTATACAAAAATACTTTAAAATACCAATTGCTGAAAGT

TTTCTCTTCAATATCTGGTCTCGAAAAATATCCTTTAGATAGATTTAAAAATAATATAGT

AATTTATGATGATAAAATAGAATTGGAAAAGACTCACAACTTAATAAAAAGTAATTTTTC

AACAGAAGAGATAATGAAGCAACTTGAAGTTACAGAAAAAGATTTCGATTTTTACTATAA

AAAAGCTAGACATAAAAATCCTGATTATAGTATCCAAGAAAATCAAGTAAATCTAACTGA

ATTAAACTTAGTTAGGTTATTGAAAAGTTTTCAAAAAACATATCTTGATAACCCACTATA

ATCAATCTTTTTAACTAGTAAAAAAGATAAAACAAGGCATAGAAATTAGTTTATCTAAAA

ACTAATTTCTATGCCTTAATCATTATTAATTAATAACATCAACTAAATTGGATTGAGTCA

GCAATGAATATATCTCATTAATATTTCTATTATTTTGCGGACTATTATTTCTCAATTTAC

AATATGTGAATATTAAGTGACTTTTTGCTCTTGAAAGTGCGACAAAGAATGCACTTTTAT

CTTCTTCCGGCTGATTATTAAAACTCCAGAATGCAGAATCCTCCAAGCCTAGAAAATAGA

CTACCTCATATTCTAATCCTTTACTCTTATGGATAGTCATAATTGGAATTGAGTTTTCAC

CCTTGAAACTAGAGACTGTATCTAGCCATTCACCTTGTGTTTGAGAATATTCTATGTATA

ATAACTTTGAAAAGTTCTTGACAATGATATCTAGGTCACTTTTTCCATTGTATGTTGAAA

AATTGGAAATAATTCTTTTTTCATCTATTTTTTCAATAATACAATCAATTAATTTTAGCA

TACTTTCTTCATTGGGAATAAAATTTGAAATTAGGTAAGTAATATCACTAACTATATTGT

CAATCTCCTTATAGGATTTTGCTAAAATTAATTCATCAGTTAACTCATCTATTCCATTGA

TATTTCCATAAAAATTACTAATATTTTCCCAAATTAAAGGATCTCGTTTTCCTTGACTGC

ATGATATTAAATCTAATAATAGGTTACACGTAGGATCTTTTAAAATATCCTGATATTCAT

TTTCAATTCTTGCTTTAATCCCTTTGCTATTTAATATAGATATCAATTCGAAACTATAAA

TACCAACCTTTTGTTTTGCTAGGATACAAATTTCTGATGGTCGTATACCTCCTTGAATTT

TTAATTCTATATCATTTGCAATTAACTTAGCTTCTAAACTTTCATTTTCAAATTCAAATA

ATGTTATTTCACCCTCTTGAAATTCTGGATAATTATTTGTCTGAATAGAACTGTGATTAC

TATTTAATATCTGATGAACCTCTTTTTGAAATTCTACAAGCTTAGGTACAGAGCGGTGAT

TCATCAACAATTGATATTCATTTGGATTAAAGTCTCGAATATAGTCTGGAAAAATATCAG

GCTTTGCACCTGCCCATCTCATAATGGCTTGCTTATCATCTCCAACTGCTGTTAATTTAC

AAGACGAACCTAAAAAACAAGTTTTTAATAAATCATACTGGGCATATGTAGTATCCTGAA

ATTCATCTAGAAATACAAAATCGTATGTCATTTGAAGTGCTTTACGGATGTATTCGTTAG

TATCTATTATTTGAGTACTCAACTTTGTTATTTGCCTATACAAGAGGACCGGCTTATTAT

CTTGTGTTCCTTTTAGTAAGTCAGTTTTAAACTTGTGATTATCCCCATTGTTAAGTATGA

TGTTTTCGACATATCTTCTTATATCTGACATTCTCCATCCATTAACATTAATTCCATTCA

TAGAGAGTAGTTCTTTTATAGTATACCAATCTTCAATCAAATAATCTCTTGATGGTCGTA

TATCCTCAGGTAGAACATCTCTAAATTGGTCTAAAATTCTCTTTTCAAAAGCCGAATAGG

TTAATGAGGTAAAACGAGAAGCATATTCATCACCATAACGTTTCTTAACTCTTTCTTTCA

AATTTGATGCAGCATCCGTTTTGAAACTTAATGCTAGAATTTTTTTCGGAGAAACGCATT

TATTTGTAGAGAATAGATAGTCTAACTTTTGAGCTAGTAATTCAGTTTTCCCAGCACCTG

GTCCAGCAATGACTAAACAGTTAGTCACATCTTTGACGGCTCCTAATGCAGTCTCTTCTA

AAATGATATCTCCTTTAGGAAACCACTCCTCACTCTTTACCATGTTGAATATCTCCAAGT

AATTCTTCAGCACTGTTAACTATTTTTTCAAATACAGGGGGTAGATTTCTTGTTAATTCA

TCATCACTAATAGATGATAAAAATTGCATATGAGTTGTTGGTTTTCCTCTACCTAAAAAG

AAATATTGATACCAAATCATTAATTCTTTTTCTTCTTCATTGAAACTATCGCCATGTCCA

CTCTTATCTTTTAAAGTAGCTCTTATTGCTTCCTCTTTACGTTTGTTGAAACCTTCTAAC

TGCAATCTATCAAGACAGTCTAAATCACTCAATTTCACTTTTTTACTACTTCCATCCGAA

TCATTATATGAAACAACTGGTCCTTCTATTGGGGATAAAGTATTCAGATAATTGTCTTTA

TAATGTTGTAGCATCAAGAAATCAATATCTAAAGGCGATGAAAAGTAGACATTAAATTCT

TCGAGTTTATTAAACCAATTAATCAGACGTTGAGCTCCTATCGATTCACAACCTCGTGAC

CCAATTTCATTAAAATCAAGTCTCTGAGTATGGAACCATTCTTGAAACTCTGTGTTCAGC

TCATACAGATGTTGAGAAATGTATTTTATTCTTCCCCAACCACCTCCGTATCTCTCATTA

TCAAAATCCAACAAGGTAATATGAGGAATTCTGAGAGCATTCAATAATTTCCAAAAATAG

TTGACATGTCTGCCCCCTAAAGGGACAATTGAAATCTGAGAGCTATCTACCTCTTTCCCA

AGTAAATCAAAAAACTTTGGTAATAGCAGTTCCTCACTGTCGCCTTCACCTAGAACTACT

AACTTCGCAAAATATAGTTCAGGATAAGCTTGAATTGCTCCTTTAATATATTTATAAGAT

TCATCTATAGCTGGAGGTAGCTGTATATCAGAAACAATGGTTTGAAGAACTCTGTCATTA

TTTTCTATTCTTAAATACTTCAAATCTTCTGGGTCAATTCTTTTTACAATAGCTGGAGAA

TGTGACGTTAAAATTACTTGAGAGTTGTCATTATTGCCTAATTGCTTAAACCTTTTTATC

AGTTTTCCAATGTGATGTGGAGCAATATGATTTTCTGGTTCTTCTATTGCTAGGATAGTT

AGTACTGGTGGAATCAACTTAAATCTTGGATTATCAGGATTTTCTTCACGGTCTTTGGTA

ATTTCTAACTCAATATCAAGTATCGAATCAACAAGTGAAAAATAAAAATAGATCTCAGTC

CATCTCCTAGATCTGAAACTGTAAATGCTTCTTCAGTGGTTGTTGGTGAAAATTTTAAGG

CAATCTGTCTAAGTGCTGCCGCCATCTCAGAAGAATTAATAATCAACTCTGCTTGAGAAA

AACGGTTATCTTCATGATATAATTCCCATGACTTCTGAATCTCATTGTTGATCTGAGTCA

ATGCTCCATTTTCAGATAAAAAGGTGTTATTCAACTCATCAATTTTATCTGTAATTTCAT

TTATCTCATCTTCGGTCCAATTTATACTATTTACCAACCTACTTAACATACTACCTGAAG

CATTACCCAACTCCTTCTCAGGCGTTCTTGAAGCAGGTACATAAAGTACCCTAATTTTAT

CCAAATCCTTTCTAGGAGCACGATGTTTGTCTTCATCTCTAATAGTATCTTCATCAGATG

AAATATAATAGAATTGAGTATCAATACTTCCTTCAACTGTTCCATCATCTTCCCAAGAAG

ATTCTAACCTGATTCGTAGAAATGGTTTGGCACCATCTTTAGAAACTGTAAAGTGTTCGA

AAAACGATGGAATTGCTTGACTATAGGGAGTTCCATCAAGTTCATCAAACTCAAAAATAG

TTTCTATAAAGAGGTTTCTAGTATTTTCTCCGGGCCTTGAACCTTTTGGAAGGTGAAAAT

CACTTTTTTTTATTATTCTATCATTTTGTTTGTCCGAAAACAGCTTACTCAATGCTTGTA

ATACAGTTGTTTTTCCCGAACTATTATTCCCAATCAGTACAGTTTGATTGTTTAATTCAA

TAATTTGACTTTCACCGAATGATCTAAAATTATTAATAATTACTTTTGTTAACTTCATTT

CTTATCTCCATTACCTGAAACTAATTCACACAACCATTCCTGTCCCACCTTCTACATCTC

TTCCTCAACCCTACTCTATTACCCAAAAAGGCATTATTGAAGAGTGACAGAAGCTTAACC

TTGATCTATTTTTGAAACATAGACACATCTTCTATGAATTCCTAGCAAATAGTTGAAATA

GTACTTGTTCCGTTTTTATAACAGCTAAGGGCTATTGCGTAAAATTTATACAATTCGAGC

AAATCTGTTTTTGTCAGGCCATGTGTCTTTCGTAGAATTTTATATTGTTACCAATTATAG

CATGCTCTTTTTACTTGATAACACTATTATAACATTTAAAAATAAAAACAGATTCTGAAC

TAATAGAAAATATAATTCTAAAATTAGAATTTTTCTTGATAATCATATAAAATGCTACAT

TTCTAATAAACTTGTACTTATTTCCTTCAAACTACACAATTCTCGAGTTGTTGTAAGTTT

GTAATTGACTCAAAAGTATTAGATATAATTTCAAAACAGTATTTTACTTTATAGTTGACC

AATAACATTTAATTCTAACTAAAAAACCATTATACAAATCAAGCTACTAAAAATGACTTT

ATGGTATACTAGTGATAATAAATTGTTATCGGGAGGAAAGTTGAGAAATGACCAGTTGTT

TTGTAGTTATGGGGTTTAATACTAAAAAAATTCCTAATACAAATATAGAAGTGGACTTGA

ATCAAGTCTATAATAATCTAATTAAGCCAACAATTTTAGAAAAAGAATTAGTTTCTGTTC

ATGGTAAAAACCACTTTAGAGCTGATGAAGTTTTCAGTACACAGAGTATAACGAAAACAT

TTATCGAAGGTATATTAAAAGCAGATATTGTAATTGCAGATATTACTACTTTAAACCAAA

ATGCTATCTATGAGTTAGGTCTACGTCATGCTATGAAACCCAAATCAACCATTATACTGT

GTGATCATCACACAGCTAAGATTAGCTTTTTTGATATTGCACACTTACCTCAAATCAGAT

ATGATTCTGATAAGTTAAACGAAGTGGATGAAGTTAACAAGGTGAAAAAACTGCTTTCTG

AATATATTGATTCCGCTATAAAGTCAGATGAGACATTTACTGATAGTCCAGCCTTTGAAT

CTGCTCTATACCGTGTAATAATTAACGATTTAATCGATAAAACTGAAATTCAAAGTGAAG

AAGCATTAGACAAGTCAATAGCTGAACTTTATGATCAAGCCACGGAACTCAAGAATTCAG

AAAAGTATCCTGAGGCAGAAGAAATATTTCAGCAAATTTTAGAAAGTGGATATATTGATG

AAGAAATTTTGGCAGGCTATTTACTATCATCCTATAAGAAAAATGAATCTTCGCTAGCAA

ATTTAAAAATGGCACAGCAAAATATTAGTAAATATATAGATATAAACACCACAACATATC

ATAAGTTATTAGGTATTTATGGAGCTATTTTCTTACGTATATTTTATATTACAAAGAATA

GGAGTGACTTAATGTCTGCAATTAATTACTATAGGTTAGGCATGAATTTTGAAGATAGAA

ATATTTATTGTGCAAGGAATTACTGTGCTAACCTGTTAAAAATAGCCTTAGTTGAGAAAG

ATGTTGAAGTTTTAAAGGAGTTCTACTATACATCTGTTTATACAGCAAAAACTATTTTAG

GATCTTTAGAAAAAATACATCGTAAATCTAGTGAATATGACGACATTTGGTTTCTTTCCA

ATCAAGAGGATCTAATGCTGATTTCAGGCCTTCTTTCAAAACCTATTAATGATATTGAGG

GTTTAACTGAACGACAGAAAAAAACAATTAATGAAGGTAGTATTATGTTAAGTGAGGATC

TCCAGAGGATTAAAACTGCAATTGTAAATGGTAATTAATAGTTTATTTCCCCTCCTTTGA

TAAAGTTATACTTATCATCAAGAAGGTTTTTCCATACATGAAAACTTTTTTGAAACCCTT

CTGATTGCAAGTACATTAATATATTTTCTTTTGTTTCTAATGAGAATTCAGGTGATGTTA

TAGAATATTCTCCCTTCTTTAAAATCCCTTCTTCAGTTAAACTTTCTTGAGGAATAGATA

GCATTGGAAGATTATAAGTTAACGCTAAAATCCCTTCAATATGACAATAAACACTTGTAA

CCCACTTATTCTTTAAGTGGGTCTCTTTGGATGCAAAGAAGTCTGGATTATCCTCAGCTC

CCATTTTTCTGGTTCCACTAGAAATAAATGTCTGACCAAATGCAACAATTATAATTCCGT

AACTCCTTTGTATCATCTCACTCAACGAATTCATGACTTCGTAAGGAGTATACTCAGCAG

CTTGTAATGTTACACATTCAATATTGTGTCGTCTTAAAAATTTAATCAATTCTTTAATAA

ATAAATTTTGATTCTCTGTAAATGGATTAGGTCTGCTTAAAAATACTCTTATTTTTTATT

TGTCATATTCGCTATCTCCTACCGATAACAATTTATTATCACTATACAAAGCACTCAAAT

AGGTTTATAATAGCATGATTATTGATAAAGGAGATTTTTATGGATTATAAGCTTATTTCT

ACTTACTTAGATTATTGCAAAACTCATAAGCGTTTGAGTTCACACACGATTCGAGCTTAT

AAGAATGATCTTATGCAATTTTATAATTCAAACTATGATAATGTCGAATCCTATATCGAA

GAGTTGACACAATCTAACATAAAAACGAATACATTAAGAAGAAAAATTGCTTGTATAAAG

GTGTTTTATAACTATCTAAAATACCAGCACATAATTGAAGAGAATCCCTTCAATCAATTA

CGCTTTCAATTTAGAACTGAAAAAATATTGCCTAAAACGATTCCGTATGATATTCTGAAA

AATATTTTTTCATATTTAGAGCAGAGAGTAGTTATATCTAAAACTGACTATCAAAAACAA

AAAGCTGAAAGAAATCTACTAATTATTTCCCTATTACTTTCAACAGGTATCAGAATTTCT

GAACTTTGTCACATTCATCTCAAAGATATTAATCTTTCCAATAAGACACTCCATATTATA

GGAAAAGGTAAGAAAGAGCGTATCCTATTTTTAGGAGATCAAACAACCTTCAATTTATTA

GAAACATATATAAATAAAAACGGAAAGGAATCTAATGATTTCCTATTCCCAGGAAAACAT

TCGCCTAAACCATTGTCAGAGCAAAGTGTACGTTTAATTTTAAAGAGAATCGTTGAACAA

AATGGCTTATCTAAAACTATTACACCACATATGTTTAGACATAGCTTTGCAACAATGCTT

CTAGATAATGATGTAGATATTCGATATATTCAACAAATTCTTGGACACAGTTCTATATCA

ATCACACAAATCTATACTCACGTATCTCATTCAAAACAAAAAGAAATACTTAGTTCTTTC

AATCCTATGTCAGTGATTCATTCTGAAATCGAGTAAGAGACATTTCCGAAGGTTACAGTT

TACTTCATCTTAAATTAATGTTTGTAGTTGACTTTAGTTAATAGATTTTATACTCAACAA

AAGCAAAAAGCATGCAAAACTCCACTTTGAATCAGCATTTATACTATATTCCGTATTAAG

AACTCCATAATTGTTTTTCTAAAATATAACTTTCAAGTATTCTTAAATATAATAAAAAGA

GATGGGCAAAATCTCAATTCCTGAAAAAAATGGAGTAAATCTTCCCACAAGAAACGCATA

ATTTCAAGTTTTTCAACACCTGAGACTATGCGTTTTTTGCTTCTAAAAAAGTTTCGCCCA

CCTTAAACAACTAACAAAAGTTATAAGTAGACTATATTTTAAAACTCAAGATCTAACAAC

CATTTCGATTAGTTAAATCTTTCAATTTTTCTTCTCCTGATTTAAGCATCTCTTTCTCAA

CTTGATTCCATTCGCCAAATAGTAAATCATGAAGAACGGTTGCTACTGAAGTTTTATCTT

CTTCCGAATCGTATACACAACTTCTATCTCGTTGGTAAATTTGAATTCTTTCAAAGATAG

CTAGTTTTTCCAATTGTCTTGTATTATCAACTAGATGATTTACAATGAAATCATGATGTT

CTTTTGGAGTTGCGCGTGCTTGATCTGGATTGATAGCGTACAGTTTTTCATAACGGATAA

GGGTGCTCAGATAGGACAGCTTAGGCTTTGTCGCAATCAAAGCTAATTGTACTTCATATC

CCATACTTTTCAAGAGTTGTGCTGTTTCCTTTGGAAGATCAACGCCCTTTAACTACCCTA

TTCTAACGCCAAATTGGAAGCGACTAATAATCTCATTAAACTCATCAAACGAAATGCCTT

TGGTTTTCGAAACTTTGAAAACTTCAAAAAAACGGATTTTTATCGCTCTGAACATCAAAA

AAGAAAGGACGAAATGTGTCCTTTCAAGATCTTAGCTTTTCTTCAACCCACTGCAGTTGA

CAAAGAGCCATAACATTTAGAAAAAAGAAATGCACCATTTTTGGTGCGTTACTTCTTGTT

TTTCTAGCCTAAACTTTACTTCTAAAAAAGCCACCTTAAAGAGTAATTCCTTTTTCGGTG

GCTTTTTAAAATACTATACATTAAAATACTCTTGTTTAATTTTTGTAAGCAGATTGTTAG

CAACCGAAGTTACACCAACATGCATCAGTTCAGGATCAGAATAATCTAGATTACGAGCAT

AGCCTTCTGCAACAGTCTGAGCAATACGCTTATCCAATTTATAATTAGAGGCTTTTAATA

GTGCTTGAAATAATTCTGAACTATATAGTTTAATATTATTTTCCAATGATACTACTGCCA

TTTTTGTAACTCCTCTCCTGTTTTTGATTGATAAACCTTCTCTATTTTCATTATATCACA

AAAAGATTGGTTGTGGATGGATAATTGTTTAAAAGAATATGGTTTTCTAAACTCAGCAAG

CATTTCTTCTTTTGTAATTTCATTCTGACGAAAACGAGCTAAAGCTTGGGTTGGATTGTC

ATCAGTCTGAACACCATAGATAAAATCAAAATGGTGTTGTAATTCATCTGGATTTTCCCG

ATTTTGAACGACAAATTCAGCAAAATCTAAATCTGACTCTGACTTTTTATGTTTTGCAAA

ATAGTGACAATGATAGTCAGGGTTCTTAAAAATTTCTATAAAATTTGAAATACGAAATTC

TACTATAATTCCTACTTCTTCTTCTGAGTCAAAAATATTATTATTAGAGCTGCTTCGATT

TAATACTTCAACTTGCTTGTTAATAAATTTTCTAGCTTGTTCAAAGTCAGGCGTGATATA

GAAACCAGGACCAAAATCTAGCTCACTTCCTAAATTAAATTTAACATCTATACCACTTTT

TAAGGATTCTAAATGTCTCAATAATGTAGCATGAAACCACTGCGTCTGACCTAGTTGCTT

TTCCTGCTTGGTAGTCAACTCCAAAATATTTACTTCCTTCTCTAGTCTATACTAAAAGTA

TAACACAAGACTTTATTCAAGTAAAAGAAAATTGTATAAAACTATTCACTTCCTTCCGTC

TTTCCTAAATCTCTCTAAATTCTTCTCCAATTGTTCCTTATGAAGTTGATTCTTAGCTTC

CTTTAATGTGTCGTCCAAAGTTTCTTCCCTATAAGTTGATTCTGTTTTTACTTGTTCTGA

TTCTTGATCTGGAGTGAAAATGATATCTAACAGTCGGTCTATTTTTTCAAGATCTTGGAC

AGACATATCAGATAGTCTATGAATCAGCTTTTTAAATACATCACTATTGTCGTGATTTTT

CTTAAAAAATGTTGTAAACATCTATAACCTCTCAAAAAAAGCAGCCTATCAGGACTGCTT

AGTGTAATTCCGAAATCGCATCATAAATGGACTGTAATTTCTTATTGTCTTTATTCTTTG

TGTCAATCAACGTATGTAGCTCTTCAATTTTCTTTTGACTGCTTTGAATATCGCTATTGT

TATCAGCAATAAGTCTTTTAAGATGTTGCTGATAACTTGTAGTGATATCTGTCTCTTTTC

CTGTTCTAACCTCTGTAACTTTAGGTTTAGAACTAGAATTTGATTCCGTCACAGGCGCTT

GTTTTCGCTTTTGAAAAGCTGCTTCATAATTTACATCATTACCTCCTTGATGATTTCCAA

ATAAGGCCGCAATTTTATCTGAATCCCCTTGATTTTCTGATTTACTTTCTAATGGTTGAT

TAAAATTCCAATCTTTTGTCATTCACTCATTTCATCCTTTCTAAATTCAGAATCATCGAA

TTGTCTTTCTTTACCAAAGGCATGGTCAAGAGCTTCTTCAAAACTCATGTGACTGATGCT

TTGACGCCTTTTGAACGTCGCAAACATCGCTGTCTCCAGTTGCTCTTTGTAAGCAGCGAG

TTTTTCTGTCTCTCTTGCTACCTTGCTTTCTTGCCTAGTGACCTTTTCTTCTAAGCGCTC

GATTATGGTCATATACGATCCTCCTTCATTCTAATATTAGCTAAAACATCTTGATTTTGT

TATCACAATTCTAAACATTGAGAGTTTTGTCTTACTGGTGGATTTCCAAATGTCTCTTTT

ACAGTTTCAAGCTTAAATTGAAAATCTGATTTCTTTTCTCTAAGAATATCGTTCCAGTCT

ATTTTTTCTTTCCCAGATTCATTATTAGGTAAATCCAGTAAAACAGGAAATCCTGATTGA

GATAACTTATCAGAAAAATCTTTTCCTGCATCATCACAATCTACCGCAAGTGTCAATAAA

TCAGGATGATTATCGAAATAGGTGGTGGTATCACGAATTGTATTGATCAAATGCAATAAC

TTTGAAGGTGTTACTGTATCTAAAAATTCCAACTTCTGATTTTCTTCAGCTATTAGTCGT

AAAGTTTGATAAGCAACAACAGACCTTTTTAATCCTTCCATAGATACCAAACGAACATTA

GTTAGACTTTGTTGATGAAGTTCGTAATAGCTCATCAAGTCGATGAACGATTCACAAAAG

ACCAGTCTATTTGGTTTACCAATGTCAAAGGATATTCCAACATGTCCATGGCTTCCTTTT

AGAATCGTTTTTAACCTTTCTCTAGGAAGAGAGTGATTCTTATAAATTCCTTGTAAGCTT

GCTGCCTGCAGCTTGTGGCGATGATCAAAGCTTTTAAAAACGTTAACAGGTTCAACTGTT

TCATTTGTTTTCCAACTAGCTTGTGCTATCAAACCTTGTTGAATCATCTTTTGTATGATT

TCTTCTGAGATTCCTCTACATTCTGTTAAGTAATATCTAGTCAAACTGCAGTTAGAATCT

TCTACTCTCTTTAAAGGATAATAAAATGGTCTCTCTCTTTTTCTTGAATAGCTTCTTTTT

GAAAAGGTTCTTCAGAAAGAAAGGCTAGAGCTTCTTTAAAGGAAATTCCCTTAACAAGTC

GAACAAAATCAATGACATCACCTTGAATATCTCTTGAAAACCATTTAAAAGTATTGGTAG

TTGAAAAAATCCGAAATGAATCGTGTTCAGGATGTTCATAGACACTGCTCGAAACTTGTT

TAAAGGAGATACCTAAACGATTGGCTACATCAAGAATTGAAATTTGCTTACATTCTTCTA

TTCCCATGCAATATCATCATTTTGTCGTAGTATTTGGCAGTGATGGTATTGATGATGATT

CTTCTAAAGTTTTAGGAGTTGCCTTATCTAAATCATCTAACCCAGATTTCCAAACCTGCA

CTTGATTGACCAATAACTTACCTGGTAGTTTAGAATAGGATAACTTAACCGTTCTGGTTT

CTGTCTGATTGGTCTTTGATTGGTTGGCATTCTTTAAATCAGATACATAGGTTACATTAT

AAGAGACCATGGCAATAGCTTGATTCGTAGTCTGATTGACAAAGATATCAGCTTTTTCAA

AATGATAATCCAAAATATAATCCTTATACACTTGGTTCATGGCATCATTTTGACTTGACA

ATTCTTGAGAATAAGCCGATTCAGTCATATAAGGTTGAATACGTGTATTATTTTCTCCGA

GCTTTTCTTTCGTATAGTACTGCGTCAAAAATTCTTTTACAGTATCCGATGACAAAATGC

TGGCTTTATCTTCTTCTTGTTTGTCCTCTACAAGTTTAGCTGCAGCCAATTCGATTTCTT

TGCGACTTTGTTTAGCAGTCGAGTGTTGACCAGCAGTATATCCCATCATGAGAATAAAGC

TAGTTGCGGCTACTGTTCCAACACTAATTAAGGCTTTAGTTTTGACTTTATTTAACATCT

TAGACCTTCTTTCTATAAAAACTAGGTCAAGAATACCAGATAAATCTTGAAAAAAGTATC

ACAATAGAAAAAGGACTCTACATTGAAAAAAGAATCCTTAATTTTTTATAATACTATCAA

ATAATTGTCTCAAATTCACCGAGCTTACCACGGGAAAAACGAGGCCTGAGTAAAAAAAGC

GCACGGTATTTAGACCCCCAAAAAACCGAAATGCGTTGAAAGCGTATGATCAGTTTTATC

ATGACTAGATAATAGATGATGAAAATCAGGAATATCCAAAATCATATTCTTATGAAAACT

CTTTTGAACTTGTGAAAAGCTTATGTTAGTCATGTTTTAGAACTCTTTTCACGACAACCA

ACTCCCCATCCTTGATTTCCCAAATCTGATCAAAGCGTGATAGCATCTCTTCTTGACGGT

GAAGTGTAAGGACGACAGCACTTGACAAGCTAAGTACCTTGTCTAATTCCTTTTGAAATT

GGCTGGCATCCAAAGCAGAAAAAGGCTCATCCATGATAATTAGCGGACTATGACGATTTT

TCTCCCGATTGAGATACATGTGCTGTTGCTGACCACCTGACAGGGACTGGGGTGGTATCT

TTTCAAACTTTTCATCCCTAAAATTAGCCTCAAAAGTCTTGAAAATAGTGACATTATTTT

CATAAGATGAGATAAAGGTGTGCTCCTGTTGCAAAATCATACCAAATTTGCCCCAGAGTT

GATGAACAGGGAGATGATCAAGTAAGATTTCACCTTGATAATAGGGGTAACTATTGCCGG

CGAGGCTAGTTACCCTTAAGTTATTGGTATGACTGGTTTTAAGCGCAAAAAAAGTTGCCT

TTTCGTACCTATTAATGTATCGTTTTAAATGACTAGTAAAAAACATACATAGAAAGGGGA

AAAAGCAACTTTTTTTATTGTCATAGTTTGTGAAAACTAAGTTGTTTTTATGTGTTATAA

CATGGAAAAGTATACTGAGAAAAACAAAGAAATCAAGTATTTCAGAAATTTATTAAACGT

CATATTGGAGAGAATCAAATGGATTTAGTTGAAGATTGCAATACATTTCTGTCTTTTGTA

GCTGATAAAACTTTAGAAAAACAGAAATTATATAAAGCTAATTCTTGTAAAAATCGATTT

TGTCCTGTCTGTGCTTGGAGAAAAGCTAGAAAAGATGCATTGGGTTTATCTTTGATGATG

CAATATATTAAGCAGCAAGAGAAAAAGGAGTTTATCTTTTTAACTTTGACTACACCTAAT

GTAATGAGTGATGAATTAGAAAATGAAATAAAACGTTATAATAATTCTTTTAGAAAACTT

ATAAAGAGAAAAAAGTAGGTAGTGTTATAAAGGGATATGTTCGTAAGTTAGAGATTACAT

ATAATAAAAAAAGAGATGATTATAATCCTCATTTTCATGTGTTAATTGCAGTAAATAAAT

CGTATTTCACAGATAAAAGATATTATATTAGCCAACAAGAATGGTTAGATTTATGGCGTG

ATGTAACGGGCATTTCAGAAATAACACAAGTTCAAGTTCAAAAAATAAGACAAAATAATA

ATAAAGAATTATATGAAATGGCTAAGTATTCTGGTAAAGATAGTGATTATTTAATAAATC

AAAAAGTCTTTGATGCATTTTATAAATCACTTAAAGGTAAACAGGTATTAGTTTATTCAG

GATTATTTAAAGAGGCTAAAAAGAAATTAAAAAATGGGGATTTAGATTACTTAAAAGAAA

TTGATCCAACCGAATATATCTATCAAATTTTTTATATTTGGAAACAAAAAGAGTATTTAG

CTAGTGAACTTTATGACTTAACAGAACAAGAAAAAAGAGAAATTAATCACAAAATGATAG

ACGAAATCGAGGAAGAACAATAACAAAATATAAGTGCTAACAGCTGACCTCCCGATAACA

CCATGTAGTTATTGGGAGGTCAGCTGTTGAATTATGCACGAGTATTTTAAAAGTTATTGT

GATGACGACGATAAACGATTATCAAAAGTATAATGTTAAAATGCTTTATTATACTAACGT

TATATAAACATTATACTTTCGTTATACAAATTTTAACCCTGTTAGGAACTATAAAAAATC

ATGAAAATTTTAATTTGCATGTAACTGGGCAGTGTCTTAAAAAATCGACACTGAATTTGC

TCAAATTTTTGTTTGTAGAATTAGAATATATTTATTTGGCTCATATTTGCTTTTTAAAAG

CTTCTGTAGGTTTTTAGGCATAAAACTATATGATTTACCCCTAAATCTTTAAAATGCCCC

TTAAAATTCAAAATAAAGGCATTTAAAATTTAAATATTTCTTGTGATAAAGTTTGTTAAA

AAGGAGTGGTTTTATGACTGTTATGTGGTTATCGATTATAGGTATGTGGTTTTGTATTGG

AATGGCATTTTTTGCTATCAAGGTTATTAAAAATAAAAATTAGACCACGCATTTATGCCG

AGAAAATTTATTGTGCGTTGAGAAGAACCCTTAACTAAACTTGCAGACGAATGTCGGCAT

AGCGTGAGCTATTAAGCCGACCATTCGACAAGTTTTAGGATTGTTAAGGGTTCCGAGGCT

CAACGTCAATAAAGCAATTGGAATAAAGAAGCGAAAAAGGAGAAGTCGGTTCAGAAAAAG

AAGGATATGGATCTGGAGCTGTAATATAAAAACCTTCTTCAACTAACGGGGCAGGTTAGT

GACATTAGAAAACCGACTGTAAAAAGTACAGTCGGCATTATCTCATATTATAAAAGCCAG

TCATTAGGCCTATCTGACAATTCCTGAATAGAGTTCATAAACAATCCTGCATGATAACCA

TCACAAACAGAATGATGTACCTGTAAAGATAGCGGTAAATATATTGAATTACCTTTATTA

ATGAATTTTCCTGCTGTAATAATGGGTAGAAGGTAATTACTATTATTATTGATATTTAAG

TTAAACCCAGTAAATGAAGTCCATGGAATAATAGAAAGAGAAAAAGCATTTTCAGGTATA

GGTGTTTTGGGAAACAATTTCCCCGAACCATTATATTTCTCTACATCAGAAAGGTATAAA

TCATAAAACTCTTTGAAGTCATTCTTTACAGGAGTCCAAATACCAGAGAATGTTTTAGAT

ACACCATCAAAAATTGTATAAAGTGGCTCTAACTTATCCCAATAACCTAACTCTCCGTCG

CTATTGTAACCAGTTCTAAAAGCTGTATTTGAGTTTATCACCCTTGTCACTAAGAAAATA

AATGCAGGGTAAAATTTATATCCTTCTTGTTTTATGTTTCGGTATAAAACACTAATATCA

ATTTCTGTGGTTATACTAAAAGTCGTTTGTTGGTTCAAATAATGATTAAATATCTCTTTT

CTCTTCCAATTGTCTAAATCAATTTTATTAAAGTTCATTTGATATGCCTCCTAAATTTTT

ATCTAAAGTGAATTTAGGAGGCTTACTTGTCTGCTTTCTTCATTAGAATCAATCCTTTTT

TAAAAGTCAATATTACTGTAACATAAATATATATATTTTAAAAATATCCCACTTTATCCA

ATTTTCGTTTGTTGAACTAATGGGTGCTTTAAATGATTACAGGTATAATTTTGCCCTTAT

GTTTTCTAATAATTTGAATTTTTTTACTAATTTGTATGTTTTCTTCTTGATTTATCTCTG

TAAGCGCTTCATCGATATGTGATATTAGGCGAGTTCCCTCTCCTTTACCAATATAGAAAG

GTAGTTGCTTACCTTCTTCATAGAGTCCGTAAACATAGTATTTATCTTTTTCATTTTTGG

TAGCACCAGTAATATCATTTAATTTTTGATAATTCATTTTTTTACTCCTATTTAAATATT

TTACTCCTATTTAAATATATTATAAAAGTTCTGCTCCATACTATTAAAAATTAATTAAAA

TAATCCTCTTATTCTACCTTCATTCTGATTCTGAACTATTTATATGTTAAACTATATTTT

CTTTAAAATCAAGTAAAATCCTAGAGAATAATCTAAATAAATTTGGGGACAATATATAGG

TTTACTAATAACTACACAATACAGTATTTACGATAGCATTAAAAAGAGTCTGTTATATTT

ATTTTATAGATCTTTAAATAATTTGCTCAAAGTTTTTGAAAATTCTTCTGTAGCATAAAT

TTTAGCTTTCCCATCTCCTTTTAATAAGTGCTTAGCTCTTATAGTGGCATTTCTTGGGTT

ACTAGAGCGATAACATTTTTCATAAGTTTTTAATTCAGGAATAGCGTTAGTCCATTCAGC

ATAACCACGCGCGATAAGAATAGTTACAGGTGGTTTGCTTTTATCCGCAATTCTTTCTTT

AATCAAATTAAATAAAAATTGTTGAGTAGGAAGATATCCTTTTTCTTCAACTATTTTACT

AATTTCCCTATTATAATTATCAGAATGATAGGGGAAAAACTCAGCTAAAGCTATATGCTT

TGTAAACCAGTCTACATGTTCTTTATAATCTTTGTTTGTTTTAAATTGTTCGCCCATACC

TGCATCTTCAAACCAGAACTTAAATTTATCTGCTGTGTATCCAAGTTCATCACCTTCATC

AAGTTTGAAGGCGTGGAAAACTACTTTTTTCAGTTGTAAGCTATCCAAACAGGTTTGCTG

ATAATCTTTATTGTTGTTGTAAAGTTCTTCATATCCTTCAGTATATCTAGGATTTTTTGA

CAAAATGATAACATCAGCATTTTTAACATTCCCTACAAATTGTTGAGGATAGAAATTCAA

GTGTAGTTCAGTGCTTTCAGTGGAGTTATTTTTATTTTCGGATTCTAGTATGGTTTGAAT

AATTTCCCAATCATCACGCGCAACAATAGCCTCTTTGCCTTTGTGAGTTGTTGTTAAAAT

ATCATTCCAAGGGTTTTTAGTTGTAGTCATTTTGTTTTCCTTTTTTTGTAGAATAACTAT

ATTTTACCATGTTTTAATGTCAAATACAAAAATAGATTTTATTAATATATTTTCAATTAT

AAAAGCAGCTGATTTAAGAACACTTTTTAATATCAATAAGTCGGACATTATTTATATACC

CTTTCACACCATGGTTCATGACGTTCGGTATACATCAGTTCTACTGAACTGTAACGAACT

CTCATTTCCCCGTCTACAAATTATTTTCAATACTGTAGAACAGATAGAATTAACTAAGAA

GTATAAGACTAGTACAAAAAGTCCAATAAATTGGAAATATTTTGAGAACAACGATTAATT

AAAAAAAGAATGGCGGTGAGATTTATTACTTTTAATTATACTCACTTTGTTAATTTATTT

TAAAGCTATTTTTTGACCATAATCTAAGAGCTAGCATTTTTTAATAAACTTTACTTTAAA

AGTTGTGTTCTAACAATCATTTCGATTAGTTAAATCTTCCAATCTTTCTTCTCCCACCTT

CAACATCTCTTCCTCTACCTTGCTCCATTTTCCGAAGAGGTATTCTTGTAGCACTTTTGC

TGCTGAAATTTTATCTTCTCCTGAATCATATACACAACTTCTATCTCGTTGATAAATTTG

AATTCTTTCAAAGATAGCCAATTCTTCCAATTGTCGTGTATTATCAACTAGATGATTTAC

AATGAAATCATGATGTTCTTTTGGAGTTGCGCGTGCTTGATTTGGATTGATAGCGTACAG

TTCTTCGTATCGGATAAGGGTGCTCAGATAGGACAACTTAGGCTTTGTCGCAATCAAGGC

TAATTGTACTTCATATCCCCTACTTTTCAAGAGTTGTGCTGTTTTCTTTGGAACATCAAT

TGTTCGTAAAGTTCCTTCAATCAAAAGATTGTATCCCAAATGACTAAATTCGGTTAGTAA

AGACTCTACCATTTTCCCTGCAAAATCTTTGGTGTATTCTACACTATCTTTGCCATATTC

TCGCTGCAGTTCTAAATAGTGTGGATGCTGAGAACGAAAACTATCACCATCTATGATAAC

AATATTTCCTTGAAATTCTTTCTGTTTAATACGATGAATTGTAGTCTTACCGGCACCACT

TTGCCCTCCAAGCAAAATCGCTATAGGTTGCTTACTGGACTTTTTTCCTCTTGTCAGTGA

ACGAAGATTCCGTGCTAGAGCATGTTTGAATTTACTATCAGTATAATCTTGGATTTCCAT

TAGGCTACCATCCGTTTTTCAGATATCTCTAACATCCGTTCAATTCCATCCAAATAGCCA

GTATATCTCTCTATTTCATCAAAAGTTTCTACTAAATAGATATTCGTATGAATCAAGTCA

GACAGATCGTCACTCATTAAAATCCAAGGATTAGATTCATCATCAATGCTAATTCCATGA

CTATCTTGATAACGATAGAGTCGAGATAATAGATTAGCACCTCTTTCTTTCACAATTTCA

ATTTTTAAAGTCAATTCATAATCTTCAACAGGATTGAGCATTTTATCTTCTCCTACAATA

TCGACATAAGATACATTAAACTTCTGGCAGATGATGTCTATTAGTTCCGTAGAGACTGAA

CTCGTTCCATTTTCATAACGACTTAAGCTATTTCGAGAAATACCTACAATCCGTGCAAAT

TCGGGTTGGGTTAAATCATGTGTTTTACGTAAGGATTTTATATTCTTTCCAATCATGGCA

AACTCCTTTTATTTTTATACCACCATTATAACATTTAGAAAAAGTAAATGCACCATTTTT

GGTGCATTATTGGCTGTTTTTCCTAAATCTCTCTAAATTCTTTTCCAATTGTTCCTTATG

AAGTTGATTCTTAGCTTCCTTTAATGTGTCGTCCAAAGTTTCTTCTCTATAAGTTGCTTC

TGTTTTTAGTTGTTGTGATTCTTGATTTGGAGTGAAAATGATATCTAGCAGTCGGTCTAT

TTTTTCAAGATCTTGGACAGACATATCAGATAGTCTATGAATCAGCTTTTTAAATACATC

ACTATTGTCGTGATTTTTCTTAAAAAATGTTGTAAACATCTATAACCTCTCAAAAAAAGC

AGCCTATCAGGACTGCTTAGTGTAATTCGGAAATCGCATCATAAATGGACTGCAATTTCT

TATTGTCTTTATTCTTTGTGTCGATCAACGTATGTAGCTCTTCAATTTTCTTTTGACTAC

TTTGAATATCGCTATTGTTATCTGCAATCAGTCTTTTAAGATGTTGCTGATAACTTGTAG

TGATATCTGTCTCTTTTCCTGTTCTAACCTCTGTAACTTTAGGTTTAGAACTAGAATTTG

ATTCCGTCACAGGTGCTTGTTTACGCTTTTGAAAAGCTGCTTCATAATTTACATCATTAC

CTCCTTGATGATTTCCAAATAAGGCCGCAATTTTATCTGGATCTTCTTGATTTTCTGATT

TACTTTCTAATGGTTGATTAAAAATCCAATCTTTTGTCATTCACTTATTTCATCCTTTCT

AAATTCAGAATCATCGAATTGTCTTTCTTTACCAAAGGCATGGTCAAGAGCTTCTTCAAA

ACTCATGTGACTAATGCTTTGACGCCTTTTGAACGTCGCAAACATCGCTGTCTCCAGTTG

CTCTTTGTAAGCAGCGAGTTTTTCTGTCTCTCTTGCTACCTTGCTTTCTTGCCTAGTGAC

CTTTTCTTCTAAGCGTTCAATAATGGTCATATACTATCCTCCTTCATTCTAATATTAGCT

AAAACATCTTGATTTTGTTATCACAATTCTAAACATTGAGAGGTTTGCCTTACTGGTTGA

TTCCTCAATGTCTCTTTTGCACTCTCGATCATCAATTGTAAATCTGATTTCTTTTCTCTA

AGGACATCATTCCAGTCTACTTTTTCTTTCCCAGATTCATTATCAGGGAAATCCAGGAAA

ACAGGAAATCCTGATTGAGATAACTTATCAGAAAAATCTTTTCCTGCATCATCACAATCT

ACCGCAAGTGTCAATAAATCAGGATGATTATCAAAATAGCTGGTGGTATCACGAATTGTA

TTCATCAAAGGCAATAACTTTGAAGGTATTACTGTATCTAAGAATTCCAACTTCTGATTT

TCTTCAGCTATTAGTCGTAAAGTTTGATAAGCAACAACAGACCTTTTTAATCCCTCCATA

GATACCAAACGAACATCAAATAGATTTTGTTGATGAAGTTCGTAATAGCTCATCAAGTCG

ATAAACGATTCACAAAAGACCAGTCTATTTGGTTTACCAATGTCAAAGGATATTCCAACA

TGTCCATGGCTTCCTTTTAGAATCGTTTTTAACCTCTCTCTTGGAAAAGAGTGATTCTTA

TATATCCCTTGTAAGCTTGCTGCCTGCAGCTTGTGACGATGATCAAAGCTTTTAAAAACG

ATAACAGGTTCAACTGTTTCATTTGTTTTCCAACTGGCTTGTGCTATCAAACCTTGTTGA

ATCATCTTTTGTACGATTTCTTCTGAAATTCCTCTACATTCTGTTAAGTAATGTCTGGCC

AGACTGCAGTTAGAATCTTCTATTCTCTTTAAAGGATAATAAAATGGTCTCTCTCTATTT

TCTTGAACAGCTTCTTTTTGAAAAGGTTCTTTAGAAAGAAAGGCTAGAGCTTCTTTAAAG

GAAATTCCCTTAACAAGTTGAACAAAATCAATGACATCACCTTGAATATCTCTTGAAAAC

CATTTAAAAGTATTGGTAGTTGAAAAAATCCGAAATGAATCGTGTTCAGGATGTTCATAG

ACACTGCTCGAAACTTGTTTAAAGGAGATACCTAAACGATTGGCTACATCAAGAATTGAA

ATTTGCTTACATTCTTCTATTTCCATGCAATATCATCATTTTGTTGTAGTATTTGGCAGT

GATGGAACAGATGAGGATTCTTCCAAAGTTTTGGGAGTGGCCTTATCCAAATCATCTAAC

CCAGATTTCCAAACCTGCACTTGATTGACCAATAACTTACCTGGTAGTTTCGAATAAGAC

AACTTAACCGTTCTGGTTTCTGTCTGATTGGTCTTTGATTGGTTGGCATTCTTTAAATCG

GATACATAGGTTACATTATAAGACACCATAGCAATGGCTTGATTCGTAGTCTGATTGACA

AAGATATCAGCTTTTTCAAAATGATAATCCAAAATATAATCCTTATACACTTGGTTCATG

GCATCATTTTGACTTGACAATTCTTGAGAATAAGCTGATTCAGTCATATAAGGTTGAATA

CGTGTATTATTTTCTCCGAGCTTTTCTTTCGTATAGTACTGTGTCAAAAATTCTTTTACA

CCCAAATCATTCATACCTCTCTCAACTAGATGTAACTTACAAAACCCCTGACCTCATGAG

CCACTTTCTTCCTCCTCATGAGGTCAGTTTTACTTTCTGCTGTTCCAGTATCGTTTTTCC

TCGCTAGATTTCCTCAAAAGGGCAGACTCCTCCCTTGGTGCGTCACACGATTTTTCATCT

CGACTGTTCTTTAATGCATCATTAACGACGCTTTTCTTCCAGGTGGTTCATAAGGAACAG

GAAGATTCAGGTTGACTTTTCTAATCCTAGAATAAAGTGCTGAAAACAATTCGGAATAGG

CATAGAGACTAGACAATTTGAGGAGCTGCTTGCGTCCTGTTCGAACACATTTTCCTACCA

CGTGAAGAAAAAGATGGCGGAAGCGTTTGATTGTTAAAGTTTGGAAGTCACCTCCAGCTA

GATGTTTGAGAAAAAGATAGAGATTGTAGGCGATACAGCTCATCATCATACGAACTTCGT

TTTTGATTAAGGTTGAACTATCCGTTTTATCGCCAAAAAATCCCTCCTTCATCTCCTTGA

TGAAATTCTCGGCTTGACCACGTCCACGATAAAGCTGAAACTGGTCTTGGCTTGTTCCAC

TCGTCATATTTGTAACGAGAGAAATAACATCGTAGAACAAGTTTCCTTCTTTTCGTTCAG

AGAACTGGCAGACACGACGCTTGTGCGACCAAGATCCTGCTTGATAGAGAGTTTCTGAGT

AGGCGGAGTGGGGCAAGATGGTTAAGTCCTCATCCTGTGGGCAAGGGAGGGAAAGGTCTC

CAAGACGGCTCAGAACAGTATTTTCTTGAGTTTTATGAGGTAGCATTGCCCTGTTTTTTC

AATTAAATCGTATAATTTTGGGGTCGCAAAGCCACTATCCATTCGAAAGAGAAGTTGATT

AAACCGTTCTAAAACAGGTGTGATAAAGCTGTCTGCCTCTTCAGAACAATAACGATTACC

AGGACGAAGCTGGGCATTGAAACAATAACCTGTCTTCCCCTCGAAAGCATAAAGAGGATG

ATAGCCATGAGCACGATAGTGGGCGTTATAAGCAACACCTTCTTGCTTGCCATAAGTTGT

GAAATGGGTAGAATCGATATCTACAATGAGTTGGTTTAGCTGGTGAAACTGTAAAAAGAA

TTCGACCAATTCAAGGTTGAGGCATCGCAAACTATGGACTGTTTCCTCGTCAGTTCTGGA

AAGAAAACGGGATAAGGTTGGCTGTGAAGCAAGCTGCCCTCCTTCCAACAATTTTGGAAA

GTAGGCATCAGCTGACAATTCTTTACAAGCATAGTCCGTTCCATAGCCTGTTAACAGTTG

AAAGAGGAACTGGACAAGGATATCTGAATCCGAATAACGACAGTAGCGGCGTTGGTCATT

CGTTACTAAATACTTAGAAATCCGCTCTTTTAGTTTCAACTGGGAAAAAAGTTCCTGAAA

AAAGATAAGACCACCATACTGGGTTAAATGACCTCCATCGAAAGATAGTTGGTAAAAAGA

CTTGTTTTGGAAGTGATGATTTGGTAAACTGTTCATATGAGTTTCCTTTCTTTTTGTGTT

TTTTTCTACACTTATACCATAAAGGGGAAACTCTTTTTTGTCTAGTAAAAAACACCCATT

GGGTGAAAAAGAAACCATCCAGGATCTAAGCTAAGGCAAGGATTCTGGATGGTTTTTAGA

TTAGGGGTGCATAATTGGGGTTTACAGTATCTGATGACAAAATACTTGCTTTATCTTCTG

CTTGTTTGTCCTCTACAAGTTTAGCTGCAGCCAATTCAATCTCTTTACGACTTTGTTTAG

CAGTAGAATGTTGACCAGCAGTATATCCCATCATGAGAATAAAGCTAGTTGCAGCCACTG

CTCCAACACTAATTAAGGCTTTAGTTTTGACTTTATTTAACATCTTAGACCTTCTTTCTA

TAAAAACTAGGTCAAGAATAACAAAAAGAACTTGTAAGTTTTATCACACCACAACGAAAG

GTAATTCCTACATTTCAGGCGCAAAATCGTACAGTTCAGAGAAAAAAATAAAACTAGCTG

TCTTTGAAGTATAATAGACTTATTGGAAAAAAATAAAATGCATACTGTACTATGCTATAC

TATAGTTAAGAAAACTATTTACAAAAGATCATGCAACTAACTTCTGAGAACTTTAAATTA

TTTATTAGTGTAAAAAAGGACACCTTCCCAACGAAGATATCCTGTCTAAATAAAGCTTAT

AAAACCAAACACTATAAAGGAGGACGTCCAAAAACGCTAAGTCTATAAAAACAGTTGATG

CTAAACTTGCGTTATTTATTCTATTGACCAACTCTACGTCTCCTAGCTTTTGATTTTGGT

CTTGGATTAGCGACAGTCACCGAACGATAATATTGGTAGAAGATACTCTTCGTTCTTCAG

GTCTATTTGCCTTAGACAACTTAAAAGCAGCAAGTGTCACTATAGCTATTGATGTCATAG

AAAGTCCTATTTCAACGTCCTAAAAGAAACCAAAGTAAAATTATTCAGGAAAAAAGAGAC

ACACCATGAAGACACAAATCATACTCGATTTAACACCACTTCATGCCTGCTAATTAGTTT

TTTAAGAAGGTCATACACATGATTTTACTCTTTTTAAGAAAGGATAGGTCAAGTTTGCCA

GAGCAATCACTTGATTTTCTGTATTTGGATTATTTATGCATTATGAATATTCATAATATA

ACCTTCATTCCTGTTAAAAATTCTAAAAAGCATCTATTGACTGAAGAGGATAAGAAGTTT

AATAGAGATACTTGTAATACGTATTAAAATTGAGCATTTTAATGCTAAGTATAAAACTTT

TCAATTTATGGAATCATCAACTATGAGTTAAAATAATTTTCGAACAAAGGCTGTCCAAAA

AACTTGATATAATGCGTTTTATTATGGGAATATTCATTTCATTTACTCCTGAAATTGAGT

TTTTACCCAGACTCATTAATGTTATTGATATAATCCAATAATAATGATAATATCAAATAG

TAATAAAAAAATTTGATAGTATTTTTTCATAATCTGTCACGCTTTCTAATGATTTTTTAT

GGTAAATGGAGATAAATATGATTATAGGTGAAACTTATAGAAAAATACGAGAAGGAAAAG

GTATTTCTATTTCTTCATTAGCAGGTGCAGAAATTTCAAAGTCTCAAATATCTAGATTTG

AATTAGGAGAGACAGAAATCTCATTTTTTAAATTATTATATCTACTTGAAAAAATAGGTG

TAACACTAGAGGAGTTTTTGCTTTCATGTAATAATTATCAGCCTTCAGACTTCAATACCT

TGATACGCTTAGTTCAACAGGCTGCATACAATCAAGAAATCAAATCATTACTTAATATGG

TAAGCAAAGAGATGGAACTGTTTAGAGAAACCAAATCTCATTATCATAAGTTAAACGCTA

TTTTCATTGAAAGTATTATCTATGGAATTGATAATACTCATCAATTGAGTAATCAAGACA

CCTCTTATCTTACTAATTATTTATTTTCTGTTGAAAACTGGGGATATTACGAAATTCTTA

TTCTTGGAAATTGTTGTCGAGCAATATTACCAAATTTATTATTTAGATATGCCAAAGAGG

CGCTTAAAAAAGGAAAGTTGTATAGTTCTATACCTAGAAATAAACAAGCTCTCGTTCAAT

TACTTCTTAATTCACTTCTTATTATGATTGAAAACAGCTTATATGAGGAAGCTTTATTTT

TAGAACAAGCTACTAAGAATATATTATCTAATTCCACAGATTTTTTTGAACAAACTATTC

TTTTGTATTTAGAAGGATTTTTTGAACTTAAATTTCATCATAATCAAAAATCAATTTTAA

AAATAGAAGATGCTTTAAAAATTTTTGAATTATTCAATAAAACATTATACAAAAATTATA

AAGATTACTACAAAAAAAATATTATCATTTTATTACAATGTGGTAATTCCTATTTTGAAT

AATTGCTTTTTTTGAAAAACTTTTTAATTCATCTAAAGAAATCAATTCCAGCTCTTCAGC

AATACTATCCCAACTTGGAATATCAAATAATTTTTCTCTACAAATTGTTTTTAAATATTC

ATTTTTATTAACTTCACAATTTATGTACAATTCATTGATTACTCTTTTTTTAGCCTTATA

AAATAATAGTTCATCAGATAAATCGGATTCATTAACAAGATTGTTCTCCAGTGTTTTAAT

CAATTTTTTCTTATAGATTGTAGGTGTTATAGTTACTATTTTTAGTAAAGTCATATTACT

ATAAAATATTGGGTATGATACAATACTATATATCCATTGCTTTTGTGTCCTTAACTTATG

GCCTAATAATGAATCTTTAATACCAGTTAACATCGAGTTTAAGATACTTAACATTGCCAT

TTCATGAGATGATGCTACCATACCAGATATATCTAGATACATAGAAATTTCAGAAAGTTC

TTGTTGTTTATCTAATTCCGTTAAACCAGTTGAAAAAGCCGGAGAGTTAATTTCTACAGA

TGATCCACTTAAAGGTAACATATTTTCAATACTTATGATTTCACTTTGTAGAAAACTTGG

ACCAGCAATAGCAATCTGCAATTCTCCTGCTTGAATATTTTTCATATAAACAAAGTATAA

ATTTCTAATTATCTTTTTATATTTTCTTAAATGCTCGTAAAGCCTTATTCTATGTGCTTT

CGAGTATTTTTACTGTAGGAAGATACTTCACGTTTCTTTGCATATTTCCTCATGTCTTAG

CTGTCAGAAGTGGTAAATAAGTAGTAAATTCATTTGTACTACTAAGCAACAAGACGCTCC

TGTTGCTTCTCTTTATTCAAGCGTTTCATTTCTGCCATTGCAGAATCGAATGTTGCATGT

GCGTAATAGTTCAGCGTCATGGCTATATTAGCATGTCCCATAATGTACTGTAATGCCTTT

GGATTCATTCCTGCATTTGCATAGTTGGTACAGAATGTATGTCGCAAACTATGTGGAGTG

ATGTGTGGCAATTTATCCTCGTTATACTTATTGTATTTCTTAACAAGACCTTTCATCATG

CCGTTGTAATCACTTGCCACTTTTGGATAGTTCTTTCTATTAAGAAAGAGGAAATCACTA

TATCCATCAATCTCAACACGCTTATCATTCTTTCGATTCGCTAACACTCGCTTAAATGCT

TGATAGGCTTCTTCAACCATAGGAACTTGACGTTCGCCACTTTTGGTCTTTGGTGTTTCA

ATGTAGTACCCAATTTCAGTATCTCTCAATAGCTGATGGTCTATATTGACAAGACGATTC

TCAAAATCTAAATCTGGAAGTGTCAAACCACCAAACTCTGAAATACGAAGACCTGTTTTT

AAGAGTATCAGAATTTCATCATAATTTTTGCTGTAGGTTTTATCAGCTTTTGCAAAGGCT

AACAGTTTTTCTTCCTGTTCTTCTGTTAGTACGGTCTTAGGGACAGTATCATCATCAAGA

ACTGCTTTCAGTTGAAAGTCAAATGGATTCTTCCGAACACAATCATCTTGTATAGCAATA

TAGAATGAAGCCTTTAAAGAACGTTTGTAGTTATTGATGGTTTGATAAGCATAACCATTT

TCACTCATTCTAATAGCCCATTCTTTAGCGTCTGATGGCTTAATACTGTCAATACTTCTT

ACACCTAACTTGTCTTTCTTCAAAATATCCATAAGATATTTGCGTCCAGTTTCAGTGTTT

TTTCTAACCTTTGGTCTTTGAGCGTTCTGTTTTGCGTAAAGCTGGCAGAGTGTCATTTTC

TTTCCTACAACATCAATACCATCATGAATGTCTTTCTGTAACTCTGCGATTTTCTCTCTA

AGTGAGATACAATCACGCTTTCCTGCTGGTACTAGGTCTGTAGCCACAAGTTTCCACGAG

TAAACAAATTGCGGTTCTCCAAATGAATCTATATATTTGTATAAGTATCTTCCGTCTTTT

CGTTGGCTCTCTCCAGTCTTTAAGATTCGACCTTTATTGTCACGTCTTTTTTCTGACATG

GCATTTGCTCCTTTCCTTTATGGAAAGAGCCTTGATACGACTTAATACTATTTTATCATA

TACAAGACCCTTTGGCGACGCTAGATTGCGTCCAATGTATCTATAATTTTTTCAAATTGT

TTTCGTTTAATCTGAATACGATTGCCATTCATAATCAGCCAATTTGCATTTTTATTTTCC

TCTGCCAAGCGTCGTAGCTTGTTTTCGCCAATACGAAAATATTTTGACGCTTCTTCAATG

GTTAGGGTATAACGTTCCCAAATAGGAATGTCAGTCTGCTTCATAAAATCCTCCTTTCCA

AATCACTTATTTGGATTTCATAAAAGTTGTTTTACCAGCAATCGAACAGCTTTAGCAAAG

CTCACGGGAGTTCCACCCCTGCATGGTTCTCATGTAGCCATACTCATTGCCTGCGACGGT

TTTATCACGCTCGGACTATTGACTGTATGGGAGTATCATTATCACGATAAGAATGTCGTT

GCAGGCAATCCTGCTAAAGATTGCTTCTCGGATCACTAACATGAATCGCTCGCTATCTTT

ATAAGATAGGTCATGGCGGTTAGTTCCGTTGGCTCTTTTCTTATCGAAACGTATTCGATT

ACTTTTATTCAGTTTTCAAAGAACAATGGCTCGTTAGCCTATCAAAACACATTGAAAGCT

CAATATGCTTTGGTGGAATAACAAACCTCCCTGTTCGGGAAGCGTGGAATGGTTTAGCAC

GCTTCCACGAAAGGAGAGAGGATATTACTTAATTTCAAATGACAAAATCTTTGTAATCAG

TCTGGTTTCCATTCTTCCACGTAAGACTTCATCAACGACCATACTTTGATTGCCATATTC

ATCTTTCATAAGTCGTAGGGAACGCTTCGTTATGTACCCTCTGTAATGATGTAGAATCTG

GTTAATCGCTTCGGTATCGCCATCTGTTGCCTTTACAATGAGAGGAAAGGGAATCATAGG

ATATTGTGTTTTCATTCTTCAAATTCCTCCATAAACTTTTTAATTAAGGCTAGTCCACTG

GTTCTATGCCGATAGACAGTAGAACGGTTCAATTTCAACAGGTCTGCAATTTCTGAATCG

CTCATGTCCATAAAGTAAAACAGCAGTAGAATTTCACGTTTCTTGTCTGGCAACTCACGT

AATGCTTCACTCAACAAATCATTTTCAACGCCTACTGATAACCCATTGAGTGTAAAAATC

TGAAAGTCAGTTGAATAGTTATCTGTTGTCGCAAACTGGCTAACAAGATAATCGCCAACA

TCCGAAAAGGACACCTCACGCTTTGCAATCCTTGAAAGATAAAGCATATAATTCTTTCGC

TCGTCTTCCATAGCACGTTTACAGATATAGTCAAACTGATTTTCTATTGTGGTCTGAAAA

GAAGATGGTTTCATGTTTCTCACCCCCTTTCTGTCTAGGAAAGGAAGTGAGCCTTGCTCG

TTTATCTCCTTTCACTCTTAGTCCCAATGTGAAAGGGGGATTTGTTGCATTACTGATAAA

TAAACTTTGTAAAAAAGTTCTGAATAGCCAAAAAAGCATATAAACAGATTTATTTCTCTG

TTTACATGCTTCTGTTATTCTATCTATATGATTTATAAAACCACATTGGTGGACGTACTT

ATCTATTGCAGATAGACGACTTTTTTTGACAAGAACCCAATGTAAGGAAATTTATTGTAT

ATGATGTACTTCATGGCGACGTTGACCTCCAACAAACCGCCATTTGGAAGTAATATACAA

TATTTTAACAGCGTAAATAGCACTACCATATAACGGTTTTTTTTATTGGCGTTTAGTAGT

GCTTTTTATTAAATATAAACCTATAAACCATATAACACGTTTTTCTATACCTGTTTTTAA

TTCAGTAGGGACAATAAAATGTATAGAGGTGGTCTACTATGCGTAAAAAAGAAGATAAAT

ATGATTTTAGAGCCTTTGGTTTAGCCATTAAAGAAGCTCGATTGAAACGAGGTTTAACTC

GTGAACAAGTGGGAGCATTGATTGAAATTGACCCACGGTACTTAACTAATATTGAAAATA

AAGGGCAACACCCCAGCATACAAGTTCTTTATGACCTTGTATCGTTACTTCATGTTTCCG

TTGATGAATTTTTCTTACCTGCTAATAACTTGGTAAAAAGCACCCGACGATTACAGATAG

AGAAATACATGGATAGCTTTACAGACAAAGAACTATCCTTAATGGAATCTTTAGCCAGCG

GTATCAACGAAGCAAGAAACATCGAAGACTAATTAAAAGAATCCATACATAACGGAAAGA

GCCGATAAAATGAGATTGTATTAATCTCATTTTATCGGCTCTGCGTCTTTGCGTCTGGCT

CTGTAATCACAGTTACTTTGAACTGCTTTATTTCAATTAAATTTTCTTGTCTGCATTTCG

GACAATAGAGGGGGAATTTTTTTAATTCAGTATCTTCCCTTATCTTTAATCGTGTTTTAT

TTCCACATACAGGACACAATATCCACTTGTAGTTTATAATAACTATCTCCTCCTTTACAC

TTTAATTCAAATCTTTATTAAAAAATATTTCATCTTATTTAACAAGAAACCATATTTATA

TAACAACATAAAATACACTAAGTTATTTTATTGAACATATATCGTACTTTATCTATCCGA

CTATTTGGACGACGGGGCTGGCAAACAGGTTCACCGGTAGTAACATGGTACCCTTTTAAC

TCTGTTAAACAAACACTACGTCCATTTGTAAAGAAAGTTAAATCACTACGATATTCTTGA

ATACACCGAGCAGGGATTTCTCCACTAAGAATGACCTCATTATTTTTCAATTGAGTGTCT

ACGATGTTCGCACAATATTTAGGAGCATCGTTGTATGCTCGTGAAAGATATTCCTGTGGC

GCATAAATTTTAAAACTAAGATATGGCTCTAACAATTCTGTTCCAGCTTTTTTTAAGACT

TGTTCCAATACAATAGGAGCAAGCATCCGAAAATCTGCTGGGGTACTAACAGGGCTATAG

TATAAGCCATACTTAAAACAGATTTTACAGTCCGTCACATTCCAACCATACAATCCTTGT

TCACAGCCATAGCGTATCCCCTCCATAACTGCATTTTGAAACGATTGATTTAAGTATCCA

AGAGAAACCGAGCTCTCATACTGTACTCCGCTCCCTAATGGAAGCTGTGCTACAGATAGA

CCAATGGAAGCCCAGAAAGGATTCGGTGGAACTTCGATGTGAATGGTATACTCTGCTTTT

TTTAACGGTCTTTCCATATAAATGACTGTAGGCTCTTTTATTTCTATCTCCACATGATAC

TTTTCTTGCAGCAGAGCACAAGTCACTTCCATTTGTACTTTCCCTAAGAAAGAAAGTATG

ATTTCATGTGTCGCAGAATCCACATAATATCGCAGAAGCGGGTCACTGTCGGAGATTTCT

AAAAGTGCATCAAGTAACATTTCCCTTTGTTGAGGTTTGCTCGGTTCAACAGTCGTTTGC

AGCAGAGGGAGGGGATTTTCAATTCTCTCTCTCTGTGGCAATAGCTTTGTATCTCCAAGA

ACACTATTTAACTTCAAAAACTCATTCTGCAAAATAACAATTTCCCCGGAATAAGCCTTA

TCGATTTTACATAATTCACCATTTATTGAAGTATACATTTCTGTAATTTTTATTTTTCCT

TTTCCGATATTCTAACCGAATCTCGCAAATGCAGTACGCCACTATAAAGACGTATATATG

CAAGACGCTGTCTTTTTTCCGAATACTCAATTTTGAAAACATTTCCGCAAAGTTCAGACG

GACCTCGATGTGTTGATGAATAAAATTTATTAGTAATAACTTCTATAAGGTTATCAATCC

CTATATTACTTTTTGCACTTCCATGATAAAGAGGGAACAGAGAACAATTCTGAAATCTTA

TGCTTTCCTCTTGTTCGAGTTCCAATGCTTCTAATGATTTACCGGACATATATTTCTCTA

AAAGGTCATCGTTTCCCTCTATTACCGTATCCCATTGTTCAGATTCGGTAAAGTTCGTCA

CACACATATTAGGATACAGTTCTACCTTCTGTTTGATTACAATTTCGGCAGAAAGTTTCT

CTTTAATATCCTGATAAACCGTTGATAAATCAATTCCATTTTGGTCAATCTTATTGATAA

AAAAGATTGTGGGAATCCCCATTTTCCTAAGTGCATGAAATAATATACGAGTTTGTGCTT

GTACGCCATCTTTTGCAGAAATCAGTAGAATTGCCCCATCTAAAACTGATAATGAACGAT

ATACTTCTGCTAAGAAATCCATATGTCCTGGCGTGTCTATGATGTTCACCTTCGTATTTT

CCCACTGAAAAGAGGTTATTCCTGTCTGAATTGTAATTCCTCTCTGACGTTCTAAAAGCG

TATTATCCGTCCTCGTTGTACCTTTGTCCACGCTTCCTAATTCTGTAATCGCTCCACTGT

TATATAATAAGCTTTCTGTTAAGGTAGTTTTTCCCGCATCAACATGAGCTAAAACTCCAA

TATTAATAATTTTCATGTGATTTTCCTCCATTCAAAAGCCCAAAAGGGCATNNNNNCACA

CACTTAATTAATTAAGTGTGTGNNNNNTTTTTATGCATAACCATAGGCATACAAAGCATA

CAGATATTCTCCGGATACTTTAGAATCACATGATAAAGGTATTCTTAAACTGGGTACAAA

AAACTAAGCCCTCCTAAAAAAGGACATCCAATTATTTGTTCCCACTATCAAATTGACAGT

TTATTTAAGAATACCTTGCCGCATATTTATTAACTCCTTTTAAATAGATACTTAAATAAT

AGCACGTAAGAGCATATTTGTCAAGGAATCTCCAATTTTTTATCAAAGAGAGTACGTGAT

TACAAAATAGCTGTAATAATGTACCAATATTTGTTATTCTATAATCTTCCAATTACTCCC

GTTCTTTTCAAGTACCAAATCAAATTGAGATACCTGCGTTGCTTTGGTCTGCTGGTCGAT

ATACTCCACTGTCAGCGATACCGTGACTTGATTATCCTTACGATTGTGAATAGGATTTAC

CAGTTCTTGAAAGATGTACTCTTTTCCGATTGGTTTTAATATCCCGTCATTCACATAGTA

GGAAAGTTCACTGGCTGTCGCTGTAGGATAGAGCTTGAAGAACGTCGTTAAAAACTCATT

GATTTCATTGGTTGTAATGGAATCAACCGTCCCCTCACTTTCAATGGCTTTTGGTTTATA

ACTTGATTTCTTAGGTATGTTGGTAATGGTCGGATTCTTAACCAGTACCATATTTCCAGA

ACCATCTACATAGACACTCACTATATAAGCAGAGTGGACGGTCTTTGTATTTTCTCCCTC

TGTAATGAGCTGGTCTACACTGTAGGTTACATTAAACTCATTGTCGCCAGTTGGCTCTAC

CGTCCATATCTGAAATCCTCTTACAGAAGACGATACAGGAATATCTTTGCGTACTGTATC

AACATTGAGAGCTTGAAGTTCATCTGTCAGATAGCCTTTTAGACTTTCCATTCGATTATC

AATGGACTTATCGGATTGCTCCCATGAATAGTAGACTTTCGCAAAGTTCTCTACAAAATT

TTCTACATGATGAGTATCAACGTATTCCTTTTCTATGATAGTTGTTTCGTGAATAGTATG

AGTATCTATAGCTGTAAAGTGCTTGAATATCGCAAAGCTGAAACTAAGCCCTAAAAGTAC

CCACAAGGCAATCACAACCTTTTTATGAGGATTGACCTTATAGTAGACACGAGGTTTCTT

TTCCTTTGGTATCTGTTTTTCTTTATTCTGATTTTTTCTAAATTTCATCATTAAATCTTC

CTTTCTCATTGTTTGATTCGTCCTGCTCCCACTAAATGCTGTTGCCAGTAGGGGCTTGTT

AAGTCGGCATAACCGATTGGGTCGCCTGCATGAAACATACGGTTATTGCCAAGGTATATC

CCAACATGAGTAATATAAGAGCCAGCGTTATAGGTAGAATGAAAGAAAACCAAATCGCCA

GCTTGTGCTTCCGATAGTGGGATATGCTGGGTCACATCATATTGCTGTTGTGCGGTTCGT

GGTAAGTTAATTCCAGCTTTTCCATACGTCCATTGTGTCAGTCCGCTACAATCAAAAGAA

GTAGTCGGGGAAGCTCCACCGTAAACGTATCGCCAGCCCTCATATTTCAGTGCTTCGTCC

ATGATGGCTTGTACCGTATCATCATCAAACTCTGTTGTGACAAGATACTGCGTTACCAGT

TGCACATAAAACATATTGCCATAGTTGTATCGCCAGCCCCCATTGATAGGTATGGCTATG

GGATTGGGGTAAGACACTTTTTCGCCACCTGAATACTCTTTTGAGAAACTTTGAGCCAGT

TCAAAGGTATATTTATTTCCACGATTAGCCACATACCCTAAGAAACCACCACCATAATTG

TAGGACTGGATAACCGATTCTAAATCTACACTGAGCCTTTCGCTACTGGCTAATAATTCA

CTGAAATACTTCACACCTTGCTTAATGGATTCTTCTGTACTCAATGAATTAGGTGGAAGA

CCGAGGGATTCCGAGGACTGCATAACATCTTCCGCAGTACCGCCCGATTCCACCTGTATA

ATCGCAAGAAGTATGTTGACATATTCTTCAACGCCATATTCTTTGGCATATTTTTCTACC

ATAGGCTTATGAGCCAGCACTTCTGCGGAAACATTCACACCTCCATAATGAATATTGGAA

ATTCCGCTGTCCTGTTCATCTGAAAATAAAATGGCAACAAACAGAAGCAGTGAGAAGACC

ATCAAGAATAATCCAGAACCACCAATCACTAAAGTTTTCAACTTCATGGTTTCTTACCGA

CTTTCTTAATGGTGGCGGTTTTGATTGGTGGTCTACTTCTTGTATTTTGTAGTGGTACTC

TTTGAACAGTAGACGGACGTTCTTTTGTGATTGGACGTTGTGAAGTTCTATCTGCTGTAG

TGGTTGAAGTTGCTGGCTTTTGAACGGTTTTTTCTTGAACGGTATTGCCTTGGCGTTCCA

CTTTTGGACTTGAAAAATCGGACTTAACTGCTGGACGCTCTTGTTTGGCTTGTTGAGATT

CCTTATATGAAGTCTGAATATTAGACTGTTTTGAGGTCTGTTCATCATGATATTGTTCTT

GTCTTGTAGTCGGTCTTTCATGAACAGAAGAAGCAGGCTGTTTTTTCTGTTTGACCTGTT

CCATTTCAGAGCGACGCTTCGCAATGGTTTTTCGCCTTTGTTCCTGCTGTTCCTTGCGTC

CACTGGCTCTGTCCGCTTTGGTTTGAGAAATACTACTGGTTAAATCACGGACATTCTCTT

TTACTTTGGATTTTCCTTGATATACTGCATATCTTGCATTGGTCGGCAAATCTTTAACCT

GTTCTTTCAAACCACTAGCAGTGTCTACCATTCTGTCTTTGGTATCAGCTACTGTACCGA

TGGTTTGACCGATACGTTTTCCAAGTGTTGATTTTTCCTTTCCGTCTGGTCGGGAGTGAT

CTGCTTGTGTCCTTGCAGAACTCCCCGAACCCGACTGTCCTTTTTTACCTGTAACAATGG

CAGACCCAGCCCCTAGAGTAGTCATGGAACGTCCAAGTTTCCGCTGTAGACGGTGCATGT

GAGCGTGCATAAGCATACGAGGTTTTCTCATCACACGACTTCCCACACTTTGAGAATCGT

TACTCTGTAGAGAAAACATACTCATTAAATCGCCCAGCTTGAAGTAGATTCCTGCAAAGG

TCACAATCTGTAGAAAAGCAATCAAAAAGAACGGATAACCAGCCGATAAGGTATAGAGCA

TGGTTGAAATACTAAATGCTGTCGTAATAATCAATGTGATTCCAGCTCGTGTCAAAATGG

TATTAAAGAGCTTTGTTATGGCTCGTTTTGACATACCATCAAATGATGGAATCATGCTTA

AAATAAAGCTCACAGGCAGAAACATAGCATAGATGATAAAAAGTACCTGCGAGAAAATCA

TGATTCCTGTTAATAGGAATACAAATATGGAAATCCCAATATTGAAGACAAATAGGAAGA

AGACTGTACCTAAACGGTTAATGGTCTTTGTAATGGTTAGATTGGTATTGCTTCTGTCTT

CAATTTCTTCCGCAACAATTTTTTCTCTGTCTTCGCCATTGTTGGAATCTGGGCTGGTGG

AGAGCAGGCTTTCCACACGGTCAATACCGATACTTTCAATGTCTGAACTGTTGTATTGAA

GCAGTAGCCACGGTTGCTGAACCTGTATGGAAAACAGGCTATCTCTGATTAAGTCCACGC

TGTCCTTGCCTTGACTATCGGAATGGGGCATGACAATCTTCGTGCCAAGTGATAAACTGG

CATTACTGATGTCTGATGAAAAGTCATTGATTTTTTTAATGTAGTCGGGAGCGTAGGCAA

TAAAGGAAGCCGATAGGATAAACACCAGCACAAAATTCATAATGGCATGAATTGCCTTTG

TGGTTTCTCTCTTTATCAGTCCCGTATAGGCAACATAAACCCCAAGAACCAAAATCAAGA

GTAAGAGGAATCCAACATAGAAACCCTCTGTTGAAAATCCGTTTGCACTCACACCAGCTA

AGGTCTGCATATTCTTACCAATGGAATCTGCTGTAGCGGAAATGAAGTCTAAGGAATAGG

CTTCCTGTACTAAGTAACCTGTCGCATTGGAAACATACAAACTGATTGTCCAAATAAAAT

TGGTAATGGCATATAGTCCATACATGACCTGTTTTCCAATCCCGTCCGACCAGTTCCACG

GAAGCCAGCCCCAGCTATTATCCACATAAAAATCCAGTTGATAGTTTTCAAGTGGGTATC

GGCTGTATTCATTTGCCACATTGACCGTATCATCTACCAAGCCCGCAGCTTGAACCACCG

TTCCCAGCATGGCTAAAAGAAAAATGGCAATCACAAGTGTGAAAGCCACTGTCATTGCCA

CTTTACCTAGACGTTTCAGCGTCCAGTTTGATTTTATTCTGTTTACTATTGATGGTTTCA

CATTTACACCTCTTTTCGCACAGGTGGTCTGGTATCAAAGGCATGGAGCAGTTCTTCAAA

TACAGGGTGGAACTGTATCACACCGACACGACCATATAAATCACTGATAAGGCATTGCCC

GTTTTCCAAATCACGCAATCGCTTCTGATTGTTTTCGTCCTCTGGGTCTACACCAAAAAA

GGCTAAGGTCTTTTTAATCTCGTTAAGGTCAGTGGAACGAAATGCAAATTTTAAGCCGAG

GTTATTTTTCAGTTTTTCATCTAAGAGGTCGTCTGTATTTTGGGTCACGAAATATACCCC

AGCGTTCATAGCACGACCAGCCCGAACCAGCTTCATAGATAGTGTTTTTCCTTGTGCTAC

CTGTAAAAAGCTCCATGCTTCGTCTAAATCTACAATCTTGAAAATGCTTCGGTCTGTATG

GATAAAGTCTAAAGCAAAGGTACTAATGACAATCAGCATAGCAACGGATAAAAGCTCCAT

AGTGGTATATTCCTCAAAGGAAGTTTCCTTGTCGGGAAGTACCAAGTCCGCAACCTGTAT

AATGTTCAGTTGTTTTTCTAAGCTGATAGACTGCTCCACATAACCATTACTGAATAATAA

ATGTGCAAAGTCATAGTCTGTAAAACTTTCGATATGGTCGGCTATACTGGTACTTAGTGG

CGTATTCTCAACCCGTAATTCCTCAATCACTTTCATCAACCCTCGTACTTCACTATTGGT

TACTGCACGAATGGCTTTTCTAAGGATTGGGAAGCGTTCCCCATCACGAGAGGAAATCCC

CGTAAGGAATGTCAGAATATCAATAGCCAGTGATTCAGAATCTTTGGGATTTTTCATAAT

CACATAAGGGTCAAGTAAGCCTTTGTTTTTCTCATCAGAAGTCAGAGTGACGATATTGAT

TTCATGGGAAATCTCTGGCAAGGTTTCTTTCCATCTGCCACGTTCTGCTTTTGGGTCTAC

AATCACTGCTTGTGCCCCATAAAGCACCGCATAATAGACGATAAGGTTATTCGCAAAGGA

TTTACCACCACCCAGCGAACCAACAAAAGCCGACGCTAACGCATTGGTTACTGAACCCTT

AACCCCTTGACTGGCAAGAGCAGGTTTCAGATAGACATTGCGTCCAGTATCTAAGCTGTA

GCCAACATAAATCCCCTCATTTTCCCCCAGCATTTGAGTAGCACCAAAACCTAAACCAGC

GAGGAAATCAGAGGTCACGTATTGAATATAATCATTCATATAACGCTTGCTGGCAGGTAA

AAATTCTTCATGTAAGCCGAGCATATCCCCAAATGGTCGTACCAGTTTTACGCTTAAATC

GTCATAAAAATCTTTCACTTCATTACAACGACGTTTGAGTTCGTCAAGATCATTTGCTGA

TACCCTTACCACATAAGACAGCTTGTACATAGATTCCTTGCTTTGGTCTAAATTGGTTTC

CAGCTCATTCACACTTTCCAGAGCTTCCGCCACATTGGAGCTGGTTTCATTATCACTTTG

CCAAGCGTGGTTATCCAAGTCTTTCAGTTCTTTCTTTTTATTGCGGACAGTAGATAGGGC

TTTACGATTCGCTACAATTTCCACATTCATTGACGTATCAATCGGGAATGTAAATTGCTG

TTGCTGGTAGTAGAAGATTTCAGAGGACGGGAAGTCCAGTTCTCCGACAATGCTGTTAAT

GGTAAAGTAAGCTACATAGACGGTTTCATCTTCCTGCTGGATTTTCAAATATCGCTGTTT

TTCTTCCACCAAACAGCGAGTAGGCTTAATCAAGTCATAGTATTTAATCAGCGTTTCATT

ATCCAGCTTTTTCTTTGATAGATGGTACTCATACTCTTCATAGGCAGTGCCTGTCTGTCC

GTAAAGGTGTTCAATCAGATAGCCGAAGTCGTCCTTATCTAACCTGCGGATTTTGAAACG

ACGAGAGATTTTATTTTCTAAGAGCTTTTCCATCTTCTGAAAACGCAGGATTTCATCATT

ACTCATACTAACAAAATCGCCCATCAGCTTATGGTTCACATCATAGACAAAATCAGACAA

AGCATTTTTTGCTTCAACGGTAAGACTTTTCATAGAAAACTCCTGATCGTTGAGAAGCAA

CTTAAAGCCGATAAAGAAACGGTAGTTCACTTGATTTTCGCCAATCATGGATATTAAAGC

GTCTGTCTGTTGGTCGATTTTGTCATAGGCAACCGCTTTGAGCTTGCCAGTGACTTCATT

TTTGGAACGCTCTTGTGCAGAACGTATGCTGGATTCTGTACTGATTTGTAAAGCATGAAT

TTTGCCATCACGATTTTGTGCGATAAGCTGTCTGAAAGAATCATGCACTTGTATTTTCTG

TTCTGGACTTAGAAATGAGTAATTGTAAGGAACAAGCTCATAGTAAGCATAACATTCCCC

GTCTTTATTCCAGACGAGATTGTTTTCAATGTATTTAATTGGATATGCCATAAAATTCAC

TCCTAACTGCTGTAATGGCTTCTTGTGGCTGGTTTCTGCCAAGCGTTACTTTTTTCCTGC

ATAGGTCAGCTTTGGTCGCAGTGCATAAGCAATGACAGACTTCAAAAATCCATAAGGCTT

TTTACCATCAAAAGTTTTTGTAGACATAAACCATGTGAAAGCCACAGGAATCCCAAAGTA

TTTGAGAAATGCTCCCTCTATCATGGAAAGAGGGGGCAAGTTGCCAAGTATCATCACTGC

AAAGAGTGACACGACAAACCATGTCATTTGCGTAAAGGTTATGGGAAACGGAAGTCTAAA

ATCATTGATAGAATACAGTACCTTTTCCACAGACCAGATACTGGTATAGCTTCGTATTTT

CTTCATGTAATCAATCCTTTCATAAAAAATAGGGGTAGCTGATTGAGCCACCCCGTAAAA

TAGAAAATCTGCCAGTAGTAATGTACCGACAGATTTAATAGACGATTTCAAAAATCCCAT

GATTGGTTGAGATAAACGTTCCTGAAAGGTCTAAATCCCGACCATAGGCTTGATAATCAA

TATAGTTTTGAAGACTAGCTGGTACTTCGCCTAAAGCACCCGTTTCTTCAATGTAGTAGC

GTGCCACGTCATACATATCATCACAATCGGAATGAATGATAATATCCTCTTGATGTTCGC

TTAGTTCTTCAATGCTTGAAAAATGAGTGAGCAGAGCAGATAGCTCCGATTGTAATTCTT

CGGGTAATTCCGATACCATTTCCCATAGTCGATTGAGTTCGCCAATGGAAGTGTATTCGT

CAACCGTAAAGGGTAACTCGTAGTCATGAATGGCGTATTCCTCATATTCATCATTCAAGC

CGATTTTCTCTTTGACTTCCTCAAAGTCAATGGGAAAGGTAAACCACGCACCGACCAATT

CGCCCTCATTGTATTTGCCTAAATTCGCAATATAGACTTGCATATCGTCCATATATTCAC

GTCCTTTCTTTGTAGAGATTCAAAAATCCCTACCGCACTTCGTTTGGTGTACCATTCCTT

TGCGGAACATAAGAAAACCACTTATATTCCACAAAAGAACGGTTTTATTTAAGCACCAAT

AATGCGATTGAATAGCTCTAGTAAAATGTCTTTTACTCCAGCAGCGTTGAAGACTAAGCC

AACCGCAATAATCGCAATAATTAAAAAGCCAATCAGTTTGCTAAACTCACGCTTGAAGCC

AAGATACAAGCCAATCACAACGATTGCTAAAAGCACCAGTGATTGAGCGTTTGATAGAAA

CCAGTTATAAAGGTTTTGTCCAAAATTCATAAAAATGTTCTCCTCTCTATATTCAATGAA

TTTGTATTTGAGTTATTTTTTGGGGTATACACTTTCTGGTATGGATAGAGATTCCATGCC

AGATTTTTTTATACAAAAAAGAGGACATTTGCTGTCCCCTCGTTATACAATCAATTCACC

ACAAAAATCATGAAAGAAGGTTAAACAAATGTCCCATAATGATTCTATCCTAAATATTCT

TGGAATTAAAGATAAAAATATTAAAATTATTTCTGTTGAAGAAGCTGAACACAACAACGA

TTCTGTTAAAGAGTATATAACGCTAATAACAGCTACTCTTTCTTATCCGATTAATCGTTG

TCGTAACTGTGGCTTTCCCACAGTTAATAAGGATGGCTTTCGCAAAACTCATGTACGACT

GGCAAGTTTAAATGGGAGAAGATATGAACTAGAGCTTCGTAAACAACGCTATAAATGTAA

ATCATGCCATACTACTTTTGGTGCTATTACTAATTTAACCAAAGAAAATCAAACCTTATC

CAGTGATCTCAAAAATCAAATCATGCTTTTAGCTCGTAAAGGCTTATCTGGTCAGCTTAT

TGCTGAAATGTGTCACTGCTCTCCTAGCAGTGTTCGTCGAACAATCTTAGAGCGCATGGA

ACCACACTATCGTGTGGCTAAGTTGCCTAAGCATCTATGTTTTGACGAGTTTCGTTCAAT

TAAGTCTGTGATGTCCTTTATCTGTTGTGACGCTGAAACCCACCAAATTGTCACAAAGTT

ACAGGATCGTCTATCACCTACCATTGTTGATTATTTTGAAAGTCGTTATTCAAAAGCCGA

ACGCGAATGCGTTCAATCAGTTGTAATTGATTTAAATGCTCAATATCAAAGTTTTATCTA

TCGCCTTTTCCCTAATGCCAATATCATTATTGATCGCTTCCACCTTGTACAATTAGCTGG

TCGCGCTTTGGACAATTGTCGTATCTCTATCCTAAAGCAACTTGATAAACAGAGCCAAGA

ATATAAAATTATGAAGTCACATTGGAAGCTATTCCATAAAAAAGCTGAAGATCTTCACCC

TGAAGAAGTAGTTTTTCTTCGCGGCGTTAAACAATATATGACTCGCCAAAATGCTGTTGA

TCTCATTACTAGTAAATTTTCCAAGTTCGCTGAAGTATACCAAACTTACCAAGATATCAC

GAAAGCCCTAAACGAGCGCAATAGTGAATTACTAGAGTCAACCATCTTAGACTACCAAAA

AACCAATACAGAAATGGATACTGCTATTCAAACCCTTCGTCAAAACAGAAAATATGTCTT

AAATAGCGCTAAATTTGAATACTCTAATGGTCCTTTAGAAGGCATCAATCGCAAAATCAA

AACCCTAAAACGAACTTGTTATGGTTTTGCCAATCAAAAATTTTTCTTTTTAAGAATCGA

TTGTATTTTTCGTAAAAAATACCCCCTACATTTTCGTAGGAAGTATTTTTAGTCAACCAT

ACCAGTTGACAGATATCCCACTTAGGACATTTTCCTACAAGGGGTCCCGAGCGCTTAGTG

GGAATTTGTACCCCTTATCGATACAAATTCCCCGTAGGCGCTAGGGACCTCTTTAGCTTC

TTGGAAGCTGTCAGTAGTATATCTAATAATTTATCTCCATTCCCTTTAGTAACGTGTAAC

TTTCCAAATTTAAAAAAGCGACTCATAGAATTATTTCCTCCCGTTAAATAATAGATAACT

ATTAAAAATAGACAATACTTGCTCATAAGTAATGGTACTTAAATTGTTTACTTTGGCGTG

TTTCATTGCTTGATGAAACTGATTTTTAGTAAACAGTTGACGATATTCTCGATTGACCCA

TTTTGAAACAAAGTACGTATATAGCTTCCAATATTTATCTGGAACATCTGTGGTATGGCG

GGTAAGTTTTATTAAGACACTGTTTACTTTTGGTTTAGGATGAAAGCATTCCGCTGGCAG

CTTAAGCAATTGCTGAATCGAGACTTGAGTGTGCAAGAGCAACCCTAGTGTTCGGTGAAT

ATCCAAGGTACGCTTGTAGAATCCTTCTTCAACAATCAGATAGATGTCAGACGCACGGCT

TTCAAAAACCACTTTTTTAATAATTTGTGTGCTTAAATGGTAAGGAATATTCCCAACAAT

TTTATACCTCTGTTTGTTAGGGAATTGAAACTGTAGAATATCTTGGTGAATTAAAGTGAC

ACGAGTATTCAGTTTTAATTTTTCTGACGATAAGTTGAATAGATGACTGTCTAATTCAAT

AGACGTTACCTGTTTACTTATTTTAGCCAGTTTCGTCGTTAAATGCCCTTTACCTGTTCC

AATTTCGTAAACGGTATCGGTTTCTTTTAAATTCAATTGTTTTATTATTTGGTTGAGTAC

TTTTTCACTCGTTAAAAAGTTTTGAGAATATTTTATATTTTTGTTCATGTAATCTCTCCT

GAAGTGATTACATCTATAAACAAATACAGAAGTTAAACGATTTGTTTGTAATTTTAGTTA

TCTGTTTAAAAAGTCATAAGATTAGTCACTGGTAGGAATTAATCTAACGTATTTATTTAT

CTGCGTAATCACTGTTTTTAGTCTGTTTCAAAACAGTAGATGTTTTATCTACATTACGCA

TTTGGAATACCAACATGACGAATCCCTCCTTCTTAATTACAAATTTTTAGCATCTAATTT

AACTTCAATTCCTATTATACAAAATTTTAAGATAATGCACTATCAACACACTCTTAAGTT

TGCTTCTAAGTCTTATTTCCATAACTTCTTTTACGTTTCCGCCATTCTTTGCTGTTTCGA

TTTTTATGATATGGTGCAAGTCAGCACGAACACGAACCGTCTTATCTCCCATTATATCTT

TTTTTGGATATCTGTCAACTGGTATGGTTGACAGATATCCTTTTTTTGTTGTTATCACGT

CCTGTTCTTTTACTGACTGTTGCTTCAAAATCTGCTTGTGTCGGTCTGTCAGTTTCGCAT

GGTCGAGAATGTCTTTTACAACCTGCGTCTGGTTGATTTCATCAAGTTTAATCGCAACCT

TTAAGGTCGGGGCAACTTGATGAGATAGCCAGTTCAGCGTCCTTTGGAAGGAGTAAGGCT

CTGGTTTTGTGGTTAGTTTTAATCGTTCACGATTGTTCCCAATAAACCAAGCCCATTCTT

CATTCAGTTTCCAATCAGAACGAGGTTTGGAATCGTCTTTATCTACAAAACGGATATACC

GATTGATAATTTTAAAGGCGGTATGCTCTGGATTGTCATAGACGAGTAAATCACGGACTG

CATAATAGGCACGCTCATTTTTCAATCGAATCTCAAAACGGTTTTTTACTTCTGCGTCTT

CAATGGGAATATCATTTTTCTTGTACTGCTCGTAGTCCTTTTCATAGATACAGAAATAAA

CTTCACTTTGTAATGAACCGATATAGAGGGTGTTTCCCATACATTCCTTTTCCTCTTTGC

GTACCAGTTCGCCACTGCGATAGCTTTTAAAACTGCGGAAGACGGAGATACATTCTTCCT

GTTGGCACTTTTCAGTGAGTACAGGGATATTTAAAATCCCTGTCTTATCGTTAATGGCAA

GGTCAAGGCGTTTCATCACACCGCCAGCCACCAAAACGTCCATAAAGAACTCATACCAGC

TTCTTTGTTGTGCCAGAAGATAGCTTTCAAATTGTCTGCACCCACGACCTTTCAATTCCA

CCAGAACTCCTTTGTCCAGTTCATGGGAGCAAAGGACGAATATGTCGCCTAAAGCATAAT

GCTCTGAATAAGAATAGAAACCATAGTCCTCATGAAGAAAATAGGACAGTTTCAGTTGTA

AGATGTTTTCGACCACCTGCTGTACGTCTGTTGTCGGAAAGCGAATTCTTACATAATCAA

ACAGCATTTCAAGGGGAGCGTCGGGATTGAAGCGTTCCAGAGCTTCCCAAAGGGACTGCT

GTAAATCCTCTGATGGCTTGACTTTTCCTGTTTCAATATCGCTTAGATACTGCCTTGTAA

TACCAGTCGCAACAGCTAAACGGTTTTGAGATAGTCCATAAGCCAAGCGTTTTTCTTTTA

AATGCTGTAACCAAGTTTGTTCATTCAGTAAAAATCCCTCCAATCAAAAAGGCGTATGTC

AACTTTTAAAGCCCATTTGACATACGCTGAAATTTTGTAAATCCCTTGTAACCAAAGGAT

TTTCTAATGTTTTTTTGACTGTTTCCTGTCGATTTGTACCCCCCTGTTAGATACGGGGGG

GTAAGTGCTGGCGTGGCTATTGCCACACCAGCCAGCAAGATCAGTCCACACCTGCGACTT

CCGCTTCGCACGTCGCCTGCGTGGACTGTCTGCTGTTGGATAACTTTTTAATTTCCTCCA

AGAAATCATATCCTTTTGGTACAAGGGGAGTATAAAACTCTGATATGACACTTGTTCCTA

CATCAACATAGCCACGACCTTTGATTCGCTTTAAGAAGAAATCCTTTTGTACGTCACTGC

CAAACATCATGCCATAGCCCATTTCAGACATACGACCTAAAGCCACTCTGAAATTAAACT

GATCACGGATTCCGTCGCCTAAATATTTTGCGTCTGGACGTTGACAAGCCAGTATTAGAA

AGAAGCCAGCTTGACGACCTAACATGACAATCTGTTTCAGCTTATTCATAACTGCGGTGT

TTTCTTTTGTTCCCAGCATTTCCATGAAAGCGACGTATTCATCAAAGATTAAGAAGTGTG

CCGGGAGACCTAAGTAAGCATAATTTTTGCCAGTCTTATAGTTCTTCATCTGCTTCATTT

CCTCACTACGTTTCATCATTTCTTCATAGAATGTTTCAATGCAAGAAAGCAAGTCTTCTT

TTCTATAGTAGACATTTGCCATCACAGAACCTAAGTCCGCAAGGTCAGCATTTTTCGGGT

CAAGAATATACAGTTTTGAATCTGTATGAAGCAAGGCTTCAATCAGTGTCAGTATAAAGT

AAGTTTTACCGCCACCTGTACCACCAGCAATCAACATATGAGGGAGCTTATCATATTCCC

ACCATACGTTTTTCATTAAGCGAAGTTTACCATCTTTAGCTTCTACTTCATCAATAGAAA

TACGACTGGCTATGGTGTCATAGAGCAAAGTATATTCCACATAGGAATCCTTTAACTCTT

TATCCGTCAGCTCACAGTACAAGCCACTCTCTAATTTCTTTTCCAAGTGTAAGAGTTGGT

CTTGATATTTTCCCAGCGTGATTTCCACCCGTATCTGTATCAAGCCATTTTTAAGTCGAT

AATACATTTTAGGGAAGTAGGTTATCTTTTCCTTTGTACGACCAGCACTATCTTTAAAGA

AACCCTCTGTTTTGACCTGTTCAGATTCATACCACTTGTTTTCAAGTATCATCTTTGCCA

GTTTTTGACGGTGGTAAAGTTGTTTAACCGTATCATAGCGAACCCGTTTGAATACAAACG

CTACCAGCAAGCAGATAAGAATTGCGACACTGAAACTGATAATTAAATAGGGAATGTCAA

TCTTATCTGCTTGTGATAGGTTAAAATCCTGCCAGTTGATCTGCTGGATTGTCTTCACAT

GAAACAGTCCGACAACCAGCAGGAAAACAGGCAGGAGTGACGCTATCGTAAAATGAAAGA

CTAAATCTTTACCAGATGGGCGAATCCTTTTACCACGCTGTTTCATGCGAAAAAGTCTCC

TTTCTACCTAGCGACTATTTGTCTTGTGTCGGTTCTTTCTTTGCTTGTGGTTGAGCTTTG

AATGAACTAGAATCCTTTGTCAGCACAATATCGTCTGCCTTGATATACCAGTCAACATCT

GCTCCTTGATAGGTGGCAGTAGCAACGGTGTCCGCAATGGGATTGATAAGTTCCACCCGT

GCGTTATAATCAAACTCTTTCAAAGGCACGCTGGCAGGAATACTTACTTGAATCATGCGT

CCTTGTCCTTTGGATTTTAAGTCATAGGTACGTTCCTTGATTTCATCTGAAACCGACCCG

TCTTCATTTTGGATTCTCACTTCACGACGTAGAGCAGAGAATTTCAATTCTCCAAAAGTC

GTGTCTTTATCTAATACAATGCCATTTGCTAATCTCATCATTTTTCCTCTCTTTCTTTAT

TCTTTTATCATGTCGTCAGCATGTAAAAGGTAATTTGTAAAACCACGAGTGCCGATTTTG

TAGCCCTCTGCGGTAATACGTGGATTGACTAACTTCACACGTTCCTCAAAGCCGAAATGT

TTTTCGCCAGCTTCAGCAGGAAGCACCACCACAATATCATCTGCTCTTTGAACATCAGAA

TAGAGATTATAGCTTCTTGATAAGACAGTTAGCCGTCCGTTGATTCTTCGCTGAACGACT

TTATCCTCGCCAGCAAATTCTAAATTGCCGAATGTTTTTTCCATGTTGGGAATCACAAAT

TTAAGTTCCATATTTTTACCTATCCTTTCTTTTTTATTGGCTGAATGAATGTTTGATGGT

CTTAAAGAGTGGGGAACGACCTTTTGATTCTTGATTTTTTGTTTTCATAAGTTCACTTCC

TTTCAAAATCGGGTAAAAAAATAGACACCTCATTTTTTGAAGTGTCTACCTATTAAATAT

TCAAATTTTATTGGAAGTATCTTTATATCTTCACTTTTCAAGGATAAATCGTCGTATCAA

AGCTCATTCATAAGTAGTAAATTAGTAGTAAATTGAGTGGTTTTGACCTTGATAAAGTGT

GATAAGTCCAGTTTTTATGCGGATAACTAGATTTTTATGCTATTTTTTATATAAAATTTA

TATATAGTTTCTAAACTAACATACTCAATATTACCTCTAGTACCAATAATATCTATTGAC

CAAGAACTATTTTTAAAAATTACTCGATTATCATTAATAGATTTTTTACTATTTTCATAC

TGATTTAATTCAATTAAGACTATTTTCTTTTCCCTCAATAGTTCATCTTCAGTAATAGAA

AGATTGAAAATCCTACTTATCAGTATTTTAAAAATTTTATTGAAATCTTCAGCTAGACAG

TAACCACTTAAATTAATATAATCTCTAGATGTCTCTCCTTTAATAGCTGCACCATTCATA

TCAAACAATTCATTTAGTGATTGTTCATTCCCTGCTCGTATTAGTAAATGTTCAATAAGA

TGTGAAAATCCTAATTCATTTACTTGCTCAATACTAGTACCTGCAACAAACATCAATGAA

AAATATGCAAATTTACTTGCTTGATTTTTTTTAAATATAATTTTCTTATTCATCAATTTT

TAGAATCTCCCAGAGTTTACTTTTATCGAAATCATGACCACATGTAGGACAATGAGATTG

CTTAGAGAAGTGCTTGAATTCATCTTCCCTAAAACTATAAAAATCAAATCTAAATAAAGT

ATTCCAAACATTACTATTTTTCTCTGTTACACAATCTAACCATTTCTTCACAACCCATGA

TGACAATAATAGAATATTAGTTAAAATATTTGCAGTTATTAATTTTCCATCATAGTTTGA

AAATTTTTTAACTAAATTTTGAAAGTTATCACTATCTTGTTCATAAAGTAAACAATCAAC

ACAGCCTATATTTTGAATATTAGGATTACAAAATAAGAGTTTACCTGCGCTACGTTGGCT

AAAACAGTATATACTAGGGATATTAAATAAATAGCATGCTCTATTTACTAATCTTTGTGC

TATATAAGGAGGTTCATCCATACAACAAAATACCCAGTCACTTTCAGAAATTTCTTTTTT

TACAGCATCAACAGTGCTCATTTTTAATTTTCTTTTTGTGACTACACACTGATAATTACG

TTTTAGAATACAATCTTTTACACAGTCAACTTTATATTTACCAATATCGTTAAAGGTAAA

CATAGATTGCCTATTTATATTATTTTCTTTAACAATATCATCATCGACTAAATGAAGTCG

CCCTACCCCTAATTGGGCTAATTGTAATGCGATATGAGAACCACCGGCTCCACATCCTAG

TATGGTAACTTTTAAATTACTCAATTTTTCAAGTAATTTAATCCCACCAATACCTTCACT

AGAATAATAAGTTTCTAGTGATTCATTAATTAAATAGTGATCAATTTGATTTACAGGTCT

ATCTAATATTTCTAATAAATTACGATCAGAAAATTTAGATATATATTTTTCTATAATATT

TTCGGATATATCATATTTTTTTCTTAATAAATCTTTAATTTCTTCAAAGCTATTAATTCC

ATTGCAACAAGAAATTAAATCTGACCAAAAATCTGAGTATCCTGTAATTTCTAATACATC

AACATCATCAGCTCCAATTCTTAATCTGTCGTTCTTCCATTGGTAAACGATTCTATCCTG

GTTCCATTTTGGGAAATAATTATCCATTTTATATCCTCAACTATTTGATAAAAGCATGCA

AGATGAACATGCATGCTTCTGTTATACACGAGAAGTCGATTAATATTATTGACTTCTTTT

TTATTAATACAAACTAATTTAAACTCTCTTTTTAAGAGTTTAAATTAACTAAATCTAAAA

TTATCTTAGATAAAGTACCAATTAGTTGTATCTTCTTCACGAATTTCTTCAAATTCTAAC

TCCAAAATGTTGAAATCTTCCATAATCATGATCATCACCTCCTTTCTATTATAAAACAAT

AATCTGATCAAACTCTTCCAATATTTCTGGATTTTGATCATGAGTAATGACAATAACAAT

TGCATCTTTAATGGATAGAATGTAATCCATTATTTCTTTTGCTTTTATTGGATCAAGGTT

ACTTGTAGGTTCGTCAAATATATAAACTGAATATGTTTTGACTAAAAACCTAGCTAAATC

AATTCGTTGCTTTTCTCCTTCTGAAATGATTTGGTTTGATTGAGCTAGGTTTTTACGAAG

GAAGTCTTCATTAAAACCTAAGGATTTTCTTAATTTTTCAGTTAAATGAAGGTTTCTAGC

CAATTTGATATTCTCTAGAATATTGCCTTCAATCAGAAAGTCATTTTTTTGCACAAACGC

AATATCTTTATATAACTCCTCTGAGGAAATATCAATATTCTGTTTTCCGTTGATTTTTAT

TTCTCCATCATAAAGATTTTTTGGATAATAATTTAGTATTAATTTTACTAAAGTTGTTTT

CCCAGCTCCTGAAGCACCTATAATTGCATAGCGCTTGCCTTTTTTAAATTCATAGGAAAA

ATGATCAAATAATATTTTATTTTCAATTTCATAATGTAATTGGCTAATAGAAATGGTCGA

AATGTTATCACAATCAATTGTACTTGTTTTATGGGAAGTTCCTTGATTTGTCTCATATAG

ATTTTCCTGGATATTATCAATAATGGACTTGCTACTGCTGATTAAATTTTTATTGTAAAG

AATAGATTGTAATGGAGCAAAAACACCGTTTAATAATTGAATACTTGCTACTAGTAATCC

AATAGTCAGTAAGTTATTTCTTACAAAAAAGATACCAGCGACCATACAGGATAGTTGAGA

GCCAAAACTTAGTAAAATCCCAGTAGTTGAAGCTAAGTCTTTTAAGAATTGATATGTTTT

TCTGGACTCTTCAAATTCTAAACTGATTCCCTGAATTTTTTCTTGGGTCCAGCTTTGAAT

ATTTAATAACTTAATTTGTTCAAAACCATTAATAAAGTTTGTCATTTTTGACAAATAGTG

ATTATTTTGTAGAGAATAATTATTTGTTGATTTAGTCATTAACTTTCCAGGAATTTGTGA

TAGGAAAACCGTTATACTCGACAATAAAAGGAAGACTAATGCTAATCGCCATTCTATATA

AATAATAGCAACCACACTCATGATAAGGCTCCCTAAATTTGCAATTAGAGAGATTTTAGG

GATTAATAAATTTTCTTGAATAAGGTCACTGTTTTTGGTGATATTATTTAAGAAATCTGA

ATGGTTACGGTTGTCAGTTTGGTTAAATTCACGCGATAAATAATGTAATAAGAGACGCCT

TTTTAGATCTAAAATAACTTTTTTTACATATTCGTTTTTAAGATAGGAATAGGAAGAAGA

AATCAAGAACCACAGTAAAATCGAACCTACAGAAATAGTGATATGAAAGAGTAATCCACT

ATTTGAATTAGATATACTATCAAGTATTCTTCCTAATTGCAGAGAGAAGAATGTCATTAA

AAGAGCACTTGTCAAACTTAATAAAATAATCAAGAAATATAGTAATTTATATTGTTTCAT

AATCTGGTTCATATCTTACTCCTTAAGTAACTCTGATGAGAATATTATAATCCAAATATG

AAAATCAGAAACAAACTTCCCGTATTGGGGACGCAATGAATAGTTTACACTAAATATAAA

AAAATTATAAAATAATTATTTTTTAAAATAAGAGGAGAACTCATATTTTTATTTAACAAT

GAATATATCACACTAAACCAGATAGAATATACAAGTTAATTTGTACCGAAATTACTATAT

ATTATAAGAAAAATAGAAACTCTAAAGCATTCTGTACACTTTAATTTTAATTCATCTCTT

TTTGTTTCTAATACAGAGTATTGAGACTGGATTATAAGCACATCAACAAAAAACTCTATT

CAAACCTACCTTTGAATAAAGTCTTTTAACTATCTAAAAAGTTCACATTTTATGCTTATT

TATGGAGTTAATGAAGCGCCAACTAAATCCTAGAACCAACATCAGATTGATGAAAAAGTA

CACAGCCGTAAAACTAGTTAAACCATGTTGTCTGATATCATCAAAATATAAACTAGGTTC

AGTAAAGAACAGATAAATTCCATAGACCTCCACGAAAATAAGAAAACTGATAAAACCAAT

AAGGAAAAGTCCACTGGCAATAAGAAAGAGTCTCCATAAAAGAATGAGAATTCCTCCTAC

TGCTAAAAAAATTACTGGGATAAGGAGTAAATCATTCATGACTTTCTCCTTTCTAATCAA

TCAACTGACGATAATGGGTGGCAAGAGAGGAATCAAACTCTTCTAATTTTTGAACTAAGT

CCAAATACTCTTCTTTTTCATGTTCTTCAAAGTCCACACCTTGATGATTCTGAAGAGCGA

TTTCCAATTGACTAGTGAGCTCTCGTTTAGAAAAACTGTAATCCCCTGTCACTTCCTCAT

AATGTTCCTTTAACTCCTTGTAGGCTTGGTATTCTTCAGGTGTTTGAAATCCCTGTACCA

AAGCCTCTTCTAATTGATAATAGGTATCTTCCATCATAATCGTTACCTCGTTTCTTTCTA

TCTTTATTCTACCGCGCTTTTCTTTTCTTGTCCGCTATTTATATCTATTTTTTAATCTAA

TTCAAAAGAAAGTTGTTCTTGCTTTTCTTTTCCTTTTTCGTGGAATTGTCGTAAGGCCTG

ATCAATAATATCTAAATCCGTTTCTGTTTCAATCAATGGCGCGAGTACATCGTATTCGGC

CTTTTTAGTTTGATAATCCTCTTCCTTTGGAAAATTTTTCTCAATTTCTACCTTAGCAGT

AGTCCATTTATCCTTTAATTCATCCAATAAGTTCTGAGTTTTCACTTGGTCATCTTTAAT

GTGGTCTATCGTATGCTGAAGCCTTTGAATTGTCCCCAAAGGAGAATACAAATCCAAACT

GACAGAATACTGATTTTCTCCTACAATCTTAACAGAGAAGGTTTCAGGAAGAGGCTGATT

CGTTGGTAGACTAAGCATTTTAATGTCAAATCCTCGATAACTTGCTAGGGTTCGAAATTC

TTTGCTGTCAGATTGATTATGACGGATAAGACGGTGTAGGGATTCCCCTGCTTCAGCTCG

TTGCTCAAACGCTTGCTTACCTACCGTCATAGAAAATGCCTGGTCTTTCGACATTTCAGA

CTGTTGAATGTCGCCTTCATACTTGCTTAATCGTTTCTCAAGAATGGGCATATTTTCTTC

ACAGTAGGAGATTGTATGACGATAGTGATCCTTGCTGCGTTGAAAGGCGCGTCTTTGATT

TTCTAATAGAGTTAGATCATTCTCTAGTTCCATCTTATATTTGAGATAAGGATTACCTGT

TGCTAGTGCCTTAAAATCAGAAGCTGTCATGGTCTGCTCATCAATATCTTCTGCAGCACG

AATCGGCTCCTTAGAAGTCATAATCTGCTTAATATAACGGAGTTTGTTCTCCTGAGTTGC

CCATAGATAATTATCAAATGAACCTTTGGTAATGTAGTGGTAAATATCCACTTCCTTGTT

TTCATTTCCCTGTCGGATAATACGTCCATTGCGTTGCTGAATGTCACTTGGTCTCCATGG

CACATCCAGGTGATGAACTGCTTTCATCTTGCTCTGAACATTTAAACCTGTTCCTCCTTT

TTCAGTTGAGGCAAGGAGAATCCGCACCTCTCCTGCATTGACCTTTCGAGACAAGCTATT

CTTCTTTTCATCACTATTGGCATCATGTACAAAGGCAATTTCCTTACTAGGGATTCCTCT

ATCCACTAATAAAGCCTTTATTTCAGAATAAACATCAAAGCCATTATCCTTTTTCTTAGG

TGTGCCAATATCTGAGAAAATCATCTGAGTAGCCTTATTTCCCATTCCCTCACGATAAAT

TCTTTCAACATTATCCACTACCTGAAGCAGTTTATGATTATCTGCTAGACTATAGCTAGA

GTCCAATAAACGCATATCAATAGCTAGTTTCCGTGCCTCACCTGTTATCTTTAACATGTT

ATCCTGACTCGGATCAACTGTTCCACATTTGACCATATCTGACCTCATAACTAATTCTTC

TAAATAGAGTTTCTGGTTTTCAGTTAACTCACTCTCAATAGGGATAATATGAGCTTCTGG

AACAGGTAAATCCAACATATCTTGTGTTTGAATGTCTGCTGTTTCTTTATAGATTTTCAT

CAACTCAGGTAGATTGACAAACTTTTTAAATCGTTTCTTAGGCTGGTACTTATCCCCTGT

AGGAGCTAATTCCATAGAGTTTTGAATTTCTCCAAAAGCACCTACCCAAGAGTCAAAATA

ATCAACTTGATAGCGTTTTAAGATATCTGGTTGAATATAGTTCATCATAGTATATAACTC

ACTAATAGAATTGGAAACAGGTGTTCCTGTCGCAAAAACAATATTCTTAAAATCATGTTC

TTCCTGAATCTGTCGAACCTTCATTTCCATATCCACGTTCTTCTTAGAGGTTGTATTGGT

AATACCTGCTACATTCCCAAGTCCAGTAATTGGACGAATATTTTTAAAGTGATGTGCTTC

ATCCACAAAGAGAAAATCAATTCCTAAGTTTTCAAAATCAATAAAACTATCACGATTAAA

GCGTTGCAGTTCTTCCAATTGTTTCTCTAGACCACTTATTGATTGCTCTGCTTCTTTAAC

AGTATACTTATTTTCAGAATGTGTTTTAATCTCTCGTAGTTCATTGAGTTTATCCTCGAT

ATAATTCATCTGTCTTTCCTTACTGACAGGGATTTTTTCAAATTGAGAATCCCCAATGAC

AATGGCATCGTAATCTCCTGTAATAATACGCGACACAAATTGTTTTCTTCTCGACTTCAC

AAAATCTTTCTTAGTGGTCACAAAGACCTTTTTAGTAGGGAAAAATTTCATGATTTCTTG

GCCAAATTGAGCAGACAAACTAGAGGGCACCACATACAAGGGCTTATGAACCATGCCCAA

CTCCTTTAATTTAAACCCAGCACCAAGCATGGTCAAGGTCTTTCCTGAACCTACCTCATG

AGCTAACAAGGCTCTTTTTTCTTCTACGATTCTTTGAATGGCATTCTCTTGGTGAGGACG

AAGACTGATGTTTTGTGCCAAGCCATCAATGACTAGATGGCTACCGTCATACTCTCGACT

AACCGTTCGATTATAAAGACGATTATAGCTTTCCTCAATGACTTGCTGGACTTCTGGATA

CCGTGAGACAAATTCTTGAAAGAGCTCTTGTAAATGCTGCTCTTTTGCTCTTAGAACAGA

GGTTTTTTCCAAATCTGTGATGGTCTTTTTCTTTTCTCCTTCCGTAACAGTCATGGTAAT

CGTCGGTTGGTTCGAATTAAGTAAATTCTCAAAAATCTTTCTTCCTGTATCATAACGTGA

CCCACTAACTCCAAGACTACTGTCTTTGGCACTTGGATAGCGATAAGCAAATGATGTCTT

TAAATGAACCTGGCCATCGACAGGATTCACTTCAATGACTTGTTCAACATCAGGCGAAGA

CAATTCAAATTCACGATTGGTAAAACATTCAAAGGCAAATTTACCATAAACGGATTGAGG

AATCCAACGTGACCCTATTTTAAACTCAATATCTGCCAGATGAATCCGTGGAGGGCGAAC

AGACTCTAACAAATCTAAAGCATGGGACCAGTCACATTCTTGGTTGTTTTCCTCCACTAA

TAGTTGAACTACTTCTATCTTGTTGAGAATGTCTCCTGATAAAAACTGGTTCTTAGAAAG

ATATTTTCTTTCTCCTCTTAAATAGCTTTCTGGATCCATTAAAACCTGATCACCTAACTC

ATCTAAAATAGCAGCTTGGCTATGTTCGGGATAAATTGATACCATATAGTCTAAATCAAC

CCCTCTACCATCCGATAAACTAGAGTTTACGGCATCTAGAGCCGTTGATACTCTTGCAAT

CACTCTCTCCGGCCTAACCAATGCTTTCTCAAAGGCTAAAGATTTTTTATATTTTACTTT

CTGATCTTTAGAATCAATGTATTCATCTTCTAAACTTGCAAGCAAAGAATACTTATCGTC

ACTATCAAATAAGTTCCGATTGACTGAGGCATTCAAGTATCCAAATTGACTTACAAAGCG

GTCATAGTCACGATTGAGTTTACTAAGTAATACCTGAAAATCTGTCCGACTATAATCTTG

ATGACGTTGAATTTCAATTAAGGATTGATAGGTCTCTCTCAAATCAACCATTCCCTTAAT

GCGACTAATATCCTTATCCGATAAGGGACTTTCATAAAAGACAGTTTTTTTGAACAGTCC

CTTATATTTCCCCCTTTTACTCGCTTCTTCTGACTTGTAAACATCTAGTGCTTCCTCATC

AGTCAAATGAAGTTGCACGAATCGGTCTATTTTATGTTCAGACAAAGAACTGTCCCAAGC

TTTAAACTCTCCCTTCTCGTCTACATAATAACTAATTTCGTCTACTTTTGAACTTTTCCG

AATGCCATGTGTATCTCGGTAATAAATTTGATTTCCCTCATATCCAAAAGAATAGAGCGC

TAAGTTCTCACGAATACGACTTGGGATAGAATGATCCACTTTTTCTTGGATAAAAACAGG

TTCTTTCAAAGAATTGTCAATTGGTTTAGGTGCCTCCACATTCTCTAATGCTTCCATGAT

GTCAGTAGTGAATGTTTCTGATTCCCCCTTAACATTGAGAGTTCCTCCATTAAAATTGCG

TACCTCATATTGACCCAAAACTTGTGTATTGTATTTCCCATCAAAATAAGGATTGATCCA

GACACGCTTATCCTCCTCAAAGGGAATAGAGCCACTAAAGACAAGCTCCTCCTCATGAAG

ATTCTTTGCTTGATCCTTTTGAAAGAAGAGGAGGTCTGTGGTCACTCGAGTACCTGCAAT

CTTTTTAAAAGCCGTATCCGGCAAACGAACTCCCCCTAAAAAATGAGTGCTGGATTTAAT

CTCTTGTAAGACATTATCCGTCCGCTTATCCATTGTGCCAATAGATGAGATAATCGAAAC

TTGTCCTCCGTCTCTTACTAAATCAAGTGAGTGTTTGACAAAGTAGTCATGAATCATATA

AGGTCTATCATAATTTTTATCTGCAATGCGAAAATTTCCAAATGGTACATTCGTTAAGAC

TAAATCAAAACTATTATTTTGATAGGGAACTTCTTCAAATCCTCGCACTTCAATATGAGT

ATTGGGATGAAGTTGTTTTGCGATTGCGCCTGTCACACTGTCTAATTCAACTCCATAGAG

TTCTGATTTCTCTCGTATACTTCTAGGCATCGCCGCAAAGAAGTTCCCAGTCCCCATAGA

AGGATCTAAAATTCTTCCTCCCTCAAAACCATCATCCAGTAACTTTTGCCAAATCTGGCG

AATAATCATCGGGTCTGTATAATAGGCTGTGAGAGAACTTTGTTTCATGGTAGAGTATTC

TGATTTACTTACTAAGCTCTTAAGAGTTAAACGTTCTGCCTCATACTTTGGATTGAGTTC

ATCAAAAAATTCATTGGCAAGACCACCCCATCCAACATACTTGGCTAGTAGTTCTTGTTC

TTCTGGACTTGCTTGTCGTCCCTCTTTTTCTAATCTTTTAACAAGTTCAATTGCGGCGAT

ATTCGTTTCAATCTTTTCTCGATTTGTCTTAGGATAAAAGTCCTCTAAATCATCTGGAAA

AACAAAATCTTGAACATGGACATCCGTCTCTTTTATACCCGAAATTAAAGGTTTTGTTTC

CTTGTCCTTATCCTTTTCATTTTCATTTTCTTCTTCCAGGTAGGAAAACAAATCTATTTC

CTGACTTTTACTTGATGAATCAATCTCAATCTCTGAATCTTCTTTTTCAAGTTCTACGAG

AGACAATACTTGTTCAATCTCTTCCAAAGTGTTCAAGTATAAGATAGGATTCTCTTCAAA

TAACTGGTTGGAATCATTGAATAACTCTAGGCGAACTAAGTCATTTAACTGTGCATTTTC

AATCGAAACCAACTGAAATACTTGTCCTTTATAACTTACTTGTGAACCGATTGGATATTC

CTTCAAAGCTTCTTCTACAATTTTATCGACATTAGAAAAAGAGTTCGTTTCTTCCGTTTC

CTGTTCAATACCACTTGAAGGAGATAAAACTTCCTCTACTTGATTAGACTTTTCGCTTCT

AGCTTTTACATCATCAAAATGAGTAATAATCTTCAGCTTTTGTGTCAGTGGGAGTTGTTG

GTAGTTTTCTTCATGATGGACAAGATCCGTCACGGTATCTAAAGTAGCCACTAATTCAGA

ACCCAAATAAGCTAGAACATCTCCCTCTTTTTCTTCAAAATGAATCTCAAGAACTTCTTT

TTTAAGCTCTTTTGAGTCAACCATTAGAAGAAATTCCTCTATATCTCTACTAACACGGTC

AGATAGATTATTCGCAACCCGATAGACATTGACTAAATTGACATCCTGATTCTGATGAAA

TACAAGCTCACTTAATGGTTCTAACTTCTCTTTTTCAGATTGGATGTAAAATCGAGTGGA

AAGGTTATAGGTTGCGACTTCTAGCACCAAGTTTTTCTCCCAACTACTTAATTGAGATAA

TTTACTTAGACCTGCTTGTCCAAAATTTCTTGTATAACTTTCTAAATCTTGGTCATTATT

TTCAGAACTAGCCTCTAAGAAAGAAACAATATCTTGATCAAAAGAATAGACGTGAGGAAT

CTCTTCTATCTTGCCCTCTTCAGCCAAAAAACTCTCTACCAAATCCTGCCATTTCTCATC

AAAATGTTGCGCTTGATAACGAAGAAATAGGTCCATTTTCTCCCTATCTTTTGGAATTGT

TCCACGAAACATAGCCAATAATTGCATTACTTCCATACTCGCTCCTTTCATCTATATGAA

AATAGGAGAATCACATGATTCCCCTACAACTCTATCTCCATTTCAACCTCTTGAGGACTG

GTTTCTAAATCACTAGACAAAAGAGAAACAGAATCTGTTCTCATGTAGTAAGTATATAAG

TCTTCTAGGTCTTGTTGGTTTTCAATCCCTGTTTCAATACTAATCAGAGCCTGTATAAAT

TCCTTGTAGTGACTCTCCATCATATCTGAAATCTTTTGTTTCATTTCCATAAATACTGAA

GATGGTTCAGTTATACTTGTTTGGTCTATGTCTTGTAAATCCTTTTCTAAGGGATTGGTT

CCTTCTTCTACTTCCACTAAATCTGAACGACCATCACCATCAGAATCTGCACTAAGAGGA

TTGGTTCCTAGCGCTAATTCTTGAGCATCAGTTAGTCCATCTTGATCCGAATCACGTTGA

TAAATGGCTTCCATATACTTCCTTCTTTCCTAGTTAATCTTTTTTCACTCCAATCCTACC

AAATATTCCTTGAAAGAAGTATCACAACAAAAAAAGAGAACGAGCCAAAACCTCGTTCTC

CTTAAAATGATAAAACGTTACTATCAAAGTAAAAAGATGACTTACAGAACCTGTAAGCCA

CCTAGTCAGTCGGTTTATTCTGGTGTCTGCCACCGCTTGGCCCTTACGTCCAAGATTGCT

ATCGGATTTTGTTCTGGTGTCCGCCACCGCTTGGCCCTTATGTCCAAGATTACTATCAGA

TTGACCATGAGCCAACTACTCATCTACTCCATTATTCTAACGAATATGAGCTACAAACGC

AAGAGAAAAACTTATTTTGGATTTATACACTATAAAAAACATACACTATCTTCAGATTGA

ACTTTTGTTCAAAATAAGCTATAATGAATTTAAGTAGAAACAGGAGGTAGAACAAATGGC

TACAATCAGTTTAACACGTGATATAAAACTAACAAACGAAGATGCTCTAAAAATTCTTAA

TCATAAACCATCTAAAAAGCTACAAGCCGTCTTGAAATCTATTGAATCACAGGACGCTAC

CGCTCCAACGAAAAATAAACTCATTTCAAAGTATCTGTAAGATGACAATTCAAGTAACAC

CACTTAAGCAATATCTTGAAAATATTCAAGTGCTAGCTCCTGATAAAACTGAGAAACAAG

TTCAGGATCTATTTAAAACTATCATTTTAGAAAATGTTGATTTCAACGGTAATGAAGAGA

TGTTGACTTATCTATCTGATAAAGCTCCCAATTTTGAAAAACAACATCGTTCTCGTAATT

TTATCGTTGAAGAAACTGAAACAAATAACATCATAGGATTCTTTAGTTTATCTCTCAAAG

TTGTTGATATTTCTGATTTAGAGAAATCCTTAAAAAAGAAACTCGTCCTAAAAGGGAAAA

GTCCCAAAAATATTGATTATCTCCCTGTGTTACTTATCGGTCAATTCGGCAAAAACACAA

AGCTAAATAAACTATCAGGACAAGAGTTATTTGAAATTGTAATTCAAAAAATTGAAGAAT

TTCGAGCAATTGTAGGAACTCAGATGGTATTTTTAGATAGTATCAATCATCCTAAAGTCA

TTCAATTTTATGAACAGTTCGGATTTGTTGCTTACAGTCAATTGATTAAAGATGATCATC

AAGTAAGTTATCAGCCTATGGCATTAAATATGTCGCTCTATAAAAAATAAGAATCATGTC

ACCTAAACTGATGACATGATTCTTTTATTTCGTAGTATAGGCAAAACTGATGGCACTATC

TGCTTGAGAGATTTTTCTAAAGGTATAGTTAGGATTACCCCCATAGTTTGTCTCAGACAC

AAGGAAAGAACCATCATCATATACTTTCTCGACAAAAGCCACATGACCGTAGATGGCTGG

TGTGCCATGTGTACCTCCCACAAAAGAAACAATGGCACCTGCTCTTGGTGTAGAGCCAGT

TTCCCCACCAAGACTTGAAGCTGTCGCAACCCAGTCCTGACCATTTCCCATGGTATTAAT

GATTGAAATCTTCTCTCCGTTTCTACCTTTTAGCTTTAATCCTAACTGGTTCATACGAGC

CGCAACACCCCATGTACATTGTCCATAGGCATAGGCCATACCATCTCCACCACCAGGAAC

AGAATGATCATACAAGTCCCCACGAACCCCTTCAAGGGATTGTGGGTCACTTTTTGCCTG

TCCTCCATTTGTTTGACTAAAGCCTTTTTCAATTTGATAATACCATTCCGTTGCTCTGGT

TTGTCTTTCCAGTAGTTTATCACCAGAATTTCCTTCCCAATAGGTCAGAAAGAGTTGGGC

GAGATTGGCTGCACTGCCCGTATTTTTAAAAAATCTTTTAACCAACTTTGATAGTAAGGA

CTATCCCCATGAAGCATAAAATCAAGTTGGAGGTCTAAATCATACCACTTCTTATTTTGG

GTGCGTGCATAATTTAACAAAGCTGTATGACGTGTTGAACCATCTGCGGTATCCGTCCAT

TGACCTAACCCCAAACCTCTATGAAGGATATTAGGATAAGCACCACTATAAATGGCTGGA

CCTCCAATCGCTAACCAGCTTTCATCATCCCATGAGGAATCGGTAGCGCCAACAGGAGGA

GATAAATAATCTCCTTCAGCTCGTTTAGGATTAATAGAAGACTCTACCGACCAATTTCCT

AAAATTGCCGCAATGGCTTGGGGACTTGCCCCTTGAGATTTCAAAAACTCATAAATATGT

TTTGCTCGTTCAAACTCATCCCCACCAAACTGACCAATGGCAGGTAAGATAGTTGTCTGA

AGTTGAATGACTTTTGGAAAATAAAATTGCGGATTGACATACACCAATTTTTCTTTCTTG

TTCTTATACTTTTGATAGGAAACTTTCAAACCTGTATCGTCTGGTGTTTCACCAATAATA

TCACCCGTTAGGACTCTTGTCCCCTCAATCGCACGACCATTATGAATAGAATACAAGGTC

AATCGACTCTCATTCTCTCCTTTTCCGTTAGTGAGAATAACATCATCTCCGTCTAGAGAT

ACAACACCATCCATTGGTGCGACAATTGTTTGGTGAACCTTCGCTTCTAGTAGAATGTAC

TCCTGAAGAGTAGGTTTTCCGTCTAAATCATAGTATCCATAACGATAAGTCATGGTTAGA

CTATCTTCGTTGCTTTTCCCCTCAAATGGATTGTCCAATTCCTGCATGGAAGCATAGACA

CCTTCTTCTTTTAGTTCCTTAATTTCCTCTTGATCGTCTTTCGATAGTTTATACTTAGGA

CTTTCATAGAGGTCTTGCATGGATTTCAAATCTTCCCCATCGTTTAAATCATGCCACAAA

GTAGACAGATAATCCTTGTAAGTTTCTGAACTAAATAAGTGAACTGGTTTGTGTAACTCA

TAGTCATGGAATTTAAAGTTCATATACCCCATCACATCATCAACTTTTGTGTAATAAGTA

ATTCCTTTGTCATTTGTGCGAGTATGTTCTGCATCTTCCCAAGTTAGGTGGGTATAAGCT

TTTGTTAATTCAAATTCATCTTGTTGAATCAAACTAGCAGATGAAAATCCTAAAAAGAAG

CTCATTATAAGTAAAAGAAGAAAGACTATTCCTCCAACTATCCAGGTTACAGGATTTCCA

GCCGCAAATGTAAAGAAGGAAAAGGCTGCTTTTAGTTTTTGATAGATATTTCGGACACTT

GTAAGACCTTGTTTCTTTAATTTTCGAAACCGATTTTTAAAGGAACTTGGCTTATCTTTC

GCTAGTTTCCATCCTTTTCCATCCTTAAAATGATGGTATCGCTCTTTTGTGTTGGTCAGT

CTTTTCTTGGTAAAACGACCTGTTGCTTGTCCTGTTTTGACACTAGCTTTTCCAAGGTTA

TAAGAAAGGCGACTGTAGCGTTTCCCTTTTCTAATTGTCTCTTGAAGTGTGCGATAGCCT

TCTAAATCTTCATTTTCTGAAGCTAACTCTCCACCTTCACGTCCAAGGACATAAAGAAAA

GTTTTGGCTTTCCTACTAACCTTTTTGGATTTATAGGCTTGTTTAGTAGATTTTAAATTT

TCTTTGGCGGCCTTGACTTCTTTCTTAGCTTTTAATTCTTCTAAACTCTTCCCTTGAAAG

AAAAAATTAGATTTTTGTTTCGATTCCTGACCGTAGAGAAATTTTTGATTGGTTTTTCTT

TCTTTACGACTCTCTTTTCTTTCTTCTTTTGCTTCTACCTTGGCTTCTCTAAATTGCTTC

TTGGCTATTTTCAATCGTTTCCTAGCATGAGGCAACCGTCTGTCTCTTAATTCTTTGCGA

TTCAAAAGAGATGGAGGACTATTTTGAAGAATATGATTGTAGTCTTCATTTGCTTGTTTT

ACTCTCTCCTTTGAAGCTTCTCTCATCTCCTCTAGCTTTTGCTTTATCTCTTTTTTCCAT

GCTTTTTCATCCAGTACAGCGGAATCTTTTTTCTGTTTCCTTACCTCCTTCTTTCCTTTT

TTCAAGAATTTTTTCTCATCTTTTAGGCTTCTTCTAAATGCCTTTCGGGCACGTATGATT

TTTCTTTTATCCTTCATTTACCTTCCCCTTAGTTAGAAGCCATTTTATCAGGATCCGTAC

TCATGATATCAAACAATTGAGTACCTTGAGGAATCTTATTTTTAAAGGGTACGACAACTG

AACCAGCTTTTATCAGTCCTGCCCCTTTTTCTGGATTGACAAGGTATTTTTCGAGTTCTT

TTGACAAGCCTAAGAGTTGAACCAGTTCTTCTCGGTCATTTTTTGCTTGCTTGAGGAGAA

TCATAAATTCACTATTGGCAATAATCCGTCTACCATTTGGATCTAACAATAAGGTTTCGA

CGTTTTGAGTTATTCCAGTCGGACTGGCTCCATATTTTCTGACACGACTCCACAATTTAA

AGAAGAAATCACTGGCATATTTATCTAATAAGAGAAGCTGCATTTCATCAAAATAAATCC

AGGTCTTCTTCCCTAATTTTTGGTTCCGAACAACACGATTCCATATCTGATCAAAAACAA

CCATGAGAGCGATTTGTTTCAGCTCATCTCCTAACTTTTTAACATTATAGATTAAGAAAT

TAGATCCTGTCTGAATATTGGTCTTATGAGAAAAAATATCAAGAGAACCTTCGACATACA

GTTCCATATCAAGTGCCAAATTCTGCGCTTCTTCTTCTGGTTGTTGGCTCAAGACAAAGA

CCCATTCTTCCAAAGAAGGTTCTTTAAATGACTGATAGGTGAGTCTGGTGACTCGGTCGA

TAATCGATTTTTCTCTTCCATCCATTTTCCTATCCAATAATTTGCCGATAAAAGATAAAA

GAAATTCTGATTTTACCTTTACAGGATCCTCATCCATATTTTCCTCAGACAAGTCAAGGA

CATTGAGATAAGTTTGGGAATCAGGCGCAATATCAATCATTTCTCCACCAAAAGCTCGTC

CAATGACACTGTATTCTGCTTCTGGATCCACGATGATAATTTCAGTATTTTCACCAGATT

CCTTGATTTTAGTCGTGATAATTTCATGCTTGGTTGCCATCCCTTTCCCAGCTCCAGATG

TCCCTAATATCAGACCAGACGGTGTATTTAATAGGCTGCGATCAATGGTAATAATATTAC

TTGAGATTTGATTGATACCGTAATATTTCCCACTACGGTCTTGTAAATCTACTGAAGTCC

AAGGTGAGTTCACTGCTACATTAGATGTTAATAAACTCCGTGATACTCCCTCTAGAAAAT

CACAACCAAATGGCAGCAAACTATTAAAGGCTGCTTCTTGCATATATGGAAGTTTATCAA

TCATTAGGTCATTTGAGCCGGCCACTTGTTGAATCGTGTCTAGAGCTTGTTTAAGTTCTT

CTTCATCCTGACCAAAAACACCAATCAAGAAGACTGTTTGAAATAGTTTATCTCCTGTCT

CGGTCATGGTTTTTAAGAGTTCCTCAGCTTCATCAATATTGCTTTCTAAAACATGGCCTA

CTTTTTCCAAATAGATACCTGTACGAGCAAGTTTTTGTTGTTCCCCAATCTTTTGGGATT

CCATTAAGGTCTTCTTTGTTCGTAGTTTCTTCATGGCATCCGCCTTGGTCGAACTTTGAG

CATGAAGGCTCACTATCAATTCCAAATCTCCTTGCATGAGGTCTCGGATAAACTGATCAC

CTAATTCCATGCCGTAGTCTCTCACATAGACAATCTGCAATAAGCGGTCATTGATTTGTA

GGTAATTCTTGTTTTTAAAATCTAAAAGGTTAGGGGCTATGAAGTGACGAGTTGTCTGAC

CAGATCTCGTTAAATCACGGTAAGAAAAAGGAAGATGGTGTTCTCCTCTAAGCATATCGG

CCAACAAGTTCACCCGTTCTTCTCCAGCCAAGGATTCAAATCGTGCATCAATTTCTGAGA

AACCACTCTTGAAATATTCGCCTATTTGAGACAAGGAACGATAGGCTTGTTTGGGATTAG

AATCCTTTCTACCAAAGCTAATCAGTTTCACAGCTGAAAAATTATTTTCACCACTGTCTA

AATTCTGATTCATCATTCGATTCAATTCTTGACGATAGCTATCATATCCATCTTCTTTTT

CCTCATACAAAACACTTTGTCTAAACTTTTCTAAATTTAATCTTTTATTAAAGATAGTCA

ATTGGAAGTTGGTTTGGTCGTCTAAAGAGTTAATCAAATCAGAATACTTCTCAATGATTG

CGCCCTTATCTTCTAAACCAACAGTTTGGTAATTGACATCACCAAGTAAATAGCTTTGTG

AGAAATAATCTTCTTTTACCTGCATCAGACCATTTTGATACAGGGCTTGATAGGAAAGAG

TATTAGCCGTTGAGGGTAACACTTCCTCTTTTTTCCCTTTAACTTTTTCCTTTTTATTAG

TCATTGAAGTTTTTTGTTTCTTTAATGTATTTGATTTTCTTTTCATGTTCAGGTCCTTTC

TTTCCTGTAATTGTGCGTAGGGGAACCGTTAATTCAAAATGAAGACGGTATTTCAAATAA

TGTTCAAAATATAAATCATTGGGTTTATAGACTCCAAAAAGCATGAGGGGGATGGTAAAA

GCAAACACAAAACCGTAAACAAACCAATCTCCAAATTGCCAGAAAAAGAGATTCAAGCCC

AAAACAATAATTGTGACAATAAAGGCTGGTAAAACAAAGATGATTTGTCTTGTGGTGAAA

CCTAACCAAGCCCTGTGTTGGTATTTTGAGATGTCTTTAAAGACACGTGTATTCATGACT

TTCCTTTCTAAAAAGGCTAAGAAGCATTCACTTCCTAGCCTTTATCTAATTACATACCTA

AGATTGAGCGAGCCGTACGTTGAGAACCAACGAGGGCAATAATCAGTAAGATAGCTTGTA

CCAAACTACCAAACATAATCGCAAGTGATTGCAAGACTCCTGCACCATTTGAGACAGCTA

TTTTCCCAGCAGATTCAAACAAAGGAACAAGAGAAACAATCAGAAAAATAAGAACCCCTT

GTACCGCATAGACCATAATATTTTTTAAATAGCCAATACCAATAGACTTCCATTCATCAC

TTAAAAATGTTGGAATCGTAAGAGGGGCAAATGGAATCATAAGGTAGAGTTGAATAAATC

GAATAGATACCAAAAGGTTAACCATGGCTGCACTTACTATCCGAACAAGCCAAATGAGGA

GGGCGAAAAAGCCCACAATCATCCGGCCAATAAATCCTGAACCTTTTAATCCAGAGATTG

TATCATACTTCGCCCCACCGTGAGCCACAATCGAGGCCACTTGTTCAATGGCGTGACTCG

CAATCCCGATGATGGCTTCTACAATCACGGTAGTGTTGGTAATTACAACTGCGACCATGA

TATAACTAATCAACATCGGCGCCAATGCTTCAAAGGTCATCGCTCCTCCTGAGTTAGCAA

TTTTCTTTGCCATCTTTGAAAATTCTAAGATGAGAACAACTGATAGAATCGCAACTCCAA

GAGGCTGCATGACACTTTTAGTAATACTAGACATATAAGTCCAAACAGTTGGATTGTAGC

TAGATAGAGATTTAATCAGATCTACCGTAGATTGTAAATCTACATTAAACCCTTCAAATA

AATTTTCAGCTGATATTTTTTCAGATGCAAGGTAAACAAAGGGTGAGACTAAACTAAGAT

TCATGTCATTGTTTATCCTCCTAAATTGAAATCTGGGTTACAAAGGCTCCAGCAGCCCCT

ACCATAACTCCACCGACAATTTCAAGAATGGCATTCCGAACACCTGGTCCACCATCTTTA

ATGTTGGTTGCAAGATTGACAATCCCCACAACAACGAGAAAGGCACCAACCGCAATCAAT

CCCTTCTGTAACAAAGACATAGCTTGTGCAAACATGGCACTTGCGTCTACTCCATAAACA

AAACCTTTAAAATGCGTAATCATCTATTTCCTCTTTTCTATTTTTATTTTAAACTAGATT

CAAAAGTTAAATCACGAATTCTAAGGCCTTCAAGATGATTTTCCTGTCTTTGATTCAAAG

GATTAATTTGATAGTTCCACCACCGTTCATCGGTTTCTTGATTGGCTAGGTACTTCCAGT

TTGGATGCTTAGTGGAATAGTATTTTTTGCTTTTAAAGACAGGCATATTGGCAATTCGAA

CCAAGCATTCATGCCGTTTCATATTTCCGATCTCATCAGGTGTCATTAAATCACGAGCAA

TCTTTTGATGAGAAAGGGATCCTGAACCTGTCTGGCCAAAGGAACGACTCGTATTTCGAA

CATCAATGGTTTGTTTACCGAGTAAGCCACTCATAAATTTAAAGGTATCTTCATCATTCC

CACCTAAGTAGACTAAGCTATCACAGTTTCCTAAAATGGTTTTCCAAGCTTCTTTTTCTT

TATAGAGCCCTTGAAGTTGGGCAATATTTTGTAGAATAGGAACGAGACTCATATTACGAG

AGCGGACTGTTGAGGTTTGTTCAGCAAAATCTGGAATTTCTCCGATATTTGCGAACTCAT

CTAAATAGACTCTCACATGAAGAGGCAATTGACCCTTAAAATCAATATCTGCTTGTCTTG

TTAGGGTTTGAAATACGGTTGAAAAAAAGAGGGCTGAAAGAAAGCGAAAGGTACTATCGT

TATCTGGGATAACTAAGTAAACCATTGATTTTTCCTGGCCCCATGTCTTCATATCAAGGG

TATCTCTTTTGGTCAAATCCATGACACTTTGAATATTGAAGAGGGCAAATTTAGCAGTGG

TTACAGCTATAACAGAATCCAGAGTCTTATCCTTATAATTTTGAAAATCTGCCCAATTTC

GCATGGTGAAATTTTCAGTCCCATACTTTTTAGCATAATTTTCAAATAGAATTTCTAAGA

CACTTTTTTCTTGATTTTCACCCTTGGATAAGTGTTTAATGAGTTTTGAGATTTCCGCAA

AACTTGGATAACGCCCTCGTTTTTTTCGCTCTTCCACTTCTTTTTTTGACGTTTCAACAA

GTTTTGGTATTCTTTTTGACTTAAACGACTTTCTTCTATGAGCTGTTCTCTTGTTTTAGG

TGGGTTATAGAAATCGACCAAGTAAGAGGCTAAAGCTCGAACCAAAGTCATAGAAGCTTC

ATCCCAAAATGGATCACTTCGGGAGCCTGAGCCTTTGGTGTTATTGAAATAAACCGTCAG

CATTCGATTCAAATCGTTTTCTGTCTCTATATAGCGAAAAGGATTAAAGCCATCTGAGTT

CTTCATATTGACTAAATCTAACACCTTTACTTGGTAGCCACGTTCTAAAAAGAGTTTTCC

AGTTTTCTCAGCTAAGTGATCTTTAGGATCCACTACAATATTTGAACTATTCATCTGAAT

CAGATTGGGTTTCACAAAGCGAAATGTCTTCCCACTTCCTGAACCTCCAATCACCGCAAT

ATTCTTATTTCTATCATATTGGGGTGGTTTTTTATCTAATAATGTCAAACGAACATCTTG

TGCTAAGATCGTATCATGAGAAAATTCCTTACCGTAAAAGAGCTTCTTTTCTTTTAGAGT

TCCAAAACGGGCACTCCCGTATTCTACCCCTTCTCGGTATTGTTTTTTACCAGTCTCTAG

ATAGAGATAAACCAGCAACATCATCACAAAGCCTAGTAGAAAAAAAGCACTTGATTTTCC

AGTAAAGGAAACATTCCATGGCGACTGAAGAACTTCATCTTGACCTTCCATCAGAAGATG

AGTCCATTTATCTAATGTATTTCCAGTATAGGAATCATACAAAAGCGTCAAACGATGAAA

AAGATAGCCTAGTAAGATACCTAACAGTGAGAATAGTAGGAATTTCTTTCCACTGTACAT

CATCTCACCATCTCTTTCTGTTTGACGGCCCCTTCTTGTCTAAAGGTTATTTGGGATTTA

GCCTCATCAATTGCATCGTCCAATGACTTATCCATGGTAAAATCAGCTAATTTATCCGGA

TCATTAACCATTTTTTCTAACAAATGCTCTAAATGATTGTCCAGAATCGAACGGTCTTTC

GTGTAGAAATGTAGAGAATCCCCTTGCCAAGCGATGGCTAAAGGAATCTCTTCTTTTTCT

AAAAAAGCTTTAAATTTCTCTATATCAATTGGTTTGTCTAAAAAATCTTTTTTCAGATTA

ATCGTATCAATCGAATAAGGAGATTGTAGCAATTCTTCTAATTTCTGCACTCCTATCTTA

TAGGCGGAATCCTGTGCTAAAGCCTGACGTCTAGACCATTCTAGAATCTTTAAAAGACTT

TTTACAGTAAATAAAAGACTACGCTCAGCATATTGAACTGCCATTCGTTCCTGTTGTTCA

GAGGACATCCGATGCCTCCTTCTTCACAAATAGCAGCTTTCCTTCTTTATAACGATAAGC

TATCAATTTCTGACGTTGCTTAATCGACTTGACAACCTGCAGTAGATCTCTCGAATAAGG

TTCTCGAAGAGTAACTTCTACTGCTTTCCCATCAACGATACCAACACGTTTAAATTCAAT

GTAGTCTTTTTTGAGATGCCCTAAGCCCATACCAAAAGGAAAGTGATGAATCAATTGGAT

GGTACAACCACCTTTATTTCCCATTTGTTTCAAATCATACAAGTTTACTACCTTCATGAT

TAACTCCTTTATCTAGTTTGTCGCATCGTTTAAGTCTGGTAACGATAAACTCCGTGTCTG

TTAGAAAATTCTCACACACGTCTTGTGCCAGTTGCCCTTCACAGGGAAATACTCTCAGTC

CCTACTTACACAGGCACGCTAATCAAGACGGAGTGGATTCAATTTTCAAAGAACAGGTAG

CTTTATTATAGATAAGAGTAGTTGAAATTTTTATCACATTTTTGAGGTTGTTGGAATAAA

AAAAGCAACTCACTGCTTTACGAATGAGTTACTTTGTACTAAAATATTTGTTGCATAATT

TCCCTAATAACCTGAACAGAGTTATCAAATGAGATATCTTTAGCATACTTAAATTGGCGT

TGATAACGAATCCATAGCTCTTTCTGATAATCAGAAATTTCAATTCCTCTCACTTGTTCT

TCCCAATTATAAATAGTTTCTTTACTTTTACGTTTTTCTGCCGTATTCTCAAGTGCATTT

TTTAATACTTCTAGATTAATCTCTTCTTTTCTAAGTTTATACAAGGTGAAAAGATCATAT

CGATCACGTGGGCGTGTCGAAGCTAATCCCCTACTGATGATTGTTTCTAACTTTTCAGCA

AGTACAGTCTCTAGATTATATGTCCATATCTTGATGCTTTCATTAGTAAAGATAGAAGTC

ATTGAATAAGTAATTTCTCTTGGTGTGATCTTATCACCAGTAGTAATATCAATAAAAACA

ACCTCCTTTAATGTATCAAAAGTTGCATTTAATTTTAATGAAAATCCTCCATATTCATCA

TCTTGGCGAATAGGTTCTAGCTTATCTACTGAAAATGAAAAACCATCAGTTTTAGAACAA

AGAATCTCTTCAAAGATATCAATCAAATTTTCTCTGCTCATTTCGGTTCCCTTCAGAGTG

ACATCTAAATCCATTGTAGTTCGCTTATCTAGTCCAATCATTTGACCTATTAGATACCCT

CCTTTTACAATGAAGGAATCTCTATAAGAACTAGTAGAAATCAGCTTTAACACTTGCTCA

ATTAAATAATGTTGTTGTACTTGTTGAGCTGGAATTCCCTTATCTTTTGAAATATTTTTG

ATTTTTGCTTTAAAGCTATTCGCATTTGAAAACATTAAGATAGCACCTCCGTATAAGATT

GTAACTTATCAGTTACTTTAAATAACTGGGCATAATAAAATAATTTGTGTAAATGAATTT

GATTCTGTTGAAAATATTTCTTAAATGCTGGTGCAATAATTTGAAGATCCACCTGATGAA

CTGGTCTTAGACACTCGACCAAAACTCGTTCAACTTCATAAACTTTAATAAATTGACCAG

GAAGACGCTCTATTTCAATAATTCCCTCACTATAGTGAGAACGTAAAATAATAGGACATA

TATCTGCTTCCTTAATGTTTTTGGTATTCGTACCATAAGGAAAACTCATCGTCATGTTAA

AAGGGATAGTCAAAGATAAACCATGTAACCAAAGTGCTGTCTCCAAAGAAAAGATTCCCT

TTGGAAATCGATACTGAAGGACAAACCACTCATCTAAATAAATATCAGGTAAGCGATATA

AGCCTTTTTCTTCAGCATCGATTTTTCCTTCTGTAACCATTTTTAATAAGGTTTTGTAGT

GTAAATTTTCATCTATTACTTGTTTAAAAGTTAAAAAACCATACTGATTGAAAGAGTTCA

TCAGTTTCTCTCTTTTATCAACCATATCATTCTCCTTTCTTGGAAACAAAATGCTAACAT

CCTCTATATATGTTAGCATTTTATTTCTTCAAAATCAATTCATTTAGTTTTTTATTTCAA

ACTGAATCTATAAGTAAGACAGAGCACCTATTTTAAAAAAGCAGAAAACTATAAACTAGT

AGGTTCCACACCAAATGTAGCCCCATACTGCCCCATAAATCAGATTTATAGCGCACCATC

CCTAAAAACATTCCCAATGAAACATACAAACACCAAGCTAGAATGGTTCCTGGATGATGA

GCCAAAGCGAACAAAACACTTGTCAACGCAATTCTGATATCTAATTTTCTGACCAAGTTC

CATAAAATTTCTCGACACAAAAATTCTTCAACCATACTCGCATTGATTAAGAAAAGAATG

GTGATGTAATCATAATTTCAATTTTTAAATCATGATGCCCATTTTACCTTTCTTGTCTTA

ACTGGTTTAAATCGAATCTTATTAATGAGTCTAAGCTCTATTTCTCTACAATCTGTAACG

GAGGTATCATTGATTTTAAGGCTTCTTGGAAAGTATTGATTACCTTTTTCATGTGATAAT

TCTACTGTATAACTGATACCATTACTTAATTTAAAGACTACAAAATAAATATGATCTCTT

TTAAATCGGCGACCGACTTTTCTATTCAAAATGCCATCAGTTGTATAGAATTTACACTCC

ATTACATGTAACATTTGATAAAGCAACAAGGTTCCAACTATTTTGAATTTATTTTCGTTG

AACCAACCATTATCAGCTTTATTAAGACGTTCCAATGTCCAATCATCTTTTAATCCTTCA

AAAATACTCCCTGCATTATTAGTTGGTAAATTAACATGATGTTCTCTAGATAATCCTAAA

AGATGAGGAATGGAGGAATTTGGGACTGATAGAATAATTTCAGGACAGTTCTCCATATTT

GTCTGATAAATATATTTGTACTGAGAAACATTTTTCTCAATAGTATTGGCCAAGCAATCC

ATCTTAACATTGAGAATATCAATCTCTCCATCAAAAGTCTTTAGCCATTCCAAAGTATAA

GTCATTTGAATTTTCTCCTAAAAAAGAGAAGGAAGCTCCTGCTTCCTTCTCTCCCACGTA

TTGAGTTTCACCTCACGCTAGATATGCCCTATCTGTTCCTCAAGGGTAGGCAGTCCTGCT

ATTCTTTTTCTCAACAAACAGTCTGAACTGAGAGCGGAGCCCACAGGCGGGTGCAGACCA

TATCCACTAGCCCACAAGTAATTATATCATTTAATTACCTTCATTATACGCAAATAGTCC

ATATTTGTCAACAATTACATTCATTATGAATATCTAAATAAATCAGGCCACCTATTTTAA

AAAAGCAGCAAACTATAAACTAGCAGGTTCCACACCAAATGTAGCCCCATACTGCCCCAT

AAGTCCGATTTATAGCGCACCATCCCTAAAACCATCCCAAGTGAAACATACAAACACCAA

GCTAGAATGGTTCCTGGATGATGAGCTAAGGCAAATAAAACACTTGTCAACGCAATTCTG

ATATCTAATTTTTTGACCAAGTTCCATAAAATTTCTCGATACAAAAATTCTTCAACCATA

CTCGCATTGATTAAAAACAATAAAAATGAAAACCAAGGAACTTGATGTTGAAGGCCAATT

AAGTTTGCTTGATTCGTGCTTCCTTGAGCATGAATCAGACTAAAACATAGACTTATAATC

AGTAGGCTGACAAATCCAATACCAAGCCATTTTATCCTAGTTTTCATATTAACCTTGATC

ACTTGTTTTCGTTGACCATACATCCATAAAAAAGAAATGAGCGACGCTCCATAGAGAATC

TGTAGTATAGTTAACTCACCGATACAAAGCATTTTCAGTAAGTATAGAGATACCAATAGG

ACATTTGCTTGTTGGAATATATAAACTGGAATTATTCTTTTCATAGTTACCTCCGAAATA

AATCTTCATAATCTAAATCTAATACCTGCACAATCCTTTCTACCCATGGACTTTGAGGCA

TTCGTTGTTCCATCTTATAGTGGCGAATCTTTTGATACAAACGATTCAATTCACTTGGAT

AGTGAAACTCTCCCGCAAACATTTTTCTGGTTAACTCAATCCAGCTGATATTTCTTTCAG

CCAAAATAATGGACAAGTTCTCCCAAAATCGTTCAGCCATATTGCTTCTCCTTTAGTTAG

ATAAATAATGTGTTTGTGCCATGTAAATCAATTGTTTCGTATCTCTTGGCAATAAAGCTC

TAACCTCTTCCAAATTCAGATTTGGATAAACCCGCTTATTTGAAACCGCAAGAGGAAGTC

TGATGTTTAGTTCAGGATTTTTTAAAATCATCTCAACGAAATCCGTTAATCTTAGATTAT

CACGGTTCTTAAATCGTAATAAATTGGGAGATAAAAATTCAAAACAATCTGAAGAATAGC

TCATCATCTCAATTAATTTATCCTTTGTCATTTCAGAAACTGAATGACAAGATACCTCAA

TGCCATAGTTTTGGAAGAATTCTAAAAGAAGTTGATTTCTATGGTTATTTTTACTTAGAT

AGAGATCAATCACGGGAGACCTCCCAAAGATTCGGTTCCATTTGATATTCTGAGACGATT

AAGGAATCTAATAAATTGGAGAAGTTAATCGGTTTCTTGTCTTCATCATAAGCTTTTACA

GTTACTTGGGTTGTAAGTATTCCCTCTTTTCCCTCGGCTCGATAGCCTTGCCCATATAAA

ACAAAAACAAGATTCTGATTATCATCTACAAAAGCATCAGCTCCGTTCTTTATATCCTGA

CTTTCAAGGAATTCCATAATGTTTTGAAGATAGGATTCATAAAATAGTGGGTAATTATGT

TTTTTATGGTAATAATCTAAAAATTTTACTTCAAACTCACATGGATAATTGGGCATCAAA

AATATTTGTTCATCCAGCTGTCTGATTTCTGCATCATGTAATTCTGTTTCTAATTCATCA

CAATCTAGTATTGATTCTTTATTTAGTGCTTTCATCTTTTTCCTCTATTTCTTTTAATTT

CTTTGCGATTGCGGCAATCACAGGAACGGTTACACTATTACCAGCTTGTTTATAGAGCTG

ACTATTACTAGAGACTTTTCTAGCAGCTTCAAAAGCCCAATCAGGAAAGCCCTGCAATCG

AAAACACTCTTTAGGGGTGATTCGTCGTATTCTCAAACGGTAAAATTGTCCATCTATTAA

AACACCAGCTACTTGGTAAACTTGTTTATCTTCTCCTTCATAGCTAGCCACTACTACTCC

CATTTGGCCACTAGTTGTTAACGTATTCGCTATACCTTTTCCAACTCTACCACGACGATA

CTGAGAACTTGGTCTTTCTAAATTGATTGAATCCCCAATCTCTGCTTGAGCATATCCTTT

TTTCGTTGCCTCTCGGACTTTAAGAAATTGGATTGGTTCTGGAATCAGTATTTTGGGGAT

TTTATCTCCTCCTTGCATCGTAGTCAGTGTTGGAGATAAGCCCTCACTTCCATAGACGCG

ACCAGTTTCCTTAAAGCTAGTTGGTAAATCTCCAACAACGACAATACCATGGCGATCCTG

AGTATTTAAAGTAAACATTGGCTCTTGATTATCCTTGAAACGTCTACCATTTTGTCTCTT

GTCTAATCTGTCTGGTGTCATACAAGGAATCGCAACTTTAAATCCTTCTCCTTTTCCACG

AACTAAGGTTGGCGCAAGACCTTCTGAATAATAGACTTTACCGCTCATTCCACTTTTTGA

TGGATTCAAATTCCCTAATATTTTTAAAGTCTCAGAGTTAGTTGCTTGACCTTCTCGTCT

GAAAGGAAATAAGAGTCTGGTACCTCTCTTTCTAGAATGTCCGATAATAAACACCCTCTC

TCTGTTTTGGGGAACGCCAAAATCCTTACTGTTAAGCACCTGCCACTCAACATCAAACCC

CAATTCATCAAGCGTGGTAAGGATTGTGGTGAACGTCCGTCCCTTATCGTGATTGAGTAG

GCCTTTAACATTTTCAAGAAAAAGAAAACGTGGTTGGATTTGTTTGGCCGCCCGAGCAAT

TTCAAAGAATAAAGTGCCTCTAGTATCTTCAAATCCCAATCGTCTTCCTGCGATTGAAAA

TGCTTGACAAGGGAATCCCCCACAGATGACATCGACTTTCCCTCTAAGTTTTTTAAATTC

GTCATCTGAAACATCTCGTATGTCATGAAATTCAATTTCTCCTTCCGTTTGAAAAATGGA

CTTATAAGATTCTCTAGCAAATTTATCAATCTCACAAAATCCAATACACTCATGTCCAAC

ACTTTCCATTCCAAGTCGAAAACCACCGATACCTGAAAATAAATCAATAAATCTCATTTC

TTTATCACCCCTTTCTTATCGACTACCATTGTCAAAACTGTCACTCCACTTGAAATGACA

ATAAGCATAACTAATATCCACTCGATTGGCGACATTTAACCACCTCCTTTTCTAACACGG

TTATTCCAAACAGAATACAAGCCATTAAACACAAATTCAGCAAGTACTTCCGCTCGTCTA

ACAAAGCGAACATTGTCAAGAGCATACTGATAGATTCTCTCAAAATCCGTCATAGCAATT

TCACTGGCTGTTTCAGAAAAACCTTCTCGTCTAAATTGATCTTGTACCAATCCCCAAATA

TAATCTCGATCATATTTTGTGACCTTTTCTACTTTTCTTTTCAAGATAGGATGAGTATTC

CTATCCTCCTCATCCTCAATAAATAAAGAATCAGTCTCACTATATTTAGTCTCACTAACT

TCAGTCTCACTAGGGGCTGAATGTGAGACGGGGGCCGTTTCATTTTCAACCTGCCCTAGT

CTTTTTTTAACACTAGCCCTGTTTGAATTACCTACTGGGGTAGAAGATAATTCCCCTAAA

TAAATCTTATTAGCTAGTCTTCCTTTCTCACTTGAAGACTGTTGAACTTCATCAATTAAG

TCATATTCTTTAAGAGTTTTTTTGATGGACAGTAATTTTGACTTCGAACAACCTAACAGC

CTCATCAGTTTAGAATTAGAAAATACTAAATAGACTGATCCTTCTTCATCTATCCAACCA

CGACTAAGAGATAATTCTAAACGATCTTTTAAAATAGAATAAGCCACCTTTACTTCTAGT

TTCATATCCATATATTTCTCATCCTCAAAAAGAATTTTAGGTAATTTGTAATACCGTTCT

GAAGTTTGGTATTGATTTGCGGTAATTCGTTTCATAGCGCTCCTCCAAGTTCTTTGAGTA

TTAAATCTCCACTTGATTCCAATCGAACCAAGCCATTTTTTTCTAATTCAGCCATAAGAG

AGATGGCTTCAACAATGTCAATTCCCATTTCACGTACTAAAAATGAAATGACAATATAGC

GTGATGATTGTAGTTCTTTTGTCATTTTCCCTCCTGAAAATAAAAAAACGGAAAGATTGT

GAAATTCCTTTCCGTAACATAATAAATATTTGAACACCAAACTCAACACCAAAAAAATGT

TTTTATTGATAATTATTGCTCAATCAAGCACTTATAACAATGTAAAAAATAAAAATCTGT

ACTCACAAAAACCCTTGAAATTCAAGGGTTTTAAGCATTTATTCTACTTCACAATGTTTT

ATTATTTTAAATCTTCTAGACCAACCATTGAATAGTAGCCATTGAGTTTTTTCCTTTCGT

AGC

>ICESpnGA13494

TTTTTGGCAGGAAACCAAATCAATTTATCAGTTTCTATCAATCGCTTATCGCTCTCAAAG

ACTGGTAAATAGGGATTCCGCAATCAAATTGCGATACTCTATTATTTAAGAGTAACAATT

TGCAAATCCTATTTTCTCCCAGTATTAGTGGGTTAATTCCTCAATTTTCAACTCTGGCAG

GAATTTGGTAGGTAAATTGATAATCCAAGATTATCTAAACTGTTTGTAATCTCTTCTGAA

ATTGTTGAATCACCTGAATAATACCAATGACTATATGTATTCAACGTGGTTGACTTATCA

GCGTGTCCTAATCGATATGAAACATATAAAGTATCCTTATGTAACACATTTATAAGATAA

GATGCATGACTATGTCTTAATCCTTTCCCCGTAATTTCTGGTACACCAGTAATCTTAGCA

TGCCTCTTTATTATCCTAGAAATTGTAGACTTACACAAAGGGGCACCAAAACGTGATATT

ACATAATCTTTATCATCATTCTTGATTTGAACTTCTCGCCATTTTTTAAGTATTGTAATC

GTAAAATCATCTAAATCAATTTTACGATTCCCTGCTACTGTCTTTGTTTGTTGTTTAGCA

TACCATACACCGTTTTTGTCTTTCTCAATTGTCGAATGAACATGAATCCATTTACGTTCA

AAATCAATATCTTCCCATTTAAGTGCAATACCCTCACTAACTCTTAAGCCAGTCATAAAA

TATAACCAGATCGTCATGTAATTATGGAGTCCCTCATACTCACTAATATCAAAAGAGTTT

ATTATCTTCTTAAATTCATCAAATGTCCAAAATTTTGTATCAGGGTGCTTTCCTCTAGGA

TTATCTAATCCTTTAAAAGGAACTCTATCAATATAACCCAATCTTTCTGCATAGCCCAGA

CATGCTTTAAATCTAGACCACATATTTTTAGCGTAGTTACTAGAATACTTGTCTATAATA

GCTAAGCGAAATCTTTCACAGTCAATAGTACTAATATCTGATAATTTTTTACTACCAAAT

TGCTTAATAAATAACTGATGATGTGGTAAAGCGGTCTGATAAGTTACAAATTGAACTTTT

TGTTGATAATATTTTAAATAAATCTCTTCCATAAACTCTCTAAAAGTTAAAAAACTATTA

TTAATAGTAGAGTTCACAAATTCATGTTTTAAACGAAGTATCTCTTCATATGCTTCCTTA

AAAGAATTAAACGGTAGTCCCTGTTGATTTTTCCTTCCTTTCTTTTGAATTCTTTTCCCA

GTTATTGGATCAACTCCTAATTCAATTTGAAAATAAATTTTTCCTTTTGAATCTTTGTAT

ACACCTTTATATTTCTTTGTCTTTGATATAACCATTTTTCTCACCTTCTATATCTGAAAA

AGAAATACCAATTAAATTTTCTACAATATTTTTAGGAGCAATGCCTAACCTTTTATTATC

GAAAGGTGAACATCCTAATTGTACCGCATTCTTATCATTTTTTCTAGCTTCTTCAAACTT

TTTTACAGCAATCTTTTTTGCTTGTCTAATAATATTTCTCGATGTGTGCTCTGGAAAACC

AATCGCAACTAAATCTTTATAGTTTACTGTTTCCATGATTTTACTACTTCCTTTCTTAGA

TTTTTTCACCTCCATGGCATGACTGATGAAGTCATCATAAGAATTACACTTGCTGCTCTT

TTACGGTGGCAGCCTGAACGGTCAGAAGTATCATTGTATATCATTTGTATCATGGCATAT

AAAGTATCGCTCTATTTTATTGATGGTTTTAATGTCGCTCTTATCATGGCGAGCCATTCA

AAACTGCTCCACAAATGAGGAAAGTACCATTTTGGACTATTCAATTGTCAATGTGCTTTC

TTAACCTACTACTTTTTGGAGTAATGGTCTTCTTTATTATCCACTCGACCTACAAGGTAG

TCTAGACTCACCTTATAAAAATCTGCAAGTTTGATAAGGTCATTTATAGAGATTAGCCTG

CTACCAGCTTCCATTTTAGAATAAGATGATCTTGTACAATTCAAAACGATTTTCGCAATA

TATTCTTGTGTCAAATCATCATCTTCTCGTAAATCTCTAATCCTTTTCAACATTTGTTGT

CTCCTAAAACAAGTATAATATAGTTATTTTCTTATTTTTAAATATGTGACAAATTGGCAC

ATGAAACTATTCTATATATACTTTTTAGGAAACTGTTTACTCTAAATGTATCTCATTTTC

CTCAATAGATTTCATATTTTGTTTTTCTTATATTTCTTTATTGTTTTATTAAACATTTCA

AATAAATTTATTGCTTCTCCATGTTGTTCCTTCAGTGTTTCAATCTTTCCTTTATATATG

TATAAATCCTTCTCTATCGTATCTACTGAACTTTTACCTTGAATACTATTTTCTAAATTG

CTAGAACTATTTGTCGTATTATTCAACAATACTTCCTCTAAATTAGTTAAATCATCAATT

TTATTTTGGACTTGTTCAATCATATTCTCTAGATATGATATTTGGGCAATGAAATCATTT

GTTATATCTTCAAAATCATTCGGACTATTTTCCGCAGATATAAGAAAATCTAATTGTTCT

ATTTTTTCTTTAATTTTGCTAAGAGGTATTCTTCTATACATATGCTGCGGTTCATGCTGA

AGCGTAAATTGCCTAATTAAAGTTTTACCTTTCATAAAGCGATTTTTGTCTGCTGAATCT

TTATGATATACATAGTAAGAACTAGTTTCTCTGAGAAATACTTTAACTTTTTCTTCTTCC

ATATTGATTTGAATATTTGGTACAAAAATAAGTCCTTCTTGTCGGATACCAAACTGTACC

TTAATGTAAATTCCATCGTCTACTACTGCTTCTATTTGATTAAGATTCAACTCTACTTCT

AACTCATGAACAGCATCCCTATTTCTCTTGAAATCTTGATAGGATTTCCATACCACCTCT

TCTGTCGGCAACTCTTTTTCTTTAATTAGCTTTTCTTTATTGTAAAGTTCAATTAAATTA

TTATTATCTAAGACAAAAGTTTCATTTTTGTTATTAAAATAGTCTTGAAAATAACTAACA

CTGTACTGATTCATTTTTCCCAATTCCTGTTCTGAAAGCTTAATCCCATTAAATTCAAAT

AGAACATGTTTTTCTTTCGGAATTATTTTTAAGTCAAAAAATTCTGCTTGTTGAATCAAT

TCATTCATATTCTTCATTTTCGGAAGTAGAAATTCTAAGATGTTTATAATTTCCCTTTGA

ACAAACTTTTGCTTAAAATAAGTTTCATTATAAGGTTGTTTTCTATTCAATTTATCATCA

CGTACGACTTGTTTCATATTTGAATCAGTCATAAAAAAGTAACATGCTTGTGTCTAAAAT

CAATTATTAAATGTAAAGCTTTTGCTTTTTTCCTGAAATCTTCAAAATTTTTCGAGTTCT

CAATTAGAAAATATACCCGTTGTTTTATTTCATATTTGTAATTGGTTTTTCGATAAACTT

CATACTGACGATGCGAATAACGATTTTCTATAATCTTTGCACCTGCAATTTTTGAGAGAC

GATCTGAAACCATTCGTAGATTACGTTCTGACTTATAATTCCATAGAAACTTTTTATCAG

AATTCTGATCAATTGAATTTAGGATGATGTGATTGTGGATATGACCTTTATCGACATGTG

TTGCTACGATAAAACGAAATCTACCTCCTGTCAACTCTTTAACTGTCTCATAACCAATCC

GATTGATTTGTTCAGGAGTAAGATGATCATCTGGAGAAAAAGACTGAATAATATGATGAG

AATAAATTCTTCGTTGATTTACTTCTTGCCTATCATGACGAAATTCATAAAGACCATCAT

TACTTAAAAAATTATCATTGTACATCTTCACTAGTTCTTTATAACTAGGAAAATCTAAAT

AATTTCTCATACCAAAATCTGAAACTAGTGCTAGATTTTTTGTTTTACTTGGATTCAAAA

TATATTTGACTAGTTTGCTACGATAATTTTTTCCATGAATCGCAAAGTGTTTAGTGATGA

CCATAAAATTTCCTCAATTTTTCAGATTGAATAAGAAAATCTTTCTCCACTTCTACTATT

AACTCTTCTATACCTTTTCTCAACTCATTTAATTCAACCTCCGTTATTAAATTCGAATAA

TTTATGCTTCTGGCTATTTGATTAATATTATTTCCGATTCGTTTTAATTCAAAAATCAAA

TCTTGATAACTATTTGTATCAATGGTGATAAAATTCATACCAGGATCTAGTAGAGTTCGT

CTAGCATATTCTGAGAATGATAAGCAGTTACTTTTTGAGATATTTTCATTCAATTTGACT

AATTCTAGGTCAGATAAAAAGACTTTTTTTAAATTTGTTCTATATCGGTGCTCCACTCTA

CTACCTCATGTATTTGTTACGAAAATTTTCACTAAGAGGACTTGTTTTTTCTACCTCTTT

AATTAATTCCTGAATACAAGTTAACAAAATAGAAACATGTTCTTGAGTCACCTGATGATT

TATTTTAGCAATTATTAATATTTCATGAACATCACGGCTAATTTGTTCCAACTTTTGTTT

TTTCCAAAGGTTGAACCATTTTTCCATCTGTTTTTGTCCATCAGACAATAGTAAGCTTTT

ACGGAGAAAATCAGAAAAATTATCCTCTCCTTTCTCTCTCATTAAATCTAGTATTTGTTT

TTCTTCCGTTTCTGTTAAGCGAAATTGTTTCCGAATACTACGTATATCTCTTTTCATATG

GCTTTTCCTCCCTCATAGAAACTTGTACTGACAATGTAAGCTTACAGACACTGTCAGTAC

GTTTTTCAAAATAGATTCAACATTTTTGTAAGGCTTTGCGAGCTCAGATATTGTGTCCAC

AATATCCCAAAAATCATATCGCATGCAATTTCAACCCCCACGGATAAAAAAGCAAACGAT

ATGAAAATGTGGTGGCACGCCCCCACCCCCCAGAGAAAATCTAATATAGATTTTTAGTAA

GTTGAGAAAAGTATAGCGTAAAAAACTTGGAGAAGTTATCACAAAAAAACTCCATAATAT

TCATATTAGTTTTCACCATTACGAATATTATAGAGTTAATAACTGATGTTGATTTTAGAT

ATTGTTATTTTATATAATGCCTAATCTTCCTGCCAAAATATATACGTAATATTTTCTAAA

TGAACGATATACTTAAACTCTAAAAAATCTGGCTCTTCATAATAATCAATAGAATCAAAG

AAAAAATAATCATACTCAATTTTTTTAGAACGTTTGTATATGTACAAGCTATGAGGATGA

TTTTTGTTACCCCAAAAATCTATCTCTATATGACCAATATCCTTCTCTTTATAATGTCTC

TCTATAAATTGATTAACAGCCTCAACATATTCCATTATTTACCTCGTAATTACTAGGAAA

CTTAACTTATCGTTCTATACTAGCAAATGAATTAAACCACTTAATTAACTAAATTACTTT

CTTTTCCGGATTCTATAGTTTCTTTTAATAATTCAAAATCAACAAACTCAGCTAATCGAC

AATAATCACAATCGTTTTTACCACAACTACCAGTAAAATCAGCTGTATTCATATGAGACC

AGAAGTTTTGAATTTGTGATGTAACTAAGTCTAAATCCTCTTTCGTAACTTGTATAGTGT

GAATTGAATAGCCACTTTCAGTACTATTATCATCAAGAAAAATATACTGAGTTTCAATTC

GTTTATCAGTCGTATCTATTTCAGAAGAATTTTCTAATAAAAGATTATAGAAGACTGCTT

GACGCCAATAATCATGTCCAACTTCTAGTTCCTCAACACCTCTTTGAGCAGAACCTGTCT

TATAATCTACAACTCGAATAAGATCATTATCAAATTCAAGCTTATCGATAATCCCATTGA

TTGAGTAATTATCTCCTAATTTAACACGAAGTTCTTTTTCTAAGGAAACATGCTCCCCTT

TTAAACTATTAGGAATAAAATGTTCAAAAAGATTAGTCAACAAGTTATGTCCATATTCTT

TTAATTCTTCTACAGACATTGTATCAATGATTTCCTCTTGATGAGAGTACAATGAACTAT

CATAGAATTCAATTGCGTCCTCTAATTGTAAATCTTCTTTATAGATTTTTTCCATTGTTT

CATGAATTGCAGTCCCAAATACGGGACCTGTGGGGGCATGATTGATTGTTATTTTTGTAG

ATTTTTCTTTTGCTCCTGCTGATAATCCAGCTGCTTGAGATAACTGTAAAACATCTTCAA

AGTAGATACTCTTCGGACAACGCAGGTAACGATTCAATTTGCTAACGCTTAAACTCTTAT

AGTTTCCCCGTGTTTGTTGATAAGTCGCAACTTCTAAGGCTTCAGTATTATCAAATTTAG

GTGTTGTGTTAGCTGATACTTGGAGACAGTAGTCGCATGGGTAATTATCTCTGTCCTTTT

TCAATACTCCACAACCACAGTTAAAATCGCCTTGTTTAATTTTCATAATAGATTCTTTAA

TCTGATTAAGCACTATATCAACTTCTTTTTGAGTGATTCGTATATCTTCAGTTTCAGAAA

AACCATCTTCATTAGTTGAATTCTCAACCAAAACATATTTAACAAGTATTTCTTTGTCAG

AAATATCGATACCGGCATTTTTGAAGAGGATATAATAGAATACAGCCTGACGCCAATAAT

CACCACCAAGGGGTTCTTTTTCACTCGGAGCTACTAATTTCTTTTTAGCATTTTCAAAAT

TTCCTGTTTTATAGTCGACTAGACACACTATATTGCCGTCGCACTCAATTTTATCAATTT

TTCCGTTTATCTCGATATTAGATACTTCCGATAGGTCTACGGTGGTATTTAAAATATCGC

CTAGTCGAATACCTTGAATAGGTACCTCAACTGCAACTACACCATCTTTGAGGTATCCAG

ATTTCTTGTAAAGATTTTCAATAATCTTTTTCCCTCTAGCATAGTCATCTTTCTTTATTT

TATTTGAAGTCAATTGATAAGATTTTTCTTCAAAGACTGTCTCAAATAATTTTAAAGCTT

CTTCTAGAGAAAGTACTGTTTTTGCAGTTAATTCTGAACTTTGTGAACCATCTACAGAAA

TATATATTTTTTCTAAGACTTCGTGAATAATGCTACCGAATACCATAGCTTCGTTTGTTT

CAGATGGTAATTTTAATCCCTTATTGAAATAAAAACTCAAAGGACATTTAAGAAAAGAAT

TTAATGTACTAACGCTTAAACTCAATTCGTCAAAAATTTCATTGATAACATTGTTAGAAA

ATTTCGGCATTACCACTTTATCTACAGATTGACAATCCTCAAAAGATTCTGTCACGTCTA

CCTCATCAATAAATTCCTCAAATTCATTAATTAAGTGGGTTTTAGTAGAATCATTAGCAT

AAGAGAGATGGAGCACCTTCTTTGCCCGAGTCATAGCAACATAAAACAAGCGACGGTTCT

CCTCTTCTTTTAGTTGAGATTCATTTTCATCTTTTCCATTTAAACTTGGTGGATAAGAGA

ATCTACCTGAATTATTTTCACCTCCTGGCCAAGCTTCACTCTTTTTCTTTCCATCATTGC

ATTTCATTATAAACACATGATCAAACTCGAGTCCTTTGGAACCATGAGCTGTCATTAATT

GAACGCAGTTACTTGGAGAACCTGAGATATCTTCAATTGGTAGACTAATTCCCATTTCTT

CTAAAGCTGATAATTGATTTAAAAATCCTTTTACAGTCAATTTAGGATGGATGTGGCATT

CCATCAACATTAACTTGTAAAGTTCATTTAAAACAGAAACCAAATGATATTTCTCTTTTG

ACTTCATAATATACTCATCGATACTTAAAGATTTTAATACATCGCTAAGGACATACATAG

GCGATAGGTATTTTGATGATTGATTTACTTTTTTCGAATGATTTCTAATTTTTGATCAAT

TTCTGCTATAGTGGGGATTGAAATTTTTTCATCTTTCTTTAGATTCCGTATCATTACGAT

TTGTGATAGTACTACCTCAAAGAAATCAAAATATACAATTTTTCGAAGTTCTCTAACATC

TCTTGATGGTTTACAAAGATATTTTAAAATAGCTACAATTTTTTTGAAGAAAGGTTCACT

AAATAAATCTTTATTTTCTTTCATTTGAACGAAAATACCTTTATCACGAAGAATCTTCGC

AAACTCTTCTCCGTAAGAATTTCTTCCATAAATGACTCCTATTTCATTAGGAGAAACACC

CGAATCAATCAGCTCTTTAATCTCAGTAAGCACACCAAACATTTCAGCTTTTGCATTTTC

GTAGGATTTCAAGATTGGCAATTGATAGTCATAATCTTTGTTAGCGCCTGCTGCAATAAG

AAGTTTCTCTTCTCTTGGATTATGACTGATTAGTTGTCTTGAAGCGTCAATGATAGCTTG

GGAAGAACGGTAATTTTCATCAAGTACTATTTTCGTAGGTTTATACTTCTTATCAAAATC

TCGAATATTATTCGCACTTACTCCTTGGAAACGATAAATACTTTGATCATCATCCCCTAC

AACAAAAATATTTGGATTGTCTTTTCCTTTTACTAGTAAATCTACAAGTTTATTCTGAAC

AACTGAAGTATCCTGAAACTCATCAACAAATAAATATCTTATAGTATCAGATACACTTCT

TTGAAAACCTTCATTTTCCTCCAACTTTTCAATTGTCCATAAAATCATATCATCAAAGTC

AAAATAATTGTGATTGCTAATATCAGAGCTATACTTTTCAATAATTTCAACTCCAGCAAG

TAACTTTTGAGTATTTTCTTTTAATTCGTTTATAGCCTTATCTTTATAATCTCCAACTTT

CTTGTCTTTAGACTTAGAATTTCTAGCATATTTGAATTTACTATACAAATCATCTCCAGG

TATGGATAAATCAATCATTTTAAAGTATTCATCTATCTCTTTTTCAAAATCCTCTTTGTT

CAGGTTTTCGCGTTTCATTTTATAAAAAAGTTCTAATAGCTGGTCTTTAGCAGAATAACG

TTTACCAGAGTTTTTATAAAGAGGATTTCCTTCTATAACATATTCATCCATAATTTCTTT

TAGGATTTCATATTTGGCTGCTGTTGAGATAATTTTTGGTTGTCCACCGAAAATTTCAGA

GTTTCTTAAGATTAATTCACTACAAAAAGCATGGATAGTTGATACTCTTACCTTACGCCC

TTCTTCTCCAATTAATTTTTCTAATCTATCCAACATTTCTGATGCACCAGCCTCTGTATA

TGTTAAACAGACAATTTCTTCTGAACTTGTATGATAATTTGTTAAAATATTTGCGACACG

ACGTGACAGAATTTGAGTTTTACCTGTTCCAGGACCAGCAATAACCATAACAGGTCCTTC

CACCGTATCAACTGCTTTTTTCTGTTGTTTATTAAGTTTATCATATTCTTCTTTAAATAA

AGTTTTAAAATCTAATGTTGTCATCGTTTTACCTCGCTTAACTATAAGTACTTCTTTTGT

GTTAATTATACCATATTTAAAAGCGTTTTCACATATGGTGTTAAATACTTACTTAAGTAT

TTTAGAATCTATAAGATAAGAAAATCTAAATATTAAAATCATTACGTTTTGTTTCAATAA

CTCTCATTGCCGCTTTAACTGCATGAAATTCATCAATCAACTTTCCAAGAAGATAGTAGT

TTTGTCGAGTTAATCTTCCTGACATTATTTTTTGGGAAATTTCTTGGAAATTATATAAAT

GACTCTCTACACTTAATAACATTTCCTTAGCATCTTCATCAAGATATAGTACTTGTGAAT

ACACATCCTCTACATTCCAAGCAAGAAATTTCCCCTCTGCTAAGTGTCTTAGATCAGATA

AAGTTAGCGAAATCATATCACCTGTAACATACCTCCCATGAAACTCTATTACTTCCATTA

TATTTTCTAACCATGCTGTTTCAAAATCTTCTCTTATTTCACATTTATACATCTACTGTT

ACCTTCTTTCCTCATCTGTATCTCTATTATAAATGAAGAGGTAGACATTTTTTGTCTACC

TCTTCATTTTTTATAATTTCTTAATGTGTTTTCAAGAGATTCTTGTATTTCTTCAATCAA

CTTTGAAGGTGATAAAAGAGTGACTGCATCACCTTGTCCTAGAATCCATCGTTTGAGACC

TGGTGTGTCTTGAGTTTTAATTTTTACTTTTATTTCATCGTGATTCTCAGAAAGAATTTC

ACGCTCTGGAAACTGATCTAAAACAATTTCAGTATTTCCTTTATAGATTAAAGTAACATC

AATTTTTCTTCCTTCAAAAGCATCTACTTTTTGATTTCGAACCTCCCCATCTCGGTACTT

ACGCCCATATGAAATTTCTGGTTTCTTTACATGAGATTCACTCAGACTCTCCATCCTATC

AACACGATACGTTGTATAATTTTCATGTTTCAAATGGTAAGCAACTAGATAAAAATAGTG

GGCATCATAATAGAGGGAAACAGGAAAAATGATGTGGCTTTTGATATCTTTTAACGGAGC

TTTATATTCTATTTCGAGCATTTGCTCCCTACGAATAGCTTCCGACAAAATCCAAATTGT

ATCAATCCTGTCCTTGTCATTAGTCAGTGATTTATAGTTAAAACGCTCACTGCCAATAAT

TGCATCTATCTCTTTTTGTTCTTCGCTTGAGAGTAAAGATAGAAGATCTTCGAGTAATGA

ATATAGTTCTGATTTGTTTAGTGCACGATTCTCCAAAAGAATTTTTGAAATTACTAAAAT

ATCCTTTTTATTAAAAGTTGTTTTAGCAATCAAACGATAGGTATTATCTGAAGTATCGAA

TTTTATCTCTGTATTTGATATACGCTGCTCTTGCAAAAAATTTTTTAATGTGCTCATATA

TCTTTGTACAGTCCTATAATCAATCTCATATTCATCGCTTAATTGAGCTTTTGATAGCCT

CTCGCCTGCTTGTAAACGGAAAAAGATTGTTAAAAGTCGTTCTTGTGGTTTCATATCTTA

CCAATCAATTCTATTAATTTAAAATAATTTATTGAAATCTTTATTTTCTAAAGCTGATAA

CATAAATACAACTTCACTTTATTTTTTCACATATTCCCCTAAGGTTTCCTGAACTAAGTG

TTTAAAACTACTATTACTAATTCTTTCATTATTTTTACAAAAATTTCGCCCAAAATATCC

TGATTGACCGTTATAATCTTTTTTCCTAACAGTAAAAAAGTATTCTTCATCTAGTGCGTT

TATGATTTGATTTATTTCTTCTTTTGTAAAATTCAAGTCTTGTTCTAAAACTTTAGATAT

AGTAGGTAACCAAAAAGTATTAAACCTTCTCAATATAAAGGCGGGTTTATGTTGAGATGA

CTCTAAATGCTTAACGATTCTCCAAATAATCACTCTTGCACTTAACAAATTAACTTTACT

ATCTGATTCAATCAGTAGATTTGTAAAGTTTGTTGCTCTTTTATTACTTTTATAAGTGTA

ATTTGGATTTTGGCTTCTAAATGGGAAAGCCTCTAAATTAGCTATTTTTTTTGACATTTT

TTCTAACTCATCCCACTCATCAGTCAAATTGTGAATAACATTCTTAAAAGAGCCTTTTTT

ATTTAAAAATTCTTTTATAAAGTGTGGCAAGTAATGTCCTAGATAGTAAGTATCTTTATA

AGAGCGAGTTTGTTTTACAATTTGTAATTCATTGTATAAAATACTGCTCGTTTCTCTTAC

ATCCAAGATATGTTCTTTGATATATACTTTAGGATCTATTCTCAATTTGCCTTTATCATT

TAAGATGTTTAATGAGTCGTCTCCACTCTCCATTTCTCTTGCTTTTTTATAATATGTGTG

AATTCCAACATCCTTCTTCTCGGTTTTTACTTCATCAATATTTGGATTCACTAAACATAA

AAATAATGTTGCTTCATCAATATTTCCTTTTATGTGATTAGGTAATCCATACTTGATATC

AACATCTGTACTTTTAGGTAAATCTTCCTCAATATCTAACCAAGAAAAATTATTACTATT

AGATTGTAAAGCCTTATTAAAAGATTCTATCATTTTTTCTATATTAAAATTATTCCACGG

GCTATATATATCGAATAGTTCTGAAGAGTTGTTAATTTTTCCCACTTTATCATGATATTC

ATCAAGCAATGTTTTTAAAGGTTCATTTAATGTATTTTTGTTTTCTGTACTGACATAAAT

CTATCCTCAATTTTATTATTTTTATGTTAATTTCATTAACAATATCATTTTACAACACTC

CATAGACAATTAATGTCTATATATAAAATTTCGTCAAGATTTTATTAATCTGTATCAATT

ACAAAACTAAATCATATAGCGTGTTATTTAATTTTTTAGGAAAAGATAATTTAAAACAGA

AATCATCTTTTCATCAGTATCTATTTGGGCATGATTACTAAGTTACAGTTTTATAATTTA

GTTATTTTTAAATTTATTTAATTATTTAATATTTTACTTGCCTTAGATAATAGTTTATTT

AAGTTATCTCTAAAATTCATCTCAAATTCGTGATAAAGTTCTTCACTTCGCAAGTCACTA

GCTAAAATATTATCTTTTATCTTTTCTACCAAATTGTTATTAACAACAATTGCCATTCCA

TCATTAAGCATTCGACCGTGCGTATCCCCTGAAATATAAATAAAATTTGATTTATTATGT

GAAGAGTATATATTTAAACCTTGTCCTTTTCCTCTTGTTAATCTGATTCTATCAAATTCA

TTTGGAATCGAAATAAATTCCTCAGATAAAGGAGCTTCTCGTTTTTCTCGTGTTTCTTTA

CCGAATAACTCTGGCTTTTTAAGATCAAAGTAATTTTCATCACCAACTAAAGTATTAAAT

AGTTTACGAATTACAAACTGCACATCTTCGTTTGGAGCATGATTCTGTTTTTTCTTATCT

GCTGTAATTGTATCTGTATTTCCAATTGAAACAGCATTTGAGAATGTCATTTTTTCAATT

AGTTTGTTAAATTGTTCCATGGTTAATCCTTGTTCATTATATCCAAGTAATTCCTTCAAA

GTTCTATAGAAATTGGTAAAATCATCTCTATCCTTCTTATCTTTAAATGTAAATTTTCCA

AGAAGCGATACTAATGAATTATAATAAACTTCATCATCTAATAATTCAAAAGAATTTTCT

AATAAATCTATCAAGTAAGATTCTATATCATCGCTATCTTCGGGACTATTATTATATGCT

GTCAATAAATAGTTGTATGCTTCCTCGAATCTTAATTGGAAACATACCATTGCTAATATC

ATCAATTTTTCATCATGATCTACTTTGTCTCCATTATTGATATACATTAAGAGGCTGACT

GAATTAAAAAGTCTATTAATGCTACGAGGATTATTTCCTATAGAATATCTAATTAATTGA

GTAATTGTTTCTAACTGATTTCTTTCTTTATTATTTTTATCAAAACAAAAATCAAGTTTT

TTAAGTGAGGATTCAATAAAATTCTGCAAATCATAATTTGCTACTGGTACTGTAAATGGA

ACTTGAATAATTTTTTCAAAGAAAGCTTTCCCCTTTTCATCATCTAAATCTTTACCATAT

TTACTTTTTGCACCTCTAACAACAACATTATAATCTATAGCCAATACAAAGACGCAATGT

TCACAGTCTAAAAATAATTTTAAAACCTCTAAGAGTTCTATGGCCTTTTCAGGAACTAAT

CGATCCAGATCGTCAATAAAAATAACTATTCGATCACTTTTATTTTCCTTAATGGCATCA

TTAATAATGTCTTGTAAGTTTTCTTTTAAATGCTTAATATCATCAGTTCTCTCGCCAAAA

CCTATGATTAAATCTTGAAATTTTTCAGTTAATTTTTCTCCATTTAGTATGCCAAAGTCT

AAATTTACATATTCTAAGCCTTTAGATAGCGCACCTTTTGCTTTTTTAAAAAACTGTTTC

TTTTTTGAATCTTCTACATTCAACTCACTAATTAAATCTGTAATTAATGCTACTGCTAAA

TTATTTCCCATATCAAACTGTGAGTATTGCCAAGTGTTGAAAAAAATAGTTTGAATTTTT

TTATCTTGTTTTTCAGGATTTAATCTTTTTTCAACTTGGTACATAATACTGGTTTTTCCA

GTACCCCAGTCTCCTTGAATGGCAACCGTTAAGGGTGTTTCACACTCAATAATAAAATTT

GATAAACCATTAATATAGTTCTCAATGCTAAATTTGTCATCATGGGTAGGACTATCAGAA

AATGAGCCACTCATAAGCAATCCTTTCATAAATATAAAAATAAGACAAATCACTCCGTAA

TAGAGTGATTAAACATATATATTGAATCTTTTTTGTTTTTAGCAAGATTCGATTATTTTT

TGATAACATATTTATTAAAAAACTGTACTTCCCCCTCACATAAAAAATGTAACACCTATC

TCAGATGTATTTTTGAATGAAGTCTTACAAGCTAAATTAAATTCTACGTTATAAACTACG

ATTACATTTTCAATTTGTGCAACATATTGTAAGTGTTTTCATATCTGTACTTAATTTTAT

CATAAATATTAACCATGAGCAATTTATAACCTACTTAATACCTATCGGTATACTCATTTA

ATTTCTTACCAGCACAAATTTTTAAACTTTATTTTCTAAGAGCTGAAATCTAATGTCTTA

GTTGTTAAAATTAATAATCTTTCCAAAACACATAGGAACTATTCTCTAAGTTAATAATCT

GTTTAAAATGAACAGAATCATCCACTACATAATAATCTATGTTATCAAAATTAAAGAAGT

CACACTCAAACTCTTCAGATTGTTTTTGAATTTCTAAAAAATATCGAGAATAATTTTCAG

AATCCCTTATTCCTATCTCTATACTTTCAATACGGTCAAGATCTTCTCTTGGATAGTAAT

TTTCTATAAATTGATTCGCAGCTCTAATAAATTCCATGCGTGACCTCATCTAGTTTATAA

ATCACATCTTTTCTCTCAATAATGATTTTACAATCTAGTTTCTCCATTGTAATTTTGTCC

AATAATTTCTTATTGGGATTTATTGCTGTCGGAAATCCTACAGATTGAAGCATTGTAAAA

TCACCAGTTGTATCTCCATAAGCGTAGGATTTTTCGAAATCAATAAACAATTTTTTTAAA

ACTTGTAATTTTGAAGTTGAATCCCACATAGGAATCACTTCTCCAGTATACTTACTATCA

AGTTGAAGATAATTGCTTGCATACCAGATATCTGCGCCCAATTTTTCGGCCATTTTTGAG

ACTAAGAAATCAGGAGACCCCGAAATAAATATAATTTGGTGCCCCTGTTCCTTATGCCAT

TTGATTCGATCACGAGTGTATCGATAAAGTTTTTTACTTTCTTTTTCAATAACTTTTTTG

GCAACAAAATCAATATCAAGTATGTCTTTATCCGCAATATATTTTGTATAAAGTGTTGCA

GCAATATAGAGATAGTCATCATAGTCTAGTTCTCTATTTTCCCAAGCATTTTTGTGAAAT

TTAATTTCAGAATTGACACTATTTGGAAATACATCATAGGAAACACACTTTTCCATATGC

TTTAAAAGTAAGGAATCTCTAAAAATTGTTCCGTCGATATCAAATATAGCTGCTATTGGT

TTATTCATTTGTATCCTTTCTATTTACATTTAATTAAAAGATAATTTTAAAAACATATTT

TTAATTGTTATAAAATAAAAGCTTTAAAATTTCTTTATAGTTATTTAAAATATCTTTGTT

TTTCGAAAATAAATCTTGGTTATCTTTAAAGATTTTATAAAACTCGTTAAAGTACCATCG

TTGTTTTTTCGGATCCTTTGAATTAAAACGGTCCCATAGTAAACCACCAATTATTAAGTA

ATCTTGATACAGTTCCATTAAATTTGCTAATTTATCAGAAAAAGTTACAAGTAAAACTTT

TCTTAGCGACTCATCTTCTGCTGCTTCTCGAAATTTTGCCAATTGAAGCTCTTTACGAGT

TTGCCAATCTTTAATTGATTTATCTTCAGATTCCAAAGACATATATGAATAAATTTCATC

ATTAAATTCTTGTTTAATATCTTCAAGACTGACATCGGTATCTTCAACAGTATCATGTAA

CCATCCGGTAGCTATAATATGAGGATCTTCTGTTAGGGTTTCTAATATATTATTGACCAA

AAATAAGTGAAATGAATAAGGTGTGATTTTCCCTTTTCTTGTTTGATTTTTGTGAGCAGT

TAATGCGAATTCTTTGGCATTTGTCATAAAACTATTTCCTCCTTTTGTCTCTGTTGAATA

TATATTTTTTATTGTACTTTTAAATTTAAAAATTTAGTAAAGTTAATCAACAACATACAC

TACTCTTCTGGAAGACATTCTACTAAATCTAAACCTTGATTTACTAGAAATTCAAACATC

TTTTTAGATAAATGAAGTTCATTGTTATGGAGATAAATCTTTTCATTTTCATCTTCTTTA

TAAGCATAAGCTGGTACATGAACTACTTTCCTACCTTTTACTGGAACAAAATATAATCTC

AAAAGTTCAAATAGTCTTTTTGGATTTTTTACAATAGCTTTCAATTTATTACTCCTTATT

CTTTTCTACATATTCTTTTAACTCAGGAATTGATGATTCAAGTGATTCTAATAAAAAGAA

GATTTCTTCTTTTTATTGTTGCCTTTCTAAAATAGGGACAATTCCATCTTTATGTTTTAA

ATAAAATTCATTTCCTCTTAAATAATCATCATAGTAATCAAGCAAAAATTGAACGGAAAC

AATTTCATCACATTCATAGTATCCATAAACTATTAGGTTTTCTGCTGTGTAAGGTAGGTC

CTTTATGGTTTTAACATCTATACTTATATCTTTTGTTTTTGAATTATATGTAATTGTTGC

TTCTTGTTCCTTAACAAAATTCTGACTAGCTTCAGCATCTACATACTCAAATTTGATTTG

AATATCTTGACTACAAACATATTCCTTCATTTGTCTTATAAGAACATCATCTGTATCTTG

AAATAAAGTACAATCAAAAAAGTCATCTATATTATTCTCAAAACACCATCTACCCTCAGC

AGTAAACCACATGTTGCATATATAATAACCATTTTCTTGAGTTGTTCTTGAGGAGACTAT

ATCTGATATGTTAGATTGGCTATCTATAATATCAGTTGGATAATAAACATACTTATTTCT

TTCTTCTTGAATTAACAGAAAATCCTCAATAGTTTTAAGATTTGATGCTTTAATGTAAAC

TCTGCCATGTGCTTCTGATGAATTTGCCATATATCATTTACCTTCTTTCTCTTATGTCTA

TATAAGTATTATACATCATAAGGTAGACATATTTTGTCTATGGTATAAAATTTTTAACAA

ATCAAAAACACATAATACAAGGCATTAAAAAGCATTGATACTACGTGTCTTGACATAAAA

ATATTGACTTTAATTAAATTCTAATAAAAAACTATACAGAAGTTTAAAAATGAAATGGTT

AACAAAATAATATTTTGACGTTTATACAATGGAATCATATTTTAAGAAGATAGATTGCTT

TAAACTTACTACAATTAATTAAAAAAGTTATTACGAGATCTAGATTTAAAGCTATTTAAG

ATCATTATAGATAACAGATGCTTGATTATCAATAGATTTAAATTCTTTAACAGATTTAGT

GAATTTAGGGATGTTAATAATTCGCTTATTTAGACATTGAGCAAGGTGTTGAGGTACATC

TGATCCATCCCAGTTGCCATAAAAGAAACTTCTATTTCTCCATTTATCTAAAGATTTCTG

GGGAAATTCTGAATTAGAGAAACTGCGTTTATATAATTCTGGATAATCTTTTTTAAATCC

TCTATATTAGAATATTTCACAATAGTTTCTTCACTAGTTTCTATTTTACCCCCTTCTTTC

CTTGTTGGGAAAGGATACTCTGAGGAATGTTCGTAAGCTTTCTTCCAACCTTGAATTTTA

TAAACACCAACACAAACACCACGGTATAAGGCAAGAGCATACTCTAAGTCTTTTTTACGT

GATTCAGAAATATTCCAAACACCACGTACATTGTGATAAATATCTTCTGTAGTGTCATCA

GCTTGAAAGCTATTATTGATTTTAATGAATACACATTTTTCTTTAATCGTTGAAAAATCA

AAATCAGATAAGGCATAGTTATCAATAAAGTTCTCAATTGATCTAGCCTGAATCAGTCCA

TCTTTGGAGATTGTTGCTTTTTCTCTTTTTGACGCATGTCCACTAACTAAATTTGTTAAT

TCTTCATTCTCTTTTGAAAAATTGATAAGATTAATTAATGCCGATTCAGCCATAAAAGCT

TCGTGCTCTGTTAAACCAAATTTGTTGAAGAATAAAAGACCACATTAAAAAATGTGGTCT

TTTGTGTTTTTTTAAAGGATTTGAGCGTAGCGAAAAATCCTTTTCTTTCTTATCTTGATA

ATAGGGGTAACTATTGCCGGCGAGGCTAGTTACCCTTAAGTTATTGGTATGACTGGTTTT

AAGCGCAAAAAAAGTTGCTTTTTCGTACCTATTAATGTATCGTTTTAAATGACTAGTAAA

AAACATACATAGAAAGGGGAAAAGCAACTTTTTTTATTGTCATAGTTTGTGAAAACTAAG

TTGTTTTTATGTGTTATAACATGGAAAAGTATACTGAGAAAAAACAAAGAAATCAAGTAT

TTCAGAAATTTATTAAACGTCATATTGGAGAGAATCAAATGGATTTAGTTGAAGATTGCA

ATACATTTCTGTCTTTTGTAGCTGATAAAACTTTAGAAAAACAGAAATTATATAAAGCTA

ATTCTTGTAAAAATCGATTTTGTCCTGTCTGTGCTTGGAGAAAAGCTAGAAAAGATGCAT

TGGGTTTATCTTTGATGATGCAATATATTAAGCAGCAAGAGAAAAAGGAGTTTATCTTTT

TAACTTTGACTACACCTAATGTAATGAGTGATGAATTAGAAAATGAAATAAAACGTTATA

ATAATTCTTTTAGAAAACTTATAAAGAGAAAAAAAGTAGGTAGTGTTATAAAGGGATATG

TTCGTAAGTTAGAGATTACATATAATAAAAAAGAGATGATTATAATCCTCATTTTCATGT

GTTAATTGCAGTAAATAAATCGTATTTCACAGATAAAAGATATTATATTAGCCAACAAGA

ATGGTTAGATTTATGGCGTGATGTAACGGGCATTTCAGAAATAACACAAGTTCAAGTTCA

AAAAATAAGACAAAATAATAATAAAGAATTATATGAAATGGCTAAGTATTCTGGTAAAGA

TAGTGATTATTTAATAAATCAAAAAGTCTTTGATGCATTTTATAAATCACTTAAAGGTAA

ACAGGTATTAGTTTATTCAGGATTATTTAAAGAGGCTAAAAAGAAATTAAAAAATGGGGA

TTTAGATTACTTAAAAGAAATTGATCCAACCGAATATATCTATCAAATTTTTTATATTTG

GAAACAAAAAGAGTATTTAGCTAGTGAACTTTATGACTTAACAGAACAAGAAAAAAGAGA

AATTAATCACAAAATGATAGACGAAATCGAGGAAGAACAATAACAAAATATAAGTGCTAA

CAGCTGACCTCCCGATAACACCATGTAGTTATTGGGAGGTCAGCTGTTGAATTATGCACG

AGTATTTTAAAAGTTATTGTGATGACGACGATAAACGATTATCAAAAGTATAATGTTAAA

ATGCTTTATTATACTAACGTTATATAAACATTATACTTTCGTTATACAAATTTTAACCCT

GTTAGGAACTATAAAAAATCATGAAAATTTTAATTTGCATGTAACTGGGCAGTGTCTTAA

AAAATCGACACTGAATTTGCTCAAATTTTTGTTTGTAGAATTAGAATATATTTATTTGGC

TCATATTTGCTTTTTAAAAGCTTCTGTAGGTTTTTAGGCATAAAACTATATGATTTACCC

CTAAATCTTTAAAATGCCCCTTAAAATTCAAAATAAAGGCATTTAAAATTTAAATATTTC

TTGTGATAAAGTTTGTTAAAAAGGAGTGGTTTTATGACTGTTATGTGGTTATCGATTATA

GGTATGTGGTTTTGTATTGGAATGGCATTTTTTGCTATCAAGGTTATTAAAAATAAAAAT

TAGACCACGCATTTATGCCGAGAAAATTTATTGTGCGTTGAGAAGAACCCTTAACTAAAC

TTGCAGACGAATGTCGGCATAGCGTGAGCTATTAAGCCGACCATTCGACAAGTTTTGGGA

TTGTTAAGGGTTCCGAGGCTCAACGTCAATAAAGCAATTGGAATAAAGAAGCGAAAAAGG

AGAAGTCGGTTCAGAAAAAGAAGGATATGGATCTGGAGCTGTAATATAAAAACCTTCTTC

AACTAACGGGGCAGGTTAGTGACATTAGAAAACCGACTGTAAAAAGTACAGTCGGCATTA

TCTCATATTATAAAAGCCAGTCATTAGGCCTATCTGACAATTCCTGAATAGAGTTCATAA

ACAATCCTGCATGATAACCATCACAAACAGAATGATGTACCTGTAAAGATAGCGGTAAAT

ATATTGAATTACCTTTATTAATGAATTTTCCTGCTGTAATAATGGGTAGAAGGTAATTAC

TATTATTATTGATATTTAAGTTAAACCCAGTAAATGAAGTCCATGGAATAATAGAAAGAG

AAAAAGCATTTTCAGGTATAGGTGTTTTGGGAAACAATTTCCCCGAACCATTATATTTCT

CTACATCAGAAAGGTATAAATCATAAAACTCTTTGAAGTCATTCTTTACAGGAGTCCAAA

TACCAGAGAATGTTTTAGATACACCATCAAAAATTGTATAAAGTGGCTCTAACTTATCCC

AATAACCTAACTCTCCGTCGCTATTGTAACCAGTTCTAAAAGCTGTATTTGAGTTTATCA

CCCTTGTCACTAAGAAAATAAATGCAGGGTAAAATTTATATCCTTCTTGTTTTATGTTTC

GGTATAAAACACTAATATCAATTTCTGTGGTTATACTAAAAGTCGTTTGTTGGTTCAAAT

AATGATTAAATATCTCTTTTCTCTTCCAATTGTCTAAATCAATTTTATTAAAGTTCATTT

GATATGCCTCCTAAATTTTTATCTAAAGTGAATTTAGGAGGCTTACTTGTCTGCTTTCTT

CATTAGAATCAATCCTTTTTTAAAAGTCAATATTACTGTAACATAAATATATATATTTTA

AAAATATCCCACTTTATCCAATTTTCGTTTGTTGAACTAATGGGTGCTTTAAATGATTAC

AGGTATAATTTTGCCCTTATGTTTTCTAATAATTTGAATTTTTTACTAATTTGTATGTTT

TCTTCTTGATTTATCTCTGTAAGCGCTTCATCGATATGTGATATTAGGCGAGTTCCCTCT

CCTTTACCAATATAGAAAGGTAGTTGCTTACCTTCTTCATAGAGTCCGTAAACATAGTAT

TTATCTTTTTCATTTTTGGTAGCACCAGTAATATCATTTAATTTTTGATAATTCATTTTT

TTACTCCTATTTAAATATATTATAAAAGTTCTGCTCCATACTATTAAAAATTAATTAAAA

TAATCCTCTTATTCTACCTTCATTCTGATTCTGAACTATTTATATGTTAAACTATATTTT

CTTTAAAATCAAGTAAAATCCTAGAGAATAATCTAAATAAATTTGGGGACAATATATAGG

TTTACTAATAACTACACAATACAGTATTTACGATAGCATTAAAAAGAGACTGTTTTATTT

ATGCTCTAGCTTTTTCAATGAATCTTCTAATACTTTTTCAACCTCTTTTTTAGCAGAATA

TCGTTTGACTTTTAATAAATTCTCAGGTTTTAAAGAAGGATTACTAGGATTACTCGTTTC

ATAACAATTTTTATACTCTTTTAATTGAGGAATTGCATCGTACCACTTATTGTAAGATCG

TGTGATGATAATGATTACAGGATCATTGTCATCCAAAACACGTTCTCTAATTAAATTGAA

TAAAAACTTTTGTGTTGGGAAATAACCTTCTTTGCTTATAAAATCATTTAATTTATCATC

GTACTTAGTAGAGTAATATGGAAAATATTCAGCTAAAGCAACGTGTTTTGAAAACCAAGG

AAGCTTTTCTTTTAAATCCTGTAAATTGTCTACATCATCAAACCATACTTTAAACTTTTT

AGCCCAGTAACCAAATTCATTCGTATCTAGGTCAAAAGCATGAAAATAGAGGTTTCCTTT

TAGCTGTAGATTATTTAAAAGAGTCTTTTGGTAGTTTTTATCGTGGTCATAAAGTGTTTT

AAATTCAGGGGTGTAGCCAGGATTTTTAGATAAAACAATGATATCAGCATTTTGAATATC

CCCCACAAATTGTTGCGGACAAAAACCTAATTGTAAACGGTAGATATCATCTTTTAGATT

TTTGTAGTGTTTTGTAGAGGTGTATTTTTTGATTAGTTCAACATCTTCGGTAGCAAGAAT

AGCTTGCTCTCCATTTATATCGACGTTAGATAATTGATTCCAGGGATTTTTAGTTGTAGT

CATTTTATTTTTCCTTTCTTTTTGTAGAATTACTATATTTTACGATGTTTTAATATTAAA

TACAAAAATAGATTTTATTAATATATTCTCGATTATAAAAGAATCTGAGTGAGGAAACGA

TTTTGAATATCAATAGATATAATATTATTTATATCACCTTTCACACCGCCGTAAATGATG

TTCGGAATACGTCGGCTCTACTAAACTGTAACGAATTCTCATTGCCAAGTCTACAAATTA

TTTTCAATACTATAGGCCAGATAGAATTAACTAAAAAGTATAAGACTAGTACAAAAAGTC

CGATAAATTGGAAATATTTTGAGAACAACGATTAATTTAAAAAAGAATGACGGTGAGATT

TATTACTTTTATTTATACTCACTTTGTTAGTTTAATTAAAAGCCATTTTTTTACCATAAT

CTAAGAGATAGCAATTTTTAATAGGCTTTATTTTAAAACTCAAGATCTAACAACCATTTC

AATTAGTTAAATCTTTCAATCTTTCTTCTCCAGATTTAAGCATATCCTTCTCTACTTGAC

TCCACTCCCCAAACAGTAACTCTTGAAGAACATCTGCCGCTGAAGTTGTATTTTCTTTTG

CATCATATACACAACCTCTATCTCGTTGGTAAATTTGAATTCTTTCAAAGATAGCTAGTT

CTTCCAATTGTCGTGTATTATCAACTAGATGATTTACAATGAAATCATGATGTTCTTTTG

GAGTTGCGCGTGCTTGATTTGGGTTAATAGCGTACAGTTCTTCGTATCGGATAAGGGTGC

TCAGATAGGACAGCTTAGGCTTTGTCGCAATCAAGGCTAATTGTATTTCATATCCCCTAC

TTTTCAAGAGTTGTGCTGTTTTCTTTGGAACATCAATAGTTCGTAAAGTTCCCTCTATCA

AAAGATTGTATCCCAAATGACTCAATTCTCTTACTAAAGACTCTACCATTTTACCTGCAA

AATATTTGGTGTATTCAACACTGTCTTTGCCATATTCTTGCTGAAGTTCTAAATAGTGTG

GATGCTGAGAACGAAAACTATCTCCATCTATGATAACAATATTTCCTTGAAATTCTTTCT

GTTTAATACGATGAATTGTAGTCTTACCGGCACCACTTTGCCCTCCGAGCAAAATCGCTA

TAGGTTGCTTACTGGACTTTTTTCCTCTTGTCAGTGAGCGAATGTTCCTTGCTAAAGCCT

GTTTAAATTGACTATCGGTATAATCTTGGATTCCCATTAGGCTGCCATCCGTTTTTCAAA

TATCTCTAACATACGTTCAATTCCATCCAAATAGCCACTATATCTCTCTATTTCATCAAA

AGTTTCTACTAAATAGATATTCGTATGAATCAAGTCAGATAGATCATCACTCATTAAAAT

CCAAGGATTAGATTCATCATCAATGCTAATTCCCTGACTATCTTGATAACGATAGAGTCG

AGATAATAGATTAGCACCTCTTTCTTTCACAATTTCAATTTTTAAAGTCAATTCATAATC

TTCAACAGGATTGAGCATTTTATCTTCTCCTACAATATCGACATAAGATACATTAAACTT

CTGACAAATGATGTCTATTAATTCGGTAGAGACTGAACTCGTTCCATTTTCATAACGACT

CAGACTATTTCGTGAAATACCTACAATCCGTGCAAATTCGAGTTGTGTTAAGTCATGTGT

TTTACGCAAGGATTTTATGTTCTTTCCAATCATGGCAAACTCCTTTCATTTGATACCACC

ATTATAACATTTAGAAAAATTAAATGCACCAATTTTGGTGTGTTATTGACTATTTTTCCT

AAACCTCTCTAAATTCTTCTCCAATTGTTCCATATGAAGTTGATTCTTGGCTTCCTTTAA

TGTGTCGTCCAAAGTTTCTTCTCTATAAGTTGCTTCTGTTTTTAGTTGTTCTGATCCTTG

ATCTGGAGTGAAAATGATATCTAACAGTCGATCTATTTTTTTAAGATCTTGGATAGACAT

ATCAGATAGTCTATGAATTAGCTTCTTAAATACATCACTATTGTCTTGGTTTTTCTTAAA

AAGTTTTGTAAACATCTATAACCTCTCAAAAAAAGCAGCCTTTTAAGACTGCTTAGTGTA

ATTCCGAAATCGCATCATAAATGGACTGCAATTTCTTATTGTCTTTATTCTTTGTGTCAA

TCAAAGTATGTAGCTCTTCAATTTTCTTTTGACTACTTTGAATATCGCTATTGTTATCTG

CAATAAGTCTTTTAAGATGTTGCTGATAACTTGTAGTGATATCTGTCTCTTTTCCTGTTC

TAACCTCTGTAACTTTAGGTTTAGAACTAGAATTTGATTCCGTCACAGGTGCTTGTTTAC

GCTTTTGAAAAGCTGCTTCATAATTTACCTCATTACCTCCTTGATGATTTCCAAATAAGG

CCGCAATTTTATCTGGATCTTCTTGATTTTCTGATTTGCTTTCTAATGGTTGATTAAAAT

TCCAATCTTTTGTCATTCACTCATTTCATCCTTTCTAAATTCAGAATCATCGAATTGTCT

TTCTTTGCCAAATGCATGGTCAAGAGCTTCTTCAAAACTCATGTGACTAATGCTTTGACG

CCTTTTGAACGTCGCAAACATCGCTGTCTCCAGTTGCTCTTTGTAAGCAGCTAGTTTTTC

TGTCTCTCTTGCTACCTTGCTTTCTTGCCTAGTGACCTTTTCTTCTAAGCGTTCAATAAT

GGTCATATACGATCCTCCTTCATTCTAATATTAGCTAAAACATCTTGATTTTGTTATCAC

AATTCTAAACATTGAGATGTTTGTCTTACTGGTTGATTCCTCAATGTCTCTTTTGCATTC

TCGATCATCAATTGTAAATCTGATTTCTTTTCTCGAAGAATATCGTTCCAGTCTCGTTTT

TCTTTCCCAGATTCATTATCAGGTAAATCCAGAAAAACAGGAAATCCTGATCTAGATAAC

TTATCAGAAAAATCTTTACCTGCATCATCACAATCTACTGCCAGTGTCAATAAATCAGGA

TGATTATCAAAATAGCTGGTGGTATCACGAATTGTATTGATCAAAGGCAATAACTTTGAA

GGTATTACTGTATCTAAAAATTCCAACTTCTGATTTTCTTCAGCTATCAGTCGTAAAGTT

TGATAAGCAACAACAGACCTTTTTAATCCCTCCATAGATACCAAACGAACATCAAATAGA

TTTTGTTGATGAAGTTCGTAATAGCTCATCAAGTCGATAAACGATTCACAAAAGACCAGT

CTATTTGGTTTACCAATGTCAAAGGATATTCCAACATGTCCGTGGCTTCCTTTTAGAATC

GTTTTTAACCTCTCTCTAGGAAGAGAGTGATTCTTATAAATCCCTTGTAAGCTTGCTGCC

TGCAGTTTGTGTCGATGATCAAAGCTTTTAAAAACAATAACAGGTTCAACAGTTTCATTT

GTTTTCCAACTGGCTTGTGACATCAAACCTTGTTGAATCATCTTTTGTATGATTTCTTCT

GAAATTCCTCTACATTCTGTTAAATAATATCTGGCCAGACTACAGTTAGAATCTTCTATT

CTCTTTAAAGGGTAATAAAATGGTCTCTCTCTTTTTTCTTGAACAGCTTCTTTTTGAAAA

GGTTCTTCAGAAAGAAAGGCTAGAGCTTCTTTAAAGGAAATTCCCTTAACAAGTCGAACA

AAATCAATGACATCACCTTGAATATCTCTTGAAAACCATTTAAAAGTATTGGTAGTTGAA

AAAATCCGAAATGAATCGTGTTCAGGATGTTCATAGACACTGCTCGAAACTTGTTTAAAG

GAGATCCCCAAACGATTGGCTACATCAAGAATTGAAATTTTCTTACATTCTTCTATTTCC

ATGCAACATCATCATTTTGTCGTAGTATTTGGCAGTGATGGTATTGATGATGATTCTTCT

AAAGTTTTAGGAGTTACCTTATCTAAATCATCTAACCCAGATTTCCAAACCTGTACTTGA

TTGACCAATAACTTACCTGGTAGTTTCGAATAAGACAACTTAACCGTTCTGGTTTCTGTC

TGATTAGTCTTTGATTGGTTGGCATTCTTTAAATCAGATACATAGGTTACATTATAAGAG

ACCATGGCAATAGCTTGATTCGTAGTCTGATTGACAAAGATATCAGCTTTTTCAAAATGA

TAATCCAAAATATAATCCTTATACACTTGGTTCATGGCATCATTTTGACTTGACAATTCT

TGAGAATAAGCCGATTCAGTCATATAAGGTTGAATACGTGTATTATTTTCCCCTAGTTTT

TCTTTCGTATAGTACTGCGTCAAAAATTCTTTTACAGTATCCGATGACAAAATGCTGGCT

TTATCTTCAGCTTGTTTGTCCTCTACAAGTTTAGTTGCAGCCAATTCGATTTCTTTGCGA

CTTTGTTTAGCAGTAGAATGTTGACCAATAGTATATCCCACCATGAGAATAAAGCTAGTT

GCGGCTACTGCTCCAACACTAATTAAGGCTTTAGTTTTGACTTTATTTAACATCTTAGAC

CTTCTTTCTATAAAAACTAGGTCAAGAATAACAAAAAGAACTTGTAAGTTTTATCACAAG

AAAAAGTATAGTCACTAAAACTCAATTACAAGAGAAAATTAAGAAAATCCTCCGACAATA

AAACGCATAGTATCAAGGTTTTTGTGATTCTTGATATTATGCGTGTTCTGCTTTTCGAGA

CTTTTTGTACAGACTCATAAAACCTAATCACAATTAATTATATGAGGTTTCAAAAAGCCT

TTTATACATACCTTCTTTACCACATAACTCATCATGTTTTCCGCTATCTATGATTTTACC

GTTATCCATAACAATAATATTATCGTATTCCTGAGCGTTAGATAGTCTATGAGTTATAAA

AATTTGGGTAATTCCTTTATGTTTTAAATTATCCATTATTTTCTTCTCTGTTATAGTGTC

CAATTGACCAGTACCTTCATCAAAAATTATAATCTCTGGATTTTGCAAAATGGCTCGAGC

TATTGCTAAGCGTTGCCTTTGCCCACCTGATAAATTGTTACCATTATCCCCAATACTAGT

ATAGTATCCCATAGGCATATTATTAATCTCTATATCTAATTCAGCTAAACTTGCTGCAGA

AATGACATCCTCCATAGAATGGCTATGAGTTAAATCTATATTGGAAATCACAGTGTCGTT

AAACATTACATCATTTTGTAAAACAACTCCGAAACTCTTCCTATAACTATTTAAATCAAA

TAAATTAATATCATTACCATTATATAAAATCGTATTTTTCTCTACATCATACAGCCGTAG

CAATAATTTAATAAGTGTAGTTTTTCCGCAACCTGTCCTCCCCACTATAGCAATACGTTC

ACCTTTCTTAATAGTTAATGATATATCATAAATAACTTTACAGGCACTACTATAAGCAAA

ACTCAAATTTCTGAAAGTCATCATTTCAAATGGGGGTAGTTTAGAAATAGAAAATTGATT

TTTTTCTTCTGGAGTATAAATGATATCAAACACTCTATCTAAAATTGTAAAACAAAATTG

AAATTGAAAACAGTTTTGTACGATTAGAGTAATAGGTGAAAGAAGCAAAGATACCAATGA

TAAAAATGACATTAAACTCCCCAGAGACATCATATGCTGCTGTACTTCTAGTCCTCCCAA

AATTAAAATTAGTAAGTTTGGTACGAGTCTATAAATAGAAATAAAAGTCTGAAAAATATT

TAGATAATTTTCACGCTCTACATCAAATTTAAGCTCATTACTAAACATTTCTTTCCACTT

AGTATATGAAACATCTCCTTTTGCTGAGACTTTCAAAAATAATAAATTAGAAAAAATTTC

CATCAAATAACCTTGAGTCAATTCTTTAGAACTGATAAAGTTTGAGTTCTTTTCTTCCAA

TTTTTTAACAAAAAATATAAAACAATAAACAAAAATATAGCAAAACATGTGATTATAAGT

GTGAAATATAATGAAATCATTGCCATAGCTATAAAAGCGATAATAATTGTAGATAGATCA

ATTAACAAACTTGATATTCCACTTGAAATTATTTTCTCTAAAGAATCTAAATTATTGATT

CGAGTTACAATTTCACCTGAAGATCTACTATCAAAAAATTTCATAGGTAGAGTGAAAATT

TTGCCAACAATTTTATCTTTTAATGTAAATATGTATCTCTTTTCAACTGTAATAATTAAT

TTAGTTCGTATAAAGCTAAATAATGCATAAAATGTTATTAAAGAAAAGATTGAAAAAAGC

ATCCCTAAACGATACATTTCTCCTTTGCTAATTACATTATCAATAATAAACCTTGTTAGC

AAAGGTAATAATAAGGTGAGACTCTGTACCAAAATTGATATTAAAGAAATATAACATAGC

CTTTTAATATCAATTTTACCTATCTTCAATATTTTAAAAAATTACTATGCTTCTTTTTTT

AGTGAATTTCCTATTTGGTGAGAAGAAAATTACAATACCAGATATATTATTCGCAAAGTT

TGATATATCTTCTGTAATTCTACCTCGTTCTGGATCAATGAATACTACACTATGCTCATT

ATGTTTTTCCACCACAATATAATGAGCATTTTGAGTTAAAGCTATATAAGGTTCTACAAA

TTTGTTCCCCAAAAATTTTTTAATATTTAAGATCTCAGTCGCCTTTGAATCCAATCCAAA

ATAAGAAGCTATATCTTTAATCTCTCTAATGGATAATCCATCCCTTGAATTCTCAAAATC

ACAGCATAATTCATATAAGTTTGCCTCATGCCCATAATAATCTAAAATCATTGCCATGCA

TGCTAAACCACAACTGGTCTCAGATAATTGTTGTATGAATTTTACCTTTTTCAATTTGAA

TCACCAAATTTCTAGATTAATTACAGAAGGAATTGATAAATTATTCAAAGTTAGTAAGCC

TAATCCTATTCCAGCTATCCCACTCATTAGACTTGGAGAATTCTTATACGGAAGACCGCA

TTTCCAATTTTCTGATTCTAAATCATCAAGAATCGTATTAAGATAGGAGTAAACTACATT

GTATAAATGCTCAGAGGACATTTTCTTAGCAACATAAAATAGAATGTTTAGATTTCCTAA

GTCTCCATGACATAGACAGTGGCTTTTTCCAAAACCATATTTTATTGTTGTATCAATTGC

TACATCCATATCAGATTTCATTGTTCTTCGAAACTCTACATTCAATGTATTATTTTTATA

CAGTTCTAATCTACTTATAAGTATACCTGGAGCACCATGGCACCACGAAACAGGGATATT

TTCAACAATATCCTTATATTTGATACCACTTTCGGTTTCTCTATAATCCGACCAATTAGA

TTTTTCAGTATCAAACATAGAATTTTCAAAAATTATACCTTGTTTAATAATAGTTTTAAG

CTTTTTATCTTCTGGGAAATATCTACTAAGTTTTGACAGTGCCCACACAATTCCTGAGAC

TCCGTGTGCAAAACCCGCTAAAGGATTGGAAGCCATCGTTCCTTTCCATGCTCTTCCATC

TTGCCCTTCAACAGTAATTTTTGTAGAATTTTTTTCTAAATGACTAAATAAATCATGCGC

TAATATCAAGAGTTCTTCGTCTCTAGTAACTTCAAATACATTTAGCAAATGTATAATCGC

ACCAGCAGAACCTACAAGAACATCAAAATTTTCATCATATCTATACCCTTTTTTTAAATC

ATCAATAATATCATAGATATAAACATCTAGTTCATCAGAATTAAAGTGTTCAGGCATCCT

TGTCTTCAGTAACAATAATACATATATAATAGAGGTCTCTCCAGAAAAACCTCCAAACAG

TACCTGAGAGTGAGTAGCAAATTGCTTTCTTGAATCTAAATAATACTTGAGAGATCGGTA

ACATTTTTTCAAGTATATGAGATAATCTTTTTTCTTGGTCTCCAAATATAAGAATAAATA

GAATAACATAATACCTGATATACCATTATACAGATCGCATCCGATTGGAACCATATTCCA

ATCATTCGTTTTCTCTCCCAGTATGTTCATGTCTACCCAGCATATATCTCCTGAAGTACC

ACTATATGAAATTTTAGATAAATAGTCAGCAATTTTTTCTGTTAGAGGTACAATTCTACT

TTTAGTTTCTTGTATTTCTTTCTGATTTAAATTACGTATCACTTTTACAGATGGTTTTAG

AGGTACAAAAGAATCCTGAACTAAATTATAAGAAGGATCGTAATTCAAAGTTGTTTCAAT

CAAATTTAATTGAAATTCCATATCCTTCTGACAAAAGTCTTTTATTTGTTGAATTGCTAA

TTCCATTCCTGAAATATTAAAGAAATTTTGATATTTCGTTAGTTCAGAACATAAGAATCT

ATCAGATATTTTTGTCTGAAATAATGGAATATCACCCAATAATAATGATTCGAATTCTGA

ACTAGCTATTCTTTGTACTTTTTTATCCTCAAAAACTATTTTCCAAACTTTTGAAAATAA

CATTTCCCTATCTAGACCACTCCTTAAAAAACTAGGATGATAAGATGTTTGTATTAAATT

AGAATAAAATTGTGTGGGCCTCATTATTTGTCTAGAATACGCATTATTAAATTTTTCTAT

CTCAACCAGAAACTCTTCTATTGATGAAGGATCTTTTGAAATAATTTCATAAGCTTCTTT

AAAGCCAGCAGAAACGTAATTTTTATATGAATTCAAATCAACATCCTTGCCCGATTGAAT

AAAAGGCTGATTATCTTTACTTTGCAATGCGGATTGTTCATAACCAATTTTCATTTCATC

CGTCATACTATGCATGATACTTGCACGTTCAATCGGAATCATCTCTCCTGCGCTTGAACT

TAGACCGCTAATATCCACCCCAGAGTTCCCATCTTTACCCCAAACTATATTAGGTAAAAT

ACCTACTCTTTTCACAGATTTTTCTAATAAAGAGAATATTAGTTTATCTGCCGTTTCGTA

TTCCTTTCTGTATTCCGTTGTATTATGAAATAATGTTTCAATATCGACAAGAACTGGTCT

ATTACCCTTAGCTATGATATTTTCAAAATGAATATCGTTTCCCCTCATAGCAAATAATAA

GAATAACAAGTATCCTAGATGTGTATAAAACTGAATAAAATCAGATTCATATGTGCATGG

TAAGTGTTCTATGTACTCTACCCATCCATAATTTCCACGATTCAAAATATTTGTTACATA

TAGATTTTTATTGAATTTCAGGTTGTACCAGCTAAGCAAGTTTTGAAACTTCACATCCAA

TATTAAATCTCTTGGTTTATATACTAACTTATTTGTTCCAAACCAAAGGAGATATACTGA

TTTTCCATGATTATGAAAATCTGACACACCAGATTCAATCTTTGTTAACTCCCCATTGAT

AGAAAAATGTTCCTGTAATAATGCTTTATCTTCTTTTAGGTTTGTTAAAAATTCTACATT

ATTAGTAACCCAATTTTGAATATTTTCAATAATTAATCGAAACATAACAGGATATTCTTT

TAATATATCCCAAAAATGGTCTGATAAGTATATTTCTGAAAAATATTTAAATCTTTTTTC

TCCAGTTTCACCTTTTAACATATTTTGTTCTTTCAGAACTTGTAATTCTAATATCAATGT

TCTGTAAGAAATATTCAAAATTTGTTGGAATAACGCAATTAATAAACTATTCAAGACGTC

TCTAGTGACTTTCAAATTCTGACTACTCAAATTATCTTCAAGTTTATAGGCGGCAAATTT

TAAAAATGGTATGACAAAATTACTGAAAATAGGTTTTCGTTTTCCCTCACTGTCAACTAT

ATAAAACTCTGGTAATGTATAATCATTTCTCTTAAAAAAGAACTCTATAAATTCGTCCAT

ATGTGAAAATGAATCTATATCTTTCACATCATATGTATGTTCTTCCAAAAAAATATCTAA

ATCATGTTTAGAAAAATCATTTTTTTCTTGTAAATATTTCCAAAATTCATTAGAACCTTC

TCCAATAAAATTATCCCAATACTCTTTTCTTTTTCTTGATTTCAAACCATCCCCTAAATC

ATGACTAGAAATTTTTCTTTCAGAGAAATAAGAAGCATCTTTAAAATCTGTTATGTTTAA

TTTTCGATTCATTTTCAACTCCTAAATATAAAATAACAGGTGACATCCTACTTTTAGGTG

TCACCTATCCCAAAGATTCAATAGAATTAATCTATTAACAACAAATGAATGTTAAGAAAG

GACACTCTGTGGTGATAGTACAACCACGACCACAAGAATGCTCTCCTGAATAACATGCGC

AATAAGATCTGGCTGTTCCGCCAGCCATAGCTTGATCCATCTCGCTATCGCTAAGTTCAC

GCAAGATATCCCCAGCTGGATTACCTTCTGTACTTAGTGATTCGTAAATAGGTTTTTTCC

AATCAGACATAATTGTTGAATCCCTTTCTAGTTACTGATTTCTCTTATTAAATATAGTGA

CACCTCAAATCTTTGGGATTAAAAATTCAAAAGAGATACCAGTAGCAAATATTTAAATCC

GCTGTAATTATAACTCTGAAAGTTATGATTACAGTCATTTATAAGCAAAAATAATGTTCA

TCATGCGTTTATCTGCCAACAAATAAAGGAGACATGCCACCAAGGTAACCAACCAAATGG

CATATACAGAAACTGTTAAAGCCGTAAATTGCTCCATGATGATTAGTAGTAAATTTGCCA

GATAATACCGCCCAACAACCGTTGTTAAAGCAAACAAACCTGCAAATGACAATAAAAAGG

CAAGTGGTAGGGCAAGGATAAGTTCCTTCCGATATTTTCCTAAGAAGAGGACAATAATAC

CAGCAGACAGTGTATAAGCAAAAGCAATTGCTCCATAAACAACTCCCCAAGATAACATTG

CAAGACTAGAGAAAGCAAAATCACCATCCATTAACTCTAGGACCAGCATAGCCAAAAAAG

TGAATTCACTGACTAGTAGATTAATCAACAAAGCAAGCATAAATTTCGCTTGGAGTAATT

GTTTCAAATCGACAGGAATCAGTCTGAGATTTGAAATCGTTTTAGACACTTGTTCTATTC

TTATCAAATCCACTAAAATCATGCTCGCAAAAGGGAGGAGAAAATAGTAGTTCAAAAAGA

AGAAACCATTTTCTACATACCAGCCATTCACACTACCGTACGAATGCCCTTGATAAATCT

GATGACCAATCATAAACTCCAACAAAAAGAGCAGGATGGCTACAAAGGGAATCATATACA

GTATCTTTCGCCTTCTTAGTTTAGTAAATTCTAGCATCAACATTTATTCATCCACCTTTC

TTAACCAAGATTTAATGGACAAGAAAGACAAAACAAGTAAAGATAAACCTAGTACAACAA

TCGATAAGATAGCTGGCTGTTGAAGCACCACTCCTTCTCTGTTTCCTTCCCAGATTAAAC

TGTTAGCACTTGTTACTGGACTGATATTTGGACTCGTCCTTGCTAAAAAAAATGACACTA

AAACATACATGACACCGACCAAATTCCCAACTATCTCCTTGCCTCTGACTTTTATCATGA

CCAAAGCAACTAAAGGAAATACTCCCATGGGAATTAAGATACCACTACTCATACAAAGAT

AGAAGAATCTTAATAATAAAGACAAACTGACTGTCGAAGAAAAGCGGGATAGTAGTGCGA

ATAAGGAAACCGAAATATAGGTGAATAACATGATACACAGAGCGTTGATTTCTATATACA

AAATCTTAGCCCAGAATGTCTTCATTAGATTTGCTGAAGCGAGTTTCAAGTTGTTCATGA

TGCGATAGCGATATTCTAAACCAAATGCCTGTATTGTATAGGAAACACAATACAAAGGGA

GAATCAAAAAGTTTAAATAAGACAAAGAGAATTTATAGACAAAGGGAAAGGAGTCAAACA

AGCCTCTCGCATCATTTACATAATAACAGATGAGAGCTAGGTAGAGGGAAACAACACCAT

ATACGACAAAATTCAGTTTTTGATGCCTTTGTTTGAGAAATTCAATCTTCACTAAATCAA

GCAATTCCCTCACCTCCTGTTATCCGCTTAAAGTAATCCTCTAAACTTTCATTGTTTTTA

TAAGAATATTCTTTTAGAACTATTCCGTTGTCTTTTAAGGAGTTAGATACGACTGCAATG

TCAAAAGTAGGTGATTCTCCCTGAAGTTCAAGTTCTATATCCGAAAGAATACTAATCTGT

TCTTGCAGATTTATCCTGCAAAGCACTTCCTTAGCTTGAGGAGCATCCGAAACAATTAGG

TGGATTTTCCTTTTATCTGCACCATCTATTAATTCCTTCATAGTCTTTTCAGCCATAAGG

TGGGCATCATGCAGTATCCCAACTCTATCTACAAGCTTTTCAAGTTCTGAAATAATGTGG

CTGGATATTAAAATCGAAATGCCATGATTGGTACTGAGTTCCTTTAGATAACGGCGCATT

TCAAGGATACCAATAGGATCTAAACCATTTGTTGGTTCATCTAATATCAGAATTTTCGGC

TGGTGCATAATAGCGTTAGCAATTGCTAACCTTTGCTTCATTCCCAGAGAATACTGCTTG

AATAGTTTTTTATTTTCCCCATCTAATCCTACTACTTGTAAAGCTTGCTCCACCTGTTTT

AGCGAAATCCCTCTCAATCTTGCAAAAATAGAGAGATTTTCAGGTCCCGTTAAATTAGGA

TAAAAAGAAGCACTGTCCAATACCACACCGATTTGAGATAGCAAATCCTTAGAAAATTCG

CCTAACAGTTTTTCAGATAGAATATTCACTTCACCACTATCCATAGCTGTCAAGCCTAAA

ATAGCCTTGATAAAGGTTGTCTTTCCAGTACCATTTCTTCCTAGTAAGCCATAGATTTCT

CCCTGTCTTAATGTAAAATCTATCCTATCTAAGACAATTTTCCCATCATAGGATTTTGAT

AAATTAAATGTCTGTATTACTGGATTTCTCATTTTTCTCCTCCGAACATTCGTACACCCA

CATAAGAGATATAATAAAGTAGTTCCTGTATAACTAGTTAGTTATATGAGAGGGTAATAG

GGTATATCAAACTTACAAGCTCCCTTTAAGCCCTTCAATCTGTGGCATGATCCATTGTAT

AAATGTTACTTCACAAAAAGTTTAACAAATCAATCCGCCAGTCCCAGATCAACTTCATTA

GTTGTTACAGCTTGTTGTATAATTCTCGATTCCCAGGTATATTGTGATTGATTTACAGGT

TCCTTGAAAAAGAAACCGCTTCCAAACAATCCTAAAAAGGACAACATAAAAATGATTTTA

CGAAACTTTGCTAAAATACTCATATTAACCACCTTTTTCTTATCTTTACACTTGTAAGTG

TACTACTTTAAATATTATTTGTCAATAAAAATAATTCTTTTTTTCATTTTTTTTGTTTTA

TTTTTTATGACTATTTTTGTATTAGAAAGTTGAAAAATAGAGATTAAAAGTATATAATTA

ATCTTAAATTTATTTAAAGAGGGTTAGAGATGACCATATTATCTGATAAACTAAAAGCCA

AGAGAAAAGAAAAAGGATTCTCACAAAAAACACTTTCCGAAGGAATTTGTGAGCAAAGCC

AAATAAGCAAAATAGAACGTGGCAACTATATGCCTGCCGCTGATTTACTCTATAAACTTG

CAAATCGGCTTCAAGTTCCCTTAGATTATTTTTTCGATGAACAGATTGAAATGACTTCCA

ATATAACACCGTTTAAGAAATTAGCAGAAAAATTACTTGAAGATCGTAATTACGAGGATT

TAGAATACCTCTTAAACTTAGAAAAAGAAAAGTCTCAGTATCTATCTACTGAGGATGAAT

TTTATCTTCTATGGATTCAATCGATTATCCTTTTCTACTTACATAGTTCCAAAGATGAAG

CTATCGCCAGTCTAGAAAACGCTTTGCCAAAGCTTTCGGTCAGCTCATCTGTGTATCTTA

AACTCCTCAATACTTTATCTAATTTTTATTTTTCTGTAGGGCGTGATGCAGAATACGAAG

AAAATAACTCACTTCTTATCAGTTTGTATCAGGAAAAAGATTTAAACCACCAAGAGTATT

TATTTGGTTATATTCGAGTTAAACATAATTTCGCCTATTATTTACACTCTAAAGGGAAGG

AATTAGAAGCCGTCCAAGAAGCCTTAGAAACCATTGACTTTTGTAAACAAAAAGAGACCA

GCTATCAGCTGGCTCCTCTTTTAACTATCGTTGCGAATGCCGGAAAAGATTTTTTAAAGC

ACGATGAAATTCTTGACTATTATTTACAGGCTCGTGATATCTGTAAAATATACGAACACA

AGCTCATGATGGCTAAAATTGACCATTTTTTGAAAGATAAGGATAGGTGACTTCGATTGT

AATAAGTTATAATTACTAATTAATTAGAAACATAAAAGTGGAGAGAAACTAATGTCGTTT

TCTCCACTTTTGTTAATATCCCCTCTTCCACTTGTATCCTGCCCAGCATGCTAAAGCAAG

TAAAACTAAATTAAAGATGATAAAGAAAGTCGTAAAACCAAATAATCCATTTTGAGCTAA

ATCTGCCGTGTAAAGTTCAGTTTCTGTAAAGAGAAGATAAATTCCATAGACCTCCACGAA

AATAAGGAAACTGACAAAACCAATCAAGAAAAGTCCACTGGCAATAAGAAAGAGTCTCCA

TAAAAGAATGAGAATTCCTCCTACTGCTAAAAAAATTACTGGGATAAGGAGTAAATCATT

CATGACTTTCTCCTTTCTAGTCAATCAATTGACGATAATGGGTGGCAAGAGAGGAATCAA

ACTCTTCTAGTTTTTGAACCAATTCCAGATAGTCCTTTTTTTCATATTCTTCAAAGTCCA

CACCTCGATGATTCTGAAGAGCGATTTCCAATTGGCTAGTAAGTTCTCGTTTGGAAAAGC

TGTAATCCCCTGTCACTTCCTCATAATGTTCCTTTAACTCCTTGTAGGCTTGGTATTCTT

CAGGTGTTTGAAATCCCTGTACCAAAGCCTCTTCTAATTGATAATAGGTATCTTCCATCA

TAATCGTTACCTCGTTTCTTTCTATCTTTATTTTACCGCGCTTTTCTTTTCTTGTCCGCT

ATTTGTATCTATTTTTAATCTAATTCAAAAGAAAGTTGTTCTTGCTTTTCTTTTCCTTTT

TCGTGGAATTGTCTTAAGGCCTGATCAATAGTATCTAAATCCGTTTCTGTTTCAATCAAT

GGCGCGAGTACATCGTATTCGGCCTTTTTAGTTTGATAATCCTCTTCCTTTGGAAAATTT

TTCTCAATTTCAACTTTAGCAGTATTCCATTTATCCTTTAATTCATCCAATAAGTTCTGA

GTTTTCACTTGGTCCTCTTTAATGTGATCAATCGTATGCTGAAGCCTTTGAATTGTCCCC

AAAGGAGAATATAAATCTAAACTGACAGAATACTGATTCTCTCCTACAATTTTAACAGAG

AAGGTTTCAGGAAGAGGTTGATTTGTTGGAAGACTAAGCATTTTAATGTCAAATCCTCGA

TAGCTTGCTAGGGTACGAAATTCTTTGCTGTCAGCTTGATTATGACGAATAAGACGGTGT

AGGGATTCCCCTGCTTCAGCTCGTTGATCAAAAGATTGCTTACCTACCCTCATAGAAAAT

GCCTGGTCTTTCGATATTTCAGACTGTTGAATATCGCCTTCATACTTGCTTAATCGTTTC

TCAAGAATGGGCATATTTTCTTCACAGTAAGAGATTGTATGACGATAGTGATCCTTGCTG

CGTTGAAAGGCGCGTCTTTGATTTTCTAATAGAGTTAGATCATTCTCTAGTTCCATCTTA

TATTTGAGATAAGGATTACCTGTTGCTAGTGCCTTAAAATCAGAAGCTGTCATAGTCTGT

TCATCAATGTCTTCTGCAGCACGAATCGGCTCCTTAGAAGTCATAATCTGCTTAATATAA

CGGAGTTTGTTCTCCTGAGTTGCCCATAGATAATTATCAAATGAACCTTTGGTAATGTAG

TGGTAAATATCCACTTCCTTATTTTCATTTCCCTGTCGGATAATACGTCCATTGCGTTGT

AGGCTAAGTACCCAGCGCCTTACCATATTACTACGTCAGGTTTCCAAATACTCGCCCCCG

AACCGTACGTACACCTTTCAATGTATACGGCTCTCCGTTTATAAACTCAATCTAACTTGC

CATGATGTAATTTCTTATGGCAATCTTTACACAGTGCTATTGTTTTCCGTTGTCTAGAAA

TCATCAAACGTTCCCAAAAAGTCTTTCCCTTTAAATCCTTTAGTTTTCTCACATGATGGA

TTTCTAGGGAACTGTCGGTCGCTTGACAATACTCACAACGGCTTGCTTTTAATCTATCAA

TTAAATTTGTTCGACTAAAATATTTCGCCGTATTTGGCAAATTATCATTTTCAAGGAAAG

ATTTCTTTTGACGTTTAAATCCTCCGTTATATAAATAACGGGTCATTGTATCCCCTTTAC

GTCCAATATACTGTACACTAAATTGACCATCCTTTTTATATTTACGGATGATGTGGGATT

TAGTCGTACGATATTTAGTCGCATAAGTTTTATACATGCTATATTCCATGATGTACTTAA

AGCGATGAAGGATAGAGCTGTTATTAGCGATACAATAATAGTTATAAAAACCTCTTATTT

CTGCATTGTAGCGTTCTAAAATCTCTAAATCATCGCAATCTTTCATGAAATAACGGGCCG

TTGGTTTCCAAACTTCGTAGCCTCTATGATAAGTCATTTTCATAGCTCCGTAAGACAGTA

ATCTGTCTCGAATCGTTTCAATAGAAACCTCTAGGACTAATCTACCTGTATAATTTCTTA

CCAATCGTCCTGCCGAATCACGCTTAGCTAAATTCGATTGGCGAATATACAGATGATATC

CCAGAAACTTAGCTTTATCTCTCGCATTGGTAATCAAGGTTTTCTCCACAGAGAGTTCCA

ATTTGAGAACCGCTTCCAAATAGTCCTTAATATCTGCTTTAATGCGATGAGCGTCCTCTT

TACTGCCAATCACACCGCAGATAAAATCATCTGCGTATCGGGTATAGGTTAGACGTTTAA

AACTACTATCCATAGGATCACTATGAGGAATTAACACTCTTTCCTTTTCCAATTGACGAA

TACGCTGGATAACTTCTTGTCGTTGATTTTCCGTTGAAACACATTCTAATGCTCTTCTTG

CTTTTCCCAACGCTATCTCATTTTGGCGATATTCTGGGGTACGTTTCCGGTATTTCCCTT

GGCAGAAGTTCTTCACATAATCCGTCATATATTTATCAAACTTATCCAAATAGATATTGG

CTAGAATGGGACTGATTATCCCTCCTTGTGGAGTTCCTGAGTAGGTTTTGTAGAATTTCC

AATCTTCCACGTATCCCGCATTGAGGAATTTACGAATCAAGCGTAAGAACCGCTCATCAG

TAATTCTTTCTCGGAGAATGTGAATCATCACATCATGATTGATATTATCAAAGAATGACT

TGATATCTCCTTCAATAAACCATTTTGTTCCAGTGTAGGTCTTCTGAATCTGAGTAAGTG

CTGTATGGCAACTTTTATTCGGACGAAAACCATGGGAAGAGGGTTCAAATTGTCCCTCGT

AGATAGCTTCCAAAATCATTTTAATAACCTGTTGTAAGAGCTTATCATCAAATGATGGAA

TACCTAGTGGACGTAGCTTCCCATTCTTTTTAGGAATATAGGTTCGTCTTGATGGGTGTG

GCTGATAGGATTCATCTTTCAGGGAATCAATCAATTGGTCAATTCTAGCTATACTCATAC

CATCAATCGTCAACTCGTCTACTCCAGGTGTCAAGTGTCCTGGGTTAGCGTAAATCGTTT

GATAGGCAACTAAGTACATTTCTTTGTTATAAAGCAAACGATACAGTCGCTCAAATTGAT

AGTTTTTATCTTTACTGTGTTTTGTTAGATTGTTTAACACATTTTGAGGATTTCTCATGG

TGTCTCACACACCTTCCTTTCCATAATGTTAATGTTATAAACTGTTCCCCTTCGCCATGT

ACGTGCCATTAACACGCTCGGACTACTATGGGAACTCCGTTACCTTATCAAATATTCATA

AACCTTATGTTTAATAGCTTTTACAAGCGTTTTGACTTAGGTAATCTCCGTTTAGCTGGT

ATAGTAACATAGCTTGATAATTTCATCGGATAGGACGTTCATCTGTTTTCATTTACTATG

AATACGCTATGGAATCCCTTTGCTTCACTATCTTCAACAGTGATAATGAAGTTCCATAGT

ATGAGGATTTTAGTTATCTTACCTCACAGTCCCGACACAGACCGTTTAGACTATCCTTCA

ATCAGTTTAGATTTTATCCTTATAATTATCTCAATAACATCTGTTACATTATCATCTCCT

CATTCAGTCGTGATGTGATAACTCATCAACTTATGAGTTTCCCAACGTGCTTTGTTCCCT

GATGCTTGGTTTCCCTCACAGGTCAGTTGGGTGATGATAGGCATTAAGGTCACGCCTACT

ACTTTGCCAAGAGTAGATTTACTATACCGCCTTTACGGACGCACGCTGAATGTCACTTGG

TCTCCACGGTACATCCAGATGATGAACTGCTTTCATCTTGCTCTGAACATTTAAACCTGT

TCCTCCTTTTTCAGTTGAGGCAAGGAGAATCCGCACCTCTCCTGCATTGACCTTTCGAGA

CAAACTATTCTTCTTTTCATCACTATTGGCATCATGTACAAAGGCAATTTCTTTACTAGG

GATTCCTCTATCAACTAATAAAGCCTTAATCTCAGAATAAACATCAAAGCCATTATCTTT

TTTCTTAGGTGTGCCAATATCTGAAAAAATCATCTGAGTAGCCTTATTTTCCATTCCCTC

ACGATAAATTCTTTCAACATTATCCACTACCTGAAGCAGTTTATGATTGTCTGCTAGACT

ATAACTAGAGTCCAATAAACGCATATCAATAGCTAGTTTTCGTGCCTCACCCGTAATTTT

TAACATGTTATCCTGGCTCGGATCAACTGTTCCACATTTGACCATATCTGACCTCATAAC

TAATTCTTCTAAATAGAGTTTCTGGTTTTCAGTTAACTCACTCTCAATAGGGATAATATG

AGCTTCTGGAACAGGTAAATCCAACATATCTTGTGTTTGAATGTCGGCTGTTTCTTTATA

GATTTTCATCAACTCAGGTAGATTGACAAACTTTTTAAATCGTTTCTTAGGTTGGTACTT

ATCCCCTGTAGGAGCTAATTCCATAGAGTTTTGAATTTCTCCAAAAGCACCTACCCAAGA

GTCAAAATAATCAACTTGATAGCGTTTTAAGATATCTGGTTGAATATAGTTCATCATAGT

ATACAACTCACTAATAGAATTGGAAACAGGTGTTCCTGTCGCAAAGACAATATTTTTAAA

ATCATGTTCTTCCTGAATCTGTCGAACCTTCATCTCCATATCCACGTTCTTCTTAGAGGT

TGTATTGGTAATACCTGCTACATTCCCAAGTCCAGTAATTGGACGAATATTTTTAAAGTG

ATGTGCTTCATCCACAAAGAGAAAATCAATTCCTAAGTTCTCAAAATCAATAAAACTATC

ACGATTAAAGCGTTGCAGTTCTTCCAATTGTTTCTCTAGACCACTTATTGATTGTTCTGC

TTCTTTAACGGTATACTTATTTTCAGAATGTGTTTTAATCTCTCGTAGTTCATTGAGTTT

ATCCTCTATATAGTTCATCTGTCTTTCCTTACTGACAGGGATTTTTTCAAATTGAGAATC

CCCAATGACAATAGCATCGTAATCTCCTGTAATAATACGTGATACAAATTGTTTTCTTCT

CGCCTTCACAAAATCTTTCTTAGTGGTCACAAAGACTTTTTTAGTAGGGAAAAATTTCAT

GATTTCTTGGCCAAATTGAGCAGACAAACTAGAGGGCACCACATATAATGGCTTATGAAC

CATCCCCAACTCCTTTAATTTAAACCCAGCACCAAGCATGGTCAAGGTCTTTCCTGAACC

TACCTCATGAGCTAATAGGGCTCTTTTTTCTTCTACGATTCTTTGAATGGCATTTTCTTG

GTGAGGACGAAGACTGATGTTTTGTGCTAAGCCATCAATGACTAGATGGCTACCGTCATA

CTCTCGACTAACCGTTCGGTTATAAAGACGATTGTAACTTTCTTCAATGACTTGTTGGAC

TTCTGGATACCGTGAGACAAAGTCTTGAAAGAGCTCTTGTAAATGCTGTTCTTTTGCTCT

TAGAACAGAGGTTTTTTCCAAATCTGTGATGGTCTTTTTCTTTTCTCCTTCCGTAACAGT

CATAGTAATCGTCGGTTGGTTCGAATTAAGTAAATTCTCAAAAATCTTTCTTCCTGTATC

ATAACGTGAGCCACTGACTCCAAGACTACTATCTTTGGCACTTGGATAGCGATAAGCAAA

TGATGTCCTTAAATGAACCTGCCCATCGACAGGATTCGCTTCAATGACCTGTTCAACATC

AGGCGAAGATAATTCAAATTCACGATTGGTAAAACATTCAAAGGCAAATTTACCATAAAC

GGATTGAGGAATCCAACGTGACCCTATTTTAAACTCAATATCTGCCAGATGAATCCTTGG

AGGGCGAACAGATTCTAACAAATCTAAAGCATGGTTCCAATCATATTCTTGGTTGTTTTC

CTCCACTAATAGTTGAACTACTTCTATCTTGTTGAGAATGTCTCCTGATAAAAACTGGTT

CTTAGAAAGATATTTTCTTTCCCCTCTTAAATAGCTTTCTGGATCTATTAAAATCTGGTC

TCCTAACTCATCTAAAATAGCAGCTTGGCTATGTTCGGGATAAATGGATACCATATAGTC

TAAATCAACCCCTCTACCATCCGATAAACTAGAGTTTAAGGCATCTAGAGCCGTTGAAAC

TCTTGCAATCACTCTCTCTGGCCTAACCAATGCTTTCTCAAAGGCTAAAGATTTTTTATA

TTTTACTTTCTGATCTTTAGAATCAATGTATTCATCTTCTAAACTTGCCAGTAAAGAATA

CTTATCGTCACTATCAAATAAGTTCCGATTGACTGAGGCATTCAAGTATCCAAATTGACT

TACAAAACGGTCATAGTTATGATTGAGTTTACTAAGTAACACCTGAAAATCTGTCCGACT

ATAATCTTGATGGCGTTGAATTTCAATTAAGGATTGATAGGTCTCTCTCAAATCAACCAT

GCCCTTAATGCGACTAATATCCTTATCCGATAAGGGACTTTCATAAAAGACAGTTTTTTT

GAACAATCCCTTATATTTCCCTCTTTTACTCGCTTCTTCTGACTTGTAAACATCTAGTGC

TTCCTCATCTGTCAAATGAAGTTGCACGAATCGGTCTATTTTATGTTCAGACAAAGAACT

GTCCCAAGCTTTAAAATCTCCCTTTTCATCTACATAATAACTAATTTCGTCTACTTTTGA

ACTTTTCCGAATGCCATGCGTATCTCGGTAATAAATTTGATTTCTCTCATATCCAAAAGA

ATAGAGCGCTAAGTCCTCACGTATACGACTTGGGAGCGAATTATCCACTTCTTCTTGGAT

AAAAACAGGTGCTTTCAAAAAATTGTCAATTTGTTTAGGTGCTTCCACATTCTCTAATGC

TTTCATTATATCAGTAGATAATGTTTCTGATACCCCCTTAACATTGAGAGTTCCTCCATT

AAAATTACGTACCTCATATTCACCCAAAACTTGTGTGTTGTATTTCCCATCAAAATAAGG

ATTGATCCAGACACGCTTATCCTCCTCAAAGGGAATAGAGCCACTAAAAACAAGTTCCTC

TTCATTAAGATTCTTTGCTTGATCCTTTTGAAAGAAGAGGATATCTGTGGTCACTCGGGT

ACCTGCAATGCTTTTAAAAGCCGTATCTGGCAACCGAACTCCCCCTAAAAAATGAGTGTT

GGTTTTAATCTCTTGTAAGACATTATCTGTCCGCTTATCCATTGTCCCGATAGATGAGAT

AATCGAAACTTGTCCTCCGTCTCTTACTAAATCAAGTGAGTGTTTGACAAAGTAATCATG

AATCATATAAGGTTTATCATAGTTTTTATCGGCAATGCGAAAATTTCCAAATGGAACATT

CGTTAAGACTAAATCAAAACTATTATTTTGATAGGGAACTTCTTCAAATCCTCGCACTTC

AATATGGGTATTGGGGTGGAGTTTTTTTGCGATTGCGCCTGTCACACTGTCTAATTCAAC

CCCATAGAGTTCTGATTTATCTCGTATACTTCTAGGCATCGCCGCAAAGAAGTTCCCAGT

CCCCATAGAAGGATCTAATATCCTTCCTCCCTCAAAACCATCATCCAGTAATTTTTGCCA

AATCTGGCGAATAATCATTGGGTCTGTATAATAGGCTGTGAGAGAACTTTGTTTCATGGT

CGAGTATTCTGATTTACTTACTAAGCTCTTAAGAGTTAAACGTTCTGTTTCATACTTTGG

ATTGAGTTCATCGAAAAATTCATTGGCAAGGCCACCCCATCCGACATACTTGGCTAGTAG

TTCTTGTTCTTCTGGATTCGCTTGTCGTCCCTCTTTTTCTAATCTTTTAACAAGTTCAAT

TGCGGCGATATTCGTTTCAATCTTTTCTCGATTTGTCTTAGGATAAAAGTCCTCTAAATC

ATCTGGAAAAACAAAATCTTGAACAGGGACATCCGTCTCTTCTATGCCTACAATAAGCGT

TTCTGTTTCCTTATCCTTTTCATTTTCTTTTTCTTCTTCCAGGTAGGAAAACAAATCTAT

TTCCTGACTTTCACTTGATGAATCAATCTCAATCTCTGAATCTTCTTTTTCAAGTTCTAC

AAGAGACAATACTTGTTCAATCTCTTCCAAACTGTTCAAGTATAAGATAGGATTTTCTTC

AAATAACTGGTTGGAATCATTGAATAGTTCTAGGCGAATTAAGTCATTTAACTGTGCATT

TTCAATCGAAACCAACTGAAAAACTTGTCCTTTATAACTTACTTGTGAACCGATTGGATA

TTCCCTCAAAGCTTCTTCTACAATTTTATCGACATTAGAAAAAGAGTTCGTTTCTTCCGT

TTCCTGTTCAATATCACTTGAAGGAGATAAAACTTCCTCTACTTGATTAGACTTTTCGCT

TCTAGCTTTTACATCATCAAAATGAGTAATAATCTTCAGCTTTTGTGTCAGTGGGAGTTG

TGTGTAGTTTTCTTCATGATGGACAAGATCCGTCACAGTATCTAAAGTAGCCATCAATTC

TGAACCCAAATAAGCTAGAACATCTCCTTCTTTTTCTTCAAAATGAATCTCAAGAACTTC

TTTTGTTAGTTCTTTGGAATCAACCATTAGAAGAAACTCCTCTATATCTCTACTAATGCG

GTCAGATAGATTATTCGCAACCCGATAGACATTGACTAAATTGACATCCTGATTCTGAAG

ACATACAAGTTCACTTAATGGTTCTAACTTCTCTTTTTCAGATTGGATGTAAAATCGAGT

GGAAAGATTATAGGTTGCGACTTCTAGCACCAAGTTTTTCTCAAAATTACTTAATTGAGA

TAATTTACTTAGACCTGCTTGTCCAAAATTTCTTGTGTAACTTTCTAAATCTTGGTCATT

ATTTTCAGAACTAGCCTCTAGGAAAGAAATAATATCTTGATGAAACGAATAGACATGAGG

TATCTCTTCTATCTTGCCCTCTTCAGCCAAAAAACTCTCTACCAAGTCCTGCCATTTCTC

ATCAAAATGTTGCGCTTGATAACGAAGAAATAGGTCCATTTTCTCCCTATCTTTTGGAAT

TGTTCCACGAAACATAGCCAATAATTTCATTACTTCCATACTCGCTCCTTTCATCTATAC

GAAAATAGGAGAATCACATGATTCCCCTACAACTCTATCTCCATTTCAACCTTTTGAGGA

CTGGTTTCTAAATCACTAGACAAAAGAGAAATAGAATCTGTTCTCATGTAGTAAGTATAT

AAGTCTTCTAGGTCTTGTTCGTTTTCAATCCCTGTTTCAATACTAATAAGAGCCTGTATA

AATTCCTTGTAGTGACTCTCCATCATATCTGAAATCTTTTGTTTCATTTCCATAAATACT

GAAGACGGTTCAGTTATACTTGTTTGGTCTATGTCTTGTAAATCCTTTTCTAAGGGATTG

GTTCCTTCTTCTACTTCCACTAAATCTGAACGTCCATCACCATCAGAATCTGCGCTAAGA

GGATTGGTTCCTAGCGCCAATTCTTGAGCATCAGTTAATCCATCTTGATCCGAATCACGT

TGATAAATGCCTTCCATATACTTCCTTCTTTCCTAGTTAATCTTTTTTCACTCCAATCCT

ACCAAATATTCCTTGAAAGAAGTATCACAACAAAAAAAGAGAACGAGCCAAAACTGCGTT

CTCCTGAAAATGATAAAAATATATTTTATGCCACAAAAGATATGTAAACTAATCTTTTAA

ATAATAAAAAAGTTCCGACGTTTGTAAATGTTTTGAGTAAATTGAAATTACTTTTTGACC

TTTTGGAAATACTGTCTTTCTCTTCAAATCTAATAAAGATTGAGGAACAATTTTCCTTGA

TGTCTCTTTCAATGTTAATGCTAGTATCTGTTTTCTTGTACGTAAAGCATAATCAAACTC

CAAATACAAATAACGTCCTGAACCAGTTAGATGAACATGCTTTCCCAATAACTCCTCAAT

GGATCCTAAAACTTGTAACTTTTGCATTGTGGTTCCATCTTTTTTTATTTTCAACTCATC

AATTATGACATGCCTATTTAAACAATCGTCAAAGAAACTGCCTGCACCTTTTCGGTAATC

AACACCACATAGGTGCATGAAATTAGTAGGTGAAAAATGAAGTTCAACACTTTCTATTTC

TGTTTCATAATACATGATTTTGCCAACGAAGTGTTCTTTAAAAAAAGAAGCTGCAATTTG

TAACTGAGTAAAAAAACGCTGAAGTTTTATTTTTTCGGTGTAATTTGGATTACGATAATC

TCTAGAATTTGCCATATTTTCCTCACAAAAAAGGTGGTTTACAGAGTCTGTAAGCCACCT

AGTCAGTCGGTTTATTCTGGTGTCTGCCACCGCTTGGCCCTTACGTCCAAGATTGCTATC

GGATTTCGTTCTGGTGTCCGCCACCGCTTGGCCCTTATGTCCAAGATTACTATCAGATTG

ACCATGAGCCGACTACTCATCTACACTATTTATTCTACCTGAAAATGGTTAAAATTTCAA

GTATAAACTCTTCTATTTCGTAGTATAGGCAAAACTAATGGCGCTATCTGCTTGCGAGAT

TTTTCTAAAGGTATAGTTAGGATTGCCACCATAGTTGGTTTCAGACACAAGGAAAGAACC

ATCATCATAGACCTTCTCTACAAAAGCCACATGACCGTATATAGCTGGTGTACCATGTGT

ACCTCCTACAAAAGAGACAATAGCACCTGCTTTTGGTGTGGAACCCGTTTCTCCACCAAG

ACTTGAAGCTGTCGCAACCCAGTCCTGACCATTTCCCATGGTATTAATGATTGAAATCTT

TTCTCCATTTCTACCTTTTAATTTTAAGCCTAACTGGTTCATACGAGCCGCAACACCCCA

TGTACATTGTCCATAAGCATAGGCCATACCATCTCCACCACCAGGAACAGAATGATCATA

CAAGTCCCCACGAACCCCTTCAAGGGATTGTGGATCACTCTTTGCCTGTCCTCCATTTGT

TTGACTAAAGCCTTTTTCAATTTGGTAATACCATTCCGTTGCTCTGGTTTGTCTTTCCAG

TAGTTTGTCACCAGAATTTCCCTCCCAATAGGTGAGGAAGAGTTGGGCCAGATTGGCTGC

ACTGCCTGTATTTCCAAAGAAATCCTTTAACCAACTTTGATAGTAAGGACTATCCCCATG

AAGCATAAAATCAAGTTGGAGGTCTAAATCATACCACTTCTTATTTTTGCTATGTGCATA

ATTCAATAAAGCTGTATGACGTGTTGAACCATCTGCAGTATCCGTCCATTGCCCTAAACC

TAAACCTCTATGAAGAATATTAGGATAAGCACCACTATAAATGGCTGGTCCTCCTATCGC

TAACCAGGTTTCATTATCCCATGAGGAATCGGTAGCGCCAACTGGAGGAGATAAATAATC

TCCCTCAGCTCGTTTAGGATTGATAGAAGACTCTACCGACCAATTTCCTAAAATAGCCGC

AATGGCTTGGGGACTTGCCCCTTGAGATTTCAAAAACTCATAAATATGTTTTGCTCGTTC

AAACTCATCCCCACCAAACTGACCAATGGCTGGTAAGATAGTGGTCTGAAGTTGAATGAC

TTTTGGAAAATAAAATTGCGGATTGACATACACCAATTTTTCTTTCTTGTTCTTATACTT

TTGATAGGAAACTTTCAAACCTGTATCGTCTGGTGTTTCACCAATAATATCACCTGTTAG

GACTCTTGTTCCCTCAATCGCACGGCCATTATGAATAGAATACAAGGTCAATCGACTCTC

ATTCTCTCCTTTTCCGTTAGTGAGAATAACATTGTCTCCATCTAGAGATACAACTCCATC

CATTGGTGCGACAATTGTTTGGTGAGCCTTTGCTTCTAGTAGAATGTACTCCTGAAGGGT

AGGTTTTCCGTCTAGATCATAATATCCGTAACGATAAGTCATGGTCAAACTATCTTCGTT

ACTTTTCCCTTCAAATGGATTGTCCAATTCCTGCATGGAAGCATAGATACCTTCTTCTTT

TAGTTCCTTCATTTCCTCTTGATCGTCTTTCGATAGTTTATACTTAGGAGTTTCATAGAG

GTCTTGCATGGATTTCAAATCTTCCCCATCGTTTAAATCATGCCACAAAGTAGATAGATA

ATCCTTGTAAGTTTCTGAACTAAATAAGTGAACTGGTTTGTGTAACTCATAGTCATGGAA

TTTAAAGTTCATATACCCCATCACATCATCAACTTTTGTGTAATAGGTAATTCCTTTGTC

ATTTGTACGAGTATGTTCTGCGTCTTCCCAAGTTAGGTGGGTATAAGCTTTTGTTAATTC

AAATTCATCTTGTTGAATCAAACTAGCAGATGAAAATCCTAAAAAGAAGCTCATTATAAG

TAAAAGAAGAAAGACTATTCCTCCAACTATCCAGGTTACAGGATTTCCAGCCGCAAATGT

AAAGAAGGAAAAGGCTGCTTTTAGTTTTTGATAGATATTTCGGACACTTGTAAGACCTTG

TTTCTTTAATTTTCGAAACCGATTTTTAAAGGAACTTGGCTTATCTTTCGCTAGTTTCCA

TCCTTTTCCATCCTTAAAATGATGGTATCGCTCTTTTGTGTTGGTCAGTCTTTTCTTGGT

AAAACGACATGTTGCTTGTCCTGTTTTGACACTAGCTTTTCCAAGGTTATAAGAAAGGCG

ACTGTAGCGTTTCCCTTTTCTAATTGTCTCTTGAAGTGTGCGATAACCATCTAAATCTTC

ATTTTCTGAAGCTAACTCTCCACCTTCACGTCCAAGGACATAAAGAAAAGTTTTGGCTTT

CCTACTGACTTTTTTGGACTTATAGGCTTGTTTAGTAGATTTTAGATTCTCTTTTGCGGT

CTTGACTTCTTTCTTAACTTTTAATTCTTCTAAACTCTTCCCTTGAAAGAAAAAATTGGA

TTTTGCTTTCGATTCCTGACCATAAAGAAATTTTTGATTGGTTTTTCTTTCTTTACGACT

CTCTTTTCTTTCTTCTTTTGCTTCTACCTTGGCTTCTCTATATTGCTTCTTTGCTATTTT

CAATCGTTTCCTAGCATGAGGTAATCGTCTGTCTCTTAATTCTTTGCGATTTAAAAGAGA

TGGAGGACTATTTTGAAGAATATGATTGTAGTCTTCATTTGCTTGTTTTACTCTAGCCTT

TGAAGCTTCTCTCATCTCCTCTAGCTTTTTCTTTATCTCTTTTTTCCATGCTTTTTCATC

CAGTACAGCGGAATCTTTTTTCTGTTTCTTCACCTCCTTCTTTCCTTTTTTCAAGAATTT

CTTCTCATCTTTTAGACTTCTTCTAAATGCCTTTCGGGCACGTATGATTTCTCTTTTATC

CTTCATTTACCTTCCCCTTAATTAGAAGCCATTTTATCAGGATCTGTACTCATGATATCA

AACAATTGAGTGCCTTTAGGAATCTTATTTTTAAAGGGAACTACAACGGAACCAGCTTTT

ATCAGTCCTGCACCTTTTTCTGGATTGACTAGGTATTTTTCAAGTTCTTTTGACAATCCT

AAGAGTTGAACCAATTCTTCTCGATCATTTTTTGCTTGCTTTAGGAGAATCATAAATTCA

CTATTTGCAATAATCCGTCTACCGTTTGGATCTAACAATAAGGTTTCGACATTTTGGGTT

ATCCCAGTCGGACTGGCTCCATATTTTCTGACACGACTCCACAATTTAAAGAAGAAATCA

CTGGCATATTTATCTAATAAGAGAAGTTGCATTTCATCAAAATAAATCCAGGTCTTCTTC

CCTAATTTTTGGTTCCGAACAACACGATTCCATATCTGATCAAACACTACCATAAGAGCG

ATTTGTTTCAACTCATCTCCTAACTTTTTAACGTTATAGATTAAGAAGTTAGATCCTGTC

TGAATATTGGTCTTATGAGAAAAAATATCAAGAGAACCTTCGACATACAGTTCCATATCA

AGTGCCAAATTCTGCGCTTCTTCTTCTAGTTGTTGGCTCAAGACAAAGACCCATTCTTCC

AAAGAAGGCTCTTTAAATGACTGATAAGTGAGTCTCGTAACTCGGTCGATAATCGATTTT

TCTCTCCCATCCATTTTTCTATCCAATAACTTACCGATAAAGGATAAAAGAAATTCTGAT

TTTACCTTTACAGGATCCTCATCCATATTTTCCTCAGACAAGTCAAGGACATTGAGATAA

GTTTGGGAATCGGGCGCAATATCAATCATTTCTCCCCCAAAAGCTCGTCCAATGACACTG

TATTCTGCTTCTGGATCCACGATGATAATTTCAGTATTTTCACCAGATTCCTTGATTTTG

GTCGTGATAATTTCATGCTTAGTTGCCATCCCTTTCCCAGCTCCAGATGTTCCTAAAATC

AGACCAGACGGTGTATTTAATAGGCTGCGATCAATGGTAATAATATTGCTTGAGATTTGA

TTGATACCATAATATTTCCCACTACGGTCTTGTAAGTCTACTGAAGTCCAAGGTGAGTTC

ACTGCTATATTGGACGTTAATAAACTCCGTGATACTCCCTCTAAAAAATCACAACCAAAT

GGCAGCAAACAATTAAAGGCTGCTTCTTGCATATATGGTAGTTTATCAATCATTAGGTCA

TTTGATCCGGCCACTTGTTGGATAGTGTCTAGTGCTTGTTTGAGTTCTTCTTCATCCTGA

CCAAAGACCCCAATCAAGAAGACTGTTTGAAACAGTTTATCTCCTGTCTCTGTCATGGTT

TTTAAGAGTTCCTCAGCTTCATCGATATTGCTTTCTAAAACATGACCTACTTTTTCCAAA

TAGATACCTGTACGAGCTAGTTTTTGTTGTTCCCCAATCTTTTGGGATTCCATCAAGGTC

TTCTTTGTTCGTAGTTTCTTCATAGCATCTGCCTTGGTCGAACTTTGAGCATGGAGACTC

ACAATCAATTCCAAATCTCCTTGCATGAGGTCTCTTATAAACTGATCCCCTAATTCCATG

CCGTAGTCTCTCACATAGACAATCTGCAATAAGCGGTCATTAATCTGTAGGTAATTCTTG

TTTTTAAAATCTAAGAGATTAGGTGCTATGAAGTGACGAGTTGTCTGACCAGATCTCGTT

AAATCACAGTAAGAAAAGGAAGATGGTGTTCTCCTCTAAGCATATCTGCCAACAAGTTGA

CACGTTCTTCTCCAGCCAAGGATTCAAAACGTGCATCAATTTCTGAAAAACCACTCTTGA

AATATTCTCCTATTTGAGACAAGGAACGATAGGCTTGTTTGGGATTAGAATCCTTTCTAC

CAAAGCTAATCAGTTTCACAGCTGAAAAATTATTTTCACCACTGTCTAAATTCTGATTCA

TCATCCGATTCAATTCTTTACGATAGGTATCATATCCATCTTCTTTTTCCTCATACAAAA

CACTTTGTCTAAACTTTTCTAAATTCAATCTTTTATTAAAGATGGTCAATTGGAAGTTGG

TTTGGTCATCTAGGGAGTTAATCAAATCAGAATACTTCTCAATGATTGCCCCCTTATCTT

CTAAACCAACGGTTTGGTAATTGACATCCCCAAGTAAGTAGCTTTGTGAGAAATAATCTT

CTTTTACCTGCATTAGACCATTTTGATACAGGGCTTGATAGGAAATGGTATTCGCCGTAG

AGGGTAACACTTCCTCTTTTTCCCTTTAACTTCTTCCTTTTTATAAGTCATTGAAGCTTT

TTGTTTCTTTAATGTATTTGATTTTCTTTTCATGTTCATGTCCTTTCTTTCCTGTAATTG

TGCGTAGGGGAACCGTTAATTCAAAATGAAGACGGTATTTCAAATAATGTTCAAAATATA

AATCATTGGGTTTATAGACTCCAAAAAGCATGAGGGGGATGGTAAAGGCAAACACAAAAC

CGTAAACAAACCAATCTCCAAATTGCCAGAAAAAGAGATTCAAGCCCAAAACAATAATTG

TGACAATAAAGGCTGGTAAAACAAAGATGATTTGCCTTGTAGTGAAGCCTAACCAAGCCC

TGTGTTGGTATTTTGAGATGTCTTTAAAGACACGTGTATTCATGACTTTCCTTTCTAAAA

AGGCTAAGAAGCAATCACTTCCTAGCCTTTATCTAATTACATACCTAAGATTGAGCGAGC

CGTACGTTGAGAACCAACGAGGGCAATAATCAGTAAGATAGCTTGTACCAAACTACCAAA

CATAATCGCAAGTGATTGCAAGACTCCTGCACCATTTGAAACAGCTATTTTCCCAGCAGA

TTCAAACAAAGGAACAAGAGAAACAATCAGAAAAATAAGAACCCCTTGTACCGCATAGAC

CATAATATTTTTAAATAGCCAATACCAATAGACTTCCATTCATCACTTAAAAATGTTGGA

ATCGTAAGAGGGGCAAATGGAATCATAAGGTAGAGTTGAATAAATCGAATAGATACCAAA

AGATTAACCATGGCAGCACTTACTATCCGAACAAGCCAAATGAGGAGGGCGAAAAAGCCC

ACAATCATCCGGCCAATAAATCCTGACCCTTTTAATCCAGAGATGGTATCATACTTTGCC

CCACCGTGAGCCACAATCGAGGCCACTTGCTCAATGGCGTGACTCGCAATCCCGATGATG

GCTTCTACAATCACGGTAGTGTTGGTAATTACAACTGCGACCATAATATAACTAATCAAC

ATCGGTGCTAATGCTTCAAAGGTCATCGCTCCTCCTGAGTTAGCAATTTTCTTTGCCATC

TTCGAAAATTCTAAGATGAGAACAACTGATAAAATCGCAACTCCAAGAGGCTGCATGACG

CTTTTAGTAATACTAGACATATAAGTCCAAACTGTTGGATTGTAGCTAGATAGAGATTTA

ATCAGATCTACCGTAGATTGTAAATCCACACTAAATCCTTCAAATAAATTTTCAGCTGAT

ATTTTTTCAGATGCAAGGTAAACAAAGGGTGAGACTAAACTAAGATTCATGTCATTGTTT

ATCCTCCTAAATTGAAATTTGAGTTACAAAGGCTCCAGCAGCCCCGACCATAACACCACC

GACAATTTCAAGAATGGCATTCCGAACACCTGGTCCACCATCTTTAATATTGGTTGCAAG

ATTGACAATCCCAACAACAACGAGAAAGGCACCAACCGCAATCAATCCCTTCTGTAACAA

AGACATAGCTTGTGCAAACATAGCACTTGCGTCTACTCCATAAACAAAACCTTTAAAATG

CGTAATCATCTATTTCCTCTTTTCTATTTTTATTTTAAACTAGATTCAAAAGTCAAATCA

CGAATTCTAAGGCCTTCAAGATGATTTTCCTGACTTTGATTCAAAGGATTGATCTGATAG

TCCCACCACCTTTCATCGGTTTCTTGATTGGCTAGGTACTTCCAGTTTGGATGCTTAGTG

GAATTGTATTTTTTGCTTTTAAAGACAGGCATATTGGCAATTCGAACTAAGCATTCATGC

CGTTTCATATTTCCGACTTCATCAGGTGTCATTAAATCACGAGCAATCTTTTGATGAGAA

AGGGATCCTGAACCTGTTTGGCCAAAGGAACGACTAGTATTTCGAACATCAATGGTTTGT

TTACCGAGTAACCCACTCATAAATTTAAAAGTATCTTCATCATTACCACCTAAGTAGACT

AAGCTATCACAGTTCCCAAGAATAGTTTTCCAAGCTTCTTTTTCTTTATAGAGTCCTTGA

AGTTGAGCAATATTTTGTAGAATAGGAACGAGACTCATATTCCGAGAACGGACTGTTGAG

GTTTGTTCAGCAAAATCGGGAATTTCTCCGATATTTGCGAACTCATCTAAGTAAACTCTC

ACATGAAGAGGCAATTGACCCTTAAAATCAATATCTGCTTGTCTTGTTAGGGTTTGAAAT

ACGGTTGAAAAAAAGAGGGCTGATAGAAAGCGAAAGGTACTATCGTTATCTGGGATAACT

AAGTAAACCATTGATTTTTCCTTGCCCCATGTCTTCATATCAAGGGTATCTCTTTTGGTC

AAATCCATGACACTTTGAATATTGAAGAGGGCAAATTTAGCAGTGGTTACAGCTATGACA

GAATCCAGAGTCTTATCCTTATAATTTTGGAAATCTGCCCAATTTCGCATGGTAAAATTT

TCAGTCCCATACTTTTTAGCATAATTTTCAAATAGAATTTCTAAGACACTTTTTTCTTGG

TTTTCACCCTTGGATAAGTGTTTAATGAGTTTTGAGATTTCAGCAAAACTTGGATAACGC

CCTCGTTTTTTTCGCTCTTCCACTTCTTTTTTTGACGTTTCAACAAGTTTTGGTGTTCTT

TTTGACTTAAACGACTCTCTTCAATGAGCTGTTCTCTTGTTTTAGGTGGATTATAGAAAT

CGACCAAGTAAGAGGCTAAAGCTCGTACCAAAGTCATAGAAGCTTCATCCCAAAATGGAT

CACTACGAGATCCAGAGCCTTTGGTGTTATTAAAATAAACCGTCAGCATGCGATTCAAAT

CATTTTCTGTCTCTATATAGCGAAAAGGATTGAAGCCATCTGAGTTCTTCATATTAACTA

AATCTAACACCTTTACTTGGTAGCCATGTTCTAAAAAGAGTTTGCCTGTTTTCTCGGCCA

AGTGATCTTTAGGATCCACCACAATATTAGAACTATTCATCTGAATTAGATTGGGTTTCA

CAAAGCGAAATGTCTTCCCACTTCCTGAACCTCCTATCACCGCAATATTCTTGTTTCTAT

CATATTGGGGTGGTTTTTTATCTAATAATGTCAGACGAACATCTTGGGCTAAGATTGTAT

CATGAGAAAATTCCTTGCCGTAAAAGAGCTTCTTTTCTTTTAGAGTTCCAAAATGGGCGC

TCCCGTATTCTATCCCTTCTCGGTATTGTTTTTTGCCAGTCTCTAAATAAAGATAAACCA

GCAACATCATCACAAAGCCTAGTAGAAAAAAAGCACTGGATTTTCCAGTAAAGGAAACAT

TCCATGGCGACTGAAGAACTTCATCTTGACCTTCCATTAGAAGATGAGTCCATTTATCTA

AGCTATTTCCAGTATAGGAATCATACAAAAGCGTCAAACGATGAAAAAGATAACCTAGTA

AGATACCTAACAGTGAGAATAGTAGGAATTTCTTTCCACTGTACATCATCTCACCATCTC

TTTCTGTTTGACGGCTCCTTCTTGTCTAAAGGTAATTTGGGATTTAGCCTCATCAATTGC

ATCGTCTAATGACTTATCCATGGTAAAATCAGCTAATTTCTCTGGATCATTAACCATTTT

TTCTAACAGTTGGTCTAAATGATTGTCCAGAATCGAACGGTCTTTCGTATAGAAATGCAG

AGAATCCCCTTGCCAAGCGATGGCTAAAGGAATCTCTTCTTTTTCTAAAAAAGCTTTAAA

TTTCTCTATATCAATTGGTTTGTCTAAAAAATCTTTTTTCAGATTAATCGTATCAATCGA

ATAAGGAGATTGTAGCAATTCTTCTAATTTCTGTACCCCTATTTTATAGGCGGAATCCTG

TGCTAAAGCCTGACGTCTAGACCATTCTAGAATCTTTAAGAGACTTTTTACAGTAAATAA

AAGACTACGCTCTGCATATTGAACCGCCATTCGTTCCTGTTGTTCAGAGGACATCTGATG

CCTCCTCCTTAACAAATAGCAGCTTTCCTTCTTTATAACGATAAGCTATCAATTTCTGAC

GTTGCTTAATCGACTTGACAACCTGCAGTAGATCTCTCGAATAAGGTTCTCGAAGAGTAA

CTTCTACTGCTTTCCCATCAACGATACCAACACGTTTAAATTCAATGTAGTCTTTTTTGA

GATGTCCTAAGCCCATACCAAAAGGAAAGTGATGAATCAATTGGATGGTACAACCACCTT

TATTTCCCATTTGTTTCAAATCATACAAGTTTACTACCTTCATGATTAACTCCTTTATCT

AGTTTGTCGCATCGTTTAAGTCTGGTAACGATAAACTCCGTGTCTGTTAGAAAATTCTCA

CACACGTCTTGTGCCAGTTGCCCTTCACAGGGAAATACTCTCAGTCCCTACTTACACAGG

CACGCTAATCAAGACGGAGTGGATTCAATTTTCAAAGAACAGGTAGCTTTATTATAGATA

AGAGTAGTTGAAATTTTTATCACATTTTTGGGGTTGTTGGAATAAAAAAAGCAACTCACT

GCTTTACGAATGAGTTGCTTTTGTACTAAAATATTTGTTGCATAATTTCCCTAATGACCT

GAACAGAGTTATCAAATGAGATATCTTTAGCATACTTAAATTGGCGTTGATAACGAATCC

ATAGCTCTTTCTGATAATCAGAAATTTCAATTCCTCTCACTTGTTCTTCCCAATTATAAA

TAGTATCTTTACTTTTACGTTTTTCTGCCGTATTCTCAAGTGCATTTTTTAATACTTCTA

GATTAATCTCTTCTTTTCTAAGTTTATACAAGGTGAAAAGATCATATCGATCACGTGGGC

GTGTCGAAGCTAATCCCCTACTGATAATCGTTTCTAACTTTTCAGCAAGTACAGTCTCTA

GATTATATGTCCATATCTTGATACTTTCATTAGTAAAGATAGAAGTCATTGAATAAGTAA

TTTCTCTTGGTGTAATCTTATCACCAGTAGTAATATCAATAAAAACAACCTCCTTTAATG

TATCAAAAGTTGCATTTAATTTTAATGAAAATCCTCCATATTCATCATCTTGGCGAATAG

GTTCTAGCTTATCTACTGAAAATGAAAATCCATCAGTTTTAGAACAAAGAATCTCTTCAA

AGATATGAATCAAATTTTCTCTGCTCATTTCGGTTCCCTTCAGAGTGACATCTAAATCCA

TTGTGGTTCGCTTATCTAGTCCAATCATTTGACCTATTAGATACCCTCCTTTTACAATGA

AGGAATCTCTATAAGAACTAGTAGAAATCAGCTTTAATACTTGCTCAATTAAATAATGTT

GTTGTACTTGTTGAGCTGGAATTCCCTTATCTTTTGAAATGTTTTTGATTTTTGCTTTAA

AGCTATTCGCATTTGAAAACATTAAGATAGTACCTCCGTATAAGATTGTAACTTATCAGT

TACTTTAAATAGCTGGGCATAATAAAATAATTTGTGTAAATGAATTTTATTTTGTTGAAA

ATATTTTTTAAACGCTGGTGCAATAATTTGAAGATCCACCTGATGAACTGGTCTTAGACA

TTCAACCAAAACTCGTTCAACTTCATAAACTTTAATAAATTGACCAGGAAGGCGCTCAAT

TTCAATAATTCCTTCACTATAGTGAGAGCGTAAAATAATAGGACATATATCTGCTTCCTT

AATGTTTTTGGTATTCGTACCATAAGGAAAACTCATCGTCATATTAAAAGGGATAGTCAA

AGATAAACCATGTAACCAAAGTGCTGTCTCCAAAGAAAAGATTCCCTTTGGAAATCGATA

CTGAAGGACAAACCACTCATCTAAATAAATATCAGGTAAGCGATATAACCCTTTTTCTTC

AGCATCGATTTTTCCTTCTGCAACCATTTTTAATAAGGTTTTGTAGTGTAAATTTTCATC

TATTACTTGTTTAAAAGTTAAAAAACCATACTGATTGAAAGAGTTCATCAGTTTCTCTCT

TTTATCAACCATATCATTCTCCTTTCTTAGAAATAAAATGCTAACACTTACTATATATGT

TAGCATTTTATTTCTTTAAAATCAATTCATTTAGTTTTTTTAAAATAAAAATAGCATAAA

AATCTAGTTATCCGCATAAAAACTGGACTTATCACACTTTATCAAGGTCAAAACCACTCA

ATTTACTACTAATTTACTACTTATGAATGAGCTTTGATACGACGATTTATCCTTGAAAAG

TGAAGATATAAAGATACTTCCAATAAAATTTGAATATTTAATAGGTAGACACTTCAAAAA

ATGAGGTGTCTATTTTTTTACCCGATTTTGAAAGGAAGTGAACTTATGAAAACAAAAAAT

CAAGAATCAAAAGGTCGTTCCCCACTCTTTAAGACCATCAAACATTCATTCAGCCAATAA

AAAGAAAGGATAGGTAAAAATATGGAACTTAAATTTGTGATTCCCAACATGGAAAAAACA

TTCGGCAATTTAGAATTTGCTGGCGAGGATAAAGTCGTTCAGCGAAGAATCAACGGACGG

CTAACTGTCTTATCAAGAAGCTATAATCTCTATTCTGATGTTCAAAGAGCAGATGATATT

GTGGTGGTGCTTCCTGCTGAAGCTGGCGAAAAACATTTCGGCTTTGAGGAACGTGTGAAG

TTAGTCAATCCACGTATTACCGCAGAGGGCTACAAAATCGGCACTCGTGGTTTTACAAAT

TACCTTTTACATGCTGACGACATGATAAAAGAATAAAGAAAGAGAGGAAAAATGATGAGA

TTAGCAAATGGCATTGTATTAGATAAAGACACGACTTTTGGAGAATTGAAATTCTCTGCT

CTACGTCGTGAAGTGAGAATCCAAAATGAAGACGGGTCGGTTTCAGATGAAATCAAGGAA

CGTACCTATGACTTAAAATCCAAAGGACAAGGACGCATGATTCAAGTAAGTATTCCTGCC

AGCGTGCCTTTGAAAGAGTTTGATTATAACGCACGGGTGGAACTTATCAATCCCATTGCG

GACACCGTTGCTACTGCCACCTATCAAGGAGCAGATGTTGACTGGTATATCAAGGCAGAC

GATATTGTGCTGACAAAGGATTCTAGTTCATTCAAAGCTCAACCACAAGCAAAGAAAGAA

CCGACACAAGACAAATAGTCGCTAGGTAGAAAGGAGACTTTTTCGCATGAAACAGCGTGG

TAAAAGGATTCGCCCATCTGGTAAAGATTTAGTCTTTCATTTTACGATAGCGTCACTCCT

GCCTGTTTTCCTGCTGGTTGTCGGACTGTTTCATGTGAAGACAATCCAGCAGATCAACTG

GCAGGATTTTAACCTATCACAAGCAGATAAGATTGACATTCCCTATTTAATTATCAGTTT

CAGTGTCGCAATTCTTATCTGCTTGCTGGTAGCGTTTGTATTCAAACGGGTTCGCTATGA

TACGGTTAAACAACTTTACCACCGTCAAAAACTGGCAAAGATGATACTTGAAAACAAGTG

GTATGAATCTGAACAGGTCAAAACAGAGGGTTTCTTTAAAGATAGTGCTGGTCGTACAAA

GGAAAAGATAACCTACTTCCCTAAAATGTATTATCGACTTAAAAATGGCTTGATACAGAT

ACGGGTGGAAATCACGCTGGGAAAATATCAAGACCAACTCTTACACTTGGAAAAGAAATT

AGAGAGTGGCTTGTACTGTGAGCTGACGGATAAAGAGTTAAAGGATTCCTATGTGGAATA

TACTTTGCTCTATGACACCATAGCCAGTCGTATTTCTATTGATGAAGTAGAAGCTAAAGA

TGGTAAACTTCGCTTAATGAAAAACGTATGGTGGGAATATGATAAGCTCCCTCATATGTT

GATTGCTGGTGGTACAGGTGGCGGTAAAACTTACTTTATACTGACACTGATTGAAGCCTT

GCTTCATACAGATTCAAAACTGTATATTCTTGACCCGAAAAATGCTGACCTTGCGGACTT

AGGTTCTGTGATGGCAAATGTCTACTATAGAAAAGAAGACTTGCTTTCTTGCATTGAAAC

ATTCTATGAAGAAATGATGAAACGTAGTGAGGAAATGAAGCAGATGAAGAACTATAAGAC

TGGCAAAAATTATGCTTACTTAGGTCTCCCGGCACACTTCTTAATCTTTGATGAATACGT

CGCTTTCATGGAAATGCTGGGAACAAAAGAAAACACCGCAGTTATGAATAAGCTGAAACA

GATTGTCATGTTAGGTCGTCAAGCTGGCTTCTTTCTAATACTGGCTTGTCAACGTCCAGA

CGCAAAATATTTAGGCGACGGAATCCGTGATCAGTTTAATTTCAGAGTGGCTTTAGGTCG

TATGTCTGAAATGGGCTATGGCATGATGTTTGGCAGTGACGTACAAAAGGATTTCTTCTT

AAAGCGAATCAAAGGTCGTGGCTATGTTGATGTAGGAACAAGTGTCATATCAGAGTTTTA

TACTCCCCTTGTACCAAAAGGATATGATTTCTTGGAGGAAATTAAAAAGTTATCCAACAG

CAGACAGTCCACGCAGGCGACGTGCGAAGCGGAAGTCGCAGGTGTGGACTGATCTTGCTG

GCTGGTGTGGCAATAGCCACGCCAGCACTTAACCCCCCGTATCTAACAGGGGGGTACAAA

TCGACAGGAAACAGTCAAAAAAACATTAGAAAATCCTTTGGTTACAAGGGATTTACAAAA

TTTCAGCGTATGTCAAATGGGCTTTAAAAGTTGACATACGCCTTTTTGATTGGAGGGATT

TTTACTGAATGAACAAACTTGGTTACAGCATTTAAAAGAAAAACGCTTGGCTTATGGACT

ATCTCAAAACCGTTTAGCTGTTGCGACTGGTATTACAAGGCAGTATCTAAGCGATATTGA

AACAGGAAAAGTCAAGCCATCAGAGGATTTACAGCAGTCCCTTTGGGAAGCTCTGGAACG

CTTCAATCCCGACGCTCCCCTTGAAATGCTGTTTGATTATGTAAGGATTCGCTTTCCGAC

AACAGACGTACAGCAGGTGGTCGAAAACATCTTACAACTGAAACTGTCCTATTTTCTTCA

TGAGGACTATGGTTTCTATTCTTATTCAGAGCATTATGCTTTAGGCGACATATTCGTCCT

TTGCTCCCATGAACTGGACAAAGGAGTTCTGGTGGAATTGAAAGGTCGTGGGTGCAGACA

ATTTGAAAGCTATCTTCTGGCACAACAAAGAAGCTGGTATGAGTTCTTTATGGACGTTTT

GGTGGCTGGCGGTGTGATGAAACGCCTTGACCTTGCCATTAACGATAAGACAGGGATTTT

GAATATCCCTGTACTCACTGAAAAGTGCCAACAGGAAGAATGTATCTCCGTCTTCCGCAG

TTTTAAAAGCTATCGCAGTGGCGAACTGGTACGCAAAGAGGAAAAGGAATGTATGGGAAA

CACCCTCTATATCGGTTCATTACAAAGTGAAGTTTATTTCTGTATCTATGAAAAGGACTA

CGAGCAGTACAAGAAAAATGATATTCCCATTGAAGACGCAGAAGTAAAAAACCGTTTTGA

GATTCGATTGAAAAATGAGCGTGCCTATTATGCAGTCCGTGATTTACTCGTCTATGACAA

TCCAGAGCATACCGCCTTTAAAATTATCAATCGGTATATCCGTTTTGTAGATAAAGACGA

TTCCAAACCTCGTTCTGATTGGAAACTGAATGAAGAATGGGCTTGGTTTATTGGGAACAA

TCGTGAACGATTAAAACTAACCACAAAACCAGAGCCTTACTCCTTCCAAAGGACGCTGAA

CTGGCTATCTCATCAAGTTGCCCCGACCTTAAAGGTTGCGATTAAACTTGATGAAATCAA

CCAGACGCAGGTTGTAAAAGACATTCTCGACCATGCGAAACTGACAGACCGACACAAGCA

GATTTTGAAGCAACAGTCAGTAAAAGAACAGGACGTGATAACAACAAAAAAATAACTCAA

ATACAAATTCATTGAATATAGAGAGGAGAACATTTTTATGAATTTTGGACAAAACCTTTA

TAACTGGTTTCTATCAAACGCTCAATCACTGGTGCTTTTAGCAATCGTTGTGATTGGCTT

GTATCTTGGCTTCAAGCGTGAGTTTAGCAAACTGATTGGCTTTTTAATTATTGCGATTAT

TGCGGTTGGCTTAGTCTTCAACGCTGCTGGAGTAAAAGACATTTTACTAGAGCTATTCAA

TCGCATTATTGGTGCTTAAATAAAACCGTTCTTTTGTGGAATATAAGTGGTTTTCTTATG

TTCCGCAAAGGAATGGTACACCAAACGAAGTGCGGTAGGGATTTTTGAATCTCTACAAAG

AAAGGACGTGAATATATGGACGATATGCAAGTCTATATTGCGAATTTAGGCAAATACAAT

GAGGGCGAATTGGTCGGTGCGTGGTTTACCTTTCCCATTGACTTTGAGGAAGTCAAAGAG

AAAATCGGCTTGAATGATGAATATGAGGAATACGCCATTCATGACTACGAGTTACCCTTT

ACGGTTGACGAATACACTTCCATTGGCGAACTCAATCGACTATGGGAAATGGTATCGGAA

TTACCCGAAGAATTACAATCGGAGCTATCTGCTCTGCTCACTCATTTTTCAAGCATTGAA

GAACTAAGCGAACATCAAGAGGATATTATCATTCATTCCGATTGTGATGATATGTATGAC

GTGGCACGCTACTACATTGAAGAAACGGGTGCTTTAGGCGAAGTACCAGCTAGTCTTCAA

AACTATATTGATTATCAAGCCTATGGTCGGGATTTAGACCTTTCAGGAACGTTTATCTCA

ACCAATCATGGGATTTTTGAAATCGTCTATTAAATCTGTCGGTACATTACTACTGGCAGA

TTTTCTATTTTACGGGGTGGCTCAATCAGCTACCCCTATTTTTTATGAAAGGATTGATTA

CATGAAGAAAATACGAAGCTATACCAGTATCTGGTCTGTGGAAAAGGTACTGTATTCTAT

CAATGATTTTAGACTTCCGTTTCCCATAACCTTTACGCAAATGACATGGTTTGTCGTGTC

ACTCTTTGCAGTGATGATACTTGGCAACTTGCCCCCTCTTTCCATGATAGAGGGAGCATT

TCTCAAATACTTTGGGATTCCTGTGGCTTTCACATGGTTTATGTCTACAAAAACTTTTGA

TGGTAAAAAGCCTTATGGATTTTTGAAGTCTGTCATTGCTTATGCACTGCGACCAAAGCT

GACCTATGCAGGAAAAAAAGTAACGCTTGGCAGAAACCAGCCACAAGAAGCCATTACAGC

AGTTAGGAGTGAATTTTATGGCATATCCAATTAAATACATTGAAAACAATCTCGTCTGGA

ATAAAGACGGGGAATGTTATGCTTACTATGAGCTTGTTCCTTACAATTACTCATTTCTAA

GTCCAGAACAGAAAATACAAGTGCATGATTCTTTCAGACAGCTTATCGCACAAAATCGTG

ATGGCAAAATTCATGCTTTACAAATCAGTACAGAATCCAGCATACGTTCTGCACAAGAGC

GTTCCAAAAATGAAGTCACTGGCAAGCTCAAAGCGGTTGCCTATGACAAAATCGACCAAC

AGACAGACGCTTTAATATCCATGATTGGCGAAAATCAAGTGAACTACCGTTTCTTTATCG

GCTTTAAGTTGCTTCTCAACGATCAGGAGTTTTCTATGAAAAGTCTTACCGTTGAAGCAA

AAAATGCTTTGTCTGATTTTGTCTATGATGTGAACCATAAGCTGATGGGCGATTTTGTTA

GTATGAGTAATGATGAAATCCTGCGTTTTCAGAAGATGGAAAAGCTCTTAGAAAATAAAA

TCTCTCGTCGTTTCAAAATCCGCAGGTTAGATAAGGACGACTTCGGCTATCTGATTGAAC

ACCTTTACGGACAGACAGGCACTGCCTATGAAGAGTATGAGTACCATCTATCAAAGAAAA

AGCTGGATAATGAAACGCTGATTAAATACTATGACTTGATTAAGCCTACTCGCTGTTTGG

TGGAAGAAAAACAGCGATATTTGAAAATCCAGCAGGAAGATGAAACCGTCTATGTAGCTT

ACTTTACCATTAACAGCATTGTCGGAGAACTGGACTTCCCGTCCTCTGAAATCTTCTACT

ACCAGCAACAGCAATTTACATTCCCGATTGATACGTCAATGAATGTGGAAATTGTAGCGA

ATCGTAAAGCCCTATCTACTGTCCGCAATAAAAAGAAAGAACTGAAAGACTTGGATAACC

ACGCTTGGCAAAGTGATAATGAAACCAGCTCCAATGTGGCGGAAGCTCTGGAAAGTGTGA

ATGAGCTGGAAACCAATTTAGACCAAAGCAAGGAATCTATGTACAAGCTGTCTTATGTGG

TAAGGGTATCAGCAAATGATCTTGACGAACTCAAACGTCGTTGTAATGAAGTGAAAGATT

TTTATGACGATTTAAGCGTAAAACTGGTACGACCATTTGGGGATATGCTCGGCTTACATG

AAGAATTTTTACCTGCCAGCAAGCGTTATATGAATGATTATATTCAATACGTGACCTCTG

ATTTCCTCGCTGGTTTAGGTTTTGGTGCTACTCAAATGCTGGGGGAAAATGAGGGGATTT

ATGTTGGCTACAGCTTAGATACTGGACGCAATGTCTATCTGAAACCTGCTCTTGCCAGTC

AAGGGGTTAAGGGTTCAGTAACCAATGCGTTAGCGTCGGCTTTTGTTGGTTCGCTGGGTG

GTGGTAAATCCTTTGCGAATAACCTTATCGTCTATTATGCGGTGCTTTATGGGGCACAAG

CAGTGATTGTAGACCCAAAAGCAGAACGTGGCAGATGGAAAGAAACCTTGCCAGAAATTT

CCCATGAAATCAATATCGTCACTCTGACTTCTGATGAGAAAAACAAAGGCTTACTTGACC

CTTATGTGATTATGAAAAATCCCAAAGATTCTGAATCACTGGCTATTGATATTCTGACAT

TCCTTACGGGGATTTCCTCTCGTGATGGGGAACGCTTCCCAATCCTTAGAAAAGCCATTC

GTGCAGTAACCAATAGTGAAGTACGAGGGTTGATGAAAGTGATTGAGGAATTACGGGTTG

AGAATACGCCACTAAGTACCAGTATAGCCGACCATATCGAAAGTTTTACAGACTATGACT

TTGCACATTTATTATTCAGTAATGGTTATGTGGAGCAGTCTATCAGCTTAGAAAAACAAC

TGAACATTATACAGGTTGCGGACTTGGTACTTCCCGACAAGGAAACTTCCTTTGAGGAAT

ATACCACTATGGAGCTTTTATCCGTTGCTATGCTGATTGTCATTAGTACCTTTGCTTTAG

ACTTTATCCATACAGACCGAAGCATTTTCAAGATTGTAGATTTAGACGAAGCATGGAGCT

TTTTACAGGTAGCACAAGGAAAAACACTATCTATGAAGCTGGTTCGGGCTGGTCGTGCTA

TGAACGCTGGAGTATATTTCGTGACCCAAAATACAGACGACCTCTTAGATGAAAAACTGA

AAAATAACCTCGGCTTAAAATTTGCATTTCGTTCCACTGACCTTAACGAGATTAAAAAGA

CCTTAGCCTTTTTTGGTGTAGACCCAGAGGACGAAAACAATCAGAAGCGATTGCGTGATT

TGGAAAACGGGCAATGCCTTATCAGTGATTTATATGGTCGTGTCGGTGTGATACAGTTCC

ACCCTGTATTTGAAGAACTGCTCCATGCCTTTGATACCAGACCACCTGTGCGAAAAGAGG

TGTAAATGTGAAACCATCAATAGTAAACAGAATAAAATCAAACTGGACGCTGAAACGTCT

AGGTAAAGTGGCAATGACAGTGGCTTTCACACTTGTGATTGCCATTTTTCTTTTAGCCAT

GCTGGGAACGGTGGTTCAAGCTGCGGGCTTGGTAGATGATACGGTCAATGTGGCAAATGA

ATACAGCCGATACCCACTTGAAAACTATCAACTGGATTTTTATGTGGATAATAGCTGGGG

CTGGCTTCCGTGGAACTGGTCGGACGGGATTGGAAAACAGGTCATGTATGGACTATATGC

CATTACCAATTTTATTTGGACAATCAGTTTGTATGTTTCCAATGCGACAGGTTACTTAGT

ACAGGAAGCCTATTCCTTAGACTTCATTTCCGCTACAGCAGATTCCATTGGTAAGAATAT

GCAGACCTTAGCTGGTGTGAGTGCAAACGGATTTTCAACAGAGGGTTTCTATGTTGGATT

CCTCTTACTCTTGATTTTGGTTCTTGGGGTTTATGTTGCCTATACGGGACTGATAAAGAG

AGAAACCACAAAGGCAATTCATGCCATTATGAATTTTGTGCTGGTGTTTATCCTATCGGC

TTCCTTTATTGCCTACGCTCCCGACTACATTAAAAAAATCAATGACTTTTCATCAGACAT

CAGTAATGCCAGTTTATCACTTGGCACGAAGATTGTCATGCCCCATTCCGATAGTCAAGG

CAAGGACAGCGTGGACTTAATCAGAGATAGCCTGTTTTCCATACAGGTTCAGCAACCGTG

GCTACTGCTTCAATACAACAGTTCAGACATTGAAAGTATCGGTATTGACCGTGTGGAAAG

CCTGCTCTCCACCAGCCCAGATTCCAACAATGGCGAAGACAGAGAAAAAATTGTTGCGGA

AGAAATTGAAGACAGAAGCAATACCAATCTAACCATTACAAAGACCATTAACCGTTTAGG

TACAGTCTTCTTCCTATTTGTCTTCAATATTGGGATTTCCATATTTGTATTCCTATTAAC

AGGAATCATGATTTTCTCGCAGGTACTTTTTATCATCTATGCTATGTTTCTGCCTGTGAG

CTTTATTTTAAGCATGATTCCATCATTTGATGGTATGTCAAAACGAGCCATAACAAAGCT

CTTTAATACCATTTTGACACGAGCTGGAATCACATTGATTATTACGACAGCATTTAGTAT

TTCAACCATGCTCTATACCTTATCGGCTGGTTATCCGTTCTTTTTGATTGCTTTTCTACA

GATTGTGACCTTTGCAGGAATCTACTTCAAGCTGGGCGATTTAATGAGTATGTTTTCTCT

ACAGAGTAACGATTCTCAAAGTGTGGGAAGTCGTGTGATGAGAAAACCTCGTATGCTTAT

GCACGCTCACATGCACCGTCTACAGCGGAAACTTGGACGTTCCATGACTACTCTAGGGGC

TGGGTCTGCCATTGTTACAGGTAAAAAAGGACAGTCGGGTTCGGGGAGTTCTGCAAGGAC

ACAAGCAGATCACTCCCGACCAGACGGAAAGGAAAAATCAACACTTGGAAAACGTATCGG

TCAAACCATCGGTACAGTAGCTGATACCAAAGACAGAATGGTAGACACTGCTAGTGGTTT

GAAAGAACAGGTTAAAGATTTGCCGACCAATGCAAGATATGCAGTATATCAAGGAAAATC

CAAAGTAAAAGAGAATGTCCGTGATTTAACCAGTAGTATTTCTCAAACCAAAGCGGACAG

AGCCAGTGGACGCAAGGAACAGCAGGAACAAAGGCGAAAAACCATTGCGAAGCGTCGCTC

TGAAATGAAACAGGTCAAACAGAAAAAACAGCCTGCTTCTTCTGTTCATGAAAGACCGAC

TACAAGACAAGAACAATATCATGATGAACAGACCTCAAAACAGTCTAATATTCAGACTTC

ATATAAGGAATCTCAACAAGCCAAACAAGAGCGTCCAGCAGTTAAGTCCGATTTTTCAAG

TCCAAAAGTGGAACGCCAAGGCAATACCGTTCAAGAAAAAACCGTTCAAAAGCCAGCAAC

TTCAACCACTACAGCAGATAGAACTTCACAACGTCCAATCACAAAAGAACGTCCGTCTAC

TGTTCAAAGAGTACCACTACAAAATACAAGAAGTAGACCACCAATCAAAACCGCCACCAT

TAAGAAAGTCGGTAAGAAACCATGAAGTTGAAAACTTTAGTGATTGGTGGTTCTGGATTA

TTCTTGATGGTCTTCTCACTGCTTCTGTTTGTTGCCATTTTATTTTCAGATGAACAGGAC

AGCGGAATTTCCAATATTCATTATGGAGGTGTGAATGTTTCCGCAGAAGTGCTGGCTCAT

AAGCCTATGGTAGAAAAATATGCCAAAGAATATGGCGTTGAAGAATATGTCAACATACTT

CTTGCGATTATACAGGTGGAATCGGGCGGTACTGCGGAAGATGTTATGCAGTCCTCGGAA

TCCCTCGGTCTTCCACCTAATTCATTGAGTACAGAAGAATCCATTAAGCAAGGTGTGAAG

TATTTCAGTGAATTATTAGCCAGTAGCGAAAGGCTCAGTGTAGATTTAGAATCGGTTATC

CAGTCCTACAATTATGGTGGTGGTTTCTTAGGGTATGTGGCTAATCGTGGAAATAAATAT

ACCTTTGAACTGGCTCAAAGTTTCTCAAAAGAGTATTCAGGTGGCGAAAAAGTGTCTTAC

CCCAATCCCATAGCCATACCTATCAATGGGGGCTGGCGATACAACTATGGCAATATGTTT

TATGTGCAACTGGTAACGCAGTATCTTGTCACAACAGAGTTTGATGATGATACGGTACAA

GCCATCATGGACGAAGCACTGAAATATGAGGGCTGGCGATACGTTTACGGTGGAGCTTCC

CCGACTACTTCTTTTGATTGTAGCGGACTGACACAATGGACGTATGGAAAAGCTGGAATT

AACTTACCACGAACCGCACAACAGCAATATGATGTGACCCAGCATATCCCACTATCGGAA

GCACAAGCTGGCGATTTGGTTTTCTTTCATTCTACCTATAACGCTGGCTCTTATATTACT

CATGTTGGGATATACCTTGGCAATAACCGTATGTTTCATGCAGGCGACCCAATCGGTTAT

GCCGACTTAACAAGCCCCTACTGGCAACAGCATTTAGTGGGAGCAGGACGAATCAAACAA

TGAGAAAGGAAGATTTAATGATGAAATTTAGAAAAAATCAGAATAAAGAAAAACAGATAC

CAAAGGAAAAGAAACCTCGTGTCTATAAGGTCAATCCTCATAAAAAGGTTGTGATTGCCT

TGTGGGTACTTTTAGGGCTTAGTTTCAGCTTTGCGATATTCAAGCACTTTACAGCTATAG

ATACTCATACTATTCACGAAACAACTATCATAGAAAAGGAATACGTTGATACTCATCATG

TAGAAAATTTTGTAGAGAACTTTGCGAAAGTCTACTATTCATGGGAGCAATCCGATAAGT

CCATTGATAATCGAATGGAAAGTCTAAAAGGCTATCTGACAGATGAACTTCAAGCTCTCA

ATGTTGATACAGTACGCAAAGATATTCCTGTATCGTCTTCTGTAAGAGGATTTCAGATAT

GGACGGTAGAGCCAACTGGCGACAATGAGTTTAATGTAACCTACAGTGTAGACCAGCTCA

TTACAGAGGGAGAAAATACAAAGACCGTCCACTCTGCTTATATAGTGAGTGTCTATGTAG

ATGGTTCTGGAAATATGGTACTGGTTAAGAATCCGACCATTACCAACATACCTAAGAAAT

CAAGTTATAAACCAAAAGCCATTGAAAGTGAGGGGACGGTTGATTCCATTACAACCAATG

AAATCAATGAGTTTTTAACGACGTTCTTCAAGCTCTATCCTACAGCGACAGCCAGTGAAC

TTTCCTACTATGTGAATGACGGGATATTAAAACCAATCGGAAAAGAGTACATCTTTCAAG

AACTGGTAAATCCTATTCACAATCGTAAGGATAATCAAGTCACGGTATCGCTGACAGTGG

AGTATATCGACCAGCAGACCAAAGCAACGCAGGTATCTCAATTTGATTTGGTACTTGAAA

AGAACGGGAGTAATTGGAAGATTATAGAATAACAAATATTGGTACATTATTACAGCTATT

TTGTAATCACGTACTCTCTTTGATAAAAAATTGGAGATTCCTTTACAAATATGCTCTTAT

GTGCTATTATTTAAGTATCTATTTAAAAGGAGTTAATAAATATGCGGCAAGGTATTCTTA

AATAAACTGTCAATTTGATAGTGGGAACAAATAATTGGATGTCCTTTTTTAGGAGGGCTT

AGTTTTTGTACCCAGTTTAAGAATACCTTTATCATGTGATTCTAAAGTATCCGGAGAATA

TCTGTATGCTTTGTATGCCTATGGTTATGCATNNNNNCACACACTTAATTAATTAAGTGT

GTGNNNNNATGCCCTTTTGGGTTTTTGAATGGAGGAAAATCACATGAAAATTATTAATAT

TGGAGTTTTAGCTCATGTTGATGCAGGAAAAACTACCTTAACAGAAAGCTTATTATATAA

CAGTGGAGCGATTACAGAATTAGGAAGCGTGGACAAAGGTACAACGAGGACGGATAATAC

GCTTTTAGAACGTCAGAGAGGAATTACAATTCAGACAGGAATAACCTCTTTTCAGTGGGA

AAATACGAAGGTGAACATCATAGACACGCCAGGACATATGGATTTCTTAGCAGAAGTATA

TCGTTCATTATCAGTTTTAGATGGGGCAATTCTACTGATTTCTGCAAAAGATGGCGTACA

AGCACAAACTCGTATATTATTTCATGCACTTAGGAAAATGGGGATTCCCACAATCTTTTT

TATCAATAAGATTGACCAAAATGGAATTGATTTATCAACGGTTTATCAGGATATTAAAGA

GAAACTTTCTGCCGAAATTGTAATCAAACAGAAGGTAGAACTGTATCCTAATATGTGTGT

GACGAACTTTACCGAATCTGAACAATGGGATACGGTAATAGAGGGAAACGATGACCTTTT

AGAGAAATATATGTCCGGTAAATCATTAGAAGCATTGGAACTCGAACAAGAGGAAAGCAT

AAGATTTCAGAATTGTTCTCTGTTCCCTCTTTATCATGGAAGTGCAAAAAGTAATATAGG

GATTGATAACCTTATAGAAGTGATTACGAATAAATTTTATTCATCAACACATCGAGGTCA

GTCTGAACTTTGCGGAAAAGTTTTCAAAATTGAGTATTCGGAAAAAAGACAGCGTCTTGC

ATATATACGTCTTTATAGTGGCGTACTGCATTTGCGAGATTCGGTTAGAATATCGGAAAA

GGAAAAATAAAAATTACAGAAATGTATACTTCAATAAATGGTGAATTATGTAAAATCGAT

AAGGCTTATTCCGGGGAAATTGTTATTTTGCAGAATGAGTTTTTGAAGTTAAATAGTGTT

CTTGGAGATACAAAGCTATTGCCACAGAGAGAGAGAATTGAAAATCCCCTCCCTCTGCTG

CAAACGACTGTTGAACCGAGCAAACCTCAACAAAGGGAAATGTTACTTGATGCACTTTTA

GAAATCTCCGACAGTGACCCGCTTCTGCGATATTATGTGGATTCTGCGACACATGAAATC

ATACTTTCTTTCTTAGGGAAAGTACAAATGGAAGTGACTTGTGCTCTGCTGCAAGAAAAG

TATCATGTGGAGATAGAAATAAAAGAGCCTACAGTCATTTATATGGAAAGACCGTTAAAA

AAAGCAGAGTATACCATTCACATCGAAGTGCCGCCAAATCCTTTCTGGGCTTCCATTGGT

TTATCTGTATCACCGCTTCCGTTGGGAAGTGGAATGCAGTATGAGAGCTCGGTTTCTCTT

GGATACTTAAATCAATCATTTCAAAATGCAGTTATGGAAGGGATACGCTATGGTTGCGAA

CAAGGATTATATGGTTGGAATGTGACGGACTGTAAAATCTGTTTTAAGTATGGCTTATAC

TATAGCCCTGTTAGTACCCCAGCAGATTTTCGGATGCTTGCTCCTATTGTATTGGAACAA

GTCTTAAAAAAAGCTGGAACAGAATTGTTAGAGCCATATCTTAGTTTTAAAATTTATGCG

CCACAGGAATATCTTTCACGAGCATACAACGATGCTCCTAAATATTGTGCGAACATCGTA

GACACTCAATTGAAAAATAATGAGGTCATTCTTAGTGGAGAAATCCCTGCTCGGTGTATT

CAAGAATATCGTAGTGATTTAACTTTCTTTACAAATGGACGTAGTGTTTGTTTAACAGAG

TTAAAAGGGTACCATGTTACTACCGGTGAACCTGTTTGCCAGCCCCGTCGTCCAAATAGT

CGGATAGATAAAGTACGATATATGTTCAATAAAATAACTTAGTGTATTTTATGTTGTTAT

ATAAATATGGTTTCTTGTTAAATAAGATGAAATATTTTTTAATAAAGATTTGAATTAAAG

TGTAAAGGAGGAGATAGTTATTATAAACTACAAGTGGATATTGTGTGCTGAGAGCTTTCT

TCTATACTAATAGACGAAAGGGTGTGAAAATGATTTTTAAATGATACTGTGGAACGGAAC

AGTAGCCCTAGTATTGACTACTGTCGTTTCTATTCATATTGGCTATTCTAGGACTGAGAT

GAAAAAATCTATAAATGCTCAGAATAAAATTGAACCCGCAAATCTCCCCAAAACAATGGT

GAGTCATGTACTTGTATTATTCCGAAAAAATACACCTCTGGTGCAGTGAGACAAATTGGT

GTATCTTATAGTGGCTTCGTAGATGAAAGCTATACTCTACTATCACTCTTTGATGATGTA

GAACAAATTGAAAAAGATAATAGACTTCAGACAGCTATTGATGTTGTCAGAGAACAGTTT

GGTTTTTTAGCCATACAAAAAGGAACCGTCCTAACTGAAGGTTCCAGAAATATTGAACGC

AGTAAACTTATCGGTGGTCATTCCGCGGGTGGATTGGAGGGATTAAAATGAAACAAGAAA

AAATACAGTACAATTTTCAGAAATCCGTAGCAAAGGATGTAATGATATTGAAATGCTTGA

AAGATTTTTACATGGAATCGTTGAAACAGCAACTTCAAAACTTCGTCAGAGAAAACTCAA

AACAACTGAAATATCGATACGACTAGTACATGCTAAATCTGAAAACCGATTACCATTGGA

ATTTACATTTAGCATTAAGCCAACAAGCTCATCTGTGATAATCTATACTGAGGTAATCAA

TCGCTTTAAAGAATGTTACACAGGTGGGGGAATTCAAGGTTTTACGATTCAATTTGATAA

AAATACCCTTGCCTCTGCATAGAAAGGATTTGATATGATTGACCGTTCATATTTACCATT

TCAATCAGCAAGAGAGTACCAGGATACAAAGATGCAAAAATGGATGGGCTTTTTCCTATC

TGAACATGCATCAGCACTCTCTGATGATACAAACAAAGTAACGTACATGTCTGACTTATC

ACTAGAGAAGAAATTATTACTCCTCAGTCAAGTATACGCCGGGCAGCTACGCACACGCAT

TCAAGTGATTGAAAAAACAAGCGTGTTTCCTACACTGGAACAATACCAAGTCTGACCAAA

GATTTCATTTTGATAAAAACTACAACAGGTCACATCAATTTGAAATTAAAAGACATTATT

AGTATTGAACTTGTCGAGGAGGTGCTCTATGAATCAGCTTGAGTTTCAGCGTAATCACCT

ACAAATGGACTATTATAGCGAGAGCTACCAAGATTTTGAACGTGACTTCTACCGCTACTC

TAACATGAATATTCCATTGACCTTCCTAACTGATGATATCCTAAAAACAATGGCGACTTC

ACGTAAGAATTACTTTGTCCTCAATAAGGAAAAGTCCAGAGATAACCGCGATCACTTCTT

CATATTTGAAGTAAGTACCGTAGATGAGAATCCGCTAATCTATCATTATACATATAAGAA

AACTACAATATATTTAGCAGAAAAATAGGAGCAGTTCAATTGACTGTTCCTATTTTTAAT

ATTCATAAAATCTAAAGTCTTTATACTCTTTAACAATGGAGTCGCCAACCAGAACAGACT

ATACTGACCAGCGACTACCTTAAATTTAATGTTTCAGATTTATTTTCTTATCTCTAATTT

CATAAACTACATCTGCTACATTTTCGAGTAATCGTTTATCGTGGGTGATAAACACGATAG

TTCCGGTGTACTCCTTCATTAGTATTTCCAAAGCCTCTAAACTTGGTATGTCAAGGAAGT

TACTGGGTTCATCCATTATTAGGATGTTATATCTACCCATGAGCATTTTAGCAAGCAACA

ATTTTATAATTTCTCCACCGCTTAAAACAGATAAACTTTTTCCAATATCGTTCTGTTTGA

ACCCCATAGATGCTAGCACTGAACGAATTTCTGATATATTGTAGTCACAATCCTTCTGCA

TAAACTCCATAACATTCTGATTACTGTTGTACTTGTAACCATTCTGTGCAAAGTAACCTA

TTTTTGCCTTAGGCGAAATAGAAATTCCTTCTTCATGGTTTAAGATCATTTGGATTAAAG

TTGTTTTTCCGATTCCATTACCACCAGTTAACGCCACTTTTGCTCCTAACGGAATTTGAA

AAGATGCATTTTCAAACAGAGCCTTATCCCCAAATACTTTATTAATTTCTGCACCGACTA

TAGGGTATGGATTATGGAGCTCCAATGCTTTACTTTGCCTGAAACGAATTCTGCGAATGC

CTTCCGGAGCTTCTACTTTTCCTAAGGCCGCAATCCTGTGCTCTAGGGTTTTAGCAGCAT

TATACATCTTTTTTCCTTACTTCCTATTGATTTTTGATGAGCTAAACGCCCTCCGTCTTC

AGTACTTTTTTCTTTGAAGAACCTTTTGCCTTCTGTTCTATTTTACGAGCCTGTTTTCGC

TTTTCCTCCGCAGCCCTTTCCAATCGGGCACGTTCCGCAATAAATTGTTCGTATTCTGCA

GCTTGGCTCTTACGTTCTTCCTCTTTCTGACGAAGATAATCAGAATAGTTTCCCCAATAC

TCAGTGATTTTGCCATCTTTCAGTTCCCATATTTTATCTACTATTTCATCAAGAAAATAG

CGGTCATGGCTAATAACTAACAGTGCACCTGTAAAATATTTTAGCTGTCCTATTAGAAAA

TCAATTCCTTCACGGTCTAAATGGCTCGTAGGTTCATCCGCTAAAATACCATGAACCTGT

GCCGATAAGGCCTGTGCTATTTTAAGCCTTGTTTCTTCACCACCGCTCATAGTCTGTATA

TTTAATTGCTCAACACCTAGCTTGCCTACAAGTGCAAAATCTTTTTCCTCCTGCAGAGTT

ACTTCGTCCAACTGGGGAATATAGGCAAGTTCACCCAGACGATTCATTTTACATCCTGGG

GGAGTTAATTCTCCTAAAAGTACCCTGAGTAAAGTGCTTTTTCCAGCACCATTTGCTCCT

ACTAAACCAATACGGTCATAATCATATACTTCTAATTCATTTATATCTAAAACATCGCGT

CCTTTGAATTCCACACGAATGTCTTTTGCTTTTAATATTAATTCCATAACATTTCCTCCT

GTCTATAATCGCATGCTTTCATTTGCTTGTATGCAGGGAAAACCCTGCGATTTTAGCAGG

AAGAGTTACATGAAAATAAGATACATAAATATTCCTCCAATATTGTTTATTTTAAATCTA

ATTTTCTAACCTCAGTTATCATTTGGCAAACTATAGCAATGCCAATAATTAAAATACCTG

ATAGTAAAAACCAATGATTTACACCGATTTTATCAGCAAAGAATCCAGAAAGAATTAACC

CAATTGGCATAGCAAGTGACATGATACTTCCGATCAAAGAAAATACACGTCCTAAATATT

CAGGCTTAATTTTCTCCTGAAAAAGAGCTGTTTGCACACCGCTATAAAATGGCACCGAAA

GCCCCATTATTGCACAGCAAACTACGAATATTACAAATCCATTTGGAGGAAGTATTCCCG

AAACGGCTAAACTGGTCCCCATTATAAAAAATGAACTTGTTATTAGTAATACATGCTTTT

CGAAGCCCCCTAATCTTCCTAATAATAAGCCTCCTGCTAGCATCCCAAATGCAAAGGAAA

TTTCCGTAATAGAAATATGCACAGGCGTTCCATTAAAGTGTTCCATGCTTATTAAAGGAA

ATAGTGCATTGATTGGCATATAAACAAAAGTATATAGTGTTCCTAAGAGTAATAAGGCAA

ACAATCCTTTGTTTTGTCTCAGAACCACAACTCCTTCTTTCATCTCCCTTATGAAATTTG

GTTCTAAACTTTGCACTTGATTACCCAGCTTAGGTATACGTACAATTGCTACCGTAATAG

ATGCAATCACAGCACCCAATACGTCGATGGCAATAATAGCATTTAAATCCCAAACGGAGT

ATAAGAGTGCTGCAACTGCCGGACTAACAATATAGCTTATAGACTGCAAAGACTGACTAT

AGCCTGCGCATTTCGTTAGCTGTTCTTCTGGTACTAAAAGTGGTGTAACCGCATTGAGTG

CTGGGGTATGAAAAGCTGTTCCAATGCTACGGATAAACAATACTATCATAATCATCCAGA

CAGGTAGCTCCATACAGAATGCAACAATAGCAAGCACTGCACCAGCTGCTGCGATAATTA

AATCGGCACCAATCATTATCTTCTTCCTATCATGACGATCCACTAGCACACCAATGGCAG

GTCCCAAAATCGCATAGGGTAAAAAACCTACTAATGAAGCCATAGACAAGACCATCGCAG

ATCCTGTTTTTTCTGTAAGGTAAAAATAATCGCCATTTGCAGGATGGCACTAGTGATTAA

TGATACTGCTTGCCCTGCCCATATTGCATAAAATTTTCGTTTCCAATTGTTGTATTTTTC

CATTTATATTATCTCCTGCATATTATTTTGCTTGAATTTCTATTTTGAATAGCATTCTAG

GCAATAAAAAATGCAGGCCAAACCCCACAATGTGGCTTTTGGTCTGCATACATACAATTT

GGAAACATTCATATTAAAGACATAGTTAAATAAAGGTATAGTTAAATAACCAATATCCTC

ACCGTAACTAATGAATGCTCAATATCGTATAAATAAGCACAACAAAAAAGCCTATCATCG

GGTATAGATTCTGCTTTTTTTATTGCCAGCTTATCTTAAACGCATTGAGGCTGTCATAGT

TTCGGTTCCTCCTACATCTTTGTTTATATCAATTTATAGTATAACACAACAAGATGATAT

GTTCAATATAAAAGTTATGGAATGAGACTCATACTTCCAATTCGATGCCAGATTTAAAGG

ATATGACGAAGTTTTCTTCATAGACTGTAACGCTCTGGATTATCTTCCTTAGTAGCAAGC

GATTAGCTTTCACAAAATCTTCTGTTTGTAGTTTTAAAAATTCATCAGGATTTTCTAACT

CAACCTCAAAATATTTCATTTTACATTCCCTCATTTCATTTATTGATAAATTGAGTTTGC

AAAAAAGAGTGGACAATTTTTGTCTACTCTTAACCTTTAAAATAGTTTTTTTAATCGATT

TGAAGTTGCCTAAATTATTACTTATTCGGTAAAATGAAGTATTGCTTTCAACAGATTTCC

TTCAACTACACTTCACTTGATTCAAACAAGGTGGGTACATTTCTATTCCCACAAACTCCT

TGTCAATGGAAACAAACACGTACCCACAGGGTAAATGGAAATAGAAACTGATAATTTCTA

GCTATCACTTCTACTCATTCCAAAAATTTTCTCACTCTGATACTTACCCACCATAAAGCA

AAAAGCCTTGCAATCAAGGCTTTCATTATCCCTTTCGTTCAAAGGTTTCTAAGCTTTTAC

GAGCAGAGCGACACACTCAGCGGTTCGCTATCTCCGTTCTGTCTGCGTGCTAGCACTTGT

CAATCACGGACAGCTATCGCATGGGCGGAAGTAAATGCTAATCTTCGTCGTTTTACTCCT

TGACTAGCAAACTTACCGCCTCAACATGTCCTGTATGTGGAAATAAAACACGATTAAAGA

TAAGGGAAGATACTGAATTAAAAAAATTCCCCCTCTATTGTCCGAAATGCAGACAAGAAA

ATTTAATTGAAATAAAGCAGTTCAAAGTAACTGTGATTACAGAGCCAGACGCAAAGACGC

AGAGCCGATAAAATGAGATTAATACAATCTCATTTTATCGGCTCTTTCCGTTATGTATGG

ATTCTTTTAATTAGTCTTCGATGTTTCTTGCTTCGTTGATACCGCTGGCTAAAGATTCCA

TTAAGGATAGTTCTTTGTCTGTAAAGCTATCCATGTATTTCTCTATCTGTAATCGTCGGG

TGCTTTTTACCAAGTTATTAGCAGGTAAGAAAAATTCATCAACGGAAACATGAAGTAACG

ATACAAGGTCATAAAGAACTTGTATGCTGGGGTGTTGCCCTTTATTTTCAATATTAGTTA

AGTACCGTGGGTCAATTTCAATCAATGCTCCCACTTGTTCACGAGTTAAACCTCGTTTCA

ATCGAGCTTCTTTAATGGCTAAACCAAAGGCTCTAAAATCATATTTATCTTCTTTTTTAC

GCATAGTAGACCACCTCTATACATTTTATTGTTCCTACTGAATTAAAAACAGGTATAGAA

AAACGTGTTATATGGTTTATAGGTTTATATTTAATAAAAAGCACTACTAAACGCCAATAA

AAAAACCGTTATATGGTAGTGCTATTTACGCTGTTAAAATATTGTATATTACTTCCAAAT

GGCGGTTTGTTGGAGGTCAACGTCGCCATGAAGTACATCATATACAATAAATTTCCTTAC

ATTGGGTTCTTGTCAAAAAAAGTCGTCTATCTGCAATAGATAAGTACGTCCACCAATGTG

GTTTTATAAATCATATAGATAGAATAACAGAAGCATGTAAACAGAGAAATAAATCTGTTT

ATATGCTTTTTTGGCTATTCAGAACTTTTTTACAAAGTTTATTTATCAGTAATGCAACAA

ATCCCCCTTTCACATTGGGACTAAGAGTGAAAGGAGATAAACGAGCAAGGCTCACTTCCT

TTCCTAGACAGAAAGGGGGTGAGAAACATGAAACCATCTTCTTTTCAGACCACAATAGAA

AATCAGTTTGACTATATCTGTAAACGTGCTATGGAAGACGAGCGAAAGAATTATATGCTT

TATCTTTCAAGGATTGCAAAGCGTGAGGTGTCCTTTTCGGATGTTGGCGATTATCTTGTT

AGCCAGTTTGCGACAACAGATAACTATTCAACTGACTTTCAGATTTTTACACTCAATGGG

TTATCAGTAGGCGTTGAAAATGATTTGTTGAGTGAAGCATTACGTGAGTTGCCAGACAAG

AAACGTGAAATTCTACTGCTGTTTTACTTTATGGACATGAGCGATTCAGAAATTGCAGAC

CTGTTGAAATTGAACCGTTCTACTGTCTATCGGCATAGAACCAGTGGACTAGCCTTAATT

AAAAAGTTTATGGAGGAATTTGAAGAATGAAAACACAATATCCTATGATTCCCTTTCCTC

TCATTGTAAAGGCAACAGATGGCGATACCGAAGCGATTAACCAGATTCTACATCATTACA

GAGGGTACATAACGAAGCGTTCCCTACGACTTATGAAAGATGAATATGGCAATCAAAGTA

TGGTCGTTGATGAAGTCTTACGTGGAAGAATGGAAACCAGACTGATTACAAAGATTTTGT

CATTTGAAATTAAGTAATATCCTCTCTCCTTTCGTGGAAGCGTGCTAAACCATTCCACGC

TTCCCGAACAGGGAGGTTTGTTATTCCACCAAAGCATATTGAGCTTTCAATGTGTTTTGA

TAGGCTAACGAGCCATTGTTCTTTGAAAACTGAATAAAAGTAATCGAATACGTTTCGATA

AGAAAAGAGCCAACGGAACTAACCGCCATGACCTATCTTATAAAGATAGCGAGCGATTCA

TGTTAGTGATCCGAGAAGCAATCTTTAGCAGGATTGCCTGCAACGACATTCTTATCGTGA

TAATGATACTCCCATACAGTCAATAGTCCGAGCGTGATAAAACCGTCGCAGGCAATGAGT

ATGGCTACATGAGAACCATGCAGGGGTGGAACTCCCGTGAGCTTTGCTAAAGCTGTTCGA

TTGCTGGTAAAACAACTTTTATGAAATCCAAATAAGTGATTTGGAAAGGAGGATTTTATG

AAGCAGACTGACATTCCTATTTGGGAACGTTATACCCTAACCATTGAAGAAGCGTCAAAA

TATTTTCGTATTGGCGAAAACAAGCTACGACGCTTGGCAGAGGAAAATAAAAATGCAAAT

TGGCTGATTATGAATGGCAATCGTATTCAGATTAAACGAAAACAATTTGAAAAAATTATA

GATACATTGGACGCAATCTAGCGTCGCCAAAGGGTCTTGTATATGATAAAATAGTATTAA

GTCGTATCAAGGCTCTTTCCATAAAGGAAAGGAGCAAATGCCATGTCAGAAAAAAGACGT

GACAATAAAGGTCGAATCTTAAAGACTGGAGAGAGCCAACGAAAAGACGGAAGATACTTA

TACAAATATATAGATTCATTTGGAGAACCGCAATTTGTTTACTCGTGGAAACTTGTGGCT

ACAGACCGAGTACCAGCAGGAAAGCGTGATTGTATCTCACTTAGAGAGAAAATCGCAGAG

TTACAGAAAGACATTCATGATGGTATTGATGTTGTAGGAAAGAAAATGACACTCTGCCAG

CTTTACGCAAAACAGAACGCTCAAAGACCAAAGGTTAGAAAAAACACTGAAACTGGACGC

AAATATCTTATGGATATTTTGAAGAAAGACAAGTTAGGTGTAAGAAGTATTGACAGTATT

AAGCCATCAGACGCTAAAGAATGGGCTATTAGAATGAGTGAAAATGGTTATGCTTATCAA

ACCATCAATAACTACAAACGTTCTTTAAAGGCTTCATTCTATATTGCTATACAAGATGAT

TGTGTTCGGAAGAATCCATTTGACTTTCAACTGAAAGCAGTTCTTGATGATGATACTGTC

CCTAAGACCGTACTAACAGAAGAACAGGAAGAAAAACTGTTAGCCTTTGCAAAAGCTGAT

AAAACCTACAGCAAAAATTATGATGAAATTCTGATACTCTTAAAAACAGGTCTTCGTATT

TCAGAGTTTGGTGGTTTGACACTTCCAGATTTAGATTTTGAGAATCGTCTTGTCAATATA

GACCATCAGCTATTGAGAGATACTGAAATTGGGTACTACATTGAAACACCAAAGACCAAA

AGTGGCGAACGTCAAGTTCCTATGGTTGAAGAAGCCTATCAAGCATTTAAGCGAGTGTTA

GCGAATCGAAAGAATGATAAGCGTGTTGAGATTGATGGATATAGTGATTTCCTCTTTCTT

AATAGAAAGAACTATCCAAAAGTGGCAAGTGATTACAACGGCATGATGAAAGGTCTTGTT

AAGAAATACAATAAGTATAACGAGGATAAATTGCCACACATCACTCCACATAGTTTGCGA

CATACATTCTGTACCAACTATGCAAATGCAGGAATGAATCCAAAGGCATTACAGTACATT

ATGGGACATGCTAATATAGCCATGACGCTGAACTATTACGCACATGCAACATTCGATTCT

GCAATGGCAGAAATGAAACGCTTGAATAAAGAGAAGCAACAGGAGCGTCTTGTTGCTTAG

TAGTACAAATGAATTTACTACTTATTTACCACTTCTGACAGCTAAGACATGAGGAAATAT

GCAAAGAAACGTGAAGTATCTTCCTACAGTAAAAATACTCGAAAGCACATAGAATAAGGC

TTTACGAGCATTTAAGAAAATATAAAAAGATAATTAGAAATTTATACTTTGTTTATTTCA

AAATTAATCCATGCTTAAGACAGACCAGCTATTATTAAAGAAAAATAAGACATAGACTGA

TAGGTTCCACACCAAATGTAGCCCCATACTGCCCCATAAGTCCGATTTGTAGCGCACAAG

CCCTAAAAACATCCCAAGTGAAACATACAAACACCAAGCTAAAATGGTTCCTGGATGATG

AGCTAAGACAAATAAAATACTTGTCAAAGCAACTCGAATATCTAATTTTCTAACCAAGTT

CCATAAAATTTCTCGATACAGAAATTCTTCAACCATACTCGCATTGATTAATAACAATAA

AAATGAAAACCAAGGGACTTGATGTTGAAGGCCAATTAAGTTTGCTTGATTCGTAGTTCC

TTGAGCATGGATTAGGCTAAAACATAGATTTATAATCAGTAGGCTAACGAATCCAATACC

AAGCCATTTCATCCTAGTTTTCATATTGACCTTGACAACTTGTTTTCGTTGACCATACAT

CCATAAAAAAGAAATGAGCGACGCACAATAGAGAATCTGTAGTATGGTTAACTCACTAAT

ACAAAGCAATTTCAGTAAGTATAGGGATACCAATAAGACATTTACTTGTTGGAATATATA

AACTGGAATTATTCTTTTCATAGTTACCTCCGAAATAAATCTTCATAATCTAAATCTAAT

ACCTGCACAATCCTTTCTACCCATGGACTTTGAGGCATTCGTTGTTCCATCTTGTAGTGG

CGTATCTTTTGATACAAACGATTCAATTCACTTGGATAGTGAAACTCTCCCGCAAACATT

TTTCTGGTTAACTCAATCCAGCTGATATTCCTTTCAGCCAAAATAATGGACAAGTTCTCC

CAAAATCGTTCTGCCATATTGATTCTCCTTTAGTTAGATAAATAATGTGTTTGTGCCATG

TAAATCAATTGTTTCGTATCTCTTGGCAATAAAGCTCTAGCCTCCTCCAGATTCAAATTT

GGATAAACTCGCTTATTTGAAACCGCAAGAGGAAGTCTGATGGTTAGTTCAGGATTTTTT

AATATCATTTCGATGAAATCTGTTAATTTTAGGTTATCTCGATTCTTAAAACGTAATAAA

TTGGGAGATAAAAACTCGAAACAATCTGAAGAATAGCTCATCATCTCAATTAATTTATCC

TTTGTCATTTCAGAAATAGAATGAGGAGTGACCTCTATGCCATACTTTTGAAAGAAATCC

AAAAGAAGTTGATTTCTATGACAATTTTTACTTAGATAGAGATCAATCATGGGAGACCTC

CCAAAGATTCGGTTCCATTTGATATTCTGAGACGATTAAGGAATCTAATAAATTTGAGAA

GTTAATCGGTTTCTTGTCTTCATCATAAGCTTTTACAGTTACTTGGGTTGTAAGTATTCC

CTCTTTTCCCTCGGCTCGATAGCCTTGTCCATATAAAACAAAAACAAGATTTTGATGATC

ATCTACAAAGGCATCAACCCCATTCTTTATGTCTTGACTTTCAAGGAATTCCATCACGTT

TTGAAGATAGGACTCGTAAAATAGTGGGTAGTTGTGTTTTTATGGTAATCATCTAAAAAT

GTTACTTCAAACTCACATGGATAATTGGGTATCAAAAATAATTGTTCATCCAGCTGTTTG

ATTTCTGCATCATGTAATTCTGTTTCTAATTCATCACAATCTAGTATTGATTCTTTATTT

AATGCTTTCATCTTTTTCCTCTATTTCTTTTAATTTCTTTGCGATTGCGGCAATCACAGG

AACGGTTACACTATTACCAGCTTGTTTATAGAGCTGACTATTACTAGAGACTCTTCTAGC

AGCTTCAAAAGCCCAATCAGGAAAGCCCTGCAAGCGAAAACACTCTTTAGGAGTGATTCG

TCGTATCCTCAGACGGTAAAATTGTCCATCTATTAATATACCGGCTACATGATAAACTTG

TTTATCTTCTCCTTCATAACTAGCAACTACTACTCCCATTTGTCCACTAGTTGTTAACGT

ATTAGCTATACCTTTTCCAACTCTACCACGACGATACTGAGAACTTGGTCTTTCCAAATT

GATTGAATCTCCTATTTCTGCTTGAGCGTATCCTTTTTTCGTTGCTTCCCGGACTTTTAG

AAATTGAATTGGTTCTGGAATCAGTATTTTGGGGATTTTATCTCCACCTTGCATCGTAGT

CAGTGTTGGAGATAACCCCTCACTTCCATAGACACGACCGGTCTCCTTAAAGCTAGTCGG

TAAATCTCCAACAACGACAATGCCATGACGATCCTGAGTATTTAAAGTAAACATTGGCTC

TTGGTTTTCCTTAAAGCGTCTACCATTTTGTCTCTTGTCTAATCTATCTGGTGTCATACA

AGGAATCGCAATCTTAAATCCTTCTCCTTTCCCACGAACTAAGGTTGGCGCAAGACCTTC

TGAATAATAAACTTTACCGCTCATTCCACTTTTTGATGGATTCAAATTTCCTAATGTTTT

TAAAGTCTCAGAGTTAGTTGCTTGACCTTCTCGTCTGAAAGGAAATAAGAGTCTGATACC

TTTCTTTCTAGAATGTCCGATAATAAACACCCTCTCTCTGTTTTGGGGAACGCCAAAATC

CTTACTGTTAAGCACCTGCCATTCAACATCAAACCCCAACTCATCAAGTGTGGTAAGGAT

TGTGGTGAACGTCCGTCCCTTATCGTGATTGAGTAGACCTTTAACATTTTCAAGAAAAAG

AAAACGTGGTTGGATTTGTTTGGCCGCCCGAGCAATTTCAAAGAACAAAGTTCCTCTAAT

ATCTTCAAATCCCAATCGTCTTCCTGCGATTGAAAATGCTTGACAAGGAAATCCCCCACA

GATGATATCGACTTTCCCTCTAAGTTTTTAAATTCGTCATCTGAAACATCTCGTATGTCA

TGAAATTCTATTTCTCCTTCCGTTTGAAAAATGGACTTATAAGATTCTCTAGCAAATTTA

TCAATCTCACAAAATCCAATACACTCGTGTCCTACACTTTCCATTCCAAGTCGAAAACCA

CCGATACCTGAAAATAAATCAATAAATCTCATTTCTTGATTACCCCTTTCTGATCAACTA

TCAATGTCCAAAGTGTCACACCACTTGAAATGACAATGAGCATAACTAATATCCACTCGA

TTGGCGACATTTAACCACCTCCTTTTCTAACACGATTATTCCAAACAGAATACAAGCCGT

TAAATACAAATTCAGCAAGTACTTCCGCTCTTCTAACAAAGCGAACATTGTCAAGAGCAT

ACTGATAGATTCTCTCAAAATCAGTCATAGCAATTTCACTGGCTGTTTCAGAAAAACCTT

CTCGTCTAAATTGATCTTGTACCAATCCCCAAATATAATCTTGATCATATTTTGTGACCT

TTTCTACTTTTCTTTTCAAGATAGGTTGAGTATACCTCTCCTCCTCATCCTCACTAAATA

AAGAATCAGTCTCACTATATTTAGTCTCACTAACTTCAGTCTCACTAGGGGCTGAATGTG

AGACGGGGGCAGTTTCATTTTCAACCTGCCCTATTTTTTTTTAACACTAGGCCTGTTTGA

ACTAGCTACTGGGGTAGAAGACAACTCCCCTAAATAAATCTTATTAGCTAGCCTCCCTTT

CTCACTGGAAGACTGTTGGACTTCATCAATTAAGTCATATTCTTTAAGAATTTTTTTATA

GACAGTAATTTTGACTTCGAACAACCTAACAGTTTCATCAGTTTAGAGTTAGAAAATACT

AAATAAACCGCTCCTTCTTCATCTATCCAACCACGACTGAGAGATAATTCTAAACGATCT

TTTAAAATAGAATAAGCCACCTTTACTTCTAGTTTCATATCCATATATTTCTCATCCTCA

AAAAGAATTTTAGGTAATTTATAATACCGTTCTGAAGTTTGATATTGATTTGCGGTAATT

CGTTTCATAGCGCTCCTCCAAGTTCTTTGAGTATTAAATCTCCACTTGATTCCAATCGAA

CCAAGCCACTTTTTTCTAATTCAGCCATAAGAGAGATGGCTTCAACAATGTCAATTCCCA

TTTCACGTACTAAAAATGAAATGACAATATAGCGTGATGATTGTAATTCTTTTGTCATTT

TTCCTCCAGAAAATAAAAAAGGAGAACAATATTCAACTGTTCTCCAAAATATTTTTATTA

AAAAGAAAAACCCTGCCAAATTAATTTTTGGTAGGGTTTTTGGCAGGAAACTAAATTAAT

TTATCAGTTTCTA

>ICESpnGA05245

AACCATTGAATAGTAGCCATTGCATTTTTTTCCTTTCGTAGCAAGGGTTTAGCACCCCTC

ATTTATTTTACTATCGTCTAAACACCAAGCGAACACCAAAACTACCACGAAATGGAAAAA

CCTTTGATTTGATTCCCGCTTAATTTCACAATCTTTATATCAAACTGTGGGTGATATTTG

ACAATATCTTTTTTGATTTTTAACAGTAAATTTGAAACAATATGTTTGGGTGAGTAACGT

GAACCAAGGTGTAATAAGTCTTTAAATTCATCAACACTTAACTCTACTTTATTGTTATCA

TCACAAGTTTCAATGAATTGTTCAATCATTCTCGAATATTTACAGGTGTAACTCTTCAAT

TCTTTAAAGTGAAAATTGTGCTTTTCTACAAATTGATTTAAAGCAGTTACAGTATTTTCT

TGTGAACGACTGATGTTATGGGTGTAGCCCATCGTGGTTTCAAAATTCGCATGACCAACT

CTAGTCATAATATCCTTTACAGCTATGTACATTTCATTACTTTGAAGATAACTAATGTGC

ATATGTCTGAAAGAATGAGGGGTCACATGCTTGACCCATTTAAAACCATAATCCTTCTGA

CAATTTTTTAACAACTTCTCCTCAACCCTCTTCAAAACTTGACGAAATGAGTGAGAAGTT

ATCGGAGAACCATACTCAGTTTGAAATACACTATCACACTTTTTGAAAGCAGGACTAGGA

TTTTGTTCCATATAGGCTTCAAATTCCTGATTTCTTTTTATAGCTCTCTGGATTGCTTCA

CTCGCTGCTATTGGGAGGGCTACCTCTCTATCAGCATTGACGGTCTTAGTCGTATCAAAA

TAAAATTCATCAACTTTTAAATCATGATATTGGAGAGCCTTATCAATATGTAAAATCCCC

TTATCAAAATCAACATCTGATGGTAAAAAAGCAGCCTCTCCAATCCGAATCCCTGTCAGT

AATAATACAATAGCAAGGTCATAATAGCTAGCATTTCTACACTGCCCTAAAACATCAAAA

AAGGCTTGCAATTCATGAATTTCAAGAAATTTAGCATCAAGCCTTTTTCTTGCTTCTCTC

TTTTTCTCCAGAGGCACATCTATTTTAACAGCGGTCATTGGAGAGTACTTAATGACATTG

TATAGCACACCGTGATTAAATATCTTGTTACACGTACTTTTTATATGTGTCATTGTTGAA

GGTGAAGCGTCGTATTTATCCAGATATTTATTAAGACAATTTTTCATCAGTAGTGGTGTA

ATTCGATCGAGTAAAAATTCATCACCGATAATTTTACCAAGCCGTTCTATCACCAACTCT

TCCCTTTTTATTGTCTGGGGCTTGACAGATACGCACCAAGTTTGAAACCAACTCTCTTTT

AACTCTCCAAAAGTTGTAATCATTTCAGGATTGTACTGACTTTCAAGGGAAGAGGTCAGT

TTTTCTATTTTATCCATTACCTCTCTTTCAGCTTGCTTTCGTGCTCTACTAGTATTTCTA

GTAGAGCTGACAGTTACTGTTCTCCACTTATCAGTAAGTGGATCTTTACATTTTTCAACC

ACTTGATACAAGGGTTGTCCTTTTGAATTTGTTTTAGTTACATAATACATAATTTAATTC

CTCGAAAAGAAATAAATCATTGTTGTATAGAATAAATAACTACTCTATATTATACCATGA

CTTCCTTATTTTGTCCGCTTTTGTTTCCATCTCAAAAAGCATTCGAATCCCTCAAGATTT

ATAAACACCAGCTTATGTGTAGGATTGATGACGTATAAAGAAAATCTCTTATTTTCTCTC

ATTTCTTTGATCCATCTGTTAAGAGTGTAGAGATTCAAACCTTCCCATCGTTTCAGTATG

CTTTTCTTATCCGCCCACTCAGCTACCGAATTTTCAACAGATACGTATTTTATCTCCATA

TTATCATCCTTTCGAATAATTTCTTCTCCCTCCATGGCATGACTGATGGGGTCATCATAA

GAATCTCACTTACTGCTCTTTTACGACGGCAGCCTGAACGTTCAGAAGTATCATTGTATA

TCATTTGCATCATGGCAGATAGAGTATCGCTCTATTTTATTGGTGATTGTCCCCGCTCCT

ATCTTGGCGAATCAAGCAAAACTGCTCCACAAATGAGGAAAGTACCATTTTAGGCTATTC

AGTTGTCAAAGGGCAAATAAAAAATCTCACTCATTTTCAAACTCTATGAAATTGAGGAGA

TTATTTTCTAAAGCGAATTTTATCAGGAAAATCACAGTTAATCCCAATAAATAGTCTGTA

CTGACGTCATAGAAATCTGAGAGTTTAACCAATACATCAGCCGTCAACGCATGCTCACCC

CGTTCAATTTTGGCATAAGCTGAATTTGTAAACGAAAGTATTGTAGCTATTTGCTTTTGG

GGCAGATCATGATCCTCCCTCAAATCTCTCAAACGTCTGTACATCTAGCTTTCTCCTAAG

AAAAAGTATAGACCATATATTGTAACTATTTAGGATATTGTCCAAATCGGACAACAGGAA

TTTTACCATCCTTTATATAGACTTTTATTTTTATGTATCAAGTTTTTTAAATCAAAACCA

CCAGTGTGAACTGGTGGTTTGTTCTGCGGCTATAAGCCGCTTCTACCGGCCAGGGCCAAA

GGCCCACCGAAATAGCTTCCTCGCGCACCACTTTCCCGAGCAGGTGCTAAAGCACCTTAG

TTACTTCCTCTTATTTATTTCGCCAGTAAACGGATCTACTGACTCGAATAACGTGAGCTG

GTCTGCTACTCTGTCTTCTTGTAATTGATTCTGAATATATTCAGCTATCACTTTCTGATT

ACGGCCTACCGTATCTACATAATAGCCTCTACACCAAAACTTGCGATTGCCATATTTGTA

TTTTAAATTCGCATGCTTATCAAAAATCATCAAACTGCTCTTGCCCTTTAAATAGCCCAT

AAAGGACAAAACACTAAGTTTCGGAGGAATACTGATAAGCATGTGAATATGGTCTGAACA

AGCATTCGCTTCATGGATTATTACACCCTTACGCTCACATAAGTCACGTATGATTCTTCC

GATACTAGCTTTGTATCTGCCATAAATGATTTGACGACGATATTTGGGTGCAAAAACAAT

ATGATATTTACAATTCCATGTGGTATGTGATAAACTTTGATTATCCTCTCTCATGAGGTA

CCTCCTGTATGATATGTTGTAGTGGCGGAGAAACCACTTCTATCTTATCATTTTAGGAGG

TTCTTTTTGTTACCACGCTAAAAGCTCTATGGAACCACTAGCATAGCTAGTGGTTTTCGG

GAGACAACAAAAAAGTTGGATGACCCAACTTTTATCTTTCTATCAGTATCAATTTTATCT

TTGGTATAATAAAAGATACAGATATAAGGACAAGTAATGATGTTAACAATAAGAAAACTA

ATTCCAAAGTTGTTGAATTTTTAAAATAAAAGTTTCTCAAAATAGTTAATTGATAAGTTA

AGGGATTTAACTGACCAATCCATCTCACAATACTAGGAGCAGAATCTATTAAGTAGAAAA

CAGGAGCTGTAAAACCAAGCGGTGTTAGTACAAAACGTATTACAATATCACGTCTTCTGT

AATCATTGATAAACATTGTAAGTAAAATTGCAAGGGAGTCCCAAAATAATAAAACAACAA

TAGATAACAAAACTGTATAAAAAAATCTATCCATTGCGATATTTATCTCAAACGCTAACG

TAATTATATATATAATAATTTCTTGAACTATCAACCCTAATATAGAATAGATACTCATCC

CTAAAATATAATATAAAGGACGAACTCCACTGCATAACTTAAGAGCAAGTAGCCCATATT

TTTTATCTATTGTCACCCTATAAATAACTTGAGTCATCTGTCCGATTATCAATAAAGCTA

TAACACCAATGATTGTATATTCTGCATAAGAGGTTTTTATATCATTAAACTGAATGTTCT

TTATCGAAGAATTTAATCCTATTATTATAAAAACAACATATAAGATTGGTTGTAGAAACT

GAGAAATAATTAAACCTTTACTTCTAAAAAAAGCAATAATCTCATTTTTAGCAATAATCG

CTAGACCTCTTAAACCTAAGTCCGATTTGTGCCTTAATTGATCAGCCTTCATTTTTCTCC

TCCTATTCTTTGCAAATAACTTTCTTGTAAGGTTAATTTACTTGTTGAAAAGTTTTTAAT

TTCACATGCTTTTCCTACCTCATTGATAACATCTAAGATATTTTCTTCTATAGGGACTTC

TATTGTAAAACTATCATTATCTTCGATGTGAACTTTAAATCTAAAATTTTCTAAAAATTC

AATTTGATATCTAGAAATTCTATTCTGCATTGAAAAATTTAATTTGATAGTTGAATTATC

TACAAAGTCACGCATATCACCAAAAAATGATATGGAGCCATTTTGTAAAAAAAGTATTTT

TTTACAAAACTTTTCGAGTAGATTTATGTCATGTGAAGATATGATAATAGTTTTTCCTTC

CAAACTCTTATCTTTTAAATACATTAAAAATTTTTCGGCAGATTCAGTATCTAAACCAAC

GGTAGGTTCATCTAAAATATAAAAATCTGGATTATGAGCTATTGCTCTAGCAATCTGGAC

GCGTTGCAGTTGTCCACCTGACAATGTTTCTACCAAATTATTTTTTTTATCAGCTAACCC

AACAATTTCTAAGGCTATTTGACACAACTTCTCAGCATTTTTCCCAAACTTTCCAGCAAG

GTTCAGCCCCAATATTACATTGTCCTTCACAGTAGTATAAAAATCAATTACTGTGGTTTG

AGGAGTAAAGCCTAAATGAGCAAAAGGTTGAGAACTAACTATCAAATCTTTATCACAATA

CTCTATTTCTCCTAAATAAATCTCTTGAAAACCTACAATAGAATTAATTAACGTAGATTT

ACCTGCACCATTTGAACCTAATAAGCCAATGAAATCTCCCTTATTTATTTGTAAAGTTAT

ATCTTTTAAAGCTATTACGTCATCATAACGAACACTTACATTATTCATTTTCAAGAAACA

ATTCATAACACCCCTACCTTTATTTTAATACCTCTATAAAATCTTTTTTTAATTCAGAAA

AAGTATCATATTTATAATTGAACGCTTTTTTATAAATATCATAAAGCCAATCTGATTTAT

CAAGTGTGTCTAAGCGACGCGAATTAAAATTCATTGCATACTCCATCGCTTCTAAAAAAC

TCATTTTTGAAAAGACGTTAAAATCATCTAAATTCTGACTCCAATATAATAACAAAACCA

ATCCCATAATATCCTCTGGTTGATTATTCAATAAATTTAAGTTGGTTTCATAAAACCCTG

GAGTTCCAAATAGAGGCAACTTTTTTTCTTCAATTTGAGTTTCTTTCCTTAGGGCATGCT

CAAAGTCTATAATATAAATATTATTTCTATTATCAATAAGTATATTATTAAATGATAAAT

CTCTATAGGAAAGATTATATTTGGAGTTTATTATCTCCATATAATCAATTAATGTTAAAA

ACCAATCATACGAGCCACTAACCATATTATACTCGCTTAATTTATCTGCAATAATAAACT

CAAATTCCACAAAATACGAATTCTTTATGTAAAAATCGTTAAAAACTTTTGGAGTAAATT

CCTCCTTTTCCAATTCTACTAATATTTCTCTTTCATTTATTAAACGATTCACAGAATCTC

TATTTGTAAAATCAACCAACGATAAATCACTAGCTTCTTTTAATAAAGAATAAACTCGCT

TTTGAGTATTAAATACTTTATAAACTCCACCTTTGGCATTTTTAGAAATCACTTCCAAAA

TAATATATTGATCAGGAATAGTGTTATATCTTGGAATATAGTAATCCCTTATTGGAACAT

TCACATTTGAAGGGATTTTCTTATCTCTTTTATCCTTGAAAGTGCTATCTTTTACGAACT

CCCCATATCTGTAATATACAACCTCGCTAAGTTGAAATCTGAAATCTGATGGTATGTTTA

CACCCTTTACACCTTTATACAATATTTCTAATTTGTGTAACAAACGTTGAAACTCTTTAT

TATCTTTTGGATAAATTGTAATGAATTTCCCGACTTGTGAATAACCATTAAGCCCTGTAT

TTTGCAAAGAAAGTTCTTTAATGCTAACCAAAATTTTGAAATTTATCTTCTTCTCTCTAG

AAAATATAAAATCAAAGAATTTTTTAGCAACCAAATTAGCATTTAATATTGAGGCGCTCA

GGTGTATTTTAAATCCCTTAGATTGGGTGATATTAGACGGCAAATTATATAACCAATGTT

CATCACTAAAATTATCACTAATTTTATATTCTAATAATAAATTATGGTATGCGTCTTCTA

TTTCAGTTTCATAGTCCAAATAGTTTAAATACTTTTCGTAATTCATATTAAGAAATCTTC

TCCATAAATTTTTAGACCATCATTTAAAGCCAAACAATTTAAAGCGTGATAATAAATGTT

GATAATCAATGTAACTTTCAGTCCTCTATTTTGTAATTCCTTCACCAATAATTTTATGCT

ATATCTATTTTCTCGAGGCAATTTATAGGACTTCAAGATAAAACCATAAAAGAGATAAGT

ATTATAATCTGACAATCCAGTTTCAGAATAATTTTTTAGAAAAATATCTAGTGATTCTGA

TAATTCATCCGGAATAATTCTTTTAACATCGTATTTATTTTTCATATCGGCCACTCTTCC

TTAAAAAGCTCACAATAAAATTTTAAATTTCTATACAACAATCCGAGAGTAGTCTCACAA

TTTGAACATTTCACATCACTCTTAATATATAAAAAATGAATTAATCAGAAACCTCTGACT

AAGATTTCCTAATTAATTCACTTTCTATATCATAGTAAGGAATTCTATTATCCCTAATTG

AAAATTGAAATTTTATGTTTTATATATTAACAATTATGCGGATTGTAAATCTTGTCTAAC

AAAATGGCAAGTGCTACTATGTGCCCCAGAAGGCGATGCAACGCTATTTTGAATTGAAAG

AGCATAATCATCCATATCATTTAAGTCACGGATTAGCAATGCTTCCTTCTCTCTTCCGAC

AATTCCAAATTTTCTAATTACCTTTTCAGGATTATCAAAAAATTCTCCAACAACTTCCAT

ATTTCCTTGAAGTTCATTCAAGAAAGCTTTCATTTGACTACTCATTATATAGCTCCTTTT

CTATTACTTTATTTGGAATCAAAACTTACTTGTACATTGGAAACACCTCTATTCTACGCT

TTCATATTGCTGCATGACACTTTCAAAATCAAATTGCTAAAAATAATTTTTTAAAGCTTA

ATTTAGATTTAATTACATATATCTCAAAAAATTGTTTTGAAATTAGTAAATTAAAATAGG

TTTCTGTACTTATAGGAACTAGTTATAAAAACTTCGCCCATCATAAAATATCTATTTAAG

TAAAACAAAAATTTTATAATTTTTTGATTTTTAAGTGACTATAATCTCCTATCTATAAAT

ACCATTCGCAGGACCTGGATCAATCCCTCTAGCCATCTTATGAACTTGAGTTCCTCCAGC

AGTCCCTAATAGGATTAATCCTATGACTAACAAAGCAACAATTTTCCTAAACAAACTTCT

CATATACCTCTCCTTTCGTTTTCTTGCATTTATTTTATCACTTCAAAATTAGCTTGTCAA

GTATTTTTATAATCTTTTTTTATTTTTTTTATTTAATACTTTTTTGTATTCAAATTAGAT

TTAAAAAACGATGCTCACTAGAACACCGTATTCCTTATTCTGTTATATCTGCAAAATAAT

GTTCGACTTTCAAAGCCATTGACATGTTTTCCTCAAGTTTGTAGAGAAAATGAGCAGTCT

CAAAATATTCTTTTACTGAACTTTTAGAAGAAAAATTTTCACTTACACTTCCCATCAATA

AATACAAATCAGCTAATAGATAAGTTGTCCTGTACTCCTTACATTGCTTTATCGTAGCTG

TGATTTTCGTAATAGCTTCTTCAATATTTTTCTGTAACCATAGGTATCGACAAACGTTAT

AATTGAACTTAATTGATAGTTCTAACTCTTCAATTGTGTTTAAATTAAGCTGATTCACTT

GATGCTCTAGCTTGTCTCTAATCTCATTAAAACGTTCTAAATCTTCAATATCATAATAAA

AATTGAATAGAGTGTTTGAAACTTGAAGGTAGTTCAAATCAGATACATTTAACTGAGGTA

GCATATTCTCTAATCGTGTAATTGCCTCTTCTTTCTGTCCATAAAAATAAAAATCTATAA

GAGACTTTATCCATTCCATGTAAAATTTATCTGCTAGAGATAAGCGATGGGCTTTTACAT

TTTCCAATTCATATATATATTTTAAAGATTCATAATTACGATTTGTGATAAATGTCTGTG

CTAACTTCTTAAACTCTGATAACTCATTGACTTCCTCTACAACCTGCTCATCAAAAAAGT

AGTCCATACTAACTTTTAATTTCTTAGCAAGGGCATGTAAAAAATCCGCTCCAGGGGTGA

ACTCTCCACTCTCTAATCGACTAATTTGCCCCTGTTTACAGATACCCTCAGCTAATTCTC

GCTGAGACATTTTTAATTCTTTTCGTCTATTTTTTAATCTTGTTGCTAATAGTGTACCCA

CGTTTGCATCTTCCTTTTCTATTTGATTTCAATGTTAATTGTAACATGATTAAATCTAAA

TTTCAAATATATTTTACAAAAGATACACATTGTATATAATTATTGGCTAGATTCCTATTT

TTGAAAATCTATCCCATCTCCTTTTGAAAGTCCTGTATTTAAGCGATTTATAAATTTATC

TAGCCTGACTACCATCTTTTCATACCTGCCCCTTTCCAATTCCAGTTGATTCCCCAGGCT

CTTCAATTTTTTCTCAACAATATTAGCACTGATATTCTCAGTTATATTCAGTTTTGAAAG

ATTTAATTTCATTTCTTGCTTACTTTCAACTGAAGCCAGTAGATATTCTGCTACTTGATT

CAAGGTTGACATTCGTTCTTGCACCTCATTGATTCTCAATTCAGACGCTGCTAGTTCATG

CACTAACTCATCTTTAATCGTGACATAAGATTGATTTTCTACTTCCAGTTCAATCAAAGC

ATCCACTTCCGCAATCTTCTCTTTAATCATATCGACTGTCGCTTTTCTGCGTAATGGAAT

GGTTTGATTTTCATAGCTGAACTGTCTAATTAAGGTTCGACCTTTCATATAACAATTTTT

CTCGGCAGCGTCTTTGTGGTAGACATAGTAGGAGCTGGTTTCCCTGATGAAAACCTTCAC

CTTATCCTCTTCCATATCAAGCTGCATGTTCGGCACAAAGATAAGTCCCTCCTGACGAAT

ACCAAACTTGACCTTGACGTAAATTCCATCATCCACTACTTTTTCAATTTGATTGAGTGA

CAACTCCACCTCAAATTCATGAACGGCATCTCTGTTACTCTTGAACTCTTGATAGGACTC

CCAGAACTTCTCATCGCTTGGAAGTTCTTTTTCTTTGGATAACTTTTCTTCTTGATAAAG

TTGAACGAAATCCTCAGTTTCTGGAGCTTGCCAATCTTTTCTATTTTTAAAATAATCTTG

GAAAAACTCTACATCATAAAGATTTTTCTGGTCTAGCTCTGTCTCCTTTACCTCCACTCC

TGCAAATTGAAAAGAAACATGCTTTTGTTTAGGATTGATAGTTAGTCCAAAAAGTTTTGC

TTTCTGAAGTAAATCATCCATATTCTCAACTTTCAGCAATAAAAATTCCATGAGACTTTC

TATTTCTCTTTTGGCAAAGTAGTTCTTAAAAAATTCTTCTGTGTAAGGCTGCTTGCGATT

GAGTTGCTTGCCACGCACCACCTGTTTCATAGTTGAGTCCGTAATAAAAAAGGTGGCATG

CTTGTGACGGAAATCCATCTCCACATGTAGTAGCGGAGCATTCTTTTTGAAATCCTCAAA

GTCCCTAGAATGTTCCATCAAAAAATAGAGTCGCTGCTTGAGTTCATACTTGTGATTAGT

CTTACGATAGACTTCATACCGCTGGTGAGAATAGCGGTTCTCAATGATTTTAGCACCTGC

GATTTTAGAAAAACGGTCAGAAATCATGCGAAGATTTCGCTCCACCTTGTAGTCCCACTT

GAGCTTTTTGTCAGAATTGCTATCTACTGAATTGATAATGATGTGATTGTGCAGGTGGTC

TTTATCAACATGGGTCGCAACGATAAAACGAAATTTGCCACCAGTTAATTCCTTCACAGT

CTCATAACCTATCCGATTGATTTGTTCAGGAGTGAGATGATCCTCTGGCGAGAAAGACTG

AATGATGTGGTGAGCGTGTATTTTTCGTTGATTTTCTTCCATCCTGTCGTGGCGAAAATC

GTAAAGCGTATCGTTGCTGATGAAATTTTCATGATACATCTGCACCATTTCCTCATAGCT

AGGAAAGTCCAGAAAATTCTTCATGCCATAGTCCGACACCAAGGCAAGATTATTGGTTTT

CTCAGGATTGAGAATGTACTTGATAAGCTTTCTGCGGTAACTCTTTCCGTGAATGGCAAA

GTGTTTAGTGATGACCATGGAACTCCTTTAGCTTCTGCGCTTGCAGATTAAATTCCTTAT

CAACTTCTTTTATCAATTCACCAATTCCTTTTTTCAACTCCTGCAATTCTTCACCAGAAA

TTAACTGAGATTGATTAACACTTCGAGCAATCTGGTTGATATTATTGCCAATCCTCTTTA

ACTCAAACACTAAATCTTGGTAACCGTTTGTGTCAATCGTGATAAAATTCATACCAGGAT

CGAGTAGAGTTCGTCTCGCATATTCAGAAAAGGATTTACAACCACTTTGATCGATATTTA

TATTTAGTTGGTTCAACTCACTATCAGACAAAAACACTTTCTTGAGATTGGTTCGGTAAC

GATGTTCCATGTGCTTACCTCATGTACTTCTCACGAAATTCTTTACTGAGGGGTATGGTG

TTTGCAACCTCTTGAATCAATTCCTGCACGCACGTTAAGAGAATAGATACATGCTCTTGG

GTGACTTGACGTTCTGACTGTGCCAAGATTAAAACTTCATGAACGTCACGACTGATTTGT

TCTAGTTTTTGGGATTGCCAGAGGGCAAACCATGTCTCCATCTGTTTTTGTAAATCAGAG

GAAAGTAAACTTTTACGAAGAAAATCAGAGAAATTAGTCTCTCCCCGCTCTCTCATCAAA

GCTAGAATTTGCTTTTCTTCTGCTTCTGTTAAACGAAATTGTTTCCGAATATCACGCACA

TCTCGTTTCATACTGCTACTTCCTTTCCAAAATACTTGTACTGACAATGTGAGCTACCAT

ACAATGTCAGTACACTTTTCCAACAACTGATTTTCATTCTTGTAAGGCTTTGCGAGCTCA

GATATTGTGTCCACAATATCCCAAAAATCATATCGCCAGCCTATCAAAACTTTCTAGTTT

TGACAGCCAACGATAAGATAACTTGGTGACTTCGCCCCCAAACCCCCATAGAAAATCAAA

AATTGATTTTCTATGAATGATATTAGGATAACAGGGAAGATTTGAAAAAGGTATCACAGC

TAATATAGTTATGATTGACTTTCTCTGATTCTATGATAAAATTTCTGTAAACTAATATTT

GGAGAAATAAAAATGCTGAGTCTAGATCAAATACACTTACTATTGAATACACCCGAAGAT

GAGTTTCATGATTTTAAACAAAAATGGCATCATTCAAAAACTGAATTGGTGCGTGATATC

TTAAATTTTGTCAATACATCACATCACGAAGATTGTTATATCATCTTTGGAATTGATAAT

ATCACTTTAGATATAATCGGTGTAAACAATGATGATAATAGAAGAAATGAAGAAGATCTA

ACAGATTTACTACATAAACTCTTTATATCAACAAATAATCAAATTAAAATTAGCATACAA

ACTGAAACTATAGACAACAAAGAAATTGACATCTTAATTATTCATGATACAGATAAAGTT

CCTGTATTTTTAACAAAAGATTATAAGCCTAAGAAAGATACTGCATTACCAAAAGGATTA

ATATATGCTAGAAATGGCTCTATAAATACCCCTAAAGACTCCTCTGCTCCTTTTGAGTTA

ATAAATGAGTTGTTTCAAAAGTTTAATCATACCGACTTAAATATCAAAGAGCAGTATTTT

CATGTTTTAAAAGACTATAAAAATTGGTTCTTCATTGAAAATGAAGACGGAAGATTTTTT

ATTTATAATCCTAATCCCGATTTTTATATTAAACTTAACGACGATGATGCAAATCGTTTC

AAAACTATGTCTTATAGTTTAAATCAATACCAGACTAATATTGATTGGCAATTAGTACAA

CTTCGATATCGTCATCTTACAATTGACGAGTGTATGGCTATGTATTTAGATCAAGGTAAT

TGTCTAGTCCCTTCCCCTGAAGTAGAAAGTTTTAAAATAGATTATCAGGAGACTATTTAT

TATCACTGCTTATACAAAAATACTTTAAAGTACCAATTGCTGAAAGTATTTTCTTCAATA

CCTGGTCTCGAAAAATATCCTTTAACTAGATTTAAAAATAGTATAGTAATTTATGACACA

AAATTAGAATTGAAAAAGACTCACAACTTGATAAATAGTAAATTCTCATCAAAAGACATA

GTGAATCAACTTGAAGTTACAGAAAAAGATTTCGATTTTTACTATAAAAAAGCTAGACAT

AAAAATCCTGATTATAGCATCCAAGAAAATCAAGTAAATCTAACTGAATTAAACTTAGTT

AGGTTATTGAAAAGTTTTCAAAAAACATATCTTGATAACCCACTATAATCAATCTTTTTA

ACTAGTAAAAAAGATAAAACAAGGCATAGAAATTAGTTTATCTAAAAAACTAATTTCTAT

GCCTTAATCATTATTAATTAATAACATCAACTAAATTGGATTGAGTCAGCAATGAATATA

TCTCATTAATATTTCTATTATTTTGCGGACTATTATTTCTTAATTTACAGTATGTGAATA

TTAAGTGACTTTTTGCTCTTGAAAGTGCAACAAAGAACGCACTTTTATCTTCTTCTGGTT

GATCATTAAAACTCCAGAATGCAGAATCCTCCAGCCCTAAAAAATAAACTGCCTCATATT

CTAATCCTTTACTTTTGTGTATAGTCATGATTGAAATTGAGTTTTCACCTTTGAAATTAG

ATATTATATCTATCCATGCACCTTGTGATTGAGAATACTCTTCATATAATAGTTTTGAAA

ACTTATTGATGGTGATAAGCAAGTCATTTTTCCCATTGTACATTGGAAAGTTGGAAATAA

TTCTTTTGTCACCTATTTTTTCGATAATACAATTAATTAAATTTAGCATCCTGTCTTTAT

CGGGAATAGGATTTGAAATTAGGTAAGTAATATCACTAACTATATTGTCAATCTCCTTAT

AGGATTTTGCTAAAATTAATTCATCAGTTAACTCATCTATTCCATTGATATTTCCATAAA

AATTACTAATATTTTCCCAAATTAAAGGATCTCGTTTTCCTTGACTGCATGATATTAAAT

CTAATAATAGGTTACACGTAGGATCTTTTAAAATATCCTGATATTCATTTTCAATTCTTG

CTTTAATCCCTTTGCCATTTAATATAGATATCAATTCGAAACTATAAATACCAACCTTTT

GTTTTGCTAGGATACAAATTTCTGATGGTCGTATACCTCCTTGAATTTTTGATTCTATAT

CATTTGCAATTAACTTAGCTTCTAAACTTTCATTTTCAAATTCAAATAATGTTATTTCAC

CCTCTTGAAATTCTAGATAATTATTTGTCTGAATAGAACTGTGATTACTATTTAATATCT

GATGAACCTCTTTTTGAAATTCTACAAGTTTAGGTACAGAGCGGTGATTCATCAACAATT

GATATTCATTTGAATTAAAGTCTCGAATATAGTCTGGAAAAATATCAGGCTTTGCACCTG

CCCATCTCATAATGGCTTGCTTATCATCTCCAACTGCTGTTAATTTACAAGACGAACCTA

AAAAACAAGTTTTTAATAAACTATACTGGGCATATGTAGTATCCTGAAATTCATCTAGAA

ATACAAAATCGTATGTCAGTTGAAGTGCTTTACGGATGTATTCGTTAGTAGCTATTATTT

GAGTACTCAACTTTGTTATTTGACTATACAAGAGGACCGGCTTATTATCTTGTGTTCCTT

TTAGTAGATTCTTTTTAAATTTGTGAGTATTCCCGTCATTAAGTATAATATTTTCGACAA

AATTTCTTATATCTGACATTCTCCGTCCATTAACATTAATTCCATTCATAGAGAGTAGTT

CTTTTATAGTATCCCAATCTTCAATCAAATAATCTCTTGATGGTCGTATATCCTCAGGTA

GAACATCTCTAAATTGGTCTAGAATTCTCTTTTCAAAAGCCGAATAGGTTAATGAGGTAA

AACGGGAAGCATATTCATCACCATAACGTTTCTTAACTCTTTCTTTCAAATTTGATGCAG

CATCCGTTTTGAAACTTAATGCTAGAATTTTTTTCGGAGAAACGCATTTATTTGTAGAGA

ATAGATAGTCTAACTTTTGAGCTAGTAATTCAGTTTTCCCCGCACCTGGTCCAGCAATGA

CTAAACAGTTAGTCACATCTTTGACGGCTCCTAATGCAGTCTCTTCTAAAATGATATCTC

CTTTAGGAAACCACTCCTCACTCTTTACCATGTTCAATATCTCCAAGTAATTCTTCAGCA

CGGTTAAATACAGGGGGTAGATTTCTTGTTAATTCATCATCACTAATAGATGATAAAAAT

TGCATATGAGTTGTTGGCTTTCCTCTACCTAAAAAGAAATATTGATATCAAATCATTAGT

TCCTTTTCCTCTTCAGTGAAGCTATCACCGGGTCCGCTCTTATCTTTTAAAGTTGCTTGT

ATAGCTTCACTGATGCGATTTTTAAAACCAATTAATTGATAATCATTTGTTCTATCCATT

TTAGTTAATTTTATTTTTTTATTGTCACTATTTTCATCAGTATAAGAAACAACAGGTCCC

TCATTGGTAGATAAAGTTTCCAGATAATGTTCTTTATAATACTGTAACATTAAGAAATCA

ATATCAAGTGGTGCAGAAAAGAAAACATTGAATTTTTCTAACATTTTAATCCACCCCTCA

AGCAACTGATTATCAGGTCTATCAATCTTCCAGTCCTTTATTTTATCAATCTCATCATCA

TCTAGAACTCGTGATTTTTGAGATTCGGCTGTTACAGTAACAAGTTTTTCTTTAGCAACC

CCATTTAGTATCAATTGTTTTATTGCATATTGAATTCTTCCCCAGCCACCTCCATATCTT

TCGTTATCGAAATCTAATAAGGTAATATGAGGAATCCTGAGAGCGTTCAATAATTTCCAA

AAATAATTAACATGCCTACCTCCTAAAGGAACTACTGAAATCTGAGAACTATCAATTTCC

AGTCCAAGTAAATCAAAAAACTTTGGTAATAGCAGTTCCTCACTATCACCTTCACCCAGT

ACTACTAACTTTGCAAAATATAGTTCAGGATAGGCTTGAATTGCTCCCTTAATGTATTTA

TAAGACTCATCATTCGATTGAGGTAACTGTATATCAGAAACAATGGTTTGAAGAACACTG

TCATTATTTTCTATTCTTAAATACCTCAAATCTTCTGGGTCAATTCTTTTTACAATAACT

GGAGAATGTGAAGTTAAAATTAATTGAGAGTTGTCATTATTGCCTAATTGCTTAAACCTT

TTTATCAGTTTTCCAATGTGATGTGGAGCAATATGATTTTCTGGTTCCTCTATTGCTAAG

ATAGTTAGTATTGGAGGTACCAACTTAAATCTTGGATTATCAGGATTCTCTTCACGATCT

TTGGTAATTTCTAACTCAATATCAAGTATCGAATCAACAAGTGAAAAATAAAAAATAGAT

CTCAGTCCATCTCCTAGATCTGAAACTGTAAATGCTTCTTCAGTGGTTGTTGGTGAAAAT

TTTAAGGCAATCTGTCTAAGTGCTGCCGCCATCTCAGAAGAATTAATAATCAACTCTGCT

TGAGAAAAACGGTTATCTTCATGATATAATTCCCATGATTTCTGAATCTCATTGTTGATC

TGAGTTAATGCTCCATTTTCAGATAAAAAAGTGTTATTCAACTCATCAATTTTATCTTTA

ATTTCTTTTATCTCATCTTCAGTCCAATTTATACTATTTACCAACCTACTTAACATACTA

CCTGAAGCATTACCCAACTCCTTCTCAGGCGTTCTTGAAGCTGGTACATAAAGTACCCTA

ATTTTATCCAAATCCTTTCTAGGAGCACGATGTTTGTCTTCATCTCTAATAGTATCTTCA

TCAGATGAAATATAATAAATTTGAGTATCAATACTTCCTTCAACTGTTCCATCATCTTCC

CAGGAAGATTCTAATCTGATTCGTAAAAAAGGTTTTGCACCATCTTGAGAAACTGTAAAG

TGCTCGAAAAATGTTGGAATGGCTGGTCCATAGACAGTTCCATCAAGTTCATCAAACTCA

AAAATAGTTTCTATAAAAAGATTTCTTGTGTTTTCACCAGGTCTTAACCCTTTTGGTAAA

TGAAAATCACTTTTTCGAATGATTCTATCACTTTGTTTATCTGAAAACAACTTACTCAAT

GCCTGTAATACAGTTGTCTTTCCTGAACTATTATTCCCTATCAGTACAGTTTGATTATTA

AAGTTAATTGCTTGACACTCCCCAAAAGATTTAAAATTATTAATAACCATTTTTGTTAAC

TTCATTACTTTTCCTCGTTTCCTAAATTCAACTTTTTTAATTTTAAGTGCTATTTTCCAC

CTTCAACATATCTTCCTCAACCTTACTCTATTACCCAAAAAAGGCATCATTTAAGAGTGA

CAGATGCTTAACCTTGATCTATTTTTGAAGCATATACACATCTTCTATAAATTCTGTTGA

AATGGTACTTGTTCTGTTTTTATAACAGCTAAGGGCTATTGCGTAAAATTTATACAATTC

GAGCAAATCTGTTTTTGTCAGGCCATGTGTCTTTAGTAGAATTTTATATTGTTACCAATT

ATGTATGCTCCTTTTACTTGATAACACTATTATAACATTTAAAAATAAAAAACATGTTCT

AAATTAATAGAAAGTATAATTCTAAAATTAGAATTTTTCGTGATAATCCTATAAAATGCT

ACTCTTCTAATAAACTTGTACTTATTTCCTTCAAACTACACAATTCTCTAGTTGTTGTAA

ATTTGTAATTGACTCAAAAGTATTCGATATAATTTCAAAACAGTATTTTACTTTATAGTT

GACCAATAACATTTAATTCTAACTAAAAAAATCATTATACAAATCAAGTTACTAAAGATG

ACTTTATGGTATACTAGTGATAATAAATTGTTATCGGTAGGAGAAATCTGGTGTGAATAA

TAAAAAAATAAGAGTTTTTTTGAGTAGACCCAATCCATTTACTAAAGAACAACAGTACTT

TATCGAAAAATTAAAAAGTACCTTAGATAAGTACAATATTGAAAGTATAACATTGCAAGC

AAAAGATTATTCACCATATGAATCTTTAACTGTTTTGAATGAAATGATAAAAAGATGTTA

CGGTATGGTCATCTTAGCTTTTGGACATACATATATTCAATCTGGTATTTTAAAAAAAGG

AGCAGTTAAGAATAAAAATTTTTTTGAAGCTGAAGAACAATCATTAGATGGAAAATGGAT

TACTAGTTCTTTTTGTCAAATTGAAGGTGCTATGGCAATTTCAAATAATATTCCCATTTT

AATTATTAAGCAAAAAAATTTACGTATTGATGGTATTTTAAAGGATGATCAAAAAATTGT

ATCTGTATCAGATTTTACTTTAGAAAACACTTCACAAATTGATTTCTTTTTTGAAAAAAT

TTTAGAAAAAGAAATATACTGTTGGAAAAAATCACTAGAGAAAATATTTAATAAAATTGA

AGGAAAAATAGTATAAAGCGTTTTCTAAAGTGGTATAATAAAGTCAAAACTTTTATAGGA

GAAAATAGATGAAAAGAATTTTTATAAGCTTTGCAATTGAAGATGAAAATCTTCGAGATT

TACTAAGGGGACAATCGCGAAATACGAACTCCCCATTTGAATTTGTAGATATGTCTGTTA

AGCAACCGTGGGATTCTCAATGGAAAACTAAATGTAGAACAAGAATTAGAGGGTGTGACG

GTATGATTTCTATCATTACTAGGAATACCAAGAATGCCGATGGGCAAATTTGGGAAATGA

ACTGTGCCAAAGATGAGGGTATCCCACTTCTTGCAATTTATGGTAATAATAGTCATATCG

GTGCTACTATTCCAAATGAATGTGGCTATCTTCCTGTAGTAGATTGGAATTGGGAAAAGA

TTAGCGCTTGGATAAAACAATTGTGAGGAATAATTTATGTTAAAAAATAAGGCATTAATT

GTTGGTATCGATGCATATTCTCAGGCACCATTAGTGGGGTGTGTAAATGATGCAGAGGAG

ATAGCGAAATTATTGGAAGAAAACGAGGATGGTTCCCCTAATTTCAGTGTTAAGCTAAAA

CGTAATGTACAAACAAAAGCAGAGTTATTGGAGGACCTTCATTCTTTATTTAAAGAAGGA

GAATCCGATATTGCACTTTTTTATTTCTCAGGACACGGGACAGGTGAGATGGCTGGTCAA

ATCGTAACACCAGATTTTAATGGCTATGATATGGGAATTTCTATGAATGATATACTTCGA

CTGGCAAACACTTCTAAAAGTCGAAATAAAATTATCATCTTAGATTGTTGTTATTCTGGA

AAGTTAGGAGATTTTAGTGCTATAGACTCATCGGATGCCATTATTGGAAAAGGTGTTACT

ATTCTAACAGCAAGCAATCGTGATGAAGTTGCTATTGAAGATGGAATAACTGGTCATGGT

GTGTTTACTGAATTACTAATTCAGGGGCTAAAAGGTGGAGCAGCAGATGTTAGCGGGAAT

GTCACTCCTGCTAGTTTGTATTCTTTTGTCGATCAGGCACTTGGAGTATGGGAACAAAGG

CCTCTATTTAAAACAAATATAACAGGATTTTTACCTATCAGAACTGTTGAAGCTAAAGTA

TCCAAAAAGGTATTACGAAAACTTCATCATTACTTTGTGGAACCAACTTCTGAATTTCAA

TTAGACCCATCATTTGAATTTACCAATACACCTGATATTACTCATGAATATAAAGAGCCG

TTTGCTCAGCAAACAAATGTAGACAAATTTAAAGAATTGCAACTATATGAAAGTGTTGGT

TTGATCGAACCTGTTGGGGAGGAACACATGTATTTTGCTGCTATGAACAGCAAGTCTTGT

CGTTTGACACCACTAGGTTTACATTATTGGAAATTATCAAGAGATAAGAGATTTTAAAAG

GGAAGTGAGTAAATTGACTCACTTCCCTTTTATTATTTTTAGAGTTTCGTAATAATCTTC

CTTTAGCTCTTTACGACCTGAGGTAACAGTCTCTTCTTGCCTTTTTGTAATAGGCTTAAA

TTTAATAATTGATTCGTCAGTACCAAATGCAATTAAAGTCAAATCTTTAACATTTTCATT

AAACCAAGTATCATCGAGCTCAGTTTCCTCTCGTTTGAGAAATTGTAATTCTGTTAAGCA

AGTTTTAGCAAAATGAACAGCAGAGTAATAGTATTCTTTTACTGATTCGATGTCCTTAGA

TATACGATATTGTTTTAATAAAAGAGCACAGTAATTTCTAGCACAATAGAGATTCCCACT

CTCAAAATTTGAACCATTTTTGTAATATCTTCTAGCTTGTTCTAAGAACTTACATTCTCT

ATTAAGTGCAAAAATTTGCTTATTTATAGCTGCAACAATACCTAGCAAGTTTTCATTTGT

AGAGATGGTTAAGTCTATTTTTTTATCAATGTATGATAAGGTCGAAAGTAGATTTTCTAT

GGTTATATCTTTTTTATAGCGACATAAAATGTATTGAGACAACATGTCTTCATCAAAAGA

TAAGTTTAAAATCTGAAGATACTTTTTCTCGGCTTCTTCAAACTGCATATTTTCCTTCAA

ATTTTCAGCTTCTTGAATCATAGTCCTTATCGAGTCGCCTTCTGTATCTTCGTTTTTTGA

CAATTGAATTAATGGTTCAATTCTATATAAATTATAATCAAATACTGGACTATCAATGTA

TTCTGGATTAGAATTTAAACAAGCGATTAAAGTTTTTTCCAATTGCTCCTGAACGGATTT

TCTATAGTTTTCATCCGATAATATTTTCTTTCTATGATATCTTATCTGTGGACTCATTGA

GATATCAAAAAATGGATATTTGCGAATGGTAATTTCCTCACAAAGAATAATTGTAGACTT

AGGTTTCATTGCATGACGAAGTCCTAACTCATAGATCGCATTTTGATTCAGAGTACTTAT

ATCAGCAATTACTATGTCAGCTAAAAAAATAGATGTAATGAAATTTTTGTTAATAGCTAC

TGAAGTAGGGACTTCATCACCTCGATATGCATTTGTGGAGTAAATGGAGTTCAAATTATT

CCGTGATAAAACTGGCTTTATAAACTTAAAATATATATCATCTAAGTTTAGCTTCACCAA

CGTCCCAGGAATTTTCTTTACTCCGTAACCCATAACTACAAAGCATGTTTTATCATCCAT

TTTTCATACTCCTACCGATAACAATTTATTATCACTATACAAAGCACTCAAATAGGTTTA

TAATAGCATGATTATTGATAAAGGAGATTTTTATGGATTATAAACTTATTTCTACTTACT

TAGATTATTGCAAAACTCATAAGCGTTTGAGTTCACACACGATTCGCGCTTATAAGAATG

ATCTTATGCAATTTTATAACTCAGACTATGATAATGTCGAATCCTATATAGAAAAGTTGA

CACGATCTAACATAAAAACGAATACATTAAGAAGAAAAATTGCTTGTATGAAGGTGTTTT

ATAACTATCTAAAATACCAGAACATCATTGAAGAGAATCCCTTCAATCAATTGCGCTTTC

AATTTAGAACTGAAAAAGTATTGCCTAAAACGATTCCGTATGACATTCTGAAAAGCATTT

TTATATATTTAGAACAGAAAGTAATTGTATCTAAAACTGACTATCAAAAACAACACGCTG

AAAGAAATCTACTAATTATTTCACTTTTACTTTCAACAGGCATCAGAATTTCTGAACTTT

GCCACATTCATCTCAAAGACATTAATCTTTCCAATAAGACACTCCATATTATAGGAAAGG

GTAAGAAAGAACGTATCCTATTTTTAGGAGATCAAAAAACATTCAATTTATTAGAAACAT

ATATAAATAAAACAAGAAATGAATCCAATGATTTCTTGTTCCCAGGGAAACATTCACTTA

AACCATTGTCAGAGCAAAGTGTACGTTTAGTAATAAAGAGAATCGTTGAACAAAATAACT

TTTCTAGAACTATTACACCGCATATGTTTAGACATAGCTTTGCGACAATGCTTCTAGATA

GTGATGTAGATATTCGATATATTCAACAAATTCTTGGACATAGTTCTATATCAATCACAC

AAATCTATACTCACGTATCTCATTCAAAACAAAAAGAAATACTTAGTTCTTTTAATCCCG

TATCAGTAATTCATTCTGAAATCGAGTAAGAGACATCTCCAAAGTCTACAGTTCACTTCA

TCTTAAATTAATTTTTGTAGTTCGACTTTAGTTAATAGATTTTATACTCAACAAAAGCAA

AAAACATATAAAACTTCACTTTGAATCAGCATTTATACTATATTCCGTATTAAGAACTCC

ATAATTGTTTTTCTAAAATACATCTTTCAAATATCCTTAAATATAATAAAAAGAGATGGG

CAAAATCTCAATTCCTGAAGAAAACGCATAATTTCAAGTTTTTCAACACCTGAGACTATG

CGTTTTTGGGTTCTAAAAAATTTTCGCCCACCTTAAACAACTAACAAAAGTTATAAGTAG

ATTATATTTTAAAATTTGTGTTCTAACAACCATTTCGATTAGTTAAATCTTTCAATCTTT

CTTCTCCAGATTTAAGCATCTCTTCCTCTACCTTGCTCCATTTTCCGAAGAGGTATTCTT

GTAGCACTTCTGCTGCTGAAGTTGTATTTTCTTTTGAATCATATACACAACTTCTATCTC

GTTGGTAAATTTGAATTCTTTCAAAGATAGCCAGTTCTTCCAATTGTCGTGTATTATCAA

CTAGATGATTTACAATGAAATCATGATGTTCTTTTGGAGTTGCGCGTGCTTGATTTGGAT

TGAGAATGTACAGTTCTTCGTATCGGATAAGAGTGCTCAGATAGGACAGCTCAGGCTTTG

TCGCAATTAAGGCCAATTGTACTTCATATCCCTTATTTTTCAAGAGTTGTGCTGTTTTCT

TTGGAACATCAACTGTTCGTAAAGTTCCCTCTATCAAAAGATTGTATCTCAAACTACTCA

ATTTTGTTACTAAAGACTCTACCATTTTTCCTGCAAAATCTTTGGTATATTCTACACTGT

CTTTGCCATATTCTTGCTGCAGTTCTAAATAGTGTGTATGCTGAGAACGAAAACTATCAC

CATCTATGATAACAATATTTCCTTGAAATTCTTTCTGTTTAATACGATGAATTGTAGTCT

TACCGGCACCACTTTGACCTCCAAGCAAAATCGCTATAGGTTGTTTACTGGACTTTTTTC

CTCTTGTCAGTGAACGAATATTCCTTGCTAAAGCATGTTTGAATTCACTATCAGTATAAT

CTTGGATTTCCATTAAGCTACCATCCGCTTTTCAGATATCTCTAACATACGTTCAATTCC

ATCCAAATAGCCGCTATATCTCTCTATTTCATCAAAATTTTCTACTAAATAGATATTTGT

CTGAATCAAGTCAGATAGATCATCACTCATTAAAATCCAAGGATTAGATTCATCATCAAT

GCTAATTCCCTGACTATCTTGATAACGATAGAGTCGAGATAATAGATTAGCACCTCTTTC

TTTCACAATTTCAATTTTCAAAGTCAATTCATAATCTTCAACAGGATTGAGCATTTTATC

TTCTCCTACAATATCGACATAAGATACATTAAACTTCTGACAAATGATGTCAATTAATTC

CGTAGAGACTGAACTAGTTCCATTTTCATAACGACTCAAGCTATTTCGAGAAATTCCTAT

AATTCGTGCAAATTCGTGTTGTGTTAAGTCATGTGTTTTACGTAAGGATTTTATGTTTTT

TCCAATCATGGCAAACTCCTTTTATTTGATACCACCATTATAACATTTAGAAAAAGCAAA

TGCACCATTTTTGGTGCGTTATTGACTGTTTTTCCTAAATCTCTCTAAATTCTTCTCCAA

TTGTTCCTTATGAAGTTGATTCTTAGCTTCTTTTAATGTGTCGTCCAAAGTTTCTTCTCT

GTAAGTTGCTTCTGTTTTTAGTTGTTCTGATCCTTGATCTGGAGTGAAAATGATATCTAA

CAGTCGGTCTATTTTTTCAAGATCTTGGACAGACATATCAGATAGTCTATGAATTAGCTT

CTTAAATACATCACTATTGTCTTGGTTTTTCTTAAAAAATGTTGTAAACATCTATAACCT

CTCAAAAAAAGCAGCCTATCAGGACTGCTTAGTGTAATTCCGAAATCGCATCATAAATGG

ACTGCAATTTCTTATTGTCTTTATTCTTTGTGTCAATCAACGTATGTAGCTCTTCAATTT

TCTTTTGACTACTTTGAATATCACTATTGTTATCCGCAATCAGTCTTTTAAGATGTTGCT

GATAACTTGTAGTGATATCTGTCTCTTTTCCTGTTCTAACCTCTGTAACTTTGGGTTTAG

AACTAGAATTTGATTCCGTCACAGGTGCTTGTTTGCGCTTTTGAAAAGCCGCTTCATAAT

TTACATCATTATCTCCTTGATGATTTCCAAATAAGGCCGCAATTTTATCTGGATCTTCTT

GATTTTCTGATTTACTTTCTAATGGTTGATTAAAATTCCAATCTTTTGTCATTCACTCAT

TTCATCCTTTCTAAATTCAGATTCATCGAATTGTCTTTCTCTACCGAAGGCATGGTCAAG

AGCTTCTTCAAAACTCATATGACTAATGCTTTGACGCCTTTTGAACGTCGCAAACATCGC

TGTCTCCAGTTGTTCTTTGTAAGCAGCGAGTTTTTCTGTCTCTCTTGCTACCTTACTTTC

TTGCCTAGTGACCTTTTCTTCTAAGCGTTCAATAATGGTCATATACGATCCTCCTTCATT

CTAATATTAGCTAAAACATCTTGATTTTGTTATCACAATTCTAAACATTGAGAGGTTTTT

CTTACTGGTTGATTCCTCAATGTCTCTTTTGCATTCTCGATCATCAATTGTAAATCTGAT

TTCTTTTCTCTAAGGACATCATTCCAGTCTACTTTTTCTTTCCCAGATTTATTATCAGGG

AAATCCAGGAAAACAGGAAATCCTGATTGAGATAACTTATCAGAAAAATCTTTTCCTGCA

TCATCACAATCTACCGCAAGTGTCAATAAATCAGGATGATTATCAAAATAGCTGGTGGTA

TCACGAATTGTATTGATTAAAGGCAATAACTTTGAAGGTATTACTGTATCCAAAAATTCC

AACTTCTGATTTTCTTCAGCTATCAGTCGTAAAATTTGATAAGCAACAACAGACCTTTTT

AATCCTTCCATAGATACCAAACGAACATCAGATAGACTTTGTTGATGAAGTTCGTAATAG

CTCATTAAGTCGATGAACGATTCACAAAAGACCAGTCTATTAGGTTTACCAATATCAAAG

GATATTCCAACATGTCCATGGCTTCCTTTTAGAATCGTTTTTAACCTCTCTCTAGGAAGA

GAGTGATTCTTATAAATTCCTTGTAAGCTTGCTGCCTGCAGCTTGTGGCGATGATCAAAG

CTTTTAAAAACGTTAACAGGTTCAACTGTTTCATTTGTTTTCCAACTAGCTTGTGCTATC

AAACCTTGTTGAATCATCTTTTGTATGATTTCTTCTGAGATTCCTCTACATTCTGTTAAG

TAATATCTAGTCAAACTGCAGTTAGAATCTTCTACTCTCTTTAAAGGATAATAAAATGGT

CTCTCTCTTTTTTCTTGAATAGCTTCTTTTTGAAAAGGTTCTTCAGAAAGAAAGGCTAGA

GCTTCTTTAAAGGAAATTCCCTTAACAAGTCGAACAAAATCAATGACATCACCTTGAATA

TCTCTTGAAAACCATTTAAAAGTATTGGTAGTTGAAAAAATCCGAAATGAATCGTGTTCA

GGATGTTCATAGACACTGCTCGAAACTTGTTTAAAGGAGATACCTAAACGATTGGCTACA

TCAAGAATTGAAATTTGCTTACATTCTTCTATTCCCATGCAATATCATCATTTTGTCGTA

GTATTTGGCAGTGATGGTATTGATGATGATTCTTCTAAAGTTTTAGGAGTTGCATTATCT

AAATCATCTAACCCAGATTTCCAAACCTGTACTTGATTGACCAATAACTTATCTGGTAGT

TTCGAATAAGACAACTTAACCGTTCTGGTTTCTGTCTGATTGGTCTTTGATTGGTTGGCA

TTCTTTAAATCGGATACATAGGTTACATTATAAGACACCATAGCAATGGCTTGATTCGTA

GTCTGATTGACAAAGATATCAGCTTTTTCAAAATGATAATCCAAAATATAATCCTTATAC

ACTTGGTTCATGGCATCATTTTGACTTGACAATTCTTGAGAATAAGCTGATTCAGTCATA

TAAGGTTGAATACGTGTATTATTTTCTCCGAGCTTTTCTTTCGTATAGTACTGTGTCAAA

AATTCTTTTACAGTATCTGATGACAAAATACTTGCTTTATCTTCTGCTTGTTTGTCCTCT

ACAAGTTTAGCTGCAGCCAATTCAATCTCTTTACGACTTTGTTTAGCAGTAGAATGTTGA

CCAGCAGTATATCCCATCATGAGAATAAAGCTAGTTGCAGCCACTGCTCCAACACTAATT

AAGGCTTTAGTTTTGACTTTATTTAACATCTTAGACCTTCTTTCTATAAAAACTAGGTCA

AGAATAACAAAAAGAACTTGTAAGTTTTATCACATCACAACGAAAGGTAATTCCTACATT

TCAGGCGCAAAATCGTACAGTTCAGAGAAAAAAATAAAACTAGCTGTCTTTGAAGTATAA

TAGACTTATTGGAAAAAAATAAAATGCATACTGTACTATGCTATACTATAGTTAAGAAAA

CTATTTACAAAAGATCATGCAACTAACTTCTGAGAACTTTAAATTATTTATTAGTGTAAA

AAAGGACACCTTCCCAACGAAGATATCCTGTCTAAATAAAGCTTATAAAACCAAACACTA

TAAAGGAGGACGTCCAAAAACGCTAAGTCTATAAAAACAGTTGATGCTAAACTTGCGTTA

TTTATTCTATTGACCAACTCTACGTCTCCTAGCTTTTGATTTTGGTCTTGGATTAGCGAC

AGTCACCGAACGATAATATTGGTAGAAGATACTCTTCGTTCTTCAGGTCTATTTGCCTTA

GACAACTTAAAAGCAGCAAGTGTCACTATAGCTATTGATGTCATAGAAAGTCCTATTTCA

ACGTCCTAAAAGAAACCAAAGTAAAATTATTCAGGAAAAAAGAGACACACCATGAAGACA

CAAATCATACTCGATTTAACACCACTTCATGCCTGCTAATTAGTTTTTTAAGAAGGTCAT

ACACATGATTTTACTCTTTTTAAGAAAGGATAGGTCAAGTTTGCCAGAGCAATCACTTGA

TTTTCTGTATTTGGATTATTTATGCATTATGAATATTCATAATATAACCTTCATTCCTGT

TAAAAATTCTAAAAAGCATCTATTGACTGAAGAGGATAAGAAGTTTAATAGAGATACTTG

TAATACGTATTAAAATTGAGCATTTTAATGCTAAGTATAAAACTTTTCAATTTATGGAAT

CATCAACTATGAGTTAAAATAATTTTCGAACAAAGGCTGTCCAAAAAACTTGATATAATG

CGTTTTATTATGGGAATATTCATTTCATTTACTCCTGAAATTGAGTTTTTACCCAGACTC

ATTAATGTTATTGATATAATCCAATAATAATGATAATATCAAATAGTAATAAAAAAATTT

GATAGTATTTTTTCATAATCTGTCACGCTTTCTAATGATTTTTTATGGTAAATGGAGATA

AATATGATTATAGGTGAAACTTATAGAAAAATACGAGAAGGAAAAGGTATTTCTATTTCT

TCATTAGCAGGTGCAGAAATTTCAAAGTCTCAAATATCTAGATTTGAATTAGGAGAGACA

GAAATCTCATTTTTTAAATTATTATATCTACTTGAAAAAATAGGTGTAACACTAGAGGAG

TTTTTGCTTTCATGTAATAATTATCAGCCTTCAGACTTCAATACCTTGATACGCTTAGTT

CAACAGGCTGCATACAATCAAGAAATCAAATCATTACTTAATATGGTAAGCAAAGAGATG

GAACTGTTTAGAGAAACCAAATCTCATTATCATAAGTTAAACGCTATTTTCATTGAAAGT

ATTATCTATGGAATTGATAATACTCATCAATTGAGTAATCAAGACACCTCTTATCTTACT

AATTATTTATTTTCTGTTGAAAACTGGGGATATTACGAAATTCTTATTCTTGGAAATTGT

TGTCGAGCAATATTACCAAATTTATTATTTAGATATGCCAAAGAGGCGCTTAAAAAAGGA

AAGTTGTATAGTTCTATACCTAGAAATAAACAAGCTCTCGTTCAATTACTTCTTAATTCA

CTTCTTATTATGATTGAAAACAGCTTATATGAGGAAGCTTTATTTTTAGAACAAGCTACT

AAGAATATATTATCTAATTCCACAGATTTTTTTGAACAAACTATTCTTTTGTATTTAGAA

GGATTTTTTGAACTTAAATTTCATCATAATCAAAAATCAATTTTAAAAATAGAAGATGCT

TTAAAAATTTTTGAATTATTCAATAAAACATTATACAAAAATTATAAAGATTACTACAAA

AAAATATTATCATTTTATTACAATGTGGTAATTCCTATTTTGAATAATTGCTTTTTTTGA

AAAACTTTTTAATTCATCTAAAGAAATCAATTCCAGCTCTTCAGCAATACTATCCCAACT

TGGAATATCAAATAATTTTTCTCTACAAATTGTTTTTAAATATTCATTTTTATTAACTTC

ACAATTTATGTACAATTCATTGATTACTCTTTTTTTAGCCTTATAAAATAATAGTTCATC

AGATAAATCGGATTCATTAACAAGATTGTTCTCCAGTGTTTTAATCAATTTTTTCTTATA

GATTGTAGGTGTTATAGTTACTATTTTTAGTAAAGTCATATTACTATAAAATATTGGGTA

TGATACAATACTATATATCCATTGCTTTTGTGTCCTTAACTTATGGCCTAATAATGAATC

TTTAATACCAGTTAACATCGAGTTTAAGATACTTAACATTGCCATTTCATGAGATGATGC

TACCATACCAGATATATCTAGATACATAGAAATTTCAGAAAGTTCTTGTTGTTTATCTAA

TTCCGTTAAACCAGTTGAAAAAGCCGGAGAGTTAATTTCTACAGATGATCCACTTAAAGG

TAACATATTTTCAATACTTATGATTTCACTTTGTAGAAAACTTGGACCAGCAATAGCAAT

CTGGAATTCTCCTGCTTGAATATTTTTATATGAAAAATAGCATAAAAATCTAGTTATCCG

CATAAAAACTGGACTTATCACACTTTATCAAGGTCAAAACCACTCAATTTACTACTAATT

TACTACTTATGAATGAGCTTTGATACGACGATTTATCCTTGAAAAGTGAAGATATAAAGA

TACTTCCAATAAAATTTGAATATTTAATAGGTAGACACTTCAAAAAATGAGGTGTCTATT

TTTTTACCCGATTTTGAAAGGAAGTGAACTTATGAAAACAAAAAATCAAGAATCAAAAGG

TCGTTCCCCACTCTTTAAGACCATCAAACATTCATTCAGCCAATAAAAAAGAAAGGATAG

GTAAAAATATGGAACTTAAATTTGTGATTCCCAACATGGAAAAAACATTCGGCAATTTAG

AATTTGCTGGCGAGGATAAAGTCGTTCAGCGAAGAATCAACGGACGGCTAACTGTCTTAT

CAAGAAGCTATAATCTCTATTCTGATGTTCAAAGAGCAGATGATATTGTGGTGGTGCTTC

CTGCTGAAGCTGGCGAAAAACATTTCGGCTTTGAGGAACGTGTGAAGTTAGTCAATCCAC

GTATTACCGCAGAGGGCTACAAAATCGGCACTCGTGGTTTTACAAATTACCTTTTACATG

CTGACGACATGATAAAAGAATAAAGAAAGAGAGGAAAAATGATGAGATTAGCAAATGGCA

TTGTATTAGATAAAGACACGACTTTTGGAGAATTGAAATTCTCTGCTCTACGTCGTGAAG

TGAGAATCCAAAATGAAGACGGGTCGGTTTCAGATGAAATCAAGGAACGTACCTATGACT

TAAAATCCAAAGGACAAGGACGCATGATTCAAGTAAGTATTCCTGCCAGCGTGCCTTTGA

AAGAGTTTGATTATAACGCACGGGTGGAACTTATCAATCCCATTGCGGACACCGTTGCTA

CTGCCACCTATCAAGGAGCAGATGTTGACTGGTATATCAAGGCAGACGATATTGTGCTGA

CAAAGGATTCTAGTTCATTCAAAGCTCAACCACAAGCAAAGAAAGAACCGACACAAGACA

AATAGTCGCTAGGTAGAAAGGAGACTTTTTCGCATGAAACAGCGTGGTAAAAGGATTCGC

CCATCTGGTAAAGATTTAGTCTTTCATTTTACGATAGCGTCACTCCTGCCTGTTTTCCTG

CTGGTTGTCGGACTGTTTCATGTGAAGACAATCCAGCAGATCAACTGGCAGGATTTTAAC

CTATCACAAGCAGATAAGATTGACATTCCCTATTTAATTATCAGTTTCAGTGTCGCAATT

CTTATCTGCTTGCTGGTAGCGTTTGTATTCAAACGGGTTCGCTATGATACGGTTAAACAA

CTTTACCACCGTCAAAAACTGGCAAAGATGATACTTGAAAACAAGTGGTATGAATCTGAA

CAGGTCAAAACAGAGGGTTTCTTTAAAGATAGTGCTGGTCGTACAAAGGAAAAGATAACC

TACTTCCCTAAAATGTATTATCGACTTAAAAATGGCTTGATACAGATACGGGTGGAAATC

ACGCTGGGAAAATATCAAGACCAACTCTTACACTTGGAAAAGAAATTAGAGAGTGGCTTG

TACTGTGAGCTGACGGATAAAGAGTTAAAGGATTCCTATGTGGAATATACTTTGCTCTAT

GACACCATAGCCAGTCGTATTTCTATTGATGAAGTAGAAGCTAAAGATGGTAAACTTCGC

TTAATGAAAAACGTATGGTGGGAATATGATAAGCTCCCTCATATGTTGATTGCTGGTGGT

ACAGGTGGCGGTAAAACTTACTTTATACTGACACTGATTGAAGCCTTGCTTCATACAGAT

TCAAAACTGTATATTCTTGACCCGAAAAATGCTGATCTTGCGGACTTAGGTTCTGTGATG

GCAAATGTCTACTATAGAAAAGAAGACTTGCTTTCTTGCATTGAAACATTCTATGAAGAA

ATGATGAAACGTAGTGAGGAAATGAAGCAGATGAAGAACTATAAGACTGGCAAAAATTAT

GCTTACTTAGGTCTCCCGGCACACTTCTTAATCTTTGATGAATACGTCGCTTTCATGGAA

ATGCTGGGAACAAAAGAAAACACCGCAGTTATGAATAAGCTGAAACAGATTGTCATGTTA

GGTCGTCAAGCTGGCTTCTTTCTAATACTGGCTTGTCAACGTCCAGACGCAAAATATTTA

GGCGACGGAATCCGTGATCAGTTTAATTTCAGAGTGGCTTTAGGTCGTATGTCTGAAATG

GGCTATGGCATGATGTTTGGCAGTGACGTACAAAAGGATTTCTTCTTAAAGCGAATCAAA

GGTCGTGGCTATGTTGATGTAGGAACAAGTGTCATATCAGAGTTTTATACTCCCCTTGTA

CCAAAAGGATATGATTTCTTGGAGGAAATTAAAAAGTTATCCAACAGCAGACAGTCCACG

CAGGCGACGTGCGAAGCGGAAGTCGCAGGTGTGGACTGATCTTGCTGGCTGGTGTGGCAA

TAGCCACGCCAGCACTTAACCCCCCGTATCTAACAGGGGGGTACAAATCGACAGGAAACA

GTCAAAAAAACATTAGAAAATCCTTTGGTTACAAGGGATTTACAAAATTTCAGCGTATGT

CAAATGGGCTTTAAAAGTTGACATACGCCTTTTTGATTGGAGGGATTTTTACTGAATGAA

CAAACTTGGTTACAGCATTTAAAAGAAAAACGCTTGGCTTATGGACTATCTCAAAACCGT

TTAGCTGTTGCGACTGGTATTACAAGGCAGTATCTAAGCGATATTGAAACAGGAAAAGTC

AAGCCATCAGAGGATTTACAGCAGTCCCTTTGGGAAGCTCTGGAACGCTTCAATCCCGAC

GCTCCCCTTGAAATGCTGTTTGATTATGTAAGAATTCGCTTTCCGACAACAGACGTACAG

CAGGTGGTCGAAAACATCTTACAACTGAAACTGTCCTATTTTCTTCATGAGGACTATGGT

TTCTATTCTTATTCAGAGCATTATGCTTTAGGCGACATATTCGTCCTTTGCTCCCATGAA

CTGGACAAAGGAGTTCTGGTGGAATTGAAAGGTCGTGGGTGCAGACAATTTGAAAGCTAT

CTTCTGGCACAACAAAGAAGCTGGTATGAGTTCTTTATGGACGTTTTGGTGGCTGGCGGT

GTGATGAAACGCCTTGACCTTGCCATTAACGATAAGACAGGGATTTTAAATATCCCTGTA

CTCACTGAAAAGTGCCAACAGGAAGAATGTATCTCCGTCTTCCGCAGTTTTAAAAGCTAT

CGCAGTGGCGAACTGGTACGCAAAGAGGAAAAGGAATGTATGGGAAACACCCTCTATATC

GGTTCATTACAAAGTGAAGTTTATTTCTGTATCTATGAAAAGGACTACGAGCAGTACAAG

AAAAATGATATTCCCATTGAAGACGCAGAAGTAAAAAACCGTTTTGAGATTCGATTGAAA

AATGAGCGTGCCTATTATGCAGTCCGTGATTTACTCGTCTATGACAATCCAGAGCATACC

GCCTTTAAAATTATCAATCGGTATATCCGTTTTGTAGATAAAGACGATTCCAAACCTCGT

TCTGATTGGAAACTGAATGAAGAATGGGCTTGGTTTATTGGGAACAATCGTGAACGATTA

AAACTAACCACAAAACCAGAGCCTTACTCCTTCCAAAGGACGCTGAACTGGCTATCTCAT

CAAGTTGCCCCGACCTTAAAGGTTGCGATTAAACTTGATGAAATCAACCAGACGCAGGTT

GTAAAAGACATTCTCGACCATGCGAAACTGACAGACCGACACAAGCAGATTTTGAAGCAA

CAGTCAGTAAAAGAACAGGACGTGATAACAACAAAAAAATAACTCAAATACAAATTCATT

GAATATAGAGAGGAGAACATTTTTATGAATTTTGGACAAAACCTTTATAACTGGTTTCTA

TCAAACGCTCAATCACTGGTGCTTTTAGCAATCGTTGTGATTGGCTTGTATCTTGGCTTC

AAGCGTGAGTTTAGCAAACTGATTGGCTTTTTAATTATTGCGATTATTGCGGTTGGCTTA

GTCTTCAACGCTGCTGGAGTAAAAGACATTTTACTAGAGCTATTCAATCGCATTATTGGT

GCTTAAATAAAACCGTTCTTTTGTGGAATATAAGTGGTTTTCTTATGTTCCGCAAAGGAA

TGGTACACCAAACGAAGTGCGGTAGGGATTTTTGAATCTCTACAAAGAAAGGACGTGAAT

ATATGGACGATATGCAAGTCTATATTGCGAATTTAGGCAAATACAATGAGGGCGAATTGG

TCGGTGCGTGGTTTACCTTTCCCATTGACTTTGAGGAAGTCAAAGAGAAAATCGGCTTGA

ATGATGAATATGAGGAATACGCCATTCATGACTACGAGTTACCCTTTACGGTTGACGAAT

ACACTTCCATTGGCGAACTCAATCGACTATGGGAAATGGTATCGGAATTACCCGAAGAAT

TACAATCGGAGCTATCTGCTCTGCTCACTCATTTTTCAAGCATTGAAGAACTAAGCGAAC

ATCAAGAGGATATTATCATTCATTCCGATTGTGATGATATGTATGACGTGGCACGCTACT

ACATTGAAGAAACGGGTGCTTTAGGCGAAGTACCAGCTAGTCTTCAAAACTATATTGATT

ATCAAGCCTATGGTCGGGATTTAGACCTTTCAGGAACGTTTATCTCAACCAATCATGGGA

TTTTTGAAATCGTCTATTAAATCTGTCGGTACATTACTACTGGCAGATTTTCTATTTTAC

GGGGTGGCTCAATCAGCTACCCCTATTTTTTATGAAAGGATTGATTACATGAAGAAAATA

CGAAGCTATACCAGTATCTGGTCTGTGGAAAAGGTACTGTATTCTATCAATGATTTTAGA

CTTCCGTTTCCCATAACCTTTACGCAAATGACATGGTTTGTCGTGTCACTCTTTGCAGTG

ATGATACTTGGCAACTTGCCCCCTCTTTCCATGATAGAGGGAGCATTTCTCAAATACTTT

GGGATTCCTGTGGCTTTCACATGGTTTATGTCTACAAAAACTTTTGATGGTAAAAAGCCT

TATGGATTTTTGAAGTCTGTCATTGCTTATGCACTGCGACCAAAGCTGACCTATGCAGGA

AAAAAGTAACGCTTGGCAGAAACCAGCCACAAGAAGCCATTACAGCAGTTAGGAGTGAAT

TTTATGGCATATCCAATTAAATACATTGAAAACAATCTCGTCTGGAATAAAGACGGGGAA

TGTTATGCTTACTATGAGCTTGTTCCTTACAATTACTCATTTCTAAGTCCAGAACAGAAA

ATACAAGTGCATGATTCTTTCAGACAGCTTATCGCACAAAATCGTGATGGCAAAATTCAT

GCTTTACAAATCAGTACAGAATCCAGCATACGTTCTGCACAAGAGCGTTCCAAAAATGAA

GTCACTGGCAAGCTCAAAGCGGTTGCCTATGACAAAATCGACCAACAGACAGACGCTTTA

ATATCCATGATTGGCGAAAATCAAGTGAACTACCGTTTCTTTATCGGCTTTAAGTTGCTT

CTCAACGATCAGGAGTTTTCTATGAAAAGTCTTACCGTTGAAGCAAAAAATGCTTTGTCT

GATTTTGTCTATGATGTGAACCATAAGCTGATGGGCGATTTTGTTAGTATGAGTAATGAT

GAAATCCTGCGTTTTCAGAAGATGGAAAAGCTCTTAGAAAATAAAATCTCTCGTCGTTTC

AAAATCCGCAGGTTAGATAAGGACGACTTCGGCTATCTGATTGAACACCTTTACGGACAG

ACAGGCACTGCCTATGAAGAGTATGAGTACCATCTATCAAAGAAAAAGCTGGATAATGAA

ACGCTGATTAAATACTATGACTTGATTAAGCCTACTCGCTGTTTGGTGGAAGAAAAACAG

CGATATTTGAAAATCCAGCAGGAAGATGAAACCGTCTATGTAGCTTACTTTACCATTAAC

AGCATTGTCGGAGAACTGGACTTCCCGTCCTCTGAAATCTTCTACTACCAGCAACAGCAA

TTTACATTCCCGATTGATACGTCAATGAATGTGGAAATTGTAGCGAATCGTAAAGCCCTA

TCTACTGTCCGCAATAAAAAGAAAGAACTGAAAGACTTGGATAACCACGCTTGGCAAAGT

GATAATGAAACCAGCTCCAATGTGGCGGAAGCTCTGGAAAGTGTGAATGAGCTGGAAACC

AATTTAGACCAAAGCAAGGAATCTATGTACAAGCTGTCTTATGTGGTAAGGGTATCAGCA

AATGATCTTGACGAACTCAAACGTCGTTGTAATGAAGTGAAAGATTTTTATGACGATTTA

AGCGTAAAACTGGTACGACCATTTGGGGATATGCTCGGCTTACATGAAGAATTTTTACCT

GCCAGCAAGCGTTATATGAATGATTATATTCAATACGTGACCTCTGATTTCCTCGCTGGT

TTAGGTTTTGGTGCTACTCAAATGCTGGGGGAAAATGAGGGGATTTATGTTGGCTACAGC

TTAGATACTGGACGCAATGTCTATCTGAAACCTGCTCTTGCCAGTCAAGGGGTTAAGGGT

TCAGTAACCAATGCGTTAGCGTCGGCTTTTGTTGGTTCGCTGGGTGGTGGTAAATCCTTT

GCGAATAACCTTATCGTCTATTATGCGGTGCTTTATGGGGCACAAGCAGTGATTGTAGAC

CCAAAAGCAGAACGTGGCAGATGGAAAGAAACCTTGCCAGAGATTTCCCATGAAATCAAT

ATCGTCACTCTGACTTCTGATGAGAAAAACAAAGGCTTACTTGACCCTTATGTGATTATG

AAAAATCCCAAAGATTCTGAATCACTGGCTATTGATATTCTGACATTCCTTACGGGGATT

TCCTCTCGTGATGGGGAACGCTTCCCAATCCTTAGAAAAGCCATTCGTGCAGTAACCAAT

AGTGAAGTACGAGGGTTGATGAAAGTGATTGAGGAATTACGGGTTGAGAATACGCCACTA

AGTACCAGTATAGCCGACCATATCGAAAGTTTTACAGACTATGACTTTGCACATTTATTA

TTCAGTAATGGTTATGTGGAGCAGTCTATCAGCTTAGAAAAACAACTGAACATTATACAG

GTTGCGGACTTGGTACTTCCCGACAAGGAAACTTCCTTTGAGGAATATACCACTATGGAG

CTTTTATCCGTTGCTATGCTGATTGTCATTAGTACCTTTGCTTTAGACTTTATCCATACA

GACCGAAGCATTTTCAAGATTGTAGATTTAGACGAAGCATGGAGCTTTTTACAGGTAGCA

CAAGGAAAAACACTATCTATGAAGCTGGTTCGGGCTGGTCGTGCTATGAACGCTGGGGTA

TATTTCGTGACCCAAAATACAGACGACCTCTTAGATGAAAAACTGAAAAATAACCTCGGC

TTAAAATTTGCATTTCGTTCCACTGACCTTAACGAGATTAAAAAGACCTTAGCCTTTTTT

GGTGTAGACCCAGAGGACGAAAACAATCAGAAGCGATTGCGTGATTTGGAAAACGGGCAA

TGCCTTATCAGTGATTTATATGGTCGTGTCGGTGTGATACAGTTCCACCCTGTATTTGAA

GAACTGCTCCATGCCTTTGATACCAGACCACCTGTGCGAAAAGAGGTGTAAATGTGAAAC

CATCAATAGTAAACAGAATAAAATCAAACTGGACGCTGAAACGTCTAGGTAAAGTGGCAA

TGACAGTGGCTTTCACACTTGTGATTGCCATTTTTCTTTTAGCCATGCTGGGAACGGTGG

TTCAAGCTGCGGGCTTGGTAGATGATACGGTCAATGTGGCAAATGAATACAGCCGATACC

CACTTGAAAACTATCAACTGGATTTTTATGTGGATAATAGCTGGGGCTGGCTTCCGTGGA

ACTGGTCGGACGGGATTGGAAAACAGGTCATGTATGGACTATATGCCATTACCAATTTTA

TTTGGACAATCAGTTTGTATGTTTCCAATGCGACAGGTTACTTAGTACAGGAAGCCTATT

CCTTAGACTTCATTTCCGCTACAGCAGATTCCATTGGTAAGAATATGCAGACCTTAGCTG

GTGTGAGTGCAAACGGATTTTCAACAGAGGGTTTCTATGTTGGATTCCTCTTACTCTTGA

TTTTGGTTCTTGGGGTTTATGTTGCCTATACGGGACTGATAAAGAGAGAAACCACAAAGG

CAATTCATGCCATTATGAATTTTGTGCTGGTGTTTATCCTATCGGCTTCCTTTATTGCCT

ACGCTCCCGACTACATTAAAAAAATCAATGACTTTTCATCAGACATCAGTAATGCCAGTT

TATCACTTGGCACGAAGATTGTCATGCCCCATTCCGATAGTCAAGGCAAGGACAGCGTGG

ACTTAATCAGAGATAGCCTGTTTTCCATACAGGTTCAGCAACCGTGGCTACTGCTTCAAT

ACAACAGTTCAGACATTGAAAGTATCGGTATTGACCGTGTGGAAAGCCTGCTCTCCACCA

GCCCAGATTCCAACAATGGCGAAGACAGAGAAAAAATTGTTGCGGAAGAAATTGAAGACA

GAAGCAATACCAATCTAACCATTACAAAGACCATTAACCGTTTAGGTACAGTCTTCTTCC

TATTTGTCTTCAATATTGGGATTTCCATATTTGTATTCCTATTAACAGGAATCATGATTT

TCTCGCAGGTACTTTTTATCATCTATGCTATGTTTCTGCCTGTGAGCTTTATTTTAAGCA

TGATTCCATCATTTGATGGTATGTCAAAACGAGCCATAACAAAGCTCTTTAATACCATTT

TGACACGAGCTGGAATCACATTGATTATTACGACAGCATTTAGTATTTCAACCATGCTCT

ATACCTTATCGGCTGGTTATCCGTTCTTTTTGATTGCTTTTCTACAGATTGTGACCTTTG

CAGGAATCTACTTCAAGCTGGGCGATTTAATGAGTATGTTTTCTCTACAGAGTAACGATT

CTCAAAGTGTGGGAAGTCGTGTGATGAGAAAACCTCGTATGCTTATGCACGCTCACATGC

ACCGTCTACAGCGGAAACTTGGACGTTCCATGACTACTCTAGGGGCTGGGTCTGCCATTG

TTACAGGTAAAAAAGGACAGTCGGGTTCGGGGAGTTCTGCAAGGACACAAGCAGATCACT

CCCGACCAGACGGAAAGGAAAAATCAACACTTGGAAAACGTATCGGTCAAACCATCGGTA

CAGTAGCTGATACCAAAGACAGAATGGTAGACACTGCTAGTGGTTTGAAAGAACAGGTTA

AAGATTTGCCGACCAATGCAAGATATGCAGTATATCAAGGAAAATCCAAAGTAAAAGAGA

ATGTCCGTGATTTAACCAGTAGTATTTCTCAAACCAAAGCGGACAGAGCCAGTGGACGCA

AGGAACAGCAGGAACAAAGGCGAAAAACCATTGCGAAGCGTCGCTCTGAAATGGAACAGG

TCAAACAGAAAAAACAGCCTGCTTCTTCTGTTCATGAAAGACCGACTACAAGACAAGAAC

AATATCATGATGAACAGACCTCAAAACAGTCTAATATTCAGACTTCATATAAGGAATCTC

AACAAGCCAAACAAGAGCGTCCAGCAGTTAAGTCCGATTTTTCAAGTCCAAAAGTGGAAC

GCCAAGGCAATACCGTTCAAGAAAAAACCGTTCAAAAGCCAGCAACTTCAACCACTACAG

CAGATAGAACTTCACAACGTCCAATCACAAAAGAACGTCCGTCTACTGTTCAAAGAGTAC

CACTACAAAATACAAGAAGTAGACCACCAATCAAAACCGCCACCATTAAGAAAGTCGGTA

AGAAACCATGAAGTTGAAAACTTTAGTGATTGGTGGTTCTGGATTATTCTTGATGGTCTT

CTCACTGCTTCTGTTTGTTGCCATTTTATTTTCAGATGAACAGGACAGCGGAATTTCCAA

TATTCATTATGGAGGTGTGAATGTTTCCGCAGAAGTGCTGGCTCATAAGCCTATGGTAGA

AAAATATGCCAAAGAATATGGCGTTGAAGAATATGTCAACATACTTCTTGCGATTATACA

GGTGGAATCGGGCGGTACTGCGGAAGATGTTATGCAGTCCTCGGAATCCCTCGGTCTTCC

ACCTAATTCATTGAGTACAGAAGAATCCATTAAGCAAGGTGTGAAGTATTTCAGTGAATT

ATTAGCCAGTAGCGAAAGGCTCAGTGTAGATTTAGAATCGGTTATCCAGTCCTACAATTA

TGGTGGTGGTTTCTTAGGGTATGTGGCTAATCGTGGAAATAAATATACCTTTGAACTGGC

TCAAAGTTTCTCAAAAGAGTATTCAGGTGGCGAAAAAGTGTCTTACCCCAATCCCATAGC

CATACCTATCAATGGGGGCTGGCGATACAACTATGGCAATATGTTTTATGTGCAACTGGT

AACGCAGTATCTTGTCACAACAGAGTTTGATGATGATACGGTACAAGCCATCATGGACGA

AGCACTGAAATATGAGGGCTGGCGATACGTTTACGGTGGAGCTTCCCCGACTACTTCTTT

TGATTGTAGCGGACTGACACAATGGACGTATGGAAAAGCTGGAATTAACTTACCACGAAC

TGCACAACAGCAATATGATGTGACCCAGCATATCCCACTATCGGAAGCACAAGCTGGCGA

TTTGGTTTTCTTTCATTCTACCTATAACGCTGGCTCTTATATTACTCATGTTGGGATATA

CCTTGGCAATAACCGTATGTTTCATGCAGGCGACCCAATCGGTTATGCCGACTTAACAAG

CCCCTACTGGCAACAGCATTTAGTGGGAGCAGGACGAATCAAACAATGAGAAAGGAAGAT

TTAATGATGAAATTTAGAAAAAATCAGAATAAAGAAAAACAGATACCAAAGGAAAAGAAA

CCTCGTGTCTATAAGGTCAATCCTCATAAAAAGGTTGTGATTGCCTTGTGGGTACTTTTA

GGGCTTAGTTTCAGCTTTGCGATATTCAAGCACTTTACAGCTATAGATACTCATACTATT

CACGAAACAACTATCATAGAAAAGGAATACGTTGATACTCATCATGTAGAAAATTTTGTA

GAGAACTTTGCGAAAGTCTACTATTCATGGGAGCAATCCGATAAGTCCATTGATAATCGA

ATGGAAAGTCTAAAAGGCTATCTGACAGATGAACTTCAAGCTCTCAATGTTGATACAGTA

CGCAAAGATATTCCTGTATCGTCTTCTGTAAGAGGATTTCAGATATGGACGGTAGAGCCA

ACTGGCGACAATGAGTTTAATGTAACCTACAGTGTAGACCAGCTCATTACAGAGGGAGAA

AATACAAAGACCGTCCACTCTGCTTATATAGTGAGTGTCTATGTAGATGGTTCTGGAAAT

ATGGTACTGGTTAAGAATCCGACCATTACCAACATACCTAAGAAATCAAGTTATAAACCA

AAAGCCATTGAAAGTGAGGGGACGGTTGATTCCATTACAACCAATGAAATCAATGAGTTT

TTAACGACGTTCTTCAAGCTCTATCCTACAGCGACAGCCAGTGAACTTTCCTACTATGTG

AATGACGGGATATTAAAACCAATCGGAAAAGAGTACATCTTTCAAGAACTGGTAAATCCT

ATTCACAATCGTAAGGATAATCAAGTCACGGTATCGCTGACAGTGGAGTATATCGACCAG

CAGACCAAAGCAACGCAGGTATCTCAATTTGATTTGGTACTTGAAAAGAACGGGAGTAAT

TGGAAGATTGTAAAATAACAAATATTGGTACATGATTACAGATACTTTGTAATCATGTAC

TCTTTTTGATAAAAAATTGGAGATTCCTTTACAAATATGCTCTTATGTGCTATTATTTAA

GTGACTATTTAAAAGGAGTTAATAAATATGCGGCAAGGTATTCTTAAATAAACTGTCAAT

TTGATAGCGGAAACAAATAATTAGATGTCCTTTTTTAGGAGGGCTTAGTTTTTTGTACCC

AGTTTAAGAATACCTTTATCATGTGATTCTAAAGTATCCAGAGAATATCTGTATGCTTTG

TATACCTATGGTTATGCATAAAAAATCCCANNNNNCACACACTTAATTAATTAAGTGTGT

GNNNNNTATTTATCACTGGGATTTTTATGCCCTTTTGGGTTTTTGAATGGAGGAAAATCA

CATGAAAATTATTAATATTGGAGTTTTAGCTCATGTTGATGCAGGAAAAACTACCTTAAC

AGAAAGCTTATTATATAACAGTGGAGCGATTACAGAATTAGGAAGCGTGGACAAAGGTAC

AACGAGGACGGATAATACGCTTTTAGAACGTCAGAGAGGAATTACAATTCAGACAGGAAT

AACCTCTTTTCAGTGGGAAAATACGAAGGTGAACATCATAGACACGCCAGGACATATGGA

TTTCTTAGCAGAAGTATATCGTTCATTATCAGTTTTAGATGGGGCAATTCTACTGATTTC

TGCAAAAGATGGCGTACAAGCACAAACTCGTATATTATTTCATGCACTTAGGAAAATGGG

GATTCCCACAATCTTTTTTATCAATAAGATTGACCAAAATGGAATTGATTTATCAACGGT

TTATCAGGATATTAAAGAGAAACTTTCTGCCGAAATTGTAATCAAACAGAAGGTAGAACT

GTATCCTAATATGTGTGTGACGAACTTTACCGAATCTGAACAATGGGATACGGTAATAGA

GGGAAACGATGACCTTTTAGAGAAATATATGTCCGGTAAATCATTAGAAGCATTGGAACT

CGAACAAGAGGAAAGCATAAGATTTCATAATTGTTCCCTGTTCCCTGTTTATCACGGAAG

TGCAAAAAACAATATAGGGATTGATAACCTTATAGAAGTGATTACGAATAAATTTTATTC

ATCAACACATCGAGGTCCGTCTGAACTTTGCGGAAATGTTTTCAAAATTGAATATACAAA

AAAAAGACAACGTCTTGCATATATACGCCTTTATAGTGGAGTACTACATTTACGAGATTC

GGTTAGAGTATCAGAAAAAGAAAAAATAAAAGTTACAGAAATGTATACTTCAATAAATGG

TGAATTATGTAAGATTGATAGAGCTTATTCTGGAGAAATTGTTATTTTGCAAAATGAGTT

TTTGAAGTTAAATAGTGTTCTTGGAGATACAAAACTATTGCCACAGAGAAAAAAGATTGA

AAATCCGCACCCTCTACTACAAACAACTGTTGAACCGAGTAAACCTGAACAGAGAGAAAT

GTTGCTTGATGCCCTTTTGGAAATCTCAGATAGTGATCCGCTTCTACGATATTACGTGGA

TTCTACGACACATGAAATTATACTTTCTTTCTTAGGGAAAGTACAAATGGAAGTGATTAG

TGCACTGTTGCAAGAAAAGTATCATGTGGAGATAGAACTAAAAGAGCCTACAGTCATTTA

TATGGAGAGACCGTTAAAAAATGCAGAATATACCATTCACATCGAAGTGCCGCCAAATCC

TTTCTGGGCTTCCATTGGTTTATCTGTATCACCGCTTCCGTTGGGAAGTGGAATGCAGTA

TGAGAGCTCGGTTTCTCTTGGATACTTAAATCAATCATTTCAAAATGCAGTTATGGAAGG

GATACGCTATGGTTGCGAACAAGGATTATATGGTTGGAATGTGACGGATTGTAAAATCTG

TTTTAAGTATGGCTTATACTATAGCCCTGTTAGTACCCCAGCAGATTTTCGGATGCTTGC

TCCTATTGTATTGGAACAAGTCTTAAAAAAAGCTGGAACAGAATTGTTAGAGCCATATCT

TAGTTTTAAAATTTATGCGCCACAGGAATATCTTTCACGAGCATACACCGATGCTCCTAA

ATATTGTGCGAACATCGTAGACACTCAATTGAAAAATAATGAGGTCATTCTTAGTGGAGA

AATCCCTGCTCGGTGTATTCAAGAATATCGTAGTGATTTAACTTTCTTTACAAATGGACG

TAGTGTTTGTTTAACAGAGTTAAAAGGGTACCATGTTACTACCGGTGAACCTGTTTGCCA

GCCCCGTCGTCCAAATAGTCGGATAGATAAAGTACGATATATGTTCAATAAAATAACTTA

GTGTATTTTATGTTGTTATATAAATATGGTTTCTTGTTAAATAAGATGAAATATTTTTTA

ATAAAGATTTGAATTAAAGTGTAAAGGAGGAGATAGTTATTATAAACTACAAGTGGATAT

TGTGTGCTGAGAGCTTTCTTCTATACTAATAGACGAAAGGGTGTGAAAATGATTTTTAAA

TGATACTGTGGAACGGAACAGTAGCCCTAGTATTGACTACTGTCGTTTCTATTCATATTG

GCTATTCTAGGACTGAGATGAAAAAATCTATAAATGCTCAGAATAAAATTGAACCCGCAA

ATCTCCCCAAAACAATGGTGAGTCATGTACTTGTATTATTCCGAAAAAATACACCTCTGG

TGCAGTGAGACAAATTGGTGTATCTTATAGTGGCTTCGTAGATGAAAGCTATACTCTACT

ATCACTCTTTGATGATGTAGAACAAATTGAAAAAGATAATAGACTTCAGACAGCTATTGA

TGTTGTCAGAGAACAGTTTGGTTTTTTAGCCATACAAAAAGGAACCGTCCTAACTGAAGG

TTCCAGAAATATTGAACGCAGTAAACTTATCGGTGGTCATTCCGCGGGTGGATTGGAGGG

ATTAAAATGAAACAAGAAAAAAATACAGTACAATTTTCAGAAATCCGTAGCAAAGGATGT

AATGATATTGAAATGCTTGAAAGATTTTTACATGGAATCGTTGAAACAGCAACTTCAAAA

CTTCGTCAGAGAAAACTCAAAACAACTGAAATATCGATACGACTAGTACATGCTAAATCT

GAAAACCGATTACCATTGGAATTTACATTTAGCATTAAGCCAACAAGCTCATCTGTGATA

ATCTATACTGAGGTAATCAATCGCTTTAAAGAATGTTACACAGGTGGGGGAATTCAAGGT

TTTACGATTCAATTTGATAAAAATACCCTTGCCTCTGCATAGAAAGGATTTGATATGATT

GACCGTTCATATTTACCATTTCAATCAGCAAGAGAGTACCAGGATACAAAGATGCAAAAA

TGGATGGGCTTTTTCCTATCTGAACATGCATCAGCACTCTCTGATGATACAAACAAAGTA

ACGTACATGTCTGACTTATCACTAGAGAAGAAATTATTACTCCTCAGTCAAGTATACGCC

GGGCAGCTACGCACACGCATTCAAGTGATTGAAAAAAACAAGCGTGTTTCCTACACTGGA

ACAATACCAAGTCTGACCAAAGATTTCATTTTGATAAAAACTACAACAGGTCACATCAAT

TTGAAATTAAAAGACATTATTAGTATTGAACTTGTCGAGGAGGTGCTCTATGAATCAGCT

TGAGTTTCAGCGTAATCACCTACAAATGGACTATTATAGCGAGAGCTACCAAGATTTTGA

ACGTGACTTCTACCGCTACTCTAACATGAATATTCCATTGACCTTCCTAACTGATGATAT

CCTAAAAACAATGGCGACTTCACGTAAGAATTACTTTGTCCTCAATAAGGAAAAGTCCAG

AGATAACCGCGATCACTTCTTCATATTTGAAGTAAGTACCGTAGATGAGAATCCGCTAAT

CTATCATTATACATATAAGAAAACTACAATATATTTAGCAGAAAAATAGGAGCAGTTCAA

TTGACTGTTCCTATTTTTAATATTCATAAAATCTAAAGTCTTTATACTCTTTAACAATGG

AGTCGCCAACCAGAACAGACTATACTGACCAGCGACTACCTTAAATTTAATGTTTCAGAT

TTATTTTCTTATCTCTAATTTCATAAACTACATCTGCTACATTTTCGAGTAATCGTTTAT

CGTGGGTGATAAACACGATAGTTCCGGTGTACTCCTTCATTAGTATTTCCAAAGCCTCTA

AACTTGGTATGTCAAGGAAGTTACTGGGTTCATCCATTATTAGGATGTTATATCTACCCA

TGAGCATTTTAGCAAGCAACAATTTTATAATTTCTCCACCGCTTAAAACAGATAAACTTT

TTCCAATATCGTTCTGTTTGAACCCCATAGATGCTAGCACTGAACGAATTTCTGATATAT

TGTAGTCACAATCCTTCTGCATAAACTCCATAACATTCTGATTACTGTTGTACTTGTAAC

CATTCTGTGCAAAGTAACCTATTTTTGCCTTAGGCGAAATAGAAATTCCTTCTTCATGGT

TTAAGATCATTTGGATTAAAGTTGTTTTTCCGATTCCATTACCACCAGTTAACGCCACTT

TTGCTCCTAACGGAATTTGAAAAGATGCATTTTCAAACAGAGCCTTATCCCCAAATACTT

TATTAATTTCTGCACCGACTATAGGGTATGGATTATGGAGCTCCAATGCTTTACTTTGCC

TGAAACGAATTCTGCGAATGCCTTCCGGAGCTTCTACTTTTCCTAAGGCCGCAATCCTGT

GCTCTAGGGTTTTAGCAGCATTATACATCTTTTTTTCCTTACTTCCTATTGATTTTTGAT

GAGCTAAACGCCCTCCGTCTTCAGTACTTTTTTTCTTTGAAGAACCTTTTGCCTTCTGTT

CTATTTTACGAGCCTGTTTTCGCTTTTCCTCCGCAGCCCTTTCCAATCGGGCACGTTCCG

CAATAAATTGTTCGTATTCTGCAGCTTGGCTCTTACGTTCTTCCTCTTTCTGACGAAGAT

AATCAGAATAGTTTCCCCAATACTCAGTGATTTTGCCATCTTTCAGTTCCCATATTTTAT

CTACTATTTCATCAAGAAAATAGCGGTCATGGCTAATAACTAACAGTGCACCTGTAAAAT

ATTTTAGCTGTCCTATTAGAAAATCAATTCCTTCACGGTCTAAATGGCTCGTAGGTTCAT

CCGCTAAAATACCATGAACCTGTGCCGATAAGGCCTGTGCTATTTTAAGCCTTGTTTCTT

CACCACCGCTCATAGTCTGTATATTTAATTGCTCAACACCTAGCTTGCCTACAAGTGCAA

AATCTTTTTCCTCCTGCAGAGTTACTTCGTCCAACTGGGGAATATAGGCAAGTTCACCCA

GACGATTCATTTTACATCCTGGGGGAGTTAATTCTCCTAAAAGTACCCTGAGTAAAGTGC

TTTTTCCAGCACCATTTGCTCCTACTAAACCAATACGGTCATAATCATATACTTCTAATT

CATTTATATCTAAAACATCGCGTCCTTTGAATTCCACACGAATGTCTTTTGCTTTTAATA

TTAATTCCATAACATTTCCTCCTGTCTATAATCGCATGCTTTCATTTGCTTGTATGCAGG

GAAAACCCTGCGATTTTAGCAGGAAGAGTTACATGAAAATAAGATACATAAATATTCCTC

CAATATTGTTTATTTTAAATCTAATTTTCTAACCTCAGTTATCATTTGGCAAACTATAGC

AATGCCAATAATTAAAATACCTGATAGTAAAAACCAATGATTTACACCGATTTTATCAGC

AAAGAATCCAGAAAGAATTAACCCAATTGGCATAGCAAGTGACATGATACTTCCGATCAA

AGAAAATACACGTCCTAAATATTCAGGCTTAATTTTCTCCTGAAAAAGAGCTGTTTGCAC

ACCGCTATAAAATGGCACCGAAAGCCCCATTATTGCACAGCAAACTACGAATATTACAAA

TCCATTTGGAGGAAGTATTCCCGAAACGGCTAAACTGGTCCCCATTATAAAAAATGAACT

TGTTATTAGTAATACATGCTTTTCGAAGCCCCCTAATCTTCCTAATAATAAGCCTCCTGC

TAGCATCCCAAATGCAAAGGAAATTTCCGTAATAGAAATATGCACAGGCGTTCCATTAAA

GTGTTCCATGCTTATTAAAGGAAATAGTGCATTGATTGGCATATAAACAAAAGTATATAG

TGTTCCTAAGAGTAATAAGGCAAACAATCCTTTGTTTTGTCTCAGAACCACAACTCCTTC

TTTCATCTCCCTTATGAAATTTGGTTCTAAACTTTGCACTTGATTACCCAGCTTAGGTAT

ACGTACAATTGCTACCGTAATAGATGCAATCACAGCACCCAATACGTCGATGGCAATAAT

AGCATTTAAATCCCAAACGGAGTATAAGAGTGCTGCAACTGCCGGACTAACAATATAGCT

TATAGACTGCAAAGACTGACTATAGCCTGCGCATTTCGTTAGCTGTTCTTCTGGTACTAA

AAGTGGTGTAACCGCATTGAGTGCTGGGGTATGAAAAGCTGTTCCAATGCTACGGATAAA

CAATACTATCATAATCATCCAGACAGGTAGCTCCATACAGAATGCAACAATAGCAAGCAC

TGCACCAGCTGCTGCGATAATTAAATCGGCACCAATCATTATCTTCTTCCTATCATGACG

ATCCACTAGCACACCAATGGCAGGTCCCAAAATCGCATAGGGTAAAAAACCTACTAATGA

AGCCATAGACAAGACCATCGCAGATCCTGTTTTTTCTGTAAGGTAAAAAATAATCGCCAT

TTGCAGGATGGCACTAGTGATTAATGATACTGCTTGCCCTGCCCATATTGCATAAAATTT

TCGTTTCCAATTGTTGTATTTTTCCATTTATATTATCTCCTGCATATTATTTTGCTTGAA

TTTCTATTTTGAATAGCATTCTAGGCAATAAAAAATGCAGGCCAAACCCCACAATGTGGC

TTTTGGTCTGCATACATACAATTTGGAAACATTCATATTAAAGACATAGTTAAATAAAGG

TATAGTTAAATAACCAATATCCTCACCGTAACTAATGAATGCTCAATATCGTATAAATAA

GCACAACAAAAAAGCCTATCATCGGGTATAGATTCTGCTTTTTTTATTGCCAGCTTATCT

TAAACGCATTGAGGCTGTCATAGTTTCGGTTCCTCCTACATCTTTGTTTATATCAATTTA

TAGTATAACACAACAAGATGATATGTTCAATATAAAAGTTATGGAATGAGACTCATACTT

CCAATTCGATGCCAGATTTAAAGGATATGACGAAGTTTTCTTCATAGACTGTAACGCTCT

GGATTATCTTCCTTAGTAGCAAGCGATTAGCTTTCACAAAATCTTCTGTTTGTAGTTTTA

AAAATTCATCAGGATTTTCTAACTCAACCTCAAAATATTTCATTTTACATTCCCTCATTT

CATTTATTGATAAATTGAGTTTGCAAAAAAGAGTGGACAATTTTTGTCTACTCTTAACCT

TTAAAATAGTTTTTTTTAATCGATTTGAAGTTGCCTAAATTATTACTTATTCGGTAAAAT

GAAGTATTGCTTTCAACAGATTTCCTTCAACTACACTTCACTTGATTCAAACAAGGTGGG

TACATTTCTATTCCCACAAACTCCTTGTCAATGGAAACAAACACGTACCCACAGGGTAAA

TGGAAATAGAAACTGATAATTTCTAGCTATCACTTCTACTCATTCCAAAAATTTTCTCAC

TCTGATACTTACCCACCATAAAGCAAAAAGCCTTGCAATCAAGGCTTTCATTATCCCTTT

CGTTCAAAGGTTTCTAAGCTTTTACGAGCAGAGCGACACACTCAGCGGTTCGCTATCTCC

GTTCTGTCTGCGTGCTAGCACTTGTCAATCACGGACAGCTATCGCATGGGCGGAAGTAAA

TGCTAATCTTCGTCGTTTTACTCCTTGACTAGCAAACTTACCGCCTCAACATGTCCTGTA

TGTGGAAATAAAACACGATTAAAGATAAGGGAAGATACTGAATTAAAAAAATTCCCCCTC

TATTGTCCGAAATGCAGACAAGAAAATTTAATTGAAATAAAGCAGTTCAAAGTAACTGTG

ATTACAGAGCCAGACGCAAAGACGCAGAGCCGATAAAATGAGATTAATACAATCTCATTT

TATCGGCTCTTTCCGTTATGTATGGATTCTTTTAATTAGTCTTCGATGTTTCTTGCTTCG

TTGATACCGCTGGCTAAAGATTCCATTAAGGATAGTTCTTTGTCTGTAAAGCTATCCATG

TATTTCTCTATCTGTAATCGTCGGGTGCTTTTTACCAAGTTATTAGCAGGTAAGAAAAAT

TCATCAACGGAAACATGAAGTAACGATACAAGGTCATAAAGAACTTGTATGCTGGGGTGT

TGCCCTTTATTTTCAATATTAGTTAAGTACCGTGGGTCAATTTCAATCAATGCTCCCACT

TGTTCACGAGTTAAACCTCGTTTCAATCGAGCTTCTTTAATGGCTAAACCAAAGGCTCTA

AAATCATATTTATCTTCTTTTTTACGCATAGTAGACCACCTCTATACATTTTATTGTTCC

TACTGAATTAAAAACAGGTATAGAAAAACGTGTTATATGGTTTATAGGTTTATATTTAAT

AAAAAGCACTACTAAACGCCAATAAAAAAACCGTTATATGGTAGTGCTATTTACGCTGTT

AAAATATTGTATATTACTTCCAAATGGCGGTTTGTTGGAGGTCAACGTCGCCATGAAGTA

CATCATATACAATAAATTTCCTTACATTGGGTTCTTGTCAAAAAAAGTCGTCTATCTGCA

ATAGATAAGTACGTCCACCAATGTGGTTTTATAAATCATATAGATAGAATAACAGAAGCA

TGTAAACAGAGAAATAAATCTGTTTATATGCTTTTTTGGCTATTCAGAACTTTTTTACAA

AGTTTATTTATCAGTAATGCAACAAATCCCCCTTTCACATTGGGACTAAGAGTGAAAGGA

GATAAACGAGCAAGGCTCACTTCCTTTCCTAGACAGAAAGGGGGTGAGAAACATGAAACC

ATCTTCTTTTCAGACCACAATAGAAAATCAGTTTGACTATATCTGTAAACGTGCTATGGA

AGACGAGCGAAAGAATTATATGCTTTATCTTTCAAGGATTGCAAAGCGTGAGGTGTCCTT

TTCGGATGTTGGCGATTATCTTGTTAGCCAGTTTGCGACAACAGATAACTATTCAACTGA

CTTTCAGATTTTTACACTCAATGGGTTATCAGTAGGCGTTGAAAATGATTTGTTGAGTGA

AGCATTACGTGAGTTGCCAGACAAGAAACGTGAAATTCTACTGCTGTTTTACTTTATGGA

CATGAGCGATTCAGAAATTGCAGACCTGTTGAAATTGAACCGTTCTACTGTCTATCGGCA

TAGAACCAGTGGACTAGCCTTAATTAAAAAGTTTATGGAGGAATTTGAAGAATGAAAACA

CAATATCCTATGATTCCCTTTCCTCTCATTGTAAAGGCAACAGATGGCGATACCGAAGCG

ATTAACCAGATTCTACATCATTACAGAGGGTACATAACGAAGCGTTCCCTACGACTTATG

AAAGATGAATATGGCAATCAAAGTATGGTCGTTGATGAAGTCTTACGTGGAAGAATGGAA

ACCAGACTGATTACAAAGATTTTGTCATTTGAAATTAAGTAATATCCTCTCTCCTTTCGT

GGAAGCGTGCTAAACCATTCCACGCTTCCCGAACAGGGAGGTTTGTTATTCCACCAAAGC

ATATTGAGCTTTCAATGTGTTTTGATAGGCTAACGAGCCATTGTTCTTTGAAAACTGAAT

AAAAGTAATCGAATACGTTTCGATAAGAAAAGAGCCAACGGAACTAACCGCCATGACCTA

TCTTATAAAGATAGCGAGCGATTCATGTTAGTGATCCGAGAAGCAATCTTTAGCAGGATT

GCCTGCAACGACATTCTTATCGTGATAATGATACTCCCATACAGTCAATAGTCCGAGCGT

GATAAAACCGTCGCAGGCAATGAGTATGGCTACATGAGAACCATGCAGGGGTGGAACTCC

CGTGAGCTTTGCTAAAGCTGTTCGATTGCTGGTAAAACAACTTTTATGAAATCCAAATAA

GTGATTTGGAAAGGAGGATTTTATGAAGCAGACTGACATTCCTATTTGGGAACGTTATAC

CCTAACCATTGAAGAAGCGTCAAAATATTTTCGTATTGGCGAAAACAAGCTACGACGCTT

GGCAGAGGAAAATAAAAATGCAAATTGGCTGATTATGAATGGCAATCGTATTCAGATTAA

ACGAAAACAATTTGAAAAAATTATAGATACATTGGACGCAATCTAGCGTCGCCAAAGGGT

CTTGTATATGATAAAATAGTATTAAGTCGTATCAAGGCTCTTTCCATAAAGGAAAGGAGC

AAATGCCATGTCAGAAAAAAGACGTGACAATAAAGGTCGAATCTTAAAGACTGGAGAGAG

CCAACGAAAAGACGGAAGATACTTATACAAATATATAGATTCATTTGGAGAACCGCAATT

TGTTTACTCGTGGAAACTTGTGGCTACAGACCGAGTACCAGCAGGAAAGCGTGATTGTAT

CTCACTTAGAGAGAAAATCGCAGAGTTACAGAAAGACATTCATGATGGTATTGATGTTGT

AGGAAAGAAAATGACACTCTGCCAGCTTTACGCAAAACAGAACGCTCAAAGACCAAAGGT

TAGAAAAAACACTGAAACTGGACGCAAATATCTTATGGATATTTTGAAGAAAGACAAGTT

AGGTGTAAGAAGTATTGACAGTATTAAGCCATCAGACGCTAAAGAATGGGCTATTAGAAT

GAGTGAAAATGGTTATGCTTATCAAACCATCAATAACTACAAACGTTCTTTAAAGGCTTC

ATTCTATATTGCTATACAAGATGATTGTGTTCGGAAGAATCCATTTGACTTTCAACTGAA

AGCAGTTCTTGATGATGATACTGTCCCTAAGACCGTACTAACAGAAGAACAGGAAGAAAA

ACTGTTAGCCTTTGCAAAAGCTGATAAAACCTACAGCAAAAATTATGATGAAATTCTGAT

ACTCTTAAAAACAGGTCTTCGTATTTCAGAGTTTGGTGGTTTGACACTTCCAGATTTAGA

TTTTGAGAATCGTCTTGTCAATATAGACCATCAGCTATTGAGAGATACTGAAATTGGGTA

CTACATTGAAACACCAAAGACCAAAAGTGGCGAACGTCAAGTTCCTATGGTTGAAGAAGC

CTATCAAGCATTTAAGCGAGTGTTAGCGAATCGAAAGAATGATAAGCGTGTTGAGATTGA

TGGATATAGTGATTTCCTCTTTCTTAATAGAAAGAACTATCCAAAAGTGGCAAGTGATTA

CAACGGCATGATGAAAGGTCTTGTTAAGAAATACAATAAGTATAACGAGGATAAATTGCC

ACACATCACTCCACATAGTTTGCGACATACATTCTGTACCAACTATGCAAATGCAGGAAT

GAATCCAAAGGCATTACAGTACATTATGGGACATGCTAATATAGCCATGACGCTGAACTA

TTACGCACATGCAACATTCGATTCTGCAATGGCAGAAATGAAACGCTTGAATAAAGAGAA

GCAACAGGAGCGTCTTGTTGCTTAGTAGTACAAATGAATTTACTACTTATTTACCACTTC

TGACAGCTAAGACATGAGGAAATATGCAAAGAAACGTGAAGTATCTTCCTACAGTAAAAA

TACTCGAAAGCACATAGAATAAGGCTTTACGAGCATTTAAGAAAATATAAAGGCTCTATA

ATATTTGTAGTGGGTAAATCCACTATAGATATTATGGAGCCTATTTTATTGTAGAAAAAA

AGTCCCATAAGATCTATAATGAAAAGCGACCAAACAACTCATTAGAAAGATTCATATGGA

ACAATTACATTTTATCACAAAACTACTCGATATCAAAGACACAAATATCCAAATTATAGA

TGTCGTCAATAGGGATTCACACAAGGAAATCATCGCCAAACTGGACTACGACGCCCCATC

TTGCCCTGAGTGCGGAAACCAATTGAAGAAATATGACTTTCAAAAACCTTCTAAAATTCC

TTATCTTGAAACGACTGGTATGCCTACTAGAATTCTCCTTAGAAAGCGTCGATTCAAGTG

CTATCACTGTTCAAAAATGATGGTTGCTGAAACTTCTATCGTCAAGAAGAATCACCAAAT

CCCTCGTATCATCAACCAAAAGATTGCTCAAAAGTTAATTGAAAAGATTTCTATGACTGA

TATTGCCCATCAGCTTTCCATCTCAACTTCAACTGTTATTCGTAAGCTCAATGATTTTCA

CTTTAAACATGATTTTTCTTGTCTTCCTGAGATTATGTCTTGGGATGAGTATGCTTTTAC

AAAGGGAAAGATGAGCTTCATTGCGCAAGATTTTAACAATCTCAATATCATCACTGTTCT

TGAGGGAAGAACACAAGCTATCATTCGAAATCACTTTCTTAAATATGATAGAGCCGTCCG

ATGTCGCGTCAAAATTATTACTATGGATATGTTTAGTCCTTACTATGACTTAGCTAGACA

ACTTTTCCCATGTGCTAAAATCGTGTTGGATCGCTTTCACATTGTACAACATCTTAGCCG

TGCTATGAGTCGTGTGCGTGTTCAAATCATGAATCAATTGGATCGAAAGTCTCATGAATA

CAAGGCTATCAAGCGCTACTGGAAGCTCATACAACAGGATAGTCGTAAACTCAGCGATAA

ACATTTTTATCGCCCTACTTTTCGTATGCATTTAACCAATAAAGAGATTTTAAACAAGCT

TTTGAGCTATTCACAAGACTTGAAACATCACTATCAGCTCTATCAACTCTTGCTGTTTCA

CTTTCAGAATAAGGAACCGGAGAAATTTTTCGGACTCATTGAGGACAATCTAAAGCAGGT

TCATCCTCTTTTTCAGACTGTCTTTAAAACCTTTCTAAAGGACAAAGAGAAAATCGTCAA

CGCCCTTCAACTACACTATTCTAACGCCAAATTGGAAGCGACCAATAATCTCATCAAACT

TATCAAGCGCAATGCCTTTGGTTTTCGGAACTTTGAAAACTTCAAAAAACGGATTTTTAT

CGCCCTGAATATCAAAAAAGAAAGGACGAAATTTGTCCTTTCTCGAGCTTAGCCGACTTC

AACCCACTGCAGTTGACAAAGAGCCCTTTTTTTCAACCCACTACAGTTGACAAAGAGTCA

AAAAAGGGCTCTATAATATTTGTAGTGGGTAAATCCACTATAGATATTATGGAGCCTTTT

CGGCTCTATAATATTTGTAGTGGGTAACCCCCCTATGGATATTATGGAGCCTATTTTGTT

GTAGAAAAAAAGTCCCATATGACCTATAATGAAAAGCGACAAAACCATCATTAGAAAGAT

TCATATGGAACAATTACATTTTATCACAAAATTACTAGACATTAAAGACCCTAATATCCA

AATTATAGATGTCGTCAATAGGGATTCACACAAGGAAATCATCGCCAAACTGGACTACGA

CGCCCCATCTTGCCCTGAGTGCGGAAACCAATTGAAGAAATATGACTTTCAAAAACCTTC

TAAAATTCCTTATCTTGAAACGACTGGTATGCCTACTAGAATTCTCCTTAGAAAGCGTCG

ATTCAAGTGCTATCACTGTTCAAAAATGATGGTCGCTGAAACTTCTATCGTCAAGAAGAA

TCACCAAATCCCTCGTATCATCAACCAAAAGATTGCTCAAAAGTTAATTGAAAAGATTTC

TATGACTGAGATTGCCCATCAGCTTTCCATCTCAACTTCAACTGTCATTCGAAAGCTCAA

TGACTTTCACTTTGAGCATGATTTTTCTCGGCTTCCAAAGATTATGTCTTGGGATGAGTA

TGCCTTCACTAAGGGAAAGATGAGTTTCATTGCACAAGATTTTGATAATCTTAATATTAT

CACTGTTCTTGAAGGCAGAACACAAGCTGTCATCCGAAATCACTTTCTTCGCTACGATAG

AGCCGTTCGTTGTCAAGTGAAAATCATTACGATGGATATGTTTAGTCCTTACTATGACTT

GGCTAAACAGCTTCGCTTTCAAATTTCTAGGCTCAGGCTGAAACAGTCTCCCAGGCTGTT

TCACTCCCGAATGCTAAAATCGTTCTTGATTGCTTTCACATTGTACAACATCTTAGCCGT

GCCATGAGGCGTTTTCGTGTTCAAATTATGAATCAGTTTGAACGAAAATCTCATGAATAC

AAGGCTATCAAGCGTTACTGGAAACTCATCCAACAGGATAGTCGTAAATTGAGCGATAAA

CGTTTTTATCGCCCTACTTTTCGCATGCACTTAACAAATAAAGAAATTCTTGACAAGATT

TTAAGCTATTCAGAAGACTTGAAACACCACTATCAGATCTATCAACTCTTACTTTTTCAC

TTTCAGAATAAGGAACCGGAGAAATTTTTCGGACTCATTGAGGACAATCTGAAGCAGGTT

CATCCTCTTTTTCAGACTGTCTTTAAAACCTTTCTAAAGAACAAAGAGAAAATCGTCAAC

GCCCTTCAACTACACTATTCTAATGCCAAACTGGAAGCGACCAATAATCTCATCAAACTT

ATCAAGCGCAATGCCTTTGGTTTTCGGAACTTTGAAAACTTCAAAAAACGGATTTTTATC

GCCCTGAATATCAAAAAAGAAAGGACAAAATTTGTCCTTTCTCAAGCTTAGCTTTTTTTC

AACCCACTACAGTTGACAAAGAGCCAATATAAAAAGATAATTAGAAATTTATACTTTGTT

TACTGTTAAAATTTCTATTTCCTCTGAATCTCGAATAAAGGTCTTCAGCGAACTAAGAAT

TTTTTTCTATTACCTTAATAGTATCATAAATGAGGCTTGATTGAAAGCGTAAACATAGCT

TTGATAAGTGATACAACTATTCTATACAAAATTTAAACATAAAACTTAGGTATACTTAAT

AAATGAGAATTTAAGTTAGCTATAACGAAGATAGTAGCATATGTAAAACTCTAAGCAGGC

AGAGCCTGCTAACTCATGACAGAAAAAGATGTTTAGCCTATAGTGCTGTCATTAAAAAAT

CCTCACACATCACGCAAGGAAATCGCTAAATAAAACAGGCTTTGACCATGTCGGGATTAA

TTGCAGCACATTCCAAGGATCCAGCTTTTTCTTTTTTTTATAATCGAATTTCCCAAAGAG

TAAGCAAGATGAAGGCCGTCATTGCTTATGCTCATAAGATTCTCAGAATCATTTACAAAC

TACTTTCCGCCAAGCATACTTATCAAAAAGAGAAGGGGCTAGGACTGAGGAAACAGTTCT

AACGCCAAAACTAAAAAATTTCAATTACACGCTAGTACAGGAATTGATTTTTTACGCTTT

CTTACTGTTTTTCCAAATAAAAAAGATGACTTACTGAACCTGTAAGCCACCTAATCAGTC

GGTTTATTCTGGTGTCTGCCACCGCTTGGCCCGTACGTCCAAGATTGCTATCGGATTTTG

TTCTGGTGTCCGCCACCGCTTGGCCCTTATGTCCAATATTACTATCAGATTGACCATGAG

CCGACCACTCATTTATAGACATATTGTATCTTTAAATAGCTACAATTTCAAGTGTAAACT

CTTCTATTTGGTCGTATAAGCAAAACTGATGGCACTATCTGCTTGAGAGATTTTTCTAAA

GGTATAGTTAGGGTTGCCCCCATAGTTGGTTTCAGACACAAGGAAAGAACCATCATCATA

GACCTTCTCTACAAAAGCCACATGACCATAGCTGGCTGGTGTACCATGTGTACCTCCTAC

AAAAGAAACAATAGCACCTGCTCTTGGTGTAGAGCCCGTTTCCCCACCAAGACTTGAAGC

TGTCGCAACCCAGTCTTGACCATTTCCCATGGTATTAATGATTGAAATCTTTTCTCCATT

TCTACCTTTTAATTTTAAGCCTAACTGGTTCATACGAGCCGCAACACCCCATGTACATTG

TCCATAAGCATAGGCCATACCATCTCCACCACCAGGAACAGAATGATCATACAAGTCCCC

ACGAACCCCTTCAAGGGATTGTGGATCACTTTTTGCCTGTCCTCCATTTGTTTGACTAAA

GCCTTTTTCAATTTGGTAATACCATTCCGTTGCTCTGGTTTGTCTTTCCAGTAGTTTGTC

ACCAGAATTTCCCTCCCAATAGGTCAGAAAGAGTTGGGCCAGATTGGCTGCACTGCCTGT

ATTTTTAAAGAAATCCTTTAACCAACTTTGATAGTAAGGACTATCCCCATGAAGCATAAA

ATCAAGTTGTAGGTCTAAATCATACCATTTCTTATTTTGGGTGCGTGCATAATTTAACAA

GGCTGTATGCCGTGTTGACCCATCTGCAGTATCCGTCCATTGCCCTAAACCCAGACCTCT

ATGAAGAATATTAGGATAAGCACCACTATAAATGGCTGGACCTCCAATCGCTAACCAGCT

TTCATCATCCCATGAGGAATCGGTAGCGCCAACAGGAGGAGATAAATAATCTCCTTCAGC

TCGTTTAGGATTAATAGAAGACTCTACCGACCAATTTCCTAAAATTGCCGCAATGGCTTG

GGGACTTGCCCCTTGAGATTTCAAAAACTCATAAATATGTTTTGCTCGTTCAAACTCATC

CCCACCAAACTGACCAATGGCAGGTAAGATAGTTGTCTGAAGTTGAATGACTTTTGGAAA

ATAAAATTGCGGATTGACATACACCAATTTTTCTTTCTTGTTCTTATACTTTTGATAGGA

AACTTTCAAACCTGTATCGTCTGGTGTTTCACCAATAATATCACCCGTTAGGACTCTTGT

CCCCTCAATCGCACGGCCATTATGAATAGAATACAAGGTCAATCGACTCTCATTCTCTCC

TTTTCCGTTAGTGAGAATAACATCATCTCCGTCTAGAGATACAACACCATCCATTGGTGC

GACAATTGTTTGGTGAGCCTTCGCTTCTAGTAGAATATACTCCTGAAGAGTAGGTTTTCC

GTCTAAATCATAGTATCCATAACGATAAGTCATGGTTAGACTATCTTCGTTGCTTTTCCC

CTCAAATGGATTGTCCAATTCCTGCATGGAAGCATAGACACCCTCTTCTTTTAGTTCCTT

TATTTCCTCTTGATCGTCTCTCGATAGTTTATACTTAGGAGTTTCATAGAGGTCTTGCAT

GGATTTCAAATCATCCCCATCGTTTAAATCATGCCACAAAGTAGACAGATAATCCTTGTA

AGATTCTGAACTAAATAAGTGAACTGGTTTGTGTAACTCATAGTCATGGAATTTAAAGTT

CATATACCCCATCACATCATCAACTTTTGTGTAATAAGTAATTCCTTTGTCATTTGTGCG

AGTATGTTCTGCATCTTCCCAAGTTAGGTGGGTATAAGCTTTTGTTAATTCAAATTCATC

TTGTTGAATCAAACTAGCAGATGAAAATCCTAAAAAGAAGCTCATCATAAGTAAAAGAAG

AAAGACTATTCCTCCAACTATCCAGGTTACAGGATTTCCAGCCACAAATGTAAAGAAGGA

AAAGGCTGCTTTTAATTTTTGATAGATATTTCGGACACTTAAAAGACCTTGTTTCTTTAA

TTTTCGATACCGATTTTTAAAGGAACTTGGATTATCTTTAGTTAGTTTCCATCCTTTTCC

ATCCTTAAAATGATGGTATCGCTCTTTTGTGTTGGTCAGTCTTTTCTTGGTAAAACGACC

TGTTGCTTGTCCTGTTTTGACACTAGCTTTTCCAAGATTATAAGAAAGCCGACTGTAGCG

TTTCCCTTTTCTAATGGTCTCATGAAGTGTGCGATAACTTTCTAAATCTTCATTTTCTGA

AGCTAACTCTCCACCTTCACGTCCAAGGACATAAAGAAAGGTTTTGGCTTTCCTACTGAT

TTTTTTGAACTTATAGGCTTGCTTAGTAGATTTTAGATTCTCTTTTGCGGCTTTGACTTC

TTTCTTAGCTTTTAATTCTTCTAGATTCTTCCCTTGAAGGAAAAAATTGGATCTTACTTT

CGTTTCCTGACCGTAGAAAAATTTTTGATTGGTTTTTCTTTCTTTACGACTCTCTTTTCT

TTCTTCTTTTGCTTCAACCTTAGCTTCCTTAAATTGTTTCTTGGCTATTTTCAATCGTTT

CCTAGCATGAGGCAATCGTCTATCTCTTAATTCTTTCCGATTCAAAAGTGATGGAGGACT

ATTTTGAAGAATATGATTGTAGTCTTCATTTGCTTGTTTTACTCTAGCCTTTGAAGCTTC

TCTCATATTCTCCAGCTTTTGCTTTATCTCATTTTTCCATGCTTTTTCATCCAGTACAGC

GGAATCTTTTTTCTGTTTTCTCACCTCCTTCTTTCCTTGTTTTAAGAATTTCTTCTCATC

TTTTAGACTTCTTCTAAATGCCTTTCGGGCACGTATGATTTCTCTCTTATCCTTCATTTA

CCTTCCCCTTAATTAGAAGCCATTTTATCAGGATCTGTACTCATGATATCAAACAATTGT

GTACCTTGGGGAATCTTATTTTTAAAGGGAACGACAACTGAACCAGCTTTTATTAGTCCT

GCCCCTTTTTCTGGATTGACTAGGTATTTTTCGAGTTCTTTTGACAAGCCTAAGAGTTGA

ACCAGTTCTTCCCGGTCATTTTTTGCTTGCTTGAGGAGAATCATAAATTCACTATTGGCA

ATAATCCGTCTACCATTTGGATCTAACAATAAGGTTTCGACATTTTGAGTTATTCCAGTC

GGACTGGCTCCATATTTTCTGACACGACTCCACAATTTAAAGAAGAAATCACTGGCATAT

TTATCTAATAAGAGAAGCTGCATTTCATCAAAATAAATCCAGGTCTTCTTCCCTAATTTT

TGATTCCGAACGACACGATTCCATATCTGATCAAAAACAACCATAAGAGCAATTTGTTTC

AACTCATCTCCTAACTTTTTAACATTATAGATTAAGAAATTAGATCCTGTCTGAATATTG

GTCTTATGAGAAAAAATATCAAGAGAACCTTCGACATACAGTTCCATATCAAGTGCCAAA

TTCTGCGCTTCTTCTTCTGGTTGTTGGCTCAAGACAAAGACCCATTCTTCCAAAGAAGGC

TCTTTAAATGACTGATAGGTGAGTCTGGTAACTCGGTCAATAATCGATTTTTCTCTTCCA

TCCATTTTTCTATCCAATAACTTGCCGATAAAGGATAAAAGAAATTCTGATTTTACTTTT

ACAGGATCTTCATCCATATTTTCTTCAGACAAGTCAAGGACATTGAGATATGTTTGGGAA

TCTGGCGCAATATCAATCATTTCTCCCCCAAAAGCTCGTCCAATGACACTGTATTCTGCT

TCTGGATCCACGATGATAATTTCTGTATTTTCACCAGATTCCTTGATTTTTGTCGTGATA

ATTTCATGCTTGGTTGCCATCCCTTTACCGGCTCCAGATGTCCCTAAAATTAGACCAGAT

GGTGTATTTAATAGGCTGCGATCAATGGTAATAATATTACTTGATATTTGATTGATACCG

TAATATTTCCCACTACGGTCTTGTAAATCTACTGAAGTCCATGGAGAGTTCACTGCTACA

TTAGATGTTAATAAACTCCGTGATACTCCCTCTAAAAAATCACAACCAAATGGCAGCAAA

CTATTAAAGGCTGCTTCTTGCATATATGGAAGTTTATCAATCATTAGGTCATTTGAGCCG

GCCACTTGTTGAATCGTATCTAGAGCTTGTTTGAGTTCTTCTTCATCCTGACCAAAAACA

CCAATCAAAAAGACTGTTTGAAACAGTTTATCTCCTGTCTCTGTCATGGTTTTTAAGAGT

TCCTCAGCTTCATCGATATTGCTTTCTAAAACATGGCCTACTTTTTCCAAATAGATACCT

GTACGAGCTAGTTTTTGTTGTTCCCCAATCTTTTGGGATTCCATCAAGGTCTTCTTTGTT

CGTAGTTTCTTCATGGCATCTGCTTTGGTCGAACTTTGAGCATGGAGGCTCACAATCAAT

TCCAAATCTCCTTGCATGAGGTCTCGAATAAACTGATCCCCTAATTCCATGCCGTAGTCT

CTCACATAGACAATCTGCAATAAGCGGTCATTGATTTGTAGGTAATTCTTGTTTTTGAAA

TCTAAGAGATTAGGCGCTATAAAGTGACGAGTTGTCTGACCCGATCTCGTTAAATCACGG

TAAGAAAACGGAAGATGGTGTTCTCTTCTAAGCATATCTGCCAACAAGTTCACACGGTTT

TCTCCAGCCAAGGATTCAAAGCGAGCATCAATTTCTGAGAAACCACTCTTGAAATATTCT

CCTATTTGGGACAAGGAACGATAGGCTTGTTTGGGATTAGAATCCTTTCTACCAAAGGTA

ATCAGTTTCACAGCTGAAAAGTTATTTTCACCACTGTCTAAATTCTGATTCATCATCCGA

TTCAATTCTTTACGATAGCTATCATACCCATCTTCTTTTTCCTCATACAAAACACTTTGT

CTAAACTTTTCTAAATTCAATCTTTTATTAAAGATGGTCAATTGGAAGTTGGTTTGGTCA

TCTAGGGAGTTAATCAAATCAGAATACTTCTCAATGATTGCGCCCTTATCTTCTAAACCA

ACAGTTTGGTAATTGACATCACCAAGTAAGTAGCTTTGTGAGAAATAATCTTCTTTTACC

TGCATTAGACCATTTTGATACAGGGCTTGATAATAAAGAGTATTCGCCGTAGAGGGTAAC

ACTTCCTCTTTTTTCCCTTTAACTACTTCTTTTTTATTAGTCATTGAAGTTTTTTGTTTC

TTTAATGTATTTGATTTTCTTTTCATGTTCAGGTCCTTTCTTTCCTGTAATTGTGCGTAG

GGGAACCGTTAATTCAAAATGAAGACGGTATTTCAAATAATGTTCAAAATATAAATCATT

GGGTTTATAGACTCCAAAAAGCATGAGGGGGATGGTAAAGGCAAACACAAAACCGTAAAC

AAACCAATCTCCAAATTGCCAGAAAAAGAGATTCAAGCCCAAAACAATAATTGTGACAAT

AAAGGCTGGTAAAACAAAGATAATTTGTCTTGTGGTGAAGCCTAACCAAGCCCTGTGTTG

GTATTTTGAGATGTCTTTAAAGACACGTGTATTCATGACTTTCCTTTCTAAAAAGGCTAA

GAAGCATTCACTTCCTAGCCTTTATCTAATTACATACCTAAAATTGAGCGAGCCGTACGT

TGAGAACCAACGAGAGCGATAATCAGCAAGATAGCTTGTACCAAACTACCAAACATAATC

GCAAGTGATTGCAAGACTCCTGCACCATTTGAAACAGCTATTTTCCCAGCAGATTCAAAC

AAAGGAACAAGAGAAACAATCAGAAAAATAAGAACCCCTTGTACCGCATAGACCATAATA

TTTTTTAAATAGCCAATACCAATAGACTTCCATTCATCACTTAAAAATGTTGGAATCGTA

AGAGGGGCAAATGGAATCATAAGGTAGAGTTGAATAAATCGAATAGATACCAAAAGATTG

ACCATGGCTGCACTTACTATCCGAACTAGCCAAATGAGGAGGGCGAAAAAGCCCACAATC

ATCCGGCCAATAAATCCTGAACCTTTTAATCCAGAGATTGTATCATACTTTGCCCCACCG

TGAGCCACAATCGAGGCCACTTGTTCAATGGCGTGACTCGCAATACCGATGATAGCTTCT

ACAATGACGGTAGTGTTGGTAATTACAACTGCGACCATAATATAACTAATCAACATCGGC

GCTAATGCTTCAAAGGTCATCGCTCCTCCTGAGTTAGCAATCTTCTTTGCCATCTTCGAA

AATTCTAGGATGAGAACAACTGATAAAATCGCAACTCCAAGAGGCTGCATGACACTTTTA

GTAATACTAGACATATAAGTCCAAACTGTTGGATTGTAGCTAGATAGAGATTTAATCAGA

TCTACCGTAGATTGTAAATCTACATTAAATCCTTCAAATAAATTTTCAGCTGATATTTTT

TCAGATGCAAGGTAAACAAAGGGTGAGACTAAACTAAGATTCATGTCATTGTTTATCCTC

CTAAATTGAAATTTGAGTTACAAAGGCTCCAGCAGCCCCTACCATAACACCACCGACAAT

TTCCAGAATGGCATTCCGAACACCTGGTCCACCATCTTTAATGTTGGTTGCAAGATTGAC

AATTCCTACAACAACGAGAAAGGCACCAACCGCAATCAATCCCTTCTGTAACAAAGACAT

AGCTTGTGCAAACATGGCACTTGCGTCTACTCCATAAACAAAACCTTTAAAATGCGTAAT

CATCTATTTCCTCTTTTCTATTTTTATTTTAAACTAGATTCAAAAGTTAAATCACGAATT

CTAAGGCCTTCAAGATGATTTTCTTGTCTTTGATTCAAAGGATTGATTTGATAGTTCCAC

CACCGTTCATCGGTTTCTTGGTTGGCTAGGTACTTCCAGTTTGGATGCTTAGTTGAATTG

TATTTTTTGCTTTTAAAGACTGGCATATTGGCAATTCGAACCAGGCATTCATGCCGTTTC

ATATTTCCGACTTCATCAGGTGTCATTAACCTGTCTGGCCAAAGGAACGACTAGTATTTC

GAACATCAATGGTTTGTTTACCGAGTAAGCCACTCATAAATTTAAAGGTATCTTCATCAT

TCCCACCTAAGTAGACTAAGCTATCACAGTTCCCCAAAATGGTTTTCCAAGCTTCTTTTT

CTTTATAGAGCCCTTGAAGTTGGGCAATATTTTGTAGAATAGGAACGAGACTCATGTTCC

GAGAGCGGACTGTTGAGGTTTGTTCAGCAAAATCTGGGATTTCTCCGATATTTGCGAATT

CATCTAAGTAGACTCTTACATGAAGAGGTAATTGACCCTTAAAATCAATATCTGCTTGTC

TTGTTAGGGTTTGAAATACGGTTGAAAAAAAGAGGGCTGAAAGAAAGCGAAAGGTACTAT

CGTTATCTGGGATAACTAAGTAAACCATTGATTTTTCCTTGCTCCATGTCTTCATATCAA

GGGTATCTCTTTTGGTCAAATCCATGACACTTTGAATATTGAAGAGGGCAAATTTAGCAG

TGGTTACAGCTATAACAGAATCCAGAGTCTTATCCTTATAATTTTGGAAATCTGCCCAAT

TTCGCATGGTGAAATTTTCAGTCCCATACTTTTTAGCATAATTTTCAAATAGAATTTCTA

AGACACTTTTTTCTTGGTTTTCACCCTTGGATAAGTGTTTAATGAGTTTTGAGATTTCAG

CAAAACTTGGATAACACCCTCGTTTTTTTCGCTCTTCCACTTCTTTTTTTTGACGTTTCA

ACAAGTTTTGGTATTCTGTTTGACTCAAACGACTTTCTTCTATGAGCTGTTCTCTTGTTT

TGGGTGGGTTATAGAAATCGACCAAGTAGGAGGCTAAAGCTCGTACCAAAGTCATAGAAG

CTTCATCCCAAAATGGATCACTACGGGAGCCAGAGCCTTTGGTGTTATTAAAATAAACCG

TCAGCATGCGATTCAAATCATTTTCTGTCTCTATATAGCGAAAAGGATTGAAGCCATCTG

AGTTCTTCATATTGACTAAATCTAGCACCTTTACTTGGTAGCCATGTTCTAAAAAGAGTT

TGCCTGTTTTTTCGGCCAAGTGATCTTTAGAATCCACTACAATATTAGAACTATTCATCT

GAATAAGATTAGGTTTCACAAAGCGAAATGTCTTCCCACTTCCTGAACCTCCAATCACCG

CAATATTCTTATTTCTGTCATATTGGGGTGATTTTTTATCTAATAATGTCAAACGAACAT

CTTGTGCTAAGATCGTATCATGAGAAAATTCTTTACCGTAAAAGAGCTTCTTTTCTTTTA

GAGTTCCAAAACGGGCACTCCCGTATTCTACTCCTTCTCGGTATTGTTTCTTACCGGTTT

CTAGATAGAGATAAACCAGTAACATCATCACAAAACCTAGTAGAAAAAAAGCACTTGATT

TTCCAGTAAAAGAAATATTCCATGGCGACTGAAGAACTTCATCTTGACCTTCCATCAGAA

GATGAGTCCATTTATCTAGTGTATTTCCAGTATAGGAATCATACAAAAGCGTCAAACGAT

GAAAAAGATAGCCTAGTAAGATACCTAACAGTGAGAATAGTAGGAATTTCTTTCCACTGT

ACATCATCTCACCATCTCTTTCTGTTTGACGGCTCCTTCTTGTCTAAAGGTAATTTGGGA

TTTAGCCTCATCAATTGCATCGTCTAATGACTTATCCATTGTAAAATCAGCTAATTTCTC

CGGATCATTAACCATTTTTTCTAACAGATGGTCTAAATGATTGTCTAGAATCGAACGGTC

TTTCGTGTAGAAATGCAGAGAATCCCCTTGCCAAGCGATGGCTAAAGGAATCTCTTCTTT

TTCTAAAAAATCTTTAAATTTCTCTATATCAATTGGTTTGTCTAAAAAATCTTTTTTCAG

ATTAATCGTATCAATCGAATAAGGAGATTGTAGCAATTCTTCTAATTTCTGCACCCCTAC

CTTATAAGCAGAATCTTGTGCTAAAGCCTGACGTCTAGACCATTCTAGAATCTTTAAAAG

ACTTTTTACAGTAAATAAAAGACTACGCTCAGCATATTGAACTGCCATTCGTTCCTGTTG

TTCAGAGGACATCTGATGCCTCCTTCTTCACAAATAGCAGTTTTCCTTCTTTATAACGAT

AAGCTATCAATTTCTGACGTTGCTTAATCGACTTGACAACCTGCAGTAGATCTCTCGAAT

AAGGTTCTCGAAGAGTGACTTCTACTGCTTTCCCATCAAAGATACCAACACGTTTAAACT

CAATGTAGTCTTTTTTGAGATGTCCTAAGCCCATACCAAAAGGAAAGTGATGAATCAATT

GGATGGTACAACCACCTTTATTTCCCATTTGTTTCAAATCATACAAGTTTACTACCTTCA

TGATTAACTCCTTTATCTAGTTTGTCGCATCGTTTAAGTCTGGTAACGATAAACTCCGTG

TCTGTTAGAAAATTCTCACACACGTCTTGTGCCAGTTGCCCTTCACAGGGAAATACTCTC

AGTCCCTACTTACACAGGCACGCTAATCAAGACGGAGTGGATTCAATTTTCAAAGAACAG

GTAGCTTTATTATAGATAAGAGTAGTTGAAATTTTTATCACATTTTTTATTTTTCTGTCG

AGCTAAATTCATATTAAACAAAGATTTTGAAATAAAAGACTAACATTGTTACATAACGTT

AGCATTTCATTTCCCTAAAAAAACTAATTTGGTCTAAAAAAGCAGCAAACTATAAACTAG

TAGGTTCCACACCAAATGTAGCCCCATACTGCCCCATAAGTCCAATTTGTAGCGTACAAG

CCCTAAAAACATCCCCAGCGATACATACAAACACCAAGCTATAATGGTTCCTGGATGATG

TGCTAAGGCAAATAAAACACTTGTCAAAGCAACTCGAATATCTAATTTTCTAACCAAGTT

CCATAAAATTTCTCTATACAGAAATTCTTCAACCATACTCGCATTGATTAAAAACAATAA

AAATGAAAACCAAGGAACTTGATGTTGAAGGCCAATTAAGTTTGCTTGATTCGTGCTTTC

TTGCGCATGGATTAGACTAAAACATAGACTTATAATCAGTAGGCTAACGAATCCAATACC

AAGCCATTTCATCCTAGATTTCATATTTACCTTACGAGCTTGTTTGCGTTGGCCATACAT

CCATAAAAAAGAAATGAGTGACGAACCATAGAGAATCTGTAGTATAGTTAACTCACCGAT

ACAAAGAAATTTCAATAAGTATAGAGATACCAATAGAACATTTACTTGTTGGAATATATA

AACTGGAATTATTCTTTTCATAGTTACCTCCGAAATAAATCTTCATAATCTAAATCTAAT

ACCTGCACAATCCTTTCTACCCATGGACTTTGAGGCATTCGTTGTTCCATCTTCTAGTGG

CGAATCTTTTGATACAAACGATTCAATTCACTTGGATAGTGAAACTCTCCCGCAAACATT

TTTCTGGTTAACTCAATCCAGCTGATATTTCTTTCAGCCAAAATAATGGACAAGTTCTCC

CAAAATCGTTCAGCCATATTGCTTCTCCTTTAGTTAGATAAATAATGTGTTTGCGCCATG

TAAATCAATTGTTTCGTATCTCTTGGCAATAGAGCTCTAGCCTCTTCCAGATTCAGATTT

GGATAAACTCGCTTATTTGAAACCGCAAGAGGAAGTCTGATGGTTAGTTCAGGATTTTTT

AATATCATTTCGATGAAATCTGTTAATTTTAGGTTATCTCGATTCTTAAAACGTAATAAA

TTTGGAGATAAAAACTCAAAACAATCTGAAGAATAGCTCATCATCTCAATTAATTTGTCC

TTTGTCATTTCAGAAACTGAATGACAAGATACCTCGATGCCATAGTTTTGGAAGAAGTCT

AAAAGAAGTTGATTTCTTTGGCTATTTTTACTTAGATAGAGATCAATCATAGGAGACCTC

CCAAAGATTCGGTTCCATTTGATATTCTGAGACGATTAAGGAATCTAATAAATTTGCGAA

GTTAATCGGTTTCTTGTCTTCATCATAAGCTTTTACAGTTACTTGGGGTGTAAGTATCCC

CTCTTTTCCCTCGGCTCGATAGCCTTGTCCATATAAAACAAAAACGAGATTTTGATGATC

ATCTACAGAGGCATCAACCCCATTCTTTATGTCTTGACTTTCAAGGAATTCCATAACGTT

TTGAAGATAGGATTCGTAAAATAGTGGGTAGTTATGTTTTTTATGGTAATCATCTAAAAA

TGTCACTTCAAACTCACATGGATAATTGAGCATCAAAAATATTTGTTCATCTAGCTGTTT

GATCTCTGCATCATGTAATTGTGTTTCTAATTCATCACAATCTAGTATTGACTCTTTATT

TAAAGCTTTCATCTTTTTCCTCTATTTCTTTTAATTTCTTTGCGATTGCAGCAATCACAG

GAACGGTTACACTATTACCAGCTTGTTTATAGAGCTGACTATTACTAGAGACTTTTCTAG

CAGCTTCAAAAGCCCAATCAGGAAAGCCCTGTAGGCGAAAACACTCTTTAGGAGTGATTC

GTCGTATTCTCAAACGGTAAAATTGTCCATCTATTAAGACACCGGCTACATGATAAACTT

GTTTATCTTCTCCTTCATAGCTAGCAACTACTACTCCCATTTGTCCACTAGTTGTTAACG

TATTCGCTATACCTTTTCCAACTCTACCTCGACGATACTGAGAACTTGGTCTTTCCAAAT

TGATGGAATCTCCTATTTCTGCTTGAGCATATCCTTTTTTCGTTGCTTCCCGTACTTTTA

GAAATTGGATTGGTTCTGGAATTAGTATTTTGGGGATTTTATCTCCTCCTTGCATTGTAG

TCAGTGTTGGAGATAATCCCTCACTTCCATAAACTCGACCAGTTTCCTTAAAGCTAGTTG

GTAAATCTCCAACAACGACAATACCATGACGATCCTGAGTATTTAAAGTAAACATCGGCT

CTTGGTTGTTCTTAAAGCGTCTATCATTTTGTCTCTTGTCTAATCTATCTGGTGTCATAC

AAGGAATCGCAATCTTAAATCCTTCTCCTTTCCCACGAACTAAGGTTGGCGCAAGACCTT

CTGAATAATAAACTTTACCGCTCATTCCACTTTTTGATGGATTCAAATTTCCTAATGTTT

TTAAAGTCTCAGAGTTAGTTGCTTGACCTTCTCGTCTGAAAGGAAATAAGAGTCTGGTAC

CTTTCTTTCTAGAATGTCCGATAATAAACACCCTCTCTCTGTTTTGGGGAACGCCAAAAT

CCTTACTGTTAAGCACCTGCCACTCAACATCAAACCCCAACTCATCAAGTGTGGTAAGAA

TTGTGGTGAACGTCCGTCCCTTATCGTGATTGAGTAGGCCTTTAACATTTTCAAGAAAAA

GAAAACGTGGTTGGATTTGTTTGGCCGCCCGAGCAATTTCAAAGAACAAAGTTCCTCTAG

TATCTTCAAATCCCAATCGTCTTCCTGCGATTGAAAATGCTTGACAAGGGAATCCCCCAC

AGATGACATCGACTTCCCCTCTAAGTTTTTTAAATTCGTCATCTGAAACATCTCGTATGT

CATGAAATTCTATTTCTCCTTCCGTTTGAAAAATGGACTTATAAGATTCTCTAGCAAATT

TATCAATCTCACAAAATCCAATACACTCGTGTCCAACACTTTCCATTCCAAGTCGAAAGC

CACCGATACCTGAAAATAAATCAATAAATCTCATTTCTTTATCACCCCTTTCTTATCGAC

TATCATTGTCAGAACTGTCACTCCACTTGAAATGACAATGAGCATAACTAATATCCACTC

GATTGGCGACATTTAACCACCTCCTTTTCTAACACGGTTATTCCAAACGGAATACAAGCC

ATTAAACACAAATTCAGCAAGGACTTCCGCTCGTCTAACAAAGCGAACATTGTCAAGAGC

ATACTGATAGATTCTCTCAAAATCAGTCATAGCAATTTCACTGGCTGTTTCAGAAAAACC

CTCTCGTCTAAATTGATCTTGTACCAATCCCCAAATATAATCTCGATCATATTTAGTGAC

CTTTTCTACTTTTCTTTTCAAGATAAGTTGAGTATTCCTATCCTCCTCATCCTCAATAAA

TAAAGAATCAGTCTCACTATATTTAGTCTCACTAACTTCAGTCTCACTAGGGGCTGAATA

TGAGACGGGGCCCGTTTCATTTTCAACCTGCCCTAGTCTTTTTTTACACTAGGCCTGTTT

GAATTACCTACTGGGGTAGAAGATAATTCCCCTAAATAAATCTTATTAGCTAGTCTTCCT

TTCTCACTTGAAGACTGTTGAACTTCATCAATTAAGTCATATTCTTTAAGTGTTTTTTTG

ATGGACAATAATTTGGACTTCGAACAACCTAACAGCCTCATCAGTTTAGAATTAGAAAAT

ACTAAATAGACCGCGCCCTCTTCATCTATCCAACCACGACTGAGAGATAATTCTAAACGA

TCTTTTAAAATAGAATAAGCCACCTTTACTTCTAGTTTCATATCCATATATTTCTCATCC

TCAAAAAGAATTTTAGGTAATTTGTAATACCGTTCTGAAGTTTGGTATTGATTTGCGGTG

ATTCGTTTCATAGCGCTCCTCCAAGTTCTTTGAGAATTAAATCTCCACCTGATTCCAAGC

GAACCAAGCCACTTTTTTCCAATTCAGCCATAAGAGATATAGCCTCAACAATATCTATTC

CCATTTCACGTACTAAAAATGAAATGACAATATAGCGTGATGATTGTAATTCTTTTGTCA

TTTTCCCCCCTGAAAATAAAAAAACGGAAAGATTGTGAAATTCCTTTCCGTAACATAATA

AATATTTGAACACCAAACTCAACACCAAAAAAATGTTTTTATTGATGATTATTGCTCAAT

CAAGCACATATAACAATGTGAAGAATTAAAATCTAAACCAATAAAAACCCTTGAATTTCA

AGGGTTTTAAGCATTTATTCTACTTCACAATGTTTTATTATTTTAAATCTTCTAGACCAA

CCATTGAATAGTAGCCATTGAGTTTTTTCCTTTCATAGCAAGGATTTAGATCCCCTATTT

TATTTTACTATTGTTTGAACA

>ICESpnGA17227

AACCATTGAATAGTAGCCATTGCATTTTTTTCCTTTCGTAGCAAGGGTTTAGCACCCCTC

ATTTATTTTACTATCGTCTAAACACCAAGCGAACACCAAAACTACCACGAAATGGAAAAA

CCTTTGATTTGATTCCCGCTTAATTTCACAATCTTTATATCAAACTGTGGGTGATATTTG

ACAATATCTTTTTTGATTTTTAACAGTAAATTTGAAACAATATGTTTGGGTGAGTAACGT

GAACCAAGGTGTAATAAGTCTTTAAATTCATCAACACTTAACTCTACTTTATTGTTATCA

TCACAAGTTTCAATGAATTGTTCAATCATTCTCGAATATTTACAGGTGTAACTCTTCAAT

TCTTTAAAGTGAAAATTGTGCTTTTCTACAAATTGATTTAAAGCAGTTACAGTATTTTCT

TGTGAACGACTGATGTTATGGGTGTAGCCCATCGTGGTTTCAAAATTCGCATGACCAACT

CTAGTCATAATATCCTTTACAGCTATGTACATTTCATTACTTTGAAGATAACTAATGTGC

ATATGTCTGAAAGAATGAGGGGTCACATGCTTGACCCATTTAAAACCATAATCCTTCTGA

CAATTTTTTAACAACTTCTCCTCAACCCTCTTCAAAACTTGACGAAATGAGTGAGAAGTT

ATCGGAGAACCATACTCAGTTTGAAATACACTATCACACTTTTTGAAAGCAGGACTAGGA

TTTTGTTCCATATAGGCTTCAAATTCCTGATTTCTTTTTATAGCTCTCTGGATTGCTTCA

CTCGCTGCTATTGGGAGGGCTACCTCTCTATCAGCATTGACGGTCTTAGTCGTATCAAAA

TAAAATTCATCAACTTTTAAATCATGATATTGGAGAGCCTTATCAATATGTAAAATCCCC

TTATCAAAATCAACATCTGATGGTAAAAAAGCAGCCTCTCCAATCCGAATCCCTGTCAGT

AATAATACAATAGCAAGGTCATAATAGCTAGCATTTCTACACTGCCCTAAAACATCAAAA

AAGGCTTGCAATTCATGAATTTCAAGAAATTTAGCATCAAGCCTTTTTCTTGCTTCTCTC

TTTTTCTCCAGAGGCACATCTATTTTAACAGCGGTCATTGGAGAGTACTTAATGACATTG

TATAGCACACCGTGATTAAATATCTTGTTACACGTACTTTTTATATGTGTCATTGTTGAA

GGTGAAGCGTCGTATTTATCCAGATATTTATTAAGACAATTTTTCATCAGTAGTGGTGTA

ATTCGATCGAGTAAAAATTCATCACCGATAATTTTACCAAGCCGTTCTATCACCAACTCT

TCCCTTTTTATTGTCTGGGGCTTGACAGATACGCACCAAGTTTGAAACCAACTCTCTTTT

AACTCTCCAAAAGTTGTAATCATTTCAGGATTGTACTGACTTTCAAGGGAAGAGGTCAGT

TTTTCTATTTTATCCATTACCTCTCTTTCAGCTTGCTTTCGTGCTCTACTAGTATTTCTA

GTAGAGCTGACAGTTACTGTTCTCCACTTATCAGTAAGTGGATCTTTACATTTTTCAACC

ACTTGATACAAGGGTTGTCCTTTTGAATTTGTTTTAGTTACATAATACATAATTTAATTC

CTCGAAAAGAAATAAATCATTGTTGTATAGAATAAATAACTACTCTATATTATACCATGA

CTTCCTTATTTTGTCCGCTTTTGTTTCCATCTCAAAAAGCATTCGAATCCCTCAAGATTT

ATAAACACCAGCTTATGTGTAGGATTGATGACGTATAAAGAAAATCTCTTATTTTCTCTC

ATTTCTTTGATCCATCTGTTAAGAGTGTAGAGATTCAAACCTTCCCATCGTTTCAGTATG

CTTTTCTTATCCGCCCACTCAGCTACCGAATTTTCAACAGATACGTATTTTATCTCCATA

TTATCATCCTTTCGAATAATTTCTTCTCCCTCCATGGCATGACTGATGGGGTCATCATAA

GAATCTCACTTACTGCTCTTTTACGACGGCAGCCTGAACGTTCAGAAGTATCATTGTATA

TCATTTGCATCATGGCAGATAGAGTATCGCTCTATTTTATTGGTGATTGTCCCCGCTCCT

ATCTTGGCGAATCAAGCAAAACTGCTCCACAAATGAGGAAAGTACCATTTTAGGCTATTC

AGTTGTCAAAGGGCAAATAAAAAATCTCACTCATTTTCAAACTCTATGAAATTGAGGAGA

TTATTTTCTAAAGCGAATTTTATCAGGAAAATCACAGTTAATCCCAATAAATAGTCTGTA

CTGACGTCATAGAAATCTGAGAGTTTAACCAATACATCAGCCGTCAACGCATGCTCACCC

CGTTCAATTTTGGCATAAGCTGAATTTGTAAACGAAAGTATTGTAGCTATTTGCTTTTGG

GGCAGATCATGATCCTCCCTCAAATCTCTCAAACGTCTGTACATCTAGCTTTCTCCTAAG

AAAAAGTATAGACCATATATTGTAACTATTTAGGATATTGTCCAAATCGGACAACAGGAA

TTTTACCATCCTTTATATAGACTTTTATTTTTATGTATCAAGTTTTTTAAATCAAAACCA

CCAGTGTGAACTGGTGGTTTGTTCTGCGGCTATAAGCCGCTTCTACCGGCCAGGGCCAAA

GGCCCACCGAAATAGCTTCCTCGCGCACCACTTTCCCGAGCAGGTGCTAAAGCACCTTAG

TTACTTCCTCTTATTTATTTCGCCAGTAAACGGATCTACTGACTCGAATAACGTGAGCTG

GTCTGCTACTCTGTCTTCTTGTAATTGATTCTGAATATATTCAGCTATCACTTTCTGATT

ACGGCCTACCGTATCTACATAATAGCCTCTACACCAAAACTTGCGATTGCCATATTTGTA

TTTTAAATTCGCATGCTTATCAAAAATCATCAAACTGCTCTTGCCCTTTAAATAGCCCAT

AAAGGACAAAACACTAAGTTTCGGAGGAATACTGATAAGCATGTGAATATGGTCTGAACA

AGCATTCGCTTCATGGATTATTACACCCTTACGCTCACATAAGTCACGTATGATTCTTCC

GATACTAGCTTTGTATCTGCCATAAATGATTTGACGACGATATTTGGGTGCAAAAACAAT

ATGATATTTACAATTCCATGTGGTATGTGATAAACTTTGATTATCCTCTCTCATGAGGTA

CCTCCTGTATGATATGTTGTAGTGGCGGAGAAACCACTTCTATCTTATCATTTTAGGAGG

TTCTTTTTGTTACCACGCTAAAAGCTCTATGGAACCACTAGCATAGCTAGTGGTTTTCGG

GAGACAACAAAAAAGTTGGATGACCCAACTTTTATCTTTCTATCAGTATCAATTTTATCT

TTGGTATAATAAAAGATACAGATATAAGGACAAGTAATGATGTTAACAATAAGAAAACTA

ATTCCAAAGTTGTTGAATTTTTAAAATAAAAGTTTCTCAAAATAGTTAATTGATAAGTTA

AGGGATTTAACTGACCAATCCATCTCACAATACTAGGAGCAGAATCTATTAAGTAGAAAA

CAGGAGCTGTAAAACCAAGCGGTGTTAGTACAAAACGTATTACAATATCACGTCTTCTGT

AATCATTGATAAACATTGTAAGTAAAATTGCAAGGGAGTCCCAAAATAATAAAACAACAA

TAGATAACAAAACTGTATAAAAAAATCTATCCATTGCGATATTTATCTCAAACGCTAACG

TAATTATATATATAATAATTTCTTGAACTATCAACCCTAATATAGAATAGATACTCATCC

CTAAAATATAATATAAAGGACGAACTCCACTGCATAACTTAAGAGCAAGTAGCCCATATT

TTTTATCTATTGTCACCCTATAAATAACTTGAGTCATCTGTCCGATTATCAATAAAGCTA

TAACACCAATGATTGTATATTCTGCATAAGAGGTTTTTATATCATTAAACTGAATGTTCT

TTATCGAAGAATTTAATCCTATTATTATAAAAACAACATATAAGATTGGTTGTAGAAACT

GAGAAATAATTAAACCTTTACTTCTAAAAAAGCAATAATCTCATTTTTAGCAATAATCGC

TAGACCTCTTAAACCTAAGTCCGATTTGTGCCTTAATTGATCAGCCTTCATTTTTCTCCT

CCTATTCTTTGCAAATAACTTTCTTGTAAGGTTAATTTACTTGTTGAAAAGTTTTTAATT

TCACATGCTTTTCCTACCTCATTGATAACATCTAAGATATTTTCTTCTATAGGGACTTCT

ATTGTAAAACTATCATTATCTTCGATGTGAACTTTAAATCTAAAATTTTCTAAAAATTCA

ATTTGATATCTAGAAATTCTATTCTGCATTGAAAAATTTAATTTGATAGTTGAATTATCT

ACAAAGTCACGCATATCACCAAAAAATGATATGGAGCCATTTTGTAAAAAAAGTATTTTT

TACAAAACTTTTCGAGTAGATTTATGTCATGTGAAGATATGATAATAGTTTTTCCTTCCA

AACTCTTATCTTTTAAATACATTAAAAATTTTTCGGCAGATTCAGTATCTAAACCAACGG

TAGGTTCATCTAAAATATAAAAATCTGGATTATGAGCTATTGCTCTAGCAATCTGGACGC

GTTGCAGTTGTCCACCTGACAATGTTTCTACCAAATTATTTTTTTATCAGCTAACCCAAC

AATTTCTAAGGCTATTTGACACAACTTCTCAGCATTTTTCCCAAACTTTCCAGCAAGGTT

CAGCCCCAATATTACATTGTCCTTCACAGTAGTATAAAAATCAATTACTGTGGTTTGAGG

AGTAAAGCCTAAATGAGCAAAAGGTTGAGAACTAACTATCAAATCTTTATCACAATACTC

TATTTCTCCTAAATAAATCTCTTGAAAACCTACAATAGAATTAATTAACGTAGATTTACC

TGCACCATTTGAACCTAATAAGCCAATGAAATCTCCCTTATTTATTTGTAAAGTTATATC

TTTTAAAGCTATTACGTCATCATAACGAACACTTACATTATTCATTTTCAAGAAACAATT

CATAACACCCCTACCTTTATTTTAATACCTCTATAAAATCTTTTTTAATTCAGAAAAAGT

ATCATATTTATAATTGAACGCTTTTTTATAAATATCATAAAGCCAATCTGATTTATCAAG

TGTGTCTAAGCGACGCGAATTAAAATTCATTGCATACTCCATCGCTTCTAAAAAACTCAT

TTTTGAAAAGACGTTAAAATCATCTAAATTCTGACTCCAATATAATAACAAAACCAATCC

CATAATATCCTCTGGTTGATTATTCAATAAATTTAAGTTGGTTTCATAAAACCCTGGAGT

TCCAAATAGAGGCAACTTTTTTTCTTCAATTTGAGTTTCTTTCCTTAGGGCATGCTCAAA

GTCTATAATATAAATATTATTTCTATTATCAATAAGTATATTATTAAATGATAAATCTCT

ATAGGAAAGATTATATTTGGAGTTTATTATCTCCATATAATCAATTAATGTTAAAAACCA

ATCATACGAGCCACTAACCATATTATACTCGCTTAATTTATCTGCAATAATAAACTCAAA

TTCCACAAAATACGAATTCTTTATGTAAAAATCGTTAAAAACTTTTGGAGTAAATTCCTC

CTTTTCCAATTCTACTAATATTTCTCTTTCATTTATTAAACGATTCACAGAATCTCTATT

TGTAAAATCAACCAACGATAAATCACTAGCTTCTTTTAATAAAGAATAAACTCGCTTTTG

AGTATTAAATACTTTATAAACTCCACCTTTGGCATTTTTAGAAATCACTTCCAAAATAAT

ATATTGATCAGGAATAGTGTTATATCTTGGAATATAGTAATCCCTTATTGGAACATTCAC

ATTTGAAGGGATTTTCTTATCTCTTTTATCCTTGAAAGTGCTATCTTTTACGAACTCCCC

ATATCTGTAATATACAACCTCGCTAAGTTGAAATCTGAAATCTGATGGTATGTTTACACC

CTTTACACCTTTATACAATATTTCTAATTTGTGTAACAAACGTTGAAACTCTTTATTATC

TTTTGGATAAATTGTAATGAATTTCCCGACTTGTGAATAACCATTAAGCCCTGTATTTTG

CAAAGAAAGTTCTTTAATGCTAACCAAAATTTTGAAATTTATCTTCTTCTCTCTAGAAAA

TATAAAATCAAAGAATTTTTTAGCAACCAAATTAGCATTTAATATTGAGGCGCTCAGGTG

TATTTTAAATCCCTTAGATTGGGTGATATTAGACGGCAAATTATATAACCAATGTTCATC

ACTAAAATTATCACTAATTTTATATTCTAATAATAAATTATGGTATGCGTCTTCTATTTC

AGTTTCATAGTCCAAATAGTTTAAATACTTTTCGTAATTCATATTAAGAAATCTTCTCCA

TAAATTTTTAGACCATCATTTAAAGCCAAACAATTTAAAGCGTGATAATAAATGTTGATA

ATCAATGTAACTTTCAGTCCTCTATTTTGTAATTCCTTCACCAATAATTTTATGCTATAT

CTATTTTCTCGAGGCAATTTATAGGACTTCAAGATAAAACCATAAAAGAGATAAGTATTA

TAATCTGACAATCCAGTTTCAGAATAATTTTTTAGAAAAATATCTAGTGATTCTGATAAT

TCATCCGGAATAATTCTTTTAACATCGTATTTATTTTTCATATCGGCCACTCTTCCTTAA

AAAGCTCACAATAAAATTTTAAATTTCTATACAACAATCCGAGAGTAGTCTCACAATTTG

AACATTTCACATCACTCTTAATATATAAAAAATGAATTAATCAGAAACCTCTGACTAAGA

TTTCCTAATTAATTCACTTTCTATATCATAGTAAGGAATTCTATTATCCCTAATTGAAAA

TTGAAATTTTATGTTTTATATATTAACAATTATGCGGATTGTAAATCTTGTCTAACAAAA

TGGCAAGTGCTACTATGTGCCCCAGAAGGCGATGCAACGCTATTTTGAATTGAAAGAGCA

TAATCATCCATATCATTTAAGTCACGGATTAGCAATGCTTCCTTCTCTCTTCCGACAATT

CCAAATTTTCTAATTACCTTTTCAGGATTATCAAAAAATTCTCCAACAACTTCCATATTT

CCTTGAAGTTCATTCAAGAAAGCTTTCATTTGACTACTCATTATATAGCTCCTTTTCTAT

TACTTTATTTGGAATCAAAACTTACTTGTACATTGGAAACACCTCTATTCTACGCTTTCA

TATTGCTGCATGACACTTTCAAAATCAAATTGCTAAAAATAATTTTTTAAAGCTTAATTT

AGATTTAATTACATATATCTCAAAAAATTGTTTTGAAATTAGTAAATTAAAATAGGTTTC

TGTACTTATAGGAACTAGTTATAAAAACTTCGCCCATCATAAAATATCTATTTAAGTAAA

ACAAAAATTTTATAATTTTTTGATTTTTAAGTGACTATAATCTCCTATCTATAAATACCA

TTCGCAGGACCTGGATCAATCCCTCTAGCCATCTTATGAACTTGAGTTCCTCCAGCAGTC

CCTAATAGGATTAATCCTATGACTAACAAAGCAACAATTTTCCTAAACAAACTTCTCATA

TACCTCTCCTTTCGTTTTCTTGCATTTATTTTATCACTTCAAAATTAGCTTGTCAAGTAT

TTTTATAATCTTTTTTATTTTTTTATTTAATACTTTTTTGTATTCAAATTAGATTTAAAA

AACGATGCTCACTAGAACACCGTATTCCTTATTCTGTTATATCTGCAAAATAATGTTCGA

CTTTCAAAGCCATTGACATGTTTTCCTCAAGTTTGTAGAGAAAATGAGCAGTCTCAAAAT

ATTCTTTTACTGAACTTTTAGAAGAAAAATTTTCACTTACACTTCCCATCAATAAATACA

AATCAGCTAATAGATAAGTTGTCCTGTACTCCTTACATTGCTTTATCGTAGCTGTGATTT

TCGTAATAGCTTCTTCAATATTTTTCTGTAACCATAGGTATCGACAAACGTTATAATTGA

ACTTAATTGATAGTTCTAACTCTTCAATTGTGTTTAAATTAAGCTGATTCACTTGATGCT

CTAGCTTGTCTCTAATCTCATTAAAACGTTCTAAATCTTCAATATCATAATAAAAATTGA

ATAGAGTGTTTGAAACTTGAAGGTAGTTCAAATCAGATACATTTAACTGAGGTAGCATAT

TCTCTAATCGTGTAATTGCCTCTTCTTTCTGTCCATAAAAATAAAAATCTATAAGAGACT

TTATCCATTCCATGTAAAATTTATCTGCTAGAGATAAGCGATGGGCTTTTACATTTTCCA

ATTCATATATATATTTTAAAGATTCATAATTACGATTTGTGATAAATGTCTGTGCTAACT

TCTTAAACTCTGATAACTCATTGACTTCCTCTACAACCTGCTCATCAAAAAAGTAGTCCA

TACTAACTTTTAATTTCTTAGCAAGGGCATGTAAAAAATCCGCTCCAGGGGTGAACTCTC

CACTCTCTAATCGACTAATTTGCCCCTGTTTACAGATACCCTCAGCTAATTCTCGCTGAG

ACATTTTTAATTCTTTTCGTCTATTTTTTAATCTTGTTGCTAATAGTGTACCCACGTTTG

CATCTTCCTTTTCTATTTGATTTCAATGTTAATTGTAACATGATTAAATCTAAATTTCAA

ATATATTTTACAAAAGATACACATTGTATATAATTATTGGCTAGATTCCTATTTTTGAAA

ATCTATCCCATCTCCTTTTGAAAGTCCTGTATTTAAGCGATTTATAAATTTATCTAGCCT

GACTACCATCTTTTCATACCTGCCCCTTTCCAATTCCAGTTGATTCCCCAGGCTCTTCAA

TTTTTTCTCAACAATATTAGCACTGATATTCTCAGTTATATTCAGTTTTGAAAGATTTAA

TTTCATTTCTTGCTTACTTTCAACTGAAGCCAGTAGATATTCTGCTACTTGATTCAAGGT

TGACATTCGTTCTTGCACCTCATTGATTCTCAATTCAGACGCTGCTAGTTCATGCACTAA

CTCATCTTTAATCGTGACATAAGATTGATTTTCTACTTCCAGTTCAATCAAAGCATCCAC

TTCCGCAATCTTCTCTTTAATCATATCGACTGTCGCTTTTCTGCGTAATGGAATGGTTTG

ATTTTCATAGCTGAACTGTCTAATTAAGGTTCGACCTTTCATATAACAATTTTTCTCGGC

AGCGTCTTTGTGGTAGACATAGTAGGAGCTGGTTTCCCTGATGAAAACCTTCACCTTATC

CTCTTCCATATCAAGCTGCATGTTCGGCACAAAGATAAGTCCCTCCTGACGAATACCAAA

CTTGACCTTGACGTAAATTCCATCATCCACTACTTTTTCAATTTGATTGAGTGACAACTC

CACCTCAAATTCATGAACGGCATCTCTGTTACTCTTGAACTCTTGATAGGACTCCCAGAA

CTTCTCATCGCTTGGAAGTTCTTTTTCTTTGGATAACTTTTCTTCTTGATAAAGTTGAAC

GAAATCCTCAGTTTCTGGAGCTTGCCAATCTTTTCTATTTTTAAAATAATCTTGGAAAAA

CTCTACATCATAAAGATTTTTCTGGTCTAGCTCTGTCTCCTTTACCTCCACTCCTGCAAA

TTGAAAAGAAACATGCTTTTGTTTAGGATTGATAGTTAGTCCAAAAAGTTTTGCTTTCTG

AAGTAAATCATCCATATTCTCAACTTTCAGCAATAAAAATTCCATGAGACTTTCTATTTC

TCTTTTGGCAAAGTAGTTCTTAAAAAATTCTTCTGTGTAAGGCTGCTTGCGATTGAGTTG

CTTGCCACGCACCACCTGTTTCATAGTTGAGTCCGTAATAAAAAAGGTGGCATGCTTGTG

ACGGAAATCCATCTCCACATGTAGTAGCGGAGCATTCTTTTTGAAATCCTCAAAGTCCCT

AGAATGTTCCATCAAAAAATAGAGTCGCTGCTTGAGTTCATACTTGTGATTAGTCTTACG

ATAGACTTCATACCGCTGGTGAGAATAGCGGTTCTCAATGATTTTAGCACCTGCGATTTT

AGAAAAACGGTCAGAAATCATGCGAAGATTTCGCTCCACCTTGTAGTCCCACTTGAGCTT

TTTGTCAGAATTGCTATCTACTGAATTGATAATGATGTGATTGTGCAGGTGGTCTTTATC

AACATGGGTCGCAACGATAAAACGAAATTTGCCACCAGTTAATTCCTTCACAGTCTCATA

ACCTATCCGATTGATTTGTTCAGGAGTGAGATGATCCTCTGGCGAGAAAGACTGAATGAT

GTGGTGAGCGTGTATTTTTCGTTGATTTTCTTCCATCCTGTCGTGGCGAAAATCGTAAAG

CGTATCGTTGCTGATGAAATTTTCATGATACATCTGCACCATTTCCTCATAGCTAGGAAA

GTCCAGAAAATTCTTCATGCCATAGTCCGACACCAAGGCAAGATTATTGGTTTTCTCAGG

ATTGAGAATGTACTTGATAAGCTTTCTGCGGTAACTCTTTCCGTGAATGGCAAAGTGTTT

AGTGATGACCATGGAACTCCTTTAGCTTCTGCGCTTGCAGATTAAATTCCTTATCAACTT

CTTTTATCAATTCACCAATTCCTTTTTTCAACTCCTGCAATTCTTCACCAGAAATTAACT

GAGATTGATTAACACTTCGAGCAATCTGGTTGATATTATTGCCAATCCTCTTTAACTCAA

ACACTAAATCTTGGTAACCGTTTGTGTCAATCGTGATAAAATTCATACCAGGATCGAGTA

GAGTTCGTCTCGCATATTCAGAAAAGGATTTACAACCACTTTGATCGATATTTATATTTA

GTTGGTTCAACTCACTATCAGACAAAAACACTTTCTTGAGATTGGTTCGGTAACGATGTT

CCATGTGCTTACCTCATGTACTTCTCACGAAATTCTTTACTGAGGGGTATGGTGTTTGCA

ACCTCTTGAATCAATTCCTGCACGCACGTTAAGAGAATAGATACATGCTCTTGGGTGACT

TGACGTTCTGACTGTGCCAAGATTAAAACTTCATGAACGTCACGACTGATTTGTTCTAGT

TTTTGGGATTGCCAGAGGGCAAACCATGTCTCCATCTGTTTTTGTAAATCAGAGGAAAGT

AAACTTTTACGAAGAAAATCAGAGAAATTAGTCTCTCCCCGCTCTCTCATCAAAGCTAGA

ATTTGCTTTTCTTCTGCTTCTGTTAAACGAAATTGTTTCCGAATATCACGCACATCTCGT

TTCATACTGCTACTTCCTTTCCAAAATACTTGTACTGACAATGTGAGCTACCATACAATG

TCAGTACACTTTTCCAACAACTGATTTTCATTCTTGTAAGGCTTTGCGAGCTCAGATATT

GTGTCCACAATATCCCAAAAATCATATCGCCAGCCTATCAAAACTTTCTAGTTTTGACAG

CCAACGATAAGATAACTTGGTGACTTCGCCCCCAAACCCCCATAGAAAATCAAAAATTGA

TTTTCTATGAATGATATTAGGATAACAGGGAAGATTTGAAAAAGGTATCACAGCTAATAT

AGTTATGATTGACTTTCTCTGATTCTATGATAAAATTTCTGTAAACTAATATTTGGAGAA

ATAAAAATGCTGAGTCTAGATCAAATACACTTACTATTGAATACACCCGAAGATGAGTTT

CATGATTTTAAACAAAAATGGCATCATTCAAAAACTGAATTGGTGCGTGATATCTTAAAT

TTTGTCAATACATCACATCACGAAGATTGTTATATCATCTTTGGAATTGATAATATCACT

TTAGATATAATCGGTGTAAACAATGATGATAATAGAAGAAATGAAGAAGATCTAACAGAT

TTACTACATAAACTCTTTATATCAACAAATAATCAAATTAAAATTAGCATACAAACTGAA

ACTATAGACAACAAAGAAATTGACATCTTAATTATTCATGATACAGATAAAGTTCCTGTA

TTTTTAACAAAAGATTATAAGCCTAAGAAAGATACTGCATTACCAAAAGGATTAATATAT

GCTAGAAATGGCTCTATAAATACCCCTAAAGACTCCTCTGCTCCTTTTGAGTTAATAAAT

GAGTTGTTTCAAAAGTTTAATCATACCGACTTAAATATCAAAGAGCAGTATTTTCATGTT

TTAAAAGACTATAAAAATTGGTTCTTCATTGAAAATGAAGACGGAAGATTTTTTATTTAT

AATCCTAATCCCGATTTTTATATTAAACTTAACGACGATGATGCAAATCGTTTCAAAACT

ATGTCTTATAGTTTAAATCAATACCAGACTAATATTGATTGGCAATTAGTACAACTTCGA

TATCGTCATCTTACAATTGACGAGTGTATGGCTATGTATTTAGATCAAGGTAATTGTCTA

GTCCCTTCCCCTGAAGTAGAAAGTTTTAAAATAGATTATCAGGAGACTATTTATTATCAC

TGCTTATACAAAAATACTTTAAAGTACCAATTGCTGAAAGTATTTTCTTCAATACCTGGT

CTCGAAAAATATCCTTTAACTAGATTTAAAAATAGTATAGTAATTTATGACACAAAATTA

GAATTGAAAAAGACTCACAACTTGATAAATAGTAAATTCTCATCAAAAGACATAGTGAAT

CAACTTGAAGTTACAGAAAAAGATTTCGATTTTTACTATAAAAAAGCTAGACATAAAAAT

CCTGATTATAGCATCCAAGAAAATCAAGTAAATCTAACTGAATTAAACTTAGTTAGGTTA

TTGAAAAGTTTTCAAAAAACATATCTTGATAACCCACTATAATCAATCTTTTTAACTAGT

AAAAAAGATAAAACAAGGCATAGAAATTAGTTTATCTAAAAAACTAATTTCTATGCCTTA

ATCATTATTAATTAATAACATCAACTAAATTGGATTGAGTCAGCAATGAATATATCTCAT

TAATATTTCTATTATTTTGCGGACTATTATTTCTTAATTTACAGTATGTGAATATTAAGT

GACTTTTTGCTCTTGAAAGTGCAACAAAGAACGCACTTTTATCTTCTTCTGGTTGATCAT

TAAAACTCCAGAATGCAGAATCCTCCAGCCCTAAAAAATAAACTGCCTCATATTCTAATC

CTTTACTTTTGTGTATAGTCATGATTGAAATTGAGTTTTCACCTTTGAAATTAGATATTA

TATCTATCCATGCACCTTGTGATTGAGAATACTCTTCATATAATAGTTTTGAAAACTTAT

TGATGGTGATAAGCAAGTCATTTTTCCCATTGTACATTGGAAAGTTGGAAATAATTCTTT

TGTCACCTATTTTTTCGATAATACAATTAATTAAATTTAGCATCCTGTCTTTATCGGGAA

TAGGATTTGAAATTAGGTAAGTAATATCACTAACTATATTGTCAATCTCCTTATAGGATT

TTGCTAAAATTAATTCATCAGTTAACTCATCTATTCCATTGATATTTCCATAAAAATTAC

TAATATTTTCCCAAATTAAAGGATCTCGTTTTCCTTGACTGCATGATATTAAATCTAATA

ATAGGTTACACGTAGGATCTTTTAAAATATCCTGATATTCATTTTCAATTCTTGCTTTAA

TCCCTTTGCCATTTAATATAGATATCAATTCGAAACTATAAATACCAACCTTTTGTTTTG

CTAGGATACAAATTTCTGATGGTCGTATACCTCCTTGAATTTTTGATTCTATATCATTTG

CAATTAACTTAGCTTCTAAACTTTCATTTTCAAATTCAAATAATGTTATTTCACCCTCTT

GAAATTCTAGATAATTATTTGTCTGAATAGAACTGTGATTACTATTTAATATCTGATGAA

CCTCTTTTTGAAATTCTACAAGTTTAGGTACAGAGCGGTGATTCATCAACAATTGATATT

CATTTGAATTAAAGTCTCGAATATAGTCTGGAAAAATATCAGGCTTTGCACCTGCCCATC

TCATAATGGCTTGCTTATCATCTCCAACTGCTGTTAATTTACAAGACGAACCTAAAAAAC

AAGTTTTTAATAAACTATACTGGGCATATGTAGTATCCTGAAATTCATCTAGAAATACAA

AATCGTATGTCAGTTGAAGTGCTTTACGGATGTATTCGTTAGTAGCTATTATTTGAGTAC

TCAACTTTGTTATTTGACTATACAAGAGGACCGGCTTATTATCTTGTGTTCCTTTTAGTA

GATTCTTTTTAAATTTGTGAGTATTCCCGTCATTAAGTATAATATTTTCGACAAAATTTC

TTATATCTGACATTCTCCGTCCATTAACATTAATTCCATTCATAGAGAGTAGTTCTTTTA

TAGTATCCCAATCTTCAATCAAATAATCTCTTGATGGTCGTATATCCTCAGGTAGAACAT

CTCTAAATTGGTCTAGAATTCTCTTTTCAAAAGCCGAATAGGTTAATGAGGTAAAACGGG

AAGCATATTCATCACCATAACGTTTCTTAACTCTTTCTTTCAAATTTGATGCAGCATCCG

TTTTGAAACTTAATGCTAGAATTTTTTTCGGAGAAACGCATTTATTTGTAGAGAATAGAT

AGTCTAACTTTTGAGCTAGTAATTCAGTTTTCCCCGCACCTGGTCCAGCAATGACTAAAC

AGTTAGTCACATCTTTGACGGCTCCTAATGCAGTCTCTTCTAAAATGATATCTCCTTTAG

GAAACCACTCCTCACTCTTTACCATGTTCAATATCTCCAAGTAATTCTTCAGCACGGTTA

AATACAGGGGGTAGATTTCTTGTTAATTCATCATCACTAATAGATGATAAAAATTGCATA

TGAGTTGTTGGCTTTCCTCTACCTAAAAAGAAATATTGATATCAAATCATTAGTTCCTTT

TCCTCTTCAGTGAAGCTATCACCGGGTCCGCTCTTATCTTTTAAAGTTGCTTGTATAGCT

TCACTGATGCGATTTTTAAAACCAATTAATTGATAATCATTTGTTCTATCCATTTTAGTT

AATTTTATTTTTTATTGTCACTATTTTCATCAGTATAAGAAACAACAGGTCCCTCATTGG

TAGATAAAGTTTCCAGATAATGTTCTTTATAATACTGTAACATTAAGAAATCAATATCAA

GTGGTGCAGAAAAGAAAACATTGAATTTTTCTAACATTTTAATCCACCCCTCAAGCAACT

GATTATCAGGTCTATCAATCTTCCAGTCCTTTATTTTATCAATCTCATCATCATCTAGAA

CTCGTGATTTTTGAGATTCGGCTGTTACAGTAACAAGTTTTTCTTTAGCAACCCCATTTA

GTATCAATTGTTTTATTGCATATTGAATTCTTCCCCAGCCACCTCCATATCTTTCGTTAT

CGAAATCTAATAAGGTAATATGAGGAATCCTGAGAGCGTTCAATAATTTCCAAAAATAAT

TAACATGCCTACCTCCTAAAGGAACTACTGAAATCTGAGAACTATCAATTTCCAGTCCAA

GTAAATCAAAAAACTTTGGTAATAGCAGTTCCTCACTATCACCTTCACCCAGTACTACTA

ACTTTGCAAAATATAGTTCAGGATAGGCTTGAATTGCTCCCTTAATGTATTTATAAGACT

CATCATTCGATTGAGGTAACTGTATATCAGAAACAATGGTTTGAAGAACACTGTCATTAT

TTTCTATTCTTAAATACCTCAAATCTTCTGGGTCAATTCTTTTTACAATAACTGGAGAAT

GTGAAGTTAAAATTAATTGAGAGTTGTCATTATTGCCTAATTGCTTAAACCTTTTTATCA

GTTTTCCAATGTGATGTGGAGCAATATGATTTTCTGGTTCCTCTATTGCTAAGATAGTTA

GTATTGGAGGTACCAACTTAAATCTTGGATTATCAGGATTCTCTTCACGATCTTTGGTAA

TTTCTAACTCAATATCAAGTATCGAATCAACAAGTGAAAAATAAAAAATAGATCTCAGTC

CATCTCCTAGATCTGAAACTGTAAATGCTTCTTCAGTGGTTGTTGGTGAAAATTTTAAGG

CAATCTGTCTAAGTGCTGCCGCCATCTCAGAAGAATTAATAATCAACTCTGCTTGAGAAA

AACGGTTATCTTCATGATATAATTCCCATGATTTCTGAATCTCATTGTTGATCTGAGTTA

ATGCTCCATTTTCAGATAAAAAAGTGTTATTCAACTCATCAATTTTATCTTTAATTTCTT

TTATCTCATCTTCAGTCCAATTTATACTATTTACCAACCTACTTAACATACTACCTGAAG

CATTACCCAACTCCTTCTCAGGCGTTCTTGAAGCTGGTACATAAAGTACCCTAATTTTAT

CCAAATCCTTTCTAGGAGCACGATGTTTGTCTTCATCTCTAATAGTATCTTCATCAGATG

AAATATAATAAATTTGAGTATCAATACTTCCTTCAACTGTTCCATCATCTTCCCAGGAAG

ATTCTAATCTGATTCGTAAAAAAGGTTTTGCACCATCTTGAGAAACTGTAAAGTGCTCGA

AAAATGTTGGAATGGCTGGTCCATAGACAGTTCCATCAAGTTCATCAAACTCAAAAATAG

TTTCTATAAAAAGATTTCTTGTGTTTTCACCAGGTCTTAACCCTTTTGGTAAATGAAAAT

CACTTTTTCGAATGATTCTATCACTTTGTTTATCTGAAAACAACTTACTCAATGCCTGTA

ATACAGTTGTCTTTCCTGAACTATTATTCCCTATCAGTACAGTTTGATTATTAAAGTTAA

TTGCTTGACACTCCCCAAAAGATTTAAAATTATTAATAACCATTTTTGTTAACTTCATTA

CTTTTCCTCGTTTCCTAAATTCAACTTTTTTAATTTTAAGTGCTATTTTCCACCTTCAAC

ATATCTTCCTCAACCTTACTCTATTACCCAAAAAAGGCATCATTTAAGAGTGACAGATGC

TTAACCTTGATCTATTTTTGAAGCATATACACATCTTCTATAAATTCTGTTGAAATGGTA

CTTGTTCTGTTTTTATAACAGCTAAGGGCTATTGCGTAAAATTTATACAATTCGAGCAAA

TCTGTTTTTGTCAGGCCATGTGTCTTTAGTAGAATTTTATATTGTTACCAATTATGTATG

CTCCTTTTACTTGATAACACTATTATAACATTTAAAAATAAAAAACATGTTCTAAATTAA

TAGAAAGTATAATTCTAAAATTAGAATTTTTCGTGATAATCCTATAAAATGCTACTCTTC

TAATAAACTTGTACTTATTTCCTTCAAACTACACAATTCTCTAGTTGTTGTAAATTTGTA

ATTGACTCAAAAGTATTCGATATAATTTCAAAACAGTATTTTACTTTATAGTTGACCAAT

AACATTTAATTCTAACTAAAAAAATCATTATACAAATCAAGTTACTAAAGATGACTTTAT

GGTATACTAGTGATAATAAATTGTTATCGGTAGGAGTATGAAAAATGGATGATAAAACAT

GCTTTGTAGTTATGGGTTACGGAGTAAAGAAAATTCCTGGGACGTTGGTGAAGCTAAACT

TAGATGATATATATTTTAAGTTTATAAAGCCAGTTTTATCACGGAATAATTTGAACTCCA

TTTACTCCACAAATGCATATCGAGGTGATGAAGTCCCTACTTCAGTAGCTATTAACAAAA

ATTTCATTACATCTATTTTTTTAGCTGACATAGTAATTGCTGATATAAGTACTCTGAATC

AAAATGCGATCTATGAGTTAGGACTTCGTCATGCAATGAAACCTAAGTCTACAATTATTC

TTTGTGAGGAAATTACCATTCGCAAATATCCATTTTTTGATATCTCAATGAGTCCACAGA

TAAGATATCATAGAAAGAAAATATTATCGGATGAAAACTATAGAAAATCCGTTCAGGAGC

AATTGGAAAAAACTTTAATCGCTTGTTTAAATTCTAATCCAGAATACATTGATAGTCCAG

TATTTGATTATAATTTATATAGAATTGAACCATTAATTCAATTGTCAAAAAACGAAGATA

CAGAAGGCGACTCGATAAGGACTATGATTCAAGAAGCTGAAAATTTGAAGGAAAATATGC

AGTTTGAAGAAGCCGAGAAAAAGTATCTTCAGATTTTAAACTTATCTTTTGATGAAGACA

TGTTGTCTCAATACATTTTATGTCGCTATAAAAAAGATATAACCATAGAAAATCTACTTT

CGACCTTATCATACATTGATAAAAAAATAGACTTAACCATCTCTACAAATGAAAACTTGC

TAGGTATTGTTGCAGCTATAAATAAGCAAATTTTTGCACTTAATAGAGAATGTAAGTTCT

TAGAACAAGCTAGAAGATATTACAAAAATGGTTCAAATTTTGAGAGTGGGAATCTCTATT

GTGCTAGAAATTACTGTGCTCTTTTATTAAAACAATATCGTATATCTAAGGACATCGAAT

CAGTAAAAGAATACTATTACTCTGCTGTTCATTTTGCTAAAACTTGCTTAACAGAATTAC

AATTTCTCAAACGAGAGGAAACTGAGCTCGATGATACTTGGTTTAATGAAAATGTTAAAG

ATTTGACTTTAATTGCATTTGGTACTGACGAATCAATTATTAAATTTAAGCCTATTACAA

AAAGGCAAGAAGAGACTGTTACCTCAGGTCGTAAAGAGCTAAAGGAAGATTATTACGAAA

CTCTAAAAATAATAAAAGGGAAGTGAGTCAATTTACTCACTTCCCTTTTAAAATCTCTTA

TCTCTTGATAATTTCCAATAATGTAAACCTAGTGGTGTCAAACGACAAGACTTGCTGTTC

ATAGCAGCAAAATACATGTGTTCCTCCCCAACAGGTTCGATCAAACCAACACTTTCATAT

AGTTGCAATTCTTTAAATTTGTCTACATTTGTTTGCTGAGCAAACGGCTCTTTATATTCA

TGAGTAATATCAGGTGTATTGGTAAATTCAAATGATGGGTCTAATTGAAATTCAGAAGTT

GGTTCCACAAAGTAATGATGAAGTTTTCGTAATACCTTTTTGGATACTTTAGCTTCAACA

GTTCTGATAGGTAAAAATCCTGTTATATTTGTTTTAAATAGAGGCCTTTGTTCCCATACT

CCAAGTGCCTGATCGACAAAAGAATACAAACTAGCAGGAGTGACATTCCCGCTAACATCT

GCTGCTCCACCTTTTAGCCCCTGAATTAGTAATTCAGTAAACACACCATGACCAGTTATT

CCATCTTCAATAGCAACTTCATCACGATTGCTTGCTGTTAGAATAGTAACACCTTTTCCA

ATAATGGCATCCGATGAGTCTATAGCACTAAAATCTCCTAACTTTCCAGAATAACAACAA

TCTAAGATGATAATTTTATTTCGACTTTTAGAAGTGTTTGCCAGTCGAAGTATATCATTC

ATAGAAATTCCCATATCATAGCCATTAAAATCTGGTGTTACGATTTGACCAGCCATCTCA

CCTGTCCCGTGTCCTGAGAAATAAAAAAGTGCAATATCGGATTCTCCTTCTTTAAATAAA

GAATGAAGGTCCTCCAATAACTCTGCTTTTGTTTGTACATTACGTTTTAGCTTAACACTG

AAATTAGGGGAACCATCCTCGTTTTCTTCCAATAATTTCGCTATCTCCTCTGCATCATTT

ACACACCCCACTAATGGTGCCTGAGAATATGCATCGATACCAACAATTAATGCCTTATTT

TTTAACATAAATTATTCCTCACAATTGTTTTATCCAAGCGCTAATCTTTTCCCAATTCCA

ATCTACTACAGGAAGATAGCCACATTCATTTGGAATAGTAGCACCGATATGACTATTATT

ACCATAAATTGCAAGAAGTGGGATACCCTCATCTTTGGCACAGTTCATTTCCCAAATTTG

CCCATCGGCATTCTTGGTATTCCTAGTAATGATAGAAATCATACCGTCACACCCTCTAAT

TCTTGTTCTACATTTAGTTTTCCATTGAGAATCCCACGGTTGCTTAACAGACATATCTAC

AAATTCAAATGGGGAGTTCGTATTTCGCGATTGTCCCCTTAGTAAATCTCGAAGATTTTC

ATCTTCAATTGCAAAGCTTATAAAAATTCTTTTCATCTATTTTCTCCTATAAAAGTTTTG

ACTTTATTATACCACTTTAGAAAACGCTTTATACTATTTTTCCTTCAATTTTATTAAATA

TTTTCTCTAGTGATTTTTTCCAACAGTATATTTCTTTTTCTAAAATTTTTTCAAAAAAGA

AATCAATTTGTGAAGTGTTTTCTAAAGTAAAATCTGATACAGATACAATTTTTTGATCAT

CCTTTAAAATACCATCAATACGTAAATTTTTTGCTTAATAATTAAAATGGGAATATTATT

TGAAATTGCCATAGCACCTTCAATTTGACAAAAAGAACTAGTAATCCATTTTCCATCTAA

TGATTGTTCTTCAGCTTCAAAAAAATTTTTATTCTTAACTGCTCCTTTTTTTAAAATACC

AGATTGAATATATGTATGTCCAAAAGCTAAGATGACCATACCGTAACATCTTTTTATCAT

TTCATTCAAAACAGTTAAAGATTCATATGGTGAATAATCTTTTGCTTGCAATGTTATACT

TTCAATATTGTACTTATCTAAGGTACTTTTTAATTTTTCGATAAAGTACTGTTGTTCTTT

AGTAAATGGATTGGGTCTACTCAAAAAAACTCTTATTTTTTATTATTCACACCAGATTTC

TCCTACCGATAACAATTTATTATCACTATACAAAGCACTCAAATAGGTTTATAATAGCAT

GATTATTGATAAAGGAGATTTTTATGGATTATAAACTTATTTCTACTTACTTAGATTATT

GCAAAACTCATAAGCGTTTGAGTTCACACACGATTCGCGCTTATAAGAATGATCTTATGC

AATTTTATAACTCAGACTATGATAATGTCGAATCCTATATAGAAAAGTTGACACGATCTA

ACATAAAAACGAATACATTAAGAAGAAAAATTGCTTGTATGAAGGTGTTTTATAACTATC

TAAAATACCAGAACATCATTGAAGAGAATCCCTTCAATCAATTGCGCTTTCAATTTAGAA

CTGAAAAAGTATTGCCTAAAACGATTCCGTATGACATTCTGAAAAGCATTTTTATATATT

TAGAACAGAAAGTAATTGTATCTAAAACTGACTATCAAAAACAACACGCTGAAAGAAATC

TACTAATTATTTCACTTTTACTTTCAACAGGCATCAGAATTTCTGAACTTTGCCACATTC

ATCTCAAAGACATTAATCTTTCCAATAAGACACTCCATATTATAGGAAAGGGTAAGAAAG

AACGTATCCTATTTTTAGGAGATCAAAAAACATTCAATTTATTAGAAACATATATAAATA

AAACAAGAAATGAATCCAATGATTTCTTGTTCCCAGGGAAACATTCACTTAAACCATTGT

CAGAGCAAAGTGTACGTTTAGTAATAAAGAGAATCGTTGAACAAAATAACTTTTCTAGAA

CTATTACACCGCATATGTTTAGACATAGCTTTGCGACAATGCTTCTAGATAGTGATGTAG

ATATTCGATATATTCAACAAATTCTTGGACATAGTTCTATATCAATCACACAAATCTATA

CTCACGTATCTCATTCAAAACAAAAAGAAATACTTAGTTCTTTTAATCCCGTATCAGTAA

TTCATTCTGAAATCGAGTAAGAGACATCTCCAAAGTCTACAGTTCACTTCATCTTAAATT

AATTTTTGTAGTTCGACTTTAGTTAATAGATTTTATACTCAACAAAAGCAAAAAACATAT

AAAACTTCACTTTGAATCAGCATTTATACTATATTCCGTATTAAGAACTCCATAATTGTT

TTTCTAAAATACATCTTTCAAATATCCTTAAATATAATAAAAAGAGATGGGCAAAATCTC

AATTCCTGAAGAAAACGCATAATTTCAAGTTTTTCAACACCTGAGACTATGCGTTTTTGG

GTTCTAAAAAATTTTCGCCCACCTTAAACAACTAACAAAAGTTATAAGTAGATTATATTT

TAAAATTTGTGTTCTAACAACCATTTCGATTAGTTAAATCTTTCAATCTTTCTTCTCCAG

ATTTAAGCATCTCTTCCTCTACCTTGCTCCATTTTCCGAAGAGGTATTCTTGTAGCACTT

CTGCTGCTGAAGTTGTATTTTCTTTTGAATCATATACACAACTTCTATCTCGTTGGTAAA

TTTGAATTCTTTCAAAGATAGCCAGTTCTTCCAATTGTCGTGTATTATCAACTAGATGAT

TTACAATGAAATCATGATGTTCTTTTGGAGTTGCGCGTGCTTGATTTGGATTGAGAATGT

ACAGTTCTTCGTATCGGATAAGAGTGCTCAGATAGGACAGCTCAGGCTTTGTCGCAATTA

AGGCCAATTGTACTTCATATCCCTTATTTTTCAAGAGTTGTGCTGTTTTCTTTGGAACAT

CAACTGTTCGTAAAGTTCCCTCTATCAAAAGATTGTATCTCAAACTACTCAATTTTGTTA

CTAAAGACTCTACCATTTTTCCTGCAAAATCTTTGGTATATTCTACACTGTCTTTGCCAT

ATTCTTGCTGCAGTTCTAAATAGTGTGTATGCTGAGAACGAAAACTATCACCATCTATGA

TAACAATATTTCCTTGAAATTCTTTCTGTTTAATACGATGAATTGTAGTCTTACCGGCAC

CACTTTGACCTCCAAGCAAAATCGCTATAGGTTGTTTACTGGACTTTTTTCCTCTTGTCA

GTGAACGAATATTCCTTGCTAAAGCATGTTTGAATTCACTATCAGTATAATCTTGGATTT

CCATTAAGCTACCATCCGCTTTTCAGATATCTCTAACATACGTTCAATTCCATCCAAATA

GCCGCTATATCTCTCTATTTCATCAAAATTTTCTACTAAATAGATATTTGTCTGAATCAA

GTCAGATAGATCATCACTCATTAAAATCCAAGGATTAGATTCATCATCAATGCTAATTCC

CTGACTATCTTGATAACGATAGAGTCGAGATAATAGATTAGCACCTCTTTCTTTCACAAT

TTCAATTTTCAAAGTCAATTCATAATCTTCAACAGGATTGAGCATTTTATCTTCTCCTAC

AATATCGACATAAGATACATTAAACTTCTGACAAATGATGTCAATTAATTCCGTAGAGAC

TGAACTAGTTCCATTTTCATAACGACTCAAGCTATTTCGAGAAATTCCTATAATTCGTGC

AAATTCGTGTTGTGTTAAGTCATGTGTTTTACGTAAGGATTTTATGTTTTTTCCAATCAT

GGCAAACTCCTTTTATTTGATACCACCATTATAACATTTAGAAAAAGCAAATGCACCATT

TTTGGTGCGTTATTGACTGTTTTTCCTAAATCTCTCTAAATTCTTCTCCAATTGTTCCTT

ATGAAGTTGATTCTTAGCTTCTTTTAATGTGTCGTCCAAAGTTTCTTCTCTGTAAGTTGC

TTCTGTTTTTAGTTGTTCTGATCCTTGATCTGGAGTGAAAATGATATCTAACAGTCGGTC

TATTTTTTCAAGATCTTGGACAGACATATCAGATAGTCTATGAATTAGCTTCTTAAATAC

ATCACTATTGTCTTGGTTTTTCTTAAAAAATGTTGTAAACATCTATAACCTCTCAAAAAA

AGCAGCCTATCAGGACTGCTTAGTGTAATTCCGAAATCGCATCATAAATGGACTGCAATT

TCTTATTGTCTTTATTCTTTGTGTCAATCAACGTATGTAGCTCTTCAATTTTCTTTTGAC

TACTTTGAATATCACTATTGTTATCCGCAATCAGTCTTTTAAGATGTTGCTGATAACTTG

TAGTGATATCTGTCTCTTTTCCTGTTCTAACCTCTGTAACTTTGGGTTTAGAACTAGAAT

TTGATTCCGTCACAGGTGCTTGTTTGCGCTTTTGAAAAGCCGCTTCATAATTTACATCAT

TATCTCCTTGATGATTTCCAAATAAGGCCGCAATTTTATCTGGATCTTCTTGATTTTCTG

ATTTACTTTCTAATGGTTGATTAAAATTCCAATCTTTTGTCATTCACTCATTTCATCCTT

TCTAAATTCAGATTCATCGAATTGTCTTTCTCTACCGAAGGCATGGTCAAGAGCTTCTTC

AAAACTCATATGACTAATGCTTTGACGCCTTTTGAACGTCGCAAACATCGCTGTCTCCAG

TTGTTCTTTGTAAGCAGCGAGTTTTTCTGTCTCTCTTGCTACCTTACTTTCTTGCCTAGT

GACCTTTTCTTCTAAGCGTTCAATAATGGTCATATACGATCCTCCTTCATTCTAATATTA

GCTAAAACATCTTGATTTTGTTATCACAATTCTAAACATTGAGAGGTTTTTCTTACTGGT

TGATTCCTCAATGTCTCTTTTGCATTCTCGATCATCAATTGTAAATCTGATTTCTTTTCT

CTAAGGACATCATTCCAGTCTACTTTTTCTTTCCCAGATTTATTATCAGGGAAATCCAGG

AAAACAGGAAATCCTGATTGAGATAACTTATCAGAAAAATCTTTTCCTGCATCATCACAA

TCTACCGCAAGTGTCAATAAATCAGGATGATTATCAAAATAGCTGGTGGTATCACGAATT

GTATTGATTAAAGGCAATAACTTTGAAGGTATTACTGTATCCAAAAATTCCAACTTCTGA

TTTTCTTCAGCTATCAGTCGTAAAATTTGATAAGCAACAACAGACCTTTTTAATCCTTCC

ATAGATACCAAACGAACATCAGATAGACTTTGTTGATGAAGTTCGTAATAGCTCATTAAG

TCGATGAACGATTCACAAAAGACCAGTCTATTAGGTTTACCAATATCAAAGGATATTCCA

ACATGTCCATGGCTTCCTTTTAGAATCGTTTTTAACCTCTCTCTAGGAAGAGAGTGATTC

TTATAAATTCCTTGTAAGCTTGCTGCCTGCAGCTTGTGGCGATGATCAAAGCTTTTAAAA

ACGTTAACAGGTTCAACTGTTTCATTTGTTTTCCAACTAGCTTGTGCTATCAAACCTTGT

TGAATCATCTTTTGTATGATTTCTTCTGAGATTCCTCTACATTCTGTTAAGTAATATCTA

GTCAAACTGCAGTTAGAATCTTCTACTCTCTTTAAAGGATAATAAAATGGTCTCTCTCTT

TTTCTTGAATAGCTTCTTTTTGAAAAGGTTCTTCAGAAAGAAAGGCTAGAGCTTCTTTAA

AGGAAATTCCCTTAACAAGTCGAACAAAATCAATGACATCACCTTGAATATCTCTTGAAA

ACCATTTAAAAGTATTGGTAGTTGAAAAAATCCGAAATGAATCGTGTTCAGGATGTTCAT

AGACACTGCTCGAAACTTGTTTAAAGGAGATACCTAAACGATTGGCTACATCAAGAATTG

AAATTTGCTTACATTCTTCTATTCCCATGCAATATCATCATTTTGTCGTAGTATTTGGCA

GTGATGGTATTGATGATGATTCTTCTAAAGTTTTAGGAGTTGCATTATCTAAATCATCTA

ACCCAGATTTCCAAACCTGTACTTGATTGACCAATAACTTATCTGGTAGTTTCGAATAAG

ACAACTTAACCGTTCTGGTTTCTGTCTGATTGGTCTTTGATTGGTTGGCATTCTTTAAAT

CGGATACATAGGTTACATTATAAGACACCATAGCAATGGCTTGATTCGTAGTCTGATTGA

CAAAGATATCAGCTTTTTCAAAATGATAATCCAAAATATAATCCTTATACACTTGGTTCA

TGGCATCATTTTGACTTGACAATTCTTGAGAATAAGCTGATTCAGTCATATAAGGTTGAA

TACGTGTATTATTTTCTCCGAGCTTTTCTTTCGTATAGTACTGTGTCAAAAATTCTTTTA

CAGTATCTGATGACAAAATACTTGCTTTATCTTCTGCTTGTTTGTCCTCTACAAGTTTAG

CTGCAGCCAATTCAATCTCTTTACGACTTTGTTTAGCAGTAGAATGTTGACCAGCAGTAT

ATCCCATCATGAGAATAAAGCTAGTTGCAGCCACTGCTCCAACACTAATTAAGGCTTTAG

TTTTGACTTTATTTAACATCTTAGACCTTCTTTCTATAAAAACTAGGTCAAGAATAACAA

AAAGAACTTGTAAGTTTTATCACATCACAACGAAAGGTAATTCCTACATTTCAGGCGCAA

AATCGTACAGTTCAGAGAAAAAAATAAAACTAGCTGTCTTTGAAGTATAATAGACTTATT

GGAAAAAAATAAAATGCATACTGTACTATGCTATACTATAGTTAAGAAAACTATTTACAA

AAGATCATGCAACTAACTTCTGAGAACTTTAAATTATTTATTAGTGTAAAAAAGGACACC

TTCCCAACGAAGATATCCTGTCTAAATAAAGCTTATAAAACCAAACACTATAAAGGAGGA

CGTCCAAAAACGCTAAGTCTATAAAAACAGTTGATGCTAAACTTGCGTTATTTATTCTAT

TGACCAACTCTACGTCTCCTAGCTTTTGATTTTGGTCTTGGATTAGCGACAGTCACCGAA

CGATAATATTGGTAGAAGATACTCTTCGTTCTTCAGGTCTATTTGCCTTAGACAACTTAA

AAGCAGCAAGTGTCACTATAGCTATTGATGTCATAGAAAGTCCTATTTCAACGTCCTAAA

AGAAACCAAAGTAAAATTATTCAGGAAAAAAGAGACACACCATGAAGACACAAATCATAC

TCGATTTAACACCACTTCATGCCTGCTAATTAGTTTTTTAAGAAGGTCATACACATGATT

TTACTCTTTTTAAGAAAGGATAGGTCAAGTTTGCCAGAGCAATCACTTGATTTTCTGTAT

TTGGATTATTTATGCATTATGAATATTCATAATATAACCTTCATTCCTGTTAAAAATTCT

AAAAAGCATCTATTGACTGAAGAGGATAAGAAGTTTAATAGAGATACTTGTAATACGTAT

TAAAATTGAGCATTTTAATGCTAAGTATAAAACTTTTCAATTTATGGAATCATCAACTAT

GAGTTAAAATAATTTTCGAACAAAGGCTGTCCAAAAAACTTGATATAATGCGTTTTATTA

TGGGAATATTCATTTCATTTACTCCTGAAATTGAGTTTTTACCCAGACTCATTAATGTTA

TTGATATAATCCAATAATAATGATAATATCAAATAGTAATAAAAAATTTGATAGTATTTT

TTCATAATCTGTCACGCTTTCTAATGATTTTTTATGGTAAATGGAGATAAATATGATTAT

AGGTGAAACTTATAGAAAAATACGAGAAGGAAAAGGTATTTCTATTTCTTCATTAGCAGG

TGCAGAAATTTCAAAGTCTCAAATATCTAGATTTGAATTAGGAGAGACAGAAATCTCATT

TTTTAAATTATTATATCTACTTGAAAAAATAGGTGTAACACTAGAGGAGTTTTTGCTTTC

ATGTAATAATTATCAGCCTTCAGACTTCAATACCTTGATACGCTTAGTTCAACAGGCTGC

ATACAATCAAGAAATCAAATCATTACTTAATATGGTAAGCAAAGAGATGGAACTGTTTAG

AGAAACCAAATCTCATTATCATAAGTTAAACGCTATTTTCATTGAAAGTATTATCTATGG

AATTGATAATACTCATCAATTGAGTAATCAAGACACCTCTTATCTTACTAATTATTTATT

TTCTGTTGAAAACTGGGGATATTACGAAATTCTTATTCTTGGAAATTGTTGTCGAGCAAT

ATTACCAAATTTATTATTTAGATATGCCAAAGAGGCGCTTAAAAAGGAAAGTTGTATAGT

TCTATACCTAGAAATAAACAAGCTCTCGTTCAATTACTTCTTAATTCACTTCTTATTATG

ATTGAAAACAGCTTATATGAGGAAGCTTTATTTTTAGAACAAGCTACTAAGAATATATTA

TCTAATTCCACAGATTTTTTTGAACAAACTATTCTTTTGTATTTAGAAGGATTTTTTGAA

CTTAAATTTCATCATAATCAAAAATCAATTTTAAAAATAGAAGATGCTTTAAAAATTTTT

GAATTATTCAATAAAACATTATACAAAAATTATAAAGATTACTACAAAAAAATATTATCA

TTTTATTACAATGTGGTAATTCCTATTTTGAATAATTGCTTTTTTTGAAAAACTTTTTAA

TTCATCTAAAGAAATCAATTCCAGCTCTTCAGCAATACTATCCCAACTTGGAATATCAAA

TAATTTTTCTCTACAAATTGTTTTTAAATATTCATTTTTATTAACTTCACAATTTATGTA

CAATTCATTGATTACTCTTTTTTTAGCCTTATAAAATAATAGTTCATCAGATAAATCGGA

TTCATTAACAAGATTGTTCTCCAGTGTTTTAATCAATTTTTTCTTATAGATTGTAGGTGT

TATAGTTACTATTTTTAGTAAAGTCATATTACTATAAAATATTGGGTATGATACAATACT

ATATATCCATTGCTTTTGTGTCCTTAACTTATGGCCTAATAATGAATCTTTAATACCAGT

TAACATCGAGTTTAAGATACTTAACATTGCCATTTCATGAGATGATGCTACCATACCAGA

TATATCTAGATACATAGAAATTTCAGAAAGTTCTTGTTGTTTATCTAATTCCGTTAAACC

AGTTGAAAAAGCCGGAGAGTTAATTTCTACAGATGATCCACTTAAAGGTAACATATTTTC

AATACTTATGATTTCACTTTGTAGAAAACTTGGACCAGCAATAGCAATCTGGAATTCTCC

TGCTTGAATATTTTTATATGAAAAATAGCATAAAAATCTAGTTATCCGCATAAAAACTGG

ACTTATCACACTTTATCAAGGTCAAAACCACTCAATTTACTACTAATTTACTACTTATGA

ATGAGCTTTGATACGACGATTTATCCTTGAAAAGTGAAGATATAAAGATACTTCCAATAA

AATTTGAATATTTAATAGGTAGACACTTCAAAAAATGAGGTGTCTATTTTTTTACCCGAT

TTTGAAAGGAAGTGAACTTATGAAAACAAAAAATCAAGAATCAAAAGGTCGTTCCCCACT

CTTTAAGACCATCAAACATTCATTCAGCCAATAAAAAGAAAGGATAGGTAAAAATATGGA

ACTTAAATTTGTGATTCCCAACATGGAAAAAACATTCGGCAATTTAGAATTTGCTGGCGA

GGATAAAGTCGTTCAGCGAAGAATCAACGGACGGCTAACTGTCTTATCAAGAAGCTATAA

TCTCTATTCTGATGTTCAAAGAGCAGATGATATTGTGGTGGTGCTTCCTGCTGAAGCTGG

CGAAAAACATTTCGGCTTTGAGGAACGTGTGAAGTTAGTCAATCCACGTATTACCGCAGA

GGGCTACAAAATCGGCACTCGTGGTTTTACAAATTACCTTTTACATGCTGACGACATGAT

AAAAGAATAAAGAAAGAGAGGAAAAATGATGAGATTAGCAAATGGCATTGTATTAGATAA

AGACACGACTTTTGGAGAATTGAAATTCTCTGCTCTACGTCGTGAAGTGAGAATCCAAAA

TGAAGACGGGTCGGTTTCAGATGAAATCAAGGAACGTACCTATGACTTAAAATCCAAAGG

ACAAGGACGCATGATTCAAGTAAGTATTCCTGCCAGCGTGCCTTTGAAAGAGTTTGATTA

TAACGCACGGGTGGAACTTATCAATCCCATTGCGGACACCGTTGCTACTGCCACCTATCA

AGGAGCAGATGTTGACTGGTATATCAAGGCAGACGATATTGTGCTGACAAAGGATTCTAG

TTCATTCAAAGCTCAACCACAAGCAAAGAAAGAACCGACACAAGACAAATAGTCGCTAGG

TAGAAAGGAGACTTTTTCGCATGAAACAGCGTGGTAAAAGGATTCGCCCATCTGGTAAAG

ATTTAGTCTTTCATTTTACGATAGCGTCACTCCTGCCTGTTTTCCTGCTGGTTGTCGGAC

TGTTTCATGTGAAGACAATCCAGCAGATCAACTGGCAGGATTTTAACCTATCACAAGCAG

ATAAGATTGACATTCCCTATTTAATTATCAGTTTCAGTGTCGCAATTCTTATCTGCTTGC

TGGTAGCGTTTGTATTCAAACGGGTTCGCTATGATACGGTTAAACAACTTTACCACCGTC

AAAAACTGGCAAAGATGATACTTGAAAACAAGTGGTATGAATCTGAACAGGTCAAAACAG

AGGGTTTCTTTAAAGATAGTGCTGGTCGTACAAAGGAAAAGATAACCTACTTCCCTAAAA

TGTATTATCGACTTAAAAATGGCTTGATACAGATACGGGTGGAAATCACGCTGGGAAAAT

ATCAAGACCAACTCTTACACTTGGAAAAGAAATTAGAGAGTGGCTTGTACTGTGAGCTGA

CGGATAAAGAGTTAAAGGATTCCTATGTGGAATATACTTTGCTCTATGACACCATAGCCA

GTCGTATTTCTATTGATGAAGTAGAAGCTAAAGATGGTAAACTTCGCTTAATGAAAAACG

TATGGTGGGAATATGATAAGCTCCCTCATATGTTGATTGCTGGTGGTACAGGTGGCGGTA

AAACTTACTTTATACTGACACTGATTGAAGCCTTGCTTCATACAGATTCAAAACTGTATA

TTCTTGACCCGAAAAATGCTGATCTTGCGGACTTAGGTTCTGTGATGGCAAATGTCTACT

ATAGAAAAGAAGACTTGCTTTCTTGCATTGAAACATTCTATGAAGAAATGATGAAACGTA

GTGAGGAAATGAAGCAGATGAAGAACTATAAGACTGGCAAAAATTATGCTTACTTAGGTC

TCCCGGCACACTTCTTAATCTTTGATGAATACGTCGCTTTCATGGAAATGCTGGGAACAA

AAGAAAACACCGCAGTTATGAATAAGCTGAAACAGATTGTCATGTTAGGTCGTCAAGCTG

GCTTCTTTCTAATACTGGCTTGTCAACGTCCAGACGCAAAATATTTAGGCGACGGAATCC

GTGATCAGTTTAATTTCAGAGTGGCTTTAGGTCGTATGTCTGAAATGGGCTATGGCATGA

TGTTTGGCAGTGACGTACAAAAGGATTTCTTCTTAAAGCGAATCAAAGGTCGTGGCTATG

TTGATGTAGGAACAAGTGTCATATCAGAGTTTTATACTCCCCTTGTACCAAAAGGATATG

ATTTCTTGGAGGAAATTAAAAAGTTATCCAACAGCAGACAGTCCACGCAGGCGACGTGCG

AAGCGGAAGTCGCAGGTGTGGACTGATCTTGCTGGCTGGTGTGGCAATAGCCACGCCAGC

ACTTAACCCCCCGTATCTAACAGGGGGGTACAAATCGACAGGAAACAGTCAAAAAAACAT

TAGAAAATCCTTTGGTTACAAGGGATTTACAAAATTTCAGCGTATGTCAAATGGGCTTTA

AAAGTTGACATACGCCTTTTTGATTGGAGGGATTTTTACTGAATGAACAAACTTGGTTAC

AGCATTTAAAAGAAAAACGCTTGGCTTATGGACTATCTCAAAACCGTTTAGCTGTTGCGA

CTGGTATTACAAGGCAGTATCTAAGCGATATTGAAACAGGAAAAGTCAAGCCATCAGAGG

ATTTACAGCAGTCCCTTTGGGAAGCTCTGGAACGCTTCAATCCCGACGCTCCCCTTGAAA

TGCTGTTTGATTATGTAAGAATTCGCTTTCCGACAACAGACGTACAGCAGGTGGTCGAAA

ACATCTTACAACTGAAACTGTCCTATTTTCTTCATGAGGACTATGGTTTCTATTCTTATT

CAGAGCATTATGCTTTAGGCGACATATTCGTCCTTTGCTCCCATGAACTGGACAAAGGAG

TTCTGGTGGAATTGAAAGGTCGTGGGTGCAGACAATTTGAAAGCTATCTTCTGGCACAAC

AAAGAAGCTGGTATGAGTTCTTTATGGACGTTTTGGTGGCTGGCGGTGTGATGAAACGCC

TTGACCTTGCCATTAACGATAAGACAGGGATTTTAAATATCCCTGTACTCACTGAAAAGT

GCCAACAGGAAGAATGTATCTCCGTCTTCCGCAGTTTTAAAAGCTATCGCAGTGGCGAAC

TGGTACGCAAAGAGGAAAAGGAATGTATGGGAAACACCCTCTATATCGGTTCATTACAAA

GTGAAGTTTATTTCTGTATCTATGAAAAGGACTACGAGCAGTACAAGAAAAATGATATTC

CCATTGAAGACGCAGAAGTAAAAAACCGTTTTGAGATTCGATTGAAAAATGAGCGTGCCT

ATTATGCAGTCCGTGATTTACTCGTCTATGACAATCCAGAGCATACCGCCTTTAAAATTA

TCAATCGGTATATCCGTTTTGTAGATAAAGACGATTCCAAACCTCGTTCTGATTGGAAAC

TGAATGAAGAATGGGCTTGGTTTATTGGGAACAATCGTGAACGATTAAAACTAACCACAA

AACCAGAGCCTTACTCCTTCCAAAGGACGCTGAACTGGCTATCTCATCAAGTTGCCCCGA

CCTTAAAGGTTGCGATTAAACTTGATGAAATCAACCAGACGCAGGTTGTAAAAGACATTC

TCGACCATGCGAAACTGACAGACCGACACAAGCAGATTTTGAAGCAACAGTCAGTAAAAG

AACAGGACGTGATAACAACAAAAAAATAACTCAAATACAAATTCATTGAATATAGAGAGG

AGAACATTTTTATGAATTTTGGACAAAACCTTTATAACTGGTTTCTATCAAACGCTCAAT

CACTGGTGCTTTTAGCAATCGTTGTGATTGGCTTGTATCTTGGCTTCAAGCGTGAGTTTA

GCAAACTGATTGGCTTTTTAATTATTGCGATTATTGCGGTTGGCTTAGTCTTCAACGCTG

CTGGAGTAAAAGACATTTTACTAGAGCTATTCAATCGCATTATTGGTGCTTAAATAAAAC

CGTTCTTTTGTGGAATATAAGTGGTTTTCTTATGTTCCGCAAAGGAATGGTACACCAAAC

GAAGTGCGGTAGGGATTTTTGAATCTCTACAAAGAAAGGACGTGAATATATGGACGATAT

GCAAGTCTATATTGCGAATTTAGGCAAATACAATGAGGGCGAATTGGTCGGTGCGTGGTT

TACCTTTCCCATTGACTTTGAGGAAGTCAAAGAGAAAATCGGCTTGAATGATGAATATGA

GGAATACGCCATTCATGACTACGAGTTACCCTTTACGGTTGACGAATACACTTCCATTGG

CGAACTCAATCGACTATGGGAAATGGTATCGGAATTACCCGAAGAATTACAATCGGAGCT

ATCTGCTCTGCTCACTCATTTTTCAAGCATTGAAGAACTAAGCGAACATCAAGAGGATAT

TATCATTCATTCCGATTGTGATGATATGTATGACGTGGCACGCTACTACATTGAAGAAAC

GGGTGCTTTAGGCGAAGTACCAGCTAGTCTTCAAAACTATATTGATTATCAAGCCTATGG

TCGGGATTTAGACCTTTCAGGAACGTTTATCTCAACCAATCATGGGATTTTTGAAATCGT

CTATTAAATCTGTCGGTACATTACTACTGGCAGATTTTCTATTTTACGGGGTGGCTCAAT

CAGCTACCCCTATTTTTTATGAAAGGATTGATTACATGAAGAAAATACGAAGCTATACCA

GTATCTGGTCTGTGGAAAAGGTACTGTATTCTATCAATGATTTTAGACTTCCGTTTCCCA

TAACCTTTACGCAAATGACATGGTTTGTCGTGTCACTCTTTGCAGTGATGATACTTGGCA

ACTTGCCCCCTCTTTCCATGATAGAGGGAGCATTTCTCAAATACTTTGGGATTCCTGTGG

CTTTCACATGGTTTATGTCTACAAAAACTTTTGATGGTAAAAAGCCTTATGGATTTTTGA

AGTCTGTCATTGCTTATGCACTGCGACCAAAGCTGACCTATGCAGGAAAAAAAGTAACGC

TTGGCAGAAACCAGCCACAAGAAGCCATTACAGCAGTTAGGAGTGAATTTTATGGCATAT

CCAATTAAATACATTGAAAACAATCTCGTCTGGAATAAAGACGGGGAATGTTATGCTTAC

TATGAGCTTGTTCCTTACAATTACTCATTTCTAAGTCCAGAACAGAAAATACAAGTGCAT

GATTCTTTCAGACAGCTTATCGCACAAAATCGTGATGGCAAAATTCATGCTTTACAAATC

AGTACAGAATCCAGCATACGTTCTGCACAAGAGCGTTCCAAAAATGAAGTCACTGGCAAG

CTCAAAGCGGTTGCCTATGACAAAATCGACCAACAGACAGACGCTTTAATATCCATGATT

GGCGAAAATCAAGTGAACTACCGTTTCTTTATCGGCTTTAAGTTGCTTCTCAACGATCAG

GAGTTTTCTATGAAAAGTCTTACCGTTGAAGCAAAAAATGCTTTGTCTGATTTTGTCTAT

GATGTGAACCATAAGCTGATGGGCGATTTTGTTAGTATGAGTAATGATGAAATCCTGCGT

TTTCAGAAGATGGAAAAGCTCTTAGAAAATAAAATCTCTCGTCGTTTCAAAATCCGCAGG

TTAGATAAGGACGACTTCGGCTATCTGATTGAACACCTTTACGGACAGACAGGCACTGCC

TATGAAGAGTATGAGTACCATCTATCAAAGAAAAAGCTGGATAATGAAACGCTGATTAAA

TACTATGACTTGATTAAGCCTACTCGCTGTTTGGTGGAAGAAAAACAGCGATATTTGAAA

ATCCAGCAGGAAGATGAAACCGTCTATGTAGCTTACTTTACCATTAACAGCATTGTCGGA

GAACTGGACTTCCCGTCCTCTGAAATCTTCTACTACCAGCAACAGCAATTTACATTCCCG

ATTGATACGTCAATGAATGTGGAAATTGTAGCGAATCGTAAAGCCCTATCTACTGTCCGC

AATAAAAAGAAAGAACTGAAAGACTTGGATAACCACGCTTGGCAAAGTGATAATGAAACC

AGCTCCAATGTGGCGGAAGCTCTGGAAAGTGTGAATGAGCTGGAAACCAATTTAGACCAA

AGCAAGGAATCTATGTACAAGCTGTCTTATGTGGTAAGGGTATCAGCAAATGATCTTGAC

GAACTCAAACGTCGTTGTAATGAAGTGAAAGATTTTTATGACGATTTAAGCGTAAAACTG

GTACGACCATTTGGGGATATGCTCGGCTTACATGAAGAATTTTTACCTGCCAGCAAGCGT

TATATGAATGATTATATTCAATACGTGACCTCTGATTTCCTCGCTGGTTTAGGTTTTGGT

GCTACTCAAATGCTGGGGGAAAATGAGGGGATTTATGTTGGCTACAGCTTAGATACTGGA

CGCAATGTCTATCTGAAACCTGCTCTTGCCAGTCAAGGGGTTAAGGGTTCAGTAACCAAT

GCGTTAGCGTCGGCTTTTGTTGGTTCGCTGGGTGGTGGTAAATCCTTTGCGAATAACCTT

ATCGTCTATTATGCGGTGCTTTATGGGGCACAAGCAGTGATTGTAGACCCAAAAGCAGAA

CGTGGCAGATGGAAAGAAACCTTGCCAGAGATTTCCCATGAAATCAATATCGTCACTCTG

ACTTCTGATGAGAAAAACAAAGGCTTACTTGACCCTTATGTGATTATGAAAAATCCCAAA

GATTCTGAATCACTGGCTATTGATATTCTGACATTCCTTACGGGGATTTCCTCTCGTGAT

GGGGAACGCTTCCCAATCCTTAGAAAAGCCATTCGTGCAGTAACCAATAGTGAAGTACGA

GGGTTGATGAAAGTGATTGAGGAATTACGGGTTGAGAATACGCCACTAAGTACCAGTATA

GCCGACCATATCGAAAGTTTTACAGACTATGACTTTGCACATTTATTATTCAGTAATGGT

TATGTGGAGCAGTCTATCAGCTTAGAAAAACAACTGAACATTATACAGGTTGCGGACTTG

GTACTTCCCGACAAGGAAACTTCCTTTGAGGAATATACCACTATGGAGCTTTTATCCGTT

GCTATGCTGATTGTCATTAGTACCTTTGCTTTAGACTTTATCCATACAGACCGAAGCATT

TTCAAGATTGTAGATTTAGACGAAGCATGGAGCTTTTTACAGGTAGCACAAGGAAAAACA

CTATCTATGAAGCTGGTTCGGGCTGGTCGTGCTATGAACGCTGGGGTATATTTCGTGACC

CAAAATACAGACGACCTCTTAGATGAAAAACTGAAAAATAACCTCGGCTTAAAATTTGCA

TTTCGTTCCACTGACCTTAACGAGATTAAAAAGACCTTAGCCTTTTTTGGTGTAGACCCA

GAGGACGAAAACAATCAGAAGCGATTGCGTGATTTGGAAAACGGGCAATGCCTTATCAGT

GATTTATATGGTCGTGTCGGTGTGATACAGTTCCACCCTGTATTTGAAGAACTGCTCCAT

GCCTTTGATACCAGACCACCTGTGCGAAAAGAGGTGTAAATGTGAAACCATCAATAGTAA

ACAGAATAAAATCAAACTGGACGCTGAAACGTCTAGGTAAAGTGGCAATGACAGTGGCTT

TCACACTTGTGATTGCCATTTTTCTTTTAGCCATGCTGGGAACGGTGGTTCAAGCTGCGG

GCTTGGTAGATGATACGGTCAATGTGGCAAATGAATACAGCCGATACCCACTTGAAAACT

ATCAACTGGATTTTTATGTGGATAATAGCTGGGGCTGGCTTCCGTGGAACTGGTCGGACG

GGATTGGAAAACAGGTCATGTATGGACTATATGCCATTACCAATTTTATTTGGACAATCA

GTTTGTATGTTTCCAATGCGACAGGTTACTTAGTACAGGAAGCCTATTCCTTAGACTTCA

TTTCCGCTACAGCAGATTCCATTGGTAAGAATATGCAGACCTTAGCTGGTGTGAGTGCAA

ACGGATTTTCAACAGAGGGTTTCTATGTTGGATTCCTCTTACTCTTGATTTTGGTTCTTG

GGGTTTATGTTGCCTATACGGGACTGATAAAGAGAGAAACCACAAAGGCAATTCATGCCA

TTATGAATTTTGTGCTGGTGTTTATCCTATCGGCTTCCTTTATTGCCTACGCTCCCGACT

ACATTAAAAAATCAATGACTTTTCATCAGACATCAGTAATGCCAGTTTATCACTTGGCAC

GAAGATTGTCATGCCCCATTCCGATAGTCAAGGCAAGGACAGCGTGGACTTAATCAGAGA

TAGCCTGTTTTCCATACAGGTTCAGCAACCGTGGCTACTGCTTCAATACAACAGTTCAGA

CATTGAAAGTATCGGTATTGACCGTGTGGAAAGCCTGCTCTCCACCAGCCCAGATTCCAA

CAATGGCGAAGACAGAGAAAAAATTGTTGCGGAAGAAATTGAAGACAGAAGCAATACCAA

TCTAACCATTACAAAGACCATTAACCGTTTAGGTACAGTCTTCTTCCTATTTGTCTTCAA

TATTGGGATTTCCATATTTGTATTCCTATTAACAGGAATCATGATTTTCTCGCAGGTACT

TTTTATCATCTATGCTATGTTTCTGCCTGTGAGCTTTATTTTAAGCATGATTCCATCATT

TGATGGTATGTCAAAACGAGCCATAACAAAGCTCTTTAATACCATTTTGACACGAGCTGG

AATCACATTGATTATTACGACAGCATTTAGTATTTCAACCATGCTCTATACCTTATCGGC

TGGTTATCCGTTCTTTTTGATTGCTTTTCTACAGATTGTGACCTTTGCAGGAATCTACTT

CAAGCTGGGCGATTTAATGAGTATGTTTTCTCTACAGAGTAACGATTCTCAAAGTGTGGG

AAGTCGTGTGATGAGAAAACCTCGTATGCTTATGCACGCTCACATGCACCGTCTACAGCG

GAAACTTGGACGTTCCATGACTACTCTAGGGGCTGGGTCTGCCATTGTTACAGGTAAAAA

AGGACAGTCGGGTTCGGGGAGTTCTGCAAGGACACAAGCAGATCACTCCCGACCAGACGG

AAAGGAAAAATCAACACTTGGAAAACGTATCGGTCAAACCATCGGTACAGTAGCTGATAC

CAAAGACAGAATGGTAGACACTGCTAGTGGTTTGAAAGAACAGGTTAAAGATTTGCCGAC

CAATGCAAGATATGCAGTATATCAAGGAAAATCCAAAGTAAAAGAGAATGTCCGTGATTT

AACCAGTAGTATTTCTCAAACCAAAGCGGACAGAGCCAGTGGACGCAAGGAACAGCAGGA

ACAAAGGCGAAAAACCATTGCGAAGCGTCGCTCTGAAATGGAACAGGTCAAACAGAAAAA

ACAGCCTGCTTCTTCTGTTCATGAAAGACCGACTACAAGACAAGAACAATATCATGATGA

ACAGACCTCAAAACAGTCTAATATTCAGACTTCATATAAGGAATCTCAACAAGCCAAACA

AGAGCGTCCAGCAGTTAAGTCCGATTTTTCAAGTCCAAAAGTGGAACGCCAAGGCAATAC

CGTTCAAGAAAAAACCGTTCAAAAGCCAGCAACTTCAACCACTACAGCAGATAGAACTTC

ACAACGTCCAATCACAAAAGAACGTCCGTCTACTGTTCAAAGAGTACCACTACAAAATAC

AAGAAGTAGACCACCAATCAAAACCGCCACCATTAAGAAAGTCGGTAAGAAACCATGAAG

TTGAAAACTTTAGTGATTGGTGGTTCTGGATTATTCTTGATGGTCTTCTCACTGCTTCTG

TTTGTTGCCATTTTATTTTCAGATGAACAGGACAGCGGAATTTCCAATATTCATTATGGA

GGTGTGAATGTTTCCGCAGAAGTGCTGGCTCATAAGCCTATGGTAGAAAAATATGCCAAA

GAATATGGCGTTGAAGAATATGTCAACATACTTCTTGCGATTATACAGGTGGAATCGGGC

GGTACTGCGGAAGATGTTATGCAGTCCTCGGAATCCCTCGGTCTTCCACCTAATTCATTG

AGTACAGAAGAATCCATTAAGCAAGGTGTGAAGTATTTCAGTGAATTATTAGCCAGTAGC

GAAAGGCTCAGTGTAGATTTAGAATCGGTTATCCAGTCCTACAATTATGGTGGTGGTTTC

TTAGGGTATGTGGCTAATCGTGGAAATAAATATACCTTTGAACTGGCTCAAAGTTTCTCA

AAAGAGTATTCAGGTGGCGAAAAAGTGTCTTACCCCAATCCCATAGCCATACCTATCAAT

GGGGGCTGGCGATACAACTATGGCAATATGTTTTATGTGCAACTGGTAACGCAGTATCTT

GTCACAACAGAGTTTGATGATGATACGGTACAAGCCATCATGGACGAAGCACTGAAATAT

GAGGGCTGGCGATACGTTTACGGTGGAGCTTCCCCGACTACTTCTTTTGATTGTAGCGGA

CTGACACAATGGACGTATGGAAAAGCTGGAATTAACTTACCACGAACCGCACAACAGCAA

TATGATGTGACCCAGCATATCCCACTATCGGAAGCACAAGCTGGCGATTTGGTTTTCTTT

CATTCTACCTATAACGCTGGCTCTTATATTACTCATGTTGGGATATACCTTGGCAATAAC

CGTATGTTTCATGCAGGCGACCCAATCGGTTATGCCGACTTAACAAGCCCCTACTGGCAA

CAGCATTTAGTGGGAGCAGGACGAATCAAACAATGAGAAAGGAAGATTTAATGATGAAAT

TTAGAAAAAATCAGAATAAAGAAAAACAGATACCAAAGGAAAAGAAACCTCGTGTCTATA

AGGTCAATCCTCATAAAAAGGTTGTGATTGCCTTGTGGGTACTTTTAGGGCTTAGTTTCA

GCTTTGCGATATTCAAGCACTTTACAGCTATAGATACTCATACTATTCACGAAACAACTA

TCATAGAAAAGGAATACGTTGATACTCATCATGTAGAAAATTTTGTAGAGAACTTTGCGA

AAGTCTACTATTCATGGGAGCAATCCGATAAGTCCATTGATAATCGAATGGAAAGTCTAA

AAGGCTATCTGACAGATGAACTTCAAGCTCTCAATGTTGATACAGTACGCAAAGATATTC

CTGTATCGTCTTCTGTAAGAGGATTTCAGATATGGACGGTAGAGCCAACTGGCGACAATG

AGTTTAATGTAACCTACAGTGTAGACCAGCTCATTACAGAGGGAGAAAATACAAAGACCG

TCCACTCTGCTTATATAGTGAGTGTCTATGTAGATGGTTCTGGAAATATGGTACTGGTTA

AGAATCCGACCATTACCAACATACCTAAGAAATCAAGTTATAAACCAAAAGCCATTGAAA

GTGAGGGGACGGTTGATTCCATTACAACCAATGAAATCAATGAGTTTTTAACGACGTTCT

TCAAGCTCTATCCTACAGCGACAGCCAGTGAACTTTCCTACTATGTGAATGACGGGATAT

TAAAACCAATCGGAAAAGAGTACATCTTTCAAGAACTGGTAAATCCTATTCACAATCGTA

AGGATAATCAAGTCACGGTATCGCTGACAGTGGAGTATATCGACCAGCAGACCAAAGCAA

CGCAGGTATCTCAATTTGATTTGGTACTTGAAAAGAACGGGAGTAATTGGAAGATTGTAA

AATAACAAATATTGGTACATGATTACAGATACTTTGTAATCATGTACTCTTTTTGATAAA

AAATTGGAGATTCCTTTACAAATATGCTCTTATGTGCTATTATTTAAGTGACTATTTAAA

AGGAGTTAATAAATATGCGGCAAGGTATTCTTAAATAAACTGTCAATTTGATAGCGGAAA

CAAATAATTAGATGTCCTTTTTTAGGAGGGCTTAGTTTTTTGTACCCAGTTTAAGAATAC

CTTTATCATGTGATTCTAAAGTATCCAGAGAATATCTGTATGCTTTGTATACCTATGGTT

ATGCATAAAAATCCCAGTGATAAAAGTATTTATCACTGGGATTTTTATGCCCTTTTGGGT

TTTTGAATGGAGGAAAATCACATGAAAATTATTAATATTGGAGTTTTAGCTCATGTTGAT

GCAGGAAAAACTACCTTAACAGAAAGCTTATTATATAACAGTGGAGCGATTACAGAATTA

GGAAGCGTGGACAAAGGTACAACGAGGACGGATAATACGCTTTTAGAACGTCAGAGAGGA

ATTACAATTCAGACAGGAATAACCTCTTTTCAGTGGGAAAATACGAAGGTGAACATCATA

GACACGCCAGGACATATGGATTTCTTAGCAGAAGTATATCGTTCATTATCAGTTTTAGAT

GGGGCAATTCTACTGATTTCTGCAAAAGATGGCGTACAAGCACAAACTCGTATATTATTT

CATGCACTTAGGAAAATGGGGATTCCCACAATCTTTTTTATCAATAAGATTGACCAAAAT

GGAATTGATTTATCAACGGTTTATCAGGATATTAAAGAGAAACTTTCTGCCGAAATTGTA

ATCAAACAGAAGGTAGAACTGTATCCTAATATGTGTGTGACGAACTTTACCGAATCTGAA

CAATGGGATACGGTAATAGAGGGAAACGATGACCTTTTAGAGAAATATATGTCCGGTAAA

TCATTAGAAGCATTGGAACTCGAACAAGAGGAAAGCATAAGATTTCATAATTGTTCCCTG

TTCCCTGTTTATCACGGAAGTGCAAAAAACAATATAGGGATTGATAACCTTATAGAAGTG

ATTACGAATAAATTTTATTCATCAACACATCGAGGTCCGTCTGAACTTTGCGGAAATGTT

TTCAAAATTGAATATACAAAAAAAGACAACGTCTTGCATATATACGCCTTTATAGTGGAG

TACTACATTTACGAGATTCGGTTAGAGTATCAGAAAAAGAAAAATAAAAGTTACAGAAAT

GTATACTTCAATAAATGGTGAATTATGTAAGATTGATAGAGCTTATTCTGGAGAAATTGT

TATTTTGCAAAATGAGTTTTTGAAGTTAAATAGTGTTCTTGGAGATACAAAACTATTGCC

ACAGAGAAAAAAGATTGAAAATCCGCACCCTCTACTACAAACAACTGTTGAACCGAGTAA

ACCTGAACAGAGAGAAATGTTGCTTGATGCCCTTTTGGAAATCTCAGATAGTGATCCGCT

TCTACGATATTACGTGGATTCTACGACACATGAAATTATACTTTCTTTCTTAGGGAAAGT

ACAAATGGAAGTGATTAGTGCACTGTTGCAAGAAAAGTATCATGTGGAGATAGAACTAAA

AGAGCCTACAGTCATTTATATGGAGAGACCGTTAAAAAATGCAGAATATACCATTCACAT

CGAAGTGCCGCCAAATCCTTTCTGGGCTTCCATTGGTTTATCTGTATCACCGCTTCCGTT

GGGAAGTGGAATGCAGTATGAGAGCTCGGTTTCTCTTGGATACTTAAATCAATCATTTCA

AAATGCAGTTATGGAAGGGATACGCTATGGTTGCGAACAAGGATTATATGGTTGGAATGT

GACGGATTGTAAAATCTGTTTTAAGTATGGCTTATACTATAGCCCTGTTAGTACCCCAGC

AGATTTTCGGATGCTTGCTCCTATTGTATTGGAACAAGTCTTAAAAAAAGCTGGAACAGA

ATTGTTAGAGCCATATCTTAGTTTTAAAATTTATGCGCCACAGGAATATCTTTCACGAGC

ATACACCGATGCTCCTAAATATTGTGCGAACATCGTAGACACTCAATTGAAAAATAATGA

GGTCATTCTTAGTGGAGAAATCCCTGCTCGGTGTATTCAAGAATATCGTAGTGATTTAAC

TTTCTTTACAAATGGACGTAGTGTTTGTTTAACAGAGTTAAAAGGGTACCATGTTACTAC

CGGTGAACCTGTTTGCCAGCCCCGTCGTCCAAATAGTCGGATAGATAAAGTACGATATAT

GTTCAATAAAATAACTTAGTGTATTTTATGTTGTTATATAAATATGGTTTCTTGTTAAAT

AAGATGAAATATTTTTTAATAAAGATTTGAATTAAAGTGTAAAGGAGGAGATAGTTATTA

TAAACTACAAGTGGATATTGTGTGCTGAGAGCTTTCTTCTATACTAATAGACGAAAGGGT

GTGAAAATGATTTTTAAATGATACTGTGGAACGGAACAGTAGCCCTAGTATTGACTACTG

TCGTTTCTATTCATATTGGCTATTCTAGGACTGAGATGAAAAAATCTATAAATGCTCAGA

ATAAAATTGAACCCGCAAATCTCCCCAAAACAATGGTGAGTCATGTACTTGTATTATTCC

GAAAAAATACACCTCTGGTGCAGTGAGACAAATTGGTGTATCTTATAGTGGCTTCGTAGA

TGAAAGCTATACTCTACTATCACTCTTTGATGATGTAGAACAAATTGAAAAAGATAATAG

ACTTCAGACAGCTATTGATGTTGTCAGAGAACAGTTTGGTTTTTTAGCCATACAAAAAGG

AACCGTCCTAACTGAAGGTTCCAGAAATATTGAACGCAGTAAACTTATCGGTGGTCATTC

CGCGGGTGGATTGGAGGGATTAAAATGAAACAAGAAAAAATACAGTACAATTTTCAGAAA

TCCGTAGCAAAGGATGTAATGATATTGAAATGCTTGAAAGATTTTTACATGGAATCGTTG

AAACAGCAACTTCAAAACTTCGTCAGAGAAAACTCAAAACAACTGAAATATCGATACGAC

TAGTACATGCTAAATCTGAAAACCGATTACCATTGGAATTTACATTTAGCATTAAGCCAA

CAAGCTCATCTGTGATAATCTATACTGAGGTAATCAATCGCTTTAAAGAATGTTACACAG

GTGGGGGAATTCAAGGTTTTACGATTCAATTTGATAAAAATACCCTTGCCTCTGCATAGA

AAGGATTTGATATGATTGACCGTTCATATTTACCATTTCAATCAGCAAGAGAGTACCAGG

ATACAAAGATGCAAAAATGGATGGGCTTTTTCCTATCTGAACATGCATCAGCACTCTCTG

ATGATACAAACAAAGTAACGTACATGTCTGACTTATCACTAGAGAAGAAATTATTACTCC

TCAGTCAAGTATACGCCGGGCAGCTACGCACACGCATTCAAGTGATTGAAAAAAACAAGC

GTGTTTCCTACACTGGAACAATACCAAGTCTGACCAAAGATTTCATTTTGATAAAAACTA

CAACAGGTCACATCAATTTGAAATTAAAAGACATTATTAGTATTGAACTTGTCGAGGAGG

TGCTCTATGAATCAGCTTGAGTTTCAGCGTAATCACCTACAAATGGACTATTATAGCGAG

AGCTACCAAGATTTTGAACGTGACTTCTACCGCTACTCTAACATGAATATTCCATTGACC

TTCCTAACTGATGATATCCTAAAAACAATGGCGACTTCACGTAAGAATTACTTTGTCCTC

AATAAGGAAAAGTCCAGAGATAACCGCGATCACTTCTTCATATTTGAAGTAAGTACCGTA

GATGAGAATCCGCTAATCTATCATTATACATATAAGAAAACTACAATATATTTAGCAGAA

AAATAGGAGCAGTTCAATTGACTGTTCCTATTTTTAATATTCATAAAATCTAAAGTCTTT

ATACTCTTTAACAATGGAGTCGCCAACCAGAACAGACTATACTGACCAGCGACTACCTTA

AATTTAATGTTTCAGATTTATTTTCTTATCTCTAATTTCATAAACTACATCTGCTACATT

TTCGAGTAATCGTTTATCGTGGGTGATAAACACGATAGTTCCGGTGTACTCCTTCATTAG

TATTTCCAAAGCCTCTAAACTTGGTATGTCAAGGAAGTTACTGGGTTCATCCATTATTAG

GATGTTATATCTACCCATGAGCATTTTAGCAAGCAACAATTTTATAATTTCTCCACCGCT

TAAAACAGATAAACTTTTTCCAATATCGTTCTGTTTGAACCCCATAGATGCTAGCACTGA

ACGAATTTCTGATATATTGTAGTCACAATCCTTCTGCATAAACTCCATAACATTCTGATT

ACTGTTGTACTTGTAACCATTCTGTGCAAAGTAACCTATTTTTGCCTTAGGCGAAATAGA

AATTCCTTCTTCATGGTTTAAGATCATTTGGATTAAAGTTGTTTTTCCGATTCCATTACC

ACCAGTTAACGCCACTTTTGCTCCTAACGGAATTTGAAAAGATGCATTTTCAAACAGAGC

CTTATCCCCAAATACTTTATTAATTTCTGCACCGACTATAGGGTATGGATTATGGAGCTC

CAATGCTTTACTTTGCCTGAAACGAATTCTGCGAATGCCTTCCGGAGCTTCTACTTTTCC

TAAGGCCGCAATCCTGTGCTCTAGGGTTTTAGCAGCATTATACATCTTTTTTCCTTACTT

CCTATTGATTTTTGATGAGCTAAACGCCCTCCGTCTTCAGTACTTTTTTTCTTTGAAGAA

CCTTTTGCCTTCTGTTCTATTTTACGAGCCTGTTTTCGCTTTTCCTCCGCAGCCCTTTCC

AATCGGGCACGTTCCGCAATAAATTGTTCGTATTCTGCAGCTTGGCTCTTACGTTCTTCC

TCTTTCTGACGAAGATAATCAGAATAGTTTCCCCAATACTCAGTGATTTTGCCATCTTTC

AGTTCCCATATTTTATCTACTATTTCATCAAGAAAATAGCGGTCATGGCTAATAACTAAC

AGTGCACCTGTAAAATATTTTAGCTGTCCTATTAGAAAATCAATTCCTTCACGGTCTAAA

TGGCTCGTAGGTTCATCCGCTAAAATACCATGAACCTGTGCCGATAAGGCCTGTGCTATT

TTAAGCCTTGTTTCTTCACCACCGCTCATAGTCTGTATATTTAATTGCTCAACACCTAGC

TTGCCTACAAGTGCAAAATCTTTTTCCTCCTGCAGAGTTACTTCGTCCAACTGGGGAATA

TAGGCAAGTTCACCCAGACGATTCATTTTACATCCTGGGGGAGTTAATTCTCCTAAAAGT

ACCCTGAGTAAAGTGCTTTTTCCAGCACCATTTGCTCCTACTAAACCAATACGGTCATAA

TCATATACTTCTAATTCATTTATATCTAAAACATCGCGTCCTTTGAATTCCACACGAATG

TCTTTTGCTTTTAATATTAATTCCATAACATTTCCTCCTGTCTATAATCGCATGCTTTCA

TTTGCTTGTATGCAGGGAAAACCCTGCGATTTTAGCAGGAAGAGTTACATGAAAATAAGA

TACATAAATATTCCTCCAATATTGTTTATTTTAAATCTAATTTTCTAACCTCAGTTATCA

TTTGGCAAACTATAGCAATGCCAATAATTAAAATACCTGATAGTAAAAACCAATGATTTA

CACCGATTTTATCAGCAAAGAATCCAGAAAGAATTAACCCAATTGGCATAGCAAGTGACA

TGATACTTCCGATCAAAGAAAATACACGTCCTAAATATTCAGGCTTAATTTTCTCCTGAA

AAAGAGCTGTTTGCACACCGCTATAAAATGGCACCGAAAGCCCCATTATTGCACAGCAAA

CTACGAATATTACAAATCCATTTGGAGGAAGTATTCCCGAAACGGCTAAACTGGTCCCCA

TTATAAAAAATGAACTTGTTATTAGTAATACATGCTTTTCGAAGCCCCCTAATCTTCCTA

ATAATAAGCCTCCTGCTAGCATCCCAAATGCAAAGGAAATTTCCGTAATAGAAATATGCA

CAGGCGTTCCATTAAAGTGTTCCATGCTTATTAAAGGAAATAGTGCATTGATTGGCATAT

AAACAAAAGTATATAGTGTTCCTAAGAGTAATAAGGCAAACAATCCTTTGTTTTGTCTCA

GAACCACAACTCCTTCTTTCATCTCCCTTATGAAATTTGGTTCTAAACTTTGCACTTGAT

TACCCAGCTTAGGTATACGTACAATTGCTACCGTAATAGATGCAATCACAGCACCCAATA

CGTCGATGGCAATAATAGCATTTAAATCCCAAACGGAGTATAAGAGTGCTGCAACTGCCG

GACTAACAATATAGCTTATAGACTGCAAAGACTGACTATAGCCTGCGCATTTCGTTAGCT

GTTCTTCTGGTACTAAAAGTGGTGTAACCGCATTGAGTGCTGGGGTATGAAAAGCTGTTC

CAATGCTACGGATAAACAATACTATCATAATCATCCAGACAGGTAGCTCCATACAGAATG

CAACAATAGCAAGCACTGCACCAGCTGCTGCGATAATTAAATCGGCACCAATCATTATCT

TCTTCCTATCATGACGATCCACTAGCACACCAATGGCAGGTCCCAAAATCGCATAGGGTA

AAAAACCTACTAATGAAGCCATAGACAAGACCATCGCAGATCCTGTTTTTTCTGTAAGGT

AAAAAATAATCGCCATTTGCAGGATGGCACTAGTGATTAATGATACTGCTTGCCCTGCCC

ATATTGCATAAAATTTTCGTTTCCAATTGTTGTATTTTTCCATTTATATTATCTCCTGCA

TATTATTTTGCTTGAATTTCTATTTTGAATAGCATTCTAGGCAATAAAAAATGCAGGCCA

AACCCCACAATGTGGCTTTTGGTCTGCATACATACAATTTGGAAACATTCATATTAAAGA

CATAGTTAAATAAAGGTATAGTTAAATAACCAATATCCTCACCGTAACTAATGAATGCTC

AATATCGTATAAATAAGCACAACAAAAAAGCCTATCATCGGGTATAGATTCTGCTTTTTT

TATTGCCAGCTTATCTTAAACGCATTGAGGCTGTCATAGTTTCGGTTCCTCCTACATCTT

TGTTTATATCAATTTATAGTATAACACAACAAGATGATATGTTCAATATAAAAGTTATGG

AATGAGACTCATACTTCCAATTCGATGCCAGATTTAAAGGATATGACGAAGTTTTCTTCA

TAGACTGTAACGCTCTGGATTATCTTCCTTAGTAGCAAGCGATTAGCTTTCACAAAATCT

TCTGTTTGTAGTTTTAAAAATTCATCAGGATTTTCTAACTCAACCTCAAAATATTTCATT

TTACATTCCCTCATTTCATTTATTGATAAATTGAGTTTGCAAAAAAGAGTGGACAATTTT

TGTCTACTCTTAACCTTTAAAATAGTTTTTTTAATCGATTTGAAGTTGCCTAAATTATTA

CTTATTCGGTAAAATGAAGTATTGCTTTCAACAGATTTCCTTCAACTACACTTCACTTGA

TTCAAACAAGGTGGGTACATTTCTATTCCCACAAACTCCTTGTCAATGGAAACAAACACG

TACCCACAGGGTAAATGGAAATAGAAACTGATAATTTCTAGCTATCACTTCTACTCATTC

CAAAAATTTTCTCACTCTGATACTTACCCACCATAAAGCAAAAAGCCTTGCAATCAAGGC

TTTCATTATCCCTTTCGTTCAAAGGTTTCTAAGCTTTTACGAGCAGAGCGACACACTCAG

CGGTTCGCTATCTCCGTTCTGTCTGCGTGCTAGCACTTGTCAATCACGGACAGCTATCGC

ATGGGCGGAAGTAAATGCTAATCTTCGTCGTTTTACTCCTTGACTAGCAAACTTACCGCC

TCAACATGTCCTGTATGTGGAAATAAAACACGATTAAAGATAAGGGAAGATACTGAATTA

AAAAAATTCCCCCTCTATTGTCCGAAATGCAGACAAGAAAATTTAATTGAAATAAAGCAG

TTCAAAGTAACTGTGATTACAGAGCCAGACGCAAAGACGCAGAGCCGATAAAATGAGATT

AATACAATCTCATTTTATCGGCTCTTTCCGTTATGTATGGATTCTTTTAATTAGTCTTCG

ATGTTTCTTGCTTCGTTGATACCGCTGGCTAAAGATTCCATTAAGGATAGTTCTTTGTCT

GTAAAGCTATCCATGTATTTCTCTATCTGTAATCGTCGGGTGCTTTTTACCAAGTTATTA

GCAGGTAAGAAAAATTCATCAACGGAAACATGAAGTAACGATACAAGGTCATAAAGAACT

TGTATGCTGGGGTGTTGCCCTTTATTTTCAATATTAGTTAAGTACCGTGGGTCAATTTCA

ATCAATGCTCCCACTTGTTCACGAGTTAAACCTCGTTTCAATCGAGCTTCTTTAATGGCT

AAACCAAAGGCTCTAAAATCATATTTATCTTCTTTTTTACGCATAGTAGACCACCTCTAT

ACATTTTATTGTTCCTACTGAATTAAAAACAGGTATAGAAAAACGTGTTATATGGTTTAT

AGGTTTATATTTAATAAAAAGCACTACTAAACGCCAATAAAAAAACCGTTATATGGTAGT

GCTATTTACGCTGTTAAAATATTGTATATTACTTCCAAATGGCGGTTTGTTGGAGGTCAA

CGTCGCCATGAAGTACATCATATACAATAAATTTCCTTACATTGGGTTCTTGTCAAAAAA

AGTCGTCTATCTGCAATAGATAAGTACGTCCACCAATGTGGTTTTATAAATCATATAGAT

AGAATAACAGAAGCATGTAAACAGAGAAATAAATCTGTTTATATGCTTTTTTGGCTATTC

AGAACTTTTTTACAAAGTTTATTTATCAGTAATGCAACAAATCCCCCTTTCACATTGGGA

CTAAGAGTGAAAGGAGATAAACGAGCAAGGCTCACTTCCTTTCCTAGACAGAAAGGGGGT

GAGAAACATGAAACCATCTTCTTTTCAGACCACAATAGAAAATCAGTTTGACTATATCTG

TAAACGTGCTATGGAAGACGAGCGAAAGAATTATATGCTTTATCTTTCAAGGATTGCAAA

GCGTGAGGTGTCCTTTTCGGATGTTGGCGATTATCTTGTTAGCCAGTTTGCGACAACAGA

TAACTATTCAACTGACTTTCAGATTTTTACACTCAATGGGTTATCAGTAGGCGTTGAAAA

TGATTTGTTGAGTGAAGCATTACGTGAGTTGCCAGACAAGAAACGTGAAATTCTACTGCT

GTTTTACTTTATGGACATGAGCGATTCAGAAATTGCAGACCTGTTGAAATTGAACCGTTC

TACTGTCTATCGGCATAGAACCAGTGGACTAGCCTTAATTAAAAAGTTTATGGAGGAATT

TGAAGAATGAAAACACAATATCCTATGATTCCCTTTCCTCTCATTGTAAAGGCAACAGAT

GGCGATACCGAAGCGATTAACCAGATTCTACATCATTACAGAGGGTACATAACGAAGCGT

TCCCTACGACTTATGAAAGATGAATATGGCAATCAAAGTATGGTCGTTGATGAAGTCTTA

CGTGGAAGAATGGAAACCAGACTGATTACAAAGATTTTGTCATTTGAAATTAAGTAATAT

CCTCTCTCCTTTCGTGGAAGCGTGCTAAACCATTCCACGCTTCCCGAACAGGGAGGTTTG

TTATTCCACCAAAGCATATTGAGCTTTCAATGTGTTTTGATAGGCTAACGAGCCATTGTT

CTTTGAAAACTGAATAAAAGTAATCGAATACGTTTCGATAAGAAAAGAGCCAACGGAACT

AACCGCCATGACCTATCTTATAAAGATAGCGAGCGATTCATGTTAGTGATCCGAGAAGCA

ATCTTTAGCAGGATTGCCTGCAACGACATTCTTATCGTGATAATGATACTCCCATACAGT

CAATAGTCCGAGCGTGATAAAACCGTCGCAGGCAATGAGTATGGCTACATGAGAACCATG

CAGGGGTGGAACTCCCGTGAGCTTTGCTAAAGCTGTTCGATTGCTGGTAAAACAACTTTT

ATGAAATCCAAATAAGTGATTTGGAAAGGAGGATTTTATGAAGCAGACTGACATTCCTAT

TTGGGAACGTTATACCCTAACCATTGAAGAAGCGTCAAAATATTTTCGTATTGGCGAAAA

CAAGCTACGACGCTTGGCAGAGGAAAATAAAAATGCAAATTGGCTGATTATGAATGGCAA

TCGTATTCAGATTAAACGAAAACAATTTGAAAAAATTATAGATACATTGGACGCAATCTA

GCGTCGCCAAAGGGTCTTGTATATGATAAAATAGTATTAAGTCGTATCAAGGCTCTTTCC

ATAAAGGAAAGGAGCAAATGCCATGTCAGAAAAAAGACGTGACAATAAAGGTCGAATCTT

AAAGACTGGAGAGAGCCAACGAAAAGACGGAAGATACTTATACAAATATATAGATTCATT

TGGAGAACCGCAATTTGTTTACTCGTGGAAACTTGTGGCTACAGACCGAGTACCAGCAGG

AAAGCGTGATTGTATCTCACTTAGAGAGAAAATCGCAGAGTTACAGAAAGACATTCATGA

TGGTATTGATGTTGTAGGAAAGAAAATGACACTCTGCCAGCTTTACGCAAAACAGAACGC

TCAAAGACCAAAGGTTAGAAAAAACACTGAAACTGGACGCAAATATCTTATGGATATTTT

GAAGAAAGACAAGTTAGGTGTAAGAAGTATTGACAGTATTAAGCCATCAGACGCTAAAGA

ATGGGCTATTAGAATGAGTGAAAATGGTTATGCTTATCAAACCATCAATAACTACAAACG

TTCTTTAAAGGCTTCATTCTATATTGCTATACAAGATGATTGTGTTCGGAAGAATCCATT

TGACTTTCAACTGAAAGCAGTTCTTGATGATGATACTGTCCCTAAGACCGTACTAACAGA

AGAACAGGAAGAAAAACTGTTAGCCTTTGCAAAAGCTGATAAAACCTACAGCAAAAATTA

TGATGAAATTCTGATACTCTTAAAAACAGGTCTTCGTATTTCAGAGTTTGGTGGTTTGAC

ACTTCCAGATTTAGATTTTGAGAATCGTCTTGTCAATATAGACCATCAGCTATTGAGAGA

TACTGAAATTGGGTACTACATTGAAACACCAAAGACCAAAAGTGGCGAACGTCAAGTTCC

TATGGTTGAAGAAGCCTATCAAGCATTTAAGCGAGTGTTAGCGAATCGAAAGAATGATAA

GCGTGTTGAGATTGATGGATATAGTGATTTCCTCTTTCTTAATAGAAAGAACTATCCAAA

AGTGGCAAGTGATTACAACGGCATGATGAAAGGTCTTGTTAAGAAATACAATAAGTATAA

CGAGGATAAATTGCCACACATCACTCCACATAGTTTGCGACATACATTCTGTACCAACTA

TGCAAATGCAGGAATGAATCCAAAGGCATTACAGTACATTATGGGACATGCTAATATAGC

CATGACGCTGAACTATTACGCACATGCAACATTCGATTCTGCAATGGCAGAAATGAAACG

CTTGAATAAAGAGAAGCAACAGGAGCGTCTTGTTGCTTAGTAGTACAAATGAATTTACTA

CTTATTTACCACTTCTGACAGCTAAGACATGAGGAAATATGCAAAGAAACGTGAAGTATC

TTCCTACAGTAAAAATACTCGAAAGCACATAGAATAAGGCTTTACGAGCATTTAAGAAAA

TATAAAAAGATAATTAGAAATTTATACTTTGTTTACTGTTAAAATTTCTATTTCCTCTGA

ATCTCGAATAAAGGTCTTCAGCGAACTAAGAATTTTTTTCTATTACCTTAATAGTATCAT

AAATGAGGCTTGATTGAAAGCGTAAACATAGCTTTGATAAGTGATACAACTATTCTATAC

AAAATTTAAACATAAAACTTAGGTATACTTAATAAATGAGAATTTAAGTTAGCTATAACG

AAGATAGTAGCATATGTAAAACTCTAAGCAGGCAGAGCCTGCTAACTCATGACAGAAAAA

GATGTTTAGCCTATAGTGCTGTCATTAAAAAATCCTCACACATCACGCAAGGAAATCGCT

AAATAAAACAGGCTTTGACCATGTCGGGATTAATTGCAGCACATTCCAAGGATCCAGCTT

TTTCTTTTTTTATAATCGAATTTCCCAAAGAGTAAGCAAGATGAAGGCCGTCATTGCTTA

TGCTCATAAGATTCTCAGAATCATTTACAAACTACTTTCCGCCAAGCATACTTATCAAAA

AGAGAAGGGGCTAGGACTGAGGAAACAGTTCTAACGCCAAAACTAAAAAATTTCAATTAC

ACGCTAGTACAGGAATTGATTTTTTACGCTTTCTTACTGTTTTTCCAAATAAAAAAGATG

ACTTACTGAACCTGTAAGCCACCTAATCAGTCGGTTTATTCTGGTGTCTGCCACCGCTTG

GCCCGTACGTCCAAGATTGCTATCGGATTTTGTTCTGGTGTCCGCCACCGCTTGGCCCTT

ATGTCCAAGATTACTATCAGATTGACCATGAGCCGACCACTCATTTATAGACATATTGTA

TCTTTAAATAGCTACAATTTCAAGTGTAAACTCTTCTATTTGGTCGTATAAGCAAAACTG

ATGGCACTATCTGCTTGAGAGATTTTTCTAAAGGTATAGTTAGGGTTGCCCCCATAGTTG

GTTTCAGACACAAGGAAAGAACCATCATCATAGACCTTCTCTACAAAAGCCACATGACCA

TAGCTGGCTGGTGTACCATGTGTACCTCCTACAAAAGAAACAATAGCACCTGCTCTTGGT

GTAGAGCCCGTTTCCCCACCAAGACTTGAAGCTGTCGCAACCCAGTCTTGACCATTTCCC

ATGGTATTAATGATTGAAATCTTTTCTCCATTTCTACCTTTTAATTTTAAGCCTAACTGG

TTCATACGAGCCGCAACACCCCATGTACATTGTCCATAAGCATAGGCCATACCATCTCCA

CCACCAGGAACAGAATGATCATACAAGTCCCCACGAACCCCTTCAAGGGATTGTGGATCA

CTTTTTGCCTGTCCTCCATTTGTTTGACTAAAGCCTTTTTCAATTTGGTAATACCATTCC

GTTGCTCTGGTTTGTCTTTCCAGTAGTTTGTCACCAGAATTTCCCTCCCAATAGGTCAGA

AAGAGTTGGGCCAGATTGGCTGCACTGCCTGTATTTTTAAAGAAATCCTTTAACCAACTT

TGATAGTAAGGACTATCCCCATGAAGCATAAAATCAAGTTGTAGGTCTAAATCATACCAT

TTCTTATTTTGGGTGCGTGCATAATTTAACAAGGCTGTATGCCGTGTTGACCCATCTGCA

GTATCCGTCCATTGCCCTAAACCCAGACCTCTATGAAGAATATTAGGATAAGCACCACTA

TAAATGGCTGGACCTCCAATCGCTAACCAGCTTTCATCATCCCATGAGGAATCGGTAGCG

CCAACAGGAGGAGATAAATAATCTCCTTCAGCTCGTTTAGGATTAATAGAAGACTCTACC

GACCAATTTCCTAAAATTGCCGCAATGGCTTGGGGACTTGCCCCTTGAGATTTCAAAAAC

TCATAAATATGTTTTGCTCGTTCAAACTCATCCCCACCAAACTGACCAATGGCAGGTAAG

ATAGTTGTCTGAAGTTGAATGACTTTTGGAAAATAAAATTGCGGATTGACATACACCAAT

TTTTCTTTCTTGTTCTTATACTTTTGATAGGAAACTTTCAAACCTGTATCGTCTGGTGTT

TCACCAATAATATCACCCGTTAGGACTCTTGTCCCCTCAATCGCACGGCCATTATGAATA

GAATACAAGGTCAATCGACTCTCATTCTCTCCTTTTCCGTTAGTGAGAATAACATCATCT

CCGTCTAGAGATACAACACCATCCATTGGTGCGACAATTGTTTGGTGAGCCTTCGCTTCT

AGTAGAATATACTCCTGAAGAGTAGGTTTTCCGTCTAAATCATAGTATCCATAACGATAA

GTCATGGTTAGACTATCTTCGTTGCTTTTCCCCTCAAATGGATTGTCCAATTCCTGCATG

GAAGCATAGACACCCTCTTCTTTTAGTTCCTTTATTTCCTCTTGATCGTCTCTCGATAGT

TTATACTTAGGAGTTTCATAGAGGTCTTGCATGGATTTCAAATCATCCCCATCGTTTAAA

TCATGCCACAAAGTAGACAGATAATCCTTGTAAGATTCTGAACTAAATAAGTGAACTGGT

TTGTGTAACTCATAGTCATGGAATTTAAAGTTCATATACCCCATCACATCATCAACTTTT

GTGTAATAAGTAATTCCTTTGTCATTTGTGCGAGTATGTTCTGCATCTTCCCAAGTTAGG

TGGGTATAAGCTTTTGTTAATTCAAATTCATCTTGTTGAATCAAACTAGCAGATGAAAAT

CCTAAAAAGAAGCTCATCATAAGTAAAAGAAGAAAGACTATTCCTCCAACTATCCAGGTT

ACAGGATTTCCAGCCACAAATGTAAAGAAGGAAAAGGCTGCTTTTAATTTTTGATAGATA

TTTCGGACACTTAAAAGACCTTGTTTCTTTAATTTTCGATACCGATTTTTAAAGGAACTT

GGATTATCTTTAGTTAGTTTCCATCCTTTTCCATCCTTAAAATGATGGTATCGCTCTTTT

GTGTTGGTCAGTCTTTTCTTGGTAAAACGACCTGTTGCTTGTCCTGTTTTGACACTAGCT

TTTCCAAGATTATAAGAAAGCCGACTGTAGCGTTTCCCTTTTCTAATGGTCTCATGAAGT

GTGCGATAACTTTCTAAATCTTCATTTTCTGAAGCTAACTCTCCACCTTCACGTCCAAGG

ACATAAAGAAAGGTTTTGGCTTTCCTACTGATTTTTTTGAACTTATAGGCTTGCTTAGTA

GATTTTAGATTCTCTTTTGCGGCTTTGACTTCTTTCTTAGCTTTTAATTCTTCTAGATTC

TTCCCTTGAAGGAAAAAATTGGATCTTACTTTCGTTTCCTGACCGTAGAAAAATTTTTGA

TTGGTTTTTCTTTCTTTACGACTCTCTTTTCTTTCTTCTTTTGCTTCAACCTTAGCTTCC

TTAAATTGTTTCTTGGCTATTTTCAATCGTTTCCTAGCATGAGGCAATCGTCTATCTCTT

AATTCTTTCCGATTCAAAAGTGATGGAGGACTATTTTGAAGAATATGATTGTAGTCTTCA

TTTGCTTGTTTTACTCTAGCCTTTGAAGCTTCTCTCATATTCTCCAGCTTTTGCTTTATC

TCATTTTTCCATGCTTTTTCATCCAGTACAGCGGAATCTTTTTTCTGTTTTCTCACCTCC

TTCTTTCCTTGTTTTAAGAATTTCTTCTCATCTTTTAGACTTCTTCTAAATGCCTTTCGG

GCACGTATGATTTCTCTCTTATCCTTCATTTACCTTCCCCTTAATTAGAAGCCATTTTAT

CAGGATCTGTACTCATGATATCAAACAATTGTGTACCTTGGGGAATCTTATTTTTAAAGG

GAACGACAACTGAACCAGCTTTTATTAGTCCTGCCCCTTTTTCTGGATTGACTAGGTATT

TTTCGAGTTCTTTTGACAAGCCTAAGAGTTGAACCAGTTCTTCCCGGTCATTTTTTGCTT

GCTTGAGGAGAATCATAAATTCACTATTGGCAATAATCCGTCTACCATTTGGATCTAACA

ATAAGGTTTCGACATTTTGAGTTATTCCAGTCGGACTGGCTCCATATTTTCTGACACGAC

TCCACAATTTAAAGAAGAAATCACTGGCATATTTATCTAATAAGAGAAGCTGCATTTCAT

CAAAATAAATCCAGGTCTTCTTCCCTAATTTTTGATTCCGAACGACACGATTCCATATCT

GATCAAAAACAACCATAAGAGCAATTTGTTTCAACTCATCTCCTAACTTTTTAACATTAT

AGATTAAGAAATTAGATCCTGTCTGAATATTGGTCTTATGAGAAAAAATATCAAGAGAAC

CTTCGACATACAGTTCCATATCAAGTGCCAAATTCTGCGCTTCTTCTTCTGGTTGTTGGC

TCAAGACAAAGACCCATTCTTCCAAAGAAGGCTCTTTAAATGACTGATAGGTGAGTCTGG

TAACTCGGTCAATAATCGATTTTTCTCTTCCATCCATTTTTCTATCCAATAACTTGCCGA

TAAAGGATAAAAGAAATTCTGATTTTACTTTTACAGGATCTTCATCCATATTTTCTTCAG

ACAAGTCAAGGACATTGAGATATGTTTGGGAATCTGGCGCAATATCAATCATTTCTCCCC

CAAAAGCTCGTCCAATGACACTGTATTCTGCTTCTGGATCCACGATGATAATTTCTGTAT

TTTCACCAGATTCCTTGATTTTTGTCGTGATAATTTCATGCTTGGTTGCCATCCCTTTAC

CGGCTCCAGATGTCCCTAAAATTAGACCAGATGGTGTATTTAATAGGCTGCGATCAATGG

TAATAATATTACTTGATATTTGATTGATACCGTAATATTTCCCACTACGGTCTTGTAAAT

CTACTGAAGTCCATGGAGAGTTCACTGCTACATTAGATGTTAATAAACTCCGTGATACTC

CCTCTAAAAAATCACAACCAAATGGCAGCAAACTATTAAAGGCTGCTTCTTGCATATATG

GAAGTTTATCAATCATTAGGTCATTTGAGCCGGCCACTTGTTGAATCGTATCTAGAGCTT

GTTTGAGTTCTTCTTCATCCTGACCAAAAACACCAATCAAAAAGACTGTTTGAAACAGTT

TATCTCCTGTCTCTGTCATGGTTTTTAAGAGTTCCTCAGCTTCATCGATATTGCTTTCTA

AAACATGGCCTACTTTTTCCAAATAGATACCTGTACGAGCTAGTTTTTGTTGTTCCCCAA

TCTTTTGGGATTCCATCAAGGTCTTCTTTGTTCGTAGTTTCTTCATGGCATCTGCTTTGG

TCGAACTTTGAGCATGGAGGCTCACAATCAATTCCAAATCTCCTTGCATGAGGTCTCGAA

TAAACTGATCCCCTAATTCCATGCCGTAGTCTCTCACATAGACAATCTGCAATAAGCGGT

CATTGATTTGTAGGTAATTCTTGTTTTTGAAATCTAAGAGATTAGGCGCTATAAAGTGAC

GAGTTGTCTGACCCGATCTCGTTAAATCACGGTAAGAAAACGGAAGATGGTGTTCTCTTC

TAAGCATATCTGCCAACAAGTTCACACGGTTTTCTCCAGCCAAGGATTCAAAGCGAGCAT

CAATTTCTGAGAAACCACTCTTGAAATATTCTCCTATTTGGGACAAGGAACGATAGGCTT

GTTTGGGATTAGAATCCTTTCTACCAAAGGTAATCAGTTTCACAGCTGAAAAGTTATTTT

CACCACTGTCTAAATTCTGATTCATCATCCGATTCAATTCTTTACGATAGCTATCATACC

CATCTTCTTTTTCCTCATACAAAACACTTTGTCTAAACTTTTCTAAATTCAATCTTTTAT

TAAAGATGGTCAATTGGAAGTTGGTTTGGTCATCTAGGGAGTTAATCAAATCAGAATACT

TCTCAATGATTGCGCCCTTATCTTCTAAACCAACAGTTTGGTAATTGACATCACCAAGTA

AGTAGCTTTGTGAGAAATAATCTTCTTTTACCTGCATTAGACCATTTTGATACAGGGCTT

GATAATAAAGAGTATTCGCCGTAGAGGGTAACACTTCCTCTTTTTTCCCTTTAACTACTT

CTTTTTTATTAGTCATTGAAGTTTTTTGTTTCTTTAATGTATTTGATTTTCTTTTCATGT

TCAGGTCCTTTCTTTCCTGTAATTGTGCGTAGGGGAACCGTTAATTCAAAATGAAGACGG

TATTTCAAATAATGTTCAAAATATAAATCATTGGGTTTATAGACTCCAAAAAGCATGAGG

GGGATGGTAAAGGCAAACACAAAACCGTAAACAAACCAATCTCCAAATTGCCAGAAAAAG

AGATTCAAGCCCAAAACAATAATTGTGACAATAAAGGCTGGTAAAACAAAGATAATTTGT

CTTGTGGTGAAGCCTAACCAAGCCCTGTGTTGGTATTTTGAGATGTCTTTAAAGACACGT

GTATTCATGACTTTCCTTTCTAAAAAGGCTAAGAAGCATTCACTTCCTAGCCTTTATCTA

ATTACATACCTAAAATTGAGCGAGCCGTACGTTGAGAACCAACGAGAGCGATAATCAGCA

AGATAGCTTGTACCAAACTACCAAACATAATCGCAAGTGATTGCAAGACTCCTGCACCAT

TTGAAACAGCTATTTTCCCAGCAGATTCAAACAAAGGAACAAGAGAAACAATCAGAAAAA

TAAGAACCCCTTGTACCGCATAGACCATAATATTTTTTAAATAGCCAATACCAATAGACT

TCCATTCATCACTTAAAAATGTTGGAATCGTAAGAGGGGCAAATGGAATCATAAGGTAGA

GTTGAATAAATCGAATAGATACCAAAAGATTGACCATGGCTGCACTTACTATCCGAACTA

GCCAAATGAGGAGGGCGAAAAAGCCCACAATCATCCGGCCAATAAATCCTGAACCTTTTA

ATCCAGAGATTGTATCATACTTTGCCCCACCGTGAGCCACAATCGAGGCCACTTGTTCAA

TGGCGTGACTCGCAATACCGATGATAGCTTCTACAATGACGGTAGTGTTGGTAATTACAA

CTGCGACCATAATATAACTAATCAACATCGGCGCTAATGCTTCAAAGGTCATCGCTCCTC

CTGAGTTAGCAATCTTCTTTGCCATCTTCGAAAATTCTAGGATGAGAACAACTGATAAAA

TCGCAACTCCAAGAGGCTGCATGACACTTTTAGTAATACTAGACATATAAGTCCAAACTG

TTGGATTGTAGCTAGATAGAGATTTAATCAGATCTACCGTAGATTGTAAATCTACATTAA

ATCCTTCAAATAAATTTTCAGCTGATATTTTTTCAGATGCAAGGTAAACAAAGGGTGAGA

CTAAACTAAGATTCATGTCATTGTTTATCCTCCTAAATTGAAATTTGAGTTACAAAGGCT

CCAGCAGCCCCTACCATAACACCACCGACAATTTCCAGAATGGCATTCCGAACACCTGGT

CCACCATCTTTAATGTTGGTTGCAAGATTGACAATTCCTACAACAACGAGAAAGGCACCA

ACCGCAATCAATCCCTTCTGTAACAAAGACATAGCTTGTGCAAACATGGCACTTGCGTCT

ACTCCATAAACAAAACCTTTAAAATGCGTAATCATCTATTTCCTCTTTTCTATTTTTATT

TTAAACTAGATTCAAAAGTTAAATCACGAATTCTAAGGCCTTCAAGATGATTTTCTTGTC

TTTGATTCAAAGGATTGATTTGATAGTTCCACCACCGTTCATCGGTTTCTTGGTTGGCTA

GGTACTTCCAGTTTGGATGCTTAGTTGAATTGTATTTTTTGCTTTTAAAGACTGGCATAT

TGGCAATTCGAACCAGGCATTCATGCCGTTTCATATTTCCGACTTCATCAGGTGTCATTA

ACCTGTCTGGCCAAAGGAACGACTAGTATTTCGAACATCAATGGTTTGTTTACCGAGTAA

GCCACTCATAAATTTAAAGGTATCTTCATCATTCCCACCTAAGTAGACTAAGCTATCACA

GTTCCCCAAAATGGTTTTCCAAGCTTCTTTTTCTTTATAGAGCCCTTGAAGTTGGGCAAT

ATTTTGTAGAATAGGAACGAGACTCATGTTCCGAGAGCGGACTGTTGAGGTTTGTTCAGC

AAAATCTGGGATTTCTCCGATATTTGCGAATTCATCTAAGTAGACTCTTACATGAAGAGG

TAATTGACCCTTAAAATCAATATCTGCTTGTCTTGTTAGGGTTTGAAATACGGTTGAAAA

AAAGAGGGCTGAAAGAAAGCGAAAGGTACTATCGTTATCTGGGATAACTAAGTAAACCAT

TGATTTTTCCTTGCTCCATGTCTTCATATCAAGGGTATCTCTTTTGGTCAAATCCATGAC

ACTTTGAATATTGAAGAGGGCAAATTTAGCAGTGGTTACAGCTATAACAGAATCCAGAGT

CTTATCCTTATAATTTTGGAAATCTGCCCAATTTCGCATGGTGAAATTTTCAGTCCCATA

CTTTTTAGCATAATTTTCAAATAGAATTTCTAAGACACTTTTTTCTTGGTTTTCACCCTT

GGATAAGTGTTTAATGAGTTTTGAGATTTCAGCAAAACTTGGATAACACCCTCGTTTTTT

TCGCTCTTCCACTTCTTTTTTTGACGTTTCAACAAGTTTTGGTATTCTGTTTGACTCAAA

CGACTTTCTTCTATGAGCTGTTCTCTTGTTTTGGGTGGGTTATAGAAATCGACCAAGTAG

GAGGCTAAAGCTCGTACCAAAGTCATAGAAGCTTCATCCCAAAATGGATCACTACGGGAG

CCAGAGCCTTTGGTGTTATTAAAATAAACCGTCAGCATGCGATTCAAATCATTTTCTGTC

TCTATATAGCGAAAAGGATTGAAGCCATCTGAGTTCTTCATATTGACTAAATCTAGCACC

TTTACTTGGTAGCCATGTTCTAAAAAGAGTTTGCCTGTTTTTTCGGCCAAGTGATCTTTA

GAATCCACTACAATATTAGAACTATTCATCTGAATAAGATTAGGTTTCACAAAGCGAAAT

GTCTTCCCACTTCCTGAACCTCCAATCACCGCAATATTCTTATTTCTGTCATATTGGGGT

GATTTTTTATCTAATAATGTCAAACGAACATCTTGTGCTAAGATCGTATCATGAGAAAAT

TCTTTACCGTAAAAGAGCTTCTTTTCTTTTAGAGTTCCAAAACGGGCACTCCCGTATTCT

ACTCCTTCTCGGTATTGTTTCTTACCGGTTTCTAGATAGAGATAAACCAGTAACATCATC

ACAAAACCTAGTAGAAAAAAAGCACTTGATTTTCCAGTAAAAGAAATATTCCATGGCGAC

TGAAGAACTTCATCTTGACCTTCCATCAGAAGATGAGTCCATTTATCTAGTGTATTTCCA

GTATAGGAATCATACAAAAGCGTCAAACGATGAAAAAGATAGCCTAGTAAGATACCTAAC

AGTGAGAATAGTAGGAATTTCTTTCCACTGTACATCATCTCACCATCTCTTTCTGTTTGA

CGGCTCCTTCTTGTCTAAAGGTAATTTGGGATTTAGCCTCATCAATTGCATCGTCTAATG

ACTTATCCATTGTAAAATCAGCTAATTTCTCCGGATCATTAACCATTTTTTCTAACAGAT

GGTCTAAATGATTGTCTAGAATCGAACGGTCTTTCGTGTAGAAATGCAGAGAATCCCCTT

GCCAAGCGATGGCTAAAGGAATCTCTTCTTTTTCTAAAAAATCTTTAAATTTCTCTATAT

CAATTGGTTTGTCTAAAAAATCTTTTTTCAGATTAATCGTATCAATCGAATAAGGAGATT

GTAGCAATTCTTCTAATTTCTGCACCCCTACCTTATAAGCAGAATCTTGTGCTAAAGCCT

GACGTCTAGACCATTCTAGAATCTTTAAAAGACTTTTTACAGTAAATAAAAGACTACGCT

CAGCATATTGAACTGCCATTCGTTCCTGTTGTTCAGAGGACATCTGATGCCTCCTTCTTC

ACAAATAGCAGTTTTCCTTCTTTATAACGATAAGCTATCAATTTCTGACGTTGCTTAATC

GACTTGACAACCTGCAGTAGATCTCTCGAATAAGGTTCTCGAAGAGTGACTTCTACTGCT

TTCCCATCAAAGATACCAACACGTTTAAACTCAATGTAGTCTTTTTTGAGATGTCCTAAG

CCCATACCAAAAGGAAAGTGATGAATCAATTGGATGGTACAACCACCTTTATTTCCCATT

TGTTTCAAATCATACAAGTTTACTACCTTCATGATTAACTCCTTTATCTAGTTTGTCGCA

TCGTTTAAGTCTGGTAACGATAAACTCCGTGTCTGTTAGAAAATTCTCACACACGTCTTG

TGCCAGTTGCCCTTCACAGGGAAATACTCTCAGTCCCTACTTACACAGGCACGCTAATCA

AGACGGAGTGGATTCAATTTTCAAAGAACAGGTAGCTTTATTATAGATAAGAGTAGTTGA

AATTTTTATCACATTTTTTATTTTTCTGTCGAGCTAAATTCATATTAAACAAAGATTTTG

AAATAAAAGACTAACATTGTTACATAACGTTAGCATTTCATTTCCCTAAAAAAACTAATT

TGGTCTAAAAAAGCAGCAAACTATAAACTAGTAGGTTCCACACCAAATGTAGCCCCATAC

TGCCCCATAAGTCCAATTTGTAGCGTACAAGCCCTAAAAACATCCCCAGCGATACATACA

AACACCAAGCTATAATGGTTCCTGGATGATGTGCTAAGGCAAATAAAACACTTGTCAACG

CAACTCGAATATCTAATTTTCTAACCAAGTTCCATAAAATTTCTCTATACAGAAATTCTT

CAACCATACTCGCATTGATTAAAAACAATAAAAATGAAAACCAAGGAACTTGATGTTGAA

GGCCAATTAAGTTTGCTTGATTCGTGCTTTCTTGCGCATGGATTAGACTAAAACATAGAC

TTATAATCAGTAGGCTAACGAATCCAATACCAAGCCATTTCATCCTAGATTTCATATTTA

CCTTACGAGCTTGTTTGCGTTGGCCATACATCCATAAAAAAGAAATGAGTGACGAACCAT

AGAGAATCTGTAGTATAGTTAACTCACCGATACAAAGAAATTTCAATAAGTATAGAGATA

CCAATAGAACATTTACTTGTTGGAATATATAAACTGGAATTATTCTTTTCATAGTTACCT

CCGAAATAAATCTTCATAATCTAAATCTAATACCTGCACAATCCTTTCTACCCATGGACT

TTGAGGCATTCGTTGTTCCATCTTCTAGTGGCGAATCTTTTGATACAAACGATTCAATTC

ACTTGGATAGTGAAACTCTCCCGCAAACATTTTTCTGGTTAACTCAATCCAGCTGATATT

TCTTTCAGCCAAAATAATGGACAAGTTCTCCCAAAATCGTTCAGCCATATTGCTTCTCCT

TTAGTTAGATAAATAATGTGTTTGCGCCATGTAAATCAATTGTTTCGTATCTCTTGGCAA

TAGAGCTCTAGCCTCTTCCAGATTCAGATTTGGATAAACTCGCTTATTTGAAACCGCAAG

AGGAAGTCTGATGGTTAGTTCAGGATTTTTTAATATCATTTCGATGAAATCTGTTAATTT

TAGGTTATCTCGATTCTTAAAACGTAATAAATTTGGAGATAAAAACTCAAAACAATCTGA

AGAATAGCTCATCATCTCAATTAATTTGTCCTTTGTCATTTCAGAAACTGAATGACAAGA

TACCTCGATGCCATAGTTTTGGAAGAAGTCTAAAAGAAGTTGATTTCTTTGGCTATTTTT

ACTTAGATAGAGATCAATCATAGGAGACCTCCCAAAGATTCGGTTCCATTTGATATTCTG

AGACGATTAAGGAATCTAATAAATTTGCGAAGTTAATCGGTTTCTTGTCTTCATCATAAG

CTTTTACAGTTACTTGGGGTGTAAGTATCCCCTCTTTTCCCTCGGCTCGATAGCCTTGTC

CATATAAAACAAAAACGAGATTTTGATGATCATCTACAGAGGCATCAACCCCATTCTTTA

TGTCTTGACTTTCAAGGAATTCCATAACGTTTTGAAGATAGGATTCGTAAAATAGTGGGT

AGTTATGTTTTTTATGGTAATCATCTAAAAATGTCACTTCAAACTCACATGGATAATTGA

GCATCAAAAATATTTGTTCATCTAGCTGTTTGATCTCTGCATCATGTAATTGTGTTTCTA

ATTCATCACAATCTAGTATTGACTCTTTATTTAAAGCTTTCATCTTTTTCCTCTATTTCT

TTTAATTTCTTTGCGATTGCAGCAATCACAGGAACGGTTACACTATTACCAGCTTGTTTA

TAGAGCTGACTATTACTAGAGACTTTTCTAGCAGCTTCAAAAGCCCAATCAGGAAAGCCC

TGTAGGCGAAAACACTCTTTAGGAGTGATTCGTCGTATTCTCAAACGGTAAAATTGTCCA

TCTATTAAGACACCGGCTACATGATAAACTTGTTTATCTTCTCCTTCATAGCTAGCAACT

ACTACTCCCATTTGTCCACTAGTTGTTAACGTATTCGCTATACCTTTTCCAACTCTACCT

CGACGATACTGAGAACTTGGTCTTTCCAAATTGATGGAATCTCCTATTTCTGCTTGAGCA

TATCCTTTTTTCGTTGCTTCCCGTACTTTTAGAAATTGGATTGGTTCTGGAATTAGTATT

TTGGGGATTTTATCTCCTCCTTGCATTGTAGTCAGTGTTGGAGATAATCCCTCACTTCCA

TAAACTCGACCAGTTTCCTTAAAGCTAGTTGGTAAATCTCCAACAACGACAATACCATGA

CGATCCTGAGTATTTAAAGTAAACATCGGCTCTTGGTTGTTCTTAAAGCGTCTATCATTT

TGTCTCTTGTCTAATCTATCTGGTGTCATACAAGGAATCGCAATCTTAAATCCTTCTCCT

TTCCCACGAACTAAGGTTGGCGCAAGACCTTCTGAATAATAAACTTTACCGCTCATTCCA

CTTTTTGATGGATTCAAATTTCCTAATGTTTTTAAAGTCTCAGAGTTAGTTGCTTGACCT

TCTCGTCTGAAAGGAAATAAGAGTCTGGTACCTTTCTTTCTAGAATGTCCGATAATAAAC

ACCCTCTCTCTGTTTTGGGGAACGCCAAAATCCTTACTGTTAAGCACCTGCCACTCAACA

TCAAACCCCAACTCATCAAGTGTGGTAAGAATTGTGGTGAACGTCCGTCCCTTATCGTGA

TTGAGTAGGCCTTTAACATTTTCAAGAAAAAGAAAACGTGGTTGGATTTGTTTGGCCGCC

CGAGCAATTTCAAAGAACAAAGTTCCTCTAGTATCTTCAAATCCCAATCGTCTTCCTGCG

ATTGAAAATGCTTGACAAGGGAATCCCCCACAGATGACATCGACTTCCCCTCTAAGTTTT

TTAAATTCGTCATCTGAAACATCTCGTATGTCATGAAATTCTATTTCTCCTTCCGTTTGA

AAAATGGACTTATAAGATTCTCTAGCAAATTTATCAATCTCACAAAATCCAATACACTCG

TGTCCAACACTTTCCATTCCAAGTCGAAAGCCACCGATACCTGAAAATAAATCAATAAAT

CTCATTTCTTTATCACCCCTTTCTTATCGACTATCATTGTCAGAACTGTCACTCCACTTG

AAATGACAATGAGCATAACTAATATCCACTCGATTGGCGACATTTAACCACCTCCTTTTC

TAACACGGTTATTCCAAACGGAATACAAGCCATTAAACACAAATTCAGCAAGGACTTCCG

CTCGTCTAACAAAGCGAACATTGTCAAGAGCATACTGATAGATTCTCTCAAAATCAGTCA

TAGCAATTTCACTGGCTGTTTCAGAAAAACCCTCTCGTCTAAATTGATCTTGTACCAATC

CCCAAATATAATCTCGATCATATTTAGTGACCTTTTCTACTTTTCTTTTCAAGATAAGTT

GAGTATTCCTATCCTCCTCATCCTCAATAAATAAAGAATCAGTCTCACTATATTTAGTCT

CACTAACTTCAGTCTCACTAGGGGCTGAATATGAGACGGGGCCCGTTTCATTTTCAACCT

GCCCTAGTCTTTTTTTACACTAGGCCTGTTTGAATTACCTACTGGGGTAGAAGATAATTC

CCCTAAATAAATCTTATTAGCTAGTCTTCCTTTCTCACTTGAAGACTGTTGAACTTCATC

AATTAAGTCATATTCTTTAAGTGTTTTTTTGATGGACAATAATTTGGACTTCGAACAACC

TAACAGCCTCATCAGTTTAGAATTAGAAAATACTAAATAGACCGCGCCCTCTTCATCTAT

CCAACCACGACTGAGAGATAATTCTAAACGATCTTTTAAAATAGAATAAGCCACCTTTAC

TTCTAGTTTCATATCCATATATTTCTCATCCTCAAAAAGAATTTTAGGTAATTTGTAATA

CCGTTCTGAAGTTTGGTATTGATTTGCGGTGATTCGTTTCATAGCGCTCCTCCAAGTTCT

TTGAGAATTAAATCTCCACCTGATTCCAAGCGAACCAAGCCACTTTTTTCCAATTCAGCC

ATAAGAGATATAGCCTCAACAATATCTATTCCCATTTCACGTACTAAAAATGAAATGACA

ATATAGCGTGATGATTGTAATTCTTTTGTCATTTTCCCCCCTGAAAATAAAAAACGGAAA

GATTGTGAAATTCCTTTCCGTAACATAATAAATATTTGAACACCAAACTCAACACCAAAA

AAATGTTTTTATTGATGATTATTGCTCAATCAAGCACATATAACAATGTGAAGAATTAAA

ATCTAAACCAATAAAAACCCTTGAATTTCAAGGGTTTTAAGCATTTATTCTACTTCACAA

TGTTTTATTATTTTAAATCTTCTAGACCAACCATTGAATAGTAGCCATTGAGTTTTTTCC

TTTCATAGCAAGGATTTAGATCCCCTATTTTATTTTACTATTGTTTGAACA

>ICESpnGA19101

AATTTTAAATTTTCCTGCACCTGTCGACGCGCTTTAGACATGTGACCAGGAAACCATTGA

ATAGTAGCCATTGAGTTTTTTTCCTTTCGTAGCAAGGGTTTAGAGCCCCTATTTTATTTT

ACTATTGTCTAAACACCAAGCGAACACCAAAACTACCATGCAATGGAAAAACCTCTGATT

TGATTCTCACTTGATTTCACAATCTTTATATCAAACTGTGGGTGGTATTTGACAATATCT

TTTTTGATTTTTAATAGTAAATTCGAAATAATATTTTTAGGTGAGTAACGTGGACTAAGA

TGTAACAAGTCTTTGAACTCATCGACACTTAATTCTACTTTATTGCTATTATCACTAGTT

TCAATGAATTTTTCAATTATTCTGGAATATTTACAGGTATAACTTTTCAATTCTTCAAAA

TGGAAATTGTGATTTTCTACAAATTGATTTAAGGCTTTTACAGTATTTTCTTGTGAACGA

TTTATATTATGTGTATAGCCCATTGTTGTCTCAAAGTTAGCGTGTCCTACTCTAGTCATA

ATATCTTTCACTGCTATGTGCATCTCATTACTTTGAAGGTAACTAATATGCATATGCCTA

AACGAATGGGGAGTAACATGTTTTACCCACTTAAAACCATAGTCACTTAAACAATTTGTC

AATAATTTTCCTTCTATTCGTTTCAAAATTTGACGAAAAGTGCTTGATGTTATTGGAGAG

CCGTATTCTGTTCTAAATACACTTTCAGAATGTGTAAAAGCAGGACAGGGATGTTTCTCC

ATATAAGCATCAAACTCTTTATTTCTCTGTATTGTCCTTTTAATAGCTTCGCTTGCAGCT

TCAGGCAAAGCTACTTCTCTAATTGAATTGAGTGTTTTAGTTGTATCAAAGTGAAATTGT

TCAACTTTTAAACAATGATATTGAAGTGCCTTATCAATATGCAAGATTCCTTTTTCAAAA

TCAATATCTGATGGTAAAAAAGCAGCTTCACTAATTCGAATACCTGTAAGCAACAATACT

ATAGCAAGATCATAATAGTTTGCATTTCTGCATTGGCTTAATACATCAAAAAATGCGTGT

AATTCATGGATTTCTAGAAATTTAGAATCATGTCTTTCTTTTGCTTTACGCCTTTTCTCT

AGTGAAATATCTAGTTTTACCGCAGTCATAGGAGAGAACTTAATGACATTATATAACACA

CCATGATTAAAAATCTTATTACAAGTACTTTTTATATGAGTCATTGTTGAAGGTGATGCA

TCATATATTTCTAAATATTTATTGAGACTTTTTTTCATCAGAAGTGGAGTAATCCTGTCT

AATAAAAAATCATCTCCTATAATTTTCCCAAGACGCTTCATCACCAGTAGTTCTCTCTGA

ATTGTTTGTGGTTTAACAGAGACACACCAAGTCTGAAACCAATTTTCTTTTAACTCTCCA

AATGTTGTAATCAGTTCAGGGCTAAACTGACTTTCAAATGAAGTAGTTAGTCTATCTATC

TTATCAAGAACCTCTCTTTCAGCTTGTTTCCTCGCCCTACTAGTATTCCTAGTGTAACTT

ACAGTTACTGATTTCCACTTTCCTGTTAGTGGATCTTTGTACTTTTCAACCACTTGATAT

AAGGGTTGCCCTTTTGAATTTGTTTTAGTTACATAATACATAATTTAATTCCTCTTAACT

CTTAAAAAGAAATAAATCATTGTTGTATAGAATAAATAACTACCCTATATTATACCATGA

CTTCCTTATTTTGTCCGCTTTTGCTTCCATCTTAAGAAACTTTCAAATCCTTCTAGATTA

ATGAAAACAAGTTTATGGGTTGGGTTAATTACATACATGGAAAATGTTCTGTTTTCTCGC

ATTTCTTTAATCCATCTGTTCAATGTATATTGATTTAAACCATCCCATCTTTTTAGAATT

CCTTTTTTATCGGCCCATTCTGCAATGGAATTTTCGACAGATATATATTTTATATTCATG

TCATCATCCTTCCTAAAAAATATTTTCTCCTCCATGGCATGACTGATGAAGTCATCATAA

GAATTTCACTTGCTGCTCTTTCACGGTGGCAGCCTGAACGGTCAGAAGTATCATTGTATA

TCATTTGTATCATGGCATATAAAGTATCGCTCTATTTTATTGATGGTTTTAATATCGCTC

TTATCATGGCGAGCCATTCAAAACTGCTCCACAAATGAGGAAAGTACCATTTTGGACTAT

TCAATTGTCAATGTGCTTTCTTAACCTAATACTTTTTGGAGTAATGGTCTTCTTTATTAT

CCACTCGACCTACAAGGTAATCTAAACTTACATTATAAAAATCTGCAAGTTTGATAAGGT

CATCTATAGAGATTAACCTGGTACCTGATTCCATTTTAGAATACGCTGATCTTGTACAAT

TTAAGATTGTTTTTGCAACATATTCTTGTGTCAAATCATCATCCTCGCGTAAATCTCTAA

TCCTTTTCAACATCCAATGTCTCCTAAAACAAGTATAATATAGTTATTTTCTTATTTTTA

AATATGTGACAAATTGGCACATGAAACTATTCTATATATACCTTTTAGGAAACTGTTTAC

TCTAAATGTATCTCATTTTCCTTAATAGATTTCATATTTTGTTTTTCCTTATATTTCTTT

ATTGTTTTATTAAACATTTCAAATAAATTTATTGCTTCTCTATGTTGTTCCTTCAGTGTT

TCAATCTTTCCTTTATATATGTATAAATCCTTCTCTATTGTATCTACTGAACTTTTACCT

TGAATACTATTTTCTAAATTGCTAGAACTATTTGTCGTATCTTTCAACAATACTTCCTCT

AAATTAGTTAAATCATTAATTTTATTTTGGACTTGTTCAATCATATTCTCTAGATATGAT

ATTTGGGCAATGAAATCATTTGTTATATCTTCAAAATCATTCGAACTATTTTCCGCAGAT

ATAAGAAAATCTAATTGTTCTATTTTTTCTTTAATTTTGCTAAGAGGAATTCTTCTATAC

ATATACTGTGGTTCATACTGAAGATTAAATTGTCTAATCAAAGTTTTACCTTTCATAAAT

CGATTTTTTTCTACTGAATCTTTATGATATACATAGTAAGAACTAGTTTCTCTGAGAAAT

ACTTTAACTTTTTCTTCTTCCATATTGATTTGAATATTTGGTACAAAAATAAGTCCTTCT

TGTCGGATGCCAAAATGTACCTTAATGTACATTCCATCGTCTACTACTTCTTCTATTTGA

TTAAGATTCAACTCTACCTCAAACTCATGAACTGCATCCCTATTTCTCTTGAAATCTTGA

TAGGATTTCCATACCACCTCTTCTGTCGGCAACTCTTTTTCTTTAATTAGCTTTTCTTCA

TTGTAAAGTTCAACTAAATTTTTATTATCTAAGACAACAGTTTCATTTTTGTTATTAAAA

TAGTCTTGAAAATAACTAACACTATACCGATTCGTTTTTACCAATTCCTGCTCTGAAATC

TTAATCTCATCAAATTTAAATTGAACATGTTTTTCTTTCGGAATTATTTTTAATCCAAAA

AATTCTGCTCGTTGAATCAATTCATTCATATTCTTCATTTTCGGAAGTAAAAATTCTAAG

ATGTTTATGATTTCCCTTTGAACAAACTTTTTCTTAAAATAAGTTTCATTATAAGGTTGT

TTTCTACTCAATTTACTATCACGTACGACTTGTTTCATATTTGAATCAGTCATAAAAAAA

GTAACATGCTTGTGTCTAAAATCAATTTTTAAATGTAAAGCTTTTGCTTTATTCTTGAAA

TCTTCAAAATTTTTCGAGTTCTCGATTAGAAAATATACTCGTTGTTTTATTTCATATTTG

TAATTTGTTTTGCGATAAACATCATACTGACGATGCGAATAACGATTTTCTATAATTTTT

GCCCCTGCAATTTTTGAAAGACGATCAGAAACCATTCGTAGATTATGTTCTGCCTTATAA

TCCCATAGAAACTTTTTATCAGAATTCTGATCAATTGAATTTAGGATGATGTGATTGTGG

ATATGATCTTTATCGACATGAGTTGCCACGATAAATCGAAATCTACCTCCTGTCAACTCT

TTAACTGTCTCATAACCAATCCGATTGATTTGTTCAGGAGTGAGATGGTCATCTGGAGAA

AAGGACTGAATGATGTGATGAGAATGAATTTTTCGTTGATTTACTTCTTGCCTATCATGA

CGAAATTCATAAAGAGTATCATTACTTAAAAAATTATCATTGTACATCTTCACTAGTTCT

TTATAACTAGGAAAATCTAAATAATTTCTCATACCAAAATCTGAAACTAGTGTTAGATTT

TTTGTTTTACTTGGATTCAAAATATATTTGATTAGTTTACTACGATAATTTTTTCCATGA

ATCGCAAAGTGTTTAGTGATGACCATAAAATTTCCTCAATTTTTCAGATCGAATAAGAAA

ATCTTTCTCCACTTCTACTATTAACTCTTCTATACCTTTTCTCAACTCATTTAATTCAAC

TTCCGTTATTAAATTCGAATAATTTATGCTTCTGGCTATTTGATTAATATTATTTCCGAT

TCGTTTTAATTCAAAAATCAAATCTTGATAACTATTTGTATCAATGGTGATAAAATTCAT

ACCAGGATCTAGTAGAGTTCGTCTAGCATATTCTGAGAATGATAAGCAGTTACTTTTTGA

GATATTTTCATTCAATTTGACTAATTCTAGATCAGATAGAAAGACTTTTTTTAAATTTGT

TCTATATCTGTGCTCCACTCTACTACCTCATGTATTTATTACAAAAATCTTCACTAAGAG

GACCTGTTTTTTCTACCTCTTTAATTAATTCCTGAATACAAGTTAACAAAATAGAAACAT

GTTCGTGAGTAACCTGATGATTTGTTTTAGCAATTATGAATACTTCATGAACATCACGAC

TAATTTGTTCCAACTTTTGTTTTTTCCAAAGGTTGAACCATTTTTCCATCTGTTTTTGTC

CATCAGATAATAGTAAGCTTTTACGGAGAAAATCAGAAAAATTATCATCTCCTTTCTCTC

TCATTAAATCTAGTATTTGTTTTTCTTCCGTTTCTGTTAAGCGAAATTGTTTCCGAATAC

TACGTATATTTCTTTTCATATGATTTATCCTCTCTCATAGATACTTGTACTGACAATGTA

AGCTTACATACACTGTCAGTACGTTTTACAAAATAGATTCAACATTTTTGTATGGCTTTG

CGAGCTCAGATATTGTGTCCACAATATCCCAAAAATCATATCGCCTGCCATTTCAAACCT

CACGGTTCAAAAAACAAACGATATGAAAATGTGGTGGCACGCCCCCAAACCCCCAAGATA

AATCTAAATTAAAAAAAGTATAACAAATACTTTTTGATAAAATTATCACAGAAAAAAGAG

CCCTGTCGGACTCTTACAAATTAAAACAATGTCTCTGATATTCATACTGTCTAATACCAC

TACTGTCTTTGTTCATCTTGATAGAAAAAACAAAGTAATGATCACGACCGTCTAAAGATT

TACTTTTGTCAAGTTTAAAATAATGTTTTTGAGACATAGCCATTGTGCGTAAAATATCAT

CGGTAATAAATGTTAATGGTATATCTAAAGCTGAATACTTGTAAAAATCTGACTCAAACT

GTAAATAAGAATCTGAAAAATAATCTAGCTGTAATTTATCATCATATAATTCTTTACGAT

TCATGTAATTCCTCCCCTTCTATCTGTATATCGAGTATATCTTCCCATTTTAATCGAAGA

AAACCAGTGTCTGATTTTATAGATATAAACTCTTTTCCAATCTCTTTAACAATACCTGAT

ACTACTTTTCTTTGATTAGAAAACTTAAAAACAAAAGTTGCAGGGAATACATTCGTATAA

AGTTGACGAACAAATAGAACTTTCTCTTCCAATGATAGGGAAATGGAGATATCTTCTTTA

TTTTTTTCTGCCCAAAGAGAACTGGAATGTTCTGATAAAAAGAAGCCCATCCACTTAGCC

ATGCCACGATCCTGATATACTCGGGCAGACTCAAAAGGTAAATAAGAACGATTCATTATT

TTAGTCCATCTAATCCTCCAGCTGAATGACCACCAATGAGTTTGCTTCTAGCAATTGTTC

TAGAAGCTTGATCTAGGGCATTACCTTTCAATAGTGAAGTGAACCCAAATTCTGTTCGAA

TAGCATCGATTGCGGACTGGAGCCTTTCTTCTTTTTCTATTTTCTCAATATCATCAAATA

ATGAAATCAATCCAAAGGATTCATCCACTAAACCAGAATAGTTAACCGCAACATTCCTGA

TAGCTCCTGAAGTATATTTAGTGTTAAATAACTTTAAAACGTAATTTGTCAGTAATGCTG

TTTGATTGGTCGGTTCAATCTTCATTTGAGTATGAATAGATTGTTTCTGTTCTACTTTAG

AATATCCTAAGTGTATAGAAACCACAGTCGCTTTTTTACCAGCTCTTCTCAATCTAACTG

CAACTTGTTCTGCCATTTCACGAAGTATAATTTCAATATCTCTTTGTTTGACATAATCTC

TAGGTAAAACTTGAGAGTTCCCTATCCCTTTTGACTTCGGCTTATAAGATTTATGAACAT

TGCTTTCATCAATACCATTAGCGTGAAACCACAACTCTAGTCCCATAATCCCAAGCTCTT

TTTTAATCAAGTCAGGATTAGCCTGTGCCAATTCTTTAATCGAAAAGATACTTAGATTAT

GTAATCTCTTCTCCATTCTATTTCCAATTCCCCAGAAATCTGTCATTTTAGGAATAGCCC

ATACTTTCTTTTCTATATCCTCATAGGACCAATTAGCTCTCATTGTCGGAGTCTTTTTAG

CTTCATTATCAAGTGCCAACTTAGCTAATAAGGGATTGGCGTTAGACATACCTACCGTTG

AATAGATTCCTGTTTTTCTCCAAATCTTTTTTTGAATAGCAGCCGAGATAATATCTAATT

TATCTTTCCGACTAATACTTTTATCTGGCACAAAATAATTTAATGAACTTGTTAAATCAA

TAAATCCTTCATCAATTGAATAAGGATAAATATCGTCTGGTGCCGCAAAATCTTGAAAGA

TTTTTTGAATTTCCATATTGACTGCTATATAAGTATCCATTCTCGGAGGAACAATCACAG

TTGATTTTGCCCATTCCTCAATAAAACGCACATAATCTATCGTTGTCGGTAAACCTTGCT

TCTTAGCGTTATAGTAAGAGAACTTCCTAGTTTTTACATCAAATGGTAAATCATAGGAAC

GTCCAACGTTTGATTTTCCAAAGACCTTTTTAAACATAGGAGAGGAAGCAAGGATTAACC

CGGCAGAATTATCTGCACGACTCATAACACAAAGCGAAGTCTTAAGTGGATGTAAGCCTC

TATCGACACACTCTACACTTGCGTAAAAAGATTTCATATCAATAAATGCAACGTCACTCT

TTGGTTCGAGACTATAATCAAACCAAGTCATACTCAATCCTCTAAAGGTATAAAGTTTCC

AACGATTTTCCCTATGATACGAGGATTCTCATCATAAGGCGCAAACTTATCTGAATAGTT

CCGATTGAGTGAAACTAATCGAAGCCCATTTTCTTCACGATACACTTTCTTAATATAGGT

TTGACCATCCCAATCTATCGCATAGATAGCCCCGTCATAATCAAATCCCGTTTGCTTAAT

AAGGGCTACCGAACCATTTTCATATGTCGGTTCCATTGAATTACCAAACACCCACGATGC

AAAATCATAATCAAAGTGATGATCAAAGTAAACTGTATCGTAATTACCATCATCAAAATA

AGCTGTTCCTGTACCAGCTGATAATTTTTCGTAGACCTTATAGGCAAAAAGCTGAGGAAT

GTCTACAACCTTTGCATTCTGTTTGTTCAACAATTCTGTAGCATAATGAAGTGTTGCTTC

TTGATTCCTTTCCGTTAGTTTGAGATAGATTTCAACTATTTCATACTCTGATTCAAAATA

TCGAAGATCTACATTCAAAATCTCACTCAATTTACTTAAATTGTTTTGATTAGGCTTTGT

TTTACCTGATTCCCAATTAAAGTATGAGGCTCTACTAATTCCCAAACTGGATGCAAGTTG

AGATTGAGATAATCCTTGACTTTCTCTTTTTCTTTTTAATTTAGTAGGTGAAAACATTTA

TTCTCCTTTTGTAAGTTTATTAACTAACAAAAGGATACCATAGCTTGTTGAAAAAATCAA

GAGTTTAATAACCCTCAAGATTTAAAATAATCATAAACTGCGCTAAATTCGTACTTTTAT

TGTTAGGAAATCATCACTGAAGAATGCTAAAACTTTTGAAAAAATATGAAGAAGAAAATA

GAGAATATAAAAAAGAATCGTTATTTTTTAATTAACGATCCAATAGACTTTATTTTAAAA

ATCTAACTCTACAAACCATTTCAATTAGTTAAATCTTTCAATCTTTCTTCTCCAGATTTA

AGCATCTCTTTCTCCACTTGATTCCATTCTCCAAATAGTAAATAATGAAGAACGGTTGCT

GCTGAAGTTGTATTTTCTTTTGAATCATATACACAACTTCTATCTCGTTGGTAAATTTGA

ATTCTTTCAAAGATAGCTAGTTCTTCCAATTGCCGTGTATTATCAACTAGATGATTTACA

ATGAAATCATGATGTTCTTTTGGAGTTGCGCGTGCTTGATTTGGATTGATAGCGTATAGT

TCTTCATATCGGATAAGGGTGCTCAGATAGGACAGCTTAGGCTTTGTCGCAATCAAGGCT

AATTGTACTTCATATCCCCTACTTTTCAAGAGTTGTGCTGTTTTCTTTGGAACATCAATC

GTTCGTAAAGTTCCCTCTATCAAAAGATTGTATCCCAAATGACTCAATTCTGTTACTAAA

GACTCTACCATTTTCCCTGCAAAATCTTTGGTGTATTCTACACTATCTTTGCCATATTCT

TGCTGCAGTCCTGAATAGTGTGGATGCTGAGAACGAAAACTATCACCATCTATGATAACA

ATATTTCCTTGAAATTCTTTCTGTTTAATACGATGAATTGTAGTCTTACCAGCACCACTT

TGCCCTCCAAGCAAAATTGCTATAGGTTGCTTACTGGACTTTTTTCCTCTTGTCAGTGAA

CGAAGATTCCGTGCTAGAGCATGTTTGAATTCACTGTCAGTATAATCTTGGATTTCCACT

AGGCTACCATCCGTTTTTCAGATATCTCTAACATACGTTCAATTCCATCCAAATAGCCGC

TATATCTCTCTATTTCATCAAAAGTTTCTACTAAATAGATATTCGTATGAATCAAGTCAG

ATAGATCATCACTCATTAAAATCCAAGGATTAGATTCATCATCAATGCTAATTCCCTGAC

TATCTTGATAACGATAGAGTCGAGATAAAAGATTAGCACCTCTTTCTTTCACAATTTCAA

TTTTTAAAGTCAATTCATAGTCTTCAACAGGATTGAGCATTTTATCTTCTCCTACAATAT

CGACATAAGATACATTAAACTTCTGACAAATGATGTCTATTAATTCGGTAGAGACTGAAC

TCGTTCCATTTTCATAACGACTCAAGCTATTTCGAGAAATTCCTATAATTCGTGCAAATT

CGGGTTGTGTTAAGTCATGTGTTTTACGTAAGGATTTTATGTTCTTTCCAATCATGGCAA

ACTCCTTTATTTTTATAATTCAATTATAGCATAAAAAAGTATAACGCACCAATTTTGGTG

CGTTATACTTTTTTTCTAGCCTAAACTTTACTTCTAAAAAAGCCACCTAAAGAGCCATAA

ATATTTATACTTGTATTATTAGGCCATTAGTAAAGCGTATCCAACATCTCAATAAAGTGT

TGTTTTTACTGATATTTTAACTATATTGTATAGTCTACTATTTAAATCTCAATTGGGATA

TTTTATAGAATACCCAAAATTCAACAATAACTAAAAGTAAGATTCCATTGTATTTCCTTG

TTGATCTTTCATATCACGAACTTTATTTTTCAGAAGATCAAAGTTACAAATTTCATCTCT

AATATCGTTATCCGCTCTGTAGCCATTAATCATCTCAGTTAGAGTTTTCCCTTGACTACC

ATTCTTTATAGTGGTATTCACTCTTTTTATCTTGATATCAAAAGGCTCATATGTTTTATA

CTTAATTACTTCGTCTTTAAAATCAAATAAAGGTGTATCCGGTTTAGCATCCCATAAGAT

AATCAACCACATTATCGCATTATAAAGCTGTTTTGCAGTTTTATTCCACTCTTCAGGTTG

AAAATTTCCACTCCCTCTATTGTAATGTTCTTGTCTATTATTTACATGAGAATTACCTAC

AAGCGGATTAAAAAGAGGTAGCCCTTTGTGATTAATTAGTTCTAAAAAATCCCTTGCCGC

ACCCATGCCACACTGAATAACTTCCTTCTTGCCATCAATTTTACTCTCAGCGATAAATAC

ATAATATTCATCTTCAATAGTTGCTCCTGCATCACTATGTTTTTGTTGATTTGCTAACAG

CTTAATATGTGCTTTAGGCTGAACTTCAAAATCTCTAACAATTCTTTGTCTTGTTTCATG

TCCTCTACAATTCATTTAAGTTCTCCTTTACAAGTTTAGTAACTAGCCTTATTATACTAT

ACAAGTTGAGTAAAAAATCATAACCTTCATCAACTAGTTTTTGAAAATCACAAATCCTTT

TGGAGAGATGACAAGCTGATTCTCTAATTCTTGGCAATTGCTTGCTAGTGCTACTGTTTC

CTTCTCTAAAAGATAATCTTCTTTGGACTGGTTAAAGATAGCTTTAAGAATTTGTCCATC

GCATTTCCATTCCAATGCTACTACATCTTGTTTAATTTCTTGGAGACTATACTTGCCATG

CTGAATGATTGCTGACGCGGGTTTCCGAATCTCAATCAGCTTCTTCATAAAGTTCAGCAT

ATCATTATCACTTGAGACGCGTTCCCAAGGCATACAACGACGGCAATCTGGATCTGGACC

TCCAGTTAAGGATAACTCTGTTCCGTAATAGATACACGGTGTTCCTTTTTGTAGAAAGAG

AAAGGCTAAGGCTGATTTGACCAGTTGAACATCCTCATTGGCCGTCCACAAGATTCGCTC

TGTATCATGCGAATCCAAGAGATTAAACATAACTTCTGAAATCTGCTGCTTGTAATACAT

AGATTGGTCATTGATTTCATCGATGAACTGGTCTGTCTTCTTAACTCCTCGTAAGAAATA

GTCCTTGATGCTATCAGATAAAGGATAATTCATGACCACGTGGAACTCATCTCCATTTAG

CCAAGGCTGAGACGTATGCCAGACTTCTCCTAAAATATAAAGATCAGGCTTTTTAGCTAG

AACTGTCTTGCGAAAATCCCTCCAAAACTGATGGTCAATCTCATTAGCCACATCCAAACG

CCAAGCATCGATATCAAACTCTTCAATCCAATAAGTCGCAACCTTTAAAAGATAATCCTT

GACCTCTGGATTAGCTGTATTTAATAATAGGGGTAACTATTGCCGGCGAGGCTAGTTACC

CTTAAGTTATTGGTATGACTGGTTTTAAGCGCAAAAAAAGTTGCTTTTTCGTACCTATTA

ATGTATCGTTTTAAATGACTAGTAAAAAACATACATAGAAAGGGGAAAAGCAACTACTTT

TTTTATTGTCATAGTTTGTGAAAACTAAGTTGTTTTTATGTGTTATAACATGGAAAAGTA

TACTGAGAAAAAACAAAGAAATCAAGTATTTCAGAAATTTATTAAACGTCATATTGGAGA

GAATCAAATGGATTTAGTTGAAGATTGCAATACATTTCTGTCTTTTGTAGCTGATAAAAC

TTTAGAAAAACAGAAATTATATAAAGCTAATTCTTGTAAAAATCGATTTTGTCCTGTCTG

TGCTTGGAGAAAAGCTAGAAAAGATGCATTGGGTTTATCTTTGATGATGCAATATATTAA

GCAGCAAGAGAAAAAGGAGTTTATCTTTTTAACTTTGACTACACCTAATGTAATGAGTGA

TGAATTAGAAAATGAAATAAAACGTTATAATAATTCTTTTAGAAAACTTATAAAGAGAAA

AAAGTAGGTAGTGTTATAAAGGGATATGTTCGTAAGTTAGAGATTACATATAATAAAAAA

AGAGATGATTATAATCCTCATTTTCATGTGTTAATTGCAGTAAATAAATCGTATTTCACA

GATAAAAGATATTATATTAGCCAACAAGAATGGTTAGATTTATGGCGTGATGTAACGGGC

ATTTCAGAAATAACACAAGTTCAAGTTCAAAAAATAAGACAAAATAATAATAAAGAATTA

TATGAAATGGCTAAGTATTCTGGTAAAGATAGTGATTATTTAATAAATCAAAAAGTCTTT

GATGCATTTTATAAATCACTTAAAGGTAAACAGGTATTAGTTTATTCAGGATTATTTAAA

GAGGCTAAAAAGAAATTAAAAAATGGGGATTTAGATTACTTAAAAGAAATTGATCCAACC

GAATATATCTATCAAATTTTTTATATTTGGAAACAAAAAGAGTATTTAGCTAGTGAACTT

TATGACTTAACAGAACAAGAAAAAAGAGAAATTAATCACAAAGTGATAGACGAAATCGAG

GAAGAACAATAACAAAATATAAGTGCTAACAGCTGACCTCCCGATAACACCATGTAGTTA

TTGGGAGGTCAGCTGTTGAATTATGCACGAGTATTTTAAAAGTTATTGTGATGACGACGA

TAAACGATTATCAAAAGTATAATGTTAAAATGCTTTATTATACTAACGTTATATAAACAT

TATACTTTCGTTATACAAATTTTAACCCTGTTAGGAACTATAAAAAATCATGAAAATTTT

AATTTGCATGTAACTGGGCAGTGTCTTAAAAAATCGACACTGAATTTGCTCAAATTTTTG

TTTGTAGAATTAGAATATATTTATTTGGCTCATATTTGCTTTTTAAAAGCTTCTGTAGGT

TTTTAGGCATAAAACTATATGATTTACCCCTAAATCTTTAAAATGCCCCTTAAAATTCAA

AATAAAGGCATTTAAAATTTAAATATTTCTTGTGATAAAGTTTGTTAAAAAGGAGTGGTT

TTATGACTGTTATGTGGTTATCGATTATAGGTATGTGGTTTTGTATTGGAATGGCATTTT

TTGCTATCAAGGTTATTAAAAATAAAAATTAGACCACGCATTTATGCCGAGAAAATTTAT

TGTGCGTTGAGAAGAACCCTTAACTAAACTTGCAGACGAATGTCGGCATAGCGTGAGCTA

TTAAGCCGACCATTCGACAAGTTTTGGGATTGTTAAGGGTTCCGAGGCTCAACGTCAATA

AAGCAATTGGAATAAAGAAGCGAAAAAGGAGAAGTCGGTTCAGAAAAAGAAGGATATGGA

TCTGGAGCTGTAATATAAAAACCTTCTTCAACTAACGGGGCAGGTTAGTGACATTAGAAA

ACCGACTGTAAAAAGTACAGTCGGCATTATCTCATATTATAAAAGCCAGTCATTAGGCCT

ATCTGACAATTCCTGAATAGAGTTCATAAACAATCCTGCATGATAACCATCACAAACAGA

ATGATGTACCTGTAAAGATAGCGGTAAATATATTGAATTACCTTTATTAATGAATTTTCC

TGCTGTAATAATGGGTAGAAGGTAATTACTATTATTATTGATATTTAAGTTAAACCCAGT

AAATGAAGTCCATGGAATAATAGAAAGAGAAAAAGCATTTTCAGGTATAGGTGTTTTGGG

AAACAATTTCCCCGAACCATTATATTTCTCTACATCAGAAAGGTATAAATCATAAAACTC

TTTGAAGTCATTCTTTACAGGAGTCCAAATACCAGAGAATGTTTTAGATACACCATCAAA

AATTGTATAAAGTGGCTCTAACTTATCCCAATAACCTAACTCTCCGTCGCTATTGTAACC

AGTTCTAAAAGCTGTATTTGAGTTTATCACCCTTGTCACTAAGAAAATAAATGCAGGGTA

AAATTTATATCCTTCTTGTTTTATGTTTCGGTATAAAACACTAATATCAATTTCTGTGGT

TATACTAAAAGTCGTTTGTTGGTTCAAATAATGATTAAATATCTCTTTTCTCTTCCAATT

GTCTAAATCAATTTTATTAAAGTTCATTTGATATGCCTCCTAAATTTTTATCTAAAGTGA

ATTTAGGAGGCTTACTTGTCTGCTTTCTTCATTAGAATCAATCCTTTTTTAAAAGTCAAT

ATTACTGTAACATAAATATATATATTTTAAAAATATCCCACTTTATCCAATTTTCGTTTG

TTGAACTAATGGGTGCTTTAAATGATTACAGGTATAATTTTGCCCTTATGTTTTCTAATA

ATTTGAATTTTTTTACTAATTTGTATGTTTTCTTCTTGATTTATCTCTGTAAGCGCTTCA

TCGATATGTGATATTAGGCGAGTTCCCTCTCCTTTACCAATATAGAAAGGTAGTTGCTTA

CCTTCTTCATAGAGTCCGTAAACATAGTATTTATCTTTTTCATTTTTGGTAGCACCAGTA

ATATCATTTAATTTTTGATAATTCATTTTTTTACTCCTATTTAAATATATTATAAAAGTT

CTGCTCCATACTATTAAAAATTAATTAAAATAATCCTCTTATTCTACCTTCATTCTGATT

CTGAACTATTTATATGTTAAACTATATTTTCTTTAAAATCAAGTAAAATCCTAGAGAATA

ATCTAAATAAATTTGGGGACAATATATAGGTTTACTAATAACTACACAATACAGTATTTA

CGATAGCATTAAAAAGAGACTGTTTTATTTATGCTCTAGCTTTTTCAATGAATCTTCTAA

TACTTTTTCAACCTCTTTTTTAGCAGAATATCGTTTGACTTTTAATAAATTCTCAGGTTT

TAAAGAAGGATTACTAGGATTACTCGTTTCATAACAATTTTTATACTCTTTTAATTGAGG

AATTGCATCGTACCACTTATTGTAAGATCGTGTGATGATAATGATTACAGGATCATTGTC

ATCCAAAACACGTTCTCTAATTAAATTGAATAAAAACTTTTGTGTTGGGAAATAACCTTC

TTTGCTTATAAAATCATTTAATTTATCATCGTACTTAGTAGAGTAATATGGAAAATATTC

AGCTAAAGCAACGTGTTTTGAAAACCAAGGAAGCTTTTCTTTTAAATCCTGTAAATTGTC

TACATCATCAAACCATACTTTAAACTTTTTAGCCCAGTAACCAAATTCATTCGTATCTAG

GTCAAAAGCATGAAAATAGAGGTTTCCTTTTAGCTGTAGATTATTTAAAAGAGTCTTTTG

GTAGTTTTTATCGTGGTCATAAAGTGTTTTAAATTCAGGGGTGTAGCCAGGATTTTTAGA

TAAAACAATGATATCAGCATTTTGAATATCCCCCACAAATTGTTGCGGACAAAAACCTAA

TTGTAAACGGTAGATATCATCTTTTAGATTTTTGTAGTGTTTTGTAGAGGTGTATTTTTT

GATTAGTTCAACATCTTCGGTAGCAAGAATAGCTTGCTCTCCATTTATATCGACGTTAGA

TAATTGATTCCAGGGATTTTTAGTTGTAGTCATTTTATTTTTCCTTTCTTTTTGTAGAAT

TACTATATTTTACGATGTTTTAATATTAAATACAAAAATAGATTTTATTAATATATTCTC

GATTATAAAAGAATCTGAGTGAGGAAACGATTTTGAATATCAATAGATATAATATTATTT

ATATCACCTTTCACACCGCCGTAAATGATGTTCGGAATACGTCGGCTCTACTAAACTGTA

ACGAATTCTCATTGCCAAGTCTACAAATTATTTTCAATACTATAGGCCAGATAGAATTAA

CTAAAAAGTATAAGACTAGTACAAAAAGTCCGATAAATTGGAAATATTTTGAGAACAACG

ATTAATTTAAAAAAGAATGACGGTGAGATTTATTACTTTTATTTATACTCACTTTGTTAG

TTTAATTAAAAGCCATTTTTTTACCATAATCTAAGAGATAGCAATTTTTAATAGGCTTTA

TTTTAAAACTCAAGATCTAACAACCATTTCAATTAGTTAAATCTTTCAATCTTTCTTCTC

CAGATTTAAGCATATCCTTCTCTACTTGACTCCACTCCCCAAACAGTAACTCTTGAAGAA

CATCTGCCGCTGAAGTTGTATTTTCTTTTGCATCATATACACAACCTCTATCTCGTTGGT

AAATTTGAATTCTTTCAAAGATAGCTAGTTCTTCCAATTGTCGTGTATTATCAACTAGAT

GATTTACAATGAAATCATGATGTTCTTTTGGAGTTGCGCGTGCTTGATTTGGGTTAATAG

CGTACAGTTCTTCGTATCGGATAAGGGTGCTCAGATAGGACAGCTTAGGCTTTGTCGCAA

TCAAGGCTAATTGTATTTCATATCCCCTACTTTTCAAGAGTTGTGCTGTTTTCTTTGGAA

CATCAATAGTTCGTAAAGTTCCCTCTATCAAAAGATTGTATCCCAAATGACTCAATTCTC

TTACTAAAGACTCTACCATTTTACCTGCAAAATATTTGGTGTATTCAACACTGTCTTTGC

CATATTCTTGCTGAAGTTCTAAATAGTGTGGATGCTGAGAACGAAAACTATCTCCATCTA

TGATAACAATATTTCCTTGAAATTCTTTCTGTTTAATACGATGAATTGTAGTCTTACCGG

CACCACTTTGCCCTCCAAGCAAAATCGCTATAGGTTGCTTACTGGACTTTTTTCCTCTTG

TCAGTGAACGAAGATTCCGTGCTAGAGCATGTTTGAATTCACTATCAGTATAATCTTGGA

TATCCATTAGGCTACCATCCGTTTTTCAGATATCTCTAACATACGTTCAATTCCATCCAA

ATAGCCGCTATATCTCTCTATTTCATCAAAAGTTTCTACTAGATAGATATTCGTATGAAT

CAAATCAGATAAATCATCACTCATTAAAATCCAAGGATTAGATTCATCATCAATGCTAAT

TCCCCGATTATCTTGATAACGATAGAGTCGAGATAATAGATTCGCACCTCTTTCTTTCAC

AATTTCAATTTTTAAAGTTAATTCATAATCTTCAACAGGATTGAGCATTTTATCTTCTCC

TACAATATCGACATAAGATACATTAAACTTCTGGCAAATGATGTCTATTAGTTCCGTAGA

AACTGAACTCGTTCCATTTTCATATCGACTCAGACTATTACGTGAAATACCTACAATCCG

TGCAAATTCGGGTTGGGTTAAGTCATGTGTTTTACGTAAGGATTTTATGTTCTTTCCAAT

CATAGCAAACTCCTTTATTTTTATAATTCCATTATAGCATAAAAAAGCATAACGCACCAA

AATTGGTGCGTTATTGACTGTTTTTCCTAAACCTCTCTAAATTCTTCTCCAATTGTTCCT

TATGAAGTTGATTCTTAGCTTCCTTTAATGTGTCGTCCAAAGTTTCTTCTCTGTAAGTTG

ATTCTGTTTTTAGTTGTTCTGATCCTTGATCTGGAGTGAAAATGATATCTAGCAGTCGGT

CTATTTTTTCAAGATCTTGGGAAGACATATCAGATAGTCTATGAATCAGCTTTTTAAATA

TATCACTATTGTCGTGATTTTTCTTAAAAAATGTTGTAAACATCTATAACCTCTCAAAAA

AAGCAGCCTATCAGGACTGCTTAGTGTAATTCGGAAATCGCATCATAAATGGACTGCAAT

TTCTTATTGTCTTTATTCTTTGTGTCAATCAACGTATGTAGCTCTTCAATTTTCTTTTGA

CTACTTTGAATATCGCTATTGTTATCTGCAATCAGTCTTTTAAGATGTTGCTGATAACTT

GTAGTGATATCTGTCTCTTTTCCTGTTCTAACCTCTGTAACTTTGGGTTTAGAACTAGAA

TTTGATTCCGTCACAGGTGCTTGTTTTCGCTTTTGAAAAGCTGCTTCATAATTAACATCA

TTACCTCCTTGATGATTTCTAAATAAAGCCGCAATTTTATCTGGATCTTCTTGATTTTCT

GATTTACTTTCTAATGGTTGATTAAAATTCCAATCCTTTGTCATTCACTCATTTCATCCT

TTCTAAATTCAGAATCATCGAATTGTCTTTCTTTACCAAAGGCATGGTCAAGAGCTTCTT

CAAAACTCATGTGACTAATGCTTTGACGCCTTTTGAACGTCGCAAACATCGCTGTCTCCA

GTTGTTCTTTGTAAGCAGCGAGTTTTTCTGTCTCTCTTGCTACCTTACTTTCTTGCCTAG

TGACCTTTTCTTCTAAGCGTTCAATAATGGTCATATACGATCCTCCTTCATTTTAATATT

AGCTAAAACATCTTGATTTTGTTATCACAATTCTAAACATTGAGAGATTTGTCTTACTGG

TGGATTTCCAAATGTCTCTTTTGCAGTCTCAAGCATAAATTGTAAATCTGATTTCTTTTC

TCTAAGAATATCATTCCAGTCTATTTTTTCTTTCCCAGATTCATTATCGGGTAAATCCAG

TAAAACGGGAAATCCTGATTGAGATAACTTATCAGAAAAATCTTTACCTGCATCATCACA

ATCTACCGCCAGTGTCAATAAATCAGGATGATTATCGAAATAGGTAGTGGTATCACGAAT

TGTATTCATCAAAGGCAATAACTTTGAAGGTATTACTGTATCTAAAAATTCCAATTTCTG

ATTTTCTTCAGCTATCAGTCGTAAAGTTTGATAAGCAACAACAGACCTTTTTAATCCCTC

CATAGATACCAAGCGAACATCAAATAGACTTTGTTGATGAAGTTCGTAATAGCTCATCAA

GTCGATGAACGATTCACAAAAGACTAGTCTATTTGGTTTACCAATGTCAAAGGATATTCC

AACATGTCCATGGCTTCCTTTTAGAATCGTTTTTAACCTCTCTCTAGGAAGAGAGTGATT

CTTATATAACCCTTGTAAGCTTGCTGCCTGCAGTTTGTGTCGATGATCAAAGCTTTTAAA

AACAATAACAGGTTCAACAGTTTCATTTGTTTTCCAACTGGCTTGTGACATCAAACCTTG

TTGAATCATCTTTTGTATGATTTCTTCTGAAATTCCTCTACATTCTGTTAAATAATATCT

GGCCAGACTACAGTTAGAATCTTCTATTCTCTTTAAAGGGTAATAAAATGGTCTCTCTCT

TTTTTCTTGAACAGCTTCTTTTTGAAAAGGTTCTTCAGAAAGAAAGGCTAGAGCTTCTTT

AAAGGAAATTCCCTTAACAAGTCGAACAAAATCAATGACATCACCTTGAATATCTCTTGA

AAACCATTTAAAAGTATTGGTAGTTGAAAAAATCCGAAATGAATCGTGTTCAGGATGTTC

ATAGACACTGCTCGAAACTTGTTTAAAGGAGATCCCCAAACGATTGGCTACATCAAGAAT

TGAAATTTGCTTACATTCTTCTATTTCCATGCAATATCATCATTTTGTTGTAGTATTTGG

CAGTGATGGAACAGATGAGGATTCTTCCAAAGTTTTGGGAGTGGCCTTATCCAAATCATC

TAACCCAGATTTCCAAACCTGTACTTGATTGACCAATAACTTACCTGGTAGTTTCGAATA

AGACAACTTAACCGTTCTGGTTTCTGTCTGATTGGTCTTTGATTGGTTGGCATTCTTTAA

ATCAGATACATAGGTTACATTATAAGAGACCATGGCAATGGCTTGATTCGTAGTCTGATT

GACAAAGATATCTGCTTTTTCAAAATGATAATCCAAAATATAGTCCTTATAAACTTGGTT

CATGGCATCATTTTGACTTGACAATTCTTGAGAATAAGCCGATTCAGTCATATAAGGTTG

AATACGTGTATTATTTTCTCCAAGCTTTTCTTTCGTATAGTACTGCGTCAAAAATTCTTT

TACAGTATCCGATGACAAAATGCTGGCTTTATCTTCTGCTTGTTTGTCCTCTACAAGTTT

AGCTGCAGCCAATTCGATTTCTTTGCGACTTTGTTTAGCAGTAGAATGTTGACCAGCAGT

ATATCCCATCATGAGAATAAAGCTAGTTGCGGCTACTGCTCCAACACTAATTAAGGCTTT

AGTTTTGACTTTATTTAACATCTTAGACCTTCTTTCTATAAAAACTAGGTCAAGAATAAC

AAAAAGAACTTGTAAGTTTTATCACACCACAACGAAAGGTAATTCCTACATTTCAGGCGC

AAAATCGTACAGTTCAGAGAAAAATATCAAAATTAGCTGTCTTCAGTTTATAATATAAAT

ATGAACAAATTAAAAGAAGAAAATTTGAGAAGAGCTCTTTCTCATATTGAACGCCATAAA

CAAGCCATAAATACAAGTAATAATAGTGAGGATAATGATTTTCATAAATTATTACTCCAA

TTTAGCTATGATGTATATGAAAGAATCAAGGCAAATAAAAAACCTTATCCGAATTTAGAT

TCAGATAAAGTCTTTTGACTATCTAGAAAGTTCACATTTTTTTCTTATTTATGGAGTTAA

TGAAGCGCCAACTGAATCCTAGAACCAACATCAGATTGATGAAAAGGTACACAGCTGTAA

AACTAGTTAAACCATGTTGTCTGATATCGTCAAAGTATAAAGTCGGTTCAGTAAAGAACA

GATAAATTCCATAGCCCTCTACGAAAATAAGAAAACTGACAAAACCAATCAGGAAAAGTC

CACTGGCAATAAGAAAGAGTCTCCATAAAAGAATGAGAATTCCTCCTACTGCTAAAAAAA

TTACTGGGATAAGGAGTAAATCATTCATGACTTTCTCCTTTCTAGTCAATCAATTGACGA

TAATGGGTGGCAAGAGAGGAATCAAACTCTTCTAATTTTTGAACTAAGTCCAAATACTCC

TCTTTTTCATGTTCTTCAAAGTCCACACCTCGATGATTCTGAAGAGAGATTTCCAATTGA

CTAGTGAGTTCTCGTTTAGAAAAACTGTAATCGCCTGTCACTTCCTCATAATGTTCCTTT

AGCTTCTTGTAGGCTTGGTATTCTTCAGGCGTTTGAAATCCCTGTACCAAGGCCTCTTCT

AATTGATAATAGGTATCTTCCATCATAATTATTACCTCGTTTCTTTCTATCTTTATTCTA

CCGCGCTTTTCTTTTCTTGTCCGCTATTTGTATCTATTTTTTAATCTAATTCAAAAGAAA

GTTGTTCTTGCTTTTTGTTTCCTTTTTCGTGGAATTGTCTTAAGGCCTGATCAATAATAT

CTAAATCCGTTTCTGTTTCAATCAATGGCGCGAGTACATCGTATTCGGCCTTTTTTGTTT

GATAATCCTCTTCCTTTGGAAAATTTTTCTCAATTTCTACTTTAGCAGTAGTCCATTTAT

CCTTTAATTCTTCCAATAAGTTCTGAGTTTTTACTTGGTCCTCTTTAATGTGATCAATCG

TATGCTGAAGCCTTTGAATTGTGCCTAAAGGAGAATACAGATCTAAACTGACAGAATACT

GATTTTCTCCCACAATCTTAACAGAGAAGGTTTCAGGAAGAGGTTGATTTGTTGGAAGAC

TAAGCATTTTAATGTCAAATCCTCGATAGCTTGCTAGGGTTCGGAATTCTTTGCTGTCAG

CTTGATTATGACGGATAAGACGGTGTAGGGATTCACCTGCTTCAGCTCGTTGCTCAAAAG

CTTGCTTGCCTACTGTCATAGAAAATGCCTGGTCTTTCGACATTTCAGACTGTTGAATGT

CGCCTTCATACTTGCTTAATCGTTTCTCAAGAATGGGCATATTTTCTTCACAGTAAGAGA

TTGTATGACGATAGTGATCCTTGCTGCGTTGAAAGGCGCGTCTTTGATTTTCTAATAGAG

TTAGATCATTCTCTAGTTCCATCTTATATTTGAGATAAGGATTACCTGTTGCTAGTGCCT

TAAAATCAGAAGCTGTCATGGTCTGCTCATCAATGTCTTCTGCAGCACGAATCGGCTCCT

TAGAAGTCATAATCTGCTTAATATAGCGGAGTTTGTTCTCCTGAGTTGCCCATAGATAAT

TATCAAACGAACCTTTGGTAATGTAGTGGTAAATATCCACTTCCTTATTTTCATTTCCTT

GTCGGATAATCCGTCCATTGCGTTGCTGAATGTCACTTGGTCTCCACGGTACATCCAGAT

GATGAACTGCTTTCATCTTGCTCTGAACATTTAAACCTGTTCCTCCTTTTTCAGTTGAGG

CAAGAAGAATCCGCACCTCTCCTGCATTGACCTTTCGAGACAAGCTATTCTTCTTTTCAT

CACTATTGGCATCATGTACAAAGGCAATTTCCTTACTAGGGACTCCTCTATCAACTAATA

AAGCCTTAATCTCAGAATAAACATCAAAGCCATTGTCCTTTTTCTTAGGTGTTCCAATAT

CTGAAAAAATTATCTGAGTAGCCTTATTTTCCATTCCCTCACGATAAATTCTTTCAACAT

TATCCACTACCTGAAGCAGTTTATGATTGTCTGCTAGACTATAACTAGAGTCCAATAAAC

GCATATCAATTGCTAGTTTTCGTGCCTCACCTGTTATCTTTAACATGTTATCCTGGCTCG

GATCAACTGTTCCACATTTGACCATATCTGACCTCATAACCAATTCTTCTAAATAGAGTT

TCTGGTTTTCAGTTAACTCACTCTCAATAGGGATAATATGAGCTTCTGGAACAGGTAAAT

CCAACATATCTTGTGTTTGAATGTCGGCTGTTTCTTTATAGATTTTCATCAACTCAGGTA

GATTGACAAACTTTTTGAATCGTTTCTTAGGTTGGTACTTATCCCCTGTAGGAGCTAATT

CCATAGAGTTTTGAATTTCTCCAAAAGCACCTACCCAAGAGTCAAAATAATCAACTTGAT

AGCGTTTTAAGATATCCGGTTGAATGTAGTTCATCATAGTGTAGAGCTCACTAATAGAAT

TGGAAACAGGTGTTCCTGTCGCAAAGACAATATTCTTAAAATCATGTTCTTCCTGAATCT

GTCGAACCTTCATTTCCATATCCACATTCTTCTTAGACGTTGTATTGGTAATCCCTGCTA

CATTTCCAAGGCCAGTAATCGGACGAATATTTTTAAAGTGATGTGCTTCGTCCACAAAGA

GAAAATCAATTCCTAAGTTCTCAAAATCAATAAAACTATCACGATTAAAGCGTTGGAGTT

CTTCCAATTGTCTCTCTAGACCACTTATTGATTGTTCTGCTTCTTTAACGGTATACTTAT

TTTCTGAATGTGTTTTAATCTCTCGTAGTTCATTGAGTTTATCCTCGATATAATTCATCT

GTCTTTCCTTACTGACAGGGATTTTTTCAAATTGAGAATCCCCAATGACAATGGCATCGT

AATCTCCTGTAATAATACGAGACACAAATTGTTTTCTTCTCGCCTTCACAAAATCTTTCT

TAGTAGTCACAAAGACTTTTTTAGTAGGAAAAAATTTCATGATCTCTTGGCCAAATTGAG

CAGACAAACTAGAGGGCACCACATACAAGGGCTTATGAACCATCCCCAACTCCTTTAATT

TAAACCCAGCACCAAGCATGGTCAAGGTCTTTCCTGAACCTACCTCATGAGCTAACAAGG

CTCTTTTTTCTTCTACGATTCTTTGAATGGCGTTCTCTTGGTGAGGACGAAGACTGATGT

TTTGTGCCAATCCATCAATGACTAGATGGCTACCGTCATACTCTCGACTAACCGTCCGAT

TATAAAGACGATTATAGCTTTCTTCAATGACTTGTTGGACTTCTGAATAACGTGAGACAA

ATTCTTGAAAGAGCTCTTGTAAATGCTGCTCTTTTGCTCTTAGAACAGAGGTTTTTTCCA

AATCTGTGATAGTCTTTTTCTTTTCTCCTTCCGTAACAGTCATGGTAATCGTCGGTTGGT

TCGAATTAAGTAAATTCTCAAAAATCTTTCTTCCTGTATCATAACGTGACCCACTGACTC

CAAGACTACTATCTTTGGCACTTGGATAGCGATAAGCAAATGATGTCCTTAAATGAACCT

GCCCATCGACAGGATTCACTTCAATGACTTGTTCAACATCTGGCGAAGACAATTCAAATT

CACGATTGGTAAAACATTCAAAAGCAAATTTACCATAAACGGATTGAGGAATCCAACGTG

ACCCTATTTTAAACTCAATATCTGCCAGATGAATCCGTGGAGGGCGAACAGATTCTAACA

AATCTAAAGCATGGTTCCAATCATATTCTTGGTTGTTTTCCTCTACTAATAGTTGAACTA

CTTCTATCTTGTTGAGAATGTCTCCTGATAAAAACTGGTTCTTAGAAAGATATTTTCTTT

CTCCTCTTAAATAGCTTTCTGGATCCATTAAAATCTGGTCACCTAACTCATCTAAAATAG

CAGCTTGGCTATGTTCGGGATAAATTGATACCATATAGTCTAAATCAACCCCTCTACCAT

CCGATAAACTAGAGTTTAAGGCATCTAGAGCCGTTGAGACTCTTGCGATCACTCTCTCTG

GCCTAACCAATGCTTTCTCAAAGGCTAAAGATTTTTTATATTTTACTTTCTGATCTTTAG

AATCGATGTATTCATCTTCTAAACTTGCTAGTAAAGAATACTTATCGTCACTATCAAATA

AGTTCCGATTAACGGAAGCATTCAAGTATCCAAATTGACTTACAAAACGGTCATATTCAC

GATTGAGTTTACTAAGCAATTCCTGAAAATCAGCCCGACTATAATCTTGATGACGTTGAA

TTTCAATTAAGGATTGATAGGTCTCTCTCAAATCAACCATTCCCTTAATGCGACCAATGT

CCTTATCCGATAAGGGGCTTTCATAAAATACAGTTTTTTTGAACAGTCCCTTATATTTCC

CTCTTTTACTCGCTTCTTCTGACTTGTAAACATCTAGTGCTTCCTCATCTGTCAAATGAA

GTTGCACGAATCGGTCTATTTTATGTTCAGACAAAGAACTGTCCCAAGCTTTAAAATCTC

CCTTCTCATCTACATAATAACTAATTTCGTCTACTTTTGAACTTTTCCGAATGCCATGCG

TATCTCGGTAATAAATTTGATTTCCCTCATATCCAAAAGAATAGAGCGCTATGTCCTCAC

GTATACGACTTGGGATAGAATTATCCACTTCTTCTTGGATAAAAACAGGTACTTTCAAAG

AATTGTCAATTTGTTTAGGTGCTCCCACATTCTCTAATGCTTTCATTATGTCAGTAGATA

ATGTTTCTGATACCCCCTTAACATTGAGGGTTCCTCCATTAAAATTTCGTACCTCATATT

GACCCAAAACTTGTATATTGTGTTTCCCATCAAAATAGGGATTGATCCAGACACGCTTAT

CCTCCTCAAAGGGAATAGAGCCACTAAAGACAAGTTCCTCCTCATTAAGATTCTTTGCTT

GATCCTTTTGAAAGAAGAGGAGATCTGTGGTCACTCGAGTACCTGCAATGCTTTTAAAAG

CCGTATCCGGCAAACGGACTCCCCCTAAAAAATGAGTGTTGGTTTTAATCTCTTGTAAGA

CATTATCTGTCCGCTTATCCATTGTCCCGATAGATGAGATAATCGACACTTGTCCTCCGT

CTCTTACTAAATCAAGTGAGTGTTTGACAAAGTAGTCGTGAATCATATAAGGTTTATCAT

AGTTTTTATCAGCAATCCGAAAATTTCCAAATGGAACATTCGTTAAGACTAAATCAAAAC

TATTATTTTGATAGGGAACTTCTTCAAATCCTCGCACTTCAATATGGACATTTGGATGGA

GTTGTTTTGCGATTGCGCCTGTCACACTGTCTAATTCAACCCCATAGAGTTCTGATTTCT

CTCGTATACTTCTAGGAATAGCCGCAAAGAAGTTCCCAGTCCCCATAGAAGGATCTAATA

TCCTTCCTCCCTCAAAACCATCATCCAGTAACTTTTGCCAAATCTGGCGAATAATCATTG

GGTCTGTATAATAGGCTGTGAGAGAACTTTGTTTCATAGTGGAGTATTCTGATTTACTTA

CTAAACTCTTAAGAGTTAAACGTTCTGTTTCATACTTTGGATTGAGTTCATCGAAAAATT

CATTGGCAAGACCGCCCCAGCCGACATACTTAGCTAGTAGCTCTTGTTCTTCTGGACTTG

CTTGTCGTCCCTCTTTTTCTAATCTTTTAACAAGATCAATTGCGGCGATATTCGTTTCAA

TCTTTTCTCGATTTGTCTTAGGATAAAAGTCCTCTAAATCATCTGGAAAAACAAAATCTT

GAACAGGGACATCCGTCTCTTCTATGCCTACAATAAGCGTTTCTGTTTCCTTATCCTTTT

CATTTTCTTCTTCCAGGTAGGAAAACAAATCTATTTCCTGACTTTCACTTGATGAATCAA

TCTCAATCTCTGAATCTTCTTTCTCAAGTTCTACACGAGACAATACTTGTTCAATCTCTT

CCAAACTGTTCAAGTATAAGATAGGATTCTCTTCAAATAACTGCTTGGAATCATTTAATA

GCTCTAGGCGAATTAAGTCATTTAACTGTGCATTTTCAATCGAAACCAATTGAAATACTT

GTCCTTTATAACTTACTTGTGAACCGATTGGATATTCCCTCAAAGCTTCTTCTACAATTT

TATCGACATTAGAAAAAGAGTTCGTTTCTTCCGTTTCCTGTTCAATATCACTTGAAGGAG

ATAAAACTTCCTCTACTTGATTAGACTTTTCTCTTATAGCCTTTACTTCATCAAAATGAG

TAATAATCTTCAGCTTTTGTGTCAGTGGGAGTTGTGTGTAGTTTTCTTCATGTTGGACAA

GATCCGTAACGGTATCTAAAGTAGCCATCAATTCTGAACCCAAATAGGCTAGAACATCTC

CCTCTTTTTCTTCAAAATGAATCTCAAGAACTTCTTTTTTAAGCTCTTTTGAGTCAACCA

TTAGAAGAAATTCCTCTATATCTCTACTAATACGGTCAGATAGATTATTCGCAACTCGAT

AGACATTGACTAAATTGACATCCTGATTCTGATGAAATACAAGCTCACTTAATGGTTCTA

ACTTCTCTTTTTCAGATTGGATGTAAAATCGAGTGGAAAGGTTATAGATTGCGACTTCTT

GCACCAAGTATTTCTCCCAACTACTTAATTGAGATAATTTACTTAGACCTGCTTGTCCAA

AATTTCTTGTATAACTTTCTAAATCTTGGTCATTATTTTCAGAACTGTCCTCTAGGAAAG

AAATAATATCTTGATGAAACGAATAGACATGAGGTATCTCTTCTATTTTGCCCTCTTCAG

TCAAAAAACTCTCTACCAAATCCTGCCATTTCTCATCAAAATGTTGCGCTTGATAACGAA

GAAATAAGTCCATTTTCTCTCTATCTTTTGGAATTGTTCCACGAAACATAGCCAATAATT

GTATTACTTCCATACTCGCTCCTTTCATCTATACGAAAATAGGAGAATCACATGATTCCC

CTACAACTCTATCTCCATTTCAACCTCTTGAGGACTGATTTCTAAATCACTAGATAAAAG

AGAAACGGCATCCGTTCTCATGTAGTAAGTATATAAGTCTTCTAGGTCTTGTTGGTTTTC

AATCCCTGTTTCAATACTAATCAGAGCCTGTATAAATTCCTTGTAGTGACTCTCCATCAT

ATCTGAAATCTCTTGTTTCATTTCCATAAATACTGAGGACGGTTCAGTGATACTTGTTTG

GTCTATGTCTTGTAAATCCTTTTCTAAGGGATTGGTTCCTTCTTCTATTTCCACTAAATC

TGAACGTCCATCACCATCAGAATCTGCGCTAAGAGGATTGGTTCCTAGCGCCAATTCTTG

AGCATCAGTTAGTCCATCTTGATCCGAATCACGTTGATAAATGGCTTCCATATACTTCCT

TCTTTCCTAGTTAATCTTTTTTCACTCCAATCCTACCAAATATTCCTTGAAAGAAGTATC

ACAACAAAAAAGAGAACGAGCTAAAACTGCGTTCTCTTAAAAATGATAAAACATTACTAT

TAAAATAGATTACATGTAAAACTCTAAGCAGGCAGAGCCTGCTAACTCATGACAGAAAAA

GATGTTTAGCCTATAATAAAGGTGAACAATACCAATACGTGATCTTTAATAGGGCTTTTC

CTAGCCTGTATGTCAAACTGGTTGAACAAGAGTCATGAGATGACTCGAATATGCCTTGAG

CATGAAAATATCTTACTAGTAGCCTCTTGTTCTATTGTTCAAATAGTTAACAGGAGGTTT

TTATAATGGAAGTCATGATTGAAACGTGTTTGTATTCTTTTGAATCAGTAACTAATCCAA

TTTATCTATCTCTAAATATATAAAATCCCTTTGTAATACTATAAAAAATAACTAACAAAG

AAATAACTATAAGACTCGGTTGCCAGAAACCTGATATGTCTATTAAACTACTGCCTAAAA

GTTGGCCAATAAAGCTAACTAAAATTAAAATAATTAAATTGAAAACTACTGATACTAATA

AAATTAACACCGAATGTATTATTGCCTCGCCTATACGTATTTTAAAAAGTTGTGATCTTG

ACACTCCCAGTGTCTTTAACTGTTTAATGTCTTCAATATCTTTATTAGAAGATAAAATTG

TAAGAGAAATAGAACTAGTAATAATCAGAATAATTGGAGCAGACAGATAAGCAATAAAGG

AAGCTCTTGCTTCGAGTACACCGTTTGCTGTATTCATGAATGAATACATATTTTGGGTGT

AAAGAATAAACCCAGAAATGAGAGTTACTCCCATGCTCATTGAAGTTTGTATACTTTTCA

AATAACTAGGATTGGATAGTAGATTCCAAAAGCCTGTATTAATTACATAATTTTCAGTTG

GAAATATCCTCATTAAAAGTTTTATTAGAAACACTTGAATTGATGGAGATAGTGATTGAA

TAATAAGGATGTGAATAATTAATAAAAATAATATTATGCTTGACTGTGTTGAAATTATTT

CTTTGTTTCTACTAAAACTTGCTGAAACAATCAAACCTATACATAGTAACCATAGGATTA

AACGAACAGAGATGTTAATAAAACCAGTTACTGTAACTTTTCTTTTTTCTTTTTTGGTTT

TAATATTGATGATATTTTAAGTATACGACTTGAATAAAAGTAAGCCCCTATCCCGACAAT

TGTTGGGACTATGAAAACACTCAATAAAATTGATTGAATGTTGGCTGTAATAACTAAATC

AGGTAACTCATCTCTTCCTAATAAATTTTGTAAAAATTTGTAGTAACTATCTGCCATGAT

AAATGAAAGTATTGTACCAATACTACTAACAATCACTGCCATTAAATAAAACTGTCCAGA

TACTAATAAAGATAGCTGAGTTCTGCTTGCGCCTAGGGTAGCCCATAATTGGTAATCCTT

TTTAAAGATATCTATTAGCAGTCTTATATTATTTGAAATAAGGAAAAATAGAGTAGTCCC

GCCAAAAACTATTAGCATTTGAAAAAGTTGTGAAGCGTTAATATTAGCAGTCTTTATTGC

ACTAGCAATACCAAATAAAGATGTTCCAACAATCAGGCTAGATACAAATAGTAATGGTAT

TGTTCCTAACCATTGTCTTTTTGAGTACTGAAATTGGTAAATGATTAATTTTAGCATAAT

AAATCCTCCTCAAATTAATCTTTACTACTTTCAAGTGCTTGATAGAGCTCATCAGCTGAA

GGTTTAATAATTTGTCGAGATATTTTTCCGTCTTTTAAAATGAGTGCTCTGTCAGTTTTT

GAAGCCAACTCAATATCATGAGTGACCATAAGAACACATTTTCCTTGACTCGCTAAATTA

CGAAGTGTTTCAAAAATAAGTTTTCTTGAGACGCTGTCCAGAGCTCCTGTTGGCTCATCA

GCAAAAATAATTTTACTATCAGCTATTATTGCACGAGTAATAGCCACTTTTTGCTGTTCT

CCACCTGATAAGGTAGCAACTAATGATGATAGTTCTGCTTTAAAATTCAAACTATCCAAC

TATTTTTTTTACTTTATTTCTATCAACACTCTTTCCTGAGAGTCTCAGAGGAAGTGTAAC

ATTTTCTAGTACAGGTAGAGCGGGTACCAGATTATAGTTTTGAAAAATAATTGCAATATC

TTGCCTTCTAAATTTAGCAAGCTTCCCTTCTTTTAAGGTATATGGATTTATACCATTGAT

AACAACTTCGCCACTTGTTGGTTCAGATAAACTTGATAAGCAACTTAGCAAAGTAGACTT

TCCAGAACCACTGACACCAAGTATTGATACAAATTCCCCGTAATTGACAGATAAAGAAAC

ATCTTTCAAAATAGAGACAGTTTTGTCTTGCCCTAACAAAAATTCTTTTGAAAGGTTTGA

GACTTTAACAGCTACATTATTAACCATAATAAATCCTCCTGATTCCAGTGTCATCTTTGA

TTTAATTATACAAATTGGAATTCTATTAATATTAAGTAAGTAACTTATGAAACGTATTTT

TAATGGATAAAATTTCTATTTCCTCTGGATCTCGAATAAAGGTTTTTAGCGAACTAAGAA

TTTTTTCTATTACCATAATAGTATCATAAATGAGGCTTGATTGAAAGCGTAAACATAGCT

TTGTTAAGTGATACAACTATTCTATACAAAATTTAAACATAAAACTTAGGTATACTTAAT

AAATGAGAATTTAAGTTAGCTATAACTAAGATAGTAGCATATGTAAAACTCTAAGCAGGC

AGAGCCTGCTAACTCATGACAGAAAAAGATGTTTAGCCTATAGTGCTGGCATTAAAAAAT

CCTCACACATCACGCAAGGAAATCGCTAAATAAAACAGGCTTTGACCATGTCGGGATTAA

TTGCAGCACATTCCAAGGATCCAGCTTTTTCTTTTTTTTATAATAGAATTTCCCAAAGAG

TAAGCAAGATGAAGGCCGTCATTGCTTATGCTCATAAGATTCTCAGAATCATTTACAAAC

TACTTTCCGCCAAGCATACTTATCAAAAAGAGAAGGGGCTAGGACTGAGGAAACAGTTCT

AACGCCAAAACTAAAAAATTTCAATTACACGCTAGTACAGGAATTGATTTTTTACGCTTT

CTTACTGTTTTTTCAAATAAACAAAGTATAAATTTCTAATTATCTTTTTATATTTTCTTA

AATGCTCGTAAAGCCTTATTCTATGTGCTTTCGAGTATTTTTACTGTAGGAAGATACTTC

ACGTTTCTTTGCATATTTCCTCATGTCTTAGCTGTCAGAAGTGGTAAATAAGTAGTAAAT

TCATTTGTACTACTAAGCAACAAGACGCTCCTGTTGCTTCTCTTTATTCAAGCGTTTCAT

TTCTGCCATTGCAGAATCGAATGTTGCATGTGCGTAATAGTTCAGCGTCATGGCTATATT

AGCATGTCCCATAATGTACTGTAATGCCTTTGGATTCATTCCTGCATTTGCATAGTTGGT

ACAGAATGTATGTCGCAAACTATGTGGAGTGATGTGTGGCAATTTATCCTCGTTATACTT

ATTGTATTTCTTAACAAGACCTTTCATCATGCCGTTGTAATCACTTGCCACTTTTGGATA

GTTCTTTCTATTAAGAAAGAGGAAATCACTATATCCATCAATCTCAACACGCTTATCATT

CTTTCGATTCGCTAACACTCGCTTAAATGCTTGATAGGCTTCTTCAACCATAGGAACTTG

ACGTTCGCCACTTTTGGTCTTTGGTGTTTCAATGTAGTACCCAATTTCAGTATCTCTCAA

TAGCTGATGGTCTATATTGACAAGACGATTCTCAAAATCTAAATCTGGAAGTGTCAAACC

ACCAAACTCTGAAATACGAAGACCTGTTTTTAAGAGTATCAGAATTTCATCATAATTTTT

GCTGTAGGTTTTATCAGCTTTTGCAAAGGCTAACAGTTTTTCTTCCTGTTCTTCTGTTAG

TACGGTCTTAGGGACAGTATCATCATCAAGAACTGCTTTCAGTTGAAAGTCAAATGGATT

CTTCCGAACACAATCATCTTGTATAGCAATATAGAATGAAGCCTTTAAAGAACGTTTGTA

GTTATTGATGGTTTGATAAGCATAACCATTTTCACTCATTCTAATAGCCCATTCTTTAGC

GTCTGATGGCTTAATACTGTCAATACTTCTTACACCTAACTTGTCTTTCTTCAAAATATC

CATAAGATATTTGCGTCCAGTTTCAGTGTTTTTTCTAACCTTTGGTCTTTGAGCGTTCTG

TTTTGCGTAAAGCTGGCAGAGTGTCATTTTCTTTCCTACAACATCAATACCATCATGAAT

GTCTTTCTGTAACTCTGCGATTTTCTCCCTAAGTGAGATACAATCACGCTTTCCTGCTGG

TACTCTGTCTGTAGCCACAAGTTTCCACGAGTAAACAAATTGCGGTTCTCCAAATGAATC

TGTATATTTGTATAAGTATCTTCCGTCTTTTCGTTGGCTCTCTCCAGTCTTTAAAATTCG

ACCTTTATTGTCACGTCTTTTTTCTGACATGGCATTTGCTCCTTTCCATTATGGAAAGAG

CCCTGATACGACTTAATACTATTTTATCATATACAAGACCCTTTGGCTACGCTAGATTGC

GTTCAATGTATCTATAATTTTTTCAAATTGTTTTCGTTTAACCTGAATACGATTGCCATT

CATAATCAGCCAGTTTGCATTTTTATTTTCTTCTGCCAAACGACGCAGCTTGTTTTCGCC

AATACGAAAATATTTTGACGCTTCTTCAATAGTTAGGGTATAACGCTCCCAAATCGGAAT

GTCAGTCTGCTTCATAAAATCCTCCTTTCCAAATCACTTATTGGATTTCATAAAAGTTGT

TTTACAAGCAATCGAACAGCTCTAGCAAAGCTCACGGGAGTTCCACCCCTGCATGGTTCT

CATGTAGCCATACTCATTGCCTGCGACGCTTTTAACGCTCGGACTATTGACTGTATGGGA

GTATCATTATCACGATAAGAAAGTCGTCGCTGGCAATCCTGCTAAAGATTGTTTTTCGGA

ACACTAACTGAAACGCTCGCTATCTTTAGAAGATAGGTCATGGCGGTTAGCTCCGTTGGC

TCTTTTCTTATCGAAACGTATTCAATTACTTTTATTCAGTTTTCAAAGAACAATGGCTCG

TTAGCCTATCAAAACACATTGAAAGCTCAATATGCTTTGATGGAATAACAAACCTCCCTG

TTCGGGAAGCGTGGAATAGTATGGCACGCTTCCACGAAAAGAGAGAGGTTATTACTTAAT

TTCAAATGACAGAATCTTTGTAATCAGTCTGGTTTCCATTCTTCCACGTAAGACTTCATC

AACGACCATACTTTGATTGCCATATTCATCTTTCATAAGTCGTAGGGAACGTTTCGTTAT

GTACCCTCTGTAATGATGTAGAATCTGGTTAATCGCTTCTGTATCGCCATCTGTTGCCTT

TACAATGAGAGGAAAGGGAATCATAGGATATTGTGTTTTCATTCTTCAAATTCCTCCATA

AACTTTTTGATTAAGGCTAGTCCACTGGTTCTATGCCGATAGACAGTAGAACGGTTCAAT

TTCAACAGGTCTGCAATTTCTGAATCGCTCATGTCCATAAAGTAAAACAGCAGTAGAATT

TCACGTTTCTTGTCTGGCAACTCACGTAATGCTTCACTCAATAAATCATTTTCAACACCA

ACTGATATTCCATTGAGTGTAAAAATCTGAAAGTCAGTTGAATAGTTATCTGTTGTCGCA

AACTGGCTAACAAGATAATCGCCAACATCAGAAAAGGACACTTCACGCTTTGCGATCCTT

GAAAGATAAAGCAGATAATTCTTTCGCTCGTCTTCCATAGCACGTTTACAGATATAGTCA

AACTGATTTTCTATTGTGGTCTGAAAAAAAGATGGTTTCATGTTTCTCACCCCCTTTCTG

TCTAGGAAAGGAAGTGAGCCTTGCTCTTTTATCTCCTTTCACTCTTAGTCCCAATGTGAA

AGGGGGATTTGTTGCATTACTGATGAATAAACTTTGTAAAAAAGTTCTGAATAACCAAAA

AAGCACACAAACAGATTGATTTCTCTGTTTACATGCTTTTATTTTTCTATCTATATGATT

TATAAAACCACATTGGTGGACGTACTTATCTATTGCAGATAGACGACTTTTTTTGATAAA

TACTCAATGTGGGGAACTTGATTGTATGTGATATACTTCATGGCGACTTTGACCTCCAAC

AAACCGCCATTTGGAAGTAAGATACAATATTTTAACAGCATAAATAGCACTACCATATAA

CGGTTTTTTTATTGGCGTTTAGTAGTGCTTTTTATTAAATATGAACCTATAAACTATATA

ACATGTTTTTCTATACCTGTTTCTAATTCAATAGGAACAGTAAAATGTATAGAGGTGGTC

TACTATGCGTAAAAAAGAAGATAAATATGATTTTAGAGCCTTTGGTTTAGCCATTAAAGA

AGCTCGATTGAAACGAGGTTTAACTCGTGAACAAGTGGGAGCATTGATTGAAATTGACCC

ACGTTACTTAACTAATATTGAAAATAAGGGGCAACACCCCAGCATACAAGTTCTTTATGA

CCTTGTATCGTTACTTCATGTTTCCGTTGATGAATTTTTCTTACCTGCTAATAACTTGGT

AAAAAGCACCCGACGATTACAGATAGAGAAATACATGGATAGCTTTACAGACAAAGAACT

ATCCTTAATGGAATCTTTAGCCAGCGGTATCAACGAAGCAAGAAACATCGAAGACTAATT

AAAAGAATCCATACATAACGGAAAGAGCCGATAAAATGAGATTGTATTAATCTCATTTTA

TCGGCTCTGCGTCTTTGCGTCTGGCTCTGTAATCACAGTTACTTTGAACTGCTTTATTTC

AATTAAATTTTCTTGTCTGCATTTCGGACAATAGAGGGGGAATTTTTTTAATTCAGTATC

TTCCCTTATCTTTAATCGTGTTTTATTTCCACATACAGGACACAATATCCACTTGTAGTT

TATAATAACTATCTCCTCCTTTACACTTTAATTCAAATCTTTATTAAAGAATATTTCATC

TTATTTAACAAGAAACCATATTTATATAACAACATAAAATACACTAAGTTATTTTATTGA

ACATATATCGTACTTTATCTATCCGACTATTTGGACGACGGGGCTGGCAAACAGGTTCAC

CGGTAGTAACATGGTACCCTTTTAACTCTGTTAAACAAACACTACGTCCATTTGTAAAGA

AAGTTAAATCACTACGATATTCTTGAATACACCGAGCAGGGATTTCTCCACTAAGAATGA

CCTCATTATTTTTCAATTGAGTGTCTACGATGTTCGCACAATATTTAGGAGCATCGTTGT

ATGCTCGTGAAAGATATTCCTGTGGCGCATAAATTTTAAAACTAAGATATGGCTCTAACA

ATTCTGTTCCAGCTTTTTTTAAGACTTGTTCCAATACAATAGGAGCAAGCATCCGAAAAT

CTGCTGGGGTACTAACAGGGCTATAGTATAAGCCATACTTAAAACAGATTTTACAGTCCG

TCACATTCCAACCATACAATCCTTGTTCACAGCCATAGCGTATCCCCTCCATAACTGCAT

TTTGAAACGATTGATTTAAGTATCCAAGAGAAACCGAGCTCTCATACTGTACTCCGCTCC

CTAATGGAAGCGGTGCTACAGATAGACCAATGGAAGCCCAGAAAGGATTCGGTGGAACTT

CGATGTGAATGGTATACTCTGCTTTTTTTAACGGTCTTTCCATATAAATGACTGTAGGCT

CTTTTATTTCTATCTCCACATGATACTTTTCTTGCAGCAGAGCACAAGTCACTTCCATTT

GTACTTTCCCTAAGAAAGAAAGTATGATTTCATGTGTCGCAGAATCCACATAATATCGCA

GAAGCGGGTCACTGTCGGAGATTTCTAAAAGTGCATCAAGTAACATTTCCCTTTGTTGAG

GTTTGCTCGGTTCAACAGTCGTTTGCAGCAGAGGGAGGGGATTTTCAATTCTCTCTCTCT

GTGGCAATAGCTTTGTATCTCCAAGAACACTATTTAACTTCAAAAACTCATTCTGCAAAA

TAACAATTTCCCCGGAATAAGCCTTATCGATTTTACATAATTCACCATTTATTGAAGTAT

ACATTTCTGTAATTTTTATTTTTTCCTTTTCCGATATTCTAACCGAATCTCGCAAATGCA

GTACGCCACTATAAAGACGTATATATGCAAGACGCTGTCTTTTTTCCGAATACTCAATTT

TGAAAACTTTTCCGCAAAGTTCAGACTGACCTCGATGTGTTGATGAATAAAATTTATTCG

TAATCACTTCTATAAGGTTATCAATCCCTATATTGTTTTTTGCACTTCCGTGATAAACAG

GGAACAGGGAACAATTATGAAATCTTATGCTTTCCTCTTGTTCGAGTTCTAATGCTTCCA

ATAATTTCCCAGACGTATATTTCTCCAAAAGGTAATCATTTCCTTCTATTACCATATCCC

ATTGTTCAGATTCGGTAAAGTTCATTACACGCATATTAGGATGCAGTTCTACCTTCTGTT

TGATTACAATTTCCGCAGAAAGTTTCTCTTTAATATCCTGATAAACCGTTGATAAATCAA

TTCCATTTTGGTCAATCTTATTGATAAAAAAGATTGTGGGAATACCTATTTTCCTAAGTG

CATGAAACAATATACGAGTTTGTGCTTGTACGCCATCTTTTGCAGAAATCAGTAGAATTG

CCCCATCTAAAACTGATAATGAACGATATACTTCTGCTAAGAAATCCATATGTCCTGGCG

TGTCTATGATGTTCACCTTCGTATTTTCCCACTGAAAAGAGGTTATTCCTGTCTGAATTG

TAATTCCTCTCTGACGTTCTAAAAGCGTATTATCCGTCCTCGTTGTACCTTTGTCCACGC

TTCCTAATTCTGTAATCGCTCCACTGTTATATAATAAGCTTTCTGTTAAGGTAGTTTTTC

CTGCATCAACATGAGCTAAAACTCCAATATTAATAATTTTCATGTGATTTTCCTCCATTC

AAAAACCCAAAAGGGCATAAAAATCCNNNNNCACACACTTAATTAATTAAGTGTGTGNNN

NNACTGGGATTTTTATGCATAACCATAGGTATACAAAGCATACAGATATTCTCTGGATAC

TTTAGAATCACATGATAAAGGTATTCTTAAACTGGGTACAAAAAACTAAGCCCTCCTAAA

AAAGGACATCCAATTATTTGTTCCCACTATCAAATTGACAGTTTATTTAAGAATACCTTG

CCGCATATTTATTAACTCCTTTTAAATAGATACTTAAATTATAGCACGTAAGAGCATATT

TGTAAAGGAATCTCCAATTTTTTATCAAAGAGAGTACGTGATTACAAAATAGCTGTAATA

ATGTACCAATATTTGTTATTCTATAATCTTCCAATTACTCCCGTTCTTTTCAAGTACCAA

ATCAAATTGAGATACCTGCGTTGCTTTGGTCTGCTGGTCGATATACTCCACTGTCAGCGA

TACCGTGACTTGATTATCCTTACGATTGTGAATAGGATTTACCAGTTCTTGAAAGATGTA

CTCTTTTCCGATTGGTTTTAATATCCCGTCATTCACATAGTAGGAAAGTTCACTGGCTGT

CGCTGTAGGATAGAGCTTGAAGAACGTCGTTAAAAACTCATTGATTTCATTGGTTGTAAT

GGAATCAACCGTCCCCTCACTTTCAATGGCTTTTGGTTTATAACTTGATTTCTTAGGTAT

GTTGGTAATGGTCGGATTCTTAACCAGTACCATATTTCCAGAACCATCTACATAGACACT

CACTATATAAGCAGAGTGGACGGTCTTTGTATTTTCTCCCTCTGTAATGAGCTGGTCTAC

ACTGTAGGTTACATTAAACTCATTGTCGCCAGTTGGCTCTACCGTCCATATCTGAAATCC

TCTTACAGAAGACGATACAGGAATATCTTTGCGTACTGTATCAACATTGAGAGCTTGAAG

TTCATCTGTCAGATAGCCTTTTAGACTTTCCATTCGATTATCAATGGACTTATCGGATTG

CTCCCATGAATAGTAGACTTTCGCAAAGTTCTCTACAAAATTTTCTACATGATGAGTATC

AACGTATTCCTTTTCTATGATAGTTGTTTCGTGAATAGTATGAGTATCTATAGCTGTAAA

GTGCTTGAATATCGCAAAGCTGAAACTAAGCCCTAAAAGTACCCACAAGGCAATCACAAC

CTTTTTATGAGGATTGACCTTATAGTAGACACGAGGTTTCTTTTCCTTTGGTATCTGTTT

TTCTTTATTCTGATTTTTTCTAAATTTCATCATTAAATCTTCCTTTCTCATTGTTTGATT

CGTCCTGCTCCCACTAAATGCTGTTGCCAGTAGGGGCTTGTTAAGTCGGCATAACCGATT

GGGTCGCCTGCATGAAACATACGGTTATTGCCAAGGTATATCCCAACATGAGTAATATAA

GAGCCAGCGTTATAGGTAGAATGAAAGAAAACCAAATCGCCAGCTTGTGCTTCCGATAGT

GGGATATGCTGGGTCACATCATATTGCTGTTGTGCGGTTCGTGGTAAGTTAATTCCAGCT

TTTCCATACGTCCATTGTGTCAGTCCGCTACAATCAAAAGAAGTAGTCGGGGAAGCTCCA

CCGTAAACGTATCGCCAGCCCTCATATTTCAGTGCTTCGTCCATGATGGCTTGTACCGTA

TCATCATCAAACTCTGTTGTGACAAGATACTGCGTTACCAGTTGCACATAAAACATATTG

CCATAGTTGTATCGCCAGCCCCCATTGATAGGTATGGCTATGGGATTGGGGTAAGACACT

TTTTCGCCACCTGAATACTCTTTTGAGAAACTTTGAGCCAGTTCAAAGGTATATTTATTT

CCACGATTAGCCACATACCCTAAGAAACCACCACCATAATTGTAGGACTGGATAACCGAT

TCTAAATCTACACTGAGCCTTTCGCTACTGGCTAATAATTCACTGAAATACTTCACACCT

TGCTTAATGGATTCTTCTGTACTCAATGAATTAGGTGGAAGACCGAGGGATTCCGAGGAC

TGCATAACATCTTCTGCTGTACCACCCGATTCCACCTGTATAATCGCAAGAAGTATATTG

ACGTATTCTTCAATTCCATATTCTTTGGCAACTTTTTCTACCATAGGCTTATGAGCCAGC

ACTTCTGCGGAAACATCCACACCGCCATAGTGAATGTTGGAAAAACCGCCATCTTGTTCA

TCTGAAAATAAAATGGCGACAAACAGAAGCAGTGAGAAGACCATCAAGAATAATCCCGAA

CCACCAATCACTAAAGTTTTCAGCTTCATGGTTTCTTACTGCCTTTCTTAATGGTGGCGG

TTTTGATTGGTGGTCTACTTTTTGTATTTTGTAGTGGTACTCTTTGAACAGTAGACTGAC

GTTCTTTTGTGATTGAACGTTGTGAAGCTCTATCTGTTGGTGCAGTTGAAGTTACTGGCT

TTTGAACCGTTCTTTCTTGAACTGTATTACTTTGACGTTCCGTTTTTAAACTTGAAGAAT

CGGACTTAACTGTTGGACGCTCTTGTTTGGCTTGTTGAGATTCCCTATAGGAAGCCTGTA

CAGTTGTTTGCCTTGAAGTCTGTCCATCATGAGATTGTTCTTGCTTAGTGGCTGGTCTTT

CATGAACGGAAGAAGCTGGCTGTTTTTTCTGTTTGACCTGTTCCATTTCAGAGCGACGCT

CCGCAATGGTTTTCCGTCTTTGTTCCTGCTGTTTCTTGCGTCCACTGGCTTTATCCGCTT

TGGTTTGAGAAATACTTCTGGTTAAATCATGGACATTATCCGTCACTTTAGATTTTCCTT

GATATACTGCATATCTTGCATTGGTCGGCAAATCCTTAACCTGTTCTTTCAAATTACCAG

CAGTATCTACCATTCTGTCTTTGGTATCAGCTACTGTACCGATGGTTTGACCGATACGCT

TTCCTAGTGTTGATTTTTCCTGCCCGTTTGGTCGAGAGTGATCTGCTTGTGTACTCGCAG

AACTTCCCGAACCCGACTGTCCTTTTTTACCCGTAGCACTGGCAATAGCAGACCCAGCCC

CTAAAGCAGTCACGGAGCGTCCAAGTTTCCGCTGTAGACGGTGCATGTGAGCGTGCATAA

GCATACGAGGTTTTCTCATCATACGACTTCCCACACTTTGAGAATCGTTACTCTGTAGAG

AAAACATACTCATTAAATCGCCCAGCTTAAAGTAGATTCCTGCAAAGGTCACAATCTGTA

GAAAAGCAATCAAAAAGAACGGATAACCAGCCGATAAGGTATAGAGCATGGTTGAAATAC

TAAATGCTGTCGTAATAATCAATGTGATTCCAGCTCGTGTCAAAATGGTATTAAAGAGCT

TTGTTATGGCTCGTTTTGACATACCATCAAATGATGGAATCATGCTTAAAATAAAGCTCA

CAGGCAGAAACATAGCATAGATGATAAAAAGTACCTGCGAGAAAATCATGATTCCTGTTA

ATAGGAATACAAATATGGAAATCCCAATATTGAAGACAAATAGGAAGAAGACTGTACCTA

AACGGTTAATCGTCTTTGTAATCGTCATATTGGTATTGCTTCTATCTTCAATTTCTTCCG

CAACAATTTTTTCTCTGTCTTCCCCATTGTTGGAATCTGGACTGGTTGAGAGCAGGCTTT

CCACACGGTCAATACCAATACTTTCAATGTCTGAACTGTTATATTGAAGCAGTAGCCACG

GTTGCTGAACCTGTATGGAAAACAGGCTATCTCTGATTAAGTCCACGCTGTCCTTGCCTT

GACTATCGGAATGGGGCATGACAATCTTCGTGCCAAGTGATAAACTGGCATTACTGATGT

CTGATGAAAAGTCATTGATTTTTTTAATGTAGTCGGGAGCGTAGGCAATAAAGGAAGCCG

ATAGGATAAACACCAGCACAAAATTCATAATGGCATGAATTGCCTTTGTGGTTTCTCTCT

TTATCAGTCCCGTATAGGCAACATAAACCCCAAGAACCAAAATCAAGAGTAAGAGGAATC

CAACATAGAAACCCTCTGTTGAAAATCCGTTTGCACTCACACCAGCTAAGGTCTGCATAT

TCTTACCAATGGAATCTGCTGTAGCGGAAATGAAGTCTAAGGAATAGGCTTCCTGTACTA

AGTAACCTGTCGCATTGGAAACATACAAACTGATTGTCCAAATAAAATTGGTAATGGCAT

ATAGTCCATACATGACCTGTTTTCCAATCCCGTCCGACCAGTTCCACGGAAGCCAGCCCC

AGCTATTATCCACATAAAAATCCAGTTGATAGTTTTCAAGTGGGTATCGGCTGTATTCAT

TTGCCACATTGACCGTATCATCTACCAAGCCCGCAGCTTGAACCACCGTTCCCAGCATGG

CTAAAAGAAAAATGGCAATCACAAGTGTGAAAGCCACTGTCATTGCCACTTTACCTAGAC

GTTTCAGCGTCCAGTTTGATTTTATTCTGTTTACTATTGATGGTTTCACATTTACACCTC

TTTTCGCACAGGTGGTCTGGTATCAAAGGCATGGAGCAGTTCTTCAAATACAGGGTGGAA

CTGTATCACACCGACACGACCATATAAATCACTGATAAGGCATTGCCCGTTTTCCAAATC

ACGCAATCGCTTCTGATTGTTTTCGTCCTCTGGGTCTACACCAAAAAAGGCTAAGGTCTT

TTTAATCTCGTTAAGGTCAGTGGAACGAAATGCAAATTTTAAGCCGAGGTTATTTTTCAG

TTTTTCATCTAAGAGGTCGTCTGTATTTTGGGTCACGAAATATACCCCAGCGTTCATAGC

ACGACCAGCCCGAACCAGCTTCATAGATAGTGTTTTTCCTTGTGCTACCTGTAAAAAGCT

CCATGCTTCGTCTAAATCTACAATCTTGAAAATGCTTCGGTCTGTATGGATAAAGTCTAA

AGCAAAGGTACTAATGACAATCAGCATTGCTACCGATAGTAACTCCATTGTGGTATATTC

CTCAAAGGAAGTTTCCTTGTCAGGAAGTACCAAGTCGGCAACCTGTATAATGTTCAGTTG

TTTTTCAAGGCTGATAGACTGCTCTACATAACCATCACTAAAAAGCAGATGGGCAAAGTC

ATAGTCTGTAAAACTTTCGATATGGTCGGCTATGCTGGTACTTAGTGGCGTATTCTCAAC

CCGTAATTCTTCAATCACTTTCATCAACCCTCGCACTTCACTATTGGTTACTGCACGAAT

GGCTTTTCTAAGGATTGGGAAGCGTTCCCCATCACGAGAGGAAATCCCCGTAAGGAATGT

CAAAATATCAATAGCCAGTGATTCAGAATCTTTGGTATTTTTCATAATTACATAAGGGTC

AAGTAAGCCTTTGTTTTTCTCATCAGAAGTCAGATTGACGATATTGATTTCATGGGAAAT

CTCTGGCAAGGTTTCTTTCCATCTGCCACGTTCTGCTTTTGGGTCTACAATCACTGCTTG

TGCCCCATAAAGCACTGCATAATAGACGATAAGGTTATTCGCAAAGGATTTACCACCACC

CAGCGAACCGACAAAGGCAGACGCTAACGCATTGGTTACTGAACCCTTAACCCCTTGACT

GGCAAGAGCAGGTTTCAGATAGACATTGCGTCCAGTATCTAAGCTGTAGCCAACATAAAT

CCCCTCATTTTCACCCAGCATTTGAGTAGCACCAAAACCTAAACCAGCGAGGAAATCAGA

GGTCACGTATTGAATATAGTCATTCATATATCTTTTGCTGGCAGGTAAAAATTCTTCATG

TAAGCCCAGCATATCCCCAAAAGGTCGTACCAGTTTCACGCTCAAATCATCATAGAAATC

TTTCACTTCATTACAACGACGTTTAAGTTCGTCAAGGTCATTTGCTGATACCCTTACAAC

ATAGGACAGCTTATACATAGATTCCTTGCTTTGGTCTAAATTGGTTTCCAGCTCGTTCAC

ACTTTCCAGAGCTTCCGCCACATTAGAGCTGGTTTCATTATCACTTTGCCATGCGTGGTT

ATCCAAGTCTTTCAGTTCTTTCTTTTTATTGCGGACAGTAGATAGGGCTTTACGATTCGC

TACAATTTCCACATTCATTGACGTATCAATCGGAAAGGTAAATTGCTGTTGCTGGTAGTA

GAAGATTTCAGAGGACGGGAAGTCCAGTTCGCCGACAATACTGTTGATGGTAAAGTAAGC

TACATAGGCGGTTTCGTCTTCCTGCTGGATTTTCAAATATCGCTGTTTTTCTTCCACCAG

ACAGCGAGTAGGCTTAATGAGGTCATAGGTTTTAATCAGTGTTTCATGTTCCAGCTTTTT

CTTTGATAGAGGATACTCATACTCTTCATAGGCAGTGCCTGTCTGTCCGTAAAGGTGTTC

AATCAGATAGCCGAAGTCGTCCTTATCTAATCTTCGGATTTTGAAACGACGGGAGATTTT

ATTTTCTAAAAGCTTTTCCATCTTCTGAAAACGCAGGATTTCATCATTACTCATACTAAC

AAAATCGCCCATCAGCTTATGGTTCACATCATAGACAAAATCAGTAAAAGCATTTTTTGC

TTCAACGGTAAGACTTTTCATAGAAAACTCCTGATCGTTGAGAAGCAACTTAAAACCGAT

AAAGAAACGGTAGTCCACTTGATTTTCGCCAATCATGGATATTAAAGCGTCTGTCTGTTG

GTCGATTTTGTCATAGGCAACCGTTTTGAGCTTTCCAGTGACTTCATTTTTGGAACGTTC

TTGTGCAGAAAGTATGCTGGATTCTGTACTGATTTGTAAAGCATGAATCTTGCCATCACG

ATTTTGTGCGATAAGCTGTCTGAAAGAATCATGTACTTGTATTTTCTGTTCCGGACTTAG

AAATGAGTAATTGTAGGGAACAAGCTCATAGTAAGCGTAACATTCCCCATCTTTATTCCA

GACCAGATTGTTTTCAATATATTTAATTGGATATGCCATAAAATTCACTCCTAACTGCTG

TAATGGCTTCTTGTGGCTGGTTTCTGCCAAGCGTTACTTTTTTTCCTGCATAGGTCAGTT

TTCGTCGCAGTGCATAAGCAATGACAGACTTCAAAAATCCATAAGGCTTTTTACCATCAA

AGGTTTTTGTAGACATAAACCATGTGAAAGCCACAGGAATCCCAAAGTATTTGAGAAATG

CTCCCTCTATCATAGAAAGAGGGGGCAAGTTGCCAAGTATCATAACTGCAAAGAGTGACA

CGACAAACCATGTCATTTGCGTAAAGGTTATGGGAAACGGAAGTCTAAAGTCATTGATGG

AGTACAACACTTTCTCCACAGACCAGATACTGGTATAGCTTCGTATTTTCTTCATGTAAT

CAATCCTTTCATAAAAAATAGGGGTAGCTGATTGAGCCACCCCGTAAAATAGAAAATCTG

CCAGTAGTAATGTACCGACAGATTTAATAGACGATTTCAAAAATTCCATGATTGGTTGAG

ATAAACGTTCCTGAAAGGTCTAAATCCCGACCATAGGCTTGATAATCAATATAGTTTTGA

AGACTAGCTGGTACTTCGCCTAAAGCACCCGTTTCTTCAATGTAGTAGCGTGCCACGTCA

TACATATCATCACAATCGGAATGAATGATAATATCCTCTTGATGTTCGCTTAGTTCTTCA

ATGCTTGAAAAATGAGTGAGCAGAGCAGATAGCTCCGATTGTAATTCTTCGGGTAATTCC

GATACCATTTCCCATAGTCGATTGAGTTCGCCAATGGAAGTGTATTCGTCAACCGTAAAG

GGTAACTCGTAGTCATGAATGGCGTATTCCTCATATTCATCATTCAAGCCGATTTTCTCT

TTGACTTCCTCAAAGTCAATGGGAAAGGTAAACCACGCACCGACCAATTCGCCCTCATTG

TATTTGCCTAAATTCGCAATATAGACTTGCATATCGTCCATATATTCACGTCCTTTCTTT

GTAGAGATTCAAAAATCCCTACCGCACTTCGTTTGGTGTACCATTCCTTTGCGGAACATA

AGAAAACCACTTATATTCCACAAAAGAACGGTTTTATTTAAGCACCAATAATGCGATTGA

ATAGCTCTAGTAAAATGTCTTTTACTCCAGCAGCGTTGAAGACTAAGCCAACCGCAATAA

TCGCAATAATTAAAAAGCCAATGAGTTTACTGAACTCACGCTTGAAGCCAAGATACAAGC

CAATCACAACGATTGCTAAAAGCACCAGTGATTGAGCGTTTGATAGAAACCAGTTATAAA

GGTTTTGTCCAAAATTCATAAAAATGTTCTCCTCTCTATATTCAATGAATTTGTATTTGA

GTTATTTTTTTGGGGTATACACTTTCTGGTATGGATAGAGATTCCATGCCAGATTTTTTT

ATACAAAAAAGAGGACATTTGCTGTCCCCTCGTTATACAATCAATTCACCACAAAAATCA

TGAAAGAAGGTTAAACAAATGTCCCATAATGATTCTATCCTAAATATTCTTGGAATTAAA

GATAAAAATATTAAAATTATTTCTGTTGAAGAAGCTGAACACAACAACGATTCTGTTAAA

GAGTATATAACGCTAATAACAGCTACTCTTTCTTATCCGATTAATCGTTGTCGTAACTGT

GGCTTTCCCACAGTTAATAAGGATGGCTTTCGCAAAACTCATGTACGACTGGCAAGTTTA

AATGGGAGAAGATATGAACTAGAGCTTCGTAAACAACGCTATAAATGTAAATCATGCCAT

ACTACTTTTGGTGCTATTACTAATTTAACCAAAGAAAATCAAACCTTATCCAGTGATCTC

AAAAATCAAATCATGCTTTTAGCTCGTAAAGGCTTATCTGGTCAGCTTATTGCTGAAATG

TGTCACTGCTCTCCTAGCAGTGTTCGTCGAACAATCTTAGAGCGCATGGAACCACACTAT

CGTGTGGCTAAGTTGCCTAAGCATCTATGTTTTGACGAGTTTCGTTCAATTAAGTCTGTG

ATGTCCTTTATCTGTTGTGACGCTGAAACCCACCAAATTGTCACAAAGTTACAGGATCGT

CTATCACCTACCATTGTTGATTATTTTGAAAGTCGTTATTCAAAAGCCGAACGCGAATGC

GTTCAATCAGTTGTAATTGATTTAAATGCTCAATATCAAAGTTTTATCTATCGCCTTTTC

CCTAATGCCAATATCATTATTGATCGCTTCCACCTTGTACAATTAGCTGGTCGCGCTTTG

GACAATTGTCGTATCTCTATCCTAAAGCAACTTGATAAACAGAGCCAAGAATATAAAATT

ATGAAGTCACATTGGAAGCTATTCCATAAAAAAGCTGAAGATCTTCACCCTGAAGAAGTA

GTTTTTCTTCGCGGCGTTAAACAATATATGACTCGCCAAAATGCTGTTGATCTCATTACT

AGTAAATTTTCCAAGTTCGCTGAAGTATACCAAACTTACCAAGATATCACGAAAGCCCTA

AACGAGCGCAATAGTGAATTACTAGAGTCAACCATCTTAGACTACCAAAAAACCAATACA

GAAATGGATACTGCTATTCAAACCCTTCGTCAAAACAGAAAATATGTCTTAAATAGCGCT

AAATTTGAATACTCTAATGGTCCTTTAGAAGGCATCAATCGCAAAATCAAAACCCTAAAA

CGAACTTGTTATGGTTTTGCCAATCAAAAATTTTTCTTTTTAAGAATCGATTGTATTTTT

TCGTAAAAAAATACCCCCTACATTTTCGTAGGAAGTATTTTTAGTCAACCATACCAGTTG

ACAGATATCCCACTTAGGACATTTTCCTACAAGGGGTCCCGAGCGCTTAGTGGGAATTTG

TACCCCTTATCGATACAAATTCCCCGTAGGCGCTAGGGACCTCTTTAGCTTCTTGGAAGC

TGTCAGTAGTATATCTAATAATTTATCTCCATTCCCTTTAGTAACGTGTAACTTTCCAAA

TTTAAAAAAGCGACTCATAGAATTATTTCCTCCCGTTAAATAATAGATAACTATTAAAAA

TAGACAATACTTGCTCATAAGTAATGGTACTTAAATTGTTTACTTTGGCGTGTTTCATTG

CTTGATGAAACTGATTTTTAGTAAACAGTTGACGATATTCTCGATTGACCCATTTTGAAA

CAAAGTACGTATATAGCTTCCAATATTTATCTGGAACATCTGTGGTATGGCGGGTAAGTT

TTATTAAGACACTGTTTACTTTTGGTTTAGGATGAAAGCATTCCGCTGGCAGCTTAAGCA

ATTGCTGAATCGAGACTTGAGTGTGCAAGAGCAACCCTAGTGTTCGGTGAATATCCAAGG

TACGCTTGTAGAATCCTTCTTCAACAATCAGATAGATGTCAGACGCACGGCTTTCAAAAA

CCACTTTTTTAATAATTTGTGTGCTTAAATGGTAAGGAATATTCCCAACAATTTTATACC

TCTGTTTGTTAGGGAATTGAAACTGTAGAATATCTTGGTGAATTAAAGTGACACGAGTAT

TCAGTTTTAATTTTTCTGACGATAAGTTGAATAGATGACTGTCTAATTCAATAGACGTTA

CCTGTTTACTTATTTTAGCCAGTTTCGTCGTTAAATGCTCTTTACCTGTTCCAATTTCGT

AAACGGTATCGGTTTCTTTTAAATTCAATTGTTTTATTATTTGGTTGAGTACTTTTTCAC

TCGTTAAAAAGTTTTGAGAATATTTTATATTTTTGTTCATGTAATCTCTCCTGAAGTGAT

TACATCTATAAACAAATACAGAAGTTAAACGATTTGTTTGTAATTTTAGTTATCTGTTTA

AAAAGTCATAAGATTAGTCACTGGTAGGAATTAATCTAACGTATTTATTTATCTGCGTAA

TCACTGTTTTTAGTCTATTTCAAAACAGTAGATGTTTTATCTACATTACGCATTTGGAAT

ACCAACATGACGAATCCCTCCTTCTTAATTACAAATTTTTAGCATCTAATTTAACTTCAA

TTCCTATTATACAAAATTTTAAGATAATGCACTATCAACACACTCTTAAGTTTGCTTCTA

AGTCTTATTTCCATAACTTCTTTTACGTTTCCGCCATTCTTTGCTGTTTCGATTTTTATG

ATATGGTGCAAGTCAGCACGAACACGAACCGTCTTATCTCCCATTATATCTTTTTTTGGA

TATCTGTCAACTGGTATGGTTGACAGATATCCTTTTTTTGTTGTTATCACGTCCTGTTCT

TTTACTGACTGTTGCTTCAAAATCTGCTTGTGTCGGTCTGTCAGTTTCGCATGGTCGAGA

ATGTCTTTTACAACCTGCGTCTGGTTGATTTCATCAAGTTTAATCGCAACCTTTAAGGTC

GGGGCAACTTGATGAGATAGCCAGTTCAGCGTCCTTTGGAAGGAGTAAGGCTCTGGTTTT

GTGGTTAGTTTTAATCGTTCACGATTGTTCCCAATAAACCAAGCCCATTCTTCATTCAGT

TTCCAATCAGAACGAGGTTTGGAATCGTCTTTATCTACAAAACGGATATACCGATTGATA

ATTTTAAAGGCGGTATGCTCTGGATTGTCATAGACGAGTAAATCACGGACTGCATAATAG

GCACGCTCATTTTTCAATCGAATCTCAAAACGGTTTTTTACTTCTGCGTCTTCAATGGGA

ATATCATTTTTCTTGTACTGTTCATAGTCCTTTTCATAGATACAGAAATAAACTTCACTC

TGTAATGAACCGATATAGAGGGTATTTCCCATACATTCCTTTTCATCTTTGCGTACCAGT

TCGCCACTGCGATAGCTTTTGAAACTGCGGAAGACGGATATACATTCTTCCTGTCTGCAT

TTTTCAGTGAGTACAGGGATATTCAAAATCCCTGTCTTATCGTTAATGGCAAGGTCAAGG

CGTTTCATCACACCGCCAGCCACCAAAGCGTCCATAAAGAACTCATACCAGCTTCTTTGT

TGAGCCAGAAGATAGCTTTCAAATTGCCGACACCCACGACCTTTCAATTCCACCAGAACT

CCTTTGTCCAGTTCGTGGGAGCAGAGAACGAATATATCGCCTAAAGCATAATGCTCTGAA

TAAGAATAGAAACCATAGTCCTCATGAAGAAAATAGGACAGTTTCAGTTGTAAGATGTTT

TCGACCACATGCTGTACGTCCGTTGTGGGAAAGCGAATCCTTACATAATCAAAGAGCATT

TCAAGGGGAGCGTCGGGATTGAAGCGTTCCAGAGCTTCAAAAAGAGATTGCTGTAAATCC

TCTGATGGCTTGACTTTTCCTGTTTCAATATCGCTTAGATACTGCCTTGTAATACCAGTC

GCAACCGCTAAACGATTTTGAGATAGTCCATAAGCCAAGCGTTTTTCTTTTAACTGCTGT

AACCAAGTTTGTTCATTCAGTAAAAATCCCTCCAATCAAAAAGGCGTATGTCAACTTTTA

AAGCCCATTTTACATACGCTGAAATTTTGTAAATCCCTTGTAACCAAAGGATTTTCTAAT

GTTTTTTTGACTGTTTCCTGTCGATTTGTACCCCCCTGTTAGATACGGGGGGTTAAGTGC

TGGCGTGGCTATTGCCACACCAGCCAGCAAGATCAGTCCACACCTGCGACTTCCGCTTCG

CACGTCGCCTGCGTGGACTGTCTGCTGTTGGATAACTTTTTAATTTCCTCCAAGAAATCA

TATCCTTTTGGTACAAGGGGAGTATAAAACTCTGATATGACACTTGTTCCTACATCAACA

TAGCCACGACCTTTGATTCGCTTTAAGAAGAAATCCTTTTGTACGTCACTGCCAAACATC

ATGCCATAGCCCATTTCAGACATACGACCTAAAGCCACTCTGAAATTAAACTGGTCACGG

ATTCCGTCGCCTAGATATTTTGCGTCTGGACGCTGACAAGCCAGTATTAGAAAGAAGCCA

GCTTGACGACCTAACATGACAATCTGTTTCAACTTATTTATAACTGCGGTGTTTTCTTTT

GTTCCCAGCATTTCCATGAAAGCGACATATTCATCAAAGATTAAGAAGTGTGCTGGAAGT

CCTAAGTAAGCATAATTTTCGCCAGTCTTATAATTTTCCATCTGCTTCATTTCCTCACTG

CGTTTTATCATTTCTTCATAGAATGTATCAATGCAGGAAAGCAAGTCTTCTTTTCTGTAG

TGGACATTTGCCATCACAGAGCCTAAATCGGCTAAGTCTGCATTTTTCGGGTCTAAGATA

TACAGCTTAGAATCTGTATGAAGCAAGGCTTCAATCAGTGTCAGGATAAAGTAGGTCTTA

CCGCCACCTGTACCACCAGCTATGAGCATGTGAGGCAGATTGTCATATTCCCACCACATA

TTTTTCATTAAGCAAAGTTTCCCATCATGAGCTTGTACTTCATCAATAGAAATACGACGG

GCTATCATATCATAGAGCAAGGTATATTCCACGTAGGAATCCTTTAACTCTTTATCCGTC

AGCTCACAGTACAAGCCACTTTCTAATTTCTTTTCCAAGTGTAGGAGCTGGTCTTGATAT

TTCCCCAGAGTGATTTCCACTTGTATCTGTATTAAGCCATTTTTAAGGCGATAATAGATT

TTAGGGAAGTAGGTTATCTTTTCTTTGGTACGACTGGGGGAATCCTTGAAGAAGCCATCT

GTTTTGACCTGTTCTGATTCATACCACTTATTTTCAAGAACCATCTTCGCCAGCTTTTGA

CGGTGGTAGAGTTGTTTAATCGTATCATAGCGATACCGTTTGAATAGAAACGCCACCAGC

AAGCAGACAAGAATTGCGACACTGAAACTGATACTTAAATACGGAATGTCAATCTTATCT

GCTTGTGATAGGTTAAAGTCCTGCCAGTTGACCTGCTGGATTGTCTTCACATGAAACAGT

CCGACAACAAGCAGTAAAATAGGCAGTAAGGACGCTACTGTAAAATGAAAGACTAAATCT

TTGTCACTCGGACGAATCCTTTTACCACGAAAGACACGCTGTTTCATGTTTGATTTTCTC

CTTTCTCTCTAATGGAAGATACGTGGCTATTTGTCCGTAGCAGGTTCTTTCTTAGGTGGC

TGTTGATTTCTAAAGGAATTAGAATCCTTTGTCAGCACAATATCGTCTGCCTTGATGTAC

CAGTCAACATCTGCTCCTTGAAAGGTAGCAGTAGCGACAGTATCCGCAATGGGATTGATA

AGTTCCACCCGTGCGTTATATTCAAACTCTTTCAAAGGCACGCTGGCAGGAATACTTACT

TGAATCATGCGTCCTTGCCCTTTGGATTTTAAGTCATAGGTACGTTCTTTGATTTCCTCT

GAAACCGTACCGTCTTCATTTTGGATTCTCACTTCACGACGTAGAGCAGAGAATTTTAAT

TCTCCAAAAGTCGTGTCTTTATCTAATACGATACCATTTGCTAATCTCATCATTTTTCCT

CTCTTTCTTTATTCTTTTACCATGTCGTCAGCATGTAAAAGGTAATTTGTAAAACCACGA

GTGCCGATTTTATAGCCCTCGGCGGTAATACGTGGATTGACTAACTTAACACGTTCCTCA

AAGCCGAAATGTTTTTCGCCAGCTTCAGCAGGAAGTACGACCACAATATCATCTGCTCTT

TGAACGTCTGAATAGAGATTATAGCTTCGAGATAAGACGGTTAGATGTCCGTTGATTCTT

CGCTGTACGACTTTATCCTCGCCAGCAAATTCTAAATTGCCGAATGTTTTTTCCATCTTG

GGAATCACAAATTTAAGTTCCATATTTTTACCTATCCTTTCTTTTTTATGGTTGAATGAA

TCTCGTTTGTTTGATGGTCTTTCACGGTGGGGAGCGACCTTTATTCTCTTGTTTTTCTAT

TTTCGTAGTATCACGTCCTTTCAAATCGGGTAAAAAAATAGACACCTCATTTTTGAAGTG

TCCACCTTTTGCCTATTCAATTTTTATTGGAAGTATCTTTGTTTCTTCACTTTTCAAGGG

TAAATCGTCGTATCAAAGCTCATTCATAAGTAGTAAATTAGTAGTAAATTGATTGGTTTC

GACCTTGATAAAGTGTGATAAGTCCAGTTTTTATGCGGATAACTAGATTTTTATGCTATT

TTTTATATAAAAAAGATGACTTACTGAACCTGTAAGCCACCTAATCAGTCGGTTTATTCT

GGTGTCTGCCACCGCTTGGCCCGTACGTCCAAGATTGCTATCGGATTTTGTTCTGGTGTC

CGCCACCGCTTGGCCCTTATGTCCAAGATTACTATCAGATTGACCATGAGCCGACCACTC

ATTTATAGACATATTGTATCTTTAAATAGCTACAATTTCAAGTGTAAACTCTTCTATTTG

GTCGTATAAGCAAAACTGATGGCACTATCTGCTTGAGAGATTTTTCTAAAGGTATAGTTA

GGGTTGCCCCCATAGTTGGTTTCAGACACAAGGAAAGAACCATCATCATAGACCTTCTCG

ACAAAAGCCACATGACCATAGCTGGCTGGTGTACCATGTGTACCTCCTACAAAAGAAACA

ATAGCACCTGCTCTTGGTGTAGAGCCCGTTTCCCCACCAAGACTTGAAGCTGTCGCAACC

CAGTCTTGACCATTTCCCATGGTATTAATGATTGAAATCTTTTCTCCATTTCTACCTTTT

AATTTTAAGCCTAACTGGTTCATACGAGCCGCAACACCCCATGTACATTGTCCATAAGCA

TAGGCCATACCATCTCCACCACCAGGAACAGAATGATCATACAAGTCCCCACGAACCCCT

TCAAGGGATTGTGGATCACTTTTTGCCTGTCCTCCATTTGTTTGACTAAAGCCTTTTTCA

ATTTGGTAATACCATTCCGTTGCTCTGGTTTGTCTTTCCAGTAGTTTGTCACCAGAATTT

CCCTCCCAATAGGTCAGAAAGAGTTGGGCCAGATTGGCTGCACTGCCTGTATTTTTAAAG

AAATCCTTTAACCAACTTTGATAGTAAGGACTATCCCCATGAAGCATAAAATCAAGTTGT

AGGTCTAAATCATACCATTTCTTATTTTGGGTGCGTGCATAATTTAACAAGGCTGTATGC

CGTGTTGACCCATCTGCAGTATCCGTCCATTGCCCTAAACCCAGACCTCTATGAAGAATA

TTAGGATAAGCACCACTATAAATGGCTGGACCTCCAATCGCTAACCAGCTTTCATCATCC

CATGAGGAATCGGTAGCGCCAACAGGAGGAGATAAATAATCTCCTTCAGCTCGTTTAGGA

TTAATAGAAGACTCTACCGACCAATTTCCTAAAATTGCCGCAATGGCTTGGGGACTTGCC

CCTTGAGATTTCAAAAACTCATAAATATGTTTTGCTCGTTCAAACTCATCCCCACCAAAC

TGACCAATGGCAGGTAAGATAGTTGTCTGAAGTTGAATGACTTTTGGAAAATAAAATTGC

GGATTGACATACACCAATTTTTCTTTCTTGTTCTTATACTTTTGATAGGAAACTTTCAAA

CCTGTATCATCTGGTGTTTCACCAATAATATCACCAGTTAAGACCCTTGTCCCCTCAATC

GCACGGCCATTATGGATGGAATATAATGTTAAACGACTCTCATTCTCTCCTTTTCCGTTA

GTGAGAATAACATTGTCACCATCTAGAGATACAACTCCATCCATTGGTGCGACAATCGTT

TGGTGAGCCTTCGCTTCTAGTAGAATGTACTCCTGAAGGGTAGGTTTTCCGTCTAAATCA

TAGTATCCATAACGATAAGTCATGGTTAGACTATCTTCGTTGCTTTTCCCCTCAAATGGA

TTGTCTAATTCCTGCATGGAAGCATACACACCCTCTTCTTTTAGTTCCTTCATTTCCTCT

TGATCGTCTTTCGATAGTTTATACTTAGGAGTTTCATATAGGTCTTGCATGGATTTCAAA

TCTTCCCCATCGTTTAAATCATGCCACAAAGTAGACAGATAATCCTTGTAAGTTTCTGAA

CTAAATAAGTAAACTGGTTTGTGTAACTCATAGTCATGGAATTTAAAGTTCATATACCCC

ATCACATCATCAACTTTTGTGTAATAAGTAATGCCTTTATCATTTGTTCGAGTATGTTCT

GCGTCTTCCCAAGTTAGGTGGGTATAAGCTTTTGTTAATTCAAATTCATCTTGTTGAATC

AAACTAGCAGATGAAAATCCTAAAAAGAAGCTCATCATAAGTAAAAGAAGAAAGACTAAT

CCTCCAACTATCCAGGTTGCAGGATTTCCAGCCGCAAATGTAAAGAAGGAAAAGGCTGCT

TTTAGTTTTTGATAGATATTTCGGACACTTGTAAGACCTTGTTTCTTTAATTTTCGATAC

CGATTTTTAAAGGAACTTGGGTTATCTTTCGCTAGTTTCCATCCTTTTCCAGCCTTAAAA

TGATGGTATCGCTCTTTTGTGTTGGTCAGTCTTTTCTTGGTAAAACGACCTGTTGCTTGT

CCTGTTTTGACACTAGCTTTTCCAAGATTATAAGAAAGCCGACTGTAGCGTTTCCCTTTT

CTAATGGTCTCTTGAAGTGTGCGATAGCCTTCTAAATCTTCATTTTCTGAAGCTAACTCT

CCACCTTCACGTCCAAGGACATAAAGAAAAGTTTTGGCTTTCCTACTGACTTTTTTGGAC

TTATAGGCTTGTTTAGTAGATTTTAGATTCTCTTTTGCGGCCTTGACTTCTTTCTTAGCT

TTTAATTCTTCTAAACTCTTCCCTTGAAAGAAAAAATTAGATTTTTGTTTCGATTCCTGA

CCGTAGAGAAATTTTTGATTGATTTTTCTTTCTTTACGACTCTCTTTTCTTTCTTCTTTT

GCTTCTACCTTGGCTTCTCTAAATTGCTTCTTGGCTATTTTCAATCGTTTCCTAGCATGA

GGCAACCGTCTGTCTCTTAATTCTTTCCGATTCAAAAGAGATGGAGGACTATTTTGAAGA

ATATGATTGTAGTCTTCATTTGCTTGTCTTACTCTCTCCTTTGAAGCTTCTCTCATCTCC

TCTAGCTTTTCTTTTATCTCTTTTTTCCATCTTTTTTCATCCAGTCCAGCGGAATCTTTT

TTCTGTTTCCTCACCTCCTTCTTTCCTTGTTTCAAGAATTTCTTCTCATCTTTTAGACTT

CTTCTAAATGCCTTTCGGGCACGTATGATTTCTCTTTTATCCTTCATTTACCTTCCCCTT

AATTAGAAGCCATTTTATCAGGATCTGTACTCATGATATCAAACAATTGAGTACCTTGAG

GAATCTTATTTTTAAAGGGAACAACAACTGAACCAGCTTTTATCAGTCCTGCCCCTTTTT

CTGGATTGACAAGGTATTTTTCAAGTTCTTTTGACAAGCCTAAGAGTTGAACCAGTTCTT

CTCTATCATTTTTTGCTTGCTTGAGGAGAATCATAAATTCACTATTTGCAATAATCCGTC

TACCATTTGGATCTAACAATAAGGTTTCGACGTTTTGAGTTATTCCAGTAGGACTGGCTC

CATATTTTCTGACACGACTCCACAATTTAAAGAAGAAATCACTGGCATATTTATCTAATA

AGAGAAGCTGCATTTCATCAAAATAAATCCAGGTCTTCTTCCCTAATTTTTGGTTCCGAA

CAACACGATTCCATATCTGATCAAACACTACCATAAGGGCGATTTGTTTCAGCTCATCTC

CTAACTTCTTAACGTTATAAATCAAGAAATTAGATCCTGTCTGAATATTGGTCTTATGAG

AAAAAATATCAAGAGAACCTTCGACATACAGTTCCATATCAAGTGCCAAATTTTGCGCTT

CTTCTTCTGGTTGTTGACTCAAGACAAAAACCCATTCTTCCAAAGAAGGCTCTTTAAATG

ACTGATAGGTAAGTCTGGTGACTCGGTCGATAATCGATTTTTCTCTCCCATCCATTTTTC

TATCCAATAACTTGCCAATAAAGGATAAAAGAAATTCTGATTTTACTTTTACAGGATCTT

CATCCATATTTTCCTCAGACAAGTCAAGAACATTGAGATAGGTTTGGGAATCGGGCGCAA

TATCAATCATTTCTCCCCCAAAAGCCCGTCCAATGACACTGTACTCTGCTTCTGGATCCA

CAATGATAATTTCAGTATTTTCACCAGATTCCTTGATTTTGGTCGTGATAATTTCATGCT

TGGTTGCCATCCCTTTCCCAGCTCCAGATGTTCCTAAAATCAGACCAGACGGTGTATTTA

ATAGGCTACGATCAATGGTAATAATATTGCTTGAGATTTGATTGATACCATAATATTTCC

CACTACGGTCTTGTAAGTCTACTGAAGTCCATGGAGAGTTCACTGCTATATTGGACGTTA

ATAAACTCCGTGATACTCCCTCTAAAAAATCACAACCAAATGGCAGCAAACTATTAAAGG

CTGCTTCTTGCATATATGGAAGTTTATCAATCATTAGGTCATTTGAGCCGGCCACTTGTT

GGATCGTGTCTAGGGCTTGTTTGAGTTCTTCTTCATCCTGACCAAAGACCCCAATCAAAA

AGACTGTTTGAAATAGTTTATCTCCTGTCTCTGTCATGGTTTTTAAGAGTTCTTCAGCTT

CATCGATATTACTTTCTAATACATGGCCTACTTTTTCTAAATAGATACCTGTACGGGCAA

GTTTTTGTTGTTCCCCAATCTTTTGGGATTCCATCAAGGTCTTCTTTGTTCGTAGTTTCT

TCATAGCATCCGCCTTGGTCGAACTTTGAGCATGGAGGCTCACAATCAATTCCAAATCTC

CTTGCATGAGATCTCGGATAAACTGATCACCTAATTCCATGCCGTAGTCTCTCACATAGA

CAATCTGCAATAAGCGGTCATTGATTTGTAGGTAATTCTTGTTTTTAAAATCCAAGAGAT

TAGGTGCTATGAAGTGACGAGTTGTCTGACCAGATTTCGTTAAATCACGGTAAGAAAAAG

GAAGATGGTGTTCTCCTCTAAGCATATCGGCCAACAAGTTGACACGCTCTTCTCCAGCCA

AGGATTCAAATCGTGCATCAATTTCTGAGAAACCACTCTTGAAATATTCTCCTATTTGGG

ACAAGGAACGATAGGCTTGTTTGGGATTAGAATCCTTTCTACCAAAGCTAATCAGTTTCA

CAGCTGAAAAGTTATTTTCACCACTGTCTAAATTCTGATTCATCATCCGATTCAATTCTT

TACGATAGCTATCATATCCATCTTCTTTTTCCTCATACAAAACACTTTGTCTAAACTTTT

CTAAATTCAATCTTTTATTAAAGATAGTCAATTGGAAGTTAGTTTGGTCATCTAGTGAGT

TAATCAAATCAGAATACTTCTCAATGATTGCCCCCTTGTCTTCTAAACCAACAGTCTGGT

AATTGACATCACCAAGTAAATAGCTTTGTGAGAAATAATCTTCTTTCACCTGCATCAGAC

CATTTTGATACAGGGCTTGATAGGAAAGAGTATTAGCCGTTGATGGTAACACTTCCTCTT

TTTTATCTTTAACTTTTTCCTTTTTATTAGTCATTGAAGTTTTTTGTTTCTTTAATGTAT

TTGATTTTCTTTTCATGTTCAGATCCTTTCTTTCCTGTAATTGTGCGTAGGGGAACCGTT

AATTCAAAATGTAGACGGTATTTCAAATAATGTTCAAAATATAAATCATTGGGTTTATAG

ACTCCAAAAAGCATGAGGGGGATGGTAAAGGCAAACACAAAACCGTAAACAAACCAATCT

CCAAATTGCCAGAAAAAGAGGTTCAAGCCCAAAACAATAATTGTGACAATAAAGGCTGGT

AAAACAAAGATGATTTGCCTTGTAGTGAAGCCTAACCAAGCCCTGTGTTGGTATTTTGAA

ATGTCTTTAAAGACACGTGTATTCATGACTTTCCTTTCTAAAAAGGCTAAGAAGCAATTA

CTTCCTAGCCTTTATCTAATTACATACCTAAGATTGAACGAGCCGTACGTTGAGAACCAA

CGAGGGCAATAATCAGTAAGATAGCTTGTACCAAACTACCAAACATAATCGCAAGTGATT

GCAAGACTCCTGCACCATTTGAAACAGCTATTTTCCCAGCAGATTCAAACAAAGGAACAA

GAGAAACAATCAGAAAAATGAGAACCCCTTGTACCGCATAGACCATAATATTTTTTAAAT

AGCCAATACCAATAGACTTCCATTCATCACTTAAAAATGTTGGAATCGTAAGAGGTGCAA

ATGGGATCATAAGGTAGAGTTGAATAAATCGAATAGATACCAAAAGATTGACCATGGCAG

CACTTACTATCCGAACAAGCCAAATGAGGAGGGCGAAAAAGCCCACAATCATCCGGCCAA

TAAATCCTGACCCTTTTAATCCAGAGATGGTATCATACTTTGCCCCACCGTGAGCCACAA

TCGAAGCCACTTGTTCAATGGCGTGACTCGCAATCCCGATGATAGCTTCTACAATCACAG

TTGTATTGGTAATCACAACTGCGACCATAATATAACTAATCAACATCGGCGCTAATGCTT

CAAAGGTCATTGCTCCACCAGAGTTAGCAATTTTCTTTGCCATCTTCGAAAATTCTAAGA

TGAGAACAACTGATAAAATCGCAACTCCAAGAGGCTGCATGACACTTTTAGTAATACTAG

ACATGTAAGTCCAAACTGTTGGATTGTAGCTAGATAGGGATTTAATCAGATCTACCGTAG

ATTGTAAATCCACATTAAATCCTTCAAATAAATTTTCAGCTGATATTTTTTCAGATGTAA

GGTAAACAAAGGGTGAGACTAAACTTAGATTCATGTCATTGTTTATCCTCCTAAATTGAA

ATCTGGGTTACAAAGGCTCCAGCAGCCCCTACCATAACACCACCGACAATTTCCAGAATG

GCATTCCGAACACCTGGTCCACCATCTTTAATATTGGTTGCAAGGTTAACAATCCCCACA

ACAACGAGAAAGGCACCAACTGCAATCAATCCCTTCTGTAACAAAGACATAGCTTGTGCA

AACATGGCACTTGCGTCTACTCCATAAACAAAACCTTTAAAATGCGTAATCATCTATTTC

CTCTTTTCTATTTTTATTTTAAACTAGATTCAAAAGTTAGATCACGAATTCTAAGGCCGT

CAAGATGATTTTGTTGTCTTTGATTCAAAGGATTGATCTGATAGTTCCACCAACGTTCAT

CAGTTTCTTGATTGGCTAGGTACTTCCAGTTTGGATGCTTGATGGAATTGTATTTTTTGC

TTTTAAAGACTGGCATATTAGCAATTCGGACCAAGCATTCATGCCGTTTCATATTTCCTA

CTTCATCAGGTGTCATTAAATCACGAGCAATCTTTTGATGAGAAAGGGATCCTGAACCTG

TTTGGCCAAAGGAACGACTAGTATTTCGAACATCAATGGTTTGTTTACCGAGTAACCCAC

TCATAAATTTAAATGTATCTTCATCATTACCACCTAAGTAGACTAAGCTATCACAGTTCC

CAAGAATAGTTTTCCAAGCTTCTTTTTCTTTATAGAGTCCTTGAAGTTGAGCAATATTTT

GTAGAATAGGAACGAGACTCATATTCCGAGAACGGACTGTTGAGGTTTGTTCCGCAAAAT

CTGGGATTTCTCCGATATTTGCGAATTCATCTAAGTAGACTCTCACATGAAGAGGTAATT

GCCCCTTAAAATCAATATCTGCTTGTCTTGTTAGGGTTTGAAATACGGTTGAAAAAAAGA

GGGCTGAAAGAAAGCGAAAGGTACTATCGTTATCTGGGATAACTAAGTAAACCATTGATT

TTTCCTGGCCCCATGTCTTCATATCAAGAGTATCTCTTTTGGTCAAATCCATGACACTTT

GAATATTGAAGAGGGCAAATTTAGCAGTGGTTACAGCTATAACAGAATCCAGAGTCTTAT

CCTTATAATTTTGAAAATCTGCCCAATTTCGCATGGTAAAATTTTCAGTTCCATACTTTT

TAGCATAATTTTCAAATAGAATTTCTAAGACACTTTTTTCTTGATTTTCACCCTTGGATA

AGTGTTTAATGAGTTTTGAGATTTCAGCAAAACTTGGATAACGCCCTCGTTTTTTTCGCT

CTTCCACTTCTTTTTTTTGACGTTTCAACAAGTTTTGGTATTCCTTTTGACTTAAACGAC

TTTCTTCTATGAGCTGTTCTCTTGTTTTGGGTGGATTATAGAAATCGACCAAGTAAGAGG

CTAAAGCACGGACTAAAGTCATAGAAGCCTCATCCCAAAATGGATCACTACGGGAGCCAG

AGCCTTTGGTGTTATTAAAATAAACCGTCAGCATGCGATTCAAATCGTTTTCTGTCTCTA

TATAGCGAAAAGGATTAAAGCCATCTGAGTTCTTCATATTGACTAAATCTAATACCTTTA

CTTGGTAGCCATGTTCTAAAAAGAGTTTGCCTGTTTTCTCGGCCAAGTGATCTTTAGGAT

CCACTACAATATTAGAAGTATTCATCTGAATCAGATTGGGTTTCACAAAGCGAAATGTCT

TCCCACTTCCTGAACCTCCAATCACCGCAATATTCTTATTTCTATCATATTGGGGTGGTT

TTTTATCTAATAATGTCAAACGAACATCTTGTGCTAAGATCGTATCATGAGAAAATTCCT

TACCGTAAAAGAGCTTCTTTTCTTTTAGAGTTCCAAAACGGGCGCTCCCGTATTCTACCC

CTTCTCGGTATTGTTTTTTACCAGTCTCTAGATAGAGATAAACCAGTAACATCATCACAA

AACCTAGTAGAAAAAAAGCACTTGATTTTCCAGTAAAAGAAACATTCCATGGCGACTGAA

GAACTTCATCTTGACCTTCCATCAGAAGATGAGTCCATTTATCTAAGCTATTTCCAGTAT

AGGAATCATACAAAAGCGTCAAACGATGAAAAAGATAGCCTAGTAAGATACCTAACAGTG

AGAATAGTAGGAATTTCTTTCCACTGTACATCATATCACCAACTCTTTCTGTTTGACGGC

TCCTTCTTGTCTAAAGGTAATTTGTGATTTAGCTTCATCAATAGCATCGTCTAATGACTT

ATCCATGGTAAAATCAGCTAATTTCTCCGGATCATTAACCATTTTTTCTAACAGATAGTC

TAAATGATTGTCTAGAATCGAACGATCTTTCGTGTAGAAATGCAGAGATTCCCCTTGCCA

AGCGATGGCTAAAGGAATCTCTTCTTTTTCTAAAAAAGCTTTAAATTTCTCTATATCAAT

TGGTTTGTCTAAGAAATCTTTCTTCAGATTAATCGTATCAATCGCATAGGGAGATTGTAG

CAATTCTTCTAATTTCTGCACCCCTATCTTATAGGCGGAATCCTGTGCTAAAGCCTGACG

TCTAGACCATTCTAGAATCTTTAAAAGACTTTTTACAGTAAATAAAAGACTACGCTCAGC

ATATTGAACTGCCATTCGTTCCTGTTGTTCAGAGGACATCTGAGGCCTCCTTCTTAACAA

ATAGCAGCTTTCCTTCTTTATAACGATAAGCTATCAATTTCTGACGTTGCTTAATCGACT

TGACAACCTGCAGTAGATCTCTCGAATAAGGTTCTCGAAGAGTAACTTCTACTGCTTTCC

CATCAAAGATACCAACACGTTTAAATTCAATGTAGTCTTTTTTGAGATGTCCTAAGCCCA

TACCAAAGGGAAAGTGATGAATCAATTGGATGGTACAACCACCTTTATTTCCCATTTGTT

TCAAATCATACAAGTTTACTACTTTCATGATTAACTCCTTTATCTAGTTTGTCGCATCGT

TTAAGTCTGGTAACGATAAACTCCGTGTCTGTTAGAAAATTCTCACACACGTCTTGTGCC

AGTTGCCCTTCACAGGGAAATACTCTCAGTCCCTACTTACACAGGCACGCTAATCAAGAC

GGAGTGGATTCAATTTTCAAAGAACAGGTAGCTTTATTATAGATAAGAGTAGTTGAAATT

TTTATCACATTTTTGGAGTTATTGGAACAAAAAAAGCAACTCACTGCTTTACTAATGAGT

TACTTTTGTACTAAAATATTTGTTGCATAATTTCCTTAATAACCTGAACAGAGTTATCAA

ATGAGATATCTTTAGCATACTTAAATTGGCGTTGATAACGAATCCATAGCTCTTTCTGAT

AATCAGAAATTTCAATTCCTCTCACTTGTTCTTCCCAATTATAAATAGTATCTTTACTTT

CACGTTTTTCTGCCGTATTTTCAAGTGCATTTTTTAATACTTCAAGATTAATCTCTTCTT

TTCTAAGTTTATACAAGGTGAAAAGATCATATCGATCACGTGGGCGTGTCGAAGCTAATC

CCCTACTAATGATCGTTTCTAACTTTTCAGCAAGTACAGTCTCTAAATTATATGTCCATA

TCTTGATGCTTTCATTAGTAAAGATAGAAGTCATTGAATAAGTTATCTCTCTTGGTGTGA

TCTTATCACCAGTAGTAATATCAATAAAAACAACCTCCTTTAATGTATCAAAAGTCGCAT

TTAATTTTAATGAAAATCCTCCATATTCATCATCTTGGCGAATAGGCTCTAGCTTATCTA

CTGAAAATGAAAATCCATCAGTTTTTGAACAAAGAATCTCTTCAAAGATATGAATCAAAT

TTTCTCTGCTCATTTCGGTTCCCTTCAGAGTGACATCTAAATCCATTGTGGTTCGCTTAT

CTAGTCCAATCATTTGACCTATTAGATACCCTCCTTTTACAATGAAGGAATCTCTATAAG

GACTAGTAGAAATCATCTTTAACACTTGCTCAATTAAATAATGTTGTTGTACTTGTTGAG

CTGGAATTCCCTTATCTTTTGAAATATTTTTGATTTTTGCTTTAAAGCTATTCGCATTTG

AAAACATTAAGATAGCACCTCCGTATAAGATTGTAACTTATCAGTTACTTTAAATAGTTG

GGCATAATAAAATAATTTGTGTAAATGAATTTGATTCTGTTGAAAATATTTCTTAAACGC

TGGTGCAATAATTTGAAGATCCACCTGATGAACTGGTCTTAGACATTCAACCAAAACACG

TTCAACTTCATAAACTTTAATAAATTGACCAGGAAGACGCTCTATTTCAATAATTCCTTC

ACTATAGTGAGAGCGTAAAATAATAGGACATATATCTGCTTCCTTAATGTTTTTGGTATT

CGTACCATAAGGAAAACTCATCGTCATGTTAAAAGGGATAGTCAAAGATAAACCATGTAA

CCAAAGTGCTGTCTCCAAAGAATAGATTCCCTTTGGAAATCGATACTGAAGGACAAACCA

CTCATCTAAATAAATATCAGGTAAGCGATATAGCCCTTTTTCTTCAGCATCGATTTTTCC

TTCTGTAATCATTTTCAATAAGGTTTTGTAGTGTAAATTTTCATCTATTACTTGTTTAAA

AGTTAAAAAACCATACTGATTGAAATAGTTCATCAGTTTTTCTCTTTTATCAACCATATC

ATTCTCCTTTCTTAGAAACAAAATACTAACATCTTCTATATATGTTAGCATTTTATTTCT

TTAAAATCAATTCATTTAGTTTTTTATTTCAAACTGAATCTATAAGTAAGACAGACCACC

TATTTTAAACAAGCATCAAACTATAAACTAGTAGGTTCCACACCAAATGTAGCCCCATAC

TGCCCCATAAATCCGATTTATAGCGCACCATCCCTAAAAACATTCCCAATGAAACATACA

AACACCAAGCTAGAATGGTTCCTGGATGATGTACTAAGGCAAATAAAACACTTGTCAAAG

CAACTTGAATATCTAATTTTCTAACCAAGTTCCATAAAATTTCTCGATACAGAAATTCTT

CAACCATACTCGCATTGATTAAAAACAATAAAAATGAAAACCAAGGAACTTGATGTTGAA

GCCCAATTAAGTTTGCTTGATTCGTGGTTCCTTGAGCATGGATTAGGCTAAAACATAGAC

TTATAATCAGTAGGCTAACAAATCCAACACCAAGCCATTTCATCCTAGTTTTCATATTGA

CCTTATGAGCTTGTTTGCGTTGATCATACATCCATAAAAAAGAAATGAGCGACGCTCCAT

AGAGAATCTCTAGTATAGTTAACTCACCGATACAAAGAAATTTCAGTAAGTATAGAGATA

CCAATAGGACATTTACTTGTTGGAATATATAAACTGGAATTATTCTTTTCATAGTTACCT

CCGAAATAAATCTTCATAATCTAAATCTAATACCTGCACAATCCTTTCTACCCATGGACT

TTGAGGCATTCGTTGTTCCATCTTATAGTGACGAATCTTTTGATACAAACGATTCAATTC

ACTTGGATAGTGAAACTCTCCCGCAAACATTTTTCTGGTTAACTCAATCCAGCTGATATT

TCTTTCAGCCAAAATAATGGACAAGTTCTCCCAAAATCGTTCTGCCATATTGCTTCTCCT

TTAGTTAGATAAATAATGTGTTTGTGCCATGTAAATCAATTGTTTCGTATCTCTTGGCAA

TAAAGCTCTAGCCTCCTCCAGATTCAGATTTGGATAAACTCGCTTATTTGAAACCGCAAG

AGGAAGTCTGATGGTTAGTTCAGGATTTTTTAATATCATTTCGATGAAATCTGTTAATTT

TAGGTTATCTCGATTCTTAAAACGTAATAAATTTGGAGATAAAAACTCGAAACAATCTGA

AGAATAGCTCATCATCTCAATTAATTTGTCCTTTGTCATTTCAGAAACTGAATGACAAGA

TACATCGATGCCATAGTTTTGGAAGAAGTCTAAAAGAAGTTGATTTCTTTGGCTATTTTT

ACTTAGATAGAGATCAATCATGGGAGACCTCCCAAAGATTCGGTTCCATTTGATATTCTG

AGACGATTAAGGAATCTAATAAATTTGCGAAGTTAATCGGTTTCTTGTCTTCATCAAAAG

CTTTTACAGTTACTTGGGTTGTAAGTATTCCCTCTTTTCCCTCAGCTCTATAGCCTTGTC

CATATAAAACAAAAACAAGATTCTGATGATCATCTACAAAGGCATCAGCTCCGTTCTTTA

TATCCTGACTTTCAAGGAATTCCATAATGTTTTGAAGATAGGATTCGTAAAATAGGGGGT

AGTTGTGTTTTTTATGGTAATCATCTAAAAATGTCACCTCAAACTCACATGGATAATTGG

GCATCAAAAATATTTGTTCATCCAGCTGTTTGATTTCTGCGTCATGTAATTCTGTTTCTA

ATTCATCACAATCTAGTATTGATTCTTTATTTAATGCTTTCATCTTTTTCCTCTACTTCT

TTTAATTTCTTTGCGATTGCGGCAATCACAGGAACGGTTACACTATTACCAGCTTGTTTA

TAGAGCTGACTATTACTAGAGACTTTTCTAGCAGCTTCAAAAGCCCAATCAGGAAAGCCC

TGCAATCGAAAACACTCTTTAGGAGTGATTCGTCGTATTCTCAAACGGTAAAATTGTCCA

TCTATTAAAACACCAGCTACTTGGTAAACTTGTTTATCTTCTCCTTCATAGCTAGCCACT

ACTACTCCCATTTGCCCACTAGTTGTTAACGTATTAGCTATACCTTTTCCAACTCTACCA

CGACGATGCTGAGAACTTGGTCTTTCTAAATTGATTGAATCCCCAATCTCTGCTTGAGCA

TATCCTTTTTTCGTTGCCTCTCGGACTTTTAGAAATTGGATTGGTTCTGGAATCAGTATT

TTGGGGATTTTATCTCCTCCTTGCATCGTAGTCAGTGTTGGAGATAAGCCCTCACTTCCA

TAGACTCTACCAGTTTCCTTAAAGCTAGTTGGTAAATCTCCAACAACGACAATACCATGG

CGATCCTGAGTATTTAAAGTAAACATTGGCTCTTGATTATCCTTGAAACGTCTACCATTT

TGTCTCTTGTCTAATCTGTCTGGTGTCATACAAGGAATCGCAATTTTAAATCCTTCTCCT

TTTCCACGAACTAAGGTTGGCGCAAGACCTTCTGAATAATAGACTTTACCACTCATTCCA

CTTTTTGATGGATTCAAATTCCCTAATATTTTTAAAGTCTCAGGGTTAGTTGCTTGACCT

TCTCGTCTGAAAGGAAATAAGAGTCTGGTACCTCTCTTTCTAGAATGTCCGATAATAAAC

ACCCTCTCTCTGTTTTGGGGAACGCCAAAATCCTTACTGTTAAGCATCTGCCACTCAACA

TCAAACCCCAATTCATCAAGCGTGGTAAGGATTGTGGTGAACGTCCGTCCCTTATCGTGA

TTGAGTAGGCCTTTAACATTTTCAAGAAAAAGAAAACGTGGTTGGATTTGTTTGGCCGCT

CGAGCAATTTCAAAGAATAAAGTTCCTCTAGTATCTTCAAATCCCAATCGTCTTCCTGCG

ATTGAAAATGCTTGACAAGGGAATCCTCCACAGATGACATCGACTTTCCCTCTAAGTTTT

TTAAATTCGTCATCTGAAACATCTCGTATGTCATGAAATTCAATTTCTCCTTCCGTTTGA

AAAATGGACTTATAAGATTCTCTAGCAAATTTATCAATCTCACAAAATCCAATACACTCG

TGTCCGACACTTTCCATTCCTAGTCGAAAGCCACCGATACCTGAAAATAAATCAATAAAT

CTCATTTCTTTATCCCCCCTTTCTTATCGACTATCATTGTCAGAACTGTCACTCCACTTG

AAATGACAATGAGCATAACTAATATCCACTCGATTGGCGACATTTAACCACCTCCTTTTC

TAACACGGTTGTTCCAAACGGAATACAAGCCATTAAACACAAATTCAGCAAGTACTTCCG

CTCGTCTAACAAAGCGAACATTGTCAAGAGCATACTGATAGATTCTCTCAAAATCAGTCA

TAGCAATTTCACTGGCTGTTTCAGAAAACCCCTCTCGTCTAAATTGATCTTGCACCAATC

CCCAAATATAATCTCGATCATATTTTGTGACCTTTTCTACTTTTCTTTTCAAGATAGGTT

GAGTATCCCTCTCCTCCTCATCCTCAATAAATAAAGAATCAGTCTCACTATATTTAGTCT

CACTAACTTCAGTCTCACTAGGGGCTGAATGTGAGACGGGGGCCGTTTCATTTTCACCCT

GCCCTAGTCTTTTTTTAACACTAGGCCTGTTTGAATTAGCTACTGGGGTAGAAGATAATT

CCCCTAAATAAATCTTATTAGCTAGTCTTCCTTTCTCACTTGAAGACTGTTGAACTTCAT

CAATTAAGTCATATTCTTTAAGAGTTTTTTTGATGGACAGTAATTTTGACTTCGAACAAC

CTAATAGCTTCATCAGTTTAGAATTAGAAAATACTAAATAGACCGCTCCCTCTTCATCTA

TCCAACCACGACTGAGAGATAATTCTAAACGATCTTTTAAAATAGAATAAGCCACCTTTA

CTTCTAGTTTCATATCCATATATTTCTCATCCTCAAAAAGAATTTTAGGTAATTTGTAAT

ACCGTTCTGAAGTTTGGTATTGATTTGCGGTAATTCGTTTCATAGCGTTCCTCCAAGTTC

TTTGAGTATTAAATCTCCACTTGATTCGAAGCGAACCAAGCCACTTTTTTCTAATTCAGC

CATAAGAGATATGGCCTCAACAATATCAATTCCCATTTCACGTACTAAAAATGAAATGAC

AATATAGCGTGATGATTGTAATTCTTTTGTCATTTTTCCTCCAGAAAATAAAAAAACGGA

AAGATTGTGAAATTCCTTTCCGTAACATAATAAATATTTGAACACCAAACTCAACACCAA

AAAAATGTTTTTATTGATGATTATTGCTCAATCAAGCACATATAACAATGTGAATAATTA

AAATCTAAACCAATAAAAACCCTTGAATTTCAAGGGTTTTAAGTATTTATTCTACTTCAC

AATGTTTTATTATTTTAAATCTTCTAGACCAACCATTGAATAGTAGCCATTGAGTTTTTT

CCTTTCGTAGCAAGGATTTAGATCCCCTATTTTATTTTACTATAGTTTGAACA

>ICESpnGA47597

AATTTTAAATTTTCCTGCACCTGTCGACGCGCTTTAGACATGTGACCAGGAAACCATTGA

ATAGTAGCCATTGAGTTTTTTTCCTTTCGTAGCAAGGGTTTAGAGCCCCTATTTTATTTT

ACTATTGTCTAAACACCAAGCGAACACCAAAACTACCATGCAATGGAAAAACCTCTGATT

TGATTCTCACTTGATTTCACAATCTTTATATCAAACTGTGGGTGGTATTTGACAATATCT

TTTTTGATTTTTAATAGTAAATTCGAAATAATATTTTTAGGTGAGTAACGTGGACTAAGA

TGTAACAAGTCTTTGAACTCATCGACACTTAATTCTACTTTATTGCTATTATCACTAGTT

TCAATGAATTTTTCAATTATTCTGGAATATTTACAGGTATAACTTTTCAATTCTTCAAAA

TGGAAATTGTGATTTTCTACAAATTGATTTAAGGCTTTTACAGTATTTTCTTGTGAACGA

TTTATATTATGTGTATAGCCCATTGTTGTCTCAAAGTTAGCGTGTCCTACTCTAGTCATA

ATATCTTTCACTGCTATGTGCATCTCATTACTTTGAAGGTAACTAATATGCATATGCCTA

AACGAATGGGGAGTAACATGTTTTACCCACTTAAAACCATAGTCACTTAAACAATTTGTC

AATAATTTTCCTTCTATTCGTTTCAAAATTTGACGAAAAGTGCTTGATGTTATTGGAGAG

CCGTATTCTGTTCTAAATACACTTTCAGAATGTGTAAAAGCAGGACAGGGATGTTTCTCC

ATATAAGCATCAAACTCTTTATTTCTCTGTATTGTCCTTTTAATAGCTTCGCTTGCAGCT

TCAGGCAAAGCTACTTCTCTAATTGAATTGAGTGTTTTAGTTGTATCAAAGTGAAATTGT

TCAACTTTTAAACAATGATATTGAAGTGCCTTATCAATATGCAAGATTCCTTTTTCAAAA

TCAATATCTGATGGTAAAAAAGCAGCTTCACTAATTCGAATACCTGTAAGCAACAATACT

ATAGCAAGATCATAATAGTTTGCATTTCTGCATTGGCTTAATACATCAAAAAATGCGTGT

AATTCATGGATTTCTAGAAATTTAGAATCATGTCTTTCTTTTGCTTTACGCCTTTTCTCT

AGTGAAATATCTAGTTTTACCGCAGTCATAGGAGAGAACTTAATGACATTATATAACACA

CCATGATTAAAAATCTTATTACAAGTACTTTTTATATGAGTCATTGTTGAAGGTGATGCA

TCATATATTTCTAAATATTTATTGAGACTTTTTTTCATCAGAAGTGGAGTAATCCTGTCT

AATAAAAAATCATCTCCTATAATTTTCCCAAGACGCTTCATCACCAGTAGTTCTCTCTGA

ATTGTTTGTGGTTTAACAGAGACACACCAAGTCTGAAACCAATTTTCTTTTAACTCTCCA

AATGTTGTAATCAGTTCAGGGCTAAACTGACTTTCAAATGAAGTAGTTAGTCTATCTATC

TTATCAAGAACCTCTCTTTCAGCTTGTTTCCTCGCCCTACTAGTATTCCTAGTGTAACTT

ACAGTTACTGATTTCCACTTTCCTGTTAGTGGATCTTTGTACTTTTCAACCACTTGATAT

AAGGGTTGCCCTTTTGAATTTGTTTTAGTTACATAATACATAATTTAATTCCTCTTAACT

CTTAAAAAGAAATAAATCATTGTTGTATAGAATAAATAACTACCCTATATTATACCATGA

CTTCCTTATTTTGTCCGCTTTTGCTTCCATCTTAAGAAACTTTCAAATCCTTCTAGATTA

ATGAAAACAAGTTTATGGGTTGGGTTAATTACATACATGGAAAATGTTCTGTTTTCTCGC

ATTTCTTTAATCCATCTGTTCAATGTATATTGATTTAAACCATCCCATCTTTTTAGAATT

CCTTTTTTATCGGCCCATTCTGCAATGGAATTTTCGACAGATATATATTTTATATTCATG

TCATCATCCTTCCTAAAAAATATTTTCTCCTCCATGGCATGACTGATGAAGTCATCATAA

GAATTTCACTTGCTGCTCTTTCACGGTGGCAGCCTGAACGGTCAGAAGTATCATTGTATA

TCATTTGTATCATGGCATATAAAGTATCGCTCTATTTTATTGATGGTTTTAATATCGCTC

TTATCATGGCGAGCCATTCAAAACTGCTCCACAAATGAGGAAAGTACCATTTTGGACTAT

TCAATTGTCAATGTGCTTTCTTAACCTAATACTTTTTGGAGTAATGGTCTTCTTTATTAT

CCACTCGACCTACAAGGTAATCTAAACTTACATTATAAAAATCTGCAAGTTTGATAAGGT

CATCTATAGAGATTAACCTGGTACCTGATTCCATTTTAGAATACGCTGATCTTGTACAAT

TTAAGATTGTTTTTGCAACATATTCTTGTGTCAAATCATCATCCTCGCGTAAATCTCTAA

TCCTTTTCAACATCCAATGTCTCCTAAAACAAGTATAATATAGTTATTTTCTTATTTTTA

AATATGTGACAAATTGGCACATGAAACTATTCTATATATACCTTTTAGGAAACTGTTTAC

TCTAAATGTATCTCATTTTCCTTAATAGATTTCATATTTTGTTTTTCCTTATATTTCTTT

ATTGTTTTATTAAACATTTCAAATAAATTTATTGCTTCTCTATGTTGTTCCTTCAGTGTT

TCAATCTTTCCTTTATATATGTATAAATCCTTCTCTATTGTATCTACTGAACTTTTACCT

TGAATACTATTTTCTAAATTGCTAGAACTATTTGTCGTATCTTTCAACAATACTTCCTCT

AAATTAGTTAAATCATTAATTTTATTTTGGACTTGTTCAATCATATTCTCTAGATATGAT

ATTTGGGCAATGAAATCATTTGTTATATCTTCAAAATCATTCGAACTATTTTCCGCAGAT

ATAAGAAAATCTAATTGTTCTATTTTTTCTTTAATTTTGCTAAGAGGAATTCTTCTATAC

ATATACTGTGGTTCATACTGAAGATTAAATTGTCTAATCAAAGTTTTACCTTTCATAAAT

CGATTTTTTTCTACTGAATCTTTATGATATACATAGTAAGAACTAGTTTCTCTGAGAAAT

ACTTTAACTTTTTCTTCTTCCATATTGATCTGAATATTTGGTACAAAAATAAGTCCTTCT

TGTCGGATACCAAACTGTACCTTAATGTAAATTCCATCGTCTACTACTTCTTCTATTTGA

TTAAGATTCAACTCTACTTCAAACTCATGAACAGCATCTCTATTTCTCTTGAAATCTTGA

TAAGATTTCCATACCATCTCTTCTGACGGCAACTCTTTTTCTTTAATTATCTTTTCTTCA

TTGTAAAGTTCAACTAAATTTTTATTATCTAAGACAAAAGTTTCATTTTTGTTATTAAAA

TAGTCTTGAAAATAACTAACACTATACAGATTCGTTTTTACCAATTCCTGCTCTGCAAGC

TTAATCCCATCAAATTCAAATAGAACATGTTTTTCTTTCGGAATTATTTTTAAGCCAAAA

ACTTCTGCTCGTTGAATCAATTCATTCATATTCTTCATTTTCGGAAGTAAAAATTCTAAT

ATGTTTATGATTTCCCTTTGAACAAACTTTTTCTCAAAATAAGTTTCATTATAAGGTTGT

TTTCTACTCAATTTACTATCACGTACGACTTGTTTCATATTTGAATCAGTCATAAAATAA

GTAACATGCTTGTGTCTAAAATCAATTTTTAAATGTAAAGCTTTAGCTTTTTTCTTAAGA

TCTTCAAAATTTTTCGAGTTCTCGATTAGAAAATATACCCGTTGTTTTATTTCATATTTG

TAATTTGTTTTGCGATAAACTTCATACTGACGATGCGAATAACGATTTTCTATAATTTTT

GCCCCTGCAATTTTTGAAAGACGATCAGAAACCATTCGTAGATTATGTTCTGCCTTATAA

TCCCATAGAAACTTTTTATCAGAATTCTGATCAATTGAATTTAGGATGATGTGATTGTGG

ATATGACCTTTATCGACATGAGTTGCTACAATAAAACGAAATCTACCTCCTGTCAACTCT

TTAGCTGCCTCATAACCAATTCGATTGATTTGTTCAGGAGTGAGATGGTCATCTGGAGAA

AAGGACTGAATGATGTGATGAGAATGAATTTTTCGTTGATTTACTTCTTGCCTATCATGA

CGAAATTCATAAAGAGTATCATTACTTAAAAAATTATCATTGTACATCTTCACTAGTTCT

TTATAACTAGGAAAATCTAAATAATTTCTCATACCAAAATCTGAAACTAGTGTTAGATTT

TTTGTTTTACTTGGATTCAAAATATATTTGATTAGTTTACTACGATAATTTTTTCCATGA

ATCGCAAAGTGTTTAGTGATGACCATAAAATTTCCTCAATTTTTCAGATCGAATAAGAAA

ATCTTTCTCCACTTCTACTATTAACTCTTCTATACCTTTTCTCAACTCATTTAATTCAAC

TTCCGTTATTAAATTCGAATAATTTATGCTTCTGGCTATTTGATTAATATTATTTCCGAT

TCGTTTTAATTCAAAAATCAAATCTTGATAACTATTTGTATCAATGGTGATAAAATTCAT

ACCAGGATCTAGTAGAGTTCGTCTAGCATATTCTGAGAATGATAAGCAGTTACTTTTTGA

GATATTTTCATTCAATTTGACTAATTCTAGATCAGATAGAAAGACTTTTTTTAAATTTGT

TCTATATCTGTGCTCCACTCTACTACCTCATGTATTTATTACAAAAATCTTCACTAAGAG

GACCTGTTTTTTCTACCTCTTTAATTAATTCCTGAATACAAGTTAACAAAATAGAAACAT

GTTCGTGAGTAACCTGATGATTTGTTTTAGCAATTATGAATACTTCATGAACATCACGAC

TAATTTGTTCCAACTTTTGTTTTTTCCAAAGGTTGAACCATTTTTCCATCTGTTTTTGTC

CATCAGATAATAGTAAGCTTTTACGGAGAAAATCAGAAAAATTATCATCTCCTTTCTCTC

TCATTAAATCTAGTATTTGTTTTTCTTCCGTTTCTGTTAAGCGAAATTGTTTCCGAATAC

TACGTATATTTCTTTTCATATGATTTATCCTCTCTCATAGATACTTGTACTGACAATGTA

AGCTTACATACACTGTCAGTACGTTTTACAAAATAGATTCAACATTTTTGTATGGCTTTG

CGAGCTCAGATATTGTGTCCACAATATCCCAAAAATCATATCGCCTGCCATTTCAAACCT

CACGGTTCAAAAAACAAACGATATGAAAATGTGGTGGCACGCCCCCAAACCCCCAAGATA

AATCTAAATTAAAAAAAGTATAACAAATACTTTTTGATAAAATTATCACAGAAAAAAGAG

CCCTGTCGGACTCTTACAAATTAAAACAATGTCTCTGATATTCATACTGTCTAATACCAC

TACTGTCTTTGTTCATCTTGATAGAAAAAACAAAGTAATGATCACGACCGTCTAAAGATT

TACTTTTGTTAAGTTTAAAATAATGTTTTTGAGACATAGCCATTGTGCGTAAAATATCAT

CTGTGATAAATGTTAATGGTATATCTAAAGCTGAATACTTGTAAAAATCTGACTCAAACT

GTAAATAAGAATCTGAAAAATAATCTAGCTGTAATTTATCATCATATAATTCTTTACGAT

TCATGTAATTCCTCCCCTTCTATCTGTATATCGAGTATATCTTCCCATTTTAATCGAAGA

AAACCAGTGTCTGATTTTATAGATATAAACTCTTTTCCAATCTCTTTAACAATACCTGAT

ACTACTTTTCTTTGATTAGAAAACTTAAAAACAAAAGTTGCAGGGAATACATTCGTATAA

AGTTGACGAACAAATAGAACTTTCTCTTCCAATGATAGGGAAATGGAGATATCTTCTTTA

TTTTTTTCTGCCCAAAGAGAACTGGAATGTTCTGATAAAAAGAAGCCCATCCACTTAGCC

ATGCCACGATCCTGATATACTCGGGCAGACTCAAAAGGTAAATAAGAACGATTCATTATT

TTAGTCCATCTAATCCTCCAGCTGAATGACCACCAATGAGTTTGCTTCTAGCAATTGTTC

TAGAAGCTTGATCTAGGGCATTACCTTTCAATAGTGAAGTGAACCCAAATTCTGTTCGAA

TAGCATCGATTGCGGACTGGAGCCTTTCTTCTTTTTCTATTTTCTCAATATCATCAAATA

ATGAAATCAATCCAAAGGATTCATCCACTAAACCAGAATAGTTAACCGCAACATTCCTGA

TAGCTCCTGAAGTATATTTAGTGTGAAATAACTTTAAAACGTAGTTTGTCAGTAGTGCTG

TTTGATTAGTTGGTTCAATTTTCATTTGAGTATTGATAGAACGCTTTTGTTCCACTTTAG

AGTAGCCTAGATGTATAGAAACTACTGTTGCTTTTTTACCAGATCTTCTCAATCTAACTG

CAACTTGTTCTGCCATCTCACGAAGTATGATCTCAATATCTCTTTGTTTAATATAATCTT

TTGGTAAAACTTGAGAGTTCCCTATCCCTTTTGACTTTGGCTTATAAGGTTTATGAACAT

TACTTTCATCAATCCCATTGGCATGGAACCATAACTCTAGACCCATGATACCAAGCTCTT

TTTTAATCAAGTCAGGATTAGCCTGAGCCAATTCTTTAATCGAAAAGATACCTAAATTAT

GTAATCTTTTCTCCATGCGATTTCCAATTCCCCAGAAATCTGTCATTTTAGGAATAGTCC

ATACTTTCTTTTCGACATCCTCATAGGACCAATTGGCTCTCATTGTCGGAGTCTTTTTAG

CTTCATTATCAAGTGCCAGCTTAGCTAATAAGGGATTGGCATTAGACATGCCTACAGTTG

AATAGATTCCAGTTTTTCTCCAAATCTTTTTTTGAATAGCAGCCGAAATAATATCTAATT

TATCTTTCCTACTAATACTTTTATCTGGTACAAAATAATTTAATGAACTTGTTAAATCAA

TAAATCCTTCATCAATTGAGTAGGGATAAATATCATCTGGTGCTGCAAAATCTTGAAAGA

TTTTTTGAATCTCCATGTTGACAGCAATGTAAGTATCCATTCTCGGAGGAACAATCACGG

TTGATTTTGCCCATTCCTCTATATAGCGGACATAGTCTATCGTTGTCGGTAAACCTTGCT

TCTTAGCATTATAATAAGAGAATTTGCGAGTCTTTACATCAAATGGTAAATCATAGGAAC

GTCCAACGTTCGACTTTCCAAAGACCTTTTTAAACATAGGAGAGGAAGCAAGGATTAAAC

CCGCAGAATTATCTGCACGACTCATAACACAAAGCGAAGTCTTAAGTGGGTGTAATCCTC

TATCTACACATTCTACACTTGCGTAAAAAGATTTCATATCAATAAAGGCAATGTCACTTT

GAGGTTCGAGACTATAATCAAACCAACTCATACTTAGTCCTCTAAAGGCATAAAGTTCCC

AACTATTTTCCCTATAATGCGAGGATTCTCATCATAAGGCGCAAACTTATCTGAATAGTT

CCGATTGAGTGAAACTAAACGAAGCCCATTTTCTTCACGATACACTTTCTTAATATATGT

TTGACCATCCCAATCTATGGCATAGATAGCCCCGTCATAATCAAATCCCGTTTGCTTAAT

AAGGGCTACAGAACCATCTTCATATGTCGGTTCCATTGAATTTCCAAACACCCATGATGC

AAAGTCATAATCAAATTGATGATTAAAATAAACTGTATCGTAATTACCATCATCAAAATA

AGCTGTTCCTGTACCAGCTGATAATTTTTCGTAAACTTTATAAGCAAAACGCTCAGGAAT

TTCTACAACCTTCGCATTCTGTTTGTTCAATAGTTCTGTAGCATAATGAAGTGTTGCTTC

TTGATTCCTTTCCGTTAGTTTGAGATAGGTTTCTACTATTTCATACTCTGATTCAAAATA

TCGAGGGTCTACATTCAAAATCTCACTCAATTTGCTTAGATTATTTTGATTAGGTTTTGT

TTTACCTGATTCCCAATTAAAGTACGAAGCTCTACTAATTCCCAAACTGGATGCAAGTTG

AGATTGAGATAATCCTTGACTTTCTCTTTTTTCTTTTAATTTAGTAGGTGAAAACATTTG

TTCTCCTTTTGTAAGTTTATTAACTAACAAAAGGATACCATAGCTTATTGAAAAAATCAA

GAGTTTAATAACCCCCAAGATTTAAAATAATCATAAACTGTGCTTAATTTGTACTTATAT

TGCTAGGAAACCATCACTTAAGAAAGTTATAACTTTTGAAAAAGATAAAGAACAAGCAAA

AATAGAAAATAAAAAAAAGGATCGTTATTTTTAAAATGACGATCCAATAGACTATATTTT

AAAACTCATGATCTAACAACCATTTCGATTAGTTAAATCTTTCAATCTTTCTTCTCCAGA

TTTAAGCATCTCTTTCTCCACTTGATTCCATTCTCCAAATAGTAAATCATGAAGAACGGT

TGCTGCTGAAGTTTTATCTTCTCCTGAATCATATACACAACTTCTATCTCGTTGGTAAAT

TTGAATTCTTTCAAAGATAGCTAGTTCTTCCAATTGTCGTGTATTATTAACTAGATGATT

TACAATGAAATCATGATGTTCTTTTGGAGTTGCGCGTGCTTGATTTGGATTGATAGCGTA

TAGTTCTTCGTATCGGATAAGGGTGCTCAGATAGGACAGCTTAGGTTTTGTCGCAATCAA

GGCTAATTGTACTTCATATCCCTTATTTTTCAAGAGTTGTGCTGTTTTCTTTGGAACATC

AACTGTTCGTAAAGTTCCCTCTATCAAAAGATTGTATCCCAAATGACTCAATTCTGTTAC

TAAAGACTCTACCATTTTACCTGCAAAATCTTTGGTGTATTCTACACTATCTTTGCCATA

TTCTTGCTGCAGTTCTAAATAGTGTGGATGCTGAGAACGAAAACTATCTCCATCTATGAT

AACAATATTTCCTTGAAATTCTTTCTGTTTAATACGATGAATTGTAGTCTTACCGGCACC

ACTTTGCCCTCCGAGCAAAATCGCTATAGGTTGCTTACTGGACTTTTTTCCTCTTGTCAG

TGAACGAAGATTCCGTGCTAGAGCATGTTTGAATTCACTGTCAGTATAATCTTGGATTTC

CACTAGGCTACCATCCGTTTTTCAGATATCTCTAACATACGTTCAATTCCATCCAAATAG

CCGCTATATCTCTCTATTTCATCAAAAGTTTCTACTAAATAGATATTCGTATGAATCAAG

TCAGATAGATCATCACTCATTAAAATCCAAGGATTAGATTCATCATCAATGCTAATTCCC

TGACTATCTTGATAACGATAGAGTCGAGATAAAAGATTAGCACCTCTTTCTTTCACAATT

TCAATTTTTAAAGTCAATTCATAGTCTTCAACAGGATTGAGCATTTTATCTTCTCCTACA

ATATCGACATAAGATACATTAAACTTCTGACAAATGATGTCTATTAATTCGGTAGAGACT

GAACTCGTTCCATTTTCATAACGACTCAAGCTATTTCGAGAAATTCCTATAATTCGTGCA

AATTCGGGTTGTGTTAAGTCATGTGTTTTACGTAAGGATTTTATGTTCTTTCCAATCATG

GCAAACTCCTTTATTTTTATAATTCAATTATAGCATAAAAAAGTATAACGCACCAATTTT

GGTGCGTTATACTTTTTTTCTAGCCTAAACTTTACTTCTAAAAAAGCCACCTAAAGAGCC

ATAAATATTTATACTTGTATTATTAGGCCATTAGTAAAGCGTATCCAACATCTCAATAAA

GTGTTGTTTTTACTGATATTTTAACTATATTGTATAGTCTACTATTTAAATCTCAATTGG

GATATTTTATAGAATACCCAAAATTCAACAATAACTAAAAGTAAGATTCCATTGTATTTC

CTTGTTGATCTTTCATATCACGAATTTTATTTTTCAGAAGATCAAAGTTACAAATTTCAT

CTCTAATATCGTTATCCGCTCTGTAGCCATTAATCATCTCAGTTAGAGTTTTCCCTTGAC

TACCATTCTTTATAGTGGTATTCACTCTTTTTATCTTGATATCAAAAGGCTCATATGTTT

TATACTTAATTACTTCGTCTTTAAAATCAAATAAAGGTGTATCCGGTTTAGCATCCCATA

AGATAATCAACCACATTATCGCATTATAAAGCTGTTTTGCAGTTTTATTCCACTCTTCAG

GTTGAAAATTTCCACTCCCTCTATTGTAATGTTCTTGTCTATTATTTACATGAGAATTAC

CTACAAGCGGATTAAAAAGAGGTAGCCCTTTGTGATTAATTAGTTCTAAAAAATCCCTTG

CCGCACCCATGCCACACTGAATAACTTCCTTCTTGCCATCAATTTTACTCTCAGCGATAA

ATACATAATATTCATCTTCAATAGTTGCTCCTGCATCACTATGTTTTTGTTGATTTGCTA

ACAGCTTAATATGTGCTTTAGGCTGAACTTCAAAATCTCTAACAATTCTTTGTCTTGTTT

CATGTCCTCTACAATTCATTTAAGTTCTCCTTTACAAGTTTAGTAACTAGCCTTATTATA

CTATACAAGTTGAGTAAAAAATCATAACCTTCATCAACTAGTTTTTGAAAATCACAAATC

CTTTTGGAGAGATGACAAGCTGATTCTCTAATTCTTGGCAATTGCTTGCTAGTGCTACTG

TTTCCTTCTCTAAAAGATAATCTTCTTTGGACTGGTTAAAGATAGCTTTAAGAATTTGTC

CATCGCATTTCCATTCCAATGCTACTACATCTTGTTTAATTTCTTGGAGACTATACTTGC

CATGCTGAATGATTGCTGACGCGGGTTTCCGAATCTCAATCAGCTTCTTCATAAAGTTCA

GCATATCATTATCACTTGAGACGCGTTCCCAAGGCATACAACGACGGCAATCTGGATCTG

GACCTCCAGTTAAGGATAACTCTGTTCCGTAATAGATACACGGTGTTCCTTTTTGTAGAA

AGAGAAAGGCTAAGGCTGATTTGACCAGTTGAACATCCTCATTGGCCGTCCACAAGATTC

GCTCTGTATCATGCGAATCCAAGAGATTAAACATAACTTCTGAAATCTGCTGCTTGTAAT

ACATAGATTGGTCATTGATTTCATCGATGAACTGGTCTGTCTTCTTAACTCCTCGTAAGA

AATAGTCCTTGATGCTATCAGATAAAGGATAATTCATGACCGCGTGGAACTCATCTCCAT

TTAGCCAAGGCTGAGACGTATGCCAGACTTCTCCTAAAATATAAAGATCAGGCTTTTTAG

CTAGAACTGTCTTGCGAAAATCCCTCCAAAACTGATGGTCAATCTCATTAGCCACATCCA

AACGCCAAGCATCGATATCAAACTCTTCAATCCAATAAGTCGCAACCTTTAAAAGATAAT

CCTTGACCTCTGGATTAGCTGTATTTAATAATAGGGGTAACTATTGCCGGCGAGGCTAGT

TACCCTTAAGTTATTGGTATGACTGGTTTTAAGCGCAAAAAAAGTTGCTTTTTCGTACCT

ATTAATGTATCGTTTTAAATGACTAGTAAAAAACATACATAGAAAGGGGAAAAAGCAAAG

AAATCAAGTATTTCAGAAATTTATTAAACGTCATATTGGAGAGAATCAAATGGATTTAGT

TGAAGATTGCAATACATTTCTGTCTTTTGTAGCTGATAAAACTTTAGAAAAACAGAAATT

ATATAAAGCTAATTCTTGTAAAAATCGATTTTGTCCTGTCTGTGCTTGGAGAAAAGCTAG

AAAAGATGCATTGGGTTTATCTTTGATGATGCAATATATTAAGCAGCAAGAGAAAAAGGA

GTTTATCTTTTTAACTTTGACTACACCTAATGTAATGAGTGATGAATTAGAAAATGAAAT

AAAACGTTATAATAATTCTTTTAGAAAACTTATAAAGAGAAAAAAAGTAGGTAGTGTTAT

AAAGGGATATGTTCGTAAGTTAGAGATTACATATAATAAAAAAAGAGATGATTATAATCC

TCATTTTCATGTGTTAATTGCAGTAAATAAATCGTATTTCACAGATAAAAGATATTATAT

TAGCCAACAAGAATGGTTAGATTTATGGCGTGATGTAACGGGCATTTCAGAAATAACACA

AGTTCAAGTTCAAAAAATAAGACAAAATAATAATAAAGAATTATATGAAATGGCTAAGTA

TTCTGGTAAAGATAGTGATTATTTAATAAATCAAAAAGTCTTTGATGCATTTTATAAATC

ACTTAAAGGTAAACAGGTATTAGTTTATTCAGGATTATTTAAAGAGGCTAAAAAGAAATT

AAAAAATGGGGATTTAGATTACTTAAAAGAAATTGATCCAACCGAATATATCTATCAAAT

TTTTTATATTTGGAAACAAAAAGAGTATTTAGCTAGTGAACTTTATGACTTAACAGAACA

AGAAAAAAGAGAAATTAATCACAAAATGATAGACGAAATCGAGGAAGAACAATAACAAAA

TATAAGTGCTAACAGCTGACCTCCCGATAACACCATGTAGTTATTGGGAGGTCAGCTGTT

GAATTATGCACGAGTATTTTAAAAGTTATTGTGATGACGACGATAAACGATTATCAAAAG

TATAATGTTAAAATGCTTTATTATACTAACGTTATATAAACATTATACTTTCGTTATACA

AATTTTAACCCTGTTAGGAACTATAAAAAATCATGAAAATTTTAATTTGCATGTAACTGG
[truncated: 772,812 more chars]
