# Supplementary figures and images for: Composite mobile genetic elements disseminating macrolide resistance in Streptococcus pneumoniae
Source: Front Microbiol. 2015 Feb 9;6:26. doi: 10.3389/fmicb.2015.00026 (PMC4321634; doi:10.3389/fmicb.2015.00026)

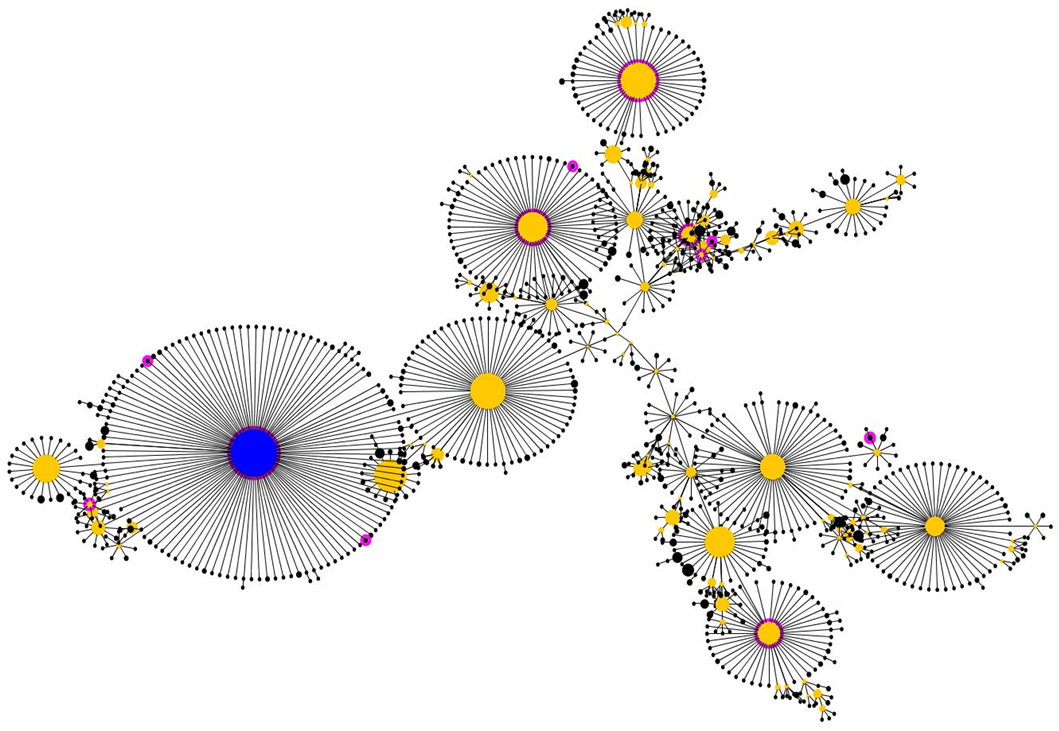

Supplement: Supplementary file 1 [file DataSheet1.ZIP › Supplementary Material/Figure S1.TIF]
